# Genome-Wide Scans for Delineation of Candidate Genes Regulating Seed-Protein Content in Chickpea

**DOI:** 10.3389/fpls.2016.00302

**Published:** 2016-03-23

**Authors:** Hari D. Upadhyaya, Deepak Bajaj, Laxmi Narnoliya, Shouvik Das, Vinod Kumar, C. L. L. Gowda, Shivali Sharma, Akhilesh K. Tyagi, Swarup K. Parida

**Affiliations:** ^1^International Crops Research Institute for the Semi-Arid TropicsPatancheru, India; ^2^National Institute of Plant Genome ResearchNew Delhi, India; ^3^National Research Centre on Plant BiotechnologyNew Delhi, India

**Keywords:** chickpea, GBS, GWAS, QTL, seed-protein content, SNP

## Abstract

Identification of potential genes/alleles governing complex seed-protein content (SPC) is essential in marker-assisted breeding for quality trait improvement of chickpea. Henceforth, the present study utilized an integrated genomics-assisted breeding strategy encompassing trait association analysis, selective genotyping in traditional bi-parental mapping population and differential expression profiling for the first-time to understand the complex genetic architecture of quantitative SPC trait in chickpea. For GWAS (genome-wide association study), high-throughput genotyping information of 16376 genome-based SNPs (single nucleotide polymorphism) discovered from a structured population of 336 sequenced *desi* and *kabuli* accessions [with 150–200 kb LD (linkage disequilibrium) decay] was utilized. This led to identification of seven most effective genomic loci (genes) associated [10–20% with 41% combined PVE (phenotypic variation explained)] with SPC trait in chickpea. Regardless of the diverse *desi* and *kabuli* genetic backgrounds, a comparable level of association potential of the identified seven genomic loci with SPC trait was observed. Five SPC-associated genes were validated successfully in parental accessions and homozygous individuals of an intra-specific *desi* RIL (recombinant inbred line) mapping population (ICC 12299 × ICC 4958) by selective genotyping. The seed-specific expression, including differential up-regulation (>four fold) of six SPC-associated genes particularly in accessions, parents and homozygous individuals of the aforementioned mapping population with a high level of contrasting SPC (21–22%) was evident. Collectively, the integrated genomic approach delineated diverse naturally occurring novel functional SNP allelic variants in six potential candidate genes regulating SPC trait in chickpea. Of these, a non-synonymous SNP allele-carrying zinc finger transcription factor gene exhibiting strong association with SPC trait was found to be the most promising in chickpea. The informative functionally relevant molecular tags scaled-down essentially have potential to accelerate marker-assisted genetic improvement by developing nutritionally rich chickpea cultivars with enhanced SPC.

## Introduction

Chickpea (*Cicer arietinum* L.) is the second most consumed important food legume crop globally and stands third in production among all legume crops world-wide (Kumar et al., 2011; FAOSTAT: Production-Crops, 2012; Varshney et al., 2013b). The chickpea seeds are considered highly nutritious and largely cultivated in arid and semi-arid regions of the world (Kumar et al., 2011; Varshney et al., 2013b). The seeds of chickpea serve as a staple source of protein in human diets. The seed-protein content (SPC) is thus an extremely vital component of nutrition for both humans and animals (Millerd, 1975; Monti and Grillo, 1983; Duranti and Gius, 1997; Kumar et al., 2011; Varshney et al., 2013b; Jadhav et al., 2015). Protein malnutrition, a commonly observed problem in infants/young children, especially of developing countries can be addressed by adequate intake of diet enriched with high protein content (Monti and Grillo, 1983; Haider and Haider, 1984; Duranti and Gius, 1997; World Health Organization [WHO], 2013). Besides, the chickpea seeds being significantly rich in almost all essential amino acids (except sulfur-containing types) can be consumed along with cereals as a daily human diet to mitigate the problem of protein-calorie malnutrition (Monti and Grillo, 1983; Haider and Haider, 1984; Duranti and Gius, 1997; Jukanti et al., 2012; World Health Organization [WHO], 2013). Considering the importance of SPC in global food as well as nutritional security, improving the protein content and quality in the most consumed part (seeds) of chickpea is now the most challenging task in molecular breeding and genomics research. The SPC is an important quality component agronomic trait in chickpea. The protein content in the chickpea seeds is considered the best among all legume proteins that significantly varies (17–22% of total dry seed mass) across core and mini-core germplasm lines, landraces, and cultivated *desi* and *kabuli* accessions (Upadhyaya and Ortiz, 2001; Upadhyaya et al., 2002, 2006; Jukanti et al., 2012; Jadhav et al., 2015). However, the proportionate storage and accumulation of protein content in chickpea seeds is a quantitative trait and regulated by many major genes/QTLs (quantitative trait loci) through a complex molecular genetic mechanism (Frimpong et al., 2009; Jadhav et al., 2015). Henceforth, it would be interesting to decipher the genetic factors and functional gene regulatory mechanism underlying SPC variation in chickpea.

The genomes of *desi* (∼520 of ∼740 Mb estimated genome size) and *kabuli* (sim544 Mb) chickpea cultivars with contrasting agro-morphological features representing diverse genepools, have been sequenced (Jain et al., 2013; Varshney et al., 2013a; Parween et al., 2015). These available genomic resources have assisted in NGS (next-generation sequencing)-based genome/transcriptome sequencing of a diverse array of *desi, kabuli*, and wild chickpea cultivars. These efforts further accelerated development of numerous informative genomic/genic microsatellite and single nucleotide polymorphism (SNP) markers and their high-throughput genotyping in numerous natural and mapping populations by NGS-/array-based assay at a genome-wide scale in chickpea (Garg et al., 2011, 2014; Agarwal et al., 2012; Hiremath et al., 2012; Jhanwar et al., 2012; Singh et al., 2013, 2014; Kudapa et al., 2014; Pradhan et al., 2014; Parida et al., 2015). These inputs are known to expedite various high-throughput genetic analysis, including construction of high-resolution genetic linkage maps, molecular mapping of QTLs/genes and trait association analysis in chickpea (Nayak et al., 2010; Gujaria et al., 2011; Thudi et al., 2011, 2014; Gaur et al., 2012; Hiremath et al., 2012; Kujur et al., 2013, 2014, 2015a,b,c; Saxena et al., 2014a,b; Bajaj et al., 2015a,b,c; Das et al., 2015). Most of these genomics-assisted breeding efforts have uncovered informative markers tightly linked to the major QTLs/genes controlling abiotic (drought and salinity tolerance)/biotic (*Ascochyta* blight and *Fusarium* wilt resistance) stress tolerance and yield-contributing traits for marker-assisted genetic improvement of chickpea (Kujur et al., 2013, 2014, 2015a,c; Sabbavarapu et al., 2013; Saxena et al., 2014b; Thudi et al., 2014; Varshney et al., 2014a,b,c; Bajaj et al., 2015b,c; Das et al., 2015). However, no comprehensive efforts have been made so far to understand the genetic inheritance pattern as well as to identify the functionally relevant genetic loci governing SPC in chickpea. To decipher the complex genetic architecture of SPC, tremendous progress has been made in the area of genomics-assisted breeding by identification (fine mapping/map-based isolation and genetic association mapping) and introgression of useful genes/QTLs governing this vital quality trait in multiple crop plants, including cereals and legumes (Bourgeois et al., 2011; Krajewski et al., 2012; Lestari et al., 2013; Lu et al., 2013; Hwang et al., 2014; Peng et al., 2014; Jadhav et al., 2015; Wang et al., 2015; Warrington et al., 2015). However, very limited attention has been paid toward identification of QTLs/genes regulating SPC, which can be deployed in marker-assisted genetic improvement of chickpea. Only one such preliminary attempt has been made recently to identify SSR marker loci associated with SPC through 23 microsatellite marker-based trait association mapping strategy in chickpea (Jadhav et al., 2015). The added-advantage and broader applicability of integrated genomic approach combining marker-based genome-wide association study (GWAS), QTL mapping, and differential gene expression profiling in rapid quantitative dissection of complex yield component traits is well documented in chickpea (Kujur et al., 2013, 2014, 2015a,b,c; Saxena et al., 2014a; Bajaj et al., 2015b,c). Therefore, this integrated approach can be employed in natural/mapping populations for quantitative dissection of a quality component trait like SPC, with an ultimate aim of marker-assisted genetic enhancement to develop high protein-rich cultivars of chickpea.

In this context, our study utilized genome-wide SNPs discovered by genotyping-by-sequencing (GBS) of natural germplasm accessions in GWAS to identify potential genomic loci (genes) governing SPC in chickpea. Large-scale validation of these trait-associated genomic loci/genes in natural and mapping populations was performed by selective genotyping and differential expression profiling to delineate functionally relevant allelic variants in the candidate genes regulating SPC in chickpea.

## Materials and Methods

### Genotyping of Genome-Wide SNPs

For discovery and high-throughput genotyping of genome-wide SNPs, 336, including 92 (39 *desi* and 43 *kabuli*) and 244 (167 *desi* and 77 *kabuli*) diverse accessions from the available chickpea germplasm collection were screened as per Kujur et al. (2014, 2015a,b). The genotyping as well as structural/functional annotation information of *kabuli* reference genome-based SNPs mined from the sequenced 92 diverse chickpea accessions using a GBS assay were acquired (Kujur et al., 2015b). Large-scale gene-based SNPs selected from afore-said GBS data were further genotyped in the genomic DNA of 244 accessions using Illumina GoldenGate SNP genotyping assay (following Bajaj et al., 2015c). The SNP genotyping information generated among accessions was utilized further for trait association mapping.

### Phenotyping for Seed-Protein Content

A set of 336, including 92 and 244 chickpea accessions were grown in the field (randomized complete block design with two replications) during crop season for two consecutive years (2012 and 2013) at two diverse geographical locations (New Delhi: 28° 4′ N/77° 2′ E and Patancheru, Hyderabad: latitude 17° 3′ N/longitude 77° 2′ E) of India. The mature seeds of each accession (2–4 representative plants from each accession) were harvested and collected individually. To estimate the protein content, healthy (free from dust and metal particles contamination) mature seeds (20 g) of chickpea accessions were analyzed at Central Analytical Services Laboratory, ICRISAT (International Crops Research Institute for the Semi-Arid Tropics), Patancheru, Hyderabad. Before grinding, the seed samples from each accession were washed with distilled water and oven-dried at 60°C for 48 h. The dried-seed samples (20 g) were powdered in a mill with Teflon chambers. The ground samples were further kept overnight in an oven at 60°C for drying. The digestion of the standards and samples was performed simultaneously with appropriate blanks in duplicates (two independent analyses). One g of ground sample was transferred to a digestion tube of 75 ml capacity containing 10 ml of tri-acid mixture of nitric, sulfuric and perchloric acid in the ratio of 10:0.5:2 (v/v). The contents were left overnight in a digestion chamber for cold digestion. To get clear and colorless digests, the samples were digested initially at 120°C for 1 h following digestion at 230°C for about 2 h. With the subsequent cooling of the digests, the contents were dissolved in distilled water and volume made up to 75 ml, and shaken well. Aliquots were retrieved from the digests and the protein content (% of total dry seed weight) of seed samples was determined in the digest using an Autoanalyzer (Singh and Jambunathan, 1980). The quality of SPC of accessions was estimated on a plot basis and their mean values were used for statistical analysis. The broad-sense heritability (H^2^), coefficient of variation (CV), analysis of variance (ANOVA), and frequency distribution of SPC among accessions were estimated using SPSS v17.0 as per Bajaj et al. (2015c).

### Association Mapping

The phylogenetic tree and population genetic structure information generated from 92 and 244 chickpea accessions were acquired from the earlier study of Kujur et al. (2014, 2015a,b), respectively. The principal component analysis (PCA) among accessions was performed using TASSEL v5.0^1^ and GAPIT following Bajaj et al. (2015a). To determine genome-wide linkage disequilibrium (LD) decay, the genome-wide genotyping data of SNPs physically mapped on eight *kabuli* chromosomes were analyzed with PLINK and the full-matrix approach of TASSEL v5.0 as per Kujur et al. (2015b). Consequently, the genome-wide LD decay by plotting average *r*^2^ (frequency correlation among pair of alleles across a pair of SNP loci) against 50 kb uniform physical intervals across chromosomes was determined. The genome-wide SNP genotyping and SPC phenotyping information along with population structure ancestry coefficient (Q), kinship matrix (K), and PCA (P) data of accessions were analyzed by mixed model (P+K, K, and Q+K)-based CMLM (compressed mixed linear model) approach of GAPIT (as per Kujur et al., 2015a and Kumar et al., 2015). The relative distribution of observed and expected –log_10_(*P*)-value in each trait-associated genomic loci was compared based on quantile-quantile plot. The corrections of these adjusted *P*-value threshold of significance for multiple comparisons were performed by false discovery rate (FDR cut-off ≤ 0.05) to determine the accuracy and robustness of SNP marker-trait association. The genomic (gene-derived) SNP loci exhibiting significant association with SPC at lowest FDR adjusted *P*-values (threshold *P* < 1 × 10^-7^) and highest *R*^2^ (degree of SNP marker-trait association) were identified in chickpea.

### Validation of SPC-Associated SNPs in a Bi-Parental Mapping Population

To ascertain the potential of detected genomic SNP loci for SPC trait association, these SNPs were targeted to validate in a traditional bi-parental intra-specific F_7_ RIL (recombinant inbred line) mapping population [*C. arietinum desi* accession ICC 12299 (SPC: 16.5%) × *C. arietinum desi* accession ICC 4958 (SPC: 21.1%)] contrasting for SPC trait. For this, two parental accessions and five of each homozygous mapping individuals with contrasting level of low and high SPC (%) were selected for DNA isolation. The SPC-associated SNPs showing polymorphism between the parents of a mapping population were further genotyped in the above-mentioned homozygous mapping individuals using MALDI-TOF MS (Matrix-Assisted Laser Desorption/Ionization Time-of-Flight Mass Spectrometry) SNP genotyping assay as per Saxena et al. (2014a,b). The presence of SPC-associated SNP allelic variants in low and high SPC-containing homozygous mapping individuals and parental accessions was determined to validate the trait association potential of target genomic loci.

### Expression Profiling

To assess the regulatory pattern of SNPs-carrying genes associated (validated by GWAS and selective genotyping in mapping population) with SPC, the differential expression profiling of these genes was performed using quantitative RT-PCR assay. The leaves as well as early cell division [10–20 days after podding (DAP)] and late seed maturation (21–30 DAP) developmental stages (as defined by Bajaj et al., 2015b) of four chickpea accessions as well as parents and four homozygous RIL individuals of a mapping population (ICC 12299 × ICC 4958) with contrasting levels of low and high SPC (%) were used for RNA isolation. RNA isolated from three independent biological replicates of each sample and two technical replicates of each biological replicate with no template and primer as control were used in the quantitative RT-PCR assay. The quality of purified RNA was tested by denaturing agarose gel-based assay and NANODROP 2000 Spectrophotometer (Thermo Scientific, NanoDrop products, USA). One microgram of high quality total RNA was utilized for cDNA synthesis using first strand cDNA synthesis kit (Applied Biosystems, USA). The cDNA (1:100 dilution) along with 1X Fast SYBR Green Master Mix (Applied Biosystems) and 200 nM of forward and reverse gene-specific primers (Supplementary Table S1) in a total reaction volume of 10 μl were amplified in 7500 Fast Real-Time PCR system (Applied Biosystems). The differential expression profile of genes among tissues/stages of accessions, mapping parents and individuals was determined following Bajaj et al. (2015b). An internal control gene elongation factor 1-alpha (*EF1α*) exhibiting consistent expression across diverse tissues and developmental stages of chickpea accessions (Garg et al., 2010) was utilized for normalization of expression value. Significant difference in gene expression at two seed developmental stages of low and high SPC-containing accessions as compared to leaf (considered as reference calibrator and assigned as 1) was determined by LSD-ANOVA significance test using SPSS 17.0^2^. The significant difference in gene expression was visualized by a heat map using MultiExperiment Viewer (MeV^3^).

## Results

### GWAS of Seed-Protein Content in Chickpea

**FIGURE 1:**
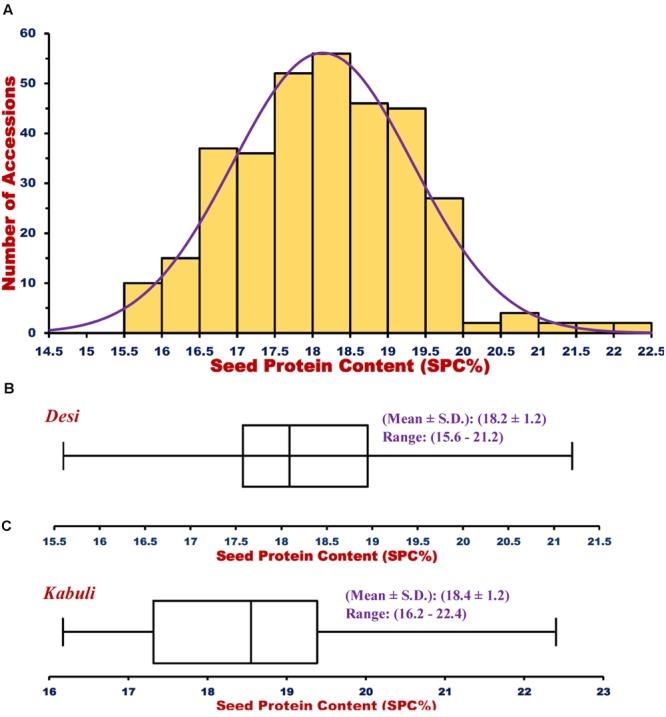
**(A)** Frequency distribution of seed-protein content (SPC %) trait variation measured in a structured population of 336 *desi* and *kabuli* chickpea accessions illustrated a goodness of fit to the normal distribution. Boxplots depicting the variation of SPC trait across 206 *desi* **(B)** and 120 *kabuli* **(C)** chickpea accessions. Box edges signify the lower and upper quantiles with median values in the middle of the box.

**FIGURE 2:**
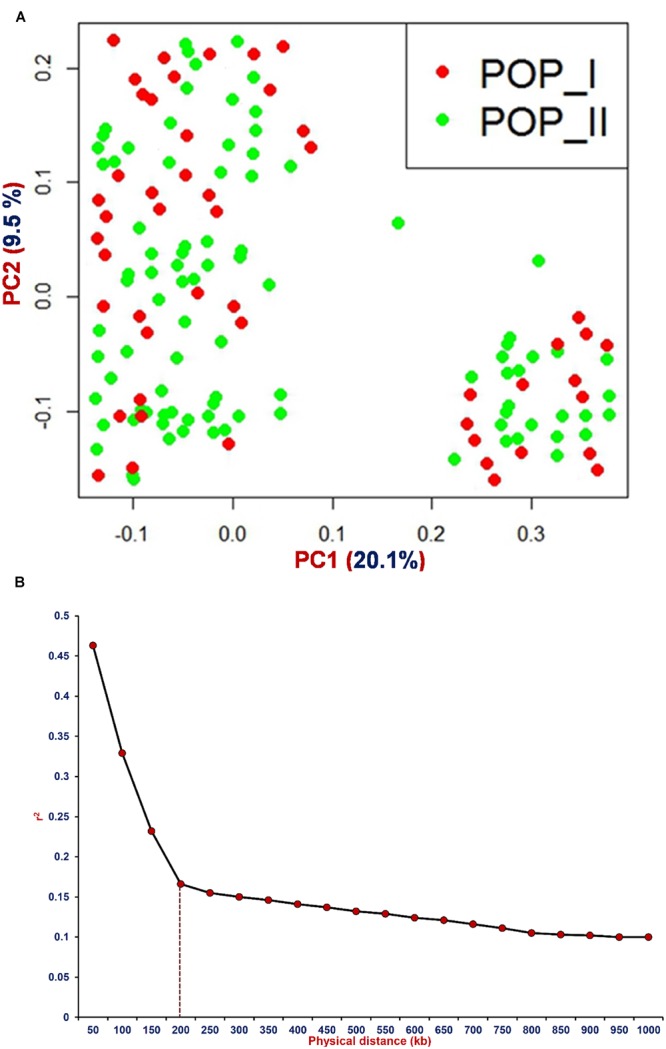
**The determination of **(A)** principal component analysis (PCA) employing 16376 genome-wide GBS-SNPs classified 336 *desi* and *kabuli* chickpea accessions into two major populations [POP I (red) and POP II (green)].** In PCA, the PC1 and PC2 explained 20.1 and 9.5% of the total variance, respectively. **(B)** LD decay (mean *r*^2^) measured in a population of 336 chickpea accessions using 14115 chromosome-wise physically mapped SNPs. The plotted curved line denotes the average *r*^2^ values among SNPs spaced with uniform 50 kb physical intervals from 0 to 1000 kb across *kabuli* chromosomes.

For GWAS, 2 years replicated phenotyping data of SPC (% of total dry seed weight) measured for 336 (including 92 and 244 *desi* and *kabuli* accessions) chickpea accessions (association panel) were analyzed with diverse statistical measures to infer the genetic inheritance pattern of traits under study (**Table 1**). This revealed a wider phenotypic variation [15.6–22.4% with a mean (17.6) ±*SD* (1.2) and 80% H^2^] along with normal frequency distribution of SPC among chickpea accessions (**Figure 1**, **Table 1**). However, almost a similar level of phenotypic variation for SPC in 206 *desi* (15.6–21.2% with 18.2 ± 1.2) and 120 *kabuli* (16.2–22.4% with 18.4 ± 1.2) accessions was observed (**Figure 1**). To perform GWAS, the high-throughput genotyping data of 16376 reference genome-based GBS-SNPs generated from 92 sequenced and genotyped accessions (by GBS assay) were utilized. This included genotyping information of 3072 SNPs generated by their high-throughput genotyping in 244 accessions using Illumina GoldenGate assay (Supplementary Table S2). Of these, 14115 and 2261 SNPs were physically mapped on eight chromosomes and unanchored scaffolds of *kabuli* genome, respectively. The neighbor-joining phylogenetic tree, high-resolution population genetic structure, and PCA classified 336, including 92 and 244 *desi* and *kabuli* chickpea accessions into two distinct population groups (POP I and POP II; Kujur et al., 2014, 2015b) (**Figure 2A**). The determination of LD pattern (*r*^2^) based on genotyping information of 14115 genome-wide SNPs (mapped on chromosomes) in 336 accessions exhibited 150–200 kb LD decay across chromosomes (**Figure 2B**). A non-linear regression curve exhibiting a decreasing trend of LD decay with an increase in the physical distance (kb) was observed. All these accessions sustained a significant level of LD (*r*^2^ ≥ 0.1) up to a physical distance of 1000 kb. However, *r*^2^ decreased to half of its maximum value at approximately 150–200 kb physical distance in chickpea chromosomes.

**Table 1:** Diverse statistical measures estimated for seed-protein content variation observed in a chickpea association panel.

Years	336 *desi* and *kabuli* accessions				
	(Mean ±*SD*)	Range (% of total dry seed weight)	Heritability (H^2^%)	Coefficient of variation (CV%)	ANOVA significance (*P*)
2012	18.1 ± 1.2	15.6–22.4	80	6.6	<0.0001
2013	17.6 ± 1.5	15.9–22.1	81	8.5	<0.0001

**FIGURE 3:**
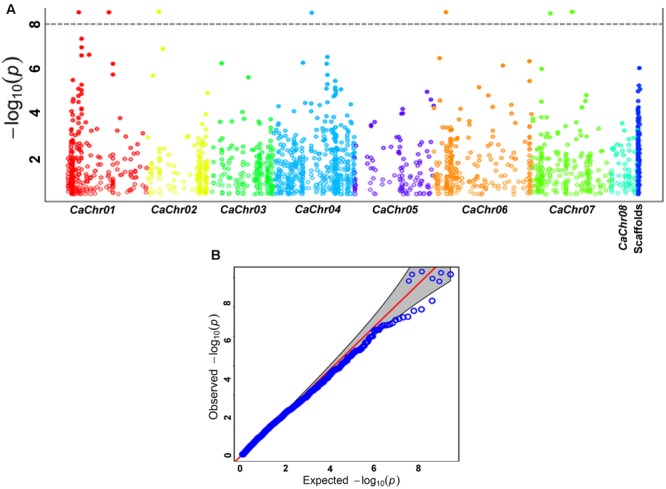
**(A)** GWAS-based Manhattan plot illustrating significant *P*-values (measured by CMLM model) associated with seed-protein content (SPC %) using 16376 genome-wide GBS-SNPs in chickpea. The x-axis represents the relative density of SNPs physically mapped on eight chromosomes and scaffolds of *kabuli* genome. The *y*-axis denotes the –log_10_ (*P*)-value for significant association of seven SNPs with SPC trait. The SNPs revealing significant association with SPC trait at cut-off *P*-value ≤ 1 × 10^-8^ are demarcated with dotted lines. **(B)** Quantile-quantile plot depicting the comparison between expected and observed –log_10_ (*P*)-values at a FDR cut-off < 0.05 to scan the significant genomic SNP loci associated with SPC trait in chickpea.

The CMLM model-based GWAS in 336 accessions using 16376 genome-wide GBS-SNPs identified seven maximum effect genomic loci (gene-based SNPs) revealing significant association (*P* ≤ 10^-8^) with SPC of chickpea (**Figure 3A**, **Table 2**). The association potential of these seven genomic SNP loci with SPC trait was further ascertained by quantile–quantile plot analysis of their observed and expected –log_10_(*P*)-value at a FDR cut-off ≤ 0.05 (**Figure 3B**). These seven SPC-associated SNPs were physically mapped on five *kabuli* chromosomes (except chromosomes 3, 5, and 8). A maximum of two SPC-associated genomic loci were mapped on each of the chromosomes 1 and 7. Four and two of seven SPC-associated genomic loci was represented from diverse coding (two of each synonymous and non-synonymous SNPs) and non-coding (two intronic) sequence components of six genes, respectively (**Figure 3A**, **Table 2**). The remaining one genomic SNP was derived from the intergenic region of *kabuli* genome. The proportion of phenotypic variation for SPC explained (*R*^2^) by maximum effect seven genomic SNP loci among 336 *desi* and *kabuli* chickpea accessions varied from 10 to 20% (*P*: 1.7 × 10^-8^ to 1.2 × 10^-9^; **Table 2**). The phenotypic variation for SPC explained (*R*^2^) by all significant seven SNPs in combination was 41%. Notably, one non-synonymous SNP-containing zinc finger transcription factor gene exhibited strong association (*R*^2^: 20% at *P*: 1.2 × 10^-9^) with SPC in chickpea (**Table 2**). This was followed by another high SPC-associated (*R*^2^: 17% at *P*: 2.5 × 10^-9^) non-synonymous SNPs-containing candidate gene that encode cytidine (CMP) and deoxycytidylate (dCMP) deaminases. To determine the effect of diverse *desi* and *kabuli* genetic backgrounds in SPC trait association, GWAS was performed by selecting genotyping, phenotyping, population genetic structure and PCA data of 206 *desi* and 120 *kabuli* chickpea accessions independently. A similar level (*R*^2^ and *P*-values) of association potential for seven genomic loci with SPC was observed irrespective of any genetic backgrounds in chickpea.

**Table 2:** Seven SNPs-containing genes regulating seed protein content delineated by using an integrated genomic approach in chickpea.

SNP IDs	*Kabuli* chromosomes	SNP physical positions (bp)	SNPs	Gene accession IDs	Structural annotation	Putative functions	Association analysis	
							*P*	*R*^2^ (%)
^∗^#CakSNP881	1	8112343	C/T	Ca08057	CDS (Syn)	ATP-dependent RNA helicase DEAD-box	3.7 × 10^-9^	13
^∗^#CakSNP1726	1	27017765	A/T	Ca20299	INTRON	Cystathionine-beta synthase	1.7 × 10^-8^	10
CakSNP2438	2	9756955	G/A	–	INTERGENIC	–	2.1 × 10^-8^	10
^#^CakSNP6395	4	17920616	C/G	Ca18632	CDS (Syn)	ABC transporter transmembrane domain-encoding protein	3.3 × 10^-9^	14
^∗^#CakSNP9644	6	3548121	C/A	Ca05955	CDS (NSyn)	Cytidine (CMP) and deoxycytidylate (dCMP) deaminases	2.5 × 10^-9^	17
^∗^#CakSNP12015	7	5854598	C/T	Ca06772	INTRON	G10 protein	1.5 × 10^-8^	12
^∗^#CakSNP12753	7	24181321	C/T	Ca19912	CDS (NSyn)	Zinc finger transcription factor	1.2 × 10^-9^	20

*^∗^Validated in an intra-specific mapping population (ICC 12299 × ICC 4958) contrasting for SPC. ^#^Validated by differential expression profiling. CDS: Coding sequence; Syn: Synonymous; NSyn: Non-synonymous.*

### Validation of SPC-Associated Genomic Loci in an Intra-specific Mapping Population

**FIGURE 4:**
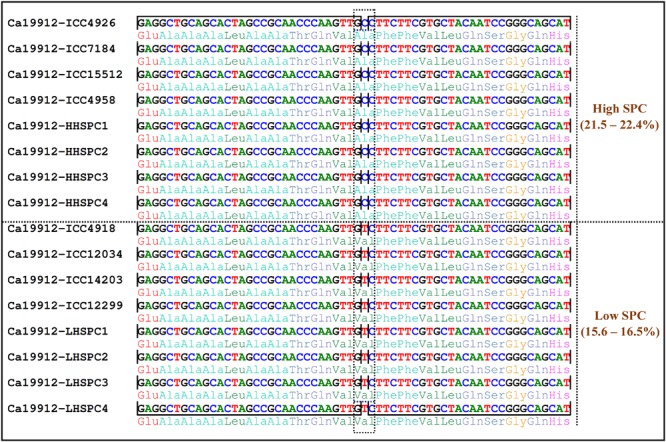
**SNP (C to T) exhibiting missense non-synonymous amino acid substitution [alanine (GCC) to valine (GTC)] in a zinc finger transcription factor gene (Ca19912) of *kabuli* differentiated the high from low SPC-containing accessions, parents, and homozygous RIL individuals of an intra-specific mapping population (ICC 12299 × ICC 4958).** The sequence region containing the non-synonymous SNP is highlighted with a dotted box.

To ascertain the association potential of GWAS-derived genomic loci for SPC trait, seven SPC-associated SNPs revealing parental polymorphism were genotyped in 10 of each low (15.1–16.3%) and high (21.0–22.2%) SPC-containing homozygous individuals derived from an intra-specific F_7_ RIL mapping population (ICC 12299 × ICC 4958). Five SNPs in the candidate genes (encode ATP-dependent RNA helicase DEAD-box, cystathionine-beta synthase, CMP and dCMP deaminases, and G10 and zinc finger protein) associated with SPC, were also validated in a chickpea mapping population with contrasting level of SPC by selective genotyping. Interestingly, the presence of identical low and high SPC-associated alleles derived from these five gene-derived SNP loci in parental accessions (ICC 12299 and ICC 4958) and homozygous mapping individuals with contrasting low (15.6–16.5%) and high (21.5–22.4%) levels of SPC was observed. Like-wise, the five gene-derived SNP alleles were validated three of each low (*desi* cv. ICC 4918, *kabuli* cv. ICC 12034 and *kabuli* cv. ICC 14203) and high (*desi* cv. ICC 4926, *desi* cv. ICC 7184 and *kabuli* cv. ICC 15512) SPC-containing natural germplasm accessions (**Figure 4**, **Table 2**). Therefore, strong marker allele effect of these five genes with high and low SPC differentiation was apparent in chickpea. In contrast, low and high SPC-associated alleles of an intergenic SNP and a candidate gene (ABC transporter transmembrane domain-encoding protein)-derived SNP could not correspond to the phenotypes of low and high SPC-containing accessions/parents and homozygous mapping individuals. Henceforth, five SNPs-carrying genes validated by GWAS and selective genotyping in a mapping population were considered as target candidates for SPC trait regulation in chickpea (**Table 2**).

### Validation of SPC-Associated Genes by Expression Profiling

**FIGURE 5:**
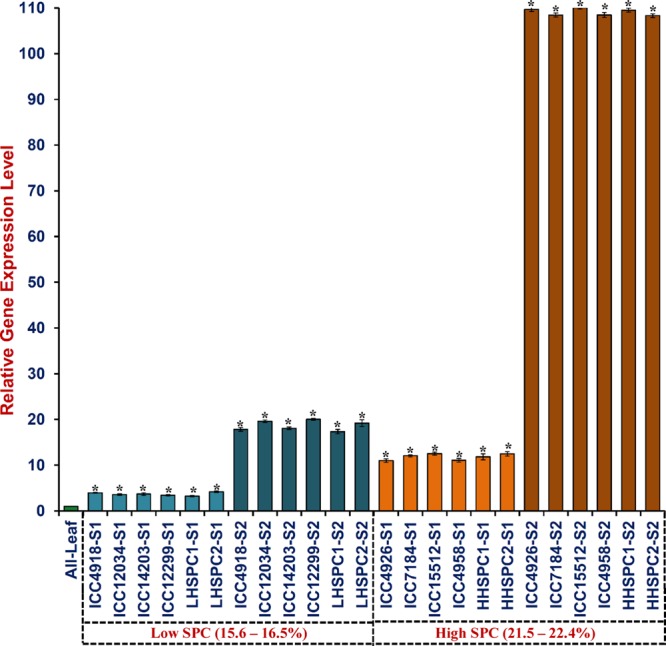
**Differential expression profile of a SPC-associated non-synonymous SNP-carrying zinc finger transcription factor gene in the leaves (green colored) as well as early cell division phase and late seed maturation developmental stages of low and high SPC-containing chickpea accessions, parents, and homozygous RIL individuals of an intra-specific mapping population (ICC 12299 × ICC 4958).** The endogenous control *elongation factor-1 alpha* was used to normalize the expression value across different tissues/developmental stages of accessions, parents, and mapping individuals. The gene expression in leaf tissues (indicated as All-Leaf) of all the accessions/parents and mapping individuals was considered as reference calibrator (assigned 1). Each bars denote the mean (±SE) of three independent biological replicates with two technical replicates for each sample used in quantitative RT-PCR assay. LHSPC, Low homozygous seed protein containing mapping individual; HHSPC, High homozygous seed protein containing mapping individual; S1, seed development stage 1 (10–20 DAP); and S2, seed development stage 2 (21–30 DAP). The level of gene expression in S1 and S2 developmental stages of low SPC-containing accessions are depicted by light and dark blue-colored bars, respectively. The level of gene expression in S1 and S2 developmental stages of high SPC-containing accessions are illustrated by light and dark brown-colored bars, respectively. ^∗^Significant difference in expression of genes at two seed developmental stages of low and high SPC-containing accessions, parents and RIL mapping individuals as compared to leaf at *p* < 0.01 (LSD-ANOVA significance test).

The SPC-associated six SNPs-carrying genes (detected by GWAS), including five genes validated by selective genotyping in a mapping population were used for differential expression analysis to infer the functional regulatory pattern of these genes controlling SPC in chickpea. The differential expression profiling of SPC-associated genes in the leaves as well as early cell division phase and late seed maturation developmental stages of eight chickpea accessions with contrasting levels of low (ICC 4918, IC 12034, ICC 14203, and ICC 12299 with 15.6–16.5%) and high (ICC 4926, ICC 7184, ICC 15512, and ICC 4958 with 21.5–22.4%) SPC content was performed. This analysis was further extended to low and high SPC-containing parental accessions and four homozygous RIL individuals of a mapping population (ICC 12299 × ICC 4958). The SPC-associated six SNPs-containing genes (including five genes validated by selective genotyping in a mapping population) exhibited seed-specific expression (>three fold, *P* ≤ 0.0001) as compared to the leaves of low and high SPC-containing accessions/mapping individuals (Supplementary Figure S1, **Figure 5**). Interestingly, all six seed-specific genes revealed higher differential up-regulation (>four fold, *P* ≤ 0.0001) in two seed developmental stages of high (21.5–22.4%) SPC-containing accessions/mapping individuals than that of low (15.6–16.5%) SPC-containing accessions/mapping individuals (Supplementary Figure S1, **Figure 5**). Remarkably, one non-synonymous SNP-containing zinc finger transcription factor gene showing strong association (*R*^2^: 20%) with SPC based on GWAS exhibited pronounced up-regulation (>five fold, *P* ≤ 0.00001) in two seed developmental stages of high SPC-containing accessions/mapping individuals than that of low SPC-containing accessions/mapping individuals of chickpea (Supplementary Figure S1, **Figure 5**). A direct positive correlation between expression level of six genes with seed protein content both at early cell division and late seed maturation developmental stages of chickpea accessions, mapping parents and individuals was evident. Altogether, the GWAS, selective genotyping in bi-parental RIL mapping population and differential expression profiling in the present study delineated natural functional SNP allelic variants in the six potential novel candidate genes regulating SPC in chickpea (**Table 2**).

## Discussion

The integrated genomics-assisted breeding strategy (combining association and QTL mapping with differential gene expression profiling) is found to be efficient for rapid delineation of potential candidate genes and alleles regulating complex yield component quantitative traits in chickpea (Kujur et al., 2013, 2014, 2015a,b,c; Saxena et al., 2014a; Bajaj et al., 2015b,c). The clues obtained from these studies have essentially encouraged us to utilize this integrated genomic approach for quantitative dissection of complex seed protein content trait in chickpea. In present investigation, GWAS was integrated with selective genotyping in RIL mapping population and differential gene expression profiling to scale-down the natural allelic variants of candidate genes regulating SPC in chickpea. Primarily, we utilized a well-established GBS assay for simultaneous large-scale discovery and genotyping of genome-wide SNPs in chickpea (Deokar et al., 2014; Jaganathan et al., 2015; Kujur et al., 2015a,b,c). The high-throughput sequencing and genotyping of 92 *desi* and *kabuli* accessions (association panel) by GBS approach and subsequent genotyping of these mined genic SNPs in 244 *desi* and *kabuli* accessions by Illumina GoldenGate assay altogether discovered 16376 reference *kabuli* genome-based SNPs for performing GWAS of SPC trait in chickpea. These genome and gene-derived SNPs thus have potential to be utilized for various genomics-assisted breeding applications, including association and genetic mapping to identify potential genes/QTLs governing traits of agronomic importance in chickpea.

The observed wider phenotypic variation and normal frequency distribution of SPC among 336, including 92 and 244 *desi* and *kabuli* accessions reflect the complex quantitative genetic inheritance pattern of target trait in chickpea. Considering its efficacy for quantitative dissection of complex traits in diverse crop plants (Huang et al., 2010, 2012; Chen et al., 2014; Peng et al., 2014; Thudi et al., 2014; Yang et al., 2014; Kujur et al., 2015a; Kumar et al., 2015), the GWAS strategy can be deployed for understanding the complex genetic architecture of SPC trait in chickpea. The use of 16376 genome-wide GBS-SNP genotyping information scanned from a structured population of 336 accessions (with 150–200 kb chromosomal LD decay) in CMLM-based GWAS, identified seven genomic loci significantly associated with SPC trait in chickpea. The LD decay estimated for 336 accessions using 14115 chromosome-wise physically mapped SNPs in the present investigation is comparable with that documented by previous association mapping studies of complex yield component traits in chickpea (Bajaj et al., 2015a; Kujur et al., 2015a,b). This indicates that the SNP density required for effective GWAS is adequate to identify the valid potential genomic loci governing SPC trait in a larger chickpea genome with narrow genetic base. The added-advantage of computationally efficient CMLM model-based strategy adopted in our study for GWAS over other association model-based approaches as documented hitherto for rapid genome-wide scanning of non-spurious SNP marker-trait association (maximal statistical power and high prediction) is well-demonstrated in crop plants (Lipka et al., 2012; Stanton-Geddes et al., 2013; Saxena et al., 2014a; Thudi et al., 2014; Kumar et al., 2015). The SPC-associated seven SNP loci being derived from diverse non-synonymous coding sequence components of six candidate genes are assumed to be functionally relevant. The non-synonymous substitutions of SNP loci in the CDS (coding sequence) of genes encoding variable amino acid residues might create altered secondary structure of proteins that possibly affects the DNA binding and transcriptional activity of target genes associated with multiple agronomic traits in crop plants. Such possible transcriptional mechanism of trait regulation due to non-synonymous SNP substitutions has already been demonstrated in one of the high seed weight-associated transcription factor gene in chickpea (Kujur et al., 2013) as well as grain size (Mao et al., 2010) and stress-responsive (Parida et al., 2012) genes in rice. Irrespective of any genetic backgrounds (*desi*/*kabuli*) in chickpea, we observed a similar level of association potential for seven genomic loci with SPC trait. This could be due to a comparable level of SPC phenotypic trait variation observed in *desi* and *kabuli* cultivar groups. Regardless of the genetic backgrounds, robust genomic loci (genes) especially the non-synonymous genic SNP allelic variants identified through GWAS in the present investigation can be effectively utilized for rapidly establishing marker-trait linkages and identification of genes/QTLs controlling SPC traits in chickpea. This in turn will expedite marker-assisted genetic improvement of chickpea for high protein content.

To ascertain the validity and robustness of identified seven SPC-associated candidate genes, the outcome of GWAS was further correlated with that of selective genotyping in mapping population and differential gene expression profiling. The seed-specific up-regulation and transcript accumulation of six SPC-associated genes specifically in early cell division and late maturation seed developmental stages of high SPC-containing accessions and mapping individuals was apparent. This included five genes that were validated by selective genotyping in a RIL mapping population. The seed protein content is a quantitative trait and being regulated by a complex genetic network involving a diverse array of genes in crop plants (Frimpong et al., 2009; Jadhav et al., 2015). In the present study, a combinatorial strategy involving GWAS, selective genotyping in mapping population and differential gene expression profiling helped us to narrow-down SNP allelic variants in six potential genes (encode ATP-dependent RNA helicase DEAD-box, cystathionine-beta synthase, ABC transporter transmembrane domain, CMP, and dCMP deaminases, and G10 and zinc finger protein) regulating SPC trait in chickpea.

Notably, one strong SPC-associated non-synonymous SNP-containing zinc finger transcription factor gene identified (integrating GWAS, selective genotyping in mapping population and differential expression profiling) in our study is known to be predominantly expressed in seed embryos and also involved in regulating seed storage protein content by accumulation of glutelins during seed development of rice (Chen et al., 2014). In maize, this zinc finger protein is known to bind prolamin box that are conserved across cereals and play a vital role in cereal storage protein gene expression and transcript accumulation (Vicente-Carbajosa et al., 1997). Such non-synonymous SNPs is known to be involved in amino acid substitutions and alteration of secondary structure of proteins and thus possibly affect the DNA binding and transcriptional activity of target genes regulating diverse complex yield component quantitative traits in chickpea (Kujur et al., 2013, 2014, 2015b). In this context, non-synonymous SNP identified in a zinc finger protein-coding gene regulating seed protein content by employing an integrated genomic approach is functionally relevant and thus could be utilized for genetic dissection of this complex trait in chickpea. A SPC-associated ATP-dependent RNA helicase DEAD-box protein-coding gene identified in chickpea regulates ribosomal biogenesis as well as protein synthesis in multiple crop plants (Xu et al., 2015). Another SPC-associated ABC transporter gene detected in chickpea has a potential involvement in transport and storage of proteins inside the cell and thus plays a crucial role in plant nutrition (Kang et al., 2011). The genes code CMP/dCMP deaminases and G10 proteins associated with SPC trait in chickpea regulate nucleotide metabolism and translational recruitment, respectively, in crop plants (McGrew et al., 1989; Armenta-Medina et al., 2014). The gene code cystathionine-beta synthase is known to be involved in cysteine biosynthesis and methionine metabolism in plant species (Amir, 2008). The elevation of two sulfur-containing proteinogen amino acids (precursors of proteins), methionine and cysteine by altering the cystathionine-beta synthase is essential for improving the protein content, including nutritive value of plant seeds (Hesse et al., 2004; Nguyen et al., 2012). Collectively, the SPC-associated novel molecular tags (markers and alleles) of genes narrowed-down by integrated genomics-assisted breeding approach, essentially function by regulating overall signaling, transport, metabolism, and accumulation of proteins in crop plants. Therefore, these functionally relevant molecular tags can be utilized for marker-assisted genetic enhancement to develop superior cultivars with enriched seed protein content in chickpea.

## Author Contributions

HU and DB conducted experiments and drafted the manuscript. LN, SD, and VK involved in data analysis. HU, CG, and SS helped in constitution of association panel and performed phenotyping. SP and AT conceived and designed the study, guided data analysis and interpretation, participated in drafting and correcting the manuscript critically, and gave the final approval of the version to be published. All authors have read and approved the final manuscript.

## Conflict of Interest Statement

The authors declare that the research was conducted in the absence of any commercial or financial relationships that could be construed as a potential conflict of interest.

## Footnotes

1 http://www.maizegenetics.net

2 http://www.spss.com/statistics

3 http://www.tm4.org/mev.html
