# Supplementary material for: Genome-Wide Scans for Delineation of Candidate Genes Regulating Seed-Protein Content in Chickpea
Source: Front Plant Sci. 2016 Mar 23;7:302. doi: 10.3389/fpls.2016.00302 (PMC4803732; doi:10.3389/fpls.2016.00302)
Supplement: Supplementary file 1 [file Table_1.PDF]

Table S1. Primers used for differential expression profiling of SPC-associated genes in chickpea

**Gene Accession IDs**	**Forward primer sequence (5'-3')**	**Reverse primer sequences (5'-3')**	**Amplified fragment size (bp)**	**Annealing temperature (^{0}C)**
Ca08057	AAAACCAGATGAGCCCCAG	TCCTCGTGTCCCATCTTTTG	79	60
Ca20299	AGTCCCTACGAGCTTAGAAGTC	TGAGACAATGCCTTCTACACG	147	60
Ca18632	AGGAGATGGAAGAGATGGAGG	AGCCAACTACCATGAGAAACC	108	60
Ca05955	GTCCATCAGAGAATTAGGCGG	GCTCTTTTCACCCTGCAATC	93	60
Ca06772	AAGATGAGGGAAGCTGAGAATG	CCGATGGTAGAGGTCAAATACG	117	60
Ca19912	AGGCTGGACAATTTCTGGTAC	CTGAACGACAAAATGCAGCTC	143	60
