# Supplementary material for: Genome-Wide Scans for Delineation of Candidate Genes Regulating Seed-Protein Content in Chickpea
Source: Front Plant Sci. 2016 Mar 23;7:302. doi: 10.3389/fpls.2016.00302 (PMC4803732; doi:10.3389/fpls.2016.00302)
Supplement: Supplementary file 2 [file Table_2.PDF]

**Table S2.** 16376 genome-wide GBS-SNPs utilized for GWAS of seed-protein content in chickpea				
**SNP IDs**	**Cultivars**	**Chromosomes/scaffolds**	**Physical positions (bp)**	**SNPs**
CakSNP1	*Kabuli*	*Ca_Kabuli_Chr01*	523	(G/C)
CakSNP2	*Kabuli*	*Ca_Kabuli_Chr01*	462	(G/C)
CakSNP3	*Kabuli*	*Ca_Kabuli_Chr01*	548	(G/C)
CakSNP4	*Kabuli*	*Ca_Kabuli_Chr01*	578	(G/A)
CakSNP5	*Kabuli*	*Ca_Kabuli_Chr01*	579	(T/C)
CakSNP6	*Kabuli*	*Ca_Kabuli_Chr01*	589	(A/C)
CakSNP7	*Kabuli*	*Ca_Kabuli_Chr01*	627	(C/A)
CakSNP8	*Kabuli*	*Ca_Kabuli_Chr01*	839	(A/T)
CakSNP9	*Kabuli*	*Ca_Kabuli_Chr01*	903	(G/T)
CakSNP10	*Kabuli*	*Ca_Kabuli_Chr01*	927	(C/A)
CakSNP11	*Kabuli*	*Ca_Kabuli_Chr01*	98930	(T/C)
CakSNP12	*Kabuli*	*Ca_Kabuli_Chr01*	98992	(G/T)
CakSNP13	*Kabuli*	*Ca_Kabuli_Chr01*	99242	(G/A)
CakSNP14	*Kabuli*	*Ca_Kabuli_Chr01*	108882	(C/A)
CakSNP15	*Kabuli*	*Ca_Kabuli_Chr01*	108984	(G/A)
CakSNP16	*Kabuli*	*Ca_Kabuli_Chr01*	108979	(T/G)
CakSNP17	*Kabuli*	*Ca_Kabuli_Chr01*	129394	(A/G)
CakSNP18	*Kabuli*	*Ca_Kabuli_Chr01*	129401	(A/G)
CakSNP19	*Kabuli*	*Ca_Kabuli_Chr01*	173669	(C/T)
CakSNP20	*Kabuli*	*Ca_Kabuli_Chr01*	202954	(G/T)
CakSNP21	*Kabuli*	*Ca_Kabuli_Chr01*	208244	(G/A)
CakSNP22	*Kabuli*	*Ca_Kabuli_Chr01*	214979	(T/A)
CakSNP23	*Kabuli*	*Ca_Kabuli_Chr01*	215066	(G/T)
CakSNP24	*Kabuli*	*Ca_Kabuli_Chr01*	215058	(T/C)
CakSNP25	*Kabuli*	*Ca_Kabuli_Chr01*	215053	(G/T)
CakSNP26	*Kabuli*	*Ca_Kabuli_Chr01*	215048	(C/T)
CakSNP27	*Kabuli*	*Ca_Kabuli_Chr01*	215029	(C/A)
CakSNP28	*Kabuli*	*Ca_Kabuli_Chr01*	215027	(T/C)
CakSNP29	*Kabuli*	*Ca_Kabuli_Chr01*	215006	(C/T)
CakSNP30	*Kabuli*	*Ca_Kabuli_Chr01*	215239	(C/G)
CakSNP31	*Kabuli*	*Ca_Kabuli_Chr01*	292962	(T/C)
CakSNP32	*Kabuli*	*Ca_Kabuli_Chr01*	292972	(G/A)
CakSNP33	*Kabuli*	*Ca_Kabuli_Chr01*	292998	(T/C)
CakSNP34	*Kabuli*	*Ca_Kabuli_Chr01*	347620	(A/C)
CakSNP35	*Kabuli*	*Ca_Kabuli_Chr01*	372530	(A/G)
CakSNP36	*Kabuli*	*Ca_Kabuli_Chr01*	406570	(A/G)
CakSNP37	*Kabuli*	*Ca_Kabuli_Chr01*	425149	(T/C)
CakSNP38	*Kabuli*	*Ca_Kabuli_Chr01*	425076	(A/G)
CakSNP39	*Kabuli*	*Ca_Kabuli_Chr01*	435259	(A/G)
CakSNP40	*Kabuli*	*Ca_Kabuli_Chr01*	435274	(G/T)

SNP IDs	Cultivars	Chromosomes/scaffolds	Physical positions (bp)	SNPs
CakSNP41	*Kabuli*	*Ca_Kabuli_Chr01*	435280	(T/C)
CakSNP42	*Kabuli*	*Ca_Kabuli_Chr01*	435297	(G/A)
CakSNP43	*Kabuli*	*Ca_Kabuli_Chr01*	435304	(G/T)
CakSNP44	*Kabuli*	*Ca_Kabuli_Chr01*	435343	(C/A)
CakSNP45	*Kabuli*	*Ca_Kabuli_Chr01*	435338	(T/C)
CakSNP46	*Kabuli*	*Ca_Kabuli_Chr01*	435264	(G/T)
CakSNP47	*Kabuli*	*Ca_Kabuli_Chr01*	441283	(G/A)
CakSNP48	*Kabuli*	*Ca_Kabuli_Chr01*	441286	(C/T)
CakSNP49	*Kabuli*	*Ca_Kabuli_Chr01*	441288	(T/A)
CakSNP50	*Kabuli*	*Ca_Kabuli_Chr01*	441343	(T/A)
CakSNP51	*Kabuli*	*Ca_Kabuli_Chr01*	446409	(T/C)
CakSNP52	*Kabuli*	*Ca_Kabuli_Chr01*	446443	(A/G)
CakSNP53	*Kabuli*	*Ca_Kabuli_Chr01*	446484	(C/T)
CakSNP54	*Kabuli*	*Ca_Kabuli_Chr01*	446558	(G/T)
CakSNP55	*Kabuli*	*Ca_Kabuli_Chr01*	459968	(T/G)
CakSNP56	*Kabuli*	*Ca_Kabuli_Chr01*	464039	(A/C)
CakSNP57	*Kabuli*	*Ca_Kabuli_Chr01*	464040	(G/C)
CakSNP58	*Kabuli*	*Ca_Kabuli_Chr01*	464043	(C/G)
CakSNP59	*Kabuli*	*Ca_Kabuli_Chr01*	471007	(C/T)
CakSNP60	*Kabuli*	*Ca_Kabuli_Chr01*	471037	(C/T)
CakSNP61	*Kabuli*	*Ca_Kabuli_Chr01*	527271	(T/G)
CakSNP62	*Kabuli*	*Ca_Kabuli_Chr01*	527321	(C/T)
CakSNP63	*Kabuli*	*Ca_Kabuli_Chr01*	554581	(G/T)
CakSNP64	*Kabuli*	*Ca_Kabuli_Chr01*	554677	(G/A)
CakSNP65	*Kabuli*	*Ca_Kabuli_Chr01*	571020	(T/G)
CakSNP66	*Kabuli*	*Ca_Kabuli_Chr01*	591609	(T/C)
CakSNP67	*Kabuli*	*Ca_Kabuli_Chr01*	609709	(A/G)
CakSNP68	*Kabuli*	*Ca_Kabuli_Chr01*	609671	(C/T)
CakSNP69	*Kabuli*	*Ca_Kabuli_Chr01*	609669	(C/G)
CakSNP70	*Kabuli*	*Ca_Kabuli_Chr01*	644318	(C/T)
CakSNP71	*Kabuli*	*Ca_Kabuli_Chr01*	644353	(G/A)
CakSNP72	*Kabuli*	*Ca_Kabuli_Chr01*	644359	(T/A)
CakSNP73	*Kabuli*	*Ca_Kabuli_Chr01*	644440	(T/C)
CakSNP74	*Kabuli*	*Ca_Kabuli_Chr01*	644425	(G/A)
CakSNP75	*Kabuli*	*Ca_Kabuli_Chr01*	661882	(A/G)
CakSNP76	*Kabuli*	*Ca_Kabuli_Chr01*	662341	(A/C)
CakSNP77	*Kabuli*	*Ca_Kabuli_Chr01*	662489	(T/A)
CakSNP78	*Kabuli*	*Ca_Kabuli_Chr01*	671420	(C/A)
CakSNP79	*Kabuli*	*Ca_Kabuli_Chr01*	671410	(T/C)
CakSNP80	*Kabuli*	*Ca_Kabuli_Chr01*	671391	(T/C)
CakSNP81	*Kabuli*	*Ca_Kabuli_Chr01*	671389	(A/G)

**SNP IDs**	**Cultivars**	**Chromosomes/scaffolds**	**Physical positions (bp)**	**SNPs**
CakSNP82	*Kabuli*	*Ca_Kabuli_Chr01*	671388	(A/T)
CakSNP83	*Kabuli*	*Ca_Kabuli_Chr01*	690156	(C/A)
CakSNP84	*Kabuli*	*Ca_Kabuli_Chr01*	690431	(G/A)
CakSNP85	*Kabuli*	*Ca_Kabuli_Chr01*	691102	(A/T)
CakSNP86	*Kabuli*	*Ca_Kabuli_Chr01*	695962	(A/C)
CakSNP87	*Kabuli*	*Ca_Kabuli_Chr01*	712110	(T/C)
CakSNP88	*Kabuli*	*Ca_Kabuli_Chr01*	712073	(C/T)
CakSNP89	*Kabuli*	*Ca_Kabuli_Chr01*	741514	(G/T)
CakSNP90	*Kabuli*	*Ca_Kabuli_Chr01*	741535	(C/T)
CakSNP91	*Kabuli*	*Ca_Kabuli_Chr01*	741565	(T/C)
CakSNP92	*Kabuli*	*Ca_Kabuli_Chr01*	741549	(C/T)
CakSNP93	*Kabuli*	*Ca_Kabuli_Chr01*	741980	(C/G)
CakSNP94	*Kabuli*	*Ca_Kabuli_Chr01*	742043	(G/A)
CakSNP95	*Kabuli*	*Ca_Kabuli_Chr01*	742026	(T/G)
CakSNP96	*Kabuli*	*Ca_Kabuli_Chr01*	750693	(C/T)
CakSNP97	*Kabuli*	*Ca_Kabuli_Chr01*	777273	(C/T)
CakSNP98	*Kabuli*	*Ca_Kabuli_Chr01*	777240	(G/A)
CakSNP99	*Kabuli*	*Ca_Kabuli_Chr01*	790004	(A/C)
CakSNP100	*Kabuli*	*Ca_Kabuli_Chr01*	790000	(C/T)
CakSNP101	*Kabuli*	*Ca_Kabuli_Chr01*	790102	(G/T)
CakSNP102	*Kabuli*	*Ca_Kabuli_Chr01*	864205	(T/C)
CakSNP103	*Kabuli*	*Ca_Kabuli_Chr01*	864382	(T/C)
CakSNP104	*Kabuli*	*Ca_Kabuli_Chr01*	864381	(G/A)
CakSNP105	*Kabuli*	*Ca_Kabuli_Chr01*	864351	(T/C)
CakSNP106	*Kabuli*	*Ca_Kabuli_Chr01*	866872	(T/C)
CakSNP107	*Kabuli*	*Ca_Kabuli_Chr01*	872443	(G/T)
CakSNP108	*Kabuli*	*Ca_Kabuli_Chr01*	872452	(C/T)
CakSNP109	*Kabuli*	*Ca_Kabuli_Chr01*	876616	(T/C)
CakSNP110	*Kabuli*	*Ca_Kabuli_Chr01*	876633	(T/A)
CakSNP111	*Kabuli*	*Ca_Kabuli_Chr01*	881543	(C/A)
CakSNP112	*Kabuli*	*Ca_Kabuli_Chr01*	881673	(G/A)
CakSNP113	*Kabuli*	*Ca_Kabuli_Chr01*	881703	(G/T)
CakSNP114	*Kabuli*	*Ca_Kabuli_Chr01*	890949	(G/A)
CakSNP115	*Kabuli*	*Ca_Kabuli_Chr01*	890948	(C/T)
CakSNP116	*Kabuli*	*Ca_Kabuli_Chr01*	910486	(T/A)
CakSNP117	*Kabuli*	*Ca_Kabuli_Chr01*	970315	(A/T)
CakSNP118	*Kabuli*	*Ca_Kabuli_Chr01*	996439	(T/C)
CakSNP119	*Kabuli*	*Ca_Kabuli_Chr01*	1007698	(A/G)
CakSNP120	*Kabuli*	*Ca_Kabuli_Chr01*	1008895	(A/G)
CakSNP121	*Kabuli*	*Ca_Kabuli_Chr01*	1008971	(C/G)
CakSNP122	*Kabuli*	*Ca_Kabuli_Chr01*	1009058	(A/C)

**SNP IDs**	**Cultivars**	**Chromosomes/scaffolds**	**Physical positions (bp)**	**SNPs**
CakSNP123	*Kabuli*	*Ca_Kabuli_Chr01*	1018966	(C/G)
CakSNP124	*Kabuli*	*Ca_Kabuli_Chr01*	1020800	(A/G)
CakSNP125	*Kabuli*	*Ca_Kabuli_Chr01*	1023550	(C/T)
CakSNP126	*Kabuli*	*Ca_Kabuli_Chr01*	1023538	(T/C)
CakSNP127	*Kabuli*	*Ca_Kabuli_Chr01*	1032734	(A/T)
CakSNP128	*Kabuli*	*Ca_Kabuli_Chr01*	1059209	(A/G)
CakSNP129	*Kabuli*	*Ca_Kabuli_Chr01*	1059311	(A/G)
CakSNP130	*Kabuli*	*Ca_Kabuli_Chr01*	1083274	(T/C)
CakSNP131	*Kabuli*	*Ca_Kabuli_Chr01*	1083388	(G/A)
CakSNP132	*Kabuli*	*Ca_Kabuli_Chr01*	1083403	(C/T)
CakSNP133	*Kabuli*	*Ca_Kabuli_Chr01*	1085228	(A/C)
CakSNP134	*Kabuli*	*Ca_Kabuli_Chr01*	1085230	(T/G)
CakSNP135	*Kabuli*	*Ca_Kabuli_Chr01*	1085238	(A/G)
CakSNP136	*Kabuli*	*Ca_Kabuli_Chr01*	1085254	(A/C)
CakSNP137	*Kabuli*	*Ca_Kabuli_Chr01*	1085263	(A/C)
CakSNP138	*Kabuli*	*Ca_Kabuli_Chr01*	1085293	(G/A)
CakSNP139	*Kabuli*	*Ca_Kabuli_Chr01*	1085348	(G/A)
CakSNP140	*Kabuli*	*Ca_Kabuli_Chr01*	1091548	(T/A)
CakSNP141	*Kabuli*	*Ca_Kabuli_Chr01*	1091534	(A/C)
CakSNP142	*Kabuli*	*Ca_Kabuli_Chr01*	1091504	(C/T)
CakSNP143	*Kabuli*	*Ca_Kabuli_Chr01*	1098703	(C/G)
CakSNP144	*Kabuli*	*Ca_Kabuli_Chr01*	1098789	(A/G)
CakSNP145	*Kabuli*	*Ca_Kabuli_Chr01*	1115668	(C/T)
CakSNP146	*Kabuli*	*Ca_Kabuli_Chr01*	1115809	(A/G)
CakSNP147	*Kabuli*	*Ca_Kabuli_Chr01*	1116043	(C/T)
CakSNP148	*Kabuli*	*Ca_Kabuli_Chr01*	1140313	(A/C)
CakSNP149	*Kabuli*	*Ca_Kabuli_Chr01*	1140405	(A/T)
CakSNP150	*Kabuli*	*Ca_Kabuli_Chr01*	1150973	(C/T)
CakSNP151	*Kabuli*	*Ca_Kabuli_Chr01*	1159383	(C/A)
CakSNP152	*Kabuli*	*Ca_Kabuli_Chr01*	1159411	(T/G)
CakSNP153	*Kabuli*	*Ca_Kabuli_Chr01*	1215024	(T/C)
CakSNP154	*Kabuli*	*Ca_Kabuli_Chr01*	1269212	(C/G)
CakSNP155	*Kabuli*	*Ca_Kabuli_Chr01*	1269180	(C/A)
CakSNP156	*Kabuli*	*Ca_Kabuli_Chr01*	1278884	(G/T)
CakSNP157	*Kabuli*	*Ca_Kabuli_Chr01*	1278997	(T/A)
CakSNP158	*Kabuli*	*Ca_Kabuli_Chr01*	1295527	(T/G)
CakSNP159	*Kabuli*	*Ca_Kabuli_Chr01*	1335518	(A/C)
CakSNP160	*Kabuli*	*Ca_Kabuli_Chr01*	1385553	(C/A)
CakSNP161	*Kabuli*	*Ca_Kabuli_Chr01*	1385554	(G/T)
CakSNP162	*Kabuli*	*Ca_Kabuli_Chr01*	1390073	(A/G)
CakSNP163	*Kabuli*	*Ca_Kabuli_Chr01*	1411348	(C/T)

SNP IDs	Cultivars	Chromosomes/scaffolds	Physical positions (bp)	SNPs
CakSNP164	*Kabuli*	*Ca_Kabuli_Chr01*	1411351	(A/C)
CakSNP165	*Kabuli*	*Ca_Kabuli_Chr01*	1411501	(C/T)
CakSNP166	*Kabuli*	*Ca_Kabuli_Chr01*	1411498	(T/A)
CakSNP167	*Kabuli*	*Ca_Kabuli_Chr01*	1411495	(A/G)
CakSNP168	*Kabuli*	*Ca_Kabuli_Chr01*	1411490	(T/C)
CakSNP169	*Kabuli*	*Ca_Kabuli_Chr01*	1428222	(C/G)
CakSNP170	*Kabuli*	*Ca_Kabuli_Chr01*	1428251	(T/G)
CakSNP171	*Kabuli*	*Ca_Kabuli_Chr01*	1428264	(T/G)
CakSNP172	*Kabuli*	*Ca_Kabuli_Chr01*	1434121	(A/G)
CakSNP173	*Kabuli*	*Ca_Kabuli_Chr01*	1434883	(A/G)
CakSNP174	*Kabuli*	*Ca_Kabuli_Chr01*	1434875	(A/G)
CakSNP175	*Kabuli*	*Ca_Kabuli_Chr01*	1449848	(G/A)
CakSNP176	*Kabuli*	*Ca_Kabuli_Chr01*	1449913	(T/G)
CakSNP177	*Kabuli*	*Ca_Kabuli_Chr01*	1449949	(G/A)
CakSNP178	*Kabuli*	*Ca_Kabuli_Chr01*	1449980	(A/G)
CakSNP179	*Kabuli*	*Ca_Kabuli_Chr01*	1461574	(T/A)
CakSNP180	*Kabuli*	*Ca_Kabuli_Chr01*	1461600	(T/C)
CakSNP181	*Kabuli*	*Ca_Kabuli_Chr01*	1467056	(A/G)
CakSNP182	*Kabuli*	*Ca_Kabuli_Chr01*	1527513	(A/G)
CakSNP183	*Kabuli*	*Ca_Kabuli_Chr01*	1528325	(G/A)
CakSNP184	*Kabuli*	*Ca_Kabuli_Chr01*	1533723	(G/A)
CakSNP185	*Kabuli*	*Ca_Kabuli_Chr01*	1546679	(G/C)
CakSNP186	*Kabuli*	*Ca_Kabuli_Chr01*	1599821	(A/G)
CakSNP187	*Kabuli*	*Ca_Kabuli_Chr01*	1654823	(C/T)
CakSNP188	*Kabuli*	*Ca_Kabuli_Chr01*	1654855	(A/C)
CakSNP189	*Kabuli*	*Ca_Kabuli_Chr01*	1655018	(G/A)
CakSNP190	*Kabuli*	*Ca_Kabuli_Chr01*	1655012	(G/T)
CakSNP191	*Kabuli*	*Ca_Kabuli_Chr01*	1693010	(T/A)
CakSNP192	*Kabuli*	*Ca_Kabuli_Chr01*	1693013	(C/T)
CakSNP193	*Kabuli*	*Ca_Kabuli_Chr01*	1693022	(T/C)
CakSNP194	*Kabuli*	*Ca_Kabuli_Chr01*	1693023	(T/C)
CakSNP195	*Kabuli*	*Ca_Kabuli_Chr01*	1693031	(C/A)
CakSNP196	*Kabuli*	*Ca_Kabuli_Chr01*	1738179	(G/C)
CakSNP197	*Kabuli*	*Ca_Kabuli_Chr01*	1772813	(T/C)
CakSNP198	*Kabuli*	*Ca_Kabuli_Chr01*	1782760	(G/A)
CakSNP199	*Kabuli*	*Ca_Kabuli_Chr01*	1782793	(G/T)
CakSNP200	*Kabuli*	*Ca_Kabuli_Chr01*	1782808	(G/C)
CakSNP201	*Kabuli*	*Ca_Kabuli_Chr01*	1782953	(G/A)
CakSNP202	*Kabuli*	*Ca_Kabuli_Chr01*	1782948	(T/C)
CakSNP203	*Kabuli*	*Ca_Kabuli_Chr01*	1782930	(G/A)
CakSNP204	*Kabuli*	*Ca_Kabuli_Chr01*	1782929	(C/T)

SNP IDs	Cultivars	Chromosomes/scaffolds	Physical positions (bp)	SNPs
CakSNP205	*Kabuli*	*Ca_Kabuli_Chr01*	1783207	(G/A)
CakSNP206	*Kabuli*	*Ca_Kabuli_Chr01*	1783277	(G/A)
CakSNP207	*Kabuli*	*Ca_Kabuli_Chr01*	1783280	(A/C)
CakSNP208	*Kabuli*	*Ca_Kabuli_Chr01*	1783342	(A/T)
CakSNP209	*Kabuli*	*Ca_Kabuli_Chr01*	1783923	(C/A)
CakSNP210	*Kabuli*	*Ca_Kabuli_Chr01*	1784006	(T/C)
CakSNP211	*Kabuli*	*Ca_Kabuli_Chr01*	1788958	(T/A)
CakSNP212	*Kabuli*	*Ca_Kabuli_Chr01*	1789087	(T/C)
CakSNP213	*Kabuli*	*Ca_Kabuli_Chr01*	1802992	(C/G)
CakSNP214	*Kabuli*	*Ca_Kabuli_Chr01*	1858073	(C/T)
CakSNP215	*Kabuli*	*Ca_Kabuli_Chr01*	1858078	(T/C)
CakSNP216	*Kabuli*	*Ca_Kabuli_Chr01*	1858084	(C/T)
CakSNP217	*Kabuli*	*Ca_Kabuli_Chr01*	1858132	(T/A)
CakSNP218	*Kabuli*	*Ca_Kabuli_Chr01*	1858156	(C/T)
CakSNP219	*Kabuli*	*Ca_Kabuli_Chr01*	1858177	(T/C)
CakSNP220	*Kabuli*	*Ca_Kabuli_Chr01*	1858159	(A/G)
CakSNP221	*Kabuli*	*Ca_Kabuli_Chr01*	1858509	(C/T)
CakSNP222	*Kabuli*	*Ca_Kabuli_Chr01*	1858608	(T/A)
CakSNP223	*Kabuli*	*Ca_Kabuli_Chr01*	1858600	(G/A)
CakSNP224	*Kabuli*	*Ca_Kabuli_Chr01*	1859789	(C/T)
CakSNP225	*Kabuli*	*Ca_Kabuli_Chr01*	1859857	(A/G)
CakSNP226	*Kabuli*	*Ca_Kabuli_Chr01*	1859914	(G/A)
CakSNP227	*Kabuli*	*Ca_Kabuli_Chr01*	1859902	(G/A)
CakSNP228	*Kabuli*	*Ca_Kabuli_Chr01*	1861696	(C/T)
CakSNP229	*Kabuli*	*Ca_Kabuli_Chr01*	1861707	(T/G)
CakSNP230	*Kabuli*	*Ca_Kabuli_Chr01*	1861722	(T/A)
CakSNP231	*Kabuli*	*Ca_Kabuli_Chr01*	1861729	(T/C)
CakSNP232	*Kabuli*	*Ca_Kabuli_Chr01*	1861837	(T/G)
CakSNP233	*Kabuli*	*Ca_Kabuli_Chr01*	1904233	(T/C)
CakSNP234	*Kabuli*	*Ca_Kabuli_Chr01*	1904230	(G/T)
CakSNP235	*Kabuli*	*Ca_Kabuli_Chr01*	1904365	(G/A)
CakSNP236	*Kabuli*	*Ca_Kabuli_Chr01*	1904391	(A/G)
CakSNP237	*Kabuli*	*Ca_Kabuli_Chr01*	1935729	(A/G)
CakSNP238	*Kabuli*	*Ca_Kabuli_Chr01*	1962876	(A/G)
CakSNP239	*Kabuli*	*Ca_Kabuli_Chr01*	1968828	(G/A)
CakSNP240	*Kabuli*	*Ca_Kabuli_Chr01*	2002844	(G/A)
CakSNP241	*Kabuli*	*Ca_Kabuli_Chr01*	2002922	(G/T)
CakSNP242	*Kabuli*	*Ca_Kabuli_Chr01*	2040943	(C/T)
CakSNP243	*Kabuli*	*Ca_Kabuli_Chr01*	2040946	(G/T)
CakSNP244	*Kabuli*	*Ca_Kabuli_Chr01*	2041027	(C/G)
CakSNP245	*Kabuli*	*Ca_Kabuli_Chr01*	2041190	(A/C)

SNP IDs	Cultivars	Chromosomes/scaffolds	Physical positions (bp)	SNPs
CakSNP246	*Kabuli*	*Ca_Kabuli_Chr01*	2041180	(T/C)
CakSNP247	*Kabuli*	*Ca_Kabuli_Chr01*	2041178	(A/C)
CakSNP248	*Kabuli*	*Ca_Kabuli_Chr01*	2041174	(C/T)
CakSNP249	*Kabuli*	*Ca_Kabuli_Chr01*	2041154	(G/C)
CakSNP250	*Kabuli*	*Ca_Kabuli_Chr01*	2041132	(C/T)
CakSNP251	*Kabuli*	*Ca_Kabuli_Chr01*	2042071	(C/G)
CakSNP252	*Kabuli*	*Ca_Kabuli_Chr01*	2042185	(T/C)
CakSNP253	*Kabuli*	*Ca_Kabuli_Chr01*	2042152	(G/C)
CakSNP254	*Kabuli*	*Ca_Kabuli_Chr01*	2042149	(G/T)
CakSNP255	*Kabuli*	*Ca_Kabuli_Chr01*	2044338	(G/A)
CakSNP256	*Kabuli*	*Ca_Kabuli_Chr01*	2044304	(A/C)
CakSNP257	*Kabuli*	*Ca_Kabuli_Chr01*	2044289	(A/G)
CakSNP258	*Kabuli*	*Ca_Kabuli_Chr01*	2044263	(A/C)
CakSNP259	*Kabuli*	*Ca_Kabuli_Chr01*	2044359	(C/T)
CakSNP260	*Kabuli*	*Ca_Kabuli_Chr01*	2044374	(C/G)
CakSNP261	*Kabuli*	*Ca_Kabuli_Chr01*	2044380	(G/C)
CakSNP262	*Kabuli*	*Ca_Kabuli_Chr01*	2044408	(A/C)
CakSNP263	*Kabuli*	*Ca_Kabuli_Chr01*	2044414	(G/A)
CakSNP264	*Kabuli*	*Ca_Kabuli_Chr01*	2044474	(A/G)
CakSNP265	*Kabuli*	*Ca_Kabuli_Chr01*	2050232	(A/T)
CakSNP266	*Kabuli*	*Ca_Kabuli_Chr01*	2050187	(C/G)
CakSNP267	*Kabuli*	*Ca_Kabuli_Chr01*	2050479	(A/C)
CakSNP268	*Kabuli*	*Ca_Kabuli_Chr01*	2050469	(T/C)
CakSNP269	*Kabuli*	*Ca_Kabuli_Chr01*	2050467	(A/C)
CakSNP270	*Kabuli*	*Ca_Kabuli_Chr01*	2050465	(A/G)
CakSNP271	*Kabuli*	*Ca_Kabuli_Chr01*	2050463	(C/T)
CakSNP272	*Kabuli*	*Ca_Kabuli_Chr01*	2050443	(G/C)
CakSNP273	*Kabuli*	*Ca_Kabuli_Chr01*	2050421	(C/T)
CakSNP274	*Kabuli*	*Ca_Kabuli_Chr01*	2051360	(A/G)
CakSNP275	*Kabuli*	*Ca_Kabuli_Chr01*	2051375	(G/T)
CakSNP276	*Kabuli*	*Ca_Kabuli_Chr01*	2051379	(C/T)
CakSNP277	*Kabuli*	*Ca_Kabuli_Chr01*	2053732	(C/T)
CakSNP278	*Kabuli*	*Ca_Kabuli_Chr01*	2053744	(C/A)
CakSNP279	*Kabuli*	*Ca_Kabuli_Chr01*	2053749	(T/A)
CakSNP280	*Kabuli*	*Ca_Kabuli_Chr01*	2053756	(G/T)
CakSNP281	*Kabuli*	*Ca_Kabuli_Chr01*	2053757	(C/T)
CakSNP282	*Kabuli*	*Ca_Kabuli_Chr01*	2053758	(A/G)
CakSNP283	*Kabuli*	*Ca_Kabuli_Chr01*	2053784	(C/A)
CakSNP284	*Kabuli*	*Ca_Kabuli_Chr01*	2053788	(A/G)
CakSNP285	*Kabuli*	*Ca_Kabuli_Chr01*	2053790	(G/A)
CakSNP286	*Kabuli*	*Ca_Kabuli_Chr01*	2053808	(G/T)

SNP IDs	Cultivars	Chromosomes/scaffolds	Physical positions (bp)	SNPs
CakSNP287	*Kabuli*	*Ca_Kabuli_Chr01*	2053856	(T/G)
CakSNP288	*Kabuli*	*Ca_Kabuli_Chr01*	2053843	(T/A)
CakSNP289	*Kabuli*	*Ca_Kabuli_Chr01*	2053841	(C/A)
CakSNP290	*Kabuli*	*Ca_Kabuli_Chr01*	2053840	(A/C)
CakSNP291	*Kabuli*	*Ca_Kabuli_Chr01*	2053833	(T/G)
CakSNP292	*Kabuli*	*Ca_Kabuli_Chr01*	2053830	(A/G)
CakSNP293	*Kabuli*	*Ca_Kabuli_Chr01*	2053819	(C/A)
CakSNP294	*Kabuli*	*Ca_Kabuli_Chr01*	2053818	(G/C)
CakSNP295	*Kabuli*	*Ca_Kabuli_Chr01*	2071456	(T/A)
CakSNP296	*Kabuli*	*Ca_Kabuli_Chr01*	2071448	(A/T)
CakSNP297	*Kabuli*	*Ca_Kabuli_Chr01*	2071424	(T/A)
CakSNP298	*Kabuli*	*Ca_Kabuli_Chr01*	2071708	(C/G)
CakSNP299	*Kabuli*	*Ca_Kabuli_Chr01*	2071703	(A/C)
CakSNP300	*Kabuli*	*Ca_Kabuli_Chr01*	2071699	(T/C)
CakSNP301	*Kabuli*	*Ca_Kabuli_Chr01*	2071693	(T/C)
CakSNP302	*Kabuli*	*Ca_Kabuli_Chr01*	2071691	(A/C)
CakSNP303	*Kabuli*	*Ca_Kabuli_Chr01*	2071689	(A/G)
CakSNP304	*Kabuli*	*Ca_Kabuli_Chr01*	2071687	(C/T)
CakSNP305	*Kabuli*	*Ca_Kabuli_Chr01*	2071667	(G/C)
CakSNP306	*Kabuli*	*Ca_Kabuli_Chr01*	2071645	(C/T)
CakSNP307	*Kabuli*	*Ca_Kabuli_Chr01*	2072613	(G/A)
CakSNP308	*Kabuli*	*Ca_Kabuli_Chr01*	2072627	(A/T)
CakSNP309	*Kabuli*	*Ca_Kabuli_Chr01*	2074998	(A/C)
CakSNP310	*Kabuli*	*Ca_Kabuli_Chr01*	2075087	(A/T)
CakSNP311	*Kabuli*	*Ca_Kabuli_Chr01*	2075057	(G/T)
CakSNP312	*Kabuli*	*Ca_Kabuli_Chr01*	2075045	(C/A)
CakSNP313	*Kabuli*	*Ca_Kabuli_Chr01*	2075044	(A/G)
CakSNP314	*Kabuli*	*Ca_Kabuli_Chr01*	2075043	(C/A)
CakSNP315	*Kabuli*	*Ca_Kabuli_Chr01*	2075015	(G/A)
CakSNP316	*Kabuli*	*Ca_Kabuli_Chr01*	2075013	(A/G)
CakSNP317	*Kabuli*	*Ca_Kabuli_Chr01*	2075009	(C/A)
CakSNP318	*Kabuli*	*Ca_Kabuli_Chr01*	2082760	(C/G)
CakSNP319	*Kabuli*	*Ca_Kabuli_Chr01*	2082801	(G/A)
CakSNP320	*Kabuli*	*Ca_Kabuli_Chr01*	2083003	(G/C)
CakSNP321	*Kabuli*	*Ca_Kabuli_Chr01*	2083059	(A/T)
CakSNP322	*Kabuli*	*Ca_Kabuli_Chr01*	2126267	(G/A)
CakSNP323	*Kabuli*	*Ca_Kabuli_Chr01*	2148583	(T/G)
CakSNP324	*Kabuli*	*Ca_Kabuli_Chr01*	2170090	(G/C)
CakSNP325	*Kabuli*	*Ca_Kabuli_Chr01*	2170935	(A/C)
CakSNP326	*Kabuli*	*Ca_Kabuli_Chr01*	2172076	(T/C)
CakSNP327	*Kabuli*	*Ca_Kabuli_Chr01*	2172113	(G/A)

SNP IDs	Cultivars	Chromosomes/scaffolds	Physical positions (bp)	SNPs
CakSNP328	*Kabuli*	*Ca_Kabuli_Chr01*	2172143	(T/G)
CakSNP329	*Kabuli*	*Ca_Kabuli_Chr01*	2172149	(G/A)
CakSNP330	*Kabuli*	*Ca_Kabuli_Chr01*	2183544	(A/G)
CakSNP331	*Kabuli*	*Ca_Kabuli_Chr01*	2188472	(T/C)
CakSNP332	*Kabuli*	*Ca_Kabuli_Chr01*	2232724	(A/G)
CakSNP333	*Kabuli*	*Ca_Kabuli_Chr01*	2232764	(C/T)
CakSNP334	*Kabuli*	*Ca_Kabuli_Chr01*	2232779	(A/G)
CakSNP335	*Kabuli*	*Ca_Kabuli_Chr01*	2252270	(C/T)
CakSNP336	*Kabuli*	*Ca_Kabuli_Chr01*	2252320	(G/T)
CakSNP337	*Kabuli*	*Ca_Kabuli_Chr01*	2252328	(C/A)
CakSNP338	*Kabuli*	*Ca_Kabuli_Chr01*	2252342	(C/T)
CakSNP339	*Kabuli*	*Ca_Kabuli_Chr01*	2252343	(G/T)
CakSNP340	*Kabuli*	*Ca_Kabuli_Chr01*	2252463	(T/C)
CakSNP341	*Kabuli*	*Ca_Kabuli_Chr01*	2252401	(A/G)
CakSNP342	*Kabuli*	*Ca_Kabuli_Chr01*	2252496	(C/A)
CakSNP343	*Kabuli*	*Ca_Kabuli_Chr01*	2252513	(C/T)
CakSNP344	*Kabuli*	*Ca_Kabuli_Chr01*	2269319	(T/G)
CakSNP345	*Kabuli*	*Ca_Kabuli_Chr01*	2269489	(A/C)
CakSNP346	*Kabuli*	*Ca_Kabuli_Chr01*	2277459	(G/A)
CakSNP347	*Kabuli*	*Ca_Kabuli_Chr01*	2285879	(G/A)
CakSNP348	*Kabuli*	*Ca_Kabuli_Chr01*	2287408	(A/T)
CakSNP349	*Kabuli*	*Ca_Kabuli_Chr01*	2287478	(A/T)
CakSNP350	*Kabuli*	*Ca_Kabuli_Chr01*	2366413	(T/C)
CakSNP351	*Kabuli*	*Ca_Kabuli_Chr01*	2366409	(T/A)
CakSNP352	*Kabuli*	*Ca_Kabuli_Chr01*	2373166	(A/G)
CakSNP353	*Kabuli*	*Ca_Kabuli_Chr01*	2373862	(G/A)
CakSNP354	*Kabuli*	*Ca_Kabuli_Chr01*	2373910	(C/T)
CakSNP355	*Kabuli*	*Ca_Kabuli_Chr01*	2373954	(G/T)
CakSNP356	*Kabuli*	*Ca_Kabuli_Chr01*	2395842	(A/C)
CakSNP357	*Kabuli*	*Ca_Kabuli_Chr01*	2400799	(T/G)
CakSNP358	*Kabuli*	*Ca_Kabuli_Chr01*	2400892	(C/T)
CakSNP359	*Kabuli*	*Ca_Kabuli_Chr01*	2428531	(A/G)
CakSNP360	*Kabuli*	*Ca_Kabuli_Chr01*	2439915	(A/G)
CakSNP361	*Kabuli*	*Ca_Kabuli_Chr01*	2439917	(G/A)
CakSNP362	*Kabuli*	*Ca_Kabuli_Chr01*	2473309	(T/C)
CakSNP363	*Kabuli*	*Ca_Kabuli_Chr01*	2513476	(T/A)
CakSNP364	*Kabuli*	*Ca_Kabuli_Chr01*	2513516	(T/A)
CakSNP365	*Kabuli*	*Ca_Kabuli_Chr01*	2513535	(T/C)
CakSNP366	*Kabuli*	*Ca_Kabuli_Chr01*	2513737	(T/C)
CakSNP367	*Kabuli*	*Ca_Kabuli_Chr01*	2513860	(G/A)
CakSNP368	*Kabuli*	*Ca_Kabuli_Chr01*	2513854	(G/A)

SNP IDs	Cultivars	Chromosomes/scaffolds	Physical positions (bp)	SNPs
CakSNP369	*Kabuli*	*Ca_Kabuli_Chr01*	2513792	(A/G)
CakSNP370	*Kabuli*	*Ca_Kabuli_Chr01*	2513914	(T/C)
CakSNP371	*Kabuli*	*Ca_Kabuli_Chr01*	2542652	(G/A)
CakSNP372	*Kabuli*	*Ca_Kabuli_Chr01*	2649822	(G/C)
CakSNP373	*Kabuli*	*Ca_Kabuli_Chr01*	2649864	(C/A)
CakSNP374	*Kabuli*	*Ca_Kabuli_Chr01*	2649921	(T/C)
CakSNP375	*Kabuli*	*Ca_Kabuli_Chr01*	2649906	(C/T)
CakSNP376	*Kabuli*	*Ca_Kabuli_Chr01*	2649974	(A/C)
CakSNP377	*Kabuli*	*Ca_Kabuli_Chr01*	2671403	(G/T)
CakSNP378	*Kabuli*	*Ca_Kabuli_Chr01*	2694584	(T/C)
CakSNP379	*Kabuli*	*Ca_Kabuli_Chr01*	2745499	(C/A)
CakSNP380	*Kabuli*	*Ca_Kabuli_Chr01*	2745514	(A/C)
CakSNP381	*Kabuli*	*Ca_Kabuli_Chr01*	2745519	(G/C)
CakSNP382	*Kabuli*	*Ca_Kabuli_Chr01*	2745486	(C/T)
CakSNP383	*Kabuli*	*Ca_Kabuli_Chr01*	2754998	(C/T)
CakSNP384	*Kabuli*	*Ca_Kabuli_Chr01*	2755012	(C/T)
CakSNP385	*Kabuli*	*Ca_Kabuli_Chr01*	2755013	(T/A)
CakSNP386	*Kabuli*	*Ca_Kabuli_Chr01*	2755015	(T/C)
CakSNP387	*Kabuli*	*Ca_Kabuli_Chr01*	2755443	(T/A)
CakSNP388	*Kabuli*	*Ca_Kabuli_Chr01*	2755455	(G/A)
CakSNP389	*Kabuli*	*Ca_Kabuli_Chr01*	2755457	(C/T)
CakSNP390	*Kabuli*	*Ca_Kabuli_Chr01*	2755459	(T/G)
CakSNP391	*Kabuli*	*Ca_Kabuli_Chr01*	2755465	(T/A)
CakSNP392	*Kabuli*	*Ca_Kabuli_Chr01*	2755466	(C/G)
CakSNP393	*Kabuli*	*Ca_Kabuli_Chr01*	2755470	(A/T)
CakSNP394	*Kabuli*	*Ca_Kabuli_Chr01*	2755471	(C/A)
CakSNP395	*Kabuli*	*Ca_Kabuli_Chr01*	2755489	(G/A)
CakSNP396	*Kabuli*	*Ca_Kabuli_Chr01*	2755494	(T/C)
CakSNP397	*Kabuli*	*Ca_Kabuli_Chr01*	2755496	(A/G)
CakSNP398	*Kabuli*	*Ca_Kabuli_Chr01*	2755613	(G/T)
CakSNP399	*Kabuli*	*Ca_Kabuli_Chr01*	2755604	(A/C)
CakSNP400	*Kabuli*	*Ca_Kabuli_Chr01*	2755596	(C/A)
CakSNP401	*Kabuli*	*Ca_Kabuli_Chr01*	2757711	(T/G)
CakSNP402	*Kabuli*	*Ca_Kabuli_Chr01*	2757696	(A/G)
CakSNP403	*Kabuli*	*Ca_Kabuli_Chr01*	2757693	(C/T)
CakSNP404	*Kabuli*	*Ca_Kabuli_Chr01*	2757678	(A/G)
CakSNP405	*Kabuli*	*Ca_Kabuli_Chr01*	2758781	(G/C)
CakSNP406	*Kabuli*	*Ca_Kabuli_Chr01*	2758759	(G/C)
CakSNP407	*Kabuli*	*Ca_Kabuli_Chr01*	2758750	(A/G)
CakSNP408	*Kabuli*	*Ca_Kabuli_Chr01*	2758746	(G/A)
CakSNP409	*Kabuli*	*Ca_Kabuli_Chr01*	2758739	(A/T)

SNP IDs	Cultivars	Chromosomes/scaffolds	Physical positions (bp)	SNPs
CakSNP410	*Kabuli*	*Ca_Kabuli_Chr01*	2758738	(T/G)
CakSNP411	*Kabuli*	*Ca_Kabuli_Chr01*	2758859	(A/T)
CakSNP412	*Kabuli*	*Ca_Kabuli_Chr01*	2758835	(T/A)
CakSNP413	*Kabuli*	*Ca_Kabuli_Chr01*	2758827	(A/T)
CakSNP414	*Kabuli*	*Ca_Kabuli_Chr01*	2787006	(C/T)
CakSNP415	*Kabuli*	*Ca_Kabuli_Chr01*	2786994	(A/C)
CakSNP416	*Kabuli*	*Ca_Kabuli_Chr01*	2786989	(T/C)
CakSNP417	*Kabuli*	*Ca_Kabuli_Chr01*	2786976	(A/C)
CakSNP418	*Kabuli*	*Ca_Kabuli_Chr01*	2786974	(T/C)
CakSNP419	*Kabuli*	*Ca_Kabuli_Chr01*	2786970	(T/A)
CakSNP420	*Kabuli*	*Ca_Kabuli_Chr01*	2807605	(G/C)
CakSNP421	*Kabuli*	*Ca_Kabuli_Chr01*	2807616	(G/A)
CakSNP422	*Kabuli*	*Ca_Kabuli_Chr01*	2808197	(T/C)
CakSNP423	*Kabuli*	*Ca_Kabuli_Chr01*	2808170	(T/G)
CakSNP424	*Kabuli*	*Ca_Kabuli_Chr01*	2832792	(G/A)
CakSNP425	*Kabuli*	*Ca_Kabuli_Chr01*	2832969	(G/A)
CakSNP426	*Kabuli*	*Ca_Kabuli_Chr01*	2832939	(G/T)
CakSNP427	*Kabuli*	*Ca_Kabuli_Chr01*	2833448	(C/A)
CakSNP428	*Kabuli*	*Ca_Kabuli_Chr01*	2855919	(T/G)
CakSNP429	*Kabuli*	*Ca_Kabuli_Chr01*	2855926	(T/A)
CakSNP430	*Kabuli*	*Ca_Kabuli_Chr01*	2855927	(T/A)
CakSNP431	*Kabuli*	*Ca_Kabuli_Chr01*	2855941	(T/G)
CakSNP432	*Kabuli*	*Ca_Kabuli_Chr01*	2855962	(A/G)
CakSNP433	*Kabuli*	*Ca_Kabuli_Chr01*	2855969	(C/T)
CakSNP434	*Kabuli*	*Ca_Kabuli_Chr01*	2856003	(T/C)
CakSNP435	*Kabuli*	*Ca_Kabuli_Chr01*	2856045	(C/T)
CakSNP436	*Kabuli*	*Ca_Kabuli_Chr01*	2856079	(T/G)
CakSNP437	*Kabuli*	*Ca_Kabuli_Chr01*	2856094	(T/G)
CakSNP438	*Kabuli*	*Ca_Kabuli_Chr01*	2856120	(T/G)
CakSNP439	*Kabuli*	*Ca_Kabuli_Chr01*	2856534	(T/A)
CakSNP440	*Kabuli*	*Ca_Kabuli_Chr01*	2858263	(A/G)
CakSNP441	*Kabuli*	*Ca_Kabuli_Chr01*	2858278	(T/A)
CakSNP442	*Kabuli*	*Ca_Kabuli_Chr01*	2858287	(G/T)
CakSNP443	*Kabuli*	*Ca_Kabuli_Chr01*	2858299	(C/A)
CakSNP444	*Kabuli*	*Ca_Kabuli_Chr01*	2868942	(G/A)
CakSNP445	*Kabuli*	*Ca_Kabuli_Chr01*	2881315	(T/C)
CakSNP446	*Kabuli*	*Ca_Kabuli_Chr01*	2881321	(C/A)
CakSNP447	*Kabuli*	*Ca_Kabuli_Chr01*	2881323	(A/T)
CakSNP448	*Kabuli*	*Ca_Kabuli_Chr01*	2881327	(C/T)
CakSNP449	*Kabuli*	*Ca_Kabuli_Chr01*	2881356	(C/A)
CakSNP450	*Kabuli*	*Ca_Kabuli_Chr01*	2881357	(A/T)

SNP IDs	Cultivars	Chromosomes/scaffolds	Physical positions (bp)	SNPs
CakSNP451	*Kabuli*	*Ca_Kabuli_Chr01*	2881422	(T/C)
CakSNP452	*Kabuli*	*Ca_Kabuli_Chr01*	2881389	(G/C)
CakSNP453	*Kabuli*	*Ca_Kabuli_Chr01*	2881386	(G/T)
CakSNP454	*Kabuli*	*Ca_Kabuli_Chr01*	2883625	(T/A)
CakSNP455	*Kabuli*	*Ca_Kabuli_Chr01*	2883634	(T/A)
CakSNP456	*Kabuli*	*Ca_Kabuli_Chr01*	2883635	(A/G)
CakSNP457	*Kabuli*	*Ca_Kabuli_Chr01*	2883660	(C/G)
CakSNP458	*Kabuli*	*Ca_Kabuli_Chr01*	2883666	(G/A)
CakSNP459	*Kabuli*	*Ca_Kabuli_Chr01*	2883684	(G/T)
CakSNP460	*Kabuli*	*Ca_Kabuli_Chr01*	2883717	(C/A)
CakSNP461	*Kabuli*	*Ca_Kabuli_Chr01*	2883716	(A/C)
CakSNP462	*Kabuli*	*Ca_Kabuli_Chr01*	2883709	(A/G)
CakSNP463	*Kabuli*	*Ca_Kabuli_Chr01*	2885508	(T/C)
CakSNP464	*Kabuli*	*Ca_Kabuli_Chr01*	2885525	(T/C)
CakSNP465	*Kabuli*	*Ca_Kabuli_Chr01*	2885531	(C/T)
CakSNP466	*Kabuli*	*Ca_Kabuli_Chr01*	2885626	(A/G)
CakSNP467	*Kabuli*	*Ca_Kabuli_Chr01*	2885673	(G/C)
CakSNP468	*Kabuli*	*Ca_Kabuli_Chr01*	2885892	(T/A)
CakSNP469	*Kabuli*	*Ca_Kabuli_Chr01*	2885906	(C/T)
CakSNP470	*Kabuli*	*Ca_Kabuli_Chr01*	2885923	(C/G)
CakSNP471	*Kabuli*	*Ca_Kabuli_Chr01*	2886025	(A/G)
CakSNP472	*Kabuli*	*Ca_Kabuli_Chr01*	2886020	(G/T)
CakSNP473	*Kabuli*	*Ca_Kabuli_Chr01*	2886015	(G/A)
CakSNP474	*Kabuli*	*Ca_Kabuli_Chr01*	2886005	(G/A)
CakSNP475	*Kabuli*	*Ca_Kabuli_Chr01*	2885997	(G/A)
CakSNP476	*Kabuli*	*Ca_Kabuli_Chr01*	2885988	(C/G)
CakSNP477	*Kabuli*	*Ca_Kabuli_Chr01*	2885987	(T/A)
CakSNP478	*Kabuli*	*Ca_Kabuli_Chr01*	2948211	(C/T)
CakSNP479	*Kabuli*	*Ca_Kabuli_Chr01*	3009417	(C/T)
CakSNP480	*Kabuli*	*Ca_Kabuli_Chr01*	3032061	(C/A)
CakSNP481	*Kabuli*	*Ca_Kabuli_Chr01*	3081723	(G/T)
CakSNP482	*Kabuli*	*Ca_Kabuli_Chr01*	3081851	(T/G)
CakSNP483	*Kabuli*	*Ca_Kabuli_Chr01*	3081850	(C/A)
CakSNP484	*Kabuli*	*Ca_Kabuli_Chr01*	3082280	(G/A)
CakSNP485	*Kabuli*	*Ca_Kabuli_Chr01*	3151007	(G/A)
CakSNP486	*Kabuli*	*Ca_Kabuli_Chr01*	3150986	(T/C)
CakSNP487	*Kabuli*	*Ca_Kabuli_Chr01*	3150964	(C/G)
CakSNP488	*Kabuli*	*Ca_Kabuli_Chr01*	3259114	(G/A)
CakSNP489	*Kabuli*	*Ca_Kabuli_Chr01*	3259117	(G/C)
CakSNP490	*Kabuli*	*Ca_Kabuli_Chr01*	3259177	(G/C)
CakSNP491	*Kabuli*	*Ca_Kabuli_Chr01*	3298327	(C/G)

**SNP IDs**	**Cultivars**	**Chromosomes/scaffolds**	**Physical positions (bp)**	**SNPs**
CakSNP492	*Kabuli*	*Ca_Kabuli_Chr01*	3298783	(T/G)
CakSNP493	*Kabuli*	*Ca_Kabuli_Chr01*	3359148	(C/A)
CakSNP494	*Kabuli*	*Ca_Kabuli_Chr01*	3364952	(A/C)
CakSNP495	*Kabuli*	*Ca_Kabuli_Chr01*	3374375	(G/A)
CakSNP496	*Kabuli*	*Ca_Kabuli_Chr01*	3427555	(A/C)
CakSNP497	*Kabuli*	*Ca_Kabuli_Chr01*	3427649	(A/G)
CakSNP498	*Kabuli*	*Ca_Kabuli_Chr01*	3503059	(C/A)
CakSNP499	*Kabuli*	*Ca_Kabuli_Chr01*	3503153	(G/A)
CakSNP500	*Kabuli*	*Ca_Kabuli_Chr01*	3542263	(A/C)
CakSNP501	*Kabuli*	*Ca_Kabuli_Chr01*	3544948	(A/T)
CakSNP502	*Kabuli*	*Ca_Kabuli_Chr01*	3545011	(G/A)
CakSNP503	*Kabuli*	*Ca_Kabuli_Chr01*	3564342	(G/T)
CakSNP504	*Kabuli*	*Ca_Kabuli_Chr01*	3596241	(C/T)
CakSNP505	*Kabuli*	*Ca_Kabuli_Chr01*	3597842	(G/A)
CakSNP506	*Kabuli*	*Ca_Kabuli_Chr01*	3605582	(G/A)
CakSNP507	*Kabuli*	*Ca_Kabuli_Chr01*	3605583	(C/T)
CakSNP508	*Kabuli*	*Ca_Kabuli_Chr01*	3609580	(T/C)
CakSNP509	*Kabuli*	*Ca_Kabuli_Chr01*	3611231	(C/A)
CakSNP510	*Kabuli*	*Ca_Kabuli_Chr01*	3614966	(A/G)
CakSNP511	*Kabuli*	*Ca_Kabuli_Chr01*	3615037	(G/T)
CakSNP512	*Kabuli*	*Ca_Kabuli_Chr01*	3615064	(T/C)
CakSNP513	*Kabuli*	*Ca_Kabuli_Chr01*	3615052	(C/A)
CakSNP514	*Kabuli*	*Ca_Kabuli_Chr01*	3622757	(C/T)
CakSNP515	*Kabuli*	*Ca_Kabuli_Chr01*	3711498	(T/G)
CakSNP516	*Kabuli*	*Ca_Kabuli_Chr01*	3711525	(T/G)
CakSNP517	*Kabuli*	*Ca_Kabuli_Chr01*	3751865	(G/T)
CakSNP518	*Kabuli*	*Ca_Kabuli_Chr01*	3777223	(C/T)
CakSNP519	*Kabuli*	*Ca_Kabuli_Chr01*	3810931	(T/C)
CakSNP520	*Kabuli*	*Ca_Kabuli_Chr01*	3852035	(A/C)
CakSNP521	*Kabuli*	*Ca_Kabuli_Chr01*	3852476	(A/C)
CakSNP522	*Kabuli*	*Ca_Kabuli_Chr01*	3853408	(T/C)
CakSNP523	*Kabuli*	*Ca_Kabuli_Chr01*	3855229	(G/A)
CakSNP524	*Kabuli*	*Ca_Kabuli_Chr01*	3891319	(T/C)
CakSNP525	*Kabuli*	*Ca_Kabuli_Chr01*	3911547	(G/A)
CakSNP526	*Kabuli*	*Ca_Kabuli_Chr01*	3941250	(C/T)
CakSNP527	*Kabuli*	*Ca_Kabuli_Chr01*	3941399	(T/C)
CakSNP528	*Kabuli*	*Ca_Kabuli_Chr01*	3952819	(G/A)
CakSNP529	*Kabuli*	*Ca_Kabuli_Chr01*	3956031	(T/C)
CakSNP530	*Kabuli*	*Ca_Kabuli_Chr01*	3956028	(C/T)
CakSNP531	*Kabuli*	*Ca_Kabuli_Chr01*	3972671	(G/T)
CakSNP532	*Kabuli*	*Ca_Kabuli_Chr01*	4048854	(G/A)

SNP IDs	Cultivars	Chromosomes/scaffolds	Physical positions (bp)	SNPs
CakSNP533	*Kabuli*	*Ca_Kabuli_Chr01*	4048875	(A/T)
CakSNP534	*Kabuli*	*Ca_Kabuli_Chr01*	4073941	(A/C)
CakSNP535	*Kabuli*	*Ca_Kabuli_Chr01*	4112929	(A/T)
CakSNP536	*Kabuli*	*Ca_Kabuli_Chr01*	4189333	(C/T)
CakSNP537	*Kabuli*	*Ca_Kabuli_Chr01*	4189297	(T/C)
CakSNP538	*Kabuli*	*Ca_Kabuli_Chr01*	4245058	(C/G)
CakSNP539	*Kabuli*	*Ca_Kabuli_Chr01*	4245073	(C/G)
CakSNP540	*Kabuli*	*Ca_Kabuli_Chr01*	4245176	(G/T)
CakSNP541	*Kabuli*	*Ca_Kabuli_Chr01*	4292981	(G/C)
CakSNP542	*Kabuli*	*Ca_Kabuli_Chr01*	4302669	(G/C)
CakSNP543	*Kabuli*	*Ca_Kabuli_Chr01*	4302770	(A/C)
CakSNP544	*Kabuli*	*Ca_Kabuli_Chr01*	4302751	(T/C)
CakSNP545	*Kabuli*	*Ca_Kabuli_Chr01*	4334299	(A/T)
CakSNP546	*Kabuli*	*Ca_Kabuli_Chr01*	4334336	(A/G)
CakSNP547	*Kabuli*	*Ca_Kabuli_Chr01*	4372257	(A/G)
CakSNP548	*Kabuli*	*Ca_Kabuli_Chr01*	4375710	(T/C)
CakSNP549	*Kabuli*	*Ca_Kabuli_Chr01*	4385002	(C/T)
CakSNP550	*Kabuli*	*Ca_Kabuli_Chr01*	4386114	(T/G)
CakSNP551	*Kabuli*	*Ca_Kabuli_Chr01*	4386111	(T/C)
CakSNP552	*Kabuli*	*Ca_Kabuli_Chr01*	4392680	(A/G)
CakSNP553	*Kabuli*	*Ca_Kabuli_Chr01*	4414410	(A/T)
CakSNP554	*Kabuli*	*Ca_Kabuli_Chr01*	4423984	(A/G)
CakSNP555	*Kabuli*	*Ca_Kabuli_Chr01*	4429044	(C/T)
CakSNP556	*Kabuli*	*Ca_Kabuli_Chr01*	4432569	(A/G)
CakSNP557	*Kabuli*	*Ca_Kabuli_Chr01*	4432549	(C/T)
CakSNP558	*Kabuli*	*Ca_Kabuli_Chr01*	4432545	(C/A)
CakSNP559	*Kabuli*	*Ca_Kabuli_Chr01*	4432541	(C/A)
CakSNP560	*Kabuli*	*Ca_Kabuli_Chr01*	4456662	(G/A)
CakSNP561	*Kabuli*	*Ca_Kabuli_Chr01*	4456654	(A/C)
CakSNP562	*Kabuli*	*Ca_Kabuli_Chr01*	4456648	(T/C)
CakSNP563	*Kabuli*	*Ca_Kabuli_Chr01*	4457588	(A/G)
CakSNP564	*Kabuli*	*Ca_Kabuli_Chr01*	4460112	(A/T)
CakSNP565	*Kabuli*	*Ca_Kabuli_Chr01*	4494270	(C/T)
CakSNP566	*Kabuli*	*Ca_Kabuli_Chr01*	4494467	(A/G)
CakSNP567	*Kabuli*	*Ca_Kabuli_Chr01*	4512566	(T/C)
CakSNP568	*Kabuli*	*Ca_Kabuli_Chr01*	4528117	(A/G)
CakSNP569	*Kabuli*	*Ca_Kabuli_Chr01*	4528184	(C/T)
CakSNP570	*Kabuli*	*Ca_Kabuli_Chr01*	4530304	(T/C)
CakSNP571	*Kabuli*	*Ca_Kabuli_Chr01*	4560792	(C/T)
CakSNP572	*Kabuli*	*Ca_Kabuli_Chr01*	4560920	(G/A)
CakSNP573	*Kabuli*	*Ca_Kabuli_Chr01*	4569669	(T/A)

SNP IDs	Cultivars	Chromosomes/scaffolds	Physical positions (bp)	SNPs
CakSNP574	*Kabuli*	*Ca_Kabuli_Chr01*	4569670	(T/A)
CakSNP575	*Kabuli*	*Ca_Kabuli_Chr01*	4569690	(A/G)
CakSNP576	*Kabuli*	*Ca_Kabuli_Chr01*	4571615	(T/C)
CakSNP577	*Kabuli*	*Ca_Kabuli_Chr01*	4571540	(C/G)
CakSNP578	*Kabuli*	*Ca_Kabuli_Chr01*	4571539	(G/A)
CakSNP579	*Kabuli*	*Ca_Kabuli_Chr01*	4576888	(A/G)
CakSNP580	*Kabuli*	*Ca_Kabuli_Chr01*	4578967	(T/G)
CakSNP581	*Kabuli*	*Ca_Kabuli_Chr01*	4578999	(T/A)
CakSNP582	*Kabuli*	*Ca_Kabuli_Chr01*	4582189	(C/T)
CakSNP583	*Kabuli*	*Ca_Kabuli_Chr01*	4626099	(T/G)
CakSNP584	*Kabuli*	*Ca_Kabuli_Chr01*	4626098	(C/G)
CakSNP585	*Kabuli*	*Ca_Kabuli_Chr01*	4631467	(G/A)
CakSNP586	*Kabuli*	*Ca_Kabuli_Chr01*	4632188	(G/A)
CakSNP587	*Kabuli*	*Ca_Kabuli_Chr01*	4632965	(G/A)
CakSNP588	*Kabuli*	*Ca_Kabuli_Chr01*	4634413	(C/T)
CakSNP589	*Kabuli*	*Ca_Kabuli_Chr01*	4635347	(G/A)
CakSNP590	*Kabuli*	*Ca_Kabuli_Chr01*	4635302	(A/C)
CakSNP591	*Kabuli*	*Ca_Kabuli_Chr01*	4662564	(A/G)
CakSNP592	*Kabuli*	*Ca_Kabuli_Chr01*	4672439	(G/A)
CakSNP593	*Kabuli*	*Ca_Kabuli_Chr01*	4715563	(T/C)
CakSNP594	*Kabuli*	*Ca_Kabuli_Chr01*	4780330	(C/T)
CakSNP595	*Kabuli*	*Ca_Kabuli_Chr01*	4780345	(G/T)
CakSNP596	*Kabuli*	*Ca_Kabuli_Chr01*	4781362	(C/T)
CakSNP597	*Kabuli*	*Ca_Kabuli_Chr01*	4781416	(G/A)
CakSNP598	*Kabuli*	*Ca_Kabuli_Chr01*	4781704	(G/A)
CakSNP599	*Kabuli*	*Ca_Kabuli_Chr01*	4808910	(A/T)
CakSNP600	*Kabuli*	*Ca_Kabuli_Chr01*	4878683	(T/C)
CakSNP601	*Kabuli*	*Ca_Kabuli_Chr01*	4878691	(A/C)
CakSNP602	*Kabuli*	*Ca_Kabuli_Chr01*	4878695	(T/C)
CakSNP603	*Kabuli*	*Ca_Kabuli_Chr01*	4878823	(A/G)
CakSNP604	*Kabuli*	*Ca_Kabuli_Chr01*	4903185	(T/A)
CakSNP605	*Kabuli*	*Ca_Kabuli_Chr01*	4903203	(A/G)
CakSNP606	*Kabuli*	*Ca_Kabuli_Chr01*	4903239	(G/A)
CakSNP607	*Kabuli*	*Ca_Kabuli_Chr01*	4906759	(G/C)
CakSNP608	*Kabuli*	*Ca_Kabuli_Chr01*	4906775	(A/T)
CakSNP609	*Kabuli*	*Ca_Kabuli_Chr01*	4906777	(G/T)
CakSNP610	*Kabuli*	*Ca_Kabuli_Chr01*	4906778	(T/G)
CakSNP611	*Kabuli*	*Ca_Kabuli_Chr01*	4906800	(T/G)
CakSNP612	*Kabuli*	*Ca_Kabuli_Chr01*	4906816	(A/G)
CakSNP613	*Kabuli*	*Ca_Kabuli_Chr01*	4906868	(G/C)
CakSNP614	*Kabuli*	*Ca_Kabuli_Chr01*	4906859	(T/C)

SNP IDs	Cultivars	Chromosomes/scaffolds	Physical positions (bp)	SNPs
CakSNP615	*Kabuli*	*Ca_Kabuli_Chr01*	4906854	(A/G)
CakSNP616	*Kabuli*	*Ca_Kabuli_Chr01*	4909155	(T/C)
CakSNP617	*Kabuli*	*Ca_Kabuli_Chr01*	4909286	(T/G)
CakSNP618	*Kabuli*	*Ca_Kabuli_Chr01*	4909280	(T/G)
CakSNP619	*Kabuli*	*Ca_Kabuli_Chr01*	4909271	(A/G)
CakSNP620	*Kabuli*	*Ca_Kabuli_Chr01*	4909268	(C/T)
CakSNP621	*Kabuli*	*Ca_Kabuli_Chr01*	4909261	(G/T)
CakSNP622	*Kabuli*	*Ca_Kabuli_Chr01*	4909255	(G/A)
CakSNP623	*Kabuli*	*Ca_Kabuli_Chr01*	4909253	(A/G)
CakSNP624	*Kabuli*	*Ca_Kabuli_Chr01*	4910497	(A/G)
CakSNP625	*Kabuli*	*Ca_Kabuli_Chr01*	4910494	(C/A)
CakSNP626	*Kabuli*	*Ca_Kabuli_Chr01*	4948740	(A/T)
CakSNP627	*Kabuli*	*Ca_Kabuli_Chr01*	4949578	(A/T)
CakSNP628	*Kabuli*	*Ca_Kabuli_Chr01*	4950457	(G/A)
CakSNP629	*Kabuli*	*Ca_Kabuli_Chr01*	4950726	(G/T)
CakSNP630	*Kabuli*	*Ca_Kabuli_Chr01*	4959562	(A/C)
CakSNP631	*Kabuli*	*Ca_Kabuli_Chr01*	4995240	(T/A)
CakSNP632	*Kabuli*	*Ca_Kabuli_Chr01*	4996404	(A/C)
CakSNP633	*Kabuli*	*Ca_Kabuli_Chr01*	4996459	(T/C)
CakSNP634	*Kabuli*	*Ca_Kabuli_Chr01*	4996476	(G/A)
CakSNP635	*Kabuli*	*Ca_Kabuli_Chr01*	4996536	(C/T)
CakSNP636	*Kabuli*	*Ca_Kabuli_Chr01*	5071210	(A/G)
CakSNP637	*Kabuli*	*Ca_Kabuli_Chr01*	5071195	(T/A)
CakSNP638	*Kabuli*	*Ca_Kabuli_Chr01*	5071177	(A/G)
CakSNP639	*Kabuli*	*Ca_Kabuli_Chr01*	5071158	(G/A)
CakSNP640	*Kabuli*	*Ca_Kabuli_Chr01*	5072022	(C/A)
CakSNP641	*Kabuli*	*Ca_Kabuli_Chr01*	5080342	(A/T)
CakSNP642	*Kabuli*	*Ca_Kabuli_Chr01*	5080358	(G/A)
CakSNP643	*Kabuli*	*Ca_Kabuli_Chr01*	5087428	(T/C)
CakSNP644	*Kabuli*	*Ca_Kabuli_Chr01*	5096676	(A/T)
CakSNP645	*Kabuli*	*Ca_Kabuli_Chr01*	5096650	(C/T)
CakSNP646	*Kabuli*	*Ca_Kabuli_Chr01*	5122663	(A/G)
CakSNP647	*Kabuli*	*Ca_Kabuli_Chr01*	5129940	(G/A)
CakSNP648	*Kabuli*	*Ca_Kabuli_Chr01*	5138013	(A/C)
CakSNP649	*Kabuli*	*Ca_Kabuli_Chr01*	5140801	(C/A)
CakSNP650	*Kabuli*	*Ca_Kabuli_Chr01*	5140837	(A/T)
CakSNP651	*Kabuli*	*Ca_Kabuli_Chr01*	5141009	(G/C)
CakSNP652	*Kabuli*	*Ca_Kabuli_Chr01*	5140944	(C/G)
CakSNP653	*Kabuli*	*Ca_Kabuli_Chr01*	5146658	(C/A)
CakSNP654	*Kabuli*	*Ca_Kabuli_Chr01*	5146667	(T/G)
CakSNP655	*Kabuli*	*Ca_Kabuli_Chr01*	5146696	(T/A)

SNP IDs	Cultivars	Chromosomes/scaffolds	Physical positions (bp)	SNPs
CakSNP656	*Kabuli*	*Ca_Kabuli_Chr01*	5164127	(A/G)
CakSNP657	*Kabuli*	*Ca_Kabuli_Chr01*	5164136	(C/T)
CakSNP658	*Kabuli*	*Ca_Kabuli_Chr01*	5164198	(C/A)
CakSNP659	*Kabuli*	*Ca_Kabuli_Chr01*	5197461	(C/T)
CakSNP660	*Kabuli*	*Ca_Kabuli_Chr01*	5197475	(T/A)
CakSNP661	*Kabuli*	*Ca_Kabuli_Chr01*	5200250	(T/G)
CakSNP662	*Kabuli*	*Ca_Kabuli_Chr01*	5200289	(G/T)
CakSNP663	*Kabuli*	*Ca_Kabuli_Chr01*	5249839	(G/A)
CakSNP664	*Kabuli*	*Ca_Kabuli_Chr01*	5249846	(A/C)
CakSNP665	*Kabuli*	*Ca_Kabuli_Chr01*	5249868	(G/A)
CakSNP666	*Kabuli*	*Ca_Kabuli_Chr01*	5288427	(T/C)
CakSNP667	*Kabuli*	*Ca_Kabuli_Chr01*	5288421	(A/C)
CakSNP668	*Kabuli*	*Ca_Kabuli_Chr01*	5304576	(A/C)
CakSNP669	*Kabuli*	*Ca_Kabuli_Chr01*	5321398	(A/G)
CakSNP670	*Kabuli*	*Ca_Kabuli_Chr01*	5430700	(A/C)
CakSNP671	*Kabuli*	*Ca_Kabuli_Chr01*	5430703	(T/G)
CakSNP672	*Kabuli*	*Ca_Kabuli_Chr01*	5430809	(G/A)
CakSNP673	*Kabuli*	*Ca_Kabuli_Chr01*	5444130	(A/C)
CakSNP674	*Kabuli*	*Ca_Kabuli_Chr01*	5444142	(A/T)
CakSNP675	*Kabuli*	*Ca_Kabuli_Chr01*	5474604	(G/A)
CakSNP676	*Kabuli*	*Ca_Kabuli_Chr01*	5477196	(T/C)
CakSNP677	*Kabuli*	*Ca_Kabuli_Chr01*	5487535	(A/G)
CakSNP678	*Kabuli*	*Ca_Kabuli_Chr01*	5489806	(C/G)
CakSNP679	*Kabuli*	*Ca_Kabuli_Chr01*	5577080	(T/C)
CakSNP680	*Kabuli*	*Ca_Kabuli_Chr01*	5629027	(G/A)
CakSNP681	*Kabuli*	*Ca_Kabuli_Chr01*	5637388	(T/A)
CakSNP682	*Kabuli*	*Ca_Kabuli_Chr01*	5637442	(C/T)
CakSNP683	*Kabuli*	*Ca_Kabuli_Chr01*	5643313	(G/C)
CakSNP684	*Kabuli*	*Ca_Kabuli_Chr01*	5666178	(A/C)
CakSNP685	*Kabuli*	*Ca_Kabuli_Chr01*	5666217	(C/A)
CakSNP686	*Kabuli*	*Ca_Kabuli_Chr01*	5666260	(T/G)
CakSNP687	*Kabuli*	*Ca_Kabuli_Chr01*	5666266	(T/G)
CakSNP688	*Kabuli*	*Ca_Kabuli_Chr01*	5666263	(T/G)
CakSNP689	*Kabuli*	*Ca_Kabuli_Chr01*	5666355	(T/G)
CakSNP690	*Kabuli*	*Ca_Kabuli_Chr01*	5667605	(G/A)
CakSNP691	*Kabuli*	*Ca_Kabuli_Chr01*	5702638	(C/T)
CakSNP692	*Kabuli*	*Ca_Kabuli_Chr01*	5706858	(A/T)
CakSNP693	*Kabuli*	*Ca_Kabuli_Chr01*	5706961	(G/T)
CakSNP694	*Kabuli*	*Ca_Kabuli_Chr01*	5706948	(A/T)
CakSNP695	*Kabuli*	*Ca_Kabuli_Chr01*	5706944	(T/G)
CakSNP696	*Kabuli*	*Ca_Kabuli_Chr01*	5706920	(G/A)

**SNP IDs**	**Cultivars**	**Chromosomes/scaffolds**	**Physical positions (bp)**	**SNPs**
CakSNP697	*Kabuli*	*Ca_Kabuli_Chr01*	5721616	(A/T)
CakSNP698	*Kabuli*	*Ca_Kabuli_Chr01*	5749456	(C/T)
CakSNP699	*Kabuli*	*Ca_Kabuli_Chr01*	5791118	(T/G)
CakSNP700	*Kabuli*	*Ca_Kabuli_Chr01*	5796146	(C/T)
CakSNP701	*Kabuli*	*Ca_Kabuli_Chr01*	5796886	(A/G)
CakSNP702	*Kabuli*	*Ca_Kabuli_Chr01*	5807492	(T/C)
CakSNP703	*Kabuli*	*Ca_Kabuli_Chr01*	5812283	(G/T)
CakSNP704	*Kabuli*	*Ca_Kabuli_Chr01*	5812298	(G/A)
CakSNP705	*Kabuli*	*Ca_Kabuli_Chr01*	5862565	(C/T)
CakSNP706	*Kabuli*	*Ca_Kabuli_Chr01*	5960264	(T/C)
CakSNP707	*Kabuli*	*Ca_Kabuli_Chr01*	5978146	(G/A)
CakSNP708	*Kabuli*	*Ca_Kabuli_Chr01*	5978289	(C/T)
CakSNP709	*Kabuli*	*Ca_Kabuli_Chr01*	5981127	(C/T)
CakSNP710	*Kabuli*	*Ca_Kabuli_Chr01*	5981178	(A/G)
CakSNP711	*Kabuli*	*Ca_Kabuli_Chr01*	6011727	(G/T)
CakSNP712	*Kabuli*	*Ca_Kabuli_Chr01*	6011697	(T/G)
CakSNP713	*Kabuli*	*Ca_Kabuli_Chr01*	6011689	(G/A)
CakSNP714	*Kabuli*	*Ca_Kabuli_Chr01*	6011657	(T/C)
CakSNP715	*Kabuli*	*Ca_Kabuli_Chr01*	6011761	(C/T)
CakSNP716	*Kabuli*	*Ca_Kabuli_Chr01*	6017854	(A/C)
CakSNP717	*Kabuli*	*Ca_Kabuli_Chr01*	6066972	(C/T)
CakSNP718	*Kabuli*	*Ca_Kabuli_Chr01*	6066936	(A/G)
CakSNP719	*Kabuli*	*Ca_Kabuli_Chr01*	6066933	(C/T)
CakSNP720	*Kabuli*	*Ca_Kabuli_Chr01*	6067846	(T/C)
CakSNP721	*Kabuli*	*Ca_Kabuli_Chr01*	6088283	(C/T)
CakSNP722	*Kabuli*	*Ca_Kabuli_Chr01*	6101205	(C/T)
CakSNP723	*Kabuli*	*Ca_Kabuli_Chr01*	6126761	(T/A)
CakSNP724	*Kabuli*	*Ca_Kabuli_Chr01*	6126772	(G/A)
CakSNP725	*Kabuli*	*Ca_Kabuli_Chr01*	6155341	(A/G)
CakSNP726	*Kabuli*	*Ca_Kabuli_Chr01*	6224010	(C/G)
CakSNP727	*Kabuli*	*Ca_Kabuli_Chr01*	6224015	(C/T)
CakSNP728	*Kabuli*	*Ca_Kabuli_Chr01*	6249062	(T/C)
CakSNP729	*Kabuli*	*Ca_Kabuli_Chr01*	6249035	(T/C)
CakSNP730	*Kabuli*	*Ca_Kabuli_Chr01*	6257653	(C/G)
CakSNP731	*Kabuli*	*Ca_Kabuli_Chr01*	6257712	(A/T)
CakSNP732	*Kabuli*	*Ca_Kabuli_Chr01*	6259458	(T/C)
CakSNP733	*Kabuli*	*Ca_Kabuli_Chr01*	6262461	(A/G)
CakSNP734	*Kabuli*	*Ca_Kabuli_Chr01*	6262529	(T/G)
CakSNP735	*Kabuli*	*Ca_Kabuli_Chr01*	6262577	(A/G)
CakSNP736	*Kabuli*	*Ca_Kabuli_Chr01*	6278572	(T/C)
CakSNP737	*Kabuli*	*Ca_Kabuli_Chr01*	6278625	(T/C)

SNP IDs	Cultivars	Chromosomes/scaffolds	Physical positions (bp)	SNPs
CakSNP738	*Kabuli*	*Ca_Kabuli_Chr01*	6279644	(G/T)
CakSNP739	*Kabuli*	*Ca_Kabuli_Chr01*	6296858	(G/A)
CakSNP740	*Kabuli*	*Ca_Kabuli_Chr01*	6296986	(C/A)
CakSNP741	*Kabuli*	*Ca_Kabuli_Chr01*	6296929	(C/T)
CakSNP742	*Kabuli*	*Ca_Kabuli_Chr01*	6315337	(G/T)
CakSNP743	*Kabuli*	*Ca_Kabuli_Chr01*	6315402	(C/T)
CakSNP744	*Kabuli*	*Ca_Kabuli_Chr01*	6315399	(T/G)
CakSNP745	*Kabuli*	*Ca_Kabuli_Chr01*	6315397	(C/G)
CakSNP746	*Kabuli*	*Ca_Kabuli_Chr01*	6328801	(T/A)
CakSNP747	*Kabuli*	*Ca_Kabuli_Chr01*	6328957	(C/T)
CakSNP748	*Kabuli*	*Ca_Kabuli_Chr01*	6328963	(A/G)
CakSNP749	*Kabuli*	*Ca_Kabuli_Chr01*	6329805	(A/G)
CakSNP750	*Kabuli*	*Ca_Kabuli_Chr01*	6329814	(T/A)
CakSNP751	*Kabuli*	*Ca_Kabuli_Chr01*	6329844	(C/T)
CakSNP752	*Kabuli*	*Ca_Kabuli_Chr01*	6329847	(A/G)
CakSNP753	*Kabuli*	*Ca_Kabuli_Chr01*	6332229	(T/C)
CakSNP754	*Kabuli*	*Ca_Kabuli_Chr01*	6347626	(C/T)
CakSNP755	*Kabuli*	*Ca_Kabuli_Chr01*	6347720	(G/A)
CakSNP756	*Kabuli*	*Ca_Kabuli_Chr01*	6381114	(T/G)
CakSNP757	*Kabuli*	*Ca_Kabuli_Chr01*	6381191	(T/C)
CakSNP758	*Kabuli*	*Ca_Kabuli_Chr01*	6390541	(T/A)
CakSNP759	*Kabuli*	*Ca_Kabuli_Chr01*	6390849	(G/A)
CakSNP760	*Kabuli*	*Ca_Kabuli_Chr01*	6391017	(T/C)
CakSNP761	*Kabuli*	*Ca_Kabuli_Chr01*	6433592	(T/G)
CakSNP762	*Kabuli*	*Ca_Kabuli_Chr01*	6433826	(G/A)
CakSNP763	*Kabuli*	*Ca_Kabuli_Chr01*	6433838	(C/T)
CakSNP764	*Kabuli*	*Ca_Kabuli_Chr01*	6433841	(C/T)
CakSNP765	*Kabuli*	*Ca_Kabuli_Chr01*	6433883	(A/G)
CakSNP766	*Kabuli*	*Ca_Kabuli_Chr01*	6433934	(T/A)
CakSNP767	*Kabuli*	*Ca_Kabuli_Chr01*	6433925	(C/T)
CakSNP768	*Kabuli*	*Ca_Kabuli_Chr01*	6478736	(A/G)
CakSNP769	*Kabuli*	*Ca_Kabuli_Chr01*	6478758	(A/G)
CakSNP770	*Kabuli*	*Ca_Kabuli_Chr01*	6486446	(G/C)
CakSNP771	*Kabuli*	*Ca_Kabuli_Chr01*	6490978	(A/T)
CakSNP772	*Kabuli*	*Ca_Kabuli_Chr01*	6491008	(A/T)
CakSNP773	*Kabuli*	*Ca_Kabuli_Chr01*	6491030	(T/G)
CakSNP774	*Kabuli*	*Ca_Kabuli_Chr01*	6491017	(T/C)
CakSNP775	*Kabuli*	*Ca_Kabuli_Chr01*	6521670	(C/T)
CakSNP776	*Kabuli*	*Ca_Kabuli_Chr01*	6521673	(G/A)
CakSNP777	*Kabuli*	*Ca_Kabuli_Chr01*	6521856	(A/G)
CakSNP778	*Kabuli*	*Ca_Kabuli_Chr01*	6521859	(T/A)

SNP IDs	Cultivars	Chromosomes/scaffolds	Physical positions (bp)	SNPs
CakSNP779	*Kabuli*	*Ca_Kabuli_Chr01*	6521970	(C/T)
CakSNP780	*Kabuli*	*Ca_Kabuli_Chr01*	6540056	(C/T)
CakSNP781	*Kabuli*	*Ca_Kabuli_Chr01*	6540170	(G/A)
CakSNP782	*Kabuli*	*Ca_Kabuli_Chr01*	6553107	(A/C)
CakSNP783	*Kabuli*	*Ca_Kabuli_Chr01*	6564716	(C/T)
CakSNP784	*Kabuli*	*Ca_Kabuli_Chr01*	6564789	(C/T)
CakSNP785	*Kabuli*	*Ca_Kabuli_Chr01*	6591506	(G/C)
CakSNP786	*Kabuli*	*Ca_Kabuli_Chr01*	6591518	(G/T)
CakSNP787	*Kabuli*	*Ca_Kabuli_Chr01*	6599917	(C/A)
CakSNP788	*Kabuli*	*Ca_Kabuli_Chr01*	6634482	(G/C)
CakSNP789	*Kabuli*	*Ca_Kabuli_Chr01*	6635404	(C/T)
CakSNP790	*Kabuli*	*Ca_Kabuli_Chr01*	6635401	(T/C)
CakSNP791	*Kabuli*	*Ca_Kabuli_Chr01*	6635366	(T/C)
CakSNP792	*Kabuli*	*Ca_Kabuli_Chr01*	6679253	(A/G)
CakSNP793	*Kabuli*	*Ca_Kabuli_Chr01*	6679329	(A/G)
CakSNP794	*Kabuli*	*Ca_Kabuli_Chr01*	6803364	(G/A)
CakSNP795	*Kabuli*	*Ca_Kabuli_Chr01*	6804031	(C/T)
CakSNP796	*Kabuli*	*Ca_Kabuli_Chr01*	6804110	(A/T)
CakSNP797	*Kabuli*	*Ca_Kabuli_Chr01*	6849437	(C/T)
CakSNP798	*Kabuli*	*Ca_Kabuli_Chr01*	6849512	(G/C)
CakSNP799	*Kabuli*	*Ca_Kabuli_Chr01*	6879110	(G/C)
CakSNP800	*Kabuli*	*Ca_Kabuli_Chr01*	6880666	(A/G)
CakSNP801	*Kabuli*	*Ca_Kabuli_Chr01*	6880837	(A/G)
CakSNP802	*Kabuli*	*Ca_Kabuli_Chr01*	6904278	(A/C)
CakSNP803	*Kabuli*	*Ca_Kabuli_Chr01*	6904313	(A/C)
CakSNP804	*Kabuli*	*Ca_Kabuli_Chr01*	6904338	(C/T)
CakSNP805	*Kabuli*	*Ca_Kabuli_Chr01*	6952382	(G/T)
CakSNP806	*Kabuli*	*Ca_Kabuli_Chr01*	7050825	(G/A)
CakSNP807	*Kabuli*	*Ca_Kabuli_Chr01*	7050958	(C/T)
CakSNP808	*Kabuli*	*Ca_Kabuli_Chr01*	7050953	(C/T)
CakSNP809	*Kabuli*	*Ca_Kabuli_Chr01*	7104985	(G/T)
CakSNP810	*Kabuli*	*Ca_Kabuli_Chr01*	7127843	(A/G)
CakSNP811	*Kabuli*	*Ca_Kabuli_Chr01*	7136677	(T/C)
CakSNP812	*Kabuli*	*Ca_Kabuli_Chr01*	7147651	(T/A)
CakSNP813	*Kabuli*	*Ca_Kabuli_Chr01*	7147643	(A/G)
CakSNP814	*Kabuli*	*Ca_Kabuli_Chr01*	7155344	(A/G)
CakSNP815	*Kabuli*	*Ca_Kabuli_Chr01*	7155302	(A/G)
CakSNP816	*Kabuli*	*Ca_Kabuli_Chr01*	7156354	(G/T)
CakSNP817	*Kabuli*	*Ca_Kabuli_Chr01*	7156355	(A/T)
CakSNP818	*Kabuli*	*Ca_Kabuli_Chr01*	7157057	(A/C)
CakSNP819	*Kabuli*	*Ca_Kabuli_Chr01*	7157112	(T/C)

SNP IDs	Cultivars	Chromosomes/scaffolds	Physical positions (bp)	SNPs
CakSNP820	*Kabuli*	*Ca_Kabuli_Chr01*	7160649	(T/C)
CakSNP821	*Kabuli*	*Ca_Kabuli_Chr01*	7160648	(A/T)
CakSNP822	*Kabuli*	*Ca_Kabuli_Chr01*	7160695	(G/A)
CakSNP823	*Kabuli*	*Ca_Kabuli_Chr01*	7160698	(A/C)
CakSNP824	*Kabuli*	*Ca_Kabuli_Chr01*	7160754	(A/T)
CakSNP825	*Kabuli*	*Ca_Kabuli_Chr01*	7160781	(A/C)
CakSNP826	*Kabuli*	*Ca_Kabuli_Chr01*	7160773	(G/A)
CakSNP827	*Kabuli*	*Ca_Kabuli_Chr01*	7161122	(A/G)
CakSNP828	*Kabuli*	*Ca_Kabuli_Chr01*	7184813	(A/C)
CakSNP829	*Kabuli*	*Ca_Kabuli_Chr01*	7184773	(G/A)
CakSNP830	*Kabuli*	*Ca_Kabuli_Chr01*	7187934	(A/G)
CakSNP831	*Kabuli*	*Ca_Kabuli_Chr01*	7187969	(C/G)
CakSNP832	*Kabuli*	*Ca_Kabuli_Chr01*	7196570	(T/C)
CakSNP833	*Kabuli*	*Ca_Kabuli_Chr01*	7196601	(T/C)
CakSNP834	*Kabuli*	*Ca_Kabuli_Chr01*	7231750	(A/C)
CakSNP835	*Kabuli*	*Ca_Kabuli_Chr01*	7325164	(T/A)
CakSNP836	*Kabuli*	*Ca_Kabuli_Chr01*	7417717	(G/A)
CakSNP837	*Kabuli*	*Ca_Kabuli_Chr01*	7417699	(G/A)
CakSNP838	*Kabuli*	*Ca_Kabuli_Chr01*	7417788	(G/A)
CakSNP839	*Kabuli*	*Ca_Kabuli_Chr01*	7439421	(C/T)
CakSNP840	*Kabuli*	*Ca_Kabuli_Chr01*	7467280	(A/G)
CakSNP841	*Kabuli*	*Ca_Kabuli_Chr01*	7478022	(G/A)
CakSNP842	*Kabuli*	*Ca_Kabuli_Chr01*	7478046	(T/G)
CakSNP843	*Kabuli*	*Ca_Kabuli_Chr01*	7486102	(C/T)
CakSNP844	*Kabuli*	*Ca_Kabuli_Chr01*	7486273	(T/C)
CakSNP845	*Kabuli*	*Ca_Kabuli_Chr01*	7486282	(G/A)
CakSNP846	*Kabuli*	*Ca_Kabuli_Chr01*	7497334	(C/A)
CakSNP847	*Kabuli*	*Ca_Kabuli_Chr01*	7497362	(T/C)
CakSNP848	*Kabuli*	*Ca_Kabuli_Chr01*	7500494	(T/G)
CakSNP849	*Kabuli*	*Ca_Kabuli_Chr01*	7500524	(T/C)
CakSNP850	*Kabuli*	*Ca_Kabuli_Chr01*	7500665	(C/A)
CakSNP851	*Kabuli*	*Ca_Kabuli_Chr01*	7505779	(A/T)
CakSNP852	*Kabuli*	*Ca_Kabuli_Chr01*	7505792	(A/T)
CakSNP853	*Kabuli*	*Ca_Kabuli_Chr01*	7515002	(T/C)
CakSNP854	*Kabuli*	*Ca_Kabuli_Chr01*	7515605	(T/C)
CakSNP855	*Kabuli*	*Ca_Kabuli_Chr01*	7515578	(A/C)
CakSNP856	*Kabuli*	*Ca_Kabuli_Chr01*	7557751	(T/G)
CakSNP857	*Kabuli*	*Ca_Kabuli_Chr01*	7557811	(A/T)
CakSNP858	*Kabuli*	*Ca_Kabuli_Chr01*	7690613	(T/G)
CakSNP859	*Kabuli*	*Ca_Kabuli_Chr01*	7690620	(T/G)
CakSNP860	*Kabuli*	*Ca_Kabuli_Chr01*	7690625	(A/C)

**SNP IDs**	**Cultivars**	**Chromosomes/scaffolds**	**Physical positions (bp)**	**SNPs**
CakSNP861	*Kabuli*	*Ca_Kabuli_Chr01*	7695844	(T/C)
CakSNP862	*Kabuli*	*Ca_Kabuli_Chr01*	7811388	(C/T)
CakSNP863	*Kabuli*	*Ca_Kabuli_Chr01*	7925666	(A/C)
CakSNP864	*Kabuli*	*Ca_Kabuli_Chr01*	7925801	(C/T)
CakSNP865	*Kabuli*	*Ca_Kabuli_Chr01*	7925774	(C/T)
CakSNP866	*Kabuli*	*Ca_Kabuli_Chr01*	7925738	(C/T)
CakSNP867	*Kabuli*	*Ca_Kabuli_Chr01*	7969202	(A/C)
CakSNP868	*Kabuli*	*Ca_Kabuli_Chr01*	7969694	(A/G)
CakSNP869	*Kabuli*	*Ca_Kabuli_Chr01*	7969647	(G/A)
CakSNP870	*Kabuli*	*Ca_Kabuli_Chr01*	8036666	(C/T)
CakSNP871	*Kabuli*	*Ca_Kabuli_Chr01*	8036706	(A/G)
CakSNP872	*Kabuli*	*Ca_Kabuli_Chr01*	8036917	(T/G)
CakSNP873	*Kabuli*	*Ca_Kabuli_Chr01*	8036903	(A/T)
CakSNP874	*Kabuli*	*Ca_Kabuli_Chr01*	8075631	(A/T)
CakSNP875	*Kabuli*	*Ca_Kabuli_Chr01*	8103090	(G/T)
CakSNP876	*Kabuli*	*Ca_Kabuli_Chr01*	8103339	(T/C)
CakSNP877	*Kabuli*	*Ca_Kabuli_Chr01*	8103401	(T/A)
CakSNP878	*Kabuli*	*Ca_Kabuli_Chr01*	8107967	(A/C)
CakSNP879	*Kabuli*	*Ca_Kabuli_Chr01*	8108014	(C/T)
CakSNP880	*Kabuli*	*Ca_Kabuli_Chr01*	8111588	(G/A)
CakSNP881	*Kabuli*	*Ca_Kabuli_Chr01*	8112343	(C/T)
CakSNP882	*Kabuli*	*Ca_Kabuli_Chr01*	8113235	(G/T)
CakSNP883	*Kabuli*	*Ca_Kabuli_Chr01*	8113383	(T/G)
CakSNP884	*Kabuli*	*Ca_Kabuli_Chr01*	8113449	(T/A)
CakSNP885	*Kabuli*	*Ca_Kabuli_Chr01*	8113403	(A/T)
CakSNP886	*Kabuli*	*Ca_Kabuli_Chr01*	8113398	(C/G)
CakSNP887	*Kabuli*	*Ca_Kabuli_Chr01*	8123033	(A/G)
CakSNP888	*Kabuli*	*Ca_Kabuli_Chr01*	8123257	(A/G)
CakSNP889	*Kabuli*	*Ca_Kabuli_Chr01*	8123481	(A/G)
CakSNP890	*Kabuli*	*Ca_Kabuli_Chr01*	8134368	(T/C)
CakSNP891	*Kabuli*	*Ca_Kabuli_Chr01*	8178109	(T/C)
CakSNP892	*Kabuli*	*Ca_Kabuli_Chr01*	8178146	(G/A)
CakSNP893	*Kabuli*	*Ca_Kabuli_Chr01*	8192580	(T/C)
CakSNP894	*Kabuli*	*Ca_Kabuli_Chr01*	8220996	(T/A)
CakSNP895	*Kabuli*	*Ca_Kabuli_Chr01*	8220968	(A/G)
CakSNP896	*Kabuli*	*Ca_Kabuli_Chr01*	8343356	(G/A)
CakSNP897	*Kabuli*	*Ca_Kabuli_Chr01*	8389242	(G/A)
CakSNP898	*Kabuli*	*Ca_Kabuli_Chr01*	8464621	(C/G)
CakSNP899	*Kabuli*	*Ca_Kabuli_Chr01*	8582341	(T/C)
CakSNP900	*Kabuli*	*Ca_Kabuli_Chr01*	8607820	(T/C)
CakSNP901	*Kabuli*	*Ca_Kabuli_Chr01*	8607919	(A/C)

SNP IDs	Cultivars	Chromosomes/scaffolds	Physical positions (bp)	SNPs
CakSNP902	*Kabuli*	*Ca_Kabuli_Chr01*	8633598	(G/A)
CakSNP903	*Kabuli*	*Ca_Kabuli_Chr01*	8665705	(T/G)
CakSNP904	*Kabuli*	*Ca_Kabuli_Chr01*	8697177	(G/T)
CakSNP905	*Kabuli*	*Ca_Kabuli_Chr01*	8698059	(T/C)
CakSNP906	*Kabuli*	*Ca_Kabuli_Chr01*	8735740	(G/T)
CakSNP907	*Kabuli*	*Ca_Kabuli_Chr01*	8735727	(G/C)
CakSNP908	*Kabuli*	*Ca_Kabuli_Chr01*	8736733	(G/A)
CakSNP909	*Kabuli*	*Ca_Kabuli_Chr01*	8736711	(G/T)
CakSNP910	*Kabuli*	*Ca_Kabuli_Chr01*	8737380	(A/G)
CakSNP911	*Kabuli*	*Ca_Kabuli_Chr01*	8738691	(T/G)
CakSNP912	*Kabuli*	*Ca_Kabuli_Chr01*	8738695	(G/C)
CakSNP913	*Kabuli*	*Ca_Kabuli_Chr01*	8767122	(T/A)
CakSNP914	*Kabuli*	*Ca_Kabuli_Chr01*	8767121	(A/T)
CakSNP915	*Kabuli*	*Ca_Kabuli_Chr01*	8799685	(T/G)
CakSNP916	*Kabuli*	*Ca_Kabuli_Chr01*	8799701	(T/C)
CakSNP917	*Kabuli*	*Ca_Kabuli_Chr01*	8799708	(T/C)
CakSNP918	*Kabuli*	*Ca_Kabuli_Chr01*	8799758	(C/G)
CakSNP919	*Kabuli*	*Ca_Kabuli_Chr01*	8809188	(T/A)
CakSNP920	*Kabuli*	*Ca_Kabuli_Chr01*	8809191	(C/T)
CakSNP921	*Kabuli*	*Ca_Kabuli_Chr01*	8809295	(A/T)
CakSNP922	*Kabuli*	*Ca_Kabuli_Chr01*	8809517	(C/G)
CakSNP923	*Kabuli*	*Ca_Kabuli_Chr01*	8822121	(G/T)
CakSNP924	*Kabuli*	*Ca_Kabuli_Chr01*	8822375	(G/A)
CakSNP925	*Kabuli*	*Ca_Kabuli_Chr01*	8822366	(T/A)
CakSNP926	*Kabuli*	*Ca_Kabuli_Chr01*	8824856	(T/C)
CakSNP927	*Kabuli*	*Ca_Kabuli_Chr01*	8852863	(G/T)
CakSNP928	*Kabuli*	*Ca_Kabuli_Chr01*	8852928	(T/A)
CakSNP929	*Kabuli*	*Ca_Kabuli_Chr01*	8852981	(C/A)
CakSNP930	*Kabuli*	*Ca_Kabuli_Chr01*	8852957	(C/A)
CakSNP931	*Kabuli*	*Ca_Kabuli_Chr01*	8852943	(C/A)
CakSNP932	*Kabuli*	*Ca_Kabuli_Chr01*	8853010	(A/G)
CakSNP933	*Kabuli*	*Ca_Kabuli_Chr01*	8854211	(G/T)
CakSNP934	*Kabuli*	*Ca_Kabuli_Chr01*	8891438	(T/G)
CakSNP935	*Kabuli*	*Ca_Kabuli_Chr01*	8891485	(T/G)
CakSNP936	*Kabuli*	*Ca_Kabuli_Chr01*	8891562	(C/G)
CakSNP937	*Kabuli*	*Ca_Kabuli_Chr01*	8891561	(G/A)
CakSNP938	*Kabuli*	*Ca_Kabuli_Chr01*	8893985	(G/A)
CakSNP939	*Kabuli*	*Ca_Kabuli_Chr01*	8901769	(C/A)
CakSNP940	*Kabuli*	*Ca_Kabuli_Chr01*	8901842	(T/A)
CakSNP941	*Kabuli*	*Ca_Kabuli_Chr01*	8941004	(A/C)
CakSNP942	*Kabuli*	*Ca_Kabuli_Chr01*	8941029	(G/A)

SNP IDs	Cultivars	Chromosomes/scaffolds	Physical positions (bp)	SNPs
CakSNP943	*Kabuli*	*Ca_Kabuli_Chr01*	8941031	(G/C)
CakSNP944	*Kabuli*	*Ca_Kabuli_Chr01*	8942137	(T/C)
CakSNP945	*Kabuli*	*Ca_Kabuli_Chr01*	8942131	(T/C)
CakSNP946	*Kabuli*	*Ca_Kabuli_Chr01*	8942198	(A/G)
CakSNP947	*Kabuli*	*Ca_Kabuli_Chr01*	8942179	(C/G)
CakSNP948	*Kabuli*	*Ca_Kabuli_Chr01*	8949723	(G/T)
CakSNP949	*Kabuli*	*Ca_Kabuli_Chr01*	8960630	(T/C)
CakSNP950	*Kabuli*	*Ca_Kabuli_Chr01*	8969321	(C/T)
CakSNP951	*Kabuli*	*Ca_Kabuli_Chr01*	8971718	(C/G)
CakSNP952	*Kabuli*	*Ca_Kabuli_Chr01*	8971776	(A/G)
CakSNP953	*Kabuli*	*Ca_Kabuli_Chr01*	8976169	(A/T)
CakSNP954	*Kabuli*	*Ca_Kabuli_Chr01*	8980112	(C/A)
CakSNP955	*Kabuli*	*Ca_Kabuli_Chr01*	8993300	(T/C)
CakSNP956	*Kabuli*	*Ca_Kabuli_Chr01*	8993329	(A/C)
CakSNP957	*Kabuli*	*Ca_Kabuli_Chr01*	9018113	(A/C)
CakSNP958	*Kabuli*	*Ca_Kabuli_Chr01*	9031417	(G/T)
CakSNP959	*Kabuli*	*Ca_Kabuli_Chr01*	9031825	(G/T)
CakSNP960	*Kabuli*	*Ca_Kabuli_Chr01*	9032126	(G/A)
CakSNP961	*Kabuli*	*Ca_Kabuli_Chr01*	9035744	(G/A)
CakSNP962	*Kabuli*	*Ca_Kabuli_Chr01*	9040419	(T/A)
CakSNP963	*Kabuli*	*Ca_Kabuli_Chr01*	9048675	(A/T)
CakSNP964	*Kabuli*	*Ca_Kabuli_Chr01*	9048682	(T/G)
CakSNP965	*Kabuli*	*Ca_Kabuli_Chr01*	9054490	(G/A)
CakSNP966	*Kabuli*	*Ca_Kabuli_Chr01*	9065933	(T/C)
CakSNP967	*Kabuli*	*Ca_Kabuli_Chr01*	9093812	(C/G)
CakSNP968	*Kabuli*	*Ca_Kabuli_Chr01*	9093964	(T/A)
CakSNP969	*Kabuli*	*Ca_Kabuli_Chr01*	9097885	(A/G)
CakSNP970	*Kabuli*	*Ca_Kabuli_Chr01*	9097881	(C/G)
CakSNP971	*Kabuli*	*Ca_Kabuli_Chr01*	9097879	(C/A)
CakSNP972	*Kabuli*	*Ca_Kabuli_Chr01*	9097878	(A/G)
CakSNP973	*Kabuli*	*Ca_Kabuli_Chr01*	9097877	(C/A)
CakSNP974	*Kabuli*	*Ca_Kabuli_Chr01*	9097869	(G/T)
CakSNP975	*Kabuli*	*Ca_Kabuli_Chr01*	9097947	(C/A)
CakSNP976	*Kabuli*	*Ca_Kabuli_Chr01*	9097949	(A/C)
CakSNP977	*Kabuli*	*Ca_Kabuli_Chr01*	9098003	(C/T)
CakSNP978	*Kabuli*	*Ca_Kabuli_Chr01*	9098051	(A/G)
CakSNP979	*Kabuli*	*Ca_Kabuli_Chr01*	9105432	(C/T)
CakSNP980	*Kabuli*	*Ca_Kabuli_Chr01*	9195312	(G/A)
CakSNP981	*Kabuli*	*Ca_Kabuli_Chr01*	9237766	(T/G)
CakSNP982	*Kabuli*	*Ca_Kabuli_Chr01*	9237813	(G/T)
CakSNP983	*Kabuli*	*Ca_Kabuli_Chr01*	9269888	(T/A)

SNP IDs	Cultivars	Chromosomes/scaffolds	Physical positions (bp)	SNPs
CakSNP984	*Kabuli*	*Ca_Kabuli_Chr01*	9468041	(C/T)
CakSNP985	*Kabuli*	*Ca_Kabuli_Chr01*	9468895	(C/A)
CakSNP986	*Kabuli*	*Ca_Kabuli_Chr01*	9476207	(A/C)
CakSNP987	*Kabuli*	*Ca_Kabuli_Chr01*	9476388	(A/G)
CakSNP988	*Kabuli*	*Ca_Kabuli_Chr01*	9476385	(A/G)
CakSNP989	*Kabuli*	*Ca_Kabuli_Chr01*	9535616	(C/T)
CakSNP990	*Kabuli*	*Ca_Kabuli_Chr01*	9547223	(A/G)
CakSNP991	*Kabuli*	*Ca_Kabuli_Chr01*	9547310	(A/G)
CakSNP992	*Kabuli*	*Ca_Kabuli_Chr01*	9547854	(G/A)
CakSNP993	*Kabuli*	*Ca_Kabuli_Chr01*	9547834	(C/T)
CakSNP994	*Kabuli*	*Ca_Kabuli_Chr01*	9547819	(T/C)
CakSNP995	*Kabuli*	*Ca_Kabuli_Chr01*	9556418	(C/G)
CakSNP996	*Kabuli*	*Ca_Kabuli_Chr01*	9556486	(T/G)
CakSNP997	*Kabuli*	*Ca_Kabuli_Chr01*	9556487	(T/A)
CakSNP998	*Kabuli*	*Ca_Kabuli_Chr01*	9556488	(G/A)
CakSNP999	*Kabuli*	*Ca_Kabuli_Chr01*	9556588	(G/T)
CakSNP1000	*Kabuli*	*Ca_Kabuli_Chr01*	9556604	(G/C)
CakSNP1001	*Kabuli*	*Ca_Kabuli_Chr01*	9556628	(G/A)
CakSNP1002	*Kabuli*	*Ca_Kabuli_Chr01*	9556735	(G/A)
CakSNP1003	*Kabuli*	*Ca_Kabuli_Chr01*	9556662	(C/T)
CakSNP1004	*Kabuli*	*Ca_Kabuli_Chr01*	9567049	(C/G)
CakSNP1005	*Kabuli*	*Ca_Kabuli_Chr01*	9603896	(T/A)
CakSNP1006	*Kabuli*	*Ca_Kabuli_Chr01*	9634823	(C/T)
CakSNP1007	*Kabuli*	*Ca_Kabuli_Chr01*	9647861	(G/C)
CakSNP1008	*Kabuli*	*Ca_Kabuli_Chr01*	9659316	(C/T)
CakSNP1009	*Kabuli*	*Ca_Kabuli_Chr01*	9705162	(A/C)
CakSNP1010	*Kabuli*	*Ca_Kabuli_Chr01*	9706910	(C/T)
CakSNP1011	*Kabuli*	*Ca_Kabuli_Chr01*	9737032	(C/T)
CakSNP1012	*Kabuli*	*Ca_Kabuli_Chr01*	9737067	(T/C)
CakSNP1013	*Kabuli*	*Ca_Kabuli_Chr01*	9757006	(G/A)
CakSNP1014	*Kabuli*	*Ca_Kabuli_Chr01*	9779398	(G/A)
CakSNP1015	*Kabuli*	*Ca_Kabuli_Chr01*	9797342	(G/T)
CakSNP1016	*Kabuli*	*Ca_Kabuli_Chr01*	9810275	(T/C)
CakSNP1017	*Kabuli*	*Ca_Kabuli_Chr01*	9815448	(A/T)
CakSNP1018	*Kabuli*	*Ca_Kabuli_Chr01*	9815508	(G/C)
CakSNP1019	*Kabuli*	*Ca_Kabuli_Chr01*	9836198	(A/C)
CakSNP1020	*Kabuli*	*Ca_Kabuli_Chr01*	9896511	(C/T)
CakSNP1021	*Kabuli*	*Ca_Kabuli_Chr01*	9920951	(G/A)
CakSNP1022	*Kabuli*	*Ca_Kabuli_Chr01*	9920986	(A/C)
CakSNP1023	*Kabuli*	*Ca_Kabuli_Chr01*	9932690	(C/G)
CakSNP1024	*Kabuli*	*Ca_Kabuli_Chr01*	10038486	(T/G)

**SNP IDs**	**Cultivars**	**Chromosomes/scaffolds**	**Physical positions (bp)**	**SNPs**
CakSNP1025	*Kabuli*	*Ca_Kabuli_Chr01*	10049364	(A/G)
CakSNP1026	*Kabuli*	*Ca_Kabuli_Chr01*	10049469	(A/G)
CakSNP1027	*Kabuli*	*Ca_Kabuli_Chr01*	10142688	(A/G)
CakSNP1028	*Kabuli*	*Ca_Kabuli_Chr01*	10155496	(T/G)
CakSNP1029	*Kabuli*	*Ca_Kabuli_Chr01*	10155584	(T/C)
CakSNP1030	*Kabuli*	*Ca_Kabuli_Chr01*	10191578	(A/C)
CakSNP1031	*Kabuli*	*Ca_Kabuli_Chr01*	10201074	(C/G)
CakSNP1032	*Kabuli*	*Ca_Kabuli_Chr01*	10212925	(G/C)
CakSNP1033	*Kabuli*	*Ca_Kabuli_Chr01*	10212926	(A/C)
CakSNP1034	*Kabuli*	*Ca_Kabuli_Chr01*	10212948	(T/C)
CakSNP1035	*Kabuli*	*Ca_Kabuli_Chr01*	10212976	(T/A)
CakSNP1036	*Kabuli*	*Ca_Kabuli_Chr01*	10213111	(T/C)
CakSNP1037	*Kabuli*	*Ca_Kabuli_Chr01*	10266774	(C/T)
CakSNP1038	*Kabuli*	*Ca_Kabuli_Chr01*	10289781	(G/A)
CakSNP1039	*Kabuli*	*Ca_Kabuli_Chr01*	10340870	(G/C)
CakSNP1040	*Kabuli*	*Ca_Kabuli_Chr01*	10364986	(C/G)
CakSNP1041	*Kabuli*	*Ca_Kabuli_Chr01*	10365024	(G/A)
CakSNP1042	*Kabuli*	*Ca_Kabuli_Chr01*	10365029	(T/A)
CakSNP1043	*Kabuli*	*Ca_Kabuli_Chr01*	10366383	(G/A)
CakSNP1044	*Kabuli*	*Ca_Kabuli_Chr01*	10377700	(G/C)
CakSNP1045	*Kabuli*	*Ca_Kabuli_Chr01*	10377698	(T/C)
CakSNP1046	*Kabuli*	*Ca_Kabuli_Chr01*	10377691	(T/C)
CakSNP1047	*Kabuli*	*Ca_Kabuli_Chr01*	10377860	(T/A)
CakSNP1048	*Kabuli*	*Ca_Kabuli_Chr01*	10408322	(A/G)
CakSNP1049	*Kabuli*	*Ca_Kabuli_Chr01*	10408349	(G/T)
CakSNP1050	*Kabuli*	*Ca_Kabuli_Chr01*	10410479	(T/C)
CakSNP1051	*Kabuli*	*Ca_Kabuli_Chr01*	10439153	(C/G)
CakSNP1052	*Kabuli*	*Ca_Kabuli_Chr01*	10439177	(T/C)
CakSNP1053	*Kabuli*	*Ca_Kabuli_Chr01*	10439190	(C/A)
CakSNP1054	*Kabuli*	*Ca_Kabuli_Chr01*	10440393	(C/T)
CakSNP1055	*Kabuli*	*Ca_Kabuli_Chr01*	10440430	(G/T)
CakSNP1056	*Kabuli*	*Ca_Kabuli_Chr01*	10440533	(C/A)
CakSNP1057	*Kabuli*	*Ca_Kabuli_Chr01*	10440479	(C/T)
CakSNP1058	*Kabuli*	*Ca_Kabuli_Chr01*	10489197	(A/G)
CakSNP1059	*Kabuli*	*Ca_Kabuli_Chr01*	10527733	(A/G)
CakSNP1060	*Kabuli*	*Ca_Kabuli_Chr01*	10551440	(T/C)
CakSNP1061	*Kabuli*	*Ca_Kabuli_Chr01*	10556610	(T/G)
CakSNP1062	*Kabuli*	*Ca_Kabuli_Chr01*	10560693	(A/G)
CakSNP1063	*Kabuli*	*Ca_Kabuli_Chr01*	10560690	(T/G)
CakSNP1064	*Kabuli*	*Ca_Kabuli_Chr01*	10567303	(A/C)
CakSNP1065	*Kabuli*	*Ca_Kabuli_Chr01*	10571949	(G/A)

SNP IDs	Cultivars	Chromosomes/scaffolds	Physical positions (bp)	SNPs
CakSNP1066	*Kabuli*	*Ca_Kabuli_Chr01*	10601318	(T/G)
CakSNP1067	*Kabuli*	*Ca_Kabuli_Chr01*	10605977	(G/A)
CakSNP1068	*Kabuli*	*Ca_Kabuli_Chr01*	10623359	(T/G)
CakSNP1069	*Kabuli*	*Ca_Kabuli_Chr01*	10630658	(A/T)
CakSNP1070	*Kabuli*	*Ca_Kabuli_Chr01*	10649758	(C/T)
CakSNP1071	*Kabuli*	*Ca_Kabuli_Chr01*	10655633	(G/T)
CakSNP1072	*Kabuli*	*Ca_Kabuli_Chr01*	10657464	(T/A)
CakSNP1073	*Kabuli*	*Ca_Kabuli_Chr01*	10697972	(C/G)
CakSNP1074	*Kabuli*	*Ca_Kabuli_Chr01*	10710672	(T/G)
CakSNP1075	*Kabuli*	*Ca_Kabuli_Chr01*	10712235	(G/A)
CakSNP1076	*Kabuli*	*Ca_Kabuli_Chr01*	10713001	(T/A)
CakSNP1077	*Kabuli*	*Ca_Kabuli_Chr01*	10734511	(G/A)
CakSNP1078	*Kabuli*	*Ca_Kabuli_Chr01*	10734570	(G/A)
CakSNP1079	*Kabuli*	*Ca_Kabuli_Chr01*	10739374	(T/A)
CakSNP1080	*Kabuli*	*Ca_Kabuli_Chr01*	10761609	(G/T)
CakSNP1081	*Kabuli*	*Ca_Kabuli_Chr01*	10761648	(C/T)
CakSNP1082	*Kabuli*	*Ca_Kabuli_Chr01*	10814166	(T/C)
CakSNP1083	*Kabuli*	*Ca_Kabuli_Chr01*	10837203	(C/T)
CakSNP1084	*Kabuli*	*Ca_Kabuli_Chr01*	10861334	(A/G)
CakSNP1085	*Kabuli*	*Ca_Kabuli_Chr01*	10861332	(C/G)
CakSNP1086	*Kabuli*	*Ca_Kabuli_Chr01*	10861326	(T/G)
CakSNP1087	*Kabuli*	*Ca_Kabuli_Chr01*	10869338	(A/T)
CakSNP1088	*Kabuli*	*Ca_Kabuli_Chr01*	10869336	(G/A)
CakSNP1089	*Kabuli*	*Ca_Kabuli_Chr01*	10887927	(C/A)
CakSNP1090	*Kabuli*	*Ca_Kabuli_Chr01*	10939428	(T/C)
CakSNP1091	*Kabuli*	*Ca_Kabuli_Chr01*	10973356	(C/T)
CakSNP1092	*Kabuli*	*Ca_Kabuli_Chr01*	11065411	(T/A)
CakSNP1093	*Kabuli*	*Ca_Kabuli_Chr01*	11103176	(T/C)
CakSNP1094	*Kabuli*	*Ca_Kabuli_Chr01*	11121162	(G/A)
CakSNP1095	*Kabuli*	*Ca_Kabuli_Chr01*	11122665	(G/T)
CakSNP1096	*Kabuli*	*Ca_Kabuli_Chr01*	11191023	(G/A)
CakSNP1097	*Kabuli*	*Ca_Kabuli_Chr01*	11193334	(G/C)
CakSNP1098	*Kabuli*	*Ca_Kabuli_Chr01*	11225828	(T/C)
CakSNP1099	*Kabuli*	*Ca_Kabuli_Chr01*	11227614	(T/C)
CakSNP1100	*Kabuli*	*Ca_Kabuli_Chr01*	11230086	(A/G)
CakSNP1101	*Kabuli*	*Ca_Kabuli_Chr01*	11230109	(T/C)
CakSNP1102	*Kabuli*	*Ca_Kabuli_Chr01*	11230115	(G/C)
CakSNP1103	*Kabuli*	*Ca_Kabuli_Chr01*	11230116	(T/C)
CakSNP1104	*Kabuli*	*Ca_Kabuli_Chr01*	11230118	(A/T)
CakSNP1105	*Kabuli*	*Ca_Kabuli_Chr01*	11230125	(A/C)
CakSNP1106	*Kabuli*	*Ca_Kabuli_Chr01*	11230126	(G/T)

SNP IDs	Cultivars	Chromosomes/scaffolds	Physical positions (bp)	SNPs
CakSNP1107	*Kabuli*	*Ca_Kabuli_Chr01*	11230127	(G/T)
CakSNP1108	*Kabuli*	*Ca_Kabuli_Chr01*	11230128	(T/G)
CakSNP1109	*Kabuli*	*Ca_Kabuli_Chr01*	11230133	(G/A)
CakSNP1110	*Kabuli*	*Ca_Kabuli_Chr01*	11230972	(C/T)
CakSNP1111	*Kabuli*	*Ca_Kabuli_Chr01*	11230998	(A/T)
CakSNP1112	*Kabuli*	*Ca_Kabuli_Chr01*	11231052	(A/G)
CakSNP1113	*Kabuli*	*Ca_Kabuli_Chr01*	11231064	(A/G)
CakSNP1114	*Kabuli*	*Ca_Kabuli_Chr01*	11231077	(T/C)
CakSNP1115	*Kabuli*	*Ca_Kabuli_Chr01*	11235211	(C/T)
CakSNP1116	*Kabuli*	*Ca_Kabuli_Chr01*	11265367	(T/C)
CakSNP1117	*Kabuli*	*Ca_Kabuli_Chr01*	11277703	(T/A)
CakSNP1118	*Kabuli*	*Ca_Kabuli_Chr01*	11277964	(C/T)
CakSNP1119	*Kabuli*	*Ca_Kabuli_Chr01*	11284183	(T/C)
CakSNP1120	*Kabuli*	*Ca_Kabuli_Chr01*	11285192	(G/A)
CakSNP1121	*Kabuli*	*Ca_Kabuli_Chr01*	11285247	(T/C)
CakSNP1122	*Kabuli*	*Ca_Kabuli_Chr01*	11285291	(C/A)
CakSNP1123	*Kabuli*	*Ca_Kabuli_Chr01*	11291516	(G/A)
CakSNP1124	*Kabuli*	*Ca_Kabuli_Chr01*	11291741	(A/C)
CakSNP1125	*Kabuli*	*Ca_Kabuli_Chr01*	11291740	(T/A)
CakSNP1126	*Kabuli*	*Ca_Kabuli_Chr01*	11291737	(C/A)
CakSNP1127	*Kabuli*	*Ca_Kabuli_Chr01*	11319264	(A/C)
CakSNP1128	*Kabuli*	*Ca_Kabuli_Chr01*	11321670	(G/C)
CakSNP1129	*Kabuli*	*Ca_Kabuli_Chr01*	11324018	(C/A)
CakSNP1130	*Kabuli*	*Ca_Kabuli_Chr01*	11324023	(C/T)
CakSNP1131	*Kabuli*	*Ca_Kabuli_Chr01*	11324035	(C/T)
CakSNP1132	*Kabuli*	*Ca_Kabuli_Chr01*	11324050	(G/T)
CakSNP1133	*Kabuli*	*Ca_Kabuli_Chr01*	11342388	(T/C)
CakSNP1134	*Kabuli*	*Ca_Kabuli_Chr01*	11361646	(G/A)
CakSNP1135	*Kabuli*	*Ca_Kabuli_Chr01*	11361588	(T/G)
CakSNP1136	*Kabuli*	*Ca_Kabuli_Chr01*	11361583	(T/C)
CakSNP1137	*Kabuli*	*Ca_Kabuli_Chr01*	11374707	(G/A)
CakSNP1138	*Kabuli*	*Ca_Kabuli_Chr01*	11405015	(A/G)
CakSNP1139	*Kabuli*	*Ca_Kabuli_Chr01*	11405118	(G/A)
CakSNP1140	*Kabuli*	*Ca_Kabuli_Chr01*	11423677	(C/A)
CakSNP1141	*Kabuli*	*Ca_Kabuli_Chr01*	11487689	(A/G)
CakSNP1142	*Kabuli*	*Ca_Kabuli_Chr01*	11487630	(C/A)
CakSNP1143	*Kabuli*	*Ca_Kabuli_Chr01*	11487752	(G/T)
CakSNP1144	*Kabuli*	*Ca_Kabuli_Chr01*	11502148	(C/A)
CakSNP1145	*Kabuli*	*Ca_Kabuli_Chr01*	11514888	(T/A)
CakSNP1146	*Kabuli*	*Ca_Kabuli_Chr01*	11514898	(T/C)
CakSNP1147	*Kabuli*	*Ca_Kabuli_Chr01*	11514903	(A/G)

SNP IDs	Cultivars	Chromosomes/scaffolds	Physical positions (bp)	SNPs
CakSNP1148	*Kabuli*	*Ca_Kabuli_Chr01*	11515055	(G/A)
CakSNP1149	*Kabuli*	*Ca_Kabuli_Chr01*	11515130	(T/G)
CakSNP1150	*Kabuli*	*Ca_Kabuli_Chr01*	11522656	(G/T)
CakSNP1151	*Kabuli*	*Ca_Kabuli_Chr01*	11522743	(T/C)
CakSNP1152	*Kabuli*	*Ca_Kabuli_Chr01*	11522875	(C/T)
CakSNP1153	*Kabuli*	*Ca_Kabuli_Chr01*	11601347	(T/C)
CakSNP1154	*Kabuli*	*Ca_Kabuli_Chr01*	11624419	(C/G)
CakSNP1155	*Kabuli*	*Ca_Kabuli_Chr01*	11624464	(C/T)
CakSNP1156	*Kabuli*	*Ca_Kabuli_Chr01*	11669794	(T/G)
CakSNP1157	*Kabuli*	*Ca_Kabuli_Chr01*	11673478	(A/C)
CakSNP1158	*Kabuli*	*Ca_Kabuli_Chr01*	11677097	(C/A)
CakSNP1159	*Kabuli*	*Ca_Kabuli_Chr01*	11685769	(A/G)
CakSNP1160	*Kabuli*	*Ca_Kabuli_Chr01*	11685790	(T/C)
CakSNP1161	*Kabuli*	*Ca_Kabuli_Chr01*	11706451	(C/T)
CakSNP1162	*Kabuli*	*Ca_Kabuli_Chr01*	11706494	(G/C)
CakSNP1163	*Kabuli*	*Ca_Kabuli_Chr01*	11706583	(A/C)
CakSNP1164	*Kabuli*	*Ca_Kabuli_Chr01*	11706545	(C/G)
CakSNP1165	*Kabuli*	*Ca_Kabuli_Chr01*	11741448	(T/A)
CakSNP1166	*Kabuli*	*Ca_Kabuli_Chr01*	11793443	(C/A)
CakSNP1167	*Kabuli*	*Ca_Kabuli_Chr01*	11822248	(A/T)
CakSNP1168	*Kabuli*	*Ca_Kabuli_Chr01*	11822305	(G/T)
CakSNP1169	*Kabuli*	*Ca_Kabuli_Chr01*	11822309	(C/A)
CakSNP1170	*Kabuli*	*Ca_Kabuli_Chr01*	11870401	(A/G)
CakSNP1171	*Kabuli*	*Ca_Kabuli_Chr01*	11935333	(A/T)
CakSNP1172	*Kabuli*	*Ca_Kabuli_Chr01*	11955896	(G/T)
CakSNP1173	*Kabuli*	*Ca_Kabuli_Chr01*	11955866	(G/A)
CakSNP1174	*Kabuli*	*Ca_Kabuli_Chr01*	11955853	(A/T)
CakSNP1175	*Kabuli*	*Ca_Kabuli_Chr01*	12054809	(C/T)
CakSNP1176	*Kabuli*	*Ca_Kabuli_Chr01*	12189822	(T/G)
CakSNP1177	*Kabuli*	*Ca_Kabuli_Chr01*	12189825	(T/G)
CakSNP1178	*Kabuli*	*Ca_Kabuli_Chr01*	12193933	(T/G)
CakSNP1179	*Kabuli*	*Ca_Kabuli_Chr01*	12199691	(C/G)
CakSNP1180	*Kabuli*	*Ca_Kabuli_Chr01*	12236387	(T/A)
CakSNP1181	*Kabuli*	*Ca_Kabuli_Chr01*	12264697	(T/G)
CakSNP1182	*Kabuli*	*Ca_Kabuli_Chr01*	12326230	(A/G)
CakSNP1183	*Kabuli*	*Ca_Kabuli_Chr01*	12326234	(T/A)
CakSNP1184	*Kabuli*	*Ca_Kabuli_Chr01*	12326318	(C/G)
CakSNP1185	*Kabuli*	*Ca_Kabuli_Chr01*	12334259	(C/T)
CakSNP1186	*Kabuli*	*Ca_Kabuli_Chr01*	12340884	(C/T)
CakSNP1187	*Kabuli*	*Ca_Kabuli_Chr01*	12390756	(G/T)
CakSNP1188	*Kabuli*	*Ca_Kabuli_Chr01*	12390750	(T/C)

SNP IDs	Cultivars	Chromosomes/scaffolds	Physical positions (bp)	SNPs
CakSNP1189	*Kabuli*	*Ca_Kabuli_Chr01*	12391116	(G/C)
CakSNP1190	*Kabuli*	*Ca_Kabuli_Chr01*	12391074	(G/A)
CakSNP1191	*Kabuli*	*Ca_Kabuli_Chr01*	12391067	(C/A)
CakSNP1192	*Kabuli*	*Ca_Kabuli_Chr01*	12413012	(G/T)
CakSNP1193	*Kabuli*	*Ca_Kabuli_Chr01*	12413016	(T/A)
CakSNP1194	*Kabuli*	*Ca_Kabuli_Chr01*	12413025	(G/C)
CakSNP1195	*Kabuli*	*Ca_Kabuli_Chr01*	12413116	(G/A)
CakSNP1196	*Kabuli*	*Ca_Kabuli_Chr01*	12414040	(G/A)
CakSNP1197	*Kabuli*	*Ca_Kabuli_Chr01*	12481879	(C/A)
CakSNP1198	*Kabuli*	*Ca_Kabuli_Chr01*	12525445	(A/G)
CakSNP1199	*Kabuli*	*Ca_Kabuli_Chr01*	12525640	(G/C)
CakSNP1200	*Kabuli*	*Ca_Kabuli_Chr01*	12566565	(C/T)
CakSNP1201	*Kabuli*	*Ca_Kabuli_Chr01*	12566579	(C/T)
CakSNP1202	*Kabuli*	*Ca_Kabuli_Chr01*	12566607	(T/C)
CakSNP1203	*Kabuli*	*Ca_Kabuli_Chr01*	12566615	(C/T)
CakSNP1204	*Kabuli*	*Ca_Kabuli_Chr01*	12566641	(T/C)
CakSNP1205	*Kabuli*	*Ca_Kabuli_Chr01*	12588525	(A/G)
CakSNP1206	*Kabuli*	*Ca_Kabuli_Chr01*	12597939	(A/T)
CakSNP1207	*Kabuli*	*Ca_Kabuli_Chr01*	12639552	(T/A)
CakSNP1208	*Kabuli*	*Ca_Kabuli_Chr01*	12639584	(T/C)
CakSNP1209	*Kabuli*	*Ca_Kabuli_Chr01*	12687506	(C/T)
CakSNP1210	*Kabuli*	*Ca_Kabuli_Chr01*	12688424	(A/G)
CakSNP1211	*Kabuli*	*Ca_Kabuli_Chr01*	12688435	(A/C)
CakSNP1212	*Kabuli*	*Ca_Kabuli_Chr01*	12704406	(G/T)
CakSNP1213	*Kabuli*	*Ca_Kabuli_Chr01*	12705665	(C/T)
CakSNP1214	*Kabuli*	*Ca_Kabuli_Chr01*	12705731	(G/A)
CakSNP1215	*Kabuli*	*Ca_Kabuli_Chr01*	12715427	(C/T)
CakSNP1216	*Kabuli*	*Ca_Kabuli_Chr01*	12719734	(T/A)
CakSNP1217	*Kabuli*	*Ca_Kabuli_Chr01*	12719735	(G/T)
CakSNP1218	*Kabuli*	*Ca_Kabuli_Chr01*	12719752	(G/C)
CakSNP1219	*Kabuli*	*Ca_Kabuli_Chr01*	12736298	(A/G)
CakSNP1220	*Kabuli*	*Ca_Kabuli_Chr01*	12736353	(A/C)
CakSNP1221	*Kabuli*	*Ca_Kabuli_Chr01*	12736622	(T/G)
CakSNP1222	*Kabuli*	*Ca_Kabuli_Chr01*	12738564	(C/T)
CakSNP1223	*Kabuli*	*Ca_Kabuli_Chr01*	12754422	(G/T)
CakSNP1224	*Kabuli*	*Ca_Kabuli_Chr01*	12794075	(C/A)
CakSNP1225	*Kabuli*	*Ca_Kabuli_Chr01*	12794147	(G/T)
CakSNP1226	*Kabuli*	*Ca_Kabuli_Chr01*	12794251	(A/C)
CakSNP1227	*Kabuli*	*Ca_Kabuli_Chr01*	12800638	(T/C)
CakSNP1228	*Kabuli*	*Ca_Kabuli_Chr01*	12800610	(A/C)
CakSNP1229	*Kabuli*	*Ca_Kabuli_Chr01*	12800607	(T/A)

SNP IDs	Cultivars	Chromosomes/scaffolds	Physical positions (bp)	SNPs
CakSNP1230	*Kabuli*	*Ca_Kabuli_Chr01*	12800601	(T/A)
CakSNP1231	*Kabuli*	*Ca_Kabuli_Chr01*	12800597	(G/T)
CakSNP1232	*Kabuli*	*Ca_Kabuli_Chr01*	12831717	(G/T)
CakSNP1233	*Kabuli*	*Ca_Kabuli_Chr01*	12845445	(A/C)
CakSNP1234	*Kabuli*	*Ca_Kabuli_Chr01*	12875506	(G/A)
CakSNP1235	*Kabuli*	*Ca_Kabuli_Chr01*	12880408	(G/A)
CakSNP1236	*Kabuli*	*Ca_Kabuli_Chr01*	12912621	(C/T)
CakSNP1237	*Kabuli*	*Ca_Kabuli_Chr01*	12912625	(T/C)
CakSNP1238	*Kabuli*	*Ca_Kabuli_Chr01*	12912670	(C/T)
CakSNP1239	*Kabuli*	*Ca_Kabuli_Chr01*	12947377	(C/T)
CakSNP1240	*Kabuli*	*Ca_Kabuli_Chr01*	12947376	(C/T)
CakSNP1241	*Kabuli*	*Ca_Kabuli_Chr01*	12947358	(A/T)
CakSNP1242	*Kabuli*	*Ca_Kabuli_Chr01*	12947347	(A/G)
CakSNP1243	*Kabuli*	*Ca_Kabuli_Chr01*	12992445	(A/C)
CakSNP1244	*Kabuli*	*Ca_Kabuli_Chr01*	12992423	(A/C)
CakSNP1245	*Kabuli*	*Ca_Kabuli_Chr01*	12992569	(A/G)
CakSNP1246	*Kabuli*	*Ca_Kabuli_Chr01*	13028543	(T/C)
CakSNP1247	*Kabuli*	*Ca_Kabuli_Chr01*	13028517	(G/A)
CakSNP1248	*Kabuli*	*Ca_Kabuli_Chr01*	13028505	(A/G)
CakSNP1249	*Kabuli*	*Ca_Kabuli_Chr01*	13034708	(T/G)
CakSNP1250	*Kabuli*	*Ca_Kabuli_Chr01*	13034810	(G/A)
CakSNP1251	*Kabuli*	*Ca_Kabuli_Chr01*	13040664	(T/C)
CakSNP1252	*Kabuli*	*Ca_Kabuli_Chr01*	13066954	(C/T)
CakSNP1253	*Kabuli*	*Ca_Kabuli_Chr01*	13066968	(C/G)
CakSNP1254	*Kabuli*	*Ca_Kabuli_Chr01*	13076647	(T/A)
CakSNP1255	*Kabuli*	*Ca_Kabuli_Chr01*	13076695	(G/A)
CakSNP1256	*Kabuli*	*Ca_Kabuli_Chr01*	13156473	(A/C)
CakSNP1257	*Kabuli*	*Ca_Kabuli_Chr01*	13158244	(C/T)
CakSNP1258	*Kabuli*	*Ca_Kabuli_Chr01*	13158201	(C/T)
CakSNP1259	*Kabuli*	*Ca_Kabuli_Chr01*	13158192	(T/C)
CakSNP1260	*Kabuli*	*Ca_Kabuli_Chr01*	13199761	(T/A)
CakSNP1261	*Kabuli*	*Ca_Kabuli_Chr01*	13202986	(T/G)
CakSNP1262	*Kabuli*	*Ca_Kabuli_Chr01*	13202991	(T/G)
CakSNP1263	*Kabuli*	*Ca_Kabuli_Chr01*	13216776	(G/T)
CakSNP1264	*Kabuli*	*Ca_Kabuli_Chr01*	13216741	(T/A)
CakSNP1265	*Kabuli*	*Ca_Kabuli_Chr01*	13216710	(G/A)
CakSNP1266	*Kabuli*	*Ca_Kabuli_Chr01*	13267896	(T/C)
CakSNP1267	*Kabuli*	*Ca_Kabuli_Chr01*	13282556	(A/G)
CakSNP1268	*Kabuli*	*Ca_Kabuli_Chr01*	13282552	(T/C)
CakSNP1269	*Kabuli*	*Ca_Kabuli_Chr01*	13316992	(C/A)
CakSNP1270	*Kabuli*	*Ca_Kabuli_Chr01*	13331955	(T/A)

SNP IDs	Cultivars	Chromosomes/scaffolds	Physical positions (bp)	SNPs
CakSNP1271	*Kabuli*	*Ca_Kabuli_Chr01*	13331941	(C/T)
CakSNP1272	*Kabuli*	*Ca_Kabuli_Chr01*	13332015	(C/A)
CakSNP1273	*Kabuli*	*Ca_Kabuli_Chr01*	13332025	(T/G)
CakSNP1274	*Kabuli*	*Ca_Kabuli_Chr01*	13365594	(T/A)
CakSNP1275	*Kabuli*	*Ca_Kabuli_Chr01*	13365625	(C/G)
CakSNP1276	*Kabuli*	*Ca_Kabuli_Chr01*	13368341	(G/A)
CakSNP1277	*Kabuli*	*Ca_Kabuli_Chr01*	13368556	(T/A)
CakSNP1278	*Kabuli*	*Ca_Kabuli_Chr01*	13369730	(G/A)
CakSNP1279	*Kabuli*	*Ca_Kabuli_Chr01*	13369986	(C/A)
CakSNP1280	*Kabuli*	*Ca_Kabuli_Chr01*	13497572	(G/T)
CakSNP1281	*Kabuli*	*Ca_Kabuli_Chr01*	13549635	(T/C)
CakSNP1282	*Kabuli*	*Ca_Kabuli_Chr01*	13550600	(G/A)
CakSNP1283	*Kabuli*	*Ca_Kabuli_Chr01*	13560364	(T/G)
CakSNP1284	*Kabuli*	*Ca_Kabuli_Chr01*	13590903	(G/T)
CakSNP1285	*Kabuli*	*Ca_Kabuli_Chr01*	13591177	(G/A)
CakSNP1286	*Kabuli*	*Ca_Kabuli_Chr01*	13591203	(C/G)
CakSNP1287	*Kabuli*	*Ca_Kabuli_Chr01*	13591314	(C/T)
CakSNP1288	*Kabuli*	*Ca_Kabuli_Chr01*	13593931	(T/A)
CakSNP1289	*Kabuli*	*Ca_Kabuli_Chr01*	13596411	(G/T)
CakSNP1290	*Kabuli*	*Ca_Kabuli_Chr01*	13596528	(T/A)
CakSNP1291	*Kabuli*	*Ca_Kabuli_Chr01*	13596539	(A/G)
CakSNP1292	*Kabuli*	*Ca_Kabuli_Chr01*	13596666	(T/A)
CakSNP1293	*Kabuli*	*Ca_Kabuli_Chr01*	13596659	(T/A)
CakSNP1294	*Kabuli*	*Ca_Kabuli_Chr01*	13596631	(C/T)
CakSNP1295	*Kabuli*	*Ca_Kabuli_Chr01*	13596621	(C/T)
CakSNP1296	*Kabuli*	*Ca_Kabuli_Chr01*	13596741	(C/T)
CakSNP1297	*Kabuli*	*Ca_Kabuli_Chr01*	13596771	(C/A)
CakSNP1298	*Kabuli*	*Ca_Kabuli_Chr01*	13607486	(A/C)
CakSNP1299	*Kabuli*	*Ca_Kabuli_Chr01*	13608414	(T/C)
CakSNP1300	*Kabuli*	*Ca_Kabuli_Chr01*	13637705	(G/T)
CakSNP1301	*Kabuli*	*Ca_Kabuli_Chr01*	13637688	(A/G)
CakSNP1302	*Kabuli*	*Ca_Kabuli_Chr01*	13637687	(C/T)
CakSNP1303	*Kabuli*	*Ca_Kabuli_Chr01*	13637684	(C/T)
CakSNP1304	*Kabuli*	*Ca_Kabuli_Chr01*	13639157	(T/C)
CakSNP1305	*Kabuli*	*Ca_Kabuli_Chr01*	13641517	(A/G)
CakSNP1306	*Kabuli*	*Ca_Kabuli_Chr01*	13641644	(G/A)
CakSNP1307	*Kabuli*	*Ca_Kabuli_Chr01*	13641615	(A/G)
CakSNP1308	*Kabuli*	*Ca_Kabuli_Chr01*	13641608	(G/T)
CakSNP1309	*Kabuli*	*Ca_Kabuli_Chr01*	13641574	(A/C)
CakSNP1310	*Kabuli*	*Ca_Kabuli_Chr01*	13641564	(G/A)
CakSNP1311	*Kabuli*	*Ca_Kabuli_Chr01*	13668759	(T/A)

SNP IDs	Cultivars	Chromosomes/scaffolds	Physical positions (bp)	SNPs
CakSNP1312	*Kabuli*	*Ca_Kabuli_Chr01*	13680002	(G/T)
CakSNP1313	*Kabuli*	*Ca_Kabuli_Chr01*	13680008	(G/C)
CakSNP1314	*Kabuli*	*Ca_Kabuli_Chr01*	13762410	(C/A)
CakSNP1315	*Kabuli*	*Ca_Kabuli_Chr01*	13769805	(C/T)
CakSNP1316	*Kabuli*	*Ca_Kabuli_Chr01*	13769771	(T/C)
CakSNP1317	*Kabuli*	*Ca_Kabuli_Chr01*	13782049	(C/T)
CakSNP1318	*Kabuli*	*Ca_Kabuli_Chr01*	13814790	(T/C)
CakSNP1319	*Kabuli*	*Ca_Kabuli_Chr01*	13828703	(C/T)
CakSNP1320	*Kabuli*	*Ca_Kabuli_Chr01*	13828659	(G/A)
CakSNP1321	*Kabuli*	*Ca_Kabuli_Chr01*	13828657	(G/A)
CakSNP1322	*Kabuli*	*Ca_Kabuli_Chr01*	13828744	(A/T)
CakSNP1323	*Kabuli*	*Ca_Kabuli_Chr01*	13991356	(T/G)
CakSNP1324	*Kabuli*	*Ca_Kabuli_Chr01*	14014566	(C/A)
CakSNP1325	*Kabuli*	*Ca_Kabuli_Chr01*	14015757	(T/C)
CakSNP1326	*Kabuli*	*Ca_Kabuli_Chr01*	14015781	(G/C)
CakSNP1327	*Kabuli*	*Ca_Kabuli_Chr01*	14015818	(C/T)
CakSNP1328	*Kabuli*	*Ca_Kabuli_Chr01*	14022892	(C/A)
CakSNP1329	*Kabuli*	*Ca_Kabuli_Chr01*	14022890	(A/T)
CakSNP1330	*Kabuli*	*Ca_Kabuli_Chr01*	14023097	(A/C)
CakSNP1331	*Kabuli*	*Ca_Kabuli_Chr01*	14023095	(T/A)
CakSNP1332	*Kabuli*	*Ca_Kabuli_Chr01*	14023078	(T/A)
CakSNP1333	*Kabuli*	*Ca_Kabuli_Chr01*	14025015	(G/A)
CakSNP1334	*Kabuli*	*Ca_Kabuli_Chr01*	14025075	(A/G)
CakSNP1335	*Kabuli*	*Ca_Kabuli_Chr01*	14026347	(C/G)
CakSNP1336	*Kabuli*	*Ca_Kabuli_Chr01*	14026391	(T/C)
CakSNP1337	*Kabuli*	*Ca_Kabuli_Chr01*	14026467	(C/T)
CakSNP1338	*Kabuli*	*Ca_Kabuli_Chr01*	14026444	(G/T)
CakSNP1339	*Kabuli*	*Ca_Kabuli_Chr01*	14026437	(C/A)
CakSNP1340	*Kabuli*	*Ca_Kabuli_Chr01*	14033226	(C/T)
CakSNP1341	*Kabuli*	*Ca_Kabuli_Chr01*	14033285	(C/T)
CakSNP1342	*Kabuli*	*Ca_Kabuli_Chr01*	14033277	(G/C)
CakSNP1343	*Kabuli*	*Ca_Kabuli_Chr01*	14033951	(G/A)
CakSNP1344	*Kabuli*	*Ca_Kabuli_Chr01*	14034011	(A/G)
CakSNP1345	*Kabuli*	*Ca_Kabuli_Chr01*	14045110	(T/C)
CakSNP1346	*Kabuli*	*Ca_Kabuli_Chr01*	14045239	(A/G)
CakSNP1347	*Kabuli*	*Ca_Kabuli_Chr01*	14076924	(T/C)
CakSNP1348	*Kabuli*	*Ca_Kabuli_Chr01*	14101608	(C/T)
CakSNP1349	*Kabuli*	*Ca_Kabuli_Chr01*	14101621	(T/C)
CakSNP1350	*Kabuli*	*Ca_Kabuli_Chr01*	14101624	(C/T)
CakSNP1351	*Kabuli*	*Ca_Kabuli_Chr01*	14101644	(A/G)
CakSNP1352	*Kabuli*	*Ca_Kabuli_Chr01*	14163638	(C/T)

**SNP IDs**	**Cultivars**	**Chromosomes/scaffolds**	**Physical positions (bp)**	**SNPs**
CakSNP1353	*Kabuli*	*Ca_Kabuli_Chr01*	14163721	(T/C)
CakSNP1354	*Kabuli*	*Ca_Kabuli_Chr01*	14163836	(A/C)
CakSNP1355	*Kabuli*	*Ca_Kabuli_Chr01*	14225210	(G/T)
CakSNP1356	*Kabuli*	*Ca_Kabuli_Chr01*	14237163	(T/G)
CakSNP1357	*Kabuli*	*Ca_Kabuli_Chr01*	14237164	(T/A)
CakSNP1358	*Kabuli*	*Ca_Kabuli_Chr01*	14237273	(A/T)
CakSNP1359	*Kabuli*	*Ca_Kabuli_Chr01*	14240571	(C/T)
CakSNP1360	*Kabuli*	*Ca_Kabuli_Chr01*	14259435	(G/C)
CakSNP1361	*Kabuli*	*Ca_Kabuli_Chr01*	14259455	(A/G)
CakSNP1362	*Kabuli*	*Ca_Kabuli_Chr01*	14281643	(T/C)
CakSNP1363	*Kabuli*	*Ca_Kabuli_Chr01*	14348207	(C/T)
CakSNP1364	*Kabuli*	*Ca_Kabuli_Chr01*	14393255	(A/G)
CakSNP1365	*Kabuli*	*Ca_Kabuli_Chr01*	14393313	(C/A)
CakSNP1366	*Kabuli*	*Ca_Kabuli_Chr01*	14459908	(T/C)
CakSNP1367	*Kabuli*	*Ca_Kabuli_Chr01*	14576525	(C/T)
CakSNP1368	*Kabuli*	*Ca_Kabuli_Chr01*	14623271	(T/C)
CakSNP1369	*Kabuli*	*Ca_Kabuli_Chr01*	14698461	(A/T)
CakSNP1370	*Kabuli*	*Ca_Kabuli_Chr01*	14801406	(A/G)
CakSNP1371	*Kabuli*	*Ca_Kabuli_Chr01*	14884940	(G/T)
CakSNP1372	*Kabuli*	*Ca_Kabuli_Chr01*	14916633	(G/C)
CakSNP1373	*Kabuli*	*Ca_Kabuli_Chr01*	15126244	(T/C)
CakSNP1374	*Kabuli*	*Ca_Kabuli_Chr01*	15137319	(C/T)
CakSNP1375	*Kabuli*	*Ca_Kabuli_Chr01*	15146123	(A/G)
CakSNP1376	*Kabuli*	*Ca_Kabuli_Chr01*	15146149	(G/A)
CakSNP1377	*Kabuli*	*Ca_Kabuli_Chr01*	15167641	(T/C)
CakSNP1378	*Kabuli*	*Ca_Kabuli_Chr01*	15167643	(G/A)
CakSNP1379	*Kabuli*	*Ca_Kabuli_Chr01*	15196154	(T/A)
CakSNP1380	*Kabuli*	*Ca_Kabuli_Chr01*	15236202	(A/G)
CakSNP1381	*Kabuli*	*Ca_Kabuli_Chr01*	15253283	(G/A)
CakSNP1382	*Kabuli*	*Ca_Kabuli_Chr01*	15253285	(A/G)
CakSNP1383	*Kabuli*	*Ca_Kabuli_Chr01*	15291748	(C/T)
CakSNP1384	*Kabuli*	*Ca_Kabuli_Chr01*	15319181	(T/C)
CakSNP1385	*Kabuli*	*Ca_Kabuli_Chr01*	15611784	(T/C)
CakSNP1386	*Kabuli*	*Ca_Kabuli_Chr01*	15613071	(A/C)
CakSNP1387	*Kabuli*	*Ca_Kabuli_Chr01*	15683091	(G/A)
CakSNP1388	*Kabuli*	*Ca_Kabuli_Chr01*	15683101	(A/C)
CakSNP1389	*Kabuli*	*Ca_Kabuli_Chr01*	15683106	(C/A)
CakSNP1390	*Kabuli*	*Ca_Kabuli_Chr01*	15683117	(G/T)
CakSNP1391	*Kabuli*	*Ca_Kabuli_Chr01*	15683138	(A/G)
CakSNP1392	*Kabuli*	*Ca_Kabuli_Chr01*	15760638	(C/A)
CakSNP1393	*Kabuli*	*Ca_Kabuli_Chr01*	15784021	(C/T)

SNP IDs	Cultivars	Chromosomes/scaffolds	Physical positions (bp)	SNPs
CakSNP1394	*Kabuli*	*Ca_Kabuli_Chr01*	15802393	(G/A)
CakSNP1395	*Kabuli*	*Ca_Kabuli_Chr01*	15862493	(C/T)
CakSNP1396	*Kabuli*	*Ca_Kabuli_Chr01*	15960425	(A/C)
CakSNP1397	*Kabuli*	*Ca_Kabuli_Chr01*	15965278	(G/A)
CakSNP1398	*Kabuli*	*Ca_Kabuli_Chr01*	16061242	(A/G)
CakSNP1399	*Kabuli*	*Ca_Kabuli_Chr01*	16061293	(C/G)
CakSNP1400	*Kabuli*	*Ca_Kabuli_Chr01*	16061294	(T/A)
CakSNP1401	*Kabuli*	*Ca_Kabuli_Chr01*	16061295	(A/G)
CakSNP1402	*Kabuli*	*Ca_Kabuli_Chr01*	16061300	(A/G)
CakSNP1403	*Kabuli*	*Ca_Kabuli_Chr01*	16061385	(A/G)
CakSNP1404	*Kabuli*	*Ca_Kabuli_Chr01*	16061486	(G/A)
CakSNP1405	*Kabuli*	*Ca_Kabuli_Chr01*	16061541	(G/A)
CakSNP1406	*Kabuli*	*Ca_Kabuli_Chr01*	16061536	(A/C)
CakSNP1407	*Kabuli*	*Ca_Kabuli_Chr01*	16081438	(T/C)
CakSNP1408	*Kabuli*	*Ca_Kabuli_Chr01*	16099437	(T/G)
CakSNP1409	*Kabuli*	*Ca_Kabuli_Chr01*	16099438	(G/A)
CakSNP1410	*Kabuli*	*Ca_Kabuli_Chr01*	16099468	(G/A)
CakSNP1411	*Kabuli*	*Ca_Kabuli_Chr01*	16099444	(T/C)
CakSNP1412	*Kabuli*	*Ca_Kabuli_Chr01*	16099459	(T/C)
CakSNP1413	*Kabuli*	*Ca_Kabuli_Chr01*	16099457	(T/C)
CakSNP1414	*Kabuli*	*Ca_Kabuli_Chr01*	16104255	(G/T)
CakSNP1415	*Kabuli*	*Ca_Kabuli_Chr01*	16104351	(G/A)
CakSNP1416	*Kabuli*	*Ca_Kabuli_Chr01*	16104334	(T/G)
CakSNP1417	*Kabuli*	*Ca_Kabuli_Chr01*	16104298	(C/T)
CakSNP1418	*Kabuli*	*Ca_Kabuli_Chr01*	16114607	(C/A)
CakSNP1419	*Kabuli*	*Ca_Kabuli_Chr01*	16196930	(A/G)
CakSNP1420	*Kabuli*	*Ca_Kabuli_Chr01*	16228049	(C/A)
CakSNP1421	*Kabuli*	*Ca_Kabuli_Chr01*	16228039	(C/A)
CakSNP1422	*Kabuli*	*Ca_Kabuli_Chr01*	16289397	(G/A)
CakSNP1423	*Kabuli*	*Ca_Kabuli_Chr01*	16329161	(G/A)
CakSNP1424	*Kabuli*	*Ca_Kabuli_Chr01*	16376254	(G/T)
CakSNP1425	*Kabuli*	*Ca_Kabuli_Chr01*	16379239	(A/G)
CakSNP1426	*Kabuli*	*Ca_Kabuli_Chr01*	16379258	(C/T)
CakSNP1427	*Kabuli*	*Ca_Kabuli_Chr01*	16410186	(C/T)
CakSNP1428	*Kabuli*	*Ca_Kabuli_Chr01*	16419469	(C/T)
CakSNP1429	*Kabuli*	*Ca_Kabuli_Chr01*	16454432	(C/T)
CakSNP1430	*Kabuli*	*Ca_Kabuli_Chr01*	16454487	(G/C)
CakSNP1431	*Kabuli*	*Ca_Kabuli_Chr01*	16493894	(A/G)
CakSNP1432	*Kabuli*	*Ca_Kabuli_Chr01*	16512011	(G/T)
CakSNP1433	*Kabuli*	*Ca_Kabuli_Chr01*	16512026	(C/T)
CakSNP1434	*Kabuli*	*Ca_Kabuli_Chr01*	16512171	(G/A)

SNP IDs	Cultivars	Chromosomes/scaffolds	Physical positions (bp)	SNPs
CakSNP1435	*Kabuli*	*Ca_Kabuli_Chr01*	16512195	(G/A)
CakSNP1436	*Kabuli*	*Ca_Kabuli_Chr01*	16515112	(T/C)
CakSNP1437	*Kabuli*	*Ca_Kabuli_Chr01*	16527347	(T/A)
CakSNP1438	*Kabuli*	*Ca_Kabuli_Chr01*	16527366	(G/A)
CakSNP1439	*Kabuli*	*Ca_Kabuli_Chr01*	16575167	(A/G)
CakSNP1440	*Kabuli*	*Ca_Kabuli_Chr01*	16636587	(G/A)
CakSNP1441	*Kabuli*	*Ca_Kabuli_Chr01*	16743569	(C/T)
CakSNP1442	*Kabuli*	*Ca_Kabuli_Chr01*	16799112	(T/C)
CakSNP1443	*Kabuli*	*Ca_Kabuli_Chr01*	16799142	(C/T)
CakSNP1444	*Kabuli*	*Ca_Kabuli_Chr01*	16813600	(T/A)
CakSNP1445	*Kabuli*	*Ca_Kabuli_Chr01*	16813603	(T/G)
CakSNP1446	*Kabuli*	*Ca_Kabuli_Chr01*	16813604	(T/C)
CakSNP1447	*Kabuli*	*Ca_Kabuli_Chr01*	16813657	(A/T)
CakSNP1448	*Kabuli*	*Ca_Kabuli_Chr01*	16813616	(T/A)
CakSNP1449	*Kabuli*	*Ca_Kabuli_Chr01*	16873356	(C/T)
CakSNP1450	*Kabuli*	*Ca_Kabuli_Chr01*	16877812	(C/A)
CakSNP1451	*Kabuli*	*Ca_Kabuli_Chr01*	16877829	(C/A)
CakSNP1452	*Kabuli*	*Ca_Kabuli_Chr01*	16883153	(C/T)
CakSNP1453	*Kabuli*	*Ca_Kabuli_Chr01*	16901798	(C/A)
CakSNP1454	*Kabuli*	*Ca_Kabuli_Chr01*	16902129	(G/A)
CakSNP1455	*Kabuli*	*Ca_Kabuli_Chr01*	16908636	(G/A)
CakSNP1456	*Kabuli*	*Ca_Kabuli_Chr01*	16933423	(T/G)
CakSNP1457	*Kabuli*	*Ca_Kabuli_Chr01*	16933431	(T/G)
CakSNP1458	*Kabuli*	*Ca_Kabuli_Chr01*	17006391	(T/C)
CakSNP1459	*Kabuli*	*Ca_Kabuli_Chr01*	17023111	(G/C)
CakSNP1460	*Kabuli*	*Ca_Kabuli_Chr01*	17024083	(A/C)
CakSNP1461	*Kabuli*	*Ca_Kabuli_Chr01*	17068798	(A/C)
CakSNP1462	*Kabuli*	*Ca_Kabuli_Chr01*	17092204	(C/T)
CakSNP1463	*Kabuli*	*Ca_Kabuli_Chr01*	17092404	(G/A)
CakSNP1464	*Kabuli*	*Ca_Kabuli_Chr01*	17092413	(C/T)
CakSNP1465	*Kabuli*	*Ca_Kabuli_Chr01*	17093640	(T/C)
CakSNP1466	*Kabuli*	*Ca_Kabuli_Chr01*	17167019	(G/A)
CakSNP1467	*Kabuli*	*Ca_Kabuli_Chr01*	17170443	(A/T)
CakSNP1468	*Kabuli*	*Ca_Kabuli_Chr01*	17170727	(T/C)
CakSNP1469	*Kabuli*	*Ca_Kabuli_Chr01*	17182711	(C/T)
CakSNP1470	*Kabuli*	*Ca_Kabuli_Chr01*	17182697	(A/T)
CakSNP1471	*Kabuli*	*Ca_Kabuli_Chr01*	17182867	(G/A)
CakSNP1472	*Kabuli*	*Ca_Kabuli_Chr01*	17194835	(C/T)
CakSNP1473	*Kabuli*	*Ca_Kabuli_Chr01*	17272338	(A/C)
CakSNP1474	*Kabuli*	*Ca_Kabuli_Chr01*	17272401	(C/T)
CakSNP1475	*Kabuli*	*Ca_Kabuli_Chr01*	17314880	(A/C)

SNP IDs	Cultivars	Chromosomes/scaffolds	Physical positions (bp)	SNPs
CakSNP1476	*Kabuli*	*Ca_Kabuli_Chr01*	17314911	(A/G)
CakSNP1477	*Kabuli*	*Ca_Kabuli_Chr01*	17314945	(T/C)
CakSNP1478	*Kabuli*	*Ca_Kabuli_Chr01*	17315046	(G/A)
CakSNP1479	*Kabuli*	*Ca_Kabuli_Chr01*	17328721	(G/A)
CakSNP1480	*Kabuli*	*Ca_Kabuli_Chr01*	17328808	(G/A)
CakSNP1481	*Kabuli*	*Ca_Kabuli_Chr01*	17332981	(C/T)
CakSNP1482	*Kabuli*	*Ca_Kabuli_Chr01*	17333056	(C/G)
CakSNP1483	*Kabuli*	*Ca_Kabuli_Chr01*	17333049	(C/T)
CakSNP1484	*Kabuli*	*Ca_Kabuli_Chr01*	17340321	(T/C)
CakSNP1485	*Kabuli*	*Ca_Kabuli_Chr01*	17340350	(G/T)
CakSNP1486	*Kabuli*	*Ca_Kabuli_Chr01*	17340398	(G/T)
CakSNP1487	*Kabuli*	*Ca_Kabuli_Chr01*	17359712	(A/G)
CakSNP1488	*Kabuli*	*Ca_Kabuli_Chr01*	17359688	(A/C)
CakSNP1489	*Kabuli*	*Ca_Kabuli_Chr01*	17359686	(C/T)
CakSNP1490	*Kabuli*	*Ca_Kabuli_Chr01*	17359682	(A/C)
CakSNP1491	*Kabuli*	*Ca_Kabuli_Chr01*	17386549	(C/T)
CakSNP1492	*Kabuli*	*Ca_Kabuli_Chr01*	17386603	(A/T)
CakSNP1493	*Kabuli*	*Ca_Kabuli_Chr01*	17395543	(C/A)
CakSNP1494	*Kabuli*	*Ca_Kabuli_Chr01*	17437336	(G/C)
CakSNP1495	*Kabuli*	*Ca_Kabuli_Chr01*	17558885	(T/C)
CakSNP1496	*Kabuli*	*Ca_Kabuli_Chr01*	17558940	(A/G)
CakSNP1497	*Kabuli*	*Ca_Kabuli_Chr01*	17558975	(A/G)
CakSNP1498	*Kabuli*	*Ca_Kabuli_Chr01*	17576793	(T/G)
CakSNP1499	*Kabuli*	*Ca_Kabuli_Chr01*	17761244	(T/C)
CakSNP1500	*Kabuli*	*Ca_Kabuli_Chr01*	17763949	(G/A)
CakSNP1501	*Kabuli*	*Ca_Kabuli_Chr01*	17819374	(A/C)
CakSNP1502	*Kabuli*	*Ca_Kabuli_Chr01*	17856892	(A/G)
CakSNP1503	*Kabuli*	*Ca_Kabuli_Chr01*	17857089	(T/A)
CakSNP1504	*Kabuli*	*Ca_Kabuli_Chr01*	17880143	(T/C)
CakSNP1505	*Kabuli*	*Ca_Kabuli_Chr01*	17880185	(T/C)
CakSNP1506	*Kabuli*	*Ca_Kabuli_Chr01*	17961779	(T/C)
CakSNP1507	*Kabuli*	*Ca_Kabuli_Chr01*	17983301	(T/G)
CakSNP1508	*Kabuli*	*Ca_Kabuli_Chr01*	17983310	(T/G)
CakSNP1509	*Kabuli*	*Ca_Kabuli_Chr01*	18009665	(C/G)
CakSNP1510	*Kabuli*	*Ca_Kabuli_Chr01*	18050590	(G/T)
CakSNP1511	*Kabuli*	*Ca_Kabuli_Chr01*	18064930	(C/T)
CakSNP1512	*Kabuli*	*Ca_Kabuli_Chr01*	18065239	(T/C)
CakSNP1513	*Kabuli*	*Ca_Kabuli_Chr01*	18092704	(T/G)
CakSNP1514	*Kabuli*	*Ca_Kabuli_Chr01*	18092761	(C/T)
CakSNP1515	*Kabuli*	*Ca_Kabuli_Chr01*	18098516	(C/T)
CakSNP1516	*Kabuli*	*Ca_Kabuli_Chr01*	18099331	(C/T)

SNP IDs	Cultivars	Chromosomes/scaffolds	Physical positions (bp)	SNPs
CakSNP1517	*Kabuli*	*Ca_Kabuli_Chr01*	18099334	(T/A)
CakSNP1518	*Kabuli*	*Ca_Kabuli_Chr01*	18112318	(A/G)
CakSNP1519	*Kabuli*	*Ca_Kabuli_Chr01*	18112353	(T/C)
CakSNP1520	*Kabuli*	*Ca_Kabuli_Chr01*	18131516	(T/G)
CakSNP1521	*Kabuli*	*Ca_Kabuli_Chr01*	18131545	(A/T)
CakSNP1522	*Kabuli*	*Ca_Kabuli_Chr01*	18131898	(G/A)
CakSNP1523	*Kabuli*	*Ca_Kabuli_Chr01*	18141990	(C/A)
CakSNP1524	*Kabuli*	*Ca_Kabuli_Chr01*	18144107	(C/G)
CakSNP1525	*Kabuli*	*Ca_Kabuli_Chr01*	18144137	(T/A)
CakSNP1526	*Kabuli*	*Ca_Kabuli_Chr01*	18147011	(C/G)
CakSNP1527	*Kabuli*	*Ca_Kabuli_Chr01*	18150151	(G/A)
CakSNP1528	*Kabuli*	*Ca_Kabuli_Chr01*	18153870	(T/G)
CakSNP1529	*Kabuli*	*Ca_Kabuli_Chr01*	18154083	(A/G)
CakSNP1530	*Kabuli*	*Ca_Kabuli_Chr01*	18156634	(A/G)
CakSNP1531	*Kabuli*	*Ca_Kabuli_Chr01*	18172762	(G/T)
CakSNP1532	*Kabuli*	*Ca_Kabuli_Chr01*	18198444	(C/T)
CakSNP1533	*Kabuli*	*Ca_Kabuli_Chr01*	18488172	(G/A)
CakSNP1534	*Kabuli*	*Ca_Kabuli_Chr01*	18746045	(T/G)
CakSNP1535	*Kabuli*	*Ca_Kabuli_Chr01*	18763916	(G/A)
CakSNP1536	*Kabuli*	*Ca_Kabuli_Chr01*	18763911	(C/A)
CakSNP1537	*Kabuli*	*Ca_Kabuli_Chr01*	18764225	(C/T)
CakSNP1538	*Kabuli*	*Ca_Kabuli_Chr01*	18768563	(C/A)
CakSNP1539	*Kabuli*	*Ca_Kabuli_Chr01*	18797330	(T/C)
CakSNP1540	*Kabuli*	*Ca_Kabuli_Chr01*	18890559	(A/G)
CakSNP1541	*Kabuli*	*Ca_Kabuli_Chr01*	18890616	(G/A)
CakSNP1542	*Kabuli*	*Ca_Kabuli_Chr01*	18890833	(G/A)
CakSNP1543	*Kabuli*	*Ca_Kabuli_Chr01*	18910213	(C/T)
CakSNP1544	*Kabuli*	*Ca_Kabuli_Chr01*	18910202	(G/A)
CakSNP1545	*Kabuli*	*Ca_Kabuli_Chr01*	18910173	(T/C)
CakSNP1546	*Kabuli*	*Ca_Kabuli_Chr01*	19000445	(T/C)
CakSNP1547	*Kabuli*	*Ca_Kabuli_Chr01*	19055085	(C/T)
CakSNP1548	*Kabuli*	*Ca_Kabuli_Chr01*	19130894	(T/C)
CakSNP1549	*Kabuli*	*Ca_Kabuli_Chr01*	19130884	(C/A)
CakSNP1550	*Kabuli*	*Ca_Kabuli_Chr01*	19130883	(C/A)
CakSNP1551	*Kabuli*	*Ca_Kabuli_Chr01*	19130872	(G/A)
CakSNP1552	*Kabuli*	*Ca_Kabuli_Chr01*	19130833	(G/T)
CakSNP1553	*Kabuli*	*Ca_Kabuli_Chr01*	19130831	(A/G)
CakSNP1554	*Kabuli*	*Ca_Kabuli_Chr01*	19130848	(C/T)
CakSNP1555	*Kabuli*	*Ca_Kabuli_Chr01*	19130853	(G/A)
CakSNP1556	*Kabuli*	*Ca_Kabuli_Chr01*	19164976	(T/C)
CakSNP1557	*Kabuli*	*Ca_Kabuli_Chr01*	19166597	(A/G)

SNP IDs	Cultivars	Chromosomes/scaffolds	Physical positions (bp)	SNPs
CakSNP1558	*Kabuli*	*Ca_Kabuli_Chr01*	19218067	(G/A)
CakSNP1559	*Kabuli*	*Ca_Kabuli_Chr01*	19218093	(T/G)
CakSNP1560	*Kabuli*	*Ca_Kabuli_Chr01*	19252952	(G/T)
CakSNP1561	*Kabuli*	*Ca_Kabuli_Chr01*	19252938	(G/A)
CakSNP1562	*Kabuli*	*Ca_Kabuli_Chr01*	19253655	(C/T)
CakSNP1563	*Kabuli*	*Ca_Kabuli_Chr01*	19254161	(G/A)
CakSNP1564	*Kabuli*	*Ca_Kabuli_Chr01*	19276489	(A/G)
CakSNP1565	*Kabuli*	*Ca_Kabuli_Chr01*	19276697	(C/G)
CakSNP1566	*Kabuli*	*Ca_Kabuli_Chr01*	19359812	(A/C)
CakSNP1567	*Kabuli*	*Ca_Kabuli_Chr01*	19359844	(C/A)
CakSNP1568	*Kabuli*	*Ca_Kabuli_Chr01*	19399112	(C/T)
CakSNP1569	*Kabuli*	*Ca_Kabuli_Chr01*	19526320	(T/C)
CakSNP1570	*Kabuli*	*Ca_Kabuli_Chr01*	19526531	(C/G)
CakSNP1571	*Kabuli*	*Ca_Kabuli_Chr01*	19526548	(G/A)
CakSNP1572	*Kabuli*	*Ca_Kabuli_Chr01*	19526561	(T/C)
CakSNP1573	*Kabuli*	*Ca_Kabuli_Chr01*	19526592	(T/A)
CakSNP1574	*Kabuli*	*Ca_Kabuli_Chr01*	19526652	(G/T)
CakSNP1575	*Kabuli*	*Ca_Kabuli_Chr01*	19526632	(C/T)
CakSNP1576	*Kabuli*	*Ca_Kabuli_Chr01*	19572408	(C/T)
CakSNP1577	*Kabuli*	*Ca_Kabuli_Chr01*	19572494	(G/C)
CakSNP1578	*Kabuli*	*Ca_Kabuli_Chr01*	19591671	(A/G)
CakSNP1579	*Kabuli*	*Ca_Kabuli_Chr01*	19877654	(A/T)
CakSNP1580	*Kabuli*	*Ca_Kabuli_Chr01*	19902735	(T/A)
CakSNP1581	*Kabuli*	*Ca_Kabuli_Chr01*	19903665	(A/G)
CakSNP1582	*Kabuli*	*Ca_Kabuli_Chr01*	19903683	(G/A)
CakSNP1583	*Kabuli*	*Ca_Kabuli_Chr01*	19903686	(T/A)
CakSNP1584	*Kabuli*	*Ca_Kabuli_Chr01*	19903690	(A/G)
CakSNP1585	*Kabuli*	*Ca_Kabuli_Chr01*	19903693	(G/T)
CakSNP1586	*Kabuli*	*Ca_Kabuli_Chr01*	19903701	(G/A)
CakSNP1587	*Kabuli*	*Ca_Kabuli_Chr01*	19903704	(G/A)
CakSNP1588	*Kabuli*	*Ca_Kabuli_Chr01*	20004655	(A/G)
CakSNP1589	*Kabuli*	*Ca_Kabuli_Chr01*	20005747	(C/T)
CakSNP1590	*Kabuli*	*Ca_Kabuli_Chr01*	20016525	(C/T)
CakSNP1591	*Kabuli*	*Ca_Kabuli_Chr01*	20070045	(A/G)
CakSNP1592	*Kabuli*	*Ca_Kabuli_Chr01*	20070197	(T/C)
CakSNP1593	*Kabuli*	*Ca_Kabuli_Chr01*	20091291	(T/G)
CakSNP1594	*Kabuli*	*Ca_Kabuli_Chr01*	20092571	(C/G)
CakSNP1595	*Kabuli*	*Ca_Kabuli_Chr01*	20092677	(C/A)
CakSNP1596	*Kabuli*	*Ca_Kabuli_Chr01*	21822975	(A/G)
CakSNP1597	*Kabuli*	*Ca_Kabuli_Chr01*	21881133	(G/A)
CakSNP1598	*Kabuli*	*Ca_Kabuli_Chr01*	21881112	(C/T)

**SNP IDs**	**Cultivars**	**Chromosomes/scaffolds**	**Physical positions (bp)**	**SNPs**
CakSNP1599	*Kabuli*	*Ca_Kabuli_Chr01*	21881103	(G/A)
CakSNP1600	*Kabuli*	*Ca_Kabuli_Chr01*	21881094	(G/A)
CakSNP1601	*Kabuli*	*Ca_Kabuli_Chr01*	21918747	(G/A)
CakSNP1602	*Kabuli*	*Ca_Kabuli_Chr01*	22344003	(G/A)
CakSNP1603	*Kabuli*	*Ca_Kabuli_Chr01*	22437033	(C/T)
CakSNP1604	*Kabuli*	*Ca_Kabuli_Chr01*	22465555	(C/T)
CakSNP1605	*Kabuli*	*Ca_Kabuli_Chr01*	22465692	(A/G)
CakSNP1606	*Kabuli*	*Ca_Kabuli_Chr01*	22719581	(T/G)
CakSNP1607	*Kabuli*	*Ca_Kabuli_Chr01*	22743361	(T/G)
CakSNP1608	*Kabuli*	*Ca_Kabuli_Chr01*	23122951	(A/G)
CakSNP1609	*Kabuli*	*Ca_Kabuli_Chr01*	23128921	(T/A)
CakSNP1610	*Kabuli*	*Ca_Kabuli_Chr01*	23128979	(G/T)
CakSNP1611	*Kabuli*	*Ca_Kabuli_Chr01*	23129094	(C/T)
CakSNP1612	*Kabuli*	*Ca_Kabuli_Chr01*	23131178	(A/C)
CakSNP1613	*Kabuli*	*Ca_Kabuli_Chr01*	23131914	(T/C)
CakSNP1614	*Kabuli*	*Ca_Kabuli_Chr01*	23148736	(A/C)
CakSNP1615	*Kabuli*	*Ca_Kabuli_Chr01*	23149053	(A/G)
CakSNP1616	*Kabuli*	*Ca_Kabuli_Chr01*	23149096	(A/C)
CakSNP1617	*Kabuli*	*Ca_Kabuli_Chr01*	23341499	(C/G)
CakSNP1618	*Kabuli*	*Ca_Kabuli_Chr01*	23382483	(T/A)
CakSNP1619	*Kabuli*	*Ca_Kabuli_Chr01*	23382454	(G/T)
CakSNP1620	*Kabuli*	*Ca_Kabuli_Chr01*	23382664	(C/T)
CakSNP1621	*Kabuli*	*Ca_Kabuli_Chr01*	23382645	(C/G)
CakSNP1622	*Kabuli*	*Ca_Kabuli_Chr01*	23915127	(C/G)
CakSNP1623	*Kabuli*	*Ca_Kabuli_Chr01*	23941438	(G/A)
CakSNP1624	*Kabuli*	*Ca_Kabuli_Chr01*	23941518	(C/T)
CakSNP1625	*Kabuli*	*Ca_Kabuli_Chr01*	24070908	(C/T)
CakSNP1626	*Kabuli*	*Ca_Kabuli_Chr01*	24070968	(C/T)
CakSNP1627	*Kabuli*	*Ca_Kabuli_Chr01*	24071007	(C/A)
CakSNP1628	*Kabuli*	*Ca_Kabuli_Chr01*	24071014	(T/G)
CakSNP1629	*Kabuli*	*Ca_Kabuli_Chr01*	24173273	(A/G)
CakSNP1630	*Kabuli*	*Ca_Kabuli_Chr01*	24450415	(T/C)
CakSNP1631	*Kabuli*	*Ca_Kabuli_Chr01*	24450511	(A/G)
CakSNP1632	*Kabuli*	*Ca_Kabuli_Chr01*	24450483	(T/G)
CakSNP1633	*Kabuli*	*Ca_Kabuli_Chr01*	24693506	(C/T)
CakSNP1634	*Kabuli*	*Ca_Kabuli_Chr01*	24693602	(A/G)
CakSNP1635	*Kabuli*	*Ca_Kabuli_Chr01*	24693601	(C/T)
CakSNP1636	*Kabuli*	*Ca_Kabuli_Chr01*	24693586	(C/T)
CakSNP1637	*Kabuli*	*Ca_Kabuli_Chr01*	24693571	(G/A)
CakSNP1638	*Kabuli*	*Ca_Kabuli_Chr01*	24693550	(G/A)
CakSNP1639	*Kabuli*	*Ca_Kabuli_Chr01*	24693533	(G/A)

SNP IDs	Cultivars	Chromosomes/scaffolds	Physical positions (bp)	SNPs
CakSNP1640	*Kabuli*	*Ca_Kabuli_Chr01*	24693521	(G/A)
CakSNP1641	*Kabuli*	*Ca_Kabuli_Chr01*	24828169	(C/T)
CakSNP1642	*Kabuli*	*Ca_Kabuli_Chr01*	24828245	(A/C)
CakSNP1643	*Kabuli*	*Ca_Kabuli_Chr01*	24845148	(A/C)
CakSNP1644	*Kabuli*	*Ca_Kabuli_Chr01*	24983908	(A/G)
CakSNP1645	*Kabuli*	*Ca_Kabuli_Chr01*	25039836	(T/C)
CakSNP1646	*Kabuli*	*Ca_Kabuli_Chr01*	25083482	(A/C)
CakSNP1647	*Kabuli*	*Ca_Kabuli_Chr01*	25083534	(A/C)
CakSNP1648	*Kabuli*	*Ca_Kabuli_Chr01*	25175124	(C/T)
CakSNP1649	*Kabuli*	*Ca_Kabuli_Chr01*	25176162	(C/A)
CakSNP1650	*Kabuli*	*Ca_Kabuli_Chr01*	25192363	(T/C)
CakSNP1651	*Kabuli*	*Ca_Kabuli_Chr01*	25524382	(C/T)
CakSNP1652	*Kabuli*	*Ca_Kabuli_Chr01*	25524425	(C/T)
CakSNP1653	*Kabuli*	*Ca_Kabuli_Chr01*	25524441	(T/C)
CakSNP1654	*Kabuli*	*Ca_Kabuli_Chr01*	25524459	(C/G)
CakSNP1655	*Kabuli*	*Ca_Kabuli_Chr01*	25524532	(T/C)
CakSNP1656	*Kabuli*	*Ca_Kabuli_Chr01*	25524519	(C/T)
CakSNP1657	*Kabuli*	*Ca_Kabuli_Chr01*	25524481	(T/A)
CakSNP1658	*Kabuli*	*Ca_Kabuli_Chr01*	25524480	(G/A)
CakSNP1659	*Kabuli*	*Ca_Kabuli_Chr01*	25524473	(C/T)
CakSNP1660	*Kabuli*	*Ca_Kabuli_Chr01*	25524465	(A/G)
CakSNP1661	*Kabuli*	*Ca_Kabuli_Chr01*	25524460	(G/A)
CakSNP1662	*Kabuli*	*Ca_Kabuli_Chr01*	25524458	(G/A)
CakSNP1663	*Kabuli*	*Ca_Kabuli_Chr01*	25524456	(C/T)
CakSNP1664	*Kabuli*	*Ca_Kabuli_Chr01*	25524455	(G/A)
CakSNP1665	*Kabuli*	*Ca_Kabuli_Chr01*	25524453	(C/T)
CakSNP1666	*Kabuli*	*Ca_Kabuli_Chr01*	25524451	(A/T)
CakSNP1667	*Kabuli*	*Ca_Kabuli_Chr01*	25524450	(C/T)
CakSNP1668	*Kabuli*	*Ca_Kabuli_Chr01*	25524464	(G/A)
CakSNP1669	*Kabuli*	*Ca_Kabuli_Chr01*	25524478	(C/A)
CakSNP1670	*Kabuli*	*Ca_Kabuli_Chr01*	25524488	(G/T)
CakSNP1671	*Kabuli*	*Ca_Kabuli_Chr01*	25524498	(G/C)
CakSNP1672	*Kabuli*	*Ca_Kabuli_Chr01*	25524505	(A/G)
CakSNP1673	*Kabuli*	*Ca_Kabuli_Chr01*	25524520	(C/T)
CakSNP1674	*Kabuli*	*Ca_Kabuli_Chr01*	25524522	(G/A)
CakSNP1675	*Kabuli*	*Ca_Kabuli_Chr01*	25524524	(C/T)
CakSNP1676	*Kabuli*	*Ca_Kabuli_Chr01*	25732004	(T/C)
CakSNP1677	*Kabuli*	*Ca_Kabuli_Chr01*	25887539	(G/A)
CakSNP1678	*Kabuli*	*Ca_Kabuli_Chr01*	25987201	(A/C)
CakSNP1679	*Kabuli*	*Ca_Kabuli_Chr01*	26026817	(C/T)
CakSNP1680	*Kabuli*	*Ca_Kabuli_Chr01*	26190096	(G/A)

**SNP IDs**	**Cultivars**	**Chromosomes/scaffolds**	**Physical positions (bp)**	**SNPs**
CakSNP1681	*Kabuli*	*Ca_Kabuli_Chr01*	26190050	(G/A)
CakSNP1682	*Kabuli*	*Ca_Kabuli_Chr01*	26481730	(C/T)
CakSNP1683	*Kabuli*	*Ca_Kabuli_Chr01*	26481747	(T/G)
CakSNP1684	*Kabuli*	*Ca_Kabuli_Chr01*	26484868	(G/C)
CakSNP1685	*Kabuli*	*Ca_Kabuli_Chr01*	26574098	(C/T)
CakSNP1686	*Kabuli*	*Ca_Kabuli_Chr01*	26574146	(C/T)
CakSNP1687	*Kabuli*	*Ca_Kabuli_Chr01*	26579621	(A/C)
CakSNP1688	*Kabuli*	*Ca_Kabuli_Chr01*	26746094	(A/T)
CakSNP1689	*Kabuli*	*Ca_Kabuli_Chr01*	26767522	(A/G)
CakSNP1690	*Kabuli*	*Ca_Kabuli_Chr01*	26767514	(A/G)
CakSNP1691	*Kabuli*	*Ca_Kabuli_Chr01*	26784404	(G/A)
CakSNP1692	*Kabuli*	*Ca_Kabuli_Chr01*	26784421	(G/T)
CakSNP1693	*Kabuli*	*Ca_Kabuli_Chr01*	26784422	(C/T)
CakSNP1694	*Kabuli*	*Ca_Kabuli_Chr01*	26784464	(C/T)
CakSNP1695	*Kabuli*	*Ca_Kabuli_Chr01*	26784548	(C/A)
CakSNP1696	*Kabuli*	*Ca_Kabuli_Chr01*	26784570	(A/G)
CakSNP1697	*Kabuli*	*Ca_Kabuli_Chr01*	26784592	(A/G)
CakSNP1698	*Kabuli*	*Ca_Kabuli_Chr01*	26784618	(G/A)
CakSNP1699	*Kabuli*	*Ca_Kabuli_Chr01*	26784732	(C/G)
CakSNP1700	*Kabuli*	*Ca_Kabuli_Chr01*	26785566	(T/G)
CakSNP1701	*Kabuli*	*Ca_Kabuli_Chr01*	26807401	(A/G)
CakSNP1702	*Kabuli*	*Ca_Kabuli_Chr01*	26807395	(T/G)
CakSNP1703	*Kabuli*	*Ca_Kabuli_Chr01*	26856541	(A/T)
CakSNP1704	*Kabuli*	*Ca_Kabuli_Chr01*	26856691	(G/A)
CakSNP1705	*Kabuli*	*Ca_Kabuli_Chr01*	26921471	(C/T)
CakSNP1706	*Kabuli*	*Ca_Kabuli_Chr01*	26921487	(C/A)
CakSNP1707	*Kabuli*	*Ca_Kabuli_Chr01*	26922011	(T/G)
CakSNP1708	*Kabuli*	*Ca_Kabuli_Chr01*	26922631	(C/T)
CakSNP1709	*Kabuli*	*Ca_Kabuli_Chr01*	26953535	(C/T)
CakSNP1710	*Kabuli*	*Ca_Kabuli_Chr01*	27012495	(C/T)
CakSNP1711	*Kabuli*	*Ca_Kabuli_Chr01*	27012660	(G/C)
CakSNP1712	*Kabuli*	*Ca_Kabuli_Chr01*	27014895	(T/G)
CakSNP1713	*Kabuli*	*Ca_Kabuli_Chr01*	27014981	(A/G)
CakSNP1714	*Kabuli*	*Ca_Kabuli_Chr01*	27014951	(A/C)
CakSNP1715	*Kabuli*	*Ca_Kabuli_Chr01*	27016101	(G/A)
CakSNP1716	*Kabuli*	*Ca_Kabuli_Chr01*	27016206	(T/A)
CakSNP1717	*Kabuli*	*Ca_Kabuli_Chr01*	27017559	(G/A)
CakSNP1718	*Kabuli*	*Ca_Kabuli_Chr01*	27017560	(G/A)
CakSNP1719	*Kabuli*	*Ca_Kabuli_Chr01*	27017570	(A/G)
CakSNP1720	*Kabuli*	*Ca_Kabuli_Chr01*	27017604	(T/G)
CakSNP1721	*Kabuli*	*Ca_Kabuli_Chr01*	27017606	(C/T)

SNP IDs	Cultivars	Chromosomes/scaffolds	Physical positions (bp)	SNPs
CakSNP1722	*Kabuli*	*Ca_Kabuli_Chr01*	27017617	(G/A)
CakSNP1723	*Kabuli*	*Ca_Kabuli_Chr01*	27017626	(A/C)
CakSNP1724	*Kabuli*	*Ca_Kabuli_Chr01*	27017628	(C/A)
CakSNP1725	*Kabuli*	*Ca_Kabuli_Chr01*	27017734	(T/C)
CakSNP1726	*Kabuli*	*Ca_Kabuli_Chr01*	27017765	(A/T)
CakSNP1727	*Kabuli*	*Ca_Kabuli_Chr01*	27017771	(G/A)
CakSNP1728	*Kabuli*	*Ca_Kabuli_Chr01*	27020193	(T/C)
CakSNP1729	*Kabuli*	*Ca_Kabuli_Chr01*	27020354	(C/A)
CakSNP1730	*Kabuli*	*Ca_Kabuli_Chr01*	28088501	(T/C)
CakSNP1731	*Kabuli*	*Ca_Kabuli_Chr01*	28106756	(G/A)
CakSNP1732	*Kabuli*	*Ca_Kabuli_Chr01*	28133847	(A/G)
CakSNP1733	*Kabuli*	*Ca_Kabuli_Chr01*	28133830	(A/T)
CakSNP1734	*Kabuli*	*Ca_Kabuli_Chr01*	28133814	(C/T)
CakSNP1735	*Kabuli*	*Ca_Kabuli_Chr01*	28143955	(G/A)
CakSNP1736	*Kabuli*	*Ca_Kabuli_Chr01*	28152260	(A/G)
CakSNP1737	*Kabuli*	*Ca_Kabuli_Chr01*	28656197	(C/A)
CakSNP1738	*Kabuli*	*Ca_Kabuli_Chr01*	28832810	(C/A)
CakSNP1739	*Kabuli*	*Ca_Kabuli_Chr01*	28907617	(G/A)
CakSNP1740	*Kabuli*	*Ca_Kabuli_Chr01*	28909059	(T/A)
CakSNP1741	*Kabuli*	*Ca_Kabuli_Chr01*	29020220	(T/G)
CakSNP1742	*Kabuli*	*Ca_Kabuli_Chr01*	29116430	(T/C)
CakSNP1743	*Kabuli*	*Ca_Kabuli_Chr01*	29460440	(C/A)
CakSNP1744	*Kabuli*	*Ca_Kabuli_Chr01*	29634320	(G/T)
CakSNP1745	*Kabuli*	*Ca_Kabuli_Chr01*	29637509	(A/C)
CakSNP1746	*Kabuli*	*Ca_Kabuli_Chr01*	29637526	(C/T)
CakSNP1747	*Kabuli*	*Ca_Kabuli_Chr01*	29637572	(T/G)
CakSNP1748	*Kabuli*	*Ca_Kabuli_Chr01*	29637571	(T/G)
CakSNP1749	*Kabuli*	*Ca_Kabuli_Chr01*	30262124	(A/G)
CakSNP1750	*Kabuli*	*Ca_Kabuli_Chr01*	31080673	(T/G)
CakSNP1751	*Kabuli*	*Ca_Kabuli_Chr01*	31080792	(T/C)
CakSNP1752	*Kabuli*	*Ca_Kabuli_Chr01*	31240370	(A/G)
CakSNP1753	*Kabuli*	*Ca_Kabuli_Chr01*	31359861	(C/A)
CakSNP1754	*Kabuli*	*Ca_Kabuli_Chr01*	31369599	(T/C)
CakSNP1755	*Kabuli*	*Ca_Kabuli_Chr01*	31590263	(C/G)
CakSNP1756	*Kabuli*	*Ca_Kabuli_Chr01*	31590510	(G/A)
CakSNP1757	*Kabuli*	*Ca_Kabuli_Chr01*	31590548	(C/T)
CakSNP1758	*Kabuli*	*Ca_Kabuli_Chr01*	31838239	(G/A)
CakSNP1759	*Kabuli*	*Ca_Kabuli_Chr01*	32286538	(C/T)
CakSNP1760	*Kabuli*	*Ca_Kabuli_Chr01*	32286534	(G/C)
CakSNP1761	*Kabuli*	*Ca_Kabuli_Chr01*	32286518	(C/A)
CakSNP1762	*Kabuli*	*Ca_Kabuli_Chr01*	32286517	(G/A)

SNP IDs	Cultivars	Chromosomes/scaffolds	Physical positions (bp)	SNPs
CakSNP1763	*Kabuli*	*Ca_Kabuli_Chr01*	32286469	(G/A)
CakSNP1764	*Kabuli*	*Ca_Kabuli_Chr01*	32286466	(A/G)
CakSNP1765	*Kabuli*	*Ca_Kabuli_Chr01*	32286568	(A/C)
CakSNP1766	*Kabuli*	*Ca_Kabuli_Chr01*	32286558	(A/G)
CakSNP1767	*Kabuli*	*Ca_Kabuli_Chr01*	32286550	(G/A)
CakSNP1768	*Kabuli*	*Ca_Kabuli_Chr01*	32286536	(G/A)
CakSNP1769	*Kabuli*	*Ca_Kabuli_Chr01*	32286551	(C/T)
CakSNP1770	*Kabuli*	*Ca_Kabuli_Chr01*	32286567	(C/T)
CakSNP1771	*Kabuli*	*Ca_Kabuli_Chr01*	32467929	(G/A)
CakSNP1772	*Kabuli*	*Ca_Kabuli_Chr01*	32467954	(A/C)
CakSNP1773	*Kabuli*	*Ca_Kabuli_Chr01*	32468053	(A/C)
CakSNP1774	*Kabuli*	*Ca_Kabuli_Chr01*	32468080	(A/T)
CakSNP1775	*Kabuli*	*Ca_Kabuli_Chr01*	32468105	(C/G)
CakSNP1776	*Kabuli*	*Ca_Kabuli_Chr01*	32468116	(A/G)
CakSNP1777	*Kabuli*	*Ca_Kabuli_Chr01*	32468119	(T/C)
CakSNP1778	*Kabuli*	*Ca_Kabuli_Chr01*	33058743	(T/C)
CakSNP1779	*Kabuli*	*Ca_Kabuli_Chr01*	33063207	(C/T)
CakSNP1780	*Kabuli*	*Ca_Kabuli_Chr01*	33194600	(T/C)
CakSNP1781	*Kabuli*	*Ca_Kabuli_Chr01*	33804152	(C/T)
CakSNP1782	*Kabuli*	*Ca_Kabuli_Chr01*	33804153	(C/T)
CakSNP1783	*Kabuli*	*Ca_Kabuli_Chr01*	33804189	(C/T)
CakSNP1784	*Kabuli*	*Ca_Kabuli_Chr01*	33804201	(C/T)
CakSNP1785	*Kabuli*	*Ca_Kabuli_Chr01*	33804232	(T/G)
CakSNP1786	*Kabuli*	*Ca_Kabuli_Chr01*	33804247	(A/G)
CakSNP1787	*Kabuli*	*Ca_Kabuli_Chr01*	33804262	(C/G)
CakSNP1788	*Kabuli*	*Ca_Kabuli_Chr01*	34161921	(G/A)
CakSNP1789	*Kabuli*	*Ca_Kabuli_Chr01*	34261166	(C/A)
CakSNP1790	*Kabuli*	*Ca_Kabuli_Chr01*	34261158	(C/A)
CakSNP1791	*Kabuli*	*Ca_Kabuli_Chr01*	34311145	(T/G)
CakSNP1792	*Kabuli*	*Ca_Kabuli_Chr01*	34437114	(C/T)
CakSNP1793	*Kabuli*	*Ca_Kabuli_Chr01*	34727251	(T/G)
CakSNP1794	*Kabuli*	*Ca_Kabuli_Chr01*	34772813	(T/A)
CakSNP1795	*Kabuli*	*Ca_Kabuli_Chr01*	34866709	(C/A)
CakSNP1796	*Kabuli*	*Ca_Kabuli_Chr01*	34905580	(A/G)
CakSNP1797	*Kabuli*	*Ca_Kabuli_Chr01*	34911065	(T/A)
CakSNP1798	*Kabuli*	*Ca_Kabuli_Chr01*	34948934	(A/G)
CakSNP1799	*Kabuli*	*Ca_Kabuli_Chr01*	35041241	(T/G)
CakSNP1800	*Kabuli*	*Ca_Kabuli_Chr01*	35041324	(G/T)
CakSNP1801	*Kabuli*	*Ca_Kabuli_Chr01*	35313277	(A/G)
CakSNP1802	*Kabuli*	*Ca_Kabuli_Chr01*	35315381	(T/A)
CakSNP1803	*Kabuli*	*Ca_Kabuli_Chr01*	35315379	(A/T)

**SNP IDs**	**Cultivars**	**Chromosomes/scaffolds**	**Physical positions (bp)**	**SNPs**
CakSNP1804	*Kabuli*	*Ca_Kabuli_Chr01*	35315347	(C/T)
CakSNP1805	*Kabuli*	*Ca_Kabuli_Chr01*	35315327	(T/A)
CakSNP1806	*Kabuli*	*Ca_Kabuli_Chr01*	35315436	(G/A)
CakSNP1807	*Kabuli*	*Ca_Kabuli_Chr01*	35708841	(C/T)
CakSNP1808	*Kabuli*	*Ca_Kabuli_Chr01*	36126090	(C/T)
CakSNP1809	*Kabuli*	*Ca_Kabuli_Chr01*	36794731	(A/G)
CakSNP1810	*Kabuli*	*Ca_Kabuli_Chr01*	36794797	(G/A)
CakSNP1811	*Kabuli*	*Ca_Kabuli_Chr01*	36794800	(G/T)
CakSNP1812	*Kabuli*	*Ca_Kabuli_Chr01*	36794806	(G/T)
CakSNP1813	*Kabuli*	*Ca_Kabuli_Chr01*	36794813	(C/T)
CakSNP1814	*Kabuli*	*Ca_Kabuli_Chr01*	36794887	(G/A)
CakSNP1815	*Kabuli*	*Ca_Kabuli_Chr01*	36794870	(C/T)
CakSNP1816	*Kabuli*	*Ca_Kabuli_Chr01*	36794900	(C/T)
CakSNP1817	*Kabuli*	*Ca_Kabuli_Chr01*	36794906	(A/C)
CakSNP1818	*Kabuli*	*Ca_Kabuli_Chr01*	36794912	(G/T)
CakSNP1819	*Kabuli*	*Ca_Kabuli_Chr01*	36794913	(A/T)
CakSNP1820	*Kabuli*	*Ca_Kabuli_Chr01*	36794915	(G/A)
CakSNP1821	*Kabuli*	*Ca_Kabuli_Chr01*	36892533	(C/T)
CakSNP1822	*Kabuli*	*Ca_Kabuli_Chr01*	37026302	(C/T)
CakSNP1823	*Kabuli*	*Ca_Kabuli_Chr01*	37608030	(G/A)
CakSNP1824	*Kabuli*	*Ca_Kabuli_Chr01*	37608050	(T/G)
CakSNP1825	*Kabuli*	*Ca_Kabuli_Chr01*	37642546	(G/A)
CakSNP1826	*Kabuli*	*Ca_Kabuli_Chr01*	37643213	(T/C)
CakSNP1827	*Kabuli*	*Ca_Kabuli_Chr01*	37647602	(C/G)
CakSNP1828	*Kabuli*	*Ca_Kabuli_Chr01*	37651679	(G/A)
CakSNP1829	*Kabuli*	*Ca_Kabuli_Chr01*	37651695	(G/A)
CakSNP1830	*Kabuli*	*Ca_Kabuli_Chr01*	37651764	(A/C)
CakSNP1831	*Kabuli*	*Ca_Kabuli_Chr01*	37651711	(G/T)
CakSNP1832	*Kabuli*	*Ca_Kabuli_Chr01*	37651800	(T/A)
CakSNP1833	*Kabuli*	*Ca_Kabuli_Chr01*	37651823	(A/G)
CakSNP1834	*Kabuli*	*Ca_Kabuli_Chr01*	37651827	(C/T)
CakSNP1835	*Kabuli*	*Ca_Kabuli_Chr01*	37651843	(T/C)
CakSNP1836	*Kabuli*	*Ca_Kabuli_Chr01*	37654039	(C/G)
CakSNP1837	*Kabuli*	*Ca_Kabuli_Chr01*	37654136	(C/G)
CakSNP1838	*Kabuli*	*Ca_Kabuli_Chr01*	37654926	(C/T)
CakSNP1839	*Kabuli*	*Ca_Kabuli_Chr01*	37690416	(G/A)
CakSNP1840	*Kabuli*	*Ca_Kabuli_Chr01*	37690465	(T/C)
CakSNP1841	*Kabuli*	*Ca_Kabuli_Chr01*	37690498	(C/A)
CakSNP1842	*Kabuli*	*Ca_Kabuli_Chr01*	37690497	(A/T)
CakSNP1843	*Kabuli*	*Ca_Kabuli_Chr01*	37699039	(T/G)
CakSNP1844	*Kabuli*	*Ca_Kabuli_Chr01*	37699036	(T/C)

SNP IDs	Cultivars	Chromosomes/scaffolds	Physical positions (bp)	SNPs
CakSNP1845	*Kabuli*	*Ca_Kabuli_Chr01*	37722095	(G/A)
CakSNP1846	*Kabuli*	*Ca_Kabuli_Chr01*	37722143	(G/C)
CakSNP1847	*Kabuli*	*Ca_Kabuli_Chr01*	37768179	(T/A)
CakSNP1848	*Kabuli*	*Ca_Kabuli_Chr01*	37768206	(A/T)
CakSNP1849	*Kabuli*	*Ca_Kabuli_Chr01*	37768238	(A/G)
CakSNP1850	*Kabuli*	*Ca_Kabuli_Chr01*	37768333	(C/T)
CakSNP1851	*Kabuli*	*Ca_Kabuli_Chr01*	37768299	(A/G)
CakSNP1852	*Kabuli*	*Ca_Kabuli_Chr01*	37768276	(G/C)
CakSNP1853	*Kabuli*	*Ca_Kabuli_Chr01*	37768264	(A/G)
CakSNP1854	*Kabuli*	*Ca_Kabuli_Chr01*	37862342	(T/G)
CakSNP1855	*Kabuli*	*Ca_Kabuli_Chr01*	37880995	(T/C)
CakSNP1856	*Kabuli*	*Ca_Kabuli_Chr01*	37892088	(A/C)
CakSNP1857	*Kabuli*	*Ca_Kabuli_Chr01*	37892089	(C/T)
CakSNP1858	*Kabuli*	*Ca_Kabuli_Chr01*	37892095	(A/G)
CakSNP1859	*Kabuli*	*Ca_Kabuli_Chr01*	37892139	(A/T)
CakSNP1860	*Kabuli*	*Ca_Kabuli_Chr01*	38049721	(C/T)
CakSNP1861	*Kabuli*	*Ca_Kabuli_Chr01*	38678712	(T/C)
CakSNP1862	*Kabuli*	*Ca_Kabuli_Chr01*	38877670	(T/C)
CakSNP1863	*Kabuli*	*Ca_Kabuli_Chr01*	38877692	(G/T)
CakSNP1864	*Kabuli*	*Ca_Kabuli_Chr01*	39436291	(T/C)
CakSNP1865	*Kabuli*	*Ca_Kabuli_Chr01*	39601982	(T/G)
CakSNP1866	*Kabuli*	*Ca_Kabuli_Chr01*	39602029	(G/T)
CakSNP1867	*Kabuli*	*Ca_Kabuli_Chr01*	39616139	(A/C)
CakSNP1868	*Kabuli*	*Ca_Kabuli_Chr01*	39663432	(C/G)
CakSNP1869	*Kabuli*	*Ca_Kabuli_Chr01*	40224835	(T/A)
CakSNP1870	*Kabuli*	*Ca_Kabuli_Chr01*	40224834	(C/G)
CakSNP1871	*Kabuli*	*Ca_Kabuli_Chr01*	40224804	(A/G)
CakSNP1872	*Kabuli*	*Ca_Kabuli_Chr01*	40343216	(G/T)
CakSNP1873	*Kabuli*	*Ca_Kabuli_Chr01*	40346280	(A/C)
CakSNP1874	*Kabuli*	*Ca_Kabuli_Chr01*	40358693	(C/T)
CakSNP1875	*Kabuli*	*Ca_Kabuli_Chr01*	40358747	(T/G)
CakSNP1876	*Kabuli*	*Ca_Kabuli_Chr01*	40367940	(G/A)
CakSNP1877	*Kabuli*	*Ca_Kabuli_Chr01*	40368845	(C/T)
CakSNP1878	*Kabuli*	*Ca_Kabuli_Chr01*	40502077	(T/C)
CakSNP1879	*Kabuli*	*Ca_Kabuli_Chr01*	40570811	(G/T)
CakSNP1880	*Kabuli*	*Ca_Kabuli_Chr01*	40585026	(T/C)
CakSNP1881	*Kabuli*	*Ca_Kabuli_Chr01*	40630215	(C/T)
CakSNP1882	*Kabuli*	*Ca_Kabuli_Chr01*	40630213	(G/T)
CakSNP1883	*Kabuli*	*Ca_Kabuli_Chr01*	40630158	(T/A)
CakSNP1884	*Kabuli*	*Ca_Kabuli_Chr01*	40660069	(T/G)
CakSNP1885	*Kabuli*	*Ca_Kabuli_Chr01*	40660116	(G/A)

SNP IDs	Cultivars	Chromosomes/scaffolds	Physical positions (bp)	SNPs
CakSNP1886	*Kabuli*	*Ca_Kabuli_Chr01*	40745421	(G/T)
CakSNP1887	*Kabuli*	*Ca_Kabuli_Chr01*	40745452	(T/C)
CakSNP1888	*Kabuli*	*Ca_Kabuli_Chr01*	40745492	(A/G)
CakSNP1889	*Kabuli*	*Ca_Kabuli_Chr01*	40745846	(T/C)
CakSNP1890	*Kabuli*	*Ca_Kabuli_Chr01*	41029732	(C/T)
CakSNP1891	*Kabuli*	*Ca_Kabuli_Chr01*	41065797	(G/C)
CakSNP1892	*Kabuli*	*Ca_Kabuli_Chr01*	41125493	(T/C)
CakSNP1893	*Kabuli*	*Ca_Kabuli_Chr01*	41169218	(G/T)
CakSNP1894	*Kabuli*	*Ca_Kabuli_Chr01*	41169430	(T/G)
CakSNP1895	*Kabuli*	*Ca_Kabuli_Chr01*	41169372	(T/A)
CakSNP1896	*Kabuli*	*Ca_Kabuli_Chr01*	41178168	(G/A)
CakSNP1897	*Kabuli*	*Ca_Kabuli_Chr01*	41178183	(C/A)
CakSNP1898	*Kabuli*	*Ca_Kabuli_Chr01*	41179660	(G/T)
CakSNP1899	*Kabuli*	*Ca_Kabuli_Chr01*	41179650	(A/T)
CakSNP1900	*Kabuli*	*Ca_Kabuli_Chr01*	41185512	(T/G)
CakSNP1901	*Kabuli*	*Ca_Kabuli_Chr01*	41202173	(T/G)
CakSNP1902	*Kabuli*	*Ca_Kabuli_Chr01*	41202206	(A/G)
CakSNP1903	*Kabuli*	*Ca_Kabuli_Chr01*	41310401	(T/G)
CakSNP1904	*Kabuli*	*Ca_Kabuli_Chr01*	41339408	(T/A)
CakSNP1905	*Kabuli*	*Ca_Kabuli_Chr01*	41339445	(C/A)
CakSNP1906	*Kabuli*	*Ca_Kabuli_Chr01*	41380061	(G/T)
CakSNP1907	*Kabuli*	*Ca_Kabuli_Chr01*	41437222	(A/G)
CakSNP1908	*Kabuli*	*Ca_Kabuli_Chr01*	41437366	(T/C)
CakSNP1909	*Kabuli*	*Ca_Kabuli_Chr01*	41437453	(T/G)
CakSNP1910	*Kabuli*	*Ca_Kabuli_Chr01*	41437483	(G/A)
CakSNP1911	*Kabuli*	*Ca_Kabuli_Chr01*	41439539	(A/G)
CakSNP1912	*Kabuli*	*Ca_Kabuli_Chr01*	41439593	(A/G)
CakSNP1913	*Kabuli*	*Ca_Kabuli_Chr01*	41451910	(C/T)
CakSNP1914	*Kabuli*	*Ca_Kabuli_Chr01*	41451977	(C/T)
CakSNP1915	*Kabuli*	*Ca_Kabuli_Chr01*	41475442	(T/C)
CakSNP1916	*Kabuli*	*Ca_Kabuli_Chr01*	41484033	(T/C)
CakSNP1917	*Kabuli*	*Ca_Kabuli_Chr01*	41484085	(C/T)
CakSNP1918	*Kabuli*	*Ca_Kabuli_Chr01*	41564809	(T/C)
CakSNP1919	*Kabuli*	*Ca_Kabuli_Chr01*	41795343	(G/A)
CakSNP1920	*Kabuli*	*Ca_Kabuli_Chr01*	41795367	(C/T)
CakSNP1921	*Kabuli*	*Ca_Kabuli_Chr01*	41822419	(C/T)
CakSNP1922	*Kabuli*	*Ca_Kabuli_Chr01*	41827388	(T/C)
CakSNP1923	*Kabuli*	*Ca_Kabuli_Chr01*	41867035	(C/T)
CakSNP1924	*Kabuli*	*Ca_Kabuli_Chr01*	41897216	(C/A)
CakSNP1925	*Kabuli*	*Ca_Kabuli_Chr01*	41946005	(A/G)
CakSNP1926	*Kabuli*	*Ca_Kabuli_Chr01*	41981244	(G/A)

SNP IDs	Cultivars	Chromosomes/scaffolds	Physical positions (bp)	SNPs
CakSNP1927	*Kabuli*	*Ca_Kabuli_Chr01*	41981259	(G/A)
CakSNP1928	*Kabuli*	*Ca_Kabuli_Chr01*	42189380	(A/G)
CakSNP1929	*Kabuli*	*Ca_Kabuli_Chr01*	42452801	(G/A)
CakSNP1930	*Kabuli*	*Ca_Kabuli_Chr01*	42452939	(G/T)
CakSNP1931	*Kabuli*	*Ca_Kabuli_Chr01*	42515406	(C/T)
CakSNP1932	*Kabuli*	*Ca_Kabuli_Chr01*	42551574	(T/G)
CakSNP1933	*Kabuli*	*Ca_Kabuli_Chr01*	42551585	(C/A)
CakSNP1934	*Kabuli*	*Ca_Kabuli_Chr01*	42552658	(A/C)
CakSNP1935	*Kabuli*	*Ca_Kabuli_Chr01*	42605239	(T/G)
CakSNP1936	*Kabuli*	*Ca_Kabuli_Chr01*	42610115	(A/C)
CakSNP1937	*Kabuli*	*Ca_Kabuli_Chr01*	42613060	(T/G)
CakSNP1938	*Kabuli*	*Ca_Kabuli_Chr01*	42894518	(T/C)
CakSNP1939	*Kabuli*	*Ca_Kabuli_Chr01*	42894525	(T/C)
CakSNP1940	*Kabuli*	*Ca_Kabuli_Chr01*	42894536	(G/T)
CakSNP1941	*Kabuli*	*Ca_Kabuli_Chr01*	43053424	(G/C)
CakSNP1942	*Kabuli*	*Ca_Kabuli_Chr01*	43273910	(G/T)
CakSNP1943	*Kabuli*	*Ca_Kabuli_Chr01*	43273922	(T/C)
CakSNP1944	*Kabuli*	*Ca_Kabuli_Chr01*	43273925	(A/G)
CakSNP1945	*Kabuli*	*Ca_Kabuli_Chr01*	43273931	(G/A)
CakSNP1946	*Kabuli*	*Ca_Kabuli_Chr01*	43273941	(C/G)
CakSNP1947	*Kabuli*	*Ca_Kabuli_Chr01*	43273943	(A/G)
CakSNP1948	*Kabuli*	*Ca_Kabuli_Chr01*	43273961	(C/T)
CakSNP1949	*Kabuli*	*Ca_Kabuli_Chr01*	43273962	(A/G)
CakSNP1950	*Kabuli*	*Ca_Kabuli_Chr01*	43273968	(T/G)
CakSNP1951	*Kabuli*	*Ca_Kabuli_Chr01*	43273972	(C/T)
CakSNP1952	*Kabuli*	*Ca_Kabuli_Chr01*	43273981	(G/T)
CakSNP1953	*Kabuli*	*Ca_Kabuli_Chr01*	43273985	(G/A)
CakSNP1954	*Kabuli*	*Ca_Kabuli_Chr01*	43304584	(A/C)
CakSNP1955	*Kabuli*	*Ca_Kabuli_Chr01*	43304573	(C/T)
CakSNP1956	*Kabuli*	*Ca_Kabuli_Chr01*	43305858	(T/C)
CakSNP1957	*Kabuli*	*Ca_Kabuli_Chr01*	43305870	(G/A)
CakSNP1958	*Kabuli*	*Ca_Kabuli_Chr01*	43305886	(T/C)
CakSNP1959	*Kabuli*	*Ca_Kabuli_Chr01*	43305904	(C/A)
CakSNP1960	*Kabuli*	*Ca_Kabuli_Chr01*	43306048	(G/A)
CakSNP1961	*Kabuli*	*Ca_Kabuli_Chr01*	43306045	(C/A)
CakSNP1962	*Kabuli*	*Ca_Kabuli_Chr01*	43306036	(G/A)
CakSNP1963	*Kabuli*	*Ca_Kabuli_Chr01*	43306012	(G/A)
CakSNP1964	*Kabuli*	*Ca_Kabuli_Chr01*	43306221	(A/G)
CakSNP1965	*Kabuli*	*Ca_Kabuli_Chr01*	43509159	(A/G)
CakSNP1966	*Kabuli*	*Ca_Kabuli_Chr01*	43509409	(T/C)
CakSNP1967	*Kabuli*	*Ca_Kabuli_Chr01*	43576059	(A/G)

**SNP IDs**	**Cultivars**	**Chromosomes/scaffolds**	**Physical positions (bp)**	**SNPs**
CakSNP1968	*Kabuli*	*Ca_Kabuli_Chr01*	43585657	(T/G)
CakSNP1969	*Kabuli*	*Ca_Kabuli_Chr01*	43590106	(C/T)
CakSNP1970	*Kabuli*	*Ca_Kabuli_Chr01*	43590258	(T/C)
CakSNP1971	*Kabuli*	*Ca_Kabuli_Chr01*	43724824	(C/T)
CakSNP1972	*Kabuli*	*Ca_Kabuli_Chr01*	43724839	(C/T)
CakSNP1973	*Kabuli*	*Ca_Kabuli_Chr01*	43724963	(C/T)
CakSNP1974	*Kabuli*	*Ca_Kabuli_Chr01*	43725059	(C/T)
CakSNP1975	*Kabuli*	*Ca_Kabuli_Chr01*	43725104	(T/C)
CakSNP1976	*Kabuli*	*Ca_Kabuli_Chr01*	43727804	(C/T)
CakSNP1977	*Kabuli*	*Ca_Kabuli_Chr01*	43792565	(G/A)
CakSNP1978	*Kabuli*	*Ca_Kabuli_Chr01*	43800367	(C/T)
CakSNP1979	*Kabuli*	*Ca_Kabuli_Chr01*	43930143	(A/T)
CakSNP1980	*Kabuli*	*Ca_Kabuli_Chr01*	43930138	(T/C)
CakSNP1981	*Kabuli*	*Ca_Kabuli_Chr01*	43933881	(G/C)
CakSNP1982	*Kabuli*	*Ca_Kabuli_Chr01*	43933904	(A/G)
CakSNP1983	*Kabuli*	*Ca_Kabuli_Chr01*	43934024	(G/T)
CakSNP1984	*Kabuli*	*Ca_Kabuli_Chr01*	43934120	(A/G)
CakSNP1985	*Kabuli*	*Ca_Kabuli_Chr01*	43934117	(G/T)
CakSNP1986	*Kabuli*	*Ca_Kabuli_Chr01*	43934081	(C/T)
CakSNP1987	*Kabuli*	*Ca_Kabuli_Chr01*	43943035	(G/T)
CakSNP1988	*Kabuli*	*Ca_Kabuli_Chr01*	43954743	(C/G)
CakSNP1989	*Kabuli*	*Ca_Kabuli_Chr01*	44043281	(G/A)
CakSNP1990	*Kabuli*	*Ca_Kabuli_Chr01*	44085069	(C/T)
CakSNP1991	*Kabuli*	*Ca_Kabuli_Chr01*	44348947	(C/T)
CakSNP1992	*Kabuli*	*Ca_Kabuli_Chr01*	44349004	(T/C)
CakSNP1993	*Kabuli*	*Ca_Kabuli_Chr01*	44350953	(G/T)
CakSNP1994	*Kabuli*	*Ca_Kabuli_Chr01*	44635939	(T/C)
CakSNP1995	*Kabuli*	*Ca_Kabuli_Chr01*	44635926	(A/G)
CakSNP1996	*Kabuli*	*Ca_Kabuli_Chr01*	44635893	(A/G)
CakSNP1997	*Kabuli*	*Ca_Kabuli_Chr01*	44976738	(T/A)
CakSNP1998	*Kabuli*	*Ca_Kabuli_Chr01*	44976735	(C/T)
CakSNP1999	*Kabuli*	*Ca_Kabuli_Chr01*	44988344	(A/G)
CakSNP2000	*Kabuli*	*Ca_Kabuli_Chr01*	44988387	(C/A)
CakSNP2001	*Kabuli*	*Ca_Kabuli_Chr01*	45003190	(G/A)
CakSNP2002	*Kabuli*	*Ca_Kabuli_Chr01*	45003514	(G/A)
CakSNP2003	*Kabuli*	*Ca_Kabuli_Chr01*	45003509	(C/A)
CakSNP2004	*Kabuli*	*Ca_Kabuli_Chr01*	45003505	(A/C)
CakSNP2005	*Kabuli*	*Ca_Kabuli_Chr01*	45003938	(A/G)
CakSNP2006	*Kabuli*	*Ca_Kabuli_Chr01*	45003939	(T/A)
CakSNP2007	*Kabuli*	*Ca_Kabuli_Chr01*	45003940	(T/G)
CakSNP2008	*Kabuli*	*Ca_Kabuli_Chr01*	45003944	(C/T)

SNP IDs	Cultivars	Chromosomes/scaffolds	Physical positions (bp)	SNPs
CakSNP2009	*Kabuli*	*Ca_Kabuli_Chr01*	45003945	(T/A)
CakSNP2010	*Kabuli*	*Ca_Kabuli_Chr01*	45003946	(T/G)
CakSNP2011	*Kabuli*	*Ca_Kabuli_Chr01*	45003947	(C/A)
CakSNP2012	*Kabuli*	*Ca_Kabuli_Chr01*	45003973	(T/C)
CakSNP2013	*Kabuli*	*Ca_Kabuli_Chr01*	45003985	(G/T)
CakSNP2014	*Kabuli*	*Ca_Kabuli_Chr01*	45003998	(G/A)
CakSNP2015	*Kabuli*	*Ca_Kabuli_Chr01*	45004001	(C/T)
CakSNP2016	*Kabuli*	*Ca_Kabuli_Chr01*	45007032	(A/C)
CakSNP2017	*Kabuli*	*Ca_Kabuli_Chr01*	45007227	(T/C)
CakSNP2018	*Kabuli*	*Ca_Kabuli_Chr01*	45007259	(A/G)
CakSNP2019	*Kabuli*	*Ca_Kabuli_Chr01*	45007331	(C/T)
CakSNP2020	*Kabuli*	*Ca_Kabuli_Chr01*	45008076	(G/A)
CakSNP2021	*Kabuli*	*Ca_Kabuli_Chr01*	45008075	(T/C)
CakSNP2022	*Kabuli*	*Ca_Kabuli_Chr01*	45008073	(T/G)
CakSNP2023	*Kabuli*	*Ca_Kabuli_Chr01*	45008071	(G/C)
CakSNP2024	*Kabuli*	*Ca_Kabuli_Chr01*	45008050	(C/G)
CakSNP2025	*Kabuli*	*Ca_Kabuli_Chr01*	45008044	(T/C)
CakSNP2026	*Kabuli*	*Ca_Kabuli_Chr01*	45008041	(C/G)
CakSNP2027	*Kabuli*	*Ca_Kabuli_Chr01*	45008040	(G/C)
CakSNP2028	*Kabuli*	*Ca_Kabuli_Chr01*	45008039	(C/T)
CakSNP2029	*Kabuli*	*Ca_Kabuli_Chr01*	45008016	(T/A)
CakSNP2030	*Kabuli*	*Ca_Kabuli_Chr01*	45008015	(G/A)
CakSNP2031	*Kabuli*	*Ca_Kabuli_Chr01*	45008009	(C/T)
CakSNP2032	*Kabuli*	*Ca_Kabuli_Chr01*	45008006	(C/A)
CakSNP2033	*Kabuli*	*Ca_Kabuli_Chr01*	45008004	(C/T)
CakSNP2034	*Kabuli*	*Ca_Kabuli_Chr01*	45008074	(C/T)
CakSNP2035	*Kabuli*	*Ca_Kabuli_Chr01*	45250874	(G/A)
CakSNP2036	*Kabuli*	*Ca_Kabuli_Chr01*	45250872	(G/T)
CakSNP2037	*Kabuli*	*Ca_Kabuli_Chr01*	45250869	(G/A)
CakSNP2038	*Kabuli*	*Ca_Kabuli_Chr01*	45250863	(C/T)
CakSNP2039	*Kabuli*	*Ca_Kabuli_Chr01*	45250862	(A/T)
CakSNP2040	*Kabuli*	*Ca_Kabuli_Chr01*	45250834	(A/G)
CakSNP2041	*Kabuli*	*Ca_Kabuli_Chr01*	45250828	(G/C)
CakSNP2042	*Kabuli*	*Ca_Kabuli_Chr01*	45250807	(C/G)
CakSNP2043	*Kabuli*	*Ca_Kabuli_Chr01*	45250805	(A/C)
CakSNP2044	*Kabuli*	*Ca_Kabuli_Chr01*	45250803	(A/G)
CakSNP2045	*Kabuli*	*Ca_Kabuli_Chr01*	45250802	(C/T)
CakSNP2046	*Kabuli*	*Ca_Kabuli_Chr01*	46437023	(T/G)
CakSNP2047	*Kabuli*	*Ca_Kabuli_Chr01*	46523221	(C/T)
CakSNP2048	*Kabuli*	*Ca_Kabuli_Chr01*	46597131	(T/G)
CakSNP2049	*Kabuli*	*Ca_Kabuli_Chr01*	46745628	(C/A)

SNP IDs	Cultivars	Chromosomes/scaffolds	Physical positions (bp)	SNPs
CakSNP2050	*Kabuli*	*Ca_Kabuli_Chr01*	46778814	(A/C)
CakSNP2051	*Kabuli*	*Ca_Kabuli_Chr01*	46791939	(T/G)
CakSNP2052	*Kabuli*	*Ca_Kabuli_Chr01*	46793244	(G/A)
CakSNP2053	*Kabuli*	*Ca_Kabuli_Chr01*	46793357	(T/C)
CakSNP2054	*Kabuli*	*Ca_Kabuli_Chr01*	46793331	(C/G)
CakSNP2055	*Kabuli*	*Ca_Kabuli_Chr01*	46816041	(A/T)
CakSNP2056	*Kabuli*	*Ca_Kabuli_Chr01*	46825211	(G/A)
CakSNP2057	*Kabuli*	*Ca_Kabuli_Chr01*	46829662	(C/T)
CakSNP2058	*Kabuli*	*Ca_Kabuli_Chr01*	46832306	(T/G)
CakSNP2059	*Kabuli*	*Ca_Kabuli_Chr01*	46832395	(A/G)
CakSNP2060	*Kabuli*	*Ca_Kabuli_Chr01*	46832383	(T/C)
CakSNP2061	*Kabuli*	*Ca_Kabuli_Chr01*	46920524	(C/T)
CakSNP2062	*Kabuli*	*Ca_Kabuli_Chr01*	46920554	(T/C)
CakSNP2063	*Kabuli*	*Ca_Kabuli_Chr01*	46936196	(C/A)
CakSNP2064	*Kabuli*	*Ca_Kabuli_Chr01*	46936201	(C/T)
CakSNP2065	*Kabuli*	*Ca_Kabuli_Chr01*	46936207	(C/A)
CakSNP2066	*Kabuli*	*Ca_Kabuli_Chr01*	46936209	(C/T)
CakSNP2067	*Kabuli*	*Ca_Kabuli_Chr01*	46936329	(C/A)
CakSNP2068	*Kabuli*	*Ca_Kabuli_Chr01*	46936297	(A/G)
CakSNP2069	*Kabuli*	*Ca_Kabuli_Chr01*	46936428	(T/C)
CakSNP2070	*Kabuli*	*Ca_Kabuli_Chr01*	46936460	(A/C)
CakSNP2071	*Kabuli*	*Ca_Kabuli_Chr01*	46936456	(T/C)
CakSNP2072	*Kabuli*	*Ca_Kabuli_Chr01*	46936448	(A/C)
CakSNP2073	*Kabuli*	*Ca_Kabuli_Chr01*	46936447	(G/A)
CakSNP2074	*Kabuli*	*Ca_Kabuli_Chr01*	46941875	(T/C)
CakSNP2075	*Kabuli*	*Ca_Kabuli_Chr01*	46968740	(T/C)
CakSNP2076	*Kabuli*	*Ca_Kabuli_Chr01*	46969221	(T/A)
CakSNP2077	*Kabuli*	*Ca_Kabuli_Chr01*	46976508	(A/G)
CakSNP2078	*Kabuli*	*Ca_Kabuli_Chr01*	47042798	(T/A)
CakSNP2079	*Kabuli*	*Ca_Kabuli_Chr01*	47203123	(A/C)
CakSNP2080	*Kabuli*	*Ca_Kabuli_Chr01*	47263589	(C/T)
CakSNP2081	*Kabuli*	*Ca_Kabuli_Chr01*	47278505	(T/C)
CakSNP2082	*Kabuli*	*Ca_Kabuli_Chr01*	47331082	(T/C)
CakSNP2083	*Kabuli*	*Ca_Kabuli_Chr01*	47331080	(T/C)
CakSNP2084	*Kabuli*	*Ca_Kabuli_Chr01*	47423715	(A/T)
CakSNP2085	*Kabuli*	*Ca_Kabuli_Chr01*	47449818	(A/G)
CakSNP2086	*Kabuli*	*Ca_Kabuli_Chr01*	47449950	(T/C)
CakSNP2087	*Kabuli*	*Ca_Kabuli_Chr01*	47449891	(T/C)
CakSNP2088	*Kabuli*	*Ca_Kabuli_Chr01*	47449882	(A/C)
CakSNP2089	*Kabuli*	*Ca_Kabuli_Chr01*	47801491	(A/G)
CakSNP2090	*Kabuli*	*Ca_Kabuli_Chr01*	47890026	(C/T)

SNP IDs	Cultivars	Chromosomes/scaffolds	Physical positions (bp)	SNPs
CakSNP2091	*Kabuli*	*Ca_Kabuli_Chr01*	47897914	(C/T)
CakSNP2092	*Kabuli*	*Ca_Kabuli_Chr01*	47936748	(G/A)
CakSNP2093	*Kabuli*	*Ca_Kabuli_Chr01*	47936841	(A/G)
CakSNP2094	*Kabuli*	*Ca_Kabuli_Chr01*	47938884	(T/G)
CakSNP2095	*Kabuli*	*Ca_Kabuli_Chr01*	47947009	(T/C)
CakSNP2096	*Kabuli*	*Ca_Kabuli_Chr01*	47947281	(A/C)
CakSNP2097	*Kabuli*	*Ca_Kabuli_Chr01*	48026664	(G/T)
CakSNP2098	*Kabuli*	*Ca_Kabuli_Chr01*	48026701	(C/T)
CakSNP2099	*Kabuli*	*Ca_Kabuli_Chr01*	48026716	(T/G)
CakSNP2100	*Kabuli*	*Ca_Kabuli_Chr01*	48026727	(A/C)
CakSNP2101	*Kabuli*	*Ca_Kabuli_Chr01*	48027714	(C/A)
CakSNP2102	*Kabuli*	*Ca_Kabuli_Chr01*	48027869	(T/C)
CakSNP2103	*Kabuli*	*Ca_Kabuli_Chr01*	48028094	(G/A)
CakSNP2104	*Kabuli*	*Ca_Kabuli_Chr01*	48049293	(C/T)
CakSNP2105	*Kabuli*	*Ca_Kabuli_Chr01*	48132217	(G/A)
CakSNP2106	*Kabuli*	*Ca_Kabuli_Chr01*	48132521	(A/C)
CakSNP2107	*Kabuli*	*Ca_Kabuli_Chr01*	48154321	(A/C)
CakSNP2108	*Kabuli*	*Ca_Kabuli_Chr01*	48191460	(T/A)
CakSNP2109	*Kabuli*	*Ca_Kabuli_Chr01*	48195174	(T/A)
CakSNP2110	*Kabuli*	*Ca_Kabuli_Chr01*	48197895	(A/T)
CakSNP2111	*Kabuli*	*Ca_Kabuli_Chr01*	48197911	(T/G)
CakSNP2112	*Kabuli*	*Ca_Kabuli_Chr01*	48197979	(A/G)
CakSNP2113	*Kabuli*	*Ca_Kabuli_Chr01*	48197969	(G/A)
CakSNP2114	*Kabuli*	*Ca_Kabuli_Chr01*	48198932	(A/T)
CakSNP2115	*Kabuli*	*Ca_Kabuli_Chr01*	48265590	(T/G)
CakSNP2116	*Kabuli*	*Ca_Kabuli_Chr01*	48273097	(G/C)
CakSNP2117	*Kabuli*	*Ca_Kabuli_Chr01*	48273307	(C/T)
CakSNP2118	*Kabuli*	*Ca_Kabuli_Chr02*	113330	(C/T)
CakSNP2119	*Kabuli*	*Ca_Kabuli_Chr02*	149250	(C/A)
CakSNP2120	*Kabuli*	*Ca_Kabuli_Chr02*	149251	(A/T)
CakSNP2121	*Kabuli*	*Ca_Kabuli_Chr02*	149259	(A/C)
CakSNP2122	*Kabuli*	*Ca_Kabuli_Chr02*	149269	(C/T)
CakSNP2123	*Kabuli*	*Ca_Kabuli_Chr02*	149278	(G/T)
CakSNP2124	*Kabuli*	*Ca_Kabuli_Chr02*	149315	(C/A)
CakSNP2125	*Kabuli*	*Ca_Kabuli_Chr02*	149322	(C/A)
CakSNP2126	*Kabuli*	*Ca_Kabuli_Chr02*	149402	(C/T)
CakSNP2127	*Kabuli*	*Ca_Kabuli_Chr02*	149395	(T/C)
CakSNP2128	*Kabuli*	*Ca_Kabuli_Chr02*	189287	(A/G)
CakSNP2129	*Kabuli*	*Ca_Kabuli_Chr02*	189784	(C/A)
CakSNP2130	*Kabuli*	*Ca_Kabuli_Chr02*	221903	(A/T)
CakSNP2131	*Kabuli*	*Ca_Kabuli_Chr02*	221955	(A/G)

SNP IDs	Cultivars	Chromosomes/scaffolds	Physical positions (bp)	SNPs
CakSNP2132	*Kabuli*	*Ca_Kabuli_Chr02*	233589	(C/T)
CakSNP2133	*Kabuli*	*Ca_Kabuli_Chr02*	270139	(G/A)
CakSNP2134	*Kabuli*	*Ca_Kabuli_Chr02*	354795	(T/C)
CakSNP2135	*Kabuli*	*Ca_Kabuli_Chr02*	355905	(T/C)
CakSNP2136	*Kabuli*	*Ca_Kabuli_Chr02*	507857	(G/T)
CakSNP2137	*Kabuli*	*Ca_Kabuli_Chr02*	522943	(A/G)
CakSNP2138	*Kabuli*	*Ca_Kabuli_Chr02*	543360	(G/T)
CakSNP2139	*Kabuli*	*Ca_Kabuli_Chr02*	616251	(C/T)
CakSNP2140	*Kabuli*	*Ca_Kabuli_Chr02*	616410	(C/G)
CakSNP2141	*Kabuli*	*Ca_Kabuli_Chr02*	669304	(A/T)
CakSNP2142	*Kabuli*	*Ca_Kabuli_Chr02*	728957	(T/C)
CakSNP2143	*Kabuli*	*Ca_Kabuli_Chr02*	737183	(T/G)
CakSNP2144	*Kabuli*	*Ca_Kabuli_Chr02*	770164	(A/C)
CakSNP2145	*Kabuli*	*Ca_Kabuli_Chr02*	770183	(C/T)
CakSNP2146	*Kabuli*	*Ca_Kabuli_Chr02*	770234	(C/T)
CakSNP2147	*Kabuli*	*Ca_Kabuli_Chr02*	770237	(C/T)
CakSNP2148	*Kabuli*	*Ca_Kabuli_Chr02*	770225	(C/T)
CakSNP2149	*Kabuli*	*Ca_Kabuli_Chr02*	770260	(C/T)
CakSNP2150	*Kabuli*	*Ca_Kabuli_Chr02*	770266	(C/A)
CakSNP2151	*Kabuli*	*Ca_Kabuli_Chr02*	1231486	(A/G)
CakSNP2152	*Kabuli*	*Ca_Kabuli_Chr02*	1231500	(A/G)
CakSNP2153	*Kabuli*	*Ca_Kabuli_Chr02*	1231519	(T/C)
CakSNP2154	*Kabuli*	*Ca_Kabuli_Chr02*	1231618	(G/A)
CakSNP2155	*Kabuli*	*Ca_Kabuli_Chr02*	1234932	(C/T)
CakSNP2156	*Kabuli*	*Ca_Kabuli_Chr02*	1235018	(G/A)
CakSNP2157	*Kabuli*	*Ca_Kabuli_Chr02*	1234983	(T/C)
CakSNP2158	*Kabuli*	*Ca_Kabuli_Chr02*	1314367	(G/A)
CakSNP2159	*Kabuli*	*Ca_Kabuli_Chr02*	1368911	(A/G)
CakSNP2160	*Kabuli*	*Ca_Kabuli_Chr02*	1389661	(A/G)
CakSNP2161	*Kabuli*	*Ca_Kabuli_Chr02*	1397779	(C/T)
CakSNP2162	*Kabuli*	*Ca_Kabuli_Chr02*	1429186	(T/C)
CakSNP2163	*Kabuli*	*Ca_Kabuli_Chr02*	1429471	(G/C)
CakSNP2164	*Kabuli*	*Ca_Kabuli_Chr02*	1431368	(A/C)
CakSNP2165	*Kabuli*	*Ca_Kabuli_Chr02*	1493971	(A/G)
CakSNP2166	*Kabuli*	*Ca_Kabuli_Chr02*	1610477	(G/T)
CakSNP2167	*Kabuli*	*Ca_Kabuli_Chr02*	1668116	(A/G)
CakSNP2168	*Kabuli*	*Ca_Kabuli_Chr02*	1713343	(T/C)
CakSNP2169	*Kabuli*	*Ca_Kabuli_Chr02*	1797399	(A/G)
CakSNP2170	*Kabuli*	*Ca_Kabuli_Chr02*	1801341	(C/T)
CakSNP2171	*Kabuli*	*Ca_Kabuli_Chr02*	1828318	(T/C)
CakSNP2172	*Kabuli*	*Ca_Kabuli_Chr02*	1828406	(G/A)

**SNP IDs**	**Cultivars**	**Chromosomes/scaffolds**	**Physical positions (bp)**	**SNPs**
CakSNP2173	*Kabuli*	*Ca_Kabuli_Chr02*	1829772	(C/T)
CakSNP2174	*Kabuli*	*Ca_Kabuli_Chr02*	1835055	(G/A)
CakSNP2175	*Kabuli*	*Ca_Kabuli_Chr02*	1841290	(A/T)
CakSNP2176	*Kabuli*	*Ca_Kabuli_Chr02*	1841267	(C/T)
CakSNP2177	*Kabuli*	*Ca_Kabuli_Chr02*	1867041	(C/A)
CakSNP2178	*Kabuli*	*Ca_Kabuli_Chr02*	1882691	(C/T)
CakSNP2179	*Kabuli*	*Ca_Kabuli_Chr02*	1884583	(C/T)
CakSNP2180	*Kabuli*	*Ca_Kabuli_Chr02*	1884704	(A/G)
CakSNP2181	*Kabuli*	*Ca_Kabuli_Chr02*	1888657	(C/G)
CakSNP2182	*Kabuli*	*Ca_Kabuli_Chr02*	1953614	(T/C)
CakSNP2183	*Kabuli*	*Ca_Kabuli_Chr02*	1997914	(C/T)
CakSNP2184	*Kabuli*	*Ca_Kabuli_Chr02*	1998003	(C/T)
CakSNP2185	*Kabuli*	*Ca_Kabuli_Chr02*	2086763	(T/G)
CakSNP2186	*Kabuli*	*Ca_Kabuli_Chr02*	2220871	(C/T)
CakSNP2187	*Kabuli*	*Ca_Kabuli_Chr02*	2227118	(A/G)
CakSNP2188	*Kabuli*	*Ca_Kabuli_Chr02*	2227074	(T/C)
CakSNP2189	*Kabuli*	*Ca_Kabuli_Chr02*	2227465	(G/A)
CakSNP2190	*Kabuli*	*Ca_Kabuli_Chr02*	2230089	(G/A)
CakSNP2191	*Kabuli*	*Ca_Kabuli_Chr02*	2230056	(T/C)
CakSNP2192	*Kabuli*	*Ca_Kabuli_Chr02*	2230984	(T/G)
CakSNP2193	*Kabuli*	*Ca_Kabuli_Chr02*	2230980	(C/A)
CakSNP2194	*Kabuli*	*Ca_Kabuli_Chr02*	2230976	(T/G)
CakSNP2195	*Kabuli*	*Ca_Kabuli_Chr02*	2246678	(T/C)
CakSNP2196	*Kabuli*	*Ca_Kabuli_Chr02*	2311915	(C/A)
CakSNP2197	*Kabuli*	*Ca_Kabuli_Chr02*	2311917	(A/C)
CakSNP2198	*Kabuli*	*Ca_Kabuli_Chr02*	2312104	(C/T)
CakSNP2199	*Kabuli*	*Ca_Kabuli_Chr02*	2312037	(T/G)
CakSNP2200	*Kabuli*	*Ca_Kabuli_Chr02*	2344777	(G/A)
CakSNP2201	*Kabuli*	*Ca_Kabuli_Chr02*	2344778	(A/G)
CakSNP2202	*Kabuli*	*Ca_Kabuli_Chr02*	2344786	(G/A)
CakSNP2203	*Kabuli*	*Ca_Kabuli_Chr02*	2344835	(G/T)
CakSNP2204	*Kabuli*	*Ca_Kabuli_Chr02*	2414265	(A/G)
CakSNP2205	*Kabuli*	*Ca_Kabuli_Chr02*	2416422	(C/T)
CakSNP2206	*Kabuli*	*Ca_Kabuli_Chr02*	2441571	(G/A)
CakSNP2207	*Kabuli*	*Ca_Kabuli_Chr02*	2602831	(A/C)
CakSNP2208	*Kabuli*	*Ca_Kabuli_Chr02*	2650375	(T/C)
CakSNP2209	*Kabuli*	*Ca_Kabuli_Chr02*	2650373	(A/T)
CakSNP2210	*Kabuli*	*Ca_Kabuli_Chr02*	2652189	(A/T)
CakSNP2211	*Kabuli*	*Ca_Kabuli_Chr02*	2682235	(T/C)
CakSNP2212	*Kabuli*	*Ca_Kabuli_Chr02*	2682293	(T/C)
CakSNP2213	*Kabuli*	*Ca_Kabuli_Chr02*	2682321	(G/A)

SNP IDs	Cultivars	Chromosomes/scaffolds	Physical positions (bp)	SNPs
CakSNP2214	*Kabuli*	*Ca_Kabuli_Chr02*	2682375	(A/G)
CakSNP2215	*Kabuli*	*Ca_Kabuli_Chr02*	2682408	(G/A)
CakSNP2216	*Kabuli*	*Ca_Kabuli_Chr02*	2682471	(A/G)
CakSNP2217	*Kabuli*	*Ca_Kabuli_Chr02*	2685299	(T/C)
CakSNP2218	*Kabuli*	*Ca_Kabuli_Chr02*	2697688	(G/C)
CakSNP2219	*Kabuli*	*Ca_Kabuli_Chr02*	2708805	(G/C)
CakSNP2220	*Kabuli*	*Ca_Kabuli_Chr02*	2780283	(C/T)
CakSNP2221	*Kabuli*	*Ca_Kabuli_Chr02*	2783727	(G/A)
CakSNP2222	*Kabuli*	*Ca_Kabuli_Chr02*	2846667	(T/C)
CakSNP2223	*Kabuli*	*Ca_Kabuli_Chr02*	2846774	(C/A)
CakSNP2224	*Kabuli*	*Ca_Kabuli_Chr02*	2965867	(G/A)
CakSNP2225	*Kabuli*	*Ca_Kabuli_Chr02*	3099959	(C/A)
CakSNP2226	*Kabuli*	*Ca_Kabuli_Chr02*	3099997	(A/T)
CakSNP2227	*Kabuli*	*Ca_Kabuli_Chr02*	3100116	(A/G)
CakSNP2228	*Kabuli*	*Ca_Kabuli_Chr02*	3113560	(G/A)
CakSNP2229	*Kabuli*	*Ca_Kabuli_Chr02*	3141128	(A/C)
CakSNP2230	*Kabuli*	*Ca_Kabuli_Chr02*	3164404	(A/G)
CakSNP2231	*Kabuli*	*Ca_Kabuli_Chr02*	3164355	(G/A)
CakSNP2232	*Kabuli*	*Ca_Kabuli_Chr02*	3366023	(C/T)
CakSNP2233	*Kabuli*	*Ca_Kabuli_Chr02*	3410375	(C/T)
CakSNP2234	*Kabuli*	*Ca_Kabuli_Chr02*	3450904	(C/A)
CakSNP2235	*Kabuli*	*Ca_Kabuli_Chr02*	3503763	(T/G)
CakSNP2236	*Kabuli*	*Ca_Kabuli_Chr02*	3503759	(T/A)
CakSNP2237	*Kabuli*	*Ca_Kabuli_Chr02*	3503753	(C/G)
CakSNP2238	*Kabuli*	*Ca_Kabuli_Chr02*	3503750	(C/G)
CakSNP2239	*Kabuli*	*Ca_Kabuli_Chr02*	3789808	(G/T)
CakSNP2240	*Kabuli*	*Ca_Kabuli_Chr02*	3843814	(C/T)
CakSNP2241	*Kabuli*	*Ca_Kabuli_Chr02*	3843826	(T/C)
CakSNP2242	*Kabuli*	*Ca_Kabuli_Chr02*	3843836	(G/A)
CakSNP2243	*Kabuli*	*Ca_Kabuli_Chr02*	3852203	(C/A)
CakSNP2244	*Kabuli*	*Ca_Kabuli_Chr02*	3894041	(G/T)
CakSNP2245	*Kabuli*	*Ca_Kabuli_Chr02*	3904757	(G/A)
CakSNP2246	*Kabuli*	*Ca_Kabuli_Chr02*	3904761	(A/G)
CakSNP2247	*Kabuli*	*Ca_Kabuli_Chr02*	3908928	(A/G)
CakSNP2248	*Kabuli*	*Ca_Kabuli_Chr02*	3908955	(G/A)
CakSNP2249	*Kabuli*	*Ca_Kabuli_Chr02*	3909068	(G/A)
CakSNP2250	*Kabuli*	*Ca_Kabuli_Chr02*	3909062	(C/T)
CakSNP2251	*Kabuli*	*Ca_Kabuli_Chr02*	3915549	(A/G)
CakSNP2252	*Kabuli*	*Ca_Kabuli_Chr02*	3930211	(A/G)
CakSNP2253	*Kabuli*	*Ca_Kabuli_Chr02*	3930182	(A/G)
CakSNP2254	*Kabuli*	*Ca_Kabuli_Chr02*	3941397	(A/T)

SNP IDs	Cultivars	Chromosomes/scaffolds	Physical positions (bp)	SNPs
CakSNP2255	*Kabuli*	*Ca_Kabuli_Chr02*	3994150	(A/C)
CakSNP2256	*Kabuli*	*Ca_Kabuli_Chr02*	3998491	(A/C)
CakSNP2257	*Kabuli*	*Ca_Kabuli_Chr02*	3999615	(C/T)
CakSNP2258	*Kabuli*	*Ca_Kabuli_Chr02*	4039745	(T/C)
CakSNP2259	*Kabuli*	*Ca_Kabuli_Chr02*	4039854	(G/A)
CakSNP2260	*Kabuli*	*Ca_Kabuli_Chr02*	4325658	(A/C)
CakSNP2261	*Kabuli*	*Ca_Kabuli_Chr02*	4325684	(C/A)
CakSNP2262	*Kabuli*	*Ca_Kabuli_Chr02*	4404813	(C/T)
CakSNP2263	*Kabuli*	*Ca_Kabuli_Chr02*	4404797	(G/A)
CakSNP2264	*Kabuli*	*Ca_Kabuli_Chr02*	4966216	(C/T)
CakSNP2265	*Kabuli*	*Ca_Kabuli_Chr02*	4966168	(G/C)
CakSNP2266	*Kabuli*	*Ca_Kabuli_Chr02*	4981626	(A/G)
CakSNP2267	*Kabuli*	*Ca_Kabuli_Chr02*	4981698	(C/T)
CakSNP2268	*Kabuli*	*Ca_Kabuli_Chr02*	4981686	(T/C)
CakSNP2269	*Kabuli*	*Ca_Kabuli_Chr02*	4981666	(G/C)
CakSNP2270	*Kabuli*	*Ca_Kabuli_Chr02*	5017291	(T/A)
CakSNP2271	*Kabuli*	*Ca_Kabuli_Chr02*	5017330	(G/C)
CakSNP2272	*Kabuli*	*Ca_Kabuli_Chr02*	5078323	(T/C)
CakSNP2273	*Kabuli*	*Ca_Kabuli_Chr02*	5079639	(C/T)
CakSNP2274	*Kabuli*	*Ca_Kabuli_Chr02*	5085761	(A/C)
CakSNP2275	*Kabuli*	*Ca_Kabuli_Chr02*	5085836	(G/C)
CakSNP2276	*Kabuli*	*Ca_Kabuli_Chr02*	5100017	(T/A)
CakSNP2277	*Kabuli*	*Ca_Kabuli_Chr02*	5099985	(A/G)
CakSNP2278	*Kabuli*	*Ca_Kabuli_Chr02*	5118370	(A/T)
CakSNP2279	*Kabuli*	*Ca_Kabuli_Chr02*	5118364	(C/T)
CakSNP2280	*Kabuli*	*Ca_Kabuli_Chr02*	5141075	(C/T)
CakSNP2281	*Kabuli*	*Ca_Kabuli_Chr02*	5141081	(A/T)
CakSNP2282	*Kabuli*	*Ca_Kabuli_Chr02*	5247305	(T/C)
CakSNP2283	*Kabuli*	*Ca_Kabuli_Chr02*	5247548	(G/T)
CakSNP2284	*Kabuli*	*Ca_Kabuli_Chr02*	5247547	(A/G)
CakSNP2285	*Kabuli*	*Ca_Kabuli_Chr02*	5247540	(T/C)
CakSNP2286	*Kabuli*	*Ca_Kabuli_Chr02*	5247481	(T/A)
CakSNP2287	*Kabuli*	*Ca_Kabuli_Chr02*	5247557	(A/C)
CakSNP2288	*Kabuli*	*Ca_Kabuli_Chr02*	5247558	(G/T)
CakSNP2289	*Kabuli*	*Ca_Kabuli_Chr02*	5259254	(T/C)
CakSNP2290	*Kabuli*	*Ca_Kabuli_Chr02*	5276208	(T/C)
CakSNP2291	*Kabuli*	*Ca_Kabuli_Chr02*	5342864	(C/A)
CakSNP2292	*Kabuli*	*Ca_Kabuli_Chr02*	5342923	(A/G)
CakSNP2293	*Kabuli*	*Ca_Kabuli_Chr02*	5359177	(C/A)
CakSNP2294	*Kabuli*	*Ca_Kabuli_Chr02*	5449886	(C/T)
CakSNP2295	*Kabuli*	*Ca_Kabuli_Chr02*	5449978	(T/C)

SNP IDs	Cultivars	Chromosomes/scaffolds	Physical positions (bp)	SNPs
CakSNP2296	*Kabuli*	*Ca_Kabuli_Chr02*	5449907	(G/A)
CakSNP2297	*Kabuli*	*Ca_Kabuli_Chr02*	5462902	(A/T)
CakSNP2298	*Kabuli*	*Ca_Kabuli_Chr02*	5468575	(A/T)
CakSNP2299	*Kabuli*	*Ca_Kabuli_Chr02*	5475329	(C/A)
CakSNP2300	*Kabuli*	*Ca_Kabuli_Chr02*	5475344	(A/G)
CakSNP2301	*Kabuli*	*Ca_Kabuli_Chr02*	5475365	(T/C)
CakSNP2302	*Kabuli*	*Ca_Kabuli_Chr02*	5475407	(C/T)
CakSNP2303	*Kabuli*	*Ca_Kabuli_Chr02*	5504300	(T/G)
CakSNP2304	*Kabuli*	*Ca_Kabuli_Chr02*	5601092	(G/C)
CakSNP2305	*Kabuli*	*Ca_Kabuli_Chr02*	5787862	(G/A)
CakSNP2306	*Kabuli*	*Ca_Kabuli_Chr02*	5787887	(A/C)
CakSNP2307	*Kabuli*	*Ca_Kabuli_Chr02*	5877688	(A/G)
CakSNP2308	*Kabuli*	*Ca_Kabuli_Chr02*	5881503	(A/G)
CakSNP2309	*Kabuli*	*Ca_Kabuli_Chr02*	5897314	(T/C)
CakSNP2310	*Kabuli*	*Ca_Kabuli_Chr02*	5904368	(T/C)
CakSNP2311	*Kabuli*	*Ca_Kabuli_Chr02*	5936796	(C/A)
CakSNP2312	*Kabuli*	*Ca_Kabuli_Chr02*	5936902	(A/T)
CakSNP2313	*Kabuli*	*Ca_Kabuli_Chr02*	6042131	(G/A)
CakSNP2314	*Kabuli*	*Ca_Kabuli_Chr02*	6042139	(C/A)
CakSNP2315	*Kabuli*	*Ca_Kabuli_Chr02*	6042210	(C/A)
CakSNP2316	*Kabuli*	*Ca_Kabuli_Chr02*	6060259	(G/C)
CakSNP2317	*Kabuli*	*Ca_Kabuli_Chr02*	6130301	(G/C)
CakSNP2318	*Kabuli*	*Ca_Kabuli_Chr02*	6130257	(C/A)
CakSNP2319	*Kabuli*	*Ca_Kabuli_Chr02*	6130247	(G/C)
CakSNP2320	*Kabuli*	*Ca_Kabuli_Chr02*	6162378	(T/G)
CakSNP2321	*Kabuli*	*Ca_Kabuli_Chr02*	6162406	(G/C)
CakSNP2322	*Kabuli*	*Ca_Kabuli_Chr02*	6213582	(T/A)
CakSNP2323	*Kabuli*	*Ca_Kabuli_Chr02*	6213580	(C/A)
CakSNP2324	*Kabuli*	*Ca_Kabuli_Chr02*	6213566	(C/T)
CakSNP2325	*Kabuli*	*Ca_Kabuli_Chr02*	6213530	(C/A)
CakSNP2326	*Kabuli*	*Ca_Kabuli_Chr02*	6213520	(G/T)
CakSNP2327	*Kabuli*	*Ca_Kabuli_Chr02*	6349208	(T/G)
CakSNP2328	*Kabuli*	*Ca_Kabuli_Chr02*	6367649	(A/G)
CakSNP2329	*Kabuli*	*Ca_Kabuli_Chr02*	6367704	(A/G)
CakSNP2330	*Kabuli*	*Ca_Kabuli_Chr02*	6391819	(T/C)
CakSNP2331	*Kabuli*	*Ca_Kabuli_Chr02*	6392158	(A/T)
CakSNP2332	*Kabuli*	*Ca_Kabuli_Chr02*	6392209	(G/A)
CakSNP2333	*Kabuli*	*Ca_Kabuli_Chr02*	6392272	(T/C)
CakSNP2334	*Kabuli*	*Ca_Kabuli_Chr02*	6392254	(T/C)
CakSNP2335	*Kabuli*	*Ca_Kabuli_Chr02*	6392449	(A/G)
CakSNP2336	*Kabuli*	*Ca_Kabuli_Chr02*	6397551	(A/G)

SNP IDs	Cultivars	Chromosomes/scaffolds	Physical positions (bp)	SNPs
CakSNP2337	*Kabuli*	*Ca_Kabuli_Chr02*	6397597	(A/C)
CakSNP2338	*Kabuli*	*Ca_Kabuli_Chr02*	6408406	(C/A)
CakSNP2339	*Kabuli*	*Ca_Kabuli_Chr02*	6534776	(C/A)
CakSNP2340	*Kabuli*	*Ca_Kabuli_Chr02*	6537296	(G/T)
CakSNP2341	*Kabuli*	*Ca_Kabuli_Chr02*	6537300	(G/T)
CakSNP2342	*Kabuli*	*Ca_Kabuli_Chr02*	6727932	(G/C)
CakSNP2343	*Kabuli*	*Ca_Kabuli_Chr02*	6727905	(G/C)
CakSNP2344	*Kabuli*	*Ca_Kabuli_Chr02*	6727900	(G/C)
CakSNP2345	*Kabuli*	*Ca_Kabuli_Chr02*	6728716	(A/T)
CakSNP2346	*Kabuli*	*Ca_Kabuli_Chr02*	6728719	(C/T)
CakSNP2347	*Kabuli*	*Ca_Kabuli_Chr02*	6728763	(T/C)
CakSNP2348	*Kabuli*	*Ca_Kabuli_Chr02*	6989950	(A/G)
CakSNP2349	*Kabuli*	*Ca_Kabuli_Chr02*	6990083	(G/A)
CakSNP2350	*Kabuli*	*Ca_Kabuli_Chr02*	7007701	(G/C)
CakSNP2351	*Kabuli*	*Ca_Kabuli_Chr02*	7027093	(G/A)
CakSNP2352	*Kabuli*	*Ca_Kabuli_Chr02*	7027146	(T/C)
CakSNP2353	*Kabuli*	*Ca_Kabuli_Chr02*	7047195	(C/A)
CakSNP2354	*Kabuli*	*Ca_Kabuli_Chr02*	7047196	(T/C)
CakSNP2355	*Kabuli*	*Ca_Kabuli_Chr02*	7047197	(G/C)
CakSNP2356	*Kabuli*	*Ca_Kabuli_Chr02*	7051451	(G/A)
CakSNP2357	*Kabuli*	*Ca_Kabuli_Chr02*	7125992	(T/G)
CakSNP2358	*Kabuli*	*Ca_Kabuli_Chr02*	7126018	(T/G)
CakSNP2359	*Kabuli*	*Ca_Kabuli_Chr02*	7507259	(C/A)
CakSNP2360	*Kabuli*	*Ca_Kabuli_Chr02*	7633520	(G/A)
CakSNP2361	*Kabuli*	*Ca_Kabuli_Chr02*	7633547	(C/T)
CakSNP2362	*Kabuli*	*Ca_Kabuli_Chr02*	7633564	(G/T)
CakSNP2363	*Kabuli*	*Ca_Kabuli_Chr02*	7633572	(T/C)
CakSNP2364	*Kabuli*	*Ca_Kabuli_Chr02*	7633605	(T/A)
CakSNP2365	*Kabuli*	*Ca_Kabuli_Chr02*	7633592	(G/A)
CakSNP2366	*Kabuli*	*Ca_Kabuli_Chr02*	7633585	(G/A)
CakSNP2367	*Kabuli*	*Ca_Kabuli_Chr02*	7633869	(G/A)
CakSNP2368	*Kabuli*	*Ca_Kabuli_Chr02*	7633826	(G/A)
CakSNP2369	*Kabuli*	*Ca_Kabuli_Chr02*	7633818	(G/A)
CakSNP2370	*Kabuli*	*Ca_Kabuli_Chr02*	7633816	(G/A)
CakSNP2371	*Kabuli*	*Ca_Kabuli_Chr02*	7633813	(G/T)
CakSNP2372	*Kabuli*	*Ca_Kabuli_Chr02*	7633793	(C/T)
CakSNP2373	*Kabuli*	*Ca_Kabuli_Chr02*	7634041	(G/A)
CakSNP2374	*Kabuli*	*Ca_Kabuli_Chr02*	7679627	(A/T)
CakSNP2375	*Kabuli*	*Ca_Kabuli_Chr02*	7679693	(C/G)
CakSNP2376	*Kabuli*	*Ca_Kabuli_Chr02*	7701189	(T/G)
CakSNP2377	*Kabuli*	*Ca_Kabuli_Chr02*	7701199	(T/G)

SNP IDs	Cultivars	Chromosomes/scaffolds	Physical positions (bp)	SNPs
CakSNP2378	*Kabuli*	*Ca_Kabuli_Chr02*	7701216	(T/G)
CakSNP2379	*Kabuli*	*Ca_Kabuli_Chr02*	7701230	(T/G)
CakSNP2380	*Kabuli*	*Ca_Kabuli_Chr02*	7702468	(A/T)
CakSNP2381	*Kabuli*	*Ca_Kabuli_Chr02*	7708668	(C/T)
CakSNP2382	*Kabuli*	*Ca_Kabuli_Chr02*	7708663	(T/C)
CakSNP2383	*Kabuli*	*Ca_Kabuli_Chr02*	7708660	(G/A)
CakSNP2384	*Kabuli*	*Ca_Kabuli_Chr02*	7708630	(C/A)
CakSNP2385	*Kabuli*	*Ca_Kabuli_Chr02*	7708617	(G/T)
CakSNP2386	*Kabuli*	*Ca_Kabuli_Chr02*	7708603	(C/T)
CakSNP2387	*Kabuli*	*Ca_Kabuli_Chr02*	7708598	(C/T)
CakSNP2388	*Kabuli*	*Ca_Kabuli_Chr02*	7708596	(T/C)
CakSNP2389	*Kabuli*	*Ca_Kabuli_Chr02*	7708590	(A/T)
CakSNP2390	*Kabuli*	*Ca_Kabuli_Chr02*	7708678	(C/T)
CakSNP2391	*Kabuli*	*Ca_Kabuli_Chr02*	7712181	(C/T)
CakSNP2392	*Kabuli*	*Ca_Kabuli_Chr02*	7712155	(G/T)
CakSNP2393	*Kabuli*	*Ca_Kabuli_Chr02*	7714392	(G/A)
CakSNP2394	*Kabuli*	*Ca_Kabuli_Chr02*	7714454	(A/G)
CakSNP2395	*Kabuli*	*Ca_Kabuli_Chr02*	7714463	(T/C)
CakSNP2396	*Kabuli*	*Ca_Kabuli_Chr02*	7753419	(G/C)
CakSNP2397	*Kabuli*	*Ca_Kabuli_Chr02*	7753425	(T/C)
CakSNP2398	*Kabuli*	*Ca_Kabuli_Chr02*	7753440	(C/T)
CakSNP2399	*Kabuli*	*Ca_Kabuli_Chr02*	7819281	(T/C)
CakSNP2400	*Kabuli*	*Ca_Kabuli_Chr02*	7870919	(G/T)
CakSNP2401	*Kabuli*	*Ca_Kabuli_Chr02*	7870943	(T/G)
CakSNP2402	*Kabuli*	*Ca_Kabuli_Chr02*	7938890	(T/C)
CakSNP2403	*Kabuli*	*Ca_Kabuli_Chr02*	8337517	(C/T)
CakSNP2404	*Kabuli*	*Ca_Kabuli_Chr02*	8484840	(A/T)
CakSNP2405	*Kabuli*	*Ca_Kabuli_Chr02*	8487331	(T/A)
CakSNP2406	*Kabuli*	*Ca_Kabuli_Chr02*	8487343	(C/T)
CakSNP2407	*Kabuli*	*Ca_Kabuli_Chr02*	8489241	(C/A)
CakSNP2408	*Kabuli*	*Ca_Kabuli_Chr02*	8489237	(G/A)
CakSNP2409	*Kabuli*	*Ca_Kabuli_Chr02*	8489172	(C/A)
CakSNP2410	*Kabuli*	*Ca_Kabuli_Chr02*	8600256	(C/G)
CakSNP2411	*Kabuli*	*Ca_Kabuli_Chr02*	8600298	(A/G)
CakSNP2412	*Kabuli*	*Ca_Kabuli_Chr02*	8600309	(T/C)
CakSNP2413	*Kabuli*	*Ca_Kabuli_Chr02*	8600450	(C/T)
CakSNP2414	*Kabuli*	*Ca_Kabuli_Chr02*	8600666	(T/C)
CakSNP2415	*Kabuli*	*Ca_Kabuli_Chr02*	8685674	(G/A)
CakSNP2416	*Kabuli*	*Ca_Kabuli_Chr02*	8719725	(T/A)
CakSNP2417	*Kabuli*	*Ca_Kabuli_Chr02*	8734136	(G/A)
CakSNP2418	*Kabuli*	*Ca_Kabuli_Chr02*	8802884	(A/G)

SNP IDs	Cultivars	Chromosomes/scaffolds	Physical positions (bp)	SNPs
CakSNP2419	*Kabuli*	*Ca_Kabuli_Chr02*	9458933	(C/T)
CakSNP2420	*Kabuli*	*Ca_Kabuli_Chr02*	9458985	(A/G)
CakSNP2421	*Kabuli*	*Ca_Kabuli_Chr02*	9473339	(T/C)
CakSNP2422	*Kabuli*	*Ca_Kabuli_Chr02*	9473353	(C/A)
CakSNP2423	*Kabuli*	*Ca_Kabuli_Chr02*	9473362	(G/A)
CakSNP2424	*Kabuli*	*Ca_Kabuli_Chr02*	9473380	(A/G)
CakSNP2425	*Kabuli*	*Ca_Kabuli_Chr02*	9473393	(G/A)
CakSNP2426	*Kabuli*	*Ca_Kabuli_Chr02*	9473474	(T/A)
CakSNP2427	*Kabuli*	*Ca_Kabuli_Chr02*	9473452	(A/G)
CakSNP2428	*Kabuli*	*Ca_Kabuli_Chr02*	9473430	(A/G)
CakSNP2429	*Kabuli*	*Ca_Kabuli_Chr02*	9621953	(A/C)
CakSNP2430	*Kabuli*	*Ca_Kabuli_Chr02*	9740018	(G/A)
CakSNP2431	*Kabuli*	*Ca_Kabuli_Chr02*	9740017	(C/T)
CakSNP2432	*Kabuli*	*Ca_Kabuli_Chr02*	9740010	(A/G)
CakSNP2433	*Kabuli*	*Ca_Kabuli_Chr02*	9740007	(A/G)
CakSNP2434	*Kabuli*	*Ca_Kabuli_Chr02*	9739980	(G/C)
CakSNP2435	*Kabuli*	*Ca_Kabuli_Chr02*	9739974	(T/C)
CakSNP2436	*Kabuli*	*Ca_Kabuli_Chr02*	9739959	(T/C)
CakSNP2437	*Kabuli*	*Ca_Kabuli_Chr02*	9739965	(A/G)
CakSNP2438	*Kabuli*	*Ca_Kabuli_Chr02*	9756955	(G/A)
CakSNP2439	*Kabuli*	*Ca_Kabuli_Chr02*	9756946	(C/T)
CakSNP2440	*Kabuli*	*Ca_Kabuli_Chr02*	9756910	(G/A)
CakSNP2441	*Kabuli*	*Ca_Kabuli_Chr02*	9756887	(G/T)
CakSNP2442	*Kabuli*	*Ca_Kabuli_Chr02*	9823224	(G/A)
CakSNP2443	*Kabuli*	*Ca_Kabuli_Chr02*	9823165	(T/C)
CakSNP2444	*Kabuli*	*Ca_Kabuli_Chr02*	9825526	(C/G)
CakSNP2445	*Kabuli*	*Ca_Kabuli_Chr02*	9841625	(A/G)
CakSNP2446	*Kabuli*	*Ca_Kabuli_Chr02*	9862794	(A/G)
CakSNP2447	*Kabuli*	*Ca_Kabuli_Chr02*	9906042	(T/G)
CakSNP2448	*Kabuli*	*Ca_Kabuli_Chr02*	9906077	(T/A)
CakSNP2449	*Kabuli*	*Ca_Kabuli_Chr02*	9918536	(T/A)
CakSNP2450	*Kabuli*	*Ca_Kabuli_Chr02*	9943370	(A/G)
CakSNP2451	*Kabuli*	*Ca_Kabuli_Chr02*	9943374	(C/T)
CakSNP2452	*Kabuli*	*Ca_Kabuli_Chr02*	9943404	(T/G)
CakSNP2453	*Kabuli*	*Ca_Kabuli_Chr02*	9986639	(C/T)
CakSNP2454	*Kabuli*	*Ca_Kabuli_Chr02*	9986683	(C/T)
CakSNP2455	*Kabuli*	*Ca_Kabuli_Chr02*	10004694	(G/A)
CakSNP2456	*Kabuli*	*Ca_Kabuli_Chr02*	10025451	(G/A)
CakSNP2457	*Kabuli*	*Ca_Kabuli_Chr02*	10033991	(T/C)
CakSNP2458	*Kabuli*	*Ca_Kabuli_Chr02*	10060196	(G/T)
CakSNP2459	*Kabuli*	*Ca_Kabuli_Chr02*	10060200	(G/T)

SNP IDs	Cultivars	Chromosomes/scaffolds	Physical positions (bp)	SNPs
CakSNP2460	*Kabuli*	*Ca_Kabuli_Chr02*	10060207	(C/T)
CakSNP2461	*Kabuli*	*Ca_Kabuli_Chr02*	10067093	(G/C)
CakSNP2462	*Kabuli*	*Ca_Kabuli_Chr02*	10067078	(T/G)
CakSNP2463	*Kabuli*	*Ca_Kabuli_Chr02*	10067061	(T/G)
CakSNP2464	*Kabuli*	*Ca_Kabuli_Chr02*	10067195	(T/A)
CakSNP2465	*Kabuli*	*Ca_Kabuli_Chr02*	10067262	(A/C)
CakSNP2466	*Kabuli*	*Ca_Kabuli_Chr02*	10067278	(G/T)
CakSNP2467	*Kabuli*	*Ca_Kabuli_Chr02*	10067285	(T/G)
CakSNP2468	*Kabuli*	*Ca_Kabuli_Chr02*	10067286	(A/T)
CakSNP2469	*Kabuli*	*Ca_Kabuli_Chr02*	10067451	(A/T)
CakSNP2470	*Kabuli*	*Ca_Kabuli_Chr02*	10067516	(G/A)
CakSNP2471	*Kabuli*	*Ca_Kabuli_Chr02*	10067538	(C/T)
CakSNP2472	*Kabuli*	*Ca_Kabuli_Chr02*	10068214	(A/G)
CakSNP2473	*Kabuli*	*Ca_Kabuli_Chr02*	10220396	(T/C)
CakSNP2474	*Kabuli*	*Ca_Kabuli_Chr02*	10270474	(C/A)
CakSNP2475	*Kabuli*	*Ca_Kabuli_Chr02*	10284426	(C/G)
CakSNP2476	*Kabuli*	*Ca_Kabuli_Chr02*	10294698	(A/G)
CakSNP2477	*Kabuli*	*Ca_Kabuli_Chr02*	10294635	(T/C)
CakSNP2478	*Kabuli*	*Ca_Kabuli_Chr02*	10322943	(T/C)
CakSNP2479	*Kabuli*	*Ca_Kabuli_Chr02*	10352705	(T/A)
CakSNP2480	*Kabuli*	*Ca_Kabuli_Chr02*	10358484	(C/T)
CakSNP2481	*Kabuli*	*Ca_Kabuli_Chr02*	10358496	(G/T)
CakSNP2482	*Kabuli*	*Ca_Kabuli_Chr02*	10377695	(G/A)
CakSNP2483	*Kabuli*	*Ca_Kabuli_Chr02*	10434727	(T/C)
CakSNP2484	*Kabuli*	*Ca_Kabuli_Chr02*	10434726	(G/C)
CakSNP2485	*Kabuli*	*Ca_Kabuli_Chr02*	10434714	(C/G)
CakSNP2486	*Kabuli*	*Ca_Kabuli_Chr02*	10540735	(C/T)
CakSNP2487	*Kabuli*	*Ca_Kabuli_Chr02*	10637631	(C/A)
CakSNP2488	*Kabuli*	*Ca_Kabuli_Chr02*	10656518	(A/T)
CakSNP2489	*Kabuli*	*Ca_Kabuli_Chr02*	10705902	(C/A)
CakSNP2490	*Kabuli*	*Ca_Kabuli_Chr02*	10705923	(T/A)
CakSNP2491	*Kabuli*	*Ca_Kabuli_Chr02*	10705979	(C/A)
CakSNP2492	*Kabuli*	*Ca_Kabuli_Chr02*	10705982	(C/T)
CakSNP2493	*Kabuli*	*Ca_Kabuli_Chr02*	10760939	(G/A)
CakSNP2494	*Kabuli*	*Ca_Kabuli_Chr02*	10761036	(C/G)
CakSNP2495	*Kabuli*	*Ca_Kabuli_Chr02*	11799472	(G/A)
CakSNP2496	*Kabuli*	*Ca_Kabuli_Chr02*	11799462	(G/A)
CakSNP2497	*Kabuli*	*Ca_Kabuli_Chr02*	12252285	(T/G)
CakSNP2498	*Kabuli*	*Ca_Kabuli_Chr02*	12252370	(A/G)
CakSNP2499	*Kabuli*	*Ca_Kabuli_Chr02*	12278583	(G/A)
CakSNP2500	*Kabuli*	*Ca_Kabuli_Chr02*	12278584	(T/C)

**SNP IDs**	**Cultivars**	**Chromosomes/scaffolds**	**Physical positions (bp)**	**SNPs**
CakSNP2501	*Kabuli*	*Ca_Kabuli_Chr02*	12278606	(T/C)
CakSNP2502	*Kabuli*	*Ca_Kabuli_Chr02*	12281599	(A/C)
CakSNP2503	*Kabuli*	*Ca_Kabuli_Chr02*	12357732	(A/G)
CakSNP2504	*Kabuli*	*Ca_Kabuli_Chr02*	12389166	(G/T)
CakSNP2505	*Kabuli*	*Ca_Kabuli_Chr02*	12389112	(G/A)
CakSNP2506	*Kabuli*	*Ca_Kabuli_Chr02*	12515595	(A/G)
CakSNP2507	*Kabuli*	*Ca_Kabuli_Chr02*	12528825	(C/G)
CakSNP2508	*Kabuli*	*Ca_Kabuli_Chr02*	12710137	(G/A)
CakSNP2509	*Kabuli*	*Ca_Kabuli_Chr02*	12737814	(T/C)
CakSNP2510	*Kabuli*	*Ca_Kabuli_Chr02*	12794438	(G/A)
CakSNP2511	*Kabuli*	*Ca_Kabuli_Chr02*	12854610	(A/G)
CakSNP2512	*Kabuli*	*Ca_Kabuli_Chr02*	12854591	(T/A)
CakSNP2513	*Kabuli*	*Ca_Kabuli_Chr02*	12854586	(G/A)
CakSNP2514	*Kabuli*	*Ca_Kabuli_Chr02*	12862414	(G/C)
CakSNP2515	*Kabuli*	*Ca_Kabuli_Chr02*	13079417	(T/G)
CakSNP2516	*Kabuli*	*Ca_Kabuli_Chr02*	13079445	(T/G)
CakSNP2517	*Kabuli*	*Ca_Kabuli_Chr02*	13217781	(T/C)
CakSNP2518	*Kabuli*	*Ca_Kabuli_Chr02*	13355018	(T/G)
CakSNP2519	*Kabuli*	*Ca_Kabuli_Chr02*	13355022	(G/T)
CakSNP2520	*Kabuli*	*Ca_Kabuli_Chr02*	13355033	(A/T)
CakSNP2521	*Kabuli*	*Ca_Kabuli_Chr02*	13355615	(T/G)
CakSNP2522	*Kabuli*	*Ca_Kabuli_Chr02*	13425568	(T/A)
CakSNP2523	*Kabuli*	*Ca_Kabuli_Chr02*	13434904	(G/A)
CakSNP2524	*Kabuli*	*Ca_Kabuli_Chr02*	13434884	(T/A)
CakSNP2525	*Kabuli*	*Ca_Kabuli_Chr02*	13435043	(C/T)
CakSNP2526	*Kabuli*	*Ca_Kabuli_Chr02*	13435030	(G/C)
CakSNP2527	*Kabuli*	*Ca_Kabuli_Chr02*	13434995	(A/C)
CakSNP2528	*Kabuli*	*Ca_Kabuli_Chr02*	13437317	(A/G)
CakSNP2529	*Kabuli*	*Ca_Kabuli_Chr02*	13529057	(G/A)
CakSNP2530	*Kabuli*	*Ca_Kabuli_Chr02*	13552505	(T/A)
CakSNP2531	*Kabuli*	*Ca_Kabuli_Chr02*	13554038	(T/C)
CakSNP2532	*Kabuli*	*Ca_Kabuli_Chr02*	13554045	(T/C)
CakSNP2533	*Kabuli*	*Ca_Kabuli_Chr02*	13554047	(A/T)
CakSNP2534	*Kabuli*	*Ca_Kabuli_Chr02*	13703083	(G/A)
CakSNP2535	*Kabuli*	*Ca_Kabuli_Chr02*	13782906	(C/A)
CakSNP2536	*Kabuli*	*Ca_Kabuli_Chr02*	14101913	(T/A)
CakSNP2537	*Kabuli*	*Ca_Kabuli_Chr02*	14134413	(A/C)
CakSNP2538	*Kabuli*	*Ca_Kabuli_Chr02*	14134676	(T/C)
CakSNP2539	*Kabuli*	*Ca_Kabuli_Chr02*	14136062	(C/A)
CakSNP2540	*Kabuli*	*Ca_Kabuli_Chr02*	14136019	(A/G)
CakSNP2541	*Kabuli*	*Ca_Kabuli_Chr02*	14136013	(T/G)

**SNP IDs**	**Cultivars**	**Chromosomes/scaffolds**	**Physical positions (bp)**	**SNPs**
CakSNP2542	*Kabuli*	*Ca_Kabuli_Chr02*	14136223	(A/C)
CakSNP2543	*Kabuli*	*Ca_Kabuli_Chr02*	15160301	(T/C)
CakSNP2544	*Kabuli*	*Ca_Kabuli_Chr02*	15178565	(G/A)
CakSNP2545	*Kabuli*	*Ca_Kabuli_Chr02*	15178564	(A/G)
CakSNP2546	*Kabuli*	*Ca_Kabuli_Chr02*	15178540	(A/G)
CakSNP2547	*Kabuli*	*Ca_Kabuli_Chr02*	15181635	(G/T)
CakSNP2548	*Kabuli*	*Ca_Kabuli_Chr02*	15234951	(C/A)
CakSNP2549	*Kabuli*	*Ca_Kabuli_Chr02*	15389412	(G/A)
CakSNP2550	*Kabuli*	*Ca_Kabuli_Chr02*	15390673	(A/G)
CakSNP2551	*Kabuli*	*Ca_Kabuli_Chr02*	15390731	(G/A)
CakSNP2552	*Kabuli*	*Ca_Kabuli_Chr02*	15390684	(A/G)
CakSNP2553	*Kabuli*	*Ca_Kabuli_Chr02*	15688657	(G/T)
CakSNP2554	*Kabuli*	*Ca_Kabuli_Chr02*	15817688	(T/C)
CakSNP2555	*Kabuli*	*Ca_Kabuli_Chr02*	16460174	(G/A)
CakSNP2556	*Kabuli*	*Ca_Kabuli_Chr02*	16460142	(A/G)
CakSNP2557	*Kabuli*	*Ca_Kabuli_Chr02*	16460097	(C/A)
CakSNP2558	*Kabuli*	*Ca_Kabuli_Chr02*	16460106	(C/T)
CakSNP2559	*Kabuli*	*Ca_Kabuli_Chr02*	16460175	(C/A)
CakSNP2560	*Kabuli*	*Ca_Kabuli_Chr02*	16462095	(A/G)
CakSNP2561	*Kabuli*	*Ca_Kabuli_Chr02*	16462089	(C/T)
CakSNP2562	*Kabuli*	*Ca_Kabuli_Chr02*	16462056	(A/C)
CakSNP2563	*Kabuli*	*Ca_Kabuli_Chr02*	16462038	(G/T)
CakSNP2564	*Kabuli*	*Ca_Kabuli_Chr02*	16555733	(C/T)
CakSNP2565	*Kabuli*	*Ca_Kabuli_Chr02*	16555790	(C/G)
CakSNP2566	*Kabuli*	*Ca_Kabuli_Chr02*	16563117	(A/T)
CakSNP2567	*Kabuli*	*Ca_Kabuli_Chr02*	16563121	(A/T)
CakSNP2568	*Kabuli*	*Ca_Kabuli_Chr02*	16563123	(C/T)
CakSNP2569	*Kabuli*	*Ca_Kabuli_Chr02*	16563125	(A/T)
CakSNP2570	*Kabuli*	*Ca_Kabuli_Chr02*	16563133	(A/T)
CakSNP2571	*Kabuli*	*Ca_Kabuli_Chr02*	16705119	(C/T)
CakSNP2572	*Kabuli*	*Ca_Kabuli_Chr02*	17450780	(C/T)
CakSNP2573	*Kabuli*	*Ca_Kabuli_Chr02*	17578623	(G/A)
CakSNP2574	*Kabuli*	*Ca_Kabuli_Chr02*	17578633	(C/G)
CakSNP2575	*Kabuli*	*Ca_Kabuli_Chr02*	17578659	(A/G)
CakSNP2576	*Kabuli*	*Ca_Kabuli_Chr02*	17578689	(G/A)
CakSNP2577	*Kabuli*	*Ca_Kabuli_Chr02*	17578663	(C/A)
CakSNP2578	*Kabuli*	*Ca_Kabuli_Chr02*	17578676	(T/C)
CakSNP2579	*Kabuli*	*Ca_Kabuli_Chr02*	17578701	(A/C)
CakSNP2580	*Kabuli*	*Ca_Kabuli_Chr02*	17578706	(A/T)
CakSNP2581	*Kabuli*	*Ca_Kabuli_Chr02*	17578726	(A/T)
CakSNP2582	*Kabuli*	*Ca_Kabuli_Chr02*	17578740	(A/G)

SNP IDs	Cultivars	Chromosomes/scaffolds	Physical positions (bp)	SNPs
CakSNP2583	*Kabuli*	*Ca_Kabuli_Chr02*	17681915	(C/T)
CakSNP2584	*Kabuli*	*Ca_Kabuli_Chr02*	17716022	(A/T)
CakSNP2585	*Kabuli*	*Ca_Kabuli_Chr02*	17727340	(G/A)
CakSNP2586	*Kabuli*	*Ca_Kabuli_Chr02*	17727362	(G/C)
CakSNP2587	*Kabuli*	*Ca_Kabuli_Chr02*	17727403	(C/T)
CakSNP2588	*Kabuli*	*Ca_Kabuli_Chr02*	17839209	(T/A)
CakSNP2589	*Kabuli*	*Ca_Kabuli_Chr02*	17839297	(T/C)
CakSNP2590	*Kabuli*	*Ca_Kabuli_Chr02*	17910111	(C/G)
CakSNP2591	*Kabuli*	*Ca_Kabuli_Chr02*	17910145	(C/T)
CakSNP2592	*Kabuli*	*Ca_Kabuli_Chr02*	17910783	(A/T)
CakSNP2593	*Kabuli*	*Ca_Kabuli_Chr02*	18312200	(T/C)
CakSNP2594	*Kabuli*	*Ca_Kabuli_Chr02*	18395345	(A/C)
CakSNP2595	*Kabuli*	*Ca_Kabuli_Chr02*	18395410	(T/C)
CakSNP2596	*Kabuli*	*Ca_Kabuli_Chr02*	18395378	(A/G)
CakSNP2597	*Kabuli*	*Ca_Kabuli_Chr02*	18546345	(T/C)
CakSNP2598	*Kabuli*	*Ca_Kabuli_Chr02*	18546366	(A/T)
CakSNP2599	*Kabuli*	*Ca_Kabuli_Chr02*	18631367	(T/A)
CakSNP2600	*Kabuli*	*Ca_Kabuli_Chr02*	18631383	(A/G)
CakSNP2601	*Kabuli*	*Ca_Kabuli_Chr02*	18631404	(T/G)
CakSNP2602	*Kabuli*	*Ca_Kabuli_Chr02*	18631412	(T/G)
CakSNP2603	*Kabuli*	*Ca_Kabuli_Chr02*	18631522	(A/C)
CakSNP2604	*Kabuli*	*Ca_Kabuli_Chr02*	18671692	(C/A)
CakSNP2605	*Kabuli*	*Ca_Kabuli_Chr02*	18671672	(T/A)
CakSNP2606	*Kabuli*	*Ca_Kabuli_Chr02*	18671666	(C/T)
CakSNP2607	*Kabuli*	*Ca_Kabuli_Chr02*	18671637	(G/A)
CakSNP2608	*Kabuli*	*Ca_Kabuli_Chr02*	18671618	(T/G)
CakSNP2609	*Kabuli*	*Ca_Kabuli_Chr02*	18671723	(C/G)
CakSNP2610	*Kabuli*	*Ca_Kabuli_Chr02*	18671746	(C/T)
CakSNP2611	*Kabuli*	*Ca_Kabuli_Chr02*	18671756	(T/A)
CakSNP2612	*Kabuli*	*Ca_Kabuli_Chr02*	18671765	(C/A)
CakSNP2613	*Kabuli*	*Ca_Kabuli_Chr02*	18671767	(G/C)
CakSNP2614	*Kabuli*	*Ca_Kabuli_Chr02*	18671785	(G/A)
CakSNP2615	*Kabuli*	*Ca_Kabuli_Chr02*	18671824	(C/T)
CakSNP2616	*Kabuli*	*Ca_Kabuli_Chr02*	20392224	(A/G)
CakSNP2617	*Kabuli*	*Ca_Kabuli_Chr02*	20762187	(A/G)
CakSNP2618	*Kabuli*	*Ca_Kabuli_Chr02*	20762205	(A/T)
CakSNP2619	*Kabuli*	*Ca_Kabuli_Chr02*	21000509	(C/T)
CakSNP2620	*Kabuli*	*Ca_Kabuli_Chr02*	21000466	(C/T)
CakSNP2621	*Kabuli*	*Ca_Kabuli_Chr02*	21000463	(C/T)
CakSNP2622	*Kabuli*	*Ca_Kabuli_Chr02*	21849874	(A/T)
CakSNP2623	*Kabuli*	*Ca_Kabuli_Chr02*	21906952	(A/G)

SNP IDs	Cultivars	Chromosomes/scaffolds	Physical positions (bp)	SNPs
CakSNP2624	*Kabuli*	*Ca_Kabuli_Chr02*	21965365	(T/C)
CakSNP2625	*Kabuli*	*Ca_Kabuli_Chr02*	22158593	(C/T)
CakSNP2626	*Kabuli*	*Ca_Kabuli_Chr02*	22158602	(C/T)
CakSNP2627	*Kabuli*	*Ca_Kabuli_Chr02*	22190700	(C/T)
CakSNP2628	*Kabuli*	*Ca_Kabuli_Chr02*	22190743	(C/T)
CakSNP2629	*Kabuli*	*Ca_Kabuli_Chr02*	22190748	(G/T)
CakSNP2630	*Kabuli*	*Ca_Kabuli_Chr02*	22190751	(C/T)
CakSNP2631	*Kabuli*	*Ca_Kabuli_Chr02*	22190756	(C/A)
CakSNP2632	*Kabuli*	*Ca_Kabuli_Chr02*	22190753	(C/T)
CakSNP2633	*Kabuli*	*Ca_Kabuli_Chr02*	22190790	(G/A)
CakSNP2634	*Kabuli*	*Ca_Kabuli_Chr02*	22190815	(C/T)
CakSNP2635	*Kabuli*	*Ca_Kabuli_Chr02*	22205024	(G/C)
CakSNP2636	*Kabuli*	*Ca_Kabuli_Chr02*	22347405	(C/T)
CakSNP2637	*Kabuli*	*Ca_Kabuli_Chr02*	22415930	(C/T)
CakSNP2638	*Kabuli*	*Ca_Kabuli_Chr02*	22416899	(A/C)
CakSNP2639	*Kabuli*	*Ca_Kabuli_Chr02*	22419501	(C/G)
CakSNP2640	*Kabuli*	*Ca_Kabuli_Chr02*	22419513	(T/C)
CakSNP2641	*Kabuli*	*Ca_Kabuli_Chr02*	22419514	(C/T)
CakSNP2642	*Kabuli*	*Ca_Kabuli_Chr02*	22419529	(T/C)
CakSNP2643	*Kabuli*	*Ca_Kabuli_Chr02*	22419625	(C/T)
CakSNP2644	*Kabuli*	*Ca_Kabuli_Chr02*	22419633	(C/T)
CakSNP2645	*Kabuli*	*Ca_Kabuli_Chr02*	22419634	(G/C)
CakSNP2646	*Kabuli*	*Ca_Kabuli_Chr02*	22419679	(G/A)
CakSNP2647	*Kabuli*	*Ca_Kabuli_Chr02*	22457234	(G/A)
CakSNP2648	*Kabuli*	*Ca_Kabuli_Chr02*	22457221	(T/A)
CakSNP2649	*Kabuli*	*Ca_Kabuli_Chr02*	22457215	(G/A)
CakSNP2650	*Kabuli*	*Ca_Kabuli_Chr02*	22457181	(G/T)
CakSNP2651	*Kabuli*	*Ca_Kabuli_Chr02*	22457233	(C/T)
CakSNP2652	*Kabuli*	*Ca_Kabuli_Chr02*	22457255	(C/T)
CakSNP2653	*Kabuli*	*Ca_Kabuli_Chr02*	22457263	(G/A)
CakSNP2654	*Kabuli*	*Ca_Kabuli_Chr02*	22457257	(G/A)
CakSNP2655	*Kabuli*	*Ca_Kabuli_Chr02*	22483072	(C/T)
CakSNP2656	*Kabuli*	*Ca_Kabuli_Chr02*	22483067	(G/A)
CakSNP2657	*Kabuli*	*Ca_Kabuli_Chr02*	22483052	(C/T)
CakSNP2658	*Kabuli*	*Ca_Kabuli_Chr02*	22483019	(A/T)
CakSNP2659	*Kabuli*	*Ca_Kabuli_Chr02*	22703091	(A/C)
CakSNP2660	*Kabuli*	*Ca_Kabuli_Chr02*	22820616	(C/G)
CakSNP2661	*Kabuli*	*Ca_Kabuli_Chr02*	22820588	(A/C)
CakSNP2662	*Kabuli*	*Ca_Kabuli_Chr02*	22897694	(G/A)
CakSNP2663	*Kabuli*	*Ca_Kabuli_Chr02*	22897647	(T/C)
CakSNP2664	*Kabuli*	*Ca_Kabuli_Chr02*	23123734	(C/T)

SNP IDs	Cultivars	Chromosomes/scaffolds	Physical positions (bp)	SNPs
CakSNP2665	*Kabuli*	*Ca_Kabuli_Chr02*	23141564	(C/T)
CakSNP2666	*Kabuli*	*Ca_Kabuli_Chr02*	23181353	(C/T)
CakSNP2667	*Kabuli*	*Ca_Kabuli_Chr02*	23247801	(C/T)
CakSNP2668	*Kabuli*	*Ca_Kabuli_Chr02*	23282374	(T/C)
CakSNP2669	*Kabuli*	*Ca_Kabuli_Chr02*	23297412	(G/A)
CakSNP2670	*Kabuli*	*Ca_Kabuli_Chr02*	23329325	(G/T)
CakSNP2671	*Kabuli*	*Ca_Kabuli_Chr02*	23329424	(C/T)
CakSNP2672	*Kabuli*	*Ca_Kabuli_Chr02*	23329440	(G/A)
CakSNP2673	*Kabuli*	*Ca_Kabuli_Chr02*	23417518	(T/G)
CakSNP2674	*Kabuli*	*Ca_Kabuli_Chr02*	23435939	(A/G)
CakSNP2675	*Kabuli*	*Ca_Kabuli_Chr02*	23439966	(T/C)
CakSNP2676	*Kabuli*	*Ca_Kabuli_Chr02*	23441740	(A/G)
CakSNP2677	*Kabuli*	*Ca_Kabuli_Chr02*	23910254	(T/C)
CakSNP2678	*Kabuli*	*Ca_Kabuli_Chr02*	23961003	(G/A)
CakSNP2679	*Kabuli*	*Ca_Kabuli_Chr02*	24048087	(A/C)
CakSNP2680	*Kabuli*	*Ca_Kabuli_Chr02*	24098243	(T/C)
CakSNP2681	*Kabuli*	*Ca_Kabuli_Chr02*	24101371	(C/T)
CakSNP2682	*Kabuli*	*Ca_Kabuli_Chr02*	24164335	(A/T)
CakSNP2683	*Kabuli*	*Ca_Kabuli_Chr02*	24164840	(T/A)
CakSNP2684	*Kabuli*	*Ca_Kabuli_Chr02*	24164903	(T/C)
CakSNP2685	*Kabuli*	*Ca_Kabuli_Chr02*	24164900	(A/T)
CakSNP2686	*Kabuli*	*Ca_Kabuli_Chr02*	24221498	(G/T)
CakSNP2687	*Kabuli*	*Ca_Kabuli_Chr02*	24291665	(T/C)
CakSNP2688	*Kabuli*	*Ca_Kabuli_Chr02*	24353303	(A/G)
CakSNP2689	*Kabuli*	*Ca_Kabuli_Chr02*	24353243	(A/T)
CakSNP2690	*Kabuli*	*Ca_Kabuli_Chr02*	24511053	(G/A)
CakSNP2691	*Kabuli*	*Ca_Kabuli_Chr02*	24511114	(G/A)
CakSNP2692	*Kabuli*	*Ca_Kabuli_Chr02*	24511121	(G/A)
CakSNP2693	*Kabuli*	*Ca_Kabuli_Chr02*	24511119	(T/G)
CakSNP2694	*Kabuli*	*Ca_Kabuli_Chr02*	24511116	(C/A)
CakSNP2695	*Kabuli*	*Ca_Kabuli_Chr02*	24586360	(T/C)
CakSNP2696	*Kabuli*	*Ca_Kabuli_Chr02*	24709014	(C/G)
CakSNP2697	*Kabuli*	*Ca_Kabuli_Chr02*	24709103	(G/A)
CakSNP2698	*Kabuli*	*Ca_Kabuli_Chr02*	24709139	(A/G)
CakSNP2699	*Kabuli*	*Ca_Kabuli_Chr02*	24709159	(T/A)
CakSNP2700	*Kabuli*	*Ca_Kabuli_Chr02*	24709295	(G/A)
CakSNP2701	*Kabuli*	*Ca_Kabuli_Chr02*	24741195	(C/T)
CakSNP2702	*Kabuli*	*Ca_Kabuli_Chr02*	24744726	(A/T)
CakSNP2703	*Kabuli*	*Ca_Kabuli_Chr02*	24817957	(G/A)
CakSNP2704	*Kabuli*	*Ca_Kabuli_Chr02*	24817945	(C/T)
CakSNP2705	*Kabuli*	*Ca_Kabuli_Chr02*	24817940	(G/A)

**SNP IDs**	**Cultivars**	**Chromosomes/scaffolds**	**Physical positions (bp)**	**SNPs**
CakSNP2706	*Kabuli*	*Ca_Kabuli_Chr02*	24817934	(A/G)
CakSNP2707	*Kabuli*	*Ca_Kabuli_Chr02*	24817921	(G/A)
CakSNP2708	*Kabuli*	*Ca_Kabuli_Chr02*	24876355	(T/G)
CakSNP2709	*Kabuli*	*Ca_Kabuli_Chr02*	24911080	(T/C)
CakSNP2710	*Kabuli*	*Ca_Kabuli_Chr02*	24911154	(G/C)
CakSNP2711	*Kabuli*	*Ca_Kabuli_Chr02*	25022971	(A/G)
CakSNP2712	*Kabuli*	*Ca_Kabuli_Chr02*	25104319	(G/T)
CakSNP2713	*Kabuli*	*Ca_Kabuli_Chr02*	25104291	(A/T)
CakSNP2714	*Kabuli*	*Ca_Kabuli_Chr02*	25108249	(T/C)
CakSNP2715	*Kabuli*	*Ca_Kabuli_Chr02*	25126798	(C/T)
CakSNP2716	*Kabuli*	*Ca_Kabuli_Chr02*	25126804	(A/G)
CakSNP2717	*Kabuli*	*Ca_Kabuli_Chr02*	25126837	(G/T)
CakSNP2718	*Kabuli*	*Ca_Kabuli_Chr02*	25126910	(G/C)
CakSNP2719	*Kabuli*	*Ca_Kabuli_Chr02*	25163062	(C/G)
CakSNP2720	*Kabuli*	*Ca_Kabuli_Chr02*	25194583	(G/A)
CakSNP2721	*Kabuli*	*Ca_Kabuli_Chr02*	25317391	(A/G)
CakSNP2722	*Kabuli*	*Ca_Kabuli_Chr02*	25321993	(C/A)
CakSNP2723	*Kabuli*	*Ca_Kabuli_Chr02*	25493973	(T/G)
CakSNP2724	*Kabuli*	*Ca_Kabuli_Chr02*	25507637	(C/G)
CakSNP2725	*Kabuli*	*Ca_Kabuli_Chr02*	25507681	(A/G)
CakSNP2726	*Kabuli*	*Ca_Kabuli_Chr02*	25508579	(C/A)
CakSNP2727	*Kabuli*	*Ca_Kabuli_Chr02*	25508679	(A/G)
CakSNP2728	*Kabuli*	*Ca_Kabuli_Chr02*	25508660	(G/C)
CakSNP2729	*Kabuli*	*Ca_Kabuli_Chr02*	25508920	(G/A)
CakSNP2730	*Kabuli*	*Ca_Kabuli_Chr02*	25508969	(T/C)
CakSNP2731	*Kabuli*	*Ca_Kabuli_Chr02*	25508964	(A/C)
CakSNP2732	*Kabuli*	*Ca_Kabuli_Chr02*	25508963	(A/G)
CakSNP2733	*Kabuli*	*Ca_Kabuli_Chr02*	25508935	(A/T)
CakSNP2734	*Kabuli*	*Ca_Kabuli_Chr02*	25509087	(C/A)
CakSNP2735	*Kabuli*	*Ca_Kabuli_Chr02*	25509093	(C/G)
CakSNP2736	*Kabuli*	*Ca_Kabuli_Chr02*	25509095	(G/A)
CakSNP2737	*Kabuli*	*Ca_Kabuli_Chr02*	25509203	(T/C)
CakSNP2738	*Kabuli*	*Ca_Kabuli_Chr02*	25509202	(T/A)
CakSNP2739	*Kabuli*	*Ca_Kabuli_Chr02*	25667417	(G/C)
CakSNP2740	*Kabuli*	*Ca_Kabuli_Chr02*	25667427	(T/C)
CakSNP2741	*Kabuli*	*Ca_Kabuli_Chr02*	25788124	(T/A)
CakSNP2742	*Kabuli*	*Ca_Kabuli_Chr02*	25788139	(T/C)
CakSNP2743	*Kabuli*	*Ca_Kabuli_Chr02*	25880318	(T/C)
CakSNP2744	*Kabuli*	*Ca_Kabuli_Chr02*	25898931	(T/C)
CakSNP2745	*Kabuli*	*Ca_Kabuli_Chr02*	25930490	(G/A)
CakSNP2746	*Kabuli*	*Ca_Kabuli_Chr02*	26051667	(T/G)

**SNP IDs**	**Cultivars**	**Chromosomes/scaffolds**	**Physical positions (bp)**	**SNPs**
CakSNP2747	*Kabuli*	*Ca_Kabuli_Chr02*	26092881	(C/G)
CakSNP2748	*Kabuli*	*Ca_Kabuli_Chr02*	26092913	(C/A)
CakSNP2749	*Kabuli*	*Ca_Kabuli_Chr02*	26092928	(T/G)
CakSNP2750	*Kabuli*	*Ca_Kabuli_Chr02*	26092991	(T/C)
CakSNP2751	*Kabuli*	*Ca_Kabuli_Chr02*	26121495	(A/C)
CakSNP2752	*Kabuli*	*Ca_Kabuli_Chr02*	26121491	(T/G)
CakSNP2753	*Kabuli*	*Ca_Kabuli_Chr02*	26417311	(T/A)
CakSNP2754	*Kabuli*	*Ca_Kabuli_Chr02*	26417281	(A/G)
CakSNP2755	*Kabuli*	*Ca_Kabuli_Chr02*	26417256	(T/C)
CakSNP2756	*Kabuli*	*Ca_Kabuli_Chr02*	26431061	(T/C)
CakSNP2757	*Kabuli*	*Ca_Kabuli_Chr02*	26431058	(G/C)
CakSNP2758	*Kabuli*	*Ca_Kabuli_Chr02*	26431052	(T/C)
CakSNP2759	*Kabuli*	*Ca_Kabuli_Chr02*	26461867	(C/G)
CakSNP2760	*Kabuli*	*Ca_Kabuli_Chr02*	26461986	(A/C)
CakSNP2761	*Kabuli*	*Ca_Kabuli_Chr02*	26461960	(T/A)
CakSNP2762	*Kabuli*	*Ca_Kabuli_Chr02*	26486049	(G/A)
CakSNP2763	*Kabuli*	*Ca_Kabuli_Chr02*	26486065	(C/T)
CakSNP2764	*Kabuli*	*Ca_Kabuli_Chr02*	26486109	(T/C)
CakSNP2765	*Kabuli*	*Ca_Kabuli_Chr02*	26502153	(T/C)
CakSNP2766	*Kabuli*	*Ca_Kabuli_Chr02*	26503262	(C/A)
CakSNP2767	*Kabuli*	*Ca_Kabuli_Chr02*	26567088	(T/C)
CakSNP2768	*Kabuli*	*Ca_Kabuli_Chr02*	26585842	(T/A)
CakSNP2769	*Kabuli*	*Ca_Kabuli_Chr02*	26601337	(C/A)
CakSNP2770	*Kabuli*	*Ca_Kabuli_Chr02*	26601331	(G/C)
CakSNP2771	*Kabuli*	*Ca_Kabuli_Chr02*	26706849	(A/G)
CakSNP2772	*Kabuli*	*Ca_Kabuli_Chr02*	27057582	(C/T)
CakSNP2773	*Kabuli*	*Ca_Kabuli_Chr02*	27063749	(C/A)
CakSNP2774	*Kabuli*	*Ca_Kabuli_Chr02*	27134574	(A/G)
CakSNP2775	*Kabuli*	*Ca_Kabuli_Chr02*	27134560	(G/T)
CakSNP2776	*Kabuli*	*Ca_Kabuli_Chr02*	27182855	(T/C)
CakSNP2777	*Kabuli*	*Ca_Kabuli_Chr02*	27317272	(A/T)
CakSNP2778	*Kabuli*	*Ca_Kabuli_Chr02*	27374064	(A/T)
CakSNP2779	*Kabuli*	*Ca_Kabuli_Chr02*	27374094	(C/A)
CakSNP2780	*Kabuli*	*Ca_Kabuli_Chr02*	27377809	(T/G)
CakSNP2781	*Kabuli*	*Ca_Kabuli_Chr02*	27623258	(A/G)
CakSNP2782	*Kabuli*	*Ca_Kabuli_Chr02*	27703356	(G/A)
CakSNP2783	*Kabuli*	*Ca_Kabuli_Chr02*	27724425	(G/T)
CakSNP2784	*Kabuli*	*Ca_Kabuli_Chr02*	27757490	(T/C)
CakSNP2785	*Kabuli*	*Ca_Kabuli_Chr02*	27757824	(A/G)
CakSNP2786	*Kabuli*	*Ca_Kabuli_Chr02*	27757804	(A/G)
CakSNP2787	*Kabuli*	*Ca_Kabuli_Chr02*	27757795	(T/G)

SNP IDs	Cultivars	Chromosomes/scaffolds	Physical positions (bp)	SNPs
CakSNP2788	*Kabuli*	*Ca_Kabuli_Chr02*	27758057	(C/T)
CakSNP2789	*Kabuli*	*Ca_Kabuli_Chr02*	27758058	(A/G)
CakSNP2790	*Kabuli*	*Ca_Kabuli_Chr02*	27758065	(C/G)
CakSNP2791	*Kabuli*	*Ca_Kabuli_Chr02*	27758093	(C/T)
CakSNP2792	*Kabuli*	*Ca_Kabuli_Chr02*	27766561	(T/A)
CakSNP2793	*Kabuli*	*Ca_Kabuli_Chr02*	27766623	(T/C)
CakSNP2794	*Kabuli*	*Ca_Kabuli_Chr02*	27766605	(A/C)
CakSNP2795	*Kabuli*	*Ca_Kabuli_Chr02*	27766594	(C/A)
CakSNP2796	*Kabuli*	*Ca_Kabuli_Chr02*	27807994	(A/G)
CakSNP2797	*Kabuli*	*Ca_Kabuli_Chr02*	27808070	(G/A)
CakSNP2798	*Kabuli*	*Ca_Kabuli_Chr02*	27824962	(G/A)
CakSNP2799	*Kabuli*	*Ca_Kabuli_Chr02*	27831340	(A/C)
CakSNP2800	*Kabuli*	*Ca_Kabuli_Chr02*	27831398	(A/G)
CakSNP2801	*Kabuli*	*Ca_Kabuli_Chr02*	27835871	(G/A)
CakSNP2802	*Kabuli*	*Ca_Kabuli_Chr02*	27835850	(G/A)
CakSNP2803	*Kabuli*	*Ca_Kabuli_Chr02*	27835843	(C/A)
CakSNP2804	*Kabuli*	*Ca_Kabuli_Chr02*	27835837	(C/T)
CakSNP2805	*Kabuli*	*Ca_Kabuli_Chr02*	27835832	(C/T)
CakSNP2806	*Kabuli*	*Ca_Kabuli_Chr02*	27847670	(A/C)
CakSNP2807	*Kabuli*	*Ca_Kabuli_Chr02*	28017163	(A/G)
CakSNP2808	*Kabuli*	*Ca_Kabuli_Chr02*	28018158	(T/C)
CakSNP2809	*Kabuli*	*Ca_Kabuli_Chr02*	28060867	(T/G)
CakSNP2810	*Kabuli*	*Ca_Kabuli_Chr02*	28060850	(A/G)
CakSNP2811	*Kabuli*	*Ca_Kabuli_Chr02*	28060879	(C/T)
CakSNP2812	*Kabuli*	*Ca_Kabuli_Chr02*	28060896	(T/C)
CakSNP2813	*Kabuli*	*Ca_Kabuli_Chr02*	28060921	(C/T)
CakSNP2814	*Kabuli*	*Ca_Kabuli_Chr02*	28060922	(A/G)
CakSNP2815	*Kabuli*	*Ca_Kabuli_Chr02*	28060928	(C/T)
CakSNP2816	*Kabuli*	*Ca_Kabuli_Chr02*	28060930	(T/G)
CakSNP2817	*Kabuli*	*Ca_Kabuli_Chr02*	28060940	(C/T)
CakSNP2818	*Kabuli*	*Ca_Kabuli_Chr02*	28061049	(G/T)
CakSNP2819	*Kabuli*	*Ca_Kabuli_Chr02*	28076664	(C/T)
CakSNP2820	*Kabuli*	*Ca_Kabuli_Chr02*	28076834	(C/T)
CakSNP2821	*Kabuli*	*Ca_Kabuli_Chr02*	28076851	(G/A)
CakSNP2822	*Kabuli*	*Ca_Kabuli_Chr02*	28084910	(C/T)
CakSNP2823	*Kabuli*	*Ca_Kabuli_Chr02*	28084913	(C/A)
CakSNP2824	*Kabuli*	*Ca_Kabuli_Chr02*	28084953	(C/T)
CakSNP2825	*Kabuli*	*Ca_Kabuli_Chr02*	28229031	(G/A)
CakSNP2826	*Kabuli*	*Ca_Kabuli_Chr02*	28229046	(G/A)
CakSNP2827	*Kabuli*	*Ca_Kabuli_Chr02*	28324577	(T/A)
CakSNP2828	*Kabuli*	*Ca_Kabuli_Chr02*	28352533	(C/A)

SNP IDs	Cultivars	Chromosomes/scaffolds	Physical positions (bp)	SNPs
CakSNP2829	*Kabuli*	*Ca_Kabuli_Chr02*	28407316	(T/C)
CakSNP2830	*Kabuli*	*Ca_Kabuli_Chr02*	28407324	(C/T)
CakSNP2831	*Kabuli*	*Ca_Kabuli_Chr02*	28534235	(T/C)
CakSNP2832	*Kabuli*	*Ca_Kabuli_Chr02*	28699149	(C/T)
CakSNP2833	*Kabuli*	*Ca_Kabuli_Chr02*	28699178	(T/C)
CakSNP2834	*Kabuli*	*Ca_Kabuli_Chr02*	28699185	(C/A)
CakSNP2835	*Kabuli*	*Ca_Kabuli_Chr02*	28699219	(T/G)
CakSNP2836	*Kabuli*	*Ca_Kabuli_Chr02*	28699229	(C/T)
CakSNP2837	*Kabuli*	*Ca_Kabuli_Chr02*	28699276	(T/C)
CakSNP2838	*Kabuli*	*Ca_Kabuli_Chr02*	28699383	(A/T)
CakSNP2839	*Kabuli*	*Ca_Kabuli_Chr02*	28699403	(C/T)
CakSNP2840	*Kabuli*	*Ca_Kabuli_Chr02*	28699414	(C/T)
CakSNP2841	*Kabuli*	*Ca_Kabuli_Chr02*	28699579	(A/C)
CakSNP2842	*Kabuli*	*Ca_Kabuli_Chr02*	28701724	(A/G)
CakSNP2843	*Kabuli*	*Ca_Kabuli_Chr02*	28741764	(T/C)
CakSNP2844	*Kabuli*	*Ca_Kabuli_Chr02*	28931402	(T/C)
CakSNP2845	*Kabuli*	*Ca_Kabuli_Chr02*	28931340	(T/C)
CakSNP2846	*Kabuli*	*Ca_Kabuli_Chr02*	28944712	(T/C)
CakSNP2847	*Kabuli*	*Ca_Kabuli_Chr02*	28944707	(A/C)
CakSNP2848	*Kabuli*	*Ca_Kabuli_Chr02*	28949900	(C/T)
CakSNP2849	*Kabuli*	*Ca_Kabuli_Chr02*	28949909	(G/A)
CakSNP2850	*Kabuli*	*Ca_Kabuli_Chr02*	28949911	(A/C)
CakSNP2851	*Kabuli*	*Ca_Kabuli_Chr02*	28949919	(A/T)
CakSNP2852	*Kabuli*	*Ca_Kabuli_Chr02*	28949954	(T/A)
CakSNP2853	*Kabuli*	*Ca_Kabuli_Chr02*	28949948	(A/G)
CakSNP2854	*Kabuli*	*Ca_Kabuli_Chr02*	28970621	(A/G)
CakSNP2855	*Kabuli*	*Ca_Kabuli_Chr02*	28970618	(A/G)
CakSNP2856	*Kabuli*	*Ca_Kabuli_Chr02*	29103993	(T/C)
CakSNP2857	*Kabuli*	*Ca_Kabuli_Chr02*	29103970	(C/T)
CakSNP2858	*Kabuli*	*Ca_Kabuli_Chr02*	29122581	(G/A)
CakSNP2859	*Kabuli*	*Ca_Kabuli_Chr02*	29122589	(T/G)
CakSNP2860	*Kabuli*	*Ca_Kabuli_Chr02*	29592666	(G/A)
CakSNP2861	*Kabuli*	*Ca_Kabuli_Chr02*	29592694	(T/C)
CakSNP2862	*Kabuli*	*Ca_Kabuli_Chr02*	29601458	(C/T)
CakSNP2863	*Kabuli*	*Ca_Kabuli_Chr02*	29601463	(C/T)
CakSNP2864	*Kabuli*	*Ca_Kabuli_Chr02*	29614370	(C/T)
CakSNP2865	*Kabuli*	*Ca_Kabuli_Chr02*	29614375	(C/T)
CakSNP2866	*Kabuli*	*Ca_Kabuli_Chr02*	29693107	(A/G)
CakSNP2867	*Kabuli*	*Ca_Kabuli_Chr02*	29948356	(G/A)
CakSNP2868	*Kabuli*	*Ca_Kabuli_Chr02*	29960049	(G/A)
CakSNP2869	*Kabuli*	*Ca_Kabuli_Chr02*	29960054	(G/A)

**SNP IDs**	**Cultivars**	**Chromosomes/scaffolds**	**Physical positions (bp)**	**SNPs**
CakSNP2870	*Kabuli*	*Ca_Kabuli_Chr02*	29960130	(C/T)
CakSNP2871	*Kabuli*	*Ca_Kabuli_Chr02*	30042255	(A/C)
CakSNP2872	*Kabuli*	*Ca_Kabuli_Chr02*	30042309	(T/G)
CakSNP2873	*Kabuli*	*Ca_Kabuli_Chr02*	30042302	(A/T)
CakSNP2874	*Kabuli*	*Ca_Kabuli_Chr02*	30042297	(C/T)
CakSNP2875	*Kabuli*	*Ca_Kabuli_Chr02*	30042267	(C/T)
CakSNP2876	*Kabuli*	*Ca_Kabuli_Chr02*	30045338	(C/G)
CakSNP2877	*Kabuli*	*Ca_Kabuli_Chr02*	30091313	(G/A)
CakSNP2878	*Kabuli*	*Ca_Kabuli_Chr02*	30091311	(C/T)
CakSNP2879	*Kabuli*	*Ca_Kabuli_Chr02*	30091308	(A/T)
CakSNP2880	*Kabuli*	*Ca_Kabuli_Chr02*	30091307	(C/T)
CakSNP2881	*Kabuli*	*Ca_Kabuli_Chr02*	30091295	(T/A)
CakSNP2882	*Kabuli*	*Ca_Kabuli_Chr02*	30091290	(T/C)
CakSNP2883	*Kabuli*	*Ca_Kabuli_Chr02*	30091286	(G/A)
CakSNP2884	*Kabuli*	*Ca_Kabuli_Chr02*	30091284	(A/C)
CakSNP2885	*Kabuli*	*Ca_Kabuli_Chr02*	30091280	(C/G)
CakSNP2886	*Kabuli*	*Ca_Kabuli_Chr02*	30133162	(G/A)
CakSNP2887	*Kabuli*	*Ca_Kabuli_Chr02*	30133189	(G/A)
CakSNP2888	*Kabuli*	*Ca_Kabuli_Chr02*	30133215	(G/T)
CakSNP2889	*Kabuli*	*Ca_Kabuli_Chr02*	30133221	(A/T)
CakSNP2890	*Kabuli*	*Ca_Kabuli_Chr02*	30335160	(T/C)
CakSNP2891	*Kabuli*	*Ca_Kabuli_Chr02*	30335212	(T/A)
CakSNP2892	*Kabuli*	*Ca_Kabuli_Chr02*	30335194	(A/G)
CakSNP2893	*Kabuli*	*Ca_Kabuli_Chr02*	30363957	(C/A)
CakSNP2894	*Kabuli*	*Ca_Kabuli_Chr02*	30364073	(T/A)
CakSNP2895	*Kabuli*	*Ca_Kabuli_Chr02*	30364042	(A/G)
CakSNP2896	*Kabuli*	*Ca_Kabuli_Chr02*	30364025	(T/C)
CakSNP2897	*Kabuli*	*Ca_Kabuli_Chr02*	30370013	(C/A)
CakSNP2898	*Kabuli*	*Ca_Kabuli_Chr02*	30370003	(A/C)
CakSNP2899	*Kabuli*	*Ca_Kabuli_Chr02*	30370411	(T/C)
CakSNP2900	*Kabuli*	*Ca_Kabuli_Chr02*	30467552	(C/T)
CakSNP2901	*Kabuli*	*Ca_Kabuli_Chr02*	30467551	(A/T)
CakSNP2902	*Kabuli*	*Ca_Kabuli_Chr02*	30508454	(T/C)
CakSNP2903	*Kabuli*	*Ca_Kabuli_Chr02*	30530642	(A/T)
CakSNP2904	*Kabuli*	*Ca_Kabuli_Chr02*	30537667	(A/T)
CakSNP2905	*Kabuli*	*Ca_Kabuli_Chr02*	30537673	(A/G)
CakSNP2906	*Kabuli*	*Ca_Kabuli_Chr02*	30537687	(T/C)
CakSNP2907	*Kabuli*	*Ca_Kabuli_Chr02*	30537701	(T/A)
CakSNP2908	*Kabuli*	*Ca_Kabuli_Chr02*	30537953	(T/C)
CakSNP2909	*Kabuli*	*Ca_Kabuli_Chr02*	30614086	(C/T)
CakSNP2910	*Kabuli*	*Ca_Kabuli_Chr02*	30686920	(C/T)

SNP IDs	Cultivars	Chromosomes/scaffolds	Physical positions (bp)	SNPs
CakSNP2911	*Kabuli*	*Ca_Kabuli_Chr02*	30702121	(T/C)
CakSNP2912	*Kabuli*	*Ca_Kabuli_Chr02*	30702090	(G/A)
CakSNP2913	*Kabuli*	*Ca_Kabuli_Chr02*	30867547	(G/A)
CakSNP2914	*Kabuli*	*Ca_Kabuli_Chr02*	30880148	(A/G)
CakSNP2915	*Kabuli*	*Ca_Kabuli_Chr02*	30880243	(G/C)
CakSNP2916	*Kabuli*	*Ca_Kabuli_Chr02*	30880246	(G/A)
CakSNP2917	*Kabuli*	*Ca_Kabuli_Chr02*	30880303	(T/A)
CakSNP2918	*Kabuli*	*Ca_Kabuli_Chr02*	30880302	(T/C)
CakSNP2919	*Kabuli*	*Ca_Kabuli_Chr02*	30880282	(A/G)
CakSNP2920	*Kabuli*	*Ca_Kabuli_Chr02*	30921092	(C/A)
CakSNP2921	*Kabuli*	*Ca_Kabuli_Chr02*	30921758	(C/T)
CakSNP2922	*Kabuli*	*Ca_Kabuli_Chr02*	30921888	(T/C)
CakSNP2923	*Kabuli*	*Ca_Kabuli_Chr02*	30921882	(C/G)
CakSNP2924	*Kabuli*	*Ca_Kabuli_Chr02*	30921917	(C/A)
CakSNP2925	*Kabuli*	*Ca_Kabuli_Chr02*	30921923	(A/G)
CakSNP2926	*Kabuli*	*Ca_Kabuli_Chr02*	30954317	(G/A)
CakSNP2927	*Kabuli*	*Ca_Kabuli_Chr02*	30954305	(T/G)
CakSNP2928	*Kabuli*	*Ca_Kabuli_Chr02*	30977572	(A/G)
CakSNP2929	*Kabuli*	*Ca_Kabuli_Chr02*	30977528	(C/T)
CakSNP2930	*Kabuli*	*Ca_Kabuli_Chr02*	30977522	(A/T)
CakSNP2931	*Kabuli*	*Ca_Kabuli_Chr02*	30977508	(A/T)
CakSNP2932	*Kabuli*	*Ca_Kabuli_Chr02*	30997769	(T/A)
CakSNP2933	*Kabuli*	*Ca_Kabuli_Chr02*	30997780	(A/C)
CakSNP2934	*Kabuli*	*Ca_Kabuli_Chr02*	30997784	(T/A)
CakSNP2935	*Kabuli*	*Ca_Kabuli_Chr02*	31005847	(T/G)
CakSNP2936	*Kabuli*	*Ca_Kabuli_Chr02*	31005845	(C/T)
CakSNP2937	*Kabuli*	*Ca_Kabuli_Chr02*	31014295	(A/T)
CakSNP2938	*Kabuli*	*Ca_Kabuli_Chr02*	31055083	(C/T)
CakSNP2939	*Kabuli*	*Ca_Kabuli_Chr02*	31055007	(G/A)
CakSNP2940	*Kabuli*	*Ca_Kabuli_Chr02*	31088681	(G/T)
CakSNP2941	*Kabuli*	*Ca_Kabuli_Chr02*	31088676	(G/C)
CakSNP2942	*Kabuli*	*Ca_Kabuli_Chr02*	31088670	(C/T)
CakSNP2943	*Kabuli*	*Ca_Kabuli_Chr02*	31088648	(A/G)
CakSNP2944	*Kabuli*	*Ca_Kabuli_Chr02*	31088645	(A/C)
CakSNP2945	*Kabuli*	*Ca_Kabuli_Chr02*	31088778	(A/T)
CakSNP2946	*Kabuli*	*Ca_Kabuli_Chr02*	31088800	(T/C)
CakSNP2947	*Kabuli*	*Ca_Kabuli_Chr02*	31088882	(A/G)
CakSNP2948	*Kabuli*	*Ca_Kabuli_Chr02*	31109311	(G/T)
CakSNP2949	*Kabuli*	*Ca_Kabuli_Chr02*	31109358	(C/T)
CakSNP2950	*Kabuli*	*Ca_Kabuli_Chr02*	31109481	(G/A)
CakSNP2951	*Kabuli*	*Ca_Kabuli_Chr02*	31109441	(C/G)

SNP IDs	Cultivars	Chromosomes/scaffolds	Physical positions (bp)	SNPs
CakSNP2952	*Kabuli*	*Ca_Kabuli_Chr02*	31109435	(G/A)
CakSNP2953	*Kabuli*	*Ca_Kabuli_Chr02*	31109427	(C/T)
CakSNP2954	*Kabuli*	*Ca_Kabuli_Chr02*	31141019	(G/A)
CakSNP2955	*Kabuli*	*Ca_Kabuli_Chr02*	31167603	(G/A)
CakSNP2956	*Kabuli*	*Ca_Kabuli_Chr02*	31167594	(A/G)
CakSNP2957	*Kabuli*	*Ca_Kabuli_Chr02*	31188355	(A/G)
CakSNP2958	*Kabuli*	*Ca_Kabuli_Chr02*	31201872	(T/C)
CakSNP2959	*Kabuli*	*Ca_Kabuli_Chr02*	31201859	(T/A)
CakSNP2960	*Kabuli*	*Ca_Kabuli_Chr02*	31201795	(A/G)
CakSNP2961	*Kabuli*	*Ca_Kabuli_Chr02*	31203154	(G/A)
CakSNP2962	*Kabuli*	*Ca_Kabuli_Chr02*	31205531	(C/T)
CakSNP2963	*Kabuli*	*Ca_Kabuli_Chr02*	31427861	(A/G)
CakSNP2964	*Kabuli*	*Ca_Kabuli_Chr02*	31427902	(G/C)
CakSNP2965	*Kabuli*	*Ca_Kabuli_Chr02*	31427915	(A/G)
CakSNP2966	*Kabuli*	*Ca_Kabuli_Chr02*	31428024	(T/G)
CakSNP2967	*Kabuli*	*Ca_Kabuli_Chr02*	31428132	(G/A)
CakSNP2968	*Kabuli*	*Ca_Kabuli_Chr02*	31428405	(T/C)
CakSNP2969	*Kabuli*	*Ca_Kabuli_Chr02*	31428429	(T/G)
CakSNP2970	*Kabuli*	*Ca_Kabuli_Chr02*	31534265	(A/C)
CakSNP2971	*Kabuli*	*Ca_Kabuli_Chr02*	31534283	(A/G)
CakSNP2972	*Kabuli*	*Ca_Kabuli_Chr02*	31534455	(T/C)
CakSNP2973	*Kabuli*	*Ca_Kabuli_Chr02*	31686714	(C/T)
CakSNP2974	*Kabuli*	*Ca_Kabuli_Chr02*	31686716	(C/T)
CakSNP2975	*Kabuli*	*Ca_Kabuli_Chr02*	31686742	(A/C)
CakSNP2976	*Kabuli*	*Ca_Kabuli_Chr02*	31686751	(A/T)
CakSNP2977	*Kabuli*	*Ca_Kabuli_Chr02*	31686758	(C/A)
CakSNP2978	*Kabuli*	*Ca_Kabuli_Chr02*	31686761	(A/C)
CakSNP2979	*Kabuli*	*Ca_Kabuli_Chr02*	31686767	(C/T)
CakSNP2980	*Kabuli*	*Ca_Kabuli_Chr02*	31686779	(G/A)
CakSNP2981	*Kabuli*	*Ca_Kabuli_Chr02*	31686820	(T/C)
CakSNP2982	*Kabuli*	*Ca_Kabuli_Chr02*	31756777	(G/T)
CakSNP2983	*Kabuli*	*Ca_Kabuli_Chr02*	31847033	(A/C)
CakSNP2984	*Kabuli*	*Ca_Kabuli_Chr02*	31881918	(G/T)
CakSNP2985	*Kabuli*	*Ca_Kabuli_Chr02*	31885934	(A/G)
CakSNP2986	*Kabuli*	*Ca_Kabuli_Chr02*	31908694	(G/A)
CakSNP2987	*Kabuli*	*Ca_Kabuli_Chr02*	31908742	(G/A)
CakSNP2988	*Kabuli*	*Ca_Kabuli_Chr02*	31908803	(C/T)
CakSNP2989	*Kabuli*	*Ca_Kabuli_Chr02*	31908787	(G/A)
CakSNP2990	*Kabuli*	*Ca_Kabuli_Chr02*	31908845	(T/A)
CakSNP2991	*Kabuli*	*Ca_Kabuli_Chr02*	31908857	(C/T)
CakSNP2992	*Kabuli*	*Ca_Kabuli_Chr02*	31941463	(T/C)

SNP IDs	Cultivars	Chromosomes/scaffolds	Physical positions (bp)	SNPs
CakSNP2993	*Kabuli*	*Ca_Kabuli_Chr02*	31941554	(A/T)
CakSNP2994	*Kabuli*	*Ca_Kabuli_Chr02*	31975269	(C/T)
CakSNP2995	*Kabuli*	*Ca_Kabuli_Chr02*	32003536	(G/T)
CakSNP2996	*Kabuli*	*Ca_Kabuli_Chr02*	32017361	(G/A)
CakSNP2997	*Kabuli*	*Ca_Kabuli_Chr02*	32049523	(C/A)
CakSNP2998	*Kabuli*	*Ca_Kabuli_Chr02*	32049525	(A/C)
CakSNP2999	*Kabuli*	*Ca_Kabuli_Chr02*	32058591	(T/C)
CakSNP3000	*Kabuli*	*Ca_Kabuli_Chr02*	32061053	(C/T)
CakSNP3001	*Kabuli*	*Ca_Kabuli_Chr02*	32061054	(T/C)
CakSNP3002	*Kabuli*	*Ca_Kabuli_Chr02*	32062010	(A/C)
CakSNP3003	*Kabuli*	*Ca_Kabuli_Chr02*	32071107	(C/G)
CakSNP3004	*Kabuli*	*Ca_Kabuli_Chr02*	32080002	(T/C)
CakSNP3005	*Kabuli*	*Ca_Kabuli_Chr02*	32080007	(T/C)
CakSNP3006	*Kabuli*	*Ca_Kabuli_Chr02*	32080010	(G/C)
CakSNP3007	*Kabuli*	*Ca_Kabuli_Chr02*	32080016	(A/T)
CakSNP3008	*Kabuli*	*Ca_Kabuli_Chr02*	32080022	(C/A)
CakSNP3009	*Kabuli*	*Ca_Kabuli_Chr02*	32080037	(A/G)
CakSNP3010	*Kabuli*	*Ca_Kabuli_Chr02*	32096192	(A/C)
CakSNP3011	*Kabuli*	*Ca_Kabuli_Chr02*	32096186	(T/A)
CakSNP3012	*Kabuli*	*Ca_Kabuli_Chr02*	32096180	(C/G)
CakSNP3013	*Kabuli*	*Ca_Kabuli_Chr02*	32096177	(C/T)
CakSNP3014	*Kabuli*	*Ca_Kabuli_Chr02*	32096172	(C/T)
CakSNP3015	*Kabuli*	*Ca_Kabuli_Chr02*	32099757	(C/T)
CakSNP3016	*Kabuli*	*Ca_Kabuli_Chr02*	32099907	(T/C)
CakSNP3017	*Kabuli*	*Ca_Kabuli_Chr02*	32109805	(G/A)
CakSNP3018	*Kabuli*	*Ca_Kabuli_Chr02*	32109811	(G/A)
CakSNP3019	*Kabuli*	*Ca_Kabuli_Chr02*	32109813	(C/T)
CakSNP3020	*Kabuli*	*Ca_Kabuli_Chr02*	32109821	(G/A)
CakSNP3021	*Kabuli*	*Ca_Kabuli_Chr02*	32109827	(T/C)
CakSNP3022	*Kabuli*	*Ca_Kabuli_Chr02*	32109859	(A/C)
CakSNP3023	*Kabuli*	*Ca_Kabuli_Chr02*	32109904	(T/A)
CakSNP3024	*Kabuli*	*Ca_Kabuli_Chr02*	32109895	(A/G)
CakSNP3025	*Kabuli*	*Ca_Kabuli_Chr02*	32109892	(T/A)
CakSNP3026	*Kabuli*	*Ca_Kabuli_Chr02*	32119094	(A/T)
CakSNP3027	*Kabuli*	*Ca_Kabuli_Chr02*	32119268	(T/A)
CakSNP3028	*Kabuli*	*Ca_Kabuli_Chr02*	32119274	(T/C)
CakSNP3029	*Kabuli*	*Ca_Kabuli_Chr02*	32119331	(A/T)
CakSNP3030	*Kabuli*	*Ca_Kabuli_Chr02*	32129272	(T/C)
CakSNP3031	*Kabuli*	*Ca_Kabuli_Chr02*	32129395	(C/G)
CakSNP3032	*Kabuli*	*Ca_Kabuli_Chr02*	32140939	(A/T)
CakSNP3033	*Kabuli*	*Ca_Kabuli_Chr02*	32140948	(A/C)

SNP IDs	Cultivars	Chromosomes/scaffolds	Physical positions (bp)	SNPs
CakSNP3034	*Kabuli*	*Ca_Kabuli_Chr02*	32140987	(A/G)
CakSNP3035	*Kabuli*	*Ca_Kabuli_Chr02*	32141047	(A/G)
CakSNP3036	*Kabuli*	*Ca_Kabuli_Chr02*	32141038	(T/C)
CakSNP3037	*Kabuli*	*Ca_Kabuli_Chr02*	32141019	(G/A)
CakSNP3038	*Kabuli*	*Ca_Kabuli_Chr02*	32141013	(G/C)
CakSNP3039	*Kabuli*	*Ca_Kabuli_Chr02*	32141002	(A/T)
CakSNP3040	*Kabuli*	*Ca_Kabuli_Chr02*	32140998	(G/T)
CakSNP3041	*Kabuli*	*Ca_Kabuli_Chr02*	32140990	(G/A)
CakSNP3042	*Kabuli*	*Ca_Kabuli_Chr02*	32203032	(A/G)
CakSNP3043	*Kabuli*	*Ca_Kabuli_Chr02*	32203036	(A/T)
CakSNP3044	*Kabuli*	*Ca_Kabuli_Chr02*	32242818	(T/G)
CakSNP3045	*Kabuli*	*Ca_Kabuli_Chr02*	32348054	(T/A)
CakSNP3046	*Kabuli*	*Ca_Kabuli_Chr02*	32348087	(A/C)
CakSNP3047	*Kabuli*	*Ca_Kabuli_Chr02*	32348119	(G/T)
CakSNP3048	*Kabuli*	*Ca_Kabuli_Chr02*	32348793	(G/A)
CakSNP3049	*Kabuli*	*Ca_Kabuli_Chr02*	32348870	(T/C)
CakSNP3050	*Kabuli*	*Ca_Kabuli_Chr02*	32411994	(G/T)
CakSNP3051	*Kabuli*	*Ca_Kabuli_Chr02*	32412000	(G/T)
CakSNP3052	*Kabuli*	*Ca_Kabuli_Chr02*	32481969	(C/T)
CakSNP3053	*Kabuli*	*Ca_Kabuli_Chr02*	32481983	(G/T)
CakSNP3054	*Kabuli*	*Ca_Kabuli_Chr02*	32484223	(C/T)
CakSNP3055	*Kabuli*	*Ca_Kabuli_Chr02*	32545320	(A/G)
CakSNP3056	*Kabuli*	*Ca_Kabuli_Chr02*	32545329	(T/C)
CakSNP3057	*Kabuli*	*Ca_Kabuli_Chr02*	32545351	(G/A)
CakSNP3058	*Kabuli*	*Ca_Kabuli_Chr02*	32556992	(T/A)
CakSNP3059	*Kabuli*	*Ca_Kabuli_Chr02*	32564427	(T/C)
CakSNP3060	*Kabuli*	*Ca_Kabuli_Chr02*	32564480	(G/A)
CakSNP3061	*Kabuli*	*Ca_Kabuli_Chr02*	32564770	(G/T)
CakSNP3062	*Kabuli*	*Ca_Kabuli_Chr02*	32564796	(A/T)
CakSNP3063	*Kabuli*	*Ca_Kabuli_Chr02*	32570394	(G/A)
CakSNP3064	*Kabuli*	*Ca_Kabuli_Chr02*	32570479	(T/G)
CakSNP3065	*Kabuli*	*Ca_Kabuli_Chr02*	32570429	(G/A)
CakSNP3066	*Kabuli*	*Ca_Kabuli_Chr02*	32570504	(G/C)
CakSNP3067	*Kabuli*	*Ca_Kabuli_Chr02*	32577274	(T/A)
CakSNP3068	*Kabuli*	*Ca_Kabuli_Chr02*	32577593	(A/G)
CakSNP3069	*Kabuli*	*Ca_Kabuli_Chr02*	32579849	(T/C)
CakSNP3070	*Kabuli*	*Ca_Kabuli_Chr02*	32579900	(C/G)
CakSNP3071	*Kabuli*	*Ca_Kabuli_Chr02*	32608900	(A/T)
CakSNP3072	*Kabuli*	*Ca_Kabuli_Chr02*	32611624	(C/T)
CakSNP3073	*Kabuli*	*Ca_Kabuli_Chr02*	32611609	(A/G)
CakSNP3074	*Kabuli*	*Ca_Kabuli_Chr02*	32611601	(G/T)

SNP IDs	Cultivars	Chromosomes/scaffolds	Physical positions (bp)	SNPs
CakSNP3075	*Kabuli*	*Ca_Kabuli_Chr02*	32627727	(C/G)
CakSNP3076	*Kabuli*	*Ca_Kabuli_Chr02*	32629763	(A/G)
CakSNP3077	*Kabuli*	*Ca_Kabuli_Chr02*	32670054	(C/T)
CakSNP3078	*Kabuli*	*Ca_Kabuli_Chr02*	32670099	(C/T)
CakSNP3079	*Kabuli*	*Ca_Kabuli_Chr02*	32670181	(T/G)
CakSNP3080	*Kabuli*	*Ca_Kabuli_Chr02*	32671354	(T/A)
CakSNP3081	*Kabuli*	*Ca_Kabuli_Chr02*	32781880	(A/G)
CakSNP3082	*Kabuli*	*Ca_Kabuli_Chr02*	32844754	(T/G)
CakSNP3083	*Kabuli*	*Ca_Kabuli_Chr02*	32844842	(C/G)
CakSNP3084	*Kabuli*	*Ca_Kabuli_Chr02*	32867125	(A/G)
CakSNP3085	*Kabuli*	*Ca_Kabuli_Chr02*	32934646	(C/T)
CakSNP3086	*Kabuli*	*Ca_Kabuli_Chr02*	32934633	(G/C)
CakSNP3087	*Kabuli*	*Ca_Kabuli_Chr02*	32936855	(A/G)
CakSNP3088	*Kabuli*	*Ca_Kabuli_Chr02*	32936908	(G/A)
CakSNP3089	*Kabuli*	*Ca_Kabuli_Chr02*	32957515	(A/G)
CakSNP3090	*Kabuli*	*Ca_Kabuli_Chr02*	32957523	(T/C)
CakSNP3091	*Kabuli*	*Ca_Kabuli_Chr02*	32957538	(A/C)
CakSNP3092	*Kabuli*	*Ca_Kabuli_Chr02*	32957572	(A/C)
CakSNP3093	*Kabuli*	*Ca_Kabuli_Chr02*	32957958	(A/C)
CakSNP3094	*Kabuli*	*Ca_Kabuli_Chr02*	32958034	(C/G)
CakSNP3095	*Kabuli*	*Ca_Kabuli_Chr02*	32989134	(G/T)
CakSNP3096	*Kabuli*	*Ca_Kabuli_Chr02*	32989205	(G/T)
CakSNP3097	*Kabuli*	*Ca_Kabuli_Chr02*	33083903	(T/A)
CakSNP3098	*Kabuli*	*Ca_Kabuli_Chr02*	33084095	(T/G)
CakSNP3099	*Kabuli*	*Ca_Kabuli_Chr02*	33084132	(T/A)
CakSNP3100	*Kabuli*	*Ca_Kabuli_Chr02*	33103318	(G/A)
CakSNP3101	*Kabuli*	*Ca_Kabuli_Chr02*	33103466	(G/A)
CakSNP3102	*Kabuli*	*Ca_Kabuli_Chr02*	33103750	(A/G)
CakSNP3103	*Kabuli*	*Ca_Kabuli_Chr02*	33103766	(G/A)
CakSNP3104	*Kabuli*	*Ca_Kabuli_Chr02*	33172936	(C/T)
CakSNP3105	*Kabuli*	*Ca_Kabuli_Chr02*	33172971	(T/G)
CakSNP3106	*Kabuli*	*Ca_Kabuli_Chr02*	33178391	(G/A)
CakSNP3107	*Kabuli*	*Ca_Kabuli_Chr02*	33178407	(C/T)
CakSNP3108	*Kabuli*	*Ca_Kabuli_Chr02*	33178419	(C/A)
CakSNP3109	*Kabuli*	*Ca_Kabuli_Chr02*	33178420	(A/C)
CakSNP3110	*Kabuli*	*Ca_Kabuli_Chr02*	33178506	(T/C)
CakSNP3111	*Kabuli*	*Ca_Kabuli_Chr02*	33184262	(T/C)
CakSNP3112	*Kabuli*	*Ca_Kabuli_Chr02*	33194198	(A/G)
CakSNP3113	*Kabuli*	*Ca_Kabuli_Chr02*	33204253	(C/T)
CakSNP3114	*Kabuli*	*Ca_Kabuli_Chr02*	33326392	(T/G)
CakSNP3115	*Kabuli*	*Ca_Kabuli_Chr02*	33326398	(C/T)

SNP IDs	Cultivars	Chromosomes/scaffolds	Physical positions (bp)	SNPs
CakSNP3116	*Kabuli*	*Ca_Kabuli_Chr02*	33326444	(G/A)
CakSNP3117	*Kabuli*	*Ca_Kabuli_Chr02*	33326522	(G/A)
CakSNP3118	*Kabuli*	*Ca_Kabuli_Chr02*	33326520	(T/C)
CakSNP3119	*Kabuli*	*Ca_Kabuli_Chr02*	33326516	(G/A)
CakSNP3120	*Kabuli*	*Ca_Kabuli_Chr02*	33326510	(C/G)
CakSNP3121	*Kabuli*	*Ca_Kabuli_Chr02*	33393209	(T/C)
CakSNP3122	*Kabuli*	*Ca_Kabuli_Chr02*	33398561	(C/T)
CakSNP3123	*Kabuli*	*Ca_Kabuli_Chr02*	33398550	(G/A)
CakSNP3124	*Kabuli*	*Ca_Kabuli_Chr02*	33398549	(T/C)
CakSNP3125	*Kabuli*	*Ca_Kabuli_Chr02*	33398527	(T/G)
CakSNP3126	*Kabuli*	*Ca_Kabuli_Chr02*	33399010	(C/T)
CakSNP3127	*Kabuli*	*Ca_Kabuli_Chr02*	33399019	(T/C)
CakSNP3128	*Kabuli*	*Ca_Kabuli_Chr02*	33399045	(A/C)
CakSNP3129	*Kabuli*	*Ca_Kabuli_Chr02*	33399098	(G/A)
CakSNP3130	*Kabuli*	*Ca_Kabuli_Chr02*	33399063	(C/T)
CakSNP3131	*Kabuli*	*Ca_Kabuli_Chr02*	33399405	(C/T)
CakSNP3132	*Kabuli*	*Ca_Kabuli_Chr02*	33399404	(C/A)
CakSNP3133	*Kabuli*	*Ca_Kabuli_Chr02*	33399397	(C/T)
CakSNP3134	*Kabuli*	*Ca_Kabuli_Chr02*	33399393	(C/A)
CakSNP3135	*Kabuli*	*Ca_Kabuli_Chr02*	33399381	(C/T)
CakSNP3136	*Kabuli*	*Ca_Kabuli_Chr02*	33399342	(A/C)
CakSNP3137	*Kabuli*	*Ca_Kabuli_Chr02*	33399465	(C/G)
CakSNP3138	*Kabuli*	*Ca_Kabuli_Chr02*	33399608	(C/A)
CakSNP3139	*Kabuli*	*Ca_Kabuli_Chr02*	33401608	(A/G)
CakSNP3140	*Kabuli*	*Ca_Kabuli_Chr02*	33490058	(C/A)
CakSNP3141	*Kabuli*	*Ca_Kabuli_Chr02*	33489989	(T/C)
CakSNP3142	*Kabuli*	*Ca_Kabuli_Chr02*	33575286	(A/C)
CakSNP3143	*Kabuli*	*Ca_Kabuli_Chr02*	33582948	(G/A)
CakSNP3144	*Kabuli*	*Ca_Kabuli_Chr02*	33582900	(G/A)
CakSNP3145	*Kabuli*	*Ca_Kabuli_Chr02*	33650447	(A/T)
CakSNP3146	*Kabuli*	*Ca_Kabuli_Chr02*	33651426	(G/A)
CakSNP3147	*Kabuli*	*Ca_Kabuli_Chr02*	33651408	(A/G)
CakSNP3148	*Kabuli*	*Ca_Kabuli_Chr02*	33661177	(C/T)
CakSNP3149	*Kabuli*	*Ca_Kabuli_Chr02*	33661316	(T/C)
CakSNP3150	*Kabuli*	*Ca_Kabuli_Chr02*	33686794	(G/A)
CakSNP3151	*Kabuli*	*Ca_Kabuli_Chr02*	33692368	(C/A)
CakSNP3152	*Kabuli*	*Ca_Kabuli_Chr02*	33713018	(G/A)
CakSNP3153	*Kabuli*	*Ca_Kabuli_Chr02*	33721515	(T/A)
CakSNP3154	*Kabuli*	*Ca_Kabuli_Chr02*	33733893	(C/T)
CakSNP3155	*Kabuli*	*Ca_Kabuli_Chr02*	33745281	(A/G)
CakSNP3156	*Kabuli*	*Ca_Kabuli_Chr02*	33764634	(G/A)

SNP IDs	Cultivars	Chromosomes/scaffolds	Physical positions (bp)	SNPs
CakSNP3157	*Kabuli*	*Ca_Kabuli_Chr02*	33783871	(C/T)
CakSNP3158	*Kabuli*	*Ca_Kabuli_Chr02*	33785203	(G/A)
CakSNP3159	*Kabuli*	*Ca_Kabuli_Chr02*	33786243	(G/A)
CakSNP3160	*Kabuli*	*Ca_Kabuli_Chr02*	33788913	(A/C)
CakSNP3161	*Kabuli*	*Ca_Kabuli_Chr02*	33798717	(T/C)
CakSNP3162	*Kabuli*	*Ca_Kabuli_Chr02*	33798748	(T/C)
CakSNP3163	*Kabuli*	*Ca_Kabuli_Chr02*	34152352	(T/C)
CakSNP3164	*Kabuli*	*Ca_Kabuli_Chr02*	34152384	(T/C)
CakSNP3165	*Kabuli*	*Ca_Kabuli_Chr02*	34152476	(C/T)
CakSNP3166	*Kabuli*	*Ca_Kabuli_Chr02*	34155090	(A/G)
CakSNP3167	*Kabuli*	*Ca_Kabuli_Chr02*	34155179	(A/G)
CakSNP3168	*Kabuli*	*Ca_Kabuli_Chr02*	34178534	(G/A)
CakSNP3169	*Kabuli*	*Ca_Kabuli_Chr02*	34203162	(T/C)
CakSNP3170	*Kabuli*	*Ca_Kabuli_Chr02*	34203444	(T/C)
CakSNP3171	*Kabuli*	*Ca_Kabuli_Chr02*	34222585	(G/A)
CakSNP3172	*Kabuli*	*Ca_Kabuli_Chr02*	34249987	(C/T)
CakSNP3173	*Kabuli*	*Ca_Kabuli_Chr02*	34249953	(G/T)
CakSNP3174	*Kabuli*	*Ca_Kabuli_Chr02*	34250042	(C/T)
CakSNP3175	*Kabuli*	*Ca_Kabuli_Chr02*	34250266	(T/C)
CakSNP3176	*Kabuli*	*Ca_Kabuli_Chr02*	34266109	(A/G)
CakSNP3177	*Kabuli*	*Ca_Kabuli_Chr02*	34288116	(G/C)
CakSNP3178	*Kabuli*	*Ca_Kabuli_Chr02*	34299096	(G/A)
CakSNP3179	*Kabuli*	*Ca_Kabuli_Chr02*	34299075	(C/T)
CakSNP3180	*Kabuli*	*Ca_Kabuli_Chr02*	34299074	(A/C)
CakSNP3181	*Kabuli*	*Ca_Kabuli_Chr02*	34299063	(G/A)
CakSNP3182	*Kabuli*	*Ca_Kabuli_Chr02*	34299079	(A/C)
CakSNP3183	*Kabuli*	*Ca_Kabuli_Chr02*	34299846	(C/T)
CakSNP3184	*Kabuli*	*Ca_Kabuli_Chr02*	34300059	(A/G)
CakSNP3185	*Kabuli*	*Ca_Kabuli_Chr02*	34300057	(A/G)
CakSNP3186	*Kabuli*	*Ca_Kabuli_Chr02*	34343478	(C/G)
CakSNP3187	*Kabuli*	*Ca_Kabuli_Chr02*	34343705	(G/A)
CakSNP3188	*Kabuli*	*Ca_Kabuli_Chr02*	34346849	(C/T)
CakSNP3189	*Kabuli*	*Ca_Kabuli_Chr02*	34346842	(T/C)
CakSNP3190	*Kabuli*	*Ca_Kabuli_Chr02*	34372503	(T/G)
CakSNP3191	*Kabuli*	*Ca_Kabuli_Chr02*	34428978	(C/A)
CakSNP3192	*Kabuli*	*Ca_Kabuli_Chr02*	34428979	(G/C)
CakSNP3193	*Kabuli*	*Ca_Kabuli_Chr02*	34429029	(G/T)
CakSNP3194	*Kabuli*	*Ca_Kabuli_Chr02*	34429030	(A/T)
CakSNP3195	*Kabuli*	*Ca_Kabuli_Chr02*	34551849	(A/C)
CakSNP3196	*Kabuli*	*Ca_Kabuli_Chr02*	34554164	(A/C)
CakSNP3197	*Kabuli*	*Ca_Kabuli_Chr02*	34601444	(C/A)

SNP IDs	Cultivars	Chromosomes/scaffolds	Physical positions (bp)	SNPs
CakSNP3198	*Kabuli*	*Ca_Kabuli_Chr02*	34634128	(C/T)
CakSNP3199	*Kabuli*	*Ca_Kabuli_Chr02*	34638960	(A/C)
CakSNP3200	*Kabuli*	*Ca_Kabuli_Chr02*	34638955	(T/A)
CakSNP3201	*Kabuli*	*Ca_Kabuli_Chr02*	34734329	(G/A)
CakSNP3202	*Kabuli*	*Ca_Kabuli_Chr02*	34734379	(G/A)
CakSNP3203	*Kabuli*	*Ca_Kabuli_Chr02*	34734439	(G/A)
CakSNP3204	*Kabuli*	*Ca_Kabuli_Chr02*	34734472	(A/G)
CakSNP3205	*Kabuli*	*Ca_Kabuli_Chr02*	34736649	(T/C)
CakSNP3206	*Kabuli*	*Ca_Kabuli_Chr02*	34736658	(A/C)
CakSNP3207	*Kabuli*	*Ca_Kabuli_Chr02*	34736753	(C/T)
CakSNP3208	*Kabuli*	*Ca_Kabuli_Chr02*	34754363	(T/C)
CakSNP3209	*Kabuli*	*Ca_Kabuli_Chr02*	34754507	(C/T)
CakSNP3210	*Kabuli*	*Ca_Kabuli_Chr02*	34820075	(C/T)
CakSNP3211	*Kabuli*	*Ca_Kabuli_Chr02*	34830995	(A/G)
CakSNP3212	*Kabuli*	*Ca_Kabuli_Chr02*	34847681	(C/A)
CakSNP3213	*Kabuli*	*Ca_Kabuli_Chr02*	34848053	(G/A)
CakSNP3214	*Kabuli*	*Ca_Kabuli_Chr02*	34848480	(T/C)
CakSNP3215	*Kabuli*	*Ca_Kabuli_Chr02*	34862129	(G/T)
CakSNP3216	*Kabuli*	*Ca_Kabuli_Chr02*	34862135	(G/A)
CakSNP3217	*Kabuli*	*Ca_Kabuli_Chr02*	34862139	(A/G)
CakSNP3218	*Kabuli*	*Ca_Kabuli_Chr02*	34862150	(C/T)
CakSNP3219	*Kabuli*	*Ca_Kabuli_Chr02*	34862276	(C/G)
CakSNP3220	*Kabuli*	*Ca_Kabuli_Chr02*	34888414	(T/C)
CakSNP3221	*Kabuli*	*Ca_Kabuli_Chr02*	35013088	(G/C)
CakSNP3222	*Kabuli*	*Ca_Kabuli_Chr02*	35013084	(T/C)
CakSNP3223	*Kabuli*	*Ca_Kabuli_Chr02*	35052930	(C/A)
CakSNP3224	*Kabuli*	*Ca_Kabuli_Chr02*	35054244	(C/A)
CakSNP3225	*Kabuli*	*Ca_Kabuli_Chr02*	35059446	(A/T)
CakSNP3226	*Kabuli*	*Ca_Kabuli_Chr02*	35059434	(T/C)
CakSNP3227	*Kabuli*	*Ca_Kabuli_Chr02*	35059440	(A/C)
CakSNP3228	*Kabuli*	*Ca_Kabuli_Chr02*	35059451	(C/A)
CakSNP3229	*Kabuli*	*Ca_Kabuli_Chr02*	35060171	(A/G)
CakSNP3230	*Kabuli*	*Ca_Kabuli_Chr02*	35060178	(A/T)
CakSNP3231	*Kabuli*	*Ca_Kabuli_Chr02*	35060228	(T/C)
CakSNP3232	*Kabuli*	*Ca_Kabuli_Chr02*	35060273	(G/A)
CakSNP3233	*Kabuli*	*Ca_Kabuli_Chr02*	35060264	(A/T)
CakSNP3234	*Kabuli*	*Ca_Kabuli_Chr02*	35066138	(G/A)
CakSNP3235	*Kabuli*	*Ca_Kabuli_Chr02*	35095010	(A/G)
CakSNP3236	*Kabuli*	*Ca_Kabuli_Chr02*	35096648	(A/T)
CakSNP3237	*Kabuli*	*Ca_Kabuli_Chr02*	35096649	(A/T)
CakSNP3238	*Kabuli*	*Ca_Kabuli_Chr02*	35120761	(G/A)

SNP IDs	Cultivars	Chromosomes/scaffolds	Physical positions (bp)	SNPs
CakSNP3239	*Kabuli*	*Ca_Kabuli_Chr02*	35122109	(C/G)
CakSNP3240	*Kabuli*	*Ca_Kabuli_Chr02*	35154629	(C/A)
CakSNP3241	*Kabuli*	*Ca_Kabuli_Chr02*	35154603	(G/A)
CakSNP3242	*Kabuli*	*Ca_Kabuli_Chr02*	35171882	(T/G)
CakSNP3243	*Kabuli*	*Ca_Kabuli_Chr02*	35189471	(C/T)
CakSNP3244	*Kabuli*	*Ca_Kabuli_Chr02*	35195303	(T/C)
CakSNP3245	*Kabuli*	*Ca_Kabuli_Chr02*	35198352	(T/C)
CakSNP3246	*Kabuli*	*Ca_Kabuli_Chr02*	35209940	(C/T)
CakSNP3247	*Kabuli*	*Ca_Kabuli_Chr02*	35362587	(A/G)
CakSNP3248	*Kabuli*	*Ca_Kabuli_Chr02*	35362642	(A/G)
CakSNP3249	*Kabuli*	*Ca_Kabuli_Chr02*	35362657	(C/A)
CakSNP3250	*Kabuli*	*Ca_Kabuli_Chr02*	35362729	(A/G)
CakSNP3251	*Kabuli*	*Ca_Kabuli_Chr02*	35363488	(T/C)
CakSNP3252	*Kabuli*	*Ca_Kabuli_Chr02*	35363501	(T/C)
CakSNP3253	*Kabuli*	*Ca_Kabuli_Chr02*	35365029	(C/T)
CakSNP3254	*Kabuli*	*Ca_Kabuli_Chr02*	35367161	(G/A)
CakSNP3255	*Kabuli*	*Ca_Kabuli_Chr02*	35413400	(T/A)
CakSNP3256	*Kabuli*	*Ca_Kabuli_Chr02*	35441184	(C/T)
CakSNP3257	*Kabuli*	*Ca_Kabuli_Chr02*	35441180	(A/G)
CakSNP3258	*Kabuli*	*Ca_Kabuli_Chr02*	35441174	(T/G)
CakSNP3259	*Kabuli*	*Ca_Kabuli_Chr02*	35447976	(A/G)
CakSNP3260	*Kabuli*	*Ca_Kabuli_Chr02*	35448018	(C/G)
CakSNP3261	*Kabuli*	*Ca_Kabuli_Chr02*	35448126	(T/A)
CakSNP3262	*Kabuli*	*Ca_Kabuli_Chr02*	35449653	(G/A)
CakSNP3263	*Kabuli*	*Ca_Kabuli_Chr02*	35473250	(C/T)
CakSNP3264	*Kabuli*	*Ca_Kabuli_Chr02*	35505791	(C/G)
CakSNP3265	*Kabuli*	*Ca_Kabuli_Chr02*	35505874	(T/G)
CakSNP3266	*Kabuli*	*Ca_Kabuli_Chr02*	35533246	(G/A)
CakSNP3267	*Kabuli*	*Ca_Kabuli_Chr02*	35593439	(T/C)
CakSNP3268	*Kabuli*	*Ca_Kabuli_Chr02*	35593797	(G/T)
CakSNP3269	*Kabuli*	*Ca_Kabuli_Chr02*	35593824	(A/C)
CakSNP3270	*Kabuli*	*Ca_Kabuli_Chr02*	35593827	(A/G)
CakSNP3271	*Kabuli*	*Ca_Kabuli_Chr02*	35594018	(A/T)
CakSNP3272	*Kabuli*	*Ca_Kabuli_Chr02*	35594013	(C/T)
CakSNP3273	*Kabuli*	*Ca_Kabuli_Chr02*	35593985	(A/G)
CakSNP3274	*Kabuli*	*Ca_Kabuli_Chr02*	35593965	(C/T)
CakSNP3275	*Kabuli*	*Ca_Kabuli_Chr02*	35593956	(A/G)
CakSNP3276	*Kabuli*	*Ca_Kabuli_Chr02*	35601566	(T/C)
CakSNP3277	*Kabuli*	*Ca_Kabuli_Chr02*	35601571	(A/C)
CakSNP3278	*Kabuli*	*Ca_Kabuli_Chr02*	35601578	(C/T)
CakSNP3279	*Kabuli*	*Ca_Kabuli_Chr02*	35601605	(G/A)

SNP IDs	Cultivars	Chromosomes/scaffolds	Physical positions (bp)	SNPs
CakSNP3280	*Kabuli*	*Ca_Kabuli_Chr02*	35601691	(A/G)
CakSNP3281	*Kabuli*	*Ca_Kabuli_Chr02*	35601657	(T/C)
CakSNP3282	*Kabuli*	*Ca_Kabuli_Chr02*	35601632	(G/T)
CakSNP3283	*Kabuli*	*Ca_Kabuli_Chr02*	35612587	(C/A)
CakSNP3284	*Kabuli*	*Ca_Kabuli_Chr02*	35612590	(G/A)
CakSNP3285	*Kabuli*	*Ca_Kabuli_Chr02*	35612644	(G/A)
CakSNP3286	*Kabuli*	*Ca_Kabuli_Chr02*	35612788	(G/A)
CakSNP3287	*Kabuli*	*Ca_Kabuli_Chr02*	35612895	(T/C)
CakSNP3288	*Kabuli*	*Ca_Kabuli_Chr02*	35612900	(C/T)
CakSNP3289	*Kabuli*	*Ca_Kabuli_Chr02*	35613016	(G/A)
CakSNP3290	*Kabuli*	*Ca_Kabuli_Chr02*	35613135	(T/C)
CakSNP3291	*Kabuli*	*Ca_Kabuli_Chr02*	35658426	(C/T)
CakSNP3292	*Kabuli*	*Ca_Kabuli_Chr02*	35658537	(T/C)
CakSNP3293	*Kabuli*	*Ca_Kabuli_Chr02*	35661053	(C/T)
CakSNP3294	*Kabuli*	*Ca_Kabuli_Chr02*	35670478	(A/G)
CakSNP3295	*Kabuli*	*Ca_Kabuli_Chr02*	35704018	(A/G)
CakSNP3296	*Kabuli*	*Ca_Kabuli_Chr02*	35704069	(G/A)
CakSNP3297	*Kabuli*	*Ca_Kabuli_Chr02*	35704228	(C/A)
CakSNP3298	*Kabuli*	*Ca_Kabuli_Chr02*	35704213	(A/G)
CakSNP3299	*Kabuli*	*Ca_Kabuli_Chr02*	35704204	(T/C)
CakSNP3300	*Kabuli*	*Ca_Kabuli_Chr02*	35704200	(T/C)
CakSNP3301	*Kabuli*	*Ca_Kabuli_Chr02*	35704180	(A/G)
CakSNP3302	*Kabuli*	*Ca_Kabuli_Chr02*	35704178	(G/A)
CakSNP3303	*Kabuli*	*Ca_Kabuli_Chr02*	35728285	(T/A)
CakSNP3304	*Kabuli*	*Ca_Kabuli_Chr02*	35754617	(G/T)
CakSNP3305	*Kabuli*	*Ca_Kabuli_Chr02*	35754557	(C/T)
CakSNP3306	*Kabuli*	*Ca_Kabuli_Chr02*	35754680	(C/A)
CakSNP3307	*Kabuli*	*Ca_Kabuli_Chr02*	35754682	(A/G)
CakSNP3308	*Kabuli*	*Ca_Kabuli_Chr02*	35754806	(G/C)
CakSNP3309	*Kabuli*	*Ca_Kabuli_Chr02*	35754757	(G/C)
CakSNP3310	*Kabuli*	*Ca_Kabuli_Chr02*	35759932	(A/T)
CakSNP3311	*Kabuli*	*Ca_Kabuli_Chr02*	35759940	(T/C)
CakSNP3312	*Kabuli*	*Ca_Kabuli_Chr02*	35759981	(G/A)
CakSNP3313	*Kabuli*	*Ca_Kabuli_Chr02*	35760062	(G/C)
CakSNP3314	*Kabuli*	*Ca_Kabuli_Chr02*	35760073	(A/T)
CakSNP3315	*Kabuli*	*Ca_Kabuli_Chr02*	35760170	(A/G)
CakSNP3316	*Kabuli*	*Ca_Kabuli_Chr02*	35760155	(G/A)
CakSNP3317	*Kabuli*	*Ca_Kabuli_Chr02*	35770569	(A/G)
CakSNP3318	*Kabuli*	*Ca_Kabuli_Chr02*	35795446	(G/A)
CakSNP3319	*Kabuli*	*Ca_Kabuli_Chr02*	35795395	(A/G)
CakSNP3320	*Kabuli*	*Ca_Kabuli_Chr02*	35795494	(G/A)

SNP IDs	Cultivars	Chromosomes/scaffolds	Physical positions (bp)	SNPs
CakSNP3321	*Kabuli*	*Ca_Kabuli_Chr02*	35795496	(G/T)
CakSNP3322	*Kabuli*	*Ca_Kabuli_Chr02*	35795497	(G/T)
CakSNP3323	*Kabuli*	*Ca_Kabuli_Chr02*	35795521	(T/C)
CakSNP3324	*Kabuli*	*Ca_Kabuli_Chr02*	35828553	(G/A)
CakSNP3325	*Kabuli*	*Ca_Kabuli_Chr02*	35829782	(A/G)
CakSNP3326	*Kabuli*	*Ca_Kabuli_Chr02*	35844790	(A/G)
CakSNP3327	*Kabuli*	*Ca_Kabuli_Chr02*	35844762	(A/G)
CakSNP3328	*Kabuli*	*Ca_Kabuli_Chr02*	35862852	(C/T)
CakSNP3329	*Kabuli*	*Ca_Kabuli_Chr02*	35862878	(T/G)
CakSNP3330	*Kabuli*	*Ca_Kabuli_Chr02*	35875666	(A/G)
CakSNP3331	*Kabuli*	*Ca_Kabuli_Chr02*	35875653	(C/T)
CakSNP3332	*Kabuli*	*Ca_Kabuli_Chr02*	35930699	(T/G)
CakSNP3333	*Kabuli*	*Ca_Kabuli_Chr02*	35993062	(C/T)
CakSNP3334	*Kabuli*	*Ca_Kabuli_Chr02*	35993082	(T/G)
CakSNP3335	*Kabuli*	*Ca_Kabuli_Chr02*	35993089	(C/T)
CakSNP3336	*Kabuli*	*Ca_Kabuli_Chr02*	35993092	(A/T)
CakSNP3337	*Kabuli*	*Ca_Kabuli_Chr02*	35993178	(G/A)
CakSNP3338	*Kabuli*	*Ca_Kabuli_Chr02*	36013809	(A/G)
CakSNP3339	*Kabuli*	*Ca_Kabuli_Chr02*	36039428	(C/T)
CakSNP3340	*Kabuli*	*Ca_Kabuli_Chr02*	36039377	(C/T)
CakSNP3341	*Kabuli*	*Ca_Kabuli_Chr02*	36039364	(G/T)
CakSNP3342	*Kabuli*	*Ca_Kabuli_Chr02*	36046593	(G/T)
CakSNP3343	*Kabuli*	*Ca_Kabuli_Chr02*	36046595	(T/G)
CakSNP3344	*Kabuli*	*Ca_Kabuli_Chr02*	36046741	(G/A)
CakSNP3345	*Kabuli*	*Ca_Kabuli_Chr02*	36046720	(T/A)
CakSNP3346	*Kabuli*	*Ca_Kabuli_Chr02*	36046767	(T/A)
CakSNP3347	*Kabuli*	*Ca_Kabuli_Chr02*	36046799	(C/A)
CakSNP3348	*Kabuli*	*Ca_Kabuli_Chr02*	36046820	(T/A)
CakSNP3349	*Kabuli*	*Ca_Kabuli_Chr02*	36046844	(T/G)
CakSNP3350	*Kabuli*	*Ca_Kabuli_Chr02*	36069340	(T/C)
CakSNP3351	*Kabuli*	*Ca_Kabuli_Chr02*	36072247	(C/A)
CakSNP3352	*Kabuli*	*Ca_Kabuli_Chr02*	36074147	(T/C)
CakSNP3353	*Kabuli*	*Ca_Kabuli_Chr02*	36088577	(A/T)
CakSNP3354	*Kabuli*	*Ca_Kabuli_Chr02*	36088623	(C/T)
CakSNP3355	*Kabuli*	*Ca_Kabuli_Chr02*	36088676	(T/C)
CakSNP3356	*Kabuli*	*Ca_Kabuli_Chr02*	36088853	(G/A)
CakSNP3357	*Kabuli*	*Ca_Kabuli_Chr02*	36101358	(A/G)
CakSNP3358	*Kabuli*	*Ca_Kabuli_Chr02*	36101379	(A/G)
CakSNP3359	*Kabuli*	*Ca_Kabuli_Chr02*	36101421	(C/T)
CakSNP3360	*Kabuli*	*Ca_Kabuli_Chr02*	36117695	(T/A)
CakSNP3361	*Kabuli*	*Ca_Kabuli_Chr02*	36117647	(G/A)

SNP IDs	Cultivars	Chromosomes/scaffolds	Physical positions (bp)	SNPs
CakSNP3362	*Kabuli*	*Ca_Kabuli_Chr02*	36253581	(C/T)
CakSNP3363	*Kabuli*	*Ca_Kabuli_Chr02*	36276791	(G/A)
CakSNP3364	*Kabuli*	*Ca_Kabuli_Chr02*	36323575	(G/T)
CakSNP3365	*Kabuli*	*Ca_Kabuli_Chr02*	36326361	(A/C)
CakSNP3366	*Kabuli*	*Ca_Kabuli_Chr02*	36335252	(A/C)
CakSNP3367	*Kabuli*	*Ca_Kabuli_Chr02*	36343900	(C/T)
CakSNP3368	*Kabuli*	*Ca_Kabuli_Chr02*	36343933	(T/C)
CakSNP3369	*Kabuli*	*Ca_Kabuli_Chr02*	36346336	(A/G)
CakSNP3370	*Kabuli*	*Ca_Kabuli_Chr02*	36375224	(A/G)
CakSNP3371	*Kabuli*	*Ca_Kabuli_Chr02*	36375638	(A/G)
CakSNP3372	*Kabuli*	*Ca_Kabuli_Chr02*	36387189	(G/C)
CakSNP3373	*Kabuli*	*Ca_Kabuli_Chr02*	36387245	(T/C)
CakSNP3374	*Kabuli*	*Ca_Kabuli_Chr02*	36389041	(G/A)
CakSNP3375	*Kabuli*	*Ca_Kabuli_Chr02*	36389096	(C/T)
CakSNP3376	*Kabuli*	*Ca_Kabuli_Chr02*	36403788	(A/G)
CakSNP3377	*Kabuli*	*Ca_Kabuli_Chr02*	36427841	(C/T)
CakSNP3378	*Kabuli*	*Ca_Kabuli_Chr02*	36429512	(T/C)
CakSNP3379	*Kabuli*	*Ca_Kabuli_Chr02*	36438939	(T/G)
CakSNP3380	*Kabuli*	*Ca_Kabuli_Chr02*	36453691	(A/G)
CakSNP3381	*Kabuli*	*Ca_Kabuli_Chr02*	36453685	(G/A)
CakSNP3382	*Kabuli*	*Ca_Kabuli_Chr02*	36466837	(C/T)
CakSNP3383	*Kabuli*	*Ca_Kabuli_Chr02*	36466998	(A/G)
CakSNP3384	*Kabuli*	*Ca_Kabuli_Chr02*	36467100	(A/C)
CakSNP3385	*Kabuli*	*Ca_Kabuli_Chr02*	36468390	(A/G)
CakSNP3386	*Kabuli*	*Ca_Kabuli_Chr02*	36468505	(C/A)
CakSNP3387	*Kabuli*	*Ca_Kabuli_Chr02*	36469921	(A/G)
CakSNP3388	*Kabuli*	*Ca_Kabuli_Chr02*	36505316	(C/A)
CakSNP3389	*Kabuli*	*Ca_Kabuli_Chr02*	36521200	(T/C)
CakSNP3390	*Kabuli*	*Ca_Kabuli_Chr02*	36533821	(A/C)
CakSNP3391	*Kabuli*	*Ca_Kabuli_Chr02*	36533910	(G/A)
CakSNP3392	*Kabuli*	*Ca_Kabuli_Chr02*	36554430	(G/A)
CakSNP3393	*Kabuli*	*Ca_Kabuli_Chr02*	36609730	(T/A)
CakSNP3394	*Kabuli*	*Ca_Kabuli_Chr02*	36609743	(C/T)
CakSNP3395	*Kabuli*	*Ca_Kabuli_Chr02*	36609744	(G/C)
CakSNP3396	*Kabuli*	*Ca_Kabuli_Chr02*	36609858	(C/G)
CakSNP3397	*Kabuli*	*Ca_Kabuli_Chr02*	36609950	(C/T)
CakSNP3398	*Kabuli*	*Ca_Kabuli_Chr02*	36609968	(C/T)
CakSNP3399	*Kabuli*	*Ca_Kabuli_Chr02*	36634234	(T/C)
CakSNP3400	*Kabuli*	*Ca_Kabuli_Chr02*	36634297	(C/G)
CakSNP3401	*Kabuli*	*Ca_Kabuli_Chr03*	171409	(A/T)
CakSNP3402	*Kabuli*	*Ca_Kabuli_Chr03*	171468	(A/G)

**SNP IDs**	**Cultivars**	**Chromosomes/scaffolds**	**Physical positions (bp)**	**SNPs**
CakSNP3403	*Kabuli*	*Ca_Kabuli_Chr03*	171574	(C/A)
CakSNP3404	*Kabuli*	*Ca_Kabuli_Chr03*	171576	(G/T)
CakSNP3405	*Kabuli*	*Ca_Kabuli_Chr03*	171579	(C/T)
CakSNP3406	*Kabuli*	*Ca_Kabuli_Chr03*	171675	(T/A)
CakSNP3407	*Kabuli*	*Ca_Kabuli_Chr03*	208233	(C/A)
CakSNP3408	*Kabuli*	*Ca_Kabuli_Chr03*	220564	(A/G)
CakSNP3409	*Kabuli*	*Ca_Kabuli_Chr03*	220568	(A/T)
CakSNP3410	*Kabuli*	*Ca_Kabuli_Chr03*	220572	(T/C)
CakSNP3411	*Kabuli*	*Ca_Kabuli_Chr03*	220573	(G/T)
CakSNP3412	*Kabuli*	*Ca_Kabuli_Chr03*	223943	(G/A)
CakSNP3413	*Kabuli*	*Ca_Kabuli_Chr03*	223976	(G/C)
CakSNP3414	*Kabuli*	*Ca_Kabuli_Chr03*	286425	(G/T)
CakSNP3415	*Kabuli*	*Ca_Kabuli_Chr03*	419715	(T/G)
CakSNP3416	*Kabuli*	*Ca_Kabuli_Chr03*	988477	(G/A)
CakSNP3417	*Kabuli*	*Ca_Kabuli_Chr03*	988471	(T/C)
CakSNP3418	*Kabuli*	*Ca_Kabuli_Chr03*	1499038	(G/A)
CakSNP3419	*Kabuli*	*Ca_Kabuli_Chr03*	1499006	(A/G)
CakSNP3420	*Kabuli*	*Ca_Kabuli_Chr03*	1746388	(C/A)
CakSNP3421	*Kabuli*	*Ca_Kabuli_Chr03*	1746389	(A/G)
CakSNP3422	*Kabuli*	*Ca_Kabuli_Chr03*	1829412	(T/G)
CakSNP3423	*Kabuli*	*Ca_Kabuli_Chr03*	1829358	(T/C)
CakSNP3424	*Kabuli*	*Ca_Kabuli_Chr03*	1829352	(C/T)
CakSNP3425	*Kabuli*	*Ca_Kabuli_Chr03*	1831920	(T/G)
CakSNP3426	*Kabuli*	*Ca_Kabuli_Chr03*	1831866	(T/C)
CakSNP3427	*Kabuli*	*Ca_Kabuli_Chr03*	1831860	(C/T)
CakSNP3428	*Kabuli*	*Ca_Kabuli_Chr03*	1831931	(A/C)
CakSNP3429	*Kabuli*	*Ca_Kabuli_Chr03*	2097376	(T/G)
CakSNP3430	*Kabuli*	*Ca_Kabuli_Chr03*	2097413	(A/G)
CakSNP3431	*Kabuli*	*Ca_Kabuli_Chr03*	2391515	(T/G)
CakSNP3432	*Kabuli*	*Ca_Kabuli_Chr03*	2423760	(T/C)
CakSNP3433	*Kabuli*	*Ca_Kabuli_Chr03*	2708703	(C/T)
CakSNP3434	*Kabuli*	*Ca_Kabuli_Chr03*	2825546	(G/T)
CakSNP3435	*Kabuli*	*Ca_Kabuli_Chr03*	2927148	(T/A)
CakSNP3436	*Kabuli*	*Ca_Kabuli_Chr03*	2927150	(A/T)
CakSNP3437	*Kabuli*	*Ca_Kabuli_Chr03*	2931027	(G/C)
CakSNP3438	*Kabuli*	*Ca_Kabuli_Chr03*	2931046	(C/T)
CakSNP3439	*Kabuli*	*Ca_Kabuli_Chr03*	2975179	(T/C)
CakSNP3440	*Kabuli*	*Ca_Kabuli_Chr03*	3280478	(C/T)
CakSNP3441	*Kabuli*	*Ca_Kabuli_Chr03*	3280500	(C/T)
CakSNP3442	*Kabuli*	*Ca_Kabuli_Chr03*	3280530	(G/A)
CakSNP3443	*Kabuli*	*Ca_Kabuli_Chr03*	3280595	(G/A)

SNP IDs	Cultivars	Chromosomes/scaffolds	Physical positions (bp)	SNPs
CakSNP3444	*Kabuli*	*Ca_Kabuli_Chr03*	3617555	(G/A)
CakSNP3445	*Kabuli*	*Ca_Kabuli_Chr03*	3701394	(A/T)
CakSNP3446	*Kabuli*	*Ca_Kabuli_Chr03*	3701396	(C/T)
CakSNP3447	*Kabuli*	*Ca_Kabuli_Chr03*	3701398	(A/T)
CakSNP3448	*Kabuli*	*Ca_Kabuli_Chr03*	3701401	(A/T)
CakSNP3449	*Kabuli*	*Ca_Kabuli_Chr03*	3701403	(A/T)
CakSNP3450	*Kabuli*	*Ca_Kabuli_Chr03*	3701405	(G/T)
CakSNP3451	*Kabuli*	*Ca_Kabuli_Chr03*	3701415	(C/T)
CakSNP3452	*Kabuli*	*Ca_Kabuli_Chr03*	3701422	(C/T)
CakSNP3453	*Kabuli*	*Ca_Kabuli_Chr03*	3701426	(T/C)
CakSNP3454	*Kabuli*	*Ca_Kabuli_Chr03*	3701435	(A/T)
CakSNP3455	*Kabuli*	*Ca_Kabuli_Chr03*	3701436	(C/T)
CakSNP3456	*Kabuli*	*Ca_Kabuli_Chr03*	3701437	(A/T)
CakSNP3457	*Kabuli*	*Ca_Kabuli_Chr03*	3701440	(G/T)
CakSNP3458	*Kabuli*	*Ca_Kabuli_Chr03*	3701450	(C/T)
CakSNP3459	*Kabuli*	*Ca_Kabuli_Chr03*	3701375	(G/A)
CakSNP3460	*Kabuli*	*Ca_Kabuli_Chr03*	3701370	(G/A)
CakSNP3461	*Kabuli*	*Ca_Kabuli_Chr03*	3744908	(G/A)
CakSNP3462	*Kabuli*	*Ca_Kabuli_Chr03*	3764420	(T/A)
CakSNP3463	*Kabuli*	*Ca_Kabuli_Chr03*	3764508	(T/C)
CakSNP3464	*Kabuli*	*Ca_Kabuli_Chr03*	3764545	(C/T)
CakSNP3465	*Kabuli*	*Ca_Kabuli_Chr03*	4145743	(G/A)
CakSNP3466	*Kabuli*	*Ca_Kabuli_Chr03*	4178154	(C/T)
CakSNP3467	*Kabuli*	*Ca_Kabuli_Chr03*	4266739	(G/A)
CakSNP3468	*Kabuli*	*Ca_Kabuli_Chr03*	4266700	(T/G)
CakSNP3469	*Kabuli*	*Ca_Kabuli_Chr03*	4266691	(C/T)
CakSNP3470	*Kabuli*	*Ca_Kabuli_Chr03*	4266686	(C/T)
CakSNP3471	*Kabuli*	*Ca_Kabuli_Chr03*	4266668	(G/A)
CakSNP3472	*Kabuli*	*Ca_Kabuli_Chr03*	4555124	(G/A)
CakSNP3473	*Kabuli*	*Ca_Kabuli_Chr03*	4555142	(T/A)
CakSNP3474	*Kabuli*	*Ca_Kabuli_Chr03*	4555167	(C/G)
CakSNP3475	*Kabuli*	*Ca_Kabuli_Chr03*	4555177	(C/T)
CakSNP3476	*Kabuli*	*Ca_Kabuli_Chr03*	4596815	(C/T)
CakSNP3477	*Kabuli*	*Ca_Kabuli_Chr03*	4596782	(A/G)
CakSNP3478	*Kabuli*	*Ca_Kabuli_Chr03*	4604649	(A/G)
CakSNP3479	*Kabuli*	*Ca_Kabuli_Chr03*	4606129	(T/A)
CakSNP3480	*Kabuli*	*Ca_Kabuli_Chr03*	4606128	(A/G)
CakSNP3481	*Kabuli*	*Ca_Kabuli_Chr03*	4629742	(A/T)
CakSNP3482	*Kabuli*	*Ca_Kabuli_Chr03*	4629747	(A/C)
CakSNP3483	*Kabuli*	*Ca_Kabuli_Chr03*	4654906	(C/T)
CakSNP3484	*Kabuli*	*Ca_Kabuli_Chr03*	4654898	(G/T)

SNP IDs	Cultivars	Chromosomes/scaffolds	Physical positions (bp)	SNPs
CakSNP3485	*Kabuli*	*Ca_Kabuli_Chr03*	4654896	(T/C)
CakSNP3486	*Kabuli*	*Ca_Kabuli_Chr03*	4823828	(T/C)
CakSNP3487	*Kabuli*	*Ca_Kabuli_Chr03*	5224781	(A/G)
CakSNP3488	*Kabuli*	*Ca_Kabuli_Chr03*	5225052	(A/G)
CakSNP3489	*Kabuli*	*Ca_Kabuli_Chr03*	5225063	(G/T)
CakSNP3490	*Kabuli*	*Ca_Kabuli_Chr03*	5225108	(T/G)
CakSNP3491	*Kabuli*	*Ca_Kabuli_Chr03*	5225079	(G/A)
CakSNP3492	*Kabuli*	*Ca_Kabuli_Chr03*	5415782	(G/A)
CakSNP3493	*Kabuli*	*Ca_Kabuli_Chr03*	5436267	(A/G)
CakSNP3494	*Kabuli*	*Ca_Kabuli_Chr03*	5436272	(T/C)
CakSNP3495	*Kabuli*	*Ca_Kabuli_Chr03*	5436277	(C/T)
CakSNP3496	*Kabuli*	*Ca_Kabuli_Chr03*	5436288	(G/T)
CakSNP3497	*Kabuli*	*Ca_Kabuli_Chr03*	5436295	(G/A)
CakSNP3498	*Kabuli*	*Ca_Kabuli_Chr03*	5436296	(C/T)
CakSNP3499	*Kabuli*	*Ca_Kabuli_Chr03*	5436299	(A/C)
CakSNP3500	*Kabuli*	*Ca_Kabuli_Chr03*	5436331	(C/A)
CakSNP3501	*Kabuli*	*Ca_Kabuli_Chr03*	5514176	(A/G)
CakSNP3502	*Kabuli*	*Ca_Kabuli_Chr03*	5514287	(C/A)
CakSNP3503	*Kabuli*	*Ca_Kabuli_Chr03*	6169456	(G/T)
CakSNP3504	*Kabuli*	*Ca_Kabuli_Chr03*	6169539	(T/C)
CakSNP3505	*Kabuli*	*Ca_Kabuli_Chr03*	6169510	(A/C)
CakSNP3506	*Kabuli*	*Ca_Kabuli_Chr03*	6278104	(G/A)
CakSNP3507	*Kabuli*	*Ca_Kabuli_Chr03*	6467014	(G/A)
CakSNP3508	*Kabuli*	*Ca_Kabuli_Chr03*	6683646	(A/G)
CakSNP3509	*Kabuli*	*Ca_Kabuli_Chr03*	6683587	(T/A)
CakSNP3510	*Kabuli*	*Ca_Kabuli_Chr03*	6828181	(A/T)
CakSNP3511	*Kabuli*	*Ca_Kabuli_Chr03*	6840644	(C/T)
CakSNP3512	*Kabuli*	*Ca_Kabuli_Chr03*	6894987	(C/A)
CakSNP3513	*Kabuli*	*Ca_Kabuli_Chr03*	6895085	(T/C)
CakSNP3514	*Kabuli*	*Ca_Kabuli_Chr03*	6899667	(T/C)
CakSNP3515	*Kabuli*	*Ca_Kabuli_Chr03*	7013492	(G/A)
CakSNP3516	*Kabuli*	*Ca_Kabuli_Chr03*	7015213	(T/G)
CakSNP3517	*Kabuli*	*Ca_Kabuli_Chr03*	7024620	(G/A)
CakSNP3518	*Kabuli*	*Ca_Kabuli_Chr03*	7204053	(G/A)
CakSNP3519	*Kabuli*	*Ca_Kabuli_Chr03*	7204071	(T/C)
CakSNP3520	*Kabuli*	*Ca_Kabuli_Chr03*	7204087	(C/T)
CakSNP3521	*Kabuli*	*Ca_Kabuli_Chr03*	7204088	(G/C)
CakSNP3522	*Kabuli*	*Ca_Kabuli_Chr03*	7204098	(G/A)
CakSNP3523	*Kabuli*	*Ca_Kabuli_Chr03*	7204099	(C/T)
CakSNP3524	*Kabuli*	*Ca_Kabuli_Chr03*	7204111	(G/A)
CakSNP3525	*Kabuli*	*Ca_Kabuli_Chr03*	7204112	(G/A)

SNP IDs	Cultivars	Chromosomes/scaffolds	Physical positions (bp)	SNPs
CakSNP3526	*Kabuli*	*Ca_Kabuli_Chr03*	7204133	(G/T)
CakSNP3527	*Kabuli*	*Ca_Kabuli_Chr03*	7226689	(T/C)
CakSNP3528	*Kabuli*	*Ca_Kabuli_Chr03*	7226661	(G/T)
CakSNP3529	*Kabuli*	*Ca_Kabuli_Chr03*	7226632	(G/T)
CakSNP3530	*Kabuli*	*Ca_Kabuli_Chr03*	7246049	(T/G)
CakSNP3531	*Kabuli*	*Ca_Kabuli_Chr03*	7420705	(G/A)
CakSNP3532	*Kabuli*	*Ca_Kabuli_Chr03*	7433747	(C/A)
CakSNP3533	*Kabuli*	*Ca_Kabuli_Chr03*	7461833	(A/G)
CakSNP3534	*Kabuli*	*Ca_Kabuli_Chr03*	7482871	(C/T)
CakSNP3535	*Kabuli*	*Ca_Kabuli_Chr03*	7606843	(A/G)
CakSNP3536	*Kabuli*	*Ca_Kabuli_Chr03*	7606901	(T/C)
CakSNP3537	*Kabuli*	*Ca_Kabuli_Chr03*	7606905	(C/G)
CakSNP3538	*Kabuli*	*Ca_Kabuli_Chr03*	7608100	(A/G)
CakSNP3539	*Kabuli*	*Ca_Kabuli_Chr03*	7665123	(G/A)
CakSNP3540	*Kabuli*	*Ca_Kabuli_Chr03*	7665112	(C/T)
CakSNP3541	*Kabuli*	*Ca_Kabuli_Chr03*	7665100	(G/T)
CakSNP3542	*Kabuli*	*Ca_Kabuli_Chr03*	7665098	(C/T)
CakSNP3543	*Kabuli*	*Ca_Kabuli_Chr03*	7665090	(C/T)
CakSNP3544	*Kabuli*	*Ca_Kabuli_Chr03*	7665078	(G/A)
CakSNP3545	*Kabuli*	*Ca_Kabuli_Chr03*	7665075	(G/A)
CakSNP3546	*Kabuli*	*Ca_Kabuli_Chr03*	7665156	(G/A)
CakSNP3547	*Kabuli*	*Ca_Kabuli_Chr03*	7665149	(T/G)
CakSNP3548	*Kabuli*	*Ca_Kabuli_Chr03*	7665142	(G/A)
CakSNP3549	*Kabuli*	*Ca_Kabuli_Chr03*	7665140	(G/T)
CakSNP3550	*Kabuli*	*Ca_Kabuli_Chr03*	7665157	(C/T)
CakSNP3551	*Kabuli*	*Ca_Kabuli_Chr03*	7694198	(C/T)
CakSNP3552	*Kabuli*	*Ca_Kabuli_Chr03*	7694297	(T/C)
CakSNP3553	*Kabuli*	*Ca_Kabuli_Chr03*	8536213	(T/C)
CakSNP3554	*Kabuli*	*Ca_Kabuli_Chr03*	8536275	(C/T)
CakSNP3555	*Kabuli*	*Ca_Kabuli_Chr03*	8536295	(A/T)
CakSNP3556	*Kabuli*	*Ca_Kabuli_Chr03*	8536301	(G/T)
CakSNP3557	*Kabuli*	*Ca_Kabuli_Chr03*	8536425	(C/A)
CakSNP3558	*Kabuli*	*Ca_Kabuli_Chr03*	8536498	(T/A)
CakSNP3559	*Kabuli*	*Ca_Kabuli_Chr03*	8536559	(T/C)
CakSNP3560	*Kabuli*	*Ca_Kabuli_Chr03*	8668628	(A/C)
CakSNP3561	*Kabuli*	*Ca_Kabuli_Chr03*	8669004	(A/T)
CakSNP3562	*Kabuli*	*Ca_Kabuli_Chr03*	8669132	(T/A)
CakSNP3563	*Kabuli*	*Ca_Kabuli_Chr03*	8697186	(A/G)
CakSNP3564	*Kabuli*	*Ca_Kabuli_Chr03*	8712381	(A/C)
CakSNP3565	*Kabuli*	*Ca_Kabuli_Chr03*	8933037	(T/C)
CakSNP3566	*Kabuli*	*Ca_Kabuli_Chr03*	8933084	(G/A)

SNP IDs	Cultivars	Chromosomes/scaffolds	Physical positions (bp)	SNPs
CakSNP3567	*Kabuli*	*Ca_Kabuli_Chr03*	8933086	(C/A)
CakSNP3568	*Kabuli*	*Ca_Kabuli_Chr03*	8933221	(T/A)
CakSNP3569	*Kabuli*	*Ca_Kabuli_Chr03*	8933183	(A/G)
CakSNP3570	*Kabuli*	*Ca_Kabuli_Chr03*	8933255	(A/T)
CakSNP3571	*Kabuli*	*Ca_Kabuli_Chr03*	8933266	(G/T)
CakSNP3572	*Kabuli*	*Ca_Kabuli_Chr03*	8933409	(A/C)
CakSNP3573	*Kabuli*	*Ca_Kabuli_Chr03*	8933370	(A/G)
CakSNP3574	*Kabuli*	*Ca_Kabuli_Chr03*	8933355	(A/T)
CakSNP3575	*Kabuli*	*Ca_Kabuli_Chr03*	9221403	(G/A)
CakSNP3576	*Kabuli*	*Ca_Kabuli_Chr03*	9221528	(C/T)
CakSNP3577	*Kabuli*	*Ca_Kabuli_Chr03*	9221579	(G/C)
CakSNP3578	*Kabuli*	*Ca_Kabuli_Chr03*	9221580	(T/G)
CakSNP3579	*Kabuli*	*Ca_Kabuli_Chr03*	9223444	(G/C)
CakSNP3580	*Kabuli*	*Ca_Kabuli_Chr03*	9223875	(G/A)
CakSNP3581	*Kabuli*	*Ca_Kabuli_Chr03*	9224033	(C/A)
CakSNP3582	*Kabuli*	*Ca_Kabuli_Chr03*	9224011	(G/A)
CakSNP3583	*Kabuli*	*Ca_Kabuli_Chr03*	9296469	(T/G)
CakSNP3584	*Kabuli*	*Ca_Kabuli_Chr03*	9448525	(T/C)
CakSNP3585	*Kabuli*	*Ca_Kabuli_Chr03*	9577402	(G/A)
CakSNP3586	*Kabuli*	*Ca_Kabuli_Chr03*	9579805	(A/T)
CakSNP3587	*Kabuli*	*Ca_Kabuli_Chr03*	9579808	(C/T)
CakSNP3588	*Kabuli*	*Ca_Kabuli_Chr03*	9579811	(G/C)
CakSNP3589	*Kabuli*	*Ca_Kabuli_Chr03*	9579817	(G/A)
CakSNP3590	*Kabuli*	*Ca_Kabuli_Chr03*	9579818	(G/T)
CakSNP3591	*Kabuli*	*Ca_Kabuli_Chr03*	9579824	(A/G)
CakSNP3592	*Kabuli*	*Ca_Kabuli_Chr03*	9579830	(T/A)
CakSNP3593	*Kabuli*	*Ca_Kabuli_Chr03*	9579831	(C/G)
CakSNP3594	*Kabuli*	*Ca_Kabuli_Chr03*	9579839	(G/A)
CakSNP3595	*Kabuli*	*Ca_Kabuli_Chr03*	9894316	(C/T)
CakSNP3596	*Kabuli*	*Ca_Kabuli_Chr03*	9894310	(C/A)
CakSNP3597	*Kabuli*	*Ca_Kabuli_Chr03*	10039539	(T/A)
CakSNP3598	*Kabuli*	*Ca_Kabuli_Chr03*	10105733	(A/G)
CakSNP3599	*Kabuli*	*Ca_Kabuli_Chr03*	10142152	(C/A)
CakSNP3600	*Kabuli*	*Ca_Kabuli_Chr03*	10159836	(A/C)
CakSNP3601	*Kabuli*	*Ca_Kabuli_Chr03*	10159859	(G/A)
CakSNP3602	*Kabuli*	*Ca_Kabuli_Chr03*	10159941	(C/A)
CakSNP3603	*Kabuli*	*Ca_Kabuli_Chr03*	10159943	(G/A)
CakSNP3604	*Kabuli*	*Ca_Kabuli_Chr03*	10159944	(T/C)
CakSNP3605	*Kabuli*	*Ca_Kabuli_Chr03*	10186893	(G/C)
CakSNP3606	*Kabuli*	*Ca_Kabuli_Chr03*	10186861	(G/T)
CakSNP3607	*Kabuli*	*Ca_Kabuli_Chr03*	10186857	(A/G)

SNP IDs	Cultivars	Chromosomes/scaffolds	Physical positions (bp)	SNPs
CakSNP3608	*Kabuli*	*Ca_Kabuli_Chr03*	10186880	(A/G)
CakSNP3609	*Kabuli*	*Ca_Kabuli_Chr03*	10186889	(G/A)
CakSNP3610	*Kabuli*	*Ca_Kabuli_Chr03*	10186891	(C/G)
CakSNP3611	*Kabuli*	*Ca_Kabuli_Chr03*	10201361	(G/T)
CakSNP3612	*Kabuli*	*Ca_Kabuli_Chr03*	10722780	(G/T)
CakSNP3613	*Kabuli*	*Ca_Kabuli_Chr03*	10722849	(G/C)
CakSNP3614	*Kabuli*	*Ca_Kabuli_Chr03*	10722859	(G/A)
CakSNP3615	*Kabuli*	*Ca_Kabuli_Chr03*	11143751	(G/A)
CakSNP3616	*Kabuli*	*Ca_Kabuli_Chr03*	11143818	(G/A)
CakSNP3617	*Kabuli*	*Ca_Kabuli_Chr03*	11143816	(C/T)
CakSNP3618	*Kabuli*	*Ca_Kabuli_Chr03*	11376752	(C/A)
CakSNP3619	*Kabuli*	*Ca_Kabuli_Chr03*	11378141	(C/T)
CakSNP3620	*Kabuli*	*Ca_Kabuli_Chr03*	11378425	(G/T)
CakSNP3621	*Kabuli*	*Ca_Kabuli_Chr03*	11590767	(C/T)
CakSNP3622	*Kabuli*	*Ca_Kabuli_Chr03*	11590793	(C/T)
CakSNP3623	*Kabuli*	*Ca_Kabuli_Chr03*	11590817	(C/G)
CakSNP3624	*Kabuli*	*Ca_Kabuli_Chr03*	11590874	(A/G)
CakSNP3625	*Kabuli*	*Ca_Kabuli_Chr03*	11590860	(G/A)
CakSNP3626	*Kabuli*	*Ca_Kabuli_Chr03*	11590832	(G/A)
CakSNP3627	*Kabuli*	*Ca_Kabuli_Chr03*	11590814	(T/C)
CakSNP3628	*Kabuli*	*Ca_Kabuli_Chr03*	11610500	(A/G)
CakSNP3629	*Kabuli*	*Ca_Kabuli_Chr03*	11610494	(C/T)
CakSNP3630	*Kabuli*	*Ca_Kabuli_Chr03*	11610460	(A/G)
CakSNP3631	*Kabuli*	*Ca_Kabuli_Chr03*	11610441	(G/T)
CakSNP3632	*Kabuli*	*Ca_Kabuli_Chr03*	11610481	(C/T)
CakSNP3633	*Kabuli*	*Ca_Kabuli_Chr03*	11610451	(C/T)
CakSNP3634	*Kabuli*	*Ca_Kabuli_Chr03*	11678625	(G/A)
CakSNP3635	*Kabuli*	*Ca_Kabuli_Chr03*	11684552	(G/A)
CakSNP3636	*Kabuli*	*Ca_Kabuli_Chr03*	11709777	(G/T)
CakSNP3637	*Kabuli*	*Ca_Kabuli_Chr03*	11811685	(A/G)
CakSNP3638	*Kabuli*	*Ca_Kabuli_Chr03*	12051826	(A/C)
CakSNP3639	*Kabuli*	*Ca_Kabuli_Chr03*	12058576	(T/C)
CakSNP3640	*Kabuli*	*Ca_Kabuli_Chr03*	12058756	(A/G)
CakSNP3641	*Kabuli*	*Ca_Kabuli_Chr03*	12058748	(A/T)
CakSNP3642	*Kabuli*	*Ca_Kabuli_Chr03*	12067458	(A/C)
CakSNP3643	*Kabuli*	*Ca_Kabuli_Chr03*	12069092	(T/G)
CakSNP3644	*Kabuli*	*Ca_Kabuli_Chr03*	12095571	(T/G)
CakSNP3645	*Kabuli*	*Ca_Kabuli_Chr03*	12124099	(G/A)
CakSNP3646	*Kabuli*	*Ca_Kabuli_Chr03*	12394742	(G/A)
CakSNP3647	*Kabuli*	*Ca_Kabuli_Chr03*	12599496	(A/C)
CakSNP3648	*Kabuli*	*Ca_Kabuli_Chr03*	12622503	(A/G)

SNP IDs	Cultivars	Chromosomes/scaffolds	Physical positions (bp)	SNPs
CakSNP3649	*Kabuli*	*Ca_Kabuli_Chr03*	12622504	(G/T)
CakSNP3650	*Kabuli*	*Ca_Kabuli_Chr03*	12622518	(T/G)
CakSNP3651	*Kabuli*	*Ca_Kabuli_Chr03*	12622534	(C/T)
CakSNP3652	*Kabuli*	*Ca_Kabuli_Chr03*	12622550	(A/T)
CakSNP3653	*Kabuli*	*Ca_Kabuli_Chr03*	12622578	(G/A)
CakSNP3654	*Kabuli*	*Ca_Kabuli_Chr03*	12622567	(A/C)
CakSNP3655	*Kabuli*	*Ca_Kabuli_Chr03*	12622543	(G/A)
CakSNP3656	*Kabuli*	*Ca_Kabuli_Chr03*	12627120	(C/T)
CakSNP3657	*Kabuli*	*Ca_Kabuli_Chr03*	12627125	(T/C)
CakSNP3658	*Kabuli*	*Ca_Kabuli_Chr03*	12627131	(G/A)
CakSNP3659	*Kabuli*	*Ca_Kabuli_Chr03*	12627144	(G/A)
CakSNP3660	*Kabuli*	*Ca_Kabuli_Chr03*	12627165	(T/C)
CakSNP3661	*Kabuli*	*Ca_Kabuli_Chr03*	12627143	(T/C)
CakSNP3662	*Kabuli*	*Ca_Kabuli_Chr03*	12627164	(G/A)
CakSNP3663	*Kabuli*	*Ca_Kabuli_Chr03*	12627170	(C/G)
CakSNP3664	*Kabuli*	*Ca_Kabuli_Chr03*	12809390	(A/G)
CakSNP3665	*Kabuli*	*Ca_Kabuli_Chr03*	12809794	(T/C)
CakSNP3666	*Kabuli*	*Ca_Kabuli_Chr03*	12814631	(C/T)
CakSNP3667	*Kabuli*	*Ca_Kabuli_Chr03*	12814603	(T/C)
CakSNP3668	*Kabuli*	*Ca_Kabuli_Chr03*	12814601	(A/C)
CakSNP3669	*Kabuli*	*Ca_Kabuli_Chr03*	12814600	(A/G)
CakSNP3670	*Kabuli*	*Ca_Kabuli_Chr03*	12814599	(A/T)
CakSNP3671	*Kabuli*	*Ca_Kabuli_Chr03*	12814597	(T/A)
CakSNP3672	*Kabuli*	*Ca_Kabuli_Chr03*	12814571	(C/A)
CakSNP3673	*Kabuli*	*Ca_Kabuli_Chr03*	12814558	(A/G)
CakSNP3674	*Kabuli*	*Ca_Kabuli_Chr03*	12897820	(C/T)
CakSNP3675	*Kabuli*	*Ca_Kabuli_Chr03*	12897831	(C/A)
CakSNP3676	*Kabuli*	*Ca_Kabuli_Chr03*	12897839	(G/A)
CakSNP3677	*Kabuli*	*Ca_Kabuli_Chr03*	12897841	(C/A)
CakSNP3678	*Kabuli*	*Ca_Kabuli_Chr03*	12897876	(C/T)
CakSNP3679	*Kabuli*	*Ca_Kabuli_Chr03*	12897881	(C/T)
CakSNP3680	*Kabuli*	*Ca_Kabuli_Chr03*	12897882	(T/G)
CakSNP3681	*Kabuli*	*Ca_Kabuli_Chr03*	12897926	(A/C)
CakSNP3682	*Kabuli*	*Ca_Kabuli_Chr03*	12897906	(C/A)
CakSNP3683	*Kabuli*	*Ca_Kabuli_Chr03*	12897887	(T/C)
CakSNP3684	*Kabuli*	*Ca_Kabuli_Chr03*	12897884	(C/T)
CakSNP3685	*Kabuli*	*Ca_Kabuli_Chr03*	12897883	(G/T)
CakSNP3686	*Kabuli*	*Ca_Kabuli_Chr03*	12897886	(G/A)
CakSNP3687	*Kabuli*	*Ca_Kabuli_Chr03*	13185541	(G/A)
CakSNP3688	*Kabuli*	*Ca_Kabuli_Chr03*	13185530	(C/T)
CakSNP3689	*Kabuli*	*Ca_Kabuli_Chr03*	13185496	(G/A)

SNP IDs	Cultivars	Chromosomes/scaffolds	Physical positions (bp)	SNPs
CakSNP3690	*Kabuli*	*Ca_Kabuli_Chr03*	13185490	(G/A)
CakSNP3691	*Kabuli*	*Ca_Kabuli_Chr03*	13185493	(G/A)
CakSNP3692	*Kabuli*	*Ca_Kabuli_Chr03*	13185508	(C/T)
CakSNP3693	*Kabuli*	*Ca_Kabuli_Chr03*	13185516	(C/T)
CakSNP3694	*Kabuli*	*Ca_Kabuli_Chr03*	13185583	(T/C)
CakSNP3695	*Kabuli*	*Ca_Kabuli_Chr03*	13185581	(T/A)
CakSNP3696	*Kabuli*	*Ca_Kabuli_Chr03*	13185574	(G/A)
CakSNP3697	*Kabuli*	*Ca_Kabuli_Chr03*	13185560	(G/A)
CakSNP3698	*Kabuli*	*Ca_Kabuli_Chr03*	13185575	(C/T)
CakSNP3699	*Kabuli*	*Ca_Kabuli_Chr03*	13185582	(A/G)
CakSNP3700	*Kabuli*	*Ca_Kabuli_Chr03*	13185590	(C/A)
CakSNP3701	*Kabuli*	*Ca_Kabuli_Chr03*	13185591	(C/T)
CakSNP3702	*Kabuli*	*Ca_Kabuli_Chr03*	13185592	(A/C)
CakSNP3703	*Kabuli*	*Ca_Kabuli_Chr03*	13202989	(C/T)
CakSNP3704	*Kabuli*	*Ca_Kabuli_Chr03*	13396925	(A/T)
CakSNP3705	*Kabuli*	*Ca_Kabuli_Chr03*	13397207	(A/C)
CakSNP3706	*Kabuli*	*Ca_Kabuli_Chr03*	13420776	(T/C)
CakSNP3707	*Kabuli*	*Ca_Kabuli_Chr03*	13420758	(A/G)
CakSNP3708	*Kabuli*	*Ca_Kabuli_Chr03*	13420756	(A/G)
CakSNP3709	*Kabuli*	*Ca_Kabuli_Chr03*	13617948	(A/C)
CakSNP3710	*Kabuli*	*Ca_Kabuli_Chr03*	14200381	(A/C)
CakSNP3711	*Kabuli*	*Ca_Kabuli_Chr03*	14465236	(T/G)
CakSNP3712	*Kabuli*	*Ca_Kabuli_Chr03*	14497374	(C/A)
CakSNP3713	*Kabuli*	*Ca_Kabuli_Chr03*	14653195	(G/T)
CakSNP3714	*Kabuli*	*Ca_Kabuli_Chr03*	14653194	(C/T)
CakSNP3715	*Kabuli*	*Ca_Kabuli_Chr03*	14653193	(T/G)
CakSNP3716	*Kabuli*	*Ca_Kabuli_Chr03*	14653270	(C/A)
CakSNP3717	*Kabuli*	*Ca_Kabuli_Chr03*	14653271	(G/A)
CakSNP3718	*Kabuli*	*Ca_Kabuli_Chr03*	14658175	(A/C)
CakSNP3719	*Kabuli*	*Ca_Kabuli_Chr03*	14812960	(A/C)
CakSNP3720	*Kabuli*	*Ca_Kabuli_Chr03*	14982667	(C/T)
CakSNP3721	*Kabuli*	*Ca_Kabuli_Chr03*	14982739	(A/T)
CakSNP3722	*Kabuli*	*Ca_Kabuli_Chr03*	15744906	(T/A)
CakSNP3723	*Kabuli*	*Ca_Kabuli_Chr03*	15745010	(C/T)
CakSNP3724	*Kabuli*	*Ca_Kabuli_Chr03*	15745017	(A/G)
CakSNP3725	*Kabuli*	*Ca_Kabuli_Chr03*	15745264	(G/C)
CakSNP3726	*Kabuli*	*Ca_Kabuli_Chr03*	15745311	(G/A)
CakSNP3727	*Kabuli*	*Ca_Kabuli_Chr03*	15757804	(T/C)
CakSNP3728	*Kabuli*	*Ca_Kabuli_Chr03*	15757829	(C/T)
CakSNP3729	*Kabuli*	*Ca_Kabuli_Chr03*	15757852	(A/C)
CakSNP3730	*Kabuli*	*Ca_Kabuli_Chr03*	16071411	(G/C)

SNP IDs	Cultivars	Chromosomes/scaffolds	Physical positions (bp)	SNPs
CakSNP3731	*Kabuli*	*Ca_Kabuli_Chr03*	16396662	(C/G)
CakSNP3732	*Kabuli*	*Ca_Kabuli_Chr03*	16396673	(A/C)
CakSNP3733	*Kabuli*	*Ca_Kabuli_Chr03*	16436286	(T/A)
CakSNP3734	*Kabuli*	*Ca_Kabuli_Chr03*	16458123	(G/T)
CakSNP3735	*Kabuli*	*Ca_Kabuli_Chr03*	16458413	(T/A)
CakSNP3736	*Kabuli*	*Ca_Kabuli_Chr03*	16540328	(T/G)
CakSNP3737	*Kabuli*	*Ca_Kabuli_Chr03*	16568567	(A/G)
CakSNP3738	*Kabuli*	*Ca_Kabuli_Chr03*	16621498	(G/A)
CakSNP3739	*Kabuli*	*Ca_Kabuli_Chr03*	16621624	(G/T)
CakSNP3740	*Kabuli*	*Ca_Kabuli_Chr03*	16768867	(A/G)
CakSNP3741	*Kabuli*	*Ca_Kabuli_Chr03*	16768936	(A/G)
CakSNP3742	*Kabuli*	*Ca_Kabuli_Chr03*	16768917	(C/T)
CakSNP3743	*Kabuli*	*Ca_Kabuli_Chr03*	16948510	(G/T)
CakSNP3744	*Kabuli*	*Ca_Kabuli_Chr03*	16948854	(A/T)
CakSNP3745	*Kabuli*	*Ca_Kabuli_Chr03*	16995243	(C/T)
CakSNP3746	*Kabuli*	*Ca_Kabuli_Chr03*	16997328	(G/A)
CakSNP3747	*Kabuli*	*Ca_Kabuli_Chr03*	16997331	(C/T)
CakSNP3748	*Kabuli*	*Ca_Kabuli_Chr03*	16997400	(T/C)
CakSNP3749	*Kabuli*	*Ca_Kabuli_Chr03*	17136071	(G/C)
CakSNP3750	*Kabuli*	*Ca_Kabuli_Chr03*	17136097	(T/C)
CakSNP3751	*Kabuli*	*Ca_Kabuli_Chr03*	17145639	(A/T)
CakSNP3752	*Kabuli*	*Ca_Kabuli_Chr03*	17145643	(G/T)
CakSNP3753	*Kabuli*	*Ca_Kabuli_Chr03*	17181213	(A/G)
CakSNP3754	*Kabuli*	*Ca_Kabuli_Chr03*	17185682	(A/G)
CakSNP3755	*Kabuli*	*Ca_Kabuli_Chr03*	17334924	(G/T)
CakSNP3756	*Kabuli*	*Ca_Kabuli_Chr03*	17335851	(A/C)
CakSNP3757	*Kabuli*	*Ca_Kabuli_Chr03*	17351553	(A/T)
CakSNP3758	*Kabuli*	*Ca_Kabuli_Chr03*	17351526	(A/C)
CakSNP3759	*Kabuli*	*Ca_Kabuli_Chr03*	17459986	(T/A)
CakSNP3760	*Kabuli*	*Ca_Kabuli_Chr03*	17460002	(G/A)
CakSNP3761	*Kabuli*	*Ca_Kabuli_Chr03*	17460012	(C/A)
CakSNP3762	*Kabuli*	*Ca_Kabuli_Chr03*	17574247	(C/T)
CakSNP3763	*Kabuli*	*Ca_Kabuli_Chr03*	17635591	(T/A)
CakSNP3764	*Kabuli*	*Ca_Kabuli_Chr03*	17635531	(T/C)
CakSNP3765	*Kabuli*	*Ca_Kabuli_Chr03*	17679409	(C/T)
CakSNP3766	*Kabuli*	*Ca_Kabuli_Chr03*	17679404	(C/T)
CakSNP3767	*Kabuli*	*Ca_Kabuli_Chr03*	17914677	(T/G)
CakSNP3768	*Kabuli*	*Ca_Kabuli_Chr03*	17936321	(T/C)
CakSNP3769	*Kabuli*	*Ca_Kabuli_Chr03*	17936729	(G/A)
CakSNP3770	*Kabuli*	*Ca_Kabuli_Chr03*	17936842	(A/G)
CakSNP3771	*Kabuli*	*Ca_Kabuli_Chr03*	17984978	(T/G)

**SNP IDs**	**Cultivars**	**Chromosomes/scaffolds**	**Physical positions (bp)**	**SNPs**
CakSNP3772	*Kabuli*	*Ca_Kabuli_Chr03*	17986521	(G/C)
CakSNP3773	*Kabuli*	*Ca_Kabuli_Chr03*	17986525	(G/T)
CakSNP3774	*Kabuli*	*Ca_Kabuli_Chr03*	17986740	(T/C)
CakSNP3775	*Kabuli*	*Ca_Kabuli_Chr03*	18215321	(G/C)
CakSNP3776	*Kabuli*	*Ca_Kabuli_Chr03*	18215332	(T/C)
CakSNP3777	*Kabuli*	*Ca_Kabuli_Chr03*	18355427	(T/C)
CakSNP3778	*Kabuli*	*Ca_Kabuli_Chr03*	18355445	(T/C)
CakSNP3779	*Kabuli*	*Ca_Kabuli_Chr03*	18375547	(T/C)
CakSNP3780	*Kabuli*	*Ca_Kabuli_Chr03*	18375581	(G/C)
CakSNP3781	*Kabuli*	*Ca_Kabuli_Chr03*	18382404	(T/C)
CakSNP3782	*Kabuli*	*Ca_Kabuli_Chr03*	18382438	(G/C)
CakSNP3783	*Kabuli*	*Ca_Kabuli_Chr03*	18383884	(T/C)
CakSNP3784	*Kabuli*	*Ca_Kabuli_Chr03*	18476006	(A/G)
CakSNP3785	*Kabuli*	*Ca_Kabuli_Chr03*	18476065	(C/A)
CakSNP3786	*Kabuli*	*Ca_Kabuli_Chr03*	18497633	(T/C)
CakSNP3787	*Kabuli*	*Ca_Kabuli_Chr03*	18573701	(T/A)
CakSNP3788	*Kabuli*	*Ca_Kabuli_Chr03*	18645114	(T/C)
CakSNP3789	*Kabuli*	*Ca_Kabuli_Chr03*	18687994	(T/A)
CakSNP3790	*Kabuli*	*Ca_Kabuli_Chr03*	18709398	(G/A)
CakSNP3791	*Kabuli*	*Ca_Kabuli_Chr03*	18731098	(T/C)
CakSNP3792	*Kabuli*	*Ca_Kabuli_Chr03*	18825708	(T/C)
CakSNP3793	*Kabuli*	*Ca_Kabuli_Chr03*	18825687	(T/C)
CakSNP3794	*Kabuli*	*Ca_Kabuli_Chr03*	18939425	(C/G)
CakSNP3795	*Kabuli*	*Ca_Kabuli_Chr03*	19372722	(G/T)
CakSNP3796	*Kabuli*	*Ca_Kabuli_Chr03*	19372739	(C/T)
CakSNP3797	*Kabuli*	*Ca_Kabuli_Chr03*	19400104	(T/C)
CakSNP3798	*Kabuli*	*Ca_Kabuli_Chr03*	19400121	(G/T)
CakSNP3799	*Kabuli*	*Ca_Kabuli_Chr03*	19400276	(C/T)
CakSNP3800	*Kabuli*	*Ca_Kabuli_Chr03*	19442839	(G/A)
CakSNP3801	*Kabuli*	*Ca_Kabuli_Chr03*	19442884	(G/C)
CakSNP3802	*Kabuli*	*Ca_Kabuli_Chr03*	19442941	(G/A)
CakSNP3803	*Kabuli*	*Ca_Kabuli_Chr03*	19445925	(A/G)
CakSNP3804	*Kabuli*	*Ca_Kabuli_Chr03*	19445920	(T/A)
CakSNP3805	*Kabuli*	*Ca_Kabuli_Chr03*	19448140	(A/G)
CakSNP3806	*Kabuli*	*Ca_Kabuli_Chr03*	19475095	(G/T)
CakSNP3807	*Kabuli*	*Ca_Kabuli_Chr03*	19571396	(C/A)
CakSNP3808	*Kabuli*	*Ca_Kabuli_Chr03*	19571382	(G/T)
CakSNP3809	*Kabuli*	*Ca_Kabuli_Chr03*	19571371	(A/G)
CakSNP3810	*Kabuli*	*Ca_Kabuli_Chr03*	19742600	(G/A)
CakSNP3811	*Kabuli*	*Ca_Kabuli_Chr03*	19742530	(C/T)
CakSNP3812	*Kabuli*	*Ca_Kabuli_Chr03*	19788512	(G/A)

SNP IDs	Cultivars	Chromosomes/scaffolds	Physical positions (bp)	SNPs
CakSNP3813	*Kabuli*	*Ca_Kabuli_Chr03*	19802763	(C/T)
CakSNP3814	*Kabuli*	*Ca_Kabuli_Chr03*	19804985	(T/C)
CakSNP3815	*Kabuli*	*Ca_Kabuli_Chr03*	19805063	(A/C)
CakSNP3816	*Kabuli*	*Ca_Kabuli_Chr03*	19829510	(G/T)
CakSNP3817	*Kabuli*	*Ca_Kabuli_Chr03*	19850532	(T/C)
CakSNP3818	*Kabuli*	*Ca_Kabuli_Chr03*	19850589	(C/T)
CakSNP3819	*Kabuli*	*Ca_Kabuli_Chr03*	19855217	(T/C)
CakSNP3820	*Kabuli*	*Ca_Kabuli_Chr03*	19857613	(T/A)
CakSNP3821	*Kabuli*	*Ca_Kabuli_Chr03*	19883898	(T/C)
CakSNP3822	*Kabuli*	*Ca_Kabuli_Chr03*	19883927	(A/G)
CakSNP3823	*Kabuli*	*Ca_Kabuli_Chr03*	19883936	(T/G)
CakSNP3824	*Kabuli*	*Ca_Kabuli_Chr03*	19896800	(C/T)
CakSNP3825	*Kabuli*	*Ca_Kabuli_Chr03*	19896849	(G/A)
CakSNP3826	*Kabuli*	*Ca_Kabuli_Chr03*	19907774	(T/C)
CakSNP3827	*Kabuli*	*Ca_Kabuli_Chr03*	19956343	(C/T)
CakSNP3828	*Kabuli*	*Ca_Kabuli_Chr03*	19956391	(C/T)
CakSNP3829	*Kabuli*	*Ca_Kabuli_Chr03*	19978343	(A/C)
CakSNP3830	*Kabuli*	*Ca_Kabuli_Chr03*	19978334	(A/C)
CakSNP3831	*Kabuli*	*Ca_Kabuli_Chr03*	19978407	(C/T)
CakSNP3832	*Kabuli*	*Ca_Kabuli_Chr03*	19979288	(A/T)
CakSNP3833	*Kabuli*	*Ca_Kabuli_Chr03*	19979318	(C/A)
CakSNP3834	*Kabuli*	*Ca_Kabuli_Chr03*	19979390	(A/T)
CakSNP3835	*Kabuli*	*Ca_Kabuli_Chr03*	20000967	(C/G)
CakSNP3836	*Kabuli*	*Ca_Kabuli_Chr03*	20030262	(T/C)
CakSNP3837	*Kabuli*	*Ca_Kabuli_Chr03*	20030298	(G/A)
CakSNP3838	*Kabuli*	*Ca_Kabuli_Chr03*	20030484	(A/G)
CakSNP3839	*Kabuli*	*Ca_Kabuli_Chr03*	20103039	(G/A)
CakSNP3840	*Kabuli*	*Ca_Kabuli_Chr03*	20103060	(C/T)
CakSNP3841	*Kabuli*	*Ca_Kabuli_Chr03*	20103126	(A/C)
CakSNP3842	*Kabuli*	*Ca_Kabuli_Chr03*	20172492	(C/T)
CakSNP3843	*Kabuli*	*Ca_Kabuli_Chr03*	20240118	(T/G)
CakSNP3844	*Kabuli*	*Ca_Kabuli_Chr03*	20242663	(C/T)
CakSNP3845	*Kabuli*	*Ca_Kabuli_Chr03*	20331659	(G/A)
CakSNP3846	*Kabuli*	*Ca_Kabuli_Chr03*	20365442	(A/C)
CakSNP3847	*Kabuli*	*Ca_Kabuli_Chr03*	20387399	(T/A)
CakSNP3848	*Kabuli*	*Ca_Kabuli_Chr03*	20387471	(A/G)
CakSNP3849	*Kabuli*	*Ca_Kabuli_Chr03*	20415660	(C/G)
CakSNP3850	*Kabuli*	*Ca_Kabuli_Chr03*	20415615	(A/G)
CakSNP3851	*Kabuli*	*Ca_Kabuli_Chr03*	20462715	(T/A)
CakSNP3852	*Kabuli*	*Ca_Kabuli_Chr03*	20490488	(T/C)
CakSNP3853	*Kabuli*	*Ca_Kabuli_Chr03*	20495810	(G/A)

SNP IDs	Cultivars	Chromosomes/scaffolds	Physical positions (bp)	SNPs
CakSNP3854	*Kabuli*	*Ca_Kabuli_Chr03*	20503939	(G/T)
CakSNP3855	*Kabuli*	*Ca_Kabuli_Chr03*	20506663	(C/G)
CakSNP3856	*Kabuli*	*Ca_Kabuli_Chr03*	20552836	(T/G)
CakSNP3857	*Kabuli*	*Ca_Kabuli_Chr03*	20555942	(G/A)
CakSNP3858	*Kabuli*	*Ca_Kabuli_Chr03*	20690207	(T/G)
CakSNP3859	*Kabuli*	*Ca_Kabuli_Chr03*	20690185	(A/T)
CakSNP3860	*Kabuli*	*Ca_Kabuli_Chr03*	20794236	(A/T)
CakSNP3861	*Kabuli*	*Ca_Kabuli_Chr03*	20794206	(A/T)
CakSNP3862	*Kabuli*	*Ca_Kabuli_Chr03*	20794984	(C/T)
CakSNP3863	*Kabuli*	*Ca_Kabuli_Chr03*	20795019	(C/T)
CakSNP3864	*Kabuli*	*Ca_Kabuli_Chr03*	20795014	(C/T)
CakSNP3865	*Kabuli*	*Ca_Kabuli_Chr03*	20794976	(A/C)
CakSNP3866	*Kabuli*	*Ca_Kabuli_Chr03*	20794993	(A/G)
CakSNP3867	*Kabuli*	*Ca_Kabuli_Chr03*	20794990	(G/A)
CakSNP3868	*Kabuli*	*Ca_Kabuli_Chr03*	20799767	(A/G)
CakSNP3869	*Kabuli*	*Ca_Kabuli_Chr03*	20799778	(C/T)
CakSNP3870	*Kabuli*	*Ca_Kabuli_Chr03*	20799811	(C/T)
CakSNP3871	*Kabuli*	*Ca_Kabuli_Chr03*	20799860	(A/G)
CakSNP3872	*Kabuli*	*Ca_Kabuli_Chr03*	20799841	(C/T)
CakSNP3873	*Kabuli*	*Ca_Kabuli_Chr03*	20799819	(C/T)
CakSNP3874	*Kabuli*	*Ca_Kabuli_Chr03*	20799820	(A/G)
CakSNP3875	*Kabuli*	*Ca_Kabuli_Chr03*	20822676	(T/C)
CakSNP3876	*Kabuli*	*Ca_Kabuli_Chr03*	20823146	(C/T)
CakSNP3877	*Kabuli*	*Ca_Kabuli_Chr03*	20828272	(G/T)
CakSNP3878	*Kabuli*	*Ca_Kabuli_Chr03*	20829399	(A/G)
CakSNP3879	*Kabuli*	*Ca_Kabuli_Chr03*	20829407	(T/G)
CakSNP3880	*Kabuli*	*Ca_Kabuli_Chr03*	20841298	(T/G)
CakSNP3881	*Kabuli*	*Ca_Kabuli_Chr03*	20841352	(C/T)
CakSNP3882	*Kabuli*	*Ca_Kabuli_Chr03*	20880294	(T/A)
CakSNP3883	*Kabuli*	*Ca_Kabuli_Chr03*	21020182	(A/G)
CakSNP3884	*Kabuli*	*Ca_Kabuli_Chr03*	21020191	(C/T)
CakSNP3885	*Kabuli*	*Ca_Kabuli_Chr03*	21069672	(A/G)
CakSNP3886	*Kabuli*	*Ca_Kabuli_Chr03*	21069714	(G/A)
CakSNP3887	*Kabuli*	*Ca_Kabuli_Chr03*	21069916	(C/T)
CakSNP3888	*Kabuli*	*Ca_Kabuli_Chr03*	21069917	(G/A)
CakSNP3889	*Kabuli*	*Ca_Kabuli_Chr03*	21069938	(C/A)
CakSNP3890	*Kabuli*	*Ca_Kabuli_Chr03*	21069959	(G/A)
CakSNP3891	*Kabuli*	*Ca_Kabuli_Chr03*	21069986	(A/C)
CakSNP3892	*Kabuli*	*Ca_Kabuli_Chr03*	21127162	(C/G)
CakSNP3893	*Kabuli*	*Ca_Kabuli_Chr03*	21127156	(T/G)
CakSNP3894	*Kabuli*	*Ca_Kabuli_Chr03*	21198607	(A/T)

SNP IDs	Cultivars	Chromosomes/scaffolds	Physical positions (bp)	SNPs
CakSNP3895	*Kabuli*	*Ca_Kabuli_Chr03*	21210002	(T/A)
CakSNP3896	*Kabuli*	*Ca_Kabuli_Chr03*	21210005	(C/A)
CakSNP3897	*Kabuli*	*Ca_Kabuli_Chr03*	21210008	(T/C)
CakSNP3898	*Kabuli*	*Ca_Kabuli_Chr03*	21210049	(A/G)
CakSNP3899	*Kabuli*	*Ca_Kabuli_Chr03*	21273368	(A/G)
CakSNP3900	*Kabuli*	*Ca_Kabuli_Chr03*	21288626	(C/A)
CakSNP3901	*Kabuli*	*Ca_Kabuli_Chr03*	21288652	(G/C)
CakSNP3902	*Kabuli*	*Ca_Kabuli_Chr03*	21288695	(G/A)
CakSNP3903	*Kabuli*	*Ca_Kabuli_Chr03*	21335892	(C/T)
CakSNP3904	*Kabuli*	*Ca_Kabuli_Chr03*	21382790	(G/A)
CakSNP3905	*Kabuli*	*Ca_Kabuli_Chr03*	21421082	(A/G)
CakSNP3906	*Kabuli*	*Ca_Kabuli_Chr03*	21421085	(G/A)
CakSNP3907	*Kabuli*	*Ca_Kabuli_Chr03*	21421146	(C/A)
CakSNP3908	*Kabuli*	*Ca_Kabuli_Chr03*	21421162	(G/A)
CakSNP3909	*Kabuli*	*Ca_Kabuli_Chr03*	21421358	(G/A)
CakSNP3910	*Kabuli*	*Ca_Kabuli_Chr03*	21421362	(T/A)
CakSNP3911	*Kabuli*	*Ca_Kabuli_Chr03*	21421369	(G/A)
CakSNP3912	*Kabuli*	*Ca_Kabuli_Chr03*	21421424	(G/A)
CakSNP3913	*Kabuli*	*Ca_Kabuli_Chr03*	21421440	(C/T)
CakSNP3914	*Kabuli*	*Ca_Kabuli_Chr03*	21450650	(A/G)
CakSNP3915	*Kabuli*	*Ca_Kabuli_Chr03*	21452017	(G/A)
CakSNP3916	*Kabuli*	*Ca_Kabuli_Chr03*	21468634	(G/A)
CakSNP3917	*Kabuli*	*Ca_Kabuli_Chr03*	21468692	(A/C)
CakSNP3918	*Kabuli*	*Ca_Kabuli_Chr03*	21470784	(C/T)
CakSNP3919	*Kabuli*	*Ca_Kabuli_Chr03*	21500178	(G/A)
CakSNP3920	*Kabuli*	*Ca_Kabuli_Chr03*	21500179	(A/T)
CakSNP3921	*Kabuli*	*Ca_Kabuli_Chr03*	21507422	(T/C)
CakSNP3922	*Kabuli*	*Ca_Kabuli_Chr03*	21524084	(T/C)
CakSNP3923	*Kabuli*	*Ca_Kabuli_Chr03*	21524192	(T/C)
CakSNP3924	*Kabuli*	*Ca_Kabuli_Chr03*	21524296	(G/T)
CakSNP3925	*Kabuli*	*Ca_Kabuli_Chr03*	21524379	(A/G)
CakSNP3926	*Kabuli*	*Ca_Kabuli_Chr03*	21525184	(C/T)
CakSNP3927	*Kabuli*	*Ca_Kabuli_Chr03*	21525177	(G/A)
CakSNP3928	*Kabuli*	*Ca_Kabuli_Chr03*	21558388	(A/G)
CakSNP3929	*Kabuli*	*Ca_Kabuli_Chr03*	21558357	(T/G)
CakSNP3930	*Kabuli*	*Ca_Kabuli_Chr03*	21627610	(T/C)
CakSNP3931	*Kabuli*	*Ca_Kabuli_Chr03*	21634275	(A/C)
CakSNP3932	*Kabuli*	*Ca_Kabuli_Chr03*	21634271	(A/G)
CakSNP3933	*Kabuli*	*Ca_Kabuli_Chr03*	21646863	(C/T)
CakSNP3934	*Kabuli*	*Ca_Kabuli_Chr03*	21646872	(T/C)
CakSNP3935	*Kabuli*	*Ca_Kabuli_Chr03*	21646893	(C/A)

SNP IDs	Cultivars	Chromosomes/scaffolds	Physical positions (bp)	SNPs
CakSNP3936	*Kabuli*	*Ca_Kabuli_Chr03*	21646965	(C/T)
CakSNP3937	*Kabuli*	*Ca_Kabuli_Chr03*	21646962	(A/G)
CakSNP3938	*Kabuli*	*Ca_Kabuli_Chr03*	21654592	(T/A)
CakSNP3939	*Kabuli*	*Ca_Kabuli_Chr03*	21686470	(C/T)
CakSNP3940	*Kabuli*	*Ca_Kabuli_Chr03*	21687439	(G/T)
CakSNP3941	*Kabuli*	*Ca_Kabuli_Chr03*	21687966	(C/A)
CakSNP3942	*Kabuli*	*Ca_Kabuli_Chr03*	21687961	(C/A)
CakSNP3943	*Kabuli*	*Ca_Kabuli_Chr03*	21687960	(G/A)
CakSNP3944	*Kabuli*	*Ca_Kabuli_Chr03*	21687959	(T/C)
CakSNP3945	*Kabuli*	*Ca_Kabuli_Chr03*	21687956	(C/T)
CakSNP3946	*Kabuli*	*Ca_Kabuli_Chr03*	21687953	(C/T)
CakSNP3947	*Kabuli*	*Ca_Kabuli_Chr03*	21741914	(C/A)
CakSNP3948	*Kabuli*	*Ca_Kabuli_Chr03*	21809395	(G/A)
CakSNP3949	*Kabuli*	*Ca_Kabuli_Chr03*	21809456	(T/C)
CakSNP3950	*Kabuli*	*Ca_Kabuli_Chr03*	21809457	(G/A)
CakSNP3951	*Kabuli*	*Ca_Kabuli_Chr03*	21809546	(T/A)
CakSNP3952	*Kabuli*	*Ca_Kabuli_Chr03*	21809505	(A/G)
CakSNP3953	*Kabuli*	*Ca_Kabuli_Chr03*	21809503	(T/C)
CakSNP3954	*Kabuli*	*Ca_Kabuli_Chr03*	21950053	(C/G)
CakSNP3955	*Kabuli*	*Ca_Kabuli_Chr03*	21950186	(A/G)
CakSNP3956	*Kabuli*	*Ca_Kabuli_Chr03*	21950164	(A/G)
CakSNP3957	*Kabuli*	*Ca_Kabuli_Chr03*	22008087	(G/A)
CakSNP3958	*Kabuli*	*Ca_Kabuli_Chr03*	22026111	(A/G)
CakSNP3959	*Kabuli*	*Ca_Kabuli_Chr03*	22026647	(A/G)
CakSNP3960	*Kabuli*	*Ca_Kabuli_Chr03*	22060199	(G/T)
CakSNP3961	*Kabuli*	*Ca_Kabuli_Chr03*	22061236	(A/G)
CakSNP3962	*Kabuli*	*Ca_Kabuli_Chr03*	22062029	(G/T)
CakSNP3963	*Kabuli*	*Ca_Kabuli_Chr03*	22062958	(T/C)
CakSNP3964	*Kabuli*	*Ca_Kabuli_Chr03*	22062965	(C/T)
CakSNP3965	*Kabuli*	*Ca_Kabuli_Chr03*	22063826	(G/A)
CakSNP3966	*Kabuli*	*Ca_Kabuli_Chr03*	22063829	(G/A)
CakSNP3967	*Kabuli*	*Ca_Kabuli_Chr03*	22063835	(C/T)
CakSNP3968	*Kabuli*	*Ca_Kabuli_Chr03*	22073381	(C/T)
CakSNP3969	*Kabuli*	*Ca_Kabuli_Chr03*	22073447	(T/A)
CakSNP3970	*Kabuli*	*Ca_Kabuli_Chr03*	22075779	(C/G)
CakSNP3971	*Kabuli*	*Ca_Kabuli_Chr03*	22075990	(T/C)
CakSNP3972	*Kabuli*	*Ca_Kabuli_Chr03*	22169816	(T/C)
CakSNP3973	*Kabuli*	*Ca_Kabuli_Chr03*	22188124	(T/C)
CakSNP3974	*Kabuli*	*Ca_Kabuli_Chr03*	22188169	(T/A)
CakSNP3975	*Kabuli*	*Ca_Kabuli_Chr03*	22200927	(T/C)
CakSNP3976	*Kabuli*	*Ca_Kabuli_Chr03*	22201045	(T/A)

SNP IDs	Cultivars	Chromosomes/scaffolds	Physical positions (bp)	SNPs
CakSNP3977	*Kabuli*	*Ca_Kabuli_Chr03*	22202317	(C/T)
CakSNP3978	*Kabuli*	*Ca_Kabuli_Chr03*	22202819	(T/C)
CakSNP3979	*Kabuli*	*Ca_Kabuli_Chr03*	22203460	(A/G)
CakSNP3980	*Kabuli*	*Ca_Kabuli_Chr03*	22225186	(C/A)
CakSNP3981	*Kabuli*	*Ca_Kabuli_Chr03*	22225154	(C/G)
CakSNP3982	*Kabuli*	*Ca_Kabuli_Chr03*	22225129	(C/G)
CakSNP3983	*Kabuli*	*Ca_Kabuli_Chr03*	22233428	(G/A)
CakSNP3984	*Kabuli*	*Ca_Kabuli_Chr03*	22233409	(T/C)
CakSNP3985	*Kabuli*	*Ca_Kabuli_Chr03*	22235801	(G/A)
CakSNP3986	*Kabuli*	*Ca_Kabuli_Chr03*	22253842	(T/C)
CakSNP3987	*Kabuli*	*Ca_Kabuli_Chr03*	22331057	(C/T)
CakSNP3988	*Kabuli*	*Ca_Kabuli_Chr03*	22346294	(T/C)
CakSNP3989	*Kabuli*	*Ca_Kabuli_Chr03*	22404660	(G/T)
CakSNP3990	*Kabuli*	*Ca_Kabuli_Chr03*	22527529	(A/C)
CakSNP3991	*Kabuli*	*Ca_Kabuli_Chr03*	22539384	(G/A)
CakSNP3992	*Kabuli*	*Ca_Kabuli_Chr03*	22539702	(C/T)
CakSNP3993	*Kabuli*	*Ca_Kabuli_Chr03*	22539885	(C/T)
CakSNP3994	*Kabuli*	*Ca_Kabuli_Chr03*	22539952	(G/A)
CakSNP3995	*Kabuli*	*Ca_Kabuli_Chr03*	22636201	(T/C)
CakSNP3996	*Kabuli*	*Ca_Kabuli_Chr03*	22646897	(T/C)
CakSNP3997	*Kabuli*	*Ca_Kabuli_Chr03*	22659924	(G/A)
CakSNP3998	*Kabuli*	*Ca_Kabuli_Chr03*	22681658	(G/A)
CakSNP3999	*Kabuli*	*Ca_Kabuli_Chr03*	22719470	(T/C)
CakSNP4000	*Kabuli*	*Ca_Kabuli_Chr03*	22719487	(T/C)
CakSNP4001	*Kabuli*	*Ca_Kabuli_Chr03*	22719494	(C/T)
CakSNP4002	*Kabuli*	*Ca_Kabuli_Chr03*	22719519	(G/A)
CakSNP4003	*Kabuli*	*Ca_Kabuli_Chr03*	22727393	(G/A)
CakSNP4004	*Kabuli*	*Ca_Kabuli_Chr03*	22727439	(A/C)
CakSNP4005	*Kabuli*	*Ca_Kabuli_Chr03*	22765806	(G/A)
CakSNP4006	*Kabuli*	*Ca_Kabuli_Chr03*	22765862	(T/C)
CakSNP4007	*Kabuli*	*Ca_Kabuli_Chr03*	22765987	(G/T)
CakSNP4008	*Kabuli*	*Ca_Kabuli_Chr03*	22798281	(T/G)
CakSNP4009	*Kabuli*	*Ca_Kabuli_Chr03*	22812717	(A/T)
CakSNP4010	*Kabuli*	*Ca_Kabuli_Chr03*	22846051	(G/A)
CakSNP4011	*Kabuli*	*Ca_Kabuli_Chr03*	22847885	(G/T)
CakSNP4012	*Kabuli*	*Ca_Kabuli_Chr03*	22847984	(A/G)
CakSNP4013	*Kabuli*	*Ca_Kabuli_Chr03*	22990523	(C/T)
CakSNP4014	*Kabuli*	*Ca_Kabuli_Chr03*	23028855	(A/G)
CakSNP4015	*Kabuli*	*Ca_Kabuli_Chr03*	23033373	(A/C)
CakSNP4016	*Kabuli*	*Ca_Kabuli_Chr03*	23042249	(A/C)
CakSNP4017	*Kabuli*	*Ca_Kabuli_Chr03*	23053008	(C/G)

**SNP IDs**	**Cultivars**	**Chromosomes/scaffolds**	**Physical positions (bp)**	**SNPs**
CakSNP4018	*Kabuli*	*Ca_Kabuli_Chr03*	23053005	(G/C)
CakSNP4019	*Kabuli*	*Ca_Kabuli_Chr03*	23053066	(A/C)
CakSNP4020	*Kabuli*	*Ca_Kabuli_Chr03*	23095498	(C/T)
CakSNP4021	*Kabuli*	*Ca_Kabuli_Chr03*	23273057	(T/A)
CakSNP4022	*Kabuli*	*Ca_Kabuli_Chr03*	23273069	(C/G)
CakSNP4023	*Kabuli*	*Ca_Kabuli_Chr03*	23327648	(C/T)
CakSNP4024	*Kabuli*	*Ca_Kabuli_Chr03*	23327700	(G/C)
CakSNP4025	*Kabuli*	*Ca_Kabuli_Chr03*	23331530	(T/G)
CakSNP4026	*Kabuli*	*Ca_Kabuli_Chr03*	23335788	(A/T)
CakSNP4027	*Kabuli*	*Ca_Kabuli_Chr03*	23335789	(T/G)
CakSNP4028	*Kabuli*	*Ca_Kabuli_Chr03*	23340171	(A/G)
CakSNP4029	*Kabuli*	*Ca_Kabuli_Chr03*	23340134	(G/A)
CakSNP4030	*Kabuli*	*Ca_Kabuli_Chr03*	23340133	(T/C)
CakSNP4031	*Kabuli*	*Ca_Kabuli_Chr03*	23341384	(T/G)
CakSNP4032	*Kabuli*	*Ca_Kabuli_Chr03*	23342297	(T/C)
CakSNP4033	*Kabuli*	*Ca_Kabuli_Chr03*	23342305	(T/C)
CakSNP4034	*Kabuli*	*Ca_Kabuli_Chr03*	23342338	(G/T)
CakSNP4035	*Kabuli*	*Ca_Kabuli_Chr03*	23342347	(G/A)
CakSNP4036	*Kabuli*	*Ca_Kabuli_Chr03*	23342350	(A/G)
CakSNP4037	*Kabuli*	*Ca_Kabuli_Chr03*	23342351	(T/C)
CakSNP4038	*Kabuli*	*Ca_Kabuli_Chr03*	23360857	(C/T)
CakSNP4039	*Kabuli*	*Ca_Kabuli_Chr03*	23400864	(A/C)
CakSNP4040	*Kabuli*	*Ca_Kabuli_Chr03*	23455008	(A/G)
CakSNP4041	*Kabuli*	*Ca_Kabuli_Chr03*	23455128	(A/G)
CakSNP4042	*Kabuli*	*Ca_Kabuli_Chr03*	23461370	(C/A)
CakSNP4043	*Kabuli*	*Ca_Kabuli_Chr03*	23555586	(C/A)
CakSNP4044	*Kabuli*	*Ca_Kabuli_Chr03*	23593288	(G/A)
CakSNP4045	*Kabuli*	*Ca_Kabuli_Chr03*	23593294	(T/C)
CakSNP4046	*Kabuli*	*Ca_Kabuli_Chr03*	23593309	(T/C)
CakSNP4047	*Kabuli*	*Ca_Kabuli_Chr03*	23593269	(T/C)
CakSNP4048	*Kabuli*	*Ca_Kabuli_Chr03*	23714158	(T/A)
CakSNP4049	*Kabuli*	*Ca_Kabuli_Chr03*	23714129	(C/A)
CakSNP4050	*Kabuli*	*Ca_Kabuli_Chr03*	23745324	(C/T)
CakSNP4051	*Kabuli*	*Ca_Kabuli_Chr03*	23792975	(A/T)
CakSNP4052	*Kabuli*	*Ca_Kabuli_Chr03*	23833683	(G/A)
CakSNP4053	*Kabuli*	*Ca_Kabuli_Chr03*	23833680	(G/C)
CakSNP4054	*Kabuli*	*Ca_Kabuli_Chr03*	23833668	(G/A)
CakSNP4055	*Kabuli*	*Ca_Kabuli_Chr03*	23886582	(A/G)
CakSNP4056	*Kabuli*	*Ca_Kabuli_Chr03*	23918105	(T/C)
CakSNP4057	*Kabuli*	*Ca_Kabuli_Chr03*	23966110	(G/A)
CakSNP4058	*Kabuli*	*Ca_Kabuli_Chr03*	23997287	(A/T)

SNP IDs	Cultivars	Chromosomes/scaffolds	Physical positions (bp)	SNPs
CakSNP4059	*Kabuli*	*Ca_Kabuli_Chr03*	24000847	(G/T)
CakSNP4060	*Kabuli*	*Ca_Kabuli_Chr03*	24000818	(T/C)
CakSNP4061	*Kabuli*	*Ca_Kabuli_Chr03*	24024560	(T/C)
CakSNP4062	*Kabuli*	*Ca_Kabuli_Chr03*	24024513	(A/C)
CakSNP4063	*Kabuli*	*Ca_Kabuli_Chr03*	24025900	(G/C)
CakSNP4064	*Kabuli*	*Ca_Kabuli_Chr03*	24025924	(T/G)
CakSNP4065	*Kabuli*	*Ca_Kabuli_Chr03*	24026428	(C/T)
CakSNP4066	*Kabuli*	*Ca_Kabuli_Chr03*	24041806	(C/T)
CakSNP4067	*Kabuli*	*Ca_Kabuli_Chr03*	24095268	(T/C)
CakSNP4068	*Kabuli*	*Ca_Kabuli_Chr03*	24099191	(C/G)
CakSNP4069	*Kabuli*	*Ca_Kabuli_Chr03*	24127222	(C/T)
CakSNP4070	*Kabuli*	*Ca_Kabuli_Chr03*	24127201	(A/C)
CakSNP4071	*Kabuli*	*Ca_Kabuli_Chr03*	24162153	(C/T)
CakSNP4072	*Kabuli*	*Ca_Kabuli_Chr03*	24194574	(C/T)
CakSNP4073	*Kabuli*	*Ca_Kabuli_Chr03*	24247161	(G/C)
CakSNP4074	*Kabuli*	*Ca_Kabuli_Chr03*	24297842	(T/G)
CakSNP4075	*Kabuli*	*Ca_Kabuli_Chr03*	24297894	(C/A)
CakSNP4076	*Kabuli*	*Ca_Kabuli_Chr03*	24417477	(T/G)
CakSNP4077	*Kabuli*	*Ca_Kabuli_Chr03*	24417507	(A/T)
CakSNP4078	*Kabuli*	*Ca_Kabuli_Chr03*	24418063	(G/T)
CakSNP4079	*Kabuli*	*Ca_Kabuli_Chr03*	24418111	(A/G)
CakSNP4080	*Kabuli*	*Ca_Kabuli_Chr03*	24418130	(T/A)
CakSNP4081	*Kabuli*	*Ca_Kabuli_Chr03*	24459669	(G/A)
CakSNP4082	*Kabuli*	*Ca_Kabuli_Chr03*	24515489	(T/G)
CakSNP4083	*Kabuli*	*Ca_Kabuli_Chr03*	24538726	(A/C)
CakSNP4084	*Kabuli*	*Ca_Kabuli_Chr03*	24540199	(A/G)
CakSNP4085	*Kabuli*	*Ca_Kabuli_Chr03*	24540304	(T/C)
CakSNP4086	*Kabuli*	*Ca_Kabuli_Chr03*	24637834	(G/T)
CakSNP4087	*Kabuli*	*Ca_Kabuli_Chr03*	24637837	(A/C)
CakSNP4088	*Kabuli*	*Ca_Kabuli_Chr03*	24664582	(G/T)
CakSNP4089	*Kabuli*	*Ca_Kabuli_Chr03*	24664550	(T/G)
CakSNP4090	*Kabuli*	*Ca_Kabuli_Chr03*	24664651	(G/T)
CakSNP4091	*Kabuli*	*Ca_Kabuli_Chr03*	24666594	(T/C)
CakSNP4092	*Kabuli*	*Ca_Kabuli_Chr03*	24763730	(A/G)
CakSNP4093	*Kabuli*	*Ca_Kabuli_Chr03*	24933940	(T/C)
CakSNP4094	*Kabuli*	*Ca_Kabuli_Chr03*	24933901	(C/A)
CakSNP4095	*Kabuli*	*Ca_Kabuli_Chr03*	24933899	(G/T)
CakSNP4096	*Kabuli*	*Ca_Kabuli_Chr03*	24998316	(C/G)
CakSNP4097	*Kabuli*	*Ca_Kabuli_Chr03*	24998300	(C/T)
CakSNP4098	*Kabuli*	*Ca_Kabuli_Chr03*	25037746	(G/A)
CakSNP4099	*Kabuli*	*Ca_Kabuli_Chr03*	25046423	(C/G)

**SNP IDs**	**Cultivars**	**Chromosomes/scaffolds**	**Physical positions (bp)**	**SNPs**
CakSNP4100	*Kabuli*	*Ca_Kabuli_Chr03*	25046421	(T/C)
CakSNP4101	*Kabuli*	*Ca_Kabuli_Chr03*	25055049	(C/T)
CakSNP4102	*Kabuli*	*Ca_Kabuli_Chr03*	25062250	(C/A)
CakSNP4103	*Kabuli*	*Ca_Kabuli_Chr03*	25110037	(T/G)
CakSNP4104	*Kabuli*	*Ca_Kabuli_Chr03*	25137192	(T/A)
CakSNP4105	*Kabuli*	*Ca_Kabuli_Chr03*	25147096	(G/A)
CakSNP4106	*Kabuli*	*Ca_Kabuli_Chr03*	25205548	(C/A)
CakSNP4107	*Kabuli*	*Ca_Kabuli_Chr03*	25241115	(G/A)
CakSNP4108	*Kabuli*	*Ca_Kabuli_Chr03*	25244194	(A/C)
CakSNP4109	*Kabuli*	*Ca_Kabuli_Chr03*	25270592	(C/G)
CakSNP4110	*Kabuli*	*Ca_Kabuli_Chr03*	25399911	(C/G)
CakSNP4111	*Kabuli*	*Ca_Kabuli_Chr03*	25457474	(T/A)
CakSNP4112	*Kabuli*	*Ca_Kabuli_Chr03*	25457494	(A/T)
CakSNP4113	*Kabuli*	*Ca_Kabuli_Chr03*	25457514	(C/A)
CakSNP4114	*Kabuli*	*Ca_Kabuli_Chr03*	25462227	(A/C)
CakSNP4115	*Kabuli*	*Ca_Kabuli_Chr03*	25504708	(A/G)
CakSNP4116	*Kabuli*	*Ca_Kabuli_Chr03*	25505438	(C/T)
CakSNP4117	*Kabuli*	*Ca_Kabuli_Chr03*	25505420	(G/T)
CakSNP4118	*Kabuli*	*Ca_Kabuli_Chr03*	25534044	(C/T)
CakSNP4119	*Kabuli*	*Ca_Kabuli_Chr03*	25538867	(T/C)
CakSNP4120	*Kabuli*	*Ca_Kabuli_Chr03*	25580072	(A/T)
CakSNP4121	*Kabuli*	*Ca_Kabuli_Chr03*	25614515	(T/G)
CakSNP4122	*Kabuli*	*Ca_Kabuli_Chr03*	25641434	(C/A)
CakSNP4123	*Kabuli*	*Ca_Kabuli_Chr03*	25641433	(G/A)
CakSNP4124	*Kabuli*	*Ca_Kabuli_Chr03*	25688341	(G/A)
CakSNP4125	*Kabuli*	*Ca_Kabuli_Chr03*	25702238	(G/A)
CakSNP4126	*Kabuli*	*Ca_Kabuli_Chr03*	25718829	(C/A)
CakSNP4127	*Kabuli*	*Ca_Kabuli_Chr03*	25718827	(T/A)
CakSNP4128	*Kabuli*	*Ca_Kabuli_Chr03*	25757693	(C/T)
CakSNP4129	*Kabuli*	*Ca_Kabuli_Chr03*	25987920	(T/G)
CakSNP4130	*Kabuli*	*Ca_Kabuli_Chr03*	26119425	(T/G)
CakSNP4131	*Kabuli*	*Ca_Kabuli_Chr03*	26119461	(C/T)
CakSNP4132	*Kabuli*	*Ca_Kabuli_Chr03*	26163449	(G/A)
CakSNP4133	*Kabuli*	*Ca_Kabuli_Chr03*	26178592	(G/A)
CakSNP4134	*Kabuli*	*Ca_Kabuli_Chr03*	26198368	(G/A)
CakSNP4135	*Kabuli*	*Ca_Kabuli_Chr03*	26198372	(G/A)
CakSNP4136	*Kabuli*	*Ca_Kabuli_Chr03*	26293846	(A/G)
CakSNP4137	*Kabuli*	*Ca_Kabuli_Chr03*	26335889	(T/A)
CakSNP4138	*Kabuli*	*Ca_Kabuli_Chr03*	26339286	(T/C)
CakSNP4139	*Kabuli*	*Ca_Kabuli_Chr03*	26339449	(G/T)
CakSNP4140	*Kabuli*	*Ca_Kabuli_Chr03*	26562004	(A/G)

SNP IDs	Cultivars	Chromosomes/scaffolds	Physical positions (bp)	SNPs
CakSNP4141	*Kabuli*	*Ca_Kabuli_Chr03*	26573902	(A/G)
CakSNP4142	*Kabuli*	*Ca_Kabuli_Chr03*	26574056	(C/T)
CakSNP4143	*Kabuli*	*Ca_Kabuli_Chr03*	26574068	(T/G)
CakSNP4144	*Kabuli*	*Ca_Kabuli_Chr03*	26574831	(A/G)
CakSNP4145	*Kabuli*	*Ca_Kabuli_Chr03*	26589678	(T/A)
CakSNP4146	*Kabuli*	*Ca_Kabuli_Chr03*	26627150	(T/G)
CakSNP4147	*Kabuli*	*Ca_Kabuli_Chr03*	26636553	(A/G)
CakSNP4148	*Kabuli*	*Ca_Kabuli_Chr03*	26646152	(C/T)
CakSNP4149	*Kabuli*	*Ca_Kabuli_Chr03*	26678343	(T/C)
CakSNP4150	*Kabuli*	*Ca_Kabuli_Chr03*	26678433	(T/G)
CakSNP4151	*Kabuli*	*Ca_Kabuli_Chr03*	26726897	(C/A)
CakSNP4152	*Kabuli*	*Ca_Kabuli_Chr03*	26758452	(C/A)
CakSNP4153	*Kabuli*	*Ca_Kabuli_Chr03*	26848870	(G/C)
CakSNP4154	*Kabuli*	*Ca_Kabuli_Chr03*	26849288	(T/A)
CakSNP4155	*Kabuli*	*Ca_Kabuli_Chr03*	26878028	(A/T)
CakSNP4156	*Kabuli*	*Ca_Kabuli_Chr03*	26900271	(T/A)
CakSNP4157	*Kabuli*	*Ca_Kabuli_Chr03*	26911443	(A/T)
CakSNP4158	*Kabuli*	*Ca_Kabuli_Chr03*	26911514	(A/G)
CakSNP4159	*Kabuli*	*Ca_Kabuli_Chr03*	26911723	(T/A)
CakSNP4160	*Kabuli*	*Ca_Kabuli_Chr03*	26911792	(A/G)
CakSNP4161	*Kabuli*	*Ca_Kabuli_Chr03*	26976249	(A/G)
CakSNP4162	*Kabuli*	*Ca_Kabuli_Chr03*	26976323	(G/A)
CakSNP4163	*Kabuli*	*Ca_Kabuli_Chr03*	27024215	(T/C)
CakSNP4164	*Kabuli*	*Ca_Kabuli_Chr03*	27047840	(A/G)
CakSNP4165	*Kabuli*	*Ca_Kabuli_Chr03*	27047847	(A/C)
CakSNP4166	*Kabuli*	*Ca_Kabuli_Chr03*	27063150	(A/C)
CakSNP4167	*Kabuli*	*Ca_Kabuli_Chr03*	27074842	(C/A)
CakSNP4168	*Kabuli*	*Ca_Kabuli_Chr03*	27074896	(A/T)
CakSNP4169	*Kabuli*	*Ca_Kabuli_Chr03*	27074931	(A/T)
CakSNP4170	*Kabuli*	*Ca_Kabuli_Chr03*	27074930	(A/C)
CakSNP4171	*Kabuli*	*Ca_Kabuli_Chr03*	27076716	(T/C)
CakSNP4172	*Kabuli*	*Ca_Kabuli_Chr03*	27076735	(T/G)
CakSNP4173	*Kabuli*	*Ca_Kabuli_Chr03*	27076744	(C/A)
CakSNP4174	*Kabuli*	*Ca_Kabuli_Chr03*	27105637	(C/T)
CakSNP4175	*Kabuli*	*Ca_Kabuli_Chr03*	27157840	(T/A)
CakSNP4176	*Kabuli*	*Ca_Kabuli_Chr03*	27157848	(C/T)
CakSNP4177	*Kabuli*	*Ca_Kabuli_Chr03*	27161813	(T/G)
CakSNP4178	*Kabuli*	*Ca_Kabuli_Chr03*	27161801	(A/G)
CakSNP4179	*Kabuli*	*Ca_Kabuli_Chr03*	27194033	(C/T)
CakSNP4180	*Kabuli*	*Ca_Kabuli_Chr03*	27194069	(T/C)
CakSNP4181	*Kabuli*	*Ca_Kabuli_Chr03*	27338521	(T/C)

**SNP IDs**	**Cultivars**	**Chromosomes/scaffolds**	**Physical positions (bp)**	**SNPs**
CakSNP4182	*Kabuli*	*Ca_Kabuli_Chr03*	27342919	(G/A)
CakSNP4183	*Kabuli*	*Ca_Kabuli_Chr03*	27391199	(G/A)
CakSNP4184	*Kabuli*	*Ca_Kabuli_Chr03*	27391354	(G/A)
CakSNP4185	*Kabuli*	*Ca_Kabuli_Chr03*	27391321	(A/G)
CakSNP4186	*Kabuli*	*Ca_Kabuli_Chr03*	27417561	(A/T)
CakSNP4187	*Kabuli*	*Ca_Kabuli_Chr03*	27420276	(T/C)
CakSNP4188	*Kabuli*	*Ca_Kabuli_Chr03*	27420330	(C/T)
CakSNP4189	*Kabuli*	*Ca_Kabuli_Chr03*	27435210	(T/C)
CakSNP4190	*Kabuli*	*Ca_Kabuli_Chr03*	27435209	(G/C)
CakSNP4191	*Kabuli*	*Ca_Kabuli_Chr03*	27435198	(T/A)
CakSNP4192	*Kabuli*	*Ca_Kabuli_Chr03*	27447685	(C/A)
CakSNP4193	*Kabuli*	*Ca_Kabuli_Chr03*	27470443	(A/C)
CakSNP4194	*Kabuli*	*Ca_Kabuli_Chr03*	27470439	(G/T)
CakSNP4195	*Kabuli*	*Ca_Kabuli_Chr03*	27597331	(A/C)
CakSNP4196	*Kabuli*	*Ca_Kabuli_Chr03*	27597409	(A/G)
CakSNP4197	*Kabuli*	*Ca_Kabuli_Chr03*	27632100	(G/A)
CakSNP4198	*Kabuli*	*Ca_Kabuli_Chr03*	27632169	(T/C)
CakSNP4199	*Kabuli*	*Ca_Kabuli_Chr03*	27654798	(A/C)
CakSNP4200	*Kabuli*	*Ca_Kabuli_Chr03*	27673727	(C/A)
CakSNP4201	*Kabuli*	*Ca_Kabuli_Chr03*	27681551	(T/C)
CakSNP4202	*Kabuli*	*Ca_Kabuli_Chr03*	27716396	(C/T)
CakSNP4203	*Kabuli*	*Ca_Kabuli_Chr03*	27763236	(C/T)
CakSNP4204	*Kabuli*	*Ca_Kabuli_Chr03*	27812571	(A/T)
CakSNP4205	*Kabuli*	*Ca_Kabuli_Chr03*	27876405	(A/G)
CakSNP4206	*Kabuli*	*Ca_Kabuli_Chr03*	27877442	(A/T)
CakSNP4207	*Kabuli*	*Ca_Kabuli_Chr03*	27877479	(C/T)
CakSNP4208	*Kabuli*	*Ca_Kabuli_Chr03*	27877480	(G/A)
CakSNP4209	*Kabuli*	*Ca_Kabuli_Chr03*	27877510	(G/A)
CakSNP4210	*Kabuli*	*Ca_Kabuli_Chr03*	27888817	(G/C)
CakSNP4211	*Kabuli*	*Ca_Kabuli_Chr03*	27889107	(A/G)
CakSNP4212	*Kabuli*	*Ca_Kabuli_Chr03*	27890849	(C/T)
CakSNP4213	*Kabuli*	*Ca_Kabuli_Chr03*	27905944	(T/C)
CakSNP4214	*Kabuli*	*Ca_Kabuli_Chr03*	27905989	(G/A)
CakSNP4215	*Kabuli*	*Ca_Kabuli_Chr03*	27905995	(C/T)
CakSNP4216	*Kabuli*	*Ca_Kabuli_Chr03*	27906069	(C/A)
CakSNP4217	*Kabuli*	*Ca_Kabuli_Chr03*	27914409	(C/T)
CakSNP4218	*Kabuli*	*Ca_Kabuli_Chr03*	27968979	(T/G)
CakSNP4219	*Kabuli*	*Ca_Kabuli_Chr03*	27969014	(T/C)
CakSNP4220	*Kabuli*	*Ca_Kabuli_Chr03*	27969005	(A/G)
CakSNP4221	*Kabuli*	*Ca_Kabuli_Chr03*	27969207	(C/T)
CakSNP4222	*Kabuli*	*Ca_Kabuli_Chr03*	28044035	(A/T)

**SNP IDs**	**Cultivars**	**Chromosomes/scaffolds**	**Physical positions (bp)**	**SNPs**
CakSNP4223	*Kabuli*	*Ca_Kabuli_Chr03*	28176380	(A/C)
CakSNP4224	*Kabuli*	*Ca_Kabuli_Chr03*	28176333	(C/A)
CakSNP4225	*Kabuli*	*Ca_Kabuli_Chr03*	28277991	(C/G)
CakSNP4226	*Kabuli*	*Ca_Kabuli_Chr03*	28278018	(C/G)
CakSNP4227	*Kabuli*	*Ca_Kabuli_Chr03*	28374024	(C/T)
CakSNP4228	*Kabuli*	*Ca_Kabuli_Chr03*	28375082	(G/A)
CakSNP4229	*Kabuli*	*Ca_Kabuli_Chr03*	28409001	(G/A)
CakSNP4230	*Kabuli*	*Ca_Kabuli_Chr03*	28420948	(C/T)
CakSNP4231	*Kabuli*	*Ca_Kabuli_Chr03*	28478044	(C/T)
CakSNP4232	*Kabuli*	*Ca_Kabuli_Chr03*	28478539	(C/G)
CakSNP4233	*Kabuli*	*Ca_Kabuli_Chr03*	28478569	(C/A)
CakSNP4234	*Kabuli*	*Ca_Kabuli_Chr03*	28602055	(C/A)
CakSNP4235	*Kabuli*	*Ca_Kabuli_Chr03*	28603892	(T/C)
CakSNP4236	*Kabuli*	*Ca_Kabuli_Chr03*	28613067	(A/G)
CakSNP4237	*Kabuli*	*Ca_Kabuli_Chr03*	28614784	(A/G)
CakSNP4238	*Kabuli*	*Ca_Kabuli_Chr03*	28614919	(A/T)
CakSNP4239	*Kabuli*	*Ca_Kabuli_Chr03*	28680858	(C/T)
CakSNP4240	*Kabuli*	*Ca_Kabuli_Chr03*	28706106	(T/A)
CakSNP4241	*Kabuli*	*Ca_Kabuli_Chr03*	28706292	(G/C)
CakSNP4242	*Kabuli*	*Ca_Kabuli_Chr03*	28712993	(T/C)
CakSNP4243	*Kabuli*	*Ca_Kabuli_Chr03*	28716003	(C/T)
CakSNP4244	*Kabuli*	*Ca_Kabuli_Chr03*	28716027	(A/T)
CakSNP4245	*Kabuli*	*Ca_Kabuli_Chr03*	28716028	(A/T)
CakSNP4246	*Kabuli*	*Ca_Kabuli_Chr03*	28716029	(A/G)
CakSNP4247	*Kabuli*	*Ca_Kabuli_Chr03*	28716038	(T/G)
CakSNP4248	*Kabuli*	*Ca_Kabuli_Chr03*	28716047	(G/A)
CakSNP4249	*Kabuli*	*Ca_Kabuli_Chr03*	28755111	(A/G)
CakSNP4250	*Kabuli*	*Ca_Kabuli_Chr03*	28755149	(G/A)
CakSNP4251	*Kabuli*	*Ca_Kabuli_Chr03*	28845958	(A/C)
CakSNP4252	*Kabuli*	*Ca_Kabuli_Chr03*	28926094	(T/A)
CakSNP4253	*Kabuli*	*Ca_Kabuli_Chr03*	28926290	(T/C)
CakSNP4254	*Kabuli*	*Ca_Kabuli_Chr03*	28934196	(C/T)
CakSNP4255	*Kabuli*	*Ca_Kabuli_Chr03*	28943956	(C/T)
CakSNP4256	*Kabuli*	*Ca_Kabuli_Chr03*	28943989	(G/T)
CakSNP4257	*Kabuli*	*Ca_Kabuli_Chr03*	29056451	(T/C)
CakSNP4258	*Kabuli*	*Ca_Kabuli_Chr03*	29056528	(G/T)
CakSNP4259	*Kabuli*	*Ca_Kabuli_Chr03*	29056768	(T/C)
CakSNP4260	*Kabuli*	*Ca_Kabuli_Chr03*	29107776	(A/G)
CakSNP4261	*Kabuli*	*Ca_Kabuli_Chr03*	29107947	(C/A)
CakSNP4262	*Kabuli*	*Ca_Kabuli_Chr03*	29155741	(C/G)
CakSNP4263	*Kabuli*	*Ca_Kabuli_Chr03*	29177885	(A/C)

**SNP IDs**	**Cultivars**	**Chromosomes/scaffolds**	**Physical positions (bp)**	**SNPs**
CakSNP4264	*Kabuli*	*Ca_Kabuli_Chr03*	29188300	(T/G)
CakSNP4265	*Kabuli*	*Ca_Kabuli_Chr03*	29188308	(G/A)
CakSNP4266	*Kabuli*	*Ca_Kabuli_Chr03*	29207338	(G/A)
CakSNP4267	*Kabuli*	*Ca_Kabuli_Chr03*	29293770	(G/T)
CakSNP4268	*Kabuli*	*Ca_Kabuli_Chr03*	29293783	(G/A)
CakSNP4269	*Kabuli*	*Ca_Kabuli_Chr03*	29293840	(C/A)
CakSNP4270	*Kabuli*	*Ca_Kabuli_Chr03*	29302251	(C/T)
CakSNP4271	*Kabuli*	*Ca_Kabuli_Chr03*	29302448	(A/C)
CakSNP4272	*Kabuli*	*Ca_Kabuli_Chr03*	29303144	(A/C)
CakSNP4273	*Kabuli*	*Ca_Kabuli_Chr03*	29304116	(A/G)
CakSNP4274	*Kabuli*	*Ca_Kabuli_Chr03*	29304161	(C/T)
CakSNP4275	*Kabuli*	*Ca_Kabuli_Chr03*	29311672	(C/A)
CakSNP4276	*Kabuli*	*Ca_Kabuli_Chr03*	29348267	(C/T)
CakSNP4277	*Kabuli*	*Ca_Kabuli_Chr03*	29355268	(T/A)
CakSNP4278	*Kabuli*	*Ca_Kabuli_Chr03*	29376025	(A/G)
CakSNP4279	*Kabuli*	*Ca_Kabuli_Chr03*	29376008	(G/A)
CakSNP4280	*Kabuli*	*Ca_Kabuli_Chr03*	29439505	(A/G)
CakSNP4281	*Kabuli*	*Ca_Kabuli_Chr03*	29485590	(T/C)
CakSNP4282	*Kabuli*	*Ca_Kabuli_Chr03*	29485566	(T/A)
CakSNP4283	*Kabuli*	*Ca_Kabuli_Chr03*	29508831	(C/T)
CakSNP4284	*Kabuli*	*Ca_Kabuli_Chr03*	29509323	(G/A)
CakSNP4285	*Kabuli*	*Ca_Kabuli_Chr03*	29509310	(G/T)
CakSNP4286	*Kabuli*	*Ca_Kabuli_Chr03*	29547528	(G/A)
CakSNP4287	*Kabuli*	*Ca_Kabuli_Chr03*	29588940	(C/T)
CakSNP4288	*Kabuli*	*Ca_Kabuli_Chr03*	29588934	(T/C)
CakSNP4289	*Kabuli*	*Ca_Kabuli_Chr03*	29588930	(T/C)
CakSNP4290	*Kabuli*	*Ca_Kabuli_Chr03*	29591575	(T/G)
CakSNP4291	*Kabuli*	*Ca_Kabuli_Chr03*	29591613	(G/T)
CakSNP4292	*Kabuli*	*Ca_Kabuli_Chr03*	29603720	(T/C)
CakSNP4293	*Kabuli*	*Ca_Kabuli_Chr03*	29640075	(A/C)
CakSNP4294	*Kabuli*	*Ca_Kabuli_Chr03*	29640071	(T/C)
CakSNP4295	*Kabuli*	*Ca_Kabuli_Chr03*	29666633	(C/T)
CakSNP4296	*Kabuli*	*Ca_Kabuli_Chr03*	29690732	(C/A)
CakSNP4297	*Kabuli*	*Ca_Kabuli_Chr03*	29698166	(G/T)
CakSNP4298	*Kabuli*	*Ca_Kabuli_Chr03*	29698245	(T/A)
CakSNP4299	*Kabuli*	*Ca_Kabuli_Chr03*	29698289	(G/A)
CakSNP4300	*Kabuli*	*Ca_Kabuli_Chr03*	29698273	(A/C)
CakSNP4301	*Kabuli*	*Ca_Kabuli_Chr03*	29730019	(G/T)
CakSNP4302	*Kabuli*	*Ca_Kabuli_Chr03*	29744878	(T/G)
CakSNP4303	*Kabuli*	*Ca_Kabuli_Chr03*	29744890	(A/C)
CakSNP4304	*Kabuli*	*Ca_Kabuli_Chr03*	29803866	(C/T)

SNP IDs	Cultivars	Chromosomes/scaffolds	Physical positions (bp)	SNPs
CakSNP4305	*Kabuli*	*Ca_Kabuli_Chr03*	29803962	(A/G)
CakSNP4306	*Kabuli*	*Ca_Kabuli_Chr03*	29807091	(C/G)
CakSNP4307	*Kabuli*	*Ca_Kabuli_Chr03*	29807248	(A/G)
CakSNP4308	*Kabuli*	*Ca_Kabuli_Chr03*	29836337	(C/A)
CakSNP4309	*Kabuli*	*Ca_Kabuli_Chr03*	29840029	(C/G)
CakSNP4310	*Kabuli*	*Ca_Kabuli_Chr03*	29840028	(T/C)
CakSNP4311	*Kabuli*	*Ca_Kabuli_Chr03*	29840022	(C/A)
CakSNP4312	*Kabuli*	*Ca_Kabuli_Chr03*	29840012	(A/T)
CakSNP4313	*Kabuli*	*Ca_Kabuli_Chr03*	29844509	(T/A)
CakSNP4314	*Kabuli*	*Ca_Kabuli_Chr03*	29869190	(G/T)
CakSNP4315	*Kabuli*	*Ca_Kabuli_Chr03*	29869199	(T/C)
CakSNP4316	*Kabuli*	*Ca_Kabuli_Chr03*	29869428	(G/T)
CakSNP4317	*Kabuli*	*Ca_Kabuli_Chr03*	29869381	(G/A)
CakSNP4318	*Kabuli*	*Ca_Kabuli_Chr03*	29869380	(G/A)
CakSNP4319	*Kabuli*	*Ca_Kabuli_Chr03*	29872643	(A/G)
CakSNP4320	*Kabuli*	*Ca_Kabuli_Chr03*	29872682	(G/A)
CakSNP4321	*Kabuli*	*Ca_Kabuli_Chr03*	29898396	(A/G)
CakSNP4322	*Kabuli*	*Ca_Kabuli_Chr03*	29912488	(A/G)
CakSNP4323	*Kabuli*	*Ca_Kabuli_Chr03*	29915510	(G/A)
CakSNP4324	*Kabuli*	*Ca_Kabuli_Chr03*	29932378	(C/G)
CakSNP4325	*Kabuli*	*Ca_Kabuli_Chr03*	29932504	(T/A)
CakSNP4326	*Kabuli*	*Ca_Kabuli_Chr03*	29932502	(G/C)
CakSNP4327	*Kabuli*	*Ca_Kabuli_Chr03*	29954498	(G/A)
CakSNP4328	*Kabuli*	*Ca_Kabuli_Chr03*	29954533	(A/G)
CakSNP4329	*Kabuli*	*Ca_Kabuli_Chr03*	29954648	(A/T)
CakSNP4330	*Kabuli*	*Ca_Kabuli_Chr03*	29954612	(T/G)
CakSNP4331	*Kabuli*	*Ca_Kabuli_Chr03*	29974532	(A/G)
CakSNP4332	*Kabuli*	*Ca_Kabuli_Chr03*	29974535	(A/G)
CakSNP4333	*Kabuli*	*Ca_Kabuli_Chr03*	29974595	(C/T)
CakSNP4334	*Kabuli*	*Ca_Kabuli_Chr03*	29982638	(A/G)
CakSNP4335	*Kabuli*	*Ca_Kabuli_Chr03*	29982644	(T/C)
CakSNP4336	*Kabuli*	*Ca_Kabuli_Chr03*	29982718	(C/A)
CakSNP4337	*Kabuli*	*Ca_Kabuli_Chr03*	30004352	(T/A)
CakSNP4338	*Kabuli*	*Ca_Kabuli_Chr03*	30004311	(T/G)
CakSNP4339	*Kabuli*	*Ca_Kabuli_Chr03*	30004295	(A/G)
CakSNP4340	*Kabuli*	*Ca_Kabuli_Chr03*	30004279	(G/T)
CakSNP4341	*Kabuli*	*Ca_Kabuli_Chr03*	30009698	(C/T)
CakSNP4342	*Kabuli*	*Ca_Kabuli_Chr03*	30009751	(A/C)
CakSNP4343	*Kabuli*	*Ca_Kabuli_Chr03*	30011584	(T/C)
CakSNP4344	*Kabuli*	*Ca_Kabuli_Chr03*	30011669	(A/G)
CakSNP4345	*Kabuli*	*Ca_Kabuli_Chr03*	30011642	(T/A)

SNP IDs	Cultivars	Chromosomes/scaffolds	Physical positions (bp)	SNPs
CakSNP4346	*Kabuli*	*Ca_Kabuli_Chr03*	30011621	(C/T)
CakSNP4347	*Kabuli*	*Ca_Kabuli_Chr03*	30029874	(C/T)
CakSNP4348	*Kabuli*	*Ca_Kabuli_Chr03*	30029858	(T/A)
CakSNP4349	*Kabuli*	*Ca_Kabuli_Chr03*	30029842	(G/C)
CakSNP4350	*Kabuli*	*Ca_Kabuli_Chr03*	30029826	(T/C)
CakSNP4351	*Kabuli*	*Ca_Kabuli_Chr03*	30029817	(T/G)
CakSNP4352	*Kabuli*	*Ca_Kabuli_Chr03*	30084154	(T/C)
CakSNP4353	*Kabuli*	*Ca_Kabuli_Chr03*	30101412	(T/C)
CakSNP4354	*Kabuli*	*Ca_Kabuli_Chr03*	30101476	(A/C)
CakSNP4355	*Kabuli*	*Ca_Kabuli_Chr03*	30111154	(A/C)
CakSNP4356	*Kabuli*	*Ca_Kabuli_Chr03*	30111146	(G/C)
CakSNP4357	*Kabuli*	*Ca_Kabuli_Chr03*	30148907	(T/C)
CakSNP4358	*Kabuli*	*Ca_Kabuli_Chr03*	30194810	(C/T)
CakSNP4359	*Kabuli*	*Ca_Kabuli_Chr03*	30206121	(T/A)
CakSNP4360	*Kabuli*	*Ca_Kabuli_Chr03*	30206266	(G/A)
CakSNP4361	*Kabuli*	*Ca_Kabuli_Chr03*	30206281	(A/T)
CakSNP4362	*Kabuli*	*Ca_Kabuli_Chr03*	30206436	(G/A)
CakSNP4363	*Kabuli*	*Ca_Kabuli_Chr03*	30267761	(T/G)
CakSNP4364	*Kabuli*	*Ca_Kabuli_Chr03*	30271022	(G/T)
CakSNP4365	*Kabuli*	*Ca_Kabuli_Chr03*	30271028	(G/A)
CakSNP4366	*Kabuli*	*Ca_Kabuli_Chr03*	30271034	(G/A)
CakSNP4367	*Kabuli*	*Ca_Kabuli_Chr03*	30309625	(G/C)
CakSNP4368	*Kabuli*	*Ca_Kabuli_Chr03*	30309624	(A/T)
CakSNP4369	*Kabuli*	*Ca_Kabuli_Chr03*	30321177	(C/G)
CakSNP4370	*Kabuli*	*Ca_Kabuli_Chr03*	30321245	(C/T)
CakSNP4371	*Kabuli*	*Ca_Kabuli_Chr03*	30321272	(C/A)
CakSNP4372	*Kabuli*	*Ca_Kabuli_Chr03*	30323585	(G/A)
CakSNP4373	*Kabuli*	*Ca_Kabuli_Chr03*	30376319	(G/A)
CakSNP4374	*Kabuli*	*Ca_Kabuli_Chr03*	30385144	(G/A)
CakSNP4375	*Kabuli*	*Ca_Kabuli_Chr03*	30385153	(T/A)
CakSNP4376	*Kabuli*	*Ca_Kabuli_Chr03*	30390663	(C/T)
CakSNP4377	*Kabuli*	*Ca_Kabuli_Chr03*	30397262	(A/T)
CakSNP4378	*Kabuli*	*Ca_Kabuli_Chr03*	30425496	(G/T)
CakSNP4379	*Kabuli*	*Ca_Kabuli_Chr03*	30510392	(T/A)
CakSNP4380	*Kabuli*	*Ca_Kabuli_Chr03*	30510333	(C/T)
CakSNP4381	*Kabuli*	*Ca_Kabuli_Chr03*	30522442	(A/C)
CakSNP4382	*Kabuli*	*Ca_Kabuli_Chr03*	30522485	(C/T)
CakSNP4383	*Kabuli*	*Ca_Kabuli_Chr03*	30522498	(T/C)
CakSNP4384	*Kabuli*	*Ca_Kabuli_Chr03*	30650598	(C/A)
CakSNP4385	*Kabuli*	*Ca_Kabuli_Chr03*	30650634	(T/C)
CakSNP4386	*Kabuli*	*Ca_Kabuli_Chr03*	30650659	(C/T)

SNP IDs	Cultivars	Chromosomes/scaffolds	Physical positions (bp)	SNPs
CakSNP4387	*Kabuli*	*Ca_Kabuli_Chr03*	30659438	(T/A)
CakSNP4388	*Kabuli*	*Ca_Kabuli_Chr03*	30675468	(T/G)
CakSNP4389	*Kabuli*	*Ca_Kabuli_Chr03*	30681031	(A/G)
CakSNP4390	*Kabuli*	*Ca_Kabuli_Chr03*	30681032	(A/G)
CakSNP4391	*Kabuli*	*Ca_Kabuli_Chr03*	30688336	(T/G)
CakSNP4392	*Kabuli*	*Ca_Kabuli_Chr03*	30688354	(G/A)
CakSNP4393	*Kabuli*	*Ca_Kabuli_Chr03*	30688369	(T/C)
CakSNP4394	*Kabuli*	*Ca_Kabuli_Chr03*	30688418	(A/C)
CakSNP4395	*Kabuli*	*Ca_Kabuli_Chr03*	30688511	(G/C)
CakSNP4396	*Kabuli*	*Ca_Kabuli_Chr03*	30688485	(C/A)
CakSNP4397	*Kabuli*	*Ca_Kabuli_Chr03*	30688484	(A/C)
CakSNP4398	*Kabuli*	*Ca_Kabuli_Chr03*	30688454	(A/G)
CakSNP4399	*Kabuli*	*Ca_Kabuli_Chr03*	30688451	(T/C)
CakSNP4400	*Kabuli*	*Ca_Kabuli_Chr03*	30718334	(G/T)
CakSNP4401	*Kabuli*	*Ca_Kabuli_Chr03*	30751893	(A/C)
CakSNP4402	*Kabuli*	*Ca_Kabuli_Chr03*	30751949	(A/T)
CakSNP4403	*Kabuli*	*Ca_Kabuli_Chr03*	30791169	(A/T)
CakSNP4404	*Kabuli*	*Ca_Kabuli_Chr03*	30791229	(G/A)
CakSNP4405	*Kabuli*	*Ca_Kabuli_Chr03*	30791693	(C/T)
CakSNP4406	*Kabuli*	*Ca_Kabuli_Chr03*	30794040	(C/A)
CakSNP4407	*Kabuli*	*Ca_Kabuli_Chr03*	30794046	(A/C)
CakSNP4408	*Kabuli*	*Ca_Kabuli_Chr03*	30837147	(A/G)
CakSNP4409	*Kabuli*	*Ca_Kabuli_Chr03*	30837284	(C/T)
CakSNP4410	*Kabuli*	*Ca_Kabuli_Chr03*	30837265	(T/C)
CakSNP4411	*Kabuli*	*Ca_Kabuli_Chr03*	30880452	(T/C)
CakSNP4412	*Kabuli*	*Ca_Kabuli_Chr03*	30880610	(A/G)
CakSNP4413	*Kabuli*	*Ca_Kabuli_Chr03*	31020357	(C/A)
CakSNP4414	*Kabuli*	*Ca_Kabuli_Chr03*	31020358	(C/G)
CakSNP4415	*Kabuli*	*Ca_Kabuli_Chr03*	31073396	(T/C)
CakSNP4416	*Kabuli*	*Ca_Kabuli_Chr03*	31073425	(G/C)
CakSNP4417	*Kabuli*	*Ca_Kabuli_Chr03*	31073451	(A/G)
CakSNP4418	*Kabuli*	*Ca_Kabuli_Chr03*	31091875	(T/G)
CakSNP4419	*Kabuli*	*Ca_Kabuli_Chr03*	31267639	(T/A)
CakSNP4420	*Kabuli*	*Ca_Kabuli_Chr03*	31267675	(A/G)
CakSNP4421	*Kabuli*	*Ca_Kabuli_Chr03*	31267681	(G/A)
CakSNP4422	*Kabuli*	*Ca_Kabuli_Chr03*	31267828	(A/G)
CakSNP4423	*Kabuli*	*Ca_Kabuli_Chr03*	31267927	(T/G)
CakSNP4424	*Kabuli*	*Ca_Kabuli_Chr03*	31268143	(A/G)
CakSNP4425	*Kabuli*	*Ca_Kabuli_Chr03*	31279502	(C/T)
CakSNP4426	*Kabuli*	*Ca_Kabuli_Chr03*	31279541	(A/G)
CakSNP4427	*Kabuli*	*Ca_Kabuli_Chr03*	31279639	(C/T)

SNP IDs	Cultivars	Chromosomes/scaffolds	Physical positions (bp)	SNPs
CakSNP4428	*Kabuli*	*Ca_Kabuli_Chr03*	31279629	(C/G)
CakSNP4429	*Kabuli*	*Ca_Kabuli_Chr03*	31279628	(T/C)
CakSNP4430	*Kabuli*	*Ca_Kabuli_Chr03*	31279616	(T/A)
CakSNP4431	*Kabuli*	*Ca_Kabuli_Chr03*	31279606	(G/A)
CakSNP4432	*Kabuli*	*Ca_Kabuli_Chr03*	31279605	(G/A)
CakSNP4433	*Kabuli*	*Ca_Kabuli_Chr03*	31279604	(C/G)
CakSNP4434	*Kabuli*	*Ca_Kabuli_Chr03*	31279600	(T/C)
CakSNP4435	*Kabuli*	*Ca_Kabuli_Chr03*	31279586	(A/G)
CakSNP4436	*Kabuli*	*Ca_Kabuli_Chr03*	31287618	(C/A)
CakSNP4437	*Kabuli*	*Ca_Kabuli_Chr03*	31287909	(A/G)
CakSNP4438	*Kabuli*	*Ca_Kabuli_Chr03*	31288143	(T/C)
CakSNP4439	*Kabuli*	*Ca_Kabuli_Chr03*	31301723	(G/A)
CakSNP4440	*Kabuli*	*Ca_Kabuli_Chr03*	31301783	(A/G)
CakSNP4441	*Kabuli*	*Ca_Kabuli_Chr03*	31302060	(T/C)
CakSNP4442	*Kabuli*	*Ca_Kabuli_Chr03*	31302117	(C/T)
CakSNP4443	*Kabuli*	*Ca_Kabuli_Chr03*	31313021	(G/T)
CakSNP4444	*Kabuli*	*Ca_Kabuli_Chr03*	31317521	(T/G)
CakSNP4445	*Kabuli*	*Ca_Kabuli_Chr03*	31321725	(A/G)
CakSNP4446	*Kabuli*	*Ca_Kabuli_Chr03*	31321778	(G/A)
CakSNP4447	*Kabuli*	*Ca_Kabuli_Chr03*	31321850	(A/G)
CakSNP4448	*Kabuli*	*Ca_Kabuli_Chr03*	31341335	(C/T)
CakSNP4449	*Kabuli*	*Ca_Kabuli_Chr03*	31341323	(T/C)
CakSNP4450	*Kabuli*	*Ca_Kabuli_Chr03*	31342119	(A/T)
CakSNP4451	*Kabuli*	*Ca_Kabuli_Chr03*	31354988	(G/C)
CakSNP4452	*Kabuli*	*Ca_Kabuli_Chr03*	31355001	(C/T)
CakSNP4453	*Kabuli*	*Ca_Kabuli_Chr03*	31355026	(T/C)
CakSNP4454	*Kabuli*	*Ca_Kabuli_Chr03*	31355066	(A/T)
CakSNP4455	*Kabuli*	*Ca_Kabuli_Chr03*	31357787	(A/T)
CakSNP4456	*Kabuli*	*Ca_Kabuli_Chr03*	31431216	(C/T)
CakSNP4457	*Kabuli*	*Ca_Kabuli_Chr03*	31478804	(T/C)
CakSNP4458	*Kabuli*	*Ca_Kabuli_Chr03*	31478882	(C/T)
CakSNP4459	*Kabuli*	*Ca_Kabuli_Chr03*	31480603	(T/C)
CakSNP4460	*Kabuli*	*Ca_Kabuli_Chr03*	31516662	(G/T)
CakSNP4461	*Kabuli*	*Ca_Kabuli_Chr03*	31516766	(T/C)
CakSNP4462	*Kabuli*	*Ca_Kabuli_Chr03*	31520119	(C/T)
CakSNP4463	*Kabuli*	*Ca_Kabuli_Chr03*	31520225	(A/T)
CakSNP4464	*Kabuli*	*Ca_Kabuli_Chr03*	31520198	(G/A)
CakSNP4465	*Kabuli*	*Ca_Kabuli_Chr03*	31520190	(T/C)
CakSNP4466	*Kabuli*	*Ca_Kabuli_Chr03*	31520832	(G/A)
CakSNP4467	*Kabuli*	*Ca_Kabuli_Chr03*	31521142	(T/C)
CakSNP4468	*Kabuli*	*Ca_Kabuli_Chr03*	31521155	(T/A)

SNP IDs	Cultivars	Chromosomes/scaffolds	Physical positions (bp)	SNPs
CakSNP4469	*Kabuli*	*Ca_Kabuli_Chr03*	31589355	(A/G)
CakSNP4470	*Kabuli*	*Ca_Kabuli_Chr03*	31589329	(C/T)
CakSNP4471	*Kabuli*	*Ca_Kabuli_Chr03*	31591065	(G/A)
CakSNP4472	*Kabuli*	*Ca_Kabuli_Chr03*	31606359	(C/T)
CakSNP4473	*Kabuli*	*Ca_Kabuli_Chr03*	31630175	(T/A)
CakSNP4474	*Kabuli*	*Ca_Kabuli_Chr03*	31630178	(G/T)
CakSNP4475	*Kabuli*	*Ca_Kabuli_Chr03*	31630195	(T/C)
CakSNP4476	*Kabuli*	*Ca_Kabuli_Chr03*	31631451	(A/C)
CakSNP4477	*Kabuli*	*Ca_Kabuli_Chr03*	31631444	(T/G)
CakSNP4478	*Kabuli*	*Ca_Kabuli_Chr03*	31631561	(G/A)
CakSNP4479	*Kabuli*	*Ca_Kabuli_Chr03*	31631534	(A/G)
CakSNP4480	*Kabuli*	*Ca_Kabuli_Chr03*	31631622	(G/A)
CakSNP4481	*Kabuli*	*Ca_Kabuli_Chr03*	31631644	(A/C)
CakSNP4482	*Kabuli*	*Ca_Kabuli_Chr03*	31646735	(C/T)
CakSNP4483	*Kabuli*	*Ca_Kabuli_Chr03*	31646817	(C/T)
CakSNP4484	*Kabuli*	*Ca_Kabuli_Chr03*	31646802	(G/A)
CakSNP4485	*Kabuli*	*Ca_Kabuli_Chr03*	31683906	(A/G)
CakSNP4486	*Kabuli*	*Ca_Kabuli_Chr03*	31689128	(C/T)
CakSNP4487	*Kabuli*	*Ca_Kabuli_Chr03*	31698493	(A/C)
CakSNP4488	*Kabuli*	*Ca_Kabuli_Chr03*	31766363	(G/A)
CakSNP4489	*Kabuli*	*Ca_Kabuli_Chr03*	31770365	(A/C)
CakSNP4490	*Kabuli*	*Ca_Kabuli_Chr03*	31771314	(C/A)
CakSNP4491	*Kabuli*	*Ca_Kabuli_Chr03*	31790189	(C/T)
CakSNP4492	*Kabuli*	*Ca_Kabuli_Chr03*	31790216	(G/A)
CakSNP4493	*Kabuli*	*Ca_Kabuli_Chr03*	31790239	(G/T)
CakSNP4494	*Kabuli*	*Ca_Kabuli_Chr03*	31790352	(G/A)
CakSNP4495	*Kabuli*	*Ca_Kabuli_Chr03*	31814300	(A/G)
CakSNP4496	*Kabuli*	*Ca_Kabuli_Chr03*	31852191	(T/C)
CakSNP4497	*Kabuli*	*Ca_Kabuli_Chr03*	31857877	(C/T)
CakSNP4498	*Kabuli*	*Ca_Kabuli_Chr03*	31859152	(C/G)
CakSNP4499	*Kabuli*	*Ca_Kabuli_Chr03*	31859251	(T/C)
CakSNP4500	*Kabuli*	*Ca_Kabuli_Chr03*	31926694	(C/G)
CakSNP4501	*Kabuli*	*Ca_Kabuli_Chr03*	31931014	(A/C)
CakSNP4502	*Kabuli*	*Ca_Kabuli_Chr03*	31945504	(C/T)
CakSNP4503	*Kabuli*	*Ca_Kabuli_Chr03*	31945634	(A/G)
CakSNP4504	*Kabuli*	*Ca_Kabuli_Chr03*	31951994	(G/A)
CakSNP4505	*Kabuli*	*Ca_Kabuli_Chr03*	31953466	(G/A)
CakSNP4506	*Kabuli*	*Ca_Kabuli_Chr03*	31953549	(C/T)
CakSNP4507	*Kabuli*	*Ca_Kabuli_Chr03*	32351052	(C/T)
CakSNP4508	*Kabuli*	*Ca_Kabuli_Chr03*	32351031	(G/C)
CakSNP4509	*Kabuli*	*Ca_Kabuli_Chr03*	32357599	(C/T)

SNP IDs	Cultivars	Chromosomes/scaffolds	Physical positions (bp)	SNPs
CakSNP4510	*Kabuli*	*Ca_Kabuli_Chr03*	32357578	(G/C)
CakSNP4511	*Kabuli*	*Ca_Kabuli_Chr03*	32448959	(T/C)
CakSNP4512	*Kabuli*	*Ca_Kabuli_Chr03*	32449654	(T/G)
CakSNP4513	*Kabuli*	*Ca_Kabuli_Chr03*	32449700	(C/T)
CakSNP4514	*Kabuli*	*Ca_Kabuli_Chr03*	32510033	(T/G)
CakSNP4515	*Kabuli*	*Ca_Kabuli_Chr03*	32510090	(A/G)
CakSNP4516	*Kabuli*	*Ca_Kabuli_Chr03*	32510130	(A/G)
CakSNP4517	*Kabuli*	*Ca_Kabuli_Chr03*	32510124	(C/T)
CakSNP4518	*Kabuli*	*Ca_Kabuli_Chr03*	32510111	(C/T)
CakSNP4519	*Kabuli*	*Ca_Kabuli_Chr03*	32510071	(G/T)
CakSNP4520	*Kabuli*	*Ca_Kabuli_Chr03*	32510081	(C/T)
CakSNP4521	*Kabuli*	*Ca_Kabuli_Chr03*	32514908	(A/G)
CakSNP4522	*Kabuli*	*Ca_Kabuli_Chr03*	32514890	(A/G)
CakSNP4523	*Kabuli*	*Ca_Kabuli_Chr03*	32514889	(C/T)
CakSNP4524	*Kabuli*	*Ca_Kabuli_Chr03*	32514869	(C/T)
CakSNP4525	*Kabuli*	*Ca_Kabuli_Chr03*	32514868	(A/G)
CakSNP4526	*Kabuli*	*Ca_Kabuli_Chr03*	32514867	(C/T)
CakSNP4527	*Kabuli*	*Ca_Kabuli_Chr03*	32514863	(G/C)
CakSNP4528	*Kabuli*	*Ca_Kabuli_Chr03*	32514859	(C/T)
CakSNP4529	*Kabuli*	*Ca_Kabuli_Chr03*	32514916	(G/C)
CakSNP4530	*Kabuli*	*Ca_Kabuli_Chr03*	32525128	(G/T)
CakSNP4531	*Kabuli*	*Ca_Kabuli_Chr03*	32557850	(C/T)
CakSNP4532	*Kabuli*	*Ca_Kabuli_Chr03*	32571237	(G/A)
CakSNP4533	*Kabuli*	*Ca_Kabuli_Chr03*	32571291	(A/G)
CakSNP4534	*Kabuli*	*Ca_Kabuli_Chr03*	32571275	(T/C)
CakSNP4535	*Kabuli*	*Ca_Kabuli_Chr03*	32600003	(A/G)
CakSNP4536	*Kabuli*	*Ca_Kabuli_Chr03*	32601732	(T/C)
CakSNP4537	*Kabuli*	*Ca_Kabuli_Chr03*	32608947	(A/G)
CakSNP4538	*Kabuli*	*Ca_Kabuli_Chr03*	32662861	(A/G)
CakSNP4539	*Kabuli*	*Ca_Kabuli_Chr03*	32662858	(A/G)
CakSNP4540	*Kabuli*	*Ca_Kabuli_Chr03*	32663015	(C/T)
CakSNP4541	*Kabuli*	*Ca_Kabuli_Chr03*	32663059	(A/C)
CakSNP4542	*Kabuli*	*Ca_Kabuli_Chr03*	32694509	(T/C)
CakSNP4543	*Kabuli*	*Ca_Kabuli_Chr03*	32694454	(T/C)
CakSNP4544	*Kabuli*	*Ca_Kabuli_Chr03*	32704734	(G/A)
CakSNP4545	*Kabuli*	*Ca_Kabuli_Chr03*	32704868	(G/T)
CakSNP4546	*Kabuli*	*Ca_Kabuli_Chr03*	32704953	(G/A)
CakSNP4547	*Kabuli*	*Ca_Kabuli_Chr03*	32706200	(C/T)
CakSNP4548	*Kabuli*	*Ca_Kabuli_Chr03*	32725039	(C/G)
CakSNP4549	*Kabuli*	*Ca_Kabuli_Chr03*	32725096	(A/T)
CakSNP4550	*Kabuli*	*Ca_Kabuli_Chr03*	32758259	(C/A)

SNP IDs	Cultivars	Chromosomes/scaffolds	Physical positions (bp)	SNPs
CakSNP4551	*Kabuli*	*Ca_Kabuli_Chr03*	32758293	(T/C)
CakSNP4552	*Kabuli*	*Ca_Kabuli_Chr03*	32785228	(A/G)
CakSNP4553	*Kabuli*	*Ca_Kabuli_Chr03*	32827067	(C/G)
CakSNP4554	*Kabuli*	*Ca_Kabuli_Chr03*	32827065	(T/G)
CakSNP4555	*Kabuli*	*Ca_Kabuli_Chr03*	32845361	(A/G)
CakSNP4556	*Kabuli*	*Ca_Kabuli_Chr03*	32845395	(C/A)
CakSNP4557	*Kabuli*	*Ca_Kabuli_Chr03*	32847120	(T/A)
CakSNP4558	*Kabuli*	*Ca_Kabuli_Chr03*	32847216	(A/G)
CakSNP4559	*Kabuli*	*Ca_Kabuli_Chr03*	32865549	(C/T)
CakSNP4560	*Kabuli*	*Ca_Kabuli_Chr03*	32865530	(C/G)
CakSNP4561	*Kabuli*	*Ca_Kabuli_Chr03*	32954428	(T/C)
CakSNP4562	*Kabuli*	*Ca_Kabuli_Chr03*	33033245	(T/C)
CakSNP4563	*Kabuli*	*Ca_Kabuli_Chr03*	33091737	(A/C)
CakSNP4564	*Kabuli*	*Ca_Kabuli_Chr03*	33120619	(G/A)
CakSNP4565	*Kabuli*	*Ca_Kabuli_Chr03*	33120825	(G/A)
CakSNP4566	*Kabuli*	*Ca_Kabuli_Chr03*	33520615	(A/C)
CakSNP4567	*Kabuli*	*Ca_Kabuli_Chr03*	33520887	(G/A)
CakSNP4568	*Kabuli*	*Ca_Kabuli_Chr03*	33522974	(G/A)
CakSNP4569	*Kabuli*	*Ca_Kabuli_Chr03*	33533273	(G/A)
CakSNP4570	*Kabuli*	*Ca_Kabuli_Chr03*	33627826	(A/C)
CakSNP4571	*Kabuli*	*Ca_Kabuli_Chr03*	33715215	(G/T)
CakSNP4572	*Kabuli*	*Ca_Kabuli_Chr03*	33898250	(C/T)
CakSNP4573	*Kabuli*	*Ca_Kabuli_Chr03*	33900522	(A/G)
CakSNP4574	*Kabuli*	*Ca_Kabuli_Chr03*	33900533	(A/G)
CakSNP4575	*Kabuli*	*Ca_Kabuli_Chr03*	33904394	(T/A)
CakSNP4576	*Kabuli*	*Ca_Kabuli_Chr03*	34247736	(T/G)
CakSNP4577	*Kabuli*	*Ca_Kabuli_Chr03*	34247831	(G/A)
CakSNP4578	*Kabuli*	*Ca_Kabuli_Chr03*	34247828	(T/C)
CakSNP4579	*Kabuli*	*Ca_Kabuli_Chr03*	34247893	(A/G)
CakSNP4580	*Kabuli*	*Ca_Kabuli_Chr03*	34268356	(A/T)
CakSNP4581	*Kabuli*	*Ca_Kabuli_Chr03*	34282419	(C/T)
CakSNP4582	*Kabuli*	*Ca_Kabuli_Chr03*	34290129	(T/C)
CakSNP4583	*Kabuli*	*Ca_Kabuli_Chr03*	34290135	(G/A)
CakSNP4584	*Kabuli*	*Ca_Kabuli_Chr03*	34290173	(C/T)
CakSNP4585	*Kabuli*	*Ca_Kabuli_Chr03*	34352385	(A/C)
CakSNP4586	*Kabuli*	*Ca_Kabuli_Chr03*	34356844	(A/C)
CakSNP4587	*Kabuli*	*Ca_Kabuli_Chr03*	34357171	(T/G)
CakSNP4588	*Kabuli*	*Ca_Kabuli_Chr03*	34357296	(C/G)
CakSNP4589	*Kabuli*	*Ca_Kabuli_Chr03*	34496982	(G/C)
CakSNP4590	*Kabuli*	*Ca_Kabuli_Chr03*	34524656	(C/A)
CakSNP4591	*Kabuli*	*Ca_Kabuli_Chr03*	34542046	(T/G)

SNP IDs	Cultivars	Chromosomes/scaffolds	Physical positions (bp)	SNPs
CakSNP4592	*Kabuli*	*Ca_Kabuli_Chr03*	34543039	(C/T)
CakSNP4593	*Kabuli*	*Ca_Kabuli_Chr03*	34543111	(A/G)
CakSNP4594	*Kabuli*	*Ca_Kabuli_Chr03*	34547052	(A/T)
CakSNP4595	*Kabuli*	*Ca_Kabuli_Chr03*	34547069	(G/T)
CakSNP4596	*Kabuli*	*Ca_Kabuli_Chr03*	34547141	(A/G)
CakSNP4597	*Kabuli*	*Ca_Kabuli_Chr03*	34576769	(G/A)
CakSNP4598	*Kabuli*	*Ca_Kabuli_Chr03*	34595487	(A/G)
CakSNP4599	*Kabuli*	*Ca_Kabuli_Chr03*	34601038	(T/C)
CakSNP4600	*Kabuli*	*Ca_Kabuli_Chr03*	34603669	(T/A)
CakSNP4601	*Kabuli*	*Ca_Kabuli_Chr03*	34650308	(G/A)
CakSNP4602	*Kabuli*	*Ca_Kabuli_Chr03*	34650245	(G/A)
CakSNP4603	*Kabuli*	*Ca_Kabuli_Chr03*	34662736	(T/G)
CakSNP4604	*Kabuli*	*Ca_Kabuli_Chr03*	34699114	(C/A)
CakSNP4605	*Kabuli*	*Ca_Kabuli_Chr03*	34699076	(T/C)
CakSNP4606	*Kabuli*	*Ca_Kabuli_Chr03*	34722508	(A/T)
CakSNP4607	*Kabuli*	*Ca_Kabuli_Chr03*	34722931	(A/G)
CakSNP4608	*Kabuli*	*Ca_Kabuli_Chr03*	34722979	(T/C)
CakSNP4609	*Kabuli*	*Ca_Kabuli_Chr03*	34722992	(T/G)
CakSNP4610	*Kabuli*	*Ca_Kabuli_Chr03*	34730463	(T/C)
CakSNP4611	*Kabuli*	*Ca_Kabuli_Chr03*	34730954	(G/A)
CakSNP4612	*Kabuli*	*Ca_Kabuli_Chr03*	34800277	(G/A)
CakSNP4613	*Kabuli*	*Ca_Kabuli_Chr03*	34820472	(T/A)
CakSNP4614	*Kabuli*	*Ca_Kabuli_Chr03*	34820532	(T/A)
CakSNP4615	*Kabuli*	*Ca_Kabuli_Chr03*	34852030	(G/A)
CakSNP4616	*Kabuli*	*Ca_Kabuli_Chr03*	34852070	(T/G)
CakSNP4617	*Kabuli*	*Ca_Kabuli_Chr03*	34852047	(T/A)
CakSNP4618	*Kabuli*	*Ca_Kabuli_Chr03*	34889316	(C/G)
CakSNP4619	*Kabuli*	*Ca_Kabuli_Chr03*	34889361	(T/C)
CakSNP4620	*Kabuli*	*Ca_Kabuli_Chr03*	34911621	(G/A)
CakSNP4621	*Kabuli*	*Ca_Kabuli_Chr03*	35071821	(A/G)
CakSNP4622	*Kabuli*	*Ca_Kabuli_Chr03*	35071852	(T/G)
CakSNP4623	*Kabuli*	*Ca_Kabuli_Chr03*	35085559	(T/A)
CakSNP4624	*Kabuli*	*Ca_Kabuli_Chr03*	35085658	(C/A)
CakSNP4625	*Kabuli*	*Ca_Kabuli_Chr03*	35126649	(C/G)
CakSNP4626	*Kabuli*	*Ca_Kabuli_Chr03*	35126766	(G/A)
CakSNP4627	*Kabuli*	*Ca_Kabuli_Chr03*	35126760	(G/A)
CakSNP4628	*Kabuli*	*Ca_Kabuli_Chr03*	35167312	(T/G)
CakSNP4629	*Kabuli*	*Ca_Kabuli_Chr03*	35167359	(T/G)
CakSNP4630	*Kabuli*	*Ca_Kabuli_Chr03*	35185001	(G/T)
CakSNP4631	*Kabuli*	*Ca_Kabuli_Chr03*	35274992	(A/G)
CakSNP4632	*Kabuli*	*Ca_Kabuli_Chr03*	35275199	(T/C)

SNP IDs	Cultivars	Chromosomes/scaffolds	Physical positions (bp)	SNPs
CakSNP4633	*Kabuli*	*Ca_Kabuli_Chr03*	35332088	(T/A)
CakSNP4634	*Kabuli*	*Ca_Kabuli_Chr03*	35332091	(T/C)
CakSNP4635	*Kabuli*	*Ca_Kabuli_Chr03*	35383318	(C/T)
CakSNP4636	*Kabuli*	*Ca_Kabuli_Chr03*	35394684	(G/T)
CakSNP4637	*Kabuli*	*Ca_Kabuli_Chr03*	35403074	(A/G)
CakSNP4638	*Kabuli*	*Ca_Kabuli_Chr03*	35420061	(A/G)
CakSNP4639	*Kabuli*	*Ca_Kabuli_Chr03*	35420108	(T/A)
CakSNP4640	*Kabuli*	*Ca_Kabuli_Chr03*	35424994	(T/C)
CakSNP4641	*Kabuli*	*Ca_Kabuli_Chr03*	35483932	(T/C)
CakSNP4642	*Kabuli*	*Ca_Kabuli_Chr03*	35483960	(T/C)
CakSNP4643	*Kabuli*	*Ca_Kabuli_Chr03*	35483995	(T/C)
CakSNP4644	*Kabuli*	*Ca_Kabuli_Chr03*	35483999	(C/T)
CakSNP4645	*Kabuli*	*Ca_Kabuli_Chr03*	35484034	(G/C)
CakSNP4646	*Kabuli*	*Ca_Kabuli_Chr03*	35487736	(T/C)
CakSNP4647	*Kabuli*	*Ca_Kabuli_Chr03*	35488503	(A/C)
CakSNP4648	*Kabuli*	*Ca_Kabuli_Chr03*	35488597	(T/C)
CakSNP4649	*Kabuli*	*Ca_Kabuli_Chr03*	35488570	(G/T)
CakSNP4650	*Kabuli*	*Ca_Kabuli_Chr03*	35490991	(A/G)
CakSNP4651	*Kabuli*	*Ca_Kabuli_Chr03*	35490951	(C/T)
CakSNP4652	*Kabuli*	*Ca_Kabuli_Chr03*	35543614	(C/T)
CakSNP4653	*Kabuli*	*Ca_Kabuli_Chr03*	35631679	(C/G)
CakSNP4654	*Kabuli*	*Ca_Kabuli_Chr03*	35631689	(C/A)
CakSNP4655	*Kabuli*	*Ca_Kabuli_Chr03*	35631723	(A/G)
CakSNP4656	*Kabuli*	*Ca_Kabuli_Chr03*	35647986	(C/G)
CakSNP4657	*Kabuli*	*Ca_Kabuli_Chr03*	35747394	(C/G)
CakSNP4658	*Kabuli*	*Ca_Kabuli_Chr03*	35807222	(A/G)
CakSNP4659	*Kabuli*	*Ca_Kabuli_Chr03*	35807265	(T/C)
CakSNP4660	*Kabuli*	*Ca_Kabuli_Chr03*	35807407	(A/T)
CakSNP4661	*Kabuli*	*Ca_Kabuli_Chr03*	35829834	(A/G)
CakSNP4662	*Kabuli*	*Ca_Kabuli_Chr03*	35898320	(G/C)
CakSNP4663	*Kabuli*	*Ca_Kabuli_Chr03*	35898374	(G/A)
CakSNP4664	*Kabuli*	*Ca_Kabuli_Chr03*	35898581	(C/T)
CakSNP4665	*Kabuli*	*Ca_Kabuli_Chr03*	35920684	(A/C)
CakSNP4666	*Kabuli*	*Ca_Kabuli_Chr03*	35920860	(C/A)
CakSNP4667	*Kabuli*	*Ca_Kabuli_Chr03*	35948487	(G/A)
CakSNP4668	*Kabuli*	*Ca_Kabuli_Chr03*	35979412	(G/T)
CakSNP4669	*Kabuli*	*Ca_Kabuli_Chr03*	36003699	(G/A)
CakSNP4670	*Kabuli*	*Ca_Kabuli_Chr03*	36003682	(G/T)
CakSNP4671	*Kabuli*	*Ca_Kabuli_Chr03*	36004638	(C/T)
CakSNP4672	*Kabuli*	*Ca_Kabuli_Chr03*	36004663	(T/C)
CakSNP4673	*Kabuli*	*Ca_Kabuli_Chr03*	36004668	(A/G)

**SNP IDs**	**Cultivars**	**Chromosomes/scaffolds**	**Physical positions (bp)**	**SNPs**
CakSNP4674	*Kabuli*	*Ca_Kabuli_Chr03*	36046759	(G/A)
CakSNP4675	*Kabuli*	*Ca_Kabuli_Chr03*	36046938	(C/T)
CakSNP4676	*Kabuli*	*Ca_Kabuli_Chr03*	36046956	(A/C)
CakSNP4677	*Kabuli*	*Ca_Kabuli_Chr03*	36050259	(T/G)
CakSNP4678	*Kabuli*	*Ca_Kabuli_Chr03*	36050319	(T/C)
CakSNP4679	*Kabuli*	*Ca_Kabuli_Chr03*	36071033	(A/G)
CakSNP4680	*Kabuli*	*Ca_Kabuli_Chr03*	36085966	(G/A)
CakSNP4681	*Kabuli*	*Ca_Kabuli_Chr03*	36117521	(A/G)
CakSNP4682	*Kabuli*	*Ca_Kabuli_Chr03*	36118120	(T/C)
CakSNP4683	*Kabuli*	*Ca_Kabuli_Chr03*	36118156	(C/T)
CakSNP4684	*Kabuli*	*Ca_Kabuli_Chr03*	36119592	(T/C)
CakSNP4685	*Kabuli*	*Ca_Kabuli_Chr03*	36119602	(C/A)
CakSNP4686	*Kabuli*	*Ca_Kabuli_Chr03*	36130561	(T/A)
CakSNP4687	*Kabuli*	*Ca_Kabuli_Chr03*	36130610	(C/A)
CakSNP4688	*Kabuli*	*Ca_Kabuli_Chr03*	36132440	(T/C)
CakSNP4689	*Kabuli*	*Ca_Kabuli_Chr03*	36132437	(C/T)
CakSNP4690	*Kabuli*	*Ca_Kabuli_Chr03*	36132401	(C/T)
CakSNP4691	*Kabuli*	*Ca_Kabuli_Chr03*	36132480	(C/T)
CakSNP4692	*Kabuli*	*Ca_Kabuli_Chr03*	36132539	(C/T)
CakSNP4693	*Kabuli*	*Ca_Kabuli_Chr03*	36132586	(T/G)
CakSNP4694	*Kabuli*	*Ca_Kabuli_Chr03*	36132570	(G/A)
CakSNP4695	*Kabuli*	*Ca_Kabuli_Chr03*	36132554	(A/T)
CakSNP4696	*Kabuli*	*Ca_Kabuli_Chr03*	36150722	(G/A)
CakSNP4697	*Kabuli*	*Ca_Kabuli_Chr03*	36150717	(T/C)
CakSNP4698	*Kabuli*	*Ca_Kabuli_Chr03*	36164863	(T/G)
CakSNP4699	*Kabuli*	*Ca_Kabuli_Chr03*	36164866	(T/C)
CakSNP4700	*Kabuli*	*Ca_Kabuli_Chr03*	36165997	(C/G)
CakSNP4701	*Kabuli*	*Ca_Kabuli_Chr03*	36167136	(G/T)
CakSNP4702	*Kabuli*	*Ca_Kabuli_Chr03*	36175164	(G/A)
CakSNP4703	*Kabuli*	*Ca_Kabuli_Chr03*	36177201	(T/C)
CakSNP4704	*Kabuli*	*Ca_Kabuli_Chr03*	36205806	(T/G)
CakSNP4705	*Kabuli*	*Ca_Kabuli_Chr03*	36205819	(A/G)
CakSNP4706	*Kabuli*	*Ca_Kabuli_Chr03*	36205845	(A/G)
CakSNP4707	*Kabuli*	*Ca_Kabuli_Chr03*	36206615	(A/C)
CakSNP4708	*Kabuli*	*Ca_Kabuli_Chr03*	36212799	(A/T)
CakSNP4709	*Kabuli*	*Ca_Kabuli_Chr03*	36212809	(G/A)
CakSNP4710	*Kabuli*	*Ca_Kabuli_Chr03*	36212843	(T/A)
CakSNP4711	*Kabuli*	*Ca_Kabuli_Chr03*	36217686	(G/A)
CakSNP4712	*Kabuli*	*Ca_Kabuli_Chr03*	36227565	(A/T)
CakSNP4713	*Kabuli*	*Ca_Kabuli_Chr03*	36227566	(A/T)
CakSNP4714	*Kabuli*	*Ca_Kabuli_Chr03*	36227948	(A/G)

**SNP IDs**	**Cultivars**	**Chromosomes/scaffolds**	**Physical positions (bp)**	**SNPs**
CakSNP4715	*Kabuli*	*Ca_Kabuli_Chr03*	36227949	(C/T)
CakSNP4716	*Kabuli*	*Ca_Kabuli_Chr03*	36228078	(G/A)
CakSNP4717	*Kabuli*	*Ca_Kabuli_Chr03*	36228032	(G/T)
CakSNP4718	*Kabuli*	*Ca_Kabuli_Chr03*	36228121	(T/A)
CakSNP4719	*Kabuli*	*Ca_Kabuli_Chr03*	36228126	(T/A)
CakSNP4720	*Kabuli*	*Ca_Kabuli_Chr03*	36242506	(C/T)
CakSNP4721	*Kabuli*	*Ca_Kabuli_Chr03*	36242504	(C/T)
CakSNP4722	*Kabuli*	*Ca_Kabuli_Chr03*	36242490	(G/T)
CakSNP4723	*Kabuli*	*Ca_Kabuli_Chr03*	36242480	(G/C)
CakSNP4724	*Kabuli*	*Ca_Kabuli_Chr03*	36247792	(T/C)
CakSNP4725	*Kabuli*	*Ca_Kabuli_Chr03*	36260865	(C/A)
CakSNP4726	*Kabuli*	*Ca_Kabuli_Chr03*	36282091	(T/G)
CakSNP4727	*Kabuli*	*Ca_Kabuli_Chr03*	36293589	(A/G)
CakSNP4728	*Kabuli*	*Ca_Kabuli_Chr03*	36293625	(C/T)
CakSNP4729	*Kabuli*	*Ca_Kabuli_Chr03*	36293637	(C/T)
CakSNP4730	*Kabuli*	*Ca_Kabuli_Chr03*	36330750	(A/G)
CakSNP4731	*Kabuli*	*Ca_Kabuli_Chr03*	36330815	(T/C)
CakSNP4732	*Kabuli*	*Ca_Kabuli_Chr03*	36345158	(T/C)
CakSNP4733	*Kabuli*	*Ca_Kabuli_Chr03*	36347203	(T/A)
CakSNP4734	*Kabuli*	*Ca_Kabuli_Chr03*	36361339	(C/T)
CakSNP4735	*Kabuli*	*Ca_Kabuli_Chr03*	36378294	(G/T)
CakSNP4736	*Kabuli*	*Ca_Kabuli_Chr03*	36378299	(C/G)
CakSNP4737	*Kabuli*	*Ca_Kabuli_Chr03*	36378358	(G/A)
CakSNP4738	*Kabuli*	*Ca_Kabuli_Chr03*	36387615	(A/T)
CakSNP4739	*Kabuli*	*Ca_Kabuli_Chr03*	36387732	(G/A)
CakSNP4740	*Kabuli*	*Ca_Kabuli_Chr03*	36387718	(C/T)
CakSNP4741	*Kabuli*	*Ca_Kabuli_Chr03*	36396559	(C/A)
CakSNP4742	*Kabuli*	*Ca_Kabuli_Chr03*	36420902	(T/C)
CakSNP4743	*Kabuli*	*Ca_Kabuli_Chr03*	36432389	(A/G)
CakSNP4744	*Kabuli*	*Ca_Kabuli_Chr03*	36447274	(G/A)
CakSNP4745	*Kabuli*	*Ca_Kabuli_Chr03*	36447364	(T/A)
CakSNP4746	*Kabuli*	*Ca_Kabuli_Chr03*	36447520	(T/A)
CakSNP4747	*Kabuli*	*Ca_Kabuli_Chr03*	36447525	(C/T)
CakSNP4748	*Kabuli*	*Ca_Kabuli_Chr03*	36488722	(T/A)
CakSNP4749	*Kabuli*	*Ca_Kabuli_Chr03*	36488736	(T/C)
CakSNP4750	*Kabuli*	*Ca_Kabuli_Chr03*	36488853	(A/G)
CakSNP4751	*Kabuli*	*Ca_Kabuli_Chr03*	36488890	(A/G)
CakSNP4752	*Kabuli*	*Ca_Kabuli_Chr03*	36503906	(C/T)
CakSNP4753	*Kabuli*	*Ca_Kabuli_Chr03*	36503912	(T/C)
CakSNP4754	*Kabuli*	*Ca_Kabuli_Chr03*	36524598	(G/C)
CakSNP4755	*Kabuli*	*Ca_Kabuli_Chr03*	36524635	(A/G)

SNP IDs	Cultivars	Chromosomes/scaffolds	Physical positions (bp)	SNPs
CakSNP4756	*Kabuli*	*Ca_Kabuli_Chr03*	36524659	(T/C)
CakSNP4757	*Kabuli*	*Ca_Kabuli_Chr03*	36531151	(C/A)
CakSNP4758	*Kabuli*	*Ca_Kabuli_Chr03*	36588856	(C/T)
CakSNP4759	*Kabuli*	*Ca_Kabuli_Chr03*	36588896	(C/G)
CakSNP4760	*Kabuli*	*Ca_Kabuli_Chr03*	36606527	(G/A)
CakSNP4761	*Kabuli*	*Ca_Kabuli_Chr03*	36609043	(G/A)
CakSNP4762	*Kabuli*	*Ca_Kabuli_Chr03*	36616855	(T/C)
CakSNP4763	*Kabuli*	*Ca_Kabuli_Chr03*	36644117	(A/T)
CakSNP4764	*Kabuli*	*Ca_Kabuli_Chr03*	36733097	(G/A)
CakSNP4765	*Kabuli*	*Ca_Kabuli_Chr03*	36733157	(A/G)
CakSNP4766	*Kabuli*	*Ca_Kabuli_Chr03*	36733211	(A/G)
CakSNP4767	*Kabuli*	*Ca_Kabuli_Chr03*	36749775	(T/A)
CakSNP4768	*Kabuli*	*Ca_Kabuli_Chr03*	36749826	(G/A)
CakSNP4769	*Kabuli*	*Ca_Kabuli_Chr03*	36749829	(T/C)
CakSNP4770	*Kabuli*	*Ca_Kabuli_Chr03*	36749831	(T/C)
CakSNP4771	*Kabuli*	*Ca_Kabuli_Chr03*	36749871	(T/C)
CakSNP4772	*Kabuli*	*Ca_Kabuli_Chr03*	36749904	(C/T)
CakSNP4773	*Kabuli*	*Ca_Kabuli_Chr03*	36749916	(C/A)
CakSNP4774	*Kabuli*	*Ca_Kabuli_Chr03*	36752689	(T/G)
CakSNP4775	*Kabuli*	*Ca_Kabuli_Chr03*	36769775	(G/T)
CakSNP4776	*Kabuli*	*Ca_Kabuli_Chr03*	36783494	(T/A)
CakSNP4777	*Kabuli*	*Ca_Kabuli_Chr03*	36825463	(T/C)
CakSNP4778	*Kabuli*	*Ca_Kabuli_Chr03*	36860134	(A/G)
CakSNP4779	*Kabuli*	*Ca_Kabuli_Chr03*	36860246	(T/C)
CakSNP4780	*Kabuli*	*Ca_Kabuli_Chr03*	36873720	(T/C)
CakSNP4781	*Kabuli*	*Ca_Kabuli_Chr03*	36883268	(T/C)
CakSNP4782	*Kabuli*	*Ca_Kabuli_Chr03*	36883333	(A/T)
CakSNP4783	*Kabuli*	*Ca_Kabuli_Chr03*	36895362	(T/C)
CakSNP4784	*Kabuli*	*Ca_Kabuli_Chr03*	36938148	(A/G)
CakSNP4785	*Kabuli*	*Ca_Kabuli_Chr03*	36938271	(G/C)
CakSNP4786	*Kabuli*	*Ca_Kabuli_Chr03*	36943522	(A/G)
CakSNP4787	*Kabuli*	*Ca_Kabuli_Chr03*	36991718	(C/A)
CakSNP4788	*Kabuli*	*Ca_Kabuli_Chr03*	37003976	(C/T)
CakSNP4789	*Kabuli*	*Ca_Kabuli_Chr03*	37004026	(A/G)
CakSNP4790	*Kabuli*	*Ca_Kabuli_Chr03*	37022354	(A/G)
CakSNP4791	*Kabuli*	*Ca_Kabuli_Chr03*	37034618	(G/A)
CakSNP4792	*Kabuli*	*Ca_Kabuli_Chr03*	37067095	(T/G)
CakSNP4793	*Kabuli*	*Ca_Kabuli_Chr03*	37072289	(A/G)
CakSNP4794	*Kabuli*	*Ca_Kabuli_Chr03*	37072276	(T/G)
CakSNP4795	*Kabuli*	*Ca_Kabuli_Chr03*	37072312	(T/C)
CakSNP4796	*Kabuli*	*Ca_Kabuli_Chr03*	37077087	(G/T)

**SNP IDs**	**Cultivars**	**Chromosomes/scaffolds**	**Physical positions (bp)**	**SNPs**
CakSNP4797	*Kabuli*	*Ca_Kabuli_Chr03*	37090968	(T/C)
CakSNP4798	*Kabuli*	*Ca_Kabuli_Chr03*	37091004	(A/G)
CakSNP4799	*Kabuli*	*Ca_Kabuli_Chr03*	37094437	(T/C)
CakSNP4800	*Kabuli*	*Ca_Kabuli_Chr03*	37100897	(G/T)
CakSNP4801	*Kabuli*	*Ca_Kabuli_Chr03*	37101339	(A/T)
CakSNP4802	*Kabuli*	*Ca_Kabuli_Chr03*	37101433	(T/G)
CakSNP4803	*Kabuli*	*Ca_Kabuli_Chr03*	37101409	(A/G)
CakSNP4804	*Kabuli*	*Ca_Kabuli_Chr03*	37108762	(A/G)
CakSNP4805	*Kabuli*	*Ca_Kabuli_Chr03*	37108908	(A/G)
CakSNP4806	*Kabuli*	*Ca_Kabuli_Chr03*	37108904	(A/T)
CakSNP4807	*Kabuli*	*Ca_Kabuli_Chr03*	37108876	(C/A)
CakSNP4808	*Kabuli*	*Ca_Kabuli_Chr03*	37110070	(A/G)
CakSNP4809	*Kabuli*	*Ca_Kabuli_Chr03*	37110124	(A/T)
CakSNP4810	*Kabuli*	*Ca_Kabuli_Chr03*	37146766	(C/G)
CakSNP4811	*Kabuli*	*Ca_Kabuli_Chr03*	37146840	(A/G)
CakSNP4812	*Kabuli*	*Ca_Kabuli_Chr03*	37154761	(T/G)
CakSNP4813	*Kabuli*	*Ca_Kabuli_Chr03*	37154857	(T/C)
CakSNP4814	*Kabuli*	*Ca_Kabuli_Chr03*	37154938	(A/C)
CakSNP4815	*Kabuli*	*Ca_Kabuli_Chr03*	37154957	(C/A)
CakSNP4816	*Kabuli*	*Ca_Kabuli_Chr03*	37155925	(G/A)
CakSNP4817	*Kabuli*	*Ca_Kabuli_Chr03*	37156951	(A/G)
CakSNP4818	*Kabuli*	*Ca_Kabuli_Chr03*	37160822	(G/T)
CakSNP4819	*Kabuli*	*Ca_Kabuli_Chr03*	37160939	(A/G)
CakSNP4820	*Kabuli*	*Ca_Kabuli_Chr03*	37164633	(T/C)
CakSNP4821	*Kabuli*	*Ca_Kabuli_Chr03*	37170249	(C/T)
CakSNP4822	*Kabuli*	*Ca_Kabuli_Chr03*	37271869	(T/C)
CakSNP4823	*Kabuli*	*Ca_Kabuli_Chr03*	37300206	(T/G)
CakSNP4824	*Kabuli*	*Ca_Kabuli_Chr03*	37303478	(G/A)
CakSNP4825	*Kabuli*	*Ca_Kabuli_Chr03*	37308614	(T/C)
CakSNP4826	*Kabuli*	*Ca_Kabuli_Chr03*	37309420	(A/C)
CakSNP4827	*Kabuli*	*Ca_Kabuli_Chr03*	37316736	(T/C)
CakSNP4828	*Kabuli*	*Ca_Kabuli_Chr03*	37316737	(C/T)
CakSNP4829	*Kabuli*	*Ca_Kabuli_Chr03*	37316782	(G/A)
CakSNP4830	*Kabuli*	*Ca_Kabuli_Chr03*	37355013	(A/G)
CakSNP4831	*Kabuli*	*Ca_Kabuli_Chr03*	37444451	(C/A)
CakSNP4832	*Kabuli*	*Ca_Kabuli_Chr03*	37566557	(C/A)
CakSNP4833	*Kabuli*	*Ca_Kabuli_Chr03*	37599142	(T/C)
CakSNP4834	*Kabuli*	*Ca_Kabuli_Chr03*	37604672	(T/A)
CakSNP4835	*Kabuli*	*Ca_Kabuli_Chr03*	37630502	(T/C)
CakSNP4836	*Kabuli*	*Ca_Kabuli_Chr03*	37635909	(G/A)
CakSNP4837	*Kabuli*	*Ca_Kabuli_Chr03*	37635961	(C/T)

**SNP IDs**	**Cultivars**	**Chromosomes/scaffolds**	**Physical positions (bp)**	**SNPs**
CakSNP4838	*Kabuli*	*Ca_Kabuli_Chr03*	37639300	(A/G)
CakSNP4839	*Kabuli*	*Ca_Kabuli_Chr03*	37682816	(T/C)
CakSNP4840	*Kabuli*	*Ca_Kabuli_Chr03*	37682824	(G/A)
CakSNP4841	*Kabuli*	*Ca_Kabuli_Chr03*	37723959	(C/T)
CakSNP4842	*Kabuli*	*Ca_Kabuli_Chr03*	37759329	(G/A)
CakSNP4843	*Kabuli*	*Ca_Kabuli_Chr03*	37765767	(A/C)
CakSNP4844	*Kabuli*	*Ca_Kabuli_Chr03*	37806690	(G/A)
CakSNP4845	*Kabuli*	*Ca_Kabuli_Chr03*	37814955	(A/T)
CakSNP4846	*Kabuli*	*Ca_Kabuli_Chr03*	37814954	(T/A)
CakSNP4847	*Kabuli*	*Ca_Kabuli_Chr03*	37816895	(T/A)
CakSNP4848	*Kabuli*	*Ca_Kabuli_Chr03*	37859943	(T/C)
CakSNP4849	*Kabuli*	*Ca_Kabuli_Chr03*	37864390	(C/T)
CakSNP4850	*Kabuli*	*Ca_Kabuli_Chr03*	37866258	(A/G)
CakSNP4851	*Kabuli*	*Ca_Kabuli_Chr03*	37886983	(G/T)
CakSNP4852	*Kabuli*	*Ca_Kabuli_Chr03*	37886971	(A/G)
CakSNP4853	*Kabuli*	*Ca_Kabuli_Chr03*	37886942	(A/C)
CakSNP4854	*Kabuli*	*Ca_Kabuli_Chr03*	37908317	(T/C)
CakSNP4855	*Kabuli*	*Ca_Kabuli_Chr03*	37908492	(A/C)
CakSNP4856	*Kabuli*	*Ca_Kabuli_Chr03*	37910881	(A/G)
CakSNP4857	*Kabuli*	*Ca_Kabuli_Chr03*	37910899	(T/C)
CakSNP4858	*Kabuli*	*Ca_Kabuli_Chr03*	37910911	(C/T)
CakSNP4859	*Kabuli*	*Ca_Kabuli_Chr03*	37913266	(C/T)
CakSNP4860	*Kabuli*	*Ca_Kabuli_Chr03*	37919880	(C/T)
CakSNP4861	*Kabuli*	*Ca_Kabuli_Chr03*	37942807	(A/G)
CakSNP4862	*Kabuli*	*Ca_Kabuli_Chr03*	37942962	(T/C)
CakSNP4863	*Kabuli*	*Ca_Kabuli_Chr03*	37985612	(A/C)
CakSNP4864	*Kabuli*	*Ca_Kabuli_Chr03*	37985652	(C/T)
CakSNP4865	*Kabuli*	*Ca_Kabuli_Chr03*	37985776	(T/A)
CakSNP4866	*Kabuli*	*Ca_Kabuli_Chr03*	37985977	(A/G)
CakSNP4867	*Kabuli*	*Ca_Kabuli_Chr03*	37985947	(A/T)
CakSNP4868	*Kabuli*	*Ca_Kabuli_Chr03*	38026470	(G/C)
CakSNP4869	*Kabuli*	*Ca_Kabuli_Chr03*	38026539	(A/G)
CakSNP4870	*Kabuli*	*Ca_Kabuli_Chr03*	38026734	(G/C)
CakSNP4871	*Kabuli*	*Ca_Kabuli_Chr03*	38026725	(G/A)
CakSNP4872	*Kabuli*	*Ca_Kabuli_Chr03*	38029095	(G/C)
CakSNP4873	*Kabuli*	*Ca_Kabuli_Chr03*	38033965	(G/A)
CakSNP4874	*Kabuli*	*Ca_Kabuli_Chr03*	38033969	(A/G)
CakSNP4875	*Kabuli*	*Ca_Kabuli_Chr03*	38046510	(T/C)
CakSNP4876	*Kabuli*	*Ca_Kabuli_Chr03*	38046575	(A/C)
CakSNP4877	*Kabuli*	*Ca_Kabuli_Chr03*	38049996	(A/G)
CakSNP4878	*Kabuli*	*Ca_Kabuli_Chr03*	38059303	(T/C)

SNP IDs	Cultivars	Chromosomes/scaffolds	Physical positions (bp)	SNPs
CakSNP4879	*Kabuli*	*Ca_Kabuli_Chr03*	38059311	(T/A)
CakSNP4880	*Kabuli*	*Ca_Kabuli_Chr03*	38060814	(A/G)
CakSNP4881	*Kabuli*	*Ca_Kabuli_Chr03*	38060755	(T/C)
CakSNP4882	*Kabuli*	*Ca_Kabuli_Chr03*	38060742	(C/T)
CakSNP4883	*Kabuli*	*Ca_Kabuli_Chr03*	38074259	(C/A)
CakSNP4884	*Kabuli*	*Ca_Kabuli_Chr03*	38103375	(T/C)
CakSNP4885	*Kabuli*	*Ca_Kabuli_Chr03*	38103378	(C/T)
CakSNP4886	*Kabuli*	*Ca_Kabuli_Chr03*	38103381	(C/T)
CakSNP4887	*Kabuli*	*Ca_Kabuli_Chr03*	38103385	(C/T)
CakSNP4888	*Kabuli*	*Ca_Kabuli_Chr03*	38103524	(C/T)
CakSNP4889	*Kabuli*	*Ca_Kabuli_Chr03*	38112801	(C/T)
CakSNP4890	*Kabuli*	*Ca_Kabuli_Chr03*	38112802	(T/C)
CakSNP4891	*Kabuli*	*Ca_Kabuli_Chr03*	38112971	(A/G)
CakSNP4892	*Kabuli*	*Ca_Kabuli_Chr03*	38117169	(T/C)
CakSNP4893	*Kabuli*	*Ca_Kabuli_Chr03*	38130415	(T/C)
CakSNP4894	*Kabuli*	*Ca_Kabuli_Chr03*	38130410	(A/G)
CakSNP4895	*Kabuli*	*Ca_Kabuli_Chr03*	38145115	(C/T)
CakSNP4896	*Kabuli*	*Ca_Kabuli_Chr03*	38157519	(T/C)
CakSNP4897	*Kabuli*	*Ca_Kabuli_Chr03*	38166074	(A/G)
CakSNP4898	*Kabuli*	*Ca_Kabuli_Chr03*	38166881	(C/T)
CakSNP4899	*Kabuli*	*Ca_Kabuli_Chr03*	38202223	(C/T)
CakSNP4900	*Kabuli*	*Ca_Kabuli_Chr03*	38220910	(T/C)
CakSNP4901	*Kabuli*	*Ca_Kabuli_Chr03*	38220949	(A/T)
CakSNP4902	*Kabuli*	*Ca_Kabuli_Chr03*	38220958	(A/G)
CakSNP4903	*Kabuli*	*Ca_Kabuli_Chr03*	38222750	(A/C)
CakSNP4904	*Kabuli*	*Ca_Kabuli_Chr03*	38222747	(G/A)
CakSNP4905	*Kabuli*	*Ca_Kabuli_Chr03*	38222733	(C/G)
CakSNP4906	*Kabuli*	*Ca_Kabuli_Chr03*	38228118	(G/T)
CakSNP4907	*Kabuli*	*Ca_Kabuli_Chr03*	38237246	(T/G)
CakSNP4908	*Kabuli*	*Ca_Kabuli_Chr03*	38251660	(C/A)
CakSNP4909	*Kabuli*	*Ca_Kabuli_Chr03*	38253654	(T/C)
CakSNP4910	*Kabuli*	*Ca_Kabuli_Chr03*	38258645	(C/A)
CakSNP4911	*Kabuli*	*Ca_Kabuli_Chr03*	38264250	(C/T)
CakSNP4912	*Kabuli*	*Ca_Kabuli_Chr03*	38294286	(C/A)
CakSNP4913	*Kabuli*	*Ca_Kabuli_Chr03*	38296573	(A/G)
CakSNP4914	*Kabuli*	*Ca_Kabuli_Chr03*	38315560	(G/T)
CakSNP4915	*Kabuli*	*Ca_Kabuli_Chr03*	38321159	(T/C)
CakSNP4916	*Kabuli*	*Ca_Kabuli_Chr03*	38321153	(G/T)
CakSNP4917	*Kabuli*	*Ca_Kabuli_Chr03*	38386392	(T/C)
CakSNP4918	*Kabuli*	*Ca_Kabuli_Chr03*	38424238	(A/T)
CakSNP4919	*Kabuli*	*Ca_Kabuli_Chr03*	38452060	(A/C)

SNP IDs	Cultivars	Chromosomes/scaffolds	Physical positions (bp)	SNPs
CakSNP4920	*Kabuli*	*Ca_Kabuli_Chr03*	38465366	(G/A)
CakSNP4921	*Kabuli*	*Ca_Kabuli_Chr03*	38465335	(G/A)
CakSNP4922	*Kabuli*	*Ca_Kabuli_Chr03*	38465440	(G/A)
CakSNP4923	*Kabuli*	*Ca_Kabuli_Chr03*	38496236	(A/G)
CakSNP4924	*Kabuli*	*Ca_Kabuli_Chr03*	38501705	(T/C)
CakSNP4925	*Kabuli*	*Ca_Kabuli_Chr03*	38539721	(T/G)
CakSNP4926	*Kabuli*	*Ca_Kabuli_Chr03*	38551142	(C/T)
CakSNP4927	*Kabuli*	*Ca_Kabuli_Chr03*	38555941	(G/A)
CakSNP4928	*Kabuli*	*Ca_Kabuli_Chr03*	38555944	(A/G)
CakSNP4929	*Kabuli*	*Ca_Kabuli_Chr03*	38592077	(G/A)
CakSNP4930	*Kabuli*	*Ca_Kabuli_Chr03*	38624786	(C/T)
CakSNP4931	*Kabuli*	*Ca_Kabuli_Chr03*	38624934	(T/C)
CakSNP4932	*Kabuli*	*Ca_Kabuli_Chr03*	38672863	(C/A)
CakSNP4933	*Kabuli*	*Ca_Kabuli_Chr03*	38704356	(C/T)
CakSNP4934	*Kabuli*	*Ca_Kabuli_Chr03*	38728528	(C/T)
CakSNP4935	*Kabuli*	*Ca_Kabuli_Chr03*	38728679	(T/C)
CakSNP4936	*Kabuli*	*Ca_Kabuli_Chr03*	38731315	(C/T)
CakSNP4937	*Kabuli*	*Ca_Kabuli_Chr03*	38731466	(T/G)
CakSNP4938	*Kabuli*	*Ca_Kabuli_Chr03*	38764372	(G/A)
CakSNP4939	*Kabuli*	*Ca_Kabuli_Chr03*	38764390	(A/C)
CakSNP4940	*Kabuli*	*Ca_Kabuli_Chr03*	38766156	(T/A)
CakSNP4941	*Kabuli*	*Ca_Kabuli_Chr03*	38766174	(A/G)
CakSNP4942	*Kabuli*	*Ca_Kabuli_Chr03*	38766311	(A/C)
CakSNP4943	*Kabuli*	*Ca_Kabuli_Chr03*	38771817	(A/G)
CakSNP4944	*Kabuli*	*Ca_Kabuli_Chr03*	38776366	(T/G)
CakSNP4945	*Kabuli*	*Ca_Kabuli_Chr03*	38785843	(C/G)
CakSNP4946	*Kabuli*	*Ca_Kabuli_Chr03*	38785845	(C/T)
CakSNP4947	*Kabuli*	*Ca_Kabuli_Chr03*	38793194	(T/C)
CakSNP4948	*Kabuli*	*Ca_Kabuli_Chr03*	38805822	(T/A)
CakSNP4949	*Kabuli*	*Ca_Kabuli_Chr03*	38805831	(A/C)
CakSNP4950	*Kabuli*	*Ca_Kabuli_Chr03*	38809132	(G/A)
CakSNP4951	*Kabuli*	*Ca_Kabuli_Chr03*	38810765	(A/T)
CakSNP4952	*Kabuli*	*Ca_Kabuli_Chr03*	38818340	(A/G)
CakSNP4953	*Kabuli*	*Ca_Kabuli_Chr03*	38821990	(A/G)
CakSNP4954	*Kabuli*	*Ca_Kabuli_Chr03*	38831533	(T/C)
CakSNP4955	*Kabuli*	*Ca_Kabuli_Chr03*	38848358	(C/T)
CakSNP4956	*Kabuli*	*Ca_Kabuli_Chr03*	38848412	(C/G)
CakSNP4957	*Kabuli*	*Ca_Kabuli_Chr03*	38878577	(C/G)
CakSNP4958	*Kabuli*	*Ca_Kabuli_Chr03*	38878664	(G/C)
CakSNP4959	*Kabuli*	*Ca_Kabuli_Chr03*	38878649	(T/C)
CakSNP4960	*Kabuli*	*Ca_Kabuli_Chr03*	38878736	(G/C)

SNP IDs	Cultivars	Chromosomes/scaffolds	Physical positions (bp)	SNPs
CakSNP4961	*Kabuli*	*Ca_Kabuli_Chr03*	38887640	(C/T)
CakSNP4962	*Kabuli*	*Ca_Kabuli_Chr03*	38891567	(T/C)
CakSNP4963	*Kabuli*	*Ca_Kabuli_Chr03*	38896013	(A/G)
CakSNP4964	*Kabuli*	*Ca_Kabuli_Chr03*	38913449	(T/C)
CakSNP4965	*Kabuli*	*Ca_Kabuli_Chr03*	38914528	(A/G)
CakSNP4966	*Kabuli*	*Ca_Kabuli_Chr03*	38923321	(C/T)
CakSNP4967	*Kabuli*	*Ca_Kabuli_Chr03*	38951428	(G/A)
CakSNP4968	*Kabuli*	*Ca_Kabuli_Chr03*	38951698	(A/C)
CakSNP4969	*Kabuli*	*Ca_Kabuli_Chr03*	38965610	(T/A)
CakSNP4970	*Kabuli*	*Ca_Kabuli_Chr03*	38982481	(A/G)
CakSNP4971	*Kabuli*	*Ca_Kabuli_Chr03*	38987810	(G/A)
CakSNP4972	*Kabuli*	*Ca_Kabuli_Chr03*	38987916	(A/C)
CakSNP4973	*Kabuli*	*Ca_Kabuli_Chr03*	39003771	(G/A)
CakSNP4974	*Kabuli*	*Ca_Kabuli_Chr03*	39013988	(T/G)
CakSNP4975	*Kabuli*	*Ca_Kabuli_Chr03*	39029251	(A/C)
CakSNP4976	*Kabuli*	*Ca_Kabuli_Chr03*	39034246	(C/T)
CakSNP4977	*Kabuli*	*Ca_Kabuli_Chr03*	39034433	(A/G)
CakSNP4978	*Kabuli*	*Ca_Kabuli_Chr03*	39036130	(T/C)
CakSNP4979	*Kabuli*	*Ca_Kabuli_Chr03*	39052024	(T/C)
CakSNP4980	*Kabuli*	*Ca_Kabuli_Chr03*	39055529	(C/T)
CakSNP4981	*Kabuli*	*Ca_Kabuli_Chr03*	39055455	(T/C)
CakSNP4982	*Kabuli*	*Ca_Kabuli_Chr03*	39081040	(A/G)
CakSNP4983	*Kabuli*	*Ca_Kabuli_Chr03*	39084916	(T/A)
CakSNP4984	*Kabuli*	*Ca_Kabuli_Chr03*	39084979	(A/C)
CakSNP4985	*Kabuli*	*Ca_Kabuli_Chr03*	39093242	(G/A)
CakSNP4986	*Kabuli*	*Ca_Kabuli_Chr03*	39093243	(C/A)
CakSNP4987	*Kabuli*	*Ca_Kabuli_Chr03*	39093245	(T/A)
CakSNP4988	*Kabuli*	*Ca_Kabuli_Chr03*	39093215	(G/A)
CakSNP4989	*Kabuli*	*Ca_Kabuli_Chr03*	39115274	(A/C)
CakSNP4990	*Kabuli*	*Ca_Kabuli_Chr03*	39115986	(C/G)
CakSNP4991	*Kabuli*	*Ca_Kabuli_Chr03*	39115979	(A/G)
CakSNP4992	*Kabuli*	*Ca_Kabuli_Chr03*	39115972	(T/A)
CakSNP4993	*Kabuli*	*Ca_Kabuli_Chr03*	39115970	(T/G)
CakSNP4994	*Kabuli*	*Ca_Kabuli_Chr03*	39118510	(C/T)
CakSNP4995	*Kabuli*	*Ca_Kabuli_Chr03*	39200823	(C/A)
CakSNP4996	*Kabuli*	*Ca_Kabuli_Chr03*	39200847	(A/C)
CakSNP4997	*Kabuli*	*Ca_Kabuli_Chr03*	39202479	(G/C)
CakSNP4998	*Kabuli*	*Ca_Kabuli_Chr03*	39202481	(A/T)
CakSNP4999	*Kabuli*	*Ca_Kabuli_Chr03*	39207112	(A/C)
CakSNP5000	*Kabuli*	*Ca_Kabuli_Chr03*	39215327	(C/T)
CakSNP5001	*Kabuli*	*Ca_Kabuli_Chr03*	39215476	(G/A)

SNP IDs	Cultivars	Chromosomes/scaffolds	Physical positions (bp)	SNPs
CakSNP5002	*Kabuli*	*Ca_Kabuli_Chr03*	39215558	(C/T)
CakSNP5003	*Kabuli*	*Ca_Kabuli_Chr03*	39233774	(A/T)
CakSNP5004	*Kabuli*	*Ca_Kabuli_Chr03*	39233852	(A/C)
CakSNP5005	*Kabuli*	*Ca_Kabuli_Chr03*	39233885	(A/T)
CakSNP5006	*Kabuli*	*Ca_Kabuli_Chr03*	39234161	(A/G)
CakSNP5007	*Kabuli*	*Ca_Kabuli_Chr03*	39254527	(T/G)
CakSNP5008	*Kabuli*	*Ca_Kabuli_Chr03*	39254532	(C/G)
CakSNP5009	*Kabuli*	*Ca_Kabuli_Chr03*	39255721	(A/C)
CakSNP5010	*Kabuli*	*Ca_Kabuli_Chr03*	39255715	(G/A)
CakSNP5011	*Kabuli*	*Ca_Kabuli_Chr03*	39255713	(C/A)
CakSNP5012	*Kabuli*	*Ca_Kabuli_Chr03*	39255709	(A/T)
CakSNP5013	*Kabuli*	*Ca_Kabuli_Chr03*	39255729	(C/T)
CakSNP5014	*Kabuli*	*Ca_Kabuli_Chr03*	39255792	(C/T)
CakSNP5015	*Kabuli*	*Ca_Kabuli_Chr03*	39257226	(A/G)
CakSNP5016	*Kabuli*	*Ca_Kabuli_Chr03*	39258671	(A/C)
CakSNP5017	*Kabuli*	*Ca_Kabuli_Chr03*	39265412	(A/C)
CakSNP5018	*Kabuli*	*Ca_Kabuli_Chr03*	39289840	(T/A)
CakSNP5019	*Kabuli*	*Ca_Kabuli_Chr03*	39303386	(C/G)
CakSNP5020	*Kabuli*	*Ca_Kabuli_Chr03*	39303416	(G/T)
CakSNP5021	*Kabuli*	*Ca_Kabuli_Chr03*	39303407	(C/A)
CakSNP5022	*Kabuli*	*Ca_Kabuli_Chr03*	39323779	(A/G)
CakSNP5023	*Kabuli*	*Ca_Kabuli_Chr03*	39326387	(C/T)
CakSNP5024	*Kabuli*	*Ca_Kabuli_Chr03*	39405252	(C/A)
CakSNP5025	*Kabuli*	*Ca_Kabuli_Chr03*	39405873	(C/G)
CakSNP5026	*Kabuli*	*Ca_Kabuli_Chr03*	39472291	(G/C)
CakSNP5027	*Kabuli*	*Ca_Kabuli_Chr03*	39481371	(A/C)
CakSNP5028	*Kabuli*	*Ca_Kabuli_Chr03*	39485182	(G/A)
CakSNP5029	*Kabuli*	*Ca_Kabuli_Chr03*	39504242	(A/G)
CakSNP5030	*Kabuli*	*Ca_Kabuli_Chr03*	39515531	(A/G)
CakSNP5031	*Kabuli*	*Ca_Kabuli_Chr03*	39515710	(T/A)
CakSNP5032	*Kabuli*	*Ca_Kabuli_Chr03*	39519640	(A/G)
CakSNP5033	*Kabuli*	*Ca_Kabuli_Chr03*	39556535	(T/A)
CakSNP5034	*Kabuli*	*Ca_Kabuli_Chr03*	39587620	(T/G)
CakSNP5035	*Kabuli*	*Ca_Kabuli_Chr03*	39592340	(C/T)
CakSNP5036	*Kabuli*	*Ca_Kabuli_Chr03*	39612466	(T/C)
CakSNP5037	*Kabuli*	*Ca_Kabuli_Chr03*	39612458	(C/A)
CakSNP5038	*Kabuli*	*Ca_Kabuli_Chr03*	39612447	(C/A)
CakSNP5039	*Kabuli*	*Ca_Kabuli_Chr03*	39612568	(A/T)
CakSNP5040	*Kabuli*	*Ca_Kabuli_Chr03*	39628916	(G/A)
CakSNP5041	*Kabuli*	*Ca_Kabuli_Chr03*	39698060	(T/C)
CakSNP5042	*Kabuli*	*Ca_Kabuli_Chr03*	39701532	(G/A)

SNP IDs	Cultivars	Chromosomes/scaffolds	Physical positions (bp)	SNPs
CakSNP5043	*Kabuli*	*Ca_Kabuli_Chr03*	39722314	(C/T)
CakSNP5044	*Kabuli*	*Ca_Kabuli_Chr03*	39731437	(A/C)
CakSNP5045	*Kabuli*	*Ca_Kabuli_Chr03*	39787513	(A/C)
CakSNP5046	*Kabuli*	*Ca_Kabuli_Chr03*	39787615	(C/T)
CakSNP5047	*Kabuli*	*Ca_Kabuli_Chr03*	39787716	(A/C)
CakSNP5048	*Kabuli*	*Ca_Kabuli_Chr03*	39791717	(C/T)
CakSNP5049	*Kabuli*	*Ca_Kabuli_Chr03*	39793539	(G/A)
CakSNP5050	*Kabuli*	*Ca_Kabuli_Chr03*	39794672	(C/T)
CakSNP5051	*Kabuli*	*Ca_Kabuli_Chr03*	39794639	(C/G)
CakSNP5052	*Kabuli*	*Ca_Kabuli_Chr03*	39801213	(A/G)
CakSNP5053	*Kabuli*	*Ca_Kabuli_Chr03*	39801242	(C/T)
CakSNP5054	*Kabuli*	*Ca_Kabuli_Chr03*	39817122	(G/A)
CakSNP5055	*Kabuli*	*Ca_Kabuli_Chr03*	39839763	(T/G)
CakSNP5056	*Kabuli*	*Ca_Kabuli_Chr03*	39891075	(G/A)
CakSNP5057	*Kabuli*	*Ca_Kabuli_Chr03*	39890991	(A/T)
CakSNP5058	*Kabuli*	*Ca_Kabuli_Chr03*	39896395	(A/G)
CakSNP5059	*Kabuli*	*Ca_Kabuli_Chr03*	39908983	(C/G)
CakSNP5060	*Kabuli*	*Ca_Kabuli_Chr03*	39909824	(C/G)
CakSNP5061	*Kabuli*	*Ca_Kabuli_Chr03*	39909839	(T/C)
CakSNP5062	*Kabuli*	*Ca_Kabuli_Chr03*	39914344	(T/G)
CakSNP5063	*Kabuli*	*Ca_Kabuli_Chr03*	39941101	(T/G)
CakSNP5064	*Kabuli*	*Ca_Kabuli_Chr03*	39941090	(G/A)
CakSNP5065	*Kabuli*	*Ca_Kabuli_Chr04*	20297	(A/G)
CakSNP5066	*Kabuli*	*Ca_Kabuli_Chr04*	20495	(G/C)
CakSNP5067	*Kabuli*	*Ca_Kabuli_Chr04*	167229	(T/G)
CakSNP5068	*Kabuli*	*Ca_Kabuli_Chr04*	228864	(G/A)
CakSNP5069	*Kabuli*	*Ca_Kabuli_Chr04*	305558	(C/T)
CakSNP5070	*Kabuli*	*Ca_Kabuli_Chr04*	335941	(C/T)
CakSNP5071	*Kabuli*	*Ca_Kabuli_Chr04*	336055	(G/A)
CakSNP5072	*Kabuli*	*Ca_Kabuli_Chr04*	336050	(T/C)
CakSNP5073	*Kabuli*	*Ca_Kabuli_Chr04*	336029	(G/A)
CakSNP5074	*Kabuli*	*Ca_Kabuli_Chr04*	338641	(A/G)
CakSNP5075	*Kabuli*	*Ca_Kabuli_Chr04*	338721	(C/T)
CakSNP5076	*Kabuli*	*Ca_Kabuli_Chr04*	338707	(G/T)
CakSNP5077	*Kabuli*	*Ca_Kabuli_Chr04*	349309	(C/T)
CakSNP5078	*Kabuli*	*Ca_Kabuli_Chr04*	353069	(C/A)
CakSNP5079	*Kabuli*	*Ca_Kabuli_Chr04*	353079	(C/T)
CakSNP5080	*Kabuli*	*Ca_Kabuli_Chr04*	353119	(G/A)
CakSNP5081	*Kabuli*	*Ca_Kabuli_Chr04*	353120	(C/G)
CakSNP5082	*Kabuli*	*Ca_Kabuli_Chr04*	353124	(A/G)
CakSNP5083	*Kabuli*	*Ca_Kabuli_Chr04*	353126	(T/G)

SNP IDs	Cultivars	Chromosomes/scaffolds	Physical positions (bp)	SNPs
CakSNP5084	*Kabuli*	*Ca_Kabuli_Chr04*	369258	(C/T)
CakSNP5085	*Kabuli*	*Ca_Kabuli_Chr04*	394661	(C/G)
CakSNP5086	*Kabuli*	*Ca_Kabuli_Chr04*	411208	(A/C)
CakSNP5087	*Kabuli*	*Ca_Kabuli_Chr04*	412017	(C/A)
CakSNP5088	*Kabuli*	*Ca_Kabuli_Chr04*	455361	(A/G)
CakSNP5089	*Kabuli*	*Ca_Kabuli_Chr04*	459360	(T/C)
CakSNP5090	*Kabuli*	*Ca_Kabuli_Chr04*	480138	(C/A)
CakSNP5091	*Kabuli*	*Ca_Kabuli_Chr04*	481556	(A/G)
CakSNP5092	*Kabuli*	*Ca_Kabuli_Chr04*	481687	(T/G)
CakSNP5093	*Kabuli*	*Ca_Kabuli_Chr04*	504928	(C/A)
CakSNP5094	*Kabuli*	*Ca_Kabuli_Chr04*	586273	(A/G)
CakSNP5095	*Kabuli*	*Ca_Kabuli_Chr04*	586703	(T/C)
CakSNP5096	*Kabuli*	*Ca_Kabuli_Chr04*	589140	(T/C)
CakSNP5097	*Kabuli*	*Ca_Kabuli_Chr04*	589191	(A/G)
CakSNP5098	*Kabuli*	*Ca_Kabuli_Chr04*	589204	(G/A)
CakSNP5099	*Kabuli*	*Ca_Kabuli_Chr04*	589220	(A/C)
CakSNP5100	*Kabuli*	*Ca_Kabuli_Chr04*	589224	(T/C)
CakSNP5101	*Kabuli*	*Ca_Kabuli_Chr04*	595982	(C/G)
CakSNP5102	*Kabuli*	*Ca_Kabuli_Chr04*	613150	(G/A)
CakSNP5103	*Kabuli*	*Ca_Kabuli_Chr04*	613166	(A/G)
CakSNP5104	*Kabuli*	*Ca_Kabuli_Chr04*	645103	(T/A)
CakSNP5105	*Kabuli*	*Ca_Kabuli_Chr04*	645075	(T/A)
CakSNP5106	*Kabuli*	*Ca_Kabuli_Chr04*	646567	(C/T)
CakSNP5107	*Kabuli*	*Ca_Kabuli_Chr04*	646576	(A/G)
CakSNP5108	*Kabuli*	*Ca_Kabuli_Chr04*	651040	(C/T)
CakSNP5109	*Kabuli*	*Ca_Kabuli_Chr04*	660034	(A/C)
CakSNP5110	*Kabuli*	*Ca_Kabuli_Chr04*	660029	(T/G)
CakSNP5111	*Kabuli*	*Ca_Kabuli_Chr04*	703978	(A/T)
CakSNP5112	*Kabuli*	*Ca_Kabuli_Chr04*	703975	(G/T)
CakSNP5113	*Kabuli*	*Ca_Kabuli_Chr04*	704009	(G/A)
CakSNP5114	*Kabuli*	*Ca_Kabuli_Chr04*	705336	(T/G)
CakSNP5115	*Kabuli*	*Ca_Kabuli_Chr04*	783302	(C/T)
CakSNP5116	*Kabuli*	*Ca_Kabuli_Chr04*	783382	(G/T)
CakSNP5117	*Kabuli*	*Ca_Kabuli_Chr04*	827630	(T/C)
CakSNP5118	*Kabuli*	*Ca_Kabuli_Chr04*	848192	(G/A)
CakSNP5119	*Kabuli*	*Ca_Kabuli_Chr04*	913689	(A/G)
CakSNP5120	*Kabuli*	*Ca_Kabuli_Chr04*	951389	(T/G)
CakSNP5121	*Kabuli*	*Ca_Kabuli_Chr04*	951390	(G/A)
CakSNP5122	*Kabuli*	*Ca_Kabuli_Chr04*	1035328	(G/C)
CakSNP5123	*Kabuli*	*Ca_Kabuli_Chr04*	1047639	(G/T)
CakSNP5124	*Kabuli*	*Ca_Kabuli_Chr04*	1065107	(C/T)

SNP IDs	Cultivars	Chromosomes/scaffolds	Physical positions (bp)	SNPs
CakSNP5125	*Kabuli*	*Ca_Kabuli_Chr04*	1065245	(A/G)
CakSNP5126	*Kabuli*	*Ca_Kabuli_Chr04*	1189997	(C/T)
CakSNP5127	*Kabuli*	*Ca_Kabuli_Chr04*	1190007	(C/G)
CakSNP5128	*Kabuli*	*Ca_Kabuli_Chr04*	1190038	(G/T)
CakSNP5129	*Kabuli*	*Ca_Kabuli_Chr04*	1190208	(T/C)
CakSNP5130	*Kabuli*	*Ca_Kabuli_Chr04*	1190184	(A/C)
CakSNP5131	*Kabuli*	*Ca_Kabuli_Chr04*	1190158	(T/G)
CakSNP5132	*Kabuli*	*Ca_Kabuli_Chr04*	1259917	(G/T)
CakSNP5133	*Kabuli*	*Ca_Kabuli_Chr04*	1259915	(T/G)
CakSNP5134	*Kabuli*	*Ca_Kabuli_Chr04*	1264361	(T/C)
CakSNP5135	*Kabuli*	*Ca_Kabuli_Chr04*	1266175	(A/G)
CakSNP5136	*Kabuli*	*Ca_Kabuli_Chr04*	1266174	(C/T)
CakSNP5137	*Kabuli*	*Ca_Kabuli_Chr04*	1283369	(A/G)
CakSNP5138	*Kabuli*	*Ca_Kabuli_Chr04*	1344151	(T/C)
CakSNP5139	*Kabuli*	*Ca_Kabuli_Chr04*	1396281	(G/C)
CakSNP5140	*Kabuli*	*Ca_Kabuli_Chr04*	1396425	(C/A)
CakSNP5141	*Kabuli*	*Ca_Kabuli_Chr04*	1474771	(T/C)
CakSNP5142	*Kabuli*	*Ca_Kabuli_Chr04*	1474898	(A/G)
CakSNP5143	*Kabuli*	*Ca_Kabuli_Chr04*	1474922	(G/T)
CakSNP5144	*Kabuli*	*Ca_Kabuli_Chr04*	1493927	(T/C)
CakSNP5145	*Kabuli*	*Ca_Kabuli_Chr04*	1513664	(T/A)
CakSNP5146	*Kabuli*	*Ca_Kabuli_Chr04*	1526270	(T/C)
CakSNP5147	*Kabuli*	*Ca_Kabuli_Chr04*	1526731	(T/G)
CakSNP5148	*Kabuli*	*Ca_Kabuli_Chr04*	1613153	(G/A)
CakSNP5149	*Kabuli*	*Ca_Kabuli_Chr04*	1613193	(G/A)
CakSNP5150	*Kabuli*	*Ca_Kabuli_Chr04*	1613303	(G/A)
CakSNP5151	*Kabuli*	*Ca_Kabuli_Chr04*	1687346	(A/G)
CakSNP5152	*Kabuli*	*Ca_Kabuli_Chr04*	1691555	(T/G)
CakSNP5153	*Kabuli*	*Ca_Kabuli_Chr04*	1708370	(C/T)
CakSNP5154	*Kabuli*	*Ca_Kabuli_Chr04*	1711864	(A/C)
CakSNP5155	*Kabuli*	*Ca_Kabuli_Chr04*	1711865	(G/A)
CakSNP5156	*Kabuli*	*Ca_Kabuli_Chr04*	1720544	(A/C)
CakSNP5157	*Kabuli*	*Ca_Kabuli_Chr04*	1720658	(A/G)
CakSNP5158	*Kabuli*	*Ca_Kabuli_Chr04*	1720642	(A/G)
CakSNP5159	*Kabuli*	*Ca_Kabuli_Chr04*	1720619	(A/C)
CakSNP5160	*Kabuli*	*Ca_Kabuli_Chr04*	1742468	(C/T)
CakSNP5161	*Kabuli*	*Ca_Kabuli_Chr04*	1747181	(A/T)
CakSNP5162	*Kabuli*	*Ca_Kabuli_Chr04*	1754555	(C/T)
CakSNP5163	*Kabuli*	*Ca_Kabuli_Chr04*	1755066	(G/C)
CakSNP5164	*Kabuli*	*Ca_Kabuli_Chr04*	1773886	(C/G)
CakSNP5165	*Kabuli*	*Ca_Kabuli_Chr04*	1773939	(C/T)

SNP IDs	Cultivars	Chromosomes/scaffolds	Physical positions (bp)	SNPs
CakSNP5166	*Kabuli*	*Ca_Kabuli_Chr04*	1773951	(C/G)
CakSNP5167	*Kabuli*	*Ca_Kabuli_Chr04*	1785705	(G/C)
CakSNP5168	*Kabuli*	*Ca_Kabuli_Chr04*	1785680	(C/T)
CakSNP5169	*Kabuli*	*Ca_Kabuli_Chr04*	1807484	(G/A)
CakSNP5170	*Kabuli*	*Ca_Kabuli_Chr04*	1817779	(G/C)
CakSNP5171	*Kabuli*	*Ca_Kabuli_Chr04*	1833570	(T/C)
CakSNP5172	*Kabuli*	*Ca_Kabuli_Chr04*	1833678	(A/G)
CakSNP5173	*Kabuli*	*Ca_Kabuli_Chr04*	1833725	(C/T)
CakSNP5174	*Kabuli*	*Ca_Kabuli_Chr04*	1845301	(G/A)
CakSNP5175	*Kabuli*	*Ca_Kabuli_Chr04*	1867921	(G/A)
CakSNP5176	*Kabuli*	*Ca_Kabuli_Chr04*	1869577	(C/T)
CakSNP5177	*Kabuli*	*Ca_Kabuli_Chr04*	1870333	(A/G)
CakSNP5178	*Kabuli*	*Ca_Kabuli_Chr04*	1870317	(T/C)
CakSNP5179	*Kabuli*	*Ca_Kabuli_Chr04*	1870666	(G/C)
CakSNP5180	*Kabuli*	*Ca_Kabuli_Chr04*	1870806	(C/A)
CakSNP5181	*Kabuli*	*Ca_Kabuli_Chr04*	1870802	(C/T)
CakSNP5182	*Kabuli*	*Ca_Kabuli_Chr04*	1873733	(T/C)
CakSNP5183	*Kabuli*	*Ca_Kabuli_Chr04*	1890220	(G/C)
CakSNP5184	*Kabuli*	*Ca_Kabuli_Chr04*	1941001	(T/G)
CakSNP5185	*Kabuli*	*Ca_Kabuli_Chr04*	1941089	(C/T)
CakSNP5186	*Kabuli*	*Ca_Kabuli_Chr04*	1970471	(A/C)
CakSNP5187	*Kabuli*	*Ca_Kabuli_Chr04*	1973375	(A/C)
CakSNP5188	*Kabuli*	*Ca_Kabuli_Chr04*	2009707	(A/G)
CakSNP5189	*Kabuli*	*Ca_Kabuli_Chr04*	2025214	(A/C)
CakSNP5190	*Kabuli*	*Ca_Kabuli_Chr04*	2027063	(G/A)
CakSNP5191	*Kabuli*	*Ca_Kabuli_Chr04*	2027101	(T/A)
CakSNP5192	*Kabuli*	*Ca_Kabuli_Chr04*	2034429	(C/A)
CakSNP5193	*Kabuli*	*Ca_Kabuli_Chr04*	2034398	(C/T)
CakSNP5194	*Kabuli*	*Ca_Kabuli_Chr04*	2034396	(A/G)
CakSNP5195	*Kabuli*	*Ca_Kabuli_Chr04*	2034739	(A/C)
CakSNP5196	*Kabuli*	*Ca_Kabuli_Chr04*	2035797	(G/A)
CakSNP5197	*Kabuli*	*Ca_Kabuli_Chr04*	2035801	(G/A)
CakSNP5198	*Kabuli*	*Ca_Kabuli_Chr04*	2035841	(C/T)
CakSNP5199	*Kabuli*	*Ca_Kabuli_Chr04*	2035894	(C/A)
CakSNP5200	*Kabuli*	*Ca_Kabuli_Chr04*	2051684	(T/C)
CakSNP5201	*Kabuli*	*Ca_Kabuli_Chr04*	2051642	(G/C)
CakSNP5202	*Kabuli*	*Ca_Kabuli_Chr04*	2060244	(G/A)
CakSNP5203	*Kabuli*	*Ca_Kabuli_Chr04*	2060262	(C/T)
CakSNP5204	*Kabuli*	*Ca_Kabuli_Chr04*	2113564	(A/G)
CakSNP5205	*Kabuli*	*Ca_Kabuli_Chr04*	2125774	(G/A)
CakSNP5206	*Kabuli*	*Ca_Kabuli_Chr04*	2143155	(A/G)

**SNP IDs**	**Cultivars**	**Chromosomes/scaffolds**	**Physical positions (bp)**	**SNPs**
CakSNP5207	*Kabuli*	*Ca_Kabuli_Chr04*	2213710	(G/A)
CakSNP5208	*Kabuli*	*Ca_Kabuli_Chr04*	2238480	(A/T)
CakSNP5209	*Kabuli*	*Ca_Kabuli_Chr04*	2270360	(T/C)
CakSNP5210	*Kabuli*	*Ca_Kabuli_Chr04*	2277421	(A/T)
CakSNP5211	*Kabuli*	*Ca_Kabuli_Chr04*	2311977	(A/G)
CakSNP5212	*Kabuli*	*Ca_Kabuli_Chr04*	2313969	(G/A)
CakSNP5213	*Kabuli*	*Ca_Kabuli_Chr04*	2361088	(T/A)
CakSNP5214	*Kabuli*	*Ca_Kabuli_Chr04*	2380206	(A/G)
CakSNP5215	*Kabuli*	*Ca_Kabuli_Chr04*	2393033	(T/G)
CakSNP5216	*Kabuli*	*Ca_Kabuli_Chr04*	2396883	(A/G)
CakSNP5217	*Kabuli*	*Ca_Kabuli_Chr04*	2411925	(G/T)
CakSNP5218	*Kabuli*	*Ca_Kabuli_Chr04*	2422087	(A/G)
CakSNP5219	*Kabuli*	*Ca_Kabuli_Chr04*	2422092	(G/A)
CakSNP5220	*Kabuli*	*Ca_Kabuli_Chr04*	2422949	(C/T)
CakSNP5221	*Kabuli*	*Ca_Kabuli_Chr04*	2463064	(A/G)
CakSNP5222	*Kabuli*	*Ca_Kabuli_Chr04*	2463038	(T/C)
CakSNP5223	*Kabuli*	*Ca_Kabuli_Chr04*	2492726	(A/G)
CakSNP5224	*Kabuli*	*Ca_Kabuli_Chr04*	2493195	(T/C)
CakSNP5225	*Kabuli*	*Ca_Kabuli_Chr04*	2494101	(C/A)
CakSNP5226	*Kabuli*	*Ca_Kabuli_Chr04*	2494140	(C/A)
CakSNP5227	*Kabuli*	*Ca_Kabuli_Chr04*	2499257	(A/C)
CakSNP5228	*Kabuli*	*Ca_Kabuli_Chr04*	2562541	(G/C)
CakSNP5229	*Kabuli*	*Ca_Kabuli_Chr04*	2564500	(T/A)
CakSNP5230	*Kabuli*	*Ca_Kabuli_Chr04*	2609945	(C/T)
CakSNP5231	*Kabuli*	*Ca_Kabuli_Chr04*	2676174	(C/T)
CakSNP5232	*Kabuli*	*Ca_Kabuli_Chr04*	2740041	(T/G)
CakSNP5233	*Kabuli*	*Ca_Kabuli_Chr04*	2740068	(A/T)
CakSNP5234	*Kabuli*	*Ca_Kabuli_Chr04*	2740065	(A/G)
CakSNP5235	*Kabuli*	*Ca_Kabuli_Chr04*	2752538	(G/A)
CakSNP5236	*Kabuli*	*Ca_Kabuli_Chr04*	2752568	(C/T)
CakSNP5237	*Kabuli*	*Ca_Kabuli_Chr04*	2886775	(T/G)
CakSNP5238	*Kabuli*	*Ca_Kabuli_Chr04*	2886863	(C/T)
CakSNP5239	*Kabuli*	*Ca_Kabuli_Chr04*	2978875	(T/C)
CakSNP5240	*Kabuli*	*Ca_Kabuli_Chr04*	2978876	(T/G)
CakSNP5241	*Kabuli*	*Ca_Kabuli_Chr04*	2978882	(G/T)
CakSNP5242	*Kabuli*	*Ca_Kabuli_Chr04*	2978932	(A/C)
CakSNP5243	*Kabuli*	*Ca_Kabuli_Chr04*	2978931	(A/T)
CakSNP5244	*Kabuli*	*Ca_Kabuli_Chr04*	3002755	(A/G)
CakSNP5245	*Kabuli*	*Ca_Kabuli_Chr04*	3002810	(T/C)
CakSNP5246	*Kabuli*	*Ca_Kabuli_Chr04*	3037899	(A/C)
CakSNP5247	*Kabuli*	*Ca_Kabuli_Chr04*	3037986	(T/A)

SNP IDs	Cultivars	Chromosomes/scaffolds	Physical positions (bp)	SNPs
CakSNP5248	*Kabuli*	*Ca_Kabuli_Chr04*	3037929	(T/G)
CakSNP5249	*Kabuli*	*Ca_Kabuli_Chr04*	3038022	(C/T)
CakSNP5250	*Kabuli*	*Ca_Kabuli_Chr04*	3046920	(A/G)
CakSNP5251	*Kabuli*	*Ca_Kabuli_Chr04*	3144454	(T/C)
CakSNP5252	*Kabuli*	*Ca_Kabuli_Chr04*	3158321	(A/G)
CakSNP5253	*Kabuli*	*Ca_Kabuli_Chr04*	3158323	(T/G)
CakSNP5254	*Kabuli*	*Ca_Kabuli_Chr04*	3158328	(G/T)
CakSNP5255	*Kabuli*	*Ca_Kabuli_Chr04*	3158331	(A/T)
CakSNP5256	*Kabuli*	*Ca_Kabuli_Chr04*	3158426	(G/A)
CakSNP5257	*Kabuli*	*Ca_Kabuli_Chr04*	3158504	(A/T)
CakSNP5258	*Kabuli*	*Ca_Kabuli_Chr04*	3158506	(T/C)
CakSNP5259	*Kabuli*	*Ca_Kabuli_Chr04*	3158549	(A/G)
CakSNP5260	*Kabuli*	*Ca_Kabuli_Chr04*	3164115	(G/A)
CakSNP5261	*Kabuli*	*Ca_Kabuli_Chr04*	3164083	(G/A)
CakSNP5262	*Kabuli*	*Ca_Kabuli_Chr04*	3166657	(C/T)
CakSNP5263	*Kabuli*	*Ca_Kabuli_Chr04*	3166663	(C/T)
CakSNP5264	*Kabuli*	*Ca_Kabuli_Chr04*	3166674	(G/T)
CakSNP5265	*Kabuli*	*Ca_Kabuli_Chr04*	3171162	(G/A)
CakSNP5266	*Kabuli*	*Ca_Kabuli_Chr04*	3171384	(T/G)
CakSNP5267	*Kabuli*	*Ca_Kabuli_Chr04*	3171385	(A/G)
CakSNP5268	*Kabuli*	*Ca_Kabuli_Chr04*	3171428	(T/C)
CakSNP5269	*Kabuli*	*Ca_Kabuli_Chr04*	3171551	(C/T)
CakSNP5270	*Kabuli*	*Ca_Kabuli_Chr04*	3264351	(T/C)
CakSNP5271	*Kabuli*	*Ca_Kabuli_Chr04*	3280147	(G/A)
CakSNP5272	*Kabuli*	*Ca_Kabuli_Chr04*	3280217	(A/G)
CakSNP5273	*Kabuli*	*Ca_Kabuli_Chr04*	3281050	(T/G)
CakSNP5274	*Kabuli*	*Ca_Kabuli_Chr04*	3290298	(T/G)
CakSNP5275	*Kabuli*	*Ca_Kabuli_Chr04*	3353106	(T/C)
CakSNP5276	*Kabuli*	*Ca_Kabuli_Chr04*	3356252	(C/G)
CakSNP5277	*Kabuli*	*Ca_Kabuli_Chr04*	3356242	(C/G)
CakSNP5278	*Kabuli*	*Ca_Kabuli_Chr04*	3400828	(G/C)
CakSNP5279	*Kabuli*	*Ca_Kabuli_Chr04*	3427396	(A/G)
CakSNP5280	*Kabuli*	*Ca_Kabuli_Chr04*	3429035	(G/A)
CakSNP5281	*Kabuli*	*Ca_Kabuli_Chr04*	3429059	(C/A)
CakSNP5282	*Kabuli*	*Ca_Kabuli_Chr04*	3455029	(T/C)
CakSNP5283	*Kabuli*	*Ca_Kabuli_Chr04*	3455047	(G/A)
CakSNP5284	*Kabuli*	*Ca_Kabuli_Chr04*	3456450	(G/T)
CakSNP5285	*Kabuli*	*Ca_Kabuli_Chr04*	3456501	(C/T)
CakSNP5286	*Kabuli*	*Ca_Kabuli_Chr04*	3483687	(A/C)
CakSNP5287	*Kabuli*	*Ca_Kabuli_Chr04*	3627810	(G/T)
CakSNP5288	*Kabuli*	*Ca_Kabuli_Chr04*	3642051	(A/T)

**SNP IDs**	**Cultivars**	**Chromosomes/scaffolds**	**Physical positions (bp)**	**SNPs**
CakSNP5289	*Kabuli*	*Ca_Kabuli_Chr04*	3642070	(T/C)
CakSNP5290	*Kabuli*	*Ca_Kabuli_Chr04*	3642116	(A/T)
CakSNP5291	*Kabuli*	*Ca_Kabuli_Chr04*	3689840	(T/C)
CakSNP5292	*Kabuli*	*Ca_Kabuli_Chr04*	3689871	(G/A)
CakSNP5293	*Kabuli*	*Ca_Kabuli_Chr04*	3739386	(C/A)
CakSNP5294	*Kabuli*	*Ca_Kabuli_Chr04*	3741522	(T/G)
CakSNP5295	*Kabuli*	*Ca_Kabuli_Chr04*	3741539	(T/G)
CakSNP5296	*Kabuli*	*Ca_Kabuli_Chr04*	3752141	(C/G)
CakSNP5297	*Kabuli*	*Ca_Kabuli_Chr04*	3762559	(T/C)
CakSNP5298	*Kabuli*	*Ca_Kabuli_Chr04*	3762561	(C/A)
CakSNP5299	*Kabuli*	*Ca_Kabuli_Chr04*	3762585	(G/T)
CakSNP5300	*Kabuli*	*Ca_Kabuli_Chr04*	3794764	(G/A)
CakSNP5301	*Kabuli*	*Ca_Kabuli_Chr04*	3794885	(C/A)
CakSNP5302	*Kabuli*	*Ca_Kabuli_Chr04*	3885444	(G/C)
CakSNP5303	*Kabuli*	*Ca_Kabuli_Chr04*	3888010	(G/C)
CakSNP5304	*Kabuli*	*Ca_Kabuli_Chr04*	3888053	(C/A)
CakSNP5305	*Kabuli*	*Ca_Kabuli_Chr04*	3888164	(T/A)
CakSNP5306	*Kabuli*	*Ca_Kabuli_Chr04*	3888185	(A/G)
CakSNP5307	*Kabuli*	*Ca_Kabuli_Chr04*	3888236	(A/G)
CakSNP5308	*Kabuli*	*Ca_Kabuli_Chr04*	3888328	(C/T)
CakSNP5309	*Kabuli*	*Ca_Kabuli_Chr04*	3888462	(T/C)
CakSNP5310	*Kabuli*	*Ca_Kabuli_Chr04*	3888510	(G/A)
CakSNP5311	*Kabuli*	*Ca_Kabuli_Chr04*	3888514	(G/A)
CakSNP5312	*Kabuli*	*Ca_Kabuli_Chr04*	3888529	(T/G)
CakSNP5313	*Kabuli*	*Ca_Kabuli_Chr04*	3890556	(A/G)
CakSNP5314	*Kabuli*	*Ca_Kabuli_Chr04*	3890709	(A/G)
CakSNP5315	*Kabuli*	*Ca_Kabuli_Chr04*	3990810	(C/G)
CakSNP5316	*Kabuli*	*Ca_Kabuli_Chr04*	3990809	(G/A)
CakSNP5317	*Kabuli*	*Ca_Kabuli_Chr04*	4002917	(T/C)
CakSNP5318	*Kabuli*	*Ca_Kabuli_Chr04*	4006213	(A/C)
CakSNP5319	*Kabuli*	*Ca_Kabuli_Chr04*	4027829	(A/T)
CakSNP5320	*Kabuli*	*Ca_Kabuli_Chr04*	4027872	(A/C)
CakSNP5321	*Kabuli*	*Ca_Kabuli_Chr04*	4097989	(G/A)
CakSNP5322	*Kabuli*	*Ca_Kabuli_Chr04*	4097935	(T/C)
CakSNP5323	*Kabuli*	*Ca_Kabuli_Chr04*	4116386	(G/C)
CakSNP5324	*Kabuli*	*Ca_Kabuli_Chr04*	4148061	(T/C)
CakSNP5325	*Kabuli*	*Ca_Kabuli_Chr04*	4148250	(A/G)
CakSNP5326	*Kabuli*	*Ca_Kabuli_Chr04*	4154417	(A/C)
CakSNP5327	*Kabuli*	*Ca_Kabuli_Chr04*	4154507	(G/T)
CakSNP5328	*Kabuli*	*Ca_Kabuli_Chr04*	4229295	(T/A)
CakSNP5329	*Kabuli*	*Ca_Kabuli_Chr04*	4229345	(C/T)

**SNP IDs**	**Cultivars**	**Chromosomes/scaffolds**	**Physical positions (bp)**	**SNPs**
CakSNP5330	*Kabuli*	*Ca_Kabuli_Chr04*	4229381	(T/C)
CakSNP5331	*Kabuli*	*Ca_Kabuli_Chr04*	4229371	(C/T)
CakSNP5332	*Kabuli*	*Ca_Kabuli_Chr04*	4255863	(T/G)
CakSNP5333	*Kabuli*	*Ca_Kabuli_Chr04*	4256023	(C/T)
CakSNP5334	*Kabuli*	*Ca_Kabuli_Chr04*	4258002	(A/G)
CakSNP5335	*Kabuli*	*Ca_Kabuli_Chr04*	4258149	(A/G)
CakSNP5336	*Kabuli*	*Ca_Kabuli_Chr04*	4305891	(A/C)
CakSNP5337	*Kabuli*	*Ca_Kabuli_Chr04*	4320615	(C/T)
CakSNP5338	*Kabuli*	*Ca_Kabuli_Chr04*	4320743	(A/G)
CakSNP5339	*Kabuli*	*Ca_Kabuli_Chr04*	4327268	(A/G)
CakSNP5340	*Kabuli*	*Ca_Kabuli_Chr04*	4334307	(T/G)
CakSNP5341	*Kabuli*	*Ca_Kabuli_Chr04*	4343586	(C/T)
CakSNP5342	*Kabuli*	*Ca_Kabuli_Chr04*	4343540	(C/A)
CakSNP5343	*Kabuli*	*Ca_Kabuli_Chr04*	4410351	(T/C)
CakSNP5344	*Kabuli*	*Ca_Kabuli_Chr04*	4426659	(C/T)
CakSNP5345	*Kabuli*	*Ca_Kabuli_Chr04*	4426760	(T/C)
CakSNP5346	*Kabuli*	*Ca_Kabuli_Chr04*	4435621	(T/C)
CakSNP5347	*Kabuli*	*Ca_Kabuli_Chr04*	4451928	(T/C)
CakSNP5348	*Kabuli*	*Ca_Kabuli_Chr04*	4452018	(T/A)
CakSNP5349	*Kabuli*	*Ca_Kabuli_Chr04*	4452380	(C/G)
CakSNP5350	*Kabuli*	*Ca_Kabuli_Chr04*	4454305	(A/C)
CakSNP5351	*Kabuli*	*Ca_Kabuli_Chr04*	4454464	(A/G)
CakSNP5352	*Kabuli*	*Ca_Kabuli_Chr04*	4461562	(G/C)
CakSNP5353	*Kabuli*	*Ca_Kabuli_Chr04*	4468394	(C/T)
CakSNP5354	*Kabuli*	*Ca_Kabuli_Chr04*	4536310	(A/G)
CakSNP5355	*Kabuli*	*Ca_Kabuli_Chr04*	4536286	(G/C)
CakSNP5356	*Kabuli*	*Ca_Kabuli_Chr04*	4578836	(C/T)
CakSNP5357	*Kabuli*	*Ca_Kabuli_Chr04*	4579274	(C/T)
CakSNP5358	*Kabuli*	*Ca_Kabuli_Chr04*	4605340	(A/C)
CakSNP5359	*Kabuli*	*Ca_Kabuli_Chr04*	4622932	(T/G)
CakSNP5360	*Kabuli*	*Ca_Kabuli_Chr04*	4622913	(C/T)
CakSNP5361	*Kabuli*	*Ca_Kabuli_Chr04*	4628221	(T/G)
CakSNP5362	*Kabuli*	*Ca_Kabuli_Chr04*	4628232	(G/A)
CakSNP5363	*Kabuli*	*Ca_Kabuli_Chr04*	4628240	(C/T)
CakSNP5364	*Kabuli*	*Ca_Kabuli_Chr04*	4628243	(G/A)
CakSNP5365	*Kabuli*	*Ca_Kabuli_Chr04*	4628309	(C/T)
CakSNP5366	*Kabuli*	*Ca_Kabuli_Chr04*	4630407	(G/A)
CakSNP5367	*Kabuli*	*Ca_Kabuli_Chr04*	4630419	(A/C)
CakSNP5368	*Kabuli*	*Ca_Kabuli_Chr04*	4630514	(T/C)
CakSNP5369	*Kabuli*	*Ca_Kabuli_Chr04*	4633913	(T/G)
CakSNP5370	*Kabuli*	*Ca_Kabuli_Chr04*	4634542	(A/T)

SNP IDs	Cultivars	Chromosomes/scaffolds	Physical positions (bp)	SNPs
CakSNP5371	*Kabuli*	*Ca_Kabuli_Chr04*	4660220	(A/G)
CakSNP5372	*Kabuli*	*Ca_Kabuli_Chr04*	4670913	(A/G)
CakSNP5373	*Kabuli*	*Ca_Kabuli_Chr04*	4670975	(C/A)
CakSNP5374	*Kabuli*	*Ca_Kabuli_Chr04*	4675182	(T/G)
CakSNP5375	*Kabuli*	*Ca_Kabuli_Chr04*	4675177	(T/A)
CakSNP5376	*Kabuli*	*Ca_Kabuli_Chr04*	4675176	(T/G)
CakSNP5377	*Kabuli*	*Ca_Kabuli_Chr04*	4675172	(T/A)
CakSNP5378	*Kabuli*	*Ca_Kabuli_Chr04*	4675171	(T/A)
CakSNP5379	*Kabuli*	*Ca_Kabuli_Chr04*	4675166	(A/T)
CakSNP5380	*Kabuli*	*Ca_Kabuli_Chr04*	4679236	(A/G)
CakSNP5381	*Kabuli*	*Ca_Kabuli_Chr04*	4707682	(A/G)
CakSNP5382	*Kabuli*	*Ca_Kabuli_Chr04*	4722348	(G/A)
CakSNP5383	*Kabuli*	*Ca_Kabuli_Chr04*	4757357	(T/C)
CakSNP5384	*Kabuli*	*Ca_Kabuli_Chr04*	4757416	(G/A)
CakSNP5385	*Kabuli*	*Ca_Kabuli_Chr04*	4757498	(C/G)
CakSNP5386	*Kabuli*	*Ca_Kabuli_Chr04*	4794122	(G/A)
CakSNP5387	*Kabuli*	*Ca_Kabuli_Chr04*	4794192	(G/C)
CakSNP5388	*Kabuli*	*Ca_Kabuli_Chr04*	4799122	(C/T)
CakSNP5389	*Kabuli*	*Ca_Kabuli_Chr04*	4799116	(C/T)
CakSNP5390	*Kabuli*	*Ca_Kabuli_Chr04*	4803168	(T/A)
CakSNP5391	*Kabuli*	*Ca_Kabuli_Chr04*	4803213	(C/T)
CakSNP5392	*Kabuli*	*Ca_Kabuli_Chr04*	4874780	(A/T)
CakSNP5393	*Kabuli*	*Ca_Kabuli_Chr04*	4874782	(C/A)
CakSNP5394	*Kabuli*	*Ca_Kabuli_Chr04*	4874836	(C/T)
CakSNP5395	*Kabuli*	*Ca_Kabuli_Chr04*	4882813	(C/G)
CakSNP5396	*Kabuli*	*Ca_Kabuli_Chr04*	4897934	(C/G)
CakSNP5397	*Kabuli*	*Ca_Kabuli_Chr04*	4907987	(G/A)
CakSNP5398	*Kabuli*	*Ca_Kabuli_Chr04*	4911083	(T/C)
CakSNP5399	*Kabuli*	*Ca_Kabuli_Chr04*	4911130	(T/C)
CakSNP5400	*Kabuli*	*Ca_Kabuli_Chr04*	4911209	(A/G)
CakSNP5401	*Kabuli*	*Ca_Kabuli_Chr04*	4911217	(A/C)
CakSNP5402	*Kabuli*	*Ca_Kabuli_Chr04*	4920706	(T/C)
CakSNP5403	*Kabuli*	*Ca_Kabuli_Chr04*	4920712	(A/T)
CakSNP5404	*Kabuli*	*Ca_Kabuli_Chr04*	4920719	(A/G)
CakSNP5405	*Kabuli*	*Ca_Kabuli_Chr04*	4922290	(A/C)
CakSNP5406	*Kabuli*	*Ca_Kabuli_Chr04*	4928764	(T/C)
CakSNP5407	*Kabuli*	*Ca_Kabuli_Chr04*	4928890	(A/G)
CakSNP5408	*Kabuli*	*Ca_Kabuli_Chr04*	4929387	(T/C)
CakSNP5409	*Kabuli*	*Ca_Kabuli_Chr04*	4953629	(C/A)
CakSNP5410	*Kabuli*	*Ca_Kabuli_Chr04*	4967336	(T/C)
CakSNP5411	*Kabuli*	*Ca_Kabuli_Chr04*	4967342	(G/A)

SNP IDs	Cultivars	Chromosomes/scaffolds	Physical positions (bp)	SNPs
CakSNP5412	*Kabuli*	*Ca_Kabuli_Chr04*	4967347	(A/G)
CakSNP5413	*Kabuli*	*Ca_Kabuli_Chr04*	5018591	(G/A)
CakSNP5414	*Kabuli*	*Ca_Kabuli_Chr04*	5038300	(G/C)
CakSNP5415	*Kabuli*	*Ca_Kabuli_Chr04*	5051617	(A/G)
CakSNP5416	*Kabuli*	*Ca_Kabuli_Chr04*	5051574	(T/G)
CakSNP5417	*Kabuli*	*Ca_Kabuli_Chr04*	5051573	(A/C)
CakSNP5418	*Kabuli*	*Ca_Kabuli_Chr04*	5086705	(T/C)
CakSNP5419	*Kabuli*	*Ca_Kabuli_Chr04*	5120207	(C/T)
CakSNP5420	*Kabuli*	*Ca_Kabuli_Chr04*	5186401	(A/G)
CakSNP5421	*Kabuli*	*Ca_Kabuli_Chr04*	5187402	(G/C)
CakSNP5422	*Kabuli*	*Ca_Kabuli_Chr04*	5187344	(C/G)
CakSNP5423	*Kabuli*	*Ca_Kabuli_Chr04*	5188084	(G/A)
CakSNP5424	*Kabuli*	*Ca_Kabuli_Chr04*	5188040	(A/G)
CakSNP5425	*Kabuli*	*Ca_Kabuli_Chr04*	5197824	(T/C)
CakSNP5426	*Kabuli*	*Ca_Kabuli_Chr04*	5199907	(C/T)
CakSNP5427	*Kabuli*	*Ca_Kabuli_Chr04*	5222669	(A/C)
CakSNP5428	*Kabuli*	*Ca_Kabuli_Chr04*	5222805	(G/C)
CakSNP5429	*Kabuli*	*Ca_Kabuli_Chr04*	5222851	(C/T)
CakSNP5430	*Kabuli*	*Ca_Kabuli_Chr04*	5223100	(G/A)
CakSNP5431	*Kabuli*	*Ca_Kabuli_Chr04*	5223206	(A/T)
CakSNP5432	*Kabuli*	*Ca_Kabuli_Chr04*	5228950	(G/T)
CakSNP5433	*Kabuli*	*Ca_Kabuli_Chr04*	5232364	(T/C)
CakSNP5434	*Kabuli*	*Ca_Kabuli_Chr04*	5263199	(A/G)
CakSNP5435	*Kabuli*	*Ca_Kabuli_Chr04*	5268995	(A/C)
CakSNP5436	*Kabuli*	*Ca_Kabuli_Chr04*	5282131	(T/C)
CakSNP5437	*Kabuli*	*Ca_Kabuli_Chr04*	5310873	(A/G)
CakSNP5438	*Kabuli*	*Ca_Kabuli_Chr04*	5345520	(T/G)
CakSNP5439	*Kabuli*	*Ca_Kabuli_Chr04*	5377067	(A/C)
CakSNP5440	*Kabuli*	*Ca_Kabuli_Chr04*	5377400	(C/T)
CakSNP5441	*Kabuli*	*Ca_Kabuli_Chr04*	5390236	(C/A)
CakSNP5442	*Kabuli*	*Ca_Kabuli_Chr04*	5390317	(G/A)
CakSNP5443	*Kabuli*	*Ca_Kabuli_Chr04*	5453008	(A/C)
CakSNP5444	*Kabuli*	*Ca_Kabuli_Chr04*	5473174	(C/T)
CakSNP5445	*Kabuli*	*Ca_Kabuli_Chr04*	5484259	(G/T)
CakSNP5446	*Kabuli*	*Ca_Kabuli_Chr04*	5484257	(G/C)
CakSNP5447	*Kabuli*	*Ca_Kabuli_Chr04*	5484238	(G/A)
CakSNP5448	*Kabuli*	*Ca_Kabuli_Chr04*	5484236	(C/T)
CakSNP5449	*Kabuli*	*Ca_Kabuli_Chr04*	5484220	(A/G)
CakSNP5450	*Kabuli*	*Ca_Kabuli_Chr04*	5485052	(G/T)
CakSNP5451	*Kabuli*	*Ca_Kabuli_Chr04*	5485050	(G/C)
CakSNP5452	*Kabuli*	*Ca_Kabuli_Chr04*	5485031	(G/A)

SNP IDs	Cultivars	Chromosomes/scaffolds	Physical positions (bp)	SNPs
CakSNP5453	*Kabuli*	*Ca_Kabuli_Chr04*	5485029	(C/T)
CakSNP5454	*Kabuli*	*Ca_Kabuli_Chr04*	5485013	(A/G)
CakSNP5455	*Kabuli*	*Ca_Kabuli_Chr04*	5528421	(T/G)
CakSNP5456	*Kabuli*	*Ca_Kabuli_Chr04*	5530612	(A/G)
CakSNP5457	*Kabuli*	*Ca_Kabuli_Chr04*	5535661	(A/G)
CakSNP5458	*Kabuli*	*Ca_Kabuli_Chr04*	5575846	(G/C)
CakSNP5459	*Kabuli*	*Ca_Kabuli_Chr04*	5595079	(T/C)
CakSNP5460	*Kabuli*	*Ca_Kabuli_Chr04*	5595077	(T/A)
CakSNP5461	*Kabuli*	*Ca_Kabuli_Chr04*	5623571	(A/G)
CakSNP5462	*Kabuli*	*Ca_Kabuli_Chr04*	5627096	(C/T)
CakSNP5463	*Kabuli*	*Ca_Kabuli_Chr04*	5701873	(A/C)
CakSNP5464	*Kabuli*	*Ca_Kabuli_Chr04*	5701900	(G/T)
CakSNP5465	*Kabuli*	*Ca_Kabuli_Chr04*	5701902	(C/T)
CakSNP5466	*Kabuli*	*Ca_Kabuli_Chr04*	5701907	(G/T)
CakSNP5467	*Kabuli*	*Ca_Kabuli_Chr04*	5779698	(A/G)
CakSNP5468	*Kabuli*	*Ca_Kabuli_Chr04*	5779765	(A/T)
CakSNP5469	*Kabuli*	*Ca_Kabuli_Chr04*	5787727	(G/A)
CakSNP5470	*Kabuli*	*Ca_Kabuli_Chr04*	5787722	(G/A)
CakSNP5471	*Kabuli*	*Ca_Kabuli_Chr04*	5841444	(C/G)
CakSNP5472	*Kabuli*	*Ca_Kabuli_Chr04*	5841462	(A/C)
CakSNP5473	*Kabuli*	*Ca_Kabuli_Chr04*	5853789	(C/T)
CakSNP5474	*Kabuli*	*Ca_Kabuli_Chr04*	5853798	(T/G)
CakSNP5475	*Kabuli*	*Ca_Kabuli_Chr04*	5900194	(C/A)
CakSNP5476	*Kabuli*	*Ca_Kabuli_Chr04*	5900290	(T/C)
CakSNP5477	*Kabuli*	*Ca_Kabuli_Chr04*	5905252	(C/T)
CakSNP5478	*Kabuli*	*Ca_Kabuli_Chr04*	5905276	(C/T)
CakSNP5479	*Kabuli*	*Ca_Kabuli_Chr04*	5905303	(A/C)
CakSNP5480	*Kabuli*	*Ca_Kabuli_Chr04*	5905385	(G/A)
CakSNP5481	*Kabuli*	*Ca_Kabuli_Chr04*	5907409	(T/G)
CakSNP5482	*Kabuli*	*Ca_Kabuli_Chr04*	5907421	(T/G)
CakSNP5483	*Kabuli*	*Ca_Kabuli_Chr04*	5907437	(C/A)
CakSNP5484	*Kabuli*	*Ca_Kabuli_Chr04*	5907465	(C/A)
CakSNP5485	*Kabuli*	*Ca_Kabuli_Chr04*	5940176	(C/T)
CakSNP5486	*Kabuli*	*Ca_Kabuli_Chr04*	5999964	(T/C)
CakSNP5487	*Kabuli*	*Ca_Kabuli_Chr04*	6000010	(T/A)
CakSNP5488	*Kabuli*	*Ca_Kabuli_Chr04*	6000013	(A/C)
CakSNP5489	*Kabuli*	*Ca_Kabuli_Chr04*	6000037	(T/G)
CakSNP5490	*Kabuli*	*Ca_Kabuli_Chr04*	6006849	(T/G)
CakSNP5491	*Kabuli*	*Ca_Kabuli_Chr04*	6030948	(T/G)
CakSNP5492	*Kabuli*	*Ca_Kabuli_Chr04*	6030966	(A/G)
CakSNP5493	*Kabuli*	*Ca_Kabuli_Chr04*	6041134	(A/C)

**SNP IDs**	**Cultivars**	**Chromosomes/scaffolds**	**Physical positions (bp)**	**SNPs**
CakSNP5494	*Kabuli*	*Ca_Kabuli_Chr04*	6041130	(C/A)
CakSNP5495	*Kabuli*	*Ca_Kabuli_Chr04*	6041126	(A/G)
CakSNP5496	*Kabuli*	*Ca_Kabuli_Chr04*	6041120	(G/T)
CakSNP5497	*Kabuli*	*Ca_Kabuli_Chr04*	6041117	(C/T)
CakSNP5498	*Kabuli*	*Ca_Kabuli_Chr04*	6076530	(T/C)
CakSNP5499	*Kabuli*	*Ca_Kabuli_Chr04*	6076594	(G/C)
CakSNP5500	*Kabuli*	*Ca_Kabuli_Chr04*	6076614	(A/G)
CakSNP5501	*Kabuli*	*Ca_Kabuli_Chr04*	6077103	(G/A)
CakSNP5502	*Kabuli*	*Ca_Kabuli_Chr04*	6077113	(G/T)
CakSNP5503	*Kabuli*	*Ca_Kabuli_Chr04*	6077156	(G/T)
CakSNP5504	*Kabuli*	*Ca_Kabuli_Chr04*	6077168	(T/C)
CakSNP5505	*Kabuli*	*Ca_Kabuli_Chr04*	6157625	(T/C)
CakSNP5506	*Kabuli*	*Ca_Kabuli_Chr04*	6201137	(T/C)
CakSNP5507	*Kabuli*	*Ca_Kabuli_Chr04*	6238354	(G/A)
CakSNP5508	*Kabuli*	*Ca_Kabuli_Chr04*	6238367	(T/A)
CakSNP5509	*Kabuli*	*Ca_Kabuli_Chr04*	6238376	(A/G)
CakSNP5510	*Kabuli*	*Ca_Kabuli_Chr04*	6238414	(T/A)
CakSNP5511	*Kabuli*	*Ca_Kabuli_Chr04*	6277398	(C/T)
CakSNP5512	*Kabuli*	*Ca_Kabuli_Chr04*	6281228	(A/C)
CakSNP5513	*Kabuli*	*Ca_Kabuli_Chr04*	6296089	(G/T)
CakSNP5514	*Kabuli*	*Ca_Kabuli_Chr04*	6387938	(T/G)
CakSNP5515	*Kabuli*	*Ca_Kabuli_Chr04*	6403910	(A/C)
CakSNP5516	*Kabuli*	*Ca_Kabuli_Chr04*	6408543	(T/G)
CakSNP5517	*Kabuli*	*Ca_Kabuli_Chr04*	6415058	(A/T)
CakSNP5518	*Kabuli*	*Ca_Kabuli_Chr04*	6462228	(C/T)
CakSNP5519	*Kabuli*	*Ca_Kabuli_Chr04*	6507540	(G/A)
CakSNP5520	*Kabuli*	*Ca_Kabuli_Chr04*	6509701	(T/A)
CakSNP5521	*Kabuli*	*Ca_Kabuli_Chr04*	6509761	(C/A)
CakSNP5522	*Kabuli*	*Ca_Kabuli_Chr04*	6511977	(C/T)
CakSNP5523	*Kabuli*	*Ca_Kabuli_Chr04*	6512060	(A/T)
CakSNP5524	*Kabuli*	*Ca_Kabuli_Chr04*	6513321	(A/T)
CakSNP5525	*Kabuli*	*Ca_Kabuli_Chr04*	6513493	(C/T)
CakSNP5526	*Kabuli*	*Ca_Kabuli_Chr04*	6513576	(A/T)
CakSNP5527	*Kabuli*	*Ca_Kabuli_Chr04*	6544385	(G/A)
CakSNP5528	*Kabuli*	*Ca_Kabuli_Chr04*	6544342	(A/C)
CakSNP5529	*Kabuli*	*Ca_Kabuli_Chr04*	6544337	(A/C)
CakSNP5530	*Kabuli*	*Ca_Kabuli_Chr04*	6552776	(A/G)
CakSNP5531	*Kabuli*	*Ca_Kabuli_Chr04*	6562373	(T/A)
CakSNP5532	*Kabuli*	*Ca_Kabuli_Chr04*	6562391	(A/C)
CakSNP5533	*Kabuli*	*Ca_Kabuli_Chr04*	6562402	(G/C)
CakSNP5534	*Kabuli*	*Ca_Kabuli_Chr04*	6611820	(G/A)

SNP IDs	Cultivars	Chromosomes/scaffolds	Physical positions (bp)	SNPs
CakSNP5535	*Kabuli*	*Ca_Kabuli_Chr04*	6616016	(C/T)
CakSNP5536	*Kabuli*	*Ca_Kabuli_Chr04*	6616071	(C/T)
CakSNP5537	*Kabuli*	*Ca_Kabuli_Chr04*	6688218	(G/A)
CakSNP5538	*Kabuli*	*Ca_Kabuli_Chr04*	6715326	(C/T)
CakSNP5539	*Kabuli*	*Ca_Kabuli_Chr04*	6723103	(G/T)
CakSNP5540	*Kabuli*	*Ca_Kabuli_Chr04*	6749334	(A/G)
CakSNP5541	*Kabuli*	*Ca_Kabuli_Chr04*	6749481	(C/T)
CakSNP5542	*Kabuli*	*Ca_Kabuli_Chr04*	6749550	(G/A)
CakSNP5543	*Kabuli*	*Ca_Kabuli_Chr04*	6749522	(T/C)
CakSNP5544	*Kabuli*	*Ca_Kabuli_Chr04*	6751549	(A/T)
CakSNP5545	*Kabuli*	*Ca_Kabuli_Chr04*	6761778	(C/A)
CakSNP5546	*Kabuli*	*Ca_Kabuli_Chr04*	6765922	(T/G)
CakSNP5547	*Kabuli*	*Ca_Kabuli_Chr04*	6765884	(T/C)
CakSNP5548	*Kabuli*	*Ca_Kabuli_Chr04*	6792858	(T/G)
CakSNP5549	*Kabuli*	*Ca_Kabuli_Chr04*	6801632	(C/A)
CakSNP5550	*Kabuli*	*Ca_Kabuli_Chr04*	6841854	(G/A)
CakSNP5551	*Kabuli*	*Ca_Kabuli_Chr04*	6841856	(G/A)
CakSNP5552	*Kabuli*	*Ca_Kabuli_Chr04*	6841858	(G/A)
CakSNP5553	*Kabuli*	*Ca_Kabuli_Chr04*	6841859	(A/C)
CakSNP5554	*Kabuli*	*Ca_Kabuli_Chr04*	6841860	(G/A)
CakSNP5555	*Kabuli*	*Ca_Kabuli_Chr04*	6841861	(A/C)
CakSNP5556	*Kabuli*	*Ca_Kabuli_Chr04*	6843659	(T/C)
CakSNP5557	*Kabuli*	*Ca_Kabuli_Chr04*	6852908	(T/C)
CakSNP5558	*Kabuli*	*Ca_Kabuli_Chr04*	6889964	(T/G)
CakSNP5559	*Kabuli*	*Ca_Kabuli_Chr04*	6889974	(T/G)
CakSNP5560	*Kabuli*	*Ca_Kabuli_Chr04*	6890000	(A/G)
CakSNP5561	*Kabuli*	*Ca_Kabuli_Chr04*	6890005	(C/T)
CakSNP5562	*Kabuli*	*Ca_Kabuli_Chr04*	6890014	(G/T)
CakSNP5563	*Kabuli*	*Ca_Kabuli_Chr04*	6889994	(T/G)
CakSNP5564	*Kabuli*	*Ca_Kabuli_Chr04*	6895804	(G/A)
CakSNP5565	*Kabuli*	*Ca_Kabuli_Chr04*	6919896	(C/T)
CakSNP5566	*Kabuli*	*Ca_Kabuli_Chr04*	6920068	(G/A)
CakSNP5567	*Kabuli*	*Ca_Kabuli_Chr04*	6931668	(T/A)
CakSNP5568	*Kabuli*	*Ca_Kabuli_Chr04*	6960336	(G/A)
CakSNP5569	*Kabuli*	*Ca_Kabuli_Chr04*	6969771	(G/T)
CakSNP5570	*Kabuli*	*Ca_Kabuli_Chr04*	6969799	(T/C)
CakSNP5571	*Kabuli*	*Ca_Kabuli_Chr04*	6970808	(A/G)
CakSNP5572	*Kabuli*	*Ca_Kabuli_Chr04*	6970800	(A/G)
CakSNP5573	*Kabuli*	*Ca_Kabuli_Chr04*	6970792	(T/A)
CakSNP5574	*Kabuli*	*Ca_Kabuli_Chr04*	6970781	(G/A)
CakSNP5575	*Kabuli*	*Ca_Kabuli_Chr04*	6970856	(T/C)

**SNP IDs**	**Cultivars**	**Chromosomes/scaffolds**	**Physical positions (bp)**	**SNPs**
CakSNP5576	*Kabuli*	*Ca_Kabuli_Chr04*	7013498	(A/G)
CakSNP5577	*Kabuli*	*Ca_Kabuli_Chr04*	7040229	(A/T)
CakSNP5578	*Kabuli*	*Ca_Kabuli_Chr04*	7142498	(G/A)
CakSNP5579	*Kabuli*	*Ca_Kabuli_Chr04*	7143207	(G/T)
CakSNP5580	*Kabuli*	*Ca_Kabuli_Chr04*	7143214	(T/C)
CakSNP5581	*Kabuli*	*Ca_Kabuli_Chr04*	7143238	(C/T)
CakSNP5582	*Kabuli*	*Ca_Kabuli_Chr04*	7155826	(G/A)
CakSNP5583	*Kabuli*	*Ca_Kabuli_Chr04*	7185974	(G/A)
CakSNP5584	*Kabuli*	*Ca_Kabuli_Chr04*	7231299	(C/T)
CakSNP5585	*Kabuli*	*Ca_Kabuli_Chr04*	7239917	(T/G)
CakSNP5586	*Kabuli*	*Ca_Kabuli_Chr04*	7244301	(G/A)
CakSNP5587	*Kabuli*	*Ca_Kabuli_Chr04*	7335587	(C/T)
CakSNP5588	*Kabuli*	*Ca_Kabuli_Chr04*	7349958	(T/C)
CakSNP5589	*Kabuli*	*Ca_Kabuli_Chr04*	7349948	(T/C)
CakSNP5590	*Kabuli*	*Ca_Kabuli_Chr04*	7349900	(G/A)
CakSNP5591	*Kabuli*	*Ca_Kabuli_Chr04*	7349885	(G/A)
CakSNP5592	*Kabuli*	*Ca_Kabuli_Chr04*	7350192	(G/T)
CakSNP5593	*Kabuli*	*Ca_Kabuli_Chr04*	7371667	(G/A)
CakSNP5594	*Kabuli*	*Ca_Kabuli_Chr04*	7371708	(T/C)
CakSNP5595	*Kabuli*	*Ca_Kabuli_Chr04*	7416949	(C/T)
CakSNP5596	*Kabuli*	*Ca_Kabuli_Chr04*	7496298	(A/G)
CakSNP5597	*Kabuli*	*Ca_Kabuli_Chr04*	7520182	(A/T)
CakSNP5598	*Kabuli*	*Ca_Kabuli_Chr04*	7520184	(A/C)
CakSNP5599	*Kabuli*	*Ca_Kabuli_Chr04*	7522047	(C/T)
CakSNP5600	*Kabuli*	*Ca_Kabuli_Chr04*	7522033	(A/G)
CakSNP5601	*Kabuli*	*Ca_Kabuli_Chr04*	7587317	(A/G)
CakSNP5602	*Kabuli*	*Ca_Kabuli_Chr04*	7587516	(T/C)
CakSNP5603	*Kabuli*	*Ca_Kabuli_Chr04*	7598474	(G/C)
CakSNP5604	*Kabuli*	*Ca_Kabuli_Chr04*	7699429	(C/T)
CakSNP5605	*Kabuli*	*Ca_Kabuli_Chr04*	7717770	(A/G)
CakSNP5606	*Kabuli*	*Ca_Kabuli_Chr04*	7717935	(T/G)
CakSNP5607	*Kabuli*	*Ca_Kabuli_Chr04*	7717978	(G/A)
CakSNP5608	*Kabuli*	*Ca_Kabuli_Chr04*	7718270	(T/C)
CakSNP5609	*Kabuli*	*Ca_Kabuli_Chr04*	7718277	(G/A)
CakSNP5610	*Kabuli*	*Ca_Kabuli_Chr04*	7739202	(C/T)
CakSNP5611	*Kabuli*	*Ca_Kabuli_Chr04*	7746230	(A/C)
CakSNP5612	*Kabuli*	*Ca_Kabuli_Chr04*	7746242	(C/G)
CakSNP5613	*Kabuli*	*Ca_Kabuli_Chr04*	7812468	(C/T)
CakSNP5614	*Kabuli*	*Ca_Kabuli_Chr04*	7812491	(T/A)
CakSNP5615	*Kabuli*	*Ca_Kabuli_Chr04*	7812521	(A/G)
CakSNP5616	*Kabuli*	*Ca_Kabuli_Chr04*	7819865	(T/C)

SNP IDs	Cultivars	Chromosomes/scaffolds	Physical positions (bp)	SNPs
CakSNP5617	*Kabuli*	*Ca_Kabuli_Chr04*	7821269	(G/A)
CakSNP5618	*Kabuli*	*Ca_Kabuli_Chr04*	7868480	(A/T)
CakSNP5619	*Kabuli*	*Ca_Kabuli_Chr04*	7883707	(T/A)
CakSNP5620	*Kabuli*	*Ca_Kabuli_Chr04*	7892596	(A/C)
CakSNP5621	*Kabuli*	*Ca_Kabuli_Chr04*	7975577	(C/G)
CakSNP5622	*Kabuli*	*Ca_Kabuli_Chr04*	7975586	(T/G)
CakSNP5623	*Kabuli*	*Ca_Kabuli_Chr04*	7988367	(A/G)
CakSNP5624	*Kabuli*	*Ca_Kabuli_Chr04*	7988396	(C/T)
CakSNP5625	*Kabuli*	*Ca_Kabuli_Chr04*	7988399	(G/A)
CakSNP5626	*Kabuli*	*Ca_Kabuli_Chr04*	7988422	(T/G)
CakSNP5627	*Kabuli*	*Ca_Kabuli_Chr04*	7988444	(T/G)
CakSNP5628	*Kabuli*	*Ca_Kabuli_Chr04*	8025363	(T/C)
CakSNP5629	*Kabuli*	*Ca_Kabuli_Chr04*	8025348	(A/T)
CakSNP5630	*Kabuli*	*Ca_Kabuli_Chr04*	8025530	(A/G)
CakSNP5631	*Kabuli*	*Ca_Kabuli_Chr04*	8050770	(T/C)
CakSNP5632	*Kabuli*	*Ca_Kabuli_Chr04*	8051306	(T/C)
CakSNP5633	*Kabuli*	*Ca_Kabuli_Chr04*	8117401	(G/A)
CakSNP5634	*Kabuli*	*Ca_Kabuli_Chr04*	8178043	(G/A)
CakSNP5635	*Kabuli*	*Ca_Kabuli_Chr04*	8198702	(T/G)
CakSNP5636	*Kabuli*	*Ca_Kabuli_Chr04*	8203586	(C/T)
CakSNP5637	*Kabuli*	*Ca_Kabuli_Chr04*	8236449	(C/A)
CakSNP5638	*Kabuli*	*Ca_Kabuli_Chr04*	8237145	(C/T)
CakSNP5639	*Kabuli*	*Ca_Kabuli_Chr04*	8269099	(T/G)
CakSNP5640	*Kabuli*	*Ca_Kabuli_Chr04*	8269152	(C/T)
CakSNP5641	*Kabuli*	*Ca_Kabuli_Chr04*	8269347	(C/G)
CakSNP5642	*Kabuli*	*Ca_Kabuli_Chr04*	8302198	(A/G)
CakSNP5643	*Kabuli*	*Ca_Kabuli_Chr04*	8383890	(G/A)
CakSNP5644	*Kabuli*	*Ca_Kabuli_Chr04*	8396861	(T/A)
CakSNP5645	*Kabuli*	*Ca_Kabuli_Chr04*	8396837	(T/G)
CakSNP5646	*Kabuli*	*Ca_Kabuli_Chr04*	8401109	(A/G)
CakSNP5647	*Kabuli*	*Ca_Kabuli_Chr04*	8401126	(G/T)
CakSNP5648	*Kabuli*	*Ca_Kabuli_Chr04*	8401143	(A/T)
CakSNP5649	*Kabuli*	*Ca_Kabuli_Chr04*	8401248	(T/A)
CakSNP5650	*Kabuli*	*Ca_Kabuli_Chr04*	8401230	(A/G)
CakSNP5651	*Kabuli*	*Ca_Kabuli_Chr04*	8404108	(T/C)
CakSNP5652	*Kabuli*	*Ca_Kabuli_Chr04*	8404165	(C/A)
CakSNP5653	*Kabuli*	*Ca_Kabuli_Chr04*	8404244	(C/G)
CakSNP5654	*Kabuli*	*Ca_Kabuli_Chr04*	8471029	(G/A)
CakSNP5655	*Kabuli*	*Ca_Kabuli_Chr04*	8473120	(G/A)
CakSNP5656	*Kabuli*	*Ca_Kabuli_Chr04*	8473127	(G/A)
CakSNP5657	*Kabuli*	*Ca_Kabuli_Chr04*	8473294	(G/A)

SNP IDs	Cultivars	Chromosomes/scaffolds	Physical positions (bp)	SNPs
CakSNP5658	*Kabuli*	*Ca_Kabuli_Chr04*	8473288	(A/G)
CakSNP5659	*Kabuli*	*Ca_Kabuli_Chr04*	8473246	(T/C)
CakSNP5660	*Kabuli*	*Ca_Kabuli_Chr04*	8474442	(C/T)
CakSNP5661	*Kabuli*	*Ca_Kabuli_Chr04*	8474441	(G/A)
CakSNP5662	*Kabuli*	*Ca_Kabuli_Chr04*	8474401	(T/A)
CakSNP5663	*Kabuli*	*Ca_Kabuli_Chr04*	8475634	(A/C)
CakSNP5664	*Kabuli*	*Ca_Kabuli_Chr04*	8483482	(T/C)
CakSNP5665	*Kabuli*	*Ca_Kabuli_Chr04*	8483494	(T/C)
CakSNP5666	*Kabuli*	*Ca_Kabuli_Chr04*	8484976	(G/A)
CakSNP5667	*Kabuli*	*Ca_Kabuli_Chr04*	8484978	(A/G)
CakSNP5668	*Kabuli*	*Ca_Kabuli_Chr04*	8496641	(C/A)
CakSNP5669	*Kabuli*	*Ca_Kabuli_Chr04*	8510011	(A/C)
CakSNP5670	*Kabuli*	*Ca_Kabuli_Chr04*	8512095	(G/A)
CakSNP5671	*Kabuli*	*Ca_Kabuli_Chr04*	8512280	(C/T)
CakSNP5672	*Kabuli*	*Ca_Kabuli_Chr04*	8518979	(G/A)
CakSNP5673	*Kabuli*	*Ca_Kabuli_Chr04*	8534120	(C/T)
CakSNP5674	*Kabuli*	*Ca_Kabuli_Chr04*	8567380	(A/T)
CakSNP5675	*Kabuli*	*Ca_Kabuli_Chr04*	8567431	(C/T)
CakSNP5676	*Kabuli*	*Ca_Kabuli_Chr04*	8567490	(C/A)
CakSNP5677	*Kabuli*	*Ca_Kabuli_Chr04*	8567478	(A/C)
CakSNP5678	*Kabuli*	*Ca_Kabuli_Chr04*	8567477	(T/C)
CakSNP5679	*Kabuli*	*Ca_Kabuli_Chr04*	8567465	(C/T)
CakSNP5680	*Kabuli*	*Ca_Kabuli_Chr04*	8570458	(T/G)
CakSNP5681	*Kabuli*	*Ca_Kabuli_Chr04*	8570501	(G/C)
CakSNP5682	*Kabuli*	*Ca_Kabuli_Chr04*	8595427	(C/T)
CakSNP5683	*Kabuli*	*Ca_Kabuli_Chr04*	8601364	(T/G)
CakSNP5684	*Kabuli*	*Ca_Kabuli_Chr04*	8659774	(G/A)
CakSNP5685	*Kabuli*	*Ca_Kabuli_Chr04*	8659935	(T/A)
CakSNP5686	*Kabuli*	*Ca_Kabuli_Chr04*	8659946	(C/T)
CakSNP5687	*Kabuli*	*Ca_Kabuli_Chr04*	8659985	(T/C)
CakSNP5688	*Kabuli*	*Ca_Kabuli_Chr04*	8660115	(A/T)
CakSNP5689	*Kabuli*	*Ca_Kabuli_Chr04*	8660136	(G/A)
CakSNP5690	*Kabuli*	*Ca_Kabuli_Chr04*	8660608	(G/T)
CakSNP5691	*Kabuli*	*Ca_Kabuli_Chr04*	8660599	(A/G)
CakSNP5692	*Kabuli*	*Ca_Kabuli_Chr04*	8668648	(G/A)
CakSNP5693	*Kabuli*	*Ca_Kabuli_Chr04*	8668753	(A/G)
CakSNP5694	*Kabuli*	*Ca_Kabuli_Chr04*	8668945	(T/C)
CakSNP5695	*Kabuli*	*Ca_Kabuli_Chr04*	8669014	(C/G)
CakSNP5696	*Kabuli*	*Ca_Kabuli_Chr04*	8669104	(T/C)
CakSNP5697	*Kabuli*	*Ca_Kabuli_Chr04*	8669515	(C/T)
CakSNP5698	*Kabuli*	*Ca_Kabuli_Chr04*	8669513	(G/A)

SNP IDs	Cultivars	Chromosomes/scaffolds	Physical positions (bp)	SNPs
CakSNP5699	*Kabuli*	*Ca_Kabuli_Chr04*	8669505	(G/T)
CakSNP5700	*Kabuli*	*Ca_Kabuli_Chr04*	8681949	(G/A)
CakSNP5701	*Kabuli*	*Ca_Kabuli_Chr04*	8695562	(C/T)
CakSNP5702	*Kabuli*	*Ca_Kabuli_Chr04*	8751638	(A/C)
CakSNP5703	*Kabuli*	*Ca_Kabuli_Chr04*	8751629	(T/C)
CakSNP5704	*Kabuli*	*Ca_Kabuli_Chr04*	8751618	(A/C)
CakSNP5705	*Kabuli*	*Ca_Kabuli_Chr04*	8751591	(A/C)
CakSNP5706	*Kabuli*	*Ca_Kabuli_Chr04*	8772215	(A/C)
CakSNP5707	*Kabuli*	*Ca_Kabuli_Chr04*	8782879	(G/C)
CakSNP5708	*Kabuli*	*Ca_Kabuli_Chr04*	8791654	(A/G)
CakSNP5709	*Kabuli*	*Ca_Kabuli_Chr04*	8805504	(A/G)
CakSNP5710	*Kabuli*	*Ca_Kabuli_Chr04*	8805578	(A/C)
CakSNP5711	*Kabuli*	*Ca_Kabuli_Chr04*	8805667	(C/T)
CakSNP5712	*Kabuli*	*Ca_Kabuli_Chr04*	8805867	(A/C)
CakSNP5713	*Kabuli*	*Ca_Kabuli_Chr04*	8805839	(G/A)
CakSNP5714	*Kabuli*	*Ca_Kabuli_Chr04*	8809032	(C/T)
CakSNP5715	*Kabuli*	*Ca_Kabuli_Chr04*	8809064	(T/C)
CakSNP5716	*Kabuli*	*Ca_Kabuli_Chr04*	8809010	(A/G)
CakSNP5717	*Kabuli*	*Ca_Kabuli_Chr04*	8809456	(A/C)
CakSNP5718	*Kabuli*	*Ca_Kabuli_Chr04*	8844846	(A/G)
CakSNP5719	*Kabuli*	*Ca_Kabuli_Chr04*	8848654	(C/T)
CakSNP5720	*Kabuli*	*Ca_Kabuli_Chr04*	8848653	(T/C)
CakSNP5721	*Kabuli*	*Ca_Kabuli_Chr04*	8852334	(T/A)
CakSNP5722	*Kabuli*	*Ca_Kabuli_Chr04*	8852420	(T/C)
CakSNP5723	*Kabuli*	*Ca_Kabuli_Chr04*	8873494	(G/T)
CakSNP5724	*Kabuli*	*Ca_Kabuli_Chr04*	8873509	(G/C)
CakSNP5725	*Kabuli*	*Ca_Kabuli_Chr04*	8919264	(C/T)
CakSNP5726	*Kabuli*	*Ca_Kabuli_Chr04*	8919300	(C/T)
CakSNP5727	*Kabuli*	*Ca_Kabuli_Chr04*	8919395	(A/G)
CakSNP5728	*Kabuli*	*Ca_Kabuli_Chr04*	8952236	(A/G)
CakSNP5729	*Kabuli*	*Ca_Kabuli_Chr04*	8985949	(C/T)
CakSNP5730	*Kabuli*	*Ca_Kabuli_Chr04*	8985961	(C/T)
CakSNP5731	*Kabuli*	*Ca_Kabuli_Chr04*	8986031	(T/A)
CakSNP5732	*Kabuli*	*Ca_Kabuli_Chr04*	8986026	(T/A)
CakSNP5733	*Kabuli*	*Ca_Kabuli_Chr04*	8986024	(T/A)
CakSNP5734	*Kabuli*	*Ca_Kabuli_Chr04*	8986018	(G/A)
CakSNP5735	*Kabuli*	*Ca_Kabuli_Chr04*	8986008	(T/A)
CakSNP5736	*Kabuli*	*Ca_Kabuli_Chr04*	8986002	(T/A)
CakSNP5737	*Kabuli*	*Ca_Kabuli_Chr04*	8985998	(T/A)
CakSNP5738	*Kabuli*	*Ca_Kabuli_Chr04*	8985994	(C/A)
CakSNP5739	*Kabuli*	*Ca_Kabuli_Chr04*	8986207	(G/C)

SNP IDs	Cultivars	Chromosomes/scaffolds	Physical positions (bp)	SNPs
CakSNP5740	*Kabuli*	*Ca_Kabuli_Chr04*	8986217	(T/A)
CakSNP5741	*Kabuli*	*Ca_Kabuli_Chr04*	9075398	(G/A)
CakSNP5742	*Kabuli*	*Ca_Kabuli_Chr04*	9075395	(A/G)
CakSNP5743	*Kabuli*	*Ca_Kabuli_Chr04*	9075459	(C/T)
CakSNP5744	*Kabuli*	*Ca_Kabuli_Chr04*	9075520	(C/T)
CakSNP5745	*Kabuli*	*Ca_Kabuli_Chr04*	9075565	(A/G)
CakSNP5746	*Kabuli*	*Ca_Kabuli_Chr04*	9075543	(T/C)
CakSNP5747	*Kabuli*	*Ca_Kabuli_Chr04*	9075671	(A/G)
CakSNP5748	*Kabuli*	*Ca_Kabuli_Chr04*	9075677	(T/C)
CakSNP5749	*Kabuli*	*Ca_Kabuli_Chr04*	9075682	(G/A)
CakSNP5750	*Kabuli*	*Ca_Kabuli_Chr04*	9075737	(G/A)
CakSNP5751	*Kabuli*	*Ca_Kabuli_Chr04*	9075744	(T/G)
CakSNP5752	*Kabuli*	*Ca_Kabuli_Chr04*	9187887	(G/C)
CakSNP5753	*Kabuli*	*Ca_Kabuli_Chr04*	9187973	(T/G)
CakSNP5754	*Kabuli*	*Ca_Kabuli_Chr04*	9189976	(T/A)
CakSNP5755	*Kabuli*	*Ca_Kabuli_Chr04*	9211570	(A/G)
CakSNP5756	*Kabuli*	*Ca_Kabuli_Chr04*	9257131	(G/A)
CakSNP5757	*Kabuli*	*Ca_Kabuli_Chr04*	9331949	(C/T)
CakSNP5758	*Kabuli*	*Ca_Kabuli_Chr04*	9392373	(A/T)
CakSNP5759	*Kabuli*	*Ca_Kabuli_Chr04*	9392455	(A/G)
CakSNP5760	*Kabuli*	*Ca_Kabuli_Chr04*	9392427	(G/A)
CakSNP5761	*Kabuli*	*Ca_Kabuli_Chr04*	9427890	(T/G)
CakSNP5762	*Kabuli*	*Ca_Kabuli_Chr04*	9427892	(G/T)
CakSNP5763	*Kabuli*	*Ca_Kabuli_Chr04*	9427894	(A/C)
CakSNP5764	*Kabuli*	*Ca_Kabuli_Chr04*	9427964	(C/A)
CakSNP5765	*Kabuli*	*Ca_Kabuli_Chr04*	9451895	(T/A)
CakSNP5766	*Kabuli*	*Ca_Kabuli_Chr04*	9451850	(T/G)
CakSNP5767	*Kabuli*	*Ca_Kabuli_Chr04*	9491741	(A/T)
CakSNP5768	*Kabuli*	*Ca_Kabuli_Chr04*	9533658	(A/G)
CakSNP5769	*Kabuli*	*Ca_Kabuli_Chr04*	9533688	(T/G)
CakSNP5770	*Kabuli*	*Ca_Kabuli_Chr04*	9579383	(C/A)
CakSNP5771	*Kabuli*	*Ca_Kabuli_Chr04*	9579393	(G/A)
CakSNP5772	*Kabuli*	*Ca_Kabuli_Chr04*	9579401	(G/T)
CakSNP5773	*Kabuli*	*Ca_Kabuli_Chr04*	9579519	(A/T)
CakSNP5774	*Kabuli*	*Ca_Kabuli_Chr04*	9579517	(T/C)
CakSNP5775	*Kabuli*	*Ca_Kabuli_Chr04*	9580341	(T/A)
CakSNP5776	*Kabuli*	*Ca_Kabuli_Chr04*	9580339	(T/G)
CakSNP5777	*Kabuli*	*Ca_Kabuli_Chr04*	9614897	(G/A)
CakSNP5778	*Kabuli*	*Ca_Kabuli_Chr04*	9614905	(C/A)
CakSNP5779	*Kabuli*	*Ca_Kabuli_Chr04*	9661685	(G/A)
CakSNP5780	*Kabuli*	*Ca_Kabuli_Chr04*	9670905	(C/T)

SNP IDs	Cultivars	Chromosomes/scaffolds	Physical positions (bp)	SNPs
CakSNP5781	*Kabuli*	*Ca_Kabuli_Chr04*	9670996	(G/T)
CakSNP5782	*Kabuli*	*Ca_Kabuli_Chr04*	9671007	(G/T)
CakSNP5783	*Kabuli*	*Ca_Kabuli_Chr04*	9671089	(T/G)
CakSNP5784	*Kabuli*	*Ca_Kabuli_Chr04*	9706453	(T/G)
CakSNP5785	*Kabuli*	*Ca_Kabuli_Chr04*	9706480	(T/A)
CakSNP5786	*Kabuli*	*Ca_Kabuli_Chr04*	9706481	(A/C)
CakSNP5787	*Kabuli*	*Ca_Kabuli_Chr04*	9740099	(A/G)
CakSNP5788	*Kabuli*	*Ca_Kabuli_Chr04*	9800865	(C/T)
CakSNP5789	*Kabuli*	*Ca_Kabuli_Chr04*	9802603	(T/C)
CakSNP5790	*Kabuli*	*Ca_Kabuli_Chr04*	9836559	(G/T)
CakSNP5791	*Kabuli*	*Ca_Kabuli_Chr04*	9840763	(C/A)
CakSNP5792	*Kabuli*	*Ca_Kabuli_Chr04*	9840906	(A/C)
CakSNP5793	*Kabuli*	*Ca_Kabuli_Chr04*	9846321	(T/A)
CakSNP5794	*Kabuli*	*Ca_Kabuli_Chr04*	9947077	(A/G)
CakSNP5795	*Kabuli*	*Ca_Kabuli_Chr04*	9973728	(C/G)
CakSNP5796	*Kabuli*	*Ca_Kabuli_Chr04*	9976039	(C/T)
CakSNP5797	*Kabuli*	*Ca_Kabuli_Chr04*	9999907	(A/T)
CakSNP5798	*Kabuli*	*Ca_Kabuli_Chr04*	10007069	(A/G)
CakSNP5799	*Kabuli*	*Ca_Kabuli_Chr04*	10007151	(C/T)
CakSNP5800	*Kabuli*	*Ca_Kabuli_Chr04*	10007193	(T/A)
CakSNP5801	*Kabuli*	*Ca_Kabuli_Chr04*	10007332	(T/C)
CakSNP5802	*Kabuli*	*Ca_Kabuli_Chr04*	10007286	(C/T)
CakSNP5803	*Kabuli*	*Ca_Kabuli_Chr04*	10007372	(C/A)
CakSNP5804	*Kabuli*	*Ca_Kabuli_Chr04*	10009839	(G/A)
CakSNP5805	*Kabuli*	*Ca_Kabuli_Chr04*	10013120	(G/A)
CakSNP5806	*Kabuli*	*Ca_Kabuli_Chr04*	10013148	(G/T)
CakSNP5807	*Kabuli*	*Ca_Kabuli_Chr04*	10017467	(A/C)
CakSNP5808	*Kabuli*	*Ca_Kabuli_Chr04*	10024226	(G/A)
CakSNP5809	*Kabuli*	*Ca_Kabuli_Chr04*	10030134	(A/G)
CakSNP5810	*Kabuli*	*Ca_Kabuli_Chr04*	10116516	(T/G)
CakSNP5811	*Kabuli*	*Ca_Kabuli_Chr04*	10151711	(A/C)
CakSNP5812	*Kabuli*	*Ca_Kabuli_Chr04*	10151943	(C/T)
CakSNP5813	*Kabuli*	*Ca_Kabuli_Chr04*	10184428	(G/T)
CakSNP5814	*Kabuli*	*Ca_Kabuli_Chr04*	10184427	(C/A)
CakSNP5815	*Kabuli*	*Ca_Kabuli_Chr04*	10185602	(T/G)
CakSNP5816	*Kabuli*	*Ca_Kabuli_Chr04*	10185625	(A/G)
CakSNP5817	*Kabuli*	*Ca_Kabuli_Chr04*	10205246	(C/T)
CakSNP5818	*Kabuli*	*Ca_Kabuli_Chr04*	10290706	(G/T)
CakSNP5819	*Kabuli*	*Ca_Kabuli_Chr04*	10293203	(T/C)
CakSNP5820	*Kabuli*	*Ca_Kabuli_Chr04*	10293241	(C/G)
CakSNP5821	*Kabuli*	*Ca_Kabuli_Chr04*	10294380	(A/G)

SNP IDs	Cultivars	Chromosomes/scaffolds	Physical positions (bp)	SNPs
CakSNP5822	*Kabuli*	*Ca_Kabuli_Chr04*	10305276	(G/A)
CakSNP5823	*Kabuli*	*Ca_Kabuli_Chr04*	10305235	(A/G)
CakSNP5824	*Kabuli*	*Ca_Kabuli_Chr04*	10305285	(C/A)
CakSNP5825	*Kabuli*	*Ca_Kabuli_Chr04*	10411809	(G/A)
CakSNP5826	*Kabuli*	*Ca_Kabuli_Chr04*	10422142	(A/T)
CakSNP5827	*Kabuli*	*Ca_Kabuli_Chr04*	10422469	(C/A)
CakSNP5828	*Kabuli*	*Ca_Kabuli_Chr04*	10430622	(A/C)
CakSNP5829	*Kabuli*	*Ca_Kabuli_Chr04*	10430617	(A/C)
CakSNP5830	*Kabuli*	*Ca_Kabuli_Chr04*	10430613	(C/A)
CakSNP5831	*Kabuli*	*Ca_Kabuli_Chr04*	10430603	(G/A)
CakSNP5832	*Kabuli*	*Ca_Kabuli_Chr04*	10430595	(A/C)
CakSNP5833	*Kabuli*	*Ca_Kabuli_Chr04*	10430588	(T/C)
CakSNP5834	*Kabuli*	*Ca_Kabuli_Chr04*	10430574	(A/T)
CakSNP5835	*Kabuli*	*Ca_Kabuli_Chr04*	10464927	(T/G)
CakSNP5836	*Kabuli*	*Ca_Kabuli_Chr04*	10465328	(T/C)
CakSNP5837	*Kabuli*	*Ca_Kabuli_Chr04*	10547575	(G/A)
CakSNP5838	*Kabuli*	*Ca_Kabuli_Chr04*	10547561	(T/C)
CakSNP5839	*Kabuli*	*Ca_Kabuli_Chr04*	10642002	(A/G)
CakSNP5840	*Kabuli*	*Ca_Kabuli_Chr04*	10642043	(T/G)
CakSNP5841	*Kabuli*	*Ca_Kabuli_Chr04*	10642044	(A/T)
CakSNP5842	*Kabuli*	*Ca_Kabuli_Chr04*	10643339	(T/A)
CakSNP5843	*Kabuli*	*Ca_Kabuli_Chr04*	10643331	(T/A)
CakSNP5844	*Kabuli*	*Ca_Kabuli_Chr04*	10644245	(G/A)
CakSNP5845	*Kabuli*	*Ca_Kabuli_Chr04*	10670664	(C/A)
CakSNP5846	*Kabuli*	*Ca_Kabuli_Chr04*	10699624	(A/T)
CakSNP5847	*Kabuli*	*Ca_Kabuli_Chr04*	10780990	(A/G)
CakSNP5848	*Kabuli*	*Ca_Kabuli_Chr04*	10807993	(T/C)
CakSNP5849	*Kabuli*	*Ca_Kabuli_Chr04*	10827485	(G/A)
CakSNP5850	*Kabuli*	*Ca_Kabuli_Chr04*	10827515	(C/G)
CakSNP5851	*Kabuli*	*Ca_Kabuli_Chr04*	10901282	(T/C)
CakSNP5852	*Kabuli*	*Ca_Kabuli_Chr04*	10917723	(T/C)
CakSNP5853	*Kabuli*	*Ca_Kabuli_Chr04*	10917717	(C/G)
CakSNP5854	*Kabuli*	*Ca_Kabuli_Chr04*	10925490	(G/A)
CakSNP5855	*Kabuli*	*Ca_Kabuli_Chr04*	10928923	(T/C)
CakSNP5856	*Kabuli*	*Ca_Kabuli_Chr04*	10950823	(C/T)
CakSNP5857	*Kabuli*	*Ca_Kabuli_Chr04*	10971039	(C/A)
CakSNP5858	*Kabuli*	*Ca_Kabuli_Chr04*	10990000	(T/C)
CakSNP5859	*Kabuli*	*Ca_Kabuli_Chr04*	10990008	(A/T)
CakSNP5860	*Kabuli*	*Ca_Kabuli_Chr04*	10990049	(G/T)
CakSNP5861	*Kabuli*	*Ca_Kabuli_Chr04*	10990058	(T/C)
CakSNP5862	*Kabuli*	*Ca_Kabuli_Chr04*	10990265	(T/A)

SNP IDs	Cultivars	Chromosomes/scaffolds	Physical positions (bp)	SNPs
CakSNP5863	*Kabuli*	*Ca_Kabuli_Chr04*	10990261	(A/G)
CakSNP5864	*Kabuli*	*Ca_Kabuli_Chr04*	10991842	(G/A)
CakSNP5865	*Kabuli*	*Ca_Kabuli_Chr04*	10991910	(C/T)
CakSNP5866	*Kabuli*	*Ca_Kabuli_Chr04*	11040108	(C/T)
CakSNP5867	*Kabuli*	*Ca_Kabuli_Chr04*	11051335	(C/T)
CakSNP5868	*Kabuli*	*Ca_Kabuli_Chr04*	11059403	(T/G)
CakSNP5869	*Kabuli*	*Ca_Kabuli_Chr04*	11059404	(T/G)
CakSNP5870	*Kabuli*	*Ca_Kabuli_Chr04*	11059414	(T/G)
CakSNP5871	*Kabuli*	*Ca_Kabuli_Chr04*	11059572	(G/A)
CakSNP5872	*Kabuli*	*Ca_Kabuli_Chr04*	11059540	(T/C)
CakSNP5873	*Kabuli*	*Ca_Kabuli_Chr04*	11096124	(A/G)
CakSNP5874	*Kabuli*	*Ca_Kabuli_Chr04*	11096225	(A/G)
CakSNP5875	*Kabuli*	*Ca_Kabuli_Chr04*	11096290	(G/A)
CakSNP5876	*Kabuli*	*Ca_Kabuli_Chr04*	11113277	(A/C)
CakSNP5877	*Kabuli*	*Ca_Kabuli_Chr04*	11114238	(T/C)
CakSNP5878	*Kabuli*	*Ca_Kabuli_Chr04*	11142202	(A/G)
CakSNP5879	*Kabuli*	*Ca_Kabuli_Chr04*	11142227	(C/T)
CakSNP5880	*Kabuli*	*Ca_Kabuli_Chr04*	11196803	(T/G)
CakSNP5881	*Kabuli*	*Ca_Kabuli_Chr04*	11230403	(G/T)
CakSNP5882	*Kabuli*	*Ca_Kabuli_Chr04*	11230400	(T/A)
CakSNP5883	*Kabuli*	*Ca_Kabuli_Chr04*	11230397	(G/C)
CakSNP5884	*Kabuli*	*Ca_Kabuli_Chr04*	11231142	(A/G)
CakSNP5885	*Kabuli*	*Ca_Kabuli_Chr04*	11231137	(A/G)
CakSNP5886	*Kabuli*	*Ca_Kabuli_Chr04*	11231135	(C/T)
CakSNP5887	*Kabuli*	*Ca_Kabuli_Chr04*	11244334	(T/G)
CakSNP5888	*Kabuli*	*Ca_Kabuli_Chr04*	11244395	(G/T)
CakSNP5889	*Kabuli*	*Ca_Kabuli_Chr04*	11246093	(G/A)
CakSNP5890	*Kabuli*	*Ca_Kabuli_Chr04*	11246173	(C/T)
CakSNP5891	*Kabuli*	*Ca_Kabuli_Chr04*	11246164	(G/A)
CakSNP5892	*Kabuli*	*Ca_Kabuli_Chr04*	11273328	(T/C)
CakSNP5893	*Kabuli*	*Ca_Kabuli_Chr04*	11273405	(A/T)
CakSNP5894	*Kabuli*	*Ca_Kabuli_Chr04*	11274281	(T/C)
CakSNP5895	*Kabuli*	*Ca_Kabuli_Chr04*	11275171	(T/C)
CakSNP5896	*Kabuli*	*Ca_Kabuli_Chr04*	11276413	(C/G)
CakSNP5897	*Kabuli*	*Ca_Kabuli_Chr04*	11276484	(T/C)
CakSNP5898	*Kabuli*	*Ca_Kabuli_Chr04*	11277138	(A/G)
CakSNP5899	*Kabuli*	*Ca_Kabuli_Chr04*	11277297	(G/A)
CakSNP5900	*Kabuli*	*Ca_Kabuli_Chr04*	11277574	(G/C)
CakSNP5901	*Kabuli*	*Ca_Kabuli_Chr04*	11334350	(A/G)
CakSNP5902	*Kabuli*	*Ca_Kabuli_Chr04*	11334343	(A/T)
CakSNP5903	*Kabuli*	*Ca_Kabuli_Chr04*	11343257	(T/C)

SNP IDs	Cultivars	Chromosomes/scaffolds	Physical positions (bp)	SNPs
CakSNP5904	*Kabuli*	*Ca_Kabuli_Chr04*	11343230	(G/C)
CakSNP5905	*Kabuli*	*Ca_Kabuli_Chr04*	11379077	(T/C)
CakSNP5906	*Kabuli*	*Ca_Kabuli_Chr04*	11398699	(A/G)
CakSNP5907	*Kabuli*	*Ca_Kabuli_Chr04*	11398682	(G/A)
CakSNP5908	*Kabuli*	*Ca_Kabuli_Chr04*	11398889	(A/G)
CakSNP5909	*Kabuli*	*Ca_Kabuli_Chr04*	11414506	(C/T)
CakSNP5910	*Kabuli*	*Ca_Kabuli_Chr04*	11435651	(A/G)
CakSNP5911	*Kabuli*	*Ca_Kabuli_Chr04*	11441604	(T/A)
CakSNP5912	*Kabuli*	*Ca_Kabuli_Chr04*	11441615	(T/C)
CakSNP5913	*Kabuli*	*Ca_Kabuli_Chr04*	11441619	(G/A)
CakSNP5914	*Kabuli*	*Ca_Kabuli_Chr04*	11441655	(T/A)
CakSNP5915	*Kabuli*	*Ca_Kabuli_Chr04*	11441707	(A/C)
CakSNP5916	*Kabuli*	*Ca_Kabuli_Chr04*	11465057	(C/G)
CakSNP5917	*Kabuli*	*Ca_Kabuli_Chr04*	11465075	(T/C)
CakSNP5918	*Kabuli*	*Ca_Kabuli_Chr04*	11465113	(C/T)
CakSNP5919	*Kabuli*	*Ca_Kabuli_Chr04*	11490100	(C/T)
CakSNP5920	*Kabuli*	*Ca_Kabuli_Chr04*	11490154	(A/T)
CakSNP5921	*Kabuli*	*Ca_Kabuli_Chr04*	11490125	(G/T)
CakSNP5922	*Kabuli*	*Ca_Kabuli_Chr04*	11490496	(G/A)
CakSNP5923	*Kabuli*	*Ca_Kabuli_Chr04*	11517582	(C/T)
CakSNP5924	*Kabuli*	*Ca_Kabuli_Chr04*	11517517	(C/A)
CakSNP5925	*Kabuli*	*Ca_Kabuli_Chr04*	11572051	(C/T)
CakSNP5926	*Kabuli*	*Ca_Kabuli_Chr04*	11645738	(A/G)
CakSNP5927	*Kabuli*	*Ca_Kabuli_Chr04*	11646347	(C/G)
CakSNP5928	*Kabuli*	*Ca_Kabuli_Chr04*	11646453	(G/A)
CakSNP5929	*Kabuli*	*Ca_Kabuli_Chr04*	11658323	(A/G)
CakSNP5930	*Kabuli*	*Ca_Kabuli_Chr04*	11658314	(A/G)
CakSNP5931	*Kabuli*	*Ca_Kabuli_Chr04*	11689611	(C/T)
CakSNP5932	*Kabuli*	*Ca_Kabuli_Chr04*	11689647	(T/C)
CakSNP5933	*Kabuli*	*Ca_Kabuli_Chr04*	11752862	(A/G)
CakSNP5934	*Kabuli*	*Ca_Kabuli_Chr04*	11752855	(A/G)
CakSNP5935	*Kabuli*	*Ca_Kabuli_Chr04*	11772023	(G/T)
CakSNP5936	*Kabuli*	*Ca_Kabuli_Chr04*	11838005	(C/T)
CakSNP5937	*Kabuli*	*Ca_Kabuli_Chr04*	11852291	(A/G)
CakSNP5938	*Kabuli*	*Ca_Kabuli_Chr04*	11926352	(G/C)
CakSNP5939	*Kabuli*	*Ca_Kabuli_Chr04*	11985628	(G/A)
CakSNP5940	*Kabuli*	*Ca_Kabuli_Chr04*	11985604	(C/T)
CakSNP5941	*Kabuli*	*Ca_Kabuli_Chr04*	12004082	(A/T)
CakSNP5942	*Kabuli*	*Ca_Kabuli_Chr04*	12004209	(C/T)
CakSNP5943	*Kabuli*	*Ca_Kabuli_Chr04*	12004205	(A/C)
CakSNP5944	*Kabuli*	*Ca_Kabuli_Chr04*	12023137	(A/T)

SNP IDs	Cultivars	Chromosomes/scaffolds	Physical positions (bp)	SNPs
CakSNP5945	*Kabuli*	*Ca_Kabuli_Chr04*	12023215	(G/A)
CakSNP5946	*Kabuli*	*Ca_Kabuli_Chr04*	12045899	(T/A)
CakSNP5947	*Kabuli*	*Ca_Kabuli_Chr04*	12045903	(C/A)
CakSNP5948	*Kabuli*	*Ca_Kabuli_Chr04*	12045906	(C/A)
CakSNP5949	*Kabuli*	*Ca_Kabuli_Chr04*	12045910	(G/A)
CakSNP5950	*Kabuli*	*Ca_Kabuli_Chr04*	12070990	(T/A)
CakSNP5951	*Kabuli*	*Ca_Kabuli_Chr04*	12070994	(C/A)
CakSNP5952	*Kabuli*	*Ca_Kabuli_Chr04*	12070997	(C/A)
CakSNP5953	*Kabuli*	*Ca_Kabuli_Chr04*	12071001	(G/A)
CakSNP5954	*Kabuli*	*Ca_Kabuli_Chr04*	12167328	(A/G)
CakSNP5955	*Kabuli*	*Ca_Kabuli_Chr04*	12167568	(G/A)
CakSNP5956	*Kabuli*	*Ca_Kabuli_Chr04*	12212917	(G/A)
CakSNP5957	*Kabuli*	*Ca_Kabuli_Chr04*	12213071	(C/A)
CakSNP5958	*Kabuli*	*Ca_Kabuli_Chr04*	12213046	(T/C)
CakSNP5959	*Kabuli*	*Ca_Kabuli_Chr04*	12234358	(C/A)
CakSNP5960	*Kabuli*	*Ca_Kabuli_Chr04*	12249494	(T/C)
CakSNP5961	*Kabuli*	*Ca_Kabuli_Chr04*	12251184	(G/A)
CakSNP5962	*Kabuli*	*Ca_Kabuli_Chr04*	12274290	(A/T)
CakSNP5963	*Kabuli*	*Ca_Kabuli_Chr04*	12316847	(T/C)
CakSNP5964	*Kabuli*	*Ca_Kabuli_Chr04*	12431895	(C/T)
CakSNP5965	*Kabuli*	*Ca_Kabuli_Chr04*	12431915	(A/G)
CakSNP5966	*Kabuli*	*Ca_Kabuli_Chr04*	12484911	(A/T)
CakSNP5967	*Kabuli*	*Ca_Kabuli_Chr04*	12506440	(A/G)
CakSNP5968	*Kabuli*	*Ca_Kabuli_Chr04*	12506450	(T/A)
CakSNP5969	*Kabuli*	*Ca_Kabuli_Chr04*	12514728	(G/A)
CakSNP5970	*Kabuli*	*Ca_Kabuli_Chr04*	12514726	(C/T)
CakSNP5971	*Kabuli*	*Ca_Kabuli_Chr04*	12514724	(T/C)
CakSNP5972	*Kabuli*	*Ca_Kabuli_Chr04*	12514706	(G/A)
CakSNP5973	*Kabuli*	*Ca_Kabuli_Chr04*	12525328	(A/G)
CakSNP5974	*Kabuli*	*Ca_Kabuli_Chr04*	12525358	(C/G)
CakSNP5975	*Kabuli*	*Ca_Kabuli_Chr04*	12525639	(T/A)
CakSNP5976	*Kabuli*	*Ca_Kabuli_Chr04*	12534847	(T/C)
CakSNP5977	*Kabuli*	*Ca_Kabuli_Chr04*	12558541	(T/C)
CakSNP5978	*Kabuli*	*Ca_Kabuli_Chr04*	12558680	(C/T)
CakSNP5979	*Kabuli*	*Ca_Kabuli_Chr04*	12568707	(G/A)
CakSNP5980	*Kabuli*	*Ca_Kabuli_Chr04*	12568845	(G/T)
CakSNP5981	*Kabuli*	*Ca_Kabuli_Chr04*	12568905	(G/A)
CakSNP5982	*Kabuli*	*Ca_Kabuli_Chr04*	12568944	(A/G)
CakSNP5983	*Kabuli*	*Ca_Kabuli_Chr04*	12568995	(G/A)
CakSNP5984	*Kabuli*	*Ca_Kabuli_Chr04*	12569133	(G/A)
CakSNP5985	*Kabuli*	*Ca_Kabuli_Chr04*	12574650	(G/A)

SNP IDs	Cultivars	Chromosomes/scaffolds	Physical positions (bp)	SNPs
CakSNP5986	*Kabuli*	*Ca_Kabuli_Chr04*	12578259	(A/G)
CakSNP5987	*Kabuli*	*Ca_Kabuli_Chr04*	12578350	(A/G)
CakSNP5988	*Kabuli*	*Ca_Kabuli_Chr04*	12581371	(G/T)
CakSNP5989	*Kabuli*	*Ca_Kabuli_Chr04*	12692925	(C/T)
CakSNP5990	*Kabuli*	*Ca_Kabuli_Chr04*	12692958	(G/C)
CakSNP5991	*Kabuli*	*Ca_Kabuli_Chr04*	12693000	(T/C)
CakSNP5992	*Kabuli*	*Ca_Kabuli_Chr04*	12740330	(A/G)
CakSNP5993	*Kabuli*	*Ca_Kabuli_Chr04*	12740351	(T/G)
CakSNP5994	*Kabuli*	*Ca_Kabuli_Chr04*	12779146	(A/C)
CakSNP5995	*Kabuli*	*Ca_Kabuli_Chr04*	12780274	(C/T)
CakSNP5996	*Kabuli*	*Ca_Kabuli_Chr04*	12795036	(G/A)
CakSNP5997	*Kabuli*	*Ca_Kabuli_Chr04*	12795037	(G/A)
CakSNP5998	*Kabuli*	*Ca_Kabuli_Chr04*	12869023	(C/T)
CakSNP5999	*Kabuli*	*Ca_Kabuli_Chr04*	12869049	(C/T)
CakSNP6000	*Kabuli*	*Ca_Kabuli_Chr04*	12907910	(C/A)
CakSNP6001	*Kabuli*	*Ca_Kabuli_Chr04*	12908080	(C/T)
CakSNP6002	*Kabuli*	*Ca_Kabuli_Chr04*	12911860	(A/G)
CakSNP6003	*Kabuli*	*Ca_Kabuli_Chr04*	12911874	(T/C)
CakSNP6004	*Kabuli*	*Ca_Kabuli_Chr04*	12911888	(G/C)
CakSNP6005	*Kabuli*	*Ca_Kabuli_Chr04*	12912015	(G/A)
CakSNP6006	*Kabuli*	*Ca_Kabuli_Chr04*	12955130	(G/A)
CakSNP6007	*Kabuli*	*Ca_Kabuli_Chr04*	12973657	(C/A)
CakSNP6008	*Kabuli*	*Ca_Kabuli_Chr04*	12982420	(G/A)
CakSNP6009	*Kabuli*	*Ca_Kabuli_Chr04*	13018788	(C/G)
CakSNP6010	*Kabuli*	*Ca_Kabuli_Chr04*	13020891	(A/G)
CakSNP6011	*Kabuli*	*Ca_Kabuli_Chr04*	13050470	(T/C)
CakSNP6012	*Kabuli*	*Ca_Kabuli_Chr04*	13050516	(A/G)
CakSNP6013	*Kabuli*	*Ca_Kabuli_Chr04*	13050527	(C/T)
CakSNP6014	*Kabuli*	*Ca_Kabuli_Chr04*	13050585	(C/T)
CakSNP6015	*Kabuli*	*Ca_Kabuli_Chr04*	13062744	(T/A)
CakSNP6016	*Kabuli*	*Ca_Kabuli_Chr04*	13062761	(A/T)
CakSNP6017	*Kabuli*	*Ca_Kabuli_Chr04*	13062805	(C/G)
CakSNP6018	*Kabuli*	*Ca_Kabuli_Chr04*	13062972	(A/T)
CakSNP6019	*Kabuli*	*Ca_Kabuli_Chr04*	13063689	(T/A)
CakSNP6020	*Kabuli*	*Ca_Kabuli_Chr04*	13063699	(G/T)
CakSNP6021	*Kabuli*	*Ca_Kabuli_Chr04*	13069574	(T/C)
CakSNP6022	*Kabuli*	*Ca_Kabuli_Chr04*	13071169	(T/A)
CakSNP6023	*Kabuli*	*Ca_Kabuli_Chr04*	13071199	(A/T)
CakSNP6024	*Kabuli*	*Ca_Kabuli_Chr04*	13071230	(T/G)
CakSNP6025	*Kabuli*	*Ca_Kabuli_Chr04*	13072114	(T/A)
CakSNP6026	*Kabuli*	*Ca_Kabuli_Chr04*	13072118	(T/G)

SNP IDs	Cultivars	Chromosomes/scaffolds	Physical positions (bp)	SNPs
CakSNP6027	*Kabuli*	*Ca_Kabuli_Chr04*	13072208	(C/A)
CakSNP6028	*Kabuli*	*Ca_Kabuli_Chr04*	13082999	(A/G)
CakSNP6029	*Kabuli*	*Ca_Kabuli_Chr04*	13102381	(G/A)
CakSNP6030	*Kabuli*	*Ca_Kabuli_Chr04*	13102348	(G/T)
CakSNP6031	*Kabuli*	*Ca_Kabuli_Chr04*	13147948	(A/G)
CakSNP6032	*Kabuli*	*Ca_Kabuli_Chr04*	13147951	(C/T)
CakSNP6033	*Kabuli*	*Ca_Kabuli_Chr04*	13147960	(T/C)
CakSNP6034	*Kabuli*	*Ca_Kabuli_Chr04*	13147965	(A/G)
CakSNP6035	*Kabuli*	*Ca_Kabuli_Chr04*	13148016	(G/A)
CakSNP6036	*Kabuli*	*Ca_Kabuli_Chr04*	13179829	(T/C)
CakSNP6037	*Kabuli*	*Ca_Kabuli_Chr04*	13203008	(A/T)
CakSNP6038	*Kabuli*	*Ca_Kabuli_Chr04*	13229035	(C/T)
CakSNP6039	*Kabuli*	*Ca_Kabuli_Chr04*	13252015	(A/T)
CakSNP6040	*Kabuli*	*Ca_Kabuli_Chr04*	13252077	(C/G)
CakSNP6041	*Kabuli*	*Ca_Kabuli_Chr04*	13252126	(T/C)
CakSNP6042	*Kabuli*	*Ca_Kabuli_Chr04*	13252212	(G/A)
CakSNP6043	*Kabuli*	*Ca_Kabuli_Chr04*	13272282	(T/C)
CakSNP6044	*Kabuli*	*Ca_Kabuli_Chr04*	13282984	(A/C)
CakSNP6045	*Kabuli*	*Ca_Kabuli_Chr04*	13296221	(A/T)
CakSNP6046	*Kabuli*	*Ca_Kabuli_Chr04*	13296217	(G/T)
CakSNP6047	*Kabuli*	*Ca_Kabuli_Chr04*	13310700	(T/C)
CakSNP6048	*Kabuli*	*Ca_Kabuli_Chr04*	13318811	(C/T)
CakSNP6049	*Kabuli*	*Ca_Kabuli_Chr04*	13331280	(A/G)
CakSNP6050	*Kabuli*	*Ca_Kabuli_Chr04*	13332384	(G/A)
CakSNP6051	*Kabuli*	*Ca_Kabuli_Chr04*	13332452	(T/C)
CakSNP6052	*Kabuli*	*Ca_Kabuli_Chr04*	13376456	(A/T)
CakSNP6053	*Kabuli*	*Ca_Kabuli_Chr04*	13463481	(C/T)
CakSNP6054	*Kabuli*	*Ca_Kabuli_Chr04*	13500679	(A/C)
CakSNP6055	*Kabuli*	*Ca_Kabuli_Chr04*	13500693	(G/A)
CakSNP6056	*Kabuli*	*Ca_Kabuli_Chr04*	13588956	(C/T)
CakSNP6057	*Kabuli*	*Ca_Kabuli_Chr04*	13588928	(T/C)
CakSNP6058	*Kabuli*	*Ca_Kabuli_Chr04*	13646571	(T/C)
CakSNP6059	*Kabuli*	*Ca_Kabuli_Chr04*	13646628	(C/T)
CakSNP6060	*Kabuli*	*Ca_Kabuli_Chr04*	13687456	(G/A)
CakSNP6061	*Kabuli*	*Ca_Kabuli_Chr04*	13713380	(G/T)
CakSNP6062	*Kabuli*	*Ca_Kabuli_Chr04*	13713367	(G/C)
CakSNP6063	*Kabuli*	*Ca_Kabuli_Chr04*	13724666	(G/C)
CakSNP6064	*Kabuli*	*Ca_Kabuli_Chr04*	13787448	(A/C)
CakSNP6065	*Kabuli*	*Ca_Kabuli_Chr04*	13787649	(T/C)
CakSNP6066	*Kabuli*	*Ca_Kabuli_Chr04*	13787720	(G/C)
CakSNP6067	*Kabuli*	*Ca_Kabuli_Chr04*	13823667	(T/A)

SNP IDs	Cultivars	Chromosomes/scaffolds	Physical positions (bp)	SNPs
CakSNP6068	*Kabuli*	*Ca_Kabuli_Chr04*	13823672	(G/T)
CakSNP6069	*Kabuli*	*Ca_Kabuli_Chr04*	13823695	(T/C)
CakSNP6070	*Kabuli*	*Ca_Kabuli_Chr04*	13838241	(G/T)
CakSNP6071	*Kabuli*	*Ca_Kabuli_Chr04*	13838796	(A/G)
CakSNP6072	*Kabuli*	*Ca_Kabuli_Chr04*	13839294	(A/G)
CakSNP6073	*Kabuli*	*Ca_Kabuli_Chr04*	13839288	(G/A)
CakSNP6074	*Kabuli*	*Ca_Kabuli_Chr04*	13840191	(T/A)
CakSNP6075	*Kabuli*	*Ca_Kabuli_Chr04*	13840227	(C/A)
CakSNP6076	*Kabuli*	*Ca_Kabuli_Chr04*	13840251	(A/G)
CakSNP6077	*Kabuli*	*Ca_Kabuli_Chr04*	13840484	(T/C)
CakSNP6078	*Kabuli*	*Ca_Kabuli_Chr04*	13841340	(G/T)
CakSNP6079	*Kabuli*	*Ca_Kabuli_Chr04*	13841401	(A/G)
CakSNP6080	*Kabuli*	*Ca_Kabuli_Chr04*	13845581	(A/T)
CakSNP6081	*Kabuli*	*Ca_Kabuli_Chr04*	13845639	(A/C)
CakSNP6082	*Kabuli*	*Ca_Kabuli_Chr04*	13845675	(T/A)
CakSNP6083	*Kabuli*	*Ca_Kabuli_Chr04*	13900013	(A/G)
CakSNP6084	*Kabuli*	*Ca_Kabuli_Chr04*	13923366	(T/C)
CakSNP6085	*Kabuli*	*Ca_Kabuli_Chr04*	13949513	(A/C)
CakSNP6086	*Kabuli*	*Ca_Kabuli_Chr04*	13949580	(C/G)
CakSNP6087	*Kabuli*	*Ca_Kabuli_Chr04*	13954832	(G/A)
CakSNP6088	*Kabuli*	*Ca_Kabuli_Chr04*	13968291	(C/T)
CakSNP6089	*Kabuli*	*Ca_Kabuli_Chr04*	13968397	(A/G)
CakSNP6090	*Kabuli*	*Ca_Kabuli_Chr04*	13968354	(A/G)
CakSNP6091	*Kabuli*	*Ca_Kabuli_Chr04*	13982970	(G/A)
CakSNP6092	*Kabuli*	*Ca_Kabuli_Chr04*	14031548	(A/G)
CakSNP6093	*Kabuli*	*Ca_Kabuli_Chr04*	14056908	(A/G)
CakSNP6094	*Kabuli*	*Ca_Kabuli_Chr04*	14073059	(C/T)
CakSNP6095	*Kabuli*	*Ca_Kabuli_Chr04*	14073056	(C/T)
CakSNP6096	*Kabuli*	*Ca_Kabuli_Chr04*	14073045	(G/A)
CakSNP6097	*Kabuli*	*Ca_Kabuli_Chr04*	14073044	(C/T)
CakSNP6098	*Kabuli*	*Ca_Kabuli_Chr04*	14073004	(C/A)
CakSNP6099	*Kabuli*	*Ca_Kabuli_Chr04*	14082157	(C/A)
CakSNP6100	*Kabuli*	*Ca_Kabuli_Chr04*	14082158	(T/G)
CakSNP6101	*Kabuli*	*Ca_Kabuli_Chr04*	14082186	(G/A)
CakSNP6102	*Kabuli*	*Ca_Kabuli_Chr04*	14082210	(T/C)
CakSNP6103	*Kabuli*	*Ca_Kabuli_Chr04*	14082285	(C/G)
CakSNP6104	*Kabuli*	*Ca_Kabuli_Chr04*	14093027	(A/G)
CakSNP6105	*Kabuli*	*Ca_Kabuli_Chr04*	14104096	(A/C)
CakSNP6106	*Kabuli*	*Ca_Kabuli_Chr04*	14112911	(C/A)
CakSNP6107	*Kabuli*	*Ca_Kabuli_Chr04*	14132659	(A/C)
CakSNP6108	*Kabuli*	*Ca_Kabuli_Chr04*	14149589	(G/A)

**SNP IDs**	**Cultivars**	**Chromosomes/scaffolds**	**Physical positions (bp)**	**SNPs**
CakSNP6109	*Kabuli*	*Ca_Kabuli_Chr04*	14149588	(A/G)
CakSNP6110	*Kabuli*	*Ca_Kabuli_Chr04*	14155978	(A/G)
CakSNP6111	*Kabuli*	*Ca_Kabuli_Chr04*	14156014	(C/T)
CakSNP6112	*Kabuli*	*Ca_Kabuli_Chr04*	14163060	(C/T)
CakSNP6113	*Kabuli*	*Ca_Kabuli_Chr04*	14181527	(A/G)
CakSNP6114	*Kabuli*	*Ca_Kabuli_Chr04*	14189356	(A/C)
CakSNP6115	*Kabuli*	*Ca_Kabuli_Chr04*	14199191	(G/C)
CakSNP6116	*Kabuli*	*Ca_Kabuli_Chr04*	14225284	(A/G)
CakSNP6117	*Kabuli*	*Ca_Kabuli_Chr04*	14270811	(G/A)
CakSNP6118	*Kabuli*	*Ca_Kabuli_Chr04*	14270763	(G/A)
CakSNP6119	*Kabuli*	*Ca_Kabuli_Chr04*	14453942	(T/C)
CakSNP6120	*Kabuli*	*Ca_Kabuli_Chr04*	14515543	(T/A)
CakSNP6121	*Kabuli*	*Ca_Kabuli_Chr04*	14515531	(T/A)
CakSNP6122	*Kabuli*	*Ca_Kabuli_Chr04*	14517424	(C/T)
CakSNP6123	*Kabuli*	*Ca_Kabuli_Chr04*	14529007	(A/G)
CakSNP6124	*Kabuli*	*Ca_Kabuli_Chr04*	14565948	(A/C)
CakSNP6125	*Kabuli*	*Ca_Kabuli_Chr04*	14566064	(G/A)
CakSNP6126	*Kabuli*	*Ca_Kabuli_Chr04*	14566074	(T/A)
CakSNP6127	*Kabuli*	*Ca_Kabuli_Chr04*	14566081	(A/G)
CakSNP6128	*Kabuli*	*Ca_Kabuli_Chr04*	14611177	(A/G)
CakSNP6129	*Kabuli*	*Ca_Kabuli_Chr04*	14657743	(C/T)
CakSNP6130	*Kabuli*	*Ca_Kabuli_Chr04*	14696775	(T/C)
CakSNP6131	*Kabuli*	*Ca_Kabuli_Chr04*	14698825	(A/T)
CakSNP6132	*Kabuli*	*Ca_Kabuli_Chr04*	14699270	(T/C)
CakSNP6133	*Kabuli*	*Ca_Kabuli_Chr04*	14748051	(A/C)
CakSNP6134	*Kabuli*	*Ca_Kabuli_Chr04*	14770484	(C/T)
CakSNP6135	*Kabuli*	*Ca_Kabuli_Chr04*	14770524	(G/C)
CakSNP6136	*Kabuli*	*Ca_Kabuli_Chr04*	14770525	(G/A)
CakSNP6137	*Kabuli*	*Ca_Kabuli_Chr04*	14770532	(C/T)
CakSNP6138	*Kabuli*	*Ca_Kabuli_Chr04*	14785530	(G/A)
CakSNP6139	*Kabuli*	*Ca_Kabuli_Chr04*	14785557	(A/C)
CakSNP6140	*Kabuli*	*Ca_Kabuli_Chr04*	14786093	(C/G)
CakSNP6141	*Kabuli*	*Ca_Kabuli_Chr04*	14831204	(A/T)
CakSNP6142	*Kabuli*	*Ca_Kabuli_Chr04*	14831298	(A/T)
CakSNP6143	*Kabuli*	*Ca_Kabuli_Chr04*	14855406	(T/C)
CakSNP6144	*Kabuli*	*Ca_Kabuli_Chr04*	14855417	(T/A)
CakSNP6145	*Kabuli*	*Ca_Kabuli_Chr04*	14870658	(T/G)
CakSNP6146	*Kabuli*	*Ca_Kabuli_Chr04*	14870641	(G/A)
CakSNP6147	*Kabuli*	*Ca_Kabuli_Chr04*	14989557	(C/T)
CakSNP6148	*Kabuli*	*Ca_Kabuli_Chr04*	14989544	(C/T)
CakSNP6149	*Kabuli*	*Ca_Kabuli_Chr04*	14989526	(G/A)

**SNP IDs**	**Cultivars**	**Chromosomes/scaffolds**	**Physical positions (bp)**	**SNPs**
CakSNP6150	*Kabuli*	*Ca_Kabuli_Chr04*	15025765	(T/A)
CakSNP6151	*Kabuli*	*Ca_Kabuli_Chr04*	15025785	(T/G)
CakSNP6152	*Kabuli*	*Ca_Kabuli_Chr04*	15026058	(C/T)
CakSNP6153	*Kabuli*	*Ca_Kabuli_Chr04*	15036362	(A/G)
CakSNP6154	*Kabuli*	*Ca_Kabuli_Chr04*	15039897	(C/G)
CakSNP6155	*Kabuli*	*Ca_Kabuli_Chr04*	15039905	(T/C)
CakSNP6156	*Kabuli*	*Ca_Kabuli_Chr04*	15039948	(G/C)
CakSNP6157	*Kabuli*	*Ca_Kabuli_Chr04*	15039996	(C/T)
CakSNP6158	*Kabuli*	*Ca_Kabuli_Chr04*	15040072	(A/C)
CakSNP6159	*Kabuli*	*Ca_Kabuli_Chr04*	15053643	(T/C)
CakSNP6160	*Kabuli*	*Ca_Kabuli_Chr04*	15075489	(T/G)
CakSNP6161	*Kabuli*	*Ca_Kabuli_Chr04*	15075502	(G/T)
CakSNP6162	*Kabuli*	*Ca_Kabuli_Chr04*	15159143	(A/G)
CakSNP6163	*Kabuli*	*Ca_Kabuli_Chr04*	15190387	(T/G)
CakSNP6164	*Kabuli*	*Ca_Kabuli_Chr04*	15190423	(T/A)
CakSNP6165	*Kabuli*	*Ca_Kabuli_Chr04*	15212787	(G/A)
CakSNP6166	*Kabuli*	*Ca_Kabuli_Chr04*	15240675	(G/A)
CakSNP6167	*Kabuli*	*Ca_Kabuli_Chr04*	15240678	(T/C)
CakSNP6168	*Kabuli*	*Ca_Kabuli_Chr04*	15299232	(G/A)
CakSNP6169	*Kabuli*	*Ca_Kabuli_Chr04*	15320777	(G/A)
CakSNP6170	*Kabuli*	*Ca_Kabuli_Chr04*	15357651	(C/T)
CakSNP6171	*Kabuli*	*Ca_Kabuli_Chr04*	15357695	(A/G)
CakSNP6172	*Kabuli*	*Ca_Kabuli_Chr04*	15394470	(T/C)
CakSNP6173	*Kabuli*	*Ca_Kabuli_Chr04*	15448826	(G/T)
CakSNP6174	*Kabuli*	*Ca_Kabuli_Chr04*	15449843	(A/G)
CakSNP6175	*Kabuli*	*Ca_Kabuli_Chr04*	15542543	(T/C)
CakSNP6176	*Kabuli*	*Ca_Kabuli_Chr04*	15542597	(A/G)
CakSNP6177	*Kabuli*	*Ca_Kabuli_Chr04*	15552690	(C/T)
CakSNP6178	*Kabuli*	*Ca_Kabuli_Chr04*	15552683	(C/T)
CakSNP6179	*Kabuli*	*Ca_Kabuli_Chr04*	15697128	(A/G)
CakSNP6180	*Kabuli*	*Ca_Kabuli_Chr04*	15697303	(G/T)
CakSNP6181	*Kabuli*	*Ca_Kabuli_Chr04*	15698212	(G/T)
CakSNP6182	*Kabuli*	*Ca_Kabuli_Chr04*	15698179	(G/A)
CakSNP6183	*Kabuli*	*Ca_Kabuli_Chr04*	15698233	(A/C)
CakSNP6184	*Kabuli*	*Ca_Kabuli_Chr04*	15698305	(T/C)
CakSNP6185	*Kabuli*	*Ca_Kabuli_Chr04*	15698405	(G/A)
CakSNP6186	*Kabuli*	*Ca_Kabuli_Chr04*	15698486	(T/C)
CakSNP6187	*Kabuli*	*Ca_Kabuli_Chr04*	15698456	(T/C)
CakSNP6188	*Kabuli*	*Ca_Kabuli_Chr04*	15698444	(C/T)
CakSNP6189	*Kabuli*	*Ca_Kabuli_Chr04*	15698420	(G/C)
CakSNP6190	*Kabuli*	*Ca_Kabuli_Chr04*	15700603	(A/T)

**SNP IDs**	**Cultivars**	**Chromosomes/scaffolds**	**Physical positions (bp)**	**SNPs**
CakSNP6191	*Kabuli*	*Ca_Kabuli_Chr04*	15700630	(G/A)
CakSNP6192	*Kabuli*	*Ca_Kabuli_Chr04*	15713955	(G/A)
CakSNP6193	*Kabuli*	*Ca_Kabuli_Chr04*	15717281	(T/C)
CakSNP6194	*Kabuli*	*Ca_Kabuli_Chr04*	15735993	(T/C)
CakSNP6195	*Kabuli*	*Ca_Kabuli_Chr04*	15736015	(C/T)
CakSNP6196	*Kabuli*	*Ca_Kabuli_Chr04*	15736562	(A/T)
CakSNP6197	*Kabuli*	*Ca_Kabuli_Chr04*	15736619	(T/G)
CakSNP6198	*Kabuli*	*Ca_Kabuli_Chr04*	15736627	(T/C)
CakSNP6199	*Kabuli*	*Ca_Kabuli_Chr04*	15744703	(T/G)
CakSNP6200	*Kabuli*	*Ca_Kabuli_Chr04*	15752567	(G/C)
CakSNP6201	*Kabuli*	*Ca_Kabuli_Chr04*	15766341	(A/G)
CakSNP6202	*Kabuli*	*Ca_Kabuli_Chr04*	15766360	(G/A)
CakSNP6203	*Kabuli*	*Ca_Kabuli_Chr04*	15772235	(C/A)
CakSNP6204	*Kabuli*	*Ca_Kabuli_Chr04*	15772275	(A/G)
CakSNP6205	*Kabuli*	*Ca_Kabuli_Chr04*	15821282	(G/A)
CakSNP6206	*Kabuli*	*Ca_Kabuli_Chr04*	15821311	(T/A)
CakSNP6207	*Kabuli*	*Ca_Kabuli_Chr04*	15825621	(A/T)
CakSNP6208	*Kabuli*	*Ca_Kabuli_Chr04*	15860388	(A/G)
CakSNP6209	*Kabuli*	*Ca_Kabuli_Chr04*	15860359	(C/A)
CakSNP6210	*Kabuli*	*Ca_Kabuli_Chr04*	15900602	(G/A)
CakSNP6211	*Kabuli*	*Ca_Kabuli_Chr04*	15925936	(T/G)
CakSNP6212	*Kabuli*	*Ca_Kabuli_Chr04*	15926160	(A/G)
CakSNP6213	*Kabuli*	*Ca_Kabuli_Chr04*	15934607	(A/T)
CakSNP6214	*Kabuli*	*Ca_Kabuli_Chr04*	15934901	(T/G)
CakSNP6215	*Kabuli*	*Ca_Kabuli_Chr04*	15957070	(T/A)
CakSNP6216	*Kabuli*	*Ca_Kabuli_Chr04*	16027272	(G/A)
CakSNP6217	*Kabuli*	*Ca_Kabuli_Chr04*	16027292	(A/C)
CakSNP6218	*Kabuli*	*Ca_Kabuli_Chr04*	16027368	(G/T)
CakSNP6219	*Kabuli*	*Ca_Kabuli_Chr04*	16027299	(T/A)
CakSNP6220	*Kabuli*	*Ca_Kabuli_Chr04*	16045694	(T/G)
CakSNP6221	*Kabuli*	*Ca_Kabuli_Chr04*	16045870	(G/A)
CakSNP6222	*Kabuli*	*Ca_Kabuli_Chr04*	16046928	(T/C)
CakSNP6223	*Kabuli*	*Ca_Kabuli_Chr04*	16278600	(C/T)
CakSNP6224	*Kabuli*	*Ca_Kabuli_Chr04*	16278671	(T/A)
CakSNP6225	*Kabuli*	*Ca_Kabuli_Chr04*	16446305	(T/C)
CakSNP6226	*Kabuli*	*Ca_Kabuli_Chr04*	16446341	(T/G)
CakSNP6227	*Kabuli*	*Ca_Kabuli_Chr04*	16446336	(C/G)
CakSNP6228	*Kabuli*	*Ca_Kabuli_Chr04*	16446956	(T/C)
CakSNP6229	*Kabuli*	*Ca_Kabuli_Chr04*	16457940	(G/A)
CakSNP6230	*Kabuli*	*Ca_Kabuli_Chr04*	16501291	(T/A)
CakSNP6231	*Kabuli*	*Ca_Kabuli_Chr04*	16501306	(T/A)

**SNP IDs**	**Cultivars**	**Chromosomes/scaffolds**	**Physical positions (bp)**	**SNPs**
CakSNP6232	*Kabuli*	*Ca_Kabuli_Chr04*	16501354	(C/T)
CakSNP6233	*Kabuli*	*Ca_Kabuli_Chr04*	16501782	(G/A)
CakSNP6234	*Kabuli*	*Ca_Kabuli_Chr04*	16501781	(C/A)
CakSNP6235	*Kabuli*	*Ca_Kabuli_Chr04*	16501769	(C/T)
CakSNP6236	*Kabuli*	*Ca_Kabuli_Chr04*	16501738	(G/A)
CakSNP6237	*Kabuli*	*Ca_Kabuli_Chr04*	16501716	(T/C)
CakSNP6238	*Kabuli*	*Ca_Kabuli_Chr04*	16505159	(A/G)
CakSNP6239	*Kabuli*	*Ca_Kabuli_Chr04*	16536754	(C/T)
CakSNP6240	*Kabuli*	*Ca_Kabuli_Chr04*	16571288	(T/C)
CakSNP6241	*Kabuli*	*Ca_Kabuli_Chr04*	16586863	(G/C)
CakSNP6242	*Kabuli*	*Ca_Kabuli_Chr04*	16586913	(G/C)
CakSNP6243	*Kabuli*	*Ca_Kabuli_Chr04*	16586929	(G/C)
CakSNP6244	*Kabuli*	*Ca_Kabuli_Chr04*	16619312	(T/C)
CakSNP6245	*Kabuli*	*Ca_Kabuli_Chr04*	16622514	(C/A)
CakSNP6246	*Kabuli*	*Ca_Kabuli_Chr04*	16625148	(T/G)
CakSNP6247	*Kabuli*	*Ca_Kabuli_Chr04*	16625270	(T/G)
CakSNP6248	*Kabuli*	*Ca_Kabuli_Chr04*	16625221	(G/A)
CakSNP6249	*Kabuli*	*Ca_Kabuli_Chr04*	16628559	(T/G)
CakSNP6250	*Kabuli*	*Ca_Kabuli_Chr04*	16628591	(T/G)
CakSNP6251	*Kabuli*	*Ca_Kabuli_Chr04*	16628594	(T/G)
CakSNP6252	*Kabuli*	*Ca_Kabuli_Chr04*	16628542	(G/A)
CakSNP6253	*Kabuli*	*Ca_Kabuli_Chr04*	16628540	(G/A)
CakSNP6254	*Kabuli*	*Ca_Kabuli_Chr04*	16628536	(T/A)
CakSNP6255	*Kabuli*	*Ca_Kabuli_Chr04*	16628529	(C/A)
CakSNP6256	*Kabuli*	*Ca_Kabuli_Chr04*	16628527	(G/A)
CakSNP6257	*Kabuli*	*Ca_Kabuli_Chr04*	16628523	(T/C)
CakSNP6258	*Kabuli*	*Ca_Kabuli_Chr04*	16629560	(G/T)
CakSNP6259	*Kabuli*	*Ca_Kabuli_Chr04*	16629559	(T/G)
CakSNP6260	*Kabuli*	*Ca_Kabuli_Chr04*	16629638	(C/A)
CakSNP6261	*Kabuli*	*Ca_Kabuli_Chr04*	16630385	(G/T)
CakSNP6262	*Kabuli*	*Ca_Kabuli_Chr04*	16669916	(T/C)
CakSNP6263	*Kabuli*	*Ca_Kabuli_Chr04*	16749740	(A/G)
CakSNP6264	*Kabuli*	*Ca_Kabuli_Chr04*	16750416	(A/G)
CakSNP6265	*Kabuli*	*Ca_Kabuli_Chr04*	16751205	(T/C)
CakSNP6266	*Kabuli*	*Ca_Kabuli_Chr04*	16789476	(G/A)
CakSNP6267	*Kabuli*	*Ca_Kabuli_Chr04*	16789532	(A/C)
CakSNP6268	*Kabuli*	*Ca_Kabuli_Chr04*	16820726	(C/A)
CakSNP6269	*Kabuli*	*Ca_Kabuli_Chr04*	16837336	(T/A)
CakSNP6270	*Kabuli*	*Ca_Kabuli_Chr04*	16839081	(T/A)
CakSNP6271	*Kabuli*	*Ca_Kabuli_Chr04*	16845299	(G/A)
CakSNP6272	*Kabuli*	*Ca_Kabuli_Chr04*	16889650	(A/T)

SNP IDs	Cultivars	Chromosomes/scaffolds	Physical positions (bp)	SNPs
CakSNP6273	*Kabuli*	*Ca_Kabuli_Chr04*	16926545	(T/C)
CakSNP6274	*Kabuli*	*Ca_Kabuli_Chr04*	16926527	(T/A)
CakSNP6275	*Kabuli*	*Ca_Kabuli_Chr04*	16926614	(A/T)
CakSNP6276	*Kabuli*	*Ca_Kabuli_Chr04*	17028303	(A/G)
CakSNP6277	*Kabuli*	*Ca_Kabuli_Chr04*	17028368	(G/A)
CakSNP6278	*Kabuli*	*Ca_Kabuli_Chr04*	17028523	(C/T)
CakSNP6279	*Kabuli*	*Ca_Kabuli_Chr04*	17028495	(T/A)
CakSNP6280	*Kabuli*	*Ca_Kabuli_Chr04*	17032676	(T/C)
CakSNP6281	*Kabuli*	*Ca_Kabuli_Chr04*	17032673	(C/T)
CakSNP6282	*Kabuli*	*Ca_Kabuli_Chr04*	17059830	(G/A)
CakSNP6283	*Kabuli*	*Ca_Kabuli_Chr04*	17059833	(A/T)
CakSNP6284	*Kabuli*	*Ca_Kabuli_Chr04*	17059852	(T/C)
CakSNP6285	*Kabuli*	*Ca_Kabuli_Chr04*	17059866	(G/A)
CakSNP6286	*Kabuli*	*Ca_Kabuli_Chr04*	17059872	(A/G)
CakSNP6287	*Kabuli*	*Ca_Kabuli_Chr04*	17059887	(G/A)
CakSNP6288	*Kabuli*	*Ca_Kabuli_Chr04*	17093090	(C/G)
CakSNP6289	*Kabuli*	*Ca_Kabuli_Chr04*	17147875	(C/T)
CakSNP6290	*Kabuli*	*Ca_Kabuli_Chr04*	17147926	(T/C)
CakSNP6291	*Kabuli*	*Ca_Kabuli_Chr04*	17187560	(C/G)
CakSNP6292	*Kabuli*	*Ca_Kabuli_Chr04*	17191562	(T/A)
CakSNP6293	*Kabuli*	*Ca_Kabuli_Chr04*	17199316	(T/C)
CakSNP6294	*Kabuli*	*Ca_Kabuli_Chr04*	17199477	(G/A)
CakSNP6295	*Kabuli*	*Ca_Kabuli_Chr04*	17199432	(G/A)
CakSNP6296	*Kabuli*	*Ca_Kabuli_Chr04*	17202770	(A/G)
CakSNP6297	*Kabuli*	*Ca_Kabuli_Chr04*	17225334	(G/A)
CakSNP6298	*Kabuli*	*Ca_Kabuli_Chr04*	17264248	(C/A)
CakSNP6299	*Kabuli*	*Ca_Kabuli_Chr04*	17264592	(G/A)
CakSNP6300	*Kabuli*	*Ca_Kabuli_Chr04*	17264621	(C/G)
CakSNP6301	*Kabuli*	*Ca_Kabuli_Chr04*	17264637	(C/T)
CakSNP6302	*Kabuli*	*Ca_Kabuli_Chr04*	17282119	(T/C)
CakSNP6303	*Kabuli*	*Ca_Kabuli_Chr04*	17282139	(G/C)
CakSNP6304	*Kabuli*	*Ca_Kabuli_Chr04*	17282148	(G/C)
CakSNP6305	*Kabuli*	*Ca_Kabuli_Chr04*	17282336	(G/T)
CakSNP6306	*Kabuli*	*Ca_Kabuli_Chr04*	17282345	(G/C)
CakSNP6307	*Kabuli*	*Ca_Kabuli_Chr04*	17289594	(G/C)
CakSNP6308	*Kabuli*	*Ca_Kabuli_Chr04*	17297498	(G/T)
CakSNP6309	*Kabuli*	*Ca_Kabuli_Chr04*	17297567	(G/A)
CakSNP6310	*Kabuli*	*Ca_Kabuli_Chr04*	17297563	(A/G)
CakSNP6311	*Kabuli*	*Ca_Kabuli_Chr04*	17309656	(A/C)
CakSNP6312	*Kabuli*	*Ca_Kabuli_Chr04*	17345431	(T/C)
CakSNP6313	*Kabuli*	*Ca_Kabuli_Chr04*	17345436	(G/T)

SNP IDs	Cultivars	Chromosomes/scaffolds	Physical positions (bp)	SNPs
CakSNP6314	*Kabuli*	*Ca_Kabuli_Chr04*	17345439	(G/A)
CakSNP6315	*Kabuli*	*Ca_Kabuli_Chr04*	17345448	(T/G)
CakSNP6316	*Kabuli*	*Ca_Kabuli_Chr04*	17345500	(G/A)
CakSNP6317	*Kabuli*	*Ca_Kabuli_Chr04*	17345483	(C/A)
CakSNP6318	*Kabuli*	*Ca_Kabuli_Chr04*	17345454	(A/G)
CakSNP6319	*Kabuli*	*Ca_Kabuli_Chr04*	17358736	(C/T)
CakSNP6320	*Kabuli*	*Ca_Kabuli_Chr04*	17361143	(T/A)
CakSNP6321	*Kabuli*	*Ca_Kabuli_Chr04*	17396325	(A/G)
CakSNP6322	*Kabuli*	*Ca_Kabuli_Chr04*	17396349	(T/G)
CakSNP6323	*Kabuli*	*Ca_Kabuli_Chr04*	17396352	(T/G)
CakSNP6324	*Kabuli*	*Ca_Kabuli_Chr04*	17429456	(A/T)
CakSNP6325	*Kabuli*	*Ca_Kabuli_Chr04*	17429457	(A/T)
CakSNP6326	*Kabuli*	*Ca_Kabuli_Chr04*	17429468	(T/C)
CakSNP6327	*Kabuli*	*Ca_Kabuli_Chr04*	17429747	(G/A)
CakSNP6328	*Kabuli*	*Ca_Kabuli_Chr04*	17429742	(A/G)
CakSNP6329	*Kabuli*	*Ca_Kabuli_Chr04*	17429735	(A/G)
CakSNP6330	*Kabuli*	*Ca_Kabuli_Chr04*	17429699	(G/C)
CakSNP6331	*Kabuli*	*Ca_Kabuli_Chr04*	17433782	(G/A)
CakSNP6332	*Kabuli*	*Ca_Kabuli_Chr04*	17433914	(G/A)
CakSNP6333	*Kabuli*	*Ca_Kabuli_Chr04*	17444578	(A/C)
CakSNP6334	*Kabuli*	*Ca_Kabuli_Chr04*	17472129	(T/A)
CakSNP6335	*Kabuli*	*Ca_Kabuli_Chr04*	17472992	(T/G)
CakSNP6336	*Kabuli*	*Ca_Kabuli_Chr04*	17473174	(C/T)
CakSNP6337	*Kabuli*	*Ca_Kabuli_Chr04*	17473237	(T/C)
CakSNP6338	*Kabuli*	*Ca_Kabuli_Chr04*	17473234	(C/A)
CakSNP6339	*Kabuli*	*Ca_Kabuli_Chr04*	17603613	(T/C)
CakSNP6340	*Kabuli*	*Ca_Kabuli_Chr04*	17603677	(G/C)
CakSNP6341	*Kabuli*	*Ca_Kabuli_Chr04*	17603689	(T/C)
CakSNP6342	*Kabuli*	*Ca_Kabuli_Chr04*	17603725	(T/C)
CakSNP6343	*Kabuli*	*Ca_Kabuli_Chr04*	17609554	(A/T)
CakSNP6344	*Kabuli*	*Ca_Kabuli_Chr04*	17621035	(G/T)
CakSNP6345	*Kabuli*	*Ca_Kabuli_Chr04*	17621609	(G/A)
CakSNP6346	*Kabuli*	*Ca_Kabuli_Chr04*	17647558	(G/A)
CakSNP6347	*Kabuli*	*Ca_Kabuli_Chr04*	17652735	(T/C)
CakSNP6348	*Kabuli*	*Ca_Kabuli_Chr04*	17685058	(T/G)
CakSNP6349	*Kabuli*	*Ca_Kabuli_Chr04*	17685113	(C/A)
CakSNP6350	*Kabuli*	*Ca_Kabuli_Chr04*	17718540	(C/T)
CakSNP6351	*Kabuli*	*Ca_Kabuli_Chr04*	17718536	(C/A)
CakSNP6352	*Kabuli*	*Ca_Kabuli_Chr04*	17718894	(G/A)
CakSNP6353	*Kabuli*	*Ca_Kabuli_Chr04*	17718922	(T/G)
CakSNP6354	*Kabuli*	*Ca_Kabuli_Chr04*	17718997	(C/A)

SNP IDs	Cultivars	Chromosomes/scaffolds	Physical positions (bp)	SNPs
CakSNP6355	*Kabuli*	*Ca_Kabuli_Chr04*	17729988	(A/C)
CakSNP6356	*Kabuli*	*Ca_Kabuli_Chr04*	17748171	(A/T)
CakSNP6357	*Kabuli*	*Ca_Kabuli_Chr04*	17752013	(C/T)
CakSNP6358	*Kabuli*	*Ca_Kabuli_Chr04*	17760841	(A/G)
CakSNP6359	*Kabuli*	*Ca_Kabuli_Chr04*	17760853	(T/G)
CakSNP6360	*Kabuli*	*Ca_Kabuli_Chr04*	17768507	(T/C)
CakSNP6361	*Kabuli*	*Ca_Kabuli_Chr04*	17771708	(C/G)
CakSNP6362	*Kabuli*	*Ca_Kabuli_Chr04*	17784792	(T/A)
CakSNP6363	*Kabuli*	*Ca_Kabuli_Chr04*	17826472	(G/A)
CakSNP6364	*Kabuli*	*Ca_Kabuli_Chr04*	17826533	(A/C)
CakSNP6365	*Kabuli*	*Ca_Kabuli_Chr04*	17826601	(T/C)
CakSNP6366	*Kabuli*	*Ca_Kabuli_Chr04*	17826580	(C/G)
CakSNP6367	*Kabuli*	*Ca_Kabuli_Chr04*	17826543	(T/C)
CakSNP6368	*Kabuli*	*Ca_Kabuli_Chr04*	17843480	(G/T)
CakSNP6369	*Kabuli*	*Ca_Kabuli_Chr04*	17847737	(G/A)
CakSNP6370	*Kabuli*	*Ca_Kabuli_Chr04*	17847854	(T/C)
CakSNP6371	*Kabuli*	*Ca_Kabuli_Chr04*	17856307	(T/C)
CakSNP6372	*Kabuli*	*Ca_Kabuli_Chr04*	17856311	(T/G)
CakSNP6373	*Kabuli*	*Ca_Kabuli_Chr04*	17856334	(A/G)
CakSNP6374	*Kabuli*	*Ca_Kabuli_Chr04*	17857753	(C/T)
CakSNP6375	*Kabuli*	*Ca_Kabuli_Chr04*	17858122	(T/G)
CakSNP6376	*Kabuli*	*Ca_Kabuli_Chr04*	17858133	(C/T)
CakSNP6377	*Kabuli*	*Ca_Kabuli_Chr04*	17858135	(G/A)
CakSNP6378	*Kabuli*	*Ca_Kabuli_Chr04*	17858140	(A/C)
CakSNP6379	*Kabuli*	*Ca_Kabuli_Chr04*	17858143	(A/T)
CakSNP6380	*Kabuli*	*Ca_Kabuli_Chr04*	17858144	(G/C)
CakSNP6381	*Kabuli*	*Ca_Kabuli_Chr04*	17858152	(A/T)
CakSNP6382	*Kabuli*	*Ca_Kabuli_Chr04*	17858159	(C/A)
CakSNP6383	*Kabuli*	*Ca_Kabuli_Chr04*	17858226	(C/T)
CakSNP6384	*Kabuli*	*Ca_Kabuli_Chr04*	17871769	(C/A)
CakSNP6385	*Kabuli*	*Ca_Kabuli_Chr04*	17871763	(T/C)
CakSNP6386	*Kabuli*	*Ca_Kabuli_Chr04*	17871804	(T/C)
CakSNP6387	*Kabuli*	*Ca_Kabuli_Chr04*	17871828	(A/C)
CakSNP6388	*Kabuli*	*Ca_Kabuli_Chr04*	17871919	(A/G)
CakSNP6389	*Kabuli*	*Ca_Kabuli_Chr04*	17871915	(T/A)
CakSNP6390	*Kabuli*	*Ca_Kabuli_Chr04*	17913731	(A/C)
CakSNP6391	*Kabuli*	*Ca_Kabuli_Chr04*	17913726	(G/A)
CakSNP6392	*Kabuli*	*Ca_Kabuli_Chr04*	17913719	(G/C)
CakSNP6393	*Kabuli*	*Ca_Kabuli_Chr04*	17913698	(C/T)
CakSNP6394	*Kabuli*	*Ca_Kabuli_Chr04*	17913682	(C/A)
CakSNP6395	*Kabuli*	*Ca_Kabuli_Chr04*	17920616	(C/G)

SNP IDs	Cultivars	Chromosomes/scaffolds	Physical positions (bp)	SNPs
CakSNP6396	*Kabuli*	*Ca_Kabuli_Chr04*	17931955	(G/A)
CakSNP6397	*Kabuli*	*Ca_Kabuli_Chr04*	17970218	(T/G)
CakSNP6398	*Kabuli*	*Ca_Kabuli_Chr04*	17970221	(C/T)
CakSNP6399	*Kabuli*	*Ca_Kabuli_Chr04*	17970225	(T/C)
CakSNP6400	*Kabuli*	*Ca_Kabuli_Chr04*	17970232	(A/G)
CakSNP6401	*Kabuli*	*Ca_Kabuli_Chr04*	17970280	(T/A)
CakSNP6402	*Kabuli*	*Ca_Kabuli_Chr04*	18053756	(A/G)
CakSNP6403	*Kabuli*	*Ca_Kabuli_Chr04*	18070293	(G/T)
CakSNP6404	*Kabuli*	*Ca_Kabuli_Chr04*	18071114	(C/A)
CakSNP6405	*Kabuli*	*Ca_Kabuli_Chr04*	18071324	(G/A)
CakSNP6406	*Kabuli*	*Ca_Kabuli_Chr04*	18089423	(G/C)
CakSNP6407	*Kabuli*	*Ca_Kabuli_Chr04*	18089422	(T/C)
CakSNP6408	*Kabuli*	*Ca_Kabuli_Chr04*	18117833	(T/C)
CakSNP6409	*Kabuli*	*Ca_Kabuli_Chr04*	18222762	(T/C)
CakSNP6410	*Kabuli*	*Ca_Kabuli_Chr04*	18231443	(T/C)
CakSNP6411	*Kabuli*	*Ca_Kabuli_Chr04*	18231463	(T/C)
CakSNP6412	*Kabuli*	*Ca_Kabuli_Chr04*	18231466	(T/C)
CakSNP6413	*Kabuli*	*Ca_Kabuli_Chr04*	18242020	(C/G)
CakSNP6414	*Kabuli*	*Ca_Kabuli_Chr04*	18241995	(C/G)
CakSNP6415	*Kabuli*	*Ca_Kabuli_Chr04*	18427385	(T/G)
CakSNP6416	*Kabuli*	*Ca_Kabuli_Chr04*	18427384	(C/T)
CakSNP6417	*Kabuli*	*Ca_Kabuli_Chr04*	18429046	(G/T)
CakSNP6418	*Kabuli*	*Ca_Kabuli_Chr04*	18429139	(C/T)
CakSNP6419	*Kabuli*	*Ca_Kabuli_Chr04*	18485782	(T/C)
CakSNP6420	*Kabuli*	*Ca_Kabuli_Chr04*	18489456	(G/C)
CakSNP6421	*Kabuli*	*Ca_Kabuli_Chr04*	18495186	(A/G)
CakSNP6422	*Kabuli*	*Ca_Kabuli_Chr04*	18545489	(A/T)
CakSNP6423	*Kabuli*	*Ca_Kabuli_Chr04*	18546209	(T/G)
CakSNP6424	*Kabuli*	*Ca_Kabuli_Chr04*	18625387	(G/A)
CakSNP6425	*Kabuli*	*Ca_Kabuli_Chr04*	18628582	(A/G)
CakSNP6426	*Kabuli*	*Ca_Kabuli_Chr04*	18710941	(T/G)
CakSNP6427	*Kabuli*	*Ca_Kabuli_Chr04*	18719417	(A/G)
CakSNP6428	*Kabuli*	*Ca_Kabuli_Chr04*	18721801	(G/A)
CakSNP6429	*Kabuli*	*Ca_Kabuli_Chr04*	18779396	(A/C)
CakSNP6430	*Kabuli*	*Ca_Kabuli_Chr04*	18812610	(T/C)
CakSNP6431	*Kabuli*	*Ca_Kabuli_Chr04*	18880757	(A/G)
CakSNP6432	*Kabuli*	*Ca_Kabuli_Chr04*	18904060	(G/A)
CakSNP6433	*Kabuli*	*Ca_Kabuli_Chr04*	18912038	(G/C)
CakSNP6434	*Kabuli*	*Ca_Kabuli_Chr04*	18912137	(T/C)
CakSNP6435	*Kabuli*	*Ca_Kabuli_Chr04*	18912124	(C/A)
CakSNP6436	*Kabuli*	*Ca_Kabuli_Chr04*	18912142	(A/G)

SNP IDs	Cultivars	Chromosomes/scaffolds	Physical positions (bp)	SNPs
CakSNP6437	*Kabuli*	*Ca_Kabuli_Chr04*	18923641	(G/T)
CakSNP6438	*Kabuli*	*Ca_Kabuli_Chr04*	18953354	(C/A)
CakSNP6439	*Kabuli*	*Ca_Kabuli_Chr04*	18953423	(A/C)
CakSNP6440	*Kabuli*	*Ca_Kabuli_Chr04*	18953511	(C/G)
CakSNP6441	*Kabuli*	*Ca_Kabuli_Chr04*	18998727	(C/T)
CakSNP6442	*Kabuli*	*Ca_Kabuli_Chr04*	19005285	(T/G)
CakSNP6443	*Kabuli*	*Ca_Kabuli_Chr04*	19005416	(T/G)
CakSNP6444	*Kabuli*	*Ca_Kabuli_Chr04*	19006493	(C/T)
CakSNP6445	*Kabuli*	*Ca_Kabuli_Chr04*	19024709	(C/T)
CakSNP6446	*Kabuli*	*Ca_Kabuli_Chr04*	19024699	(T/C)
CakSNP6447	*Kabuli*	*Ca_Kabuli_Chr04*	19206563	(A/G)
CakSNP6448	*Kabuli*	*Ca_Kabuli_Chr04*	19206581	(C/T)
CakSNP6449	*Kabuli*	*Ca_Kabuli_Chr04*	19206611	(T/C)
CakSNP6450	*Kabuli*	*Ca_Kabuli_Chr04*	19206613	(T/G)
CakSNP6451	*Kabuli*	*Ca_Kabuli_Chr04*	19206678	(A/T)
CakSNP6452	*Kabuli*	*Ca_Kabuli_Chr04*	19207121	(A/T)
CakSNP6453	*Kabuli*	*Ca_Kabuli_Chr04*	19227691	(G/C)
CakSNP6454	*Kabuli*	*Ca_Kabuli_Chr04*	19263964	(G/C)
CakSNP6455	*Kabuli*	*Ca_Kabuli_Chr04*	19263967	(T/G)
CakSNP6456	*Kabuli*	*Ca_Kabuli_Chr04*	19267581	(A/G)
CakSNP6457	*Kabuli*	*Ca_Kabuli_Chr04*	19481258	(A/G)
CakSNP6458	*Kabuli*	*Ca_Kabuli_Chr04*	19540632	(A/C)
CakSNP6459	*Kabuli*	*Ca_Kabuli_Chr04*	19540646	(T/G)
CakSNP6460	*Kabuli*	*Ca_Kabuli_Chr04*	19579390	(G/T)
CakSNP6461	*Kabuli*	*Ca_Kabuli_Chr04*	19579401	(C/A)
CakSNP6462	*Kabuli*	*Ca_Kabuli_Chr04*	19579423	(A/G)
CakSNP6463	*Kabuli*	*Ca_Kabuli_Chr04*	19579433	(G/A)
CakSNP6464	*Kabuli*	*Ca_Kabuli_Chr04*	19579435	(A/G)
CakSNP6465	*Kabuli*	*Ca_Kabuli_Chr04*	19579436	(A/T)
CakSNP6466	*Kabuli*	*Ca_Kabuli_Chr04*	19579442	(C/A)
CakSNP6467	*Kabuli*	*Ca_Kabuli_Chr04*	19649499	(A/G)
CakSNP6468	*Kabuli*	*Ca_Kabuli_Chr04*	19668499	(T/C)
CakSNP6469	*Kabuli*	*Ca_Kabuli_Chr04*	19668502	(T/G)
CakSNP6470	*Kabuli*	*Ca_Kabuli_Chr04*	19668595	(T/C)
CakSNP6471	*Kabuli*	*Ca_Kabuli_Chr04*	19688016	(A/G)
CakSNP6472	*Kabuli*	*Ca_Kabuli_Chr04*	19712406	(C/T)
CakSNP6473	*Kabuli*	*Ca_Kabuli_Chr04*	19713311	(T/G)
CakSNP6474	*Kabuli*	*Ca_Kabuli_Chr04*	19734940	(G/A)
CakSNP6475	*Kabuli*	*Ca_Kabuli_Chr04*	19844376	(A/G)
CakSNP6476	*Kabuli*	*Ca_Kabuli_Chr04*	19869729	(G/T)
CakSNP6477	*Kabuli*	*Ca_Kabuli_Chr04*	19869722	(T/G)

**SNP IDs**	**Cultivars**	**Chromosomes/scaffolds**	**Physical positions (bp)**	**SNPs**
CakSNP6478	*Kabuli*	*Ca_Kabuli_Chr04*	20218430	(T/G)
CakSNP6479	*Kabuli*	*Ca_Kabuli_Chr04*	20308992	(C/A)
CakSNP6480	*Kabuli*	*Ca_Kabuli_Chr04*	20309151	(C/T)
CakSNP6481	*Kabuli*	*Ca_Kabuli_Chr04*	20398548	(T/G)
CakSNP6482	*Kabuli*	*Ca_Kabuli_Chr04*	20398685	(A/C)
CakSNP6483	*Kabuli*	*Ca_Kabuli_Chr04*	20402361	(A/G)
CakSNP6484	*Kabuli*	*Ca_Kabuli_Chr04*	20402391	(A/G)
CakSNP6485	*Kabuli*	*Ca_Kabuli_Chr04*	20412329	(A/T)
CakSNP6486	*Kabuli*	*Ca_Kabuli_Chr04*	20436136	(G/A)
CakSNP6487	*Kabuli*	*Ca_Kabuli_Chr04*	20444953	(T/C)
CakSNP6488	*Kabuli*	*Ca_Kabuli_Chr04*	20599050	(A/G)
CakSNP6489	*Kabuli*	*Ca_Kabuli_Chr04*	20599058	(T/C)
CakSNP6490	*Kabuli*	*Ca_Kabuli_Chr04*	20599081	(C/T)
CakSNP6491	*Kabuli*	*Ca_Kabuli_Chr04*	20599091	(T/G)
CakSNP6492	*Kabuli*	*Ca_Kabuli_Chr04*	20656170	(T/G)
CakSNP6493	*Kabuli*	*Ca_Kabuli_Chr04*	20669174	(T/C)
CakSNP6494	*Kabuli*	*Ca_Kabuli_Chr04*	20669442	(G/A)
CakSNP6495	*Kabuli*	*Ca_Kabuli_Chr04*	20696913	(A/G)
CakSNP6496	*Kabuli*	*Ca_Kabuli_Chr04*	20696952	(G/T)
CakSNP6497	*Kabuli*	*Ca_Kabuli_Chr04*	20696979	(C/T)
CakSNP6498	*Kabuli*	*Ca_Kabuli_Chr04*	20697338	(T/A)
CakSNP6499	*Kabuli*	*Ca_Kabuli_Chr04*	20697408	(A/G)
CakSNP6500	*Kabuli*	*Ca_Kabuli_Chr04*	20774180	(C/T)
CakSNP6501	*Kabuli*	*Ca_Kabuli_Chr04*	20905105	(G/A)
CakSNP6502	*Kabuli*	*Ca_Kabuli_Chr04*	21096178	(G/A)
CakSNP6503	*Kabuli*	*Ca_Kabuli_Chr04*	21385308	(A/T)
CakSNP6504	*Kabuli*	*Ca_Kabuli_Chr04*	21830534	(C/T)
CakSNP6505	*Kabuli*	*Ca_Kabuli_Chr04*	21835459	(T/A)
CakSNP6506	*Kabuli*	*Ca_Kabuli_Chr04*	21835457	(T/A)
CakSNP6507	*Kabuli*	*Ca_Kabuli_Chr04*	21835454	(C/A)
CakSNP6508	*Kabuli*	*Ca_Kabuli_Chr04*	21835446	(T/A)
CakSNP6509	*Kabuli*	*Ca_Kabuli_Chr04*	21931559	(A/G)
CakSNP6510	*Kabuli*	*Ca_Kabuli_Chr04*	21931558	(G/A)
CakSNP6511	*Kabuli*	*Ca_Kabuli_Chr04*	21931541	(C/T)
CakSNP6512	*Kabuli*	*Ca_Kabuli_Chr04*	22079566	(G/A)
CakSNP6513	*Kabuli*	*Ca_Kabuli_Chr04*	22237989	(A/T)
CakSNP6514	*Kabuli*	*Ca_Kabuli_Chr04*	22237971	(C/T)
CakSNP6515	*Kabuli*	*Ca_Kabuli_Chr04*	22288433	(T/C)
CakSNP6516	*Kabuli*	*Ca_Kabuli_Chr04*	22293495	(T/C)
CakSNP6517	*Kabuli*	*Ca_Kabuli_Chr04*	22850596	(C/G)
CakSNP6518	*Kabuli*	*Ca_Kabuli_Chr04*	22859422	(G/A)

**SNP IDs**	**Cultivars**	**Chromosomes/scaffolds**	**Physical positions (bp)**	**SNPs**
CakSNP6519	*Kabuli*	*Ca_Kabuli_Chr04*	22860281	(A/C)
CakSNP6520	*Kabuli*	*Ca_Kabuli_Chr04*	22865436	(A/T)
CakSNP6521	*Kabuli*	*Ca_Kabuli_Chr04*	22865390	(G/A)
CakSNP6522	*Kabuli*	*Ca_Kabuli_Chr04*	22865389	(C/T)
CakSNP6523	*Kabuli*	*Ca_Kabuli_Chr04*	22908360	(A/G)
CakSNP6524	*Kabuli*	*Ca_Kabuli_Chr04*	22971660	(G/A)
CakSNP6525	*Kabuli*	*Ca_Kabuli_Chr04*	22971766	(A/G)
CakSNP6526	*Kabuli*	*Ca_Kabuli_Chr04*	22971746	(G/A)
CakSNP6527	*Kabuli*	*Ca_Kabuli_Chr04*	22971744	(A/C)
CakSNP6528	*Kabuli*	*Ca_Kabuli_Chr04*	22971847	(C/A)
CakSNP6529	*Kabuli*	*Ca_Kabuli_Chr04*	22971853	(C/A)
CakSNP6530	*Kabuli*	*Ca_Kabuli_Chr04*	22971884	(G/A)
CakSNP6531	*Kabuli*	*Ca_Kabuli_Chr04*	23157656	(C/G)
CakSNP6532	*Kabuli*	*Ca_Kabuli_Chr04*	23267842	(T/C)
CakSNP6533	*Kabuli*	*Ca_Kabuli_Chr04*	23267856	(C/T)
CakSNP6534	*Kabuli*	*Ca_Kabuli_Chr04*	23267858	(C/T)
CakSNP6535	*Kabuli*	*Ca_Kabuli_Chr04*	23267859	(T/C)
CakSNP6536	*Kabuli*	*Ca_Kabuli_Chr04*	23314624	(C/T)
CakSNP6537	*Kabuli*	*Ca_Kabuli_Chr04*	23314665	(A/C)
CakSNP6538	*Kabuli*	*Ca_Kabuli_Chr04*	23318875	(T/C)
CakSNP6539	*Kabuli*	*Ca_Kabuli_Chr04*	23319309	(G/C)
CakSNP6540	*Kabuli*	*Ca_Kabuli_Chr04*	23333610	(C/T)
CakSNP6541	*Kabuli*	*Ca_Kabuli_Chr04*	23333554	(T/C)
CakSNP6542	*Kabuli*	*Ca_Kabuli_Chr04*	23337341	(T/C)
CakSNP6543	*Kabuli*	*Ca_Kabuli_Chr04*	23337312	(T/G)
CakSNP6544	*Kabuli*	*Ca_Kabuli_Chr04*	23481919	(G/A)
CakSNP6545	*Kabuli*	*Ca_Kabuli_Chr04*	23481927	(G/T)
CakSNP6546	*Kabuli*	*Ca_Kabuli_Chr04*	23481962	(G/A)
CakSNP6547	*Kabuli*	*Ca_Kabuli_Chr04*	23482033	(A/G)
CakSNP6548	*Kabuli*	*Ca_Kabuli_Chr04*	23481998	(T/C)
CakSNP6549	*Kabuli*	*Ca_Kabuli_Chr04*	23486812	(A/G)
CakSNP6550	*Kabuli*	*Ca_Kabuli_Chr04*	23486859	(A/G)
CakSNP6551	*Kabuli*	*Ca_Kabuli_Chr04*	23486824	(T/C)
CakSNP6552	*Kabuli*	*Ca_Kabuli_Chr04*	23671441	(T/G)
CakSNP6553	*Kabuli*	*Ca_Kabuli_Chr04*	23671443	(A/T)
CakSNP6554	*Kabuli*	*Ca_Kabuli_Chr04*	23731572	(C/T)
CakSNP6555	*Kabuli*	*Ca_Kabuli_Chr04*	23732099	(A/T)
CakSNP6556	*Kabuli*	*Ca_Kabuli_Chr04*	24050459	(G/A)
CakSNP6557	*Kabuli*	*Ca_Kabuli_Chr04*	24050464	(G/A)
CakSNP6558	*Kabuli*	*Ca_Kabuli_Chr04*	24120866	(A/G)
CakSNP6559	*Kabuli*	*Ca_Kabuli_Chr04*	24544345	(G/A)

SNP IDs	Cultivars	Chromosomes/scaffolds	Physical positions (bp)	SNPs
CakSNP6560	*Kabuli*	*Ca_Kabuli_Chr04*	24614582	(G/T)
CakSNP6561	*Kabuli*	*Ca_Kabuli_Chr04*	24729302	(T/C)
CakSNP6562	*Kabuli*	*Ca_Kabuli_Chr04*	24732991	(G/T)
CakSNP6563	*Kabuli*	*Ca_Kabuli_Chr04*	24732990	(T/C)
CakSNP6564	*Kabuli*	*Ca_Kabuli_Chr04*	24771737	(T/A)
CakSNP6565	*Kabuli*	*Ca_Kabuli_Chr04*	24782714	(C/A)
CakSNP6566	*Kabuli*	*Ca_Kabuli_Chr04*	24782715	(A/G)
CakSNP6567	*Kabuli*	*Ca_Kabuli_Chr04*	24856115	(A/C)
CakSNP6568	*Kabuli*	*Ca_Kabuli_Chr04*	24857501	(A/G)
CakSNP6569	*Kabuli*	*Ca_Kabuli_Chr04*	25401714	(T/C)
CakSNP6570	*Kabuli*	*Ca_Kabuli_Chr04*	25446677	(G/C)
CakSNP6571	*Kabuli*	*Ca_Kabuli_Chr04*	25446684	(T/C)
CakSNP6572	*Kabuli*	*Ca_Kabuli_Chr04*	25449897	(T/G)
CakSNP6573	*Kabuli*	*Ca_Kabuli_Chr04*	25455156	(G/A)
CakSNP6574	*Kabuli*	*Ca_Kabuli_Chr04*	25455204	(A/G)
CakSNP6575	*Kabuli*	*Ca_Kabuli_Chr04*	25455225	(G/A)
CakSNP6576	*Kabuli*	*Ca_Kabuli_Chr04*	25592561	(T/C)
CakSNP6577	*Kabuli*	*Ca_Kabuli_Chr04*	25592548	(A/G)
CakSNP6578	*Kabuli*	*Ca_Kabuli_Chr04*	25592528	(A/C)
CakSNP6579	*Kabuli*	*Ca_Kabuli_Chr04*	25592525	(C/A)
CakSNP6580	*Kabuli*	*Ca_Kabuli_Chr04*	25592492	(T/C)
CakSNP6581	*Kabuli*	*Ca_Kabuli_Chr04*	25592487	(A/G)
CakSNP6582	*Kabuli*	*Ca_Kabuli_Chr04*	25592484	(A/C)
CakSNP6583	*Kabuli*	*Ca_Kabuli_Chr04*	25592481	(T/C)
CakSNP6584	*Kabuli*	*Ca_Kabuli_Chr04*	25729905	(C/A)
CakSNP6585	*Kabuli*	*Ca_Kabuli_Chr04*	25729914	(C/A)
CakSNP6586	*Kabuli*	*Ca_Kabuli_Chr04*	25729974	(G/C)
CakSNP6587	*Kabuli*	*Ca_Kabuli_Chr04*	25729977	(C/T)
CakSNP6588	*Kabuli*	*Ca_Kabuli_Chr04*	25747256	(T/C)
CakSNP6589	*Kabuli*	*Ca_Kabuli_Chr04*	25747262	(G/A)
CakSNP6590	*Kabuli*	*Ca_Kabuli_Chr04*	25747274	(T/G)
CakSNP6591	*Kabuli*	*Ca_Kabuli_Chr04*	25747275	(G/A)
CakSNP6592	*Kabuli*	*Ca_Kabuli_Chr04*	25747281	(T/C)
CakSNP6593	*Kabuli*	*Ca_Kabuli_Chr04*	25747295	(G/A)
CakSNP6594	*Kabuli*	*Ca_Kabuli_Chr04*	25747296	(T/C)
CakSNP6595	*Kabuli*	*Ca_Kabuli_Chr04*	25747364	(T/A)
CakSNP6596	*Kabuli*	*Ca_Kabuli_Chr04*	25747297	(G/A)
CakSNP6597	*Kabuli*	*Ca_Kabuli_Chr04*	25809665	(T/C)
CakSNP6598	*Kabuli*	*Ca_Kabuli_Chr04*	25921165	(T/C)
CakSNP6599	*Kabuli*	*Ca_Kabuli_Chr04*	26000532	(C/T)
CakSNP6600	*Kabuli*	*Ca_Kabuli_Chr04*	26000483	(T/A)

SNP IDs	Cultivars	Chromosomes/scaffolds	Physical positions (bp)	SNPs
CakSNP6601	*Kabuli*	*Ca_Kabuli_Chr04*	26002991	(C/A)
CakSNP6602	*Kabuli*	*Ca_Kabuli_Chr04*	26008744	(G/A)
CakSNP6603	*Kabuli*	*Ca_Kabuli_Chr04*	26008745	(G/A)
CakSNP6604	*Kabuli*	*Ca_Kabuli_Chr04*	26008748	(C/T)
CakSNP6605	*Kabuli*	*Ca_Kabuli_Chr04*	26008768	(A/G)
CakSNP6606	*Kabuli*	*Ca_Kabuli_Chr04*	26008816	(C/A)
CakSNP6607	*Kabuli*	*Ca_Kabuli_Chr04*	26009438	(G/T)
CakSNP6608	*Kabuli*	*Ca_Kabuli_Chr04*	26009439	(G/T)
CakSNP6609	*Kabuli*	*Ca_Kabuli_Chr04*	26222180	(A/G)
CakSNP6610	*Kabuli*	*Ca_Kabuli_Chr04*	26282236	(A/C)
CakSNP6611	*Kabuli*	*Ca_Kabuli_Chr04*	26324460	(T/C)
CakSNP6612	*Kabuli*	*Ca_Kabuli_Chr04*	26529606	(C/T)
CakSNP6613	*Kabuli*	*Ca_Kabuli_Chr04*	26532763	(T/G)
CakSNP6614	*Kabuli*	*Ca_Kabuli_Chr04*	26536919	(C/G)
CakSNP6615	*Kabuli*	*Ca_Kabuli_Chr04*	26537053	(C/T)
CakSNP6616	*Kabuli*	*Ca_Kabuli_Chr04*	26537258	(A/C)
CakSNP6617	*Kabuli*	*Ca_Kabuli_Chr04*	26869756	(A/G)
CakSNP6618	*Kabuli*	*Ca_Kabuli_Chr04*	26915602	(G/A)
CakSNP6619	*Kabuli*	*Ca_Kabuli_Chr04*	26915615	(A/G)
CakSNP6620	*Kabuli*	*Ca_Kabuli_Chr04*	26915621	(G/A)
CakSNP6621	*Kabuli*	*Ca_Kabuli_Chr04*	26915626	(C/T)
CakSNP6622	*Kabuli*	*Ca_Kabuli_Chr04*	26989085	(G/A)
CakSNP6623	*Kabuli*	*Ca_Kabuli_Chr04*	26989090	(G/T)
CakSNP6624	*Kabuli*	*Ca_Kabuli_Chr04*	26989204	(A/C)
CakSNP6625	*Kabuli*	*Ca_Kabuli_Chr04*	27102807	(G/A)
CakSNP6626	*Kabuli*	*Ca_Kabuli_Chr04*	27102817	(A/C)
CakSNP6627	*Kabuli*	*Ca_Kabuli_Chr04*	27102833	(G/T)
CakSNP6628	*Kabuli*	*Ca_Kabuli_Chr04*	27262241	(C/T)
CakSNP6629	*Kabuli*	*Ca_Kabuli_Chr04*	27262893	(G/A)
CakSNP6630	*Kabuli*	*Ca_Kabuli_Chr04*	27262870	(C/T)
CakSNP6631	*Kabuli*	*Ca_Kabuli_Chr04*	27262830	(T/A)
CakSNP6632	*Kabuli*	*Ca_Kabuli_Chr04*	27268301	(G/A)
CakSNP6633	*Kabuli*	*Ca_Kabuli_Chr04*	27268278	(C/T)
CakSNP6634	*Kabuli*	*Ca_Kabuli_Chr04*	27493010	(G/T)
CakSNP6635	*Kabuli*	*Ca_Kabuli_Chr04*	27613450	(C/T)
CakSNP6636	*Kabuli*	*Ca_Kabuli_Chr04*	27617803	(A/G)
CakSNP6637	*Kabuli*	*Ca_Kabuli_Chr04*	27626559	(G/A)
CakSNP6638	*Kabuli*	*Ca_Kabuli_Chr04*	27626999	(C/T)
CakSNP6639	*Kabuli*	*Ca_Kabuli_Chr04*	27661241	(T/G)
CakSNP6640	*Kabuli*	*Ca_Kabuli_Chr04*	27669829	(C/G)
CakSNP6641	*Kabuli*	*Ca_Kabuli_Chr04*	27777968	(A/C)

SNP IDs	Cultivars	Chromosomes/scaffolds	Physical positions (bp)	SNPs
CakSNP6642	*Kabuli*	*Ca_Kabuli_Chr04*	27786162	(G/C)
CakSNP6643	*Kabuli*	*Ca_Kabuli_Chr04*	27970989	(T/G)
CakSNP6644	*Kabuli*	*Ca_Kabuli_Chr04*	28061002	(A/C)
CakSNP6645	*Kabuli*	*Ca_Kabuli_Chr04*	28092609	(G/A)
CakSNP6646	*Kabuli*	*Ca_Kabuli_Chr04*	28092727	(T/A)
CakSNP6647	*Kabuli*	*Ca_Kabuli_Chr04*	28092725	(T/G)
CakSNP6648	*Kabuli*	*Ca_Kabuli_Chr04*	28092724	(T/A)
CakSNP6649	*Kabuli*	*Ca_Kabuli_Chr04*	28268788	(G/A)
CakSNP6650	*Kabuli*	*Ca_Kabuli_Chr04*	28289900	(C/A)
CakSNP6651	*Kabuli*	*Ca_Kabuli_Chr04*	28299238	(G/A)
CakSNP6652	*Kabuli*	*Ca_Kabuli_Chr04*	28301853	(T/C)
CakSNP6653	*Kabuli*	*Ca_Kabuli_Chr04*	28423079	(T/C)
CakSNP6654	*Kabuli*	*Ca_Kabuli_Chr04*	28423140	(A/G)
CakSNP6655	*Kabuli*	*Ca_Kabuli_Chr04*	28431706	(G/A)
CakSNP6656	*Kabuli*	*Ca_Kabuli_Chr04*	28790085	(A/G)
CakSNP6657	*Kabuli*	*Ca_Kabuli_Chr04*	29582296	(C/T)
CakSNP6658	*Kabuli*	*Ca_Kabuli_Chr04*	29582289	(G/A)
CakSNP6659	*Kabuli*	*Ca_Kabuli_Chr04*	29582285	(C/A)
CakSNP6660	*Kabuli*	*Ca_Kabuli_Chr04*	29582282	(C/A)
CakSNP6661	*Kabuli*	*Ca_Kabuli_Chr04*	29582273	(G/A)
CakSNP6662	*Kabuli*	*Ca_Kabuli_Chr04*	29582268	(C/G)
CakSNP6663	*Kabuli*	*Ca_Kabuli_Chr04*	29582257	(G/A)
CakSNP6664	*Kabuli*	*Ca_Kabuli_Chr04*	29582255	(C/T)
CakSNP6665	*Kabuli*	*Ca_Kabuli_Chr04*	29582252	(G/A)
CakSNP6666	*Kabuli*	*Ca_Kabuli_Chr04*	29582249	(C/T)
CakSNP6667	*Kabuli*	*Ca_Kabuli_Chr04*	29582235	(T/A)
CakSNP6668	*Kabuli*	*Ca_Kabuli_Chr04*	29582232	(C/T)
CakSNP6669	*Kabuli*	*Ca_Kabuli_Chr04*	29582227	(G/A)
CakSNP6670	*Kabuli*	*Ca_Kabuli_Chr04*	29582219	(A/C)
CakSNP6671	*Kabuli*	*Ca_Kabuli_Chr04*	29582271	(C/T)
CakSNP6672	*Kabuli*	*Ca_Kabuli_Chr04*	29582286	(T/A)
CakSNP6673	*Kabuli*	*Ca_Kabuli_Chr04*	29582294	(G/A)
CakSNP6674	*Kabuli*	*Ca_Kabuli_Chr04*	29582301	(G/T)
CakSNP6675	*Kabuli*	*Ca_Kabuli_Chr04*	29671563	(G/A)
CakSNP6676	*Kabuli*	*Ca_Kabuli_Chr04*	29671606	(A/G)
CakSNP6677	*Kabuli*	*Ca_Kabuli_Chr04*	29683718	(A/T)
CakSNP6678	*Kabuli*	*Ca_Kabuli_Chr04*	29923380	(G/A)
CakSNP6679	*Kabuli*	*Ca_Kabuli_Chr04*	29958122	(T/G)
CakSNP6680	*Kabuli*	*Ca_Kabuli_Chr04*	29958113	(G/A)
CakSNP6681	*Kabuli*	*Ca_Kabuli_Chr04*	29972268	(T/G)
CakSNP6682	*Kabuli*	*Ca_Kabuli_Chr04*	30066903	(G/A)

SNP IDs	Cultivars	Chromosomes/scaffolds	Physical positions (bp)	SNPs
CakSNP6683	*Kabuli*	*Ca_Kabuli_Chr04*	30153390	(G/A)
CakSNP6684	*Kabuli*	*Ca_Kabuli_Chr04*	30153448	(T/C)
CakSNP6685	*Kabuli*	*Ca_Kabuli_Chr04*	30153505	(C/G)
CakSNP6686	*Kabuli*	*Ca_Kabuli_Chr04*	30258044	(A/T)
CakSNP6687	*Kabuli*	*Ca_Kabuli_Chr04*	30258045	(A/T)
CakSNP6688	*Kabuli*	*Ca_Kabuli_Chr04*	30258056	(A/G)
CakSNP6689	*Kabuli*	*Ca_Kabuli_Chr04*	30258484	(G/C)
CakSNP6690	*Kabuli*	*Ca_Kabuli_Chr04*	30258613	(C/A)
CakSNP6691	*Kabuli*	*Ca_Kabuli_Chr04*	30258607	(C/A)
CakSNP6692	*Kabuli*	*Ca_Kabuli_Chr04*	30258604	(C/A)
CakSNP6693	*Kabuli*	*Ca_Kabuli_Chr04*	30258596	(G/C)
CakSNP6694	*Kabuli*	*Ca_Kabuli_Chr04*	30258567	(C/A)
CakSNP6695	*Kabuli*	*Ca_Kabuli_Chr04*	30260485	(C/A)
CakSNP6696	*Kabuli*	*Ca_Kabuli_Chr04*	30281991	(A/G)
CakSNP6697	*Kabuli*	*Ca_Kabuli_Chr04*	30301002	(T/G)
CakSNP6698	*Kabuli*	*Ca_Kabuli_Chr04*	30308719	(A/C)
CakSNP6699	*Kabuli*	*Ca_Kabuli_Chr04*	30308812	(G/A)
CakSNP6700	*Kabuli*	*Ca_Kabuli_Chr04*	30308788	(T/C)
CakSNP6701	*Kabuli*	*Ca_Kabuli_Chr04*	30463063	(T/C)
CakSNP6702	*Kabuli*	*Ca_Kabuli_Chr04*	30463117	(C/A)
CakSNP6703	*Kabuli*	*Ca_Kabuli_Chr04*	30599096	(C/A)
CakSNP6704	*Kabuli*	*Ca_Kabuli_Chr04*	30599188	(C/T)
CakSNP6705	*Kabuli*	*Ca_Kabuli_Chr04*	30667916	(G/A)
CakSNP6706	*Kabuli*	*Ca_Kabuli_Chr04*	30668893	(A/C)
CakSNP6707	*Kabuli*	*Ca_Kabuli_Chr04*	30851027	(A/T)
CakSNP6708	*Kabuli*	*Ca_Kabuli_Chr04*	30851411	(T/G)
CakSNP6709	*Kabuli*	*Ca_Kabuli_Chr04*	30851840	(A/T)
CakSNP6710	*Kabuli*	*Ca_Kabuli_Chr04*	30931586	(G/T)
CakSNP6711	*Kabuli*	*Ca_Kabuli_Chr04*	31218647	(T/C)
CakSNP6712	*Kabuli*	*Ca_Kabuli_Chr04*	31221264	(T/G)
CakSNP6713	*Kabuli*	*Ca_Kabuli_Chr04*	31221253	(G/A)
CakSNP6714	*Kabuli*	*Ca_Kabuli_Chr04*	31221226	(T/A)
CakSNP6715	*Kabuli*	*Ca_Kabuli_Chr04*	31224001	(G/A)
CakSNP6716	*Kabuli*	*Ca_Kabuli_Chr04*	31262596	(T/A)
CakSNP6717	*Kabuli*	*Ca_Kabuli_Chr04*	31263205	(T/A)
CakSNP6718	*Kabuli*	*Ca_Kabuli_Chr04*	31594126	(A/C)
CakSNP6719	*Kabuli*	*Ca_Kabuli_Chr04*	31618949	(T/A)
CakSNP6720	*Kabuli*	*Ca_Kabuli_Chr04*	31875713	(A/G)
CakSNP6721	*Kabuli*	*Ca_Kabuli_Chr04*	31987890	(A/C)
CakSNP6722	*Kabuli*	*Ca_Kabuli_Chr04*	32042488	(T/G)
CakSNP6723	*Kabuli*	*Ca_Kabuli_Chr04*	32042527	(C/T)

SNP IDs	Cultivars	Chromosomes/scaffolds	Physical positions (bp)	SNPs
CakSNP6724	*Kabuli*	*Ca_Kabuli_Chr04*	32068961	(A/G)
CakSNP6725	*Kabuli*	*Ca_Kabuli_Chr04*	32073552	(T/C)
CakSNP6726	*Kabuli*	*Ca_Kabuli_Chr04*	32073550	(C/G)
CakSNP6727	*Kabuli*	*Ca_Kabuli_Chr04*	32073549	(G/C)
CakSNP6728	*Kabuli*	*Ca_Kabuli_Chr04*	32166559	(C/G)
CakSNP6729	*Kabuli*	*Ca_Kabuli_Chr04*	32166557	(T/A)
CakSNP6730	*Kabuli*	*Ca_Kabuli_Chr04*	32183307	(C/G)
CakSNP6731	*Kabuli*	*Ca_Kabuli_Chr04*	32184628	(A/G)
CakSNP6732	*Kabuli*	*Ca_Kabuli_Chr04*	32212899	(G/A)
CakSNP6733	*Kabuli*	*Ca_Kabuli_Chr04*	32297780	(C/A)
CakSNP6734	*Kabuli*	*Ca_Kabuli_Chr04*	32297758	(G/A)
CakSNP6735	*Kabuli*	*Ca_Kabuli_Chr04*	32297734	(A/G)
CakSNP6736	*Kabuli*	*Ca_Kabuli_Chr04*	32303394	(C/T)
CakSNP6737	*Kabuli*	*Ca_Kabuli_Chr04*	32429228	(A/T)
CakSNP6738	*Kabuli*	*Ca_Kabuli_Chr04*	32429222	(A/C)
CakSNP6739	*Kabuli*	*Ca_Kabuli_Chr04*	32521346	(T/C)
CakSNP6740	*Kabuli*	*Ca_Kabuli_Chr04*	32602658	(C/T)
CakSNP6741	*Kabuli*	*Ca_Kabuli_Chr04*	32604261	(G/A)
CakSNP6742	*Kabuli*	*Ca_Kabuli_Chr04*	32604538	(T/C)
CakSNP6743	*Kabuli*	*Ca_Kabuli_Chr04*	32604510	(A/C)
CakSNP6744	*Kabuli*	*Ca_Kabuli_Chr04*	32636315	(T/C)
CakSNP6745	*Kabuli*	*Ca_Kabuli_Chr04*	32636276	(T/C)
CakSNP6746	*Kabuli*	*Ca_Kabuli_Chr04*	32636428	(C/A)
CakSNP6747	*Kabuli*	*Ca_Kabuli_Chr04*	32636459	(A/G)
CakSNP6748	*Kabuli*	*Ca_Kabuli_Chr04*	32636690	(C/T)
CakSNP6749	*Kabuli*	*Ca_Kabuli_Chr04*	32636953	(A/G)
CakSNP6750	*Kabuli*	*Ca_Kabuli_Chr04*	32639121	(C/T)
CakSNP6751	*Kabuli*	*Ca_Kabuli_Chr04*	32639188	(C/G)
CakSNP6752	*Kabuli*	*Ca_Kabuli_Chr04*	32639118	(A/C)
CakSNP6753	*Kabuli*	*Ca_Kabuli_Chr04*	32639262	(A/G)
CakSNP6754	*Kabuli*	*Ca_Kabuli_Chr04*	32639315	(A/G)
CakSNP6755	*Kabuli*	*Ca_Kabuli_Chr04*	32639654	(T/C)
CakSNP6756	*Kabuli*	*Ca_Kabuli_Chr04*	32639681	(C/T)
CakSNP6757	*Kabuli*	*Ca_Kabuli_Chr04*	32639770	(C/G)
CakSNP6758	*Kabuli*	*Ca_Kabuli_Chr04*	32640911	(A/G)
CakSNP6759	*Kabuli*	*Ca_Kabuli_Chr04*	32853834	(T/C)
CakSNP6760	*Kabuli*	*Ca_Kabuli_Chr04*	32853838	(G/T)
CakSNP6761	*Kabuli*	*Ca_Kabuli_Chr04*	33068276	(G/A)
CakSNP6762	*Kabuli*	*Ca_Kabuli_Chr04*	33221361	(T/C)
CakSNP6763	*Kabuli*	*Ca_Kabuli_Chr04*	33221371	(T/C)
CakSNP6764	*Kabuli*	*Ca_Kabuli_Chr04*	33221397	(T/G)

SNP IDs	Cultivars	Chromosomes/scaffolds	Physical positions (bp)	SNPs
CakSNP6765	*Kabuli*	*Ca_Kabuli_Chr04*	33509759	(A/C)
CakSNP6766	*Kabuli*	*Ca_Kabuli_Chr04*	33509762	(G/A)
CakSNP6767	*Kabuli*	*Ca_Kabuli_Chr04*	33509804	(A/G)
CakSNP6768	*Kabuli*	*Ca_Kabuli_Chr04*	33641757	(C/T)
CakSNP6769	*Kabuli*	*Ca_Kabuli_Chr04*	33820657	(A/C)
CakSNP6770	*Kabuli*	*Ca_Kabuli_Chr04*	33874346	(T/C)
CakSNP6771	*Kabuli*	*Ca_Kabuli_Chr04*	33874327	(T/C)
CakSNP6772	*Kabuli*	*Ca_Kabuli_Chr04*	33874313	(G/A)
CakSNP6773	*Kabuli*	*Ca_Kabuli_Chr04*	33929598	(A/C)
CakSNP6774	*Kabuli*	*Ca_Kabuli_Chr04*	33929777	(C/T)
CakSNP6775	*Kabuli*	*Ca_Kabuli_Chr04*	34032176	(C/T)
CakSNP6776	*Kabuli*	*Ca_Kabuli_Chr04*	34155135	(A/C)
CakSNP6777	*Kabuli*	*Ca_Kabuli_Chr04*	34155131	(T/A)
CakSNP6778	*Kabuli*	*Ca_Kabuli_Chr04*	34155128	(C/T)
CakSNP6779	*Kabuli*	*Ca_Kabuli_Chr04*	34155127	(G/A)
CakSNP6780	*Kabuli*	*Ca_Kabuli_Chr04*	34155124	(G/A)
CakSNP6781	*Kabuli*	*Ca_Kabuli_Chr04*	34155084	(C/G)
CakSNP6782	*Kabuli*	*Ca_Kabuli_Chr04*	34155083	(C/T)
CakSNP6783	*Kabuli*	*Ca_Kabuli_Chr04*	34222792	(G/C)
CakSNP6784	*Kabuli*	*Ca_Kabuli_Chr04*	34354519	(G/T)
CakSNP6785	*Kabuli*	*Ca_Kabuli_Chr04*	34487064	(T/G)
CakSNP6786	*Kabuli*	*Ca_Kabuli_Chr04*	34744344	(T/G)
CakSNP6787	*Kabuli*	*Ca_Kabuli_Chr04*	34744398	(T/A)
CakSNP6788	*Kabuli*	*Ca_Kabuli_Chr04*	34784285	(G/A)
CakSNP6789	*Kabuli*	*Ca_Kabuli_Chr04*	34798597	(C/A)
CakSNP6790	*Kabuli*	*Ca_Kabuli_Chr04*	34916032	(C/T)
CakSNP6791	*Kabuli*	*Ca_Kabuli_Chr04*	34924575	(C/T)
CakSNP6792	*Kabuli*	*Ca_Kabuli_Chr04*	34955953	(A/G)
CakSNP6793	*Kabuli*	*Ca_Kabuli_Chr04*	34955971	(A/T)
CakSNP6794	*Kabuli*	*Ca_Kabuli_Chr04*	34956006	(C/T)
CakSNP6795	*Kabuli*	*Ca_Kabuli_Chr04*	34956019	(G/A)
CakSNP6796	*Kabuli*	*Ca_Kabuli_Chr04*	34956035	(C/T)
CakSNP6797	*Kabuli*	*Ca_Kabuli_Chr04*	34956111	(G/T)
CakSNP6798	*Kabuli*	*Ca_Kabuli_Chr04*	34956031	(G/A)
CakSNP6799	*Kabuli*	*Ca_Kabuli_Chr04*	35141281	(A/T)
CakSNP6800	*Kabuli*	*Ca_Kabuli_Chr04*	35141309	(A/C)
CakSNP6801	*Kabuli*	*Ca_Kabuli_Chr04*	35141340	(G/A)
CakSNP6802	*Kabuli*	*Ca_Kabuli_Chr04*	35141353	(C/G)
CakSNP6803	*Kabuli*	*Ca_Kabuli_Chr04*	35141359	(C/G)
CakSNP6804	*Kabuli*	*Ca_Kabuli_Chr04*	35141375	(A/G)
CakSNP6805	*Kabuli*	*Ca_Kabuli_Chr04*	35141403	(C/T)

SNP IDs	Cultivars	Chromosomes/scaffolds	Physical positions (bp)	SNPs
CakSNP6806	*Kabuli*	*Ca_Kabuli_Chr04*	35141425	(A/T)
CakSNP6807	*Kabuli*	*Ca_Kabuli_Chr04*	35141421	(T/A)
CakSNP6808	*Kabuli*	*Ca_Kabuli_Chr04*	35141408	(A/G)
CakSNP6809	*Kabuli*	*Ca_Kabuli_Chr04*	35168915	(C/T)
CakSNP6810	*Kabuli*	*Ca_Kabuli_Chr04*	35168939	(C/T)
CakSNP6811	*Kabuli*	*Ca_Kabuli_Chr04*	35229966	(C/T)
CakSNP6812	*Kabuli*	*Ca_Kabuli_Chr04*	35230144	(A/G)
CakSNP6813	*Kabuli*	*Ca_Kabuli_Chr04*	35231891	(C/T)
CakSNP6814	*Kabuli*	*Ca_Kabuli_Chr04*	35304102	(C/T)
CakSNP6815	*Kabuli*	*Ca_Kabuli_Chr04*	35304108	(C/A)
CakSNP6816	*Kabuli*	*Ca_Kabuli_Chr04*	35309709	(A/G)
CakSNP6817	*Kabuli*	*Ca_Kabuli_Chr04*	35311815	(A/C)
CakSNP6818	*Kabuli*	*Ca_Kabuli_Chr04*	35337282	(A/G)
CakSNP6819	*Kabuli*	*Ca_Kabuli_Chr04*	35344356	(A/G)
CakSNP6820	*Kabuli*	*Ca_Kabuli_Chr04*	35344353	(T/C)
CakSNP6821	*Kabuli*	*Ca_Kabuli_Chr04*	35344323	(C/T)
CakSNP6822	*Kabuli*	*Ca_Kabuli_Chr04*	35346415	(G/A)
CakSNP6823	*Kabuli*	*Ca_Kabuli_Chr04*	35346417	(C/T)
CakSNP6824	*Kabuli*	*Ca_Kabuli_Chr04*	35346495	(C/T)
CakSNP6825	*Kabuli*	*Ca_Kabuli_Chr04*	35346465	(G/T)
CakSNP6826	*Kabuli*	*Ca_Kabuli_Chr04*	35346558	(G/T)
CakSNP6827	*Kabuli*	*Ca_Kabuli_Chr04*	35346563	(G/T)
CakSNP6828	*Kabuli*	*Ca_Kabuli_Chr04*	35346573	(A/G)
CakSNP6829	*Kabuli*	*Ca_Kabuli_Chr04*	35346576	(C/G)
CakSNP6830	*Kabuli*	*Ca_Kabuli_Chr04*	35346624	(G/A)
CakSNP6831	*Kabuli*	*Ca_Kabuli_Chr04*	35393505	(G/T)
CakSNP6832	*Kabuli*	*Ca_Kabuli_Chr04*	35393643	(G/A)
CakSNP6833	*Kabuli*	*Ca_Kabuli_Chr04*	35455095	(A/C)
CakSNP6834	*Kabuli*	*Ca_Kabuli_Chr04*	35455062	(A/T)
CakSNP6835	*Kabuli*	*Ca_Kabuli_Chr04*	35455708	(G/A)
CakSNP6836	*Kabuli*	*Ca_Kabuli_Chr04*	35469308	(T/G)
CakSNP6837	*Kabuli*	*Ca_Kabuli_Chr04*	35469304	(G/A)
CakSNP6838	*Kabuli*	*Ca_Kabuli_Chr04*	35487504	(G/C)
CakSNP6839	*Kabuli*	*Ca_Kabuli_Chr04*	35487496	(C/A)
CakSNP6840	*Kabuli*	*Ca_Kabuli_Chr04*	35487471	(A/C)
CakSNP6841	*Kabuli*	*Ca_Kabuli_Chr04*	35528758	(C/A)
CakSNP6842	*Kabuli*	*Ca_Kabuli_Chr04*	35534777	(G/C)
CakSNP6843	*Kabuli*	*Ca_Kabuli_Chr04*	35534877	(T/C)
CakSNP6844	*Kabuli*	*Ca_Kabuli_Chr04*	35534891	(C/T)
CakSNP6845	*Kabuli*	*Ca_Kabuli_Chr04*	35574379	(C/A)
CakSNP6846	*Kabuli*	*Ca_Kabuli_Chr04*	35634712	(A/G)

SNP IDs	Cultivars	Chromosomes/scaffolds	Physical positions (bp)	SNPs
CakSNP6847	*Kabuli*	*Ca_Kabuli_Chr04*	35634751	(A/G)
CakSNP6848	*Kabuli*	*Ca_Kabuli_Chr04*	35634873	(G/T)
CakSNP6849	*Kabuli*	*Ca_Kabuli_Chr04*	35961593	(A/C)
CakSNP6850	*Kabuli*	*Ca_Kabuli_Chr04*	35977422	(G/A)
CakSNP6851	*Kabuli*	*Ca_Kabuli_Chr04*	36005847	(T/G)
CakSNP6852	*Kabuli*	*Ca_Kabuli_Chr04*	36010756	(T/G)
CakSNP6853	*Kabuli*	*Ca_Kabuli_Chr04*	36153818	(A/G)
CakSNP6854	*Kabuli*	*Ca_Kabuli_Chr04*	36154771	(T/C)
CakSNP6855	*Kabuli*	*Ca_Kabuli_Chr04*	36154820	(G/A)
CakSNP6856	*Kabuli*	*Ca_Kabuli_Chr04*	36154822	(T/G)
CakSNP6857	*Kabuli*	*Ca_Kabuli_Chr04*	36184969	(A/G)
CakSNP6858	*Kabuli*	*Ca_Kabuli_Chr04*	36277383	(C/T)
CakSNP6859	*Kabuli*	*Ca_Kabuli_Chr04*	36368713	(T/C)
CakSNP6860	*Kabuli*	*Ca_Kabuli_Chr04*	36445345	(G/A)
CakSNP6861	*Kabuli*	*Ca_Kabuli_Chr04*	36445367	(A/G)
CakSNP6862	*Kabuli*	*Ca_Kabuli_Chr04*	36449101	(C/T)
CakSNP6863	*Kabuli*	*Ca_Kabuli_Chr04*	36461836	(A/G)
CakSNP6864	*Kabuli*	*Ca_Kabuli_Chr04*	36467928	(C/T)
CakSNP6865	*Kabuli*	*Ca_Kabuli_Chr04*	36467943	(A/G)
CakSNP6866	*Kabuli*	*Ca_Kabuli_Chr04*	36467964	(G/A)
CakSNP6867	*Kabuli*	*Ca_Kabuli_Chr04*	36467967	(T/C)
CakSNP6868	*Kabuli*	*Ca_Kabuli_Chr04*	36467980	(C/A)
CakSNP6869	*Kabuli*	*Ca_Kabuli_Chr04*	36467988	(C/T)
CakSNP6870	*Kabuli*	*Ca_Kabuli_Chr04*	36467990	(T/G)
CakSNP6871	*Kabuli*	*Ca_Kabuli_Chr04*	36468003	(T/A)
CakSNP6872	*Kabuli*	*Ca_Kabuli_Chr04*	36468053	(A/G)
CakSNP6873	*Kabuli*	*Ca_Kabuli_Chr04*	36488966	(A/G)
CakSNP6874	*Kabuli*	*Ca_Kabuli_Chr04*	36537118	(C/T)
CakSNP6875	*Kabuli*	*Ca_Kabuli_Chr04*	36537124	(C/T)
CakSNP6876	*Kabuli*	*Ca_Kabuli_Chr04*	36537170	(C/T)
CakSNP6877	*Kabuli*	*Ca_Kabuli_Chr04*	36541088	(G/T)
CakSNP6878	*Kabuli*	*Ca_Kabuli_Chr04*	36541087	(T/C)
CakSNP6879	*Kabuli*	*Ca_Kabuli_Chr04*	36541083	(A/C)
CakSNP6880	*Kabuli*	*Ca_Kabuli_Chr04*	36639333	(A/T)
CakSNP6881	*Kabuli*	*Ca_Kabuli_Chr04*	36639382	(T/C)
CakSNP6882	*Kabuli*	*Ca_Kabuli_Chr04*	36639474	(G/C)
CakSNP6883	*Kabuli*	*Ca_Kabuli_Chr04*	36673677	(C/A)
CakSNP6884	*Kabuli*	*Ca_Kabuli_Chr04*	36682215	(G/A)
CakSNP6885	*Kabuli*	*Ca_Kabuli_Chr04*	36682213	(C/T)
CakSNP6886	*Kabuli*	*Ca_Kabuli_Chr04*	36682211	(G/A)
CakSNP6887	*Kabuli*	*Ca_Kabuli_Chr04*	36682253	(T/G)

SNP IDs	Cultivars	Chromosomes/scaffolds	Physical positions (bp)	SNPs
CakSNP6888	*Kabuli*	*Ca_Kabuli_Chr04*	36682231	(G/T)
CakSNP6889	*Kabuli*	*Ca_Kabuli_Chr04*	36709731	(G/A)
CakSNP6890	*Kabuli*	*Ca_Kabuli_Chr04*	36754121	(T/C)
CakSNP6891	*Kabuli*	*Ca_Kabuli_Chr04*	36754149	(G/A)
CakSNP6892	*Kabuli*	*Ca_Kabuli_Chr04*	36754290	(T/A)
CakSNP6893	*Kabuli*	*Ca_Kabuli_Chr04*	36754378	(C/T)
CakSNP6894	*Kabuli*	*Ca_Kabuli_Chr04*	36754377	(T/A)
CakSNP6895	*Kabuli*	*Ca_Kabuli_Chr04*	36754348	(C/T)
CakSNP6896	*Kabuli*	*Ca_Kabuli_Chr04*	36754343	(A/C)
CakSNP6897	*Kabuli*	*Ca_Kabuli_Chr04*	36754327	(G/A)
CakSNP6898	*Kabuli*	*Ca_Kabuli_Chr04*	36754468	(G/A)
CakSNP6899	*Kabuli*	*Ca_Kabuli_Chr04*	36754453	(A/G)
CakSNP6900	*Kabuli*	*Ca_Kabuli_Chr04*	36799551	(C/T)
CakSNP6901	*Kabuli*	*Ca_Kabuli_Chr04*	36831035	(A/G)
CakSNP6902	*Kabuli*	*Ca_Kabuli_Chr04*	36831199	(G/A)
CakSNP6903	*Kabuli*	*Ca_Kabuli_Chr04*	36831267	(A/C)
CakSNP6904	*Kabuli*	*Ca_Kabuli_Chr04*	36955706	(G/T)
CakSNP6905	*Kabuli*	*Ca_Kabuli_Chr04*	36977192	(C/T)
CakSNP6906	*Kabuli*	*Ca_Kabuli_Chr04*	36977194	(G/A)
CakSNP6907	*Kabuli*	*Ca_Kabuli_Chr04*	36979866	(T/G)
CakSNP6908	*Kabuli*	*Ca_Kabuli_Chr04*	36979926	(G/T)
CakSNP6909	*Kabuli*	*Ca_Kabuli_Chr04*	36979879	(C/T)
CakSNP6910	*Kabuli*	*Ca_Kabuli_Chr04*	36982955	(A/G)
CakSNP6911	*Kabuli*	*Ca_Kabuli_Chr04*	36984727	(C/G)
CakSNP6912	*Kabuli*	*Ca_Kabuli_Chr04*	36984829	(C/T)
CakSNP6913	*Kabuli*	*Ca_Kabuli_Chr04*	36984795	(T/A)
CakSNP6914	*Kabuli*	*Ca_Kabuli_Chr04*	36998201	(A/C)
CakSNP6915	*Kabuli*	*Ca_Kabuli_Chr04*	36998209	(C/T)
CakSNP6916	*Kabuli*	*Ca_Kabuli_Chr04*	37064588	(A/G)
CakSNP6917	*Kabuli*	*Ca_Kabuli_Chr04*	37064630	(G/A)
CakSNP6918	*Kabuli*	*Ca_Kabuli_Chr04*	37185576	(T/A)
CakSNP6919	*Kabuli*	*Ca_Kabuli_Chr04*	37185577	(T/A)
CakSNP6920	*Kabuli*	*Ca_Kabuli_Chr04*	37185578	(T/A)
CakSNP6921	*Kabuli*	*Ca_Kabuli_Chr04*	37237211	(G/A)
CakSNP6922	*Kabuli*	*Ca_Kabuli_Chr04*	37237276	(T/C)
CakSNP6923	*Kabuli*	*Ca_Kabuli_Chr04*	37241235	(A/G)
CakSNP6924	*Kabuli*	*Ca_Kabuli_Chr04*	37241262	(G/A)
CakSNP6925	*Kabuli*	*Ca_Kabuli_Chr04*	37260697	(T/C)
CakSNP6926	*Kabuli*	*Ca_Kabuli_Chr04*	37260873	(A/G)
CakSNP6927	*Kabuli*	*Ca_Kabuli_Chr04*	37260841	(T/A)
CakSNP6928	*Kabuli*	*Ca_Kabuli_Chr04*	37264628	(G/A)

SNP IDs	Cultivars	Chromosomes/scaffolds	Physical positions (bp)	SNPs
CakSNP6929	*Kabuli*	*Ca_Kabuli_Chr04*	37286591	(T/A)
CakSNP6930	*Kabuli*	*Ca_Kabuli_Chr04*	37286625	(C/A)
CakSNP6931	*Kabuli*	*Ca_Kabuli_Chr04*	37286632	(G/A)
CakSNP6932	*Kabuli*	*Ca_Kabuli_Chr04*	37286663	(A/G)
CakSNP6933	*Kabuli*	*Ca_Kabuli_Chr04*	37293752	(G/A)
CakSNP6934	*Kabuli*	*Ca_Kabuli_Chr04*	37293780	(T/A)
CakSNP6935	*Kabuli*	*Ca_Kabuli_Chr04*	37293773	(A/T)
CakSNP6936	*Kabuli*	*Ca_Kabuli_Chr04*	37293769	(C/A)
CakSNP6937	*Kabuli*	*Ca_Kabuli_Chr04*	37294720	(A/G)
CakSNP6938	*Kabuli*	*Ca_Kabuli_Chr04*	37316574	(T/A)
CakSNP6939	*Kabuli*	*Ca_Kabuli_Chr04*	37316748	(T/A)
CakSNP6940	*Kabuli*	*Ca_Kabuli_Chr04*	37316738	(C/T)
CakSNP6941	*Kabuli*	*Ca_Kabuli_Chr04*	37316713	(A/T)
CakSNP6942	*Kabuli*	*Ca_Kabuli_Chr04*	37316700	(C/A)
CakSNP6943	*Kabuli*	*Ca_Kabuli_Chr04*	37349071	(T/C)
CakSNP6944	*Kabuli*	*Ca_Kabuli_Chr04*	37349212	(A/C)
CakSNP6945	*Kabuli*	*Ca_Kabuli_Chr04*	37349239	(C/G)
CakSNP6946	*Kabuli*	*Ca_Kabuli_Chr04*	37349304	(T/C)
CakSNP6947	*Kabuli*	*Ca_Kabuli_Chr04*	37349331	(C/T)
CakSNP6948	*Kabuli*	*Ca_Kabuli_Chr04*	37349321	(T/C)
CakSNP6949	*Kabuli*	*Ca_Kabuli_Chr04*	37356865	(C/A)
CakSNP6950	*Kabuli*	*Ca_Kabuli_Chr04*	37356889	(C/T)
CakSNP6951	*Kabuli*	*Ca_Kabuli_Chr04*	37356892	(C/T)
CakSNP6952	*Kabuli*	*Ca_Kabuli_Chr04*	37356919	(A/T)
CakSNP6953	*Kabuli*	*Ca_Kabuli_Chr04*	37356951	(C/T)
CakSNP6954	*Kabuli*	*Ca_Kabuli_Chr04*	37364980	(G/A)
CakSNP6955	*Kabuli*	*Ca_Kabuli_Chr04*	37365020	(C/T)
CakSNP6956	*Kabuli*	*Ca_Kabuli_Chr04*	37371262	(G/A)
CakSNP6957	*Kabuli*	*Ca_Kabuli_Chr04*	37371263	(G/A)
CakSNP6958	*Kabuli*	*Ca_Kabuli_Chr04*	37433459	(G/T)
CakSNP6959	*Kabuli*	*Ca_Kabuli_Chr04*	37450206	(A/C)
CakSNP6960	*Kabuli*	*Ca_Kabuli_Chr04*	37450326	(A/T)
CakSNP6961	*Kabuli*	*Ca_Kabuli_Chr04*	37450308	(C/T)
CakSNP6962	*Kabuli*	*Ca_Kabuli_Chr04*	37450294	(T/C)
CakSNP6963	*Kabuli*	*Ca_Kabuli_Chr04*	37453497	(C/G)
CakSNP6964	*Kabuli*	*Ca_Kabuli_Chr04*	37453449	(A/G)
CakSNP6965	*Kabuli*	*Ca_Kabuli_Chr04*	37453590	(C/A)
CakSNP6966	*Kabuli*	*Ca_Kabuli_Chr04*	37453586	(C/A)
CakSNP6967	*Kabuli*	*Ca_Kabuli_Chr04*	37454296	(A/G)
CakSNP6968	*Kabuli*	*Ca_Kabuli_Chr04*	37492422	(C/A)
CakSNP6969	*Kabuli*	*Ca_Kabuli_Chr04*	37549883	(T/C)

SNP IDs	Cultivars	Chromosomes/scaffolds	Physical positions (bp)	SNPs
CakSNP6970	*Kabuli*	*Ca_Kabuli_Chr04*	37549871	(A/T)
CakSNP6971	*Kabuli*	*Ca_Kabuli_Chr04*	37550122	(G/A)
CakSNP6972	*Kabuli*	*Ca_Kabuli_Chr04*	37552973	(C/T)
CakSNP6973	*Kabuli*	*Ca_Kabuli_Chr04*	37558880	(C/T)
CakSNP6974	*Kabuli*	*Ca_Kabuli_Chr04*	37558881	(G/T)
CakSNP6975	*Kabuli*	*Ca_Kabuli_Chr04*	37575636	(T/C)
CakSNP6976	*Kabuli*	*Ca_Kabuli_Chr04*	37575670	(C/T)
CakSNP6977	*Kabuli*	*Ca_Kabuli_Chr04*	37575709	(C/T)
CakSNP6978	*Kabuli*	*Ca_Kabuli_Chr04*	37612154	(A/G)
CakSNP6979	*Kabuli*	*Ca_Kabuli_Chr04*	37630404	(A/G)
CakSNP6980	*Kabuli*	*Ca_Kabuli_Chr04*	37630445	(A/G)
CakSNP6981	*Kabuli*	*Ca_Kabuli_Chr04*	37630527	(G/A)
CakSNP6982	*Kabuli*	*Ca_Kabuli_Chr04*	37630520	(G/C)
CakSNP6983	*Kabuli*	*Ca_Kabuli_Chr04*	37634624	(C/T)
CakSNP6984	*Kabuli*	*Ca_Kabuli_Chr04*	37635300	(T/A)
CakSNP6985	*Kabuli*	*Ca_Kabuli_Chr04*	37685824	(T/C)
CakSNP6986	*Kabuli*	*Ca_Kabuli_Chr04*	37685842	(T/G)
CakSNP6987	*Kabuli*	*Ca_Kabuli_Chr04*	37699176	(G/A)
CakSNP6988	*Kabuli*	*Ca_Kabuli_Chr04*	37699141	(T/G)
CakSNP6989	*Kabuli*	*Ca_Kabuli_Chr04*	37699140	(C/T)
CakSNP6990	*Kabuli*	*Ca_Kabuli_Chr04*	37703656	(G/T)
CakSNP6991	*Kabuli*	*Ca_Kabuli_Chr04*	37703653	(A/C)
CakSNP6992	*Kabuli*	*Ca_Kabuli_Chr04*	37703651	(T/C)
CakSNP6993	*Kabuli*	*Ca_Kabuli_Chr04*	37703649	(A/C)
CakSNP6994	*Kabuli*	*Ca_Kabuli_Chr04*	37703648	(C/T)
CakSNP6995	*Kabuli*	*Ca_Kabuli_Chr04*	37707115	(G/A)
CakSNP6996	*Kabuli*	*Ca_Kabuli_Chr04*	37708260	(A/G)
CakSNP6997	*Kabuli*	*Ca_Kabuli_Chr04*	37708348	(T/G)
CakSNP6998	*Kabuli*	*Ca_Kabuli_Chr04*	37708334	(T/A)
CakSNP6999	*Kabuli*	*Ca_Kabuli_Chr04*	37753479	(C/T)
CakSNP7000	*Kabuli*	*Ca_Kabuli_Chr04*	37806689	(T/C)
CakSNP7001	*Kabuli*	*Ca_Kabuli_Chr04*	37812917	(G/C)
CakSNP7002	*Kabuli*	*Ca_Kabuli_Chr04*	37859069	(T/G)
CakSNP7003	*Kabuli*	*Ca_Kabuli_Chr04*	37897261	(G/T)
CakSNP7004	*Kabuli*	*Ca_Kabuli_Chr04*	37897266	(A/T)
CakSNP7005	*Kabuli*	*Ca_Kabuli_Chr04*	37897351	(C/A)
CakSNP7006	*Kabuli*	*Ca_Kabuli_Chr04*	37898404	(A/G)
CakSNP7007	*Kabuli*	*Ca_Kabuli_Chr04*	37899597	(C/T)
CakSNP7008	*Kabuli*	*Ca_Kabuli_Chr04*	37901584	(C/T)
CakSNP7009	*Kabuli*	*Ca_Kabuli_Chr04*	37901575	(G/A)
CakSNP7010	*Kabuli*	*Ca_Kabuli_Chr04*	38008062	(T/A)

**SNP IDs**	**Cultivars**	**Chromosomes/scaffolds**	**Physical positions (bp)**	**SNPs**
CakSNP7011	*Kabuli*	*Ca_Kabuli_Chr04*	38008082	(T/C)
CakSNP7012	*Kabuli*	*Ca_Kabuli_Chr04*	38008243	(C/T)
CakSNP7013	*Kabuli*	*Ca_Kabuli_Chr04*	38008227	(G/T)
CakSNP7014	*Kabuli*	*Ca_Kabuli_Chr04*	38008213	(C/T)
CakSNP7015	*Kabuli*	*Ca_Kabuli_Chr04*	38008205	(A/G)
CakSNP7016	*Kabuli*	*Ca_Kabuli_Chr04*	38008204	(G/A)
CakSNP7017	*Kabuli*	*Ca_Kabuli_Chr04*	38008186	(A/C)
CakSNP7018	*Kabuli*	*Ca_Kabuli_Chr04*	38008174	(A/G)
CakSNP7019	*Kabuli*	*Ca_Kabuli_Chr04*	38027560	(G/A)
CakSNP7020	*Kabuli*	*Ca_Kabuli_Chr04*	38029012	(T/C)
CakSNP7021	*Kabuli*	*Ca_Kabuli_Chr04*	38029050	(G/A)
CakSNP7022	*Kabuli*	*Ca_Kabuli_Chr04*	38029136	(G/T)
CakSNP7023	*Kabuli*	*Ca_Kabuli_Chr04*	38038865	(C/T)
CakSNP7024	*Kabuli*	*Ca_Kabuli_Chr04*	38038959	(A/G)
CakSNP7025	*Kabuli*	*Ca_Kabuli_Chr04*	38039037	(T/C)
CakSNP7026	*Kabuli*	*Ca_Kabuli_Chr04*	38039046	(C/T)
CakSNP7027	*Kabuli*	*Ca_Kabuli_Chr04*	38039076	(A/G)
CakSNP7028	*Kabuli*	*Ca_Kabuli_Chr04*	38039088	(T/C)
CakSNP7029	*Kabuli*	*Ca_Kabuli_Chr04*	38039251	(G/C)
CakSNP7030	*Kabuli*	*Ca_Kabuli_Chr04*	38039289	(G/A)
CakSNP7031	*Kabuli*	*Ca_Kabuli_Chr04*	38039297	(C/T)
CakSNP7032	*Kabuli*	*Ca_Kabuli_Chr04*	38039313	(G/A)
CakSNP7033	*Kabuli*	*Ca_Kabuli_Chr04*	38039371	(T/C)
CakSNP7034	*Kabuli*	*Ca_Kabuli_Chr04*	38153183	(C/A)
CakSNP7035	*Kabuli*	*Ca_Kabuli_Chr04*	38153195	(T/C)
CakSNP7036	*Kabuli*	*Ca_Kabuli_Chr04*	38153216	(T/C)
CakSNP7037	*Kabuli*	*Ca_Kabuli_Chr04*	38153277	(A/G)
CakSNP7038	*Kabuli*	*Ca_Kabuli_Chr04*	38153275	(C/A)
CakSNP7039	*Kabuli*	*Ca_Kabuli_Chr04*	38153269	(A/C)
CakSNP7040	*Kabuli*	*Ca_Kabuli_Chr04*	38179583	(A/C)
CakSNP7041	*Kabuli*	*Ca_Kabuli_Chr04*	38179614	(G/C)
CakSNP7042	*Kabuli*	*Ca_Kabuli_Chr04*	38181292	(G/A)
CakSNP7043	*Kabuli*	*Ca_Kabuli_Chr04*	38195411	(T/A)
CakSNP7044	*Kabuli*	*Ca_Kabuli_Chr04*	38195438	(C/T)
CakSNP7045	*Kabuli*	*Ca_Kabuli_Chr04*	38215267	(G/A)
CakSNP7046	*Kabuli*	*Ca_Kabuli_Chr04*	38221009	(A/G)
CakSNP7047	*Kabuli*	*Ca_Kabuli_Chr04*	38288823	(G/T)
CakSNP7048	*Kabuli*	*Ca_Kabuli_Chr04*	38288877	(C/G)
CakSNP7049	*Kabuli*	*Ca_Kabuli_Chr04*	38289066	(G/A)
CakSNP7050	*Kabuli*	*Ca_Kabuli_Chr04*	38289052	(G/A)
CakSNP7051	*Kabuli*	*Ca_Kabuli_Chr04*	38288997	(T/A)

SNP IDs	Cultivars	Chromosomes/scaffolds	Physical positions (bp)	SNPs
CakSNP7052	*Kabuli*	*Ca_Kabuli_Chr04*	38343633	(A/T)
CakSNP7053	*Kabuli*	*Ca_Kabuli_Chr04*	38343750	(C/T)
CakSNP7054	*Kabuli*	*Ca_Kabuli_Chr04*	38343687	(C/T)
CakSNP7055	*Kabuli*	*Ca_Kabuli_Chr04*	38358067	(A/G)
CakSNP7056	*Kabuli*	*Ca_Kabuli_Chr04*	38358398	(A/G)
CakSNP7057	*Kabuli*	*Ca_Kabuli_Chr04*	38358677	(G/A)
CakSNP7058	*Kabuli*	*Ca_Kabuli_Chr04*	38358887	(G/A)
CakSNP7059	*Kabuli*	*Ca_Kabuli_Chr04*	38358869	(G/A)
CakSNP7060	*Kabuli*	*Ca_Kabuli_Chr04*	38362132	(T/A)
CakSNP7061	*Kabuli*	*Ca_Kabuli_Chr04*	38362139	(T/A)
CakSNP7062	*Kabuli*	*Ca_Kabuli_Chr04*	38364369	(C/T)
CakSNP7063	*Kabuli*	*Ca_Kabuli_Chr04*	38370848	(A/G)
CakSNP7064	*Kabuli*	*Ca_Kabuli_Chr04*	38370864	(G/A)
CakSNP7065	*Kabuli*	*Ca_Kabuli_Chr04*	38370941	(A/G)
CakSNP7066	*Kabuli*	*Ca_Kabuli_Chr04*	38370930	(C/T)
CakSNP7067	*Kabuli*	*Ca_Kabuli_Chr04*	38370926	(T/C)
CakSNP7068	*Kabuli*	*Ca_Kabuli_Chr04*	38370925	(G/A)
CakSNP7069	*Kabuli*	*Ca_Kabuli_Chr04*	38370922	(G/A)
CakSNP7070	*Kabuli*	*Ca_Kabuli_Chr04*	38370921	(T/G)
CakSNP7071	*Kabuli*	*Ca_Kabuli_Chr04*	38382809	(T/C)
CakSNP7072	*Kabuli*	*Ca_Kabuli_Chr04*	38382890	(C/G)
CakSNP7073	*Kabuli*	*Ca_Kabuli_Chr04*	38421798	(A/C)
CakSNP7074	*Kabuli*	*Ca_Kabuli_Chr04*	38422037	(C/T)
CakSNP7075	*Kabuli*	*Ca_Kabuli_Chr04*	38421984	(C/T)
CakSNP7076	*Kabuli*	*Ca_Kabuli_Chr04*	38425675	(A/G)
CakSNP7077	*Kabuli*	*Ca_Kabuli_Chr04*	38425720	(T/A)
CakSNP7078	*Kabuli*	*Ca_Kabuli_Chr04*	38473540	(A/G)
CakSNP7079	*Kabuli*	*Ca_Kabuli_Chr04*	38473653	(T/C)
CakSNP7080	*Kabuli*	*Ca_Kabuli_Chr04*	38474123	(A/T)
CakSNP7081	*Kabuli*	*Ca_Kabuli_Chr04*	38474108	(T/C)
CakSNP7082	*Kabuli*	*Ca_Kabuli_Chr04*	38474192	(T/C)
CakSNP7083	*Kabuli*	*Ca_Kabuli_Chr04*	38475310	(A/C)
CakSNP7084	*Kabuli*	*Ca_Kabuli_Chr04*	38475425	(A/G)
CakSNP7085	*Kabuli*	*Ca_Kabuli_Chr04*	38475449	(T/A)
CakSNP7086	*Kabuli*	*Ca_Kabuli_Chr04*	38475442	(A/G)
CakSNP7087	*Kabuli*	*Ca_Kabuli_Chr04*	38475424	(C/G)
CakSNP7088	*Kabuli*	*Ca_Kabuli_Chr04*	38475547	(C/T)
CakSNP7089	*Kabuli*	*Ca_Kabuli_Chr04*	38540201	(C/T)
CakSNP7090	*Kabuli*	*Ca_Kabuli_Chr04*	38540236	(C/T)
CakSNP7091	*Kabuli*	*Ca_Kabuli_Chr04*	38540247	(G/A)
CakSNP7092	*Kabuli*	*Ca_Kabuli_Chr04*	38620579	(C/T)

**SNP IDs**	**Cultivars**	**Chromosomes/scaffolds**	**Physical positions (bp)**	**SNPs**
CakSNP7093	*Kabuli*	*Ca_Kabuli_Chr04*	38620620	(A/T)
CakSNP7094	*Kabuli*	*Ca_Kabuli_Chr04*	38659377	(T/C)
CakSNP7095	*Kabuli*	*Ca_Kabuli_Chr04*	38659398	(G/A)
CakSNP7096	*Kabuli*	*Ca_Kabuli_Chr04*	38659534	(T/A)
CakSNP7097	*Kabuli*	*Ca_Kabuli_Chr04*	38679987	(A/G)
CakSNP7098	*Kabuli*	*Ca_Kabuli_Chr04*	38721154	(A/G)
CakSNP7099	*Kabuli*	*Ca_Kabuli_Chr04*	38721160	(A/G)
CakSNP7100	*Kabuli*	*Ca_Kabuli_Chr04*	38721207	(A/C)
CakSNP7101	*Kabuli*	*Ca_Kabuli_Chr04*	38756507	(T/C)
CakSNP7102	*Kabuli*	*Ca_Kabuli_Chr04*	38756510	(C/G)
CakSNP7103	*Kabuli*	*Ca_Kabuli_Chr04*	38759640	(A/C)
CakSNP7104	*Kabuli*	*Ca_Kabuli_Chr04*	38770348	(T/A)
CakSNP7105	*Kabuli*	*Ca_Kabuli_Chr04*	38770370	(G/C)
CakSNP7106	*Kabuli*	*Ca_Kabuli_Chr04*	38770450	(C/G)
CakSNP7107	*Kabuli*	*Ca_Kabuli_Chr04*	38770510	(G/A)
CakSNP7108	*Kabuli*	*Ca_Kabuli_Chr04*	38770606	(G/A)
CakSNP7109	*Kabuli*	*Ca_Kabuli_Chr04*	38770582	(G/A)
CakSNP7110	*Kabuli*	*Ca_Kabuli_Chr04*	38785535	(G/A)
CakSNP7111	*Kabuli*	*Ca_Kabuli_Chr04*	38829959	(T/G)
CakSNP7112	*Kabuli*	*Ca_Kabuli_Chr04*	38833304	(C/A)
CakSNP7113	*Kabuli*	*Ca_Kabuli_Chr04*	38833280	(C/T)
CakSNP7114	*Kabuli*	*Ca_Kabuli_Chr04*	38833333	(A/C)
CakSNP7115	*Kabuli*	*Ca_Kabuli_Chr04*	38833414	(A/C)
CakSNP7116	*Kabuli*	*Ca_Kabuli_Chr04*	38834383	(G/A)
CakSNP7117	*Kabuli*	*Ca_Kabuli_Chr04*	38834405	(G/A)
CakSNP7118	*Kabuli*	*Ca_Kabuli_Chr04*	38834423	(G/C)
CakSNP7119	*Kabuli*	*Ca_Kabuli_Chr04*	38834431	(G/A)
CakSNP7120	*Kabuli*	*Ca_Kabuli_Chr04*	38834448	(A/T)
CakSNP7121	*Kabuli*	*Ca_Kabuli_Chr04*	38834477	(A/G)
CakSNP7122	*Kabuli*	*Ca_Kabuli_Chr04*	38834474	(G/A)
CakSNP7123	*Kabuli*	*Ca_Kabuli_Chr04*	38834453	(C/T)
CakSNP7124	*Kabuli*	*Ca_Kabuli_Chr04*	38888779	(C/T)
CakSNP7125	*Kabuli*	*Ca_Kabuli_Chr04*	38888791	(G/C)
CakSNP7126	*Kabuli*	*Ca_Kabuli_Chr04*	38889023	(C/T)
CakSNP7127	*Kabuli*	*Ca_Kabuli_Chr04*	38918394	(A/T)
CakSNP7128	*Kabuli*	*Ca_Kabuli_Chr04*	38918424	(G/A)
CakSNP7129	*Kabuli*	*Ca_Kabuli_Chr04*	38918430	(C/A)
CakSNP7130	*Kabuli*	*Ca_Kabuli_Chr04*	38918628	(C/T)
CakSNP7131	*Kabuli*	*Ca_Kabuli_Chr04*	38918547	(T/C)
CakSNP7132	*Kabuli*	*Ca_Kabuli_Chr04*	38918649	(G/C)
CakSNP7133	*Kabuli*	*Ca_Kabuli_Chr04*	38929681	(A/G)

SNP IDs	Cultivars	Chromosomes/scaffolds	Physical positions (bp)	SNPs
CakSNP7134	*Kabuli*	*Ca_Kabuli_Chr04*	38948414	(C/A)
CakSNP7135	*Kabuli*	*Ca_Kabuli_Chr04*	38961521	(G/A)
CakSNP7136	*Kabuli*	*Ca_Kabuli_Chr04*	38978462	(A/G)
CakSNP7137	*Kabuli*	*Ca_Kabuli_Chr04*	39031801	(C/T)
CakSNP7138	*Kabuli*	*Ca_Kabuli_Chr04*	39031823	(G/A)
CakSNP7139	*Kabuli*	*Ca_Kabuli_Chr04*	39042938	(T/G)
CakSNP7140	*Kabuli*	*Ca_Kabuli_Chr04*	39042921	(T/C)
CakSNP7141	*Kabuli*	*Ca_Kabuli_Chr04*	39057991	(T/C)
CakSNP7142	*Kabuli*	*Ca_Kabuli_Chr04*	39058011	(G/C)
CakSNP7143	*Kabuli*	*Ca_Kabuli_Chr04*	39081951	(A/G)
CakSNP7144	*Kabuli*	*Ca_Kabuli_Chr04*	39110408	(T/C)
CakSNP7145	*Kabuli*	*Ca_Kabuli_Chr04*	39110448	(A/G)
CakSNP7146	*Kabuli*	*Ca_Kabuli_Chr04*	39110735	(A/G)
CakSNP7147	*Kabuli*	*Ca_Kabuli_Chr04*	39113309	(T/C)
CakSNP7148	*Kabuli*	*Ca_Kabuli_Chr04*	39113327	(G/A)
CakSNP7149	*Kabuli*	*Ca_Kabuli_Chr04*	39113330	(G/A)
CakSNP7150	*Kabuli*	*Ca_Kabuli_Chr04*	39113401	(A/T)
CakSNP7151	*Kabuli*	*Ca_Kabuli_Chr04*	39114000	(A/C)
CakSNP7152	*Kabuli*	*Ca_Kabuli_Chr04*	39136826	(C/T)
CakSNP7153	*Kabuli*	*Ca_Kabuli_Chr04*	39189012	(A/G)
CakSNP7154	*Kabuli*	*Ca_Kabuli_Chr04*	39189016	(G/A)
CakSNP7155	*Kabuli*	*Ca_Kabuli_Chr04*	39268505	(T/C)
CakSNP7156	*Kabuli*	*Ca_Kabuli_Chr04*	39306900	(G/A)
CakSNP7157	*Kabuli*	*Ca_Kabuli_Chr04*	39635746	(T/A)
CakSNP7158	*Kabuli*	*Ca_Kabuli_Chr04*	39635721	(A/G)
CakSNP7159	*Kabuli*	*Ca_Kabuli_Chr04*	39635720	(G/A)
CakSNP7160	*Kabuli*	*Ca_Kabuli_Chr04*	39635718	(C/T)
CakSNP7161	*Kabuli*	*Ca_Kabuli_Chr04*	39650872	(T/C)
CakSNP7162	*Kabuli*	*Ca_Kabuli_Chr04*	39659757	(C/T)
CakSNP7163	*Kabuli*	*Ca_Kabuli_Chr04*	39659750	(G/A)
CakSNP7164	*Kabuli*	*Ca_Kabuli_Chr04*	39659832	(C/T)
CakSNP7165	*Kabuli*	*Ca_Kabuli_Chr04*	39715087	(A/G)
CakSNP7166	*Kabuli*	*Ca_Kabuli_Chr04*	39719606	(C/G)
CakSNP7167	*Kabuli*	*Ca_Kabuli_Chr04*	39719747	(G/T)
CakSNP7168	*Kabuli*	*Ca_Kabuli_Chr04*	39779796	(G/A)
CakSNP7169	*Kabuli*	*Ca_Kabuli_Chr04*	39803305	(T/C)
CakSNP7170	*Kabuli*	*Ca_Kabuli_Chr04*	39849612	(G/T)
CakSNP7171	*Kabuli*	*Ca_Kabuli_Chr04*	39849657	(G/A)
CakSNP7172	*Kabuli*	*Ca_Kabuli_Chr04*	39964930	(T/C)
CakSNP7173	*Kabuli*	*Ca_Kabuli_Chr04*	40010119	(A/C)
CakSNP7174	*Kabuli*	*Ca_Kabuli_Chr04*	40010201	(A/G)

SNP IDs	Cultivars	Chromosomes/scaffolds	Physical positions (bp)	SNPs
CakSNP7175	*Kabuli*	*Ca_Kabuli_Chr04*	40010177	(G/A)
CakSNP7176	*Kabuli*	*Ca_Kabuli_Chr04*	40010165	(A/C)
CakSNP7177	*Kabuli*	*Ca_Kabuli_Chr04*	40059880	(C/T)
CakSNP7178	*Kabuli*	*Ca_Kabuli_Chr04*	40060022	(C/T)
CakSNP7179	*Kabuli*	*Ca_Kabuli_Chr04*	40060063	(T/G)
CakSNP7180	*Kabuli*	*Ca_Kabuli_Chr04*	40061195	(T/C)
CakSNP7181	*Kabuli*	*Ca_Kabuli_Chr04*	40061262	(A/C)
CakSNP7182	*Kabuli*	*Ca_Kabuli_Chr04*	40065505	(T/A)
CakSNP7183	*Kabuli*	*Ca_Kabuli_Chr04*	40134460	(A/G)
CakSNP7184	*Kabuli*	*Ca_Kabuli_Chr04*	40134518	(T/C)
CakSNP7185	*Kabuli*	*Ca_Kabuli_Chr04*	40149064	(C/T)
CakSNP7186	*Kabuli*	*Ca_Kabuli_Chr04*	40149132	(G/C)
CakSNP7187	*Kabuli*	*Ca_Kabuli_Chr04*	40149324	(G/C)
CakSNP7188	*Kabuli*	*Ca_Kabuli_Chr04*	40169176	(T/G)
CakSNP7189	*Kabuli*	*Ca_Kabuli_Chr04*	40185606	(A/G)
CakSNP7190	*Kabuli*	*Ca_Kabuli_Chr04*	40199692	(A/C)
CakSNP7191	*Kabuli*	*Ca_Kabuli_Chr04*	40218375	(G/T)
CakSNP7192	*Kabuli*	*Ca_Kabuli_Chr04*	40218410	(G/A)
CakSNP7193	*Kabuli*	*Ca_Kabuli_Chr04*	40267713	(G/T)
CakSNP7194	*Kabuli*	*Ca_Kabuli_Chr04*	40267719	(G/A)
CakSNP7195	*Kabuli*	*Ca_Kabuli_Chr04*	40279513	(G/T)
CakSNP7196	*Kabuli*	*Ca_Kabuli_Chr04*	40313721	(A/G)
CakSNP7197	*Kabuli*	*Ca_Kabuli_Chr04*	40349170	(C/T)
CakSNP7198	*Kabuli*	*Ca_Kabuli_Chr04*	40368028	(C/T)
CakSNP7199	*Kabuli*	*Ca_Kabuli_Chr04*	40368278	(T/C)
CakSNP7200	*Kabuli*	*Ca_Kabuli_Chr04*	40368765	(G/A)
CakSNP7201	*Kabuli*	*Ca_Kabuli_Chr04*	40375386	(C/T)
CakSNP7202	*Kabuli*	*Ca_Kabuli_Chr04*	40375361	(A/G)
CakSNP7203	*Kabuli*	*Ca_Kabuli_Chr04*	40429387	(G/A)
CakSNP7204	*Kabuli*	*Ca_Kabuli_Chr04*	40471412	(G/C)
CakSNP7205	*Kabuli*	*Ca_Kabuli_Chr04*	40472097	(A/G)
CakSNP7206	*Kabuli*	*Ca_Kabuli_Chr04*	40513733	(C/T)
CakSNP7207	*Kabuli*	*Ca_Kabuli_Chr04*	40583205	(G/T)
CakSNP7208	*Kabuli*	*Ca_Kabuli_Chr04*	40583206	(A/T)
CakSNP7209	*Kabuli*	*Ca_Kabuli_Chr04*	40583211	(G/T)
CakSNP7210	*Kabuli*	*Ca_Kabuli_Chr04*	40583214	(G/A)
CakSNP7211	*Kabuli*	*Ca_Kabuli_Chr04*	40583216	(C/A)
CakSNP7212	*Kabuli*	*Ca_Kabuli_Chr04*	40583217	(G/A)
CakSNP7213	*Kabuli*	*Ca_Kabuli_Chr04*	40583219	(G/A)
CakSNP7214	*Kabuli*	*Ca_Kabuli_Chr04*	40597192	(G/A)
CakSNP7215	*Kabuli*	*Ca_Kabuli_Chr04*	40597534	(A/G)

SNP IDs	Cultivars	Chromosomes/scaffolds	Physical positions (bp)	SNPs
CakSNP7216	*Kabuli*	*Ca_Kabuli_Chr04*	40597464	(G/A)
CakSNP7217	*Kabuli*	*Ca_Kabuli_Chr04*	40723208	(C/G)
CakSNP7218	*Kabuli*	*Ca_Kabuli_Chr04*	40723212	(G/A)
CakSNP7219	*Kabuli*	*Ca_Kabuli_Chr04*	40734572	(T/G)
CakSNP7220	*Kabuli*	*Ca_Kabuli_Chr04*	40734765	(G/A)
CakSNP7221	*Kabuli*	*Ca_Kabuli_Chr04*	40738927	(C/T)
CakSNP7222	*Kabuli*	*Ca_Kabuli_Chr04*	40795060	(G/A)
CakSNP7223	*Kabuli*	*Ca_Kabuli_Chr04*	40795085	(C/G)
CakSNP7224	*Kabuli*	*Ca_Kabuli_Chr04*	40795137	(T/G)
CakSNP7225	*Kabuli*	*Ca_Kabuli_Chr04*	40801783	(T/C)
CakSNP7226	*Kabuli*	*Ca_Kabuli_Chr04*	40801791	(C/G)
CakSNP7227	*Kabuli*	*Ca_Kabuli_Chr04*	40805740	(C/A)
CakSNP7228	*Kabuli*	*Ca_Kabuli_Chr04*	40806993	(A/G)
CakSNP7229	*Kabuli*	*Ca_Kabuli_Chr04*	40839967	(G/A)
CakSNP7230	*Kabuli*	*Ca_Kabuli_Chr04*	40839949	(G/A)
CakSNP7231	*Kabuli*	*Ca_Kabuli_Chr04*	40852381	(A/C)
CakSNP7232	*Kabuli*	*Ca_Kabuli_Chr04*	40852475	(T/C)
CakSNP7233	*Kabuli*	*Ca_Kabuli_Chr04*	40912677	(A/G)
CakSNP7234	*Kabuli*	*Ca_Kabuli_Chr04*	40912681	(A/G)
CakSNP7235	*Kabuli*	*Ca_Kabuli_Chr04*	40912714	(C/G)
CakSNP7236	*Kabuli*	*Ca_Kabuli_Chr04*	40912731	(C/T)
CakSNP7237	*Kabuli*	*Ca_Kabuli_Chr04*	40917776	(C/A)
CakSNP7238	*Kabuli*	*Ca_Kabuli_Chr04*	40917777	(T/A)
CakSNP7239	*Kabuli*	*Ca_Kabuli_Chr04*	40917807	(C/T)
CakSNP7240	*Kabuli*	*Ca_Kabuli_Chr04*	40941283	(A/G)
CakSNP7241	*Kabuli*	*Ca_Kabuli_Chr04*	41032564	(A/G)
CakSNP7242	*Kabuli*	*Ca_Kabuli_Chr04*	41032590	(C/A)
CakSNP7243	*Kabuli*	*Ca_Kabuli_Chr04*	41032599	(T/C)
CakSNP7244	*Kabuli*	*Ca_Kabuli_Chr04*	41032608	(T/C)
CakSNP7245	*Kabuli*	*Ca_Kabuli_Chr04*	41070048	(C/G)
CakSNP7246	*Kabuli*	*Ca_Kabuli_Chr04*	41070017	(T/A)
CakSNP7247	*Kabuli*	*Ca_Kabuli_Chr04*	41094082	(T/C)
CakSNP7248	*Kabuli*	*Ca_Kabuli_Chr04*	41094554	(A/C)
CakSNP7249	*Kabuli*	*Ca_Kabuli_Chr04*	41094685	(G/C)
CakSNP7250	*Kabuli*	*Ca_Kabuli_Chr04*	41117822	(C/A)
CakSNP7251	*Kabuli*	*Ca_Kabuli_Chr04*	41141086	(G/T)
CakSNP7252	*Kabuli*	*Ca_Kabuli_Chr04*	41142299	(T/C)
CakSNP7253	*Kabuli*	*Ca_Kabuli_Chr04*	41142290	(A/G)
CakSNP7254	*Kabuli*	*Ca_Kabuli_Chr04*	41142288	(T/C)
CakSNP7255	*Kabuli*	*Ca_Kabuli_Chr04*	41144277	(T/A)
CakSNP7256	*Kabuli*	*Ca_Kabuli_Chr04*	41145767	(C/T)

SNP IDs	Cultivars	Chromosomes/scaffolds	Physical positions (bp)	SNPs
CakSNP7257	*Kabuli*	*Ca_Kabuli_Chr04*	41145875	(G/A)
CakSNP7258	*Kabuli*	*Ca_Kabuli_Chr04*	41207328	(C/T)
CakSNP7259	*Kabuli*	*Ca_Kabuli_Chr04*	41207356	(C/T)
CakSNP7260	*Kabuli*	*Ca_Kabuli_Chr04*	41207392	(T/G)
CakSNP7261	*Kabuli*	*Ca_Kabuli_Chr04*	41207354	(A/C)
CakSNP7262	*Kabuli*	*Ca_Kabuli_Chr04*	41209521	(T/G)
CakSNP7263	*Kabuli*	*Ca_Kabuli_Chr04*	41209711	(C/A)
CakSNP7264	*Kabuli*	*Ca_Kabuli_Chr04*	41215850	(A/C)
CakSNP7265	*Kabuli*	*Ca_Kabuli_Chr04*	41228918	(T/C)
CakSNP7266	*Kabuli*	*Ca_Kabuli_Chr04*	41229605	(C/T)
CakSNP7267	*Kabuli*	*Ca_Kabuli_Chr04*	41277786	(G/A)
CakSNP7268	*Kabuli*	*Ca_Kabuli_Chr04*	41277799	(T/G)
CakSNP7269	*Kabuli*	*Ca_Kabuli_Chr04*	41292152	(T/C)
CakSNP7270	*Kabuli*	*Ca_Kabuli_Chr04*	41319598	(T/C)
CakSNP7271	*Kabuli*	*Ca_Kabuli_Chr04*	41319610	(G/A)
CakSNP7272	*Kabuli*	*Ca_Kabuli_Chr04*	41344739	(A/T)
CakSNP7273	*Kabuli*	*Ca_Kabuli_Chr04*	41427081	(A/T)
CakSNP7274	*Kabuli*	*Ca_Kabuli_Chr04*	41427086	(C/A)
CakSNP7275	*Kabuli*	*Ca_Kabuli_Chr04*	41427093	(T/G)
CakSNP7276	*Kabuli*	*Ca_Kabuli_Chr04*	41445171	(T/C)
CakSNP7277	*Kabuli*	*Ca_Kabuli_Chr04*	41445212	(A/G)
CakSNP7278	*Kabuli*	*Ca_Kabuli_Chr04*	41445225	(A/T)
CakSNP7279	*Kabuli*	*Ca_Kabuli_Chr04*	41445242	(C/T)
CakSNP7280	*Kabuli*	*Ca_Kabuli_Chr04*	41458426	(A/G)
CakSNP7281	*Kabuli*	*Ca_Kabuli_Chr04*	41500284	(A/C)
CakSNP7282	*Kabuli*	*Ca_Kabuli_Chr04*	41500286	(A/G)
CakSNP7283	*Kabuli*	*Ca_Kabuli_Chr04*	41512840	(G/A)
CakSNP7284	*Kabuli*	*Ca_Kabuli_Chr04*	41568682	(A/C)
CakSNP7285	*Kabuli*	*Ca_Kabuli_Chr04*	41629852	(T/A)
CakSNP7286	*Kabuli*	*Ca_Kabuli_Chr04*	41648927	(C/T)
CakSNP7287	*Kabuli*	*Ca_Kabuli_Chr04*	41648998	(A/G)
CakSNP7288	*Kabuli*	*Ca_Kabuli_Chr04*	41675299	(A/T)
CakSNP7289	*Kabuli*	*Ca_Kabuli_Chr04*	41698291	(A/G)
CakSNP7290	*Kabuli*	*Ca_Kabuli_Chr04*	41698386	(A/G)
CakSNP7291	*Kabuli*	*Ca_Kabuli_Chr04*	41700712	(C/A)
CakSNP7292	*Kabuli*	*Ca_Kabuli_Chr04*	41721289	(G/A)
CakSNP7293	*Kabuli*	*Ca_Kabuli_Chr04*	41772690	(C/T)
CakSNP7294	*Kabuli*	*Ca_Kabuli_Chr04*	41772823	(G/A)
CakSNP7295	*Kabuli*	*Ca_Kabuli_Chr04*	41772796	(T/A)
CakSNP7296	*Kabuli*	*Ca_Kabuli_Chr04*	41772794	(T/C)
CakSNP7297	*Kabuli*	*Ca_Kabuli_Chr04*	41864826	(G/A)

SNP IDs	Cultivars	Chromosomes/scaffolds	Physical positions (bp)	SNPs
CakSNP7298	*Kabuli*	*Ca_Kabuli_Chr04*	41864846	(C/G)
CakSNP7299	*Kabuli*	*Ca_Kabuli_Chr04*	41938784	(A/G)
CakSNP7300	*Kabuli*	*Ca_Kabuli_Chr04*	41938812	(T/G)
CakSNP7301	*Kabuli*	*Ca_Kabuli_Chr04*	41956050	(C/G)
CakSNP7302	*Kabuli*	*Ca_Kabuli_Chr04*	41956085	(T/C)
CakSNP7303	*Kabuli*	*Ca_Kabuli_Chr04*	41987193	(A/G)
CakSNP7304	*Kabuli*	*Ca_Kabuli_Chr04*	42056479	(G/A)
CakSNP7305	*Kabuli*	*Ca_Kabuli_Chr04*	42092379	(T/G)
CakSNP7306	*Kabuli*	*Ca_Kabuli_Chr04*	42092381	(A/G)
CakSNP7307	*Kabuli*	*Ca_Kabuli_Chr04*	42092385	(G/A)
CakSNP7308	*Kabuli*	*Ca_Kabuli_Chr04*	42105737	(C/T)
CakSNP7309	*Kabuli*	*Ca_Kabuli_Chr04*	42105701	(A/T)
CakSNP7310	*Kabuli*	*Ca_Kabuli_Chr04*	42108980	(C/A)
CakSNP7311	*Kabuli*	*Ca_Kabuli_Chr04*	42246499	(C/T)
CakSNP7312	*Kabuli*	*Ca_Kabuli_Chr04*	42246527	(C/A)
CakSNP7313	*Kabuli*	*Ca_Kabuli_Chr04*	42246565	(T/G)
CakSNP7314	*Kabuli*	*Ca_Kabuli_Chr04*	42246566	(T/G)
CakSNP7315	*Kabuli*	*Ca_Kabuli_Chr04*	42246572	(T/G)
CakSNP7316	*Kabuli*	*Ca_Kabuli_Chr04*	42246653	(C/G)
CakSNP7317	*Kabuli*	*Ca_Kabuli_Chr04*	42273252	(G/A)
CakSNP7318	*Kabuli*	*Ca_Kabuli_Chr04*	42353949	(C/T)
CakSNP7319	*Kabuli*	*Ca_Kabuli_Chr04*	42499699	(A/T)
CakSNP7320	*Kabuli*	*Ca_Kabuli_Chr04*	42603642	(T/C)
CakSNP7321	*Kabuli*	*Ca_Kabuli_Chr04*	42768142	(T/G)
CakSNP7322	*Kabuli*	*Ca_Kabuli_Chr04*	42807483	(G/A)
CakSNP7323	*Kabuli*	*Ca_Kabuli_Chr04*	42807518	(A/G)
CakSNP7324	*Kabuli*	*Ca_Kabuli_Chr04*	42807509	(C/T)
CakSNP7325	*Kabuli*	*Ca_Kabuli_Chr04*	43319846	(A/G)
CakSNP7326	*Kabuli*	*Ca_Kabuli_Chr04*	43324547	(G/C)
CakSNP7327	*Kabuli*	*Ca_Kabuli_Chr04*	43431770	(C/T)
CakSNP7328	*Kabuli*	*Ca_Kabuli_Chr04*	43431772	(T/A)
CakSNP7329	*Kabuli*	*Ca_Kabuli_Chr04*	43431815	(G/T)
CakSNP7330	*Kabuli*	*Ca_Kabuli_Chr04*	43446753	(T/A)
CakSNP7331	*Kabuli*	*Ca_Kabuli_Chr04*	43447262	(A/T)
CakSNP7332	*Kabuli*	*Ca_Kabuli_Chr04*	43476193	(T/C)
CakSNP7333	*Kabuli*	*Ca_Kabuli_Chr04*	43653225	(A/G)
CakSNP7334	*Kabuli*	*Ca_Kabuli_Chr04*	43653226	(C/T)
CakSNP7335	*Kabuli*	*Ca_Kabuli_Chr04*	43662224	(A/C)
CakSNP7336	*Kabuli*	*Ca_Kabuli_Chr04*	43677006	(T/G)
CakSNP7337	*Kabuli*	*Ca_Kabuli_Chr04*	43677222	(C/T)
CakSNP7338	*Kabuli*	*Ca_Kabuli_Chr04*	43722728	(T/C)

SNP IDs	Cultivars	Chromosomes/scaffolds	Physical positions (bp)	SNPs
CakSNP7339	*Kabuli*	*Ca_Kabuli_Chr04*	43722727	(A/G)
CakSNP7340	*Kabuli*	*Ca_Kabuli_Chr04*	43722704	(C/G)
CakSNP7341	*Kabuli*	*Ca_Kabuli_Chr04*	43723219	(T/G)
CakSNP7342	*Kabuli*	*Ca_Kabuli_Chr04*	43723224	(C/A)
CakSNP7343	*Kabuli*	*Ca_Kabuli_Chr04*	43723248	(T/C)
CakSNP7344	*Kabuli*	*Ca_Kabuli_Chr04*	43726406	(C/T)
CakSNP7345	*Kabuli*	*Ca_Kabuli_Chr04*	43729063	(G/T)
CakSNP7346	*Kabuli*	*Ca_Kabuli_Chr04*	43729261	(C/G)
CakSNP7347	*Kabuli*	*Ca_Kabuli_Chr04*	43729266	(A/G)
CakSNP7348	*Kabuli*	*Ca_Kabuli_Chr04*	43807399	(C/T)
CakSNP7349	*Kabuli*	*Ca_Kabuli_Chr04*	43807460	(C/T)
CakSNP7350	*Kabuli*	*Ca_Kabuli_Chr04*	43840666	(C/G)
CakSNP7351	*Kabuli*	*Ca_Kabuli_Chr04*	43840650	(C/T)
CakSNP7352	*Kabuli*	*Ca_Kabuli_Chr04*	43979065	(A/G)
CakSNP7353	*Kabuli*	*Ca_Kabuli_Chr04*	44008835	(T/C)
CakSNP7354	*Kabuli*	*Ca_Kabuli_Chr04*	44059060	(C/T)
CakSNP7355	*Kabuli*	*Ca_Kabuli_Chr04*	44059061	(G/A)
CakSNP7356	*Kabuli*	*Ca_Kabuli_Chr04*	44067780	(G/A)
CakSNP7357	*Kabuli*	*Ca_Kabuli_Chr04*	44090929	(T/G)
CakSNP7358	*Kabuli*	*Ca_Kabuli_Chr04*	44186434	(C/G)
CakSNP7359	*Kabuli*	*Ca_Kabuli_Chr04*	44239783	(A/C)
CakSNP7360	*Kabuli*	*Ca_Kabuli_Chr04*	44239883	(A/T)
CakSNP7361	*Kabuli*	*Ca_Kabuli_Chr04*	44250159	(C/T)
CakSNP7362	*Kabuli*	*Ca_Kabuli_Chr04*	44261184	(A/C)
CakSNP7363	*Kabuli*	*Ca_Kabuli_Chr04*	44261909	(T/A)
CakSNP7364	*Kabuli*	*Ca_Kabuli_Chr04*	44266692	(T/A)
CakSNP7365	*Kabuli*	*Ca_Kabuli_Chr04*	44266695	(G/A)
CakSNP7366	*Kabuli*	*Ca_Kabuli_Chr04*	44266758	(C/T)
CakSNP7367	*Kabuli*	*Ca_Kabuli_Chr04*	44266819	(G/A)
CakSNP7368	*Kabuli*	*Ca_Kabuli_Chr04*	44353354	(C/T)
CakSNP7369	*Kabuli*	*Ca_Kabuli_Chr04*	44353343	(G/A)
CakSNP7370	*Kabuli*	*Ca_Kabuli_Chr04*	44375761	(T/C)
CakSNP7371	*Kabuli*	*Ca_Kabuli_Chr04*	44375831	(T/C)
CakSNP7372	*Kabuli*	*Ca_Kabuli_Chr04*	44382515	(A/G)
CakSNP7373	*Kabuli*	*Ca_Kabuli_Chr04*	44382677	(C/T)
CakSNP7374	*Kabuli*	*Ca_Kabuli_Chr04*	44382671	(C/G)
CakSNP7375	*Kabuli*	*Ca_Kabuli_Chr04*	44382614	(C/A)
CakSNP7376	*Kabuli*	*Ca_Kabuli_Chr04*	44382602	(A/G)
CakSNP7377	*Kabuli*	*Ca_Kabuli_Chr04*	44386579	(A/G)
CakSNP7378	*Kabuli*	*Ca_Kabuli_Chr04*	44431319	(A/C)
CakSNP7379	*Kabuli*	*Ca_Kabuli_Chr04*	44431353	(G/A)

SNP IDs	Cultivars	Chromosomes/scaffolds	Physical positions (bp)	SNPs
CakSNP7380	*Kabuli*	*Ca_Kabuli_Chr04*	44439372	(C/T)
CakSNP7381	*Kabuli*	*Ca_Kabuli_Chr04*	44439503	(C/A)
CakSNP7382	*Kabuli*	*Ca_Kabuli_Chr04*	44439495	(G/A)
CakSNP7383	*Kabuli*	*Ca_Kabuli_Chr04*	44439493	(A/G)
CakSNP7384	*Kabuli*	*Ca_Kabuli_Chr04*	44439492	(G/T)
CakSNP7385	*Kabuli*	*Ca_Kabuli_Chr04*	44439448	(T/C)
CakSNP7386	*Kabuli*	*Ca_Kabuli_Chr04*	44446468	(G/A)
CakSNP7387	*Kabuli*	*Ca_Kabuli_Chr04*	44446575	(G/A)
CakSNP7388	*Kabuli*	*Ca_Kabuli_Chr04*	44446506	(C/T)
CakSNP7389	*Kabuli*	*Ca_Kabuli_Chr04*	44446503	(T/G)
CakSNP7390	*Kabuli*	*Ca_Kabuli_Chr04*	44446497	(C/T)
CakSNP7391	*Kabuli*	*Ca_Kabuli_Chr04*	44472282	(G/A)
CakSNP7392	*Kabuli*	*Ca_Kabuli_Chr04*	44472278	(A/G)
CakSNP7393	*Kabuli*	*Ca_Kabuli_Chr04*	44472237	(T/A)
CakSNP7394	*Kabuli*	*Ca_Kabuli_Chr04*	44590652	(T/C)
CakSNP7395	*Kabuli*	*Ca_Kabuli_Chr04*	44590678	(G/A)
CakSNP7396	*Kabuli*	*Ca_Kabuli_Chr04*	44590697	(A/C)
CakSNP7397	*Kabuli*	*Ca_Kabuli_Chr04*	44591405	(G/T)
CakSNP7398	*Kabuli*	*Ca_Kabuli_Chr04*	44754355	(C/T)
CakSNP7399	*Kabuli*	*Ca_Kabuli_Chr04*	44802374	(G/A)
CakSNP7400	*Kabuli*	*Ca_Kabuli_Chr04*	44802364	(A/C)
CakSNP7401	*Kabuli*	*Ca_Kabuli_Chr04*	44803694	(T/A)
CakSNP7402	*Kabuli*	*Ca_Kabuli_Chr04*	44811519	(G/A)
CakSNP7403	*Kabuli*	*Ca_Kabuli_Chr04*	44814857	(A/G)
CakSNP7404	*Kabuli*	*Ca_Kabuli_Chr04*	44814981	(T/C)
CakSNP7405	*Kabuli*	*Ca_Kabuli_Chr04*	44814982	(A/G)
CakSNP7406	*Kabuli*	*Ca_Kabuli_Chr04*	44824103	(G/T)
CakSNP7407	*Kabuli*	*Ca_Kabuli_Chr04*	44824105	(T/G)
CakSNP7408	*Kabuli*	*Ca_Kabuli_Chr04*	44824178	(C/G)
CakSNP7409	*Kabuli*	*Ca_Kabuli_Chr04*	44824258	(A/G)
CakSNP7410	*Kabuli*	*Ca_Kabuli_Chr04*	44832121	(G/C)
CakSNP7411	*Kabuli*	*Ca_Kabuli_Chr04*	44832144	(T/C)
CakSNP7412	*Kabuli*	*Ca_Kabuli_Chr04*	44906128	(C/A)
CakSNP7413	*Kabuli*	*Ca_Kabuli_Chr04*	44906255	(A/G)
CakSNP7414	*Kabuli*	*Ca_Kabuli_Chr04*	44979108	(G/A)
CakSNP7415	*Kabuli*	*Ca_Kabuli_Chr04*	45019764	(T/C)
CakSNP7416	*Kabuli*	*Ca_Kabuli_Chr04*	45019778	(T/C)
CakSNP7417	*Kabuli*	*Ca_Kabuli_Chr04*	45019787	(G/C)
CakSNP7418	*Kabuli*	*Ca_Kabuli_Chr04*	45019805	(C/A)
CakSNP7419	*Kabuli*	*Ca_Kabuli_Chr04*	45019807	(C/T)
CakSNP7420	*Kabuli*	*Ca_Kabuli_Chr04*	45019814	(C/A)

**SNP IDs**	**Cultivars**	**Chromosomes/scaffolds**	**Physical positions (bp)**	**SNPs**
CakSNP7421	*Kabuli*	*Ca_Kabuli_Chr04*	45019820	(C/T)
CakSNP7422	*Kabuli*	*Ca_Kabuli_Chr04*	45019824	(A/C)
CakSNP7423	*Kabuli*	*Ca_Kabuli_Chr04*	45019867	(C/G)
CakSNP7424	*Kabuli*	*Ca_Kabuli_Chr04*	45019858	(G/T)
CakSNP7425	*Kabuli*	*Ca_Kabuli_Chr04*	45019848	(C/T)
CakSNP7426	*Kabuli*	*Ca_Kabuli_Chr04*	45035589	(G/T)
CakSNP7427	*Kabuli*	*Ca_Kabuli_Chr04*	45035629	(A/C)
CakSNP7428	*Kabuli*	*Ca_Kabuli_Chr04*	45035745	(A/G)
CakSNP7429	*Kabuli*	*Ca_Kabuli_Chr04*	45067534	(C/T)
CakSNP7430	*Kabuli*	*Ca_Kabuli_Chr04*	45104767	(G/A)
CakSNP7431	*Kabuli*	*Ca_Kabuli_Chr04*	45104779	(C/A)
CakSNP7432	*Kabuli*	*Ca_Kabuli_Chr04*	45104805	(A/G)
CakSNP7433	*Kabuli*	*Ca_Kabuli_Chr04*	45141976	(C/A)
CakSNP7434	*Kabuli*	*Ca_Kabuli_Chr04*	45141990	(A/G)
CakSNP7435	*Kabuli*	*Ca_Kabuli_Chr04*	45142020	(T/C)
CakSNP7436	*Kabuli*	*Ca_Kabuli_Chr04*	45142009	(C/T)
CakSNP7437	*Kabuli*	*Ca_Kabuli_Chr04*	45142003	(A/G)
CakSNP7438	*Kabuli*	*Ca_Kabuli_Chr04*	45142077	(C/A)
CakSNP7439	*Kabuli*	*Ca_Kabuli_Chr04*	45142087	(T/A)
CakSNP7440	*Kabuli*	*Ca_Kabuli_Chr04*	45142104	(T/C)
CakSNP7441	*Kabuli*	*Ca_Kabuli_Chr04*	45165191	(T/C)
CakSNP7442	*Kabuli*	*Ca_Kabuli_Chr04*	45165246	(C/G)
CakSNP7443	*Kabuli*	*Ca_Kabuli_Chr04*	45165240	(T/C)
CakSNP7444	*Kabuli*	*Ca_Kabuli_Chr04*	45165231	(A/C)
CakSNP7445	*Kabuli*	*Ca_Kabuli_Chr04*	45206203	(G/T)
CakSNP7446	*Kabuli*	*Ca_Kabuli_Chr04*	45206209	(G/C)
CakSNP7447	*Kabuli*	*Ca_Kabuli_Chr04*	45220084	(G/T)
CakSNP7448	*Kabuli*	*Ca_Kabuli_Chr04*	45220642	(C/T)
CakSNP7449	*Kabuli*	*Ca_Kabuli_Chr04*	45220916	(T/C)
CakSNP7450	*Kabuli*	*Ca_Kabuli_Chr04*	45225063	(T/C)
CakSNP7451	*Kabuli*	*Ca_Kabuli_Chr04*	45225059	(G/A)
CakSNP7452	*Kabuli*	*Ca_Kabuli_Chr04*	45224997	(G/C)
CakSNP7453	*Kabuli*	*Ca_Kabuli_Chr04*	45225077	(A/T)
CakSNP7454	*Kabuli*	*Ca_Kabuli_Chr04*	45225093	(G/A)
CakSNP7455	*Kabuli*	*Ca_Kabuli_Chr04*	45225145	(T/A)
CakSNP7456	*Kabuli*	*Ca_Kabuli_Chr04*	45225154	(T/A)
CakSNP7457	*Kabuli*	*Ca_Kabuli_Chr04*	45225155	(C/A)
CakSNP7458	*Kabuli*	*Ca_Kabuli_Chr04*	45225210	(G/T)
CakSNP7459	*Kabuli*	*Ca_Kabuli_Chr04*	45225193	(A/G)
CakSNP7460	*Kabuli*	*Ca_Kabuli_Chr04*	45225165	(T/C)
CakSNP7461	*Kabuli*	*Ca_Kabuli_Chr04*	45231839	(T/C)

**SNP IDs**	**Cultivars**	**Chromosomes/scaffolds**	**Physical positions (bp)**	**SNPs**
CakSNP7462	*Kabuli*	*Ca_Kabuli_Chr04*	45231867	(A/T)
CakSNP7463	*Kabuli*	*Ca_Kabuli_Chr04*	45231868	(C/A)
CakSNP7464	*Kabuli*	*Ca_Kabuli_Chr04*	45231893	(G/T)
CakSNP7465	*Kabuli*	*Ca_Kabuli_Chr04*	45292852	(G/T)
CakSNP7466	*Kabuli*	*Ca_Kabuli_Chr04*	45292918	(T/G)
CakSNP7467	*Kabuli*	*Ca_Kabuli_Chr04*	45292981	(G/T)
CakSNP7468	*Kabuli*	*Ca_Kabuli_Chr04*	45292969	(T/A)
CakSNP7469	*Kabuli*	*Ca_Kabuli_Chr04*	45292960	(A/T)
CakSNP7470	*Kabuli*	*Ca_Kabuli_Chr04*	45552675	(C/A)
CakSNP7471	*Kabuli*	*Ca_Kabuli_Chr04*	45595233	(G/A)
CakSNP7472	*Kabuli*	*Ca_Kabuli_Chr04*	45612178	(T/G)
CakSNP7473	*Kabuli*	*Ca_Kabuli_Chr04*	45615465	(C/T)
CakSNP7474	*Kabuli*	*Ca_Kabuli_Chr04*	45615457	(C/T)
CakSNP7475	*Kabuli*	*Ca_Kabuli_Chr04*	45615452	(A/C)
CakSNP7476	*Kabuli*	*Ca_Kabuli_Chr04*	45615401	(T/G)
CakSNP7477	*Kabuli*	*Ca_Kabuli_Chr04*	45646003	(A/G)
CakSNP7478	*Kabuli*	*Ca_Kabuli_Chr04*	45645996	(A/G)
CakSNP7479	*Kabuli*	*Ca_Kabuli_Chr04*	45645957	(A/C)
CakSNP7480	*Kabuli*	*Ca_Kabuli_Chr04*	45708895	(C/A)
CakSNP7481	*Kabuli*	*Ca_Kabuli_Chr04*	45735754	(G/C)
CakSNP7482	*Kabuli*	*Ca_Kabuli_Chr04*	45735819	(T/C)
CakSNP7483	*Kabuli*	*Ca_Kabuli_Chr04*	45751623	(G/A)
CakSNP7484	*Kabuli*	*Ca_Kabuli_Chr04*	45751618	(C/T)
CakSNP7485	*Kabuli*	*Ca_Kabuli_Chr04*	45752844	(G/A)
CakSNP7486	*Kabuli*	*Ca_Kabuli_Chr04*	45752869	(G/T)
CakSNP7487	*Kabuli*	*Ca_Kabuli_Chr04*	45752874	(A/T)
CakSNP7488	*Kabuli*	*Ca_Kabuli_Chr04*	45782453	(A/T)
CakSNP7489	*Kabuli*	*Ca_Kabuli_Chr04*	45870087	(T/C)
CakSNP7490	*Kabuli*	*Ca_Kabuli_Chr04*	45870042	(C/T)
CakSNP7491	*Kabuli*	*Ca_Kabuli_Chr04*	45918351	(A/C)
CakSNP7492	*Kabuli*	*Ca_Kabuli_Chr04*	45935594	(A/C)
CakSNP7493	*Kabuli*	*Ca_Kabuli_Chr04*	45942252	(G/A)
CakSNP7494	*Kabuli*	*Ca_Kabuli_Chr04*	45942341	(T/C)
CakSNP7495	*Kabuli*	*Ca_Kabuli_Chr04*	45942729	(G/A)
CakSNP7496	*Kabuli*	*Ca_Kabuli_Chr04*	46134302	(C/G)
CakSNP7497	*Kabuli*	*Ca_Kabuli_Chr04*	46140729	(A/T)
CakSNP7498	*Kabuli*	*Ca_Kabuli_Chr04*	46148985	(A/T)
CakSNP7499	*Kabuli*	*Ca_Kabuli_Chr04*	46149013	(A/G)
CakSNP7500	*Kabuli*	*Ca_Kabuli_Chr04*	46150670	(T/C)
CakSNP7501	*Kabuli*	*Ca_Kabuli_Chr04*	46202862	(A/G)
CakSNP7502	*Kabuli*	*Ca_Kabuli_Chr04*	46344790	(G/A)

**SNP IDs**	**Cultivars**	**Chromosomes/scaffolds**	**Physical positions (bp)**	**SNPs**
CakSNP7503	*Kabuli*	*Ca_Kabuli_Chr04*	46344826	(A/G)
CakSNP7504	*Kabuli*	*Ca_Kabuli_Chr04*	46345042	(A/G)
CakSNP7505	*Kabuli*	*Ca_Kabuli_Chr04*	46402035	(G/A)
CakSNP7506	*Kabuli*	*Ca_Kabuli_Chr04*	46430326	(C/T)
CakSNP7507	*Kabuli*	*Ca_Kabuli_Chr04*	46430311	(C/A)
CakSNP7508	*Kabuli*	*Ca_Kabuli_Chr04*	46430288	(G/C)
CakSNP7509	*Kabuli*	*Ca_Kabuli_Chr04*	46605167	(A/G)
CakSNP7510	*Kabuli*	*Ca_Kabuli_Chr04*	46605131	(T/A)
CakSNP7511	*Kabuli*	*Ca_Kabuli_Chr04*	46612175	(G/A)
CakSNP7512	*Kabuli*	*Ca_Kabuli_Chr04*	46612197	(G/A)
CakSNP7513	*Kabuli*	*Ca_Kabuli_Chr04*	46612244	(C/T)
CakSNP7514	*Kabuli*	*Ca_Kabuli_Chr04*	46613068	(T/G)
CakSNP7515	*Kabuli*	*Ca_Kabuli_Chr04*	46694919	(T/A)
CakSNP7516	*Kabuli*	*Ca_Kabuli_Chr04*	46695043	(A/C)
CakSNP7517	*Kabuli*	*Ca_Kabuli_Chr04*	46695008	(T/C)
CakSNP7518	*Kabuli*	*Ca_Kabuli_Chr04*	46705181	(G/T)
CakSNP7519	*Kabuli*	*Ca_Kabuli_Chr04*	46705178	(A/T)
CakSNP7520	*Kabuli*	*Ca_Kabuli_Chr04*	46705225	(A/C)
CakSNP7521	*Kabuli*	*Ca_Kabuli_Chr04*	46713972	(C/T)
CakSNP7522	*Kabuli*	*Ca_Kabuli_Chr04*	46715350	(T/C)
CakSNP7523	*Kabuli*	*Ca_Kabuli_Chr04*	46715518	(G/C)
CakSNP7524	*Kabuli*	*Ca_Kabuli_Chr04*	46717155	(T/C)
CakSNP7525	*Kabuli*	*Ca_Kabuli_Chr04*	46717153	(C/T)
CakSNP7526	*Kabuli*	*Ca_Kabuli_Chr04*	46717200	(C/T)
CakSNP7527	*Kabuli*	*Ca_Kabuli_Chr04*	46717508	(G/A)
CakSNP7528	*Kabuli*	*Ca_Kabuli_Chr04*	46763540	(C/T)
CakSNP7529	*Kabuli*	*Ca_Kabuli_Chr04*	46763555	(C/T)
CakSNP7530	*Kabuli*	*Ca_Kabuli_Chr04*	46763868	(T/C)
CakSNP7531	*Kabuli*	*Ca_Kabuli_Chr04*	46763841	(A/C)
CakSNP7532	*Kabuli*	*Ca_Kabuli_Chr04*	47223016	(T/C)
CakSNP7533	*Kabuli*	*Ca_Kabuli_Chr04*	47223047	(A/G)
CakSNP7534	*Kabuli*	*Ca_Kabuli_Chr04*	47223635	(G/A)
CakSNP7535	*Kabuli*	*Ca_Kabuli_Chr04*	47223668	(T/G)
CakSNP7536	*Kabuli*	*Ca_Kabuli_Chr04*	47296400	(C/T)
CakSNP7537	*Kabuli*	*Ca_Kabuli_Chr04*	47379992	(C/T)
CakSNP7538	*Kabuli*	*Ca_Kabuli_Chr04*	47405685	(T/A)
CakSNP7539	*Kabuli*	*Ca_Kabuli_Chr04*	47419514	(A/G)
CakSNP7540	*Kabuli*	*Ca_Kabuli_Chr04*	47419517	(C/T)
CakSNP7541	*Kabuli*	*Ca_Kabuli_Chr04*	47428580	(C/T)
CakSNP7542	*Kabuli*	*Ca_Kabuli_Chr04*	47428599	(C/T)
CakSNP7543	*Kabuli*	*Ca_Kabuli_Chr04*	47428631	(A/G)

SNP IDs	Cultivars	Chromosomes/scaffolds	Physical positions (bp)	SNPs
CakSNP7544	*Kabuli*	*Ca_Kabuli_Chr04*	47428733	(A/G)
CakSNP7545	*Kabuli*	*Ca_Kabuli_Chr04*	47440207	(G/A)
CakSNP7546	*Kabuli*	*Ca_Kabuli_Chr04*	47450409	(C/T)
CakSNP7547	*Kabuli*	*Ca_Kabuli_Chr04*	47450479	(C/G)
CakSNP7548	*Kabuli*	*Ca_Kabuli_Chr04*	47562991	(A/T)
CakSNP7549	*Kabuli*	*Ca_Kabuli_Chr04*	47572228	(G/A)
CakSNP7550	*Kabuli*	*Ca_Kabuli_Chr04*	47572276	(C/T)
CakSNP7551	*Kabuli*	*Ca_Kabuli_Chr04*	47578508	(C/T)
CakSNP7552	*Kabuli*	*Ca_Kabuli_Chr04*	47578556	(T/G)
CakSNP7553	*Kabuli*	*Ca_Kabuli_Chr04*	47578655	(C/T)
CakSNP7554	*Kabuli*	*Ca_Kabuli_Chr04*	47578596	(C/T)
CakSNP7555	*Kabuli*	*Ca_Kabuli_Chr04*	47588760	(G/A)
CakSNP7556	*Kabuli*	*Ca_Kabuli_Chr04*	47612395	(C/A)
CakSNP7557	*Kabuli*	*Ca_Kabuli_Chr04*	47612396	(G/T)
CakSNP7558	*Kabuli*	*Ca_Kabuli_Chr04*	47615800	(G/C)
CakSNP7559	*Kabuli*	*Ca_Kabuli_Chr04*	47616120	(C/T)
CakSNP7560	*Kabuli*	*Ca_Kabuli_Chr04*	47616216	(A/G)
CakSNP7561	*Kabuli*	*Ca_Kabuli_Chr04*	47622112	(G/T)
CakSNP7562	*Kabuli*	*Ca_Kabuli_Chr04*	47675446	(A/G)
CakSNP7563	*Kabuli*	*Ca_Kabuli_Chr04*	47675758	(A/T)
CakSNP7564	*Kabuli*	*Ca_Kabuli_Chr04*	47675860	(G/A)
CakSNP7565	*Kabuli*	*Ca_Kabuli_Chr04*	47697239	(C/T)
CakSNP7566	*Kabuli*	*Ca_Kabuli_Chr04*	47798580	(A/G)
CakSNP7567	*Kabuli*	*Ca_Kabuli_Chr04*	47798622	(G/A)
CakSNP7568	*Kabuli*	*Ca_Kabuli_Chr04*	47819943	(T/C)
CakSNP7569	*Kabuli*	*Ca_Kabuli_Chr04*	48008987	(T/C)
CakSNP7570	*Kabuli*	*Ca_Kabuli_Chr04*	48009201	(C/T)
CakSNP7571	*Kabuli*	*Ca_Kabuli_Chr04*	48011446	(A/G)
CakSNP7572	*Kabuli*	*Ca_Kabuli_Chr04*	48098570	(G/A)
CakSNP7573	*Kabuli*	*Ca_Kabuli_Chr04*	48098562	(G/A)
CakSNP7574	*Kabuli*	*Ca_Kabuli_Chr04*	48103202	(G/T)
CakSNP7575	*Kabuli*	*Ca_Kabuli_Chr04*	48103196	(G/A)
CakSNP7576	*Kabuli*	*Ca_Kabuli_Chr04*	48103159	(G/A)
CakSNP7577	*Kabuli*	*Ca_Kabuli_Chr04*	48103153	(A/C)
CakSNP7578	*Kabuli*	*Ca_Kabuli_Chr04*	48150277	(C/T)
CakSNP7579	*Kabuli*	*Ca_Kabuli_Chr04*	48150481	(T/C)
CakSNP7580	*Kabuli*	*Ca_Kabuli_Chr04*	48150480	(C/A)
CakSNP7581	*Kabuli*	*Ca_Kabuli_Chr04*	48150553	(A/G)
CakSNP7582	*Kabuli*	*Ca_Kabuli_Chr04*	48150641	(G/T)
CakSNP7583	*Kabuli*	*Ca_Kabuli_Chr04*	48238919	(C/T)
CakSNP7584	*Kabuli*	*Ca_Kabuli_Chr04*	48239048	(A/G)

**SNP IDs**	**Cultivars**	**Chromosomes/scaffolds**	**Physical positions (bp)**	**SNPs**
CakSNP7585	*Kabuli*	*Ca_Kabuli_Chr04*	48263603	(G/A)
CakSNP7586	*Kabuli*	*Ca_Kabuli_Chr04*	48269138	(G/C)
CakSNP7587	*Kabuli*	*Ca_Kabuli_Chr04*	48275016	(C/T)
CakSNP7588	*Kabuli*	*Ca_Kabuli_Chr04*	48322502	(C/T)
CakSNP7589	*Kabuli*	*Ca_Kabuli_Chr04*	48322507	(C/T)
CakSNP7590	*Kabuli*	*Ca_Kabuli_Chr04*	48322633	(T/C)
CakSNP7591	*Kabuli*	*Ca_Kabuli_Chr04*	48331069	(C/A)
CakSNP7592	*Kabuli*	*Ca_Kabuli_Chr04*	48345604	(A/G)
CakSNP7593	*Kabuli*	*Ca_Kabuli_Chr04*	48345608	(G/T)
CakSNP7594	*Kabuli*	*Ca_Kabuli_Chr04*	48345655	(A/C)
CakSNP7595	*Kabuli*	*Ca_Kabuli_Chr04*	48345697	(C/G)
CakSNP7596	*Kabuli*	*Ca_Kabuli_Chr04*	48355358	(A/T)
CakSNP7597	*Kabuli*	*Ca_Kabuli_Chr04*	48355468	(G/T)
CakSNP7598	*Kabuli*	*Ca_Kabuli_Chr04*	48364170	(G/A)
CakSNP7599	*Kabuli*	*Ca_Kabuli_Chr04*	48365208	(C/A)
CakSNP7600	*Kabuli*	*Ca_Kabuli_Chr04*	48365388	(G/A)
CakSNP7601	*Kabuli*	*Ca_Kabuli_Chr04*	48382666	(G/C)
CakSNP7602	*Kabuli*	*Ca_Kabuli_Chr04*	48464493	(T/C)
CakSNP7603	*Kabuli*	*Ca_Kabuli_Chr04*	48464434	(G/C)
CakSNP7604	*Kabuli*	*Ca_Kabuli_Chr04*	48470477	(A/G)
CakSNP7605	*Kabuli*	*Ca_Kabuli_Chr04*	48475447	(T/C)
CakSNP7606	*Kabuli*	*Ca_Kabuli_Chr04*	48475461	(G/A)
CakSNP7607	*Kabuli*	*Ca_Kabuli_Chr04*	48478018	(A/G)
CakSNP7608	*Kabuli*	*Ca_Kabuli_Chr04*	48478058	(T/C)
CakSNP7609	*Kabuli*	*Ca_Kabuli_Chr04*	48478303	(G/A)
CakSNP7610	*Kabuli*	*Ca_Kabuli_Chr04*	48478524	(C/T)
CakSNP7611	*Kabuli*	*Ca_Kabuli_Chr04*	48484453	(A/T)
CakSNP7612	*Kabuli*	*Ca_Kabuli_Chr04*	48497731	(A/C)
CakSNP7613	*Kabuli*	*Ca_Kabuli_Chr04*	48497725	(A/C)
CakSNP7614	*Kabuli*	*Ca_Kabuli_Chr04*	48497756	(A/G)
CakSNP7615	*Kabuli*	*Ca_Kabuli_Chr04*	48497765	(G/A)
CakSNP7616	*Kabuli*	*Ca_Kabuli_Chr04*	48497839	(G/A)
CakSNP7617	*Kabuli*	*Ca_Kabuli_Chr04*	48498133	(C/T)
CakSNP7618	*Kabuli*	*Ca_Kabuli_Chr04*	48498181	(G/C)
CakSNP7619	*Kabuli*	*Ca_Kabuli_Chr04*	48498166	(A/G)
CakSNP7620	*Kabuli*	*Ca_Kabuli_Chr04*	48498368	(C/T)
CakSNP7621	*Kabuli*	*Ca_Kabuli_Chr04*	48498334	(T/G)
CakSNP7622	*Kabuli*	*Ca_Kabuli_Chr04*	48498331	(T/A)
CakSNP7623	*Kabuli*	*Ca_Kabuli_Chr04*	48498312	(T/C)
CakSNP7624	*Kabuli*	*Ca_Kabuli_Chr04*	48501567	(G/T)
CakSNP7625	*Kabuli*	*Ca_Kabuli_Chr04*	48501618	(C/A)

SNP IDs	Cultivars	Chromosomes/scaffolds	Physical positions (bp)	SNPs
CakSNP7626	*Kabuli*	*Ca_Kabuli_Chr04*	48501662	(A/T)
CakSNP7627	*Kabuli*	*Ca_Kabuli_Chr04*	48502554	(G/T)
CakSNP7628	*Kabuli*	*Ca_Kabuli_Chr04*	48502718	(G/C)
CakSNP7629	*Kabuli*	*Ca_Kabuli_Chr04*	48502714	(T/C)
CakSNP7630	*Kabuli*	*Ca_Kabuli_Chr04*	48502687	(G/A)
CakSNP7631	*Kabuli*	*Ca_Kabuli_Chr04*	48591482	(T/G)
CakSNP7632	*Kabuli*	*Ca_Kabuli_Chr04*	48591539	(T/C)
CakSNP7633	*Kabuli*	*Ca_Kabuli_Chr04*	48591578	(T/C)
CakSNP7634	*Kabuli*	*Ca_Kabuli_Chr04*	48591625	(G/T)
CakSNP7635	*Kabuli*	*Ca_Kabuli_Chr04*	48678278	(T/C)
CakSNP7636	*Kabuli*	*Ca_Kabuli_Chr04*	48678692	(T/C)
CakSNP7637	*Kabuli*	*Ca_Kabuli_Chr04*	48688766	(A/G)
CakSNP7638	*Kabuli*	*Ca_Kabuli_Chr04*	48705815	(C/T)
CakSNP7639	*Kabuli*	*Ca_Kabuli_Chr04*	48705856	(A/T)
CakSNP7640	*Kabuli*	*Ca_Kabuli_Chr04*	48705957	(C/T)
CakSNP7641	*Kabuli*	*Ca_Kabuli_Chr04*	48714912	(G/A)
CakSNP7642	*Kabuli*	*Ca_Kabuli_Chr04*	48715028	(A/G)
CakSNP7643	*Kabuli*	*Ca_Kabuli_Chr04*	48720330	(C/T)
CakSNP7644	*Kabuli*	*Ca_Kabuli_Chr04*	48720266	(C/T)
CakSNP7645	*Kabuli*	*Ca_Kabuli_Chr04*	48784151	(T/G)
CakSNP7646	*Kabuli*	*Ca_Kabuli_Chr04*	48784176	(T/C)
CakSNP7647	*Kabuli*	*Ca_Kabuli_Chr04*	48784178	(C/G)
CakSNP7648	*Kabuli*	*Ca_Kabuli_Chr04*	48784179	(G/A)
CakSNP7649	*Kabuli*	*Ca_Kabuli_Chr04*	48784189	(A/T)
CakSNP7650	*Kabuli*	*Ca_Kabuli_Chr04*	48784202	(A/C)
CakSNP7651	*Kabuli*	*Ca_Kabuli_Chr04*	48784204	(G/C)
CakSNP7652	*Kabuli*	*Ca_Kabuli_Chr04*	48931112	(T/G)
CakSNP7653	*Kabuli*	*Ca_Kabuli_Chr04*	48931109	(A/G)
CakSNP7654	*Kabuli*	*Ca_Kabuli_Chr04*	48936643	(G/A)
CakSNP7655	*Kabuli*	*Ca_Kabuli_Chr04*	48936669	(G/A)
CakSNP7656	*Kabuli*	*Ca_Kabuli_Chr04*	48936710	(A/G)
CakSNP7657	*Kabuli*	*Ca_Kabuli_Chr04*	48936819	(C/T)
CakSNP7658	*Kabuli*	*Ca_Kabuli_Chr04*	48964200	(T/C)
CakSNP7659	*Kabuli*	*Ca_Kabuli_Chr04*	48964260	(G/C)
CakSNP7660	*Kabuli*	*Ca_Kabuli_Chr04*	48964248	(C/T)
CakSNP7661	*Kabuli*	*Ca_Kabuli_Chr04*	49012733	(T/A)
CakSNP7662	*Kabuli*	*Ca_Kabuli_Chr04*	49012756	(C/T)
CakSNP7663	*Kabuli*	*Ca_Kabuli_Chr05*	95087	(G/A)
CakSNP7664	*Kabuli*	*Ca_Kabuli_Chr05*	95253	(A/G)
CakSNP7665	*Kabuli*	*Ca_Kabuli_Chr05*	131558	(A/C)
CakSNP7666	*Kabuli*	*Ca_Kabuli_Chr05*	154084	(T/G)

SNP IDs	Cultivars	Chromosomes/scaffolds	Physical positions (bp)	SNPs
CakSNP7667	*Kabuli*	*Ca_Kabuli_Chr05*	154095	(C/G)
CakSNP7668	*Kabuli*	*Ca_Kabuli_Chr05*	154097	(A/T)
CakSNP7669	*Kabuli*	*Ca_Kabuli_Chr05*	154162	(C/T)
CakSNP7670	*Kabuli*	*Ca_Kabuli_Chr05*	154128	(G/T)
CakSNP7671	*Kabuli*	*Ca_Kabuli_Chr05*	166222	(T/G)
CakSNP7672	*Kabuli*	*Ca_Kabuli_Chr05*	166217	(G/C)
CakSNP7673	*Kabuli*	*Ca_Kabuli_Chr05*	166216	(T/G)
CakSNP7674	*Kabuli*	*Ca_Kabuli_Chr05*	166341	(C/A)
CakSNP7675	*Kabuli*	*Ca_Kabuli_Chr05*	166381	(A/G)
CakSNP7676	*Kabuli*	*Ca_Kabuli_Chr05*	210625	(T/C)
CakSNP7677	*Kabuli*	*Ca_Kabuli_Chr05*	210649	(C/A)
CakSNP7678	*Kabuli*	*Ca_Kabuli_Chr05*	222651	(A/C)
CakSNP7679	*Kabuli*	*Ca_Kabuli_Chr05*	222630	(A/G)
CakSNP7680	*Kabuli*	*Ca_Kabuli_Chr05*	222641	(C/T)
CakSNP7681	*Kabuli*	*Ca_Kabuli_Chr05*	222653	(A/C)
CakSNP7682	*Kabuli*	*Ca_Kabuli_Chr05*	222657	(G/A)
CakSNP7683	*Kabuli*	*Ca_Kabuli_Chr05*	223384	(C/G)
CakSNP7684	*Kabuli*	*Ca_Kabuli_Chr05*	234247	(T/C)
CakSNP7685	*Kabuli*	*Ca_Kabuli_Chr05*	234319	(T/A)
CakSNP7686	*Kabuli*	*Ca_Kabuli_Chr05*	234311	(T/A)
CakSNP7687	*Kabuli*	*Ca_Kabuli_Chr05*	234310	(G/A)
CakSNP7688	*Kabuli*	*Ca_Kabuli_Chr05*	234305	(T/A)
CakSNP7689	*Kabuli*	*Ca_Kabuli_Chr05*	234303	(C/A)
CakSNP7690	*Kabuli*	*Ca_Kabuli_Chr05*	234410	(A/C)
CakSNP7691	*Kabuli*	*Ca_Kabuli_Chr05*	245850	(T/G)
CakSNP7692	*Kabuli*	*Ca_Kabuli_Chr05*	245863	(A/T)
CakSNP7693	*Kabuli*	*Ca_Kabuli_Chr05*	245896	(A/T)
CakSNP7694	*Kabuli*	*Ca_Kabuli_Chr05*	246466	(A/T)
CakSNP7695	*Kabuli*	*Ca_Kabuli_Chr05*	246654	(A/G)
CakSNP7696	*Kabuli*	*Ca_Kabuli_Chr05*	256745	(G/A)
CakSNP7697	*Kabuli*	*Ca_Kabuli_Chr05*	272972	(T/A)
CakSNP7698	*Kabuli*	*Ca_Kabuli_Chr05*	277040	(G/A)
CakSNP7699	*Kabuli*	*Ca_Kabuli_Chr05*	277316	(T/A)
CakSNP7700	*Kabuli*	*Ca_Kabuli_Chr05*	343517	(C/T)
CakSNP7701	*Kabuli*	*Ca_Kabuli_Chr05*	353792	(T/A)
CakSNP7702	*Kabuli*	*Ca_Kabuli_Chr05*	438448	(C/A)
CakSNP7703	*Kabuli*	*Ca_Kabuli_Chr05*	438445	(C/A)
CakSNP7704	*Kabuli*	*Ca_Kabuli_Chr05*	438442	(T/C)
CakSNP7705	*Kabuli*	*Ca_Kabuli_Chr05*	443309	(A/T)
CakSNP7706	*Kabuli*	*Ca_Kabuli_Chr05*	469397	(G/A)
CakSNP7707	*Kabuli*	*Ca_Kabuli_Chr05*	805859	(C/T)

SNP IDs	Cultivars	Chromosomes/scaffolds	Physical positions (bp)	SNPs
CakSNP7708	*Kabuli*	*Ca_Kabuli_Chr05*	805882	(A/T)
CakSNP7709	*Kabuli*	*Ca_Kabuli_Chr05*	805883	(G/T)
CakSNP7710	*Kabuli*	*Ca_Kabuli_Chr05*	805910	(G/A)
CakSNP7711	*Kabuli*	*Ca_Kabuli_Chr05*	870867	(A/G)
CakSNP7712	*Kabuli*	*Ca_Kabuli_Chr05*	870909	(G/A)
CakSNP7713	*Kabuli*	*Ca_Kabuli_Chr05*	925554	(T/C)
CakSNP7714	*Kabuli*	*Ca_Kabuli_Chr05*	925493	(T/C)
CakSNP7715	*Kabuli*	*Ca_Kabuli_Chr05*	1141755	(C/T)
CakSNP7716	*Kabuli*	*Ca_Kabuli_Chr05*	1220867	(A/G)
CakSNP7717	*Kabuli*	*Ca_Kabuli_Chr05*	1252394	(G/A)
CakSNP7718	*Kabuli*	*Ca_Kabuli_Chr05*	1252388	(A/T)
CakSNP7719	*Kabuli*	*Ca_Kabuli_Chr05*	1270830	(T/C)
CakSNP7720	*Kabuli*	*Ca_Kabuli_Chr05*	1272581	(T/C)
CakSNP7721	*Kabuli*	*Ca_Kabuli_Chr05*	1280977	(T/G)
CakSNP7722	*Kabuli*	*Ca_Kabuli_Chr05*	1281006	(A/C)
CakSNP7723	*Kabuli*	*Ca_Kabuli_Chr05*	1281017	(G/A)
CakSNP7724	*Kabuli*	*Ca_Kabuli_Chr05*	1281021	(C/T)
CakSNP7725	*Kabuli*	*Ca_Kabuli_Chr05*	1534904	(C/T)
CakSNP7726	*Kabuli*	*Ca_Kabuli_Chr05*	1534901	(C/T)
CakSNP7727	*Kabuli*	*Ca_Kabuli_Chr05*	1534888	(T/C)
CakSNP7728	*Kabuli*	*Ca_Kabuli_Chr05*	2166586	(A/G)
CakSNP7729	*Kabuli*	*Ca_Kabuli_Chr05*	2519977	(C/T)
CakSNP7730	*Kabuli*	*Ca_Kabuli_Chr05*	2519949	(C/T)
CakSNP7731	*Kabuli*	*Ca_Kabuli_Chr05*	2519961	(G/A)
CakSNP7732	*Kabuli*	*Ca_Kabuli_Chr05*	2519976	(G/A)
CakSNP7733	*Kabuli*	*Ca_Kabuli_Chr05*	2520035	(G/A)
CakSNP7734	*Kabuli*	*Ca_Kabuli_Chr05*	2520058	(C/T)
CakSNP7735	*Kabuli*	*Ca_Kabuli_Chr05*	2520015	(C/G)
CakSNP7736	*Kabuli*	*Ca_Kabuli_Chr05*	2520049	(C/T)
CakSNP7737	*Kabuli*	*Ca_Kabuli_Chr05*	2520050	(G/C)
CakSNP7738	*Kabuli*	*Ca_Kabuli_Chr05*	2520051	(G/T)
CakSNP7739	*Kabuli*	*Ca_Kabuli_Chr05*	2520055	(C/T)
CakSNP7740	*Kabuli*	*Ca_Kabuli_Chr05*	2520068	(T/A)
CakSNP7741	*Kabuli*	*Ca_Kabuli_Chr05*	2520069	(C/T)
CakSNP7742	*Kabuli*	*Ca_Kabuli_Chr05*	2520094	(G/A)
CakSNP7743	*Kabuli*	*Ca_Kabuli_Chr05*	2520061	(C/T)
CakSNP7744	*Kabuli*	*Ca_Kabuli_Chr05*	2520093	(G/A)
CakSNP7745	*Kabuli*	*Ca_Kabuli_Chr05*	2520123	(A/G)
CakSNP7746	*Kabuli*	*Ca_Kabuli_Chr05*	2520114	(G/A)
CakSNP7747	*Kabuli*	*Ca_Kabuli_Chr05*	2520109	(G/A)
CakSNP7748	*Kabuli*	*Ca_Kabuli_Chr05*	2520101	(C/T)

SNP IDs	Cultivars	Chromosomes/scaffolds	Physical positions (bp)	SNPs
CakSNP7749	*Kabuli*	*Ca_Kabuli_Chr05*	2613423	(T/G)
CakSNP7750	*Kabuli*	*Ca_Kabuli_Chr05*	2948530	(G/T)
CakSNP7751	*Kabuli*	*Ca_Kabuli_Chr05*	2948531	(T/C)
CakSNP7752	*Kabuli*	*Ca_Kabuli_Chr05*	2948549	(C/A)
CakSNP7753	*Kabuli*	*Ca_Kabuli_Chr05*	2948592	(T/C)
CakSNP7754	*Kabuli*	*Ca_Kabuli_Chr05*	2948596	(C/T)
CakSNP7755	*Kabuli*	*Ca_Kabuli_Chr05*	2948616	(G/A)
CakSNP7756	*Kabuli*	*Ca_Kabuli_Chr05*	2948698	(G/A)
CakSNP7757	*Kabuli*	*Ca_Kabuli_Chr05*	2948977	(G/A)
CakSNP7758	*Kabuli*	*Ca_Kabuli_Chr05*	3007213	(A/G)
CakSNP7759	*Kabuli*	*Ca_Kabuli_Chr05*	3007316	(T/C)
CakSNP7760	*Kabuli*	*Ca_Kabuli_Chr05*	3040264	(G/T)
CakSNP7761	*Kabuli*	*Ca_Kabuli_Chr05*	3040948	(G/C)
CakSNP7762	*Kabuli*	*Ca_Kabuli_Chr05*	3159061	(G/A)
CakSNP7763	*Kabuli*	*Ca_Kabuli_Chr05*	3348291	(G/A)
CakSNP7764	*Kabuli*	*Ca_Kabuli_Chr05*	3348310	(A/G)
CakSNP7765	*Kabuli*	*Ca_Kabuli_Chr05*	3348366	(G/C)
CakSNP7766	*Kabuli*	*Ca_Kabuli_Chr05*	3353052	(A/G)
CakSNP7767	*Kabuli*	*Ca_Kabuli_Chr05*	4122395	(T/C)
CakSNP7768	*Kabuli*	*Ca_Kabuli_Chr05*	4122342	(T/C)
CakSNP7769	*Kabuli*	*Ca_Kabuli_Chr05*	5133644	(A/G)
CakSNP7770	*Kabuli*	*Ca_Kabuli_Chr05*	5205261	(T/G)
CakSNP7771	*Kabuli*	*Ca_Kabuli_Chr05*	5404261	(G/A)
CakSNP7772	*Kabuli*	*Ca_Kabuli_Chr05*	6190494	(T/C)
CakSNP7773	*Kabuli*	*Ca_Kabuli_Chr05*	6225426	(T/A)
CakSNP7774	*Kabuli*	*Ca_Kabuli_Chr05*	6225415	(G/C)
CakSNP7775	*Kabuli*	*Ca_Kabuli_Chr05*	6225410	(T/A)
CakSNP7776	*Kabuli*	*Ca_Kabuli_Chr05*	6290453	(G/A)
CakSNP7777	*Kabuli*	*Ca_Kabuli_Chr05*	6300337	(C/A)
CakSNP7778	*Kabuli*	*Ca_Kabuli_Chr05*	6300360	(C/A)
CakSNP7779	*Kabuli*	*Ca_Kabuli_Chr05*	6300502	(C/A)
CakSNP7780	*Kabuli*	*Ca_Kabuli_Chr05*	6316787	(C/T)
CakSNP7781	*Kabuli*	*Ca_Kabuli_Chr05*	6317014	(C/T)
CakSNP7782	*Kabuli*	*Ca_Kabuli_Chr05*	6931865	(A/G)
CakSNP7783	*Kabuli*	*Ca_Kabuli_Chr05*	7082295	(G/A)
CakSNP7784	*Kabuli*	*Ca_Kabuli_Chr05*	7082296	(T/G)
CakSNP7785	*Kabuli*	*Ca_Kabuli_Chr05*	7082301	(G/T)
CakSNP7786	*Kabuli*	*Ca_Kabuli_Chr05*	7278953	(C/T)
CakSNP7787	*Kabuli*	*Ca_Kabuli_Chr05*	7398246	(A/C)
CakSNP7788	*Kabuli*	*Ca_Kabuli_Chr05*	7425116	(C/T)
CakSNP7789	*Kabuli*	*Ca_Kabuli_Chr05*	7617914	(T/A)

SNP IDs	Cultivars	Chromosomes/scaffolds	Physical positions (bp)	SNPs
CakSNP7790	*Kabuli*	*Ca_Kabuli_Chr05*	7628097	(C/A)
CakSNP7791	*Kabuli*	*Ca_Kabuli_Chr05*	7641993	(T/C)
CakSNP7792	*Kabuli*	*Ca_Kabuli_Chr05*	7641925	(G/T)
CakSNP7793	*Kabuli*	*Ca_Kabuli_Chr05*	7641923	(A/G)
CakSNP7794	*Kabuli*	*Ca_Kabuli_Chr05*	7664421	(T/C)
CakSNP7795	*Kabuli*	*Ca_Kabuli_Chr05*	7775286	(C/A)
CakSNP7796	*Kabuli*	*Ca_Kabuli_Chr05*	7775290	(A/T)
CakSNP7797	*Kabuli*	*Ca_Kabuli_Chr05*	7775298	(A/T)
CakSNP7798	*Kabuli*	*Ca_Kabuli_Chr05*	7775329	(A/G)
CakSNP7799	*Kabuli*	*Ca_Kabuli_Chr05*	7775516	(A/G)
CakSNP7800	*Kabuli*	*Ca_Kabuli_Chr05*	7775497	(G/C)
CakSNP7801	*Kabuli*	*Ca_Kabuli_Chr05*	7775472	(G/A)
CakSNP7802	*Kabuli*	*Ca_Kabuli_Chr05*	7775456	(T/C)
CakSNP7803	*Kabuli*	*Ca_Kabuli_Chr05*	7775918	(C/T)
CakSNP7804	*Kabuli*	*Ca_Kabuli_Chr05*	7789021	(G/T)
CakSNP7805	*Kabuli*	*Ca_Kabuli_Chr05*	7789065	(A/G)
CakSNP7806	*Kabuli*	*Ca_Kabuli_Chr05*	7826336	(G/T)
CakSNP7807	*Kabuli*	*Ca_Kabuli_Chr05*	7826338	(T/G)
CakSNP7808	*Kabuli*	*Ca_Kabuli_Chr05*	7826362	(C/T)
CakSNP7809	*Kabuli*	*Ca_Kabuli_Chr05*	7826366	(A/G)
CakSNP7810	*Kabuli*	*Ca_Kabuli_Chr05*	7925356	(A/C)
CakSNP7811	*Kabuli*	*Ca_Kabuli_Chr05*	8263483	(A/G)
CakSNP7812	*Kabuli*	*Ca_Kabuli_Chr05*	8653283	(G/A)
CakSNP7813	*Kabuli*	*Ca_Kabuli_Chr05*	8676750	(T/G)
CakSNP7814	*Kabuli*	*Ca_Kabuli_Chr05*	8676807	(G/A)
CakSNP7815	*Kabuli*	*Ca_Kabuli_Chr05*	8676789	(T/C)
CakSNP7816	*Kabuli*	*Ca_Kabuli_Chr05*	8676771	(G/A)
CakSNP7817	*Kabuli*	*Ca_Kabuli_Chr05*	8946218	(G/A)
CakSNP7818	*Kabuli*	*Ca_Kabuli_Chr05*	9034100	(C/G)
CakSNP7819	*Kabuli*	*Ca_Kabuli_Chr05*	9076709	(C/T)
CakSNP7820	*Kabuli*	*Ca_Kabuli_Chr05*	9076712	(G/A)
CakSNP7821	*Kabuli*	*Ca_Kabuli_Chr05*	9076737	(C/A)
CakSNP7822	*Kabuli*	*Ca_Kabuli_Chr05*	9076738	(G/C)
CakSNP7823	*Kabuli*	*Ca_Kabuli_Chr05*	9076767	(C/A)
CakSNP7824	*Kabuli*	*Ca_Kabuli_Chr05*	9076824	(G/A)
CakSNP7825	*Kabuli*	*Ca_Kabuli_Chr05*	9076821	(G/T)
CakSNP7826	*Kabuli*	*Ca_Kabuli_Chr05*	9076809	(T/A)
CakSNP7827	*Kabuli*	*Ca_Kabuli_Chr05*	9077489	(T/C)
CakSNP7828	*Kabuli*	*Ca_Kabuli_Chr05*	9519382	(C/A)
CakSNP7829	*Kabuli*	*Ca_Kabuli_Chr05*	9519390	(G/A)
CakSNP7830	*Kabuli*	*Ca_Kabuli_Chr05*	9609055	(T/C)

SNP IDs	Cultivars	Chromosomes/scaffolds	Physical positions (bp)	SNPs
CakSNP7831	*Kabuli*	*Ca_Kabuli_Chr05*	9620998	(A/C)
CakSNP7832	*Kabuli*	*Ca_Kabuli_Chr05*	9728498	(A/T)
CakSNP7833	*Kabuli*	*Ca_Kabuli_Chr05*	9728609	(T/A)
CakSNP7834	*Kabuli*	*Ca_Kabuli_Chr05*	9728591	(A/G)
CakSNP7835	*Kabuli*	*Ca_Kabuli_Chr05*	9786213	(C/A)
CakSNP7836	*Kabuli*	*Ca_Kabuli_Chr05*	9868796	(T/C)
CakSNP7837	*Kabuli*	*Ca_Kabuli_Chr05*	9869005	(T/G)
CakSNP7838	*Kabuli*	*Ca_Kabuli_Chr05*	9961273	(T/C)
CakSNP7839	*Kabuli*	*Ca_Kabuli_Chr05*	9961396	(T/C)
CakSNP7840	*Kabuli*	*Ca_Kabuli_Chr05*	9961380	(C/T)
CakSNP7841	*Kabuli*	*Ca_Kabuli_Chr05*	9965880	(G/C)
CakSNP7842	*Kabuli*	*Ca_Kabuli_Chr05*	9965890	(T/C)
CakSNP7843	*Kabuli*	*Ca_Kabuli_Chr05*	9965913	(A/C)
CakSNP7844	*Kabuli*	*Ca_Kabuli_Chr05*	9965848	(T/C)
CakSNP7845	*Kabuli*	*Ca_Kabuli_Chr05*	9965846	(A/C)
CakSNP7846	*Kabuli*	*Ca_Kabuli_Chr05*	10004386	(G/T)
CakSNP7847	*Kabuli*	*Ca_Kabuli_Chr05*	10004427	(T/G)
CakSNP7848	*Kabuli*	*Ca_Kabuli_Chr05*	10004430	(T/G)
CakSNP7849	*Kabuli*	*Ca_Kabuli_Chr05*	10004451	(T/G)
CakSNP7850	*Kabuli*	*Ca_Kabuli_Chr05*	10054140	(G/C)
CakSNP7851	*Kabuli*	*Ca_Kabuli_Chr05*	10054146	(A/G)
CakSNP7852	*Kabuli*	*Ca_Kabuli_Chr05*	10054266	(T/C)
CakSNP7853	*Kabuli*	*Ca_Kabuli_Chr05*	10054213	(A/C)
CakSNP7854	*Kabuli*	*Ca_Kabuli_Chr05*	10072358	(G/A)
CakSNP7855	*Kabuli*	*Ca_Kabuli_Chr05*	10072403	(G/C)
CakSNP7856	*Kabuli*	*Ca_Kabuli_Chr05*	10154016	(A/G)
CakSNP7857	*Kabuli*	*Ca_Kabuli_Chr05*	10247223	(T/C)
CakSNP7858	*Kabuli*	*Ca_Kabuli_Chr05*	10247224	(T/A)
CakSNP7859	*Kabuli*	*Ca_Kabuli_Chr05*	10247233	(C/T)
CakSNP7860	*Kabuli*	*Ca_Kabuli_Chr05*	10247244	(C/T)
CakSNP7861	*Kabuli*	*Ca_Kabuli_Chr05*	10390882	(C/T)
CakSNP7862	*Kabuli*	*Ca_Kabuli_Chr05*	10406562	(C/A)
CakSNP7863	*Kabuli*	*Ca_Kabuli_Chr05*	10406602	(C/T)
CakSNP7864	*Kabuli*	*Ca_Kabuli_Chr05*	10406613	(C/T)
CakSNP7865	*Kabuli*	*Ca_Kabuli_Chr05*	10406636	(A/G)
CakSNP7866	*Kabuli*	*Ca_Kabuli_Chr05*	10406649	(A/C)
CakSNP7867	*Kabuli*	*Ca_Kabuli_Chr05*	10406650	(A/G)
CakSNP7868	*Kabuli*	*Ca_Kabuli_Chr05*	10406754	(G/T)
CakSNP7869	*Kabuli*	*Ca_Kabuli_Chr05*	10510166	(G/A)
CakSNP7870	*Kabuli*	*Ca_Kabuli_Chr05*	10544823	(A/G)
CakSNP7871	*Kabuli*	*Ca_Kabuli_Chr05*	10546944	(A/T)

SNP IDs	Cultivars	Chromosomes/scaffolds	Physical positions (bp)	SNPs
CakSNP7872	*Kabuli*	*Ca_Kabuli_Chr05*	10675252	(A/G)
CakSNP7873	*Kabuli*	*Ca_Kabuli_Chr05*	10675279	(C/G)
CakSNP7874	*Kabuli*	*Ca_Kabuli_Chr05*	10675294	(A/G)
CakSNP7875	*Kabuli*	*Ca_Kabuli_Chr05*	10769765	(C/T)
CakSNP7876	*Kabuli*	*Ca_Kabuli_Chr05*	10832275	(T/C)
CakSNP7877	*Kabuli*	*Ca_Kabuli_Chr05*	10858490	(C/T)
CakSNP7878	*Kabuli*	*Ca_Kabuli_Chr05*	10918368	(A/G)
CakSNP7879	*Kabuli*	*Ca_Kabuli_Chr05*	10919246	(C/G)
CakSNP7880	*Kabuli*	*Ca_Kabuli_Chr05*	11230617	(C/G)
CakSNP7881	*Kabuli*	*Ca_Kabuli_Chr05*	11230627	(A/G)
CakSNP7882	*Kabuli*	*Ca_Kabuli_Chr05*	11230648	(G/A)
CakSNP7883	*Kabuli*	*Ca_Kabuli_Chr05*	11230671	(T/C)
CakSNP7884	*Kabuli*	*Ca_Kabuli_Chr05*	11263571	(C/G)
CakSNP7885	*Kabuli*	*Ca_Kabuli_Chr05*	11263574	(C/A)
CakSNP7886	*Kabuli*	*Ca_Kabuli_Chr05*	11317487	(C/T)
CakSNP7887	*Kabuli*	*Ca_Kabuli_Chr05*	11317502	(C/A)
CakSNP7888	*Kabuli*	*Ca_Kabuli_Chr05*	11317534	(C/A)
CakSNP7889	*Kabuli*	*Ca_Kabuli_Chr05*	11385780	(A/C)
CakSNP7890	*Kabuli*	*Ca_Kabuli_Chr05*	11385874	(A/G)
CakSNP7891	*Kabuli*	*Ca_Kabuli_Chr05*	11765841	(C/T)
CakSNP7892	*Kabuli*	*Ca_Kabuli_Chr05*	11777112	(C/T)
CakSNP7893	*Kabuli*	*Ca_Kabuli_Chr05*	11807822	(G/T)
CakSNP7894	*Kabuli*	*Ca_Kabuli_Chr05*	11807842	(T/C)
CakSNP7895	*Kabuli*	*Ca_Kabuli_Chr05*	11845515	(C/T)
CakSNP7896	*Kabuli*	*Ca_Kabuli_Chr05*	11853090	(C/T)
CakSNP7897	*Kabuli*	*Ca_Kabuli_Chr05*	11864959	(T/C)
CakSNP7898	*Kabuli*	*Ca_Kabuli_Chr05*	12044714	(A/G)
CakSNP7899	*Kabuli*	*Ca_Kabuli_Chr05*	12157678	(A/G)
CakSNP7900	*Kabuli*	*Ca_Kabuli_Chr05*	12157683	(T/C)
CakSNP7901	*Kabuli*	*Ca_Kabuli_Chr05*	12157688	(C/T)
CakSNP7902	*Kabuli*	*Ca_Kabuli_Chr05*	12157706	(G/A)
CakSNP7903	*Kabuli*	*Ca_Kabuli_Chr05*	12157707	(C/T)
CakSNP7904	*Kabuli*	*Ca_Kabuli_Chr05*	12157710	(A/C)
CakSNP7905	*Kabuli*	*Ca_Kabuli_Chr05*	12157742	(C/A)
CakSNP7906	*Kabuli*	*Ca_Kabuli_Chr05*	12157699	(G/T)
CakSNP7907	*Kabuli*	*Ca_Kabuli_Chr05*	12162108	(T/G)
CakSNP7908	*Kabuli*	*Ca_Kabuli_Chr05*	12162156	(T/C)
CakSNP7909	*Kabuli*	*Ca_Kabuli_Chr05*	12162160	(G/T)
CakSNP7910	*Kabuli*	*Ca_Kabuli_Chr05*	12162164	(C/G)
CakSNP7911	*Kabuli*	*Ca_Kabuli_Chr05*	12198014	(G/A)
CakSNP7912	*Kabuli*	*Ca_Kabuli_Chr05*	12204521	(T/A)

**SNP IDs**	**Cultivars**	**Chromosomes/scaffolds**	**Physical positions (bp)**	**SNPs**
CakSNP7913	*Kabuli*	*Ca_Kabuli_Chr05*	12204588	(A/G)
CakSNP7914	*Kabuli*	*Ca_Kabuli_Chr05*	12427931	(C/T)
CakSNP7915	*Kabuli*	*Ca_Kabuli_Chr05*	12427940	(T/A)
CakSNP7916	*Kabuli*	*Ca_Kabuli_Chr05*	12427970	(T/C)
CakSNP7917	*Kabuli*	*Ca_Kabuli_Chr05*	12427969	(T/C)
CakSNP7918	*Kabuli*	*Ca_Kabuli_Chr05*	12427966	(T/C)
CakSNP7919	*Kabuli*	*Ca_Kabuli_Chr05*	12428027	(G/A)
CakSNP7920	*Kabuli*	*Ca_Kabuli_Chr05*	12428121	(A/T)
CakSNP7921	*Kabuli*	*Ca_Kabuli_Chr05*	12428120	(G/A)
CakSNP7922	*Kabuli*	*Ca_Kabuli_Chr05*	12428102	(A/G)
CakSNP7923	*Kabuli*	*Ca_Kabuli_Chr05*	12480948	(T/G)
CakSNP7924	*Kabuli*	*Ca_Kabuli_Chr05*	12553469	(A/C)
CakSNP7925	*Kabuli*	*Ca_Kabuli_Chr05*	12553470	(G/C)
CakSNP7926	*Kabuli*	*Ca_Kabuli_Chr05*	12590330	(A/G)
CakSNP7927	*Kabuli*	*Ca_Kabuli_Chr05*	12590361	(T/C)
CakSNP7928	*Kabuli*	*Ca_Kabuli_Chr05*	12590478	(T/C)
CakSNP7929	*Kabuli*	*Ca_Kabuli_Chr05*	12662138	(G/T)
CakSNP7930	*Kabuli*	*Ca_Kabuli_Chr05*	13982000	(T/G)
CakSNP7931	*Kabuli*	*Ca_Kabuli_Chr05*	13982041	(C/T)
CakSNP7932	*Kabuli*	*Ca_Kabuli_Chr05*	15401230	(C/T)
CakSNP7933	*Kabuli*	*Ca_Kabuli_Chr05*	15500782	(G/C)
CakSNP7934	*Kabuli*	*Ca_Kabuli_Chr05*	15548719	(G/A)
CakSNP7935	*Kabuli*	*Ca_Kabuli_Chr05*	15952395	(C/T)
CakSNP7936	*Kabuli*	*Ca_Kabuli_Chr05*	16227319	(G/T)
CakSNP7937	*Kabuli*	*Ca_Kabuli_Chr05*	16227321	(C/T)
CakSNP7938	*Kabuli*	*Ca_Kabuli_Chr05*	16227353	(G/A)
CakSNP7939	*Kabuli*	*Ca_Kabuli_Chr05*	16227395	(G/A)
CakSNP7940	*Kabuli*	*Ca_Kabuli_Chr05*	16227401	(G/A)
CakSNP7941	*Kabuli*	*Ca_Kabuli_Chr05*	16227408	(C/T)
CakSNP7942	*Kabuli*	*Ca_Kabuli_Chr05*	16426578	(G/A)
CakSNP7943	*Kabuli*	*Ca_Kabuli_Chr05*	16426575	(G/A)
CakSNP7944	*Kabuli*	*Ca_Kabuli_Chr05*	16426549	(G/A)
CakSNP7945	*Kabuli*	*Ca_Kabuli_Chr05*	16426662	(C/T)
CakSNP7946	*Kabuli*	*Ca_Kabuli_Chr05*	16426657	(C/T)
CakSNP7947	*Kabuli*	*Ca_Kabuli_Chr05*	16426656	(A/G)
CakSNP7948	*Kabuli*	*Ca_Kabuli_Chr05*	16426642	(A/G)
CakSNP7949	*Kabuli*	*Ca_Kabuli_Chr05*	16593880	(A/G)
CakSNP7950	*Kabuli*	*Ca_Kabuli_Chr05*	16751536	(C/T)
CakSNP7951	*Kabuli*	*Ca_Kabuli_Chr05*	16767134	(C/T)
CakSNP7952	*Kabuli*	*Ca_Kabuli_Chr05*	16821406	(A/G)
CakSNP7953	*Kabuli*	*Ca_Kabuli_Chr05*	16929903	(C/G)

SNP IDs	Cultivars	Chromosomes/scaffolds	Physical positions (bp)	SNPs
CakSNP7954	*Kabuli*	*Ca_Kabuli_Chr05*	16929897	(A/G)
CakSNP7955	*Kabuli*	*Ca_Kabuli_Chr05*	17037918	(G/T)
CakSNP7956	*Kabuli*	*Ca_Kabuli_Chr05*	17044340	(G/A)
CakSNP7957	*Kabuli*	*Ca_Kabuli_Chr05*	17044338	(G/A)
CakSNP7958	*Kabuli*	*Ca_Kabuli_Chr05*	17044332	(A/G)
CakSNP7959	*Kabuli*	*Ca_Kabuli_Chr05*	17045235	(T/C)
CakSNP7960	*Kabuli*	*Ca_Kabuli_Chr05*	17045244	(A/G)
CakSNP7961	*Kabuli*	*Ca_Kabuli_Chr05*	17045485	(T/G)
CakSNP7962	*Kabuli*	*Ca_Kabuli_Chr05*	17239612	(T/C)
CakSNP7963	*Kabuli*	*Ca_Kabuli_Chr05*	17494714	(A/G)
CakSNP7964	*Kabuli*	*Ca_Kabuli_Chr05*	17494702	(G/A)
CakSNP7965	*Kabuli*	*Ca_Kabuli_Chr05*	17494700	(T/C)
CakSNP7966	*Kabuli*	*Ca_Kabuli_Chr05*	17494697	(C/T)
CakSNP7967	*Kabuli*	*Ca_Kabuli_Chr05*	17494672	(C/T)
CakSNP7968	*Kabuli*	*Ca_Kabuli_Chr05*	17494699	(C/A)
CakSNP7969	*Kabuli*	*Ca_Kabuli_Chr05*	17494705	(A/T)
CakSNP7970	*Kabuli*	*Ca_Kabuli_Chr05*	17494710	(C/T)
CakSNP7971	*Kabuli*	*Ca_Kabuli_Chr05*	17494731	(A/T)
CakSNP7972	*Kabuli*	*Ca_Kabuli_Chr05*	18044808	(C/G)
CakSNP7973	*Kabuli*	*Ca_Kabuli_Chr05*	18664706	(C/T)
CakSNP7974	*Kabuli*	*Ca_Kabuli_Chr05*	18667782	(T/C)
CakSNP7975	*Kabuli*	*Ca_Kabuli_Chr05*	18667775	(T/C)
CakSNP7976	*Kabuli*	*Ca_Kabuli_Chr05*	18682788	(A/C)
CakSNP7977	*Kabuli*	*Ca_Kabuli_Chr05*	18682792	(T/C)
CakSNP7978	*Kabuli*	*Ca_Kabuli_Chr05*	18682793	(A/G)
CakSNP7979	*Kabuli*	*Ca_Kabuli_Chr05*	18682794	(A/G)
CakSNP7980	*Kabuli*	*Ca_Kabuli_Chr05*	18682812	(A/G)
CakSNP7981	*Kabuli*	*Ca_Kabuli_Chr05*	18682814	(T/A)
CakSNP7982	*Kabuli*	*Ca_Kabuli_Chr05*	18682822	(A/G)
CakSNP7983	*Kabuli*	*Ca_Kabuli_Chr05*	18682827	(C/G)
CakSNP7984	*Kabuli*	*Ca_Kabuli_Chr05*	18682830	(G/A)
CakSNP7985	*Kabuli*	*Ca_Kabuli_Chr05*	18684334	(C/T)
CakSNP7986	*Kabuli*	*Ca_Kabuli_Chr05*	18971085	(G/A)
CakSNP7987	*Kabuli*	*Ca_Kabuli_Chr05*	18971088	(C/A)
CakSNP7988	*Kabuli*	*Ca_Kabuli_Chr05*	19421820	(C/T)
CakSNP7989	*Kabuli*	*Ca_Kabuli_Chr05*	19421868	(C/T)
CakSNP7990	*Kabuli*	*Ca_Kabuli_Chr05*	19423387	(G/A)
CakSNP7991	*Kabuli*	*Ca_Kabuli_Chr05*	19423357	(C/T)
CakSNP7992	*Kabuli*	*Ca_Kabuli_Chr05*	19828124	(G/A)
CakSNP7993	*Kabuli*	*Ca_Kabuli_Chr05*	19828151	(C/A)
CakSNP7994	*Kabuli*	*Ca_Kabuli_Chr05*	19828163	(C/T)

SNP IDs	Cultivars	Chromosomes/scaffolds	Physical positions (bp)	SNPs
CakSNP7995	*Kabuli*	*Ca_Kabuli_Chr05*	19828166	(C/T)
CakSNP7996	*Kabuli*	*Ca_Kabuli_Chr05*	19828167	(A/C)
CakSNP7997	*Kabuli*	*Ca_Kabuli_Chr05*	19828226	(G/A)
CakSNP7998	*Kabuli*	*Ca_Kabuli_Chr05*	19828190	(T/C)
CakSNP7999	*Kabuli*	*Ca_Kabuli_Chr05*	19828184	(C/A)
CakSNP8000	*Kabuli*	*Ca_Kabuli_Chr05*	19828172	(T/G)
CakSNP8001	*Kabuli*	*Ca_Kabuli_Chr05*	19828144	(A/G)
CakSNP8002	*Kabuli*	*Ca_Kabuli_Chr05*	19884132	(C/T)
CakSNP8003	*Kabuli*	*Ca_Kabuli_Chr05*	19884124	(A/G)
CakSNP8004	*Kabuli*	*Ca_Kabuli_Chr05*	20008545	(C/T)
CakSNP8005	*Kabuli*	*Ca_Kabuli_Chr05*	20031711	(A/G)
CakSNP8006	*Kabuli*	*Ca_Kabuli_Chr05*	20354352	(T/C)
CakSNP8007	*Kabuli*	*Ca_Kabuli_Chr05*	20359674	(G/A)
CakSNP8008	*Kabuli*	*Ca_Kabuli_Chr05*	20460857	(G/A)
CakSNP8009	*Kabuli*	*Ca_Kabuli_Chr05*	20460859	(T/G)
CakSNP8010	*Kabuli*	*Ca_Kabuli_Chr05*	20546402	(T/C)
CakSNP8011	*Kabuli*	*Ca_Kabuli_Chr05*	20546385	(T/G)
CakSNP8012	*Kabuli*	*Ca_Kabuli_Chr05*	20644984	(C/A)
CakSNP8013	*Kabuli*	*Ca_Kabuli_Chr05*	20834519	(A/G)
CakSNP8014	*Kabuli*	*Ca_Kabuli_Chr05*	21163371	(G/A)
CakSNP8015	*Kabuli*	*Ca_Kabuli_Chr05*	21385948	(T/C)
CakSNP8016	*Kabuli*	*Ca_Kabuli_Chr05*	21495583	(C/A)
CakSNP8017	*Kabuli*	*Ca_Kabuli_Chr05*	21495631	(C/T)
CakSNP8018	*Kabuli*	*Ca_Kabuli_Chr05*	21495672	(G/A)
CakSNP8019	*Kabuli*	*Ca_Kabuli_Chr05*	21495707	(A/G)
CakSNP8020	*Kabuli*	*Ca_Kabuli_Chr05*	21495679	(G/A)
CakSNP8021	*Kabuli*	*Ca_Kabuli_Chr05*	21495674	(G/A)
CakSNP8022	*Kabuli*	*Ca_Kabuli_Chr05*	21769327	(G/A)
CakSNP8023	*Kabuli*	*Ca_Kabuli_Chr05*	21769419	(C/G)
CakSNP8024	*Kabuli*	*Ca_Kabuli_Chr05*	21769794	(A/C)
CakSNP8025	*Kabuli*	*Ca_Kabuli_Chr05*	22027215	(T/C)
CakSNP8026	*Kabuli*	*Ca_Kabuli_Chr05*	22027257	(G/T)
CakSNP8027	*Kabuli*	*Ca_Kabuli_Chr05*	22027265	(A/T)
CakSNP8028	*Kabuli*	*Ca_Kabuli_Chr05*	22029684	(C/A)
CakSNP8029	*Kabuli*	*Ca_Kabuli_Chr05*	22029686	(G/A)
CakSNP8030	*Kabuli*	*Ca_Kabuli_Chr05*	22029761	(C/T)
CakSNP8031	*Kabuli*	*Ca_Kabuli_Chr05*	22065518	(C/T)
CakSNP8032	*Kabuli*	*Ca_Kabuli_Chr05*	22065859	(G/A)
CakSNP8033	*Kabuli*	*Ca_Kabuli_Chr05*	22066034	(A/T)
CakSNP8034	*Kabuli*	*Ca_Kabuli_Chr05*	22116961	(T/G)
CakSNP8035	*Kabuli*	*Ca_Kabuli_Chr05*	22188341	(C/T)

SNP IDs	Cultivars	Chromosomes/scaffolds	Physical positions (bp)	SNPs
CakSNP8036	*Kabuli*	*Ca_Kabuli_Chr05*	22188318	(C/T)
CakSNP8037	*Kabuli*	*Ca_Kabuli_Chr05*	22296731	(G/A)
CakSNP8038	*Kabuli*	*Ca_Kabuli_Chr05*	22296728	(G/T)
CakSNP8039	*Kabuli*	*Ca_Kabuli_Chr05*	22302350	(A/C)
CakSNP8040	*Kabuli*	*Ca_Kabuli_Chr05*	22344868	(A/G)
CakSNP8041	*Kabuli*	*Ca_Kabuli_Chr05*	22445954	(T/C)
CakSNP8042	*Kabuli*	*Ca_Kabuli_Chr05*	22679380	(C/A)
CakSNP8043	*Kabuli*	*Ca_Kabuli_Chr05*	23039312	(C/T)
CakSNP8044	*Kabuli*	*Ca_Kabuli_Chr05*	23049683	(C/A)
CakSNP8045	*Kabuli*	*Ca_Kabuli_Chr05*	23157899	(A/G)
CakSNP8046	*Kabuli*	*Ca_Kabuli_Chr05*	23161492	(A/T)
CakSNP8047	*Kabuli*	*Ca_Kabuli_Chr05*	23261964	(A/G)
CakSNP8048	*Kabuli*	*Ca_Kabuli_Chr05*	23262114	(C/A)
CakSNP8049	*Kabuli*	*Ca_Kabuli_Chr05*	23262264	(C/A)
CakSNP8050	*Kabuli*	*Ca_Kabuli_Chr05*	23323538	(G/A)
CakSNP8051	*Kabuli*	*Ca_Kabuli_Chr05*	23323520	(C/A)
CakSNP8052	*Kabuli*	*Ca_Kabuli_Chr05*	23323514	(C/A)
CakSNP8053	*Kabuli*	*Ca_Kabuli_Chr05*	23323479	(A/T)
CakSNP8054	*Kabuli*	*Ca_Kabuli_Chr05*	23323478	(A/T)
CakSNP8055	*Kabuli*	*Ca_Kabuli_Chr05*	23440293	(C/T)
CakSNP8056	*Kabuli*	*Ca_Kabuli_Chr05*	23449048	(T/C)
CakSNP8057	*Kabuli*	*Ca_Kabuli_Chr05*	23449134	(A/C)
CakSNP8058	*Kabuli*	*Ca_Kabuli_Chr05*	23449127	(C/A)
CakSNP8059	*Kabuli*	*Ca_Kabuli_Chr05*	23562595	(G/A)
CakSNP8060	*Kabuli*	*Ca_Kabuli_Chr05*	23562827	(G/A)
CakSNP8061	*Kabuli*	*Ca_Kabuli_Chr05*	23674814	(C/G)
CakSNP8062	*Kabuli*	*Ca_Kabuli_Chr05*	23674815	(C/T)
CakSNP8063	*Kabuli*	*Ca_Kabuli_Chr05*	23674845	(G/C)
CakSNP8064	*Kabuli*	*Ca_Kabuli_Chr05*	23783140	(T/C)
CakSNP8065	*Kabuli*	*Ca_Kabuli_Chr05*	23783156	(C/A)
CakSNP8066	*Kabuli*	*Ca_Kabuli_Chr05*	23783157	(G/C)
CakSNP8067	*Kabuli*	*Ca_Kabuli_Chr05*	23783188	(G/A)
CakSNP8068	*Kabuli*	*Ca_Kabuli_Chr05*	23783195	(C/G)
CakSNP8069	*Kabuli*	*Ca_Kabuli_Chr05*	23783254	(A/G)
CakSNP8070	*Kabuli*	*Ca_Kabuli_Chr05*	23783252	(T/A)
CakSNP8071	*Kabuli*	*Ca_Kabuli_Chr05*	23884909	(C/T)
CakSNP8072	*Kabuli*	*Ca_Kabuli_Chr05*	23884936	(G/A)
CakSNP8073	*Kabuli*	*Ca_Kabuli_Chr05*	23930933	(C/T)
CakSNP8074	*Kabuli*	*Ca_Kabuli_Chr05*	23930988	(G/A)
CakSNP8075	*Kabuli*	*Ca_Kabuli_Chr05*	23932354	(C/T)
CakSNP8076	*Kabuli*	*Ca_Kabuli_Chr05*	23934198	(G/T)

SNP IDs	Cultivars	Chromosomes/scaffolds	Physical positions (bp)	SNPs
CakSNP8077	*Kabuli*	*Ca_Kabuli_Chr05*	24059182	(C/G)
CakSNP8078	*Kabuli*	*Ca_Kabuli_Chr05*	24090533	(C/T)
CakSNP8079	*Kabuli*	*Ca_Kabuli_Chr05*	24090523	(A/C)
CakSNP8080	*Kabuli*	*Ca_Kabuli_Chr05*	24090515	(A/C)
CakSNP8081	*Kabuli*	*Ca_Kabuli_Chr05*	24090749	(A/C)
CakSNP8082	*Kabuli*	*Ca_Kabuli_Chr05*	24097823	(T/C)
CakSNP8083	*Kabuli*	*Ca_Kabuli_Chr05*	24153061	(G/C)
CakSNP8084	*Kabuli*	*Ca_Kabuli_Chr05*	24153205	(T/C)
CakSNP8085	*Kabuli*	*Ca_Kabuli_Chr05*	24153273	(T/C)
CakSNP8086	*Kabuli*	*Ca_Kabuli_Chr05*	24153202	(A/C)
CakSNP8087	*Kabuli*	*Ca_Kabuli_Chr05*	24153199	(C/T)
CakSNP8088	*Kabuli*	*Ca_Kabuli_Chr05*	24166103	(C/T)
CakSNP8089	*Kabuli*	*Ca_Kabuli_Chr05*	24166095	(G/A)
CakSNP8090	*Kabuli*	*Ca_Kabuli_Chr05*	24166093	(A/G)
CakSNP8091	*Kabuli*	*Ca_Kabuli_Chr05*	24166046	(G/A)
CakSNP8092	*Kabuli*	*Ca_Kabuli_Chr05*	24166047	(C/T)
CakSNP8093	*Kabuli*	*Ca_Kabuli_Chr05*	24166082	(G/A)
CakSNP8094	*Kabuli*	*Ca_Kabuli_Chr05*	24181232	(C/A)
CakSNP8095	*Kabuli*	*Ca_Kabuli_Chr05*	24181228	(C/A)
CakSNP8096	*Kabuli*	*Ca_Kabuli_Chr05*	24245312	(C/A)
CakSNP8097	*Kabuli*	*Ca_Kabuli_Chr05*	24245308	(C/A)
CakSNP8098	*Kabuli*	*Ca_Kabuli_Chr05*	24346350	(A/T)
CakSNP8099	*Kabuli*	*Ca_Kabuli_Chr05*	24576960	(C/T)
CakSNP8100	*Kabuli*	*Ca_Kabuli_Chr05*	24576970	(A/G)
CakSNP8101	*Kabuli*	*Ca_Kabuli_Chr05*	24576993	(T/G)
CakSNP8102	*Kabuli*	*Ca_Kabuli_Chr05*	24596227	(T/C)
CakSNP8103	*Kabuli*	*Ca_Kabuli_Chr05*	24596257	(C/T)
CakSNP8104	*Kabuli*	*Ca_Kabuli_Chr05*	24596269	(G/A)
CakSNP8105	*Kabuli*	*Ca_Kabuli_Chr05*	24605438	(A/C)
CakSNP8106	*Kabuli*	*Ca_Kabuli_Chr05*	24843015	(G/T)
CakSNP8107	*Kabuli*	*Ca_Kabuli_Chr05*	25003171	(C/T)
CakSNP8108	*Kabuli*	*Ca_Kabuli_Chr05*	25006110	(A/C)
CakSNP8109	*Kabuli*	*Ca_Kabuli_Chr05*	25006123	(C/T)
CakSNP8110	*Kabuli*	*Ca_Kabuli_Chr05*	25184089	(T/C)
CakSNP8111	*Kabuli*	*Ca_Kabuli_Chr05*	25184133	(G/A)
CakSNP8112	*Kabuli*	*Ca_Kabuli_Chr05*	25399115	(T/G)
CakSNP8113	*Kabuli*	*Ca_Kabuli_Chr05*	25400604	(A/G)
CakSNP8114	*Kabuli*	*Ca_Kabuli_Chr05*	25474110	(A/C)
CakSNP8115	*Kabuli*	*Ca_Kabuli_Chr05*	25474105	(C/T)
CakSNP8116	*Kabuli*	*Ca_Kabuli_Chr05*	25474093	(G/C)
CakSNP8117	*Kabuli*	*Ca_Kabuli_Chr05*	25474089	(C/A)

SNP IDs	Cultivars	Chromosomes/scaffolds	Physical positions (bp)	SNPs
CakSNP8118	*Kabuli*	*Ca_Kabuli_Chr05*	25597077	(A/G)
CakSNP8119	*Kabuli*	*Ca_Kabuli_Chr05*	25678975	(G/T)
CakSNP8120	*Kabuli*	*Ca_Kabuli_Chr05*	25697487	(C/G)
CakSNP8121	*Kabuli*	*Ca_Kabuli_Chr05*	25805020	(A/G)
CakSNP8122	*Kabuli*	*Ca_Kabuli_Chr05*	26025243	(C/T)
CakSNP8123	*Kabuli*	*Ca_Kabuli_Chr05*	26025282	(A/G)
CakSNP8124	*Kabuli*	*Ca_Kabuli_Chr05*	26025356	(A/T)
CakSNP8125	*Kabuli*	*Ca_Kabuli_Chr05*	26027313	(C/A)
CakSNP8126	*Kabuli*	*Ca_Kabuli_Chr05*	26095084	(T/G)
CakSNP8127	*Kabuli*	*Ca_Kabuli_Chr05*	26124240	(T/C)
CakSNP8128	*Kabuli*	*Ca_Kabuli_Chr05*	26191896	(T/C)
CakSNP8129	*Kabuli*	*Ca_Kabuli_Chr05*	26194579	(A/G)
CakSNP8130	*Kabuli*	*Ca_Kabuli_Chr05*	26238764	(T/G)
CakSNP8131	*Kabuli*	*Ca_Kabuli_Chr05*	26238752	(A/T)
CakSNP8132	*Kabuli*	*Ca_Kabuli_Chr05*	26238748	(A/T)
CakSNP8133	*Kabuli*	*Ca_Kabuli_Chr05*	26238741	(G/T)
CakSNP8134	*Kabuli*	*Ca_Kabuli_Chr05*	26238739	(C/T)
CakSNP8135	*Kabuli*	*Ca_Kabuli_Chr05*	26384636	(C/G)
CakSNP8136	*Kabuli*	*Ca_Kabuli_Chr05*	26467869	(A/C)
CakSNP8137	*Kabuli*	*Ca_Kabuli_Chr05*	26474910	(A/C)
CakSNP8138	*Kabuli*	*Ca_Kabuli_Chr05*	26475067	(T/C)
CakSNP8139	*Kabuli*	*Ca_Kabuli_Chr05*	26477751	(A/T)
CakSNP8140	*Kabuli*	*Ca_Kabuli_Chr05*	26494631	(A/G)
CakSNP8141	*Kabuli*	*Ca_Kabuli_Chr05*	26494778	(T/C)
CakSNP8142	*Kabuli*	*Ca_Kabuli_Chr05*	26494769	(T/C)
CakSNP8143	*Kabuli*	*Ca_Kabuli_Chr05*	26496197	(A/G)
CakSNP8144	*Kabuli*	*Ca_Kabuli_Chr05*	26558406	(A/G)
CakSNP8145	*Kabuli*	*Ca_Kabuli_Chr05*	26558366	(A/G)
CakSNP8146	*Kabuli*	*Ca_Kabuli_Chr05*	26558363	(C/G)
CakSNP8147	*Kabuli*	*Ca_Kabuli_Chr05*	26558357	(G/A)
CakSNP8148	*Kabuli*	*Ca_Kabuli_Chr05*	26558356	(G/T)
CakSNP8149	*Kabuli*	*Ca_Kabuli_Chr05*	26569400	(G/C)
CakSNP8150	*Kabuli*	*Ca_Kabuli_Chr05*	26609014	(T/C)
CakSNP8151	*Kabuli*	*Ca_Kabuli_Chr05*	26608993	(A/G)
CakSNP8152	*Kabuli*	*Ca_Kabuli_Chr05*	26621093	(C/G)
CakSNP8153	*Kabuli*	*Ca_Kabuli_Chr05*	26626861	(A/G)
CakSNP8154	*Kabuli*	*Ca_Kabuli_Chr05*	26626855	(T/G)
CakSNP8155	*Kabuli*	*Ca_Kabuli_Chr05*	26638041	(G/A)
CakSNP8156	*Kabuli*	*Ca_Kabuli_Chr05*	26638042	(C/T)
CakSNP8157	*Kabuli*	*Ca_Kabuli_Chr05*	26638080	(A/G)
CakSNP8158	*Kabuli*	*Ca_Kabuli_Chr05*	26638126	(C/T)

SNP IDs	Cultivars	Chromosomes/scaffolds	Physical positions (bp)	SNPs
CakSNP8159	*Kabuli*	*Ca_Kabuli_Chr05*	26638118	(C/T)
CakSNP8160	*Kabuli*	*Ca_Kabuli_Chr05*	26679836	(C/T)
CakSNP8161	*Kabuli*	*Ca_Kabuli_Chr05*	26696390	(G/A)
CakSNP8162	*Kabuli*	*Ca_Kabuli_Chr05*	26696412	(C/A)
CakSNP8163	*Kabuli*	*Ca_Kabuli_Chr05*	26696455	(G/A)
CakSNP8164	*Kabuli*	*Ca_Kabuli_Chr05*	26696547	(C/T)
CakSNP8165	*Kabuli*	*Ca_Kabuli_Chr05*	26696533	(C/T)
CakSNP8166	*Kabuli*	*Ca_Kabuli_Chr05*	26696494	(G/A)
CakSNP8167	*Kabuli*	*Ca_Kabuli_Chr05*	26696578	(G/A)
CakSNP8168	*Kabuli*	*Ca_Kabuli_Chr05*	26696602	(C/T)
CakSNP8169	*Kabuli*	*Ca_Kabuli_Chr05*	26696620	(C/T)
CakSNP8170	*Kabuli*	*Ca_Kabuli_Chr05*	26696624	(G/A)
CakSNP8171	*Kabuli*	*Ca_Kabuli_Chr05*	26785140	(G/A)
CakSNP8172	*Kabuli*	*Ca_Kabuli_Chr05*	26785226	(C/A)
CakSNP8173	*Kabuli*	*Ca_Kabuli_Chr05*	26997335	(T/G)
CakSNP8174	*Kabuli*	*Ca_Kabuli_Chr05*	26999793	(C/T)
CakSNP8175	*Kabuli*	*Ca_Kabuli_Chr05*	27000127	(C/G)
CakSNP8176	*Kabuli*	*Ca_Kabuli_Chr05*	27000104	(C/A)
CakSNP8177	*Kabuli*	*Ca_Kabuli_Chr05*	27000278	(T/A)
CakSNP8178	*Kabuli*	*Ca_Kabuli_Chr05*	27000280	(T/G)
CakSNP8179	*Kabuli*	*Ca_Kabuli_Chr05*	27025766	(A/G)
CakSNP8180	*Kabuli*	*Ca_Kabuli_Chr05*	27025752	(A/C)
CakSNP8181	*Kabuli*	*Ca_Kabuli_Chr05*	27042679	(G/A)
CakSNP8182	*Kabuli*	*Ca_Kabuli_Chr05*	27117953	(A/G)
CakSNP8183	*Kabuli*	*Ca_Kabuli_Chr05*	27150550	(A/G)
CakSNP8184	*Kabuli*	*Ca_Kabuli_Chr05*	27230515	(T/A)
CakSNP8185	*Kabuli*	*Ca_Kabuli_Chr05*	27230514	(A/G)
CakSNP8186	*Kabuli*	*Ca_Kabuli_Chr05*	27231394	(T/C)
CakSNP8187	*Kabuli*	*Ca_Kabuli_Chr05*	27231641	(T/A)
CakSNP8188	*Kabuli*	*Ca_Kabuli_Chr05*	27232304	(T/A)
CakSNP8189	*Kabuli*	*Ca_Kabuli_Chr05*	27234806	(G/A)
CakSNP8190	*Kabuli*	*Ca_Kabuli_Chr05*	27235002	(T/G)
CakSNP8191	*Kabuli*	*Ca_Kabuli_Chr05*	27328552	(G/A)
CakSNP8192	*Kabuli*	*Ca_Kabuli_Chr05*	27328737	(G/T)
CakSNP8193	*Kabuli*	*Ca_Kabuli_Chr05*	27334056	(A/C)
CakSNP8194	*Kabuli*	*Ca_Kabuli_Chr05*	27334057	(G/C)
CakSNP8195	*Kabuli*	*Ca_Kabuli_Chr05*	27334061	(G/C)
CakSNP8196	*Kabuli*	*Ca_Kabuli_Chr05*	27349210	(T/A)
CakSNP8197	*Kabuli*	*Ca_Kabuli_Chr05*	27349237	(C/T)
CakSNP8198	*Kabuli*	*Ca_Kabuli_Chr05*	27361515	(C/T)
CakSNP8199	*Kabuli*	*Ca_Kabuli_Chr05*	27361579	(T/G)

SNP IDs	Cultivars	Chromosomes/scaffolds	Physical positions (bp)	SNPs
CakSNP8200	*Kabuli*	*Ca_Kabuli_Chr05*	27362517	(C/G)
CakSNP8201	*Kabuli*	*Ca_Kabuli_Chr05*	27362570	(G/T)
CakSNP8202	*Kabuli*	*Ca_Kabuli_Chr05*	27362573	(A/G)
CakSNP8203	*Kabuli*	*Ca_Kabuli_Chr05*	27362584	(T/G)
CakSNP8204	*Kabuli*	*Ca_Kabuli_Chr05*	27452484	(C/G)
CakSNP8205	*Kabuli*	*Ca_Kabuli_Chr05*	27516054	(T/A)
CakSNP8206	*Kabuli*	*Ca_Kabuli_Chr05*	27552106	(A/C)
CakSNP8207	*Kabuli*	*Ca_Kabuli_Chr05*	27555637	(T/G)
CakSNP8208	*Kabuli*	*Ca_Kabuli_Chr05*	27568101	(T/G)
CakSNP8209	*Kabuli*	*Ca_Kabuli_Chr05*	27568095	(C/A)
CakSNP8210	*Kabuli*	*Ca_Kabuli_Chr05*	27568064	(A/C)
CakSNP8211	*Kabuli*	*Ca_Kabuli_Chr05*	27568059	(C/T)
CakSNP8212	*Kabuli*	*Ca_Kabuli_Chr05*	27568028	(A/T)
CakSNP8213	*Kabuli*	*Ca_Kabuli_Chr05*	27568033	(G/A)
CakSNP8214	*Kabuli*	*Ca_Kabuli_Chr05*	27568082	(G/A)
CakSNP8215	*Kabuli*	*Ca_Kabuli_Chr05*	27568097	(G/A)
CakSNP8216	*Kabuli*	*Ca_Kabuli_Chr05*	27568098	(C/T)
CakSNP8217	*Kabuli*	*Ca_Kabuli_Chr05*	27568118	(A/G)
CakSNP8218	*Kabuli*	*Ca_Kabuli_Chr05*	27568133	(G/T)
CakSNP8219	*Kabuli*	*Ca_Kabuli_Chr05*	27568081	(G/C)
CakSNP8220	*Kabuli*	*Ca_Kabuli_Chr05*	27568107	(C/G)
CakSNP8221	*Kabuli*	*Ca_Kabuli_Chr05*	27568156	(C/G)
CakSNP8222	*Kabuli*	*Ca_Kabuli_Chr05*	27568141	(C/T)
CakSNP8223	*Kabuli*	*Ca_Kabuli_Chr05*	27568180	(C/T)
CakSNP8224	*Kabuli*	*Ca_Kabuli_Chr05*	27568176	(T/C)
CakSNP8225	*Kabuli*	*Ca_Kabuli_Chr05*	27579347	(C/T)
CakSNP8226	*Kabuli*	*Ca_Kabuli_Chr05*	27605037	(A/T)
CakSNP8227	*Kabuli*	*Ca_Kabuli_Chr05*	27605063	(T/A)
CakSNP8228	*Kabuli*	*Ca_Kabuli_Chr05*	27605081	(C/T)
CakSNP8229	*Kabuli*	*Ca_Kabuli_Chr05*	27605095	(G/C)
CakSNP8230	*Kabuli*	*Ca_Kabuli_Chr05*	27692816	(C/T)
CakSNP8231	*Kabuli*	*Ca_Kabuli_Chr05*	27692818	(C/T)
CakSNP8232	*Kabuli*	*Ca_Kabuli_Chr05*	27692822	(C/T)
CakSNP8233	*Kabuli*	*Ca_Kabuli_Chr05*	27692823	(A/T)
CakSNP8234	*Kabuli*	*Ca_Kabuli_Chr05*	27692831	(A/T)
CakSNP8235	*Kabuli*	*Ca_Kabuli_Chr05*	27694205	(C/A)
CakSNP8236	*Kabuli*	*Ca_Kabuli_Chr05*	27750155	(C/T)
CakSNP8237	*Kabuli*	*Ca_Kabuli_Chr05*	27786025	(T/C)
CakSNP8238	*Kabuli*	*Ca_Kabuli_Chr05*	27838003	(T/C)
CakSNP8239	*Kabuli*	*Ca_Kabuli_Chr05*	27850760	(T/C)
CakSNP8240	*Kabuli*	*Ca_Kabuli_Chr05*	27850735	(C/A)

SNP IDs	Cultivars	Chromosomes/scaffolds	Physical positions (bp)	SNPs
CakSNP8241	*Kabuli*	*Ca_Kabuli_Chr05*	27853444	(C/T)
CakSNP8242	*Kabuli*	*Ca_Kabuli_Chr05*	27872968	(C/T)
CakSNP8243	*Kabuli*	*Ca_Kabuli_Chr05*	27873001	(T/C)
CakSNP8244	*Kabuli*	*Ca_Kabuli_Chr05*	27874414	(C/A)
CakSNP8245	*Kabuli*	*Ca_Kabuli_Chr05*	27936521	(A/G)
CakSNP8246	*Kabuli*	*Ca_Kabuli_Chr05*	27936524	(C/T)
CakSNP8247	*Kabuli*	*Ca_Kabuli_Chr05*	27939162	(G/A)
CakSNP8248	*Kabuli*	*Ca_Kabuli_Chr05*	27939135	(G/C)
CakSNP8249	*Kabuli*	*Ca_Kabuli_Chr05*	27939134	(A/T)
CakSNP8250	*Kabuli*	*Ca_Kabuli_Chr05*	27939128	(A/G)
CakSNP8251	*Kabuli*	*Ca_Kabuli_Chr05*	27974691	(G/T)
CakSNP8252	*Kabuli*	*Ca_Kabuli_Chr05*	28016628	(C/T)
CakSNP8253	*Kabuli*	*Ca_Kabuli_Chr05*	28016669	(A/G)
CakSNP8254	*Kabuli*	*Ca_Kabuli_Chr05*	28071467	(A/G)
CakSNP8255	*Kabuli*	*Ca_Kabuli_Chr05*	28116818	(A/G)
CakSNP8256	*Kabuli*	*Ca_Kabuli_Chr05*	28116988	(A/T)
CakSNP8257	*Kabuli*	*Ca_Kabuli_Chr05*	28117011	(C/T)
CakSNP8258	*Kabuli*	*Ca_Kabuli_Chr05*	28164985	(G/C)
CakSNP8259	*Kabuli*	*Ca_Kabuli_Chr05*	28168910	(A/G)
CakSNP8260	*Kabuli*	*Ca_Kabuli_Chr05*	28169475	(T/C)
CakSNP8261	*Kabuli*	*Ca_Kabuli_Chr05*	28175039	(A/G)
CakSNP8262	*Kabuli*	*Ca_Kabuli_Chr05*	28266046	(T/G)
CakSNP8263	*Kabuli*	*Ca_Kabuli_Chr05*	28271638	(G/C)
CakSNP8264	*Kabuli*	*Ca_Kabuli_Chr05*	28271732	(C/T)
CakSNP8265	*Kabuli*	*Ca_Kabuli_Chr05*	28271733	(G/A)
CakSNP8266	*Kabuli*	*Ca_Kabuli_Chr05*	28271742	(G/A)
CakSNP8267	*Kabuli*	*Ca_Kabuli_Chr05*	28271767	(G/C)
CakSNP8268	*Kabuli*	*Ca_Kabuli_Chr05*	28284640	(T/C)
CakSNP8269	*Kabuli*	*Ca_Kabuli_Chr05*	28327832	(T/G)
CakSNP8270	*Kabuli*	*Ca_Kabuli_Chr05*	28327883	(G/A)
CakSNP8271	*Kabuli*	*Ca_Kabuli_Chr05*	28327870	(T/C)
CakSNP8272	*Kabuli*	*Ca_Kabuli_Chr05*	28327868	(G/A)
CakSNP8273	*Kabuli*	*Ca_Kabuli_Chr05*	28327856	(T/C)
CakSNP8274	*Kabuli*	*Ca_Kabuli_Chr05*	28335455	(A/C)
CakSNP8275	*Kabuli*	*Ca_Kabuli_Chr05*	28346497	(C/T)
CakSNP8276	*Kabuli*	*Ca_Kabuli_Chr05*	28360495	(C/T)
CakSNP8277	*Kabuli*	*Ca_Kabuli_Chr05*	28360492	(C/T)
CakSNP8278	*Kabuli*	*Ca_Kabuli_Chr05*	28360585	(A/G)
CakSNP8279	*Kabuli*	*Ca_Kabuli_Chr05*	28383374	(G/T)
CakSNP8280	*Kabuli*	*Ca_Kabuli_Chr05*	28491113	(G/A)
CakSNP8281	*Kabuli*	*Ca_Kabuli_Chr05*	28491131	(G/A)

SNP IDs	Cultivars	Chromosomes/scaffolds	Physical positions (bp)	SNPs
CakSNP8282	*Kabuli*	*Ca_Kabuli_Chr05*	28509048	(G/A)
CakSNP8283	*Kabuli*	*Ca_Kabuli_Chr05*	28509066	(G/A)
CakSNP8284	*Kabuli*	*Ca_Kabuli_Chr05*	28562734	(G/A)
CakSNP8285	*Kabuli*	*Ca_Kabuli_Chr05*	28581814	(A/G)
CakSNP8286	*Kabuli*	*Ca_Kabuli_Chr05*	28581815	(C/G)
CakSNP8287	*Kabuli*	*Ca_Kabuli_Chr05*	28581827	(T/C)
CakSNP8288	*Kabuli*	*Ca_Kabuli_Chr05*	28581866	(T/C)
CakSNP8289	*Kabuli*	*Ca_Kabuli_Chr05*	28581926	(C/A)
CakSNP8290	*Kabuli*	*Ca_Kabuli_Chr05*	28582525	(C/T)
CakSNP8291	*Kabuli*	*Ca_Kabuli_Chr05*	28582806	(T/C)
CakSNP8292	*Kabuli*	*Ca_Kabuli_Chr05*	28582763	(A/G)
CakSNP8293	*Kabuli*	*Ca_Kabuli_Chr05*	28583402	(G/A)
CakSNP8294	*Kabuli*	*Ca_Kabuli_Chr05*	28583375	(C/G)
CakSNP8295	*Kabuli*	*Ca_Kabuli_Chr05*	28682973	(A/C)
CakSNP8296	*Kabuli*	*Ca_Kabuli_Chr05*	28682995	(G/A)
CakSNP8297	*Kabuli*	*Ca_Kabuli_Chr05*	28683084	(A/C)
CakSNP8298	*Kabuli*	*Ca_Kabuli_Chr05*	28683070	(T/A)
CakSNP8299	*Kabuli*	*Ca_Kabuli_Chr05*	28683069	(A/T)
CakSNP8300	*Kabuli*	*Ca_Kabuli_Chr05*	28714658	(C/T)
CakSNP8301	*Kabuli*	*Ca_Kabuli_Chr05*	28717362	(C/T)
CakSNP8302	*Kabuli*	*Ca_Kabuli_Chr05*	28717384	(C/T)
CakSNP8303	*Kabuli*	*Ca_Kabuli_Chr05*	28721236	(C/T)
CakSNP8304	*Kabuli*	*Ca_Kabuli_Chr05*	28721237	(A/G)
CakSNP8305	*Kabuli*	*Ca_Kabuli_Chr05*	28721386	(T/C)
CakSNP8306	*Kabuli*	*Ca_Kabuli_Chr05*	28721383	(A/C)
CakSNP8307	*Kabuli*	*Ca_Kabuli_Chr05*	28746139	(A/G)
CakSNP8308	*Kabuli*	*Ca_Kabuli_Chr05*	28757467	(A/T)
CakSNP8309	*Kabuli*	*Ca_Kabuli_Chr05*	28757474	(T/C)
CakSNP8310	*Kabuli*	*Ca_Kabuli_Chr05*	28757479	(T/C)
CakSNP8311	*Kabuli*	*Ca_Kabuli_Chr05*	28757485	(A/C)
CakSNP8312	*Kabuli*	*Ca_Kabuli_Chr05*	28757507	(A/T)
CakSNP8313	*Kabuli*	*Ca_Kabuli_Chr05*	28758723	(A/G)
CakSNP8314	*Kabuli*	*Ca_Kabuli_Chr05*	28817832	(A/G)
CakSNP8315	*Kabuli*	*Ca_Kabuli_Chr05*	28827467	(A/G)
CakSNP8316	*Kabuli*	*Ca_Kabuli_Chr05*	28843854	(G/A)
CakSNP8317	*Kabuli*	*Ca_Kabuli_Chr05*	28843801	(A/G)
CakSNP8318	*Kabuli*	*Ca_Kabuli_Chr05*	28843795	(A/T)
CakSNP8319	*Kabuli*	*Ca_Kabuli_Chr05*	28843782	(C/G)
CakSNP8320	*Kabuli*	*Ca_Kabuli_Chr05*	28843775	(C/T)
CakSNP8321	*Kabuli*	*Ca_Kabuli_Chr05*	28854486	(C/T)
CakSNP8322	*Kabuli*	*Ca_Kabuli_Chr05*	28905203	(A/C)

**SNP IDs**	**Cultivars**	**Chromosomes/scaffolds**	**Physical positions (bp)**	**SNPs**
CakSNP8323	*Kabuli*	*Ca_Kabuli_Chr05*	29007895	(T/C)
CakSNP8324	*Kabuli*	*Ca_Kabuli_Chr05*	29007886	(C/A)
CakSNP8325	*Kabuli*	*Ca_Kabuli_Chr05*	29007884	(A/C)
CakSNP8326	*Kabuli*	*Ca_Kabuli_Chr05*	29007882	(A/C)
CakSNP8327	*Kabuli*	*Ca_Kabuli_Chr05*	29007879	(T/C)
CakSNP8328	*Kabuli*	*Ca_Kabuli_Chr05*	29007847	(A/C)
CakSNP8329	*Kabuli*	*Ca_Kabuli_Chr05*	29007844	(T/C)
CakSNP8330	*Kabuli*	*Ca_Kabuli_Chr05*	29061056	(T/A)
CakSNP8331	*Kabuli*	*Ca_Kabuli_Chr05*	29061025	(A/C)
CakSNP8332	*Kabuli*	*Ca_Kabuli_Chr05*	29060992	(C/T)
CakSNP8333	*Kabuli*	*Ca_Kabuli_Chr05*	29062696	(G/A)
CakSNP8334	*Kabuli*	*Ca_Kabuli_Chr05*	29062738	(A/G)
CakSNP8335	*Kabuli*	*Ca_Kabuli_Chr05*	29079931	(G/A)
CakSNP8336	*Kabuli*	*Ca_Kabuli_Chr05*	29079962	(A/G)
CakSNP8337	*Kabuli*	*Ca_Kabuli_Chr05*	29080066	(G/C)
CakSNP8338	*Kabuli*	*Ca_Kabuli_Chr05*	29080060	(C/T)
CakSNP8339	*Kabuli*	*Ca_Kabuli_Chr05*	29111292	(T/G)
CakSNP8340	*Kabuli*	*Ca_Kabuli_Chr05*	29111293	(T/C)
CakSNP8341	*Kabuli*	*Ca_Kabuli_Chr05*	29186179	(T/G)
CakSNP8342	*Kabuli*	*Ca_Kabuli_Chr05*	29220980	(T/A)
CakSNP8343	*Kabuli*	*Ca_Kabuli_Chr05*	29221003	(T/C)
CakSNP8344	*Kabuli*	*Ca_Kabuli_Chr05*	29221919	(T/A)
CakSNP8345	*Kabuli*	*Ca_Kabuli_Chr05*	29272117	(C/G)
CakSNP8346	*Kabuli*	*Ca_Kabuli_Chr05*	29272125	(C/T)
CakSNP8347	*Kabuli*	*Ca_Kabuli_Chr05*	29354269	(A/T)
CakSNP8348	*Kabuli*	*Ca_Kabuli_Chr05*	29422029	(T/A)
CakSNP8349	*Kabuli*	*Ca_Kabuli_Chr05*	29422066	(A/T)
CakSNP8350	*Kabuli*	*Ca_Kabuli_Chr05*	29422091	(C/T)
CakSNP8351	*Kabuli*	*Ca_Kabuli_Chr05*	29437139	(C/T)
CakSNP8352	*Kabuli*	*Ca_Kabuli_Chr05*	29437146	(C/A)
CakSNP8353	*Kabuli*	*Ca_Kabuli_Chr05*	29437159	(C/T)
CakSNP8354	*Kabuli*	*Ca_Kabuli_Chr05*	29437162	(A/C)
CakSNP8355	*Kabuli*	*Ca_Kabuli_Chr05*	29437171	(A/T)
CakSNP8356	*Kabuli*	*Ca_Kabuli_Chr05*	29437181	(A/C)
CakSNP8357	*Kabuli*	*Ca_Kabuli_Chr05*	29437189	(C/T)
CakSNP8358	*Kabuli*	*Ca_Kabuli_Chr05*	29437193	(A/C)
CakSNP8359	*Kabuli*	*Ca_Kabuli_Chr05*	29437182	(C/A)
CakSNP8360	*Kabuli*	*Ca_Kabuli_Chr05*	29528859	(A/T)
CakSNP8361	*Kabuli*	*Ca_Kabuli_Chr05*	29528890	(T/A)
CakSNP8362	*Kabuli*	*Ca_Kabuli_Chr05*	29528904	(T/G)
CakSNP8363	*Kabuli*	*Ca_Kabuli_Chr05*	29530996	(G/A)

SNP IDs	Cultivars	Chromosomes/scaffolds	Physical positions (bp)	SNPs
CakSNP8364	*Kabuli*	*Ca_Kabuli_Chr05*	29531122	(A/T)
CakSNP8365	*Kabuli*	*Ca_Kabuli_Chr05*	29531113	(G/A)
CakSNP8366	*Kabuli*	*Ca_Kabuli_Chr05*	29531218	(A/T)
CakSNP8367	*Kabuli*	*Ca_Kabuli_Chr05*	29531416	(A/G)
CakSNP8368	*Kabuli*	*Ca_Kabuli_Chr05*	29567676	(T/C)
CakSNP8369	*Kabuli*	*Ca_Kabuli_Chr05*	29567820	(T/C)
CakSNP8370	*Kabuli*	*Ca_Kabuli_Chr05*	29576690	(A/T)
CakSNP8371	*Kabuli*	*Ca_Kabuli_Chr05*	29576689	(G/A)
CakSNP8372	*Kabuli*	*Ca_Kabuli_Chr05*	29576716	(A/C)
CakSNP8373	*Kabuli*	*Ca_Kabuli_Chr05*	29608657	(T/C)
CakSNP8374	*Kabuli*	*Ca_Kabuli_Chr05*	29641659	(T/C)
CakSNP8375	*Kabuli*	*Ca_Kabuli_Chr05*	29658258	(G/C)
CakSNP8376	*Kabuli*	*Ca_Kabuli_Chr05*	29658280	(A/C)
CakSNP8377	*Kabuli*	*Ca_Kabuli_Chr05*	29667787	(C/G)
CakSNP8378	*Kabuli*	*Ca_Kabuli_Chr05*	29667783	(C/T)
CakSNP8379	*Kabuli*	*Ca_Kabuli_Chr05*	29667774	(G/C)
CakSNP8380	*Kabuli*	*Ca_Kabuli_Chr05*	29809862	(A/C)
CakSNP8381	*Kabuli*	*Ca_Kabuli_Chr05*	29816799	(T/C)
CakSNP8382	*Kabuli*	*Ca_Kabuli_Chr05*	29816800	(T/A)
CakSNP8383	*Kabuli*	*Ca_Kabuli_Chr05*	29824218	(T/G)
CakSNP8384	*Kabuli*	*Ca_Kabuli_Chr05*	29852130	(T/C)
CakSNP8385	*Kabuli*	*Ca_Kabuli_Chr05*	29852105	(T/G)
CakSNP8386	*Kabuli*	*Ca_Kabuli_Chr05*	29886086	(T/A)
CakSNP8387	*Kabuli*	*Ca_Kabuli_Chr05*	29981826	(A/G)
CakSNP8388	*Kabuli*	*Ca_Kabuli_Chr05*	29999414	(C/T)
CakSNP8389	*Kabuli*	*Ca_Kabuli_Chr05*	29999427	(G/A)
CakSNP8390	*Kabuli*	*Ca_Kabuli_Chr05*	29999433	(C/A)
CakSNP8391	*Kabuli*	*Ca_Kabuli_Chr05*	29999465	(T/G)
CakSNP8392	*Kabuli*	*Ca_Kabuli_Chr05*	29999476	(G/A)
CakSNP8393	*Kabuli*	*Ca_Kabuli_Chr05*	29999523	(A/T)
CakSNP8394	*Kabuli*	*Ca_Kabuli_Chr05*	30035763	(A/T)
CakSNP8395	*Kabuli*	*Ca_Kabuli_Chr05*	30049265	(G/A)
CakSNP8396	*Kabuli*	*Ca_Kabuli_Chr05*	30053540	(C/G)
CakSNP8397	*Kabuli*	*Ca_Kabuli_Chr05*	30060160	(C/T)
CakSNP8398	*Kabuli*	*Ca_Kabuli_Chr05*	30086073	(A/G)
CakSNP8399	*Kabuli*	*Ca_Kabuli_Chr05*	30086232	(A/C)
CakSNP8400	*Kabuli*	*Ca_Kabuli_Chr05*	30090906	(A/G)
CakSNP8401	*Kabuli*	*Ca_Kabuli_Chr05*	30091063	(G/A)
CakSNP8402	*Kabuli*	*Ca_Kabuli_Chr05*	30116978	(C/T)
CakSNP8403	*Kabuli*	*Ca_Kabuli_Chr05*	30116992	(T/G)
CakSNP8404	*Kabuli*	*Ca_Kabuli_Chr05*	30159226	(C/T)

SNP IDs	Cultivars	Chromosomes/scaffolds	Physical positions (bp)	SNPs
CakSNP8405	*Kabuli*	*Ca_Kabuli_Chr05*	30159173	(G/A)
CakSNP8406	*Kabuli*	*Ca_Kabuli_Chr05*	30159161	(G/A)
CakSNP8407	*Kabuli*	*Ca_Kabuli_Chr05*	30191389	(A/C)
CakSNP8408	*Kabuli*	*Ca_Kabuli_Chr05*	30248447	(C/A)
CakSNP8409	*Kabuli*	*Ca_Kabuli_Chr05*	30258178	(T/G)
CakSNP8410	*Kabuli*	*Ca_Kabuli_Chr05*	30287853	(C/T)
CakSNP8411	*Kabuli*	*Ca_Kabuli_Chr05*	30304076	(A/T)
CakSNP8412	*Kabuli*	*Ca_Kabuli_Chr05*	30319316	(A/G)
CakSNP8413	*Kabuli*	*Ca_Kabuli_Chr05*	30359047	(T/C)
CakSNP8414	*Kabuli*	*Ca_Kabuli_Chr05*	30359022	(G/A)
CakSNP8415	*Kabuli*	*Ca_Kabuli_Chr05*	30360185	(T/C)
CakSNP8416	*Kabuli*	*Ca_Kabuli_Chr05*	30360348	(T/C)
CakSNP8417	*Kabuli*	*Ca_Kabuli_Chr05*	30367349	(G/T)
CakSNP8418	*Kabuli*	*Ca_Kabuli_Chr05*	30373542	(A/G)
CakSNP8419	*Kabuli*	*Ca_Kabuli_Chr05*	30373783	(T/A)
CakSNP8420	*Kabuli*	*Ca_Kabuli_Chr05*	30376661	(C/G)
CakSNP8421	*Kabuli*	*Ca_Kabuli_Chr05*	30376722	(T/C)
CakSNP8422	*Kabuli*	*Ca_Kabuli_Chr05*	30376723	(T/C)
CakSNP8423	*Kabuli*	*Ca_Kabuli_Chr05*	30378114	(A/G)
CakSNP8424	*Kabuli*	*Ca_Kabuli_Chr05*	30378184	(A/G)
CakSNP8425	*Kabuli*	*Ca_Kabuli_Chr05*	30407725	(C/G)
CakSNP8426	*Kabuli*	*Ca_Kabuli_Chr05*	30408006	(C/T)
CakSNP8427	*Kabuli*	*Ca_Kabuli_Chr05*	30438913	(G/A)
CakSNP8428	*Kabuli*	*Ca_Kabuli_Chr05*	30443858	(A/T)
CakSNP8429	*Kabuli*	*Ca_Kabuli_Chr05*	30562430	(C/G)
CakSNP8430	*Kabuli*	*Ca_Kabuli_Chr05*	30562524	(G/T)
CakSNP8431	*Kabuli*	*Ca_Kabuli_Chr05*	30601932	(T/C)
CakSNP8432	*Kabuli*	*Ca_Kabuli_Chr05*	30601926	(T/C)
CakSNP8433	*Kabuli*	*Ca_Kabuli_Chr05*	30601920	(T/C)
CakSNP8434	*Kabuli*	*Ca_Kabuli_Chr05*	30627937	(G/T)
CakSNP8435	*Kabuli*	*Ca_Kabuli_Chr05*	30632697	(A/G)
CakSNP8436	*Kabuli*	*Ca_Kabuli_Chr05*	30655941	(T/C)
CakSNP8437	*Kabuli*	*Ca_Kabuli_Chr05*	30656058	(A/T)
CakSNP8438	*Kabuli*	*Ca_Kabuli_Chr05*	30690891	(T/G)
CakSNP8439	*Kabuli*	*Ca_Kabuli_Chr05*	30690852	(G/A)
CakSNP8440	*Kabuli*	*Ca_Kabuli_Chr05*	30690885	(A/G)
CakSNP8441	*Kabuli*	*Ca_Kabuli_Chr05*	30691510	(A/G)
CakSNP8442	*Kabuli*	*Ca_Kabuli_Chr05*	30691515	(G/A)
CakSNP8443	*Kabuli*	*Ca_Kabuli_Chr05*	30691543	(A/G)
CakSNP8444	*Kabuli*	*Ca_Kabuli_Chr05*	30697511	(G/A)
CakSNP8445	*Kabuli*	*Ca_Kabuli_Chr05*	30745571	(G/T)

SNP IDs	Cultivars	Chromosomes/scaffolds	Physical positions (bp)	SNPs
CakSNP8446	*Kabuli*	*Ca_Kabuli_Chr05*	30749553	(A/T)
CakSNP8447	*Kabuli*	*Ca_Kabuli_Chr05*	30749696	(A/T)
CakSNP8448	*Kabuli*	*Ca_Kabuli_Chr05*	30755644	(A/G)
CakSNP8449	*Kabuli*	*Ca_Kabuli_Chr05*	30755584	(G/A)
CakSNP8450	*Kabuli*	*Ca_Kabuli_Chr05*	30811250	(A/T)
CakSNP8451	*Kabuli*	*Ca_Kabuli_Chr05*	30864010	(T/C)
CakSNP8452	*Kabuli*	*Ca_Kabuli_Chr05*	30868770	(G/C)
CakSNP8453	*Kabuli*	*Ca_Kabuli_Chr05*	30868695	(G/C)
CakSNP8454	*Kabuli*	*Ca_Kabuli_Chr05*	30878047	(C/T)
CakSNP8455	*Kabuli*	*Ca_Kabuli_Chr05*	30878092	(G/T)
CakSNP8456	*Kabuli*	*Ca_Kabuli_Chr05*	30878100	(T/A)
CakSNP8457	*Kabuli*	*Ca_Kabuli_Chr05*	30890480	(G/C)
CakSNP8458	*Kabuli*	*Ca_Kabuli_Chr05*	30962640	(A/T)
CakSNP8459	*Kabuli*	*Ca_Kabuli_Chr05*	30962641	(C/A)
CakSNP8460	*Kabuli*	*Ca_Kabuli_Chr05*	30962650	(G/A)
CakSNP8461	*Kabuli*	*Ca_Kabuli_Chr05*	30964628	(A/G)
CakSNP8462	*Kabuli*	*Ca_Kabuli_Chr05*	31024408	(T/C)
CakSNP8463	*Kabuli*	*Ca_Kabuli_Chr05*	31025764	(G/T)
CakSNP8464	*Kabuli*	*Ca_Kabuli_Chr05*	31034500	(T/C)
CakSNP8465	*Kabuli*	*Ca_Kabuli_Chr05*	31034898	(A/G)
CakSNP8466	*Kabuli*	*Ca_Kabuli_Chr05*	31064173	(C/G)
CakSNP8467	*Kabuli*	*Ca_Kabuli_Chr05*	31072213	(T/C)
CakSNP8468	*Kabuli*	*Ca_Kabuli_Chr05*	31072276	(G/A)
CakSNP8469	*Kabuli*	*Ca_Kabuli_Chr05*	31084839	(A/C)
CakSNP8470	*Kabuli*	*Ca_Kabuli_Chr05*	31088698	(T/G)
CakSNP8471	*Kabuli*	*Ca_Kabuli_Chr05*	31126125	(C/T)
CakSNP8472	*Kabuli*	*Ca_Kabuli_Chr05*	31131830	(T/C)
CakSNP8473	*Kabuli*	*Ca_Kabuli_Chr05*	31132568	(A/T)
CakSNP8474	*Kabuli*	*Ca_Kabuli_Chr05*	31138650	(G/A)
CakSNP8475	*Kabuli*	*Ca_Kabuli_Chr05*	31140531	(T/C)
CakSNP8476	*Kabuli*	*Ca_Kabuli_Chr05*	31162533	(G/A)
CakSNP8477	*Kabuli*	*Ca_Kabuli_Chr05*	31164387	(G/C)
CakSNP8478	*Kabuli*	*Ca_Kabuli_Chr05*	31164539	(T/C)
CakSNP8479	*Kabuli*	*Ca_Kabuli_Chr05*	31165048	(T/C)
CakSNP8480	*Kabuli*	*Ca_Kabuli_Chr05*	31165039	(T/C)
CakSNP8481	*Kabuli*	*Ca_Kabuli_Chr05*	31165035	(T/C)
CakSNP8482	*Kabuli*	*Ca_Kabuli_Chr05*	31165002	(A/C)
CakSNP8483	*Kabuli*	*Ca_Kabuli_Chr05*	31171555	(G/A)
CakSNP8484	*Kabuli*	*Ca_Kabuli_Chr05*	31176722	(T/G)
CakSNP8485	*Kabuli*	*Ca_Kabuli_Chr05*	31176810	(C/T)
CakSNP8486	*Kabuli*	*Ca_Kabuli_Chr05*	31176844	(G/A)

SNP IDs	Cultivars	Chromosomes/scaffolds	Physical positions (bp)	SNPs
CakSNP8487	*Kabuli*	*Ca_Kabuli_Chr05*	31192415	(C/T)
CakSNP8488	*Kabuli*	*Ca_Kabuli_Chr05*	31192413	(G/A)
CakSNP8489	*Kabuli*	*Ca_Kabuli_Chr05*	31194845	(G/T)
CakSNP8490	*Kabuli*	*Ca_Kabuli_Chr05*	31194974	(T/C)
CakSNP8491	*Kabuli*	*Ca_Kabuli_Chr05*	31194988	(G/T)
CakSNP8492	*Kabuli*	*Ca_Kabuli_Chr05*	31194989	(A/C)
CakSNP8493	*Kabuli*	*Ca_Kabuli_Chr05*	31215109	(C/T)
CakSNP8494	*Kabuli*	*Ca_Kabuli_Chr05*	31310698	(C/T)
CakSNP8495	*Kabuli*	*Ca_Kabuli_Chr05*	31310702	(C/G)
CakSNP8496	*Kabuli*	*Ca_Kabuli_Chr05*	31310703	(A/G)
CakSNP8497	*Kabuli*	*Ca_Kabuli_Chr05*	31310741	(A/G)
CakSNP8498	*Kabuli*	*Ca_Kabuli_Chr05*	31314012	(A/C)
CakSNP8499	*Kabuli*	*Ca_Kabuli_Chr05*	31314056	(A/C)
CakSNP8500	*Kabuli*	*Ca_Kabuli_Chr05*	31344725	(G/A)
CakSNP8501	*Kabuli*	*Ca_Kabuli_Chr05*	31377103	(C/T)
CakSNP8502	*Kabuli*	*Ca_Kabuli_Chr05*	31377122	(C/T)
CakSNP8503	*Kabuli*	*Ca_Kabuli_Chr05*	31377134	(A/T)
CakSNP8504	*Kabuli*	*Ca_Kabuli_Chr05*	31390221	(A/G)
CakSNP8505	*Kabuli*	*Ca_Kabuli_Chr05*	31390319	(C/T)
CakSNP8506	*Kabuli*	*Ca_Kabuli_Chr05*	31522389	(T/G)
CakSNP8507	*Kabuli*	*Ca_Kabuli_Chr05*	31548555	(G/A)
CakSNP8508	*Kabuli*	*Ca_Kabuli_Chr05*	31651335	(G/T)
CakSNP8509	*Kabuli*	*Ca_Kabuli_Chr05*	31651336	(T/G)
CakSNP8510	*Kabuli*	*Ca_Kabuli_Chr05*	31653418	(T/C)
CakSNP8511	*Kabuli*	*Ca_Kabuli_Chr05*	31653423	(T/C)
CakSNP8512	*Kabuli*	*Ca_Kabuli_Chr05*	31653432	(A/G)
CakSNP8513	*Kabuli*	*Ca_Kabuli_Chr05*	31653437	(C/T)
CakSNP8514	*Kabuli*	*Ca_Kabuli_Chr05*	31653452	(C/A)
CakSNP8515	*Kabuli*	*Ca_Kabuli_Chr05*	31687881	(C/T)
CakSNP8516	*Kabuli*	*Ca_Kabuli_Chr05*	31687891	(A/C)
CakSNP8517	*Kabuli*	*Ca_Kabuli_Chr05*	31696236	(T/C)
CakSNP8518	*Kabuli*	*Ca_Kabuli_Chr05*	31710670	(G/A)
CakSNP8519	*Kabuli*	*Ca_Kabuli_Chr05*	31724217	(G/A)
CakSNP8520	*Kabuli*	*Ca_Kabuli_Chr05*	31724272	(C/T)
CakSNP8521	*Kabuli*	*Ca_Kabuli_Chr05*	31724332	(G/A)
CakSNP8522	*Kabuli*	*Ca_Kabuli_Chr05*	31750150	(T/G)
CakSNP8523	*Kabuli*	*Ca_Kabuli_Chr05*	31750242	(A/G)
CakSNP8524	*Kabuli*	*Ca_Kabuli_Chr05*	31758166	(T/C)
CakSNP8525	*Kabuli*	*Ca_Kabuli_Chr05*	31802684	(G/T)
CakSNP8526	*Kabuli*	*Ca_Kabuli_Chr05*	31802727	(G/A)
CakSNP8527	*Kabuli*	*Ca_Kabuli_Chr05*	31816202	(A/C)

SNP IDs	Cultivars	Chromosomes/scaffolds	Physical positions (bp)	SNPs
CakSNP8528	*Kabuli*	*Ca_Kabuli_Chr05*	31816228	(A/G)
CakSNP8529	*Kabuli*	*Ca_Kabuli_Chr05*	31935674	(T/C)
CakSNP8530	*Kabuli*	*Ca_Kabuli_Chr05*	31951925	(G/A)
CakSNP8531	*Kabuli*	*Ca_Kabuli_Chr05*	31951944	(C/T)
CakSNP8532	*Kabuli*	*Ca_Kabuli_Chr05*	31985390	(G/C)
CakSNP8533	*Kabuli*	*Ca_Kabuli_Chr05*	32011781	(G/T)
CakSNP8534	*Kabuli*	*Ca_Kabuli_Chr05*	32011792	(T/G)
CakSNP8535	*Kabuli*	*Ca_Kabuli_Chr05*	32015967	(T/C)
CakSNP8536	*Kabuli*	*Ca_Kabuli_Chr05*	32019864	(G/A)
CakSNP8537	*Kabuli*	*Ca_Kabuli_Chr05*	32023925	(T/C)
CakSNP8538	*Kabuli*	*Ca_Kabuli_Chr05*	32090295	(C/A)
CakSNP8539	*Kabuli*	*Ca_Kabuli_Chr05*	32144081	(G/C)
CakSNP8540	*Kabuli*	*Ca_Kabuli_Chr05*	32144108	(A/C)
CakSNP8541	*Kabuli*	*Ca_Kabuli_Chr05*	32144120	(C/T)
CakSNP8542	*Kabuli*	*Ca_Kabuli_Chr05*	32147522	(C/T)
CakSNP8543	*Kabuli*	*Ca_Kabuli_Chr05*	32171632	(G/A)
CakSNP8544	*Kabuli*	*Ca_Kabuli_Chr05*	32171652	(T/A)
CakSNP8545	*Kabuli*	*Ca_Kabuli_Chr05*	32209547	(C/G)
CakSNP8546	*Kabuli*	*Ca_Kabuli_Chr05*	32209750	(T/C)
CakSNP8547	*Kabuli*	*Ca_Kabuli_Chr05*	32229485	(A/T)
CakSNP8548	*Kabuli*	*Ca_Kabuli_Chr05*	32291158	(C/T)
CakSNP8549	*Kabuli*	*Ca_Kabuli_Chr05*	32291118	(G/A)
CakSNP8550	*Kabuli*	*Ca_Kabuli_Chr05*	32328110	(T/C)
CakSNP8551	*Kabuli*	*Ca_Kabuli_Chr05*	32346655	(A/C)
CakSNP8552	*Kabuli*	*Ca_Kabuli_Chr05*	32357511	(G/A)
CakSNP8553	*Kabuli*	*Ca_Kabuli_Chr05*	32368485	(G/C)
CakSNP8554	*Kabuli*	*Ca_Kabuli_Chr05*	32369197	(C/A)
CakSNP8555	*Kabuli*	*Ca_Kabuli_Chr05*	32399391	(T/A)
CakSNP8556	*Kabuli*	*Ca_Kabuli_Chr05*	32476432	(A/G)
CakSNP8557	*Kabuli*	*Ca_Kabuli_Chr05*	32476423	(A/G)
CakSNP8558	*Kabuli*	*Ca_Kabuli_Chr05*	32516773	(T/A)
CakSNP8559	*Kabuli*	*Ca_Kabuli_Chr05*	32524646	(T/C)
CakSNP8560	*Kabuli*	*Ca_Kabuli_Chr05*	32566131	(C/G)
CakSNP8561	*Kabuli*	*Ca_Kabuli_Chr05*	32598221	(C/A)
CakSNP8562	*Kabuli*	*Ca_Kabuli_Chr05*	32623507	(T/G)
CakSNP8563	*Kabuli*	*Ca_Kabuli_Chr05*	32631930	(C/A)
CakSNP8564	*Kabuli*	*Ca_Kabuli_Chr05*	32643759	(A/G)
CakSNP8565	*Kabuli*	*Ca_Kabuli_Chr05*	32732862	(A/T)
CakSNP8566	*Kabuli*	*Ca_Kabuli_Chr05*	32732913	(A/T)
CakSNP8567	*Kabuli*	*Ca_Kabuli_Chr05*	32759420	(T/A)
CakSNP8568	*Kabuli*	*Ca_Kabuli_Chr05*	32761420	(G/A)

SNP IDs	Cultivars	Chromosomes/scaffolds	Physical positions (bp)	SNPs
CakSNP8569	*Kabuli*	*Ca_Kabuli_Chr05*	32794288	(G/A)
CakSNP8570	*Kabuli*	*Ca_Kabuli_Chr05*	32816626	(G/T)
CakSNP8571	*Kabuli*	*Ca_Kabuli_Chr05*	32839347	(C/A)
CakSNP8572	*Kabuli*	*Ca_Kabuli_Chr05*	32840512	(A/T)
CakSNP8573	*Kabuli*	*Ca_Kabuli_Chr05*	32840522	(T/C)
CakSNP8574	*Kabuli*	*Ca_Kabuli_Chr05*	32840541	(T/G)
CakSNP8575	*Kabuli*	*Ca_Kabuli_Chr05*	32859483	(T/A)
CakSNP8576	*Kabuli*	*Ca_Kabuli_Chr05*	32859504	(T/G)
CakSNP8577	*Kabuli*	*Ca_Kabuli_Chr05*	32859515	(T/G)
CakSNP8578	*Kabuli*	*Ca_Kabuli_Chr05*	32860087	(C/T)
CakSNP8579	*Kabuli*	*Ca_Kabuli_Chr05*	32978975	(A/T)
CakSNP8580	*Kabuli*	*Ca_Kabuli_Chr05*	33052329	(C/G)
CakSNP8581	*Kabuli*	*Ca_Kabuli_Chr05*	33143038	(A/T)
CakSNP8582	*Kabuli*	*Ca_Kabuli_Chr05*	33189733	(A/G)
CakSNP8583	*Kabuli*	*Ca_Kabuli_Chr05*	33189791	(G/A)
CakSNP8584	*Kabuli*	*Ca_Kabuli_Chr05*	33189808	(A/G)
CakSNP8585	*Kabuli*	*Ca_Kabuli_Chr05*	33255823	(T/G)
CakSNP8586	*Kabuli*	*Ca_Kabuli_Chr05*	33255971	(G/T)
CakSNP8587	*Kabuli*	*Ca_Kabuli_Chr05*	33256053	(C/G)
CakSNP8588	*Kabuli*	*Ca_Kabuli_Chr05*	33256089	(T/C)
CakSNP8589	*Kabuli*	*Ca_Kabuli_Chr05*	33256245	(T/C)
CakSNP8590	*Kabuli*	*Ca_Kabuli_Chr05*	33256412	(A/T)
CakSNP8591	*Kabuli*	*Ca_Kabuli_Chr05*	33256388	(T/A)
CakSNP8592	*Kabuli*	*Ca_Kabuli_Chr05*	33336127	(C/G)
CakSNP8593	*Kabuli*	*Ca_Kabuli_Chr05*	33336135	(G/T)
CakSNP8594	*Kabuli*	*Ca_Kabuli_Chr05*	33349598	(C/T)
CakSNP8595	*Kabuli*	*Ca_Kabuli_Chr05*	33349700	(A/G)
CakSNP8596	*Kabuli*	*Ca_Kabuli_Chr05*	33379904	(A/G)
CakSNP8597	*Kabuli*	*Ca_Kabuli_Chr05*	33379873	(C/T)
CakSNP8598	*Kabuli*	*Ca_Kabuli_Chr05*	33381774	(C/T)
CakSNP8599	*Kabuli*	*Ca_Kabuli_Chr05*	33422813	(C/T)
CakSNP8600	*Kabuli*	*Ca_Kabuli_Chr05*	33422780	(A/G)
CakSNP8601	*Kabuli*	*Ca_Kabuli_Chr05*	33422779	(C/T)
CakSNP8602	*Kabuli*	*Ca_Kabuli_Chr05*	33422767	(T/C)
CakSNP8603	*Kabuli*	*Ca_Kabuli_Chr05*	33431897	(T/C)
CakSNP8604	*Kabuli*	*Ca_Kabuli_Chr05*	33431996	(T/C)
CakSNP8605	*Kabuli*	*Ca_Kabuli_Chr05*	33523676	(T/C)
CakSNP8606	*Kabuli*	*Ca_Kabuli_Chr05*	33523686	(G/A)
CakSNP8607	*Kabuli*	*Ca_Kabuli_Chr05*	33523691	(T/C)
CakSNP8608	*Kabuli*	*Ca_Kabuli_Chr05*	33532807	(A/G)
CakSNP8609	*Kabuli*	*Ca_Kabuli_Chr05*	33532804	(A/T)

SNP IDs	Cultivars	Chromosomes/scaffolds	Physical positions (bp)	SNPs
CakSNP8610	*Kabuli*	*Ca_Kabuli_Chr05*	33532798	(A/T)
CakSNP8611	*Kabuli*	*Ca_Kabuli_Chr05*	33574726	(T/C)
CakSNP8612	*Kabuli*	*Ca_Kabuli_Chr05*	33578061	(T/A)
CakSNP8613	*Kabuli*	*Ca_Kabuli_Chr05*	33578167	(A/T)
CakSNP8614	*Kabuli*	*Ca_Kabuli_Chr05*	33578438	(T/C)
CakSNP8615	*Kabuli*	*Ca_Kabuli_Chr05*	33578477	(A/G)
CakSNP8616	*Kabuli*	*Ca_Kabuli_Chr05*	33581040	(A/G)
CakSNP8617	*Kabuli*	*Ca_Kabuli_Chr05*	33626841	(C/T)
CakSNP8618	*Kabuli*	*Ca_Kabuli_Chr05*	33627132	(T/C)
CakSNP8619	*Kabuli*	*Ca_Kabuli_Chr05*	33631108	(A/G)
CakSNP8620	*Kabuli*	*Ca_Kabuli_Chr05*	33631266	(T/A)
CakSNP8621	*Kabuli*	*Ca_Kabuli_Chr05*	33656560	(C/G)
CakSNP8622	*Kabuli*	*Ca_Kabuli_Chr05*	33673894	(T/C)
CakSNP8623	*Kabuli*	*Ca_Kabuli_Chr05*	33700181	(G/C)
CakSNP8624	*Kabuli*	*Ca_Kabuli_Chr05*	33700196	(A/T)
CakSNP8625	*Kabuli*	*Ca_Kabuli_Chr05*	33721487	(C/T)
CakSNP8626	*Kabuli*	*Ca_Kabuli_Chr05*	33721544	(A/G)
CakSNP8627	*Kabuli*	*Ca_Kabuli_Chr05*	33771359	(T/C)
CakSNP8628	*Kabuli*	*Ca_Kabuli_Chr05*	33812108	(T/C)
CakSNP8629	*Kabuli*	*Ca_Kabuli_Chr05*	33812213	(A/C)
CakSNP8630	*Kabuli*	*Ca_Kabuli_Chr05*	33812363	(T/A)
CakSNP8631	*Kabuli*	*Ca_Kabuli_Chr05*	33818742	(T/A)
CakSNP8632	*Kabuli*	*Ca_Kabuli_Chr05*	33818789	(C/G)
CakSNP8633	*Kabuli*	*Ca_Kabuli_Chr05*	33818823	(A/G)
CakSNP8634	*Kabuli*	*Ca_Kabuli_Chr05*	33824516	(G/T)
CakSNP8635	*Kabuli*	*Ca_Kabuli_Chr05*	33824513	(G/A)
CakSNP8636	*Kabuli*	*Ca_Kabuli_Chr05*	33824503	(G/A)
CakSNP8637	*Kabuli*	*Ca_Kabuli_Chr05*	33835816	(T/C)
CakSNP8638	*Kabuli*	*Ca_Kabuli_Chr05*	33841544	(T/G)
CakSNP8639	*Kabuli*	*Ca_Kabuli_Chr05*	33844056	(A/T)
CakSNP8640	*Kabuli*	*Ca_Kabuli_Chr05*	33844059	(G/C)
CakSNP8641	*Kabuli*	*Ca_Kabuli_Chr05*	33844222	(T/C)
CakSNP8642	*Kabuli*	*Ca_Kabuli_Chr05*	33844436	(T/A)
CakSNP8643	*Kabuli*	*Ca_Kabuli_Chr05*	33892596	(A/C)
CakSNP8644	*Kabuli*	*Ca_Kabuli_Chr05*	33937307	(G/A)
CakSNP8645	*Kabuli*	*Ca_Kabuli_Chr05*	33937316	(G/A)
CakSNP8646	*Kabuli*	*Ca_Kabuli_Chr05*	33963525	(G/C)
CakSNP8647	*Kabuli*	*Ca_Kabuli_Chr05*	33985819	(A/G)
CakSNP8648	*Kabuli*	*Ca_Kabuli_Chr05*	33986234	(C/T)
CakSNP8649	*Kabuli*	*Ca_Kabuli_Chr05*	33986253	(A/C)
CakSNP8650	*Kabuli*	*Ca_Kabuli_Chr05*	34022362	(G/T)

SNP IDs	Cultivars	Chromosomes/scaffolds	Physical positions (bp)	SNPs
CakSNP8651	*Kabuli*	*Ca_Kabuli_Chr05*	34040479	(A/G)
CakSNP8652	*Kabuli*	*Ca_Kabuli_Chr05*	34040508	(A/C)
CakSNP8653	*Kabuli*	*Ca_Kabuli_Chr05*	34040535	(G/A)
CakSNP8654	*Kabuli*	*Ca_Kabuli_Chr05*	34041705	(G/T)
CakSNP8655	*Kabuli*	*Ca_Kabuli_Chr05*	34073378	(A/T)
CakSNP8656	*Kabuli*	*Ca_Kabuli_Chr05*	34073395	(G/A)
CakSNP8657	*Kabuli*	*Ca_Kabuli_Chr05*	34073445	(A/T)
CakSNP8658	*Kabuli*	*Ca_Kabuli_Chr05*	34201989	(C/G)
CakSNP8659	*Kabuli*	*Ca_Kabuli_Chr05*	34202000	(T/C)
CakSNP8660	*Kabuli*	*Ca_Kabuli_Chr05*	34202075	(T/G)
CakSNP8661	*Kabuli*	*Ca_Kabuli_Chr05*	34202222	(G/A)
CakSNP8662	*Kabuli*	*Ca_Kabuli_Chr05*	34241269	(C/G)
CakSNP8663	*Kabuli*	*Ca_Kabuli_Chr05*	34244499	(C/T)
CakSNP8664	*Kabuli*	*Ca_Kabuli_Chr05*	34244681	(G/A)
CakSNP8665	*Kabuli*	*Ca_Kabuli_Chr05*	34244670	(A/G)
CakSNP8666	*Kabuli*	*Ca_Kabuli_Chr05*	34244662	(C/G)
CakSNP8667	*Kabuli*	*Ca_Kabuli_Chr05*	34244754	(T/A)
CakSNP8668	*Kabuli*	*Ca_Kabuli_Chr05*	34313779	(A/G)
CakSNP8669	*Kabuli*	*Ca_Kabuli_Chr05*	34313758	(T/G)
CakSNP8670	*Kabuli*	*Ca_Kabuli_Chr05*	34334980	(A/G)
CakSNP8671	*Kabuli*	*Ca_Kabuli_Chr05*	34334993	(A/C)
CakSNP8672	*Kabuli*	*Ca_Kabuli_Chr05*	34335252	(T/A)
CakSNP8673	*Kabuli*	*Ca_Kabuli_Chr05*	34418798	(G/C)
CakSNP8674	*Kabuli*	*Ca_Kabuli_Chr05*	34441581	(A/C)
CakSNP8675	*Kabuli*	*Ca_Kabuli_Chr05*	34461159	(T/C)
CakSNP8676	*Kabuli*	*Ca_Kabuli_Chr05*	34482245	(G/T)
CakSNP8677	*Kabuli*	*Ca_Kabuli_Chr05*	34482217	(G/T)
CakSNP8678	*Kabuli*	*Ca_Kabuli_Chr05*	34511669	(G/T)
CakSNP8679	*Kabuli*	*Ca_Kabuli_Chr05*	34511678	(T/C)
CakSNP8680	*Kabuli*	*Ca_Kabuli_Chr05*	34511693	(C/T)
CakSNP8681	*Kabuli*	*Ca_Kabuli_Chr05*	34511705	(G/T)
CakSNP8682	*Kabuli*	*Ca_Kabuli_Chr05*	34521655	(T/C)
CakSNP8683	*Kabuli*	*Ca_Kabuli_Chr05*	34531222	(A/G)
CakSNP8684	*Kabuli*	*Ca_Kabuli_Chr05*	34535758	(T/C)
CakSNP8685	*Kabuli*	*Ca_Kabuli_Chr05*	34535893	(G/T)
CakSNP8686	*Kabuli*	*Ca_Kabuli_Chr05*	34535878	(G/A)
CakSNP8687	*Kabuli*	*Ca_Kabuli_Chr05*	34629028	(A/G)
CakSNP8688	*Kabuli*	*Ca_Kabuli_Chr05*	34673784	(T/A)
CakSNP8689	*Kabuli*	*Ca_Kabuli_Chr05*	34691234	(A/C)
CakSNP8690	*Kabuli*	*Ca_Kabuli_Chr05*	34691302	(T/C)
CakSNP8691	*Kabuli*	*Ca_Kabuli_Chr05*	34691453	(G/A)

SNP IDs	Cultivars	Chromosomes/scaffolds	Physical positions (bp)	SNPs
CakSNP8692	*Kabuli*	*Ca_Kabuli_Chr05*	34807071	(C/T)
CakSNP8693	*Kabuli*	*Ca_Kabuli_Chr05*	34814216	(T/C)
CakSNP8694	*Kabuli*	*Ca_Kabuli_Chr05*	34824647	(C/G)
CakSNP8695	*Kabuli*	*Ca_Kabuli_Chr05*	34824749	(A/T)
CakSNP8696	*Kabuli*	*Ca_Kabuli_Chr05*	34845398	(T/G)
CakSNP8697	*Kabuli*	*Ca_Kabuli_Chr05*	34879731	(A/G)
CakSNP8698	*Kabuli*	*Ca_Kabuli_Chr05*	34974615	(T/A)
CakSNP8699	*Kabuli*	*Ca_Kabuli_Chr05*	34974703	(C/T)
CakSNP8700	*Kabuli*	*Ca_Kabuli_Chr05*	34974795	(A/G)
CakSNP8701	*Kabuli*	*Ca_Kabuli_Chr05*	34975712	(T/A)
CakSNP8702	*Kabuli*	*Ca_Kabuli_Chr05*	34998725	(G/A)
CakSNP8703	*Kabuli*	*Ca_Kabuli_Chr05*	35126544	(A/C)
CakSNP8704	*Kabuli*	*Ca_Kabuli_Chr05*	35164295	(C/T)
CakSNP8705	*Kabuli*	*Ca_Kabuli_Chr05*	35191371	(C/G)
CakSNP8706	*Kabuli*	*Ca_Kabuli_Chr05*	35209511	(T/C)
CakSNP8707	*Kabuli*	*Ca_Kabuli_Chr05*	35213601	(C/T)
CakSNP8708	*Kabuli*	*Ca_Kabuli_Chr05*	35226049	(G/C)
CakSNP8709	*Kabuli*	*Ca_Kabuli_Chr05*	35290452	(A/G)
CakSNP8710	*Kabuli*	*Ca_Kabuli_Chr05*	35296135	(A/C)
CakSNP8711	*Kabuli*	*Ca_Kabuli_Chr05*	35330946	(G/A)
CakSNP8712	*Kabuli*	*Ca_Kabuli_Chr05*	35330995	(A/C)
CakSNP8713	*Kabuli*	*Ca_Kabuli_Chr05*	35340068	(A/G)
CakSNP8714	*Kabuli*	*Ca_Kabuli_Chr05*	35340065	(G/A)
CakSNP8715	*Kabuli*	*Ca_Kabuli_Chr05*	35374184	(G/A)
CakSNP8716	*Kabuli*	*Ca_Kabuli_Chr05*	35438327	(G/T)
CakSNP8717	*Kabuli*	*Ca_Kabuli_Chr05*	35495900	(T/A)
CakSNP8718	*Kabuli*	*Ca_Kabuli_Chr05*	35500751	(T/C)
CakSNP8719	*Kabuli*	*Ca_Kabuli_Chr05*	35534923	(T/A)
CakSNP8720	*Kabuli*	*Ca_Kabuli_Chr05*	35534897	(G/A)
CakSNP8721	*Kabuli*	*Ca_Kabuli_Chr05*	35540372	(T/C)
CakSNP8722	*Kabuli*	*Ca_Kabuli_Chr05*	35543648	(T/C)
CakSNP8723	*Kabuli*	*Ca_Kabuli_Chr05*	35554534	(C/A)
CakSNP8724	*Kabuli*	*Ca_Kabuli_Chr05*	35554593	(C/T)
CakSNP8725	*Kabuli*	*Ca_Kabuli_Chr05*	35555184	(A/G)
CakSNP8726	*Kabuli*	*Ca_Kabuli_Chr05*	35576495	(A/G)
CakSNP8727	*Kabuli*	*Ca_Kabuli_Chr05*	35617798	(A/C)
CakSNP8728	*Kabuli*	*Ca_Kabuli_Chr05*	35688558	(T/C)
CakSNP8729	*Kabuli*	*Ca_Kabuli_Chr05*	35690996	(G/A)
CakSNP8730	*Kabuli*	*Ca_Kabuli_Chr05*	35691581	(T/C)
CakSNP8731	*Kabuli*	*Ca_Kabuli_Chr05*	35715495	(C/T)
CakSNP8732	*Kabuli*	*Ca_Kabuli_Chr05*	35715550	(G/C)

SNP IDs	Cultivars	Chromosomes/scaffolds	Physical positions (bp)	SNPs
CakSNP8733	*Kabuli*	*Ca_Kabuli_Chr05*	35715571	(A/C)
CakSNP8734	*Kabuli*	*Ca_Kabuli_Chr05*	35715563	(G/A)
CakSNP8735	*Kabuli*	*Ca_Kabuli_Chr05*	35715548	(T/C)
CakSNP8736	*Kabuli*	*Ca_Kabuli_Chr05*	35773040	(C/A)
CakSNP8737	*Kabuli*	*Ca_Kabuli_Chr05*	35773044	(A/G)
CakSNP8738	*Kabuli*	*Ca_Kabuli_Chr05*	35775200	(T/C)
CakSNP8739	*Kabuli*	*Ca_Kabuli_Chr05*	35775191	(T/G)
CakSNP8740	*Kabuli*	*Ca_Kabuli_Chr05*	35775266	(G/A)
CakSNP8741	*Kabuli*	*Ca_Kabuli_Chr05*	35789373	(T/A)
CakSNP8742	*Kabuli*	*Ca_Kabuli_Chr05*	35789775	(A/G)
CakSNP8743	*Kabuli*	*Ca_Kabuli_Chr05*	35839230	(T/C)
CakSNP8744	*Kabuli*	*Ca_Kabuli_Chr05*	35839233	(G/A)
CakSNP8745	*Kabuli*	*Ca_Kabuli_Chr05*	35844295	(G/T)
CakSNP8746	*Kabuli*	*Ca_Kabuli_Chr05*	35845094	(G/A)
CakSNP8747	*Kabuli*	*Ca_Kabuli_Chr05*	35845780	(T/C)
CakSNP8748	*Kabuli*	*Ca_Kabuli_Chr05*	35845801	(T/C)
CakSNP8749	*Kabuli*	*Ca_Kabuli_Chr05*	35846404	(A/G)
CakSNP8750	*Kabuli*	*Ca_Kabuli_Chr05*	35868865	(G/A)
CakSNP8751	*Kabuli*	*Ca_Kabuli_Chr05*	35877505	(T/C)
CakSNP8752	*Kabuli*	*Ca_Kabuli_Chr05*	35926105	(T/C)
CakSNP8753	*Kabuli*	*Ca_Kabuli_Chr05*	35937904	(C/T)
CakSNP8754	*Kabuli*	*Ca_Kabuli_Chr05*	35937842	(G/T)
CakSNP8755	*Kabuli*	*Ca_Kabuli_Chr05*	35947574	(G/A)
CakSNP8756	*Kabuli*	*Ca_Kabuli_Chr05*	35954656	(T/C)
CakSNP8757	*Kabuli*	*Ca_Kabuli_Chr05*	35956534	(C/T)
CakSNP8758	*Kabuli*	*Ca_Kabuli_Chr05*	36012698	(C/G)
CakSNP8759	*Kabuli*	*Ca_Kabuli_Chr05*	36039365	(A/C)
CakSNP8760	*Kabuli*	*Ca_Kabuli_Chr05*	36128655	(G/T)
CakSNP8761	*Kabuli*	*Ca_Kabuli_Chr05*	36131156	(A/C)
CakSNP8762	*Kabuli*	*Ca_Kabuli_Chr05*	36217803	(C/T)
CakSNP8763	*Kabuli*	*Ca_Kabuli_Chr05*	36238656	(G/A)
CakSNP8764	*Kabuli*	*Ca_Kabuli_Chr05*	36238655	(G/C)
CakSNP8765	*Kabuli*	*Ca_Kabuli_Chr05*	36248568	(C/T)
CakSNP8766	*Kabuli*	*Ca_Kabuli_Chr05*	36292611	(C/G)
CakSNP8767	*Kabuli*	*Ca_Kabuli_Chr05*	36334569	(C/A)
CakSNP8768	*Kabuli*	*Ca_Kabuli_Chr05*	36334592	(C/T)
CakSNP8769	*Kabuli*	*Ca_Kabuli_Chr05*	36334625	(A/G)
CakSNP8770	*Kabuli*	*Ca_Kabuli_Chr05*	36338598	(A/C)
CakSNP8771	*Kabuli*	*Ca_Kabuli_Chr05*	36338658	(A/G)
CakSNP8772	*Kabuli*	*Ca_Kabuli_Chr05*	36397489	(A/C)
CakSNP8773	*Kabuli*	*Ca_Kabuli_Chr05*	36400943	(C/T)

**SNP IDs**	**Cultivars**	**Chromosomes/scaffolds**	**Physical positions (bp)**	**SNPs**
CakSNP8774	*Kabuli*	*Ca_Kabuli_Chr05*	36408701	(G/A)
CakSNP8775	*Kabuli*	*Ca_Kabuli_Chr05*	36408719	(G/A)
CakSNP8776	*Kabuli*	*Ca_Kabuli_Chr05*	36408724	(T/C)
CakSNP8777	*Kabuli*	*Ca_Kabuli_Chr05*	36408736	(C/A)
CakSNP8778	*Kabuli*	*Ca_Kabuli_Chr05*	36430677	(A/C)
CakSNP8779	*Kabuli*	*Ca_Kabuli_Chr05*	36441063	(C/T)
CakSNP8780	*Kabuli*	*Ca_Kabuli_Chr05*	36533996	(T/A)
CakSNP8781	*Kabuli*	*Ca_Kabuli_Chr05*	36534056	(G/C)
CakSNP8782	*Kabuli*	*Ca_Kabuli_Chr05*	36534057	(T/G)
CakSNP8783	*Kabuli*	*Ca_Kabuli_Chr05*	36534060	(G/A)
CakSNP8784	*Kabuli*	*Ca_Kabuli_Chr05*	36534063	(C/T)
CakSNP8785	*Kabuli*	*Ca_Kabuli_Chr05*	36534126	(A/G)
CakSNP8786	*Kabuli*	*Ca_Kabuli_Chr05*	36534110	(T/C)
CakSNP8787	*Kabuli*	*Ca_Kabuli_Chr05*	36566175	(G/A)
CakSNP8788	*Kabuli*	*Ca_Kabuli_Chr05*	36613610	(T/C)
CakSNP8789	*Kabuli*	*Ca_Kabuli_Chr05*	36619837	(A/G)
CakSNP8790	*Kabuli*	*Ca_Kabuli_Chr05*	36621872	(A/T)
CakSNP8791	*Kabuli*	*Ca_Kabuli_Chr05*	36672147	(C/T)
CakSNP8792	*Kabuli*	*Ca_Kabuli_Chr05*	36672164	(A/G)
CakSNP8793	*Kabuli*	*Ca_Kabuli_Chr05*	36673950	(C/A)
CakSNP8794	*Kabuli*	*Ca_Kabuli_Chr05*	36731961	(T/C)
CakSNP8795	*Kabuli*	*Ca_Kabuli_Chr05*	36735459	(A/T)
CakSNP8796	*Kabuli*	*Ca_Kabuli_Chr05*	36751625	(C/T)
CakSNP8797	*Kabuli*	*Ca_Kabuli_Chr05*	36751851	(T/A)
CakSNP8798	*Kabuli*	*Ca_Kabuli_Chr05*	36757408	(G/A)
CakSNP8799	*Kabuli*	*Ca_Kabuli_Chr05*	36797173	(A/G)
CakSNP8800	*Kabuli*	*Ca_Kabuli_Chr05*	36797187	(G/T)
CakSNP8801	*Kabuli*	*Ca_Kabuli_Chr05*	36800497	(G/T)
CakSNP8802	*Kabuli*	*Ca_Kabuli_Chr05*	36842784	(G/T)
CakSNP8803	*Kabuli*	*Ca_Kabuli_Chr05*	36855028	(G/A)
CakSNP8804	*Kabuli*	*Ca_Kabuli_Chr05*	36916560	(T/C)
CakSNP8805	*Kabuli*	*Ca_Kabuli_Chr05*	36999074	(G/C)
CakSNP8806	*Kabuli*	*Ca_Kabuli_Chr05*	37016704	(C/G)
CakSNP8807	*Kabuli*	*Ca_Kabuli_Chr05*	37039246	(A/T)
CakSNP8808	*Kabuli*	*Ca_Kabuli_Chr05*	37039300	(A/G)
CakSNP8809	*Kabuli*	*Ca_Kabuli_Chr05*	37040045	(G/A)
CakSNP8810	*Kabuli*	*Ca_Kabuli_Chr05*	37067293	(A/G)
CakSNP8811	*Kabuli*	*Ca_Kabuli_Chr05*	37067371	(T/C)
CakSNP8812	*Kabuli*	*Ca_Kabuli_Chr05*	37067384	(G/C)
CakSNP8813	*Kabuli*	*Ca_Kabuli_Chr05*	37083276	(T/G)
CakSNP8814	*Kabuli*	*Ca_Kabuli_Chr05*	37083275	(G/T)

SNP IDs	Cultivars	Chromosomes/scaffolds	Physical positions (bp)	SNPs
CakSNP8815	*Kabuli*	*Ca_Kabuli_Chr05*	37114574	(A/G)
CakSNP8816	*Kabuli*	*Ca_Kabuli_Chr05*	37126371	(A/G)
CakSNP8817	*Kabuli*	*Ca_Kabuli_Chr05*	37126354	(T/C)
CakSNP8818	*Kabuli*	*Ca_Kabuli_Chr05*	37147572	(C/T)
CakSNP8819	*Kabuli*	*Ca_Kabuli_Chr05*	37167061	(A/G)
CakSNP8820	*Kabuli*	*Ca_Kabuli_Chr05*	37167114	(A/T)
CakSNP8821	*Kabuli*	*Ca_Kabuli_Chr05*	37167108	(A/T)
CakSNP8822	*Kabuli*	*Ca_Kabuli_Chr05*	37194939	(G/A)
CakSNP8823	*Kabuli*	*Ca_Kabuli_Chr05*	37194917	(G/A)
CakSNP8824	*Kabuli*	*Ca_Kabuli_Chr05*	37197321	(C/A)
CakSNP8825	*Kabuli*	*Ca_Kabuli_Chr05*	37207821	(A/T)
CakSNP8826	*Kabuli*	*Ca_Kabuli_Chr05*	37207837	(G/T)
CakSNP8827	*Kabuli*	*Ca_Kabuli_Chr05*	37211645	(T/G)
CakSNP8828	*Kabuli*	*Ca_Kabuli_Chr05*	37213853	(C/T)
CakSNP8829	*Kabuli*	*Ca_Kabuli_Chr05*	37232307	(T/C)
CakSNP8830	*Kabuli*	*Ca_Kabuli_Chr05*	37254596	(A/G)
CakSNP8831	*Kabuli*	*Ca_Kabuli_Chr05*	37260601	(T/C)
CakSNP8832	*Kabuli*	*Ca_Kabuli_Chr05*	37274572	(C/T)
CakSNP8833	*Kabuli*	*Ca_Kabuli_Chr05*	37274583	(C/T)
CakSNP8834	*Kabuli*	*Ca_Kabuli_Chr05*	37274616	(C/G)
CakSNP8835	*Kabuli*	*Ca_Kabuli_Chr05*	37309917	(T/G)
CakSNP8836	*Kabuli*	*Ca_Kabuli_Chr05*	37309935	(T/G)
CakSNP8837	*Kabuli*	*Ca_Kabuli_Chr05*	37332101	(G/T)
CakSNP8838	*Kabuli*	*Ca_Kabuli_Chr05*	37332198	(G/A)
CakSNP8839	*Kabuli*	*Ca_Kabuli_Chr05*	37332304	(G/A)
CakSNP8840	*Kabuli*	*Ca_Kabuli_Chr05*	37332303	(A/C)
CakSNP8841	*Kabuli*	*Ca_Kabuli_Chr05*	37332370	(T/C)
CakSNP8842	*Kabuli*	*Ca_Kabuli_Chr05*	37332441	(T/C)
CakSNP8843	*Kabuli*	*Ca_Kabuli_Chr05*	37332501	(C/T)
CakSNP8844	*Kabuli*	*Ca_Kabuli_Chr05*	37339022	(T/G)
CakSNP8845	*Kabuli*	*Ca_Kabuli_Chr05*	37339358	(T/G)
CakSNP8846	*Kabuli*	*Ca_Kabuli_Chr05*	37339327	(A/G)
CakSNP8847	*Kabuli*	*Ca_Kabuli_Chr05*	37339300	(A/G)
CakSNP8848	*Kabuli*	*Ca_Kabuli_Chr05*	37342358	(G/T)
CakSNP8849	*Kabuli*	*Ca_Kabuli_Chr05*	37363626	(C/T)
CakSNP8850	*Kabuli*	*Ca_Kabuli_Chr05*	37375575	(C/G)
CakSNP8851	*Kabuli*	*Ca_Kabuli_Chr05*	37375620	(C/T)
CakSNP8852	*Kabuli*	*Ca_Kabuli_Chr05*	37445226	(A/G)
CakSNP8853	*Kabuli*	*Ca_Kabuli_Chr05*	37460980	(A/G)
CakSNP8854	*Kabuli*	*Ca_Kabuli_Chr05*	37468727	(T/C)
CakSNP8855	*Kabuli*	*Ca_Kabuli_Chr05*	37468755	(G/A)

SNP IDs	Cultivars	Chromosomes/scaffolds	Physical positions (bp)	SNPs
CakSNP8856	*Kabuli*	*Ca_Kabuli_Chr05*	37555816	(T/A)
CakSNP8857	*Kabuli*	*Ca_Kabuli_Chr05*	37555846	(T/A)
CakSNP8858	*Kabuli*	*Ca_Kabuli_Chr05*	37578999	(T/G)
CakSNP8859	*Kabuli*	*Ca_Kabuli_Chr05*	37591782	(A/T)
CakSNP8860	*Kabuli*	*Ca_Kabuli_Chr05*	37593444	(A/T)
CakSNP8861	*Kabuli*	*Ca_Kabuli_Chr05*	37594347	(T/C)
CakSNP8862	*Kabuli*	*Ca_Kabuli_Chr05*	37604030	(T/C)
CakSNP8863	*Kabuli*	*Ca_Kabuli_Chr05*	37604090	(G/T)
CakSNP8864	*Kabuli*	*Ca_Kabuli_Chr05*	37604780	(C/T)
CakSNP8865	*Kabuli*	*Ca_Kabuli_Chr05*	37796337	(T/G)
CakSNP8866	*Kabuli*	*Ca_Kabuli_Chr05*	37798313	(T/G)
CakSNP8867	*Kabuli*	*Ca_Kabuli_Chr05*	37868250	(C/T)
CakSNP8868	*Kabuli*	*Ca_Kabuli_Chr05*	37868256	(T/A)
CakSNP8869	*Kabuli*	*Ca_Kabuli_Chr05*	37869744	(T/C)
CakSNP8870	*Kabuli*	*Ca_Kabuli_Chr05*	37872223	(C/G)
CakSNP8871	*Kabuli*	*Ca_Kabuli_Chr05*	37874564	(T/A)
CakSNP8872	*Kabuli*	*Ca_Kabuli_Chr05*	37874725	(A/C)
CakSNP8873	*Kabuli*	*Ca_Kabuli_Chr05*	37874694	(C/T)
CakSNP8874	*Kabuli*	*Ca_Kabuli_Chr05*	37886417	(T/G)
CakSNP8875	*Kabuli*	*Ca_Kabuli_Chr05*	37899098	(T/C)
CakSNP8876	*Kabuli*	*Ca_Kabuli_Chr05*	37899238	(T/C)
CakSNP8877	*Kabuli*	*Ca_Kabuli_Chr05*	37908818	(A/C)
CakSNP8878	*Kabuli*	*Ca_Kabuli_Chr05*	37908807	(A/T)
CakSNP8879	*Kabuli*	*Ca_Kabuli_Chr05*	37911813	(A/C)
CakSNP8880	*Kabuli*	*Ca_Kabuli_Chr05*	38018552	(A/T)
CakSNP8881	*Kabuli*	*Ca_Kabuli_Chr05*	38018558	(G/C)
CakSNP8882	*Kabuli*	*Ca_Kabuli_Chr05*	38057582	(C/T)
CakSNP8883	*Kabuli*	*Ca_Kabuli_Chr05*	38080226	(A/G)
CakSNP8884	*Kabuli*	*Ca_Kabuli_Chr05*	38080349	(C/T)
CakSNP8885	*Kabuli*	*Ca_Kabuli_Chr05*	38092606	(G/A)
CakSNP8886	*Kabuli*	*Ca_Kabuli_Chr05*	38092619	(T/C)
CakSNP8887	*Kabuli*	*Ca_Kabuli_Chr05*	38092732	(C/T)
CakSNP8888	*Kabuli*	*Ca_Kabuli_Chr05*	38092735	(C/T)
CakSNP8889	*Kabuli*	*Ca_Kabuli_Chr05*	38092793	(C/T)
CakSNP8890	*Kabuli*	*Ca_Kabuli_Chr05*	38102941	(T/G)
CakSNP8891	*Kabuli*	*Ca_Kabuli_Chr05*	38108396	(G/A)
CakSNP8892	*Kabuli*	*Ca_Kabuli_Chr05*	38110219	(T/G)
CakSNP8893	*Kabuli*	*Ca_Kabuli_Chr05*	38220843	(G/A)
CakSNP8894	*Kabuli*	*Ca_Kabuli_Chr05*	38220908	(G/T)
CakSNP8895	*Kabuli*	*Ca_Kabuli_Chr05*	38248895	(A/C)
CakSNP8896	*Kabuli*	*Ca_Kabuli_Chr05*	38249134	(T/C)

**SNP IDs**	**Cultivars**	**Chromosomes/scaffolds**	**Physical positions (bp)**	**SNPs**
CakSNP8897	*Kabuli*	*Ca_Kabuli_Chr05*	38249132	(A/C)
CakSNP8898	*Kabuli*	*Ca_Kabuli_Chr05*	38266153	(G/A)
CakSNP8899	*Kabuli*	*Ca_Kabuli_Chr05*	38307036	(G/A)
CakSNP8900	*Kabuli*	*Ca_Kabuli_Chr05*	38333718	(C/T)
CakSNP8901	*Kabuli*	*Ca_Kabuli_Chr05*	38333746	(G/A)
CakSNP8902	*Kabuli*	*Ca_Kabuli_Chr05*	38333774	(A/G)
CakSNP8903	*Kabuli*	*Ca_Kabuli_Chr05*	38347201	(A/C)
CakSNP8904	*Kabuli*	*Ca_Kabuli_Chr05*	38455563	(A/C)
CakSNP8905	*Kabuli*	*Ca_Kabuli_Chr05*	38509657	(A/C)
CakSNP8906	*Kabuli*	*Ca_Kabuli_Chr05*	38538904	(A/G)
CakSNP8907	*Kabuli*	*Ca_Kabuli_Chr05*	38551438	(C/T)
CakSNP8908	*Kabuli*	*Ca_Kabuli_Chr05*	38551381	(G/A)
CakSNP8909	*Kabuli*	*Ca_Kabuli_Chr05*	38647040	(A/C)
CakSNP8910	*Kabuli*	*Ca_Kabuli_Chr05*	38656599	(C/T)
CakSNP8911	*Kabuli*	*Ca_Kabuli_Chr05*	38783604	(C/A)
CakSNP8912	*Kabuli*	*Ca_Kabuli_Chr05*	38784078	(G/A)
CakSNP8913	*Kabuli*	*Ca_Kabuli_Chr05*	38784074	(A/G)
CakSNP8914	*Kabuli*	*Ca_Kabuli_Chr05*	38784150	(T/A)
CakSNP8915	*Kabuli*	*Ca_Kabuli_Chr05*	38784129	(G/C)
CakSNP8916	*Kabuli*	*Ca_Kabuli_Chr05*	38857904	(T/C)
CakSNP8917	*Kabuli*	*Ca_Kabuli_Chr05*	38872143	(A/G)
CakSNP8918	*Kabuli*	*Ca_Kabuli_Chr05*	38892604	(A/C)
CakSNP8919	*Kabuli*	*Ca_Kabuli_Chr05*	38898971	(G/A)
CakSNP8920	*Kabuli*	*Ca_Kabuli_Chr05*	38909701	(C/T)
CakSNP8921	*Kabuli*	*Ca_Kabuli_Chr05*	38937304	(C/G)
CakSNP8922	*Kabuli*	*Ca_Kabuli_Chr05*	38937398	(C/A)
CakSNP8923	*Kabuli*	*Ca_Kabuli_Chr05*	38939578	(C/A)
CakSNP8924	*Kabuli*	*Ca_Kabuli_Chr05*	38984762	(A/G)
CakSNP8925	*Kabuli*	*Ca_Kabuli_Chr05*	38984769	(A/C)
CakSNP8926	*Kabuli*	*Ca_Kabuli_Chr05*	38984776	(A/G)
CakSNP8927	*Kabuli*	*Ca_Kabuli_Chr05*	39008980	(G/A)
CakSNP8928	*Kabuli*	*Ca_Kabuli_Chr05*	39137170	(A/G)
CakSNP8929	*Kabuli*	*Ca_Kabuli_Chr05*	39175007	(A/T)
CakSNP8930	*Kabuli*	*Ca_Kabuli_Chr05*	39176391	(T/A)
CakSNP8931	*Kabuli*	*Ca_Kabuli_Chr05*	39266333	(T/C)
CakSNP8932	*Kabuli*	*Ca_Kabuli_Chr05*	39277581	(T/C)
CakSNP8933	*Kabuli*	*Ca_Kabuli_Chr05*	39283856	(G/A)
CakSNP8934	*Kabuli*	*Ca_Kabuli_Chr05*	39326933	(G/T)
CakSNP8935	*Kabuli*	*Ca_Kabuli_Chr05*	39353755	(T/G)
CakSNP8936	*Kabuli*	*Ca_Kabuli_Chr05*	39364522	(T/C)
CakSNP8937	*Kabuli*	*Ca_Kabuli_Chr05*	39364569	(G/A)

SNP IDs	Cultivars	Chromosomes/scaffolds	Physical positions (bp)	SNPs
CakSNP8938	*Kabuli*	*Ca_Kabuli_Chr05*	39364571	(C/A)
CakSNP8939	*Kabuli*	*Ca_Kabuli_Chr05*	39364720	(A/T)
CakSNP8940	*Kabuli*	*Ca_Kabuli_Chr05*	39364700	(C/A)
CakSNP8941	*Kabuli*	*Ca_Kabuli_Chr05*	39364695	(A/C)
CakSNP8942	*Kabuli*	*Ca_Kabuli_Chr05*	39364667	(T/A)
CakSNP8943	*Kabuli*	*Ca_Kabuli_Chr05*	39364741	(A/T)
CakSNP8944	*Kabuli*	*Ca_Kabuli_Chr05*	39364752	(G/T)
CakSNP8945	*Kabuli*	*Ca_Kabuli_Chr05*	39364895	(A/C)
CakSNP8946	*Kabuli*	*Ca_Kabuli_Chr05*	39364856	(A/G)
CakSNP8947	*Kabuli*	*Ca_Kabuli_Chr05*	39364848	(C/T)
CakSNP8948	*Kabuli*	*Ca_Kabuli_Chr05*	39364841	(A/T)
CakSNP8949	*Kabuli*	*Ca_Kabuli_Chr05*	39366353	(C/G)
CakSNP8950	*Kabuli*	*Ca_Kabuli_Chr05*	39385603	(T/G)
CakSNP8951	*Kabuli*	*Ca_Kabuli_Chr05*	39385630	(C/T)
CakSNP8952	*Kabuli*	*Ca_Kabuli_Chr05*	39410377	(T/G)
CakSNP8953	*Kabuli*	*Ca_Kabuli_Chr05*	39410329	(T/G)
CakSNP8954	*Kabuli*	*Ca_Kabuli_Chr05*	39460567	(A/C)
CakSNP8955	*Kabuli*	*Ca_Kabuli_Chr05*	39475960	(T/C)
CakSNP8956	*Kabuli*	*Ca_Kabuli_Chr05*	39476040	(T/G)
CakSNP8957	*Kabuli*	*Ca_Kabuli_Chr05*	39498665	(G/A)
CakSNP8958	*Kabuli*	*Ca_Kabuli_Chr05*	39513563	(C/A)
CakSNP8959	*Kabuli*	*Ca_Kabuli_Chr05*	39543929	(C/T)
CakSNP8960	*Kabuli*	*Ca_Kabuli_Chr05*	39576823	(C/T)
CakSNP8961	*Kabuli*	*Ca_Kabuli_Chr05*	39576836	(T/A)
CakSNP8962	*Kabuli*	*Ca_Kabuli_Chr05*	39576839	(C/T)
CakSNP8963	*Kabuli*	*Ca_Kabuli_Chr05*	39576864	(C/A)
CakSNP8964	*Kabuli*	*Ca_Kabuli_Chr05*	39576870	(C/T)
CakSNP8965	*Kabuli*	*Ca_Kabuli_Chr05*	39576874	(G/T)
CakSNP8966	*Kabuli*	*Ca_Kabuli_Chr05*	39576841	(G/A)
CakSNP8967	*Kabuli*	*Ca_Kabuli_Chr05*	39592439	(A/G)
CakSNP8968	*Kabuli*	*Ca_Kabuli_Chr05*	39597485	(C/T)
CakSNP8969	*Kabuli*	*Ca_Kabuli_Chr05*	39597459	(T/C)
CakSNP8970	*Kabuli*	*Ca_Kabuli_Chr05*	39608204	(A/G)
CakSNP8971	*Kabuli*	*Ca_Kabuli_Chr05*	39608209	(G/T)
CakSNP8972	*Kabuli*	*Ca_Kabuli_Chr05*	39684480	(A/G)
CakSNP8973	*Kabuli*	*Ca_Kabuli_Chr05*	39684592	(A/T)
CakSNP8974	*Kabuli*	*Ca_Kabuli_Chr05*	39713021	(G/A)
CakSNP8975	*Kabuli*	*Ca_Kabuli_Chr05*	39776794	(G/A)
CakSNP8976	*Kabuli*	*Ca_Kabuli_Chr05*	39776795	(G/A)
CakSNP8977	*Kabuli*	*Ca_Kabuli_Chr05*	39776818	(C/T)
CakSNP8978	*Kabuli*	*Ca_Kabuli_Chr05*	39776850	(G/A)

SNP IDs	Cultivars	Chromosomes/scaffolds	Physical positions (bp)	SNPs
CakSNP8979	*Kabuli*	*Ca_Kabuli_Chr05*	39776869	(C/T)
CakSNP8980	*Kabuli*	*Ca_Kabuli_Chr05*	39794718	(C/T)
CakSNP8981	*Kabuli*	*Ca_Kabuli_Chr05*	39851570	(T/G)
CakSNP8982	*Kabuli*	*Ca_Kabuli_Chr05*	39869398	(C/T)
CakSNP8983	*Kabuli*	*Ca_Kabuli_Chr05*	39943298	(C/T)
CakSNP8984	*Kabuli*	*Ca_Kabuli_Chr05*	39985042	(G/A)
CakSNP8985	*Kabuli*	*Ca_Kabuli_Chr05*	39985332	(A/G)
CakSNP8986	*Kabuli*	*Ca_Kabuli_Chr05*	40018264	(A/C)
CakSNP8987	*Kabuli*	*Ca_Kabuli_Chr05*	40020062	(C/T)
CakSNP8988	*Kabuli*	*Ca_Kabuli_Chr05*	40020073	(C/G)
CakSNP8989	*Kabuli*	*Ca_Kabuli_Chr05*	40025250	(G/A)
CakSNP8990	*Kabuli*	*Ca_Kabuli_Chr05*	40025319	(T/C)
CakSNP8991	*Kabuli*	*Ca_Kabuli_Chr05*	40025326	(C/T)
CakSNP8992	*Kabuli*	*Ca_Kabuli_Chr05*	40025376	(A/G)
CakSNP8993	*Kabuli*	*Ca_Kabuli_Chr05*	40025377	(T/A)
CakSNP8994	*Kabuli*	*Ca_Kabuli_Chr05*	40027707	(A/G)
CakSNP8995	*Kabuli*	*Ca_Kabuli_Chr05*	40078085	(C/G)
CakSNP8996	*Kabuli*	*Ca_Kabuli_Chr05*	40078077	(A/G)
CakSNP8997	*Kabuli*	*Ca_Kabuli_Chr05*	40159260	(C/A)
CakSNP8998	*Kabuli*	*Ca_Kabuli_Chr05*	40159348	(G/A)
CakSNP8999	*Kabuli*	*Ca_Kabuli_Chr05*	40162755	(T/C)
CakSNP9000	*Kabuli*	*Ca_Kabuli_Chr05*	40162995	(C/T)
CakSNP9001	*Kabuli*	*Ca_Kabuli_Chr05*	40164176	(C/G)
CakSNP9002	*Kabuli*	*Ca_Kabuli_Chr05*	40239397	(G/C)
CakSNP9003	*Kabuli*	*Ca_Kabuli_Chr05*	40290082	(G/A)
CakSNP9004	*Kabuli*	*Ca_Kabuli_Chr05*	40290066	(A/G)
CakSNP9005	*Kabuli*	*Ca_Kabuli_Chr05*	40404136	(C/T)
CakSNP9006	*Kabuli*	*Ca_Kabuli_Chr05*	40404105	(G/C)
CakSNP9007	*Kabuli*	*Ca_Kabuli_Chr05*	40410891	(C/A)
CakSNP9008	*Kabuli*	*Ca_Kabuli_Chr05*	40410895	(G/T)
CakSNP9009	*Kabuli*	*Ca_Kabuli_Chr05*	40411325	(G/C)
CakSNP9010	*Kabuli*	*Ca_Kabuli_Chr05*	40464608	(A/G)
CakSNP9011	*Kabuli*	*Ca_Kabuli_Chr05*	40505498	(T/G)
CakSNP9012	*Kabuli*	*Ca_Kabuli_Chr05*	40507018	(T/A)
CakSNP9013	*Kabuli*	*Ca_Kabuli_Chr05*	40507107	(A/G)
CakSNP9014	*Kabuli*	*Ca_Kabuli_Chr05*	40507116	(A/G)
CakSNP9015	*Kabuli*	*Ca_Kabuli_Chr05*	40507158	(A/G)
CakSNP9016	*Kabuli*	*Ca_Kabuli_Chr05*	40507653	(G/C)
CakSNP9017	*Kabuli*	*Ca_Kabuli_Chr05*	40507662	(C/T)
CakSNP9018	*Kabuli*	*Ca_Kabuli_Chr05*	40507683	(G/T)
CakSNP9019	*Kabuli*	*Ca_Kabuli_Chr05*	40532435	(A/G)

**SNP IDs**	**Cultivars**	**Chromosomes/scaffolds**	**Physical positions (bp)**	**SNPs**
CakSNP9020	*Kabuli*	*Ca_Kabuli_Chr05*	40543989	(G/T)
CakSNP9021	*Kabuli*	*Ca_Kabuli_Chr05*	40553279	(C/G)
CakSNP9022	*Kabuli*	*Ca_Kabuli_Chr05*	40631069	(G/C)
CakSNP9023	*Kabuli*	*Ca_Kabuli_Chr05*	40666160	(T/A)
CakSNP9024	*Kabuli*	*Ca_Kabuli_Chr05*	40666171	(T/C)
CakSNP9025	*Kabuli*	*Ca_Kabuli_Chr05*	40666174	(C/T)
CakSNP9026	*Kabuli*	*Ca_Kabuli_Chr05*	40673241	(A/G)
CakSNP9027	*Kabuli*	*Ca_Kabuli_Chr05*	40700278	(C/G)
CakSNP9028	*Kabuli*	*Ca_Kabuli_Chr05*	40700260	(T/C)
CakSNP9029	*Kabuli*	*Ca_Kabuli_Chr05*	40700318	(C/A)
CakSNP9030	*Kabuli*	*Ca_Kabuli_Chr05*	40700772	(C/T)
CakSNP9031	*Kabuli*	*Ca_Kabuli_Chr05*	40736291	(C/G)
CakSNP9032	*Kabuli*	*Ca_Kabuli_Chr05*	40828566	(A/C)
CakSNP9033	*Kabuli*	*Ca_Kabuli_Chr05*	40828567	(G/C)
CakSNP9034	*Kabuli*	*Ca_Kabuli_Chr05*	40841690	(C/T)
CakSNP9035	*Kabuli*	*Ca_Kabuli_Chr05*	40845320	(C/T)
CakSNP9036	*Kabuli*	*Ca_Kabuli_Chr05*	40883438	(C/A)
CakSNP9037	*Kabuli*	*Ca_Kabuli_Chr05*	40955055	(A/T)
CakSNP9038	*Kabuli*	*Ca_Kabuli_Chr05*	40966058	(G/A)
CakSNP9039	*Kabuli*	*Ca_Kabuli_Chr05*	41024079	(A/G)
CakSNP9040	*Kabuli*	*Ca_Kabuli_Chr05*	41024135	(C/G)
CakSNP9041	*Kabuli*	*Ca_Kabuli_Chr05*	41024201	(G/A)
CakSNP9042	*Kabuli*	*Ca_Kabuli_Chr05*	41054578	(A/G)
CakSNP9043	*Kabuli*	*Ca_Kabuli_Chr05*	41055111	(T/A)
CakSNP9044	*Kabuli*	*Ca_Kabuli_Chr05*	41063657	(C/T)
CakSNP9045	*Kabuli*	*Ca_Kabuli_Chr05*	41066644	(C/G)
CakSNP9046	*Kabuli*	*Ca_Kabuli_Chr05*	41066664	(T/C)
CakSNP9047	*Kabuli*	*Ca_Kabuli_Chr05*	41066667	(C/A)
CakSNP9048	*Kabuli*	*Ca_Kabuli_Chr05*	41080035	(C/T)
CakSNP9049	*Kabuli*	*Ca_Kabuli_Chr05*	41191566	(A/T)
CakSNP9050	*Kabuli*	*Ca_Kabuli_Chr05*	41191676	(G/C)
CakSNP9051	*Kabuli*	*Ca_Kabuli_Chr05*	41197909	(A/T)
CakSNP9052	*Kabuli*	*Ca_Kabuli_Chr05*	41203594	(C/T)
CakSNP9053	*Kabuli*	*Ca_Kabuli_Chr05*	41253742	(A/G)
CakSNP9054	*Kabuli*	*Ca_Kabuli_Chr05*	41283253	(T/C)
CakSNP9055	*Kabuli*	*Ca_Kabuli_Chr05*	41283453	(A/C)
CakSNP9056	*Kabuli*	*Ca_Kabuli_Chr05*	41285223	(A/C)
CakSNP9057	*Kabuli*	*Ca_Kabuli_Chr05*	41302544	(C/G)
CakSNP9058	*Kabuli*	*Ca_Kabuli_Chr05*	41302601	(G/A)
CakSNP9059	*Kabuli*	*Ca_Kabuli_Chr05*	41302733	(T/C)
CakSNP9060	*Kabuli*	*Ca_Kabuli_Chr05*	41302804	(A/G)

SNP IDs	Cultivars	Chromosomes/scaffolds	Physical positions (bp)	SNPs
CakSNP9061	*Kabuli*	*Ca_Kabuli_Chr05*	41302859	(C/A)
CakSNP9062	*Kabuli*	*Ca_Kabuli_Chr05*	41302901	(C/A)
CakSNP9063	*Kabuli*	*Ca_Kabuli_Chr05*	41302900	(T/A)
CakSNP9064	*Kabuli*	*Ca_Kabuli_Chr05*	41302895	(G/A)
CakSNP9065	*Kabuli*	*Ca_Kabuli_Chr05*	41304103	(G/A)
CakSNP9066	*Kabuli*	*Ca_Kabuli_Chr05*	41399241	(C/T)
CakSNP9067	*Kabuli*	*Ca_Kabuli_Chr05*	41407077	(A/C)
CakSNP9068	*Kabuli*	*Ca_Kabuli_Chr05*	41407253	(T/C)
CakSNP9069	*Kabuli*	*Ca_Kabuli_Chr05*	41519141	(C/T)
CakSNP9070	*Kabuli*	*Ca_Kabuli_Chr05*	41521854	(T/G)
CakSNP9071	*Kabuli*	*Ca_Kabuli_Chr05*	41526026	(T/G)
CakSNP9072	*Kabuli*	*Ca_Kabuli_Chr05*	41635262	(C/T)
CakSNP9073	*Kabuli*	*Ca_Kabuli_Chr05*	41678144	(C/T)
CakSNP9074	*Kabuli*	*Ca_Kabuli_Chr05*	41696426	(T/C)
CakSNP9075	*Kabuli*	*Ca_Kabuli_Chr05*	41696496	(G/C)
CakSNP9076	*Kabuli*	*Ca_Kabuli_Chr05*	41701479	(T/C)
CakSNP9077	*Kabuli*	*Ca_Kabuli_Chr05*	41772759	(G/A)
CakSNP9078	*Kabuli*	*Ca_Kabuli_Chr05*	41887214	(A/T)
CakSNP9079	*Kabuli*	*Ca_Kabuli_Chr05*	41902419	(A/C)
CakSNP9080	*Kabuli*	*Ca_Kabuli_Chr05*	41945316	(A/G)
CakSNP9081	*Kabuli*	*Ca_Kabuli_Chr05*	41945373	(C/T)
CakSNP9082	*Kabuli*	*Ca_Kabuli_Chr05*	41945308	(G/A)
CakSNP9083	*Kabuli*	*Ca_Kabuli_Chr05*	41974690	(T/C)
CakSNP9084	*Kabuli*	*Ca_Kabuli_Chr05*	42004600	(T/G)
CakSNP9085	*Kabuli*	*Ca_Kabuli_Chr05*	42004602	(A/G)
CakSNP9086	*Kabuli*	*Ca_Kabuli_Chr05*	42004734	(G/A)
CakSNP9087	*Kabuli*	*Ca_Kabuli_Chr05*	42004742	(G/A)
CakSNP9088	*Kabuli*	*Ca_Kabuli_Chr05*	42004751	(G/A)
CakSNP9089	*Kabuli*	*Ca_Kabuli_Chr05*	42004753	(C/A)
CakSNP9090	*Kabuli*	*Ca_Kabuli_Chr05*	42008626	(A/C)
CakSNP9091	*Kabuli*	*Ca_Kabuli_Chr05*	42012972	(C/A)
CakSNP9092	*Kabuli*	*Ca_Kabuli_Chr05*	42012983	(G/A)
CakSNP9093	*Kabuli*	*Ca_Kabuli_Chr05*	42012998	(C/T)
CakSNP9094	*Kabuli*	*Ca_Kabuli_Chr05*	42020435	(A/G)
CakSNP9095	*Kabuli*	*Ca_Kabuli_Chr05*	42100070	(A/G)
CakSNP9096	*Kabuli*	*Ca_Kabuli_Chr05*	42117516	(C/T)
CakSNP9097	*Kabuli*	*Ca_Kabuli_Chr05*	42120698	(T/C)
CakSNP9098	*Kabuli*	*Ca_Kabuli_Chr05*	42120735	(G/C)
CakSNP9099	*Kabuli*	*Ca_Kabuli_Chr05*	42121472	(C/A)
CakSNP9100	*Kabuli*	*Ca_Kabuli_Chr05*	42225634	(G/A)
CakSNP9101	*Kabuli*	*Ca_Kabuli_Chr05*	42275523	(G/A)

**SNP IDs**	**Cultivars**	**Chromosomes/scaffolds**	**Physical positions (bp)**	**SNPs**
CakSNP9102	*Kabuli*	*Ca_Kabuli_Chr05*	42282297	(G/A)
CakSNP9103	*Kabuli*	*Ca_Kabuli_Chr05*	42413430	(A/C)
CakSNP9104	*Kabuli*	*Ca_Kabuli_Chr05*	42422867	(C/A)
CakSNP9105	*Kabuli*	*Ca_Kabuli_Chr05*	42422845	(T/C)
CakSNP9106	*Kabuli*	*Ca_Kabuli_Chr05*	42422837	(G/A)
CakSNP9107	*Kabuli*	*Ca_Kabuli_Chr05*	42422808	(A/G)
CakSNP9108	*Kabuli*	*Ca_Kabuli_Chr05*	42422932	(C/A)
CakSNP9109	*Kabuli*	*Ca_Kabuli_Chr05*	42435263	(C/T)
CakSNP9110	*Kabuli*	*Ca_Kabuli_Chr05*	42436972	(A/G)
CakSNP9111	*Kabuli*	*Ca_Kabuli_Chr05*	42436976	(A/T)
CakSNP9112	*Kabuli*	*Ca_Kabuli_Chr05*	42443786	(A/G)
CakSNP9113	*Kabuli*	*Ca_Kabuli_Chr05*	42477712	(G/A)
CakSNP9114	*Kabuli*	*Ca_Kabuli_Chr05*	42485130	(T/C)
CakSNP9115	*Kabuli*	*Ca_Kabuli_Chr05*	42528744	(T/G)
CakSNP9116	*Kabuli*	*Ca_Kabuli_Chr05*	42528934	(A/G)
CakSNP9117	*Kabuli*	*Ca_Kabuli_Chr05*	42540675	(A/T)
CakSNP9118	*Kabuli*	*Ca_Kabuli_Chr05*	42582262	(T/A)
CakSNP9119	*Kabuli*	*Ca_Kabuli_Chr05*	42655824	(A/G)
CakSNP9120	*Kabuli*	*Ca_Kabuli_Chr05*	42749499	(C/T)
CakSNP9121	*Kabuli*	*Ca_Kabuli_Chr05*	42792635	(G/A)
CakSNP9122	*Kabuli*	*Ca_Kabuli_Chr05*	42814621	(C/T)
CakSNP9123	*Kabuli*	*Ca_Kabuli_Chr05*	42814623	(A/G)
CakSNP9124	*Kabuli*	*Ca_Kabuli_Chr05*	42857977	(G/C)
CakSNP9125	*Kabuli*	*Ca_Kabuli_Chr05*	42863366	(C/T)
CakSNP9126	*Kabuli*	*Ca_Kabuli_Chr05*	42872074	(A/C)
CakSNP9127	*Kabuli*	*Ca_Kabuli_Chr05*	42872091	(A/G)
CakSNP9128	*Kabuli*	*Ca_Kabuli_Chr05*	42881185	(C/T)
CakSNP9129	*Kabuli*	*Ca_Kabuli_Chr05*	42882945	(C/T)
CakSNP9130	*Kabuli*	*Ca_Kabuli_Chr05*	42882965	(C/T)
CakSNP9131	*Kabuli*	*Ca_Kabuli_Chr05*	42883163	(T/C)
CakSNP9132	*Kabuli*	*Ca_Kabuli_Chr05*	42910653	(A/G)
CakSNP9133	*Kabuli*	*Ca_Kabuli_Chr05*	42910661	(A/G)
CakSNP9134	*Kabuli*	*Ca_Kabuli_Chr05*	42910732	(A/T)
CakSNP9135	*Kabuli*	*Ca_Kabuli_Chr05*	42924113	(C/T)
CakSNP9136	*Kabuli*	*Ca_Kabuli_Chr05*	42928986	(T/G)
CakSNP9137	*Kabuli*	*Ca_Kabuli_Chr05*	42929087	(A/C)
CakSNP9138	*Kabuli*	*Ca_Kabuli_Chr05*	42946580	(T/C)
CakSNP9139	*Kabuli*	*Ca_Kabuli_Chr05*	42946716	(A/T)
CakSNP9140	*Kabuli*	*Ca_Kabuli_Chr05*	42999704	(C/T)
CakSNP9141	*Kabuli*	*Ca_Kabuli_Chr05*	43000210	(A/G)
CakSNP9142	*Kabuli*	*Ca_Kabuli_Chr05*	43034618	(T/A)

SNP IDs	Cultivars	Chromosomes/scaffolds	Physical positions (bp)	SNPs
CakSNP9143	*Kabuli*	*Ca_Kabuli_Chr05*	43037863	(G/A)
CakSNP9144	*Kabuli*	*Ca_Kabuli_Chr05*	43060091	(C/T)
CakSNP9145	*Kabuli*	*Ca_Kabuli_Chr05*	43067894	(T/C)
CakSNP9146	*Kabuli*	*Ca_Kabuli_Chr05*	43067891	(G/C)
CakSNP9147	*Kabuli*	*Ca_Kabuli_Chr05*	43067905	(A/C)
CakSNP9148	*Kabuli*	*Ca_Kabuli_Chr05*	43080493	(G/A)
CakSNP9149	*Kabuli*	*Ca_Kabuli_Chr05*	43081783	(G/A)
CakSNP9150	*Kabuli*	*Ca_Kabuli_Chr05*	43110660	(G/A)
CakSNP9151	*Kabuli*	*Ca_Kabuli_Chr05*	43110669	(A/T)
CakSNP9152	*Kabuli*	*Ca_Kabuli_Chr05*	43110726	(T/G)
CakSNP9153	*Kabuli*	*Ca_Kabuli_Chr05*	43110737	(A/G)
CakSNP9154	*Kabuli*	*Ca_Kabuli_Chr05*	43175781	(G/A)
CakSNP9155	*Kabuli*	*Ca_Kabuli_Chr05*	43188824	(T/G)
CakSNP9156	*Kabuli*	*Ca_Kabuli_Chr05*	43188853	(C/A)
CakSNP9157	*Kabuli*	*Ca_Kabuli_Chr05*	43210793	(T/C)
CakSNP9158	*Kabuli*	*Ca_Kabuli_Chr05*	43212083	(G/A)
CakSNP9159	*Kabuli*	*Ca_Kabuli_Chr05*	43212066	(C/T)
CakSNP9160	*Kabuli*	*Ca_Kabuli_Chr05*	43221125	(T/C)
CakSNP9161	*Kabuli*	*Ca_Kabuli_Chr05*	43231220	(T/C)
CakSNP9162	*Kabuli*	*Ca_Kabuli_Chr05*	43231265	(C/T)
CakSNP9163	*Kabuli*	*Ca_Kabuli_Chr05*	43253586	(C/A)
CakSNP9164	*Kabuli*	*Ca_Kabuli_Chr05*	43275472	(T/C)
CakSNP9165	*Kabuli*	*Ca_Kabuli_Chr05*	43334903	(T/C)
CakSNP9166	*Kabuli*	*Ca_Kabuli_Chr05*	43341646	(A/C)
CakSNP9167	*Kabuli*	*Ca_Kabuli_Chr05*	43402230	(C/T)
CakSNP9168	*Kabuli*	*Ca_Kabuli_Chr05*	43410326	(T/G)
CakSNP9169	*Kabuli*	*Ca_Kabuli_Chr05*	43423593	(G/C)
CakSNP9170	*Kabuli*	*Ca_Kabuli_Chr05*	43521774	(A/T)
CakSNP9171	*Kabuli*	*Ca_Kabuli_Chr05*	43521789	(G/A)
CakSNP9172	*Kabuli*	*Ca_Kabuli_Chr05*	43521830	(T/C)
CakSNP9173	*Kabuli*	*Ca_Kabuli_Chr05*	43521851	(G/A)
CakSNP9174	*Kabuli*	*Ca_Kabuli_Chr05*	43523560	(A/G)
CakSNP9175	*Kabuli*	*Ca_Kabuli_Chr05*	43530567	(C/T)
CakSNP9176	*Kabuli*	*Ca_Kabuli_Chr05*	43565712	(T/C)
CakSNP9177	*Kabuli*	*Ca_Kabuli_Chr05*	43632249	(C/A)
CakSNP9178	*Kabuli*	*Ca_Kabuli_Chr05*	43632281	(C/G)
CakSNP9179	*Kabuli*	*Ca_Kabuli_Chr05*	43632349	(T/A)
CakSNP9180	*Kabuli*	*Ca_Kabuli_Chr05*	43632400	(A/G)
CakSNP9181	*Kabuli*	*Ca_Kabuli_Chr05*	43632377	(C/T)
CakSNP9182	*Kabuli*	*Ca_Kabuli_Chr05*	43632481	(G/T)
CakSNP9183	*Kabuli*	*Ca_Kabuli_Chr05*	43632508	(A/G)

SNP IDs	Cultivars	Chromosomes/scaffolds	Physical positions (bp)	SNPs
CakSNP9184	*Kabuli*	*Ca_Kabuli_Chr05*	43710359	(T/G)
CakSNP9185	*Kabuli*	*Ca_Kabuli_Chr05*	43710362	(A/T)
CakSNP9186	*Kabuli*	*Ca_Kabuli_Chr05*	43710366	(G/T)
CakSNP9187	*Kabuli*	*Ca_Kabuli_Chr05*	43710391	(A/G)
CakSNP9188	*Kabuli*	*Ca_Kabuli_Chr05*	43742448	(C/T)
CakSNP9189	*Kabuli*	*Ca_Kabuli_Chr05*	43742456	(G/A)
CakSNP9190	*Kabuli*	*Ca_Kabuli_Chr05*	43755074	(A/G)
CakSNP9191	*Kabuli*	*Ca_Kabuli_Chr05*	43755756	(A/G)
CakSNP9192	*Kabuli*	*Ca_Kabuli_Chr05*	43759059	(A/C)
CakSNP9193	*Kabuli*	*Ca_Kabuli_Chr05*	43786883	(A/G)
CakSNP9194	*Kabuli*	*Ca_Kabuli_Chr05*	43789875	(C/G)
CakSNP9195	*Kabuli*	*Ca_Kabuli_Chr05*	43790108	(G/A)
CakSNP9196	*Kabuli*	*Ca_Kabuli_Chr05*	43812119	(G/A)
CakSNP9197	*Kabuli*	*Ca_Kabuli_Chr05*	43812213	(C/T)
CakSNP9198	*Kabuli*	*Ca_Kabuli_Chr05*	43859301	(G/A)
CakSNP9199	*Kabuli*	*Ca_Kabuli_Chr05*	43904489	(T/A)
CakSNP9200	*Kabuli*	*Ca_Kabuli_Chr05*	43919009	(C/T)
CakSNP9201	*Kabuli*	*Ca_Kabuli_Chr05*	43919017	(T/G)
CakSNP9202	*Kabuli*	*Ca_Kabuli_Chr05*	43919022	(G/C)
CakSNP9203	*Kabuli*	*Ca_Kabuli_Chr05*	43919028	(A/G)
CakSNP9204	*Kabuli*	*Ca_Kabuli_Chr05*	43982994	(C/T)
CakSNP9205	*Kabuli*	*Ca_Kabuli_Chr05*	43995843	(T/C)
CakSNP9206	*Kabuli*	*Ca_Kabuli_Chr05*	44002824	(T/G)
CakSNP9207	*Kabuli*	*Ca_Kabuli_Chr05*	44002862	(C/A)
CakSNP9208	*Kabuli*	*Ca_Kabuli_Chr05*	44038229	(T/C)
CakSNP9209	*Kabuli*	*Ca_Kabuli_Chr05*	44064495	(G/A)
CakSNP9210	*Kabuli*	*Ca_Kabuli_Chr05*	44080450	(G/C)
CakSNP9211	*Kabuli*	*Ca_Kabuli_Chr05*	44109260	(G/T)
CakSNP9212	*Kabuli*	*Ca_Kabuli_Chr05*	44154671	(A/G)
CakSNP9213	*Kabuli*	*Ca_Kabuli_Chr05*	44159237	(C/G)
CakSNP9214	*Kabuli*	*Ca_Kabuli_Chr05*	44289612	(T/C)
CakSNP9215	*Kabuli*	*Ca_Kabuli_Chr05*	44334286	(C/G)
CakSNP9216	*Kabuli*	*Ca_Kabuli_Chr05*	44334318	(C/T)
CakSNP9217	*Kabuli*	*Ca_Kabuli_Chr05*	44364134	(C/T)
CakSNP9218	*Kabuli*	*Ca_Kabuli_Chr05*	44373968	(G/T)
CakSNP9219	*Kabuli*	*Ca_Kabuli_Chr05*	44378178	(C/A)
CakSNP9220	*Kabuli*	*Ca_Kabuli_Chr05*	44428965	(G/A)
CakSNP9221	*Kabuli*	*Ca_Kabuli_Chr05*	44461250	(A/G)
CakSNP9222	*Kabuli*	*Ca_Kabuli_Chr05*	44491489	(G/T)
CakSNP9223	*Kabuli*	*Ca_Kabuli_Chr05*	44555718	(A/C)
CakSNP9224	*Kabuli*	*Ca_Kabuli_Chr05*	44561095	(T/C)

SNP IDs	Cultivars	Chromosomes/scaffolds	Physical positions (bp)	SNPs
CakSNP9225	*Kabuli*	*Ca_Kabuli_Chr05*	44656921	(G/A)
CakSNP9226	*Kabuli*	*Ca_Kabuli_Chr05*	44667285	(C/T)
CakSNP9227	*Kabuli*	*Ca_Kabuli_Chr05*	44667297	(C/T)
CakSNP9228	*Kabuli*	*Ca_Kabuli_Chr05*	44667359	(C/A)
CakSNP9229	*Kabuli*	*Ca_Kabuli_Chr05*	44760403	(A/G)
CakSNP9230	*Kabuli*	*Ca_Kabuli_Chr05*	44760347	(G/T)
CakSNP9231	*Kabuli*	*Ca_Kabuli_Chr05*	44760469	(C/T)
CakSNP9232	*Kabuli*	*Ca_Kabuli_Chr05*	44760490	(C/A)
CakSNP9233	*Kabuli*	*Ca_Kabuli_Chr05*	44772326	(G/A)
CakSNP9234	*Kabuli*	*Ca_Kabuli_Chr05*	44884779	(G/C)
CakSNP9235	*Kabuli*	*Ca_Kabuli_Chr05*	44887105	(T/G)
CakSNP9236	*Kabuli*	*Ca_Kabuli_Chr05*	44887241	(T/C)
CakSNP9237	*Kabuli*	*Ca_Kabuli_Chr05*	44887234	(A/C)
CakSNP9238	*Kabuli*	*Ca_Kabuli_Chr05*	44891426	(G/C)
CakSNP9239	*Kabuli*	*Ca_Kabuli_Chr05*	44911842	(C/T)
CakSNP9240	*Kabuli*	*Ca_Kabuli_Chr05*	44911868	(C/G)
CakSNP9241	*Kabuli*	*Ca_Kabuli_Chr05*	44911881	(G/A)
CakSNP9242	*Kabuli*	*Ca_Kabuli_Chr05*	44911906	(C/T)
CakSNP9243	*Kabuli*	*Ca_Kabuli_Chr05*	44918947	(C/A)
CakSNP9244	*Kabuli*	*Ca_Kabuli_Chr05*	44938814	(A/T)
CakSNP9245	*Kabuli*	*Ca_Kabuli_Chr05*	44938832	(A/G)
CakSNP9246	*Kabuli*	*Ca_Kabuli_Chr05*	44939058	(C/G)
CakSNP9247	*Kabuli*	*Ca_Kabuli_Chr05*	44939033	(G/A)
CakSNP9248	*Kabuli*	*Ca_Kabuli_Chr05*	45087170	(G/A)
CakSNP9249	*Kabuli*	*Ca_Kabuli_Chr05*	45087156	(A/G)
CakSNP9250	*Kabuli*	*Ca_Kabuli_Chr05*	45087146	(C/G)
CakSNP9251	*Kabuli*	*Ca_Kabuli_Chr05*	45087140	(C/A)
CakSNP9252	*Kabuli*	*Ca_Kabuli_Chr05*	45087202	(G/C)
CakSNP9253	*Kabuli*	*Ca_Kabuli_Chr05*	45087267	(A/G)
CakSNP9254	*Kabuli*	*Ca_Kabuli_Chr05*	45087228	(A/C)
CakSNP9255	*Kabuli*	*Ca_Kabuli_Chr05*	45087215	(G/A)
CakSNP9256	*Kabuli*	*Ca_Kabuli_Chr05*	45215617	(G/A)
CakSNP9257	*Kabuli*	*Ca_Kabuli_Chr05*	45219125	(C/T)
CakSNP9258	*Kabuli*	*Ca_Kabuli_Chr05*	45219258	(G/T)
CakSNP9259	*Kabuli*	*Ca_Kabuli_Chr05*	45219355	(T/G)
CakSNP9260	*Kabuli*	*Ca_Kabuli_Chr05*	45254102	(C/T)
CakSNP9261	*Kabuli*	*Ca_Kabuli_Chr05*	45254112	(T/G)
CakSNP9262	*Kabuli*	*Ca_Kabuli_Chr05*	45301772	(T/G)
CakSNP9263	*Kabuli*	*Ca_Kabuli_Chr05*	45337274	(C/G)
CakSNP9264	*Kabuli*	*Ca_Kabuli_Chr05*	45337307	(C/T)
CakSNP9265	*Kabuli*	*Ca_Kabuli_Chr05*	45406853	(C/T)

SNP IDs	Cultivars	Chromosomes/scaffolds	Physical positions (bp)	SNPs
CakSNP9266	*Kabuli*	*Ca_Kabuli_Chr05*	45407063	(G/A)
CakSNP9267	*Kabuli*	*Ca_Kabuli_Chr05*	45407044	(A/C)
CakSNP9268	*Kabuli*	*Ca_Kabuli_Chr05*	45414089	(C/G)
CakSNP9269	*Kabuli*	*Ca_Kabuli_Chr05*	45428229	(A/G)
CakSNP9270	*Kabuli*	*Ca_Kabuli_Chr05*	45429513	(A/G)
CakSNP9271	*Kabuli*	*Ca_Kabuli_Chr05*	45429518	(T/G)
CakSNP9272	*Kabuli*	*Ca_Kabuli_Chr05*	45441223	(C/T)
CakSNP9273	*Kabuli*	*Ca_Kabuli_Chr05*	45444675	(G/T)
CakSNP9274	*Kabuli*	*Ca_Kabuli_Chr05*	45444924	(G/T)
CakSNP9275	*Kabuli*	*Ca_Kabuli_Chr05*	45480175	(T/G)
CakSNP9276	*Kabuli*	*Ca_Kabuli_Chr05*	45501379	(C/T)
CakSNP9277	*Kabuli*	*Ca_Kabuli_Chr05*	45540473	(G/A)
CakSNP9278	*Kabuli*	*Ca_Kabuli_Chr05*	45559874	(C/A)
CakSNP9279	*Kabuli*	*Ca_Kabuli_Chr05*	45601196	(A/G)
CakSNP9280	*Kabuli*	*Ca_Kabuli_Chr05*	45601313	(G/A)
CakSNP9281	*Kabuli*	*Ca_Kabuli_Chr05*	45642344	(C/A)
CakSNP9282	*Kabuli*	*Ca_Kabuli_Chr05*	45646235	(A/G)
CakSNP9283	*Kabuli*	*Ca_Kabuli_Chr05*	45649180	(G/A)
CakSNP9284	*Kabuli*	*Ca_Kabuli_Chr05*	45649189	(G/A)
CakSNP9285	*Kabuli*	*Ca_Kabuli_Chr05*	45661109	(T/C)
CakSNP9286	*Kabuli*	*Ca_Kabuli_Chr05*	45694453	(T/G)
CakSNP9287	*Kabuli*	*Ca_Kabuli_Chr05*	45710509	(G/A)
CakSNP9288	*Kabuli*	*Ca_Kabuli_Chr05*	45710476	(A/G)
CakSNP9289	*Kabuli*	*Ca_Kabuli_Chr05*	45726539	(T/A)
CakSNP9290	*Kabuli*	*Ca_Kabuli_Chr05*	45726995	(C/T)
CakSNP9291	*Kabuli*	*Ca_Kabuli_Chr05*	45727022	(T/C)
CakSNP9292	*Kabuli*	*Ca_Kabuli_Chr05*	45750121	(A/T)
CakSNP9293	*Kabuli*	*Ca_Kabuli_Chr05*	45798850	(C/A)
CakSNP9294	*Kabuli*	*Ca_Kabuli_Chr05*	45798966	(T/G)
CakSNP9295	*Kabuli*	*Ca_Kabuli_Chr05*	45798950	(C/T)
CakSNP9296	*Kabuli*	*Ca_Kabuli_Chr05*	45798919	(C/A)
CakSNP9297	*Kabuli*	*Ca_Kabuli_Chr05*	45808905	(G/A)
CakSNP9298	*Kabuli*	*Ca_Kabuli_Chr05*	45832009	(T/G)
CakSNP9299	*Kabuli*	*Ca_Kabuli_Chr05*	45832048	(C/G)
CakSNP9300	*Kabuli*	*Ca_Kabuli_Chr05*	45832070	(A/T)
CakSNP9301	*Kabuli*	*Ca_Kabuli_Chr05*	45842438	(A/C)
CakSNP9302	*Kabuli*	*Ca_Kabuli_Chr05*	45845081	(A/C)
CakSNP9303	*Kabuli*	*Ca_Kabuli_Chr05*	45848030	(A/G)
CakSNP9304	*Kabuli*	*Ca_Kabuli_Chr05*	45848087	(T/G)
CakSNP9305	*Kabuli*	*Ca_Kabuli_Chr05*	45952107	(A/G)
CakSNP9306	*Kabuli*	*Ca_Kabuli_Chr05*	45952842	(A/C)

**SNP IDs**	**Cultivars**	**Chromosomes/scaffolds**	**Physical positions (bp)**	**SNPs**
CakSNP9307	*Kabuli*	*Ca_Kabuli_Chr05*	45971560	(G/A)
CakSNP9308	*Kabuli*	*Ca_Kabuli_Chr05*	45978758	(A/G)
CakSNP9309	*Kabuli*	*Ca_Kabuli_Chr05*	46002514	(C/T)
CakSNP9310	*Kabuli*	*Ca_Kabuli_Chr05*	46002497	(G/A)
CakSNP9311	*Kabuli*	*Ca_Kabuli_Chr05*	46018939	(C/T)
CakSNP9312	*Kabuli*	*Ca_Kabuli_Chr05*	46019052	(T/A)
CakSNP9313	*Kabuli*	*Ca_Kabuli_Chr05*	46022566	(C/T)
CakSNP9314	*Kabuli*	*Ca_Kabuli_Chr05*	46022598	(T/C)
CakSNP9315	*Kabuli*	*Ca_Kabuli_Chr05*	46022551	(G/A)
CakSNP9316	*Kabuli*	*Ca_Kabuli_Chr05*	46022549	(G/A)
CakSNP9317	*Kabuli*	*Ca_Kabuli_Chr05*	46022547	(G/A)
CakSNP9318	*Kabuli*	*Ca_Kabuli_Chr05*	46022544	(A/G)
CakSNP9319	*Kabuli*	*Ca_Kabuli_Chr05*	46022531	(T/A)
CakSNP9320	*Kabuli*	*Ca_Kabuli_Chr05*	46049587	(A/G)
CakSNP9321	*Kabuli*	*Ca_Kabuli_Chr05*	46050168	(A/T)
CakSNP9322	*Kabuli*	*Ca_Kabuli_Chr05*	46077190	(T/C)
CakSNP9323	*Kabuli*	*Ca_Kabuli_Chr05*	46077302	(G/A)
CakSNP9324	*Kabuli*	*Ca_Kabuli_Chr05*	46083283	(G/A)
CakSNP9325	*Kabuli*	*Ca_Kabuli_Chr05*	46083343	(T/G)
CakSNP9326	*Kabuli*	*Ca_Kabuli_Chr05*	46083414	(T/G)
CakSNP9327	*Kabuli*	*Ca_Kabuli_Chr05*	46119122	(G/A)
CakSNP9328	*Kabuli*	*Ca_Kabuli_Chr05*	46119182	(A/G)
CakSNP9329	*Kabuli*	*Ca_Kabuli_Chr05*	46130289	(A/C)
CakSNP9330	*Kabuli*	*Ca_Kabuli_Chr05*	46155262	(C/T)
CakSNP9331	*Kabuli*	*Ca_Kabuli_Chr05*	46176366	(T/C)
CakSNP9332	*Kabuli*	*Ca_Kabuli_Chr05*	46176443	(G/A)
CakSNP9333	*Kabuli*	*Ca_Kabuli_Chr05*	46214229	(G/T)
CakSNP9334	*Kabuli*	*Ca_Kabuli_Chr05*	46214233	(C/T)
CakSNP9335	*Kabuli*	*Ca_Kabuli_Chr05*	46230010	(G/C)
CakSNP9336	*Kabuli*	*Ca_Kabuli_Chr05*	46230011	(G/A)
CakSNP9337	*Kabuli*	*Ca_Kabuli_Chr05*	46230109	(T/G)
CakSNP9338	*Kabuli*	*Ca_Kabuli_Chr05*	46246419	(G/C)
CakSNP9339	*Kabuli*	*Ca_Kabuli_Chr05*	46252617	(C/T)
CakSNP9340	*Kabuli*	*Ca_Kabuli_Chr05*	46252643	(T/A)
CakSNP9341	*Kabuli*	*Ca_Kabuli_Chr05*	46252605	(C/A)
CakSNP9342	*Kabuli*	*Ca_Kabuli_Chr05*	46260822	(C/T)
CakSNP9343	*Kabuli*	*Ca_Kabuli_Chr05*	46294000	(G/A)
CakSNP9344	*Kabuli*	*Ca_Kabuli_Chr05*	46300789	(G/A)
CakSNP9345	*Kabuli*	*Ca_Kabuli_Chr05*	46301639	(A/G)
CakSNP9346	*Kabuli*	*Ca_Kabuli_Chr05*	46321992	(C/T)
CakSNP9347	*Kabuli*	*Ca_Kabuli_Chr05*	46328589	(C/A)

**SNP IDs**	**Cultivars**	**Chromosomes/scaffolds**	**Physical positions (bp)**	**SNPs**
CakSNP9348	*Kabuli*	*Ca_Kabuli_Chr05*	46328627	(A/G)
CakSNP9349	*Kabuli*	*Ca_Kabuli_Chr05*	46328895	(T/C)
CakSNP9350	*Kabuli*	*Ca_Kabuli_Chr05*	46328902	(C/T)
CakSNP9351	*Kabuli*	*Ca_Kabuli_Chr05*	46344109	(G/T)
CakSNP9352	*Kabuli*	*Ca_Kabuli_Chr05*	46344121	(G/A)
CakSNP9353	*Kabuli*	*Ca_Kabuli_Chr05*	46356764	(G/A)
CakSNP9354	*Kabuli*	*Ca_Kabuli_Chr05*	46366598	(G/T)
CakSNP9355	*Kabuli*	*Ca_Kabuli_Chr05*	46368606	(G/T)
CakSNP9356	*Kabuli*	*Ca_Kabuli_Chr05*	46376496	(G/T)
CakSNP9357	*Kabuli*	*Ca_Kabuli_Chr05*	46376532	(A/C)
CakSNP9358	*Kabuli*	*Ca_Kabuli_Chr05*	46403922	(G/T)
CakSNP9359	*Kabuli*	*Ca_Kabuli_Chr05*	46422788	(C/G)
CakSNP9360	*Kabuli*	*Ca_Kabuli_Chr05*	46422869	(A/T)
CakSNP9361	*Kabuli*	*Ca_Kabuli_Chr05*	46445611	(A/T)
CakSNP9362	*Kabuli*	*Ca_Kabuli_Chr05*	46445651	(G/T)
CakSNP9363	*Kabuli*	*Ca_Kabuli_Chr05*	46491761	(C/G)
CakSNP9364	*Kabuli*	*Ca_Kabuli_Chr05*	46526963	(A/C)
CakSNP9365	*Kabuli*	*Ca_Kabuli_Chr05*	46621351	(G/A)
CakSNP9366	*Kabuli*	*Ca_Kabuli_Chr05*	46621455	(C/T)
CakSNP9367	*Kabuli*	*Ca_Kabuli_Chr05*	46668462	(T/C)
CakSNP9368	*Kabuli*	*Ca_Kabuli_Chr05*	46792131	(C/T)
CakSNP9369	*Kabuli*	*Ca_Kabuli_Chr05*	46945916	(G/A)
CakSNP9370	*Kabuli*	*Ca_Kabuli_Chr05*	46956474	(T/C)
CakSNP9371	*Kabuli*	*Ca_Kabuli_Chr05*	47061531	(C/T)
CakSNP9372	*Kabuli*	*Ca_Kabuli_Chr05*	47073011	(A/T)
CakSNP9373	*Kabuli*	*Ca_Kabuli_Chr05*	47073016	(G/C)
CakSNP9374	*Kabuli*	*Ca_Kabuli_Chr05*	47091499	(C/T)
CakSNP9375	*Kabuli*	*Ca_Kabuli_Chr05*	47151660	(C/G)
CakSNP9376	*Kabuli*	*Ca_Kabuli_Chr05*	47151840	(T/G)
CakSNP9377	*Kabuli*	*Ca_Kabuli_Chr05*	47165299	(A/C)
CakSNP9378	*Kabuli*	*Ca_Kabuli_Chr05*	47256002	(G/T)
CakSNP9379	*Kabuli*	*Ca_Kabuli_Chr05*	47256038	(A/T)
CakSNP9380	*Kabuli*	*Ca_Kabuli_Chr05*	47289004	(A/C)
CakSNP9381	*Kabuli*	*Ca_Kabuli_Chr05*	47298345	(A/G)
CakSNP9382	*Kabuli*	*Ca_Kabuli_Chr05*	47329979	(T/G)
CakSNP9383	*Kabuli*	*Ca_Kabuli_Chr05*	47362666	(T/G)
CakSNP9384	*Kabuli*	*Ca_Kabuli_Chr05*	47362814	(C/T)
CakSNP9385	*Kabuli*	*Ca_Kabuli_Chr05*	47375789	(C/T)
CakSNP9386	*Kabuli*	*Ca_Kabuli_Chr05*	47422536	(G/A)
CakSNP9387	*Kabuli*	*Ca_Kabuli_Chr05*	47518533	(G/A)
CakSNP9388	*Kabuli*	*Ca_Kabuli_Chr05*	47539345	(T/C)

**SNP IDs**	**Cultivars**	**Chromosomes/scaffolds**	**Physical positions (bp)**	**SNPs**
CakSNP9389	*Kabuli*	*Ca_Kabuli_Chr05*	47539505	(G/T)
CakSNP9390	*Kabuli*	*Ca_Kabuli_Chr05*	47570939	(T/C)
CakSNP9391	*Kabuli*	*Ca_Kabuli_Chr05*	47591327	(G/T)
CakSNP9392	*Kabuli*	*Ca_Kabuli_Chr05*	47594471	(T/C)
CakSNP9393	*Kabuli*	*Ca_Kabuli_Chr05*	47641224	(A/C)
CakSNP9394	*Kabuli*	*Ca_Kabuli_Chr05*	47641472	(T/C)
CakSNP9395	*Kabuli*	*Ca_Kabuli_Chr05*	47641628	(T/C)
CakSNP9396	*Kabuli*	*Ca_Kabuli_Chr05*	47641567	(A/G)
CakSNP9397	*Kabuli*	*Ca_Kabuli_Chr05*	47643992	(G/T)
CakSNP9398	*Kabuli*	*Ca_Kabuli_Chr05*	47669124	(A/G)
CakSNP9399	*Kabuli*	*Ca_Kabuli_Chr05*	47670649	(G/A)
CakSNP9400	*Kabuli*	*Ca_Kabuli_Chr05*	47706780	(G/A)
CakSNP9401	*Kabuli*	*Ca_Kabuli_Chr05*	47718830	(A/G)
CakSNP9402	*Kabuli*	*Ca_Kabuli_Chr05*	47718839	(A/G)
CakSNP9403	*Kabuli*	*Ca_Kabuli_Chr05*	47733645	(A/G)
CakSNP9404	*Kabuli*	*Ca_Kabuli_Chr05*	47759169	(T/G)
CakSNP9405	*Kabuli*	*Ca_Kabuli_Chr05*	47759745	(G/A)
CakSNP9406	*Kabuli*	*Ca_Kabuli_Chr05*	47761348	(A/G)
CakSNP9407	*Kabuli*	*Ca_Kabuli_Chr05*	47761322	(C/A)
CakSNP9408	*Kabuli*	*Ca_Kabuli_Chr05*	47770909	(T/G)
CakSNP9409	*Kabuli*	*Ca_Kabuli_Chr05*	47774516	(A/G)
CakSNP9410	*Kabuli*	*Ca_Kabuli_Chr05*	47774559	(C/T)
CakSNP9411	*Kabuli*	*Ca_Kabuli_Chr05*	47774718	(C/G)
CakSNP9412	*Kabuli*	*Ca_Kabuli_Chr05*	47775175	(C/T)
CakSNP9413	*Kabuli*	*Ca_Kabuli_Chr05*	47782605	(G/A)
CakSNP9414	*Kabuli*	*Ca_Kabuli_Chr05*	47782606	(C/T)
CakSNP9415	*Kabuli*	*Ca_Kabuli_Chr05*	47782697	(T/C)
CakSNP9416	*Kabuli*	*Ca_Kabuli_Chr05*	47782843	(T/C)
CakSNP9417	*Kabuli*	*Ca_Kabuli_Chr05*	47803636	(C/A)
CakSNP9418	*Kabuli*	*Ca_Kabuli_Chr05*	47812041	(C/T)
CakSNP9419	*Kabuli*	*Ca_Kabuli_Chr05*	47813712	(T/C)
CakSNP9420	*Kabuli*	*Ca_Kabuli_Chr05*	47813767	(A/G)
CakSNP9421	*Kabuli*	*Ca_Kabuli_Chr05*	47829261	(T/C)
CakSNP9422	*Kabuli*	*Ca_Kabuli_Chr05*	47829331	(T/A)
CakSNP9423	*Kabuli*	*Ca_Kabuli_Chr05*	47871094	(C/T)
CakSNP9424	*Kabuli*	*Ca_Kabuli_Chr05*	47871137	(G/A)
CakSNP9425	*Kabuli*	*Ca_Kabuli_Chr05*	47871248	(G/T)
CakSNP9426	*Kabuli*	*Ca_Kabuli_Chr05*	47871234	(A/G)
CakSNP9427	*Kabuli*	*Ca_Kabuli_Chr05*	47871194	(T/C)
CakSNP9428	*Kabuli*	*Ca_Kabuli_Chr05*	47878997	(C/T)
CakSNP9429	*Kabuli*	*Ca_Kabuli_Chr05*	47901017	(G/T)

SNP IDs	Cultivars	Chromosomes/scaffolds	Physical positions (bp)	SNPs
CakSNP9430	*Kabuli*	*Ca_Kabuli_Chr05*	47931566	(T/C)
CakSNP9431	*Kabuli*	*Ca_Kabuli_Chr05*	47941783	(C/T)
CakSNP9432	*Kabuli*	*Ca_Kabuli_Chr05*	47943743	(G/A)
CakSNP9433	*Kabuli*	*Ca_Kabuli_Chr05*	47965279	(G/A)
CakSNP9434	*Kabuli*	*Ca_Kabuli_Chr05*	47994780	(A/C)
CakSNP9435	*Kabuli*	*Ca_Kabuli_Chr05*	47997751	(A/T)
CakSNP9436	*Kabuli*	*Ca_Kabuli_Chr05*	48005123	(A/C)
CakSNP9437	*Kabuli*	*Ca_Kabuli_Chr05*	48005073	(G/A)
CakSNP9438	*Kabuli*	*Ca_Kabuli_Chr05*	48042132	(A/C)
CakSNP9439	*Kabuli*	*Ca_Kabuli_Chr05*	48063028	(A/T)
CakSNP9440	*Kabuli*	*Ca_Kabuli_Chr05*	48069744	(G/A)
CakSNP9441	*Kabuli*	*Ca_Kabuli_Chr05*	48069811	(G/C)
CakSNP9442	*Kabuli*	*Ca_Kabuli_Chr05*	48069859	(A/T)
CakSNP9443	*Kabuli*	*Ca_Kabuli_Chr05*	48069844	(T/C)
CakSNP9444	*Kabuli*	*Ca_Kabuli_Chr05*	48069833	(G/A)
CakSNP9445	*Kabuli*	*Ca_Kabuli_Chr06*	1916	(T/C)
CakSNP9446	*Kabuli*	*Ca_Kabuli_Chr06*	1847	(G/T)
CakSNP9447	*Kabuli*	*Ca_Kabuli_Chr06*	140189	(A/G)
CakSNP9448	*Kabuli*	*Ca_Kabuli_Chr06*	140279	(T/C)
CakSNP9449	*Kabuli*	*Ca_Kabuli_Chr06*	140320	(G/A)
CakSNP9450	*Kabuli*	*Ca_Kabuli_Chr06*	152513	(C/T)
CakSNP9451	*Kabuli*	*Ca_Kabuli_Chr06*	152556	(T/C)
CakSNP9452	*Kabuli*	*Ca_Kabuli_Chr06*	166823	(G/A)
CakSNP9453	*Kabuli*	*Ca_Kabuli_Chr06*	166806	(C/T)
CakSNP9454	*Kabuli*	*Ca_Kabuli_Chr06*	166959	(G/A)
CakSNP9455	*Kabuli*	*Ca_Kabuli_Chr06*	166943	(G/A)
CakSNP9456	*Kabuli*	*Ca_Kabuli_Chr06*	166938	(G/T)
CakSNP9457	*Kabuli*	*Ca_Kabuli_Chr06*	166934	(G/A)
CakSNP9458	*Kabuli*	*Ca_Kabuli_Chr06*	166932	(G/A)
CakSNP9459	*Kabuli*	*Ca_Kabuli_Chr06*	166911	(A/G)
CakSNP9460	*Kabuli*	*Ca_Kabuli_Chr06*	204368	(G/A)
CakSNP9461	*Kabuli*	*Ca_Kabuli_Chr06*	204412	(G/C)
CakSNP9462	*Kabuli*	*Ca_Kabuli_Chr06*	204438	(T/C)
CakSNP9463	*Kabuli*	*Ca_Kabuli_Chr06*	267151	(A/G)
CakSNP9464	*Kabuli*	*Ca_Kabuli_Chr06*	269269	(T/G)
CakSNP9465	*Kabuli*	*Ca_Kabuli_Chr06*	324301	(C/T)
CakSNP9466	*Kabuli*	*Ca_Kabuli_Chr06*	348283	(C/T)
CakSNP9467	*Kabuli*	*Ca_Kabuli_Chr06*	348295	(G/C)
CakSNP9468	*Kabuli*	*Ca_Kabuli_Chr06*	375684	(T/G)
CakSNP9469	*Kabuli*	*Ca_Kabuli_Chr06*	386250	(A/G)
CakSNP9470	*Kabuli*	*Ca_Kabuli_Chr06*	474489	(C/A)

**SNP IDs**	**Cultivars**	**Chromosomes/scaffolds**	**Physical positions (bp)**	**SNPs**
CakSNP9471	*Kabuli*	*Ca_Kabuli_Chr06*	505875	(T/C)
CakSNP9472	*Kabuli*	*Ca_Kabuli_Chr06*	515221	(T/G)
CakSNP9473	*Kabuli*	*Ca_Kabuli_Chr06*	548416	(A/T)
CakSNP9474	*Kabuli*	*Ca_Kabuli_Chr06*	551130	(T/G)
CakSNP9475	*Kabuli*	*Ca_Kabuli_Chr06*	561562	(A/G)
CakSNP9476	*Kabuli*	*Ca_Kabuli_Chr06*	580616	(G/C)
CakSNP9477	*Kabuli*	*Ca_Kabuli_Chr06*	588592	(C/A)
CakSNP9478	*Kabuli*	*Ca_Kabuli_Chr06*	638983	(T/A)
CakSNP9479	*Kabuli*	*Ca_Kabuli_Chr06*	645755	(G/T)
CakSNP9480	*Kabuli*	*Ca_Kabuli_Chr06*	645746	(G/T)
CakSNP9481	*Kabuli*	*Ca_Kabuli_Chr06*	647322	(A/C)
CakSNP9482	*Kabuli*	*Ca_Kabuli_Chr06*	697187	(A/G)
CakSNP9483	*Kabuli*	*Ca_Kabuli_Chr06*	697169	(A/G)
CakSNP9484	*Kabuli*	*Ca_Kabuli_Chr06*	697168	(C/T)
CakSNP9485	*Kabuli*	*Ca_Kabuli_Chr06*	697162	(A/G)
CakSNP9486	*Kabuli*	*Ca_Kabuli_Chr06*	697158	(G/A)
CakSNP9487	*Kabuli*	*Ca_Kabuli_Chr06*	697148	(C/T)
CakSNP9488	*Kabuli*	*Ca_Kabuli_Chr06*	697147	(A/G)
CakSNP9489	*Kabuli*	*Ca_Kabuli_Chr06*	697142	(G/C)
CakSNP9490	*Kabuli*	*Ca_Kabuli_Chr06*	894921	(A/G)
CakSNP9491	*Kabuli*	*Ca_Kabuli_Chr06*	902403	(G/A)
CakSNP9492	*Kabuli*	*Ca_Kabuli_Chr06*	902402	(T/C)
CakSNP9493	*Kabuli*	*Ca_Kabuli_Chr06*	949263	(C/A)
CakSNP9494	*Kabuli*	*Ca_Kabuli_Chr06*	961652	(C/T)
CakSNP9495	*Kabuli*	*Ca_Kabuli_Chr06*	961805	(C/T)
CakSNP9496	*Kabuli*	*Ca_Kabuli_Chr06*	961733	(T/A)
CakSNP9497	*Kabuli*	*Ca_Kabuli_Chr06*	997997	(T/A)
CakSNP9498	*Kabuli*	*Ca_Kabuli_Chr06*	1130293	(C/T)
CakSNP9499	*Kabuli*	*Ca_Kabuli_Chr06*	1212889	(G/A)
CakSNP9500	*Kabuli*	*Ca_Kabuli_Chr06*	1220425	(C/T)
CakSNP9501	*Kabuli*	*Ca_Kabuli_Chr06*	1220460	(G/C)
CakSNP9502	*Kabuli*	*Ca_Kabuli_Chr06*	1220465	(G/A)
CakSNP9503	*Kabuli*	*Ca_Kabuli_Chr06*	1220510	(A/T)
CakSNP9504	*Kabuli*	*Ca_Kabuli_Chr06*	1220499	(C/G)
CakSNP9505	*Kabuli*	*Ca_Kabuli_Chr06*	1287168	(C/T)
CakSNP9506	*Kabuli*	*Ca_Kabuli_Chr06*	1290413	(C/G)
CakSNP9507	*Kabuli*	*Ca_Kabuli_Chr06*	1290538	(T/G)
CakSNP9508	*Kabuli*	*Ca_Kabuli_Chr06*	1295748	(T/C)
CakSNP9509	*Kabuli*	*Ca_Kabuli_Chr06*	1312881	(T/C)
CakSNP9510	*Kabuli*	*Ca_Kabuli_Chr06*	1312932	(G/A)
CakSNP9511	*Kabuli*	*Ca_Kabuli_Chr06*	1312982	(C/A)

SNP IDs	Cultivars	Chromosomes/scaffolds	Physical positions (bp)	SNPs
CakSNP9512	*Kabuli*	*Ca_Kabuli_Chr06*	1315583	(G/C)
CakSNP9513	*Kabuli*	*Ca_Kabuli_Chr06*	1626723	(T/C)
CakSNP9514	*Kabuli*	*Ca_Kabuli_Chr06*	1626712	(C/T)
CakSNP9515	*Kabuli*	*Ca_Kabuli_Chr06*	1817599	(C/T)
CakSNP9516	*Kabuli*	*Ca_Kabuli_Chr06*	1817602	(A/T)
CakSNP9517	*Kabuli*	*Ca_Kabuli_Chr06*	1817607	(T/C)
CakSNP9518	*Kabuli*	*Ca_Kabuli_Chr06*	1817628	(A/T)
CakSNP9519	*Kabuli*	*Ca_Kabuli_Chr06*	1932114	(C/G)
CakSNP9520	*Kabuli*	*Ca_Kabuli_Chr06*	1932175	(G/A)
CakSNP9521	*Kabuli*	*Ca_Kabuli_Chr06*	1966137	(T/C)
CakSNP9522	*Kabuli*	*Ca_Kabuli_Chr06*	1978781	(C/A)
CakSNP9523	*Kabuli*	*Ca_Kabuli_Chr06*	1978864	(A/C)
CakSNP9524	*Kabuli*	*Ca_Kabuli_Chr06*	1978867	(T/A)
CakSNP9525	*Kabuli*	*Ca_Kabuli_Chr06*	2037575	(G/T)
CakSNP9526	*Kabuli*	*Ca_Kabuli_Chr06*	2037599	(A/G)
CakSNP9527	*Kabuli*	*Ca_Kabuli_Chr06*	2037620	(T/G)
CakSNP9528	*Kabuli*	*Ca_Kabuli_Chr06*	2037623	(T/G)
CakSNP9529	*Kabuli*	*Ca_Kabuli_Chr06*	2037626	(C/T)
CakSNP9530	*Kabuli*	*Ca_Kabuli_Chr06*	2050907	(A/G)
CakSNP9531	*Kabuli*	*Ca_Kabuli_Chr06*	2061459	(A/G)
CakSNP9532	*Kabuli*	*Ca_Kabuli_Chr06*	2078416	(T/A)
CakSNP9533	*Kabuli*	*Ca_Kabuli_Chr06*	2079137	(T/G)
CakSNP9534	*Kabuli*	*Ca_Kabuli_Chr06*	2079186	(G/T)
CakSNP9535	*Kabuli*	*Ca_Kabuli_Chr06*	2083784	(A/T)
CakSNP9536	*Kabuli*	*Ca_Kabuli_Chr06*	2083805	(G/A)
CakSNP9537	*Kabuli*	*Ca_Kabuli_Chr06*	2083813	(T/C)
CakSNP9538	*Kabuli*	*Ca_Kabuli_Chr06*	2126667	(C/T)
CakSNP9539	*Kabuli*	*Ca_Kabuli_Chr06*	2126655	(G/A)
CakSNP9540	*Kabuli*	*Ca_Kabuli_Chr06*	2140189	(G/T)
CakSNP9541	*Kabuli*	*Ca_Kabuli_Chr06*	2140406	(G/A)
CakSNP9542	*Kabuli*	*Ca_Kabuli_Chr06*	2193748	(T/A)
CakSNP9543	*Kabuli*	*Ca_Kabuli_Chr06*	2193763	(A/C)
CakSNP9544	*Kabuli*	*Ca_Kabuli_Chr06*	2193803	(G/A)
CakSNP9545	*Kabuli*	*Ca_Kabuli_Chr06*	2214131	(T/A)
CakSNP9546	*Kabuli*	*Ca_Kabuli_Chr06*	2214178	(T/C)
CakSNP9547	*Kabuli*	*Ca_Kabuli_Chr06*	2214247	(A/G)
CakSNP9548	*Kabuli*	*Ca_Kabuli_Chr06*	2223544	(A/T)
CakSNP9549	*Kabuli*	*Ca_Kabuli_Chr06*	2223578	(T/G)
CakSNP9550	*Kabuli*	*Ca_Kabuli_Chr06*	2223664	(A/C)
CakSNP9551	*Kabuli*	*Ca_Kabuli_Chr06*	2244901	(A/C)
CakSNP9552	*Kabuli*	*Ca_Kabuli_Chr06*	2275505	(A/G)

SNP IDs	Cultivars	Chromosomes/scaffolds	Physical positions (bp)	SNPs
CakSNP9553	*Kabuli*	*Ca_Kabuli_Chr06*	2275524	(G/A)
CakSNP9554	*Kabuli*	*Ca_Kabuli_Chr06*	2275553	(A/G)
CakSNP9555	*Kabuli*	*Ca_Kabuli_Chr06*	2280734	(T/A)
CakSNP9556	*Kabuli*	*Ca_Kabuli_Chr06*	2280752	(A/G)
CakSNP9557	*Kabuli*	*Ca_Kabuli_Chr06*	2286205	(A/G)
CakSNP9558	*Kabuli*	*Ca_Kabuli_Chr06*	2286237	(C/T)
CakSNP9559	*Kabuli*	*Ca_Kabuli_Chr06*	2294911	(T/G)
CakSNP9560	*Kabuli*	*Ca_Kabuli_Chr06*	2303123	(T/G)
CakSNP9561	*Kabuli*	*Ca_Kabuli_Chr06*	2313683	(A/C)
CakSNP9562	*Kabuli*	*Ca_Kabuli_Chr06*	2313828	(G/A)
CakSNP9563	*Kabuli*	*Ca_Kabuli_Chr06*	2314029	(T/C)
CakSNP9564	*Kabuli*	*Ca_Kabuli_Chr06*	2327842	(A/G)
CakSNP9565	*Kabuli*	*Ca_Kabuli_Chr06*	2462481	(C/T)
CakSNP9566	*Kabuli*	*Ca_Kabuli_Chr06*	2502485	(A/G)
CakSNP9567	*Kabuli*	*Ca_Kabuli_Chr06*	2527768	(A/G)
CakSNP9568	*Kabuli*	*Ca_Kabuli_Chr06*	2542942	(A/T)
CakSNP9569	*Kabuli*	*Ca_Kabuli_Chr06*	2542981	(T/C)
CakSNP9570	*Kabuli*	*Ca_Kabuli_Chr06*	2543096	(C/T)
CakSNP9571	*Kabuli*	*Ca_Kabuli_Chr06*	2543062	(A/G)
CakSNP9572	*Kabuli*	*Ca_Kabuli_Chr06*	2548412	(A/G)
CakSNP9573	*Kabuli*	*Ca_Kabuli_Chr06*	2549997	(C/T)
CakSNP9574	*Kabuli*	*Ca_Kabuli_Chr06*	2549991	(T/C)
CakSNP9575	*Kabuli*	*Ca_Kabuli_Chr06*	2550154	(A/C)
CakSNP9576	*Kabuli*	*Ca_Kabuli_Chr06*	2623370	(T/C)
CakSNP9577	*Kabuli*	*Ca_Kabuli_Chr06*	2625793	(C/G)
CakSNP9578	*Kabuli*	*Ca_Kabuli_Chr06*	2633941	(A/T)
CakSNP9579	*Kabuli*	*Ca_Kabuli_Chr06*	2639558	(T/A)
CakSNP9580	*Kabuli*	*Ca_Kabuli_Chr06*	2639606	(A/G)
CakSNP9581	*Kabuli*	*Ca_Kabuli_Chr06*	2639607	(T/C)
CakSNP9582	*Kabuli*	*Ca_Kabuli_Chr06*	2676594	(C/T)
CakSNP9583	*Kabuli*	*Ca_Kabuli_Chr06*	2714894	(T/C)
CakSNP9584	*Kabuli*	*Ca_Kabuli_Chr06*	2714897	(C/A)
CakSNP9585	*Kabuli*	*Ca_Kabuli_Chr06*	2714918	(T/G)
CakSNP9586	*Kabuli*	*Ca_Kabuli_Chr06*	2717150	(G/A)
CakSNP9587	*Kabuli*	*Ca_Kabuli_Chr06*	2740630	(A/G)
CakSNP9588	*Kabuli*	*Ca_Kabuli_Chr06*	2740765	(A/G)
CakSNP9589	*Kabuli*	*Ca_Kabuli_Chr06*	2740784	(T/C)
CakSNP9590	*Kabuli*	*Ca_Kabuli_Chr06*	2741185	(G/A)
CakSNP9591	*Kabuli*	*Ca_Kabuli_Chr06*	2777609	(A/G)
CakSNP9592	*Kabuli*	*Ca_Kabuli_Chr06*	2802942	(C/A)
CakSNP9593	*Kabuli*	*Ca_Kabuli_Chr06*	2811345	(A/G)

SNP IDs	Cultivars	Chromosomes/scaffolds	Physical positions (bp)	SNPs
CakSNP9594	*Kabuli*	*Ca_Kabuli_Chr06*	2812794	(A/C)
CakSNP9595	*Kabuli*	*Ca_Kabuli_Chr06*	2812719	(G/A)
CakSNP9596	*Kabuli*	*Ca_Kabuli_Chr06*	2813244	(G/A)
CakSNP9597	*Kabuli*	*Ca_Kabuli_Chr06*	2821836	(G/T)
CakSNP9598	*Kabuli*	*Ca_Kabuli_Chr06*	2846051	(C/T)
CakSNP9599	*Kabuli*	*Ca_Kabuli_Chr06*	2846018	(T/C)
CakSNP9600	*Kabuli*	*Ca_Kabuli_Chr06*	2855691	(C/G)
CakSNP9601	*Kabuli*	*Ca_Kabuli_Chr06*	2864713	(G/A)
CakSNP9602	*Kabuli*	*Ca_Kabuli_Chr06*	2919355	(A/C)
CakSNP9603	*Kabuli*	*Ca_Kabuli_Chr06*	2925908	(A/G)
CakSNP9604	*Kabuli*	*Ca_Kabuli_Chr06*	2925962	(G/C)
CakSNP9605	*Kabuli*	*Ca_Kabuli_Chr06*	2926006	(T/C)
CakSNP9606	*Kabuli*	*Ca_Kabuli_Chr06*	2927224	(G/A)
CakSNP9607	*Kabuli*	*Ca_Kabuli_Chr06*	2927255	(C/A)
CakSNP9608	*Kabuli*	*Ca_Kabuli_Chr06*	2927271	(G/A)
CakSNP9609	*Kabuli*	*Ca_Kabuli_Chr06*	2927386	(C/G)
CakSNP9610	*Kabuli*	*Ca_Kabuli_Chr06*	2927387	(T/C)
CakSNP9611	*Kabuli*	*Ca_Kabuli_Chr06*	2929355	(C/T)
CakSNP9612	*Kabuli*	*Ca_Kabuli_Chr06*	2930453	(T/C)
CakSNP9613	*Kabuli*	*Ca_Kabuli_Chr06*	2930459	(C/T)
CakSNP9614	*Kabuli*	*Ca_Kabuli_Chr06*	2930504	(T/C)
CakSNP9615	*Kabuli*	*Ca_Kabuli_Chr06*	2932029	(T/C)
CakSNP9616	*Kabuli*	*Ca_Kabuli_Chr06*	2932179	(T/G)
CakSNP9617	*Kabuli*	*Ca_Kabuli_Chr06*	2932168	(A/C)
CakSNP9618	*Kabuli*	*Ca_Kabuli_Chr06*	2932143	(C/A)
CakSNP9619	*Kabuli*	*Ca_Kabuli_Chr06*	2932130	(T/G)
CakSNP9620	*Kabuli*	*Ca_Kabuli_Chr06*	2932777	(A/G)
CakSNP9621	*Kabuli*	*Ca_Kabuli_Chr06*	2972319	(C/T)
CakSNP9622	*Kabuli*	*Ca_Kabuli_Chr06*	2972301	(T/C)
CakSNP9623	*Kabuli*	*Ca_Kabuli_Chr06*	2973741	(A/G)
CakSNP9624	*Kabuli*	*Ca_Kabuli_Chr06*	2973756	(A/C)
CakSNP9625	*Kabuli*	*Ca_Kabuli_Chr06*	3060508	(G/A)
CakSNP9626	*Kabuli*	*Ca_Kabuli_Chr06*	3060502	(G/A)
CakSNP9627	*Kabuli*	*Ca_Kabuli_Chr06*	3288481	(C/A)
CakSNP9628	*Kabuli*	*Ca_Kabuli_Chr06*	3288444	(G/A)
CakSNP9629	*Kabuli*	*Ca_Kabuli_Chr06*	3288441	(A/G)
CakSNP9630	*Kabuli*	*Ca_Kabuli_Chr06*	3288505	(G/T)
CakSNP9631	*Kabuli*	*Ca_Kabuli_Chr06*	3297086	(G/A)
CakSNP9632	*Kabuli*	*Ca_Kabuli_Chr06*	3298952	(A/T)
CakSNP9633	*Kabuli*	*Ca_Kabuli_Chr06*	3395024	(A/G)
CakSNP9634	*Kabuli*	*Ca_Kabuli_Chr06*	3395253	(G/A)

SNP IDs	Cultivars	Chromosomes/scaffolds	Physical positions (bp)	SNPs
CakSNP9635	*Kabuli*	*Ca_Kabuli_Chr06*	3395370	(C/T)
CakSNP9636	*Kabuli*	*Ca_Kabuli_Chr06*	3397650	(A/G)
CakSNP9637	*Kabuli*	*Ca_Kabuli_Chr06*	3401260	(A/G)
CakSNP9638	*Kabuli*	*Ca_Kabuli_Chr06*	3401272	(G/A)
CakSNP9639	*Kabuli*	*Ca_Kabuli_Chr06*	3401277	(C/T)
CakSNP9640	*Kabuli*	*Ca_Kabuli_Chr06*	3482411	(A/G)
CakSNP9641	*Kabuli*	*Ca_Kabuli_Chr06*	3505662	(C/A)
CakSNP9642	*Kabuli*	*Ca_Kabuli_Chr06*	3530094	(T/C)
CakSNP9643	*Kabuli*	*Ca_Kabuli_Chr06*	3530137	(A/G)
CakSNP9644	*Kabuli*	*Ca_Kabuli_Chr06*	3548121	(C/A)
CakSNP9645	*Kabuli*	*Ca_Kabuli_Chr06*	3548114	(A/C)
CakSNP9646	*Kabuli*	*Ca_Kabuli_Chr06*	3786843	(A/G)
CakSNP9647	*Kabuli*	*Ca_Kabuli_Chr06*	3787239	(C/A)
CakSNP9648	*Kabuli*	*Ca_Kabuli_Chr06*	3829070	(C/A)
CakSNP9649	*Kabuli*	*Ca_Kabuli_Chr06*	3962516	(A/C)
CakSNP9650	*Kabuli*	*Ca_Kabuli_Chr06*	3985519	(G/A)
CakSNP9651	*Kabuli*	*Ca_Kabuli_Chr06*	3992419	(C/T)
CakSNP9652	*Kabuli*	*Ca_Kabuli_Chr06*	4112085	(T/C)
CakSNP9653	*Kabuli*	*Ca_Kabuli_Chr06*	4226957	(G/A)
CakSNP9654	*Kabuli*	*Ca_Kabuli_Chr06*	4226947	(A/T)
CakSNP9655	*Kabuli*	*Ca_Kabuli_Chr06*	4226924	(G/T)
CakSNP9656	*Kabuli*	*Ca_Kabuli_Chr06*	4226951	(C/A)
CakSNP9657	*Kabuli*	*Ca_Kabuli_Chr06*	4228728	(T/C)
CakSNP9658	*Kabuli*	*Ca_Kabuli_Chr06*	4228759	(C/G)
CakSNP9659	*Kabuli*	*Ca_Kabuli_Chr06*	4228884	(T/C)
CakSNP9660	*Kabuli*	*Ca_Kabuli_Chr06*	4344604	(T/C)
CakSNP9661	*Kabuli*	*Ca_Kabuli_Chr06*	4350822	(A/T)
CakSNP9662	*Kabuli*	*Ca_Kabuli_Chr06*	4350867	(A/C)
CakSNP9663	*Kabuli*	*Ca_Kabuli_Chr06*	4350889	(A/C)
CakSNP9664	*Kabuli*	*Ca_Kabuli_Chr06*	4440034	(T/G)
CakSNP9665	*Kabuli*	*Ca_Kabuli_Chr06*	4562263	(C/A)
CakSNP9666	*Kabuli*	*Ca_Kabuli_Chr06*	4562264	(C/G)
CakSNP9667	*Kabuli*	*Ca_Kabuli_Chr06*	4601303	(A/C)
CakSNP9668	*Kabuli*	*Ca_Kabuli_Chr06*	4678523	(A/C)
CakSNP9669	*Kabuli*	*Ca_Kabuli_Chr06*	4681686	(C/A)
CakSNP9670	*Kabuli*	*Ca_Kabuli_Chr06*	4743563	(C/G)
CakSNP9671	*Kabuli*	*Ca_Kabuli_Chr06*	4743608	(C/T)
CakSNP9672	*Kabuli*	*Ca_Kabuli_Chr06*	4807908	(G/C)
CakSNP9673	*Kabuli*	*Ca_Kabuli_Chr06*	4858109	(T/C)
CakSNP9674	*Kabuli*	*Ca_Kabuli_Chr06*	4861184	(T/G)
CakSNP9675	*Kabuli*	*Ca_Kabuli_Chr06*	4981078	(G/T)

**SNP IDs**	**Cultivars**	**Chromosomes/scaffolds**	**Physical positions (bp)**	**SNPs**
CakSNP9676	*Kabuli*	*Ca_Kabuli_Chr06*	5185380	(A/G)
CakSNP9677	*Kabuli*	*Ca_Kabuli_Chr06*	5309445	(G/A)
CakSNP9678	*Kabuli*	*Ca_Kabuli_Chr06*	5338761	(T/G)
CakSNP9679	*Kabuli*	*Ca_Kabuli_Chr06*	5341510	(C/A)
CakSNP9680	*Kabuli*	*Ca_Kabuli_Chr06*	5366094	(A/G)
CakSNP9681	*Kabuli*	*Ca_Kabuli_Chr06*	5368455	(T/G)
CakSNP9682	*Kabuli*	*Ca_Kabuli_Chr06*	5392161	(G/A)
CakSNP9683	*Kabuli*	*Ca_Kabuli_Chr06*	5392185	(T/C)
CakSNP9684	*Kabuli*	*Ca_Kabuli_Chr06*	5396888	(T/G)
CakSNP9685	*Kabuli*	*Ca_Kabuli_Chr06*	5396890	(T/G)
CakSNP9686	*Kabuli*	*Ca_Kabuli_Chr06*	5396897	(T/G)
CakSNP9687	*Kabuli*	*Ca_Kabuli_Chr06*	5396913	(C/T)
CakSNP9688	*Kabuli*	*Ca_Kabuli_Chr06*	5396916	(C/T)
CakSNP9689	*Kabuli*	*Ca_Kabuli_Chr06*	5401307	(T/C)
CakSNP9690	*Kabuli*	*Ca_Kabuli_Chr06*	5453512	(A/G)
CakSNP9691	*Kabuli*	*Ca_Kabuli_Chr06*	5498898	(C/T)
CakSNP9692	*Kabuli*	*Ca_Kabuli_Chr06*	5500791	(C/T)
CakSNP9693	*Kabuli*	*Ca_Kabuli_Chr06*	5501363	(T/C)
CakSNP9694	*Kabuli*	*Ca_Kabuli_Chr06*	5541264	(A/G)
CakSNP9695	*Kabuli*	*Ca_Kabuli_Chr06*	5567967	(T/C)
CakSNP9696	*Kabuli*	*Ca_Kabuli_Chr06*	5589373	(T/C)
CakSNP9697	*Kabuli*	*Ca_Kabuli_Chr06*	5616372	(C/T)
CakSNP9698	*Kabuli*	*Ca_Kabuli_Chr06*	5616482	(A/G)
CakSNP9699	*Kabuli*	*Ca_Kabuli_Chr06*	5616454	(T/A)
CakSNP9700	*Kabuli*	*Ca_Kabuli_Chr06*	5628948	(C/A)
CakSNP9701	*Kabuli*	*Ca_Kabuli_Chr06*	5628956	(T/G)
CakSNP9702	*Kabuli*	*Ca_Kabuli_Chr06*	5692120	(C/A)
CakSNP9703	*Kabuli*	*Ca_Kabuli_Chr06*	5694397	(G/T)
CakSNP9704	*Kabuli*	*Ca_Kabuli_Chr06*	5701004	(A/G)
CakSNP9705	*Kabuli*	*Ca_Kabuli_Chr06*	5704845	(T/C)
CakSNP9706	*Kabuli*	*Ca_Kabuli_Chr06*	5704809	(T/A)
CakSNP9707	*Kabuli*	*Ca_Kabuli_Chr06*	5706649	(C/T)
CakSNP9708	*Kabuli*	*Ca_Kabuli_Chr06*	5841443	(G/C)
CakSNP9709	*Kabuli*	*Ca_Kabuli_Chr06*	5871537	(G/A)
CakSNP9710	*Kabuli*	*Ca_Kabuli_Chr06*	5886393	(A/G)
CakSNP9711	*Kabuli*	*Ca_Kabuli_Chr06*	5888423	(A/G)
CakSNP9712	*Kabuli*	*Ca_Kabuli_Chr06*	5924967	(G/T)
CakSNP9713	*Kabuli*	*Ca_Kabuli_Chr06*	5925028	(G/T)
CakSNP9714	*Kabuli*	*Ca_Kabuli_Chr06*	6110805	(T/C)
CakSNP9715	*Kabuli*	*Ca_Kabuli_Chr06*	6119644	(C/A)
CakSNP9716	*Kabuli*	*Ca_Kabuli_Chr06*	6131564	(G/A)

SNP IDs	Cultivars	Chromosomes/scaffolds	Physical positions (bp)	SNPs
CakSNP9717	*Kabuli*	*Ca_Kabuli_Chr06*	6131518	(G/T)
CakSNP9718	*Kabuli*	*Ca_Kabuli_Chr06*	6131514	(G/T)
CakSNP9719	*Kabuli*	*Ca_Kabuli_Chr06*	6169291	(A/G)
CakSNP9720	*Kabuli*	*Ca_Kabuli_Chr06*	6192895	(A/T)
CakSNP9721	*Kabuli*	*Ca_Kabuli_Chr06*	6193105	(G/C)
CakSNP9722	*Kabuli*	*Ca_Kabuli_Chr06*	6216766	(T/G)
CakSNP9723	*Kabuli*	*Ca_Kabuli_Chr06*	6216746	(G/C)
CakSNP9724	*Kabuli*	*Ca_Kabuli_Chr06*	6227887	(A/G)
CakSNP9725	*Kabuli*	*Ca_Kabuli_Chr06*	6248260	(T/C)
CakSNP9726	*Kabuli*	*Ca_Kabuli_Chr06*	6248203	(A/G)
CakSNP9727	*Kabuli*	*Ca_Kabuli_Chr06*	6415818	(A/G)
CakSNP9728	*Kabuli*	*Ca_Kabuli_Chr06*	6443828	(C/G)
CakSNP9729	*Kabuli*	*Ca_Kabuli_Chr06*	6444169	(A/G)
CakSNP9730	*Kabuli*	*Ca_Kabuli_Chr06*	6444226	(T/G)
CakSNP9731	*Kabuli*	*Ca_Kabuli_Chr06*	6444243	(G/A)
CakSNP9732	*Kabuli*	*Ca_Kabuli_Chr06*	6444220	(G/A)
CakSNP9733	*Kabuli*	*Ca_Kabuli_Chr06*	6517785	(T/C)
CakSNP9734	*Kabuli*	*Ca_Kabuli_Chr06*	6517849	(C/T)
CakSNP9735	*Kabuli*	*Ca_Kabuli_Chr06*	6538236	(C/T)
CakSNP9736	*Kabuli*	*Ca_Kabuli_Chr06*	6604713	(G/A)
CakSNP9737	*Kabuli*	*Ca_Kabuli_Chr06*	6604766	(C/A)
CakSNP9738	*Kabuli*	*Ca_Kabuli_Chr06*	6607773	(A/G)
CakSNP9739	*Kabuli*	*Ca_Kabuli_Chr06*	6631216	(C/T)
CakSNP9740	*Kabuli*	*Ca_Kabuli_Chr06*	6654917	(T/C)
CakSNP9741	*Kabuli*	*Ca_Kabuli_Chr06*	6764292	(G/A)
CakSNP9742	*Kabuli*	*Ca_Kabuli_Chr06*	6779147	(G/C)
CakSNP9743	*Kabuli*	*Ca_Kabuli_Chr06*	6779982	(C/T)
CakSNP9744	*Kabuli*	*Ca_Kabuli_Chr06*	6792012	(C/T)
CakSNP9745	*Kabuli*	*Ca_Kabuli_Chr06*	6800935	(A/T)
CakSNP9746	*Kabuli*	*Ca_Kabuli_Chr06*	6800927	(G/T)
CakSNP9747	*Kabuli*	*Ca_Kabuli_Chr06*	6800877	(T/A)
CakSNP9748	*Kabuli*	*Ca_Kabuli_Chr06*	6802259	(C/T)
CakSNP9749	*Kabuli*	*Ca_Kabuli_Chr06*	6802651	(A/G)
CakSNP9750	*Kabuli*	*Ca_Kabuli_Chr06*	6803433	(A/G)
CakSNP9751	*Kabuli*	*Ca_Kabuli_Chr06*	6815594	(G/A)
CakSNP9752	*Kabuli*	*Ca_Kabuli_Chr06*	6840803	(T/C)
CakSNP9753	*Kabuli*	*Ca_Kabuli_Chr06*	6853983	(G/A)
CakSNP9754	*Kabuli*	*Ca_Kabuli_Chr06*	6871801	(A/G)
CakSNP9755	*Kabuli*	*Ca_Kabuli_Chr06*	6877020	(C/G)
CakSNP9756	*Kabuli*	*Ca_Kabuli_Chr06*	6877088	(C/A)
CakSNP9757	*Kabuli*	*Ca_Kabuli_Chr06*	6877163	(T/C)

SNP IDs	Cultivars	Chromosomes/scaffolds	Physical positions (bp)	SNPs
CakSNP9758	*Kabuli*	*Ca_Kabuli_Chr06*	6877246	(C/A)
CakSNP9759	*Kabuli*	*Ca_Kabuli_Chr06*	6877284	(C/T)
CakSNP9760	*Kabuli*	*Ca_Kabuli_Chr06*	6899977	(G/A)
CakSNP9761	*Kabuli*	*Ca_Kabuli_Chr06*	6899949	(T/G)
CakSNP9762	*Kabuli*	*Ca_Kabuli_Chr06*	6913639	(T/C)
CakSNP9763	*Kabuli*	*Ca_Kabuli_Chr06*	6913622	(A/C)
CakSNP9764	*Kabuli*	*Ca_Kabuli_Chr06*	6913695	(G/A)
CakSNP9765	*Kabuli*	*Ca_Kabuli_Chr06*	6913825	(G/A)
CakSNP9766	*Kabuli*	*Ca_Kabuli_Chr06*	6913821	(A/T)
CakSNP9767	*Kabuli*	*Ca_Kabuli_Chr06*	6954468	(A/G)
CakSNP9768	*Kabuli*	*Ca_Kabuli_Chr06*	6954480	(A/G)
CakSNP9769	*Kabuli*	*Ca_Kabuli_Chr06*	7026495	(G/A)
CakSNP9770	*Kabuli*	*Ca_Kabuli_Chr06*	7026481	(G/C)
CakSNP9771	*Kabuli*	*Ca_Kabuli_Chr06*	7039578	(C/T)
CakSNP9772	*Kabuli*	*Ca_Kabuli_Chr06*	7040231	(A/C)
CakSNP9773	*Kabuli*	*Ca_Kabuli_Chr06*	7138381	(G/A)
CakSNP9774	*Kabuli*	*Ca_Kabuli_Chr06*	7259533	(T/A)
CakSNP9775	*Kabuli*	*Ca_Kabuli_Chr06*	7271634	(C/T)
CakSNP9776	*Kabuli*	*Ca_Kabuli_Chr06*	7332579	(T/C)
CakSNP9777	*Kabuli*	*Ca_Kabuli_Chr06*	7332585	(T/C)
CakSNP9778	*Kabuli*	*Ca_Kabuli_Chr06*	7332762	(C/T)
CakSNP9779	*Kabuli*	*Ca_Kabuli_Chr06*	7349517	(A/C)
CakSNP9780	*Kabuli*	*Ca_Kabuli_Chr06*	7377229	(G/A)
CakSNP9781	*Kabuli*	*Ca_Kabuli_Chr06*	7377276	(G/A)
CakSNP9782	*Kabuli*	*Ca_Kabuli_Chr06*	7406219	(A/G)
CakSNP9783	*Kabuli*	*Ca_Kabuli_Chr06*	7406233	(G/A)
CakSNP9784	*Kabuli*	*Ca_Kabuli_Chr06*	7406301	(T/C)
CakSNP9785	*Kabuli*	*Ca_Kabuli_Chr06*	7531353	(G/A)
CakSNP9786	*Kabuli*	*Ca_Kabuli_Chr06*	7531445	(G/A)
CakSNP9787	*Kabuli*	*Ca_Kabuli_Chr06*	7531456	(T/A)
CakSNP9788	*Kabuli*	*Ca_Kabuli_Chr06*	7534360	(G/T)
CakSNP9789	*Kabuli*	*Ca_Kabuli_Chr06*	7543580	(C/T)
CakSNP9790	*Kabuli*	*Ca_Kabuli_Chr06*	7554094	(T/A)
CakSNP9791	*Kabuli*	*Ca_Kabuli_Chr06*	7571950	(T/C)
CakSNP9792	*Kabuli*	*Ca_Kabuli_Chr06*	7582726	(G/T)
CakSNP9793	*Kabuli*	*Ca_Kabuli_Chr06*	7600817	(G/A)
CakSNP9794	*Kabuli*	*Ca_Kabuli_Chr06*	7603195	(G/A)
CakSNP9795	*Kabuli*	*Ca_Kabuli_Chr06*	7603178	(A/C)
CakSNP9796	*Kabuli*	*Ca_Kabuli_Chr06*	7609814	(C/T)
CakSNP9797	*Kabuli*	*Ca_Kabuli_Chr06*	7647907	(T/G)
CakSNP9798	*Kabuli*	*Ca_Kabuli_Chr06*	7647931	(G/A)

**SNP IDs**	**Cultivars**	**Chromosomes/scaffolds**	**Physical positions (bp)**	**SNPs**
CakSNP9799	*Kabuli*	*Ca_Kabuli_Chr06*	7661930	(T/G)
CakSNP9800	*Kabuli*	*Ca_Kabuli_Chr06*	7661924	(T/C)
CakSNP9801	*Kabuli*	*Ca_Kabuli_Chr06*	7667301	(T/C)
CakSNP9802	*Kabuli*	*Ca_Kabuli_Chr06*	7667328	(A/T)
CakSNP9803	*Kabuli*	*Ca_Kabuli_Chr06*	7673403	(C/T)
CakSNP9804	*Kabuli*	*Ca_Kabuli_Chr06*	7694609	(A/T)
CakSNP9805	*Kabuli*	*Ca_Kabuli_Chr06*	7694625	(G/T)
CakSNP9806	*Kabuli*	*Ca_Kabuli_Chr06*	7694696	(T/C)
CakSNP9807	*Kabuli*	*Ca_Kabuli_Chr06*	7744730	(A/G)
CakSNP9808	*Kabuli*	*Ca_Kabuli_Chr06*	7744752	(T/C)
CakSNP9809	*Kabuli*	*Ca_Kabuli_Chr06*	7745641	(A/G)
CakSNP9810	*Kabuli*	*Ca_Kabuli_Chr06*	7747254	(G/A)
CakSNP9811	*Kabuli*	*Ca_Kabuli_Chr06*	7764968	(A/G)
CakSNP9812	*Kabuli*	*Ca_Kabuli_Chr06*	7770357	(C/T)
CakSNP9813	*Kabuli*	*Ca_Kabuli_Chr06*	7805886	(G/T)
CakSNP9814	*Kabuli*	*Ca_Kabuli_Chr06*	7821850	(A/G)
CakSNP9815	*Kabuli*	*Ca_Kabuli_Chr06*	7822666	(C/A)
CakSNP9816	*Kabuli*	*Ca_Kabuli_Chr06*	7829485	(G/A)
CakSNP9817	*Kabuli*	*Ca_Kabuli_Chr06*	7829627	(T/G)
CakSNP9818	*Kabuli*	*Ca_Kabuli_Chr06*	7831214	(C/T)
CakSNP9819	*Kabuli*	*Ca_Kabuli_Chr06*	7831409	(C/T)
CakSNP9820	*Kabuli*	*Ca_Kabuli_Chr06*	7831585	(G/A)
CakSNP9821	*Kabuli*	*Ca_Kabuli_Chr06*	7831874	(A/G)
CakSNP9822	*Kabuli*	*Ca_Kabuli_Chr06*	7831898	(G/A)
CakSNP9823	*Kabuli*	*Ca_Kabuli_Chr06*	7831899	(G/A)
CakSNP9824	*Kabuli*	*Ca_Kabuli_Chr06*	7832029	(C/T)
CakSNP9825	*Kabuli*	*Ca_Kabuli_Chr06*	7832023	(C/T)
CakSNP9826	*Kabuli*	*Ca_Kabuli_Chr06*	7834595	(C/T)
CakSNP9827	*Kabuli*	*Ca_Kabuli_Chr06*	7834922	(G/A)
CakSNP9828	*Kabuli*	*Ca_Kabuli_Chr06*	7834895	(G/T)
CakSNP9829	*Kabuli*	*Ca_Kabuli_Chr06*	7834976	(G/A)
CakSNP9830	*Kabuli*	*Ca_Kabuli_Chr06*	7835427	(G/A)
CakSNP9831	*Kabuli*	*Ca_Kabuli_Chr06*	7835738	(C/T)
CakSNP9832	*Kabuli*	*Ca_Kabuli_Chr06*	7835984	(T/A)
CakSNP9833	*Kabuli*	*Ca_Kabuli_Chr06*	7851000	(G/C)
CakSNP9834	*Kabuli*	*Ca_Kabuli_Chr06*	7929263	(C/T)
CakSNP9835	*Kabuli*	*Ca_Kabuli_Chr06*	7929338	(T/C)
CakSNP9836	*Kabuli*	*Ca_Kabuli_Chr06*	7929339	(G/A)
CakSNP9837	*Kabuli*	*Ca_Kabuli_Chr06*	7929348	(C/T)
CakSNP9838	*Kabuli*	*Ca_Kabuli_Chr06*	7929384	(T/C)
CakSNP9839	*Kabuli*	*Ca_Kabuli_Chr06*	7929493	(A/G)

SNP IDs	Cultivars	Chromosomes/scaffolds	Physical positions (bp)	SNPs
CakSNP9840	*Kabuli*	*Ca_Kabuli_Chr06*	7929629	(G/A)
CakSNP9841	*Kabuli*	*Ca_Kabuli_Chr06*	7929628	(C/T)
CakSNP9842	*Kabuli*	*Ca_Kabuli_Chr06*	7929607	(A/C)
CakSNP9843	*Kabuli*	*Ca_Kabuli_Chr06*	7939277	(T/A)
CakSNP9844	*Kabuli*	*Ca_Kabuli_Chr06*	7939281	(T/G)
CakSNP9845	*Kabuli*	*Ca_Kabuli_Chr06*	7939465	(A/G)
CakSNP9846	*Kabuli*	*Ca_Kabuli_Chr06*	7943546	(C/G)
CakSNP9847	*Kabuli*	*Ca_Kabuli_Chr06*	7994732	(G/T)
CakSNP9848	*Kabuli*	*Ca_Kabuli_Chr06*	8010016	(T/C)
CakSNP9849	*Kabuli*	*Ca_Kabuli_Chr06*	8011685	(G/T)
CakSNP9850	*Kabuli*	*Ca_Kabuli_Chr06*	8029226	(C/T)
CakSNP9851	*Kabuli*	*Ca_Kabuli_Chr06*	8041582	(A/G)
CakSNP9852	*Kabuli*	*Ca_Kabuli_Chr06*	8052987	(G/C)
CakSNP9853	*Kabuli*	*Ca_Kabuli_Chr06*	8076394	(T/C)
CakSNP9854	*Kabuli*	*Ca_Kabuli_Chr06*	8076446	(G/A)
CakSNP9855	*Kabuli*	*Ca_Kabuli_Chr06*	8081800	(C/G)
CakSNP9856	*Kabuli*	*Ca_Kabuli_Chr06*	8084950	(T/A)
CakSNP9857	*Kabuli*	*Ca_Kabuli_Chr06*	8090739	(T/A)
CakSNP9858	*Kabuli*	*Ca_Kabuli_Chr06*	8090980	(A/G)
CakSNP9859	*Kabuli*	*Ca_Kabuli_Chr06*	8091050	(A/G)
CakSNP9860	*Kabuli*	*Ca_Kabuli_Chr06*	8139434	(G/A)
CakSNP9861	*Kabuli*	*Ca_Kabuli_Chr06*	8139446	(C/A)
CakSNP9862	*Kabuli*	*Ca_Kabuli_Chr06*	8166317	(T/C)
CakSNP9863	*Kabuli*	*Ca_Kabuli_Chr06*	8166339	(T/C)
CakSNP9864	*Kabuli*	*Ca_Kabuli_Chr06*	8166435	(A/G)
CakSNP9865	*Kabuli*	*Ca_Kabuli_Chr06*	8166462	(A/T)
CakSNP9866	*Kabuli*	*Ca_Kabuli_Chr06*	8170583	(C/T)
CakSNP9867	*Kabuli*	*Ca_Kabuli_Chr06*	8170633	(A/G)
CakSNP9868	*Kabuli*	*Ca_Kabuli_Chr06*	8179815	(T/C)
CakSNP9869	*Kabuli*	*Ca_Kabuli_Chr06*	8179797	(C/T)
CakSNP9870	*Kabuli*	*Ca_Kabuli_Chr06*	8221204	(C/A)
CakSNP9871	*Kabuli*	*Ca_Kabuli_Chr06*	8222666	(T/C)
CakSNP9872	*Kabuli*	*Ca_Kabuli_Chr06*	8222685	(A/T)
CakSNP9873	*Kabuli*	*Ca_Kabuli_Chr06*	8223986	(C/G)
CakSNP9874	*Kabuli*	*Ca_Kabuli_Chr06*	8237286	(A/G)
CakSNP9875	*Kabuli*	*Ca_Kabuli_Chr06*	8243936	(A/T)
CakSNP9876	*Kabuli*	*Ca_Kabuli_Chr06*	8243947	(T/C)
CakSNP9877	*Kabuli*	*Ca_Kabuli_Chr06*	8244152	(C/T)
CakSNP9878	*Kabuli*	*Ca_Kabuli_Chr06*	8244249	(C/T)
CakSNP9879	*Kabuli*	*Ca_Kabuli_Chr06*	8246037	(T/C)
CakSNP9880	*Kabuli*	*Ca_Kabuli_Chr06*	8246187	(A/C)

**SNP IDs**	**Cultivars**	**Chromosomes/scaffolds**	**Physical positions (bp)**	**SNPs**
CakSNP9881	*Kabuli*	*Ca_Kabuli_Chr06*	8248202	(A/C)
CakSNP9882	*Kabuli*	*Ca_Kabuli_Chr06*	8251365	(T/C)
CakSNP9883	*Kabuli*	*Ca_Kabuli_Chr06*	8269326	(A/C)
CakSNP9884	*Kabuli*	*Ca_Kabuli_Chr06*	8285170	(G/A)
CakSNP9885	*Kabuli*	*Ca_Kabuli_Chr06*	8285147	(A/G)
CakSNP9886	*Kabuli*	*Ca_Kabuli_Chr06*	8633231	(C/A)
CakSNP9887	*Kabuli*	*Ca_Kabuli_Chr06*	8633215	(C/A)
CakSNP9888	*Kabuli*	*Ca_Kabuli_Chr06*	8633187	(A/C)
CakSNP9889	*Kabuli*	*Ca_Kabuli_Chr06*	8634513	(G/A)
CakSNP9890	*Kabuli*	*Ca_Kabuli_Chr06*	8634494	(T/C)
CakSNP9891	*Kabuli*	*Ca_Kabuli_Chr06*	8643286	(T/C)
CakSNP9892	*Kabuli*	*Ca_Kabuli_Chr06*	8645079	(A/G)
CakSNP9893	*Kabuli*	*Ca_Kabuli_Chr06*	8710064	(A/T)
CakSNP9894	*Kabuli*	*Ca_Kabuli_Chr06*	8787556	(C/T)
CakSNP9895	*Kabuli*	*Ca_Kabuli_Chr06*	8787557	(T/C)
CakSNP9896	*Kabuli*	*Ca_Kabuli_Chr06*	8787562	(C/T)
CakSNP9897	*Kabuli*	*Ca_Kabuli_Chr06*	8787564	(G/T)
CakSNP9898	*Kabuli*	*Ca_Kabuli_Chr06*	8787568	(G/T)
CakSNP9899	*Kabuli*	*Ca_Kabuli_Chr06*	8787572	(T/A)
CakSNP9900	*Kabuli*	*Ca_Kabuli_Chr06*	8787573	(G/A)
CakSNP9901	*Kabuli*	*Ca_Kabuli_Chr06*	8794430	(A/G)
CakSNP9902	*Kabuli*	*Ca_Kabuli_Chr06*	8794552	(C/G)
CakSNP9903	*Kabuli*	*Ca_Kabuli_Chr06*	8890548	(A/G)
CakSNP9904	*Kabuli*	*Ca_Kabuli_Chr06*	8890552	(C/G)
CakSNP9905	*Kabuli*	*Ca_Kabuli_Chr06*	8893216	(A/G)
CakSNP9906	*Kabuli*	*Ca_Kabuli_Chr06*	8968068	(G/A)
CakSNP9907	*Kabuli*	*Ca_Kabuli_Chr06*	8969711	(C/T)
CakSNP9908	*Kabuli*	*Ca_Kabuli_Chr06*	8969725	(C/A)
CakSNP9909	*Kabuli*	*Ca_Kabuli_Chr06*	9053279	(A/G)
CakSNP9910	*Kabuli*	*Ca_Kabuli_Chr06*	9054596	(T/C)
CakSNP9911	*Kabuli*	*Ca_Kabuli_Chr06*	9088121	(T/C)
CakSNP9912	*Kabuli*	*Ca_Kabuli_Chr06*	9088151	(G/A)
CakSNP9913	*Kabuli*	*Ca_Kabuli_Chr06*	9088163	(A/G)
CakSNP9914	*Kabuli*	*Ca_Kabuli_Chr06*	9088184	(G/A)
CakSNP9915	*Kabuli*	*Ca_Kabuli_Chr06*	9088234	(A/C)
CakSNP9916	*Kabuli*	*Ca_Kabuli_Chr06*	9190620	(G/T)
CakSNP9917	*Kabuli*	*Ca_Kabuli_Chr06*	9221201	(A/G)
CakSNP9918	*Kabuli*	*Ca_Kabuli_Chr06*	9227767	(C/A)
CakSNP9919	*Kabuli*	*Ca_Kabuli_Chr06*	9228004	(C/A)
CakSNP9920	*Kabuli*	*Ca_Kabuli_Chr06*	9323775	(A/G)
CakSNP9921	*Kabuli*	*Ca_Kabuli_Chr06*	9324122	(A/C)

**SNP IDs**	**Cultivars**	**Chromosomes/scaffolds**	**Physical positions (bp)**	**SNPs**
CakSNP9922	*Kabuli*	*Ca_Kabuli_Chr06*	9373240	(C/A)
CakSNP9923	*Kabuli*	*Ca_Kabuli_Chr06*	9373383	(G/C)
CakSNP9924	*Kabuli*	*Ca_Kabuli_Chr06*	9378508	(A/T)
CakSNP9925	*Kabuli*	*Ca_Kabuli_Chr06*	9378557	(G/C)
CakSNP9926	*Kabuli*	*Ca_Kabuli_Chr06*	9382091	(C/T)
CakSNP9927	*Kabuli*	*Ca_Kabuli_Chr06*	9423222	(T/C)
CakSNP9928	*Kabuli*	*Ca_Kabuli_Chr06*	9424317	(T/C)
CakSNP9929	*Kabuli*	*Ca_Kabuli_Chr06*	9428522	(C/T)
CakSNP9930	*Kabuli*	*Ca_Kabuli_Chr06*	9428517	(G/A)
CakSNP9931	*Kabuli*	*Ca_Kabuli_Chr06*	9523228	(T/C)
CakSNP9932	*Kabuli*	*Ca_Kabuli_Chr06*	9544233	(T/C)
CakSNP9933	*Kabuli*	*Ca_Kabuli_Chr06*	9565932	(A/G)
CakSNP9934	*Kabuli*	*Ca_Kabuli_Chr06*	9568243	(A/C)
CakSNP9935	*Kabuli*	*Ca_Kabuli_Chr06*	9568247	(G/A)
CakSNP9936	*Kabuli*	*Ca_Kabuli_Chr06*	9568289	(A/C)
CakSNP9937	*Kabuli*	*Ca_Kabuli_Chr06*	9569170	(A/G)
CakSNP9938	*Kabuli*	*Ca_Kabuli_Chr06*	9570753	(C/T)
CakSNP9939	*Kabuli*	*Ca_Kabuli_Chr06*	9617218	(A/G)
CakSNP9940	*Kabuli*	*Ca_Kabuli_Chr06*	9617233	(A/G)
CakSNP9941	*Kabuli*	*Ca_Kabuli_Chr06*	9714240	(T/C)
CakSNP9942	*Kabuli*	*Ca_Kabuli_Chr06*	9728195	(G/T)
CakSNP9943	*Kabuli*	*Ca_Kabuli_Chr06*	9728181	(A/G)
CakSNP9944	*Kabuli*	*Ca_Kabuli_Chr06*	9814378	(G/T)
CakSNP9945	*Kabuli*	*Ca_Kabuli_Chr06*	9834199	(T/C)
CakSNP9946	*Kabuli*	*Ca_Kabuli_Chr06*	9834198	(C/A)
CakSNP9947	*Kabuli*	*Ca_Kabuli_Chr06*	9834187	(A/T)
CakSNP9948	*Kabuli*	*Ca_Kabuli_Chr06*	9834184	(A/T)
CakSNP9949	*Kabuli*	*Ca_Kabuli_Chr06*	9834183	(C/T)
CakSNP9950	*Kabuli*	*Ca_Kabuli_Chr06*	9841836	(G/A)
CakSNP9951	*Kabuli*	*Ca_Kabuli_Chr06*	9841890	(G/A)
CakSNP9952	*Kabuli*	*Ca_Kabuli_Chr06*	9845070	(C/T)
CakSNP9953	*Kabuli*	*Ca_Kabuli_Chr06*	9845076	(T/C)
CakSNP9954	*Kabuli*	*Ca_Kabuli_Chr06*	9872557	(G/A)
CakSNP9955	*Kabuli*	*Ca_Kabuli_Chr06*	9872657	(C/T)
CakSNP9956	*Kabuli*	*Ca_Kabuli_Chr06*	9872988	(A/T)
CakSNP9957	*Kabuli*	*Ca_Kabuli_Chr06*	9888237	(G/A)
CakSNP9958	*Kabuli*	*Ca_Kabuli_Chr06*	9938833	(T/C)
CakSNP9959	*Kabuli*	*Ca_Kabuli_Chr06*	9939017	(G/A)
CakSNP9960	*Kabuli*	*Ca_Kabuli_Chr06*	9959686	(T/C)
CakSNP9961	*Kabuli*	*Ca_Kabuli_Chr06*	10020177	(C/G)
CakSNP9962	*Kabuli*	*Ca_Kabuli_Chr06*	10020187	(A/G)

**SNP IDs**	**Cultivars**	**Chromosomes/scaffolds**	**Physical positions (bp)**	**SNPs**
CakSNP9963	*Kabuli*	*Ca_Kabuli_Chr06*	10025115	(A/C)
CakSNP9964	*Kabuli*	*Ca_Kabuli_Chr06*	10025113	(C/G)
CakSNP9965	*Kabuli*	*Ca_Kabuli_Chr06*	10027019	(G/A)
CakSNP9966	*Kabuli*	*Ca_Kabuli_Chr06*	10027015	(G/C)
CakSNP9967	*Kabuli*	*Ca_Kabuli_Chr06*	10028504	(A/G)
CakSNP9968	*Kabuli*	*Ca_Kabuli_Chr06*	10028508	(G/C)
CakSNP9969	*Kabuli*	*Ca_Kabuli_Chr06*	10028515	(A/G)
CakSNP9970	*Kabuli*	*Ca_Kabuli_Chr06*	10045644	(A/C)
CakSNP9971	*Kabuli*	*Ca_Kabuli_Chr06*	10045844	(A/G)
CakSNP9972	*Kabuli*	*Ca_Kabuli_Chr06*	10088963	(G/T)
CakSNP9973	*Kabuli*	*Ca_Kabuli_Chr06*	10088984	(A/G)
CakSNP9974	*Kabuli*	*Ca_Kabuli_Chr06*	10100863	(C/G)
CakSNP9975	*Kabuli*	*Ca_Kabuli_Chr06*	10111488	(C/G)
CakSNP9976	*Kabuli*	*Ca_Kabuli_Chr06*	10111520	(G/C)
CakSNP9977	*Kabuli*	*Ca_Kabuli_Chr06*	10111870	(A/G)
CakSNP9978	*Kabuli*	*Ca_Kabuli_Chr06*	10111938	(G/T)
CakSNP9979	*Kabuli*	*Ca_Kabuli_Chr06*	10116403	(A/T)
CakSNP9980	*Kabuli*	*Ca_Kabuli_Chr06*	10116475	(C/A)
CakSNP9981	*Kabuli*	*Ca_Kabuli_Chr06*	10138984	(C/T)
CakSNP9982	*Kabuli*	*Ca_Kabuli_Chr06*	10151973	(A/G)
CakSNP9983	*Kabuli*	*Ca_Kabuli_Chr06*	10151974	(A/G)
CakSNP9984	*Kabuli*	*Ca_Kabuli_Chr06*	10158850	(T/C)
CakSNP9985	*Kabuli*	*Ca_Kabuli_Chr06*	10188400	(G/T)
CakSNP9986	*Kabuli*	*Ca_Kabuli_Chr06*	10191941	(A/G)
CakSNP9987	*Kabuli*	*Ca_Kabuli_Chr06*	10215262	(T/G)
CakSNP9988	*Kabuli*	*Ca_Kabuli_Chr06*	10230617	(A/G)
CakSNP9989	*Kabuli*	*Ca_Kabuli_Chr06*	10230657	(A/G)
CakSNP9990	*Kabuli*	*Ca_Kabuli_Chr06*	10231092	(T/G)
CakSNP9991	*Kabuli*	*Ca_Kabuli_Chr06*	10231095	(G/T)
CakSNP9992	*Kabuli*	*Ca_Kabuli_Chr06*	10234241	(A/G)
CakSNP9993	*Kabuli*	*Ca_Kabuli_Chr06*	10259614	(C/T)
CakSNP9994	*Kabuli*	*Ca_Kabuli_Chr06*	10260274	(C/T)
CakSNP9995	*Kabuli*	*Ca_Kabuli_Chr06*	10314993	(C/G)
CakSNP9996	*Kabuli*	*Ca_Kabuli_Chr06*	10395411	(G/A)
CakSNP9997	*Kabuli*	*Ca_Kabuli_Chr06*	10395379	(T/C)
CakSNP9998	*Kabuli*	*Ca_Kabuli_Chr06*	10395356	(T/A)
CakSNP9999	*Kabuli*	*Ca_Kabuli_Chr06*	10441736	(A/C)
CakSNP10000	*Kabuli*	*Ca_Kabuli_Chr06*	10441705	(T/C)
CakSNP10001	*Kabuli*	*Ca_Kabuli_Chr06*	10442148	(A/C)
CakSNP10002	*Kabuli*	*Ca_Kabuli_Chr06*	10445185	(C/T)
CakSNP10003	*Kabuli*	*Ca_Kabuli_Chr06*	10445274	(G/A)

SNP IDs	Cultivars	Chromosomes/scaffolds	Physical positions (bp)	SNPs
CakSNP10004	*Kabuli*	*Ca_Kabuli_Chr06*	10456774	(A/G)
CakSNP10005	*Kabuli*	*Ca_Kabuli_Chr06*	10456854	(G/T)
CakSNP10006	*Kabuli*	*Ca_Kabuli_Chr06*	10494134	(C/T)
CakSNP10007	*Kabuli*	*Ca_Kabuli_Chr06*	10494057	(C/A)
CakSNP10008	*Kabuli*	*Ca_Kabuli_Chr06*	10494055	(T/A)
CakSNP10009	*Kabuli*	*Ca_Kabuli_Chr06*	10495747	(G/T)
CakSNP10010	*Kabuli*	*Ca_Kabuli_Chr06*	10495804	(C/T)
CakSNP10011	*Kabuli*	*Ca_Kabuli_Chr06*	10502381	(A/G)
CakSNP10012	*Kabuli*	*Ca_Kabuli_Chr06*	10502387	(C/A)
CakSNP10013	*Kabuli*	*Ca_Kabuli_Chr06*	10510996	(T/C)
CakSNP10014	*Kabuli*	*Ca_Kabuli_Chr06*	10520958	(C/T)
CakSNP10015	*Kabuli*	*Ca_Kabuli_Chr06*	10521741	(A/T)
CakSNP10016	*Kabuli*	*Ca_Kabuli_Chr06*	10531311	(A/G)
CakSNP10017	*Kabuli*	*Ca_Kabuli_Chr06*	10531399	(C/T)
CakSNP10018	*Kabuli*	*Ca_Kabuli_Chr06*	10532182	(A/T)
CakSNP10019	*Kabuli*	*Ca_Kabuli_Chr06*	10532739	(A/G)
CakSNP10020	*Kabuli*	*Ca_Kabuli_Chr06*	10539918	(A/C)
CakSNP10021	*Kabuli*	*Ca_Kabuli_Chr06*	10539977	(C/T)
CakSNP10022	*Kabuli*	*Ca_Kabuli_Chr06*	10539994	(A/C)
CakSNP10023	*Kabuli*	*Ca_Kabuli_Chr06*	10567518	(A/G)
CakSNP10024	*Kabuli*	*Ca_Kabuli_Chr06*	10588030	(C/T)
CakSNP10025	*Kabuli*	*Ca_Kabuli_Chr06*	10613546	(A/G)
CakSNP10026	*Kabuli*	*Ca_Kabuli_Chr06*	10613612	(A/G)
CakSNP10027	*Kabuli*	*Ca_Kabuli_Chr06*	10652739	(C/T)
CakSNP10028	*Kabuli*	*Ca_Kabuli_Chr06*	10667504	(A/T)
CakSNP10029	*Kabuli*	*Ca_Kabuli_Chr06*	10667559	(T/C)
CakSNP10030	*Kabuli*	*Ca_Kabuli_Chr06*	10667640	(T/G)
CakSNP10031	*Kabuli*	*Ca_Kabuli_Chr06*	10670343	(G/A)
CakSNP10032	*Kabuli*	*Ca_Kabuli_Chr06*	10670368	(G/A)
CakSNP10033	*Kabuli*	*Ca_Kabuli_Chr06*	10670409	(G/A)
CakSNP10034	*Kabuli*	*Ca_Kabuli_Chr06*	10670412	(A/C)
CakSNP10035	*Kabuli*	*Ca_Kabuli_Chr06*	10670482	(C/T)
CakSNP10036	*Kabuli*	*Ca_Kabuli_Chr06*	10670582	(G/A)
CakSNP10037	*Kabuli*	*Ca_Kabuli_Chr06*	10670675	(C/T)
CakSNP10038	*Kabuli*	*Ca_Kabuli_Chr06*	10670694	(G/A)
CakSNP10039	*Kabuli*	*Ca_Kabuli_Chr06*	10670773	(A/C)
CakSNP10040	*Kabuli*	*Ca_Kabuli_Chr06*	10670959	(T/C)
CakSNP10041	*Kabuli*	*Ca_Kabuli_Chr06*	10671458	(T/C)
CakSNP10042	*Kabuli*	*Ca_Kabuli_Chr06*	10671444	(A/C)
CakSNP10043	*Kabuli*	*Ca_Kabuli_Chr06*	10672468	(C/T)
CakSNP10044	*Kabuli*	*Ca_Kabuli_Chr06*	10677469	(A/G)

SNP IDs	Cultivars	Chromosomes/scaffolds	Physical positions (bp)	SNPs
CakSNP10045	*Kabuli*	*Ca_Kabuli_Chr06*	10677501	(C/T)
CakSNP10046	*Kabuli*	*Ca_Kabuli_Chr06*	10682221	(G/T)
CakSNP10047	*Kabuli*	*Ca_Kabuli_Chr06*	10682201	(C/G)
CakSNP10048	*Kabuli*	*Ca_Kabuli_Chr06*	10683193	(C/T)
CakSNP10049	*Kabuli*	*Ca_Kabuli_Chr06*	10696986	(A/T)
CakSNP10050	*Kabuli*	*Ca_Kabuli_Chr06*	10696990	(T/C)
CakSNP10051	*Kabuli*	*Ca_Kabuli_Chr06*	10697064	(T/A)
CakSNP10052	*Kabuli*	*Ca_Kabuli_Chr06*	10716986	(G/A)
CakSNP10053	*Kabuli*	*Ca_Kabuli_Chr06*	10716968	(G/A)
CakSNP10054	*Kabuli*	*Ca_Kabuli_Chr06*	10744029	(C/A)
CakSNP10055	*Kabuli*	*Ca_Kabuli_Chr06*	10743996	(A/T)
CakSNP10056	*Kabuli*	*Ca_Kabuli_Chr06*	10748965	(G/T)
CakSNP10057	*Kabuli*	*Ca_Kabuli_Chr06*	10779368	(G/C)
CakSNP10058	*Kabuli*	*Ca_Kabuli_Chr06*	10779465	(A/T)
CakSNP10059	*Kabuli*	*Ca_Kabuli_Chr06*	10779547	(T/G)
CakSNP10060	*Kabuli*	*Ca_Kabuli_Chr06*	10779636	(C/A)
CakSNP10061	*Kabuli*	*Ca_Kabuli_Chr06*	10785480	(T/G)
CakSNP10062	*Kabuli*	*Ca_Kabuli_Chr06*	10870187	(T/G)
CakSNP10063	*Kabuli*	*Ca_Kabuli_Chr06*	10870534	(A/G)
CakSNP10064	*Kabuli*	*Ca_Kabuli_Chr06*	10880791	(C/T)
CakSNP10065	*Kabuli*	*Ca_Kabuli_Chr06*	10880819	(T/G)
CakSNP10066	*Kabuli*	*Ca_Kabuli_Chr06*	10881002	(A/G)
CakSNP10067	*Kabuli*	*Ca_Kabuli_Chr06*	10880985	(C/T)
CakSNP10068	*Kabuli*	*Ca_Kabuli_Chr06*	10880962	(G/T)
CakSNP10069	*Kabuli*	*Ca_Kabuli_Chr06*	10991652	(C/A)
CakSNP10070	*Kabuli*	*Ca_Kabuli_Chr06*	10991717	(T/A)
CakSNP10071	*Kabuli*	*Ca_Kabuli_Chr06*	10991737	(A/C)
CakSNP10072	*Kabuli*	*Ca_Kabuli_Chr06*	11011847	(C/G)
CakSNP10073	*Kabuli*	*Ca_Kabuli_Chr06*	11128158	(A/G)
CakSNP10074	*Kabuli*	*Ca_Kabuli_Chr06*	11128170	(T/C)
CakSNP10075	*Kabuli*	*Ca_Kabuli_Chr06*	11128204	(G/A)
CakSNP10076	*Kabuli*	*Ca_Kabuli_Chr06*	11131010	(T/A)
CakSNP10077	*Kabuli*	*Ca_Kabuli_Chr06*	11131464	(G/A)
CakSNP10078	*Kabuli*	*Ca_Kabuli_Chr06*	11131462	(G/A)
CakSNP10079	*Kabuli*	*Ca_Kabuli_Chr06*	11131436	(T/G)
CakSNP10080	*Kabuli*	*Ca_Kabuli_Chr06*	11163819	(T/G)
CakSNP10081	*Kabuli*	*Ca_Kabuli_Chr06*	11163822	(C/T)
CakSNP10082	*Kabuli*	*Ca_Kabuli_Chr06*	11163836	(C/T)
CakSNP10083	*Kabuli*	*Ca_Kabuli_Chr06*	11188803	(G/A)
CakSNP10084	*Kabuli*	*Ca_Kabuli_Chr06*	11189051	(A/G)
CakSNP10085	*Kabuli*	*Ca_Kabuli_Chr06*	11229102	(C/T)

SNP IDs	Cultivars	Chromosomes/scaffolds	Physical positions (bp)	SNPs
CakSNP10086	*Kabuli*	*Ca_Kabuli_Chr06*	11229143	(A/C)
CakSNP10087	*Kabuli*	*Ca_Kabuli_Chr06*	11229302	(A/G)
CakSNP10088	*Kabuli*	*Ca_Kabuli_Chr06*	11229388	(C/T)
CakSNP10089	*Kabuli*	*Ca_Kabuli_Chr06*	11285884	(G/A)
CakSNP10090	*Kabuli*	*Ca_Kabuli_Chr06*	11300597	(C/T)
CakSNP10091	*Kabuli*	*Ca_Kabuli_Chr06*	11300549	(A/C)
CakSNP10092	*Kabuli*	*Ca_Kabuli_Chr06*	11302037	(A/C)
CakSNP10093	*Kabuli*	*Ca_Kabuli_Chr06*	11302033	(T/C)
CakSNP10094	*Kabuli*	*Ca_Kabuli_Chr06*	11314581	(T/C)
CakSNP10095	*Kabuli*	*Ca_Kabuli_Chr06*	11394963	(A/G)
CakSNP10096	*Kabuli*	*Ca_Kabuli_Chr06*	11395330	(C/T)
CakSNP10097	*Kabuli*	*Ca_Kabuli_Chr06*	11508828	(C/T)
CakSNP10098	*Kabuli*	*Ca_Kabuli_Chr06*	11508824	(T/G)
CakSNP10099	*Kabuli*	*Ca_Kabuli_Chr06*	11508817	(G/A)
CakSNP10100	*Kabuli*	*Ca_Kabuli_Chr06*	11508816	(A/C)
CakSNP10101	*Kabuli*	*Ca_Kabuli_Chr06*	11514926	(T/C)
CakSNP10102	*Kabuli*	*Ca_Kabuli_Chr06*	11514883	(C/T)
CakSNP10103	*Kabuli*	*Ca_Kabuli_Chr06*	11514935	(G/A)
CakSNP10104	*Kabuli*	*Ca_Kabuli_Chr06*	11543615	(G/C)
CakSNP10105	*Kabuli*	*Ca_Kabuli_Chr06*	11543618	(T/G)
CakSNP10106	*Kabuli*	*Ca_Kabuli_Chr06*	11665233	(G/A)
CakSNP10107	*Kabuli*	*Ca_Kabuli_Chr06*	11665338	(T/G)
CakSNP10108	*Kabuli*	*Ca_Kabuli_Chr06*	12408955	(A/G)
CakSNP10109	*Kabuli*	*Ca_Kabuli_Chr06*	12408964	(G/C)
CakSNP10110	*Kabuli*	*Ca_Kabuli_Chr06*	12434183	(G/T)
CakSNP10111	*Kabuli*	*Ca_Kabuli_Chr06*	12437275	(A/T)
CakSNP10112	*Kabuli*	*Ca_Kabuli_Chr06*	12437285	(A/G)
CakSNP10113	*Kabuli*	*Ca_Kabuli_Chr06*	12437288	(G/A)
CakSNP10114	*Kabuli*	*Ca_Kabuli_Chr06*	12437319	(C/T)
CakSNP10115	*Kabuli*	*Ca_Kabuli_Chr06*	12494824	(G/T)
CakSNP10116	*Kabuli*	*Ca_Kabuli_Chr06*	12494972	(A/G)
CakSNP10117	*Kabuli*	*Ca_Kabuli_Chr06*	12532947	(T/A)
CakSNP10118	*Kabuli*	*Ca_Kabuli_Chr06*	12535733	(C/G)
CakSNP10119	*Kabuli*	*Ca_Kabuli_Chr06*	12619757	(C/T)
CakSNP10120	*Kabuli*	*Ca_Kabuli_Chr06*	12619786	(C/A)
CakSNP10121	*Kabuli*	*Ca_Kabuli_Chr06*	12619829	(A/T)
CakSNP10122	*Kabuli*	*Ca_Kabuli_Chr06*	12647539	(G/A)
CakSNP10123	*Kabuli*	*Ca_Kabuli_Chr06*	12799890	(T/G)
CakSNP10124	*Kabuli*	*Ca_Kabuli_Chr06*	12844524	(G/A)
CakSNP10125	*Kabuli*	*Ca_Kabuli_Chr06*	12893335	(C/A)
CakSNP10126	*Kabuli*	*Ca_Kabuli_Chr06*	12893424	(C/T)

**SNP IDs**	**Cultivars**	**Chromosomes/scaffolds**	**Physical positions (bp)**	**SNPs**
CakSNP10127	*Kabuli*	*Ca_Kabuli_Chr06*	12906599	(G/A)
CakSNP10128	*Kabuli*	*Ca_Kabuli_Chr06*	12906680	(T/C)
CakSNP10129	*Kabuli*	*Ca_Kabuli_Chr06*	12906669	(G/A)
CakSNP10130	*Kabuli*	*Ca_Kabuli_Chr06*	12973765	(A/G)
CakSNP10131	*Kabuli*	*Ca_Kabuli_Chr06*	12976279	(T/A)
CakSNP10132	*Kabuli*	*Ca_Kabuli_Chr06*	12977129	(A/G)
CakSNP10133	*Kabuli*	*Ca_Kabuli_Chr06*	12977196	(A/G)
CakSNP10134	*Kabuli*	*Ca_Kabuli_Chr06*	12977204	(T/G)
CakSNP10135	*Kabuli*	*Ca_Kabuli_Chr06*	12985896	(A/C)
CakSNP10136	*Kabuli*	*Ca_Kabuli_Chr06*	12985908	(C/G)
CakSNP10137	*Kabuli*	*Ca_Kabuli_Chr06*	12985910	(C/T)
CakSNP10138	*Kabuli*	*Ca_Kabuli_Chr06*	12986776	(A/G)
CakSNP10139	*Kabuli*	*Ca_Kabuli_Chr06*	12986833	(A/C)
CakSNP10140	*Kabuli*	*Ca_Kabuli_Chr06*	13008333	(A/T)
CakSNP10141	*Kabuli*	*Ca_Kabuli_Chr06*	13008360	(C/T)
CakSNP10142	*Kabuli*	*Ca_Kabuli_Chr06*	13008465	(C/T)
CakSNP10143	*Kabuli*	*Ca_Kabuli_Chr06*	13099658	(G/T)
CakSNP10144	*Kabuli*	*Ca_Kabuli_Chr06*	13099656	(G/T)
CakSNP10145	*Kabuli*	*Ca_Kabuli_Chr06*	13107887	(C/T)
CakSNP10146	*Kabuli*	*Ca_Kabuli_Chr06*	13117820	(A/T)
CakSNP10147	*Kabuli*	*Ca_Kabuli_Chr06*	13131682	(C/G)
CakSNP10148	*Kabuli*	*Ca_Kabuli_Chr06*	13136852	(C/T)
CakSNP10149	*Kabuli*	*Ca_Kabuli_Chr06*	13136835	(C/T)
CakSNP10150	*Kabuli*	*Ca_Kabuli_Chr06*	13136976	(A/G)
CakSNP10151	*Kabuli*	*Ca_Kabuli_Chr06*	13136955	(G/A)
CakSNP10152	*Kabuli*	*Ca_Kabuli_Chr06*	13187706	(A/G)
CakSNP10153	*Kabuli*	*Ca_Kabuli_Chr06*	13187709	(T/C)
CakSNP10154	*Kabuli*	*Ca_Kabuli_Chr06*	13188124	(G/C)
CakSNP10155	*Kabuli*	*Ca_Kabuli_Chr06*	13188119	(A/C)
CakSNP10156	*Kabuli*	*Ca_Kabuli_Chr06*	13188480	(T/C)
CakSNP10157	*Kabuli*	*Ca_Kabuli_Chr06*	13192177	(G/A)
CakSNP10158	*Kabuli*	*Ca_Kabuli_Chr06*	13243282	(A/G)
CakSNP10159	*Kabuli*	*Ca_Kabuli_Chr06*	13252152	(C/T)
CakSNP10160	*Kabuli*	*Ca_Kabuli_Chr06*	13296128	(G/A)
CakSNP10161	*Kabuli*	*Ca_Kabuli_Chr06*	13326969	(C/G)
CakSNP10162	*Kabuli*	*Ca_Kabuli_Chr06*	13340012	(A/G)
CakSNP10163	*Kabuli*	*Ca_Kabuli_Chr06*	13340757	(T/C)
CakSNP10164	*Kabuli*	*Ca_Kabuli_Chr06*	13340762	(G/A)
CakSNP10165	*Kabuli*	*Ca_Kabuli_Chr06*	13340782	(G/A)
CakSNP10166	*Kabuli*	*Ca_Kabuli_Chr06*	13413848	(C/G)
CakSNP10167	*Kabuli*	*Ca_Kabuli_Chr06*	13483841	(T/G)

SNP IDs	Cultivars	Chromosomes/scaffolds	Physical positions (bp)	SNPs
CakSNP10168	*Kabuli*	*Ca_Kabuli_Chr06*	13565854	(T/C)
CakSNP10169	*Kabuli*	*Ca_Kabuli_Chr06*	13565990	(C/T)
CakSNP10170	*Kabuli*	*Ca_Kabuli_Chr06*	13566007	(C/T)
CakSNP10171	*Kabuli*	*Ca_Kabuli_Chr06*	13566012	(T/G)
CakSNP10172	*Kabuli*	*Ca_Kabuli_Chr06*	13566041	(C/T)
CakSNP10173	*Kabuli*	*Ca_Kabuli_Chr06*	13566110	(A/G)
CakSNP10174	*Kabuli*	*Ca_Kabuli_Chr06*	13566136	(A/C)
CakSNP10175	*Kabuli*	*Ca_Kabuli_Chr06*	13566130	(G/T)
CakSNP10176	*Kabuli*	*Ca_Kabuli_Chr06*	13572236	(A/T)
CakSNP10177	*Kabuli*	*Ca_Kabuli_Chr06*	13572221	(A/C)
CakSNP10178	*Kabuli*	*Ca_Kabuli_Chr06*	13576963	(T/A)
CakSNP10179	*Kabuli*	*Ca_Kabuli_Chr06*	13591590	(T/G)
CakSNP10180	*Kabuli*	*Ca_Kabuli_Chr06*	13593544	(A/G)
CakSNP10181	*Kabuli*	*Ca_Kabuli_Chr06*	13593564	(C/A)
CakSNP10182	*Kabuli*	*Ca_Kabuli_Chr06*	13593600	(T/C)
CakSNP10183	*Kabuli*	*Ca_Kabuli_Chr06*	13593685	(G/A)
CakSNP10184	*Kabuli*	*Ca_Kabuli_Chr06*	13593668	(A/G)
CakSNP10185	*Kabuli*	*Ca_Kabuli_Chr06*	13593661	(T/C)
CakSNP10186	*Kabuli*	*Ca_Kabuli_Chr06*	13593654	(T/C)
CakSNP10187	*Kabuli*	*Ca_Kabuli_Chr06*	13619538	(C/T)
CakSNP10188	*Kabuli*	*Ca_Kabuli_Chr06*	13619555	(C/T)
CakSNP10189	*Kabuli*	*Ca_Kabuli_Chr06*	13619560	(T/G)
CakSNP10190	*Kabuli*	*Ca_Kabuli_Chr06*	13619589	(C/T)
CakSNP10191	*Kabuli*	*Ca_Kabuli_Chr06*	13619658	(A/G)
CakSNP10192	*Kabuli*	*Ca_Kabuli_Chr06*	13619684	(A/C)
CakSNP10193	*Kabuli*	*Ca_Kabuli_Chr06*	13622292	(C/T)
CakSNP10194	*Kabuli*	*Ca_Kabuli_Chr06*	13625388	(A/G)
CakSNP10195	*Kabuli*	*Ca_Kabuli_Chr06*	13637387	(T/G)
CakSNP10196	*Kabuli*	*Ca_Kabuli_Chr06*	13637347	(C/T)
CakSNP10197	*Kabuli*	*Ca_Kabuli_Chr06*	13668916	(T/A)
CakSNP10198	*Kabuli*	*Ca_Kabuli_Chr06*	13668995	(A/G)
CakSNP10199	*Kabuli*	*Ca_Kabuli_Chr06*	13669052	(G/C)
CakSNP10200	*Kabuli*	*Ca_Kabuli_Chr06*	13669049	(C/T)
CakSNP10201	*Kabuli*	*Ca_Kabuli_Chr06*	13669048	(T/A)
CakSNP10202	*Kabuli*	*Ca_Kabuli_Chr06*	13669042	(A/G)
CakSNP10203	*Kabuli*	*Ca_Kabuli_Chr06*	13669018	(G/A)
CakSNP10204	*Kabuli*	*Ca_Kabuli_Chr06*	13669006	(G/C)
CakSNP10205	*Kabuli*	*Ca_Kabuli_Chr06*	13714897	(A/T)
CakSNP10206	*Kabuli*	*Ca_Kabuli_Chr06*	13764980	(T/A)
CakSNP10207	*Kabuli*	*Ca_Kabuli_Chr06*	13827484	(C/T)
CakSNP10208	*Kabuli*	*Ca_Kabuli_Chr06*	13827452	(C/G)

SNP IDs	Cultivars	Chromosomes/scaffolds	Physical positions (bp)	SNPs
CakSNP10209	*Kabuli*	*Ca_Kabuli_Chr06*	13827444	(G/A)
CakSNP10210	*Kabuli*	*Ca_Kabuli_Chr06*	13913539	(A/G)
CakSNP10211	*Kabuli*	*Ca_Kabuli_Chr06*	13917076	(T/C)
CakSNP10212	*Kabuli*	*Ca_Kabuli_Chr06*	13917135	(T/A)
CakSNP10213	*Kabuli*	*Ca_Kabuli_Chr06*	13921954	(A/T)
CakSNP10214	*Kabuli*	*Ca_Kabuli_Chr06*	13941701	(A/T)
CakSNP10215	*Kabuli*	*Ca_Kabuli_Chr06*	13942961	(A/G)
CakSNP10216	*Kabuli*	*Ca_Kabuli_Chr06*	13948732	(G/T)
CakSNP10217	*Kabuli*	*Ca_Kabuli_Chr06*	13948751	(T/G)
CakSNP10218	*Kabuli*	*Ca_Kabuli_Chr06*	13948778	(A/C)
CakSNP10219	*Kabuli*	*Ca_Kabuli_Chr06*	13986559	(A/G)
CakSNP10220	*Kabuli*	*Ca_Kabuli_Chr06*	14042859	(A/T)
CakSNP10221	*Kabuli*	*Ca_Kabuli_Chr06*	14067312	(A/C)
CakSNP10222	*Kabuli*	*Ca_Kabuli_Chr06*	14067326	(C/T)
CakSNP10223	*Kabuli*	*Ca_Kabuli_Chr06*	14112542	(A/G)
CakSNP10224	*Kabuli*	*Ca_Kabuli_Chr06*	14148771	(A/G)
CakSNP10225	*Kabuli*	*Ca_Kabuli_Chr06*	14155543	(C/T)
CakSNP10226	*Kabuli*	*Ca_Kabuli_Chr06*	14155662	(T/C)
CakSNP10227	*Kabuli*	*Ca_Kabuli_Chr06*	14155643	(A/C)
CakSNP10228	*Kabuli*	*Ca_Kabuli_Chr06*	14161084	(G/C)
CakSNP10229	*Kabuli*	*Ca_Kabuli_Chr06*	14201001	(T/C)
CakSNP10230	*Kabuli*	*Ca_Kabuli_Chr06*	14200997	(G/A)
CakSNP10231	*Kabuli*	*Ca_Kabuli_Chr06*	14204400	(T/C)
CakSNP10232	*Kabuli*	*Ca_Kabuli_Chr06*	14204469	(G/A)
CakSNP10233	*Kabuli*	*Ca_Kabuli_Chr06*	14254394	(A/G)
CakSNP10234	*Kabuli*	*Ca_Kabuli_Chr06*	14309042	(C/T)
CakSNP10235	*Kabuli*	*Ca_Kabuli_Chr06*	14330533	(T/C)
CakSNP10236	*Kabuli*	*Ca_Kabuli_Chr06*	14332018	(A/C)
CakSNP10237	*Kabuli*	*Ca_Kabuli_Chr06*	14332404	(A/G)
CakSNP10238	*Kabuli*	*Ca_Kabuli_Chr06*	14353598	(C/T)
CakSNP10239	*Kabuli*	*Ca_Kabuli_Chr06*	14353624	(G/A)
CakSNP10240	*Kabuli*	*Ca_Kabuli_Chr06*	14371493	(C/T)
CakSNP10241	*Kabuli*	*Ca_Kabuli_Chr06*	14371530	(C/T)
CakSNP10242	*Kabuli*	*Ca_Kabuli_Chr06*	14375135	(G/A)
CakSNP10243	*Kabuli*	*Ca_Kabuli_Chr06*	14375141	(G/C)
CakSNP10244	*Kabuli*	*Ca_Kabuli_Chr06*	14375172	(A/G)
CakSNP10245	*Kabuli*	*Ca_Kabuli_Chr06*	14375196	(G/A)
CakSNP10246	*Kabuli*	*Ca_Kabuli_Chr06*	14375207	(C/T)
CakSNP10247	*Kabuli*	*Ca_Kabuli_Chr06*	14401990	(G/A)
CakSNP10248	*Kabuli*	*Ca_Kabuli_Chr06*	14416997	(A/G)
CakSNP10249	*Kabuli*	*Ca_Kabuli_Chr06*	14416981	(A/G)

SNP IDs	Cultivars	Chromosomes/scaffolds	Physical positions (bp)	SNPs
CakSNP10250	*Kabuli*	*Ca_Kabuli_Chr06*	14419832	(C/T)
CakSNP10251	*Kabuli*	*Ca_Kabuli_Chr06*	14526540	(A/T)
CakSNP10252	*Kabuli*	*Ca_Kabuli_Chr06*	14589478	(T/G)
CakSNP10253	*Kabuli*	*Ca_Kabuli_Chr06*	14589480	(T/C)
CakSNP10254	*Kabuli*	*Ca_Kabuli_Chr06*	14589520	(G/C)
CakSNP10255	*Kabuli*	*Ca_Kabuli_Chr06*	14589744	(C/T)
CakSNP10256	*Kabuli*	*Ca_Kabuli_Chr06*	14589807	(T/G)
CakSNP10257	*Kabuli*	*Ca_Kabuli_Chr06*	14589873	(T/C)
CakSNP10258	*Kabuli*	*Ca_Kabuli_Chr06*	14606892	(A/C)
CakSNP10259	*Kabuli*	*Ca_Kabuli_Chr06*	14628213	(A/T)
CakSNP10260	*Kabuli*	*Ca_Kabuli_Chr06*	14628215	(T/C)
CakSNP10261	*Kabuli*	*Ca_Kabuli_Chr06*	14628216	(A/C)
CakSNP10262	*Kabuli*	*Ca_Kabuli_Chr06*	14628284	(G/A)
CakSNP10263	*Kabuli*	*Ca_Kabuli_Chr06*	14719793	(T/C)
CakSNP10264	*Kabuli*	*Ca_Kabuli_Chr06*	14719840	(G/T)
CakSNP10265	*Kabuli*	*Ca_Kabuli_Chr06*	14747608	(T/C)
CakSNP10266	*Kabuli*	*Ca_Kabuli_Chr06*	14753191	(C/T)
CakSNP10267	*Kabuli*	*Ca_Kabuli_Chr06*	14753190	(G/A)
CakSNP10268	*Kabuli*	*Ca_Kabuli_Chr06*	14753158	(A/G)
CakSNP10269	*Kabuli*	*Ca_Kabuli_Chr06*	14753113	(C/A)
CakSNP10270	*Kabuli*	*Ca_Kabuli_Chr06*	14753145	(C/A)
CakSNP10271	*Kabuli*	*Ca_Kabuli_Chr06*	14753150	(G/A)
CakSNP10272	*Kabuli*	*Ca_Kabuli_Chr06*	14762493	(A/G)
CakSNP10273	*Kabuli*	*Ca_Kabuli_Chr06*	14771564	(G/T)
CakSNP10274	*Kabuli*	*Ca_Kabuli_Chr06*	14771709	(G/A)
CakSNP10275	*Kabuli*	*Ca_Kabuli_Chr06*	14786015	(T/G)
CakSNP10276	*Kabuli*	*Ca_Kabuli_Chr06*	14789964	(A/C)
CakSNP10277	*Kabuli*	*Ca_Kabuli_Chr06*	14846794	(C/T)
CakSNP10278	*Kabuli*	*Ca_Kabuli_Chr06*	14857722	(C/A)
CakSNP10279	*Kabuli*	*Ca_Kabuli_Chr06*	14857709	(A/G)
CakSNP10280	*Kabuli*	*Ca_Kabuli_Chr06*	14857680	(G/A)
CakSNP10281	*Kabuli*	*Ca_Kabuli_Chr06*	14861487	(T/A)
CakSNP10282	*Kabuli*	*Ca_Kabuli_Chr06*	14884604	(T/G)
CakSNP10283	*Kabuli*	*Ca_Kabuli_Chr06*	14884606	(T/C)
CakSNP10284	*Kabuli*	*Ca_Kabuli_Chr06*	14891588	(T/C)
CakSNP10285	*Kabuli*	*Ca_Kabuli_Chr06*	14891533	(T/C)
CakSNP10286	*Kabuli*	*Ca_Kabuli_Chr06*	14891597	(C/T)
CakSNP10287	*Kabuli*	*Ca_Kabuli_Chr06*	14891626	(C/A)
CakSNP10288	*Kabuli*	*Ca_Kabuli_Chr06*	14941348	(C/T)
CakSNP10289	*Kabuli*	*Ca_Kabuli_Chr06*	14951877	(C/T)
CakSNP10290	*Kabuli*	*Ca_Kabuli_Chr06*	14951860	(G/A)

SNP IDs	Cultivars	Chromosomes/scaffolds	Physical positions (bp)	SNPs
CakSNP10291	*Kabuli*	*Ca_Kabuli_Chr06*	14982998	(T/C)
CakSNP10292	*Kabuli*	*Ca_Kabuli_Chr06*	14983391	(C/T)
CakSNP10293	*Kabuli*	*Ca_Kabuli_Chr06*	15024904	(C/A)
CakSNP10294	*Kabuli*	*Ca_Kabuli_Chr06*	15024881	(C/A)
CakSNP10295	*Kabuli*	*Ca_Kabuli_Chr06*	15024979	(T/G)
CakSNP10296	*Kabuli*	*Ca_Kabuli_Chr06*	15024999	(T/A)
CakSNP10297	*Kabuli*	*Ca_Kabuli_Chr06*	15025010	(T/C)
CakSNP10298	*Kabuli*	*Ca_Kabuli_Chr06*	15025012	(A/C)
CakSNP10299	*Kabuli*	*Ca_Kabuli_Chr06*	15051094	(T/C)
CakSNP10300	*Kabuli*	*Ca_Kabuli_Chr06*	15061717	(G/A)
CakSNP10301	*Kabuli*	*Ca_Kabuli_Chr06*	15123739	(C/T)
CakSNP10302	*Kabuli*	*Ca_Kabuli_Chr06*	15123771	(T/C)
CakSNP10303	*Kabuli*	*Ca_Kabuli_Chr06*	15123827	(T/G)
CakSNP10304	*Kabuli*	*Ca_Kabuli_Chr06*	15123947	(G/A)
CakSNP10305	*Kabuli*	*Ca_Kabuli_Chr06*	15173607	(G/A)
CakSNP10306	*Kabuli*	*Ca_Kabuli_Chr06*	15185723	(T/C)
CakSNP10307	*Kabuli*	*Ca_Kabuli_Chr06*	15204957	(G/C)
CakSNP10308	*Kabuli*	*Ca_Kabuli_Chr06*	15235575	(A/G)
CakSNP10309	*Kabuli*	*Ca_Kabuli_Chr06*	15235576	(C/T)
CakSNP10310	*Kabuli*	*Ca_Kabuli_Chr06*	15235615	(C/T)
CakSNP10311	*Kabuli*	*Ca_Kabuli_Chr06*	15243538	(A/T)
CakSNP10312	*Kabuli*	*Ca_Kabuli_Chr06*	15243590	(A/G)
CakSNP10313	*Kabuli*	*Ca_Kabuli_Chr06*	15243630	(C/T)
CakSNP10314	*Kabuli*	*Ca_Kabuli_Chr06*	15243674	(T/A)
CakSNP10315	*Kabuli*	*Ca_Kabuli_Chr06*	15268491	(T/G)
CakSNP10316	*Kabuli*	*Ca_Kabuli_Chr06*	15294332	(T/G)
CakSNP10317	*Kabuli*	*Ca_Kabuli_Chr06*	15294331	(C/T)
CakSNP10318	*Kabuli*	*Ca_Kabuli_Chr06*	15294329	(A/C)
CakSNP10319	*Kabuli*	*Ca_Kabuli_Chr06*	15301137	(C/T)
CakSNP10320	*Kabuli*	*Ca_Kabuli_Chr06*	15313655	(G/T)
CakSNP10321	*Kabuli*	*Ca_Kabuli_Chr06*	15328759	(C/A)
CakSNP10322	*Kabuli*	*Ca_Kabuli_Chr06*	15328805	(A/T)
CakSNP10323	*Kabuli*	*Ca_Kabuli_Chr06*	15328948	(A/C)
CakSNP10324	*Kabuli*	*Ca_Kabuli_Chr06*	15347707	(C/T)
CakSNP10325	*Kabuli*	*Ca_Kabuli_Chr06*	15393321	(A/T)
CakSNP10326	*Kabuli*	*Ca_Kabuli_Chr06*	15393349	(T/C)
CakSNP10327	*Kabuli*	*Ca_Kabuli_Chr06*	15463326	(C/G)
CakSNP10328	*Kabuli*	*Ca_Kabuli_Chr06*	15475948	(A/G)
CakSNP10329	*Kabuli*	*Ca_Kabuli_Chr06*	15488265	(C/T)
CakSNP10330	*Kabuli*	*Ca_Kabuli_Chr06*	15488314	(A/T)
CakSNP10331	*Kabuli*	*Ca_Kabuli_Chr06*	15488309	(G/A)

**SNP IDs**	**Cultivars**	**Chromosomes/scaffolds**	**Physical positions (bp)**	**SNPs**
CakSNP10332	*Kabuli*	*Ca_Kabuli_Chr06*	15510433	(A/T)
CakSNP10333	*Kabuli*	*Ca_Kabuli_Chr06*	15510397	(G/A)
CakSNP10334	*Kabuli*	*Ca_Kabuli_Chr06*	15537908	(A/C)
CakSNP10335	*Kabuli*	*Ca_Kabuli_Chr06*	15543295	(C/G)
CakSNP10336	*Kabuli*	*Ca_Kabuli_Chr06*	15543293	(T/G)
CakSNP10337	*Kabuli*	*Ca_Kabuli_Chr06*	15543286	(G/A)
CakSNP10338	*Kabuli*	*Ca_Kabuli_Chr06*	15550044	(C/T)
CakSNP10339	*Kabuli*	*Ca_Kabuli_Chr06*	15589539	(A/T)
CakSNP10340	*Kabuli*	*Ca_Kabuli_Chr06*	15589885	(G/T)
CakSNP10341	*Kabuli*	*Ca_Kabuli_Chr06*	15635126	(A/T)
CakSNP10342	*Kabuli*	*Ca_Kabuli_Chr06*	15644412	(A/T)
CakSNP10343	*Kabuli*	*Ca_Kabuli_Chr06*	15644796	(A/T)
CakSNP10344	*Kabuli*	*Ca_Kabuli_Chr06*	15857728	(T/C)
CakSNP10345	*Kabuli*	*Ca_Kabuli_Chr06*	15861278	(G/A)
CakSNP10346	*Kabuli*	*Ca_Kabuli_Chr06*	16113523	(T/C)
CakSNP10347	*Kabuli*	*Ca_Kabuli_Chr06*	16113522	(A/T)
CakSNP10348	*Kabuli*	*Ca_Kabuli_Chr06*	16113521	(G/T)
CakSNP10349	*Kabuli*	*Ca_Kabuli_Chr06*	16115933	(T/C)
CakSNP10350	*Kabuli*	*Ca_Kabuli_Chr06*	16147469	(A/G)
CakSNP10351	*Kabuli*	*Ca_Kabuli_Chr06*	16148277	(A/C)
CakSNP10352	*Kabuli*	*Ca_Kabuli_Chr06*	16189692	(C/A)
CakSNP10353	*Kabuli*	*Ca_Kabuli_Chr06*	16189693	(G/T)
CakSNP10354	*Kabuli*	*Ca_Kabuli_Chr06*	16195748	(T/C)
CakSNP10355	*Kabuli*	*Ca_Kabuli_Chr06*	16196582	(G/C)
CakSNP10356	*Kabuli*	*Ca_Kabuli_Chr06*	16315552	(T/A)
CakSNP10357	*Kabuli*	*Ca_Kabuli_Chr06*	16332377	(G/A)
CakSNP10358	*Kabuli*	*Ca_Kabuli_Chr06*	16353698	(G/A)
CakSNP10359	*Kabuli*	*Ca_Kabuli_Chr06*	16450107	(C/T)
CakSNP10360	*Kabuli*	*Ca_Kabuli_Chr06*	16460495	(A/C)
CakSNP10361	*Kabuli*	*Ca_Kabuli_Chr06*	16561334	(C/T)
CakSNP10362	*Kabuli*	*Ca_Kabuli_Chr06*	16635061	(C/T)
CakSNP10363	*Kabuli*	*Ca_Kabuli_Chr06*	16646769	(A/T)
CakSNP10364	*Kabuli*	*Ca_Kabuli_Chr06*	16646713	(T/G)
CakSNP10365	*Kabuli*	*Ca_Kabuli_Chr06*	16665385	(G/C)
CakSNP10366	*Kabuli*	*Ca_Kabuli_Chr06*	16665477	(T/C)
CakSNP10367	*Kabuli*	*Ca_Kabuli_Chr06*	16665829	(G/C)
CakSNP10368	*Kabuli*	*Ca_Kabuli_Chr06*	16677320	(C/A)
CakSNP10369	*Kabuli*	*Ca_Kabuli_Chr06*	16717984	(G/A)
CakSNP10370	*Kabuli*	*Ca_Kabuli_Chr06*	16718201	(G/A)
CakSNP10371	*Kabuli*	*Ca_Kabuli_Chr06*	16719918	(C/A)
CakSNP10372	*Kabuli*	*Ca_Kabuli_Chr06*	16719919	(T/G)

SNP IDs	Cultivars	Chromosomes/scaffolds	Physical positions (bp)	SNPs
CakSNP10373	*Kabuli*	*Ca_Kabuli_Chr06*	16741672	(C/T)
CakSNP10374	*Kabuli*	*Ca_Kabuli_Chr06*	16748675	(A/C)
CakSNP10375	*Kabuli*	*Ca_Kabuli_Chr06*	16768782	(G/A)
CakSNP10376	*Kabuli*	*Ca_Kabuli_Chr06*	16823954	(A/G)
CakSNP10377	*Kabuli*	*Ca_Kabuli_Chr06*	16932014	(C/G)
CakSNP10378	*Kabuli*	*Ca_Kabuli_Chr06*	17048493	(A/C)
CakSNP10379	*Kabuli*	*Ca_Kabuli_Chr06*	17096214	(C/T)
CakSNP10380	*Kabuli*	*Ca_Kabuli_Chr06*	17096215	(G/A)
CakSNP10381	*Kabuli*	*Ca_Kabuli_Chr06*	17096224	(C/T)
CakSNP10382	*Kabuli*	*Ca_Kabuli_Chr06*	17129574	(A/G)
CakSNP10383	*Kabuli*	*Ca_Kabuli_Chr06*	17148605	(G/A)
CakSNP10384	*Kabuli*	*Ca_Kabuli_Chr06*	17148773	(A/G)
CakSNP10385	*Kabuli*	*Ca_Kabuli_Chr06*	17175334	(C/A)
CakSNP10386	*Kabuli*	*Ca_Kabuli_Chr06*	17175446	(G/A)
CakSNP10387	*Kabuli*	*Ca_Kabuli_Chr06*	17258827	(C/A)
CakSNP10388	*Kabuli*	*Ca_Kabuli_Chr06*	17258886	(T/G)
CakSNP10389	*Kabuli*	*Ca_Kabuli_Chr06*	17262283	(C/T)
CakSNP10390	*Kabuli*	*Ca_Kabuli_Chr06*	17263126	(G/A)
CakSNP10391	*Kabuli*	*Ca_Kabuli_Chr06*	17369613	(A/G)
CakSNP10392	*Kabuli*	*Ca_Kabuli_Chr06*	17445945	(T/G)
CakSNP10393	*Kabuli*	*Ca_Kabuli_Chr06*	17477741	(A/G)
CakSNP10394	*Kabuli*	*Ca_Kabuli_Chr06*	17478220	(G/A)
CakSNP10395	*Kabuli*	*Ca_Kabuli_Chr06*	17480486	(G/C)
CakSNP10396	*Kabuli*	*Ca_Kabuli_Chr06*	17481288	(T/C)
CakSNP10397	*Kabuli*	*Ca_Kabuli_Chr06*	17481307	(C/A)
CakSNP10398	*Kabuli*	*Ca_Kabuli_Chr06*	17481403	(T/G)
CakSNP10399	*Kabuli*	*Ca_Kabuli_Chr06*	17481392	(A/G)
CakSNP10400	*Kabuli*	*Ca_Kabuli_Chr06*	17482626	(A/G)
CakSNP10401	*Kabuli*	*Ca_Kabuli_Chr06*	17482640	(C/T)
CakSNP10402	*Kabuli*	*Ca_Kabuli_Chr06*	17482649	(A/G)
CakSNP10403	*Kabuli*	*Ca_Kabuli_Chr06*	17482718	(G/A)
CakSNP10404	*Kabuli*	*Ca_Kabuli_Chr06*	17482697	(A/G)
CakSNP10405	*Kabuli*	*Ca_Kabuli_Chr06*	17573309	(A/G)
CakSNP10406	*Kabuli*	*Ca_Kabuli_Chr06*	17659067	(A/C)
CakSNP10407	*Kabuli*	*Ca_Kabuli_Chr06*	17741078	(G/A)
CakSNP10408	*Kabuli*	*Ca_Kabuli_Chr06*	18027391	(G/A)
CakSNP10409	*Kabuli*	*Ca_Kabuli_Chr06*	18094691	(C/A)
CakSNP10410	*Kabuli*	*Ca_Kabuli_Chr06*	18125613	(C/A)
CakSNP10411	*Kabuli*	*Ca_Kabuli_Chr06*	18232696	(G/A)
CakSNP10412	*Kabuli*	*Ca_Kabuli_Chr06*	18268960	(G/A)
CakSNP10413	*Kabuli*	*Ca_Kabuli_Chr06*	18268982	(A/G)

SNP IDs	Cultivars	Chromosomes/scaffolds	Physical positions (bp)	SNPs
CakSNP10414	*Kabuli*	*Ca_Kabuli_Chr06*	18389704	(T/G)
CakSNP10415	*Kabuli*	*Ca_Kabuli_Chr06*	18389661	(A/C)
CakSNP10416	*Kabuli*	*Ca_Kabuli_Chr06*	18444725	(T/C)
CakSNP10417	*Kabuli*	*Ca_Kabuli_Chr06*	18549073	(T/C)
CakSNP10418	*Kabuli*	*Ca_Kabuli_Chr06*	18562366	(C/A)
CakSNP10419	*Kabuli*	*Ca_Kabuli_Chr06*	18664114	(A/T)
CakSNP10420	*Kabuli*	*Ca_Kabuli_Chr06*	18747207	(G/A)
CakSNP10421	*Kabuli*	*Ca_Kabuli_Chr06*	18752554	(G/T)
CakSNP10422	*Kabuli*	*Ca_Kabuli_Chr06*	18767357	(G/A)
CakSNP10423	*Kabuli*	*Ca_Kabuli_Chr06*	18771093	(T/A)
CakSNP10424	*Kabuli*	*Ca_Kabuli_Chr06*	18798747	(A/C)
CakSNP10425	*Kabuli*	*Ca_Kabuli_Chr06*	18852806	(T/C)
CakSNP10426	*Kabuli*	*Ca_Kabuli_Chr06*	18852854	(T/C)
CakSNP10427	*Kabuli*	*Ca_Kabuli_Chr06*	18876221	(T/C)
CakSNP10428	*Kabuli*	*Ca_Kabuli_Chr06*	18876213	(A/G)
CakSNP10429	*Kabuli*	*Ca_Kabuli_Chr06*	18899989	(C/A)
CakSNP10430	*Kabuli*	*Ca_Kabuli_Chr06*	18900031	(C/T)
CakSNP10431	*Kabuli*	*Ca_Kabuli_Chr06*	18900034	(G/A)
CakSNP10432	*Kabuli*	*Ca_Kabuli_Chr06*	18916626	(G/T)
CakSNP10433	*Kabuli*	*Ca_Kabuli_Chr06*	18916619	(C/T)
CakSNP10434	*Kabuli*	*Ca_Kabuli_Chr06*	18925002	(A/G)
CakSNP10435	*Kabuli*	*Ca_Kabuli_Chr06*	18938479	(C/T)
CakSNP10436	*Kabuli*	*Ca_Kabuli_Chr06*	18938512	(G/A)
CakSNP10437	*Kabuli*	*Ca_Kabuli_Chr06*	18956194	(G/A)
CakSNP10438	*Kabuli*	*Ca_Kabuli_Chr06*	18987220	(T/G)
CakSNP10439	*Kabuli*	*Ca_Kabuli_Chr06*	18999175	(C/T)
CakSNP10440	*Kabuli*	*Ca_Kabuli_Chr06*	19033068	(A/C)
CakSNP10441	*Kabuli*	*Ca_Kabuli_Chr06*	19033309	(G/A)
CakSNP10442	*Kabuli*	*Ca_Kabuli_Chr06*	19034129	(A/G)
CakSNP10443	*Kabuli*	*Ca_Kabuli_Chr06*	19125254	(G/A)
CakSNP10444	*Kabuli*	*Ca_Kabuli_Chr06*	19125222	(T/C)
CakSNP10445	*Kabuli*	*Ca_Kabuli_Chr06*	19129651	(T/C)
CakSNP10446	*Kabuli*	*Ca_Kabuli_Chr06*	19169687	(T/C)
CakSNP10447	*Kabuli*	*Ca_Kabuli_Chr06*	19195656	(A/T)
CakSNP10448	*Kabuli*	*Ca_Kabuli_Chr06*	19195659	(A/C)
CakSNP10449	*Kabuli*	*Ca_Kabuli_Chr06*	19198386	(G/A)
CakSNP10450	*Kabuli*	*Ca_Kabuli_Chr06*	19217820	(G/A)
CakSNP10451	*Kabuli*	*Ca_Kabuli_Chr06*	19304834	(A/G)
CakSNP10452	*Kabuli*	*Ca_Kabuli_Chr06*	19440988	(T/C)
CakSNP10453	*Kabuli*	*Ca_Kabuli_Chr06*	19441372	(T/C)
CakSNP10454	*Kabuli*	*Ca_Kabuli_Chr06*	19450845	(T/A)

SNP IDs	Cultivars	Chromosomes/scaffolds	Physical positions (bp)	SNPs
CakSNP10455	*Kabuli*	*Ca_Kabuli_Chr06*	19458843	(C/T)
CakSNP10456	*Kabuli*	*Ca_Kabuli_Chr06*	19458898	(T/A)
CakSNP10457	*Kabuli*	*Ca_Kabuli_Chr06*	19458908	(A/T)
CakSNP10458	*Kabuli*	*Ca_Kabuli_Chr06*	19476915	(T/C)
CakSNP10459	*Kabuli*	*Ca_Kabuli_Chr06*	19476916	(A/G)
CakSNP10460	*Kabuli*	*Ca_Kabuli_Chr06*	19477006	(T/C)
CakSNP10461	*Kabuli*	*Ca_Kabuli_Chr06*	19478493	(T/A)
CakSNP10462	*Kabuli*	*Ca_Kabuli_Chr06*	19494588	(G/T)
CakSNP10463	*Kabuli*	*Ca_Kabuli_Chr06*	19494584	(C/A)
CakSNP10464	*Kabuli*	*Ca_Kabuli_Chr06*	19551459	(G/A)
CakSNP10465	*Kabuli*	*Ca_Kabuli_Chr06*	19551504	(G/C)
CakSNP10466	*Kabuli*	*Ca_Kabuli_Chr06*	19556904	(G/A)
CakSNP10467	*Kabuli*	*Ca_Kabuli_Chr06*	19557045	(C/T)
CakSNP10468	*Kabuli*	*Ca_Kabuli_Chr06*	19578972	(T/C)
CakSNP10469	*Kabuli*	*Ca_Kabuli_Chr06*	19725007	(T/A)
CakSNP10470	*Kabuli*	*Ca_Kabuli_Chr06*	20505641	(A/T)
CakSNP10471	*Kabuli*	*Ca_Kabuli_Chr06*	20558142	(C/T)
CakSNP10472	*Kabuli*	*Ca_Kabuli_Chr06*	20558138	(G/A)
CakSNP10473	*Kabuli*	*Ca_Kabuli_Chr06*	20660779	(T/C)
CakSNP10474	*Kabuli*	*Ca_Kabuli_Chr06*	20660782	(T/C)
CakSNP10475	*Kabuli*	*Ca_Kabuli_Chr06*	20660819	(T/C)
CakSNP10476	*Kabuli*	*Ca_Kabuli_Chr06*	20660820	(T/A)
CakSNP10477	*Kabuli*	*Ca_Kabuli_Chr06*	20660841	(A/G)
CakSNP10478	*Kabuli*	*Ca_Kabuli_Chr06*	20660870	(G/C)
CakSNP10479	*Kabuli*	*Ca_Kabuli_Chr06*	20671043	(T/C)
CakSNP10480	*Kabuli*	*Ca_Kabuli_Chr06*	20739258	(A/T)
CakSNP10481	*Kabuli*	*Ca_Kabuli_Chr06*	20739340	(A/G)
CakSNP10482	*Kabuli*	*Ca_Kabuli_Chr06*	20766342	(T/A)
CakSNP10483	*Kabuli*	*Ca_Kabuli_Chr06*	20766377	(C/A)
CakSNP10484	*Kabuli*	*Ca_Kabuli_Chr06*	20766397	(G/T)
CakSNP10485	*Kabuli*	*Ca_Kabuli_Chr06*	20770728	(C/T)
CakSNP10486	*Kabuli*	*Ca_Kabuli_Chr06*	20776473	(A/G)
CakSNP10487	*Kabuli*	*Ca_Kabuli_Chr06*	20786079	(T/A)
CakSNP10488	*Kabuli*	*Ca_Kabuli_Chr06*	20802747	(G/C)
CakSNP10489	*Kabuli*	*Ca_Kabuli_Chr06*	20830353	(T/G)
CakSNP10490	*Kabuli*	*Ca_Kabuli_Chr06*	20830634	(T/A)
CakSNP10491	*Kabuli*	*Ca_Kabuli_Chr06*	20890714	(A/G)
CakSNP10492	*Kabuli*	*Ca_Kabuli_Chr06*	20917713	(A/C)
CakSNP10493	*Kabuli*	*Ca_Kabuli_Chr06*	20917694	(A/T)
CakSNP10494	*Kabuli*	*Ca_Kabuli_Chr06*	21025495	(T/A)
CakSNP10495	*Kabuli*	*Ca_Kabuli_Chr06*	21025494	(C/T)

**SNP IDs**	**Cultivars**	**Chromosomes/scaffolds**	**Physical positions (bp)**	**SNPs**
CakSNP10496	*Kabuli*	*Ca_Kabuli_Chr06*	21025540	(C/T)
CakSNP10497	*Kabuli*	*Ca_Kabuli_Chr06*	21025677	(T/A)
CakSNP10498	*Kabuli*	*Ca_Kabuli_Chr06*	21025742	(T/G)
CakSNP10499	*Kabuli*	*Ca_Kabuli_Chr06*	21025706	(C/T)
CakSNP10500	*Kabuli*	*Ca_Kabuli_Chr06*	21025673	(C/T)
CakSNP10501	*Kabuli*	*Ca_Kabuli_Chr06*	21025680	(C/T)
CakSNP10502	*Kabuli*	*Ca_Kabuli_Chr06*	21025682	(C/T)
CakSNP10503	*Kabuli*	*Ca_Kabuli_Chr06*	21025712	(C/T)
CakSNP10504	*Kabuli*	*Ca_Kabuli_Chr06*	21025718	(C/A)
CakSNP10505	*Kabuli*	*Ca_Kabuli_Chr06*	21025894	(G/T)
CakSNP10506	*Kabuli*	*Ca_Kabuli_Chr06*	21054758	(T/C)
CakSNP10507	*Kabuli*	*Ca_Kabuli_Chr06*	21058286	(G/T)
CakSNP10508	*Kabuli*	*Ca_Kabuli_Chr06*	21135764	(C/T)
CakSNP10509	*Kabuli*	*Ca_Kabuli_Chr06*	21135996	(C/T)
CakSNP10510	*Kabuli*	*Ca_Kabuli_Chr06*	21139038	(C/G)
CakSNP10511	*Kabuli*	*Ca_Kabuli_Chr06*	21165963	(A/C)
CakSNP10512	*Kabuli*	*Ca_Kabuli_Chr06*	21165999	(G/T)
CakSNP10513	*Kabuli*	*Ca_Kabuli_Chr06*	21170382	(G/A)
CakSNP10514	*Kabuli*	*Ca_Kabuli_Chr06*	21170401	(C/A)
CakSNP10515	*Kabuli*	*Ca_Kabuli_Chr06*	21177330	(C/A)
CakSNP10516	*Kabuli*	*Ca_Kabuli_Chr06*	21238178	(A/G)
CakSNP10517	*Kabuli*	*Ca_Kabuli_Chr06*	21238711	(C/T)
CakSNP10518	*Kabuli*	*Ca_Kabuli_Chr06*	21238707	(G/T)
CakSNP10519	*Kabuli*	*Ca_Kabuli_Chr06*	21276479	(G/T)
CakSNP10520	*Kabuli*	*Ca_Kabuli_Chr06*	21276551	(C/G)
CakSNP10521	*Kabuli*	*Ca_Kabuli_Chr06*	21323438	(T/C)
CakSNP10522	*Kabuli*	*Ca_Kabuli_Chr06*	21323390	(C/G)
CakSNP10523	*Kabuli*	*Ca_Kabuli_Chr06*	21367869	(A/G)
CakSNP10524	*Kabuli*	*Ca_Kabuli_Chr06*	21367830	(T/A)
CakSNP10525	*Kabuli*	*Ca_Kabuli_Chr06*	21368163	(C/T)
CakSNP10526	*Kabuli*	*Ca_Kabuli_Chr06*	21385915	(C/A)
CakSNP10527	*Kabuli*	*Ca_Kabuli_Chr06*	21478820	(G/T)
CakSNP10528	*Kabuli*	*Ca_Kabuli_Chr06*	21478816	(C/T)
CakSNP10529	*Kabuli*	*Ca_Kabuli_Chr06*	21483425	(T/G)
CakSNP10530	*Kabuli*	*Ca_Kabuli_Chr06*	21483429	(C/T)
CakSNP10531	*Kabuli*	*Ca_Kabuli_Chr06*	21483494	(T/G)
CakSNP10532	*Kabuli*	*Ca_Kabuli_Chr06*	21489498	(A/G)
CakSNP10533	*Kabuli*	*Ca_Kabuli_Chr06*	21506224	(A/C)
CakSNP10534	*Kabuli*	*Ca_Kabuli_Chr06*	21540338	(A/G)
CakSNP10535	*Kabuli*	*Ca_Kabuli_Chr06*	21540382	(T/G)
CakSNP10536	*Kabuli*	*Ca_Kabuli_Chr06*	21576745	(G/A)

**SNP IDs**	**Cultivars**	**Chromosomes/scaffolds**	**Physical positions (bp)**	**SNPs**
CakSNP10537	*Kabuli*	*Ca_Kabuli_Chr06*	21602874	(C/T)
CakSNP10538	*Kabuli*	*Ca_Kabuli_Chr06*	21603102	(G/C)
CakSNP10539	*Kabuli*	*Ca_Kabuli_Chr06*	21675734	(T/G)
CakSNP10540	*Kabuli*	*Ca_Kabuli_Chr06*	21675757	(A/G)
CakSNP10541	*Kabuli*	*Ca_Kabuli_Chr06*	21675762	(G/T)
CakSNP10542	*Kabuli*	*Ca_Kabuli_Chr06*	21675816	(C/G)
CakSNP10543	*Kabuli*	*Ca_Kabuli_Chr06*	21718674	(G/T)
CakSNP10544	*Kabuli*	*Ca_Kabuli_Chr06*	21756666	(A/T)
CakSNP10545	*Kabuli*	*Ca_Kabuli_Chr06*	21756706	(C/T)
CakSNP10546	*Kabuli*	*Ca_Kabuli_Chr06*	21763468	(G/A)
CakSNP10547	*Kabuli*	*Ca_Kabuli_Chr06*	21763611	(C/T)
CakSNP10548	*Kabuli*	*Ca_Kabuli_Chr06*	21763582	(C/T)
CakSNP10549	*Kabuli*	*Ca_Kabuli_Chr06*	21804333	(T/A)
CakSNP10550	*Kabuli*	*Ca_Kabuli_Chr06*	21804337	(G/C)
CakSNP10551	*Kabuli*	*Ca_Kabuli_Chr06*	21834111	(C/G)
CakSNP10552	*Kabuli*	*Ca_Kabuli_Chr06*	21861700	(T/A)
CakSNP10553	*Kabuli*	*Ca_Kabuli_Chr06*	21914387	(T/C)
CakSNP10554	*Kabuli*	*Ca_Kabuli_Chr06*	21914380	(C/T)
CakSNP10555	*Kabuli*	*Ca_Kabuli_Chr06*	21916465	(G/A)
CakSNP10556	*Kabuli*	*Ca_Kabuli_Chr06*	21916476	(T/C)
CakSNP10557	*Kabuli*	*Ca_Kabuli_Chr06*	21940793	(T/C)
CakSNP10558	*Kabuli*	*Ca_Kabuli_Chr06*	21979846	(G/A)
CakSNP10559	*Kabuli*	*Ca_Kabuli_Chr06*	21979860	(C/T)
CakSNP10560	*Kabuli*	*Ca_Kabuli_Chr06*	21979873	(A/G)
CakSNP10561	*Kabuli*	*Ca_Kabuli_Chr06*	21979887	(G/A)
CakSNP10562	*Kabuli*	*Ca_Kabuli_Chr06*	21979889	(A/G)
CakSNP10563	*Kabuli*	*Ca_Kabuli_Chr06*	21979893	(C/T)
CakSNP10564	*Kabuli*	*Ca_Kabuli_Chr06*	21979903	(T/C)
CakSNP10565	*Kabuli*	*Ca_Kabuli_Chr06*	22015915	(G/T)
CakSNP10566	*Kabuli*	*Ca_Kabuli_Chr06*	22016086	(A/G)
CakSNP10567	*Kabuli*	*Ca_Kabuli_Chr06*	22016767	(C/T)
CakSNP10568	*Kabuli*	*Ca_Kabuli_Chr06*	22016839	(A/G)
CakSNP10569	*Kabuli*	*Ca_Kabuli_Chr06*	22038857	(A/G)
CakSNP10570	*Kabuli*	*Ca_Kabuli_Chr06*	22038906	(A/T)
CakSNP10571	*Kabuli*	*Ca_Kabuli_Chr06*	22074809	(T/C)
CakSNP10572	*Kabuli*	*Ca_Kabuli_Chr06*	22075447	(C/G)
CakSNP10573	*Kabuli*	*Ca_Kabuli_Chr06*	22079148	(A/G)
CakSNP10574	*Kabuli*	*Ca_Kabuli_Chr06*	22137477	(A/T)
CakSNP10575	*Kabuli*	*Ca_Kabuli_Chr06*	22137464	(G/A)
CakSNP10576	*Kabuli*	*Ca_Kabuli_Chr06*	22137569	(G/T)
CakSNP10577	*Kabuli*	*Ca_Kabuli_Chr06*	22175547	(A/G)

**SNP IDs**	**Cultivars**	**Chromosomes/scaffolds**	**Physical positions (bp)**	**SNPs**
CakSNP10578	*Kabuli*	*Ca_Kabuli_Chr06*	22192503	(G/A)
CakSNP10579	*Kabuli*	*Ca_Kabuli_Chr06*	22192498	(A/C)
CakSNP10580	*Kabuli*	*Ca_Kabuli_Chr06*	22194387	(C/A)
CakSNP10581	*Kabuli*	*Ca_Kabuli_Chr06*	22201688	(C/T)
CakSNP10582	*Kabuli*	*Ca_Kabuli_Chr06*	22206341	(G/T)
CakSNP10583	*Kabuli*	*Ca_Kabuli_Chr06*	22208130	(G/C)
CakSNP10584	*Kabuli*	*Ca_Kabuli_Chr06*	22208125	(G/A)
CakSNP10585	*Kabuli*	*Ca_Kabuli_Chr06*	22208093	(G/A)
CakSNP10586	*Kabuli*	*Ca_Kabuli_Chr06*	22211721	(C/T)
CakSNP10587	*Kabuli*	*Ca_Kabuli_Chr06*	22211823	(T/C)
CakSNP10588	*Kabuli*	*Ca_Kabuli_Chr06*	22217158	(A/G)
CakSNP10589	*Kabuli*	*Ca_Kabuli_Chr06*	22217157	(T/G)
CakSNP10590	*Kabuli*	*Ca_Kabuli_Chr06*	22217107	(G/A)
CakSNP10591	*Kabuli*	*Ca_Kabuli_Chr06*	22225288	(T/G)
CakSNP10592	*Kabuli*	*Ca_Kabuli_Chr06*	22235449	(G/A)
CakSNP10593	*Kabuli*	*Ca_Kabuli_Chr06*	22235421	(T/A)
CakSNP10594	*Kabuli*	*Ca_Kabuli_Chr06*	22236475	(T/A)
CakSNP10595	*Kabuli*	*Ca_Kabuli_Chr06*	22252149	(G/A)
CakSNP10596	*Kabuli*	*Ca_Kabuli_Chr06*	22262999	(A/C)
CakSNP10597	*Kabuli*	*Ca_Kabuli_Chr06*	22263011	(T/C)
CakSNP10598	*Kabuli*	*Ca_Kabuli_Chr06*	22263388	(A/G)
CakSNP10599	*Kabuli*	*Ca_Kabuli_Chr06*	22263452	(A/T)
CakSNP10600	*Kabuli*	*Ca_Kabuli_Chr06*	22279906	(T/G)
CakSNP10601	*Kabuli*	*Ca_Kabuli_Chr06*	22287922	(C/T)
CakSNP10602	*Kabuli*	*Ca_Kabuli_Chr06*	22334733	(C/T)
CakSNP10603	*Kabuli*	*Ca_Kabuli_Chr06*	22352976	(T/C)
CakSNP10604	*Kabuli*	*Ca_Kabuli_Chr06*	22370242	(C/T)
CakSNP10605	*Kabuli*	*Ca_Kabuli_Chr06*	22371908	(A/C)
CakSNP10606	*Kabuli*	*Ca_Kabuli_Chr06*	22446629	(G/A)
CakSNP10607	*Kabuli*	*Ca_Kabuli_Chr06*	22446621	(A/C)
CakSNP10608	*Kabuli*	*Ca_Kabuli_Chr06*	22446610	(G/A)
CakSNP10609	*Kabuli*	*Ca_Kabuli_Chr06*	22446570	(C/T)
CakSNP10610	*Kabuli*	*Ca_Kabuli_Chr06*	22446649	(C/T)
CakSNP10611	*Kabuli*	*Ca_Kabuli_Chr06*	22446644	(C/T)
CakSNP10612	*Kabuli*	*Ca_Kabuli_Chr06*	22482366	(A/C)
CakSNP10613	*Kabuli*	*Ca_Kabuli_Chr06*	22498553	(T/C)
CakSNP10614	*Kabuli*	*Ca_Kabuli_Chr06*	22498867	(T/C)
CakSNP10615	*Kabuli*	*Ca_Kabuli_Chr06*	22518747	(G/T)
CakSNP10616	*Kabuli*	*Ca_Kabuli_Chr06*	22879982	(C/T)
CakSNP10617	*Kabuli*	*Ca_Kabuli_Chr06*	22881176	(A/T)
CakSNP10618	*Kabuli*	*Ca_Kabuli_Chr06*	22933938	(T/A)

SNP IDs	Cultivars	Chromosomes/scaffolds	Physical positions (bp)	SNPs
CakSNP10619	*Kabuli*	*Ca_Kabuli_Chr06*	22952185	(G/A)
CakSNP10620	*Kabuli*	*Ca_Kabuli_Chr06*	22952253	(C/T)
CakSNP10621	*Kabuli*	*Ca_Kabuli_Chr06*	22966001	(C/A)
CakSNP10622	*Kabuli*	*Ca_Kabuli_Chr06*	22986891	(T/C)
CakSNP10623	*Kabuli*	*Ca_Kabuli_Chr06*	22986869	(G/C)
CakSNP10624	*Kabuli*	*Ca_Kabuli_Chr06*	23014724	(T/A)
CakSNP10625	*Kabuli*	*Ca_Kabuli_Chr06*	23039165	(T/C)
CakSNP10626	*Kabuli*	*Ca_Kabuli_Chr06*	23039147	(G/A)
CakSNP10627	*Kabuli*	*Ca_Kabuli_Chr06*	23039145	(A/G)
CakSNP10628	*Kabuli*	*Ca_Kabuli_Chr06*	23039125	(G/A)
CakSNP10629	*Kabuli*	*Ca_Kabuli_Chr06*	23039097	(C/T)
CakSNP10630	*Kabuli*	*Ca_Kabuli_Chr06*	23046076	(C/T)
CakSNP10631	*Kabuli*	*Ca_Kabuli_Chr06*	23113706	(A/G)
CakSNP10632	*Kabuli*	*Ca_Kabuli_Chr06*	23129516	(A/G)
CakSNP10633	*Kabuli*	*Ca_Kabuli_Chr06*	23129504	(C/G)
CakSNP10634	*Kabuli*	*Ca_Kabuli_Chr06*	23145532	(T/A)
CakSNP10635	*Kabuli*	*Ca_Kabuli_Chr06*	23166355	(A/C)
CakSNP10636	*Kabuli*	*Ca_Kabuli_Chr06*	23253687	(G/A)
CakSNP10637	*Kabuli*	*Ca_Kabuli_Chr06*	23253973	(G/C)
CakSNP10638	*Kabuli*	*Ca_Kabuli_Chr06*	23254642	(A/G)
CakSNP10639	*Kabuli*	*Ca_Kabuli_Chr06*	23256848	(C/G)
CakSNP10640	*Kabuli*	*Ca_Kabuli_Chr06*	23256891	(T/G)
CakSNP10641	*Kabuli*	*Ca_Kabuli_Chr06*	23380746	(G/A)
CakSNP10642	*Kabuli*	*Ca_Kabuli_Chr06*	23393426	(T/C)
CakSNP10643	*Kabuli*	*Ca_Kabuli_Chr06*	23393446	(A/G)
CakSNP10644	*Kabuli*	*Ca_Kabuli_Chr06*	23393469	(T/C)
CakSNP10645	*Kabuli*	*Ca_Kabuli_Chr06*	23393491	(T/G)
CakSNP10646	*Kabuli*	*Ca_Kabuli_Chr06*	23399103	(A/G)
CakSNP10647	*Kabuli*	*Ca_Kabuli_Chr06*	23424061	(T/G)
CakSNP10648	*Kabuli*	*Ca_Kabuli_Chr06*	23549220	(A/T)
CakSNP10649	*Kabuli*	*Ca_Kabuli_Chr06*	23638882	(A/G)
CakSNP10650	*Kabuli*	*Ca_Kabuli_Chr06*	23663204	(C/A)
CakSNP10651	*Kabuli*	*Ca_Kabuli_Chr06*	23663201	(T/C)
CakSNP10652	*Kabuli*	*Ca_Kabuli_Chr06*	23663180	(G/T)
CakSNP10653	*Kabuli*	*Ca_Kabuli_Chr06*	23688740	(C/T)
CakSNP10654	*Kabuli*	*Ca_Kabuli_Chr06*	23688723	(A/G)
CakSNP10655	*Kabuli*	*Ca_Kabuli_Chr06*	23691440	(A/C)
CakSNP10656	*Kabuli*	*Ca_Kabuli_Chr06*	23766943	(C/T)
CakSNP10657	*Kabuli*	*Ca_Kabuli_Chr06*	23768142	(A/T)
CakSNP10658	*Kabuli*	*Ca_Kabuli_Chr06*	23809120	(G/A)
CakSNP10659	*Kabuli*	*Ca_Kabuli_Chr06*	23890836	(A/G)

**SNP IDs**	**Cultivars**	**Chromosomes/scaffolds**	**Physical positions (bp)**	**SNPs**
CakSNP10660	*Kabuli*	*Ca_Kabuli_Chr06*	23891065	(G/A)
CakSNP10661	*Kabuli*	*Ca_Kabuli_Chr06*	23916260	(A/G)
CakSNP10662	*Kabuli*	*Ca_Kabuli_Chr06*	23963812	(T/C)
CakSNP10663	*Kabuli*	*Ca_Kabuli_Chr06*	23973410	(G/A)
CakSNP10664	*Kabuli*	*Ca_Kabuli_Chr06*	24007185	(C/T)
CakSNP10665	*Kabuli*	*Ca_Kabuli_Chr06*	24007863	(T/C)
CakSNP10666	*Kabuli*	*Ca_Kabuli_Chr06*	24061578	(G/T)
CakSNP10667	*Kabuli*	*Ca_Kabuli_Chr06*	24085747	(G/T)
CakSNP10668	*Kabuli*	*Ca_Kabuli_Chr06*	24085795	(C/T)
CakSNP10669	*Kabuli*	*Ca_Kabuli_Chr06*	24085807	(A/T)
CakSNP10670	*Kabuli*	*Ca_Kabuli_Chr06*	24138357	(A/G)
CakSNP10671	*Kabuli*	*Ca_Kabuli_Chr06*	24138388	(T/C)
CakSNP10672	*Kabuli*	*Ca_Kabuli_Chr06*	24174940	(C/T)
CakSNP10673	*Kabuli*	*Ca_Kabuli_Chr06*	24260527	(T/C)
CakSNP10674	*Kabuli*	*Ca_Kabuli_Chr06*	24301508	(T/A)
CakSNP10675	*Kabuli*	*Ca_Kabuli_Chr06*	24302205	(A/G)
CakSNP10676	*Kabuli*	*Ca_Kabuli_Chr06*	24381949	(A/G)
CakSNP10677	*Kabuli*	*Ca_Kabuli_Chr06*	24381937	(G/A)
CakSNP10678	*Kabuli*	*Ca_Kabuli_Chr06*	24403316	(G/T)
CakSNP10679	*Kabuli*	*Ca_Kabuli_Chr06*	24413675	(T/C)
CakSNP10680	*Kabuli*	*Ca_Kabuli_Chr06*	24413714	(G/A)
CakSNP10681	*Kabuli*	*Ca_Kabuli_Chr06*	24414512	(G/C)
CakSNP10682	*Kabuli*	*Ca_Kabuli_Chr06*	24454583	(C/T)
CakSNP10683	*Kabuli*	*Ca_Kabuli_Chr06*	24454557	(T/C)
CakSNP10684	*Kabuli*	*Ca_Kabuli_Chr06*	24516523	(A/T)
CakSNP10685	*Kabuli*	*Ca_Kabuli_Chr06*	24516528	(A/T)
CakSNP10686	*Kabuli*	*Ca_Kabuli_Chr06*	24516532	(C/T)
CakSNP10687	*Kabuli*	*Ca_Kabuli_Chr06*	24547086	(T/G)
CakSNP10688	*Kabuli*	*Ca_Kabuli_Chr06*	24644924	(T/G)
CakSNP10689	*Kabuli*	*Ca_Kabuli_Chr06*	24661895	(G/A)
CakSNP10690	*Kabuli*	*Ca_Kabuli_Chr06*	24661916	(C/T)
CakSNP10691	*Kabuli*	*Ca_Kabuli_Chr06*	24661937	(C/T)
CakSNP10692	*Kabuli*	*Ca_Kabuli_Chr06*	24661945	(T/A)
CakSNP10693	*Kabuli*	*Ca_Kabuli_Chr06*	24661986	(G/A)
CakSNP10694	*Kabuli*	*Ca_Kabuli_Chr06*	25575097	(T/C)
CakSNP10695	*Kabuli*	*Ca_Kabuli_Chr06*	25575078	(C/T)
CakSNP10696	*Kabuli*	*Ca_Kabuli_Chr06*	25575062	(G/A)
CakSNP10697	*Kabuli*	*Ca_Kabuli_Chr06*	25575045	(C/A)
CakSNP10698	*Kabuli*	*Ca_Kabuli_Chr06*	25604942	(C/A)
CakSNP10699	*Kabuli*	*Ca_Kabuli_Chr06*	25604936	(C/T)
CakSNP10700	*Kabuli*	*Ca_Kabuli_Chr06*	25604931	(C/A)

SNP IDs	Cultivars	Chromosomes/scaffolds	Physical positions (bp)	SNPs
CakSNP10701	*Kabuli*	*Ca_Kabuli_Chr06*	25604922	(A/G)
CakSNP10702	*Kabuli*	*Ca_Kabuli_Chr06*	25604954	(C/T)
CakSNP10703	*Kabuli*	*Ca_Kabuli_Chr06*	25604962	(G/A)
CakSNP10704	*Kabuli*	*Ca_Kabuli_Chr06*	25604994	(T/C)
CakSNP10705	*Kabuli*	*Ca_Kabuli_Chr06*	25605006	(C/A)
CakSNP10706	*Kabuli*	*Ca_Kabuli_Chr06*	25605023	(C/A)
CakSNP10707	*Kabuli*	*Ca_Kabuli_Chr06*	25605022	(T/C)
CakSNP10708	*Kabuli*	*Ca_Kabuli_Chr06*	25850717	(C/T)
CakSNP10709	*Kabuli*	*Ca_Kabuli_Chr06*	25850725	(T/G)
CakSNP10710	*Kabuli*	*Ca_Kabuli_Chr06*	25850736	(C/T)
CakSNP10711	*Kabuli*	*Ca_Kabuli_Chr06*	25850760	(T/A)
CakSNP10712	*Kabuli*	*Ca_Kabuli_Chr06*	25850759	(C/A)
CakSNP10713	*Kabuli*	*Ca_Kabuli_Chr06*	25850783	(G/A)
CakSNP10714	*Kabuli*	*Ca_Kabuli_Chr06*	25850816	(C/A)
CakSNP10715	*Kabuli*	*Ca_Kabuli_Chr06*	25850813	(C/T)
CakSNP10716	*Kabuli*	*Ca_Kabuli_Chr06*	25850866	(C/G)
CakSNP10717	*Kabuli*	*Ca_Kabuli_Chr06*	25850827	(C/T)
CakSNP10718	*Kabuli*	*Ca_Kabuli_Chr06*	26349363	(C/A)
CakSNP10719	*Kabuli*	*Ca_Kabuli_Chr06*	26349321	(C/T)
CakSNP10720	*Kabuli*	*Ca_Kabuli_Chr06*	26349434	(G/A)
CakSNP10721	*Kabuli*	*Ca_Kabuli_Chr06*	26349451	(T/C)
CakSNP10722	*Kabuli*	*Ca_Kabuli_Chr06*	26353512	(C/T)
CakSNP10723	*Kabuli*	*Ca_Kabuli_Chr06*	26353740	(G/C)
CakSNP10724	*Kabuli*	*Ca_Kabuli_Chr06*	26357149	(C/G)
CakSNP10725	*Kabuli*	*Ca_Kabuli_Chr06*	26360367	(C/A)
CakSNP10726	*Kabuli*	*Ca_Kabuli_Chr06*	26385448	(A/G)
CakSNP10727	*Kabuli*	*Ca_Kabuli_Chr06*	26385444	(C/T)
CakSNP10728	*Kabuli*	*Ca_Kabuli_Chr06*	26385432	(T/C)
CakSNP10729	*Kabuli*	*Ca_Kabuli_Chr06*	26426239	(C/A)
CakSNP10730	*Kabuli*	*Ca_Kabuli_Chr06*	26426252	(A/G)
CakSNP10731	*Kabuli*	*Ca_Kabuli_Chr06*	26426283	(G/A)
CakSNP10732	*Kabuli*	*Ca_Kabuli_Chr06*	26527086	(T/C)
CakSNP10733	*Kabuli*	*Ca_Kabuli_Chr06*	26527111	(G/T)
CakSNP10734	*Kabuli*	*Ca_Kabuli_Chr06*	26527161	(C/G)
CakSNP10735	*Kabuli*	*Ca_Kabuli_Chr06*	26527416	(C/T)
CakSNP10736	*Kabuli*	*Ca_Kabuli_Chr06*	26549168	(T/G)
CakSNP10737	*Kabuli*	*Ca_Kabuli_Chr06*	26549181	(C/T)
CakSNP10738	*Kabuli*	*Ca_Kabuli_Chr06*	26551390	(C/T)
CakSNP10739	*Kabuli*	*Ca_Kabuli_Chr06*	26551703	(T/G)
CakSNP10740	*Kabuli*	*Ca_Kabuli_Chr06*	26567360	(A/G)
CakSNP10741	*Kabuli*	*Ca_Kabuli_Chr06*	26656016	(T/C)

SNP IDs	Cultivars	Chromosomes/scaffolds	Physical positions (bp)	SNPs
CakSNP10742	*Kabuli*	*Ca_Kabuli_Chr06*	26698265	(A/C)
CakSNP10743	*Kabuli*	*Ca_Kabuli_Chr06*	26703130	(A/G)
CakSNP10744	*Kabuli*	*Ca_Kabuli_Chr06*	26703132	(T/C)
CakSNP10745	*Kabuli*	*Ca_Kabuli_Chr06*	26767389	(T/C)
CakSNP10746	*Kabuli*	*Ca_Kabuli_Chr06*	26769087	(C/A)
CakSNP10747	*Kabuli*	*Ca_Kabuli_Chr06*	26928255	(T/C)
CakSNP10748	*Kabuli*	*Ca_Kabuli_Chr06*	27053217	(A/G)
CakSNP10749	*Kabuli*	*Ca_Kabuli_Chr06*	27053288	(G/C)
CakSNP10750	*Kabuli*	*Ca_Kabuli_Chr06*	27139791	(T/C)
CakSNP10751	*Kabuli*	*Ca_Kabuli_Chr06*	27139816	(T/C)
CakSNP10752	*Kabuli*	*Ca_Kabuli_Chr06*	27190671	(G/A)
CakSNP10753	*Kabuli*	*Ca_Kabuli_Chr06*	27192557	(C/T)
CakSNP10754	*Kabuli*	*Ca_Kabuli_Chr06*	27310342	(A/T)
CakSNP10755	*Kabuli*	*Ca_Kabuli_Chr06*	27310341	(G/T)
CakSNP10756	*Kabuli*	*Ca_Kabuli_Chr06*	27310336	(A/T)
CakSNP10757	*Kabuli*	*Ca_Kabuli_Chr06*	27310333	(C/T)
CakSNP10758	*Kabuli*	*Ca_Kabuli_Chr06*	27310328	(T/A)
CakSNP10759	*Kabuli*	*Ca_Kabuli_Chr06*	27310327	(A/T)
CakSNP10760	*Kabuli*	*Ca_Kabuli_Chr06*	27310325	(G/T)
CakSNP10761	*Kabuli*	*Ca_Kabuli_Chr06*	27310315	(C/T)
CakSNP10762	*Kabuli*	*Ca_Kabuli_Chr06*	27326420	(G/A)
CakSNP10763	*Kabuli*	*Ca_Kabuli_Chr06*	27326978	(C/T)
CakSNP10764	*Kabuli*	*Ca_Kabuli_Chr06*	27330059	(T/C)
CakSNP10765	*Kabuli*	*Ca_Kabuli_Chr06*	27330159	(C/T)
CakSNP10766	*Kabuli*	*Ca_Kabuli_Chr06*	27331165	(T/C)
CakSNP10767	*Kabuli*	*Ca_Kabuli_Chr06*	27331318	(A/C)
CakSNP10768	*Kabuli*	*Ca_Kabuli_Chr06*	27364381	(A/G)
CakSNP10769	*Kabuli*	*Ca_Kabuli_Chr06*	27364395	(C/T)
CakSNP10770	*Kabuli*	*Ca_Kabuli_Chr06*	27364402	(T/A)
CakSNP10771	*Kabuli*	*Ca_Kabuli_Chr06*	27364428	(G/T)
CakSNP10772	*Kabuli*	*Ca_Kabuli_Chr06*	27364452	(A/T)
CakSNP10773	*Kabuli*	*Ca_Kabuli_Chr06*	27383746	(A/T)
CakSNP10774	*Kabuli*	*Ca_Kabuli_Chr06*	27383745	(G/T)
CakSNP10775	*Kabuli*	*Ca_Kabuli_Chr06*	27383744	(G/T)
CakSNP10776	*Kabuli*	*Ca_Kabuli_Chr06*	27383718	(C/T)
CakSNP10777	*Kabuli*	*Ca_Kabuli_Chr06*	27515184	(C/T)
CakSNP10778	*Kabuli*	*Ca_Kabuli_Chr06*	27515293	(G/A)
CakSNP10779	*Kabuli*	*Ca_Kabuli_Chr06*	27537433	(A/G)
CakSNP10780	*Kabuli*	*Ca_Kabuli_Chr06*	27558154	(A/G)
CakSNP10781	*Kabuli*	*Ca_Kabuli_Chr06*	27578968	(A/G)
CakSNP10782	*Kabuli*	*Ca_Kabuli_Chr06*	27592117	(G/T)

SNP IDs	Cultivars	Chromosomes/scaffolds	Physical positions (bp)	SNPs
CakSNP10783	*Kabuli*	*Ca_Kabuli_Chr06*	27592070	(A/G)
CakSNP10784	*Kabuli*	*Ca_Kabuli_Chr06*	27606193	(A/C)
CakSNP10785	*Kabuli*	*Ca_Kabuli_Chr06*	27606210	(C/T)
CakSNP10786	*Kabuli*	*Ca_Kabuli_Chr06*	27622037	(A/C)
CakSNP10787	*Kabuli*	*Ca_Kabuli_Chr06*	27622045	(A/G)
CakSNP10788	*Kabuli*	*Ca_Kabuli_Chr06*	27622053	(A/G)
CakSNP10789	*Kabuli*	*Ca_Kabuli_Chr06*	27636256	(A/G)
CakSNP10790	*Kabuli*	*Ca_Kabuli_Chr06*	27655605	(A/G)
CakSNP10791	*Kabuli*	*Ca_Kabuli_Chr06*	27660980	(C/T)
CakSNP10792	*Kabuli*	*Ca_Kabuli_Chr06*	27660971	(T/C)
CakSNP10793	*Kabuli*	*Ca_Kabuli_Chr06*	27661951	(C/T)
CakSNP10794	*Kabuli*	*Ca_Kabuli_Chr06*	27662026	(C/T)
CakSNP10795	*Kabuli*	*Ca_Kabuli_Chr06*	27710531	(T/G)
CakSNP10796	*Kabuli*	*Ca_Kabuli_Chr06*	27710547	(A/T)
CakSNP10797	*Kabuli*	*Ca_Kabuli_Chr06*	27842297	(G/A)
CakSNP10798	*Kabuli*	*Ca_Kabuli_Chr06*	27865048	(C/T)
CakSNP10799	*Kabuli*	*Ca_Kabuli_Chr06*	27865046	(G/A)
CakSNP10800	*Kabuli*	*Ca_Kabuli_Chr06*	27864997	(C/A)
CakSNP10801	*Kabuli*	*Ca_Kabuli_Chr06*	27986719	(A/G)
CakSNP10802	*Kabuli*	*Ca_Kabuli_Chr06*	28021899	(C/T)
CakSNP10803	*Kabuli*	*Ca_Kabuli_Chr06*	28044830	(C/A)
CakSNP10804	*Kabuli*	*Ca_Kabuli_Chr06*	28052463	(T/G)
CakSNP10805	*Kabuli*	*Ca_Kabuli_Chr06*	28074393	(T/C)
CakSNP10806	*Kabuli*	*Ca_Kabuli_Chr06*	28076323	(T/A)
CakSNP10807	*Kabuli*	*Ca_Kabuli_Chr06*	28076364	(T/G)
CakSNP10808	*Kabuli*	*Ca_Kabuli_Chr06*	28076436	(A/T)
CakSNP10809	*Kabuli*	*Ca_Kabuli_Chr06*	28177397	(A/C)
CakSNP10810	*Kabuli*	*Ca_Kabuli_Chr06*	28325392	(C/T)
CakSNP10811	*Kabuli*	*Ca_Kabuli_Chr06*	28372130	(C/T)
CakSNP10812	*Kabuli*	*Ca_Kabuli_Chr06*	28574699	(T/C)
CakSNP10813	*Kabuli*	*Ca_Kabuli_Chr06*	28575509	(T/C)
CakSNP10814	*Kabuli*	*Ca_Kabuli_Chr06*	28575491	(G/A)
CakSNP10815	*Kabuli*	*Ca_Kabuli_Chr06*	28615404	(G/T)
CakSNP10816	*Kabuli*	*Ca_Kabuli_Chr06*	28615475	(A/G)
CakSNP10817	*Kabuli*	*Ca_Kabuli_Chr06*	28650056	(A/G)
CakSNP10818	*Kabuli*	*Ca_Kabuli_Chr06*	28727478	(T/C)
CakSNP10819	*Kabuli*	*Ca_Kabuli_Chr06*	28728293	(A/C)
CakSNP10820	*Kabuli*	*Ca_Kabuli_Chr06*	28755298	(T/A)
CakSNP10821	*Kabuli*	*Ca_Kabuli_Chr06*	28757208	(C/A)
CakSNP10822	*Kabuli*	*Ca_Kabuli_Chr06*	28757204	(G/A)
CakSNP10823	*Kabuli*	*Ca_Kabuli_Chr06*	28757139	(C/A)

**SNP IDs**	**Cultivars**	**Chromosomes/scaffolds**	**Physical positions (bp)**	**SNPs**
CakSNP10824	*Kabuli*	*Ca_Kabuli_Chr06*	28887176	(G/A)
CakSNP10825	*Kabuli*	*Ca_Kabuli_Chr06*	28887188	(G/T)
CakSNP10826	*Kabuli*	*Ca_Kabuli_Chr06*	28887201	(A/G)
CakSNP10827	*Kabuli*	*Ca_Kabuli_Chr06*	28887212	(C/T)
CakSNP10828	*Kabuli*	*Ca_Kabuli_Chr06*	28892743	(G/C)
CakSNP10829	*Kabuli*	*Ca_Kabuli_Chr06*	28893411	(C/T)
CakSNP10830	*Kabuli*	*Ca_Kabuli_Chr06*	28917705	(T/A)
CakSNP10831	*Kabuli*	*Ca_Kabuli_Chr06*	28947529	(A/C)
CakSNP10832	*Kabuli*	*Ca_Kabuli_Chr06*	28990518	(A/G)
CakSNP10833	*Kabuli*	*Ca_Kabuli_Chr06*	29085558	(T/C)
CakSNP10834	*Kabuli*	*Ca_Kabuli_Chr06*	29163667	(C/A)
CakSNP10835	*Kabuli*	*Ca_Kabuli_Chr06*	29163714	(G/C)
CakSNP10836	*Kabuli*	*Ca_Kabuli_Chr06*	29163699	(T/A)
CakSNP10837	*Kabuli*	*Ca_Kabuli_Chr06*	29163642	(G/A)
CakSNP10838	*Kabuli*	*Ca_Kabuli_Chr06*	29163640	(A/C)
CakSNP10839	*Kabuli*	*Ca_Kabuli_Chr06*	29203129	(C/G)
CakSNP10840	*Kabuli*	*Ca_Kabuli_Chr06*	29203201	(G/C)
CakSNP10841	*Kabuli*	*Ca_Kabuli_Chr06*	29203210	(T/C)
CakSNP10842	*Kabuli*	*Ca_Kabuli_Chr06*	29205260	(A/G)
CakSNP10843	*Kabuli*	*Ca_Kabuli_Chr06*	29205940	(A/G)
CakSNP10844	*Kabuli*	*Ca_Kabuli_Chr06*	29206008	(A/G)
CakSNP10845	*Kabuli*	*Ca_Kabuli_Chr06*	29307254	(A/G)
CakSNP10846	*Kabuli*	*Ca_Kabuli_Chr06*	29495856	(C/T)
CakSNP10847	*Kabuli*	*Ca_Kabuli_Chr06*	29934610	(A/T)
CakSNP10848	*Kabuli*	*Ca_Kabuli_Chr06*	30127756	(T/G)
CakSNP10849	*Kabuli*	*Ca_Kabuli_Chr06*	30257217	(C/T)
CakSNP10850	*Kabuli*	*Ca_Kabuli_Chr06*	30257284	(A/C)
CakSNP10851	*Kabuli*	*Ca_Kabuli_Chr06*	30567073	(G/A)
CakSNP10852	*Kabuli*	*Ca_Kabuli_Chr06*	30676031	(G/T)
CakSNP10853	*Kabuli*	*Ca_Kabuli_Chr06*	30676320	(T/C)
CakSNP10854	*Kabuli*	*Ca_Kabuli_Chr06*	30676248	(C/T)
CakSNP10855	*Kabuli*	*Ca_Kabuli_Chr06*	30761620	(C/T)
CakSNP10856	*Kabuli*	*Ca_Kabuli_Chr06*	30761641	(G/T)
CakSNP10857	*Kabuli*	*Ca_Kabuli_Chr06*	30913932	(T/C)
CakSNP10858	*Kabuli*	*Ca_Kabuli_Chr06*	30926084	(G/A)
CakSNP10859	*Kabuli*	*Ca_Kabuli_Chr06*	30927221	(C/G)
CakSNP10860	*Kabuli*	*Ca_Kabuli_Chr06*	31011743	(A/G)
CakSNP10861	*Kabuli*	*Ca_Kabuli_Chr06*	31024728	(A/T)
CakSNP10862	*Kabuli*	*Ca_Kabuli_Chr06*	31025560	(C/G)
CakSNP10863	*Kabuli*	*Ca_Kabuli_Chr06*	31038808	(C/T)
CakSNP10864	*Kabuli*	*Ca_Kabuli_Chr06*	31048949	(T/C)

**SNP IDs**	**Cultivars**	**Chromosomes/scaffolds**	**Physical positions (bp)**	**SNPs**
CakSNP10865	*Kabuli*	*Ca_Kabuli_Chr06*	31057806	(C/T)
CakSNP10866	*Kabuli*	*Ca_Kabuli_Chr06*	31068678	(G/A)
CakSNP10867	*Kabuli*	*Ca_Kabuli_Chr06*	31324962	(A/C)
CakSNP10868	*Kabuli*	*Ca_Kabuli_Chr06*	31324976	(A/G)
CakSNP10869	*Kabuli*	*Ca_Kabuli_Chr06*	31408895	(G/T)
CakSNP10870	*Kabuli*	*Ca_Kabuli_Chr06*	31408969	(C/T)
CakSNP10871	*Kabuli*	*Ca_Kabuli_Chr06*	31495897	(C/A)
CakSNP10872	*Kabuli*	*Ca_Kabuli_Chr06*	31495903	(A/G)
CakSNP10873	*Kabuli*	*Ca_Kabuli_Chr06*	31592707	(A/C)
CakSNP10874	*Kabuli*	*Ca_Kabuli_Chr06*	31595142	(A/C)
CakSNP10875	*Kabuli*	*Ca_Kabuli_Chr06*	31595136	(A/T)
CakSNP10876	*Kabuli*	*Ca_Kabuli_Chr06*	31895214	(G/C)
CakSNP10877	*Kabuli*	*Ca_Kabuli_Chr06*	31895264	(C/T)
CakSNP10878	*Kabuli*	*Ca_Kabuli_Chr06*	31900781	(T/G)
CakSNP10879	*Kabuli*	*Ca_Kabuli_Chr06*	31900785	(T/G)
CakSNP10880	*Kabuli*	*Ca_Kabuli_Chr06*	31900790	(A/C)
CakSNP10881	*Kabuli*	*Ca_Kabuli_Chr06*	31966982	(C/T)
CakSNP10882	*Kabuli*	*Ca_Kabuli_Chr06*	31967089	(T/G)
CakSNP10883	*Kabuli*	*Ca_Kabuli_Chr06*	31967106	(G/A)
CakSNP10884	*Kabuli*	*Ca_Kabuli_Chr06*	31967109	(T/C)
CakSNP10885	*Kabuli*	*Ca_Kabuli_Chr06*	31967144	(T/C)
CakSNP10886	*Kabuli*	*Ca_Kabuli_Chr06*	31967201	(C/T)
CakSNP10887	*Kabuli*	*Ca_Kabuli_Chr06*	31967195	(G/C)
CakSNP10888	*Kabuli*	*Ca_Kabuli_Chr06*	31992907	(A/G)
CakSNP10889	*Kabuli*	*Ca_Kabuli_Chr06*	32035882	(C/T)
CakSNP10890	*Kabuli*	*Ca_Kabuli_Chr06*	32036033	(G/A)
CakSNP10891	*Kabuli*	*Ca_Kabuli_Chr06*	32035976	(T/C)
CakSNP10892	*Kabuli*	*Ca_Kabuli_Chr06*	32140428	(C/A)
CakSNP10893	*Kabuli*	*Ca_Kabuli_Chr06*	32140429	(G/T)
CakSNP10894	*Kabuli*	*Ca_Kabuli_Chr06*	32262807	(T/C)
CakSNP10895	*Kabuli*	*Ca_Kabuli_Chr06*	32262855	(C/T)
CakSNP10896	*Kabuli*	*Ca_Kabuli_Chr06*	32328468	(T/C)
CakSNP10897	*Kabuli*	*Ca_Kabuli_Chr06*	32717450	(G/A)
CakSNP10898	*Kabuli*	*Ca_Kabuli_Chr06*	32717665	(C/A)
CakSNP10899	*Kabuli*	*Ca_Kabuli_Chr06*	32825567	(C/A)
CakSNP10900	*Kabuli*	*Ca_Kabuli_Chr06*	32825561	(C/T)
CakSNP10901	*Kabuli*	*Ca_Kabuli_Chr06*	32825547	(A/G)
CakSNP10902	*Kabuli*	*Ca_Kabuli_Chr06*	32825579	(C/T)
CakSNP10903	*Kabuli*	*Ca_Kabuli_Chr06*	32825587	(G/A)
CakSNP10904	*Kabuli*	*Ca_Kabuli_Chr06*	32825612	(G/A)
CakSNP10905	*Kabuli*	*Ca_Kabuli_Chr06*	32825619	(T/C)

**SNP IDs**	**Cultivars**	**Chromosomes/scaffolds**	**Physical positions (bp)**	**SNPs**
CakSNP10906	*Kabuli*	*Ca_Kabuli_Chr06*	32825631	(C/A)
CakSNP10907	*Kabuli*	*Ca_Kabuli_Chr06*	32914927	(C/G)
CakSNP10908	*Kabuli*	*Ca_Kabuli_Chr06*	33502994	(C/A)
CakSNP10909	*Kabuli*	*Ca_Kabuli_Chr06*	33502997	(T/A)
CakSNP10910	*Kabuli*	*Ca_Kabuli_Chr06*	33503007	(T/C)
CakSNP10911	*Kabuli*	*Ca_Kabuli_Chr06*	33503014	(G/T)
CakSNP10912	*Kabuli*	*Ca_Kabuli_Chr06*	33503020	(G/A)
CakSNP10913	*Kabuli*	*Ca_Kabuli_Chr06*	33503032	(A/C)
CakSNP10914	*Kabuli*	*Ca_Kabuli_Chr06*	33503037	(A/T)
CakSNP10915	*Kabuli*	*Ca_Kabuli_Chr06*	33503051	(G/C)
CakSNP10916	*Kabuli*	*Ca_Kabuli_Chr06*	33503063	(C/G)
CakSNP10917	*Kabuli*	*Ca_Kabuli_Chr06*	33503065	(C/A)
CakSNP10918	*Kabuli*	*Ca_Kabuli_Chr06*	33503071	(A/G)
CakSNP10919	*Kabuli*	*Ca_Kabuli_Chr06*	33598926	(G/A)
CakSNP10920	*Kabuli*	*Ca_Kabuli_Chr06*	33704792	(C/T)
CakSNP10921	*Kabuli*	*Ca_Kabuli_Chr06*	33704807	(C/A)
CakSNP10922	*Kabuli*	*Ca_Kabuli_Chr06*	33704839	(C/T)
CakSNP10923	*Kabuli*	*Ca_Kabuli_Chr06*	33704782	(C/T)
CakSNP10924	*Kabuli*	*Ca_Kabuli_Chr06*	33733259	(A/G)
CakSNP10925	*Kabuli*	*Ca_Kabuli_Chr06*	34235650	(T/C)
CakSNP10926	*Kabuli*	*Ca_Kabuli_Chr06*	34407309	(T/C)
CakSNP10927	*Kabuli*	*Ca_Kabuli_Chr06*	34481266	(A/G)
CakSNP10928	*Kabuli*	*Ca_Kabuli_Chr06*	34481256	(C/T)
CakSNP10929	*Kabuli*	*Ca_Kabuli_Chr06*	34535695	(T/C)
CakSNP10930	*Kabuli*	*Ca_Kabuli_Chr06*	34535983	(T/C)
CakSNP10931	*Kabuli*	*Ca_Kabuli_Chr06*	34596792	(A/G)
CakSNP10932	*Kabuli*	*Ca_Kabuli_Chr06*	34634028	(T/A)
CakSNP10933	*Kabuli*	*Ca_Kabuli_Chr06*	34744433	(A/G)
CakSNP10934	*Kabuli*	*Ca_Kabuli_Chr06*	34744438	(T/G)
CakSNP10935	*Kabuli*	*Ca_Kabuli_Chr06*	34744681	(T/G)
CakSNP10936	*Kabuli*	*Ca_Kabuli_Chr06*	34744665	(G/A)
CakSNP10937	*Kabuli*	*Ca_Kabuli_Chr06*	34773476	(T/A)
CakSNP10938	*Kabuli*	*Ca_Kabuli_Chr06*	34793312	(T/G)
CakSNP10939	*Kabuli*	*Ca_Kabuli_Chr06*	34974503	(C/G)
CakSNP10940	*Kabuli*	*Ca_Kabuli_Chr06*	35126227	(C/T)
CakSNP10941	*Kabuli*	*Ca_Kabuli_Chr06*	35126273	(T/C)
CakSNP10942	*Kabuli*	*Ca_Kabuli_Chr06*	35126389	(G/T)
CakSNP10943	*Kabuli*	*Ca_Kabuli_Chr06*	35129588	(G/A)
CakSNP10944	*Kabuli*	*Ca_Kabuli_Chr06*	35596037	(C/A)
CakSNP10945	*Kabuli*	*Ca_Kabuli_Chr06*	35596048	(G/A)
CakSNP10946	*Kabuli*	*Ca_Kabuli_Chr06*	35596064	(T/C)

SNP IDs	Cultivars	Chromosomes/scaffolds	Physical positions (bp)	SNPs
CakSNP10947	*Kabuli*	*Ca_Kabuli_Chr06*	35596070	(G/A)
CakSNP10948	*Kabuli*	*Ca_Kabuli_Chr06*	35596161	(C/T)
CakSNP10949	*Kabuli*	*Ca_Kabuli_Chr06*	35596174	(G/A)
CakSNP10950	*Kabuli*	*Ca_Kabuli_Chr06*	35596184	(A/G)
CakSNP10951	*Kabuli*	*Ca_Kabuli_Chr06*	35596195	(G/C)
CakSNP10952	*Kabuli*	*Ca_Kabuli_Chr06*	35596197	(G/A)
CakSNP10953	*Kabuli*	*Ca_Kabuli_Chr06*	35596207	(G/T)
CakSNP10954	*Kabuli*	*Ca_Kabuli_Chr06*	35596218	(T/C)
CakSNP10955	*Kabuli*	*Ca_Kabuli_Chr06*	35596220	(G/A)
CakSNP10956	*Kabuli*	*Ca_Kabuli_Chr06*	35596230	(T/A)
CakSNP10957	*Kabuli*	*Ca_Kabuli_Chr06*	35596300	(G/A)
CakSNP10958	*Kabuli*	*Ca_Kabuli_Chr06*	35628131	(T/C)
CakSNP10959	*Kabuli*	*Ca_Kabuli_Chr06*	35719930	(A/C)
CakSNP10960	*Kabuli*	*Ca_Kabuli_Chr06*	35743792	(A/G)
CakSNP10961	*Kabuli*	*Ca_Kabuli_Chr06*	35962309	(C/T)
CakSNP10962	*Kabuli*	*Ca_Kabuli_Chr06*	35962320	(T/C)
CakSNP10963	*Kabuli*	*Ca_Kabuli_Chr06*	35962360	(T/C)
CakSNP10964	*Kabuli*	*Ca_Kabuli_Chr06*	35962364	(T/G)
CakSNP10965	*Kabuli*	*Ca_Kabuli_Chr06*	35962365	(T/C)
CakSNP10966	*Kabuli*	*Ca_Kabuli_Chr06*	35962366	(G/A)
CakSNP10967	*Kabuli*	*Ca_Kabuli_Chr06*	36094855	(T/G)
CakSNP10968	*Kabuli*	*Ca_Kabuli_Chr06*	36371775	(A/G)
CakSNP10969	*Kabuli*	*Ca_Kabuli_Chr06*	36374561	(C/T)
CakSNP10970	*Kabuli*	*Ca_Kabuli_Chr06*	36574836	(A/G)
CakSNP10971	*Kabuli*	*Ca_Kabuli_Chr06*	36790467	(G/A)
CakSNP10972	*Kabuli*	*Ca_Kabuli_Chr06*	36790458	(G/A)
CakSNP10973	*Kabuli*	*Ca_Kabuli_Chr06*	36790428	(G/A)
CakSNP10974	*Kabuli*	*Ca_Kabuli_Chr06*	36790455	(C/T)
CakSNP10975	*Kabuli*	*Ca_Kabuli_Chr06*	36790460	(A/G)
CakSNP10976	*Kabuli*	*Ca_Kabuli_Chr06*	37139425	(C/T)
CakSNP10977	*Kabuli*	*Ca_Kabuli_Chr06*	37139466	(G/C)
CakSNP10978	*Kabuli*	*Ca_Kabuli_Chr06*	37139484	(T/C)
CakSNP10979	*Kabuli*	*Ca_Kabuli_Chr06*	37139486	(G/A)
CakSNP10980	*Kabuli*	*Ca_Kabuli_Chr06*	37139533	(T/A)
CakSNP10981	*Kabuli*	*Ca_Kabuli_Chr06*	37139514	(C/T)
CakSNP10982	*Kabuli*	*Ca_Kabuli_Chr06*	37139499	(C/G)
CakSNP10983	*Kabuli*	*Ca_Kabuli_Chr06*	37139610	(T/G)
CakSNP10984	*Kabuli*	*Ca_Kabuli_Chr06*	37139604	(G/T)
CakSNP10985	*Kabuli*	*Ca_Kabuli_Chr06*	37321783	(A/T)
CakSNP10986	*Kabuli*	*Ca_Kabuli_Chr06*	37476426	(C/T)
CakSNP10987	*Kabuli*	*Ca_Kabuli_Chr06*	37476437	(A/C)

**SNP IDs**	**Cultivars**	**Chromosomes/scaffolds**	**Physical positions (bp)**	**SNPs**
CakSNP10988	*Kabuli*	*Ca_Kabuli_Chr06*	37476516	(G/T)
CakSNP10989	*Kabuli*	*Ca_Kabuli_Chr06*	37476465	(G/A)
CakSNP10990	*Kabuli*	*Ca_Kabuli_Chr06*	37484467	(C/T)
CakSNP10991	*Kabuli*	*Ca_Kabuli_Chr06*	37484468	(G/T)
CakSNP10992	*Kabuli*	*Ca_Kabuli_Chr06*	37484470	(C/A)
CakSNP10993	*Kabuli*	*Ca_Kabuli_Chr06*	37484489	(C/G)
CakSNP10994	*Kabuli*	*Ca_Kabuli_Chr06*	37484513	(T/C)
CakSNP10995	*Kabuli*	*Ca_Kabuli_Chr06*	38816345	(C/T)
CakSNP10996	*Kabuli*	*Ca_Kabuli_Chr06*	38838320	(A/G)
CakSNP10997	*Kabuli*	*Ca_Kabuli_Chr06*	38840279	(G/A)
CakSNP10998	*Kabuli*	*Ca_Kabuli_Chr06*	38840315	(G/A)
CakSNP10999	*Kabuli*	*Ca_Kabuli_Chr06*	38840273	(G/A)
CakSNP11000	*Kabuli*	*Ca_Kabuli_Chr06*	38881909	(C/A)
CakSNP11001	*Kabuli*	*Ca_Kabuli_Chr06*	38886735	(C/A)
CakSNP11002	*Kabuli*	*Ca_Kabuli_Chr06*	38994879	(A/G)
CakSNP11003	*Kabuli*	*Ca_Kabuli_Chr06*	38994925	(G/T)
CakSNP11004	*Kabuli*	*Ca_Kabuli_Chr06*	38994954	(A/G)
CakSNP11005	*Kabuli*	*Ca_Kabuli_Chr06*	38994962	(G/A)
CakSNP11006	*Kabuli*	*Ca_Kabuli_Chr06*	39028564	(T/G)
CakSNP11007	*Kabuli*	*Ca_Kabuli_Chr06*	39107187	(C/T)
CakSNP11008	*Kabuli*	*Ca_Kabuli_Chr06*	39107149	(A/C)
CakSNP11009	*Kabuli*	*Ca_Kabuli_Chr06*	39107188	(A/C)
CakSNP11010	*Kabuli*	*Ca_Kabuli_Chr06*	39317016	(T/G)
CakSNP11011	*Kabuli*	*Ca_Kabuli_Chr06*	39380625	(A/C)
CakSNP11012	*Kabuli*	*Ca_Kabuli_Chr06*	39908314	(T/G)
CakSNP11013	*Kabuli*	*Ca_Kabuli_Chr06*	40047823	(A/G)
CakSNP11014	*Kabuli*	*Ca_Kabuli_Chr06*	40128644	(T/C)
CakSNP11015	*Kabuli*	*Ca_Kabuli_Chr06*	40468356	(T/A)
CakSNP11016	*Kabuli*	*Ca_Kabuli_Chr06*	40477285	(G/A)
CakSNP11017	*Kabuli*	*Ca_Kabuli_Chr06*	40657695	(A/C)
CakSNP11018	*Kabuli*	*Ca_Kabuli_Chr06*	40735186	(C/A)
CakSNP11019	*Kabuli*	*Ca_Kabuli_Chr06*	40767566	(A/G)
CakSNP11020	*Kabuli*	*Ca_Kabuli_Chr06*	40821354	(C/T)
CakSNP11021	*Kabuli*	*Ca_Kabuli_Chr06*	40935441	(A/T)
CakSNP11022	*Kabuli*	*Ca_Kabuli_Chr06*	41121337	(C/T)
CakSNP11023	*Kabuli*	*Ca_Kabuli_Chr06*	41121330	(G/A)
CakSNP11024	*Kabuli*	*Ca_Kabuli_Chr06*	41363546	(G/A)
CakSNP11025	*Kabuli*	*Ca_Kabuli_Chr06*	41420918	(T/A)
CakSNP11026	*Kabuli*	*Ca_Kabuli_Chr06*	41420917	(A/G)
CakSNP11027	*Kabuli*	*Ca_Kabuli_Chr06*	41449028	(C/T)
CakSNP11028	*Kabuli*	*Ca_Kabuli_Chr06*	41682983	(T/C)

**SNP IDs**	**Cultivars**	**Chromosomes/scaffolds**	**Physical positions (bp)**	**SNPs**
CakSNP11029	*Kabuli*	*Ca_Kabuli_Chr06*	41682966	(C/T)
CakSNP11030	*Kabuli*	*Ca_Kabuli_Chr06*	41682943	(T/C)
CakSNP11031	*Kabuli*	*Ca_Kabuli_Chr06*	41697812	(T/G)
CakSNP11032	*Kabuli*	*Ca_Kabuli_Chr06*	41708309	(G/A)
CakSNP11033	*Kabuli*	*Ca_Kabuli_Chr06*	41708306	(G/A)
CakSNP11034	*Kabuli*	*Ca_Kabuli_Chr06*	41708523	(T/G)
CakSNP11035	*Kabuli*	*Ca_Kabuli_Chr06*	41708474	(G/A)
CakSNP11036	*Kabuli*	*Ca_Kabuli_Chr06*	41708530	(C/A)
CakSNP11037	*Kabuli*	*Ca_Kabuli_Chr06*	41907459	(C/G)
CakSNP11038	*Kabuli*	*Ca_Kabuli_Chr06*	42004851	(A/G)
CakSNP11039	*Kabuli*	*Ca_Kabuli_Chr06*	42004829	(T/G)
CakSNP11040	*Kabuli*	*Ca_Kabuli_Chr06*	42004818	(C/G)
CakSNP11041	*Kabuli*	*Ca_Kabuli_Chr06*	42004813	(C/T)
CakSNP11042	*Kabuli*	*Ca_Kabuli_Chr06*	42161851	(A/G)
CakSNP11043	*Kabuli*	*Ca_Kabuli_Chr06*	42161857	(C/G)
CakSNP11044	*Kabuli*	*Ca_Kabuli_Chr06*	42161866	(T/G)
CakSNP11045	*Kabuli*	*Ca_Kabuli_Chr06*	42521162	(G/T)
CakSNP11046	*Kabuli*	*Ca_Kabuli_Chr06*	42855091	(T/G)
CakSNP11047	*Kabuli*	*Ca_Kabuli_Chr06*	42904998	(C/G)
CakSNP11048	*Kabuli*	*Ca_Kabuli_Chr06*	42905012	(G/A)
CakSNP11049	*Kabuli*	*Ca_Kabuli_Chr06*	42905039	(C/T)
CakSNP11050	*Kabuli*	*Ca_Kabuli_Chr06*	42905035	(T/A)
CakSNP11051	*Kabuli*	*Ca_Kabuli_Chr06*	42905111	(T/C)
CakSNP11052	*Kabuli*	*Ca_Kabuli_Chr06*	42905094	(C/T)
CakSNP11053	*Kabuli*	*Ca_Kabuli_Chr06*	42905090	(G/A)
CakSNP11054	*Kabuli*	*Ca_Kabuli_Chr06*	42905086	(T/C)
CakSNP11055	*Kabuli*	*Ca_Kabuli_Chr06*	42905058	(C/G)
CakSNP11056	*Kabuli*	*Ca_Kabuli_Chr06*	42905059	(A/G)
CakSNP11057	*Kabuli*	*Ca_Kabuli_Chr06*	42905072	(T/C)
CakSNP11058	*Kabuli*	*Ca_Kabuli_Chr06*	42905115	(G/T)
CakSNP11059	*Kabuli*	*Ca_Kabuli_Chr06*	42905116	(A/T)
CakSNP11060	*Kabuli*	*Ca_Kabuli_Chr06*	42919899	(G/A)
CakSNP11061	*Kabuli*	*Ca_Kabuli_Chr06*	42923969	(A/C)
CakSNP11062	*Kabuli*	*Ca_Kabuli_Chr06*	42925974	(C/T)
CakSNP11063	*Kabuli*	*Ca_Kabuli_Chr06*	43148829	(C/T)
CakSNP11064	*Kabuli*	*Ca_Kabuli_Chr06*	43148824	(C/A)
CakSNP11065	*Kabuli*	*Ca_Kabuli_Chr06*	43148784	(C/G)
CakSNP11066	*Kabuli*	*Ca_Kabuli_Chr06*	43905509	(A/T)
CakSNP11067	*Kabuli*	*Ca_Kabuli_Chr06*	43907445	(T/C)
CakSNP11068	*Kabuli*	*Ca_Kabuli_Chr06*	44530888	(C/A)
CakSNP11069	*Kabuli*	*Ca_Kabuli_Chr06*	44530889	(A/G)

SNP IDs	Cultivars	Chromosomes/scaffolds	Physical positions (bp)	SNPs
CakSNP11070	*Kabuli*	*Ca_Kabuli_Chr06*	44707313	(T/A)
CakSNP11071	*Kabuli*	*Ca_Kabuli_Chr06*	44711166	(A/G)
CakSNP11072	*Kabuli*	*Ca_Kabuli_Chr06*	44792535	(C/T)
CakSNP11073	*Kabuli*	*Ca_Kabuli_Chr06*	44792529	(G/C)
CakSNP11074	*Kabuli*	*Ca_Kabuli_Chr06*	44792493	(C/T)
CakSNP11075	*Kabuli*	*Ca_Kabuli_Chr06*	44792486	(G/A)
CakSNP11076	*Kabuli*	*Ca_Kabuli_Chr06*	44937666	(C/T)
CakSNP11077	*Kabuli*	*Ca_Kabuli_Chr06*	44944917	(G/A)
CakSNP11078	*Kabuli*	*Ca_Kabuli_Chr06*	45053093	(A/G)
CakSNP11079	*Kabuli*	*Ca_Kabuli_Chr06*	45053214	(C/T)
CakSNP11080	*Kabuli*	*Ca_Kabuli_Chr06*	45131295	(C/T)
CakSNP11081	*Kabuli*	*Ca_Kabuli_Chr06*	45132545	(A/G)
CakSNP11082	*Kabuli*	*Ca_Kabuli_Chr06*	45178060	(G/A)
CakSNP11083	*Kabuli*	*Ca_Kabuli_Chr06*	45178056	(A/G)
CakSNP11084	*Kabuli*	*Ca_Kabuli_Chr06*	45178045	(G/T)
CakSNP11085	*Kabuli*	*Ca_Kabuli_Chr06*	45178044	(G/A)
CakSNP11086	*Kabuli*	*Ca_Kabuli_Chr06*	45178033	(C/A)
CakSNP11087	*Kabuli*	*Ca_Kabuli_Chr06*	45178061	(C/T)
CakSNP11088	*Kabuli*	*Ca_Kabuli_Chr06*	45201783	(C/T)
CakSNP11089	*Kabuli*	*Ca_Kabuli_Chr06*	45293271	(T/C)
CakSNP11090	*Kabuli*	*Ca_Kabuli_Chr06*	45500961	(T/A)
CakSNP11091	*Kabuli*	*Ca_Kabuli_Chr06*	45501099	(C/A)
CakSNP11092	*Kabuli*	*Ca_Kabuli_Chr06*	45505942	(A/G)
CakSNP11093	*Kabuli*	*Ca_Kabuli_Chr06*	45550031	(C/A)
CakSNP11094	*Kabuli*	*Ca_Kabuli_Chr06*	45554040	(G/A)
CakSNP11095	*Kabuli*	*Ca_Kabuli_Chr06*	45557656	(T/C)
CakSNP11096	*Kabuli*	*Ca_Kabuli_Chr06*	45614017	(T/C)
CakSNP11097	*Kabuli*	*Ca_Kabuli_Chr06*	45637271	(T/G)
CakSNP11098	*Kabuli*	*Ca_Kabuli_Chr06*	45637292	(C/A)
CakSNP11099	*Kabuli*	*Ca_Kabuli_Chr06*	45637293	(T/C)
CakSNP11100	*Kabuli*	*Ca_Kabuli_Chr06*	45637322	(C/A)
CakSNP11101	*Kabuli*	*Ca_Kabuli_Chr06*	45637309	(G/A)
CakSNP11102	*Kabuli*	*Ca_Kabuli_Chr06*	45637308	(G/T)
CakSNP11103	*Kabuli*	*Ca_Kabuli_Chr06*	45693275	(G/A)
CakSNP11104	*Kabuli*	*Ca_Kabuli_Chr06*	45695051	(T/G)
CakSNP11105	*Kabuli*	*Ca_Kabuli_Chr06*	45695043	(C/T)
CakSNP11106	*Kabuli*	*Ca_Kabuli_Chr06*	45695035	(G/A)
CakSNP11107	*Kabuli*	*Ca_Kabuli_Chr06*	45709318	(T/C)
CakSNP11108	*Kabuli*	*Ca_Kabuli_Chr06*	45773318	(C/T)
CakSNP11109	*Kabuli*	*Ca_Kabuli_Chr06*	45773309	(A/G)
CakSNP11110	*Kabuli*	*Ca_Kabuli_Chr06*	45773298	(C/G)

SNP IDs	Cultivars	Chromosomes/scaffolds	Physical positions (bp)	SNPs
CakSNP11111	*Kabuli*	*Ca_Kabuli_Chr06*	45773409	(T/A)
CakSNP11112	*Kabuli*	*Ca_Kabuli_Chr06*	45773408	(T/A)
CakSNP11113	*Kabuli*	*Ca_Kabuli_Chr06*	45773373	(G/T)
CakSNP11114	*Kabuli*	*Ca_Kabuli_Chr06*	45773367	(G/T)
CakSNP11115	*Kabuli*	*Ca_Kabuli_Chr06*	45773349	(C/T)
CakSNP11116	*Kabuli*	*Ca_Kabuli_Chr06*	45812152	(G/A)
CakSNP11117	*Kabuli*	*Ca_Kabuli_Chr06*	45976962	(G/A)
CakSNP11118	*Kabuli*	*Ca_Kabuli_Chr06*	45978206	(T/C)
CakSNP11119	*Kabuli*	*Ca_Kabuli_Chr06*	45978263	(G/A)
CakSNP11120	*Kabuli*	*Ca_Kabuli_Chr06*	46158211	(C/T)
CakSNP11121	*Kabuli*	*Ca_Kabuli_Chr06*	46734706	(C/G)
CakSNP11122	*Kabuli*	*Ca_Kabuli_Chr06*	46734725	(G/A)
CakSNP11123	*Kabuli*	*Ca_Kabuli_Chr06*	46734726	(T/C)
CakSNP11124	*Kabuli*	*Ca_Kabuli_Chr06*	46765659	(G/A)
CakSNP11125	*Kabuli*	*Ca_Kabuli_Chr06*	46765661	(A/T)
CakSNP11126	*Kabuli*	*Ca_Kabuli_Chr06*	46765673	(G/A)
CakSNP11127	*Kabuli*	*Ca_Kabuli_Chr06*	46765688	(A/C)
CakSNP11128	*Kabuli*	*Ca_Kabuli_Chr06*	46765725	(C/T)
CakSNP11129	*Kabuli*	*Ca_Kabuli_Chr06*	46765778	(C/A)
CakSNP11130	*Kabuli*	*Ca_Kabuli_Chr06*	46765696	(C/T)
CakSNP11131	*Kabuli*	*Ca_Kabuli_Chr06*	46830497	(A/T)
CakSNP11132	*Kabuli*	*Ca_Kabuli_Chr06*	46896903	(C/T)
CakSNP11133	*Kabuli*	*Ca_Kabuli_Chr06*	46896910	(A/C)
CakSNP11134	*Kabuli*	*Ca_Kabuli_Chr06*	46898225	(A/C)
CakSNP11135	*Kabuli*	*Ca_Kabuli_Chr06*	46898305	(T/G)
CakSNP11136	*Kabuli*	*Ca_Kabuli_Chr06*	46898316	(T/G)
CakSNP11137	*Kabuli*	*Ca_Kabuli_Chr06*	46898317	(C/T)
CakSNP11138	*Kabuli*	*Ca_Kabuli_Chr06*	46898363	(C/T)
CakSNP11139	*Kabuli*	*Ca_Kabuli_Chr06*	46898329	(T/A)
CakSNP11140	*Kabuli*	*Ca_Kabuli_Chr06*	46898747	(G/A)
CakSNP11141	*Kabuli*	*Ca_Kabuli_Chr06*	46898743	(A/G)
CakSNP11142	*Kabuli*	*Ca_Kabuli_Chr06*	46899229	(G/A)
CakSNP11143	*Kabuli*	*Ca_Kabuli_Chr06*	46979240	(C/T)
CakSNP11144	*Kabuli*	*Ca_Kabuli_Chr06*	46979267	(C/A)
CakSNP11145	*Kabuli*	*Ca_Kabuli_Chr06*	46998948	(G/A)
CakSNP11146	*Kabuli*	*Ca_Kabuli_Chr06*	46999012	(C/T)
CakSNP11147	*Kabuli*	*Ca_Kabuli_Chr06*	47104478	(T/G)
CakSNP11148	*Kabuli*	*Ca_Kabuli_Chr06*	47104505	(C/T)
CakSNP11149	*Kabuli*	*Ca_Kabuli_Chr06*	47106174	(A/G)
CakSNP11150	*Kabuli*	*Ca_Kabuli_Chr06*	47106238	(C/A)
CakSNP11151	*Kabuli*	*Ca_Kabuli_Chr06*	47232630	(C/T)

SNP IDs	Cultivars	Chromosomes/scaffolds	Physical positions (bp)	SNPs
CakSNP11152	*Kabuli*	*Ca_Kabuli_Chr06*	47232631	(C/G)
CakSNP11153	*Kabuli*	*Ca_Kabuli_Chr06*	47232685	(T/A)
CakSNP11154	*Kabuli*	*Ca_Kabuli_Chr06*	47440468	(A/T)
CakSNP11155	*Kabuli*	*Ca_Kabuli_Chr06*	47575655	(G/T)
CakSNP11156	*Kabuli*	*Ca_Kabuli_Chr06*	47575659	(C/G)
CakSNP11157	*Kabuli*	*Ca_Kabuli_Chr06*	47906683	(G/A)
CakSNP11158	*Kabuli*	*Ca_Kabuli_Chr06*	47906680	(C/T)
CakSNP11159	*Kabuli*	*Ca_Kabuli_Chr06*	47906629	(T/G)
CakSNP11160	*Kabuli*	*Ca_Kabuli_Chr06*	48088550	(G/A)
CakSNP11161	*Kabuli*	*Ca_Kabuli_Chr06*	48088592	(C/T)
CakSNP11162	*Kabuli*	*Ca_Kabuli_Chr06*	48255392	(G/A)
CakSNP11163	*Kabuli*	*Ca_Kabuli_Chr06*	48255538	(A/G)
CakSNP11164	*Kabuli*	*Ca_Kabuli_Chr06*	48255558	(C/T)
CakSNP11165	*Kabuli*	*Ca_Kabuli_Chr06*	48255559	(A/G)
CakSNP11166	*Kabuli*	*Ca_Kabuli_Chr06*	48255568	(A/G)
CakSNP11167	*Kabuli*	*Ca_Kabuli_Chr06*	48255599	(C/A)
CakSNP11168	*Kabuli*	*Ca_Kabuli_Chr06*	48314052	(T/A)
CakSNP11169	*Kabuli*	*Ca_Kabuli_Chr06*	48314788	(T/C)
CakSNP11170	*Kabuli*	*Ca_Kabuli_Chr06*	48315194	(C/A)
CakSNP11171	*Kabuli*	*Ca_Kabuli_Chr06*	48315340	(C/A)
CakSNP11172	*Kabuli*	*Ca_Kabuli_Chr06*	48315343	(G/T)
CakSNP11173	*Kabuli*	*Ca_Kabuli_Chr06*	48810611	(A/T)
CakSNP11174	*Kabuli*	*Ca_Kabuli_Chr06*	48810608	(C/T)
CakSNP11175	*Kabuli*	*Ca_Kabuli_Chr06*	48864622	(T/C)
CakSNP11176	*Kabuli*	*Ca_Kabuli_Chr06*	48864625	(G/A)
CakSNP11177	*Kabuli*	*Ca_Kabuli_Chr06*	48864639	(A/C)
CakSNP11178	*Kabuli*	*Ca_Kabuli_Chr06*	48864699	(T/C)
CakSNP11179	*Kabuli*	*Ca_Kabuli_Chr06*	48869462	(C/T)
CakSNP11180	*Kabuli*	*Ca_Kabuli_Chr06*	48869473	(C/A)
CakSNP11181	*Kabuli*	*Ca_Kabuli_Chr06*	48869484	(G/A)
CakSNP11182	*Kabuli*	*Ca_Kabuli_Chr06*	48869577	(C/T)
CakSNP11183	*Kabuli*	*Ca_Kabuli_Chr06*	48869539	(A/G)
CakSNP11184	*Kabuli*	*Ca_Kabuli_Chr06*	48869529	(T/C)
CakSNP11185	*Kabuli*	*Ca_Kabuli_Chr06*	48869512	(G/C)
CakSNP11186	*Kabuli*	*Ca_Kabuli_Chr06*	48940386	(A/C)
CakSNP11187	*Kabuli*	*Ca_Kabuli_Chr06*	49044772	(A/C)
CakSNP11188	*Kabuli*	*Ca_Kabuli_Chr06*	49044785	(C/T)
CakSNP11189	*Kabuli*	*Ca_Kabuli_Chr06*	49044794	(T/A)
CakSNP11190	*Kabuli*	*Ca_Kabuli_Chr06*	49044802	(T/C)
CakSNP11191	*Kabuli*	*Ca_Kabuli_Chr06*	49044805	(A/G)
CakSNP11192	*Kabuli*	*Ca_Kabuli_Chr06*	49044814	(G/T)

SNP IDs	Cultivars	Chromosomes/scaffolds	Physical positions (bp)	SNPs
CakSNP11193	*Kabuli*	*Ca_Kabuli_Chr06*	49044824	(C/T)
CakSNP11194	*Kabuli*	*Ca_Kabuli_Chr06*	49044839	(T/A)
CakSNP11195	*Kabuli*	*Ca_Kabuli_Chr06*	49044850	(A/C)
CakSNP11196	*Kabuli*	*Ca_Kabuli_Chr06*	49044847	(G/A)
CakSNP11197	*Kabuli*	*Ca_Kabuli_Chr06*	49044842	(G/A)
CakSNP11198	*Kabuli*	*Ca_Kabuli_Chr06*	49044838	(C/A)
CakSNP11199	*Kabuli*	*Ca_Kabuli_Chr06*	49044835	(C/A)
CakSNP11200	*Kabuli*	*Ca_Kabuli_Chr06*	49044826	(G/A)
CakSNP11201	*Kabuli*	*Ca_Kabuli_Chr06*	49044821	(C/G)
CakSNP11202	*Kabuli*	*Ca_Kabuli_Chr06*	49044810	(G/A)
CakSNP11203	*Kabuli*	*Ca_Kabuli_Chr06*	49044808	(C/T)
CakSNP11204	*Kabuli*	*Ca_Kabuli_Chr06*	49044788	(T/A)
CakSNP11205	*Kabuli*	*Ca_Kabuli_Chr06*	49044780	(G/A)
CakSNP11206	*Kabuli*	*Ca_Kabuli_Chr06*	49044915	(A/G)
CakSNP11207	*Kabuli*	*Ca_Kabuli_Chr06*	49044928	(C/A)
CakSNP11208	*Kabuli*	*Ca_Kabuli_Chr06*	49044936	(C/T)
CakSNP11209	*Kabuli*	*Ca_Kabuli_Chr06*	49044937	(A/G)
CakSNP11210	*Kabuli*	*Ca_Kabuli_Chr06*	49044944	(A/G)
CakSNP11211	*Kabuli*	*Ca_Kabuli_Chr06*	49101829	(G/C)
CakSNP11212	*Kabuli*	*Ca_Kabuli_Chr06*	49167341	(T/G)
CakSNP11213	*Kabuli*	*Ca_Kabuli_Chr06*	49222291	(G/A)
CakSNP11214	*Kabuli*	*Ca_Kabuli_Chr06*	49222290	(C/T)
CakSNP11215	*Kabuli*	*Ca_Kabuli_Chr06*	49222275	(A/C)
CakSNP11216	*Kabuli*	*Ca_Kabuli_Chr06*	49222268	(T/A)
CakSNP11217	*Kabuli*	*Ca_Kabuli_Chr06*	49222251	(G/A)
CakSNP11218	*Kabuli*	*Ca_Kabuli_Chr06*	49222235	(G/C)
CakSNP11219	*Kabuli*	*Ca_Kabuli_Chr06*	49377275	(G/A)
CakSNP11220	*Kabuli*	*Ca_Kabuli_Chr06*	49403430	(G/C)
CakSNP11221	*Kabuli*	*Ca_Kabuli_Chr06*	49406604	(G/A)
CakSNP11222	*Kabuli*	*Ca_Kabuli_Chr06*	49406599	(C/A)
CakSNP11223	*Kabuli*	*Ca_Kabuli_Chr06*	49466066	(A/G)
CakSNP11224	*Kabuli*	*Ca_Kabuli_Chr06*	49475716	(G/A)
CakSNP11225	*Kabuli*	*Ca_Kabuli_Chr06*	49475715	(A/G)
CakSNP11226	*Kabuli*	*Ca_Kabuli_Chr06*	49524179	(A/T)
CakSNP11227	*Kabuli*	*Ca_Kabuli_Chr06*	49565934	(C/T)
CakSNP11228	*Kabuli*	*Ca_Kabuli_Chr06*	49664132	(C/G)
CakSNP11229	*Kabuli*	*Ca_Kabuli_Chr06*	49724448	(C/T)
CakSNP11230	*Kabuli*	*Ca_Kabuli_Chr06*	49724515	(C/T)
CakSNP11231	*Kabuli*	*Ca_Kabuli_Chr06*	49726734	(C/G)
CakSNP11232	*Kabuli*	*Ca_Kabuli_Chr06*	49726732	(T/C)
CakSNP11233	*Kabuli*	*Ca_Kabuli_Chr06*	49726729	(G/A)

**SNP IDs**	**Cultivars**	**Chromosomes/scaffolds**	**Physical positions (bp)**	**SNPs**
CakSNP11234	*Kabuli*	*Ca_Kabuli_Chr06*	49726711	(C/T)
CakSNP11235	*Kabuli*	*Ca_Kabuli_Chr06*	49726723	(G/A)
CakSNP11236	*Kabuli*	*Ca_Kabuli_Chr06*	49830299	(A/G)
CakSNP11237	*Kabuli*	*Ca_Kabuli_Chr06*	49830384	(G/A)
CakSNP11238	*Kabuli*	*Ca_Kabuli_Chr06*	49833934	(C/T)
CakSNP11239	*Kabuli*	*Ca_Kabuli_Chr06*	49843667	(G/A)
CakSNP11240	*Kabuli*	*Ca_Kabuli_Chr06*	49846257	(A/T)
CakSNP11241	*Kabuli*	*Ca_Kabuli_Chr06*	49846303	(T/G)
CakSNP11242	*Kabuli*	*Ca_Kabuli_Chr06*	49931821	(C/T)
CakSNP11243	*Kabuli*	*Ca_Kabuli_Chr06*	49931879	(T/C)
CakSNP11244	*Kabuli*	*Ca_Kabuli_Chr06*	49942397	(A/G)
CakSNP11245	*Kabuli*	*Ca_Kabuli_Chr06*	50086781	(A/G)
CakSNP11246	*Kabuli*	*Ca_Kabuli_Chr06*	50152053	(C/T)
CakSNP11247	*Kabuli*	*Ca_Kabuli_Chr06*	50152129	(C/T)
CakSNP11248	*Kabuli*	*Ca_Kabuli_Chr06*	50152124	(C/T)
CakSNP11249	*Kabuli*	*Ca_Kabuli_Chr06*	50152119	(T/C)
CakSNP11250	*Kabuli*	*Ca_Kabuli_Chr06*	50152083	(C/T)
CakSNP11251	*Kabuli*	*Ca_Kabuli_Chr06*	50217734	(G/A)
CakSNP11252	*Kabuli*	*Ca_Kabuli_Chr06*	50356239	(A/G)
CakSNP11253	*Kabuli*	*Ca_Kabuli_Chr06*	50356229	(C/T)
CakSNP11254	*Kabuli*	*Ca_Kabuli_Chr06*	50356222	(C/A)
CakSNP11255	*Kabuli*	*Ca_Kabuli_Chr06*	50356217	(T/C)
CakSNP11256	*Kabuli*	*Ca_Kabuli_Chr06*	50356206	(G/T)
CakSNP11257	*Kabuli*	*Ca_Kabuli_Chr06*	50356201	(T/C)
CakSNP11258	*Kabuli*	*Ca_Kabuli_Chr06*	50382452	(C/T)
CakSNP11259	*Kabuli*	*Ca_Kabuli_Chr06*	50384101	(A/C)
CakSNP11260	*Kabuli*	*Ca_Kabuli_Chr06*	50390752	(A/G)
CakSNP11261	*Kabuli*	*Ca_Kabuli_Chr06*	50476721	(T/C)
CakSNP11262	*Kabuli*	*Ca_Kabuli_Chr06*	50476775	(G/A)
CakSNP11263	*Kabuli*	*Ca_Kabuli_Chr06*	50477672	(A/C)
CakSNP11264	*Kabuli*	*Ca_Kabuli_Chr06*	50581295	(T/G)
CakSNP11265	*Kabuli*	*Ca_Kabuli_Chr06*	50617926	(G/C)
CakSNP11266	*Kabuli*	*Ca_Kabuli_Chr06*	50617932	(G/A)
CakSNP11267	*Kabuli*	*Ca_Kabuli_Chr06*	50723595	(T/C)
CakSNP11268	*Kabuli*	*Ca_Kabuli_Chr06*	50723630	(C/T)
CakSNP11269	*Kabuli*	*Ca_Kabuli_Chr06*	50723916	(T/C)
CakSNP11270	*Kabuli*	*Ca_Kabuli_Chr06*	50723950	(A/G)
CakSNP11271	*Kabuli*	*Ca_Kabuli_Chr06*	50723980	(A/G)
CakSNP11272	*Kabuli*	*Ca_Kabuli_Chr06*	50723988	(C/A)
CakSNP11273	*Kabuli*	*Ca_Kabuli_Chr06*	51157961	(C/T)
CakSNP11274	*Kabuli*	*Ca_Kabuli_Chr06*	51157939	(A/C)

**SNP IDs**	**Cultivars**	**Chromosomes/scaffolds**	**Physical positions (bp)**	**SNPs**
CakSNP11275	*Kabuli*	*Ca_Kabuli_Chr06*	51162065	(T/C)
CakSNP11276	*Kabuli*	*Ca_Kabuli_Chr06*	51281235	(G/A)
CakSNP11277	*Kabuli*	*Ca_Kabuli_Chr06*	51281178	(C/G)
CakSNP11278	*Kabuli*	*Ca_Kabuli_Chr06*	51345460	(G/C)
CakSNP11279	*Kabuli*	*Ca_Kabuli_Chr06*	51392609	(T/C)
CakSNP11280	*Kabuli*	*Ca_Kabuli_Chr06*	51471573	(T/C)
CakSNP11281	*Kabuli*	*Ca_Kabuli_Chr06*	52007211	(T/C)
CakSNP11282	*Kabuli*	*Ca_Kabuli_Chr06*	52007217	(G/A)
CakSNP11283	*Kabuli*	*Ca_Kabuli_Chr06*	52007471	(C/T)
CakSNP11284	*Kabuli*	*Ca_Kabuli_Chr06*	52007464	(C/G)
CakSNP11285	*Kabuli*	*Ca_Kabuli_Chr06*	52007440	(C/T)
CakSNP11286	*Kabuli*	*Ca_Kabuli_Chr06*	52033279	(G/A)
CakSNP11287	*Kabuli*	*Ca_Kabuli_Chr06*	52033244	(A/T)
CakSNP11288	*Kabuli*	*Ca_Kabuli_Chr06*	52059750	(A/G)
CakSNP11289	*Kabuli*	*Ca_Kabuli_Chr06*	52065393	(C/A)
CakSNP11290	*Kabuli*	*Ca_Kabuli_Chr06*	52065527	(T/A)
CakSNP11291	*Kabuli*	*Ca_Kabuli_Chr06*	52164571	(C/A)
CakSNP11292	*Kabuli*	*Ca_Kabuli_Chr06*	52227277	(T/C)
CakSNP11293	*Kabuli*	*Ca_Kabuli_Chr06*	52304094	(C/T)
CakSNP11294	*Kabuli*	*Ca_Kabuli_Chr06*	52304112	(A/G)
CakSNP11295	*Kabuli*	*Ca_Kabuli_Chr06*	52363121	(C/T)
CakSNP11296	*Kabuli*	*Ca_Kabuli_Chr06*	52416366	(T/A)
CakSNP11297	*Kabuli*	*Ca_Kabuli_Chr06*	52417684	(C/T)
CakSNP11298	*Kabuli*	*Ca_Kabuli_Chr06*	52417717	(T/G)
CakSNP11299	*Kabuli*	*Ca_Kabuli_Chr06*	52417720	(A/T)
CakSNP11300	*Kabuli*	*Ca_Kabuli_Chr06*	52417736	(T/A)
CakSNP11301	*Kabuli*	*Ca_Kabuli_Chr06*	52417747	(G/T)
CakSNP11302	*Kabuli*	*Ca_Kabuli_Chr06*	52567474	(A/C)
CakSNP11303	*Kabuli*	*Ca_Kabuli_Chr06*	52596602	(T/A)
CakSNP11304	*Kabuli*	*Ca_Kabuli_Chr06*	52596601	(A/T)
CakSNP11305	*Kabuli*	*Ca_Kabuli_Chr06*	52858359	(A/G)
CakSNP11306	*Kabuli*	*Ca_Kabuli_Chr06*	52993231	(A/G)
CakSNP11307	*Kabuli*	*Ca_Kabuli_Chr06*	53068176	(C/T)
CakSNP11308	*Kabuli*	*Ca_Kabuli_Chr06*	53068201	(G/T)
CakSNP11309	*Kabuli*	*Ca_Kabuli_Chr06*	53097833	(G/C)
CakSNP11310	*Kabuli*	*Ca_Kabuli_Chr06*	53097803	(A/G)
CakSNP11311	*Kabuli*	*Ca_Kabuli_Chr06*	53097766	(G/A)
CakSNP11312	*Kabuli*	*Ca_Kabuli_Chr06*	53124182	(G/T)
CakSNP11313	*Kabuli*	*Ca_Kabuli_Chr06*	53168715	(T/G)
CakSNP11314	*Kabuli*	*Ca_Kabuli_Chr06*	53348993	(C/A)
CakSNP11315	*Kabuli*	*Ca_Kabuli_Chr06*	53349034	(G/A)

SNP IDs	Cultivars	Chromosomes/scaffolds	Physical positions (bp)	SNPs
CakSNP11316	*Kabuli*	*Ca_Kabuli_Chr06*	53390065	(A/G)
CakSNP11317	*Kabuli*	*Ca_Kabuli_Chr06*	53461929	(C/A)
CakSNP11318	*Kabuli*	*Ca_Kabuli_Chr06*	53461883	(A/G)
CakSNP11319	*Kabuli*	*Ca_Kabuli_Chr06*	53541031	(G/A)
CakSNP11320	*Kabuli*	*Ca_Kabuli_Chr06*	53541094	(C/G)
CakSNP11321	*Kabuli*	*Ca_Kabuli_Chr06*	53541111	(T/G)
CakSNP11322	*Kabuli*	*Ca_Kabuli_Chr06*	53551878	(C/A)
CakSNP11323	*Kabuli*	*Ca_Kabuli_Chr06*	53558430	(A/C)
CakSNP11324	*Kabuli*	*Ca_Kabuli_Chr06*	53566496	(G/A)
CakSNP11325	*Kabuli*	*Ca_Kabuli_Chr06*	53566506	(C/T)
CakSNP11326	*Kabuli*	*Ca_Kabuli_Chr06*	53625264	(A/C)
CakSNP11327	*Kabuli*	*Ca_Kabuli_Chr06*	53683753	(T/C)
CakSNP11328	*Kabuli*	*Ca_Kabuli_Chr06*	53683891	(C/A)
CakSNP11329	*Kabuli*	*Ca_Kabuli_Chr06*	53683885	(C/T)
CakSNP11330	*Kabuli*	*Ca_Kabuli_Chr06*	53683879	(C/T)
CakSNP11331	*Kabuli*	*Ca_Kabuli_Chr06*	53683876	(C/T)
CakSNP11332	*Kabuli*	*Ca_Kabuli_Chr06*	53692784	(G/A)
CakSNP11333	*Kabuli*	*Ca_Kabuli_Chr06*	53769044	(A/C)
CakSNP11334	*Kabuli*	*Ca_Kabuli_Chr06*	53769160	(C/T)
CakSNP11335	*Kabuli*	*Ca_Kabuli_Chr06*	53769240	(T/A)
CakSNP11336	*Kabuli*	*Ca_Kabuli_Chr06*	53769241	(A/T)
CakSNP11337	*Kabuli*	*Ca_Kabuli_Chr06*	54091997	(A/C)
CakSNP11338	*Kabuli*	*Ca_Kabuli_Chr06*	54151902	(T/G)
CakSNP11339	*Kabuli*	*Ca_Kabuli_Chr06*	54151966	(G/C)
CakSNP11340	*Kabuli*	*Ca_Kabuli_Chr06*	54151969	(T/G)
CakSNP11341	*Kabuli*	*Ca_Kabuli_Chr06*	54203356	(G/A)
CakSNP11342	*Kabuli*	*Ca_Kabuli_Chr06*	54203400	(C/T)
CakSNP11343	*Kabuli*	*Ca_Kabuli_Chr06*	54226451	(A/T)
CakSNP11344	*Kabuli*	*Ca_Kabuli_Chr06*	54226432	(A/C)
CakSNP11345	*Kabuli*	*Ca_Kabuli_Chr06*	54229449	(G/T)
CakSNP11346	*Kabuli*	*Ca_Kabuli_Chr06*	54237764	(A/C)
CakSNP11347	*Kabuli*	*Ca_Kabuli_Chr06*	54237815	(C/T)
CakSNP11348	*Kabuli*	*Ca_Kabuli_Chr06*	54250702	(C/T)
CakSNP11349	*Kabuli*	*Ca_Kabuli_Chr06*	54368267	(G/A)
CakSNP11350	*Kabuli*	*Ca_Kabuli_Chr06*	54869636	(C/T)
CakSNP11351	*Kabuli*	*Ca_Kabuli_Chr06*	54869666	(G/A)
CakSNP11352	*Kabuli*	*Ca_Kabuli_Chr06*	54997337	(A/T)
CakSNP11353	*Kabuli*	*Ca_Kabuli_Chr06*	55024071	(C/T)
CakSNP11354	*Kabuli*	*Ca_Kabuli_Chr06*	55024091	(G/A)
CakSNP11355	*Kabuli*	*Ca_Kabuli_Chr06*	55024146	(C/T)
CakSNP11356	*Kabuli*	*Ca_Kabuli_Chr06*	55024140	(G/A)

SNP IDs	Cultivars	Chromosomes/scaffolds	Physical positions (bp)	SNPs
CakSNP11357	*Kabuli*	*Ca_Kabuli_Chr06*	55024130	(T/A)
CakSNP11358	*Kabuli*	*Ca_Kabuli_Chr06*	55024112	(C/G)
CakSNP11359	*Kabuli*	*Ca_Kabuli_Chr06*	55024093	(G/C)
CakSNP11360	*Kabuli*	*Ca_Kabuli_Chr06*	55024108	(C/T)
CakSNP11361	*Kabuli*	*Ca_Kabuli_Chr06*	55024136	(C/T)
CakSNP11362	*Kabuli*	*Ca_Kabuli_Chr06*	55024159	(T/A)
CakSNP11363	*Kabuli*	*Ca_Kabuli_Chr06*	55024198	(T/G)
CakSNP11364	*Kabuli*	*Ca_Kabuli_Chr06*	55028980	(G/A)
CakSNP11365	*Kabuli*	*Ca_Kabuli_Chr06*	55074979	(T/A)
CakSNP11366	*Kabuli*	*Ca_Kabuli_Chr06*	55083311	(T/A)
CakSNP11367	*Kabuli*	*Ca_Kabuli_Chr06*	55083721	(T/A)
CakSNP11368	*Kabuli*	*Ca_Kabuli_Chr06*	55180211	(A/G)
CakSNP11369	*Kabuli*	*Ca_Kabuli_Chr06*	55332515	(G/A)
CakSNP11370	*Kabuli*	*Ca_Kabuli_Chr06*	55914455	(T/C)
CakSNP11371	*Kabuli*	*Ca_Kabuli_Chr06*	55914451	(A/G)
CakSNP11372	*Kabuli*	*Ca_Kabuli_Chr06*	55914443	(T/C)
CakSNP11373	*Kabuli*	*Ca_Kabuli_Chr06*	55914434	(C/T)
CakSNP11374	*Kabuli*	*Ca_Kabuli_Chr06*	55914431	(G/A)
CakSNP11375	*Kabuli*	*Ca_Kabuli_Chr06*	55922843	(T/A)
CakSNP11376	*Kabuli*	*Ca_Kabuli_Chr06*	55922850	(C/T)
CakSNP11377	*Kabuli*	*Ca_Kabuli_Chr06*	55942105	(A/G)
CakSNP11378	*Kabuli*	*Ca_Kabuli_Chr06*	55953320	(T/A)
CakSNP11379	*Kabuli*	*Ca_Kabuli_Chr06*	55953327	(C/T)
CakSNP11380	*Kabuli*	*Ca_Kabuli_Chr06*	56025902	(A/G)
CakSNP11381	*Kabuli*	*Ca_Kabuli_Chr06*	56025888	(G/A)
CakSNP11382	*Kabuli*	*Ca_Kabuli_Chr06*	56025884	(A/G)
CakSNP11383	*Kabuli*	*Ca_Kabuli_Chr06*	56029491	(T/A)
CakSNP11384	*Kabuli*	*Ca_Kabuli_Chr06*	56029544	(A/T)
CakSNP11385	*Kabuli*	*Ca_Kabuli_Chr06*	56029549	(C/T)
CakSNP11386	*Kabuli*	*Ca_Kabuli_Chr06*	56137878	(T/C)
CakSNP11387	*Kabuli*	*Ca_Kabuli_Chr06*	56553930	(G/A)
CakSNP11388	*Kabuli*	*Ca_Kabuli_Chr06*	56564249	(G/C)
CakSNP11389	*Kabuli*	*Ca_Kabuli_Chr06*	56587094	(C/G)
CakSNP11390	*Kabuli*	*Ca_Kabuli_Chr06*	56593148	(A/G)
CakSNP11391	*Kabuli*	*Ca_Kabuli_Chr06*	56593121	(T/G)
CakSNP11392	*Kabuli*	*Ca_Kabuli_Chr06*	56624316	(A/T)
CakSNP11393	*Kabuli*	*Ca_Kabuli_Chr06*	56624674	(T/C)
CakSNP11394	*Kabuli*	*Ca_Kabuli_Chr06*	56624718	(C/A)
CakSNP11395	*Kabuli*	*Ca_Kabuli_Chr06*	56694898	(T/C)
CakSNP11396	*Kabuli*	*Ca_Kabuli_Chr06*	56765524	(C/A)
CakSNP11397	*Kabuli*	*Ca_Kabuli_Chr06*	56793077	(G/A)

SNP IDs	Cultivars	Chromosomes/scaffolds	Physical positions (bp)	SNPs
CakSNP11398	*Kabuli*	*Ca_Kabuli_Chr06*	56793037	(A/T)
CakSNP11399	*Kabuli*	*Ca_Kabuli_Chr06*	56793021	(G/T)
CakSNP11400	*Kabuli*	*Ca_Kabuli_Chr06*	56793122	(C/T)
CakSNP11401	*Kabuli*	*Ca_Kabuli_Chr06*	56822084	(G/T)
CakSNP11402	*Kabuli*	*Ca_Kabuli_Chr06*	56822060	(G/A)
CakSNP11403	*Kabuli*	*Ca_Kabuli_Chr06*	56840030	(A/C)
CakSNP11404	*Kabuli*	*Ca_Kabuli_Chr06*	56840084	(A/T)
CakSNP11405	*Kabuli*	*Ca_Kabuli_Chr06*	56840063	(T/A)
CakSNP11406	*Kabuli*	*Ca_Kabuli_Chr06*	56840038	(A/T)
CakSNP11407	*Kabuli*	*Ca_Kabuli_Chr06*	56854753	(T/C)
CakSNP11408	*Kabuli*	*Ca_Kabuli_Chr06*	56946415	(A/G)
CakSNP11409	*Kabuli*	*Ca_Kabuli_Chr06*	56946431	(T/C)
CakSNP11410	*Kabuli*	*Ca_Kabuli_Chr06*	56950487	(G/T)
CakSNP11411	*Kabuli*	*Ca_Kabuli_Chr06*	56980313	(T/C)
CakSNP11412	*Kabuli*	*Ca_Kabuli_Chr06*	56980286	(T/C)
CakSNP11413	*Kabuli*	*Ca_Kabuli_Chr06*	56980321	(T/C)
CakSNP11414	*Kabuli*	*Ca_Kabuli_Chr06*	57147008	(A/G)
CakSNP11415	*Kabuli*	*Ca_Kabuli_Chr06*	57198837	(G/A)
CakSNP11416	*Kabuli*	*Ca_Kabuli_Chr06*	57259982	(C/A)
CakSNP11417	*Kabuli*	*Ca_Kabuli_Chr06*	57260020	(A/G)
CakSNP11418	*Kabuli*	*Ca_Kabuli_Chr06*	57260022	(T/C)
CakSNP11419	*Kabuli*	*Ca_Kabuli_Chr06*	57272604	(A/G)
CakSNP11420	*Kabuli*	*Ca_Kabuli_Chr06*	57272607	(G/A)
CakSNP11421	*Kabuli*	*Ca_Kabuli_Chr06*	57272608	(C/A)
CakSNP11422	*Kabuli*	*Ca_Kabuli_Chr06*	57277430	(A/G)
CakSNP11423	*Kabuli*	*Ca_Kabuli_Chr06*	57277440	(C/T)
CakSNP11424	*Kabuli*	*Ca_Kabuli_Chr06*	57344963	(G/T)
CakSNP11425	*Kabuli*	*Ca_Kabuli_Chr06*	57382220	(G/A)
CakSNP11426	*Kabuli*	*Ca_Kabuli_Chr06*	57433674	(T/A)
CakSNP11427	*Kabuli*	*Ca_Kabuli_Chr06*	57436112	(A/T)
CakSNP11428	*Kabuli*	*Ca_Kabuli_Chr06*	57436119	(T/C)
CakSNP11429	*Kabuli*	*Ca_Kabuli_Chr06*	57436149	(T/A)
CakSNP11430	*Kabuli*	*Ca_Kabuli_Chr06*	57436320	(T/G)
CakSNP11431	*Kabuli*	*Ca_Kabuli_Chr06*	57437904	(A/G)
CakSNP11432	*Kabuli*	*Ca_Kabuli_Chr06*	57449892	(A/G)
CakSNP11433	*Kabuli*	*Ca_Kabuli_Chr06*	57475853	(G/T)
CakSNP11434	*Kabuli*	*Ca_Kabuli_Chr06*	57475892	(A/G)
CakSNP11435	*Kabuli*	*Ca_Kabuli_Chr06*	57475982	(A/G)
CakSNP11436	*Kabuli*	*Ca_Kabuli_Chr06*	57533087	(G/A)
CakSNP11437	*Kabuli*	*Ca_Kabuli_Chr06*	57579770	(G/T)
CakSNP11438	*Kabuli*	*Ca_Kabuli_Chr06*	57579768	(C/T)

SNP IDs	Cultivars	Chromosomes/scaffolds	Physical positions (bp)	SNPs
CakSNP11439	*Kabuli*	*Ca_Kabuli_Chr06*	57580569	(C/T)
CakSNP11440	*Kabuli*	*Ca_Kabuli_Chr06*	57603257	(T/A)
CakSNP11441	*Kabuli*	*Ca_Kabuli_Chr06*	57625134	(G/A)
CakSNP11442	*Kabuli*	*Ca_Kabuli_Chr06*	57625320	(G/A)
CakSNP11443	*Kabuli*	*Ca_Kabuli_Chr06*	57632486	(C/T)
CakSNP11444	*Kabuli*	*Ca_Kabuli_Chr06*	57634387	(G/T)
CakSNP11445	*Kabuli*	*Ca_Kabuli_Chr06*	57634677	(G/A)
CakSNP11446	*Kabuli*	*Ca_Kabuli_Chr06*	57637091	(G/A)
CakSNP11447	*Kabuli*	*Ca_Kabuli_Chr06*	57637083	(T/G)
CakSNP11448	*Kabuli*	*Ca_Kabuli_Chr06*	57637075	(C/T)
CakSNP11449	*Kabuli*	*Ca_Kabuli_Chr06*	57662858	(T/A)
CakSNP11450	*Kabuli*	*Ca_Kabuli_Chr06*	57667515	(A/T)
CakSNP11451	*Kabuli*	*Ca_Kabuli_Chr06*	57688937	(C/T)
CakSNP11452	*Kabuli*	*Ca_Kabuli_Chr06*	57704057	(C/T)
CakSNP11453	*Kabuli*	*Ca_Kabuli_Chr06*	57704802	(T/C)
CakSNP11454	*Kabuli*	*Ca_Kabuli_Chr06*	57705011	(G/A)
CakSNP11455	*Kabuli*	*Ca_Kabuli_Chr06*	57707716	(C/T)
CakSNP11456	*Kabuli*	*Ca_Kabuli_Chr06*	57707724	(A/C)
CakSNP11457	*Kabuli*	*Ca_Kabuli_Chr06*	57707727	(T/G)
CakSNP11458	*Kabuli*	*Ca_Kabuli_Chr06*	57707848	(A/G)
CakSNP11459	*Kabuli*	*Ca_Kabuli_Chr06*	57707846	(G/A)
CakSNP11460	*Kabuli*	*Ca_Kabuli_Chr06*	57707912	(G/C)
CakSNP11461	*Kabuli*	*Ca_Kabuli_Chr06*	57708019	(A/G)
CakSNP11462	*Kabuli*	*Ca_Kabuli_Chr06*	57708130	(T/C)
CakSNP11463	*Kabuli*	*Ca_Kabuli_Chr06*	57708101	(T/G)
CakSNP11464	*Kabuli*	*Ca_Kabuli_Chr06*	57708198	(C/T)
CakSNP11465	*Kabuli*	*Ca_Kabuli_Chr06*	57710133	(C/G)
CakSNP11466	*Kabuli*	*Ca_Kabuli_Chr06*	57720446	(T/C)
CakSNP11467	*Kabuli*	*Ca_Kabuli_Chr06*	57723992	(A/C)
CakSNP11468	*Kabuli*	*Ca_Kabuli_Chr06*	57725372	(A/C)
CakSNP11469	*Kabuli*	*Ca_Kabuli_Chr06*	57746989	(C/T)
CakSNP11470	*Kabuli*	*Ca_Kabuli_Chr06*	57747101	(C/T)
CakSNP11471	*Kabuli*	*Ca_Kabuli_Chr06*	57748565	(A/G)
CakSNP11472	*Kabuli*	*Ca_Kabuli_Chr06*	57748602	(A/T)
CakSNP11473	*Kabuli*	*Ca_Kabuli_Chr06*	57753076	(T/C)
CakSNP11474	*Kabuli*	*Ca_Kabuli_Chr06*	57756699	(G/A)
CakSNP11475	*Kabuli*	*Ca_Kabuli_Chr06*	57760109	(T/G)
CakSNP11476	*Kabuli*	*Ca_Kabuli_Chr06*	57760176	(C/T)
CakSNP11477	*Kabuli*	*Ca_Kabuli_Chr06*	57760227	(C/T)
CakSNP11478	*Kabuli*	*Ca_Kabuli_Chr06*	57760290	(A/T)
CakSNP11479	*Kabuli*	*Ca_Kabuli_Chr06*	57760291	(G/T)

SNP IDs	Cultivars	Chromosomes/scaffolds	Physical positions (bp)	SNPs
CakSNP11480	*Kabuli*	*Ca_Kabuli_Chr06*	57760360	(A/G)
CakSNP11481	*Kabuli*	*Ca_Kabuli_Chr06*	57761758	(G/A)
CakSNP11482	*Kabuli*	*Ca_Kabuli_Chr06*	57761722	(T/C)
CakSNP11483	*Kabuli*	*Ca_Kabuli_Chr06*	57766444	(C/T)
CakSNP11484	*Kabuli*	*Ca_Kabuli_Chr06*	57766484	(T/A)
CakSNP11485	*Kabuli*	*Ca_Kabuli_Chr06*	57766483	(C/A)
CakSNP11486	*Kabuli*	*Ca_Kabuli_Chr06*	57766570	(C/T)
CakSNP11487	*Kabuli*	*Ca_Kabuli_Chr06*	57766588	(G/A)
CakSNP11488	*Kabuli*	*Ca_Kabuli_Chr06*	57766598	(G/A)
CakSNP11489	*Kabuli*	*Ca_Kabuli_Chr06*	57768314	(T/C)
CakSNP11490	*Kabuli*	*Ca_Kabuli_Chr06*	57801985	(A/C)
CakSNP11491	*Kabuli*	*Ca_Kabuli_Chr06*	57831050	(G/T)
CakSNP11492	*Kabuli*	*Ca_Kabuli_Chr06*	57831049	(C/T)
CakSNP11493	*Kabuli*	*Ca_Kabuli_Chr06*	57831040	(T/C)
CakSNP11494	*Kabuli*	*Ca_Kabuli_Chr06*	57835093	(T/A)
CakSNP11495	*Kabuli*	*Ca_Kabuli_Chr06*	57835120	(G/A)
CakSNP11496	*Kabuli*	*Ca_Kabuli_Chr06*	57835917	(G/C)
CakSNP11497	*Kabuli*	*Ca_Kabuli_Chr06*	57835899	(C/T)
CakSNP11498	*Kabuli*	*Ca_Kabuli_Chr06*	57836024	(G/A)
CakSNP11499	*Kabuli*	*Ca_Kabuli_Chr06*	57836237	(A/G)
CakSNP11500	*Kabuli*	*Ca_Kabuli_Chr06*	57836284	(G/A)
CakSNP11501	*Kabuli*	*Ca_Kabuli_Chr06*	57836314	(T/A)
CakSNP11502	*Kabuli*	*Ca_Kabuli_Chr06*	57836316	(G/A)
CakSNP11503	*Kabuli*	*Ca_Kabuli_Chr06*	57870893	(C/A)
CakSNP11504	*Kabuli*	*Ca_Kabuli_Chr06*	57877446	(C/A)
CakSNP11505	*Kabuli*	*Ca_Kabuli_Chr06*	57882364	(C/T)
CakSNP11506	*Kabuli*	*Ca_Kabuli_Chr06*	57894377	(A/G)
CakSNP11507	*Kabuli*	*Ca_Kabuli_Chr06*	57894495	(C/G)
CakSNP11508	*Kabuli*	*Ca_Kabuli_Chr06*	57894590	(G/C)
CakSNP11509	*Kabuli*	*Ca_Kabuli_Chr06*	57894585	(T/G)
CakSNP11510	*Kabuli*	*Ca_Kabuli_Chr06*	57904599	(T/C)
CakSNP11511	*Kabuli*	*Ca_Kabuli_Chr06*	57921373	(C/T)
CakSNP11512	*Kabuli*	*Ca_Kabuli_Chr06*	57943585	(A/T)
CakSNP11513	*Kabuli*	*Ca_Kabuli_Chr06*	57943626	(G/A)
CakSNP11514	*Kabuli*	*Ca_Kabuli_Chr06*	57943628	(A/C)
CakSNP11515	*Kabuli*	*Ca_Kabuli_Chr06*	57944439	(C/T)
CakSNP11516	*Kabuli*	*Ca_Kabuli_Chr06*	57968924	(A/T)
CakSNP11517	*Kabuli*	*Ca_Kabuli_Chr06*	58036471	(C/T)
CakSNP11518	*Kabuli*	*Ca_Kabuli_Chr06*	58174702	(T/C)
CakSNP11519	*Kabuli*	*Ca_Kabuli_Chr06*	58187822	(T/G)
CakSNP11520	*Kabuli*	*Ca_Kabuli_Chr06*	58187859	(T/A)

**SNP IDs**	**Cultivars**	**Chromosomes/scaffolds**	**Physical positions (bp)**	**SNPs**
CakSNP11521	*Kabuli*	*Ca_Kabuli_Chr06*	58187957	(A/G)
CakSNP11522	*Kabuli*	*Ca_Kabuli_Chr06*	58191050	(T/C)
CakSNP11523	*Kabuli*	*Ca_Kabuli_Chr06*	58224023	(G/T)
CakSNP11524	*Kabuli*	*Ca_Kabuli_Chr06*	58226908	(T/G)
CakSNP11525	*Kabuli*	*Ca_Kabuli_Chr06*	58227006	(T/C)
CakSNP11526	*Kabuli*	*Ca_Kabuli_Chr06*	58237503	(A/T)
CakSNP11527	*Kabuli*	*Ca_Kabuli_Chr06*	58238371	(T/C)
CakSNP11528	*Kabuli*	*Ca_Kabuli_Chr06*	58238397	(A/G)
CakSNP11529	*Kabuli*	*Ca_Kabuli_Chr06*	58243505	(G/A)
CakSNP11530	*Kabuli*	*Ca_Kabuli_Chr06*	58326344	(A/T)
CakSNP11531	*Kabuli*	*Ca_Kabuli_Chr06*	58380367	(T/C)
CakSNP11532	*Kabuli*	*Ca_Kabuli_Chr06*	58380394	(G/A)
CakSNP11533	*Kabuli*	*Ca_Kabuli_Chr06*	58380563	(G/A)
CakSNP11534	*Kabuli*	*Ca_Kabuli_Chr06*	58381203	(G/C)
CakSNP11535	*Kabuli*	*Ca_Kabuli_Chr06*	58381259	(A/G)
CakSNP11536	*Kabuli*	*Ca_Kabuli_Chr06*	58434217	(C/G)
CakSNP11537	*Kabuli*	*Ca_Kabuli_Chr06*	58452843	(A/C)
CakSNP11538	*Kabuli*	*Ca_Kabuli_Chr06*	58569576	(G/T)
CakSNP11539	*Kabuli*	*Ca_Kabuli_Chr06*	58569569	(T/C)
CakSNP11540	*Kabuli*	*Ca_Kabuli_Chr06*	58571660	(T/C)
CakSNP11541	*Kabuli*	*Ca_Kabuli_Chr06*	58596821	(T/G)
CakSNP11542	*Kabuli*	*Ca_Kabuli_Chr06*	58596878	(C/A)
CakSNP11543	*Kabuli*	*Ca_Kabuli_Chr06*	58596968	(T/C)
CakSNP11544	*Kabuli*	*Ca_Kabuli_Chr06*	58596918	(A/G)
CakSNP11545	*Kabuli*	*Ca_Kabuli_Chr06*	58600930	(C/A)
CakSNP11546	*Kabuli*	*Ca_Kabuli_Chr06*	58600952	(A/G)
CakSNP11547	*Kabuli*	*Ca_Kabuli_Chr06*	58600974	(A/G)
CakSNP11548	*Kabuli*	*Ca_Kabuli_Chr06*	58623095	(A/G)
CakSNP11549	*Kabuli*	*Ca_Kabuli_Chr06*	58623096	(A/T)
CakSNP11550	*Kabuli*	*Ca_Kabuli_Chr06*	58623220	(T/C)
CakSNP11551	*Kabuli*	*Ca_Kabuli_Chr06*	58624183	(G/A)
CakSNP11552	*Kabuli*	*Ca_Kabuli_Chr06*	58624233	(C/A)
CakSNP11553	*Kabuli*	*Ca_Kabuli_Chr06*	58624299	(A/G)
CakSNP11554	*Kabuli*	*Ca_Kabuli_Chr06*	58624281	(A/G)
CakSNP11555	*Kabuli*	*Ca_Kabuli_Chr06*	58624251	(C/T)
CakSNP11556	*Kabuli*	*Ca_Kabuli_Chr06*	58650354	(A/G)
CakSNP11557	*Kabuli*	*Ca_Kabuli_Chr06*	58650330	(G/A)
CakSNP11558	*Kabuli*	*Ca_Kabuli_Chr06*	58650318	(A/C)
CakSNP11559	*Kabuli*	*Ca_Kabuli_Chr06*	58650459	(A/T)
CakSNP11560	*Kabuli*	*Ca_Kabuli_Chr06*	58650491	(A/G)
CakSNP11561	*Kabuli*	*Ca_Kabuli_Chr06*	58652881	(A/G)

SNP IDs	Cultivars	Chromosomes/scaffolds	Physical positions (bp)	SNPs
CakSNP11562	*Kabuli*	*Ca_Kabuli_Chr06*	58672317	(A/G)
CakSNP11563	*Kabuli*	*Ca_Kabuli_Chr06*	58675788	(T/C)
CakSNP11564	*Kabuli*	*Ca_Kabuli_Chr06*	58675749	(T/C)
CakSNP11565	*Kabuli*	*Ca_Kabuli_Chr06*	58676507	(A/G)
CakSNP11566	*Kabuli*	*Ca_Kabuli_Chr06*	58676517	(A/C)
CakSNP11567	*Kabuli*	*Ca_Kabuli_Chr06*	58682427	(G/T)
CakSNP11568	*Kabuli*	*Ca_Kabuli_Chr06*	58682397	(G/A)
CakSNP11569	*Kabuli*	*Ca_Kabuli_Chr06*	58682387	(T/A)
CakSNP11570	*Kabuli*	*Ca_Kabuli_Chr06*	58730925	(T/C)
CakSNP11571	*Kabuli*	*Ca_Kabuli_Chr06*	58730969	(C/A)
CakSNP11572	*Kabuli*	*Ca_Kabuli_Chr06*	58730967	(T/G)
CakSNP11573	*Kabuli*	*Ca_Kabuli_Chr06*	58732424	(G/T)
CakSNP11574	*Kabuli*	*Ca_Kabuli_Chr06*	58732423	(C/T)
CakSNP11575	*Kabuli*	*Ca_Kabuli_Chr06*	58732514	(G/A)
CakSNP11576	*Kabuli*	*Ca_Kabuli_Chr06*	58732451	(T/C)
CakSNP11577	*Kabuli*	*Ca_Kabuli_Chr06*	58732475	(G/A)
CakSNP11578	*Kabuli*	*Ca_Kabuli_Chr06*	58745356	(A/G)
CakSNP11579	*Kabuli*	*Ca_Kabuli_Chr06*	58745473	(A/G)
CakSNP11580	*Kabuli*	*Ca_Kabuli_Chr06*	58745426	(T/C)
CakSNP11581	*Kabuli*	*Ca_Kabuli_Chr06*	58745583	(C/T)
CakSNP11582	*Kabuli*	*Ca_Kabuli_Chr06*	58761879	(G/A)
CakSNP11583	*Kabuli*	*Ca_Kabuli_Chr06*	58779344	(A/G)
CakSNP11584	*Kabuli*	*Ca_Kabuli_Chr06*	58779389	(T/G)
CakSNP11585	*Kabuli*	*Ca_Kabuli_Chr06*	58820632	(A/C)
CakSNP11586	*Kabuli*	*Ca_Kabuli_Chr06*	58844717	(T/C)
CakSNP11587	*Kabuli*	*Ca_Kabuli_Chr06*	58844744	(A/T)
CakSNP11588	*Kabuli*	*Ca_Kabuli_Chr06*	58844776	(C/G)
CakSNP11589	*Kabuli*	*Ca_Kabuli_Chr06*	58861614	(G/A)
CakSNP11590	*Kabuli*	*Ca_Kabuli_Chr06*	58878604	(C/G)
CakSNP11591	*Kabuli*	*Ca_Kabuli_Chr06*	58879570	(T/C)
CakSNP11592	*Kabuli*	*Ca_Kabuli_Chr06*	58903346	(G/A)
CakSNP11593	*Kabuli*	*Ca_Kabuli_Chr06*	58903381	(G/A)
CakSNP11594	*Kabuli*	*Ca_Kabuli_Chr06*	58903395	(A/G)
CakSNP11595	*Kabuli*	*Ca_Kabuli_Chr06*	58903416	(T/G)
CakSNP11596	*Kabuli*	*Ca_Kabuli_Chr06*	58903497	(G/C)
CakSNP11597	*Kabuli*	*Ca_Kabuli_Chr06*	58903538	(G/A)
CakSNP11598	*Kabuli*	*Ca_Kabuli_Chr06*	58908024	(G/A)
CakSNP11599	*Kabuli*	*Ca_Kabuli_Chr06*	58908045	(A/G)
CakSNP11600	*Kabuli*	*Ca_Kabuli_Chr06*	58908069	(G/A)
CakSNP11601	*Kabuli*	*Ca_Kabuli_Chr06*	58911548	(T/C)
CakSNP11602	*Kabuli*	*Ca_Kabuli_Chr06*	58951079	(T/C)

**SNP IDs**	**Cultivars**	**Chromosomes/scaffolds**	**Physical positions (bp)**	**SNPs**
CakSNP11603	*Kabuli*	*Ca_Kabuli_Chr06*	58951298	(A/G)
CakSNP11604	*Kabuli*	*Ca_Kabuli_Chr06*	58951583	(C/A)
CakSNP11605	*Kabuli*	*Ca_Kabuli_Chr06*	58953219	(A/G)
CakSNP11606	*Kabuli*	*Ca_Kabuli_Chr06*	58994014	(C/T)
CakSNP11607	*Kabuli*	*Ca_Kabuli_Chr06*	58994083	(A/G)
CakSNP11608	*Kabuli*	*Ca_Kabuli_Chr06*	58994281	(G/A)
CakSNP11609	*Kabuli*	*Ca_Kabuli_Chr06*	59001443	(C/T)
CakSNP11610	*Kabuli*	*Ca_Kabuli_Chr06*	59003792	(A/C)
CakSNP11611	*Kabuli*	*Ca_Kabuli_Chr06*	59064798	(G/T)
CakSNP11612	*Kabuli*	*Ca_Kabuli_Chr06*	59064815	(C/T)
CakSNP11613	*Kabuli*	*Ca_Kabuli_Chr06*	59081169	(C/T)
CakSNP11614	*Kabuli*	*Ca_Kabuli_Chr06*	59107027	(A/T)
CakSNP11615	*Kabuli*	*Ca_Kabuli_Chr06*	59107007	(G/T)
CakSNP11616	*Kabuli*	*Ca_Kabuli_Chr06*	59135944	(A/C)
CakSNP11617	*Kabuli*	*Ca_Kabuli_Chr06*	59194015	(C/G)
CakSNP11618	*Kabuli*	*Ca_Kabuli_Chr06*	59193994	(G/T)
CakSNP11619	*Kabuli*	*Ca_Kabuli_Chr06*	59194081	(T/A)
CakSNP11620	*Kabuli*	*Ca_Kabuli_Chr06*	59202825	(T/G)
CakSNP11621	*Kabuli*	*Ca_Kabuli_Chr06*	59219099	(T/C)
CakSNP11622	*Kabuli*	*Ca_Kabuli_Chr06*	59222848	(C/T)
CakSNP11623	*Kabuli*	*Ca_Kabuli_Chr06*	59296368	(C/T)
CakSNP11624	*Kabuli*	*Ca_Kabuli_Chr06*	59296460	(G/A)
CakSNP11625	*Kabuli*	*Ca_Kabuli_Chr06*	59407761	(G/A)
CakSNP11626	*Kabuli*	*Ca_Kabuli_Chr06*	59417984	(G/A)
CakSNP11627	*Kabuli*	*Ca_Kabuli_Chr07*	16760	(T/G)
CakSNP11628	*Kabuli*	*Ca_Kabuli_Chr07*	16850	(C/T)
CakSNP11629	*Kabuli*	*Ca_Kabuli_Chr07*	16880	(G/A)
CakSNP11630	*Kabuli*	*Ca_Kabuli_Chr07*	16881	(A/G)
CakSNP11631	*Kabuli*	*Ca_Kabuli_Chr07*	41841	(G/A)
CakSNP11632	*Kabuli*	*Ca_Kabuli_Chr07*	55131	(T/C)
CakSNP11633	*Kabuli*	*Ca_Kabuli_Chr07*	208824	(A/G)
CakSNP11634	*Kabuli*	*Ca_Kabuli_Chr07*	218346	(C/A)
CakSNP11635	*Kabuli*	*Ca_Kabuli_Chr07*	218341	(C/G)
CakSNP11636	*Kabuli*	*Ca_Kabuli_Chr07*	419301	(C/T)
CakSNP11637	*Kabuli*	*Ca_Kabuli_Chr07*	419316	(A/G)
CakSNP11638	*Kabuli*	*Ca_Kabuli_Chr07*	419334	(A/C)
CakSNP11639	*Kabuli*	*Ca_Kabuli_Chr07*	419385	(T/G)
CakSNP11640	*Kabuli*	*Ca_Kabuli_Chr07*	419328	(C/T)
CakSNP11641	*Kabuli*	*Ca_Kabuli_Chr07*	419679	(C/T)
CakSNP11642	*Kabuli*	*Ca_Kabuli_Chr07*	419638	(C/T)
CakSNP11643	*Kabuli*	*Ca_Kabuli_Chr07*	419628	(A/C)

**SNP IDs**	**Cultivars**	**Chromosomes/scaffolds**	**Physical positions (bp)**	**SNPs**
CakSNP11644	*Kabuli*	*Ca_Kabuli_Chr07*	528521	(G/A)
CakSNP11645	*Kabuli*	*Ca_Kabuli_Chr07*	601563	(T/A)
CakSNP11646	*Kabuli*	*Ca_Kabuli_Chr07*	601551	(T/C)
CakSNP11647	*Kabuli*	*Ca_Kabuli_Chr07*	601545	(C/T)
CakSNP11648	*Kabuli*	*Ca_Kabuli_Chr07*	601538	(C/A)
CakSNP11649	*Kabuli*	*Ca_Kabuli_Chr07*	601521	(G/A)
CakSNP11650	*Kabuli*	*Ca_Kabuli_Chr07*	601516	(G/A)
CakSNP11651	*Kabuli*	*Ca_Kabuli_Chr07*	601513	(C/G)
CakSNP11652	*Kabuli*	*Ca_Kabuli_Chr07*	601502	(G/T)
CakSNP11653	*Kabuli*	*Ca_Kabuli_Chr07*	601496	(C/T)
CakSNP11654	*Kabuli*	*Ca_Kabuli_Chr07*	601586	(C/T)
CakSNP11655	*Kabuli*	*Ca_Kabuli_Chr07*	601601	(C/T)
CakSNP11656	*Kabuli*	*Ca_Kabuli_Chr07*	601612	(A/G)
CakSNP11657	*Kabuli*	*Ca_Kabuli_Chr07*	601643	(G/T)
CakSNP11658	*Kabuli*	*Ca_Kabuli_Chr07*	601622	(G/C)
CakSNP11659	*Kabuli*	*Ca_Kabuli_Chr07*	601657	(C/T)
CakSNP11660	*Kabuli*	*Ca_Kabuli_Chr07*	601719	(G/T)
CakSNP11661	*Kabuli*	*Ca_Kabuli_Chr07*	601718	(G/C)
CakSNP11662	*Kabuli*	*Ca_Kabuli_Chr07*	601714	(A/T)
CakSNP11663	*Kabuli*	*Ca_Kabuli_Chr07*	601709	(A/C)
CakSNP11664	*Kabuli*	*Ca_Kabuli_Chr07*	601683	(G/A)
CakSNP11665	*Kabuli*	*Ca_Kabuli_Chr07*	601647	(C/T)
CakSNP11666	*Kabuli*	*Ca_Kabuli_Chr07*	610737	(C/A)
CakSNP11667	*Kabuli*	*Ca_Kabuli_Chr07*	610723	(G/T)
CakSNP11668	*Kabuli*	*Ca_Kabuli_Chr07*	667597	(C/T)
CakSNP11669	*Kabuli*	*Ca_Kabuli_Chr07*	674791	(A/T)
CakSNP11670	*Kabuli*	*Ca_Kabuli_Chr07*	676785	(G/T)
CakSNP11671	*Kabuli*	*Ca_Kabuli_Chr07*	676786	(G/A)
CakSNP11672	*Kabuli*	*Ca_Kabuli_Chr07*	676790	(T/C)
CakSNP11673	*Kabuli*	*Ca_Kabuli_Chr07*	683245	(T/C)
CakSNP11674	*Kabuli*	*Ca_Kabuli_Chr07*	761710	(A/G)
CakSNP11675	*Kabuli*	*Ca_Kabuli_Chr07*	767708	(G/A)
CakSNP11676	*Kabuli*	*Ca_Kabuli_Chr07*	781377	(G/T)
CakSNP11677	*Kabuli*	*Ca_Kabuli_Chr07*	781409	(A/G)
CakSNP11678	*Kabuli*	*Ca_Kabuli_Chr07*	807095	(A/T)
CakSNP11679	*Kabuli*	*Ca_Kabuli_Chr07*	807110	(T/C)
CakSNP11680	*Kabuli*	*Ca_Kabuli_Chr07*	807133	(T/A)
CakSNP11681	*Kabuli*	*Ca_Kabuli_Chr07*	815477	(A/T)
CakSNP11682	*Kabuli*	*Ca_Kabuli_Chr07*	815526	(C/A)
CakSNP11683	*Kabuli*	*Ca_Kabuli_Chr07*	838581	(C/A)
CakSNP11684	*Kabuli*	*Ca_Kabuli_Chr07*	838651	(A/C)

SNP IDs	Cultivars	Chromosomes/scaffolds	Physical positions (bp)	SNPs
CakSNP11685	*Kabuli*	*Ca_Kabuli_Chr07*	841988	(T/C)
CakSNP11686	*Kabuli*	*Ca_Kabuli_Chr07*	844248	(G/A)
CakSNP11687	*Kabuli*	*Ca_Kabuli_Chr07*	845488	(A/G)
CakSNP11688	*Kabuli*	*Ca_Kabuli_Chr07*	865007	(C/T)
CakSNP11689	*Kabuli*	*Ca_Kabuli_Chr07*	865170	(C/T)
CakSNP11690	*Kabuli*	*Ca_Kabuli_Chr07*	873207	(A/G)
CakSNP11691	*Kabuli*	*Ca_Kabuli_Chr07*	888900	(G/A)
CakSNP11692	*Kabuli*	*Ca_Kabuli_Chr07*	888987	(A/G)
CakSNP11693	*Kabuli*	*Ca_Kabuli_Chr07*	975236	(T/A)
CakSNP11694	*Kabuli*	*Ca_Kabuli_Chr07*	993031	(G/T)
CakSNP11695	*Kabuli*	*Ca_Kabuli_Chr07*	994976	(G/A)
CakSNP11696	*Kabuli*	*Ca_Kabuli_Chr07*	995691	(C/T)
CakSNP11697	*Kabuli*	*Ca_Kabuli_Chr07*	995674	(T/C)
CakSNP11698	*Kabuli*	*Ca_Kabuli_Chr07*	1022946	(C/T)
CakSNP11699	*Kabuli*	*Ca_Kabuli_Chr07*	1045113	(A/C)
CakSNP11700	*Kabuli*	*Ca_Kabuli_Chr07*	1045127	(T/A)
CakSNP11701	*Kabuli*	*Ca_Kabuli_Chr07*	1096379	(C/T)
CakSNP11702	*Kabuli*	*Ca_Kabuli_Chr07*	1153267	(A/C)
CakSNP11703	*Kabuli*	*Ca_Kabuli_Chr07*	1174188	(C/T)
CakSNP11704	*Kabuli*	*Ca_Kabuli_Chr07*	1293601	(C/G)
CakSNP11705	*Kabuli*	*Ca_Kabuli_Chr07*	1350605	(C/T)
CakSNP11706	*Kabuli*	*Ca_Kabuli_Chr07*	1350856	(G/T)
CakSNP11707	*Kabuli*	*Ca_Kabuli_Chr07*	1350846	(A/T)
CakSNP11708	*Kabuli*	*Ca_Kabuli_Chr07*	1350947	(T/G)
CakSNP11709	*Kabuli*	*Ca_Kabuli_Chr07*	1414206	(C/A)
CakSNP11710	*Kabuli*	*Ca_Kabuli_Chr07*	1505646	(G/A)
CakSNP11711	*Kabuli*	*Ca_Kabuli_Chr07*	1509156	(G/A)
CakSNP11712	*Kabuli*	*Ca_Kabuli_Chr07*	1511252	(C/T)
CakSNP11713	*Kabuli*	*Ca_Kabuli_Chr07*	1585622	(T/C)
CakSNP11714	*Kabuli*	*Ca_Kabuli_Chr07*	1635519	(G/A)
CakSNP11715	*Kabuli*	*Ca_Kabuli_Chr07*	1635471	(G/T)
CakSNP11716	*Kabuli*	*Ca_Kabuli_Chr07*	1702033	(G/T)
CakSNP11717	*Kabuli*	*Ca_Kabuli_Chr07*	1702063	(A/G)
CakSNP11718	*Kabuli*	*Ca_Kabuli_Chr07*	1702077	(G/C)
CakSNP11719	*Kabuli*	*Ca_Kabuli_Chr07*	1704780	(T/G)
CakSNP11720	*Kabuli*	*Ca_Kabuli_Chr07*	1704802	(A/G)
CakSNP11721	*Kabuli*	*Ca_Kabuli_Chr07*	1704892	(A/G)
CakSNP11722	*Kabuli*	*Ca_Kabuli_Chr07*	1704947	(C/T)
CakSNP11723	*Kabuli*	*Ca_Kabuli_Chr07*	1705197	(A/C)
CakSNP11724	*Kabuli*	*Ca_Kabuli_Chr07*	1707283	(G/T)
CakSNP11725	*Kabuli*	*Ca_Kabuli_Chr07*	1707375	(C/G)

SNP IDs	Cultivars	Chromosomes/scaffolds	Physical positions (bp)	SNPs
CakSNP11726	*Kabuli*	*Ca_Kabuli_Chr07*	1739369	(C/A)
CakSNP11727	*Kabuli*	*Ca_Kabuli_Chr07*	1787308	(T/A)
CakSNP11728	*Kabuli*	*Ca_Kabuli_Chr07*	1787417	(G/A)
CakSNP11729	*Kabuli*	*Ca_Kabuli_Chr07*	1946020	(G/A)
CakSNP11730	*Kabuli*	*Ca_Kabuli_Chr07*	1963659	(T/A)
CakSNP11731	*Kabuli*	*Ca_Kabuli_Chr07*	1963757	(A/G)
CakSNP11732	*Kabuli*	*Ca_Kabuli_Chr07*	1991642	(G/T)
CakSNP11733	*Kabuli*	*Ca_Kabuli_Chr07*	2012854	(T/A)
CakSNP11734	*Kabuli*	*Ca_Kabuli_Chr07*	2103626	(C/T)
CakSNP11735	*Kabuli*	*Ca_Kabuli_Chr07*	2106876	(C/A)
CakSNP11736	*Kabuli*	*Ca_Kabuli_Chr07*	2106917	(A/T)
CakSNP11737	*Kabuli*	*Ca_Kabuli_Chr07*	2146554	(G/A)
CakSNP11738	*Kabuli*	*Ca_Kabuli_Chr07*	2167517	(A/C)
CakSNP11739	*Kabuli*	*Ca_Kabuli_Chr07*	2167540	(G/C)
CakSNP11740	*Kabuli*	*Ca_Kabuli_Chr07*	2211897	(G/A)
CakSNP11741	*Kabuli*	*Ca_Kabuli_Chr07*	2219764	(C/T)
CakSNP11742	*Kabuli*	*Ca_Kabuli_Chr07*	2230434	(A/G)
CakSNP11743	*Kabuli*	*Ca_Kabuli_Chr07*	2262749	(C/G)
CakSNP11744	*Kabuli*	*Ca_Kabuli_Chr07*	2262865	(A/G)
CakSNP11745	*Kabuli*	*Ca_Kabuli_Chr07*	2262843	(T/A)
CakSNP11746	*Kabuli*	*Ca_Kabuli_Chr07*	2361064	(T/G)
CakSNP11747	*Kabuli*	*Ca_Kabuli_Chr07*	2387109	(C/T)
CakSNP11748	*Kabuli*	*Ca_Kabuli_Chr07*	2391392	(G/A)
CakSNP11749	*Kabuli*	*Ca_Kabuli_Chr07*	2401235	(T/C)
CakSNP11750	*Kabuli*	*Ca_Kabuli_Chr07*	2493033	(A/G)
CakSNP11751	*Kabuli*	*Ca_Kabuli_Chr07*	2538167	(C/G)
CakSNP11752	*Kabuli*	*Ca_Kabuli_Chr07*	2541501	(A/G)
CakSNP11753	*Kabuli*	*Ca_Kabuli_Chr07*	2541551	(G/A)
CakSNP11754	*Kabuli*	*Ca_Kabuli_Chr07*	2541653	(C/G)
CakSNP11755	*Kabuli*	*Ca_Kabuli_Chr07*	2545613	(T/C)
CakSNP11756	*Kabuli*	*Ca_Kabuli_Chr07*	2552454	(G/T)
CakSNP11757	*Kabuli*	*Ca_Kabuli_Chr07*	2603595	(C/T)
CakSNP11758	*Kabuli*	*Ca_Kabuli_Chr07*	2603633	(C/T)
CakSNP11759	*Kabuli*	*Ca_Kabuli_Chr07*	2603649	(T/C)
CakSNP11760	*Kabuli*	*Ca_Kabuli_Chr07*	2609831	(C/A)
CakSNP11761	*Kabuli*	*Ca_Kabuli_Chr07*	2618478	(C/T)
CakSNP11762	*Kabuli*	*Ca_Kabuli_Chr07*	2654442	(T/G)
CakSNP11763	*Kabuli*	*Ca_Kabuli_Chr07*	2654478	(C/T)
CakSNP11764	*Kabuli*	*Ca_Kabuli_Chr07*	2684213	(A/G)
CakSNP11765	*Kabuli*	*Ca_Kabuli_Chr07*	2734680	(A/G)
CakSNP11766	*Kabuli*	*Ca_Kabuli_Chr07*	2734625	(C/T)

SNP IDs	Cultivars	Chromosomes/scaffolds	Physical positions (bp)	SNPs
CakSNP11767	*Kabuli*	*Ca_Kabuli_Chr07*	2745665	(A/C)
CakSNP11768	*Kabuli*	*Ca_Kabuli_Chr07*	2745656	(A/C)
CakSNP11769	*Kabuli*	*Ca_Kabuli_Chr07*	2757260	(C/T)
CakSNP11770	*Kabuli*	*Ca_Kabuli_Chr07*	2757255	(G/C)
CakSNP11771	*Kabuli*	*Ca_Kabuli_Chr07*	2757253	(T/G)
CakSNP11772	*Kabuli*	*Ca_Kabuli_Chr07*	2762396	(G/A)
CakSNP11773	*Kabuli*	*Ca_Kabuli_Chr07*	2762406	(C/T)
CakSNP11774	*Kabuli*	*Ca_Kabuli_Chr07*	2762423	(G/A)
CakSNP11775	*Kabuli*	*Ca_Kabuli_Chr07*	2832369	(C/T)
CakSNP11776	*Kabuli*	*Ca_Kabuli_Chr07*	2857630	(T/G)
CakSNP11777	*Kabuli*	*Ca_Kabuli_Chr07*	2857818	(T/A)
CakSNP11778	*Kabuli*	*Ca_Kabuli_Chr07*	2857844	(C/T)
CakSNP11779	*Kabuli*	*Ca_Kabuli_Chr07*	2909641	(A/G)
CakSNP11780	*Kabuli*	*Ca_Kabuli_Chr07*	2915375	(C/G)
CakSNP11781	*Kabuli*	*Ca_Kabuli_Chr07*	2932309	(C/G)
CakSNP11782	*Kabuli*	*Ca_Kabuli_Chr07*	2932301	(C/A)
CakSNP11783	*Kabuli*	*Ca_Kabuli_Chr07*	2932377	(C/T)
CakSNP11784	*Kabuli*	*Ca_Kabuli_Chr07*	2956665	(C/T)
CakSNP11785	*Kabuli*	*Ca_Kabuli_Chr07*	2956767	(A/G)
CakSNP11786	*Kabuli*	*Ca_Kabuli_Chr07*	2969653	(A/G)
CakSNP11787	*Kabuli*	*Ca_Kabuli_Chr07*	2997261	(A/G)
CakSNP11788	*Kabuli*	*Ca_Kabuli_Chr07*	3023182	(T/C)
CakSNP11789	*Kabuli*	*Ca_Kabuli_Chr07*	3023154	(T/C)
CakSNP11790	*Kabuli*	*Ca_Kabuli_Chr07*	3070531	(A/C)
CakSNP11791	*Kabuli*	*Ca_Kabuli_Chr07*	3090898	(G/A)
CakSNP11792	*Kabuli*	*Ca_Kabuli_Chr07*	3093884	(T/A)
CakSNP11793	*Kabuli*	*Ca_Kabuli_Chr07*	3093953	(T/G)
CakSNP11794	*Kabuli*	*Ca_Kabuli_Chr07*	3094090	(G/A)
CakSNP11795	*Kabuli*	*Ca_Kabuli_Chr07*	3094087	(A/G)
CakSNP11796	*Kabuli*	*Ca_Kabuli_Chr07*	3107197	(C/T)
CakSNP11797	*Kabuli*	*Ca_Kabuli_Chr07*	3107239	(T/A)
CakSNP11798	*Kabuli*	*Ca_Kabuli_Chr07*	3109250	(T/C)
CakSNP11799	*Kabuli*	*Ca_Kabuli_Chr07*	3109352	(T/C)
CakSNP11800	*Kabuli*	*Ca_Kabuli_Chr07*	3110316	(A/G)
CakSNP11801	*Kabuli*	*Ca_Kabuli_Chr07*	3130070	(A/G)
CakSNP11802	*Kabuli*	*Ca_Kabuli_Chr07*	3130069	(A/G)
CakSNP11803	*Kabuli*	*Ca_Kabuli_Chr07*	3143409	(C/T)
CakSNP11804	*Kabuli*	*Ca_Kabuli_Chr07*	3186341	(C/T)
CakSNP11805	*Kabuli*	*Ca_Kabuli_Chr07*	3186262	(T/G)
CakSNP11806	*Kabuli*	*Ca_Kabuli_Chr07*	3198197	(C/A)
CakSNP11807	*Kabuli*	*Ca_Kabuli_Chr07*	3198263	(T/C)

SNP IDs	Cultivars	Chromosomes/scaffolds	Physical positions (bp)	SNPs
CakSNP11808	*Kabuli*	*Ca_Kabuli_Chr07*	3217916	(T/G)
CakSNP11809	*Kabuli*	*Ca_Kabuli_Chr07*	3236150	(A/C)
CakSNP11810	*Kabuli*	*Ca_Kabuli_Chr07*	3265823	(A/G)
CakSNP11811	*Kabuli*	*Ca_Kabuli_Chr07*	3297560	(G/A)
CakSNP11812	*Kabuli*	*Ca_Kabuli_Chr07*	3297677	(G/T)
CakSNP11813	*Kabuli*	*Ca_Kabuli_Chr07*	3315157	(A/G)
CakSNP11814	*Kabuli*	*Ca_Kabuli_Chr07*	3351037	(G/T)
CakSNP11815	*Kabuli*	*Ca_Kabuli_Chr07*	3353095	(C/T)
CakSNP11816	*Kabuli*	*Ca_Kabuli_Chr07*	3355627	(A/G)
CakSNP11817	*Kabuli*	*Ca_Kabuli_Chr07*	3355763	(A/G)
CakSNP11818	*Kabuli*	*Ca_Kabuli_Chr07*	3356399	(G/A)
CakSNP11819	*Kabuli*	*Ca_Kabuli_Chr07*	3357452	(G/A)
CakSNP11820	*Kabuli*	*Ca_Kabuli_Chr07*	3430492	(A/T)
CakSNP11821	*Kabuli*	*Ca_Kabuli_Chr07*	3430479	(G/A)
CakSNP11822	*Kabuli*	*Ca_Kabuli_Chr07*	3445031	(T/C)
CakSNP11823	*Kabuli*	*Ca_Kabuli_Chr07*	3476546	(T/C)
CakSNP11824	*Kabuli*	*Ca_Kabuli_Chr07*	3504900	(G/A)
CakSNP11825	*Kabuli*	*Ca_Kabuli_Chr07*	3505012	(A/C)
CakSNP11826	*Kabuli*	*Ca_Kabuli_Chr07*	3518048	(G/A)
CakSNP11827	*Kabuli*	*Ca_Kabuli_Chr07*	3519510	(A/G)
CakSNP11828	*Kabuli*	*Ca_Kabuli_Chr07*	3519701	(A/C)
CakSNP11829	*Kabuli*	*Ca_Kabuli_Chr07*	3521150	(A/G)
CakSNP11830	*Kabuli*	*Ca_Kabuli_Chr07*	3521257	(G/A)
CakSNP11831	*Kabuli*	*Ca_Kabuli_Chr07*	3521192	(C/A)
CakSNP11832	*Kabuli*	*Ca_Kabuli_Chr07*	3536194	(C/T)
CakSNP11833	*Kabuli*	*Ca_Kabuli_Chr07*	3536233	(T/C)
CakSNP11834	*Kabuli*	*Ca_Kabuli_Chr07*	3543765	(A/G)
CakSNP11835	*Kabuli*	*Ca_Kabuli_Chr07*	3543768	(T/C)
CakSNP11836	*Kabuli*	*Ca_Kabuli_Chr07*	3557570	(C/T)
CakSNP11837	*Kabuli*	*Ca_Kabuli_Chr07*	3560182	(G/A)
CakSNP11838	*Kabuli*	*Ca_Kabuli_Chr07*	3560250	(C/T)
CakSNP11839	*Kabuli*	*Ca_Kabuli_Chr07*	3572963	(T/C)
CakSNP11840	*Kabuli*	*Ca_Kabuli_Chr07*	3581123	(G/A)
CakSNP11841	*Kabuli*	*Ca_Kabuli_Chr07*	3600826	(C/T)
CakSNP11842	*Kabuli*	*Ca_Kabuli_Chr07*	3600833	(T/G)
CakSNP11843	*Kabuli*	*Ca_Kabuli_Chr07*	3600866	(T/C)
CakSNP11844	*Kabuli*	*Ca_Kabuli_Chr07*	3600867	(C/T)
CakSNP11845	*Kabuli*	*Ca_Kabuli_Chr07*	3600987	(A/C)
CakSNP11846	*Kabuli*	*Ca_Kabuli_Chr07*	3628086	(G/A)
CakSNP11847	*Kabuli*	*Ca_Kabuli_Chr07*	3635307	(G/A)
CakSNP11848	*Kabuli*	*Ca_Kabuli_Chr07*	3635309	(C/T)

**SNP IDs**	**Cultivars**	**Chromosomes/scaffolds**	**Physical positions (bp)**	**SNPs**
CakSNP11849	*Kabuli*	*Ca_Kabuli_Chr07*	3635318	(T/A)
CakSNP11850	*Kabuli*	*Ca_Kabuli_Chr07*	3635325	(T/C)
CakSNP11851	*Kabuli*	*Ca_Kabuli_Chr07*	3635369	(G/T)
CakSNP11852	*Kabuli*	*Ca_Kabuli_Chr07*	3651051	(T/C)
CakSNP11853	*Kabuli*	*Ca_Kabuli_Chr07*	3655451	(G/T)
CakSNP11854	*Kabuli*	*Ca_Kabuli_Chr07*	3665651	(G/C)
CakSNP11855	*Kabuli*	*Ca_Kabuli_Chr07*	3672192	(T/C)
CakSNP11856	*Kabuli*	*Ca_Kabuli_Chr07*	3672236	(G/A)
CakSNP11857	*Kabuli*	*Ca_Kabuli_Chr07*	3734030	(T/A)
CakSNP11858	*Kabuli*	*Ca_Kabuli_Chr07*	3738769	(T/C)
CakSNP11859	*Kabuli*	*Ca_Kabuli_Chr07*	3754778	(G/A)
CakSNP11860	*Kabuli*	*Ca_Kabuli_Chr07*	3754743	(G/C)
CakSNP11861	*Kabuli*	*Ca_Kabuli_Chr07*	3795825	(T/C)
CakSNP11862	*Kabuli*	*Ca_Kabuli_Chr07*	3795798	(G/A)
CakSNP11863	*Kabuli*	*Ca_Kabuli_Chr07*	3795776	(C/A)
CakSNP11864	*Kabuli*	*Ca_Kabuli_Chr07*	3796826	(A/G)
CakSNP11865	*Kabuli*	*Ca_Kabuli_Chr07*	3796894	(A/G)
CakSNP11866	*Kabuli*	*Ca_Kabuli_Chr07*	3802188	(T/A)
CakSNP11867	*Kabuli*	*Ca_Kabuli_Chr07*	3814791	(A/G)
CakSNP11868	*Kabuli*	*Ca_Kabuli_Chr07*	3817087	(C/T)
CakSNP11869	*Kabuli*	*Ca_Kabuli_Chr07*	3817733	(T/G)
CakSNP11870	*Kabuli*	*Ca_Kabuli_Chr07*	3817740	(G/T)
CakSNP11871	*Kabuli*	*Ca_Kabuli_Chr07*	3829115	(C/T)
CakSNP11872	*Kabuli*	*Ca_Kabuli_Chr07*	3843168	(A/T)
CakSNP11873	*Kabuli*	*Ca_Kabuli_Chr07*	3845953	(A/C)
CakSNP11874	*Kabuli*	*Ca_Kabuli_Chr07*	3845958	(G/A)
CakSNP11875	*Kabuli*	*Ca_Kabuli_Chr07*	3845996	(G/A)
CakSNP11876	*Kabuli*	*Ca_Kabuli_Chr07*	3856308	(C/T)
CakSNP11877	*Kabuli*	*Ca_Kabuli_Chr07*	3859451	(G/A)
CakSNP11878	*Kabuli*	*Ca_Kabuli_Chr07*	3916849	(A/C)
CakSNP11879	*Kabuli*	*Ca_Kabuli_Chr07*	3924920	(T/C)
CakSNP11880	*Kabuli*	*Ca_Kabuli_Chr07*	3946712	(G/A)
CakSNP11881	*Kabuli*	*Ca_Kabuli_Chr07*	3982635	(T/G)
CakSNP11882	*Kabuli*	*Ca_Kabuli_Chr07*	3982622	(G/A)
CakSNP11883	*Kabuli*	*Ca_Kabuli_Chr07*	4017780	(C/T)
CakSNP11884	*Kabuli*	*Ca_Kabuli_Chr07*	4017851	(T/C)
CakSNP11885	*Kabuli*	*Ca_Kabuli_Chr07*	4039347	(C/A)
CakSNP11886	*Kabuli*	*Ca_Kabuli_Chr07*	4039300	(T/C)
CakSNP11887	*Kabuli*	*Ca_Kabuli_Chr07*	4039290	(A/G)
CakSNP11888	*Kabuli*	*Ca_Kabuli_Chr07*	4042123	(T/G)
CakSNP11889	*Kabuli*	*Ca_Kabuli_Chr07*	4095293	(A/G)

**SNP IDs**	**Cultivars**	**Chromosomes/scaffolds**	**Physical positions (bp)**	**SNPs**
CakSNP11890	*Kabuli*	*Ca_Kabuli_Chr07*	4168933	(G/T)
CakSNP11891	*Kabuli*	*Ca_Kabuli_Chr07*	4194809	(T/A)
CakSNP11892	*Kabuli*	*Ca_Kabuli_Chr07*	4194890	(C/A)
CakSNP11893	*Kabuli*	*Ca_Kabuli_Chr07*	4194891	(C/G)
CakSNP11894	*Kabuli*	*Ca_Kabuli_Chr07*	4198812	(A/C)
CakSNP11895	*Kabuli*	*Ca_Kabuli_Chr07*	4209425	(C/T)
CakSNP11896	*Kabuli*	*Ca_Kabuli_Chr07*	4209465	(A/C)
CakSNP11897	*Kabuli*	*Ca_Kabuli_Chr07*	4225314	(T/C)
CakSNP11898	*Kabuli*	*Ca_Kabuli_Chr07*	4256529	(G/A)
CakSNP11899	*Kabuli*	*Ca_Kabuli_Chr07*	4256703	(C/G)
CakSNP11900	*Kabuli*	*Ca_Kabuli_Chr07*	4257726	(A/T)
CakSNP11901	*Kabuli*	*Ca_Kabuli_Chr07*	4257727	(T/A)
CakSNP11902	*Kabuli*	*Ca_Kabuli_Chr07*	4257838	(C/T)
CakSNP11903	*Kabuli*	*Ca_Kabuli_Chr07*	4257830	(G/T)
CakSNP11904	*Kabuli*	*Ca_Kabuli_Chr07*	4257799	(G/A)
CakSNP11905	*Kabuli*	*Ca_Kabuli_Chr07*	4289975	(G/A)
CakSNP11906	*Kabuli*	*Ca_Kabuli_Chr07*	4300525	(C/T)
CakSNP11907	*Kabuli*	*Ca_Kabuli_Chr07*	4300650	(T/C)
CakSNP11908	*Kabuli*	*Ca_Kabuli_Chr07*	4300645	(C/T)
CakSNP11909	*Kabuli*	*Ca_Kabuli_Chr07*	4300592	(G/A)
CakSNP11910	*Kabuli*	*Ca_Kabuli_Chr07*	4300584	(C/T)
CakSNP11911	*Kabuli*	*Ca_Kabuli_Chr07*	4305424	(G/T)
CakSNP11912	*Kabuli*	*Ca_Kabuli_Chr07*	4431138	(A/G)
CakSNP11913	*Kabuli*	*Ca_Kabuli_Chr07*	4431253	(T/C)
CakSNP11914	*Kabuli*	*Ca_Kabuli_Chr07*	4438929	(A/G)
CakSNP11915	*Kabuli*	*Ca_Kabuli_Chr07*	4455045	(T/C)
CakSNP11916	*Kabuli*	*Ca_Kabuli_Chr07*	4461990	(A/C)
CakSNP11917	*Kabuli*	*Ca_Kabuli_Chr07*	4470285	(C/G)
CakSNP11918	*Kabuli*	*Ca_Kabuli_Chr07*	4490514	(G/A)
CakSNP11919	*Kabuli*	*Ca_Kabuli_Chr07*	4490964	(G/T)
CakSNP11920	*Kabuli*	*Ca_Kabuli_Chr07*	4490966	(A/G)
CakSNP11921	*Kabuli*	*Ca_Kabuli_Chr07*	4497784	(C/T)
CakSNP11922	*Kabuli*	*Ca_Kabuli_Chr07*	4520606	(T/C)
CakSNP11923	*Kabuli*	*Ca_Kabuli_Chr07*	4525645	(A/G)
CakSNP11924	*Kabuli*	*Ca_Kabuli_Chr07*	4552790	(T/C)
CakSNP11925	*Kabuli*	*Ca_Kabuli_Chr07*	4573236	(G/A)
CakSNP11926	*Kabuli*	*Ca_Kabuli_Chr07*	4625723	(C/T)
CakSNP11927	*Kabuli*	*Ca_Kabuli_Chr07*	4648569	(A/C)
CakSNP11928	*Kabuli*	*Ca_Kabuli_Chr07*	4648697	(T/A)
CakSNP11929	*Kabuli*	*Ca_Kabuli_Chr07*	4648677	(A/T)
CakSNP11930	*Kabuli*	*Ca_Kabuli_Chr07*	4648662	(G/A)

SNP IDs	Cultivars	Chromosomes/scaffolds	Physical positions (bp)	SNPs
CakSNP11931	*Kabuli*	*Ca_Kabuli_Chr07*	4649287	(A/G)
CakSNP11932	*Kabuli*	*Ca_Kabuli_Chr07*	4651931	(T/C)
CakSNP11933	*Kabuli*	*Ca_Kabuli_Chr07*	4652083	(G/A)
CakSNP11934	*Kabuli*	*Ca_Kabuli_Chr07*	4653235	(A/C)
CakSNP11935	*Kabuli*	*Ca_Kabuli_Chr07*	4700570	(C/A)
CakSNP11936	*Kabuli*	*Ca_Kabuli_Chr07*	4749212	(T/G)
CakSNP11937	*Kabuli*	*Ca_Kabuli_Chr07*	4752954	(G/C)
CakSNP11938	*Kabuli*	*Ca_Kabuli_Chr07*	4752887	(T/G)
CakSNP11939	*Kabuli*	*Ca_Kabuli_Chr07*	4756338	(G/A)
CakSNP11940	*Kabuli*	*Ca_Kabuli_Chr07*	4832993	(A/G)
CakSNP11941	*Kabuli*	*Ca_Kabuli_Chr07*	4834177	(G/T)
CakSNP11942	*Kabuli*	*Ca_Kabuli_Chr07*	4849553	(T/G)
CakSNP11943	*Kabuli*	*Ca_Kabuli_Chr07*	4849573	(T/C)
CakSNP11944	*Kabuli*	*Ca_Kabuli_Chr07*	4907315	(C/T)
CakSNP11945	*Kabuli*	*Ca_Kabuli_Chr07*	4926759	(A/T)
CakSNP11946	*Kabuli*	*Ca_Kabuli_Chr07*	4942847	(A/G)
CakSNP11947	*Kabuli*	*Ca_Kabuli_Chr07*	4942902	(G/C)
CakSNP11948	*Kabuli*	*Ca_Kabuli_Chr07*	4945935	(C/G)
CakSNP11949	*Kabuli*	*Ca_Kabuli_Chr07*	4981846	(T/C)
CakSNP11950	*Kabuli*	*Ca_Kabuli_Chr07*	5122536	(T/G)
CakSNP11951	*Kabuli*	*Ca_Kabuli_Chr07*	5251897	(G/C)
CakSNP11952	*Kabuli*	*Ca_Kabuli_Chr07*	5361558	(T/C)
CakSNP11953	*Kabuli*	*Ca_Kabuli_Chr07*	5383564	(G/C)
CakSNP11954	*Kabuli*	*Ca_Kabuli_Chr07*	5383566	(A/T)
CakSNP11955	*Kabuli*	*Ca_Kabuli_Chr07*	5383671	(G/T)
CakSNP11956	*Kabuli*	*Ca_Kabuli_Chr07*	5383695	(T/G)
CakSNP11957	*Kabuli*	*Ca_Kabuli_Chr07*	5383767	(G/A)
CakSNP11958	*Kabuli*	*Ca_Kabuli_Chr07*	5384447	(G/C)
CakSNP11959	*Kabuli*	*Ca_Kabuli_Chr07*	5384467	(C/T)
CakSNP11960	*Kabuli*	*Ca_Kabuli_Chr07*	5406524	(T/G)
CakSNP11961	*Kabuli*	*Ca_Kabuli_Chr07*	5416862	(C/G)
CakSNP11962	*Kabuli*	*Ca_Kabuli_Chr07*	5416865	(A/G)
CakSNP11963	*Kabuli*	*Ca_Kabuli_Chr07*	5416874	(C/T)
CakSNP11964	*Kabuli*	*Ca_Kabuli_Chr07*	5464941	(G/A)
CakSNP11965	*Kabuli*	*Ca_Kabuli_Chr07*	5464942	(A/G)
CakSNP11966	*Kabuli*	*Ca_Kabuli_Chr07*	5467744	(G/C)
CakSNP11967	*Kabuli*	*Ca_Kabuli_Chr07*	5500318	(C/A)
CakSNP11968	*Kabuli*	*Ca_Kabuli_Chr07*	5508162	(A/C)
CakSNP11969	*Kabuli*	*Ca_Kabuli_Chr07*	5520720	(T/G)
CakSNP11970	*Kabuli*	*Ca_Kabuli_Chr07*	5520934	(T/C)
CakSNP11971	*Kabuli*	*Ca_Kabuli_Chr07*	5521596	(T/C)

**SNP IDs**	**Cultivars**	**Chromosomes/scaffolds**	**Physical positions (bp)**	**SNPs**
CakSNP11972	*Kabuli*	*Ca_Kabuli_Chr07*	5521659	(G/T)
CakSNP11973	*Kabuli*	*Ca_Kabuli_Chr07*	5529577	(T/G)
CakSNP11974	*Kabuli*	*Ca_Kabuli_Chr07*	5529729	(A/G)
CakSNP11975	*Kabuli*	*Ca_Kabuli_Chr07*	5529888	(A/G)
CakSNP11976	*Kabuli*	*Ca_Kabuli_Chr07*	5529844	(C/A)
CakSNP11977	*Kabuli*	*Ca_Kabuli_Chr07*	5537851	(G/C)
CakSNP11978	*Kabuli*	*Ca_Kabuli_Chr07*	5537880	(G/T)
CakSNP11979	*Kabuli*	*Ca_Kabuli_Chr07*	5561607	(G/T)
CakSNP11980	*Kabuli*	*Ca_Kabuli_Chr07*	5568114	(C/A)
CakSNP11981	*Kabuli*	*Ca_Kabuli_Chr07*	5577467	(G/T)
CakSNP11982	*Kabuli*	*Ca_Kabuli_Chr07*	5577439	(A/G)
CakSNP11983	*Kabuli*	*Ca_Kabuli_Chr07*	5581968	(G/C)
CakSNP11984	*Kabuli*	*Ca_Kabuli_Chr07*	5602106	(T/C)
CakSNP11985	*Kabuli*	*Ca_Kabuli_Chr07*	5602144	(G/T)
CakSNP11986	*Kabuli*	*Ca_Kabuli_Chr07*	5605118	(G/A)
CakSNP11987	*Kabuli*	*Ca_Kabuli_Chr07*	5605106	(G/A)
CakSNP11988	*Kabuli*	*Ca_Kabuli_Chr07*	5658445	(G/T)
CakSNP11989	*Kabuli*	*Ca_Kabuli_Chr07*	5671902	(G/T)
CakSNP11990	*Kabuli*	*Ca_Kabuli_Chr07*	5672009	(G/C)
CakSNP11991	*Kabuli*	*Ca_Kabuli_Chr07*	5672156	(T/C)
CakSNP11992	*Kabuli*	*Ca_Kabuli_Chr07*	5683412	(T/A)
CakSNP11993	*Kabuli*	*Ca_Kabuli_Chr07*	5713977	(T/C)
CakSNP11994	*Kabuli*	*Ca_Kabuli_Chr07*	5735293	(T/A)
CakSNP11995	*Kabuli*	*Ca_Kabuli_Chr07*	5735448	(A/T)
CakSNP11996	*Kabuli*	*Ca_Kabuli_Chr07*	5737341	(C/T)
CakSNP11997	*Kabuli*	*Ca_Kabuli_Chr07*	5737305	(G/A)
CakSNP11998	*Kabuli*	*Ca_Kabuli_Chr07*	5756269	(C/T)
CakSNP11999	*Kabuli*	*Ca_Kabuli_Chr07*	5756832	(A/C)
CakSNP12000	*Kabuli*	*Ca_Kabuli_Chr07*	5774218	(T/A)
CakSNP12001	*Kabuli*	*Ca_Kabuli_Chr07*	5774219	(T/A)
CakSNP12002	*Kabuli*	*Ca_Kabuli_Chr07*	5774220	(A/T)
CakSNP12003	*Kabuli*	*Ca_Kabuli_Chr07*	5774221	(A/T)
CakSNP12004	*Kabuli*	*Ca_Kabuli_Chr07*	5774230	(G/A)
CakSNP12005	*Kabuli*	*Ca_Kabuli_Chr07*	5774257	(C/T)
CakSNP12006	*Kabuli*	*Ca_Kabuli_Chr07*	5774310	(A/G)
CakSNP12007	*Kabuli*	*Ca_Kabuli_Chr07*	5811097	(G/A)
CakSNP12008	*Kabuli*	*Ca_Kabuli_Chr07*	5811060	(T/C)
CakSNP12009	*Kabuli*	*Ca_Kabuli_Chr07*	5811044	(T/A)
CakSNP12010	*Kabuli*	*Ca_Kabuli_Chr07*	5836421	(A/T)
CakSNP12011	*Kabuli*	*Ca_Kabuli_Chr07*	5846477	(A/G)
CakSNP12012	*Kabuli*	*Ca_Kabuli_Chr07*	5849936	(C/T)

SNP IDs	Cultivars	Chromosomes/scaffolds	Physical positions (bp)	SNPs
CakSNP12013	*Kabuli*	*Ca_Kabuli_Chr07*	5849904	(C/A)
CakSNP12014	*Kabuli*	*Ca_Kabuli_Chr07*	5850058	(T/C)
CakSNP12015	*Kabuli*	*Ca_Kabuli_Chr07*	5854598	(C/T)
CakSNP12016	*Kabuli*	*Ca_Kabuli_Chr07*	5929009	(C/G)
CakSNP12017	*Kabuli*	*Ca_Kabuli_Chr07*	5946113	(G/A)
CakSNP12018	*Kabuli*	*Ca_Kabuli_Chr07*	5950352	(T/A)
CakSNP12019	*Kabuli*	*Ca_Kabuli_Chr07*	5950492	(T/C)
CakSNP12020	*Kabuli*	*Ca_Kabuli_Chr07*	5957696	(T/C)
CakSNP12021	*Kabuli*	*Ca_Kabuli_Chr07*	5963611	(C/G)
CakSNP12022	*Kabuli*	*Ca_Kabuli_Chr07*	5963609	(C/A)
CakSNP12023	*Kabuli*	*Ca_Kabuli_Chr07*	6012561	(A/C)
CakSNP12024	*Kabuli*	*Ca_Kabuli_Chr07*	6012611	(A/C)
CakSNP12025	*Kabuli*	*Ca_Kabuli_Chr07*	6029991	(G/A)
CakSNP12026	*Kabuli*	*Ca_Kabuli_Chr07*	6029990	(C/G)
CakSNP12027	*Kabuli*	*Ca_Kabuli_Chr07*	6066322	(G/A)
CakSNP12028	*Kabuli*	*Ca_Kabuli_Chr07*	6074408	(T/A)
CakSNP12029	*Kabuli*	*Ca_Kabuli_Chr07*	6075124	(T/G)
CakSNP12030	*Kabuli*	*Ca_Kabuli_Chr07*	6075218	(C/G)
CakSNP12031	*Kabuli*	*Ca_Kabuli_Chr07*	6096238	(C/A)
CakSNP12032	*Kabuli*	*Ca_Kabuli_Chr07*	6096237	(C/G)
CakSNP12033	*Kabuli*	*Ca_Kabuli_Chr07*	6110019	(T/C)
CakSNP12034	*Kabuli*	*Ca_Kabuli_Chr07*	6132021	(G/A)
CakSNP12035	*Kabuli*	*Ca_Kabuli_Chr07*	6232777	(T/C)
CakSNP12036	*Kabuli*	*Ca_Kabuli_Chr07*	6282979	(C/A)
CakSNP12037	*Kabuli*	*Ca_Kabuli_Chr07*	6282974	(T/A)
CakSNP12038	*Kabuli*	*Ca_Kabuli_Chr07*	6282949	(T/A)
CakSNP12039	*Kabuli*	*Ca_Kabuli_Chr07*	6299248	(A/G)
CakSNP12040	*Kabuli*	*Ca_Kabuli_Chr07*	6299249	(C/G)
CakSNP12041	*Kabuli*	*Ca_Kabuli_Chr07*	6330237	(A/T)
CakSNP12042	*Kabuli*	*Ca_Kabuli_Chr07*	6340051	(A/G)
CakSNP12043	*Kabuli*	*Ca_Kabuli_Chr07*	6340064	(C/G)
CakSNP12044	*Kabuli*	*Ca_Kabuli_Chr07*	6340111	(C/G)
CakSNP12045	*Kabuli*	*Ca_Kabuli_Chr07*	6340113	(G/A)
CakSNP12046	*Kabuli*	*Ca_Kabuli_Chr07*	6340167	(T/A)
CakSNP12047	*Kabuli*	*Ca_Kabuli_Chr07*	6340216	(T/C)
CakSNP12048	*Kabuli*	*Ca_Kabuli_Chr07*	6372037	(A/G)
CakSNP12049	*Kabuli*	*Ca_Kabuli_Chr07*	6393366	(G/A)
CakSNP12050	*Kabuli*	*Ca_Kabuli_Chr07*	6430396	(T/A)
CakSNP12051	*Kabuli*	*Ca_Kabuli_Chr07*	6433480	(C/G)
CakSNP12052	*Kabuli*	*Ca_Kabuli_Chr07*	6467903	(C/T)
CakSNP12053	*Kabuli*	*Ca_Kabuli_Chr07*	6540624	(A/G)

**SNP IDs**	**Cultivars**	**Chromosomes/scaffolds**	**Physical positions (bp)**	**SNPs**
CakSNP12054	*Kabuli*	*Ca_Kabuli_Chr07*	6540602	(G/A)
CakSNP12055	*Kabuli*	*Ca_Kabuli_Chr07*	6540601	(G/C)
CakSNP12056	*Kabuli*	*Ca_Kabuli_Chr07*	6566322	(T/C)
CakSNP12057	*Kabuli*	*Ca_Kabuli_Chr07*	6654718	(C/T)
CakSNP12058	*Kabuli*	*Ca_Kabuli_Chr07*	6681050	(C/T)
CakSNP12059	*Kabuli*	*Ca_Kabuli_Chr07*	6682456	(T/A)
CakSNP12060	*Kabuli*	*Ca_Kabuli_Chr07*	6682454	(A/G)
CakSNP12061	*Kabuli*	*Ca_Kabuli_Chr07*	6682453	(G/T)
CakSNP12062	*Kabuli*	*Ca_Kabuli_Chr07*	6682448	(T/C)
CakSNP12063	*Kabuli*	*Ca_Kabuli_Chr07*	6682443	(C/T)
CakSNP12064	*Kabuli*	*Ca_Kabuli_Chr07*	6682439	(T/A)
CakSNP12065	*Kabuli*	*Ca_Kabuli_Chr07*	6682455	(G/T)
CakSNP12066	*Kabuli*	*Ca_Kabuli_Chr07*	6703759	(C/T)
CakSNP12067	*Kabuli*	*Ca_Kabuli_Chr07*	6703849	(G/T)
CakSNP12068	*Kabuli*	*Ca_Kabuli_Chr07*	6767204	(G/T)
CakSNP12069	*Kabuli*	*Ca_Kabuli_Chr07*	6769694	(T/G)
CakSNP12070	*Kabuli*	*Ca_Kabuli_Chr07*	6792352	(T/C)
CakSNP12071	*Kabuli*	*Ca_Kabuli_Chr07*	6792606	(T/G)
CakSNP12072	*Kabuli*	*Ca_Kabuli_Chr07*	6807817	(A/C)
CakSNP12073	*Kabuli*	*Ca_Kabuli_Chr07*	6814954	(G/T)
CakSNP12074	*Kabuli*	*Ca_Kabuli_Chr07*	6815151	(G/T)
CakSNP12075	*Kabuli*	*Ca_Kabuli_Chr07*	6815113	(T/C)
CakSNP12076	*Kabuli*	*Ca_Kabuli_Chr07*	6859511	(C/G)
CakSNP12077	*Kabuli*	*Ca_Kabuli_Chr07*	6904686	(G/C)
CakSNP12078	*Kabuli*	*Ca_Kabuli_Chr07*	7026448	(C/T)
CakSNP12079	*Kabuli*	*Ca_Kabuli_Chr07*	7026516	(T/C)
CakSNP12080	*Kabuli*	*Ca_Kabuli_Chr07*	7026500	(T/A)
CakSNP12081	*Kabuli*	*Ca_Kabuli_Chr07*	7072853	(G/A)
CakSNP12082	*Kabuli*	*Ca_Kabuli_Chr07*	7126974	(T/C)
CakSNP12083	*Kabuli*	*Ca_Kabuli_Chr07*	7126979	(C/G)
CakSNP12084	*Kabuli*	*Ca_Kabuli_Chr07*	7127030	(G/A)
CakSNP12085	*Kabuli*	*Ca_Kabuli_Chr07*	7146159	(C/G)
CakSNP12086	*Kabuli*	*Ca_Kabuli_Chr07*	7147557	(A/G)
CakSNP12087	*Kabuli*	*Ca_Kabuli_Chr07*	7234422	(T/G)
CakSNP12088	*Kabuli*	*Ca_Kabuli_Chr07*	7234507	(T/C)
CakSNP12089	*Kabuli*	*Ca_Kabuli_Chr07*	7234484	(G/A)
CakSNP12090	*Kabuli*	*Ca_Kabuli_Chr07*	7234430	(C/T)
CakSNP12091	*Kabuli*	*Ca_Kabuli_Chr07*	7261971	(C/T)
CakSNP12092	*Kabuli*	*Ca_Kabuli_Chr07*	7261941	(T/C)
CakSNP12093	*Kabuli*	*Ca_Kabuli_Chr07*	7265386	(T/A)
CakSNP12094	*Kabuli*	*Ca_Kabuli_Chr07*	7277353	(G/A)

SNP IDs	Cultivars	Chromosomes/scaffolds	Physical positions (bp)	SNPs
CakSNP12095	*Kabuli*	*Ca_Kabuli_Chr07*	7304614	(T/G)
CakSNP12096	*Kabuli*	*Ca_Kabuli_Chr07*	7304617	(T/G)
CakSNP12097	*Kabuli*	*Ca_Kabuli_Chr07*	7423419	(G/A)
CakSNP12098	*Kabuli*	*Ca_Kabuli_Chr07*	7433827	(A/T)
CakSNP12099	*Kabuli*	*Ca_Kabuli_Chr07*	7449489	(T/C)
CakSNP12100	*Kabuli*	*Ca_Kabuli_Chr07*	7451931	(G/C)
CakSNP12101	*Kabuli*	*Ca_Kabuli_Chr07*	7491960	(G/A)
CakSNP12102	*Kabuli*	*Ca_Kabuli_Chr07*	7498598	(C/T)
CakSNP12103	*Kabuli*	*Ca_Kabuli_Chr07*	7544142	(A/G)
CakSNP12104	*Kabuli*	*Ca_Kabuli_Chr07*	7573889	(C/G)
CakSNP12105	*Kabuli*	*Ca_Kabuli_Chr07*	7576668	(A/C)
CakSNP12106	*Kabuli*	*Ca_Kabuli_Chr07*	7580158	(T/C)
CakSNP12107	*Kabuli*	*Ca_Kabuli_Chr07*	7580147	(C/A)
CakSNP12108	*Kabuli*	*Ca_Kabuli_Chr07*	7580118	(G/A)
CakSNP12109	*Kabuli*	*Ca_Kabuli_Chr07*	7612046	(A/T)
CakSNP12110	*Kabuli*	*Ca_Kabuli_Chr07*	7624697	(G/A)
CakSNP12111	*Kabuli*	*Ca_Kabuli_Chr07*	7625226	(G/T)
CakSNP12112	*Kabuli*	*Ca_Kabuli_Chr07*	7625277	(A/T)
CakSNP12113	*Kabuli*	*Ca_Kabuli_Chr07*	7625901	(A/C)
CakSNP12114	*Kabuli*	*Ca_Kabuli_Chr07*	7625943	(C/G)
CakSNP12115	*Kabuli*	*Ca_Kabuli_Chr07*	7663564	(A/G)
CakSNP12116	*Kabuli*	*Ca_Kabuli_Chr07*	7706816	(A/G)
CakSNP12117	*Kabuli*	*Ca_Kabuli_Chr07*	7756455	(C/T)
CakSNP12118	*Kabuli*	*Ca_Kabuli_Chr07*	7756482	(G/A)
CakSNP12119	*Kabuli*	*Ca_Kabuli_Chr07*	7756566	(G/A)
CakSNP12120	*Kabuli*	*Ca_Kabuli_Chr07*	7809179	(T/G)
CakSNP12121	*Kabuli*	*Ca_Kabuli_Chr07*	7809192	(A/C)
CakSNP12122	*Kabuli*	*Ca_Kabuli_Chr07*	7875189	(C/A)
CakSNP12123	*Kabuli*	*Ca_Kabuli_Chr07*	7876554	(G/A)
CakSNP12124	*Kabuli*	*Ca_Kabuli_Chr07*	7876561	(C/T)
CakSNP12125	*Kabuli*	*Ca_Kabuli_Chr07*	7876570	(C/A)
CakSNP12126	*Kabuli*	*Ca_Kabuli_Chr07*	7928398	(C/T)
CakSNP12127	*Kabuli*	*Ca_Kabuli_Chr07*	7982650	(G/T)
CakSNP12128	*Kabuli*	*Ca_Kabuli_Chr07*	7982871	(T/C)
CakSNP12129	*Kabuli*	*Ca_Kabuli_Chr07*	7982890	(T/A)
CakSNP12130	*Kabuli*	*Ca_Kabuli_Chr07*	7983519	(C/T)
CakSNP12131	*Kabuli*	*Ca_Kabuli_Chr07*	8041465	(T/G)
CakSNP12132	*Kabuli*	*Ca_Kabuli_Chr07*	8054489	(G/A)
CakSNP12133	*Kabuli*	*Ca_Kabuli_Chr07*	8061808	(A/G)
CakSNP12134	*Kabuli*	*Ca_Kabuli_Chr07*	8111735	(T/C)
CakSNP12135	*Kabuli*	*Ca_Kabuli_Chr07*	8111754	(A/C)

SNP IDs	Cultivars	Chromosomes/scaffolds	Physical positions (bp)	SNPs
CakSNP12136	*Kabuli*	*Ca_Kabuli_Chr07*	8111759	(G/T)
CakSNP12137	*Kabuli*	*Ca_Kabuli_Chr07*	8169857	(A/G)
CakSNP12138	*Kabuli*	*Ca_Kabuli_Chr07*	8169866	(A/G)
CakSNP12139	*Kabuli*	*Ca_Kabuli_Chr07*	8169960	(A/T)
CakSNP12140	*Kabuli*	*Ca_Kabuli_Chr07*	8169947	(T/C)
CakSNP12141	*Kabuli*	*Ca_Kabuli_Chr07*	8187181	(G/A)
CakSNP12142	*Kabuli*	*Ca_Kabuli_Chr07*	8188004	(C/G)
CakSNP12143	*Kabuli*	*Ca_Kabuli_Chr07*	8210963	(G/A)
CakSNP12144	*Kabuli*	*Ca_Kabuli_Chr07*	8261062	(C/A)
CakSNP12145	*Kabuli*	*Ca_Kabuli_Chr07*	8286115	(A/T)
CakSNP12146	*Kabuli*	*Ca_Kabuli_Chr07*	8515092	(C/T)
CakSNP12147	*Kabuli*	*Ca_Kabuli_Chr07*	8535189	(C/T)
CakSNP12148	*Kabuli*	*Ca_Kabuli_Chr07*	8553114	(C/T)
CakSNP12149	*Kabuli*	*Ca_Kabuli_Chr07*	8569632	(A/G)
CakSNP12150	*Kabuli*	*Ca_Kabuli_Chr07*	8583890	(T/C)
CakSNP12151	*Kabuli*	*Ca_Kabuli_Chr07*	8609831	(A/T)
CakSNP12152	*Kabuli*	*Ca_Kabuli_Chr07*	8626840	(A/C)
CakSNP12153	*Kabuli*	*Ca_Kabuli_Chr07*	8626875	(A/T)
CakSNP12154	*Kabuli*	*Ca_Kabuli_Chr07*	8626916	(T/C)
CakSNP12155	*Kabuli*	*Ca_Kabuli_Chr07*	8741098	(T/C)
CakSNP12156	*Kabuli*	*Ca_Kabuli_Chr07*	8777670	(A/G)
CakSNP12157	*Kabuli*	*Ca_Kabuli_Chr07*	8777673	(A/G)
CakSNP12158	*Kabuli*	*Ca_Kabuli_Chr07*	8777690	(G/A)
CakSNP12159	*Kabuli*	*Ca_Kabuli_Chr07*	8824586	(A/G)
CakSNP12160	*Kabuli*	*Ca_Kabuli_Chr07*	8824591	(C/T)
CakSNP12161	*Kabuli*	*Ca_Kabuli_Chr07*	8830772	(A/C)
CakSNP12162	*Kabuli*	*Ca_Kabuli_Chr07*	8830985	(A/G)
CakSNP12163	*Kabuli*	*Ca_Kabuli_Chr07*	8831024	(C/T)
CakSNP12164	*Kabuli*	*Ca_Kabuli_Chr07*	8831096	(G/A)
CakSNP12165	*Kabuli*	*Ca_Kabuli_Chr07*	8835601	(G/A)
CakSNP12166	*Kabuli*	*Ca_Kabuli_Chr07*	8835602	(A/G)
CakSNP12167	*Kabuli*	*Ca_Kabuli_Chr07*	8835610	(G/A)
CakSNP12168	*Kabuli*	*Ca_Kabuli_Chr07*	8835659	(G/T)
CakSNP12169	*Kabuli*	*Ca_Kabuli_Chr07*	8835757	(T/C)
CakSNP12170	*Kabuli*	*Ca_Kabuli_Chr07*	8835718	(T/G)
CakSNP12171	*Kabuli*	*Ca_Kabuli_Chr07*	8835705	(T/A)
CakSNP12172	*Kabuli*	*Ca_Kabuli_Chr07*	8844902	(C/T)
CakSNP12173	*Kabuli*	*Ca_Kabuli_Chr07*	8894362	(A/T)
CakSNP12174	*Kabuli*	*Ca_Kabuli_Chr07*	8895087	(T/C)
CakSNP12175	*Kabuli*	*Ca_Kabuli_Chr07*	8913092	(C/G)
CakSNP12176	*Kabuli*	*Ca_Kabuli_Chr07*	8937391	(A/G)

SNP IDs	Cultivars	Chromosomes/scaffolds	Physical positions (bp)	SNPs
CakSNP12177	*Kabuli*	*Ca_Kabuli_Chr07*	8970387	(A/G)
CakSNP12178	*Kabuli*	*Ca_Kabuli_Chr07*	9128044	(A/G)
CakSNP12179	*Kabuli*	*Ca_Kabuli_Chr07*	9128050	(C/A)
CakSNP12180	*Kabuli*	*Ca_Kabuli_Chr07*	9147367	(A/T)
CakSNP12181	*Kabuli*	*Ca_Kabuli_Chr07*	9147366	(T/G)
CakSNP12182	*Kabuli*	*Ca_Kabuli_Chr07*	9147502	(C/T)
CakSNP12183	*Kabuli*	*Ca_Kabuli_Chr07*	9151709	(T/G)
CakSNP12184	*Kabuli*	*Ca_Kabuli_Chr07*	9151755	(C/T)
CakSNP12185	*Kabuli*	*Ca_Kabuli_Chr07*	9153104	(C/A)
CakSNP12186	*Kabuli*	*Ca_Kabuli_Chr07*	9153062	(G/A)
CakSNP12187	*Kabuli*	*Ca_Kabuli_Chr07*	9214457	(T/A)
CakSNP12188	*Kabuli*	*Ca_Kabuli_Chr07*	9214422	(C/T)
CakSNP12189	*Kabuli*	*Ca_Kabuli_Chr07*	9301825	(G/C)
CakSNP12190	*Kabuli*	*Ca_Kabuli_Chr07*	9301881	(C/T)
CakSNP12191	*Kabuli*	*Ca_Kabuli_Chr07*	9327709	(C/T)
CakSNP12192	*Kabuli*	*Ca_Kabuli_Chr07*	9327687	(G/A)
CakSNP12193	*Kabuli*	*Ca_Kabuli_Chr07*	9348281	(T/C)
CakSNP12194	*Kabuli*	*Ca_Kabuli_Chr07*	9350506	(T/G)
CakSNP12195	*Kabuli*	*Ca_Kabuli_Chr07*	9350535	(A/C)
CakSNP12196	*Kabuli*	*Ca_Kabuli_Chr07*	9351783	(C/G)
CakSNP12197	*Kabuli*	*Ca_Kabuli_Chr07*	9351863	(G/A)
CakSNP12198	*Kabuli*	*Ca_Kabuli_Chr07*	9422842	(T/C)
CakSNP12199	*Kabuli*	*Ca_Kabuli_Chr07*	9422859	(T/C)
CakSNP12200	*Kabuli*	*Ca_Kabuli_Chr07*	9422876	(T/C)
CakSNP12201	*Kabuli*	*Ca_Kabuli_Chr07*	9422946	(G/C)
CakSNP12202	*Kabuli*	*Ca_Kabuli_Chr07*	9442836	(A/C)
CakSNP12203	*Kabuli*	*Ca_Kabuli_Chr07*	9475275	(T/G)
CakSNP12204	*Kabuli*	*Ca_Kabuli_Chr07*	9475817	(C/A)
CakSNP12205	*Kabuli*	*Ca_Kabuli_Chr07*	9475922	(A/G)
CakSNP12206	*Kabuli*	*Ca_Kabuli_Chr07*	9475874	(C/T)
CakSNP12207	*Kabuli*	*Ca_Kabuli_Chr07*	9523902	(C/T)
CakSNP12208	*Kabuli*	*Ca_Kabuli_Chr07*	9528324	(A/G)
CakSNP12209	*Kabuli*	*Ca_Kabuli_Chr07*	9536012	(C/T)
CakSNP12210	*Kabuli*	*Ca_Kabuli_Chr07*	9550284	(G/C)
CakSNP12211	*Kabuli*	*Ca_Kabuli_Chr07*	9550230	(C/T)
CakSNP12212	*Kabuli*	*Ca_Kabuli_Chr07*	9555319	(C/G)
CakSNP12213	*Kabuli*	*Ca_Kabuli_Chr07*	9577441	(G/A)
CakSNP12214	*Kabuli*	*Ca_Kabuli_Chr07*	9577424	(G/C)
CakSNP12215	*Kabuli*	*Ca_Kabuli_Chr07*	9593677	(C/T)
CakSNP12216	*Kabuli*	*Ca_Kabuli_Chr07*	9612558	(A/C)
CakSNP12217	*Kabuli*	*Ca_Kabuli_Chr07*	9616335	(G/A)

SNP IDs	Cultivars	Chromosomes/scaffolds	Physical positions (bp)	SNPs
CakSNP12218	*Kabuli*	*Ca_Kabuli_Chr07*	9616347	(C/G)
CakSNP12219	*Kabuli*	*Ca_Kabuli_Chr07*	9616357	(C/T)
CakSNP12220	*Kabuli*	*Ca_Kabuli_Chr07*	9616363	(A/C)
CakSNP12221	*Kabuli*	*Ca_Kabuli_Chr07*	9616367	(C/G)
CakSNP12222	*Kabuli*	*Ca_Kabuli_Chr07*	9616378	(G/A)
CakSNP12223	*Kabuli*	*Ca_Kabuli_Chr07*	9637456	(C/T)
CakSNP12224	*Kabuli*	*Ca_Kabuli_Chr07*	9639993	(C/A)
CakSNP12225	*Kabuli*	*Ca_Kabuli_Chr07*	9661050	(A/C)
CakSNP12226	*Kabuli*	*Ca_Kabuli_Chr07*	9671210	(T/C)
CakSNP12227	*Kabuli*	*Ca_Kabuli_Chr07*	9696620	(A/G)
CakSNP12228	*Kabuli*	*Ca_Kabuli_Chr07*	9738801	(T/C)
CakSNP12229	*Kabuli*	*Ca_Kabuli_Chr07*	9744436	(G/T)
CakSNP12230	*Kabuli*	*Ca_Kabuli_Chr07*	9890779	(A/C)
CakSNP12231	*Kabuli*	*Ca_Kabuli_Chr07*	9890777	(A/G)
CakSNP12232	*Kabuli*	*Ca_Kabuli_Chr07*	9893688	(A/G)
CakSNP12233	*Kabuli*	*Ca_Kabuli_Chr07*	9897698	(T/G)
CakSNP12234	*Kabuli*	*Ca_Kabuli_Chr07*	9898718	(A/T)
CakSNP12235	*Kabuli*	*Ca_Kabuli_Chr07*	9899348	(C/T)
CakSNP12236	*Kabuli*	*Ca_Kabuli_Chr07*	9914751	(G/A)
CakSNP12237	*Kabuli*	*Ca_Kabuli_Chr07*	9914764	(G/A)
CakSNP12238	*Kabuli*	*Ca_Kabuli_Chr07*	9914770	(T/C)
CakSNP12239	*Kabuli*	*Ca_Kabuli_Chr07*	9914785	(T/C)
CakSNP12240	*Kabuli*	*Ca_Kabuli_Chr07*	9914794	(G/A)
CakSNP12241	*Kabuli*	*Ca_Kabuli_Chr07*	9914737	(C/G)
CakSNP12242	*Kabuli*	*Ca_Kabuli_Chr07*	9914745	(T/C)
CakSNP12243	*Kabuli*	*Ca_Kabuli_Chr07*	9914856	(G/A)
CakSNP12244	*Kabuli*	*Ca_Kabuli_Chr07*	9914845	(A/C)
CakSNP12245	*Kabuli*	*Ca_Kabuli_Chr07*	9914832	(C/T)
CakSNP12246	*Kabuli*	*Ca_Kabuli_Chr07*	9914802	(T/G)
CakSNP12247	*Kabuli*	*Ca_Kabuli_Chr07*	9932369	(A/G)
CakSNP12248	*Kabuli*	*Ca_Kabuli_Chr07*	9936661	(C/T)
CakSNP12249	*Kabuli*	*Ca_Kabuli_Chr07*	9936854	(A/G)
CakSNP12250	*Kabuli*	*Ca_Kabuli_Chr07*	9938937	(T/C)
CakSNP12251	*Kabuli*	*Ca_Kabuli_Chr07*	10050020	(A/C)
CakSNP12252	*Kabuli*	*Ca_Kabuli_Chr07*	10049998	(T/A)
CakSNP12253	*Kabuli*	*Ca_Kabuli_Chr07*	10049996	(A/G)
CakSNP12254	*Kabuli*	*Ca_Kabuli_Chr07*	10089428	(T/C)
CakSNP12255	*Kabuli*	*Ca_Kabuli_Chr07*	10089539	(T/A)
CakSNP12256	*Kabuli*	*Ca_Kabuli_Chr07*	10106084	(C/G)
CakSNP12257	*Kabuli*	*Ca_Kabuli_Chr07*	10106065	(A/G)
CakSNP12258	*Kabuli*	*Ca_Kabuli_Chr07*	10106054	(G/C)

SNP IDs	Cultivars	Chromosomes/scaffolds	Physical positions (bp)	SNPs
CakSNP12259	*Kabuli*	*Ca_Kabuli_Chr07*	10137842	(A/G)
CakSNP12260	*Kabuli*	*Ca_Kabuli_Chr07*	10137890	(A/G)
CakSNP12261	*Kabuli*	*Ca_Kabuli_Chr07*	10181630	(G/T)
CakSNP12262	*Kabuli*	*Ca_Kabuli_Chr07*	10208029	(T/C)
CakSNP12263	*Kabuli*	*Ca_Kabuli_Chr07*	10216813	(G/A)
CakSNP12264	*Kabuli*	*Ca_Kabuli_Chr07*	10223046	(C/T)
CakSNP12265	*Kabuli*	*Ca_Kabuli_Chr07*	10223068	(G/T)
CakSNP12266	*Kabuli*	*Ca_Kabuli_Chr07*	10223069	(G/T)
CakSNP12267	*Kabuli*	*Ca_Kabuli_Chr07*	10223071	(T/A)
CakSNP12268	*Kabuli*	*Ca_Kabuli_Chr07*	10223074	(G/T)
CakSNP12269	*Kabuli*	*Ca_Kabuli_Chr07*	10223075	(C/T)
CakSNP12270	*Kabuli*	*Ca_Kabuli_Chr07*	10322726	(T/C)
CakSNP12271	*Kabuli*	*Ca_Kabuli_Chr07*	10340199	(T/C)
CakSNP12272	*Kabuli*	*Ca_Kabuli_Chr07*	10435983	(C/A)
CakSNP12273	*Kabuli*	*Ca_Kabuli_Chr07*	10465140	(G/A)
CakSNP12274	*Kabuli*	*Ca_Kabuli_Chr07*	10480401	(A/C)
CakSNP12275	*Kabuli*	*Ca_Kabuli_Chr07*	10480462	(A/G)
CakSNP12276	*Kabuli*	*Ca_Kabuli_Chr07*	10484200	(G/A)
CakSNP12277	*Kabuli*	*Ca_Kabuli_Chr07*	10484225	(C/A)
CakSNP12278	*Kabuli*	*Ca_Kabuli_Chr07*	10484196	(C/T)
CakSNP12279	*Kabuli*	*Ca_Kabuli_Chr07*	10485414	(C/T)
CakSNP12280	*Kabuli*	*Ca_Kabuli_Chr07*	10521423	(G/T)
CakSNP12281	*Kabuli*	*Ca_Kabuli_Chr07*	10521449	(C/A)
CakSNP12282	*Kabuli*	*Ca_Kabuli_Chr07*	10614159	(G/A)
CakSNP12283	*Kabuli*	*Ca_Kabuli_Chr07*	10658367	(A/T)
CakSNP12284	*Kabuli*	*Ca_Kabuli_Chr07*	10658446	(G/T)
CakSNP12285	*Kabuli*	*Ca_Kabuli_Chr07*	10688189	(T/G)
CakSNP12286	*Kabuli*	*Ca_Kabuli_Chr07*	10688401	(A/G)
CakSNP12287	*Kabuli*	*Ca_Kabuli_Chr07*	10688464	(G/A)
CakSNP12288	*Kabuli*	*Ca_Kabuli_Chr07*	10688434	(T/C)
CakSNP12289	*Kabuli*	*Ca_Kabuli_Chr07*	10688499	(T/C)
CakSNP12290	*Kabuli*	*Ca_Kabuli_Chr07*	10703569	(A/T)
CakSNP12291	*Kabuli*	*Ca_Kabuli_Chr07*	10703612	(A/C)
CakSNP12292	*Kabuli*	*Ca_Kabuli_Chr07*	10703704	(G/A)
CakSNP12293	*Kabuli*	*Ca_Kabuli_Chr07*	10703679	(T/C)
CakSNP12294	*Kabuli*	*Ca_Kabuli_Chr07*	10712202	(T/C)
CakSNP12295	*Kabuli*	*Ca_Kabuli_Chr07*	10712231	(G/A)
CakSNP12296	*Kabuli*	*Ca_Kabuli_Chr07*	10758973	(A/T)
CakSNP12297	*Kabuli*	*Ca_Kabuli_Chr07*	10759142	(T/G)
CakSNP12298	*Kabuli*	*Ca_Kabuli_Chr07*	10775881	(C/T)
CakSNP12299	*Kabuli*	*Ca_Kabuli_Chr07*	10775875	(A/G)

**SNP IDs**	**Cultivars**	**Chromosomes/scaffolds**	**Physical positions (bp)**	**SNPs**
CakSNP12300	*Kabuli*	*Ca_Kabuli_Chr07*	10775851	(C/T)
CakSNP12301	*Kabuli*	*Ca_Kabuli_Chr07*	10775860	(A/G)
CakSNP12302	*Kabuli*	*Ca_Kabuli_Chr07*	10780680	(C/G)
CakSNP12303	*Kabuli*	*Ca_Kabuli_Chr07*	10780682	(G/A)
CakSNP12304	*Kabuli*	*Ca_Kabuli_Chr07*	10780686	(T/C)
CakSNP12305	*Kabuli*	*Ca_Kabuli_Chr07*	10797204	(T/C)
CakSNP12306	*Kabuli*	*Ca_Kabuli_Chr07*	10812617	(G/T)
CakSNP12307	*Kabuli*	*Ca_Kabuli_Chr07*	10816523	(T/G)
CakSNP12308	*Kabuli*	*Ca_Kabuli_Chr07*	10816674	(C/T)
CakSNP12309	*Kabuli*	*Ca_Kabuli_Chr07*	10816662	(T/A)
CakSNP12310	*Kabuli*	*Ca_Kabuli_Chr07*	10859907	(G/A)
CakSNP12311	*Kabuli*	*Ca_Kabuli_Chr07*	10859842	(G/T)
CakSNP12312	*Kabuli*	*Ca_Kabuli_Chr07*	10900910	(T/C)
CakSNP12313	*Kabuli*	*Ca_Kabuli_Chr07*	11086047	(G/A)
CakSNP12314	*Kabuli*	*Ca_Kabuli_Chr07*	11086071	(A/T)
CakSNP12315	*Kabuli*	*Ca_Kabuli_Chr07*	11105847	(C/G)
CakSNP12316	*Kabuli*	*Ca_Kabuli_Chr07*	11105961	(A/C)
CakSNP12317	*Kabuli*	*Ca_Kabuli_Chr07*	11192667	(G/A)
CakSNP12318	*Kabuli*	*Ca_Kabuli_Chr07*	11192712	(C/T)
CakSNP12319	*Kabuli*	*Ca_Kabuli_Chr07*	11192703	(A/C)
CakSNP12320	*Kabuli*	*Ca_Kabuli_Chr07*	11261924	(G/A)
CakSNP12321	*Kabuli*	*Ca_Kabuli_Chr07*	11339511	(C/T)
CakSNP12322	*Kabuli*	*Ca_Kabuli_Chr07*	11393795	(A/G)
CakSNP12323	*Kabuli*	*Ca_Kabuli_Chr07*	11393986	(A/G)
CakSNP12324	*Kabuli*	*Ca_Kabuli_Chr07*	11473766	(A/G)
CakSNP12325	*Kabuli*	*Ca_Kabuli_Chr07*	11521694	(G/A)
CakSNP12326	*Kabuli*	*Ca_Kabuli_Chr07*	11527428	(G/A)
CakSNP12327	*Kabuli*	*Ca_Kabuli_Chr07*	11545846	(A/G)
CakSNP12328	*Kabuli*	*Ca_Kabuli_Chr07*	11568963	(T/A)
CakSNP12329	*Kabuli*	*Ca_Kabuli_Chr07*	11568945	(G/T)
CakSNP12330	*Kabuli*	*Ca_Kabuli_Chr07*	11590987	(A/C)
CakSNP12331	*Kabuli*	*Ca_Kabuli_Chr07*	11630509	(A/G)
CakSNP12332	*Kabuli*	*Ca_Kabuli_Chr07*	11630511	(C/T)
CakSNP12333	*Kabuli*	*Ca_Kabuli_Chr07*	11638885	(C/A)
CakSNP12334	*Kabuli*	*Ca_Kabuli_Chr07*	11639117	(C/G)
CakSNP12335	*Kabuli*	*Ca_Kabuli_Chr07*	11685818	(G/T)
CakSNP12336	*Kabuli*	*Ca_Kabuli_Chr07*	11685899	(A/G)
CakSNP12337	*Kabuli*	*Ca_Kabuli_Chr07*	11686238	(C/T)
CakSNP12338	*Kabuli*	*Ca_Kabuli_Chr07*	11725655	(G/A)
CakSNP12339	*Kabuli*	*Ca_Kabuli_Chr07*	11730926	(T/C)
CakSNP12340	*Kabuli*	*Ca_Kabuli_Chr07*	11730946	(A/C)

**SNP IDs**	**Cultivars**	**Chromosomes/scaffolds**	**Physical positions (bp)**	**SNPs**
CakSNP12341	*Kabuli*	*Ca_Kabuli_Chr07*	11735177	(T/C)
CakSNP12342	*Kabuli*	*Ca_Kabuli_Chr07*	11735381	(T/C)
CakSNP12343	*Kabuli*	*Ca_Kabuli_Chr07*	11735368	(G/C)
CakSNP12344	*Kabuli*	*Ca_Kabuli_Chr07*	11736006	(C/A)
CakSNP12345	*Kabuli*	*Ca_Kabuli_Chr07*	11792042	(G/A)
CakSNP12346	*Kabuli*	*Ca_Kabuli_Chr07*	11792126	(T/A)
CakSNP12347	*Kabuli*	*Ca_Kabuli_Chr07*	11792136	(G/A)
CakSNP12348	*Kabuli*	*Ca_Kabuli_Chr07*	11803091	(T/G)
CakSNP12349	*Kabuli*	*Ca_Kabuli_Chr07*	11812410	(G/A)
CakSNP12350	*Kabuli*	*Ca_Kabuli_Chr07*	11812441	(A/C)
CakSNP12351	*Kabuli*	*Ca_Kabuli_Chr07*	11858425	(C/T)
CakSNP12352	*Kabuli*	*Ca_Kabuli_Chr07*	11886954	(A/G)
CakSNP12353	*Kabuli*	*Ca_Kabuli_Chr07*	11902897	(A/G)
CakSNP12354	*Kabuli*	*Ca_Kabuli_Chr07*	11902985	(G/T)
CakSNP12355	*Kabuli*	*Ca_Kabuli_Chr07*	12010432	(G/A)
CakSNP12356	*Kabuli*	*Ca_Kabuli_Chr07*	12010471	(C/T)
CakSNP12357	*Kabuli*	*Ca_Kabuli_Chr07*	12010504	(G/A)
CakSNP12358	*Kabuli*	*Ca_Kabuli_Chr07*	12010499	(C/T)
CakSNP12359	*Kabuli*	*Ca_Kabuli_Chr07*	12010496	(G/A)
CakSNP12360	*Kabuli*	*Ca_Kabuli_Chr07*	12010494	(G/A)
CakSNP12361	*Kabuli*	*Ca_Kabuli_Chr07*	12010491	(G/T)
CakSNP12362	*Kabuli*	*Ca_Kabuli_Chr07*	12010483	(C/T)
CakSNP12363	*Kabuli*	*Ca_Kabuli_Chr07*	12010457	(C/T)
CakSNP12364	*Kabuli*	*Ca_Kabuli_Chr07*	12010544	(G/A)
CakSNP12365	*Kabuli*	*Ca_Kabuli_Chr07*	12010489	(C/T)
CakSNP12366	*Kabuli*	*Ca_Kabuli_Chr07*	12030080	(A/C)
CakSNP12367	*Kabuli*	*Ca_Kabuli_Chr07*	12071764	(T/C)
CakSNP12368	*Kabuli*	*Ca_Kabuli_Chr07*	12071773	(A/T)
CakSNP12369	*Kabuli*	*Ca_Kabuli_Chr07*	12203726	(T/G)
CakSNP12370	*Kabuli*	*Ca_Kabuli_Chr07*	12203722	(T/C)
CakSNP12371	*Kabuli*	*Ca_Kabuli_Chr07*	12203706	(T/C)
CakSNP12372	*Kabuli*	*Ca_Kabuli_Chr07*	12203690	(C/T)
CakSNP12373	*Kabuli*	*Ca_Kabuli_Chr07*	12244234	(C/T)
CakSNP12374	*Kabuli*	*Ca_Kabuli_Chr07*	12244259	(G/A)
CakSNP12375	*Kabuli*	*Ca_Kabuli_Chr07*	12284289	(T/A)
CakSNP12376	*Kabuli*	*Ca_Kabuli_Chr07*	12284304	(A/G)
CakSNP12377	*Kabuli*	*Ca_Kabuli_Chr07*	12385785	(C/T)
CakSNP12378	*Kabuli*	*Ca_Kabuli_Chr07*	12390774	(G/A)
CakSNP12379	*Kabuli*	*Ca_Kabuli_Chr07*	12481556	(G/C)
CakSNP12380	*Kabuli*	*Ca_Kabuli_Chr07*	12481627	(A/C)
CakSNP12381	*Kabuli*	*Ca_Kabuli_Chr07*	12481715	(A/G)

SNP IDs	Cultivars	Chromosomes/scaffolds	Physical positions (bp)	SNPs
CakSNP12382	*Kabuli*	*Ca_Kabuli_Chr07*	12481835	(C/T)
CakSNP12383	*Kabuli*	*Ca_Kabuli_Chr07*	12482851	(G/A)
CakSNP12384	*Kabuli*	*Ca_Kabuli_Chr07*	12482822	(A/G)
CakSNP12385	*Kabuli*	*Ca_Kabuli_Chr07*	12482953	(A/C)
CakSNP12386	*Kabuli*	*Ca_Kabuli_Chr07*	12483290	(A/C)
CakSNP12387	*Kabuli*	*Ca_Kabuli_Chr07*	12492075	(G/A)
CakSNP12388	*Kabuli*	*Ca_Kabuli_Chr07*	12492052	(C/T)
CakSNP12389	*Kabuli*	*Ca_Kabuli_Chr07*	12492046	(A/T)
CakSNP12390	*Kabuli*	*Ca_Kabuli_Chr07*	12616871	(T/C)
CakSNP12391	*Kabuli*	*Ca_Kabuli_Chr07*	12616870	(G/A)
CakSNP12392	*Kabuli*	*Ca_Kabuli_Chr07*	12616957	(C/T)
CakSNP12393	*Kabuli*	*Ca_Kabuli_Chr07*	12616937	(C/T)
CakSNP12394	*Kabuli*	*Ca_Kabuli_Chr07*	12623129	(T/C)
CakSNP12395	*Kabuli*	*Ca_Kabuli_Chr07*	12623128	(G/A)
CakSNP12396	*Kabuli*	*Ca_Kabuli_Chr07*	12623215	(C/T)
CakSNP12397	*Kabuli*	*Ca_Kabuli_Chr07*	12623195	(C/T)
CakSNP12398	*Kabuli*	*Ca_Kabuli_Chr07*	12628148	(A/G)
CakSNP12399	*Kabuli*	*Ca_Kabuli_Chr07*	12628211	(A/G)
CakSNP12400	*Kabuli*	*Ca_Kabuli_Chr07*	12628265	(T/A)
CakSNP12401	*Kabuli*	*Ca_Kabuli_Chr07*	12629986	(A/G)
CakSNP12402	*Kabuli*	*Ca_Kabuli_Chr07*	12633590	(A/C)
CakSNP12403	*Kabuli*	*Ca_Kabuli_Chr07*	12633642	(T/G)
CakSNP12404	*Kabuli*	*Ca_Kabuli_Chr07*	12633643	(A/T)
CakSNP12405	*Kabuli*	*Ca_Kabuli_Chr07*	12681440	(T/C)
CakSNP12406	*Kabuli*	*Ca_Kabuli_Chr07*	12718820	(C/G)
CakSNP12407	*Kabuli*	*Ca_Kabuli_Chr07*	12718853	(G/A)
CakSNP12408	*Kabuli*	*Ca_Kabuli_Chr07*	12730600	(C/A)
CakSNP12409	*Kabuli*	*Ca_Kabuli_Chr07*	12737516	(T/C)
CakSNP12410	*Kabuli*	*Ca_Kabuli_Chr07*	12893655	(C/T)
CakSNP12411	*Kabuli*	*Ca_Kabuli_Chr07*	12895182	(A/G)
CakSNP12412	*Kabuli*	*Ca_Kabuli_Chr07*	12898640	(A/C)
CakSNP12413	*Kabuli*	*Ca_Kabuli_Chr07*	12898709	(T/A)
CakSNP12414	*Kabuli*	*Ca_Kabuli_Chr07*	12898707	(G/A)
CakSNP12415	*Kabuli*	*Ca_Kabuli_Chr07*	12899398	(T/C)
CakSNP12416	*Kabuli*	*Ca_Kabuli_Chr07*	12913811	(T/A)
CakSNP12417	*Kabuli*	*Ca_Kabuli_Chr07*	13020788	(C/T)
CakSNP12418	*Kabuli*	*Ca_Kabuli_Chr07*	13020772	(A/G)
CakSNP12419	*Kabuli*	*Ca_Kabuli_Chr07*	13021047	(A/G)
CakSNP12420	*Kabuli*	*Ca_Kabuli_Chr07*	13021030	(A/G)
CakSNP12421	*Kabuli*	*Ca_Kabuli_Chr07*	13059710	(C/A)
CakSNP12422	*Kabuli*	*Ca_Kabuli_Chr07*	13059712	(C/A)

SNP IDs	Cultivars	Chromosomes/scaffolds	Physical positions (bp)	SNPs
CakSNP12423	*Kabuli*	*Ca_Kabuli_Chr07*	13059717	(T/A)
CakSNP12424	*Kabuli*	*Ca_Kabuli_Chr07*	13059852	(G/A)
CakSNP12425	*Kabuli*	*Ca_Kabuli_Chr07*	13172542	(A/G)
CakSNP12426	*Kabuli*	*Ca_Kabuli_Chr07*	13220439	(G/A)
CakSNP12427	*Kabuli*	*Ca_Kabuli_Chr07*	13220418	(A/G)
CakSNP12428	*Kabuli*	*Ca_Kabuli_Chr07*	13262430	(C/T)
CakSNP12429	*Kabuli*	*Ca_Kabuli_Chr07*	13281255	(T/C)
CakSNP12430	*Kabuli*	*Ca_Kabuli_Chr07*	13281290	(A/G)
CakSNP12431	*Kabuli*	*Ca_Kabuli_Chr07*	13281297	(C/G)
CakSNP12432	*Kabuli*	*Ca_Kabuli_Chr07*	13281307	(C/T)
CakSNP12433	*Kabuli*	*Ca_Kabuli_Chr07*	13281313	(T/C)
CakSNP12434	*Kabuli*	*Ca_Kabuli_Chr07*	13281314	(G/A)
CakSNP12435	*Kabuli*	*Ca_Kabuli_Chr07*	13281374	(G/T)
CakSNP12436	*Kabuli*	*Ca_Kabuli_Chr07*	13281351	(T/A)
CakSNP12437	*Kabuli*	*Ca_Kabuli_Chr07*	13288828	(G/A)
CakSNP12438	*Kabuli*	*Ca_Kabuli_Chr07*	13288813	(G/A)
CakSNP12439	*Kabuli*	*Ca_Kabuli_Chr07*	13288753	(A/C)
CakSNP12440	*Kabuli*	*Ca_Kabuli_Chr07*	13319871	(A/G)
CakSNP12441	*Kabuli*	*Ca_Kabuli_Chr07*	13514074	(C/T)
CakSNP12442	*Kabuli*	*Ca_Kabuli_Chr07*	13605200	(A/C)
CakSNP12443	*Kabuli*	*Ca_Kabuli_Chr07*	13605191	(A/C)
CakSNP12444	*Kabuli*	*Ca_Kabuli_Chr07*	13605188	(A/C)
CakSNP12445	*Kabuli*	*Ca_Kabuli_Chr07*	13605578	(C/G)
CakSNP12446	*Kabuli*	*Ca_Kabuli_Chr07*	13667102	(C/T)
CakSNP12447	*Kabuli*	*Ca_Kabuli_Chr07*	13667103	(T/G)
CakSNP12448	*Kabuli*	*Ca_Kabuli_Chr07*	13730350	(A/C)
CakSNP12449	*Kabuli*	*Ca_Kabuli_Chr07*	13757159	(A/C)
CakSNP12450	*Kabuli*	*Ca_Kabuli_Chr07*	13776110	(A/C)
CakSNP12451	*Kabuli*	*Ca_Kabuli_Chr07*	13781815	(T/C)
CakSNP12452	*Kabuli*	*Ca_Kabuli_Chr07*	13819387	(C/T)
CakSNP12453	*Kabuli*	*Ca_Kabuli_Chr07*	13881348	(G/A)
CakSNP12454	*Kabuli*	*Ca_Kabuli_Chr07*	13893199	(T/C)
CakSNP12455	*Kabuli*	*Ca_Kabuli_Chr07*	13893344	(G/C)
CakSNP12456	*Kabuli*	*Ca_Kabuli_Chr07*	13893382	(G/T)
CakSNP12457	*Kabuli*	*Ca_Kabuli_Chr07*	13893383	(C/T)
CakSNP12458	*Kabuli*	*Ca_Kabuli_Chr07*	13903980	(T/G)
CakSNP12459	*Kabuli*	*Ca_Kabuli_Chr07*	13904163	(T/C)
CakSNP12460	*Kabuli*	*Ca_Kabuli_Chr07*	13913735	(A/G)
CakSNP12461	*Kabuli*	*Ca_Kabuli_Chr07*	13921214	(C/T)
CakSNP12462	*Kabuli*	*Ca_Kabuli_Chr07*	13921289	(T/C)
CakSNP12463	*Kabuli*	*Ca_Kabuli_Chr07*	13949259	(A/G)

SNP IDs	Cultivars	Chromosomes/scaffolds	Physical positions (bp)	SNPs
CakSNP12464	*Kabuli*	*Ca_Kabuli_Chr07*	13949314	(G/A)
CakSNP12465	*Kabuli*	*Ca_Kabuli_Chr07*	13951874	(T/C)
CakSNP12466	*Kabuli*	*Ca_Kabuli_Chr07*	13951856	(T/C)
CakSNP12467	*Kabuli*	*Ca_Kabuli_Chr07*	14030136	(C/T)
CakSNP12468	*Kabuli*	*Ca_Kabuli_Chr07*	14051146	(G/T)
CakSNP12469	*Kabuli*	*Ca_Kabuli_Chr07*	14179629	(T/G)
CakSNP12470	*Kabuli*	*Ca_Kabuli_Chr07*	14179733	(C/A)
CakSNP12471	*Kabuli*	*Ca_Kabuli_Chr07*	14212570	(C/A)
CakSNP12472	*Kabuli*	*Ca_Kabuli_Chr07*	14212597	(C/G)
CakSNP12473	*Kabuli*	*Ca_Kabuli_Chr07*	14233343	(T/G)
CakSNP12474	*Kabuli*	*Ca_Kabuli_Chr07*	14233368	(T/C)
CakSNP12475	*Kabuli*	*Ca_Kabuli_Chr07*	14281993	(G/T)
CakSNP12476	*Kabuli*	*Ca_Kabuli_Chr07*	14282088	(T/C)
CakSNP12477	*Kabuli*	*Ca_Kabuli_Chr07*	14320216	(T/C)
CakSNP12478	*Kabuli*	*Ca_Kabuli_Chr07*	14328655	(T/C)
CakSNP12479	*Kabuli*	*Ca_Kabuli_Chr07*	14328649	(T/C)
CakSNP12480	*Kabuli*	*Ca_Kabuli_Chr07*	14329126	(C/T)
CakSNP12481	*Kabuli*	*Ca_Kabuli_Chr07*	14329175	(G/A)
CakSNP12482	*Kabuli*	*Ca_Kabuli_Chr07*	14329196	(G/A)
CakSNP12483	*Kabuli*	*Ca_Kabuli_Chr07*	14329217	(C/T)
CakSNP12484	*Kabuli*	*Ca_Kabuli_Chr07*	14329167	(A/T)
CakSNP12485	*Kabuli*	*Ca_Kabuli_Chr07*	14345827	(T/C)
CakSNP12486	*Kabuli*	*Ca_Kabuli_Chr07*	14345799	(T/A)
CakSNP12487	*Kabuli*	*Ca_Kabuli_Chr07*	14470549	(C/G)
CakSNP12488	*Kabuli*	*Ca_Kabuli_Chr07*	14518292	(A/T)
CakSNP12489	*Kabuli*	*Ca_Kabuli_Chr07*	14526546	(A/G)
CakSNP12490	*Kabuli*	*Ca_Kabuli_Chr07*	14526495	(T/C)
CakSNP12491	*Kabuli*	*Ca_Kabuli_Chr07*	14526493	(C/T)
CakSNP12492	*Kabuli*	*Ca_Kabuli_Chr07*	14526480	(T/C)
CakSNP12493	*Kabuli*	*Ca_Kabuli_Chr07*	14526478	(G/C)
CakSNP12494	*Kabuli*	*Ca_Kabuli_Chr07*	14558741	(C/A)
CakSNP12495	*Kabuli*	*Ca_Kabuli_Chr07*	14558785	(C/T)
CakSNP12496	*Kabuli*	*Ca_Kabuli_Chr07*	14559033	(G/A)
CakSNP12497	*Kabuli*	*Ca_Kabuli_Chr07*	14666535	(T/A)
CakSNP12498	*Kabuli*	*Ca_Kabuli_Chr07*	14668939	(G/A)
CakSNP12499	*Kabuli*	*Ca_Kabuli_Chr07*	14668922	(A/C)
CakSNP12500	*Kabuli*	*Ca_Kabuli_Chr07*	14685011	(G/C)
CakSNP12501	*Kabuli*	*Ca_Kabuli_Chr07*	14685017	(C/T)
CakSNP12502	*Kabuli*	*Ca_Kabuli_Chr07*	14685024	(A/G)
CakSNP12503	*Kabuli*	*Ca_Kabuli_Chr07*	14685034	(T/G)
CakSNP12504	*Kabuli*	*Ca_Kabuli_Chr07*	14712518	(C/A)

SNP IDs	Cultivars	Chromosomes/scaffolds	Physical positions (bp)	SNPs
CakSNP12505	*Kabuli*	*Ca_Kabuli_Chr07*	14712568	(C/A)
CakSNP12506	*Kabuli*	*Ca_Kabuli_Chr07*	14712596	(T/C)
CakSNP12507	*Kabuli*	*Ca_Kabuli_Chr07*	14712601	(C/T)
CakSNP12508	*Kabuli*	*Ca_Kabuli_Chr07*	14748257	(T/A)
CakSNP12509	*Kabuli*	*Ca_Kabuli_Chr07*	14775135	(C/G)
CakSNP12510	*Kabuli*	*Ca_Kabuli_Chr07*	14782453	(A/G)
CakSNP12511	*Kabuli*	*Ca_Kabuli_Chr07*	14899028	(C/A)
CakSNP12512	*Kabuli*	*Ca_Kabuli_Chr07*	14899101	(T/C)
CakSNP12513	*Kabuli*	*Ca_Kabuli_Chr07*	14933294	(T/C)
CakSNP12514	*Kabuli*	*Ca_Kabuli_Chr07*	14933286	(G/A)
CakSNP12515	*Kabuli*	*Ca_Kabuli_Chr07*	14983364	(T/G)
CakSNP12516	*Kabuli*	*Ca_Kabuli_Chr07*	14997794	(G/A)
CakSNP12517	*Kabuli*	*Ca_Kabuli_Chr07*	14997883	(T/C)
CakSNP12518	*Kabuli*	*Ca_Kabuli_Chr07*	15028182	(T/G)
CakSNP12519	*Kabuli*	*Ca_Kabuli_Chr07*	15039606	(T/A)
CakSNP12520	*Kabuli*	*Ca_Kabuli_Chr07*	15069817	(G/A)
CakSNP12521	*Kabuli*	*Ca_Kabuli_Chr07*	15070091	(T/A)
CakSNP12522	*Kabuli*	*Ca_Kabuli_Chr07*	15119205	(T/C)
CakSNP12523	*Kabuli*	*Ca_Kabuli_Chr07*	15125638	(G/C)
CakSNP12524	*Kabuli*	*Ca_Kabuli_Chr07*	15125619	(T/G)
CakSNP12525	*Kabuli*	*Ca_Kabuli_Chr07*	15185603	(A/G)
CakSNP12526	*Kabuli*	*Ca_Kabuli_Chr07*	15201600	(A/G)
CakSNP12527	*Kabuli*	*Ca_Kabuli_Chr07*	15399421	(T/C)
CakSNP12528	*Kabuli*	*Ca_Kabuli_Chr07*	15441316	(A/T)
CakSNP12529	*Kabuli*	*Ca_Kabuli_Chr07*	15463183	(G/T)
CakSNP12530	*Kabuli*	*Ca_Kabuli_Chr07*	15463170	(G/A)
CakSNP12531	*Kabuli*	*Ca_Kabuli_Chr07*	15463169	(G/A)
CakSNP12532	*Kabuli*	*Ca_Kabuli_Chr07*	15530329	(G/T)
CakSNP12533	*Kabuli*	*Ca_Kabuli_Chr07*	15530316	(G/A)
CakSNP12534	*Kabuli*	*Ca_Kabuli_Chr07*	15530315	(G/A)
CakSNP12535	*Kabuli*	*Ca_Kabuli_Chr07*	15568060	(G/T)
CakSNP12536	*Kabuli*	*Ca_Kabuli_Chr07*	15568047	(G/A)
CakSNP12537	*Kabuli*	*Ca_Kabuli_Chr07*	15568046	(G/A)
CakSNP12538	*Kabuli*	*Ca_Kabuli_Chr07*	15622247	(G/A)
CakSNP12539	*Kabuli*	*Ca_Kabuli_Chr07*	15622245	(T/A)
CakSNP12540	*Kabuli*	*Ca_Kabuli_Chr07*	15762068	(A/T)
CakSNP12541	*Kabuli*	*Ca_Kabuli_Chr07*	15826588	(A/C)
CakSNP12542	*Kabuli*	*Ca_Kabuli_Chr07*	15856789	(A/G)
CakSNP12543	*Kabuli*	*Ca_Kabuli_Chr07*	15856955	(T/G)
CakSNP12544	*Kabuli*	*Ca_Kabuli_Chr07*	15860298	(T/C)
CakSNP12545	*Kabuli*	*Ca_Kabuli_Chr07*	15887790	(T/C)

SNP IDs	Cultivars	Chromosomes/scaffolds	Physical positions (bp)	SNPs
CakSNP12546	*Kabuli*	*Ca_Kabuli_Chr07*	15966680	(A/G)
CakSNP12547	*Kabuli*	*Ca_Kabuli_Chr07*	15966714	(G/A)
CakSNP12548	*Kabuli*	*Ca_Kabuli_Chr07*	16008746	(T/C)
CakSNP12549	*Kabuli*	*Ca_Kabuli_Chr07*	16008733	(A/G)
CakSNP12550	*Kabuli*	*Ca_Kabuli_Chr07*	16289811	(C/T)
CakSNP12551	*Kabuli*	*Ca_Kabuli_Chr07*	16289802	(C/T)
CakSNP12552	*Kabuli*	*Ca_Kabuli_Chr07*	16289794	(G/T)
CakSNP12553	*Kabuli*	*Ca_Kabuli_Chr07*	16324985	(T/C)
CakSNP12554	*Kabuli*	*Ca_Kabuli_Chr07*	16331943	(A/G)
CakSNP12555	*Kabuli*	*Ca_Kabuli_Chr07*	16381854	(T/C)
CakSNP12556	*Kabuli*	*Ca_Kabuli_Chr07*	16381891	(T/G)
CakSNP12557	*Kabuli*	*Ca_Kabuli_Chr07*	16381934	(T/C)
CakSNP12558	*Kabuli*	*Ca_Kabuli_Chr07*	16466619	(T/C)
CakSNP12559	*Kabuli*	*Ca_Kabuli_Chr07*	16581695	(C/G)
CakSNP12560	*Kabuli*	*Ca_Kabuli_Chr07*	16626047	(T/G)
CakSNP12561	*Kabuli*	*Ca_Kabuli_Chr07*	16626703	(A/G)
CakSNP12562	*Kabuli*	*Ca_Kabuli_Chr07*	16665577	(A/G)
CakSNP12563	*Kabuli*	*Ca_Kabuli_Chr07*	16735437	(G/A)
CakSNP12564	*Kabuli*	*Ca_Kabuli_Chr07*	16735427	(A/G)
CakSNP12565	*Kabuli*	*Ca_Kabuli_Chr07*	16735423	(G/C)
CakSNP12566	*Kabuli*	*Ca_Kabuli_Chr07*	16816296	(G/C)
CakSNP12567	*Kabuli*	*Ca_Kabuli_Chr07*	16821274	(A/C)
CakSNP12568	*Kabuli*	*Ca_Kabuli_Chr07*	16829081	(C/A)
CakSNP12569	*Kabuli*	*Ca_Kabuli_Chr07*	16897329	(A/C)
CakSNP12570	*Kabuli*	*Ca_Kabuli_Chr07*	16897285	(T/C)
CakSNP12571	*Kabuli*	*Ca_Kabuli_Chr07*	16936123	(C/G)
CakSNP12572	*Kabuli*	*Ca_Kabuli_Chr07*	17017466	(C/G)
CakSNP12573	*Kabuli*	*Ca_Kabuli_Chr07*	17017453	(A/G)
CakSNP12574	*Kabuli*	*Ca_Kabuli_Chr07*	17017447	(A/G)
CakSNP12575	*Kabuli*	*Ca_Kabuli_Chr07*	17017438	(A/T)
CakSNP12576	*Kabuli*	*Ca_Kabuli_Chr07*	17017421	(G/A)
CakSNP12577	*Kabuli*	*Ca_Kabuli_Chr07*	17017418	(T/G)
CakSNP12578	*Kabuli*	*Ca_Kabuli_Chr07*	17038317	(G/A)
CakSNP12579	*Kabuli*	*Ca_Kabuli_Chr07*	17064968	(T/C)
CakSNP12580	*Kabuli*	*Ca_Kabuli_Chr07*	17088603	(A/G)
CakSNP12581	*Kabuli*	*Ca_Kabuli_Chr07*	17140664	(C/T)
CakSNP12582	*Kabuli*	*Ca_Kabuli_Chr07*	17178847	(A/C)
CakSNP12583	*Kabuli*	*Ca_Kabuli_Chr07*	17181160	(G/C)
CakSNP12584	*Kabuli*	*Ca_Kabuli_Chr07*	17228904	(T/G)
CakSNP12585	*Kabuli*	*Ca_Kabuli_Chr07*	17318174	(G/T)
CakSNP12586	*Kabuli*	*Ca_Kabuli_Chr07*	17539110	(G/A)

SNP IDs	Cultivars	Chromosomes/scaffolds	Physical positions (bp)	SNPs
CakSNP12587	*Kabuli*	*Ca_Kabuli_Chr07*	17539136	(C/A)
CakSNP12588	*Kabuli*	*Ca_Kabuli_Chr07*	17539207	(G/A)
CakSNP12589	*Kabuli*	*Ca_Kabuli_Chr07*	17586154	(C/T)
CakSNP12590	*Kabuli*	*Ca_Kabuli_Chr07*	17591798	(T/A)
CakSNP12591	*Kabuli*	*Ca_Kabuli_Chr07*	17591781	(G/A)
CakSNP12592	*Kabuli*	*Ca_Kabuli_Chr07*	17591843	(G/T)
CakSNP12593	*Kabuli*	*Ca_Kabuli_Chr07*	17591974	(A/G)
CakSNP12594	*Kabuli*	*Ca_Kabuli_Chr07*	17591971	(A/G)
CakSNP12595	*Kabuli*	*Ca_Kabuli_Chr07*	17591924	(T/C)
CakSNP12596	*Kabuli*	*Ca_Kabuli_Chr07*	17592701	(C/A)
CakSNP12597	*Kabuli*	*Ca_Kabuli_Chr07*	17597348	(T/C)
CakSNP12598	*Kabuli*	*Ca_Kabuli_Chr07*	17597421	(A/G)
CakSNP12599	*Kabuli*	*Ca_Kabuli_Chr07*	17597420	(C/T)
CakSNP12600	*Kabuli*	*Ca_Kabuli_Chr07*	17624143	(G/A)
CakSNP12601	*Kabuli*	*Ca_Kabuli_Chr07*	17624225	(T/C)
CakSNP12602	*Kabuli*	*Ca_Kabuli_Chr07*	17766520	(T/C)
CakSNP12603	*Kabuli*	*Ca_Kabuli_Chr07*	17766952	(T/A)
CakSNP12604	*Kabuli*	*Ca_Kabuli_Chr07*	17766914	(C/A)
CakSNP12605	*Kabuli*	*Ca_Kabuli_Chr07*	17779414	(A/G)
CakSNP12606	*Kabuli*	*Ca_Kabuli_Chr07*	17779594	(T/C)
CakSNP12607	*Kabuli*	*Ca_Kabuli_Chr07*	17779700	(A/T)
CakSNP12608	*Kabuli*	*Ca_Kabuli_Chr07*	17782558	(C/T)
CakSNP12609	*Kabuli*	*Ca_Kabuli_Chr07*	18011604	(G/C)
CakSNP12610	*Kabuli*	*Ca_Kabuli_Chr07*	18090289	(T/A)
CakSNP12611	*Kabuli*	*Ca_Kabuli_Chr07*	18265803	(A/C)
CakSNP12612	*Kabuli*	*Ca_Kabuli_Chr07*	18265791	(G/C)
CakSNP12613	*Kabuli*	*Ca_Kabuli_Chr07*	18265782	(G/C)
CakSNP12614	*Kabuli*	*Ca_Kabuli_Chr07*	18319049	(T/G)
CakSNP12615	*Kabuli*	*Ca_Kabuli_Chr07*	18319073	(T/G)
CakSNP12616	*Kabuli*	*Ca_Kabuli_Chr07*	18365218	(G/A)
CakSNP12617	*Kabuli*	*Ca_Kabuli_Chr07*	18430468	(C/T)
CakSNP12618	*Kabuli*	*Ca_Kabuli_Chr07*	18469372	(G/A)
CakSNP12619	*Kabuli*	*Ca_Kabuli_Chr07*	18469422	(C/T)
CakSNP12620	*Kabuli*	*Ca_Kabuli_Chr07*	18638565	(A/G)
CakSNP12621	*Kabuli*	*Ca_Kabuli_Chr07*	18642538	(A/G)
CakSNP12622	*Kabuli*	*Ca_Kabuli_Chr07*	18658563	(C/G)
CakSNP12623	*Kabuli*	*Ca_Kabuli_Chr07*	18696645	(G/A)
CakSNP12624	*Kabuli*	*Ca_Kabuli_Chr07*	18796748	(T/C)
CakSNP12625	*Kabuli*	*Ca_Kabuli_Chr07*	18796768	(A/G)
CakSNP12626	*Kabuli*	*Ca_Kabuli_Chr07*	18910468	(A/G)
CakSNP12627	*Kabuli*	*Ca_Kabuli_Chr07*	19042183	(C/T)

SNP IDs	Cultivars	Chromosomes/scaffolds	Physical positions (bp)	SNPs
CakSNP12628	*Kabuli*	*Ca_Kabuli_Chr07*	19189737	(A/C)
CakSNP12629	*Kabuli*	*Ca_Kabuli_Chr07*	19217501	(A/C)
CakSNP12630	*Kabuli*	*Ca_Kabuli_Chr07*	19217598	(G/C)
CakSNP12631	*Kabuli*	*Ca_Kabuli_Chr07*	19232818	(T/A)
CakSNP12632	*Kabuli*	*Ca_Kabuli_Chr07*	19290521	(G/A)
CakSNP12633	*Kabuli*	*Ca_Kabuli_Chr07*	19470527	(T/C)
CakSNP12634	*Kabuli*	*Ca_Kabuli_Chr07*	19472719	(G/A)
CakSNP12635	*Kabuli*	*Ca_Kabuli_Chr07*	19472689	(T/A)
CakSNP12636	*Kabuli*	*Ca_Kabuli_Chr07*	19542901	(A/G)
CakSNP12637	*Kabuli*	*Ca_Kabuli_Chr07*	19549636	(C/T)
CakSNP12638	*Kabuli*	*Ca_Kabuli_Chr07*	19707812	(T/A)
CakSNP12639	*Kabuli*	*Ca_Kabuli_Chr07*	19707842	(G/A)
CakSNP12640	*Kabuli*	*Ca_Kabuli_Chr07*	19818789	(C/A)
CakSNP12641	*Kabuli*	*Ca_Kabuli_Chr07*	19818770	(T/C)
CakSNP12642	*Kabuli*	*Ca_Kabuli_Chr07*	19818759	(G/C)
CakSNP12643	*Kabuli*	*Ca_Kabuli_Chr07*	19818757	(G/C)
CakSNP12644	*Kabuli*	*Ca_Kabuli_Chr07*	20031805	(T/C)
CakSNP12645	*Kabuli*	*Ca_Kabuli_Chr07*	20032205	(A/C)
CakSNP12646	*Kabuli*	*Ca_Kabuli_Chr07*	20082842	(T/C)
CakSNP12647	*Kabuli*	*Ca_Kabuli_Chr07*	20083040	(A/T)
CakSNP12648	*Kabuli*	*Ca_Kabuli_Chr07*	20099384	(C/T)
CakSNP12649	*Kabuli*	*Ca_Kabuli_Chr07*	20111019	(T/C)
CakSNP12650	*Kabuli*	*Ca_Kabuli_Chr07*	20111123	(T/C)
CakSNP12651	*Kabuli*	*Ca_Kabuli_Chr07*	20112897	(G/A)
CakSNP12652	*Kabuli*	*Ca_Kabuli_Chr07*	20135647	(C/G)
CakSNP12653	*Kabuli*	*Ca_Kabuli_Chr07*	20168870	(T/A)
CakSNP12654	*Kabuli*	*Ca_Kabuli_Chr07*	20252133	(T/C)
CakSNP12655	*Kabuli*	*Ca_Kabuli_Chr07*	20252134	(G/A)
CakSNP12656	*Kabuli*	*Ca_Kabuli_Chr07*	20252135	(A/G)
CakSNP12657	*Kabuli*	*Ca_Kabuli_Chr07*	20252144	(G/A)
CakSNP12658	*Kabuli*	*Ca_Kabuli_Chr07*	20252145	(A/G)
CakSNP12659	*Kabuli*	*Ca_Kabuli_Chr07*	20252181	(C/T)
CakSNP12660	*Kabuli*	*Ca_Kabuli_Chr07*	20252192	(G/A)
CakSNP12661	*Kabuli*	*Ca_Kabuli_Chr07*	20252201	(C/A)
CakSNP12662	*Kabuli*	*Ca_Kabuli_Chr07*	20252212	(C/T)
CakSNP12663	*Kabuli*	*Ca_Kabuli_Chr07*	20252214	(C/G)
CakSNP12664	*Kabuli*	*Ca_Kabuli_Chr07*	20252236	(G/A)
CakSNP12665	*Kabuli*	*Ca_Kabuli_Chr07*	20252234	(A/C)
CakSNP12666	*Kabuli*	*Ca_Kabuli_Chr07*	20252233	(C/T)
CakSNP12667	*Kabuli*	*Ca_Kabuli_Chr07*	20252221	(T/C)
CakSNP12668	*Kabuli*	*Ca_Kabuli_Chr07*	20252209	(G/A)

SNP IDs	Cultivars	Chromosomes/scaffolds	Physical positions (bp)	SNPs
CakSNP12669	*Kabuli*	*Ca_Kabuli_Chr07*	20252309	(C/T)
CakSNP12670	*Kabuli*	*Ca_Kabuli_Chr07*	20252334	(A/T)
CakSNP12671	*Kabuli*	*Ca_Kabuli_Chr07*	20252340	(A/G)
CakSNP12672	*Kabuli*	*Ca_Kabuli_Chr07*	20362883	(G/A)
CakSNP12673	*Kabuli*	*Ca_Kabuli_Chr07*	20362879	(C/T)
CakSNP12674	*Kabuli*	*Ca_Kabuli_Chr07*	20362876	(G/A)
CakSNP12675	*Kabuli*	*Ca_Kabuli_Chr07*	20362838	(T/C)
CakSNP12676	*Kabuli*	*Ca_Kabuli_Chr07*	20414653	(G/A)
CakSNP12677	*Kabuli*	*Ca_Kabuli_Chr07*	20414703	(C/A)
CakSNP12678	*Kabuli*	*Ca_Kabuli_Chr07*	20486247	(T/G)
CakSNP12679	*Kabuli*	*Ca_Kabuli_Chr07*	20486280	(G/C)
CakSNP12680	*Kabuli*	*Ca_Kabuli_Chr07*	20496378	(G/A)
CakSNP12681	*Kabuli*	*Ca_Kabuli_Chr07*	20496303	(C/T)
CakSNP12682	*Kabuli*	*Ca_Kabuli_Chr07*	20496438	(C/T)
CakSNP12683	*Kabuli*	*Ca_Kabuli_Chr07*	20498552	(T/C)
CakSNP12684	*Kabuli*	*Ca_Kabuli_Chr07*	20498626	(T/G)
CakSNP12685	*Kabuli*	*Ca_Kabuli_Chr07*	20540780	(C/T)
CakSNP12686	*Kabuli*	*Ca_Kabuli_Chr07*	20540791	(G/A)
CakSNP12687	*Kabuli*	*Ca_Kabuli_Chr07*	20543119	(T/C)
CakSNP12688	*Kabuli*	*Ca_Kabuli_Chr07*	20550563	(G/A)
CakSNP12689	*Kabuli*	*Ca_Kabuli_Chr07*	20550575	(C/G)
CakSNP12690	*Kabuli*	*Ca_Kabuli_Chr07*	20552041	(G/A)
CakSNP12691	*Kabuli*	*Ca_Kabuli_Chr07*	20552053	(C/G)
CakSNP12692	*Kabuli*	*Ca_Kabuli_Chr07*	20556466	(G/C)
CakSNP12693	*Kabuli*	*Ca_Kabuli_Chr07*	20556576	(C/T)
CakSNP12694	*Kabuli*	*Ca_Kabuli_Chr07*	20563931	(G/A)
CakSNP12695	*Kabuli*	*Ca_Kabuli_Chr07*	20564024	(G/A)
CakSNP12696	*Kabuli*	*Ca_Kabuli_Chr07*	20564266	(G/A)
CakSNP12697	*Kabuli*	*Ca_Kabuli_Chr07*	20564269	(C/T)
CakSNP12698	*Kabuli*	*Ca_Kabuli_Chr07*	20564297	(A/G)
CakSNP12699	*Kabuli*	*Ca_Kabuli_Chr07*	20579963	(G/T)
CakSNP12700	*Kabuli*	*Ca_Kabuli_Chr07*	20579974	(A/T)
CakSNP12701	*Kabuli*	*Ca_Kabuli_Chr07*	20585997	(T/G)
CakSNP12702	*Kabuli*	*Ca_Kabuli_Chr07*	20605389	(G/A)
CakSNP12703	*Kabuli*	*Ca_Kabuli_Chr07*	20605384	(C/G)
CakSNP12704	*Kabuli*	*Ca_Kabuli_Chr07*	20605373	(C/A)
CakSNP12705	*Kabuli*	*Ca_Kabuli_Chr07*	20633040	(A/G)
CakSNP12706	*Kabuli*	*Ca_Kabuli_Chr07*	20637152	(G/A)
CakSNP12707	*Kabuli*	*Ca_Kabuli_Chr07*	20917747	(A/G)
CakSNP12708	*Kabuli*	*Ca_Kabuli_Chr07*	20917743	(T/G)
CakSNP12709	*Kabuli*	*Ca_Kabuli_Chr07*	20917740	(A/G)

**SNP IDs**	**Cultivars**	**Chromosomes/scaffolds**	**Physical positions (bp)**	**SNPs**
CakSNP12710	*Kabuli*	*Ca_Kabuli_Chr07*	21073385	(A/C)
CakSNP12711	*Kabuli*	*Ca_Kabuli_Chr07*	21081969	(A/G)
CakSNP12712	*Kabuli*	*Ca_Kabuli_Chr07*	21099782	(G/T)
CakSNP12713	*Kabuli*	*Ca_Kabuli_Chr07*	21402075	(A/G)
CakSNP12714	*Kabuli*	*Ca_Kabuli_Chr07*	21402117	(A/C)
CakSNP12715	*Kabuli*	*Ca_Kabuli_Chr07*	21595525	(C/T)
CakSNP12716	*Kabuli*	*Ca_Kabuli_Chr07*	21595527	(T/G)
CakSNP12717	*Kabuli*	*Ca_Kabuli_Chr07*	21616285	(A/G)
CakSNP12718	*Kabuli*	*Ca_Kabuli_Chr07*	21616287	(T/G)
CakSNP12719	*Kabuli*	*Ca_Kabuli_Chr07*	21616294	(T/G)
CakSNP12720	*Kabuli*	*Ca_Kabuli_Chr07*	21626752	(T/C)
CakSNP12721	*Kabuli*	*Ca_Kabuli_Chr07*	21633103	(C/G)
CakSNP12722	*Kabuli*	*Ca_Kabuli_Chr07*	21639234	(G/A)
CakSNP12723	*Kabuli*	*Ca_Kabuli_Chr07*	21639238	(G/A)
CakSNP12724	*Kabuli*	*Ca_Kabuli_Chr07*	21639244	(T/G)
CakSNP12725	*Kabuli*	*Ca_Kabuli_Chr07*	21639251	(T/G)
CakSNP12726	*Kabuli*	*Ca_Kabuli_Chr07*	21639254	(T/G)
CakSNP12727	*Kabuli*	*Ca_Kabuli_Chr07*	21639375	(T/G)
CakSNP12728	*Kabuli*	*Ca_Kabuli_Chr07*	21644279	(C/T)
CakSNP12729	*Kabuli*	*Ca_Kabuli_Chr07*	21644342	(G/A)
CakSNP12730	*Kabuli*	*Ca_Kabuli_Chr07*	21713147	(T/G)
CakSNP12731	*Kabuli*	*Ca_Kabuli_Chr07*	21744514	(T/C)
CakSNP12732	*Kabuli*	*Ca_Kabuli_Chr07*	21918052	(G/C)
CakSNP12733	*Kabuli*	*Ca_Kabuli_Chr07*	22707297	(T/C)
CakSNP12734	*Kabuli*	*Ca_Kabuli_Chr07*	22721356	(A/C)
CakSNP12735	*Kabuli*	*Ca_Kabuli_Chr07*	22738152	(C/G)
CakSNP12736	*Kabuli*	*Ca_Kabuli_Chr07*	22738307	(G/C)
CakSNP12737	*Kabuli*	*Ca_Kabuli_Chr07*	22738482	(A/G)
CakSNP12738	*Kabuli*	*Ca_Kabuli_Chr07*	22808696	(A/G)
CakSNP12739	*Kabuli*	*Ca_Kabuli_Chr07*	22808695	(C/T)
CakSNP12740	*Kabuli*	*Ca_Kabuli_Chr07*	22808687	(C/T)
CakSNP12741	*Kabuli*	*Ca_Kabuli_Chr07*	22808717	(C/T)
CakSNP12742	*Kabuli*	*Ca_Kabuli_Chr07*	22808679	(A/C)
CakSNP12743	*Kabuli*	*Ca_Kabuli_Chr07*	22839776	(G/A)
CakSNP12744	*Kabuli*	*Ca_Kabuli_Chr07*	22875652	(A/G)
CakSNP12745	*Kabuli*	*Ca_Kabuli_Chr07*	23087696	(T/C)
CakSNP12746	*Kabuli*	*Ca_Kabuli_Chr07*	23087732	(T/C)
CakSNP12747	*Kabuli*	*Ca_Kabuli_Chr07*	23439214	(A/G)
CakSNP12748	*Kabuli*	*Ca_Kabuli_Chr07*	23439225	(C/A)
CakSNP12749	*Kabuli*	*Ca_Kabuli_Chr07*	23574823	(C/T)
CakSNP12750	*Kabuli*	*Ca_Kabuli_Chr07*	23575629	(G/A)

SNP IDs	Cultivars	Chromosomes/scaffolds	Physical positions (bp)	SNPs
CakSNP12751	*Kabuli*	*Ca_Kabuli_Chr07*	23616050	(A/G)
CakSNP12752	*Kabuli*	*Ca_Kabuli_Chr07*	24021690	(T/A)
CakSNP12753	*Kabuli*	*Ca_Kabuli_Chr07*	24181321	(G/A)
CakSNP12754	*Kabuli*	*Ca_Kabuli_Chr07*	24190633	(G/A)
CakSNP12755	*Kabuli*	*Ca_Kabuli_Chr07*	24190636	(G/A)
CakSNP12756	*Kabuli*	*Ca_Kabuli_Chr07*	24190645	(A/T)
CakSNP12757	*Kabuli*	*Ca_Kabuli_Chr07*	24190650	(G/A)
CakSNP12758	*Kabuli*	*Ca_Kabuli_Chr07*	24190671	(C/T)
CakSNP12759	*Kabuli*	*Ca_Kabuli_Chr07*	24245096	(C/T)
CakSNP12760	*Kabuli*	*Ca_Kabuli_Chr07*	24415819	(T/A)
CakSNP12761	*Kabuli*	*Ca_Kabuli_Chr07*	25218407	(C/T)
CakSNP12762	*Kabuli*	*Ca_Kabuli_Chr07*	25362272	(T/C)
CakSNP12763	*Kabuli*	*Ca_Kabuli_Chr07*	25428518	(T/C)
CakSNP12764	*Kabuli*	*Ca_Kabuli_Chr07*	25428525	(C/G)
CakSNP12765	*Kabuli*	*Ca_Kabuli_Chr07*	25428548	(G/T)
CakSNP12766	*Kabuli*	*Ca_Kabuli_Chr07*	25428566	(T/C)
CakSNP12767	*Kabuli*	*Ca_Kabuli_Chr07*	25428660	(G/A)
CakSNP12768	*Kabuli*	*Ca_Kabuli_Chr07*	25569665	(T/G)
CakSNP12769	*Kabuli*	*Ca_Kabuli_Chr07*	25569657	(T/C)
CakSNP12770	*Kabuli*	*Ca_Kabuli_Chr07*	25740866	(G/T)
CakSNP12771	*Kabuli*	*Ca_Kabuli_Chr07*	26070513	(G/T)
CakSNP12772	*Kabuli*	*Ca_Kabuli_Chr07*	26070515	(A/G)
CakSNP12773	*Kabuli*	*Ca_Kabuli_Chr07*	26094578	(T/C)
CakSNP12774	*Kabuli*	*Ca_Kabuli_Chr07*	26094572	(C/T)
CakSNP12775	*Kabuli*	*Ca_Kabuli_Chr07*	26094562	(G/T)
CakSNP12776	*Kabuli*	*Ca_Kabuli_Chr07*	26094567	(G/A)
CakSNP12777	*Kabuli*	*Ca_Kabuli_Chr07*	26094609	(C/T)
CakSNP12778	*Kabuli*	*Ca_Kabuli_Chr07*	26137315	(T/C)
CakSNP12779	*Kabuli*	*Ca_Kabuli_Chr07*	26146779	(C/T)
CakSNP12780	*Kabuli*	*Ca_Kabuli_Chr07*	26226146	(G/C)
CakSNP12781	*Kabuli*	*Ca_Kabuli_Chr07*	26487440	(A/C)
CakSNP12782	*Kabuli*	*Ca_Kabuli_Chr07*	26692131	(T/G)
CakSNP12783	*Kabuli*	*Ca_Kabuli_Chr07*	26692130	(C/T)
CakSNP12784	*Kabuli*	*Ca_Kabuli_Chr07*	26692128	(A/C)
CakSNP12785	*Kabuli*	*Ca_Kabuli_Chr07*	26692194	(C/A)
CakSNP12786	*Kabuli*	*Ca_Kabuli_Chr07*	26692195	(G/T)
CakSNP12787	*Kabuli*	*Ca_Kabuli_Chr07*	26694365	(G/A)
CakSNP12788	*Kabuli*	*Ca_Kabuli_Chr07*	26694440	(C/A)
CakSNP12789	*Kabuli*	*Ca_Kabuli_Chr07*	26694441	(G/A)
CakSNP12790	*Kabuli*	*Ca_Kabuli_Chr07*	26897878	(A/G)
CakSNP12791	*Kabuli*	*Ca_Kabuli_Chr07*	26897910	(A/G)

SNP IDs	Cultivars	Chromosomes/scaffolds	Physical positions (bp)	SNPs
CakSNP12792	*Kabuli*	*Ca_Kabuli_Chr07*	26897920	(A/G)
CakSNP12793	*Kabuli*	*Ca_Kabuli_Chr07*	26897923	(G/A)
CakSNP12794	*Kabuli*	*Ca_Kabuli_Chr07*	26897929	(T/C)
CakSNP12795	*Kabuli*	*Ca_Kabuli_Chr07*	26897940	(A/G)
CakSNP12796	*Kabuli*	*Ca_Kabuli_Chr07*	26897948	(A/T)
CakSNP12797	*Kabuli*	*Ca_Kabuli_Chr07*	26940917	(G/A)
CakSNP12798	*Kabuli*	*Ca_Kabuli_Chr07*	26940967	(A/G)
CakSNP12799	*Kabuli*	*Ca_Kabuli_Chr07*	26940979	(A/G)
CakSNP12800	*Kabuli*	*Ca_Kabuli_Chr07*	26940985	(T/G)
CakSNP12801	*Kabuli*	*Ca_Kabuli_Chr07*	27050259	(C/G)
CakSNP12802	*Kabuli*	*Ca_Kabuli_Chr07*	27176481	(C/A)
CakSNP12803	*Kabuli*	*Ca_Kabuli_Chr07*	27210919	(C/T)
CakSNP12804	*Kabuli*	*Ca_Kabuli_Chr07*	27210881	(C/T)
CakSNP12805	*Kabuli*	*Ca_Kabuli_Chr07*	27210871	(C/A)
CakSNP12806	*Kabuli*	*Ca_Kabuli_Chr07*	27210865	(T/C)
CakSNP12807	*Kabuli*	*Ca_Kabuli_Chr07*	27210858	(C/T)
CakSNP12808	*Kabuli*	*Ca_Kabuli_Chr07*	27210853	(A/G)
CakSNP12809	*Kabuli*	*Ca_Kabuli_Chr07*	27210848	(A/T)
CakSNP12810	*Kabuli*	*Ca_Kabuli_Chr07*	27210951	(T/C)
CakSNP12811	*Kabuli*	*Ca_Kabuli_Chr07*	27210945	(C/T)
CakSNP12812	*Kabuli*	*Ca_Kabuli_Chr07*	27210988	(C/T)
CakSNP12813	*Kabuli*	*Ca_Kabuli_Chr07*	27210989	(C/T)
CakSNP12814	*Kabuli*	*Ca_Kabuli_Chr07*	27210992	(C/A)
CakSNP12815	*Kabuli*	*Ca_Kabuli_Chr07*	27211004	(T/C)
CakSNP12816	*Kabuli*	*Ca_Kabuli_Chr07*	27211036	(G/A)
CakSNP12817	*Kabuli*	*Ca_Kabuli_Chr07*	27211051	(C/A)
CakSNP12818	*Kabuli*	*Ca_Kabuli_Chr07*	27211084	(G/T)
CakSNP12819	*Kabuli*	*Ca_Kabuli_Chr07*	27211073	(C/T)
CakSNP12820	*Kabuli*	*Ca_Kabuli_Chr07*	27211060	(C/T)
CakSNP12821	*Kabuli*	*Ca_Kabuli_Chr07*	27633960	(C/A)
CakSNP12822	*Kabuli*	*Ca_Kabuli_Chr07*	27735234	(A/G)
CakSNP12823	*Kabuli*	*Ca_Kabuli_Chr07*	27899782	(T/C)
CakSNP12824	*Kabuli*	*Ca_Kabuli_Chr07*	27899743	(G/A)
CakSNP12825	*Kabuli*	*Ca_Kabuli_Chr07*	27971186	(T/G)
CakSNP12826	*Kabuli*	*Ca_Kabuli_Chr07*	28676139	(T/C)
CakSNP12827	*Kabuli*	*Ca_Kabuli_Chr07*	28931212	(A/C)
CakSNP12828	*Kabuli*	*Ca_Kabuli_Chr07*	28931223	(C/A)
CakSNP12829	*Kabuli*	*Ca_Kabuli_Chr07*	29150927	(A/C)
CakSNP12830	*Kabuli*	*Ca_Kabuli_Chr07*	29153994	(T/C)
CakSNP12831	*Kabuli*	*Ca_Kabuli_Chr07*	29154220	(C/A)
CakSNP12832	*Kabuli*	*Ca_Kabuli_Chr07*	29157286	(A/G)

**SNP IDs**	**Cultivars**	**Chromosomes/scaffolds**	**Physical positions (bp)**	**SNPs**
CakSNP12833	*Kabuli*	*Ca_Kabuli_Chr07*	29168982	(G/A)
CakSNP12834	*Kabuli*	*Ca_Kabuli_Chr07*	29168980	(C/T)
CakSNP12835	*Kabuli*	*Ca_Kabuli_Chr07*	29190659	(T/G)
CakSNP12836	*Kabuli*	*Ca_Kabuli_Chr07*	29225385	(T/G)
CakSNP12837	*Kabuli*	*Ca_Kabuli_Chr07*	29225351	(T/G)
CakSNP12838	*Kabuli*	*Ca_Kabuli_Chr07*	29225348	(G/C)
CakSNP12839	*Kabuli*	*Ca_Kabuli_Chr07*	29229965	(A/G)
CakSNP12840	*Kabuli*	*Ca_Kabuli_Chr07*	29313114	(G/T)
CakSNP12841	*Kabuli*	*Ca_Kabuli_Chr07*	29313080	(T/C)
CakSNP12842	*Kabuli*	*Ca_Kabuli_Chr07*	29313067	(G/T)
CakSNP12843	*Kabuli*	*Ca_Kabuli_Chr07*	29313059	(G/A)
CakSNP12844	*Kabuli*	*Ca_Kabuli_Chr07*	29313058	(T/C)
CakSNP12845	*Kabuli*	*Ca_Kabuli_Chr07*	29313051	(T/C)
CakSNP12846	*Kabuli*	*Ca_Kabuli_Chr07*	29313257	(G/A)
CakSNP12847	*Kabuli*	*Ca_Kabuli_Chr07*	29434818	(T/C)
CakSNP12848	*Kabuli*	*Ca_Kabuli_Chr07*	29482556	(G/A)
CakSNP12849	*Kabuli*	*Ca_Kabuli_Chr07*	29484304	(G/A)
CakSNP12850	*Kabuli*	*Ca_Kabuli_Chr07*	29607943	(C/T)
CakSNP12851	*Kabuli*	*Ca_Kabuli_Chr07*	29613699	(C/T)
CakSNP12852	*Kabuli*	*Ca_Kabuli_Chr07*	29625884	(T/C)
CakSNP12853	*Kabuli*	*Ca_Kabuli_Chr07*	29705531	(T/G)
CakSNP12854	*Kabuli*	*Ca_Kabuli_Chr07*	29705499	(T/C)
CakSNP12855	*Kabuli*	*Ca_Kabuli_Chr07*	29796564	(G/T)
CakSNP12856	*Kabuli*	*Ca_Kabuli_Chr07*	29801148	(A/G)
CakSNP12857	*Kabuli*	*Ca_Kabuli_Chr07*	29801154	(T/C)
CakSNP12858	*Kabuli*	*Ca_Kabuli_Chr07*	29837035	(G/A)
CakSNP12859	*Kabuli*	*Ca_Kabuli_Chr07*	29838073	(C/T)
CakSNP12860	*Kabuli*	*Ca_Kabuli_Chr07*	29848248	(T/C)
CakSNP12861	*Kabuli*	*Ca_Kabuli_Chr07*	29848433	(C/T)
CakSNP12862	*Kabuli*	*Ca_Kabuli_Chr07*	29849509	(C/T)
CakSNP12863	*Kabuli*	*Ca_Kabuli_Chr07*	29849544	(A/G)
CakSNP12864	*Kabuli*	*Ca_Kabuli_Chr07*	29884719	(G/T)
CakSNP12865	*Kabuli*	*Ca_Kabuli_Chr07*	29884718	(C/A)
CakSNP12866	*Kabuli*	*Ca_Kabuli_Chr07*	29925008	(G/A)
CakSNP12867	*Kabuli*	*Ca_Kabuli_Chr07*	30004767	(C/T)
CakSNP12868	*Kabuli*	*Ca_Kabuli_Chr07*	30004934	(A/C)
CakSNP12869	*Kabuli*	*Ca_Kabuli_Chr07*	30026039	(T/G)
CakSNP12870	*Kabuli*	*Ca_Kabuli_Chr07*	30026017	(C/T)
CakSNP12871	*Kabuli*	*Ca_Kabuli_Chr07*	30029793	(A/C)
CakSNP12872	*Kabuli*	*Ca_Kabuli_Chr07*	30029820	(T/C)
CakSNP12873	*Kabuli*	*Ca_Kabuli_Chr07*	30060299	(C/A)

SNP IDs	Cultivars	Chromosomes/scaffolds	Physical positions (bp)	SNPs
CakSNP12874	*Kabuli*	*Ca_Kabuli_Chr07*	30085847	(C/T)
CakSNP12875	*Kabuli*	*Ca_Kabuli_Chr07*	30085883	(T/G)
CakSNP12876	*Kabuli*	*Ca_Kabuli_Chr07*	30085906	(C/T)
CakSNP12877	*Kabuli*	*Ca_Kabuli_Chr07*	30085910	(T/A)
CakSNP12878	*Kabuli*	*Ca_Kabuli_Chr07*	30099003	(C/T)
CakSNP12879	*Kabuli*	*Ca_Kabuli_Chr07*	30107340	(A/G)
CakSNP12880	*Kabuli*	*Ca_Kabuli_Chr07*	30398855	(A/C)
CakSNP12881	*Kabuli*	*Ca_Kabuli_Chr07*	30403615	(A/G)
CakSNP12882	*Kabuli*	*Ca_Kabuli_Chr07*	30403618	(G/A)
CakSNP12883	*Kabuli*	*Ca_Kabuli_Chr07*	30403700	(C/T)
CakSNP12884	*Kabuli*	*Ca_Kabuli_Chr07*	30403692	(G/A)
CakSNP12885	*Kabuli*	*Ca_Kabuli_Chr07*	30403621	(G/T)
CakSNP12886	*Kabuli*	*Ca_Kabuli_Chr07*	30691324	(A/C)
CakSNP12887	*Kabuli*	*Ca_Kabuli_Chr07*	30691318	(A/C)
CakSNP12888	*Kabuli*	*Ca_Kabuli_Chr07*	30768315	(G/A)
CakSNP12889	*Kabuli*	*Ca_Kabuli_Chr07*	30850950	(A/C)
CakSNP12890	*Kabuli*	*Ca_Kabuli_Chr07*	31190059	(G/A)
CakSNP12891	*Kabuli*	*Ca_Kabuli_Chr07*	31193786	(T/G)
CakSNP12892	*Kabuli*	*Ca_Kabuli_Chr07*	31314421	(T/A)
CakSNP12893	*Kabuli*	*Ca_Kabuli_Chr07*	31324279	(A/G)
CakSNP12894	*Kabuli*	*Ca_Kabuli_Chr07*	31401807	(T/C)
CakSNP12895	*Kabuli*	*Ca_Kabuli_Chr07*	31442425	(A/C)
CakSNP12896	*Kabuli*	*Ca_Kabuli_Chr07*	31612697	(T/A)
CakSNP12897	*Kabuli*	*Ca_Kabuli_Chr07*	31639701	(C/T)
CakSNP12898	*Kabuli*	*Ca_Kabuli_Chr07*	31830238	(A/G)
CakSNP12899	*Kabuli*	*Ca_Kabuli_Chr07*	31851096	(T/A)
CakSNP12900	*Kabuli*	*Ca_Kabuli_Chr07*	31873079	(G/T)
CakSNP12901	*Kabuli*	*Ca_Kabuli_Chr07*	31996345	(A/C)
CakSNP12902	*Kabuli*	*Ca_Kabuli_Chr07*	32017294	(G/A)
CakSNP12903	*Kabuli*	*Ca_Kabuli_Chr07*	32017288	(G/T)
CakSNP12904	*Kabuli*	*Ca_Kabuli_Chr07*	32017286	(T/G)
CakSNP12905	*Kabuli*	*Ca_Kabuli_Chr07*	32017284	(C/T)
CakSNP12906	*Kabuli*	*Ca_Kabuli_Chr07*	32017253	(G/A)
CakSNP12907	*Kabuli*	*Ca_Kabuli_Chr07*	32017252	(G/A)
CakSNP12908	*Kabuli*	*Ca_Kabuli_Chr07*	32017238	(C/T)
CakSNP12909	*Kabuli*	*Ca_Kabuli_Chr07*	32017228	(G/A)
CakSNP12910	*Kabuli*	*Ca_Kabuli_Chr07*	32017221	(C/T)
CakSNP12911	*Kabuli*	*Ca_Kabuli_Chr07*	32017211	(C/A)
CakSNP12912	*Kabuli*	*Ca_Kabuli_Chr07*	32127288	(T/A)
CakSNP12913	*Kabuli*	*Ca_Kabuli_Chr07*	32283585	(T/G)
CakSNP12914	*Kabuli*	*Ca_Kabuli_Chr07*	32309397	(A/T)

SNP IDs	Cultivars	Chromosomes/scaffolds	Physical positions (bp)	SNPs
CakSNP12915	*Kabuli*	*Ca_Kabuli_Chr07*	32309411	(G/T)
CakSNP12916	*Kabuli*	*Ca_Kabuli_Chr07*	32309415	(C/T)
CakSNP12917	*Kabuli*	*Ca_Kabuli_Chr07*	32309429	(C/T)
CakSNP12918	*Kabuli*	*Ca_Kabuli_Chr07*	32309377	(C/T)
CakSNP12919	*Kabuli*	*Ca_Kabuli_Chr07*	32309376	(C/G)
CakSNP12920	*Kabuli*	*Ca_Kabuli_Chr07*	32399759	(C/T)
CakSNP12921	*Kabuli*	*Ca_Kabuli_Chr07*	32404932	(T/C)
CakSNP12922	*Kabuli*	*Ca_Kabuli_Chr07*	32451377	(A/C)
CakSNP12923	*Kabuli*	*Ca_Kabuli_Chr07*	32522201	(A/G)
CakSNP12924	*Kabuli*	*Ca_Kabuli_Chr07*	32522203	(A/G)
CakSNP12925	*Kabuli*	*Ca_Kabuli_Chr07*	32522207	(T/G)
CakSNP12926	*Kabuli*	*Ca_Kabuli_Chr07*	32522213	(A/G)
CakSNP12927	*Kabuli*	*Ca_Kabuli_Chr07*	32522230	(T/G)
CakSNP12928	*Kabuli*	*Ca_Kabuli_Chr07*	32557599	(G/A)
CakSNP12929	*Kabuli*	*Ca_Kabuli_Chr07*	32669570	(A/G)
CakSNP12930	*Kabuli*	*Ca_Kabuli_Chr07*	32754633	(T/G)
CakSNP12931	*Kabuli*	*Ca_Kabuli_Chr07*	32755647	(A/C)
CakSNP12932	*Kabuli*	*Ca_Kabuli_Chr07*	32755662	(G/T)
CakSNP12933	*Kabuli*	*Ca_Kabuli_Chr07*	32784903	(T/G)
CakSNP12934	*Kabuli*	*Ca_Kabuli_Chr07*	32799037	(A/G)
CakSNP12935	*Kabuli*	*Ca_Kabuli_Chr07*	32807457	(T/A)
CakSNP12936	*Kabuli*	*Ca_Kabuli_Chr07*	32807499	(A/G)
CakSNP12937	*Kabuli*	*Ca_Kabuli_Chr07*	32874847	(C/T)
CakSNP12938	*Kabuli*	*Ca_Kabuli_Chr07*	32874840	(C/T)
CakSNP12939	*Kabuli*	*Ca_Kabuli_Chr07*	32875799	(T/A)
CakSNP12940	*Kabuli*	*Ca_Kabuli_Chr07*	32942199	(A/G)
CakSNP12941	*Kabuli*	*Ca_Kabuli_Chr07*	32942951	(T/G)
CakSNP12942	*Kabuli*	*Ca_Kabuli_Chr07*	32989796	(T/A)
CakSNP12943	*Kabuli*	*Ca_Kabuli_Chr07*	32989838	(C/T)
CakSNP12944	*Kabuli*	*Ca_Kabuli_Chr07*	32989903	(A/G)
CakSNP12945	*Kabuli*	*Ca_Kabuli_Chr07*	32989902	(C/A)
CakSNP12946	*Kabuli*	*Ca_Kabuli_Chr07*	33065611	(G/T)
CakSNP12947	*Kabuli*	*Ca_Kabuli_Chr07*	33121674	(G/C)
CakSNP12948	*Kabuli*	*Ca_Kabuli_Chr07*	33121640	(C/A)
CakSNP12949	*Kabuli*	*Ca_Kabuli_Chr07*	33122473	(A/C)
CakSNP12950	*Kabuli*	*Ca_Kabuli_Chr07*	33182932	(G/A)
CakSNP12951	*Kabuli*	*Ca_Kabuli_Chr07*	33267485	(T/C)
CakSNP12952	*Kabuli*	*Ca_Kabuli_Chr07*	33267496	(T/A)
CakSNP12953	*Kabuli*	*Ca_Kabuli_Chr07*	33335978	(C/A)
CakSNP12954	*Kabuli*	*Ca_Kabuli_Chr07*	33364329	(C/T)
CakSNP12955	*Kabuli*	*Ca_Kabuli_Chr07*	33364354	(G/A)

**SNP IDs**	**Cultivars**	**Chromosomes/scaffolds**	**Physical positions (bp)**	**SNPs**
CakSNP12956	*Kabuli*	*Ca_Kabuli_Chr07*	33378427	(C/T)
CakSNP12957	*Kabuli*	*Ca_Kabuli_Chr07*	33411746	(C/A)
CakSNP12958	*Kabuli*	*Ca_Kabuli_Chr07*	33465828	(C/A)
CakSNP12959	*Kabuli*	*Ca_Kabuli_Chr07*	33465818	(G/A)
CakSNP12960	*Kabuli*	*Ca_Kabuli_Chr07*	33465805	(T/C)
CakSNP12961	*Kabuli*	*Ca_Kabuli_Chr07*	33465802	(C/A)
CakSNP12962	*Kabuli*	*Ca_Kabuli_Chr07*	33465800	(T/A)
CakSNP12963	*Kabuli*	*Ca_Kabuli_Chr07*	33465781	(G/T)
CakSNP12964	*Kabuli*	*Ca_Kabuli_Chr07*	33465855	(A/C)
CakSNP12965	*Kabuli*	*Ca_Kabuli_Chr07*	33480940	(C/T)
CakSNP12966	*Kabuli*	*Ca_Kabuli_Chr07*	33573605	(C/T)
CakSNP12967	*Kabuli*	*Ca_Kabuli_Chr07*	33573655	(A/C)
CakSNP12968	*Kabuli*	*Ca_Kabuli_Chr07*	33619268	(G/C)
CakSNP12969	*Kabuli*	*Ca_Kabuli_Chr07*	33641799	(G/T)
CakSNP12970	*Kabuli*	*Ca_Kabuli_Chr07*	33641882	(A/T)
CakSNP12971	*Kabuli*	*Ca_Kabuli_Chr07*	33674454	(T/C)
CakSNP12972	*Kabuli*	*Ca_Kabuli_Chr07*	33744521	(C/G)
CakSNP12973	*Kabuli*	*Ca_Kabuli_Chr07*	33753917	(G/A)
CakSNP12974	*Kabuli*	*Ca_Kabuli_Chr07*	33761944	(G/A)
CakSNP12975	*Kabuli*	*Ca_Kabuli_Chr07*	33762053	(T/G)
CakSNP12976	*Kabuli*	*Ca_Kabuli_Chr07*	33821658	(C/T)
CakSNP12977	*Kabuli*	*Ca_Kabuli_Chr07*	33865463	(T/C)
CakSNP12978	*Kabuli*	*Ca_Kabuli_Chr07*	33865475	(G/A)
CakSNP12979	*Kabuli*	*Ca_Kabuli_Chr07*	33867334	(C/T)
CakSNP12980	*Kabuli*	*Ca_Kabuli_Chr07*	33867491	(C/T)
CakSNP12981	*Kabuli*	*Ca_Kabuli_Chr07*	33867674	(C/T)
CakSNP12982	*Kabuli*	*Ca_Kabuli_Chr07*	33869367	(G/A)
CakSNP12983	*Kabuli*	*Ca_Kabuli_Chr07*	33879559	(C/G)
CakSNP12984	*Kabuli*	*Ca_Kabuli_Chr07*	33910324	(C/A)
CakSNP12985	*Kabuli*	*Ca_Kabuli_Chr07*	33910870	(C/A)
CakSNP12986	*Kabuli*	*Ca_Kabuli_Chr07*	33912719	(T/G)
CakSNP12987	*Kabuli*	*Ca_Kabuli_Chr07*	33912766	(G/T)
CakSNP12988	*Kabuli*	*Ca_Kabuli_Chr07*	33912866	(T/C)
CakSNP12989	*Kabuli*	*Ca_Kabuli_Chr07*	33912899	(T/A)
CakSNP12990	*Kabuli*	*Ca_Kabuli_Chr07*	33912902	(A/T)
CakSNP12991	*Kabuli*	*Ca_Kabuli_Chr07*	33912913	(A/T)
CakSNP12992	*Kabuli*	*Ca_Kabuli_Chr07*	33912916	(G/T)
CakSNP12993	*Kabuli*	*Ca_Kabuli_Chr07*	33912921	(T/G)
CakSNP12994	*Kabuli*	*Ca_Kabuli_Chr07*	33912950	(C/T)
CakSNP12995	*Kabuli*	*Ca_Kabuli_Chr07*	33912966	(T/C)
CakSNP12996	*Kabuli*	*Ca_Kabuli_Chr07*	33913094	(A/G)

SNP IDs	Cultivars	Chromosomes/scaffolds	Physical positions (bp)	SNPs
CakSNP12997	*Kabuli*	*Ca_Kabuli_Chr07*	33913047	(C/T)
CakSNP12998	*Kabuli*	*Ca_Kabuli_Chr07*	33913045	(G/T)
CakSNP12999	*Kabuli*	*Ca_Kabuli_Chr07*	34107603	(C/A)
CakSNP13000	*Kabuli*	*Ca_Kabuli_Chr07*	34152124	(G/C)
CakSNP13001	*Kabuli*	*Ca_Kabuli_Chr07*	34271486	(A/G)
CakSNP13002	*Kabuli*	*Ca_Kabuli_Chr07*	34277014	(G/T)
CakSNP13003	*Kabuli*	*Ca_Kabuli_Chr07*	34277010	(A/G)
CakSNP13004	*Kabuli*	*Ca_Kabuli_Chr07*	34279673	(G/A)
CakSNP13005	*Kabuli*	*Ca_Kabuli_Chr07*	34279725	(A/G)
CakSNP13006	*Kabuli*	*Ca_Kabuli_Chr07*	34279715	(A/C)
CakSNP13007	*Kabuli*	*Ca_Kabuli_Chr07*	34318860	(C/T)
CakSNP13008	*Kabuli*	*Ca_Kabuli_Chr07*	34318862	(A/G)
CakSNP13009	*Kabuli*	*Ca_Kabuli_Chr07*	34474614	(A/C)
CakSNP13010	*Kabuli*	*Ca_Kabuli_Chr07*	34801839	(A/T)
CakSNP13011	*Kabuli*	*Ca_Kabuli_Chr07*	34857693	(C/T)
CakSNP13012	*Kabuli*	*Ca_Kabuli_Chr07*	34947743	(A/C)
CakSNP13013	*Kabuli*	*Ca_Kabuli_Chr07*	35016516	(G/C)
CakSNP13014	*Kabuli*	*Ca_Kabuli_Chr07*	35072425	(A/T)
CakSNP13015	*Kabuli*	*Ca_Kabuli_Chr07*	35072407	(G/A)
CakSNP13016	*Kabuli*	*Ca_Kabuli_Chr07*	35072461	(A/T)
CakSNP13017	*Kabuli*	*Ca_Kabuli_Chr07*	35074345	(T/A)
CakSNP13018	*Kabuli*	*Ca_Kabuli_Chr07*	35074348	(T/C)
CakSNP13019	*Kabuli*	*Ca_Kabuli_Chr07*	35074362	(T/C)
CakSNP13020	*Kabuli*	*Ca_Kabuli_Chr07*	35112785	(T/C)
CakSNP13021	*Kabuli*	*Ca_Kabuli_Chr07*	35114049	(C/T)
CakSNP13022	*Kabuli*	*Ca_Kabuli_Chr07*	35134809	(A/C)
CakSNP13023	*Kabuli*	*Ca_Kabuli_Chr07*	35134863	(T/C)
CakSNP13024	*Kabuli*	*Ca_Kabuli_Chr07*	35181619	(T/G)
CakSNP13025	*Kabuli*	*Ca_Kabuli_Chr07*	35181661	(T/C)
CakSNP13026	*Kabuli*	*Ca_Kabuli_Chr07*	35181660	(G/A)
CakSNP13027	*Kabuli*	*Ca_Kabuli_Chr07*	35181635	(G/C)
CakSNP13028	*Kabuli*	*Ca_Kabuli_Chr07*	35227794	(A/C)
CakSNP13029	*Kabuli*	*Ca_Kabuli_Chr07*	35258029	(T/G)
CakSNP13030	*Kabuli*	*Ca_Kabuli_Chr07*	35263711	(A/T)
CakSNP13031	*Kabuli*	*Ca_Kabuli_Chr07*	35263712	(G/T)
CakSNP13032	*Kabuli*	*Ca_Kabuli_Chr07*	35263736	(C/G)
CakSNP13033	*Kabuli*	*Ca_Kabuli_Chr07*	35263758	(A/G)
CakSNP13034	*Kabuli*	*Ca_Kabuli_Chr07*	35274368	(A/G)
CakSNP13035	*Kabuli*	*Ca_Kabuli_Chr07*	35274859	(T/G)
CakSNP13036	*Kabuli*	*Ca_Kabuli_Chr07*	35274858	(G/A)
CakSNP13037	*Kabuli*	*Ca_Kabuli_Chr07*	35274828	(A/G)

SNP IDs	Cultivars	Chromosomes/scaffolds	Physical positions (bp)	SNPs
CakSNP13038	*Kabuli*	*Ca_Kabuli_Chr07*	35279675	(A/G)
CakSNP13039	*Kabuli*	*Ca_Kabuli_Chr07*	35280189	(G/A)
CakSNP13040	*Kabuli*	*Ca_Kabuli_Chr07*	35280167	(A/G)
CakSNP13041	*Kabuli*	*Ca_Kabuli_Chr07*	35576427	(C/A)
CakSNP13042	*Kabuli*	*Ca_Kabuli_Chr07*	35619530	(G/A)
CakSNP13043	*Kabuli*	*Ca_Kabuli_Chr07*	35619487	(G/A)
CakSNP13044	*Kabuli*	*Ca_Kabuli_Chr07*	35619518	(G/C)
CakSNP13045	*Kabuli*	*Ca_Kabuli_Chr07*	35622168	(A/G)
CakSNP13046	*Kabuli*	*Ca_Kabuli_Chr07*	35637056	(T/C)
CakSNP13047	*Kabuli*	*Ca_Kabuli_Chr07*	35680423	(A/C)
CakSNP13048	*Kabuli*	*Ca_Kabuli_Chr07*	35799096	(A/C)
CakSNP13049	*Kabuli*	*Ca_Kabuli_Chr07*	35799083	(T/A)
CakSNP13050	*Kabuli*	*Ca_Kabuli_Chr07*	35799074	(G/C)
CakSNP13051	*Kabuli*	*Ca_Kabuli_Chr07*	35799073	(T/C)
CakSNP13052	*Kabuli*	*Ca_Kabuli_Chr07*	35807000	(A/G)
CakSNP13053	*Kabuli*	*Ca_Kabuli_Chr07*	35806999	(C/G)
CakSNP13054	*Kabuli*	*Ca_Kabuli_Chr07*	35806990	(A/T)
CakSNP13055	*Kabuli*	*Ca_Kabuli_Chr07*	35806977	(T/G)
CakSNP13056	*Kabuli*	*Ca_Kabuli_Chr07*	35911620	(T/G)
CakSNP13057	*Kabuli*	*Ca_Kabuli_Chr07*	35911617	(A/C)
CakSNP13058	*Kabuli*	*Ca_Kabuli_Chr07*	35911615	(C/T)
CakSNP13059	*Kabuli*	*Ca_Kabuli_Chr07*	35911612	(T/G)
CakSNP13060	*Kabuli*	*Ca_Kabuli_Chr07*	35911606	(T/C)
CakSNP13061	*Kabuli*	*Ca_Kabuli_Chr07*	35947529	(T/C)
CakSNP13062	*Kabuli*	*Ca_Kabuli_Chr07*	35947550	(C/T)
CakSNP13063	*Kabuli*	*Ca_Kabuli_Chr07*	35947551	(A/G)
CakSNP13064	*Kabuli*	*Ca_Kabuli_Chr07*	35959805	(C/T)
CakSNP13065	*Kabuli*	*Ca_Kabuli_Chr07*	35959825	(G/A)
CakSNP13066	*Kabuli*	*Ca_Kabuli_Chr07*	35959878	(G/A)
CakSNP13067	*Kabuli*	*Ca_Kabuli_Chr07*	35959917	(C/T)
CakSNP13068	*Kabuli*	*Ca_Kabuli_Chr07*	35959865	(T/C)
CakSNP13069	*Kabuli*	*Ca_Kabuli_Chr07*	36711954	(C/A)
CakSNP13070	*Kabuli*	*Ca_Kabuli_Chr07*	36715249	(T/C)
CakSNP13071	*Kabuli*	*Ca_Kabuli_Chr07*	36745439	(T/G)
CakSNP13072	*Kabuli*	*Ca_Kabuli_Chr07*	36916272	(T/G)
CakSNP13073	*Kabuli*	*Ca_Kabuli_Chr07*	36916273	(T/A)
CakSNP13074	*Kabuli*	*Ca_Kabuli_Chr07*	36998096	(T/C)
CakSNP13075	*Kabuli*	*Ca_Kabuli_Chr07*	36998113	(C/T)
CakSNP13076	*Kabuli*	*Ca_Kabuli_Chr07*	37058979	(C/T)
CakSNP13077	*Kabuli*	*Ca_Kabuli_Chr07*	37187439	(A/G)
CakSNP13078	*Kabuli*	*Ca_Kabuli_Chr07*	37626729	(G/T)

SNP IDs	Cultivars	Chromosomes/scaffolds	Physical positions (bp)	SNPs
CakSNP13079	*Kabuli*	*Ca_Kabuli_Chr07*	37626653	(A/C)
CakSNP13080	*Kabuli*	*Ca_Kabuli_Chr07*	37626687	(A/G)
CakSNP13081	*Kabuli*	*Ca_Kabuli_Chr07*	37626666	(C/T)
CakSNP13082	*Kabuli*	*Ca_Kabuli_Chr07*	37703967	(G/A)
CakSNP13083	*Kabuli*	*Ca_Kabuli_Chr07*	37703966	(C/T)
CakSNP13084	*Kabuli*	*Ca_Kabuli_Chr07*	37703965	(C/T)
CakSNP13085	*Kabuli*	*Ca_Kabuli_Chr07*	37703921	(G/T)
CakSNP13086	*Kabuli*	*Ca_Kabuli_Chr07*	37703912	(G/A)
CakSNP13087	*Kabuli*	*Ca_Kabuli_Chr07*	37703913	(C/G)
CakSNP13088	*Kabuli*	*Ca_Kabuli_Chr07*	37703936	(C/T)
CakSNP13089	*Kabuli*	*Ca_Kabuli_Chr07*	37857936	(G/T)
CakSNP13090	*Kabuli*	*Ca_Kabuli_Chr07*	37857928	(A/T)
CakSNP13091	*Kabuli*	*Ca_Kabuli_Chr07*	37857917	(G/A)
CakSNP13092	*Kabuli*	*Ca_Kabuli_Chr07*	37857885	(C/T)
CakSNP13093	*Kabuli*	*Ca_Kabuli_Chr07*	38047374	(A/G)
CakSNP13094	*Kabuli*	*Ca_Kabuli_Chr07*	38047378	(C/A)
CakSNP13095	*Kabuli*	*Ca_Kabuli_Chr07*	38143361	(A/G)
CakSNP13096	*Kabuli*	*Ca_Kabuli_Chr07*	38143524	(T/A)
CakSNP13097	*Kabuli*	*Ca_Kabuli_Chr07*	38143452	(G/A)
CakSNP13098	*Kabuli*	*Ca_Kabuli_Chr07*	38460464	(G/A)
CakSNP13099	*Kabuli*	*Ca_Kabuli_Chr07*	38460452	(C/T)
CakSNP13100	*Kabuli*	*Ca_Kabuli_Chr07*	38460425	(G/A)
CakSNP13101	*Kabuli*	*Ca_Kabuli_Chr07*	38460394	(C/A)
CakSNP13102	*Kabuli*	*Ca_Kabuli_Chr07*	39181880	(G/A)
CakSNP13103	*Kabuli*	*Ca_Kabuli_Chr07*	39182564	(A/T)
CakSNP13104	*Kabuli*	*Ca_Kabuli_Chr07*	39339510	(T/G)
CakSNP13105	*Kabuli*	*Ca_Kabuli_Chr07*	39339509	(G/A)
CakSNP13106	*Kabuli*	*Ca_Kabuli_Chr07*	39339508	(G/A)
CakSNP13107	*Kabuli*	*Ca_Kabuli_Chr07*	39339503	(C/A)
CakSNP13108	*Kabuli*	*Ca_Kabuli_Chr07*	39339502	(C/A)
CakSNP13109	*Kabuli*	*Ca_Kabuli_Chr07*	39339501	(G/C)
CakSNP13110	*Kabuli*	*Ca_Kabuli_Chr07*	39339500	(G/T)
CakSNP13111	*Kabuli*	*Ca_Kabuli_Chr07*	39339499	(C/T)
CakSNP13112	*Kabuli*	*Ca_Kabuli_Chr07*	39339498	(G/T)
CakSNP13113	*Kabuli*	*Ca_Kabuli_Chr07*	39339495	(G/T)
CakSNP13114	*Kabuli*	*Ca_Kabuli_Chr07*	39399280	(T/C)
CakSNP13115	*Kabuli*	*Ca_Kabuli_Chr07*	39680221	(A/C)
CakSNP13116	*Kabuli*	*Ca_Kabuli_Chr07*	39680222	(G/A)
CakSNP13117	*Kabuli*	*Ca_Kabuli_Chr07*	39768248	(T/C)
CakSNP13118	*Kabuli*	*Ca_Kabuli_Chr07*	39944444	(T/C)
CakSNP13119	*Kabuli*	*Ca_Kabuli_Chr07*	39944937	(T/C)

SNP IDs	Cultivars	Chromosomes/scaffolds	Physical positions (bp)	SNPs
CakSNP13120	*Kabuli*	*Ca_Kabuli_Chr07*	39944889	(T/C)
CakSNP13121	*Kabuli*	*Ca_Kabuli_Chr07*	40092198	(G/A)
CakSNP13122	*Kabuli*	*Ca_Kabuli_Chr07*	40092181	(C/T)
CakSNP13123	*Kabuli*	*Ca_Kabuli_Chr07*	40092218	(A/T)
CakSNP13124	*Kabuli*	*Ca_Kabuli_Chr07*	40092213	(G/A)
CakSNP13125	*Kabuli*	*Ca_Kabuli_Chr07*	40092163	(T/C)
CakSNP13126	*Kabuli*	*Ca_Kabuli_Chr07*	40092155	(T/A)
CakSNP13127	*Kabuli*	*Ca_Kabuli_Chr07*	40092156	(C/A)
CakSNP13128	*Kabuli*	*Ca_Kabuli_Chr07*	40092168	(G/A)
CakSNP13129	*Kabuli*	*Ca_Kabuli_Chr07*	40092201	(G/A)
CakSNP13130	*Kabuli*	*Ca_Kabuli_Chr07*	40092203	(T/G)
CakSNP13131	*Kabuli*	*Ca_Kabuli_Chr07*	40092211	(C/T)
CakSNP13132	*Kabuli*	*Ca_Kabuli_Chr07*	40092214	(C/G)
CakSNP13133	*Kabuli*	*Ca_Kabuli_Chr07*	40092229	(C/A)
CakSNP13134	*Kabuli*	*Ca_Kabuli_Chr07*	40092237	(C/T)
CakSNP13135	*Kabuli*	*Ca_Kabuli_Chr07*	40092239	(G/A)
CakSNP13136	*Kabuli*	*Ca_Kabuli_Chr07*	40092250	(A/C)
CakSNP13137	*Kabuli*	*Ca_Kabuli_Chr07*	40092253	(C/T)
CakSNP13138	*Kabuli*	*Ca_Kabuli_Chr07*	40092256	(T/C)
CakSNP13139	*Kabuli*	*Ca_Kabuli_Chr07*	40092267	(G/C)
CakSNP13140	*Kabuli*	*Ca_Kabuli_Chr07*	40183449	(C/A)
CakSNP13141	*Kabuli*	*Ca_Kabuli_Chr07*	40183452	(A/C)
CakSNP13142	*Kabuli*	*Ca_Kabuli_Chr07*	40241674	(A/C)
CakSNP13143	*Kabuli*	*Ca_Kabuli_Chr07*	40242539	(A/G)
CakSNP13144	*Kabuli*	*Ca_Kabuli_Chr07*	40242765	(A/G)
CakSNP13145	*Kabuli*	*Ca_Kabuli_Chr07*	40243784	(G/A)
CakSNP13146	*Kabuli*	*Ca_Kabuli_Chr07*	40323213	(C/A)
CakSNP13147	*Kabuli*	*Ca_Kabuli_Chr07*	40323251	(T/A)
CakSNP13148	*Kabuli*	*Ca_Kabuli_Chr07*	40429548	(A/G)
CakSNP13149	*Kabuli*	*Ca_Kabuli_Chr07*	40429552	(C/T)
CakSNP13150	*Kabuli*	*Ca_Kabuli_Chr07*	40429564	(G/T)
CakSNP13151	*Kabuli*	*Ca_Kabuli_Chr07*	40429573	(G/T)
CakSNP13152	*Kabuli*	*Ca_Kabuli_Chr07*	40429629	(G/T)
CakSNP13153	*Kabuli*	*Ca_Kabuli_Chr07*	40429621	(G/T)
CakSNP13154	*Kabuli*	*Ca_Kabuli_Chr07*	40429604	(C/T)
CakSNP13155	*Kabuli*	*Ca_Kabuli_Chr07*	40429600	(C/T)
CakSNP13156	*Kabuli*	*Ca_Kabuli_Chr07*	40429596	(G/A)
CakSNP13157	*Kabuli*	*Ca_Kabuli_Chr07*	40429567	(G/A)
CakSNP13158	*Kabuli*	*Ca_Kabuli_Chr07*	40456574	(T/C)
CakSNP13159	*Kabuli*	*Ca_Kabuli_Chr07*	40645278	(A/G)
CakSNP13160	*Kabuli*	*Ca_Kabuli_Chr07*	40645276	(A/G)

SNP IDs	Cultivars	Chromosomes/scaffolds	Physical positions (bp)	SNPs
CakSNP13161	*Kabuli*	*Ca_Kabuli_Chr07*	40645208	(C/G)
CakSNP13162	*Kabuli*	*Ca_Kabuli_Chr07*	40889596	(A/C)
CakSNP13163	*Kabuli*	*Ca_Kabuli_Chr07*	40889598	(C/G)
CakSNP13164	*Kabuli*	*Ca_Kabuli_Chr07*	40889623	(G/A)
CakSNP13165	*Kabuli*	*Ca_Kabuli_Chr07*	40889632	(C/T)
CakSNP13166	*Kabuli*	*Ca_Kabuli_Chr07*	40889633	(G/A)
CakSNP13167	*Kabuli*	*Ca_Kabuli_Chr07*	40889640	(G/A)
CakSNP13168	*Kabuli*	*Ca_Kabuli_Chr07*	40889641	(C/T)
CakSNP13169	*Kabuli*	*Ca_Kabuli_Chr07*	40889643	(G/A)
CakSNP13170	*Kabuli*	*Ca_Kabuli_Chr07*	40889648	(T/A)
CakSNP13171	*Kabuli*	*Ca_Kabuli_Chr07*	40889649	(G/A)
CakSNP13172	*Kabuli*	*Ca_Kabuli_Chr07*	40889673	(C/A)
CakSNP13173	*Kabuli*	*Ca_Kabuli_Chr07*	40889670	(C/T)
CakSNP13174	*Kabuli*	*Ca_Kabuli_Chr07*	40889723	(C/G)
CakSNP13175	*Kabuli*	*Ca_Kabuli_Chr07*	40889684	(C/T)
CakSNP13176	*Kabuli*	*Ca_Kabuli_Chr07*	41282883	(A/T)
CakSNP13177	*Kabuli*	*Ca_Kabuli_Chr07*	41282961	(G/T)
CakSNP13178	*Kabuli*	*Ca_Kabuli_Chr07*	41282960	(A/C)
CakSNP13179	*Kabuli*	*Ca_Kabuli_Chr07*	41282957	(G/A)
CakSNP13180	*Kabuli*	*Ca_Kabuli_Chr07*	41325886	(A/G)
CakSNP13181	*Kabuli*	*Ca_Kabuli_Chr07*	41339748	(C/A)
CakSNP13182	*Kabuli*	*Ca_Kabuli_Chr07*	41349604	(T/C)
CakSNP13183	*Kabuli*	*Ca_Kabuli_Chr07*	41387509	(A/C)
CakSNP13184	*Kabuli*	*Ca_Kabuli_Chr07*	41387502	(A/C)
CakSNP13185	*Kabuli*	*Ca_Kabuli_Chr07*	41387475	(A/C)
CakSNP13186	*Kabuli*	*Ca_Kabuli_Chr07*	41657180	(G/T)
CakSNP13187	*Kabuli*	*Ca_Kabuli_Chr07*	41657241	(T/C)
CakSNP13188	*Kabuli*	*Ca_Kabuli_Chr07*	41821410	(T/C)
CakSNP13189	*Kabuli*	*Ca_Kabuli_Chr07*	42166004	(C/T)
CakSNP13190	*Kabuli*	*Ca_Kabuli_Chr07*	42268628	(A/C)
CakSNP13191	*Kabuli*	*Ca_Kabuli_Chr07*	42272939	(G/C)
CakSNP13192	*Kabuli*	*Ca_Kabuli_Chr07*	42355002	(T/G)
CakSNP13193	*Kabuli*	*Ca_Kabuli_Chr07*	42354958	(G/A)
CakSNP13194	*Kabuli*	*Ca_Kabuli_Chr07*	42354938	(C/T)
CakSNP13195	*Kabuli*	*Ca_Kabuli_Chr07*	42354999	(C/T)
CakSNP13196	*Kabuli*	*Ca_Kabuli_Chr07*	42355011	(G/T)
CakSNP13197	*Kabuli*	*Ca_Kabuli_Chr07*	42566717	(A/G)
CakSNP13198	*Kabuli*	*Ca_Kabuli_Chr07*	42846882	(C/T)
CakSNP13199	*Kabuli*	*Ca_Kabuli_Chr07*	42851182	(T/C)
CakSNP13200	*Kabuli*	*Ca_Kabuli_Chr07*	43080817	(T/C)
CakSNP13201	*Kabuli*	*Ca_Kabuli_Chr07*	43118954	(A/C)

SNP IDs	Cultivars	Chromosomes/scaffolds	Physical positions (bp)	SNPs
CakSNP13202	*Kabuli*	*Ca_Kabuli_Chr07*	43119266	(C/T)
CakSNP13203	*Kabuli*	*Ca_Kabuli_Chr07*	43210821	(A/T)
CakSNP13204	*Kabuli*	*Ca_Kabuli_Chr07*	43646117	(G/A)
CakSNP13205	*Kabuli*	*Ca_Kabuli_Chr07*	44232239	(G/A)
CakSNP13206	*Kabuli*	*Ca_Kabuli_Chr07*	44232204	(T/A)
CakSNP13207	*Kabuli*	*Ca_Kabuli_Chr07*	44232221	(C/T)
CakSNP13208	*Kabuli*	*Ca_Kabuli_Chr07*	44232266	(C/T)
CakSNP13209	*Kabuli*	*Ca_Kabuli_Chr07*	44540553	(T/G)
CakSNP13210	*Kabuli*	*Ca_Kabuli_Chr07*	44540595	(G/A)
CakSNP13211	*Kabuli*	*Ca_Kabuli_Chr07*	44540596	(C/A)
CakSNP13212	*Kabuli*	*Ca_Kabuli_Chr07*	44540629	(G/A)
CakSNP13213	*Kabuli*	*Ca_Kabuli_Chr07*	44540616	(C/A)
CakSNP13214	*Kabuli*	*Ca_Kabuli_Chr07*	44612651	(C/A)
CakSNP13215	*Kabuli*	*Ca_Kabuli_Chr07*	44747668	(C/T)
CakSNP13216	*Kabuli*	*Ca_Kabuli_Chr07*	44765450	(C/T)
CakSNP13217	*Kabuli*	*Ca_Kabuli_Chr07*	44765467	(G/T)
CakSNP13218	*Kabuli*	*Ca_Kabuli_Chr07*	44765522	(A/C)
CakSNP13219	*Kabuli*	*Ca_Kabuli_Chr07*	44877324	(A/C)
CakSNP13220	*Kabuli*	*Ca_Kabuli_Chr07*	44926768	(C/T)
CakSNP13221	*Kabuli*	*Ca_Kabuli_Chr07*	44926783	(C/A)
CakSNP13222	*Kabuli*	*Ca_Kabuli_Chr07*	44926815	(C/T)
CakSNP13223	*Kabuli*	*Ca_Kabuli_Chr07*	45147550	(A/G)
CakSNP13224	*Kabuli*	*Ca_Kabuli_Chr07*	45280659	(T/A)
CakSNP13225	*Kabuli*	*Ca_Kabuli_Chr07*	45289633	(T/C)
CakSNP13226	*Kabuli*	*Ca_Kabuli_Chr07*	45936581	(C/A)
CakSNP13227	*Kabuli*	*Ca_Kabuli_Chr07*	46053109	(G/A)
CakSNP13228	*Kabuli*	*Ca_Kabuli_Chr07*	46394656	(A/G)
CakSNP13229	*Kabuli*	*Ca_Kabuli_Chr07*	46485934	(A/G)
CakSNP13230	*Kabuli*	*Ca_Kabuli_Chr07*	46747255	(A/C)
CakSNP13231	*Kabuli*	*Ca_Kabuli_Chr07*	46747283	(T/C)
CakSNP13232	*Kabuli*	*Ca_Kabuli_Chr07*	46747290	(G/T)
CakSNP13233	*Kabuli*	*Ca_Kabuli_Chr07*	46747295	(G/C)
CakSNP13234	*Kabuli*	*Ca_Kabuli_Chr07*	46747309	(C/T)
CakSNP13235	*Kabuli*	*Ca_Kabuli_Chr07*	46747330	(G/A)
CakSNP13236	*Kabuli*	*Ca_Kabuli_Chr07*	46823471	(T/G)
CakSNP13237	*Kabuli*	*Ca_Kabuli_Chr07*	46823949	(G/T)
CakSNP13238	*Kabuli*	*Ca_Kabuli_Chr07*	46826036	(T/A)
CakSNP13239	*Kabuli*	*Ca_Kabuli_Chr07*	46826030	(T/C)
CakSNP13240	*Kabuli*	*Ca_Kabuli_Chr07*	46826028	(C/A)
CakSNP13241	*Kabuli*	*Ca_Kabuli_Chr07*	46826020	(G/A)
CakSNP13242	*Kabuli*	*Ca_Kabuli_Chr07*	46826015	(G/A)

SNP IDs	Cultivars	Chromosomes/scaffolds	Physical positions (bp)	SNPs
CakSNP13243	*Kabuli*	*Ca_Kabuli_Chr07*	46826008	(A/T)
CakSNP13244	*Kabuli*	*Ca_Kabuli_Chr07*	46825997	(A/G)
CakSNP13245	*Kabuli*	*Ca_Kabuli_Chr07*	46825992	(T/G)
CakSNP13246	*Kabuli*	*Ca_Kabuli_Chr07*	46825985	(T/G)
CakSNP13247	*Kabuli*	*Ca_Kabuli_Chr07*	46825957	(A/T)
CakSNP13248	*Kabuli*	*Ca_Kabuli_Chr07*	46825952	(A/G)
CakSNP13249	*Kabuli*	*Ca_Kabuli_Chr07*	47127331	(T/G)
CakSNP13250	*Kabuli*	*Ca_Kabuli_Chr07*	47127342	(T/G)
CakSNP13251	*Kabuli*	*Ca_Kabuli_Chr07*	47127343	(C/T)
CakSNP13252	*Kabuli*	*Ca_Kabuli_Chr07*	47159646	(A/C)
CakSNP13253	*Kabuli*	*Ca_Kabuli_Chr07*	47335940	(G/A)
CakSNP13254	*Kabuli*	*Ca_Kabuli_Chr07*	47335916	(C/G)
CakSNP13255	*Kabuli*	*Ca_Kabuli_Chr07*	47524173	(A/C)
CakSNP13256	*Kabuli*	*Ca_Kabuli_Chr07*	47554330	(C/G)
CakSNP13257	*Kabuli*	*Ca_Kabuli_Chr07*	47694706	(A/C)
CakSNP13258	*Kabuli*	*Ca_Kabuli_Chr07*	47839934	(G/A)
CakSNP13259	*Kabuli*	*Ca_Kabuli_Chr07*	47839952	(G/T)
CakSNP13260	*Kabuli*	*Ca_Kabuli_Chr07*	47840034	(A/G)
CakSNP13261	*Kabuli*	*Ca_Kabuli_Chr07*	47840092	(A/C)
CakSNP13262	*Kabuli*	*Ca_Kabuli_Chr07*	48102586	(G/T)
CakSNP13263	*Kabuli*	*Ca_Kabuli_Chr07*	48200865	(A/G)
CakSNP13264	*Kabuli*	*Ca_Kabuli_Chr07*	48200852	(G/A)
CakSNP13265	*Kabuli*	*Ca_Kabuli_Chr07*	48200822	(G/A)
CakSNP13266	*Kabuli*	*Ca_Kabuli_Chr07*	48200820	(A/C)
CakSNP13267	*Kabuli*	*Ca_Kabuli_Chr07*	48200823	(G/A)
CakSNP13268	*Kabuli*	*Ca_Kabuli_Chr07*	48200848	(T/G)
CakSNP13269	*Kabuli*	*Ca_Kabuli_Chr07*	48509506	(G/T)
CakSNP13270	*Kabuli*	*Ca_Kabuli_Chr08*	54095	(A/C)
CakSNP13271	*Kabuli*	*Ca_Kabuli_Chr08*	85693	(T/G)
CakSNP13272	*Kabuli*	*Ca_Kabuli_Chr08*	85697	(G/A)
CakSNP13273	*Kabuli*	*Ca_Kabuli_Chr08*	85707	(C/A)
CakSNP13274	*Kabuli*	*Ca_Kabuli_Chr08*	85710	(C/A)
CakSNP13275	*Kabuli*	*Ca_Kabuli_Chr08*	158986	(C/G)
CakSNP13276	*Kabuli*	*Ca_Kabuli_Chr08*	177457	(C/T)
CakSNP13277	*Kabuli*	*Ca_Kabuli_Chr08*	177436	(A/G)
CakSNP13278	*Kabuli*	*Ca_Kabuli_Chr08*	177405	(G/A)
CakSNP13279	*Kabuli*	*Ca_Kabuli_Chr08*	184444	(G/A)
CakSNP13280	*Kabuli*	*Ca_Kabuli_Chr08*	218813	(C/T)
CakSNP13281	*Kabuli*	*Ca_Kabuli_Chr08*	218828	(T/G)
CakSNP13282	*Kabuli*	*Ca_Kabuli_Chr08*	218957	(G/T)
CakSNP13283	*Kabuli*	*Ca_Kabuli_Chr08*	218928	(A/G)

SNP IDs	Cultivars	Chromosomes/scaffolds	Physical positions (bp)	SNPs
CakSNP13284	*Kabuli*	*Ca_Kabuli_Chr08*	249654	(A/C)
CakSNP13285	*Kabuli*	*Ca_Kabuli_Chr08*	253786	(G/A)
CakSNP13286	*Kabuli*	*Ca_Kabuli_Chr08*	281622	(G/A)
CakSNP13287	*Kabuli*	*Ca_Kabuli_Chr08*	281608	(T/A)
CakSNP13288	*Kabuli*	*Ca_Kabuli_Chr08*	281630	(A/C)
CakSNP13289	*Kabuli*	*Ca_Kabuli_Chr08*	283147	(C/T)
CakSNP13290	*Kabuli*	*Ca_Kabuli_Chr08*	285779	(G/A)
CakSNP13291	*Kabuli*	*Ca_Kabuli_Chr08*	356295	(A/T)
CakSNP13292	*Kabuli*	*Ca_Kabuli_Chr08*	411967	(G/A)
CakSNP13293	*Kabuli*	*Ca_Kabuli_Chr08*	499819	(A/C)
CakSNP13294	*Kabuli*	*Ca_Kabuli_Chr08*	499890	(T/C)
CakSNP13295	*Kabuli*	*Ca_Kabuli_Chr08*	510824	(G/A)
CakSNP13296	*Kabuli*	*Ca_Kabuli_Chr08*	560477	(C/T)
CakSNP13297	*Kabuli*	*Ca_Kabuli_Chr08*	560451	(C/T)
CakSNP13298	*Kabuli*	*Ca_Kabuli_Chr08*	560604	(T/G)
CakSNP13299	*Kabuli*	*Ca_Kabuli_Chr08*	560746	(A/G)
CakSNP13300	*Kabuli*	*Ca_Kabuli_Chr08*	566112	(A/G)
CakSNP13301	*Kabuli*	*Ca_Kabuli_Chr08*	566107	(C/T)
CakSNP13302	*Kabuli*	*Ca_Kabuli_Chr08*	566094	(A/G)
CakSNP13303	*Kabuli*	*Ca_Kabuli_Chr08*	596171	(C/T)
CakSNP13304	*Kabuli*	*Ca_Kabuli_Chr08*	614324	(G/A)
CakSNP13305	*Kabuli*	*Ca_Kabuli_Chr08*	614301	(C/A)
CakSNP13306	*Kabuli*	*Ca_Kabuli_Chr08*	619605	(T/C)
CakSNP13307	*Kabuli*	*Ca_Kabuli_Chr08*	634862	(C/T)
CakSNP13308	*Kabuli*	*Ca_Kabuli_Chr08*	634843	(G/A)
CakSNP13309	*Kabuli*	*Ca_Kabuli_Chr08*	634818	(G/T)
CakSNP13310	*Kabuli*	*Ca_Kabuli_Chr08*	634921	(C/G)
CakSNP13311	*Kabuli*	*Ca_Kabuli_Chr08*	649491	(A/C)
CakSNP13312	*Kabuli*	*Ca_Kabuli_Chr08*	660610	(T/G)
CakSNP13313	*Kabuli*	*Ca_Kabuli_Chr08*	666294	(G/A)
CakSNP13314	*Kabuli*	*Ca_Kabuli_Chr08*	666288	(A/T)
CakSNP13315	*Kabuli*	*Ca_Kabuli_Chr08*	666284	(T/A)
CakSNP13316	*Kabuli*	*Ca_Kabuli_Chr08*	666274	(T/G)
CakSNP13317	*Kabuli*	*Ca_Kabuli_Chr08*	672962	(C/T)
CakSNP13318	*Kabuli*	*Ca_Kabuli_Chr08*	673820	(A/G)
CakSNP13319	*Kabuli*	*Ca_Kabuli_Chr08*	674902	(T/G)
CakSNP13320	*Kabuli*	*Ca_Kabuli_Chr08*	701803	(C/T)
CakSNP13321	*Kabuli*	*Ca_Kabuli_Chr08*	701811	(T/G)
CakSNP13322	*Kabuli*	*Ca_Kabuli_Chr08*	701837	(G/A)
CakSNP13323	*Kabuli*	*Ca_Kabuli_Chr08*	706514	(T/C)
CakSNP13324	*Kabuli*	*Ca_Kabuli_Chr08*	706482	(G/C)

**SNP IDs**	**Cultivars**	**Chromosomes/scaffolds**	**Physical positions (bp)**	**SNPs**
CakSNP13325	*Kabuli*	*Ca_Kabuli_Chr08*	706614	(C/T)
CakSNP13326	*Kabuli*	*Ca_Kabuli_Chr08*	706793	(A/G)
CakSNP13327	*Kabuli*	*Ca_Kabuli_Chr08*	706800	(G/A)
CakSNP13328	*Kabuli*	*Ca_Kabuli_Chr08*	709063	(T/C)
CakSNP13329	*Kabuli*	*Ca_Kabuli_Chr08*	709094	(T/C)
CakSNP13330	*Kabuli*	*Ca_Kabuli_Chr08*	709124	(A/T)
CakSNP13331	*Kabuli*	*Ca_Kabuli_Chr08*	714967	(A/G)
CakSNP13332	*Kabuli*	*Ca_Kabuli_Chr08*	714974	(G/A)
CakSNP13333	*Kabuli*	*Ca_Kabuli_Chr08*	777760	(A/T)
CakSNP13334	*Kabuli*	*Ca_Kabuli_Chr08*	777772	(G/A)
CakSNP13335	*Kabuli*	*Ca_Kabuli_Chr08*	777776	(C/G)
CakSNP13336	*Kabuli*	*Ca_Kabuli_Chr08*	779589	(C/G)
CakSNP13337	*Kabuli*	*Ca_Kabuli_Chr08*	779611	(T/C)
CakSNP13338	*Kabuli*	*Ca_Kabuli_Chr08*	779640	(T/C)
CakSNP13339	*Kabuli*	*Ca_Kabuli_Chr08*	817321	(T/G)
CakSNP13340	*Kabuli*	*Ca_Kabuli_Chr08*	822302	(A/G)
CakSNP13341	*Kabuli*	*Ca_Kabuli_Chr08*	822942	(T/G)
CakSNP13342	*Kabuli*	*Ca_Kabuli_Chr08*	822976	(C/T)
CakSNP13343	*Kabuli*	*Ca_Kabuli_Chr08*	839924	(C/G)
CakSNP13344	*Kabuli*	*Ca_Kabuli_Chr08*	855332	(G/A)
CakSNP13345	*Kabuli*	*Ca_Kabuli_Chr08*	866741	(A/C)
CakSNP13346	*Kabuli*	*Ca_Kabuli_Chr08*	871666	(C/G)
CakSNP13347	*Kabuli*	*Ca_Kabuli_Chr08*	875236	(T/G)
CakSNP13348	*Kabuli*	*Ca_Kabuli_Chr08*	894184	(C/A)
CakSNP13349	*Kabuli*	*Ca_Kabuli_Chr08*	895883	(G/A)
CakSNP13350	*Kabuli*	*Ca_Kabuli_Chr08*	895947	(C/T)
CakSNP13351	*Kabuli*	*Ca_Kabuli_Chr08*	897417	(T/C)
CakSNP13352	*Kabuli*	*Ca_Kabuli_Chr08*	898089	(C/T)
CakSNP13353	*Kabuli*	*Ca_Kabuli_Chr08*	898150	(C/A)
CakSNP13354	*Kabuli*	*Ca_Kabuli_Chr08*	898152	(A/G)
CakSNP13355	*Kabuli*	*Ca_Kabuli_Chr08*	898173	(G/T)
CakSNP13356	*Kabuli*	*Ca_Kabuli_Chr08*	898164	(G/T)
CakSNP13357	*Kabuli*	*Ca_Kabuli_Chr08*	898226	(C/G)
CakSNP13358	*Kabuli*	*Ca_Kabuli_Chr08*	898251	(G/T)
CakSNP13359	*Kabuli*	*Ca_Kabuli_Chr08*	919690	(T/C)
CakSNP13360	*Kabuli*	*Ca_Kabuli_Chr08*	923820	(T/C)
CakSNP13361	*Kabuli*	*Ca_Kabuli_Chr08*	924720	(G/T)
CakSNP13362	*Kabuli*	*Ca_Kabuli_Chr08*	924681	(C/T)
CakSNP13363	*Kabuli*	*Ca_Kabuli_Chr08*	924715	(A/G)
CakSNP13364	*Kabuli*	*Ca_Kabuli_Chr08*	924732	(G/T)
CakSNP13365	*Kabuli*	*Ca_Kabuli_Chr08*	974170	(T/C)

SNP IDs	Cultivars	Chromosomes/scaffolds	Physical positions (bp)	SNPs
CakSNP13366	*Kabuli*	*Ca_Kabuli_Chr08*	974159	(T/G)
CakSNP13367	*Kabuli*	*Ca_Kabuli_Chr08*	1011333	(C/T)
CakSNP13368	*Kabuli*	*Ca_Kabuli_Chr08*	1011320	(G/T)
CakSNP13369	*Kabuli*	*Ca_Kabuli_Chr08*	1011327	(G/A)
CakSNP13370	*Kabuli*	*Ca_Kabuli_Chr08*	1043905	(T/C)
CakSNP13371	*Kabuli*	*Ca_Kabuli_Chr08*	1070728	(T/C)
CakSNP13372	*Kabuli*	*Ca_Kabuli_Chr08*	1077334	(C/T)
CakSNP13373	*Kabuli*	*Ca_Kabuli_Chr08*	1077430	(C/T)
CakSNP13374	*Kabuli*	*Ca_Kabuli_Chr08*	1119568	(T/C)
CakSNP13375	*Kabuli*	*Ca_Kabuli_Chr08*	1119781	(A/G)
CakSNP13376	*Kabuli*	*Ca_Kabuli_Chr08*	1119773	(T/G)
CakSNP13377	*Kabuli*	*Ca_Kabuli_Chr08*	1119761	(G/T)
CakSNP13378	*Kabuli*	*Ca_Kabuli_Chr08*	1138275	(G/A)
CakSNP13379	*Kabuli*	*Ca_Kabuli_Chr08*	1138394	(A/G)
CakSNP13380	*Kabuli*	*Ca_Kabuli_Chr08*	1155597	(C/T)
CakSNP13381	*Kabuli*	*Ca_Kabuli_Chr08*	1155636	(T/C)
CakSNP13382	*Kabuli*	*Ca_Kabuli_Chr08*	1155639	(T/A)
CakSNP13383	*Kabuli*	*Ca_Kabuli_Chr08*	1155641	(C/G)
CakSNP13384	*Kabuli*	*Ca_Kabuli_Chr08*	1174369	(A/G)
CakSNP13385	*Kabuli*	*Ca_Kabuli_Chr08*	1193297	(C/T)
CakSNP13386	*Kabuli*	*Ca_Kabuli_Chr08*	1194886	(G/A)
CakSNP13387	*Kabuli*	*Ca_Kabuli_Chr08*	1194888	(A/G)
CakSNP13388	*Kabuli*	*Ca_Kabuli_Chr08*	1241739	(C/T)
CakSNP13389	*Kabuli*	*Ca_Kabuli_Chr08*	1262576	(T/C)
CakSNP13390	*Kabuli*	*Ca_Kabuli_Chr08*	1262561	(C/T)
CakSNP13391	*Kabuli*	*Ca_Kabuli_Chr08*	1262660	(C/A)
CakSNP13392	*Kabuli*	*Ca_Kabuli_Chr08*	1262686	(T/G)
CakSNP13393	*Kabuli*	*Ca_Kabuli_Chr08*	1265537	(G/A)
CakSNP13394	*Kabuli*	*Ca_Kabuli_Chr08*	1265535	(A/G)
CakSNP13395	*Kabuli*	*Ca_Kabuli_Chr08*	1265509	(T/C)
CakSNP13396	*Kabuli*	*Ca_Kabuli_Chr08*	1265595	(G/T)
CakSNP13397	*Kabuli*	*Ca_Kabuli_Chr08*	1265579	(A/G)
CakSNP13398	*Kabuli*	*Ca_Kabuli_Chr08*	1265577	(T/G)
CakSNP13399	*Kabuli*	*Ca_Kabuli_Chr08*	1382266	(G/A)
CakSNP13400	*Kabuli*	*Ca_Kabuli_Chr08*	1382433	(G/T)
CakSNP13401	*Kabuli*	*Ca_Kabuli_Chr08*	1382454	(A/C)
CakSNP13402	*Kabuli*	*Ca_Kabuli_Chr08*	1433436	(T/C)
CakSNP13403	*Kabuli*	*Ca_Kabuli_Chr08*	1477523	(T/C)
CakSNP13404	*Kabuli*	*Ca_Kabuli_Chr08*	1487202	(G/C)
CakSNP13405	*Kabuli*	*Ca_Kabuli_Chr08*	1503621	(C/T)
CakSNP13406	*Kabuli*	*Ca_Kabuli_Chr08*	1503595	(C/T)

SNP IDs	Cultivars	Chromosomes/scaffolds	Physical positions (bp)	SNPs
CakSNP13407	*Kabuli*	*Ca_Kabuli_Chr08*	1503588	(T/C)
CakSNP13408	*Kabuli*	*Ca_Kabuli_Chr08*	1504859	(A/G)
CakSNP13409	*Kabuli*	*Ca_Kabuli_Chr08*	1507709	(T/G)
CakSNP13410	*Kabuli*	*Ca_Kabuli_Chr08*	1507782	(G/C)
CakSNP13411	*Kabuli*	*Ca_Kabuli_Chr08*	1518285	(A/C)
CakSNP13412	*Kabuli*	*Ca_Kabuli_Chr08*	1518297	(T/G)
CakSNP13413	*Kabuli*	*Ca_Kabuli_Chr08*	1518355	(G/A)
CakSNP13414	*Kabuli*	*Ca_Kabuli_Chr08*	1534461	(A/C)
CakSNP13415	*Kabuli*	*Ca_Kabuli_Chr08*	1544916	(C/T)
CakSNP13416	*Kabuli*	*Ca_Kabuli_Chr08*	1552082	(T/A)
CakSNP13417	*Kabuli*	*Ca_Kabuli_Chr08*	1552143	(A/G)
CakSNP13418	*Kabuli*	*Ca_Kabuli_Chr08*	1554964	(T/C)
CakSNP13419	*Kabuli*	*Ca_Kabuli_Chr08*	1555017	(C/A)
CakSNP13420	*Kabuli*	*Ca_Kabuli_Chr08*	1555122	(A/G)
CakSNP13421	*Kabuli*	*Ca_Kabuli_Chr08*	1557492	(C/T)
CakSNP13422	*Kabuli*	*Ca_Kabuli_Chr08*	1573877	(G/T)
CakSNP13423	*Kabuli*	*Ca_Kabuli_Chr08*	1612107	(G/A)
CakSNP13424	*Kabuli*	*Ca_Kabuli_Chr08*	1612099	(A/G)
CakSNP13425	*Kabuli*	*Ca_Kabuli_Chr08*	1612221	(C/G)
CakSNP13426	*Kabuli*	*Ca_Kabuli_Chr08*	1667832	(T/C)
CakSNP13427	*Kabuli*	*Ca_Kabuli_Chr08*	1675895	(C/T)
CakSNP13428	*Kabuli*	*Ca_Kabuli_Chr08*	1675888	(C/T)
CakSNP13429	*Kabuli*	*Ca_Kabuli_Chr08*	1708743	(C/T)
CakSNP13430	*Kabuli*	*Ca_Kabuli_Chr08*	1708860	(T/C)
CakSNP13431	*Kabuli*	*Ca_Kabuli_Chr08*	1718032	(C/T)
CakSNP13432	*Kabuli*	*Ca_Kabuli_Chr08*	1723593	(G/A)
CakSNP13433	*Kabuli*	*Ca_Kabuli_Chr08*	1743097	(G/A)
CakSNP13434	*Kabuli*	*Ca_Kabuli_Chr08*	1747523	(T/A)
CakSNP13435	*Kabuli*	*Ca_Kabuli_Chr08*	1747522	(T/C)
CakSNP13436	*Kabuli*	*Ca_Kabuli_Chr08*	1782034	(C/G)
CakSNP13437	*Kabuli*	*Ca_Kabuli_Chr08*	1794755	(C/A)
CakSNP13438	*Kabuli*	*Ca_Kabuli_Chr08*	1794756	(G/T)
CakSNP13439	*Kabuli*	*Ca_Kabuli_Chr08*	1794759	(C/T)
CakSNP13440	*Kabuli*	*Ca_Kabuli_Chr08*	1794810	(G/A)
CakSNP13441	*Kabuli*	*Ca_Kabuli_Chr08*	1817388	(T/C)
CakSNP13442	*Kabuli*	*Ca_Kabuli_Chr08*	1830230	(A/T)
CakSNP13443	*Kabuli*	*Ca_Kabuli_Chr08*	1830348	(T/C)
CakSNP13444	*Kabuli*	*Ca_Kabuli_Chr08*	1846478	(A/G)
CakSNP13445	*Kabuli*	*Ca_Kabuli_Chr08*	1893613	(T/C)
CakSNP13446	*Kabuli*	*Ca_Kabuli_Chr08*	1932975	(T/C)
CakSNP13447	*Kabuli*	*Ca_Kabuli_Chr08*	1932981	(T/C)

**SNP IDs**	**Cultivars**	**Chromosomes/scaffolds**	**Physical positions (bp)**	**SNPs**
CakSNP13448	*Kabuli*	*Ca_Kabuli_Chr08*	1932990	(A/C)
CakSNP13449	*Kabuli*	*Ca_Kabuli_Chr08*	1932993	(C/T)
CakSNP13450	*Kabuli*	*Ca_Kabuli_Chr08*	1932997	(G/T)
CakSNP13451	*Kabuli*	*Ca_Kabuli_Chr08*	1933026	(G/A)
CakSNP13452	*Kabuli*	*Ca_Kabuli_Chr08*	1933064	(G/C)
CakSNP13453	*Kabuli*	*Ca_Kabuli_Chr08*	1933053	(G/A)
CakSNP13454	*Kabuli*	*Ca_Kabuli_Chr08*	1933052	(T/C)
CakSNP13455	*Kabuli*	*Ca_Kabuli_Chr08*	1933039	(G/T)
CakSNP13456	*Kabuli*	*Ca_Kabuli_Chr08*	1933035	(T/A)
CakSNP13457	*Kabuli*	*Ca_Kabuli_Chr08*	1958433	(G/C)
CakSNP13458	*Kabuli*	*Ca_Kabuli_Chr08*	1958415	(T/G)
CakSNP13459	*Kabuli*	*Ca_Kabuli_Chr08*	1978964	(G/C)
CakSNP13460	*Kabuli*	*Ca_Kabuli_Chr08*	1980189	(T/A)
CakSNP13461	*Kabuli*	*Ca_Kabuli_Chr08*	2004509	(A/G)
CakSNP13462	*Kabuli*	*Ca_Kabuli_Chr08*	2004577	(G/C)
CakSNP13463	*Kabuli*	*Ca_Kabuli_Chr08*	2029405	(C/A)
CakSNP13464	*Kabuli*	*Ca_Kabuli_Chr08*	2029384	(T/C)
CakSNP13465	*Kabuli*	*Ca_Kabuli_Chr08*	2052263	(T/G)
CakSNP13466	*Kabuli*	*Ca_Kabuli_Chr08*	2052286	(T/C)
CakSNP13467	*Kabuli*	*Ca_Kabuli_Chr08*	2070694	(G/T)
CakSNP13468	*Kabuli*	*Ca_Kabuli_Chr08*	2073807	(C/G)
CakSNP13469	*Kabuli*	*Ca_Kabuli_Chr08*	2074619	(T/G)
CakSNP13470	*Kabuli*	*Ca_Kabuli_Chr08*	2075296	(T/C)
CakSNP13471	*Kabuli*	*Ca_Kabuli_Chr08*	2075290	(G/T)
CakSNP13472	*Kabuli*	*Ca_Kabuli_Chr08*	2075278	(T/G)
CakSNP13473	*Kabuli*	*Ca_Kabuli_Chr08*	2079723	(A/G)
CakSNP13474	*Kabuli*	*Ca_Kabuli_Chr08*	2081228	(C/T)
CakSNP13475	*Kabuli*	*Ca_Kabuli_Chr08*	2088903	(T/C)
CakSNP13476	*Kabuli*	*Ca_Kabuli_Chr08*	2088892	(T/G)
CakSNP13477	*Kabuli*	*Ca_Kabuli_Chr08*	2090852	(A/G)
CakSNP13478	*Kabuli*	*Ca_Kabuli_Chr08*	2095607	(A/G)
CakSNP13479	*Kabuli*	*Ca_Kabuli_Chr08*	2107370	(C/T)
CakSNP13480	*Kabuli*	*Ca_Kabuli_Chr08*	2113823	(T/G)
CakSNP13481	*Kabuli*	*Ca_Kabuli_Chr08*	2113816	(T/C)
CakSNP13482	*Kabuli*	*Ca_Kabuli_Chr08*	2128450	(A/G)
CakSNP13483	*Kabuli*	*Ca_Kabuli_Chr08*	2194927	(G/A)
CakSNP13484	*Kabuli*	*Ca_Kabuli_Chr08*	2194898	(A/T)
CakSNP13485	*Kabuli*	*Ca_Kabuli_Chr08*	2250195	(C/G)
CakSNP13486	*Kabuli*	*Ca_Kabuli_Chr08*	2250148	(T/C)
CakSNP13487	*Kabuli*	*Ca_Kabuli_Chr08*	2264108	(G/A)
CakSNP13488	*Kabuli*	*Ca_Kabuli_Chr08*	2279413	(G/A)

SNP IDs	Cultivars	Chromosomes/scaffolds	Physical positions (bp)	SNPs
CakSNP13489	*Kabuli*	*Ca_Kabuli_Chr08*	2309552	(C/G)
CakSNP13490	*Kabuli*	*Ca_Kabuli_Chr08*	2309549	(A/G)
CakSNP13491	*Kabuli*	*Ca_Kabuli_Chr08*	2413731	(A/G)
CakSNP13492	*Kabuli*	*Ca_Kabuli_Chr08*	2456352	(T/C)
CakSNP13493	*Kabuli*	*Ca_Kabuli_Chr08*	2482834	(A/G)
CakSNP13494	*Kabuli*	*Ca_Kabuli_Chr08*	2482849	(T/C)
CakSNP13495	*Kabuli*	*Ca_Kabuli_Chr08*	2483021	(G/A)
CakSNP13496	*Kabuli*	*Ca_Kabuli_Chr08*	2488709	(A/G)
CakSNP13497	*Kabuli*	*Ca_Kabuli_Chr08*	2488769	(C/G)
CakSNP13498	*Kabuli*	*Ca_Kabuli_Chr08*	2488772	(A/G)
CakSNP13499	*Kabuli*	*Ca_Kabuli_Chr08*	2515612	(A/G)
CakSNP13500	*Kabuli*	*Ca_Kabuli_Chr08*	2524188	(A/G)
CakSNP13501	*Kabuli*	*Ca_Kabuli_Chr08*	2524148	(A/G)
CakSNP13502	*Kabuli*	*Ca_Kabuli_Chr08*	2539375	(G/A)
CakSNP13503	*Kabuli*	*Ca_Kabuli_Chr08*	2540644	(G/A)
CakSNP13504	*Kabuli*	*Ca_Kabuli_Chr08*	2540704	(A/T)
CakSNP13505	*Kabuli*	*Ca_Kabuli_Chr08*	2543156	(A/G)
CakSNP13506	*Kabuli*	*Ca_Kabuli_Chr08*	2545070	(T/A)
CakSNP13507	*Kabuli*	*Ca_Kabuli_Chr08*	2604759	(C/T)
CakSNP13508	*Kabuli*	*Ca_Kabuli_Chr08*	2605423	(A/T)
CakSNP13509	*Kabuli*	*Ca_Kabuli_Chr08*	2605460	(C/T)
CakSNP13510	*Kabuli*	*Ca_Kabuli_Chr08*	2621132	(T/A)
CakSNP13511	*Kabuli*	*Ca_Kabuli_Chr08*	2622009	(G/T)
CakSNP13512	*Kabuli*	*Ca_Kabuli_Chr08*	2621987	(C/G)
CakSNP13513	*Kabuli*	*Ca_Kabuli_Chr08*	2621973	(T/G)
CakSNP13514	*Kabuli*	*Ca_Kabuli_Chr08*	2651439	(G/T)
CakSNP13515	*Kabuli*	*Ca_Kabuli_Chr08*	2651425	(C/T)
CakSNP13516	*Kabuli*	*Ca_Kabuli_Chr08*	2651422	(T/C)
CakSNP13517	*Kabuli*	*Ca_Kabuli_Chr08*	2651393	(G/T)
CakSNP13518	*Kabuli*	*Ca_Kabuli_Chr08*	2651373	(G/T)
CakSNP13519	*Kabuli*	*Ca_Kabuli_Chr08*	2651921	(C/T)
CakSNP13520	*Kabuli*	*Ca_Kabuli_Chr08*	2664625	(T/C)
CakSNP13521	*Kabuli*	*Ca_Kabuli_Chr08*	2664711	(T/G)
CakSNP13522	*Kabuli*	*Ca_Kabuli_Chr08*	2722673	(C/A)
CakSNP13523	*Kabuli*	*Ca_Kabuli_Chr08*	2735849	(T/C)
CakSNP13524	*Kabuli*	*Ca_Kabuli_Chr08*	2743075	(T/C)
CakSNP13525	*Kabuli*	*Ca_Kabuli_Chr08*	2743088	(A/G)
CakSNP13526	*Kabuli*	*Ca_Kabuli_Chr08*	2743109	(G/C)
CakSNP13527	*Kabuli*	*Ca_Kabuli_Chr08*	2743139	(A/T)
CakSNP13528	*Kabuli*	*Ca_Kabuli_Chr08*	2783576	(T/G)
CakSNP13529	*Kabuli*	*Ca_Kabuli_Chr08*	2783655	(G/C)

SNP IDs	Cultivars	Chromosomes/scaffolds	Physical positions (bp)	SNPs
CakSNP13530	*Kabuli*	*Ca_Kabuli_Chr08*	2827822	(C/T)
CakSNP13531	*Kabuli*	*Ca_Kabuli_Chr08*	2827984	(T/C)
CakSNP13532	*Kabuli*	*Ca_Kabuli_Chr08*	2838179	(T/A)
CakSNP13533	*Kabuli*	*Ca_Kabuli_Chr08*	2871095	(G/A)
CakSNP13534	*Kabuli*	*Ca_Kabuli_Chr08*	2871064	(G/A)
CakSNP13535	*Kabuli*	*Ca_Kabuli_Chr08*	2888353	(G/T)
CakSNP13536	*Kabuli*	*Ca_Kabuli_Chr08*	2888317	(G/A)
CakSNP13537	*Kabuli*	*Ca_Kabuli_Chr08*	2943631	(G/T)
CakSNP13538	*Kabuli*	*Ca_Kabuli_Chr08*	2947498	(A/G)
CakSNP13539	*Kabuli*	*Ca_Kabuli_Chr08*	2974759	(G/C)
CakSNP13540	*Kabuli*	*Ca_Kabuli_Chr08*	2976709	(C/T)
CakSNP13541	*Kabuli*	*Ca_Kabuli_Chr08*	2977543	(T/C)
CakSNP13542	*Kabuli*	*Ca_Kabuli_Chr08*	2977628	(C/T)
CakSNP13543	*Kabuli*	*Ca_Kabuli_Chr08*	2977603	(T/A)
CakSNP13544	*Kabuli*	*Ca_Kabuli_Chr08*	3013928	(G/C)
CakSNP13545	*Kabuli*	*Ca_Kabuli_Chr08*	3013917	(G/A)
CakSNP13546	*Kabuli*	*Ca_Kabuli_Chr08*	3041947	(A/G)
CakSNP13547	*Kabuli*	*Ca_Kabuli_Chr08*	3041981	(A/G)
CakSNP13548	*Kabuli*	*Ca_Kabuli_Chr08*	3050452	(T/C)
CakSNP13549	*Kabuli*	*Ca_Kabuli_Chr08*	3064477	(G/A)
CakSNP13550	*Kabuli*	*Ca_Kabuli_Chr08*	3141617	(C/T)
CakSNP13551	*Kabuli*	*Ca_Kabuli_Chr08*	3171324	(C/T)
CakSNP13552	*Kabuli*	*Ca_Kabuli_Chr08*	3210879	(C/A)
CakSNP13553	*Kabuli*	*Ca_Kabuli_Chr08*	3248044	(A/G)
CakSNP13554	*Kabuli*	*Ca_Kabuli_Chr08*	3248104	(A/G)
CakSNP13555	*Kabuli*	*Ca_Kabuli_Chr08*	3250707	(C/T)
CakSNP13556	*Kabuli*	*Ca_Kabuli_Chr08*	3263822	(T/C)
CakSNP13557	*Kabuli*	*Ca_Kabuli_Chr08*	3269124	(G/A)
CakSNP13558	*Kabuli*	*Ca_Kabuli_Chr08*	3269118	(A/G)
CakSNP13559	*Kabuli*	*Ca_Kabuli_Chr08*	3269088	(A/C)
CakSNP13560	*Kabuli*	*Ca_Kabuli_Chr08*	3269085	(A/C)
CakSNP13561	*Kabuli*	*Ca_Kabuli_Chr08*	3297410	(T/C)
CakSNP13562	*Kabuli*	*Ca_Kabuli_Chr08*	3386165	(C/A)
CakSNP13563	*Kabuli*	*Ca_Kabuli_Chr08*	3386181	(C/A)
CakSNP13564	*Kabuli*	*Ca_Kabuli_Chr08*	3392929	(A/C)
CakSNP13565	*Kabuli*	*Ca_Kabuli_Chr08*	3394796	(A/C)
CakSNP13566	*Kabuli*	*Ca_Kabuli_Chr08*	3394833	(T/C)
CakSNP13567	*Kabuli*	*Ca_Kabuli_Chr08*	3394931	(T/C)
CakSNP13568	*Kabuli*	*Ca_Kabuli_Chr08*	3396820	(T/C)
CakSNP13569	*Kabuli*	*Ca_Kabuli_Chr08*	3438274	(A/T)
CakSNP13570	*Kabuli*	*Ca_Kabuli_Chr08*	3445568	(G/T)

**SNP IDs**	**Cultivars**	**Chromosomes/scaffolds**	**Physical positions (bp)**	**SNPs**
CakSNP13571	*Kabuli*	*Ca_Kabuli_Chr08*	3448194	(C/T)
CakSNP13572	*Kabuli*	*Ca_Kabuli_Chr08*	3460627	(G/A)
CakSNP13573	*Kabuli*	*Ca_Kabuli_Chr08*	3499010	(G/A)
CakSNP13574	*Kabuli*	*Ca_Kabuli_Chr08*	3539690	(A/G)
CakSNP13575	*Kabuli*	*Ca_Kabuli_Chr08*	3539723	(G/C)
CakSNP13576	*Kabuli*	*Ca_Kabuli_Chr08*	3539745	(T/C)
CakSNP13577	*Kabuli*	*Ca_Kabuli_Chr08*	3539851	(T/C)
CakSNP13578	*Kabuli*	*Ca_Kabuli_Chr08*	3539838	(T/G)
CakSNP13579	*Kabuli*	*Ca_Kabuli_Chr08*	3539832	(T/C)
CakSNP13580	*Kabuli*	*Ca_Kabuli_Chr08*	3560271	(G/A)
CakSNP13581	*Kabuli*	*Ca_Kabuli_Chr08*	3563692	(A/C)
CakSNP13582	*Kabuli*	*Ca_Kabuli_Chr08*	3563825	(G/T)
CakSNP13583	*Kabuli*	*Ca_Kabuli_Chr08*	3563824	(T/A)
CakSNP13584	*Kabuli*	*Ca_Kabuli_Chr08*	3599091	(G/C)
CakSNP13585	*Kabuli*	*Ca_Kabuli_Chr08*	3601517	(T/C)
CakSNP13586	*Kabuli*	*Ca_Kabuli_Chr08*	3605513	(T/C)
CakSNP13587	*Kabuli*	*Ca_Kabuli_Chr08*	3617810	(T/C)
CakSNP13588	*Kabuli*	*Ca_Kabuli_Chr08*	3617757	(G/C)
CakSNP13589	*Kabuli*	*Ca_Kabuli_Chr08*	3723085	(C/T)
CakSNP13590	*Kabuli*	*Ca_Kabuli_Chr08*	3730369	(A/C)
CakSNP13591	*Kabuli*	*Ca_Kabuli_Chr08*	3775945	(G/A)
CakSNP13592	*Kabuli*	*Ca_Kabuli_Chr08*	3775960	(T/C)
CakSNP13593	*Kabuli*	*Ca_Kabuli_Chr08*	3813978	(C/T)
CakSNP13594	*Kabuli*	*Ca_Kabuli_Chr08*	3829425	(C/G)
CakSNP13595	*Kabuli*	*Ca_Kabuli_Chr08*	3829576	(A/G)
CakSNP13596	*Kabuli*	*Ca_Kabuli_Chr08*	3837237	(A/G)
CakSNP13597	*Kabuli*	*Ca_Kabuli_Chr08*	3837306	(G/C)
CakSNP13598	*Kabuli*	*Ca_Kabuli_Chr08*	3868511	(A/C)
CakSNP13599	*Kabuli*	*Ca_Kabuli_Chr08*	3877130	(G/T)
CakSNP13600	*Kabuli*	*Ca_Kabuli_Chr08*	3877176	(T/G)
CakSNP13601	*Kabuli*	*Ca_Kabuli_Chr08*	3911930	(A/C)
CakSNP13602	*Kabuli*	*Ca_Kabuli_Chr08*	3911876	(G/C)
CakSNP13603	*Kabuli*	*Ca_Kabuli_Chr08*	3951767	(C/A)
CakSNP13604	*Kabuli*	*Ca_Kabuli_Chr08*	3951768	(A/G)
CakSNP13605	*Kabuli*	*Ca_Kabuli_Chr08*	3954853	(T/A)
CakSNP13606	*Kabuli*	*Ca_Kabuli_Chr08*	4042166	(A/C)
CakSNP13607	*Kabuli*	*Ca_Kabuli_Chr08*	4042233	(G/A)
CakSNP13608	*Kabuli*	*Ca_Kabuli_Chr08*	4042424	(G/A)
CakSNP13609	*Kabuli*	*Ca_Kabuli_Chr08*	4052732	(C/T)
CakSNP13610	*Kabuli*	*Ca_Kabuli_Chr08*	4052702	(C/T)
CakSNP13611	*Kabuli*	*Ca_Kabuli_Chr08*	4073528	(C/T)

SNP IDs	Cultivars	Chromosomes/scaffolds	Physical positions (bp)	SNPs
CakSNP13612	*Kabuli*	*Ca_Kabuli_Chr08*	4073603	(A/G)
CakSNP13613	*Kabuli*	*Ca_Kabuli_Chr08*	4074135	(T/C)
CakSNP13614	*Kabuli*	*Ca_Kabuli_Chr08*	4091946	(T/C)
CakSNP13615	*Kabuli*	*Ca_Kabuli_Chr08*	4092348	(T/A)
CakSNP13616	*Kabuli*	*Ca_Kabuli_Chr08*	4092350	(G/C)
CakSNP13617	*Kabuli*	*Ca_Kabuli_Chr08*	4095131	(A/C)
CakSNP13618	*Kabuli*	*Ca_Kabuli_Chr08*	4095119	(G/A)
CakSNP13619	*Kabuli*	*Ca_Kabuli_Chr08*	4106580	(T/C)
CakSNP13620	*Kabuli*	*Ca_Kabuli_Chr08*	4106583	(T/C)
CakSNP13621	*Kabuli*	*Ca_Kabuli_Chr08*	4106644	(C/T)
CakSNP13622	*Kabuli*	*Ca_Kabuli_Chr08*	4172342	(A/G)
CakSNP13623	*Kabuli*	*Ca_Kabuli_Chr08*	4172368	(T/C)
CakSNP13624	*Kabuli*	*Ca_Kabuli_Chr08*	4183744	(A/G)
CakSNP13625	*Kabuli*	*Ca_Kabuli_Chr08*	4183733	(G/A)
CakSNP13626	*Kabuli*	*Ca_Kabuli_Chr08*	4198214	(A/C)
CakSNP13627	*Kabuli*	*Ca_Kabuli_Chr08*	4201285	(A/G)
CakSNP13628	*Kabuli*	*Ca_Kabuli_Chr08*	4237620	(T/A)
CakSNP13629	*Kabuli*	*Ca_Kabuli_Chr08*	4271620	(T/A)
CakSNP13630	*Kabuli*	*Ca_Kabuli_Chr08*	4290690	(T/A)
CakSNP13631	*Kabuli*	*Ca_Kabuli_Chr08*	4322058	(A/G)
CakSNP13632	*Kabuli*	*Ca_Kabuli_Chr08*	4322202	(C/T)
CakSNP13633	*Kabuli*	*Ca_Kabuli_Chr08*	4344459	(C/A)
CakSNP13634	*Kabuli*	*Ca_Kabuli_Chr08*	4346552	(A/G)
CakSNP13635	*Kabuli*	*Ca_Kabuli_Chr08*	4346749	(A/G)
CakSNP13636	*Kabuli*	*Ca_Kabuli_Chr08*	4346811	(C/T)
CakSNP13637	*Kabuli*	*Ca_Kabuli_Chr08*	4346827	(A/C)
CakSNP13638	*Kabuli*	*Ca_Kabuli_Chr08*	4347535	(A/C)
CakSNP13639	*Kabuli*	*Ca_Kabuli_Chr08*	4351751	(G/A)
CakSNP13640	*Kabuli*	*Ca_Kabuli_Chr08*	4353397	(A/C)
CakSNP13641	*Kabuli*	*Ca_Kabuli_Chr08*	4355388	(G/T)
CakSNP13642	*Kabuli*	*Ca_Kabuli_Chr08*	4355531	(A/C)
CakSNP13643	*Kabuli*	*Ca_Kabuli_Chr08*	4368163	(T/C)
CakSNP13644	*Kabuli*	*Ca_Kabuli_Chr08*	4368184	(T/C)
CakSNP13645	*Kabuli*	*Ca_Kabuli_Chr08*	4368213	(T/C)
CakSNP13646	*Kabuli*	*Ca_Kabuli_Chr08*	4368403	(G/A)
CakSNP13647	*Kabuli*	*Ca_Kabuli_Chr08*	4368463	(A/G)
CakSNP13648	*Kabuli*	*Ca_Kabuli_Chr08*	4392707	(T/C)
CakSNP13649	*Kabuli*	*Ca_Kabuli_Chr08*	4392767	(T/G)
CakSNP13650	*Kabuli*	*Ca_Kabuli_Chr08*	4392818	(C/A)
CakSNP13651	*Kabuli*	*Ca_Kabuli_Chr08*	4406956	(C/G)
CakSNP13652	*Kabuli*	*Ca_Kabuli_Chr08*	4421609	(A/G)

SNP IDs	Cultivars	Chromosomes/scaffolds	Physical positions (bp)	SNPs
CakSNP13653	*Kabuli*	*Ca_Kabuli_Chr08*	4421629	(T/A)
CakSNP13654	*Kabuli*	*Ca_Kabuli_Chr08*	4472423	(A/T)
CakSNP13655	*Kabuli*	*Ca_Kabuli_Chr08*	4483130	(T/C)
CakSNP13656	*Kabuli*	*Ca_Kabuli_Chr08*	4483274	(T/C)
CakSNP13657	*Kabuli*	*Ca_Kabuli_Chr08*	4484606	(T/A)
CakSNP13658	*Kabuli*	*Ca_Kabuli_Chr08*	4507729	(G/T)
CakSNP13659	*Kabuli*	*Ca_Kabuli_Chr08*	4510845	(C/T)
CakSNP13660	*Kabuli*	*Ca_Kabuli_Chr08*	4510810	(A/G)
CakSNP13661	*Kabuli*	*Ca_Kabuli_Chr08*	4512311	(T/C)
CakSNP13662	*Kabuli*	*Ca_Kabuli_Chr08*	4512436	(G/A)
CakSNP13663	*Kabuli*	*Ca_Kabuli_Chr08*	4512492	(T/G)
CakSNP13664	*Kabuli*	*Ca_Kabuli_Chr08*	4524914	(C/T)
CakSNP13665	*Kabuli*	*Ca_Kabuli_Chr08*	4557334	(A/G)
CakSNP13666	*Kabuli*	*Ca_Kabuli_Chr08*	4557328	(T/C)
CakSNP13667	*Kabuli*	*Ca_Kabuli_Chr08*	4557401	(A/C)
CakSNP13668	*Kabuli*	*Ca_Kabuli_Chr08*	4557407	(A/C)
CakSNP13669	*Kabuli*	*Ca_Kabuli_Chr08*	4557440	(A/G)
CakSNP13670	*Kabuli*	*Ca_Kabuli_Chr08*	4557488	(C/T)
CakSNP13671	*Kabuli*	*Ca_Kabuli_Chr08*	4580363	(C/A)
CakSNP13672	*Kabuli*	*Ca_Kabuli_Chr08*	4588869	(T/C)
CakSNP13673	*Kabuli*	*Ca_Kabuli_Chr08*	4588915	(A/C)
CakSNP13674	*Kabuli*	*Ca_Kabuli_Chr08*	4611094	(C/T)
CakSNP13675	*Kabuli*	*Ca_Kabuli_Chr08*	4658502	(A/G)
CakSNP13676	*Kabuli*	*Ca_Kabuli_Chr08*	4714566	(G/A)
CakSNP13677	*Kabuli*	*Ca_Kabuli_Chr08*	4744388	(C/T)
CakSNP13678	*Kabuli*	*Ca_Kabuli_Chr08*	4767567	(A/C)
CakSNP13679	*Kabuli*	*Ca_Kabuli_Chr08*	4767586	(C/G)
CakSNP13680	*Kabuli*	*Ca_Kabuli_Chr08*	4773205	(A/C)
CakSNP13681	*Kabuli*	*Ca_Kabuli_Chr08*	4810954	(A/C)
CakSNP13682	*Kabuli*	*Ca_Kabuli_Chr08*	4810968	(G/A)
CakSNP13683	*Kabuli*	*Ca_Kabuli_Chr08*	4810986	(A/C)
CakSNP13684	*Kabuli*	*Ca_Kabuli_Chr08*	4858262	(C/A)
CakSNP13685	*Kabuli*	*Ca_Kabuli_Chr08*	4899964	(T/G)
CakSNP13686	*Kabuli*	*Ca_Kabuli_Chr08*	4899983	(G/A)
CakSNP13687	*Kabuli*	*Ca_Kabuli_Chr08*	4980949	(A/T)
CakSNP13688	*Kabuli*	*Ca_Kabuli_Chr08*	4980916	(C/T)
CakSNP13689	*Kabuli*	*Ca_Kabuli_Chr08*	5044456	(T/C)
CakSNP13690	*Kabuli*	*Ca_Kabuli_Chr08*	5044423	(T/A)
CakSNP13691	*Kabuli*	*Ca_Kabuli_Chr08*	5096194	(T/G)
CakSNP13692	*Kabuli*	*Ca_Kabuli_Chr08*	5115451	(C/G)
CakSNP13693	*Kabuli*	*Ca_Kabuli_Chr08*	5115498	(A/T)

**SNP IDs**	**Cultivars**	**Chromosomes/scaffolds**	**Physical positions (bp)**	**SNPs**
CakSNP13694	*Kabuli*	*Ca_Kabuli_Chr08*	5115512	(T/G)
CakSNP13695	*Kabuli*	*Ca_Kabuli_Chr08*	5115522	(G/C)
CakSNP13696	*Kabuli*	*Ca_Kabuli_Chr08*	5134706	(A/C)
CakSNP13697	*Kabuli*	*Ca_Kabuli_Chr08*	5184227	(C/G)
CakSNP13698	*Kabuli*	*Ca_Kabuli_Chr08*	5184276	(G/A)
CakSNP13699	*Kabuli*	*Ca_Kabuli_Chr08*	5197004	(G/T)
CakSNP13700	*Kabuli*	*Ca_Kabuli_Chr08*	5197254	(G/A)
CakSNP13701	*Kabuli*	*Ca_Kabuli_Chr08*	5204956	(A/G)
CakSNP13702	*Kabuli*	*Ca_Kabuli_Chr08*	5313792	(T/C)
CakSNP13703	*Kabuli*	*Ca_Kabuli_Chr08*	5313782	(T/G)
CakSNP13704	*Kabuli*	*Ca_Kabuli_Chr08*	5313774	(C/G)
CakSNP13705	*Kabuli*	*Ca_Kabuli_Chr08*	5313770	(A/T)
CakSNP13706	*Kabuli*	*Ca_Kabuli_Chr08*	5379556	(G/A)
CakSNP13707	*Kabuli*	*Ca_Kabuli_Chr08*	5390415	(C/T)
CakSNP13708	*Kabuli*	*Ca_Kabuli_Chr08*	5390602	(T/C)
CakSNP13709	*Kabuli*	*Ca_Kabuli_Chr08*	5399503	(G/A)
CakSNP13710	*Kabuli*	*Ca_Kabuli_Chr08*	5400188	(A/G)
CakSNP13711	*Kabuli*	*Ca_Kabuli_Chr08*	5412501	(A/G)
CakSNP13712	*Kabuli*	*Ca_Kabuli_Chr08*	5412489	(T/C)
CakSNP13713	*Kabuli*	*Ca_Kabuli_Chr08*	5434223	(C/T)
CakSNP13714	*Kabuli*	*Ca_Kabuli_Chr08*	5434225	(G/C)
CakSNP13715	*Kabuli*	*Ca_Kabuli_Chr08*	5434232	(A/G)
CakSNP13716	*Kabuli*	*Ca_Kabuli_Chr08*	5434238	(T/G)
CakSNP13717	*Kabuli*	*Ca_Kabuli_Chr08*	5434242	(T/A)
CakSNP13718	*Kabuli*	*Ca_Kabuli_Chr08*	5487769	(G/A)
CakSNP13719	*Kabuli*	*Ca_Kabuli_Chr08*	5487830	(G/A)
CakSNP13720	*Kabuli*	*Ca_Kabuli_Chr08*	5536418	(T/G)
CakSNP13721	*Kabuli*	*Ca_Kabuli_Chr08*	5538696	(A/C)
CakSNP13722	*Kabuli*	*Ca_Kabuli_Chr08*	5538695	(T/C)
CakSNP13723	*Kabuli*	*Ca_Kabuli_Chr08*	5538683	(T/C)
CakSNP13724	*Kabuli*	*Ca_Kabuli_Chr08*	5539044	(C/T)
CakSNP13725	*Kabuli*	*Ca_Kabuli_Chr08*	5586106	(G/A)
CakSNP13726	*Kabuli*	*Ca_Kabuli_Chr08*	5591654	(T/C)
CakSNP13727	*Kabuli*	*Ca_Kabuli_Chr08*	5591788	(G/C)
CakSNP13728	*Kabuli*	*Ca_Kabuli_Chr08*	5650080	(C/G)
CakSNP13729	*Kabuli*	*Ca_Kabuli_Chr08*	5712245	(T/A)
CakSNP13730	*Kabuli*	*Ca_Kabuli_Chr08*	5811179	(G/C)
CakSNP13731	*Kabuli*	*Ca_Kabuli_Chr08*	5819008	(G/A)
CakSNP13732	*Kabuli*	*Ca_Kabuli_Chr08*	5840141	(G/A)
CakSNP13733	*Kabuli*	*Ca_Kabuli_Chr08*	5870455	(A/G)
CakSNP13734	*Kabuli*	*Ca_Kabuli_Chr08*	5870664	(T/C)

SNP IDs	Cultivars	Chromosomes/scaffolds	Physical positions (bp)	SNPs
CakSNP13735	*Kabuli*	*Ca_Kabuli_Chr08*	5870749	(C/T)
CakSNP13736	*Kabuli*	*Ca_Kabuli_Chr08*	5871358	(G/C)
CakSNP13737	*Kabuli*	*Ca_Kabuli_Chr08*	5871361	(T/G)
CakSNP13738	*Kabuli*	*Ca_Kabuli_Chr08*	5871364	(T/C)
CakSNP13739	*Kabuli*	*Ca_Kabuli_Chr08*	5871367	(T/A)
CakSNP13740	*Kabuli*	*Ca_Kabuli_Chr08*	5871421	(A/G)
CakSNP13741	*Kabuli*	*Ca_Kabuli_Chr08*	5910629	(C/G)
CakSNP13742	*Kabuli*	*Ca_Kabuli_Chr08*	5927870	(G/T)
CakSNP13743	*Kabuli*	*Ca_Kabuli_Chr08*	5935435	(A/C)
CakSNP13744	*Kabuli*	*Ca_Kabuli_Chr08*	5963855	(T/A)
CakSNP13745	*Kabuli*	*Ca_Kabuli_Chr08*	5992761	(A/G)
CakSNP13746	*Kabuli*	*Ca_Kabuli_Chr08*	6084594	(G/A)
CakSNP13747	*Kabuli*	*Ca_Kabuli_Chr08*	6084591	(C/T)
CakSNP13748	*Kabuli*	*Ca_Kabuli_Chr08*	6115961	(T/G)
CakSNP13749	*Kabuli*	*Ca_Kabuli_Chr08*	6115965	(T/C)
CakSNP13750	*Kabuli*	*Ca_Kabuli_Chr08*	6116096	(C/T)
CakSNP13751	*Kabuli*	*Ca_Kabuli_Chr08*	6116097	(C/A)
CakSNP13752	*Kabuli*	*Ca_Kabuli_Chr08*	6116101	(G/A)
CakSNP13753	*Kabuli*	*Ca_Kabuli_Chr08*	6145326	(G/A)
CakSNP13754	*Kabuli*	*Ca_Kabuli_Chr08*	6149097	(A/C)
CakSNP13755	*Kabuli*	*Ca_Kabuli_Chr08*	6192944	(T/G)
CakSNP13756	*Kabuli*	*Ca_Kabuli_Chr08*	6192986	(C/T)
CakSNP13757	*Kabuli*	*Ca_Kabuli_Chr08*	6193078	(G/A)
CakSNP13758	*Kabuli*	*Ca_Kabuli_Chr08*	6218250	(G/A)
CakSNP13759	*Kabuli*	*Ca_Kabuli_Chr08*	6218275	(C/T)
CakSNP13760	*Kabuli*	*Ca_Kabuli_Chr08*	6218298	(A/G)
CakSNP13761	*Kabuli*	*Ca_Kabuli_Chr08*	6218299	(T/A)
CakSNP13762	*Kabuli*	*Ca_Kabuli_Chr08*	6266432	(A/G)
CakSNP13763	*Kabuli*	*Ca_Kabuli_Chr08*	6372236	(A/T)
CakSNP13764	*Kabuli*	*Ca_Kabuli_Chr08*	6372230	(G/A)
CakSNP13765	*Kabuli*	*Ca_Kabuli_Chr08*	6372223	(G/A)
CakSNP13766	*Kabuli*	*Ca_Kabuli_Chr08*	6374794	(C/A)
CakSNP13767	*Kabuli*	*Ca_Kabuli_Chr08*	6437758	(T/G)
CakSNP13768	*Kabuli*	*Ca_Kabuli_Chr08*	6503723	(T/C)
CakSNP13769	*Kabuli*	*Ca_Kabuli_Chr08*	6525633	(A/T)
CakSNP13770	*Kabuli*	*Ca_Kabuli_Chr08*	6526612	(T/C)
CakSNP13771	*Kabuli*	*Ca_Kabuli_Chr08*	6544248	(G/T)
CakSNP13772	*Kabuli*	*Ca_Kabuli_Chr08*	6544278	(C/A)
CakSNP13773	*Kabuli*	*Ca_Kabuli_Chr08*	6624006	(C/T)
CakSNP13774	*Kabuli*	*Ca_Kabuli_Chr08*	6630663	(C/T)
CakSNP13775	*Kabuli*	*Ca_Kabuli_Chr08*	6630661	(T/C)

SNP IDs	Cultivars	Chromosomes/scaffolds	Physical positions (bp)	SNPs
CakSNP13776	*Kabuli*	*Ca_Kabuli_Chr08*	6664509	(T/A)
CakSNP13777	*Kabuli*	*Ca_Kabuli_Chr08*	6673855	(C/T)
CakSNP13778	*Kabuli*	*Ca_Kabuli_Chr08*	6673891	(A/G)
CakSNP13779	*Kabuli*	*Ca_Kabuli_Chr08*	6674024	(G/A)
CakSNP13780	*Kabuli*	*Ca_Kabuli_Chr08*	6673956	(G/A)
CakSNP13781	*Kabuli*	*Ca_Kabuli_Chr08*	6708869	(G/T)
CakSNP13782	*Kabuli*	*Ca_Kabuli_Chr08*	6708945	(A/G)
CakSNP13783	*Kabuli*	*Ca_Kabuli_Chr08*	6747091	(T/C)
CakSNP13784	*Kabuli*	*Ca_Kabuli_Chr08*	6747995	(A/G)
CakSNP13785	*Kabuli*	*Ca_Kabuli_Chr08*	6748136	(C/T)
CakSNP13786	*Kabuli*	*Ca_Kabuli_Chr08*	6748294	(T/C)
CakSNP13787	*Kabuli*	*Ca_Kabuli_Chr08*	6780592	(T/G)
CakSNP13788	*Kabuli*	*Ca_Kabuli_Chr08*	6940139	(A/G)
CakSNP13789	*Kabuli*	*Ca_Kabuli_Chr08*	6942651	(G/T)
CakSNP13790	*Kabuli*	*Ca_Kabuli_Chr08*	6943710	(A/G)
CakSNP13791	*Kabuli*	*Ca_Kabuli_Chr08*	6968036	(A/G)
CakSNP13792	*Kabuli*	*Ca_Kabuli_Chr08*	6986090	(G/A)
CakSNP13793	*Kabuli*	*Ca_Kabuli_Chr08*	6986130	(A/G)
CakSNP13794	*Kabuli*	*Ca_Kabuli_Chr08*	6987609	(G/T)
CakSNP13795	*Kabuli*	*Ca_Kabuli_Chr08*	6987615	(A/C)
CakSNP13796	*Kabuli*	*Ca_Kabuli_Chr08*	6989342	(G/T)
CakSNP13797	*Kabuli*	*Ca_Kabuli_Chr08*	6989433	(A/G)
CakSNP13798	*Kabuli*	*Ca_Kabuli_Chr08*	7013049	(G/A)
CakSNP13799	*Kabuli*	*Ca_Kabuli_Chr08*	7037715	(G/A)
CakSNP13800	*Kabuli*	*Ca_Kabuli_Chr08*	7041901	(A/C)
CakSNP13801	*Kabuli*	*Ca_Kabuli_Chr08*	7047509	(T/G)
CakSNP13802	*Kabuli*	*Ca_Kabuli_Chr08*	7047469	(T/A)
CakSNP13803	*Kabuli*	*Ca_Kabuli_Chr08*	7099455	(C/T)
CakSNP13804	*Kabuli*	*Ca_Kabuli_Chr08*	7099569	(T/C)
CakSNP13805	*Kabuli*	*Ca_Kabuli_Chr08*	7099530	(C/A)
CakSNP13806	*Kabuli*	*Ca_Kabuli_Chr08*	7099756	(A/G)
CakSNP13807	*Kabuli*	*Ca_Kabuli_Chr08*	7162826	(T/G)
CakSNP13808	*Kabuli*	*Ca_Kabuli_Chr08*	7162976	(A/G)
CakSNP13809	*Kabuli*	*Ca_Kabuli_Chr08*	7174066	(C/T)
CakSNP13810	*Kabuli*	*Ca_Kabuli_Chr08*	7175086	(A/C)
CakSNP13811	*Kabuli*	*Ca_Kabuli_Chr08*	7191825	(A/G)
CakSNP13812	*Kabuli*	*Ca_Kabuli_Chr08*	7193946	(C/T)
CakSNP13813	*Kabuli*	*Ca_Kabuli_Chr08*	7193960	(A/T)
CakSNP13814	*Kabuli*	*Ca_Kabuli_Chr08*	7295151	(T/C)
CakSNP13815	*Kabuli*	*Ca_Kabuli_Chr08*	7295206	(A/G)
CakSNP13816	*Kabuli*	*Ca_Kabuli_Chr08*	7295177	(G/T)

SNP IDs	Cultivars	Chromosomes/scaffolds	Physical positions (bp)	SNPs
CakSNP13817	*Kabuli*	*Ca_Kabuli_Chr08*	7307737	(G/T)
CakSNP13818	*Kabuli*	*Ca_Kabuli_Chr08*	7307787	(A/T)
CakSNP13819	*Kabuli*	*Ca_Kabuli_Chr08*	7357621	(A/G)
CakSNP13820	*Kabuli*	*Ca_Kabuli_Chr08*	7357583	(T/C)
CakSNP13821	*Kabuli*	*Ca_Kabuli_Chr08*	7503013	(T/A)
CakSNP13822	*Kabuli*	*Ca_Kabuli_Chr08*	7503010	(T/C)
CakSNP13823	*Kabuli*	*Ca_Kabuli_Chr08*	7506004	(A/G)
CakSNP13824	*Kabuli*	*Ca_Kabuli_Chr08*	7519758	(C/A)
CakSNP13825	*Kabuli*	*Ca_Kabuli_Chr08*	7519815	(G/A)
CakSNP13826	*Kabuli*	*Ca_Kabuli_Chr08*	7526956	(A/G)
CakSNP13827	*Kabuli*	*Ca_Kabuli_Chr08*	7563385	(C/G)
CakSNP13828	*Kabuli*	*Ca_Kabuli_Chr08*	7563370	(T/C)
CakSNP13829	*Kabuli*	*Ca_Kabuli_Chr08*	7563373	(T/A)
CakSNP13830	*Kabuli*	*Ca_Kabuli_Chr08*	7566548	(T/C)
CakSNP13831	*Kabuli*	*Ca_Kabuli_Chr08*	7614202	(A/C)
CakSNP13832	*Kabuli*	*Ca_Kabuli_Chr08*	7628690	(T/A)
CakSNP13833	*Kabuli*	*Ca_Kabuli_Chr08*	7665291	(T/C)
CakSNP13834	*Kabuli*	*Ca_Kabuli_Chr08*	7665383	(G/T)
CakSNP13835	*Kabuli*	*Ca_Kabuli_Chr08*	7694747	(A/G)
CakSNP13836	*Kabuli*	*Ca_Kabuli_Chr08*	7695990	(A/T)
CakSNP13837	*Kabuli*	*Ca_Kabuli_Chr08*	7695989	(T/A)
CakSNP13838	*Kabuli*	*Ca_Kabuli_Chr08*	7697886	(G/C)
CakSNP13839	*Kabuli*	*Ca_Kabuli_Chr08*	7726921	(G/A)
CakSNP13840	*Kabuli*	*Ca_Kabuli_Chr08*	7726894	(G/T)
CakSNP13841	*Kabuli*	*Ca_Kabuli_Chr08*	7726879	(A/G)
CakSNP13842	*Kabuli*	*Ca_Kabuli_Chr08*	7728085	(T/G)
CakSNP13843	*Kabuli*	*Ca_Kabuli_Chr08*	7728076	(T/C)
CakSNP13844	*Kabuli*	*Ca_Kabuli_Chr08*	7729693	(T/C)
CakSNP13845	*Kabuli*	*Ca_Kabuli_Chr08*	7733508	(C/T)
CakSNP13846	*Kabuli*	*Ca_Kabuli_Chr08*	7733573	(A/C)
CakSNP13847	*Kabuli*	*Ca_Kabuli_Chr08*	7733574	(G/A)
CakSNP13848	*Kabuli*	*Ca_Kabuli_Chr08*	7733670	(C/T)
CakSNP13849	*Kabuli*	*Ca_Kabuli_Chr08*	7733647	(G/T)
CakSNP13850	*Kabuli*	*Ca_Kabuli_Chr08*	7776466	(G/A)
CakSNP13851	*Kabuli*	*Ca_Kabuli_Chr08*	7776592	(T/C)
CakSNP13852	*Kabuli*	*Ca_Kabuli_Chr08*	7776897	(A/T)
CakSNP13853	*Kabuli*	*Ca_Kabuli_Chr08*	7776934	(G/T)
CakSNP13854	*Kabuli*	*Ca_Kabuli_Chr08*	7776970	(T/G)
CakSNP13855	*Kabuli*	*Ca_Kabuli_Chr08*	7777023	(G/A)
CakSNP13856	*Kabuli*	*Ca_Kabuli_Chr08*	7807065	(T/C)
CakSNP13857	*Kabuli*	*Ca_Kabuli_Chr08*	7978269	(C/T)

SNP IDs	Cultivars	Chromosomes/scaffolds	Physical positions (bp)	SNPs
CakSNP13858	*Kabuli*	*Ca_Kabuli_Chr08*	8029283	(T/A)
CakSNP13859	*Kabuli*	*Ca_Kabuli_Chr08*	8039078	(A/T)
CakSNP13860	*Kabuli*	*Ca_Kabuli_Chr08*	8059732	(A/G)
CakSNP13861	*Kabuli*	*Ca_Kabuli_Chr08*	8066006	(C/A)
CakSNP13862	*Kabuli*	*Ca_Kabuli_Chr08*	8066005	(A/G)
CakSNP13863	*Kabuli*	*Ca_Kabuli_Chr08*	8104804	(G/T)
CakSNP13864	*Kabuli*	*Ca_Kabuli_Chr08*	8104801	(T/C)
CakSNP13865	*Kabuli*	*Ca_Kabuli_Chr08*	8104884	(T/A)
CakSNP13866	*Kabuli*	*Ca_Kabuli_Chr08*	8104892	(C/T)
CakSNP13867	*Kabuli*	*Ca_Kabuli_Chr08*	8106804	(A/G)
CakSNP13868	*Kabuli*	*Ca_Kabuli_Chr08*	8106851	(G/A)
CakSNP13869	*Kabuli*	*Ca_Kabuli_Chr08*	8114316	(A/C)
CakSNP13870	*Kabuli*	*Ca_Kabuli_Chr08*	8194206	(C/T)
CakSNP13871	*Kabuli*	*Ca_Kabuli_Chr08*	8194232	(C/A)
CakSNP13872	*Kabuli*	*Ca_Kabuli_Chr08*	8194234	(C/A)
CakSNP13873	*Kabuli*	*Ca_Kabuli_Chr08*	8194342	(T/C)
CakSNP13874	*Kabuli*	*Ca_Kabuli_Chr08*	8194464	(A/G)
CakSNP13875	*Kabuli*	*Ca_Kabuli_Chr08*	8194436	(T/A)
CakSNP13876	*Kabuli*	*Ca_Kabuli_Chr08*	8202726	(G/A)
CakSNP13877	*Kabuli*	*Ca_Kabuli_Chr08*	8270488	(T/C)
CakSNP13878	*Kabuli*	*Ca_Kabuli_Chr08*	8288041	(A/G)
CakSNP13879	*Kabuli*	*Ca_Kabuli_Chr08*	8293716	(G/A)
CakSNP13880	*Kabuli*	*Ca_Kabuli_Chr08*	8293704	(T/C)
CakSNP13881	*Kabuli*	*Ca_Kabuli_Chr08*	8293693	(T/C)
CakSNP13882	*Kabuli*	*Ca_Kabuli_Chr08*	8318110	(A/G)
CakSNP13883	*Kabuli*	*Ca_Kabuli_Chr08*	8389117	(C/T)
CakSNP13884	*Kabuli*	*Ca_Kabuli_Chr08*	8458383	(G/A)
CakSNP13885	*Kabuli*	*Ca_Kabuli_Chr08*	8533479	(G/T)
CakSNP13886	*Kabuli*	*Ca_Kabuli_Chr08*	8533978	(G/A)
CakSNP13887	*Kabuli*	*Ca_Kabuli_Chr08*	8560330	(C/A)
CakSNP13888	*Kabuli*	*Ca_Kabuli_Chr08*	8560331	(C/G)
CakSNP13889	*Kabuli*	*Ca_Kabuli_Chr08*	8560332	(A/C)
CakSNP13890	*Kabuli*	*Ca_Kabuli_Chr08*	8560326	(G/T)
CakSNP13891	*Kabuli*	*Ca_Kabuli_Chr08*	8661327	(T/C)
CakSNP13892	*Kabuli*	*Ca_Kabuli_Chr08*	8670242	(T/G)
CakSNP13893	*Kabuli*	*Ca_Kabuli_Chr08*	8772131	(C/G)
CakSNP13894	*Kabuli*	*Ca_Kabuli_Chr08*	8772124	(G/A)
CakSNP13895	*Kabuli*	*Ca_Kabuli_Chr08*	8853602	(G/A)
CakSNP13896	*Kabuli*	*Ca_Kabuli_Chr08*	8853664	(A/G)
CakSNP13897	*Kabuli*	*Ca_Kabuli_Chr08*	8856046	(C/T)
CakSNP13898	*Kabuli*	*Ca_Kabuli_Chr08*	8856047	(C/T)

SNP IDs	Cultivars	Chromosomes/scaffolds	Physical positions (bp)	SNPs
CakSNP13899	*Kabuli*	*Ca_Kabuli_Chr08*	8856150	(A/G)
CakSNP13900	*Kabuli*	*Ca_Kabuli_Chr08*	8856143	(G/A)
CakSNP13901	*Kabuli*	*Ca_Kabuli_Chr08*	8864079	(C/T)
CakSNP13902	*Kabuli*	*Ca_Kabuli_Chr08*	8864157	(G/A)
CakSNP13903	*Kabuli*	*Ca_Kabuli_Chr08*	8888412	(A/G)
CakSNP13904	*Kabuli*	*Ca_Kabuli_Chr08*	8888414	(G/T)
CakSNP13905	*Kabuli*	*Ca_Kabuli_Chr08*	8888426	(T/C)
CakSNP13906	*Kabuli*	*Ca_Kabuli_Chr08*	8888476	(A/C)
CakSNP13907	*Kabuli*	*Ca_Kabuli_Chr08*	8904346	(C/A)
CakSNP13908	*Kabuli*	*Ca_Kabuli_Chr08*	8904366	(A/G)
CakSNP13909	*Kabuli*	*Ca_Kabuli_Chr08*	8914018	(A/C)
CakSNP13910	*Kabuli*	*Ca_Kabuli_Chr08*	9097585	(A/C)
CakSNP13911	*Kabuli*	*Ca_Kabuli_Chr08*	9099798	(G/T)
CakSNP13912	*Kabuli*	*Ca_Kabuli_Chr08*	9100111	(C/T)
CakSNP13913	*Kabuli*	*Ca_Kabuli_Chr08*	9109896	(T/A)
CakSNP13914	*Kabuli*	*Ca_Kabuli_Chr08*	9109907	(A/T)
CakSNP13915	*Kabuli*	*Ca_Kabuli_Chr08*	9109920	(C/A)
CakSNP13916	*Kabuli*	*Ca_Kabuli_Chr08*	9112178	(G/A)
CakSNP13917	*Kabuli*	*Ca_Kabuli_Chr08*	9112172	(A/C)
CakSNP13918	*Kabuli*	*Ca_Kabuli_Chr08*	9118582	(C/T)
CakSNP13919	*Kabuli*	*Ca_Kabuli_Chr08*	9118667	(A/G)
CakSNP13920	*Kabuli*	*Ca_Kabuli_Chr08*	9143840	(C/A)
CakSNP13921	*Kabuli*	*Ca_Kabuli_Chr08*	9151722	(G/T)
CakSNP13922	*Kabuli*	*Ca_Kabuli_Chr08*	9389347	(G/A)
CakSNP13923	*Kabuli*	*Ca_Kabuli_Chr08*	10004481	(T/C)
CakSNP13924	*Kabuli*	*Ca_Kabuli_Chr08*	10155981	(A/C)
CakSNP13925	*Kabuli*	*Ca_Kabuli_Chr08*	10258892	(T/G)
CakSNP13926	*Kabuli*	*Ca_Kabuli_Chr08*	10486601	(A/C)
CakSNP13927	*Kabuli*	*Ca_Kabuli_Chr08*	10556124	(T/C)
CakSNP13928	*Kabuli*	*Ca_Kabuli_Chr08*	10668422	(A/C)
CakSNP13929	*Kabuli*	*Ca_Kabuli_Chr08*	10728369	(G/C)
CakSNP13930	*Kabuli*	*Ca_Kabuli_Chr08*	10733404	(A/G)
CakSNP13931	*Kabuli*	*Ca_Kabuli_Chr08*	10733503	(C/T)
CakSNP13932	*Kabuli*	*Ca_Kabuli_Chr08*	10733661	(G/A)
CakSNP13933	*Kabuli*	*Ca_Kabuli_Chr08*	10733747	(C/T)
CakSNP13934	*Kabuli*	*Ca_Kabuli_Chr08*	10759078	(G/A)
CakSNP13935	*Kabuli*	*Ca_Kabuli_Chr08*	10759060	(G/A)
CakSNP13936	*Kabuli*	*Ca_Kabuli_Chr08*	10759056	(C/T)
CakSNP13937	*Kabuli*	*Ca_Kabuli_Chr08*	10759102	(C/T)
CakSNP13938	*Kabuli*	*Ca_Kabuli_Chr08*	10763656	(A/T)
CakSNP13939	*Kabuli*	*Ca_Kabuli_Chr08*	10953767	(C/T)

**SNP IDs**	**Cultivars**	**Chromosomes/scaffolds**	**Physical positions (bp)**	**SNPs**
CakSNP13940	*Kabuli*	*Ca_Kabuli_Chr08*	11015680	(G/T)
CakSNP13941	*Kabuli*	*Ca_Kabuli_Chr08*	11015678	(C/G)
CakSNP13942	*Kabuli*	*Ca_Kabuli_Chr08*	11015666	(G/C)
CakSNP13943	*Kabuli*	*Ca_Kabuli_Chr08*	11066644	(C/T)
CakSNP13944	*Kabuli*	*Ca_Kabuli_Chr08*	11066728	(G/A)
CakSNP13945	*Kabuli*	*Ca_Kabuli_Chr08*	11087143	(A/G)
CakSNP13946	*Kabuli*	*Ca_Kabuli_Chr08*	11087169	(C/T)
CakSNP13947	*Kabuli*	*Ca_Kabuli_Chr08*	11087302	(A/T)
CakSNP13948	*Kabuli*	*Ca_Kabuli_Chr08*	11087250	(T/C)
CakSNP13949	*Kabuli*	*Ca_Kabuli_Chr08*	11087247	(T/C)
CakSNP13950	*Kabuli*	*Ca_Kabuli_Chr08*	11186919	(C/G)
CakSNP13951	*Kabuli*	*Ca_Kabuli_Chr08*	11187037	(T/C)
CakSNP13952	*Kabuli*	*Ca_Kabuli_Chr08*	11186986	(A/C)
CakSNP13953	*Kabuli*	*Ca_Kabuli_Chr08*	11186974	(A/C)
CakSNP13954	*Kabuli*	*Ca_Kabuli_Chr08*	11231812	(A/G)
CakSNP13955	*Kabuli*	*Ca_Kabuli_Chr08*	11240258	(C/A)
CakSNP13956	*Kabuli*	*Ca_Kabuli_Chr08*	11243263	(A/C)
CakSNP13957	*Kabuli*	*Ca_Kabuli_Chr08*	11243256	(A/C)
CakSNP13958	*Kabuli*	*Ca_Kabuli_Chr08*	11266447	(G/A)
CakSNP13959	*Kabuli*	*Ca_Kabuli_Chr08*	11310460	(G/A)
CakSNP13960	*Kabuli*	*Ca_Kabuli_Chr08*	11310480	(T/A)
CakSNP13961	*Kabuli*	*Ca_Kabuli_Chr08*	11332934	(C/T)
CakSNP13962	*Kabuli*	*Ca_Kabuli_Chr08*	11332940	(T/C)
CakSNP13963	*Kabuli*	*Ca_Kabuli_Chr08*	11333057	(C/G)
CakSNP13964	*Kabuli*	*Ca_Kabuli_Chr08*	11334190	(A/T)
CakSNP13965	*Kabuli*	*Ca_Kabuli_Chr08*	11334210	(C/T)
CakSNP13966	*Kabuli*	*Ca_Kabuli_Chr08*	11349891	(T/A)
CakSNP13967	*Kabuli*	*Ca_Kabuli_Chr08*	11349995	(A/G)
CakSNP13968	*Kabuli*	*Ca_Kabuli_Chr08*	11403805	(G/A)
CakSNP13969	*Kabuli*	*Ca_Kabuli_Chr08*	11403754	(G/A)
CakSNP13970	*Kabuli*	*Ca_Kabuli_Chr08*	11562814	(A/C)
CakSNP13971	*Kabuli*	*Ca_Kabuli_Chr08*	11562833	(A/G)
CakSNP13972	*Kabuli*	*Ca_Kabuli_Chr08*	11631836	(C/T)
CakSNP13973	*Kabuli*	*Ca_Kabuli_Chr08*	11656700	(G/T)
CakSNP13974	*Kabuli*	*Ca_Kabuli_Chr08*	11656687	(A/G)
CakSNP13975	*Kabuli*	*Ca_Kabuli_Chr08*	11656675	(T/C)
CakSNP13976	*Kabuli*	*Ca_Kabuli_Chr08*	11656670	(A/G)
CakSNP13977	*Kabuli*	*Ca_Kabuli_Chr08*	11696021	(G/C)
CakSNP13978	*Kabuli*	*Ca_Kabuli_Chr08*	11735882	(A/C)
CakSNP13979	*Kabuli*	*Ca_Kabuli_Chr08*	11962280	(T/C)
CakSNP13980	*Kabuli*	*Ca_Kabuli_Chr08*	12875217	(A/G)

**SNP IDs**	**Cultivars**	**Chromosomes/scaffolds**	**Physical positions (bp)**	**SNPs**
CakSNP13981	*Kabuli*	*Ca_Kabuli_Chr08*	13248268	(T/C)
CakSNP13982	*Kabuli*	*Ca_Kabuli_Chr08*	13745335	(A/G)
CakSNP13983	*Kabuli*	*Ca_Kabuli_Chr08*	13840865	(A/C)
CakSNP13984	*Kabuli*	*Ca_Kabuli_Chr08*	13860922	(C/G)
CakSNP13985	*Kabuli*	*Ca_Kabuli_Chr08*	13864026	(T/G)
CakSNP13986	*Kabuli*	*Ca_Kabuli_Chr08*	13864077	(T/C)
CakSNP13987	*Kabuli*	*Ca_Kabuli_Chr08*	13997385	(C/T)
CakSNP13988	*Kabuli*	*Ca_Kabuli_Chr08*	13998102	(A/G)
CakSNP13989	*Kabuli*	*Ca_Kabuli_Chr08*	14006709	(G/A)
CakSNP13990	*Kabuli*	*Ca_Kabuli_Chr08*	14006694	(C/T)
CakSNP13991	*Kabuli*	*Ca_Kabuli_Chr08*	14007431	(A/G)
CakSNP13992	*Kabuli*	*Ca_Kabuli_Chr08*	14124588	(C/A)
CakSNP13993	*Kabuli*	*Ca_Kabuli_Chr08*	14129040	(G/A)
CakSNP13994	*Kabuli*	*Ca_Kabuli_Chr08*	14325980	(C/A)
CakSNP13995	*Kabuli*	*Ca_Kabuli_Chr08*	14437006	(C/A)
CakSNP13996	*Kabuli*	*Ca_Kabuli_Chr08*	14444424	(G/A)
CakSNP13997	*Kabuli*	*Ca_Kabuli_Chr08*	14444438	(A/C)
CakSNP13998	*Kabuli*	*Ca_Kabuli_Chr08*	14445205	(C/A)
CakSNP13999	*Kabuli*	*Ca_Kabuli_Chr08*	14460426	(T/C)
CakSNP14000	*Kabuli*	*Ca_Kabuli_Chr08*	14528882	(T/G)
CakSNP14001	*Kabuli*	*Ca_Kabuli_Chr08*	14528966	(C/G)
CakSNP14002	*Kabuli*	*Ca_Kabuli_Chr08*	14613302	(G/A)
CakSNP14003	*Kabuli*	*Ca_Kabuli_Chr08*	14640967	(A/C)
CakSNP14004	*Kabuli*	*Ca_Kabuli_Chr08*	14715495	(T/G)
CakSNP14005	*Kabuli*	*Ca_Kabuli_Chr08*	14753763	(T/C)
CakSNP14006	*Kabuli*	*Ca_Kabuli_Chr08*	14753750	(T/C)
CakSNP14007	*Kabuli*	*Ca_Kabuli_Chr08*	14754849	(A/T)
CakSNP14008	*Kabuli*	*Ca_Kabuli_Chr08*	14759784	(G/T)
CakSNP14009	*Kabuli*	*Ca_Kabuli_Chr08*	14760479	(C/G)
CakSNP14010	*Kabuli*	*Ca_Kabuli_Chr08*	14760497	(G/A)
CakSNP14011	*Kabuli*	*Ca_Kabuli_Chr08*	14760503	(A/T)
CakSNP14012	*Kabuli*	*Ca_Kabuli_Chr08*	14821416	(G/A)
CakSNP14013	*Kabuli*	*Ca_Kabuli_Chr08*	15000109	(A/G)
CakSNP14014	*Kabuli*	*Ca_Kabuli_Chr08*	15034465	(T/C)
CakSNP14015	*Kabuli*	*Ca_Kabuli_Chr08*	15131175	(G/A)
CakSNP14016	*Kabuli*	*Ca_Kabuli_Chr08*	15192125	(A/G)
CakSNP14017	*Kabuli*	*Ca_Kabuli_Chr08*	15207944	(C/T)
CakSNP14018	*Kabuli*	*Ca_Kabuli_Chr08*	15207976	(A/G)
CakSNP14019	*Kabuli*	*Ca_Kabuli_Chr08*	15220986	(C/G)
CakSNP14020	*Kabuli*	*Ca_Kabuli_Chr08*	15421764	(T/C)
CakSNP14021	*Kabuli*	*Ca_Kabuli_Chr08*	15421744	(T/G)

SNP IDs	Cultivars	Chromosomes/scaffolds	Physical positions (bp)	SNPs
CakSNP14022	*Kabuli*	*Ca_Kabuli_Chr08*	15421719	(C/G)
CakSNP14023	*Kabuli*	*Ca_Kabuli_Chr08*	15422163	(A/T)
CakSNP14024	*Kabuli*	*Ca_Kabuli_Chr08*	15546669	(T/C)
CakSNP14025	*Kabuli*	*Ca_Kabuli_Chr08*	15546671	(A/C)
CakSNP14026	*Kabuli*	*Ca_Kabuli_Chr08*	15546696	(C/T)
CakSNP14027	*Kabuli*	*Ca_Kabuli_Chr08*	15546710	(T/A)
CakSNP14028	*Kabuli*	*Ca_Kabuli_Chr08*	15546758	(G/C)
CakSNP14029	*Kabuli*	*Ca_Kabuli_Chr08*	15553251	(A/G)
CakSNP14030	*Kabuli*	*Ca_Kabuli_Chr08*	15555771	(A/C)
CakSNP14031	*Kabuli*	*Ca_Kabuli_Chr08*	15699220	(T/A)
CakSNP14032	*Kabuli*	*Ca_Kabuli_Chr08*	15699180	(T/C)
CakSNP14033	*Kabuli*	*Ca_Kabuli_Chr08*	15741863	(C/G)
CakSNP14034	*Kabuli*	*Ca_Kabuli_Chr08*	15764739	(C/T)
CakSNP14035	*Kabuli*	*Ca_Kabuli_Chr08*	15764748	(G/A)
CakSNP14036	*Kabuli*	*Ca_Kabuli_Chr08*	15764823	(G/A)
CakSNP14037	*Kabuli*	*Ca_Kabuli_Chr08*	15764808	(C/A)
CakSNP14038	*Kabuli*	*Ca_Kabuli_Chr08*	15764874	(G/A)
CakSNP14039	*Kabuli*	*Ca_Kabuli_Chr08*	15857176	(G/A)
CakSNP14040	*Kabuli*	*Ca_Kabuli_Chr08*	15857192	(A/G)
CakSNP14041	*Kabuli*	*Ca_Kabuli_Chr08*	15857359	(T/G)
CakSNP14042	*Kabuli*	*Ca_Kabuli_Chr08*	15863509	(T/C)
CakSNP14043	*Kabuli*	*Ca_Kabuli_Chr08*	15873071	(A/C)
CakSNP14044	*Kabuli*	*Ca_Kabuli_Chr08*	15873133	(A/G)
CakSNP14045	*Kabuli*	*Ca_Kabuli_Chr08*	16004976	(G/C)
CakSNP14046	*Kabuli*	*Ca_Kabuli_Chr08*	16005046	(C/T)
CakSNP14047	*Kabuli*	*Ca_Kabuli_Chr08*	16005078	(A/G)
CakSNP14048	*Kabuli*	*Ca_Kabuli_Chr08*	16005235	(A/G)
CakSNP14049	*Kabuli*	*Ca_Kabuli_Chr08*	16005253	(G/A)
CakSNP14050	*Kabuli*	*Ca_Kabuli_Chr08*	16020239	(C/A)
CakSNP14051	*Kabuli*	*Ca_Kabuli_Chr08*	16020277	(T/G)
CakSNP14052	*Kabuli*	*Ca_Kabuli_Chr08*	16020323	(G/T)
CakSNP14053	*Kabuli*	*Ca_Kabuli_Chr08*	16020305	(G/A)
CakSNP14054	*Kabuli*	*Ca_Kabuli_Chr08*	16022432	(C/T)
CakSNP14055	*Kabuli*	*Ca_Kabuli_Chr08*	16027974	(A/T)
CakSNP14056	*Kabuli*	*Ca_Kabuli_Chr08*	16028064	(G/T)
CakSNP14057	*Kabuli*	*Ca_Kabuli_Chr08*	16028062	(G/A)
CakSNP14058	*Kabuli*	*Ca_Kabuli_Chr08*	16028040	(A/G)
CakSNP14059	*Kabuli*	*Ca_Kabuli_Chr08*	16033779	(T/G)
CakSNP14060	*Kabuli*	*Ca_Kabuli_Chr08*	16076161	(C/G)
CakSNP14061	*Kabuli*	*Ca_Kabuli_Chr08*	16076168	(A/G)
CakSNP14062	*Kabuli*	*Ca_Kabuli_Chr08*	16102413	(C/T)

SNP IDs	Cultivars	Chromosomes/scaffolds	Physical positions (bp)	SNPs
CakSNP14063	*Kabuli*	*Ca_Kabuli_Chr08*	16102395	(G/A)
CakSNP14064	*Kabuli*	*Ca_Kabuli_Chr08*	16102363	(T/C)
CakSNP14065	*Kabuli*	*Ca_Kabuli_Chr08*	16102362	(G/T)
CakSNP14066	*Kabuli*	*Ca_Kabuli_Chr08*	16102348	(A/T)
CakSNP14067	*Kabuli*	*Ca_Kabuli_Chr08*	16102343	(C/T)
CakSNP14068	*Kabuli*	*Ca_Kabuli_Chr08*	16102335	(A/T)
CakSNP14069	*Kabuli*	*Ca_Kabuli_Chr08*	16102414	(G/T)
CakSNP14070	*Kabuli*	*Ca_Kabuli_Chr08*	16144279	(T/A)
CakSNP14071	*Kabuli*	*Ca_Kabuli_Chr08*	16145275	(A/G)
CakSNP14072	*Kabuli*	*Ca_Kabuli_Chr08*	16145419	(A/G)
CakSNP14073	*Kabuli*	*Ca_Kabuli_Chr08*	16145411	(C/G)
CakSNP14074	*Kabuli*	*Ca_Kabuli_Chr08*	16145370	(C/A)
CakSNP14075	*Kabuli*	*Ca_Kabuli_Chr08*	16157015	(C/T)
CakSNP14076	*Kabuli*	*Ca_Kabuli_Chr08*	16157134	(A/C)
CakSNP14077	*Kabuli*	*Ca_Kabuli_Chr08*	16158594	(A/C)
CakSNP14078	*Kabuli*	*Ca_Kabuli_Chr08*	16264310	(G/T)
CakSNP14079	*Kabuli*	*Ca_Kabuli_Chr08*	16268878	(T/C)
CakSNP14080	*Kabuli*	*Ca_Kabuli_Chr08*	16278758	(T/C)
CakSNP14081	*Kabuli*	*Ca_Kabuli_Chr08*	16283660	(C/T)
CakSNP14082	*Kabuli*	*Ca_Kabuli_Chr08*	16283661	(A/G)
CakSNP14083	*Kabuli*	*Ca_Kabuli_Chr08*	16283722	(A/G)
CakSNP14084	*Kabuli*	*Ca_Kabuli_Chr08*	16283738	(G/C)
CakSNP14085	*Kabuli*	*Ca_Kabuli_Chr08*	16283859	(T/C)
CakSNP14086	*Kabuli*	*Ca_Kabuli_Chr08*	16284269	(G/A)
CakSNP14087	*Kabuli*	*Ca_Kabuli_Chr08*	16284279	(A/C)
CakSNP14088	*Kabuli*	*Ca_Kabuli_Chr08*	16284306	(C/T)
CakSNP14089	*Kabuli*	*Ca_Kabuli_Chr08*	16309857	(G/T)
CakSNP14090	*Kabuli*	*Ca_Kabuli_Chr08*	16321524	(A/G)
CakSNP14091	*Kabuli*	*Ca_Kabuli_Chr08*	16321545	(G/T)
CakSNP14092	*Kabuli*	*Ca_Kabuli_Chr08*	16366490	(G/A)
CakSNP14093	*Kabuli*	*Ca_Kabuli_Chr08*	16381928	(T/G)
CakSNP14094	*Kabuli*	*Ca_Kabuli_Chr08*	16411550	(A/G)
CakSNP14095	*Kabuli*	*Ca_Kabuli_Chr08*	16412672	(T/C)
CakSNP14096	*Kabuli*	*Ca_Kabuli_Chr08*	16421407	(G/A)
CakSNP14097	*Kabuli*	*Ca_Kabuli_Chr08*	16421441	(C/A)
CakSNP14098	*Kabuli*	*Ca_Kabuli_Chr08*	16421442	(C/G)
CakSNP14099	*Kabuli*	*Ca_Kabuli_Chr08*	16421545	(G/T)
CakSNP14100	*Kabuli*	*Ca_Kabuli_Chr08*	16421497	(C/T)
CakSNP14101	*Kabuli*	*Ca_Kabuli_Chr08*	16421595	(A/C)
CakSNP14102	*Kabuli*	*Ca_Kabuli_Chr08*	16432907	(T/G)
CakSNP14103	*Kabuli*	*Ca_Kabuli_Chr08*	16472919	(T/A)

SNP IDs	Cultivars	Chromosomes/scaffolds	Physical positions (bp)	SNPs
CakSNP14104	*Kabuli*	*Ca_Kabuli_Chr08*	16472932	(C/T)
CakSNP14105	*Kabuli*	*Ca_Kabuli_Chr08*	16472933	(G/C)
CakSNP14106	*Kabuli*	*Ca_Kabuli_Chr08*	16472999	(A/C)
CakSNP14107	*Kabuli*	*Ca_Kabuli_Chr08*	16473047	(C/G)
CakSNP14108	*Kabuli*	*Ca_Kabuli_Chr08*	16473139	(C/T)
CakSNP14109	*Kabuli*	*Ca_Kabuli_Chr08*	16473154	(T/A)
CakSNP14110	*Kabuli*	*Ca_Kabuli_Chr08*	16473157	(C/T)
CakSNP14111	*Kabuli*	*Ca_Kabuli_Chr08*	16473311	(A/G)
CakSNP14112	*Kabuli*	*Ca_Kabuli_Chr08*	16473290	(A/G)
CakSNP14113	*Kabuli*	*Ca_Kabuli_Chr08*	16473920	(A/G)
CakSNP14114	*Kabuli*	*Ca_Kabuli_Chr08*	16473939	(A/G)
CakSNP14115	*Kabuli*	*Ca_Kabuli_Chr08*	16473987	(C/T)
CakSNP14116	*Kabuli*	*Ca_Kabuli_C11058086*	341	(G/A)
CakSNP14117	*Kabuli*	*Ca_Kabuli_C11058274*	143	(T/A)
CakSNP14118	*Kabuli*	*Ca_Kabuli_C11062332*	412	(G/A)
CakSNP14119	*Kabuli*	*Ca_Kabuli_C11062436*	381	(T/A)
CakSNP14120	*Kabuli*	*Ca_Kabuli_C11062546*	677	(G/T)
CakSNP14121	*Kabuli*	*Ca_Kabuli_C11076702*	989	(G/A)
CakSNP14122	*Kabuli*	*Ca_Kabuli_C11078452*	885	(G/A)
CakSNP14123	*Kabuli*	*Ca_Kabuli_C11079766*	1418	(A/C)
CakSNP14124	*Kabuli*	*Ca_Kabuli_C11079766*	1422	(G/C)
CakSNP14125	*Kabuli*	*Ca_Kabuli_C11079766*	1412	(A/T)
CakSNP14126	*Kabuli*	*Ca_Kabuli_C11080716*	1568	(A/C)
CakSNP14127	*Kabuli*	*Ca_Kabuli_C11084624*	656	(G/A)
CakSNP14128	*Kabuli*	*Ca_Kabuli_C11084992*	516	(A/G)
CakSNP14129	*Kabuli*	*Ca_Kabuli_C11084992*	537	(G/T)
CakSNP14130	*Kabuli*	*Ca_Kabuli_C11086066*	641	(A/C)
CakSNP14131	*Kabuli*	*Ca_Kabuli_C11086066*	624	(A/C)
CakSNP14132	*Kabuli*	*Ca_Kabuli_C11088694*	514	(G/C)
CakSNP14133	*Kabuli*	*Ca_Kabuli_C11119498*	682	(T/A)
CakSNP14134	*Kabuli*	*Ca_Kabuli_C11119498*	686	(C/A)
CakSNP14135	*Kabuli*	*Ca_Kabuli_C11119498*	689	(C/A)
CakSNP14136	*Kabuli*	*Ca_Kabuli_C11119498*	693	(G/A)
CakSNP14137	*Kabuli*	*Ca_Kabuli_C11132768*	2177	(G/A)
CakSNP14138	*Kabuli*	*Ca_Kabuli_C11142214*	2902	(G/T)
CakSNP14139	*Kabuli*	*Ca_Kabuli_C11142252*	3299	(A/C)
CakSNP14140	*Kabuli*	*Ca_Kabuli_C11143608*	1389	(A/T)
CakSNP14141	*Kabuli*	*Ca_Kabuli_C11147634*	3004	(C/A)
CakSNP14142	*Kabuli*	*Ca_Kabuli_C11147634*	3028	(C/T)
CakSNP14143	*Kabuli*	*Ca_Kabuli_C11149092*	2181	(A/G)
CakSNP14144	*Kabuli*	*Ca_Kabuli_C11149092*	2281	(T/C)

SNP IDs	Cultivars	Chromosomes/scaffolds	Physical positions (bp)	SNPs
CakSNP14145	*Kabuli*	*Ca_Kabuli_C11149092*	2474	(T/A)
CakSNP14146	*Kabuli*	*Ca_Kabuli_C11149092*	3190	(T/G)
CakSNP14147	*Kabuli*	*Ca_Kabuli_C11149092*	3284	(C/G)
CakSNP14148	*Kabuli*	*Ca_Kabuli_C11153606*	4275	(C/T)
CakSNP14149	*Kabuli*	*Ca_Kabuli_C11157320*	766	(C/G)
CakSNP14150	*Kabuli*	*Ca_Kabuli_C11157320*	2169	(T/A)
CakSNP14151	*Kabuli*	*Ca_Kabuli_C11157320*	2152	(G/C)
CakSNP14152	*Kabuli*	*Ca_Kabuli_C11158050*	2186	(A/T)
CakSNP14153	*Kabuli*	*Ca_Kabuli_C11160518*	4522	(A/G)
CakSNP14154	*Kabuli*	*Ca_Kabuli_C11162020*	3914	(C/T)
CakSNP14155	*Kabuli*	*Ca_Kabuli_C11162020*	3926	(T/G)
CakSNP14156	*Kabuli*	*Ca_Kabuli_C11162020*	5515	(G/A)
CakSNP14157	*Kabuli*	*Ca_Kabuli_C11164954*	5778	(T/A)
CakSNP14158	*Kabuli*	*Ca_Kabuli_C11164954*	5784	(G/A)
CakSNP14159	*Kabuli*	*Ca_Kabuli_C11165474*	6285	(T/A)
CakSNP14160	*Kabuli*	*Ca_Kabuli_C11165890*	154	(C/G)
CakSNP14161	*Kabuli*	*Ca_Kabuli_C11174756*	8931	(C/G)
CakSNP14162	*Kabuli*	*Ca_Kabuli_C11175430*	987	(A/G)
CakSNP14163	*Kabuli*	*Ca_Kabuli_C11175430*	3929	(T/G)
CakSNP14164	*Kabuli*	*Ca_Kabuli_C11175430*	3936	(G/T)
CakSNP14165	*Kabuli*	*Ca_Kabuli_C11175430*	3952	(T/C)
CakSNP14166	*Kabuli*	*Ca_Kabuli_C11176688*	4197	(G/C)
CakSNP14167	*Kabuli*	*Ca_Kabuli_C11177172*	2529	(T/G)
CakSNP14168	*Kabuli*	*Ca_Kabuli_C11177172*	2515	(T/A)
CakSNP14169	*Kabuli*	*Ca_Kabuli_C11177172*	5324	(G/A)
CakSNP14170	*Kabuli*	*Ca_Kabuli_C11177748*	1264	(T/A)
CakSNP14171	*Kabuli*	*Ca_Kabuli_C11178532*	2107	(A/T)
CakSNP14172	*Kabuli*	*Ca_Kabuli_C11178532*	3524	(A/G)
CakSNP14173	*Kabuli*	*Ca_Kabuli_C11181782*	6215	(A/G)
CakSNP14174	*Kabuli*	*Ca_Kabuli_C11181840*	19192	(A/G)
CakSNP14175	*Kabuli*	*Ca_Kabuli_C11181840*	19202	(T/C)
CakSNP14176	*Kabuli*	*Ca_Kabuli_Scaffold1006*	40651	(T/A)
CakSNP14177	*Kabuli*	*Ca_Kabuli_Scaffold1006*	382579	(C/T)
CakSNP14178	*Kabuli*	*Ca_Kabuli_Scaffold1006*	436923	(T/C)
CakSNP14179	*Kabuli*	*Ca_Kabuli_Scaffold1006*	644893	(A/C)
CakSNP14180	*Kabuli*	*Ca_Kabuli_Scaffold1006*	667620	(A/G)
CakSNP14181	*Kabuli*	*Ca_Kabuli_Scaffold1006*	667713	(G/A)
CakSNP14182	*Kabuli*	*Ca_Kabuli_Scaffold1006*	674495	(A/G)
CakSNP14183	*Kabuli*	*Ca_Kabuli_Scaffold1006*	674823	(G/A)
CakSNP14184	*Kabuli*	*Ca_Kabuli_Scaffold1006*	674826	(C/T)
CakSNP14185	*Kabuli*	*Ca_Kabuli_Scaffold1006*	674844	(T/C)

SNP IDs	Cultivars	Chromosomes/scaffolds	Physical positions (bp)	SNPs
CakSNP14186	*Kabuli*	*Ca_Kabuli_Scaffold1006*	674847	(T/G)
CakSNP14187	*Kabuli*	*Ca_Kabuli_Scaffold1006*	674862	(G/A)
CakSNP14188	*Kabuli*	*Ca_Kabuli_Scaffold1006*	674865	(T/C)
CakSNP14189	*Kabuli*	*Ca_Kabuli_Scaffold1006*	674871	(A/G)
CakSNP14190	*Kabuli*	*Ca_Kabuli_Scaffold1006*	674881	(G/A)
CakSNP14191	*Kabuli*	*Ca_Kabuli_Scaffold1006*	674981	(G/A)
CakSNP14192	*Kabuli*	*Ca_Kabuli_Scaffold1006*	674946	(G/A)
CakSNP14193	*Kabuli*	*Ca_Kabuli_Scaffold1010*	90092	(G/A)
CakSNP14194	*Kabuli*	*Ca_Kabuli_Scaffold1013*	56140	(G/A)
CakSNP14195	*Kabuli*	*Ca_Kabuli_Scaffold1013*	56116	(C/T)
CakSNP14196	*Kabuli*	*Ca_Kabuli_Scaffold1013*	56114	(C/A)
CakSNP14197	*Kabuli*	*Ca_Kabuli_Scaffold1013*	56075	(T/A)
CakSNP14198	*Kabuli*	*Ca_Kabuli_Scaffold1013*	56074	(C/T)
CakSNP14199	*Kabuli*	*Ca_Kabuli_Scaffold1013*	56069	(C/T)
CakSNP14200	*Kabuli*	*Ca_Kabuli_Scaffold10186*	416	(C/T)
CakSNP14201	*Kabuli*	*Ca_Kabuli_Scaffold10186*	410	(A/C)
CakSNP14202	*Kabuli*	*Ca_Kabuli_Scaffold10186*	378	(A/T)
CakSNP14203	*Kabuli*	*Ca_Kabuli_Scaffold10186*	354	(T/A)
CakSNP14204	*Kabuli*	*Ca_Kabuli_Scaffold10186*	397	(G/A)
CakSNP14205	*Kabuli*	*Ca_Kabuli_Scaffold10186*	573	(T/G)
CakSNP14206	*Kabuli*	*Ca_Kabuli_Scaffold102*	82102	(T/C)
CakSNP14207	*Kabuli*	*Ca_Kabuli_Scaffold102*	82107	(C/A)
CakSNP14208	*Kabuli*	*Ca_Kabuli_Scaffold1027*	256612	(A/C)
CakSNP14209	*Kabuli*	*Ca_Kabuli_Scaffold1034*	73684	(G/A)
CakSNP14210	*Kabuli*	*Ca_Kabuli_Scaffold1034*	73695	(G/T)
CakSNP14211	*Kabuli*	*Ca_Kabuli_Scaffold10420*	19974	(A/G)
CakSNP14212	*Kabuli*	*Ca_Kabuli_Scaffold10420*	31513	(C/T)
CakSNP14213	*Kabuli*	*Ca_Kabuli_Scaffold10420*	31563	(A/G)
CakSNP14214	*Kabuli*	*Ca_Kabuli_Scaffold10420*	31817	(C/T)
CakSNP14215	*Kabuli*	*Ca_Kabuli_Scaffold10420*	40193	(G/T)
CakSNP14216	*Kabuli*	*Ca_Kabuli_Scaffold1043*	16328	(A/T)
CakSNP14217	*Kabuli*	*Ca_Kabuli_Scaffold1043*	16331	(G/T)
CakSNP14218	*Kabuli*	*Ca_Kabuli_Scaffold1043*	16333	(A/T)
CakSNP14219	*Kabuli*	*Ca_Kabuli_Scaffold1043*	16313	(A/G)
CakSNP14220	*Kabuli*	*Ca_Kabuli_Scaffold1047*	235352	(T/C)
CakSNP14221	*Kabuli*	*Ca_Kabuli_Scaffold1047*	577874	(T/G)
CakSNP14222	*Kabuli*	*Ca_Kabuli_Scaffold1050*	3398	(G/A)
CakSNP14223	*Kabuli*	*Ca_Kabuli_Scaffold1050*	3415	(C/T)
CakSNP14224	*Kabuli*	*Ca_Kabuli_Scaffold1050*	3463	(C/T)
CakSNP14225	*Kabuli*	*Ca_Kabuli_Scaffold1050*	14057	(C/A)
CakSNP14226	*Kabuli*	*Ca_Kabuli_Scaffold1050*	126986	(T/G)

**SNP IDs**	**Cultivars**	**Chromosomes/scaffolds**	**Physical positions (bp)**	**SNPs**
CakSNP14227	*Kabuli*	*Ca_Kabuli_Scaffold1050*	127022	(G/A)
CakSNP14228	*Kabuli*	*Ca_Kabuli_Scaffold1050*	127023	(G/T)
CakSNP14229	*Kabuli*	*Ca_Kabuli_Scaffold1050*	127054	(C/T)
CakSNP14230	*Kabuli*	*Ca_Kabuli_Scaffold1050*	128763	(A/G)
CakSNP14231	*Kabuli*	*Ca_Kabuli_Scaffold1055*	344459	(G/A)
CakSNP14232	*Kabuli*	*Ca_Kabuli_Scaffold1055*	344443	(G/A)
CakSNP14233	*Kabuli*	*Ca_Kabuli_Scaffold1055*	344399	(C/T)
CakSNP14234	*Kabuli*	*Ca_Kabuli_Scaffold1060*	3278	(A/C)
CakSNP14235	*Kabuli*	*Ca_Kabuli_Scaffold1060*	3310	(G/T)
CakSNP14236	*Kabuli*	*Ca_Kabuli_Scaffold1060*	394867	(C/T)
CakSNP14237	*Kabuli*	*Ca_Kabuli_Scaffold1060*	395029	(C/T)
CakSNP14238	*Kabuli*	*Ca_Kabuli_Scaffold1060*	395042	(A/G)
CakSNP14239	*Kabuli*	*Ca_Kabuli_Scaffold1061*	63613	(A/G)
CakSNP14240	*Kabuli*	*Ca_Kabuli_Scaffold1061*	63620	(C/T)
CakSNP14241	*Kabuli*	*Ca_Kabuli_Scaffold1065*	142678	(A/C)
CakSNP14242	*Kabuli*	*Ca_Kabuli_Scaffold1067*	38623	(C/A)
CakSNP14243	*Kabuli*	*Ca_Kabuli_Scaffold1081*	768	(A/C)
CakSNP14244	*Kabuli*	*Ca_Kabuli_Scaffold1081*	798	(T/C)
CakSNP14245	*Kabuli*	*Ca_Kabuli_Scaffold1081*	800	(T/C)
CakSNP14246	*Kabuli*	*Ca_Kabuli_Scaffold1081*	805	(A/C)
CakSNP14247	*Kabuli*	*Ca_Kabuli_Scaffold1081*	818	(A/C)
CakSNP14248	*Kabuli*	*Ca_Kabuli_Scaffold1089*	1154	(C/A)
CakSNP14249	*Kabuli*	*Ca_Kabuli_Scaffold1089*	1240	(T/G)
CakSNP14250	*Kabuli*	*Ca_Kabuli_Scaffold1089*	1281	(G/A)
CakSNP14251	*Kabuli*	*Ca_Kabuli_Scaffold1089*	1278	(T/C)
CakSNP14252	*Kabuli*	*Ca_Kabuli_Scaffold1089*	3160	(C/A)
CakSNP14253	*Kabuli*	*Ca_Kabuli_Scaffold1089*	3173	(G/T)
CakSNP14254	*Kabuli*	*Ca_Kabuli_Scaffold1089*	3204	(G/C)
CakSNP14255	*Kabuli*	*Ca_Kabuli_Scaffold1089*	3236	(T/G)
CakSNP14256	*Kabuli*	*Ca_Kabuli_Scaffold1089*	3249	(T/G)
CakSNP14257	*Kabuli*	*Ca_Kabuli_Scaffold1089*	3248	(C/T)
CakSNP14258	*Kabuli*	*Ca_Kabuli_Scaffold1089*	3240	(G/C)
CakSNP14259	*Kabuli*	*Ca_Kabuli_Scaffold1089*	3228	(C/T)
CakSNP14260	*Kabuli*	*Ca_Kabuli_Scaffold1089*	3191	(G/A)
CakSNP14261	*Kabuli*	*Ca_Kabuli_Scaffold1089*	3274	(T/C)
CakSNP14262	*Kabuli*	*Ca_Kabuli_Scaffold1089*	137675	(T/C)
CakSNP14263	*Kabuli*	*Ca_Kabuli_Scaffold1089*	137679	(G/T)
CakSNP14264	*Kabuli*	*Ca_Kabuli_Scaffold109_1*	156485	(C/T)
CakSNP14265	*Kabuli*	*Ca_Kabuli_Scaffold109_1*	230701	(G/C)
CakSNP14266	*Kabuli*	*Ca_Kabuli_Scaffold109_1*	244141	(A/C)
CakSNP14267	*Kabuli*	*Ca_Kabuli_Scaffold109_1*	244150	(C/T)

SNP IDs	Cultivars	Chromosomes/scaffolds	Physical positions (bp)	SNPs
CakSNP14268	*Kabuli*	*Ca_Kabuli_Scaffold109_1*	244156	(A/C)
CakSNP14269	*Kabuli*	*Ca_Kabuli_Scaffold109_1*	244176	(A/T)
CakSNP14270	*Kabuli*	*Ca_Kabuli_Scaffold109_1*	328757	(C/T)
CakSNP14271	*Kabuli*	*Ca_Kabuli_Scaffold109_1*	343415	(G/A)
CakSNP14272	*Kabuli*	*Ca_Kabuli_Scaffold109_1*	343454	(G/A)
CakSNP14273	*Kabuli*	*Ca_Kabuli_Scaffold109_1*	385548	(T/C)
CakSNP14274	*Kabuli*	*Ca_Kabuli_Scaffold109_1*	385578	(C/G)
CakSNP14275	*Kabuli*	*Ca_Kabuli_Scaffold109_1*	385602	(C/T)
CakSNP14276	*Kabuli*	*Ca_Kabuli_Scaffold109_1*	385660	(G/A)
CakSNP14277	*Kabuli*	*Ca_Kabuli_Scaffold109_1*	385653	(C/T)
CakSNP14278	*Kabuli*	*Ca_Kabuli_Scaffold109_1*	442149	(A/T)
CakSNP14279	*Kabuli*	*Ca_Kabuli_Scaffold109_1*	535504	(G/C)
CakSNP14280	*Kabuli*	*Ca_Kabuli_Scaffold109_1*	586181	(A/C)
CakSNP14281	*Kabuli*	*Ca_Kabuli_Scaffold109_1*	586286	(C/T)
CakSNP14282	*Kabuli*	*Ca_Kabuli_Scaffold109_1*	696869	(A/C)
CakSNP14283	*Kabuli*	*Ca_Kabuli_Scaffold109_1*	734137	(G/A)
CakSNP14284	*Kabuli*	*Ca_Kabuli_Scaffold109_1*	734141	(C/T)
CakSNP14285	*Kabuli*	*Ca_Kabuli_Scaffold109_1*	741607	(G/A)
CakSNP14286	*Kabuli*	*Ca_Kabuli_Scaffold109_1*	763160	(G/A)
CakSNP14287	*Kabuli*	*Ca_Kabuli_Scaffold1109*	150672	(G/A)
CakSNP14288	*Kabuli*	*Ca_Kabuli_Scaffold1109*	150720	(G/A)
CakSNP14289	*Kabuli*	*Ca_Kabuli_Scaffold1109*	150676	(C/T)
CakSNP14290	*Kabuli*	*Ca_Kabuli_Scaffold1115*	6234	(G/C)
CakSNP14291	*Kabuli*	*Ca_Kabuli_Scaffold1115*	131039	(G/A)
CakSNP14292	*Kabuli*	*Ca_Kabuli_Scaffold1115*	153349	(T/C)
CakSNP14293	*Kabuli*	*Ca_Kabuli_Scaffold1118*	2215	(T/C)
CakSNP14294	*Kabuli*	*Ca_Kabuli_Scaffold1118*	2224	(T/A)
CakSNP14295	*Kabuli*	*Ca_Kabuli_Scaffold1118*	2225	(C/T)
CakSNP14296	*Kabuli*	*Ca_Kabuli_Scaffold1118*	2226	(T/G)
CakSNP14297	*Kabuli*	*Ca_Kabuli_Scaffold1118*	2315	(T/C)
CakSNP14298	*Kabuli*	*Ca_Kabuli_Scaffold1118*	9568	(A/G)
CakSNP14299	*Kabuli*	*Ca_Kabuli_Scaffold1118*	9556	(C/T)
CakSNP14300	*Kabuli*	*Ca_Kabuli_Scaffold1128*	94196	(T/A)
CakSNP14301	*Kabuli*	*Ca_Kabuli_Scaffold1128*	112702	(G/A)
CakSNP14302	*Kabuli*	*Ca_Kabuli_Scaffold1128*	341416	(T/A)
CakSNP14303	*Kabuli*	*Ca_Kabuli_Scaffold1128*	360567	(T/A)
CakSNP14304	*Kabuli*	*Ca_Kabuli_Scaffold1128*	360529	(A/C)
CakSNP14305	*Kabuli*	*Ca_Kabuli_Scaffold1128*	423553	(A/G)
CakSNP14306	*Kabuli*	*Ca_Kabuli_Scaffold1132*	26497	(C/T)
CakSNP14307	*Kabuli*	*Ca_Kabuli_Scaffold1132*	26492	(G/A)
CakSNP14308	*Kabuli*	*Ca_Kabuli_Scaffold1134*	50630	(T/C)

SNP IDs	Cultivars	Chromosomes/scaffolds	Physical positions (bp)	SNPs
CakSNP14309	*Kabuli*	*Ca_Kabuli_Scaffold1151*	228312	(C/G)
CakSNP14310	*Kabuli*	*Ca_Kabuli_Scaffold1151*	228304	(A/T)
CakSNP14311	*Kabuli*	*Ca_Kabuli_Scaffold1151*	228291	(C/T)
CakSNP14312	*Kabuli*	*Ca_Kabuli_Scaffold1152*	9541	(A/G)
CakSNP14313	*Kabuli*	*Ca_Kabuli_Scaffold1176_2*	52186	(C/T)
CakSNP14314	*Kabuli*	*Ca_Kabuli_Scaffold1176_2*	52146	(T/C)
CakSNP14315	*Kabuli*	*Ca_Kabuli_Scaffold1176_2*	52142	(G/T)
CakSNP14316	*Kabuli*	*Ca_Kabuli_Scaffold1176_2*	52125	(C/T)
CakSNP14317	*Kabuli*	*Ca_Kabuli_Scaffold1176_2*	52124	(C/T)
CakSNP14318	*Kabuli*	*Ca_Kabuli_Scaffold1176_2*	330840	(C/A)
CakSNP14319	*Kabuli*	*Ca_Kabuli_Scaffold118*	206562	(A/G)
CakSNP14320	*Kabuli*	*Ca_Kabuli_Scaffold118*	303784	(A/C)
CakSNP14321	*Kabuli*	*Ca_Kabuli_Scaffold118*	332673	(T/C)
CakSNP14322	*Kabuli*	*Ca_Kabuli_Scaffold1180*	114508	(C/A)
CakSNP14323	*Kabuli*	*Ca_Kabuli_Scaffold1185*	944	(G/A)
CakSNP14324	*Kabuli*	*Ca_Kabuli_Scaffold1185*	1110	(G/A)
CakSNP14325	*Kabuli*	*Ca_Kabuli_Scaffold1185*	1134	(T/A)
CakSNP14326	*Kabuli*	*Ca_Kabuli_Scaffold1185*	1098	(T/C)
CakSNP14327	*Kabuli*	*Ca_Kabuli_Scaffold119*	12259	(A/G)
CakSNP14328	*Kabuli*	*Ca_Kabuli_Scaffold119*	12263	(C/A)
CakSNP14329	*Kabuli*	*Ca_Kabuli_Scaffold119*	12267	(C/A)
CakSNP14330	*Kabuli*	*Ca_Kabuli_Scaffold119*	12279	(G/A)
CakSNP14331	*Kabuli*	*Ca_Kabuli_Scaffold1196*	70506	(G/A)
CakSNP14332	*Kabuli*	*Ca_Kabuli_Scaffold1196*	70552	(A/C)
CakSNP14333	*Kabuli*	*Ca_Kabuli_Scaffold1196*	70549	(G/A)
CakSNP14334	*Kabuli*	*Ca_Kabuli_Scaffold1196*	70547	(G/C)
CakSNP14335	*Kabuli*	*Ca_Kabuli_Scaffold1196*	70535	(G/A)
CakSNP14336	*Kabuli*	*Ca_Kabuli_Scaffold1196*	70530	(C/T)
CakSNP14337	*Kabuli*	*Ca_Kabuli_Scaffold1196*	70526	(T/C)
CakSNP14338	*Kabuli*	*Ca_Kabuli_Scaffold1196*	70520	(G/A)
CakSNP14339	*Kabuli*	*Ca_Kabuli_Scaffold1196*	70503	(C/T)
CakSNP14340	*Kabuli*	*Ca_Kabuli_Scaffold1196*	203001	(G/A)
CakSNP14341	*Kabuli*	*Ca_Kabuli_Scaffold1197*	219637	(A/T)
CakSNP14342	*Kabuli*	*Ca_Kabuli_Scaffold1197*	344239	(A/C)
CakSNP14343	*Kabuli*	*Ca_Kabuli_Scaffold1197*	402506	(T/C)
CakSNP14344	*Kabuli*	*Ca_Kabuli_Scaffold1197*	407641	(G/A)
CakSNP14345	*Kabuli*	*Ca_Kabuli_Scaffold1197*	407658	(C/G)
CakSNP14346	*Kabuli*	*Ca_Kabuli_Scaffold1197*	407691	(T/C)
CakSNP14347	*Kabuli*	*Ca_Kabuli_Scaffold1197*	407695	(T/C)
CakSNP14348	*Kabuli*	*Ca_Kabuli_Scaffold1197*	447890	(C/T)
CakSNP14349	*Kabuli*	*Ca_Kabuli_Scaffold1197*	520164	(T/A)

SNP IDs	Cultivars	Chromosomes/scaffolds	Physical positions (bp)	SNPs
CakSNP14350	*Kabuli*	*Ca_Kabuli_Scaffold1197*	520153	(C/G)
CakSNP14351	*Kabuli*	*Ca_Kabuli_Scaffold1197*	520148	(A/T)
CakSNP14352	*Kabuli*	*Ca_Kabuli_Scaffold12*	107756	(G/A)
CakSNP14353	*Kabuli*	*Ca_Kabuli_Scaffold12*	200573	(G/C)
CakSNP14354	*Kabuli*	*Ca_Kabuli_Scaffold12*	331877	(G/A)
CakSNP14355	*Kabuli*	*Ca_Kabuli_Scaffold12*	331890	(A/G)
CakSNP14356	*Kabuli*	*Ca_Kabuli_Scaffold12*	332909	(C/T)
CakSNP14357	*Kabuli*	*Ca_Kabuli_Scaffold12*	332921	(G/A)
CakSNP14358	*Kabuli*	*Ca_Kabuli_Scaffold12*	332963	(C/T)
CakSNP14359	*Kabuli*	*Ca_Kabuli_Scaffold1202*	46904	(C/T)
CakSNP14360	*Kabuli*	*Ca_Kabuli_Scaffold1202*	46945	(G/A)
CakSNP14361	*Kabuli*	*Ca_Kabuli_Scaffold1215*	40741	(G/T)
CakSNP14362	*Kabuli*	*Ca_Kabuli_Scaffold1215*	40793	(A/G)
CakSNP14363	*Kabuli*	*Ca_Kabuli_Scaffold1215*	40797	(A/T)
CakSNP14364	*Kabuli*	*Ca_Kabuli_Scaffold1215*	40817	(G/A)
CakSNP14365	*Kabuli*	*Ca_Kabuli_Scaffold1215*	40814	(A/T)
CakSNP14366	*Kabuli*	*Ca_Kabuli_Scaffold1219*	34791	(G/A)
CakSNP14367	*Kabuli*	*Ca_Kabuli_Scaffold1219*	85015	(A/C)
CakSNP14368	*Kabuli*	*Ca_Kabuli_Scaffold1259*	71428	(C/T)
CakSNP14369	*Kabuli*	*Ca_Kabuli_Scaffold1259*	71574	(A/T)
CakSNP14370	*Kabuli*	*Ca_Kabuli_Scaffold1259*	71649	(C/T)
CakSNP14371	*Kabuli*	*Ca_Kabuli_Scaffold127*	28809	(A/T)
CakSNP14372	*Kabuli*	*Ca_Kabuli_Scaffold127*	28780	(G/A)
CakSNP14373	*Kabuli*	*Ca_Kabuli_Scaffold127*	28778	(C/T)
CakSNP14374	*Kabuli*	*Ca_Kabuli_Scaffold127*	28776	(T/G)
CakSNP14375	*Kabuli*	*Ca_Kabuli_Scaffold127*	28772	(G/A)
CakSNP14376	*Kabuli*	*Ca_Kabuli_Scaffold1272*	57303	(C/A)
CakSNP14377	*Kabuli*	*Ca_Kabuli_Scaffold1272*	57356	(C/G)
CakSNP14378	*Kabuli*	*Ca_Kabuli_Scaffold128*	19256	(A/C)
CakSNP14379	*Kabuli*	*Ca_Kabuli_Scaffold128*	19315	(C/T)
CakSNP14380	*Kabuli*	*Ca_Kabuli_Scaffold128*	52881	(G/A)
CakSNP14381	*Kabuli*	*Ca_Kabuli_Scaffold128*	52855	(G/C)
CakSNP14382	*Kabuli*	*Ca_Kabuli_Scaffold128*	52844	(A/T)
CakSNP14383	*Kabuli*	*Ca_Kabuli_Scaffold128*	263590	(G/T)
CakSNP14384	*Kabuli*	*Ca_Kabuli_Scaffold128*	263586	(T/A)
CakSNP14385	*Kabuli*	*Ca_Kabuli_Scaffold128*	263572	(T/A)
CakSNP14386	*Kabuli*	*Ca_Kabuli_Scaffold128*	334180	(G/T)
CakSNP14387	*Kabuli*	*Ca_Kabuli_Scaffold128*	334129	(G/A)
CakSNP14388	*Kabuli*	*Ca_Kabuli_Scaffold128*	593782	(T/C)
CakSNP14389	*Kabuli*	*Ca_Kabuli_Scaffold128*	860975	(G/A)
CakSNP14390	*Kabuli*	*Ca_Kabuli_Scaffold128*	860981	(T/A)

SNP IDs	Cultivars	Chromosomes/scaffolds	Physical positions (bp)	SNPs
CakSNP14391	*Kabuli*	*Ca_Kabuli_Scaffold128*	860983	(A/T)
CakSNP14392	*Kabuli*	*Ca_Kabuli_Scaffold128*	860987	(C/T)
CakSNP14393	*Kabuli*	*Ca_Kabuli_Scaffold128*	861003	(C/A)
CakSNP14394	*Kabuli*	*Ca_Kabuli_Scaffold128*	861010	(C/T)
CakSNP14395	*Kabuli*	*Ca_Kabuli_Scaffold128*	861022	(T/C)
CakSNP14396	*Kabuli*	*Ca_Kabuli_Scaffold128*	861028	(T/C)
CakSNP14397	*Kabuli*	*Ca_Kabuli_Scaffold128*	861072	(G/T)
CakSNP14398	*Kabuli*	*Ca_Kabuli_Scaffold128*	861053	(G/A)
CakSNP14399	*Kabuli*	*Ca_Kabuli_Scaffold128*	861036	(G/A)
CakSNP14400	*Kabuli*	*Ca_Kabuli_Scaffold128*	861015	(C/T)
CakSNP14401	*Kabuli*	*Ca_Kabuli_Scaffold1281*	211456	(A/T)
CakSNP14402	*Kabuli*	*Ca_Kabuli_Scaffold1281*	239742	(T/C)
CakSNP14403	*Kabuli*	*Ca_Kabuli_Scaffold1281*	244153	(A/C)
CakSNP14404	*Kabuli*	*Ca_Kabuli_Scaffold1281*	258565	(G/A)
CakSNP14405	*Kabuli*	*Ca_Kabuli_Scaffold1281*	316037	(A/G)
CakSNP14406	*Kabuli*	*Ca_Kabuli_Scaffold1281*	396888	(G/A)
CakSNP14407	*Kabuli*	*Ca_Kabuli_Scaffold1281*	396996	(C/T)
CakSNP14408	*Kabuli*	*Ca_Kabuli_Scaffold1281*	396992	(A/G)
CakSNP14409	*Kabuli*	*Ca_Kabuli_Scaffold1281*	398693	(G/A)
CakSNP14410	*Kabuli*	*Ca_Kabuli_Scaffold1281*	398729	(T/G)
CakSNP14411	*Kabuli*	*Ca_Kabuli_Scaffold1281*	398887	(G/A)
CakSNP14412	*Kabuli*	*Ca_Kabuli_Scaffold1281*	408214	(C/T)
CakSNP14413	*Kabuli*	*Ca_Kabuli_Scaffold1281*	408170	(C/T)
CakSNP14414	*Kabuli*	*Ca_Kabuli_Scaffold1281*	526446	(C/T)
CakSNP14415	*Kabuli*	*Ca_Kabuli_Scaffold1281*	526442	(T/C)
CakSNP14416	*Kabuli*	*Ca_Kabuli_Scaffold1281*	527012	(C/T)
CakSNP14417	*Kabuli*	*Ca_Kabuli_Scaffold1281*	527008	(T/C)
CakSNP14418	*Kabuli*	*Ca_Kabuli_Scaffold1285*	68330	(G/A)
CakSNP14419	*Kabuli*	*Ca_Kabuli_Scaffold1285*	68327	(G/T)
CakSNP14420	*Kabuli*	*Ca_Kabuli_Scaffold1285*	68324	(T/C)
CakSNP14421	*Kabuli*	*Ca_Kabuli_Scaffold1285*	68315	(T/C)
CakSNP14422	*Kabuli*	*Ca_Kabuli_Scaffold1285*	68311	(G/A)
CakSNP14423	*Kabuli*	*Ca_Kabuli_Scaffold1285*	96310	(A/C)
CakSNP14424	*Kabuli*	*Ca_Kabuli_Scaffold1285*	96358	(T/C)
CakSNP14425	*Kabuli*	*Ca_Kabuli_Scaffold1285*	96429	(C/T)
CakSNP14426	*Kabuli*	*Ca_Kabuli_Scaffold1285*	96428	(G/A)
CakSNP14427	*Kabuli*	*Ca_Kabuli_Scaffold1285*	96414	(C/T)
CakSNP14428	*Kabuli*	*Ca_Kabuli_Scaffold1285*	96411	(G/A)
CakSNP14429	*Kabuli*	*Ca_Kabuli_Scaffold1285*	96409	(C/T)
CakSNP14430	*Kabuli*	*Ca_Kabuli_Scaffold1285*	96399	(C/T)
CakSNP14431	*Kabuli*	*Ca_Kabuli_Scaffold1285*	105553	(T/C)

SNP IDs	Cultivars	Chromosomes/scaffolds	Physical positions (bp)	SNPs
CakSNP14432	*Kabuli*	*Ca_Kabuli_Scaffold1285*	105507	(A/T)
CakSNP14433	*Kabuli*	*Ca_Kabuli_Scaffold1285*	105504	(G/A)
CakSNP14434	*Kabuli*	*Ca_Kabuli_Scaffold1285*	192068	(T/G)
CakSNP14435	*Kabuli*	*Ca_Kabuli_Scaffold1285*	240762	(C/T)
CakSNP14436	*Kabuli*	*Ca_Kabuli_Scaffold1285*	240735	(G/A)
CakSNP14437	*Kabuli*	*Ca_Kabuli_Scaffold1285*	240730	(T/C)
CakSNP14438	*Kabuli*	*Ca_Kabuli_Scaffold1285*	240723	(C/T)
CakSNP14439	*Kabuli*	*Ca_Kabuli_Scaffold1301_1*	43433	(T/C)
CakSNP14440	*Kabuli*	*Ca_Kabuli_Scaffold1301_1*	45565	(A/C)
CakSNP14441	*Kabuli*	*Ca_Kabuli_Scaffold1301_1*	45566	(G/A)
CakSNP14442	*Kabuli*	*Ca_Kabuli_Scaffold1301_1*	58243	(A/G)
CakSNP14443	*Kabuli*	*Ca_Kabuli_Scaffold1301_1*	58337	(C/T)
CakSNP14444	*Kabuli*	*Ca_Kabuli_Scaffold1301_1*	58460	(A/T)
CakSNP14445	*Kabuli*	*Ca_Kabuli_Scaffold1301_1*	63148	(T/C)
CakSNP14446	*Kabuli*	*Ca_Kabuli_Scaffold1301_1*	95495	(G/A)
CakSNP14447	*Kabuli*	*Ca_Kabuli_Scaffold1301_1*	95497	(G/A)
CakSNP14448	*Kabuli*	*Ca_Kabuli_Scaffold1301_1*	95602	(T/C)
CakSNP14449	*Kabuli*	*Ca_Kabuli_Scaffold1301_1*	95533	(C/G)
CakSNP14450	*Kabuli*	*Ca_Kabuli_Scaffold1301_1*	122496	(T/A)
CakSNP14451	*Kabuli*	*Ca_Kabuli_Scaffold1301_1*	122563	(T/C)
CakSNP14452	*Kabuli*	*Ca_Kabuli_Scaffold1301_1*	154342	(A/C)
CakSNP14453	*Kabuli*	*Ca_Kabuli_Scaffold1301_1*	157390	(A/G)
CakSNP14454	*Kabuli*	*Ca_Kabuli_Scaffold1301_1*	218538	(G/A)
CakSNP14455	*Kabuli*	*Ca_Kabuli_Scaffold1308*	44260	(A/G)
CakSNP14456	*Kabuli*	*Ca_Kabuli_Scaffold1309*	49259	(G/A)
CakSNP14457	*Kabuli*	*Ca_Kabuli_Scaffold1312*	98400	(T/C)
CakSNP14458	*Kabuli*	*Ca_Kabuli_Scaffold1313*	18173	(A/G)
CakSNP14459	*Kabuli*	*Ca_Kabuli_Scaffold1313*	18412	(G/A)
CakSNP14460	*Kabuli*	*Ca_Kabuli_Scaffold1313*	227732	(G/A)
CakSNP14461	*Kabuli*	*Ca_Kabuli_Scaffold1313*	227744	(C/T)
CakSNP14462	*Kabuli*	*Ca_Kabuli_Scaffold1315*	115480	(G/A)
CakSNP14463	*Kabuli*	*Ca_Kabuli_Scaffold1315*	115476	(A/C)
CakSNP14464	*Kabuli*	*Ca_Kabuli_Scaffold1315*	115471	(G/A)
CakSNP14465	*Kabuli*	*Ca_Kabuli_Scaffold1315*	115446	(C/A)
CakSNP14466	*Kabuli*	*Ca_Kabuli_Scaffold1315*	115481	(G/T)
CakSNP14467	*Kabuli*	*Ca_Kabuli_Scaffold132*	153364	(A/G)
CakSNP14468	*Kabuli*	*Ca_Kabuli_Scaffold132*	153361	(A/G)
CakSNP14469	*Kabuli*	*Ca_Kabuli_Scaffold1324*	228151	(G/A)
CakSNP14470	*Kabuli*	*Ca_Kabuli_Scaffold1324*	228159	(A/C)
CakSNP14471	*Kabuli*	*Ca_Kabuli_Scaffold1324*	262208	(C/G)
CakSNP14472	*Kabuli*	*Ca_Kabuli_Scaffold134*	218290	(C/T)

SNP IDs	Cultivars	Chromosomes/scaffolds	Physical positions (bp)	SNPs
CakSNP14473	*Kabuli*	*Ca_Kabuli_Scaffold134*	218352	(T/G)
CakSNP14474	*Kabuli*	*Ca_Kabuli_Scaffold134*	218336	(T/G)
CakSNP14475	*Kabuli*	*Ca_Kabuli_Scaffold134*	223318	(C/T)
CakSNP14476	*Kabuli*	*Ca_Kabuli_Scaffold134*	223303	(T/C)
CakSNP14477	*Kabuli*	*Ca_Kabuli_Scaffold134*	233007	(C/T)
CakSNP14478	*Kabuli*	*Ca_Kabuli_Scaffold134*	275661	(T/A)
CakSNP14479	*Kabuli*	*Ca_Kabuli_Scaffold1348_1*	84644	(G/C)
CakSNP14480	*Kabuli*	*Ca_Kabuli_Scaffold1348_1*	134575	(T/C)
CakSNP14481	*Kabuli*	*Ca_Kabuli_Scaffold1348_1*	134572	(G/A)
CakSNP14482	*Kabuli*	*Ca_Kabuli_Scaffold1348_1*	294458	(A/G)
CakSNP14483	*Kabuli*	*Ca_Kabuli_Scaffold1348_1*	294456	(A/G)
CakSNP14484	*Kabuli*	*Ca_Kabuli_Scaffold1348_1*	380156	(A/T)
CakSNP14485	*Kabuli*	*Ca_Kabuli_Scaffold1348_1*	380225	(T/G)
CakSNP14486	*Kabuli*	*Ca_Kabuli_Scaffold1348_1*	380227	(C/G)
CakSNP14487	*Kabuli*	*Ca_Kabuli_Scaffold1348_1*	380244	(C/G)
CakSNP14488	*Kabuli*	*Ca_Kabuli_Scaffold1348_1*	380289	(C/T)
CakSNP14489	*Kabuli*	*Ca_Kabuli_Scaffold1348_1*	380336	(G/C)
CakSNP14490	*Kabuli*	*Ca_Kabuli_Scaffold1348_1*	381026	(G/T)
CakSNP14491	*Kabuli*	*Ca_Kabuli_Scaffold1348_1*	452011	(G/T)
CakSNP14492	*Kabuli*	*Ca_Kabuli_Scaffold1348_1*	454342	(G/T)
CakSNP14493	*Kabuli*	*Ca_Kabuli_Scaffold1348_1*	515602	(A/C)
CakSNP14494	*Kabuli*	*Ca_Kabuli_Scaffold1348_1*	535984	(G/T)
CakSNP14495	*Kabuli*	*Ca_Kabuli_Scaffold1348_1*	552192	(C/A)
CakSNP14496	*Kabuli*	*Ca_Kabuli_Scaffold1348_1*	552299	(T/C)
CakSNP14497	*Kabuli*	*Ca_Kabuli_Scaffold1348_1*	552342	(G/A)
CakSNP14498	*Kabuli*	*Ca_Kabuli_Scaffold1348_1*	552328	(A/G)
CakSNP14499	*Kabuli*	*Ca_Kabuli_Scaffold1348_1*	553863	(G/T)
CakSNP14500	*Kabuli*	*Ca_Kabuli_Scaffold1348_1*	553978	(C/T)
CakSNP14501	*Kabuli*	*Ca_Kabuli_Scaffold1348_1*	554003	(G/C)
CakSNP14502	*Kabuli*	*Ca_Kabuli_Scaffold1348_1*	554134	(A/G)
CakSNP14503	*Kabuli*	*Ca_Kabuli_Scaffold1348_1*	554215	(T/G)
CakSNP14504	*Kabuli*	*Ca_Kabuli_Scaffold1348_1*	651906	(A/T)
CakSNP14505	*Kabuli*	*Ca_Kabuli_Scaffold1348_1*	652067	(A/G)
CakSNP14506	*Kabuli*	*Ca_Kabuli_Scaffold1348_1*	667541	(A/C)
CakSNP14507	*Kabuli*	*Ca_Kabuli_Scaffold1348_1*	667504	(C/T)
CakSNP14508	*Kabuli*	*Ca_Kabuli_Scaffold1348_1*	679305	(T/C)
CakSNP14509	*Kabuli*	*Ca_Kabuli_Scaffold1348_1*	680734	(G/T)
CakSNP14510	*Kabuli*	*Ca_Kabuli_Scaffold1348_1*	680730	(C/T)
CakSNP14511	*Kabuli*	*Ca_Kabuli_Scaffold1348_1*	680718	(A/T)
CakSNP14512	*Kabuli*	*Ca_Kabuli_Scaffold1348_1*	708276	(G/A)
CakSNP14513	*Kabuli*	*Ca_Kabuli_Scaffold1348_1*	708302	(G/T)

**SNP IDs**	**Cultivars**	**Chromosomes/scaffolds**	**Physical positions (bp)**	**SNPs**
CakSNP14514	*Kabuli*	*Ca_Kabuli_Scaffold1348_1*	708344	(G/A)
CakSNP14515	*Kabuli*	*Ca_Kabuli_Scaffold1348_1*	732450	(A/C)
CakSNP14516	*Kabuli*	*Ca_Kabuli_Scaffold1348_1*	732454	(T/G)
CakSNP14517	*Kabuli*	*Ca_Kabuli_Scaffold1348_1*	732460	(T/A)
CakSNP14518	*Kabuli*	*Ca_Kabuli_Scaffold1348_1*	732496	(G/T)
CakSNP14519	*Kabuli*	*Ca_Kabuli_Scaffold1348_1*	735782	(C/T)
CakSNP14520	*Kabuli*	*Ca_Kabuli_Scaffold1348_1*	735812	(G/A)
CakSNP14521	*Kabuli*	*Ca_Kabuli_Scaffold1348_1*	750170	(C/A)
CakSNP14522	*Kabuli*	*Ca_Kabuli_Scaffold1348_1*	750162	(T/C)
CakSNP14523	*Kabuli*	*Ca_Kabuli_Scaffold1348_1*	750213	(C/A)
CakSNP14524	*Kabuli*	*Ca_Kabuli_Scaffold1348_1*	771029	(T/A)
CakSNP14525	*Kabuli*	*Ca_Kabuli_Scaffold1348_1*	871724	(G/A)
CakSNP14526	*Kabuli*	*Ca_Kabuli_Scaffold1348_1*	909909	(A/G)
CakSNP14527	*Kabuli*	*Ca_Kabuli_Scaffold1348_1*	909967	(C/G)
CakSNP14528	*Kabuli*	*Ca_Kabuli_Scaffold1348_1*	909965	(T/A)
CakSNP14529	*Kabuli*	*Ca_Kabuli_Scaffold1348_1*	909959	(A/C)
CakSNP14530	*Kabuli*	*Ca_Kabuli_Scaffold1348_1*	924164	(T/G)
CakSNP14531	*Kabuli*	*Ca_Kabuli_Scaffold1348_1*	934720	(C/A)
CakSNP14532	*Kabuli*	*Ca_Kabuli_Scaffold1348_1*	934791	(C/T)
CakSNP14533	*Kabuli*	*Ca_Kabuli_Scaffold1348_1*	950995	(A/G)
CakSNP14534	*Kabuli*	*Ca_Kabuli_Scaffold1348_1*	959608	(A/C)
CakSNP14535	*Kabuli*	*Ca_Kabuli_Scaffold1348_1*	959828	(G/A)
CakSNP14536	*Kabuli*	*Ca_Kabuli_Scaffold1348_1*	959780	(G/A)
CakSNP14537	*Kabuli*	*Ca_Kabuli_Scaffold1348_1*	970927	(A/G)
CakSNP14538	*Kabuli*	*Ca_Kabuli_Scaffold1348_1*	981964	(T/C)
CakSNP14539	*Kabuli*	*Ca_Kabuli_Scaffold1348_1*	983101	(T/G)
CakSNP14540	*Kabuli*	*Ca_Kabuli_Scaffold1348_1*	1020258	(C/T)
CakSNP14541	*Kabuli*	*Ca_Kabuli_Scaffold1348_1*	1023898	(G/A)
CakSNP14542	*Kabuli*	*Ca_Kabuli_Scaffold1348_1*	1023923	(G/A)
CakSNP14543	*Kabuli*	*Ca_Kabuli_Scaffold1348_1*	1023954	(G/C)
CakSNP14544	*Kabuli*	*Ca_Kabuli_Scaffold1348_1*	1066818	(A/C)
CakSNP14545	*Kabuli*	*Ca_Kabuli_Scaffold1348_1*	1066783	(C/T)
CakSNP14546	*Kabuli*	*Ca_Kabuli_Scaffold1348_1*	1066776	(T/C)
CakSNP14547	*Kabuli*	*Ca_Kabuli_Scaffold1348_1*	1072750	(C/T)
CakSNP14548	*Kabuli*	*Ca_Kabuli_Scaffold1348_1*	1145082	(A/C)
CakSNP14549	*Kabuli*	*Ca_Kabuli_Scaffold1348_1*	1145124	(T/C)
CakSNP14550	*Kabuli*	*Ca_Kabuli_Scaffold1348_1*	1207442	(G/A)
CakSNP14551	*Kabuli*	*Ca_Kabuli_Scaffold1348_1*	1236956	(A/G)
CakSNP14552	*Kabuli*	*Ca_Kabuli_Scaffold1348_1*	1263560	(T/G)
CakSNP14553	*Kabuli*	*Ca_Kabuli_Scaffold1348_1*	1265627	(T/C)
CakSNP14554	*Kabuli*	*Ca_Kabuli_Scaffold1348_1*	1265635	(T/G)

**SNP IDs**	**Cultivars**	**Chromosomes/scaffolds**	**Physical positions (bp)**	**SNPs**
CakSNP14555	*Kabuli*	*Ca_Kabuli_Scaffold1348_1*	1273341	(G/A)
CakSNP14556	*Kabuli*	*Ca_Kabuli_Scaffold1348_1*	1273388	(G/A)
CakSNP14557	*Kabuli*	*Ca_Kabuli_Scaffold1348_1*	1288448	(A/G)
CakSNP14558	*Kabuli*	*Ca_Kabuli_Scaffold1348_1*	1294675	(A/T)
CakSNP14559	*Kabuli*	*Ca_Kabuli_Scaffold1348_1*	1296453	(G/A)
CakSNP14560	*Kabuli*	*Ca_Kabuli_Scaffold1348_1*	1330256	(A/G)
CakSNP14561	*Kabuli*	*Ca_Kabuli_Scaffold1348_1*	1330288	(A/G)
CakSNP14562	*Kabuli*	*Ca_Kabuli_Scaffold1348_1*	1330400	(A/G)
CakSNP14563	*Kabuli*	*Ca_Kabuli_Scaffold1348_1*	1346914	(G/T)
CakSNP14564	*Kabuli*	*Ca_Kabuli_Scaffold1348_1*	1346943	(G/A)
CakSNP14565	*Kabuli*	*Ca_Kabuli_Scaffold1348_1*	1372535	(G/A)
CakSNP14566	*Kabuli*	*Ca_Kabuli_Scaffold1348_1*	1403452	(G/A)
CakSNP14567	*Kabuli*	*Ca_Kabuli_Scaffold1348_1*	1403480	(A/G)
CakSNP14568	*Kabuli*	*Ca_Kabuli_Scaffold1351*	172111	(G/A)
CakSNP14569	*Kabuli*	*Ca_Kabuli_Scaffold1351*	172110	(T/C)
CakSNP14570	*Kabuli*	*Ca_Kabuli_Scaffold1351*	177159	(G/A)
CakSNP14571	*Kabuli*	*Ca_Kabuli_Scaffold1351*	177172	(G/A)
CakSNP14572	*Kabuli*	*Ca_Kabuli_Scaffold1351*	177178	(T/C)
CakSNP14573	*Kabuli*	*Ca_Kabuli_Scaffold1351*	177181	(A/G)
CakSNP14574	*Kabuli*	*Ca_Kabuli_Scaffold1351*	177194	(G/A)
CakSNP14575	*Kabuli*	*Ca_Kabuli_Scaffold1351*	177202	(G/A)
CakSNP14576	*Kabuli*	*Ca_Kabuli_Scaffold1351*	177171	(T/G)
CakSNP14577	*Kabuli*	*Ca_Kabuli_Scaffold1351*	177193	(T/C)
CakSNP14578	*Kabuli*	*Ca_Kabuli_Scaffold1351*	177256	(T/G)
CakSNP14579	*Kabuli*	*Ca_Kabuli_Scaffold1351*	177248	(G/T)
CakSNP14580	*Kabuli*	*Ca_Kabuli_Scaffold1351*	177247	(G/A)
CakSNP14581	*Kabuli*	*Ca_Kabuli_Scaffold1351*	177242	(G/A)
CakSNP14582	*Kabuli*	*Ca_Kabuli_Scaffold1351*	177241	(T/G)
CakSNP14583	*Kabuli*	*Ca_Kabuli_Scaffold1351*	177240	(C/A)
CakSNP14584	*Kabuli*	*Ca_Kabuli_Scaffold1351*	177235	(G/T)
CakSNP14585	*Kabuli*	*Ca_Kabuli_Scaffold1351*	177234	(A/C)
CakSNP14586	*Kabuli*	*Ca_Kabuli_Scaffold1351*	177210	(T/A)
CakSNP14587	*Kabuli*	*Ca_Kabuli_Scaffold1351*	177200	(T/G)
CakSNP14588	*Kabuli*	*Ca_Kabuli_Scaffold1351*	177191	(C/T)
CakSNP14589	*Kabuli*	*Ca_Kabuli_Scaffold1351*	177188	(G/T)
CakSNP14590	*Kabuli*	*Ca_Kabuli_Scaffold1351*	204656	(C/A)
CakSNP14591	*Kabuli*	*Ca_Kabuli_Scaffold1351*	335227	(T/C)
CakSNP14592	*Kabuli*	*Ca_Kabuli_Scaffold1351*	335239	(G/T)
CakSNP14593	*Kabuli*	*Ca_Kabuli_Scaffold1351*	367576	(A/G)
CakSNP14594	*Kabuli*	*Ca_Kabuli_Scaffold1351*	367578	(G/A)
CakSNP14595	*Kabuli*	*Ca_Kabuli_Scaffold1351*	367745	(G/A)

SNP IDs	Cultivars	Chromosomes/scaffolds	Physical positions (bp)	SNPs
CakSNP14596	*Kabuli*	*Ca_Kabuli_Scaffold1369*	8015	(T/A)
CakSNP14597	*Kabuli*	*Ca_Kabuli_Scaffold1369*	8115	(T/G)
CakSNP14598	*Kabuli*	*Ca_Kabuli_Scaffold1369*	8154	(T/G)
CakSNP14599	*Kabuli*	*Ca_Kabuli_Scaffold1369*	8221	(T/G)
CakSNP14600	*Kabuli*	*Ca_Kabuli_Scaffold1369*	8327	(G/A)
CakSNP14601	*Kabuli*	*Ca_Kabuli_Scaffold1369*	31000	(A/G)
CakSNP14602	*Kabuli*	*Ca_Kabuli_Scaffold1390*	98354	(A/G)
CakSNP14603	*Kabuli*	*Ca_Kabuli_Scaffold1401*	89077	(T/C)
CakSNP14604	*Kabuli*	*Ca_Kabuli_Scaffold1401*	89074	(T/C)
CakSNP14605	*Kabuli*	*Ca_Kabuli_Scaffold1417*	4863	(A/G)
CakSNP14606	*Kabuli*	*Ca_Kabuli_Scaffold1417*	4906	(G/A)
CakSNP14607	*Kabuli*	*Ca_Kabuli_Scaffold1417*	4917	(G/T)
CakSNP14608	*Kabuli*	*Ca_Kabuli_Scaffold1417*	76627	(A/C)
CakSNP14609	*Kabuli*	*Ca_Kabuli_Scaffold1417*	230210	(T/G)
CakSNP14610	*Kabuli*	*Ca_Kabuli_Scaffold1419*	24845	(C/T)
CakSNP14611	*Kabuli*	*Ca_Kabuli_Scaffold143*	18492	(A/G)
CakSNP14612	*Kabuli*	*Ca_Kabuli_Scaffold1439*	242972	(A/G)
CakSNP14613	*Kabuli*	*Ca_Kabuli_Scaffold1448*	47306	(A/G)
CakSNP14614	*Kabuli*	*Ca_Kabuli_Scaffold1448*	74436	(C/T)
CakSNP14615	*Kabuli*	*Ca_Kabuli_Scaffold1448*	74434	(C/T)
CakSNP14616	*Kabuli*	*Ca_Kabuli_Scaffold1448*	74529	(C/T)
CakSNP14617	*Kabuli*	*Ca_Kabuli_Scaffold1448*	76809	(G/A)
CakSNP14618	*Kabuli*	*Ca_Kabuli_Scaffold1449*	55474	(T/G)
CakSNP14619	*Kabuli*	*Ca_Kabuli_Scaffold1449*	55529	(G/A)
CakSNP14620	*Kabuli*	*Ca_Kabuli_Scaffold1449*	55532	(G/A)
CakSNP14621	*Kabuli*	*Ca_Kabuli_Scaffold1452*	3501	(C/A)
CakSNP14622	*Kabuli*	*Ca_Kabuli_Scaffold1452*	3496	(A/T)
CakSNP14623	*Kabuli*	*Ca_Kabuli_Scaffold1452*	3495	(C/A)
CakSNP14624	*Kabuli*	*Ca_Kabuli_Scaffold1452*	3737	(C/A)
CakSNP14625	*Kabuli*	*Ca_Kabuli_Scaffold1462*	1556	(T/G)
CakSNP14626	*Kabuli*	*Ca_Kabuli_Scaffold1466*	90846	(C/A)
CakSNP14627	*Kabuli*	*Ca_Kabuli_Scaffold1466*	165472	(C/T)
CakSNP14628	*Kabuli*	*Ca_Kabuli_Scaffold1466*	322287	(G/A)
CakSNP14629	*Kabuli*	*Ca_Kabuli_Scaffold1466*	322315	(G/T)
CakSNP14630	*Kabuli*	*Ca_Kabuli_Scaffold1466*	322450	(G/T)
CakSNP14631	*Kabuli*	*Ca_Kabuli_Scaffold1466*	322448	(T/G)
CakSNP14632	*Kabuli*	*Ca_Kabuli_Scaffold1466*	322421	(C/T)
CakSNP14633	*Kabuli*	*Ca_Kabuli_Scaffold1466*	322415	(G/A)
CakSNP14634	*Kabuli*	*Ca_Kabuli_Scaffold1466*	322414	(G/A)
CakSNP14635	*Kabuli*	*Ca_Kabuli_Scaffold1466*	322400	(C/T)
CakSNP14636	*Kabuli*	*Ca_Kabuli_Scaffold1466*	322390	(G/A)

SNP IDs	Cultivars	Chromosomes/scaffolds	Physical positions (bp)	SNPs
CakSNP14637	*Kabuli*	*Ca_Kabuli_Scaffold1466*	322383	(C/T)
CakSNP14638	*Kabuli*	*Ca_Kabuli_Scaffold1467*	205779	(A/G)
CakSNP14639	*Kabuli*	*Ca_Kabuli_Scaffold1467*	205803	(C/T)
CakSNP14640	*Kabuli*	*Ca_Kabuli_Scaffold1467*	205833	(G/A)
CakSNP14641	*Kabuli*	*Ca_Kabuli_Scaffold1467*	205840	(G/A)
CakSNP14642	*Kabuli*	*Ca_Kabuli_Scaffold1467*	205845	(A/G)
CakSNP14643	*Kabuli*	*Ca_Kabuli_Scaffold1467*	206121	(G/A)
CakSNP14644	*Kabuli*	*Ca_Kabuli_Scaffold1467*	206118	(C/T)
CakSNP14645	*Kabuli*	*Ca_Kabuli_Scaffold1467*	206100	(G/A)
CakSNP14646	*Kabuli*	*Ca_Kabuli_Scaffold1467*	206094	(T/C)
CakSNP14647	*Kabuli*	*Ca_Kabuli_Scaffold1467*	206085	(T/C)
CakSNP14648	*Kabuli*	*Ca_Kabuli_Scaffold1467*	206068	(A/G)
CakSNP14649	*Kabuli*	*Ca_Kabuli_Scaffold1467*	206067	(T/G)
CakSNP14650	*Kabuli*	*Ca_Kabuli_Scaffold1475*	4820	(G/A)
CakSNP14651	*Kabuli*	*Ca_Kabuli_Scaffold1475*	4796	(C/T)
CakSNP14652	*Kabuli*	*Ca_Kabuli_Scaffold1475*	4794	(C/A)
CakSNP14653	*Kabuli*	*Ca_Kabuli_Scaffold1475*	4755	(T/A)
CakSNP14654	*Kabuli*	*Ca_Kabuli_Scaffold1475*	4754	(C/T)
CakSNP14655	*Kabuli*	*Ca_Kabuli_Scaffold1475*	4749	(C/T)
CakSNP14656	*Kabuli*	*Ca_Kabuli_Scaffold1475*	4873	(G/T)
CakSNP14657	*Kabuli*	*Ca_Kabuli_Scaffold1475*	4845	(A/T)
CakSNP14658	*Kabuli*	*Ca_Kabuli_Scaffold1475*	4833	(G/A)
CakSNP14659	*Kabuli*	*Ca_Kabuli_Scaffold1475*	4818	(C/T)
CakSNP14660	*Kabuli*	*Ca_Kabuli_Scaffold1483*	56686	(A/T)
CakSNP14661	*Kabuli*	*Ca_Kabuli_Scaffold1483*	56721	(G/C)
CakSNP14662	*Kabuli*	*Ca_Kabuli_Scaffold1493*	81740	(C/T)
CakSNP14663	*Kabuli*	*Ca_Kabuli_Scaffold1493*	81784	(C/A)
CakSNP14664	*Kabuli*	*Ca_Kabuli_Scaffold1493*	81789	(G/A)
CakSNP14665	*Kabuli*	*Ca_Kabuli_Scaffold1496*	289997	(G/T)
CakSNP14666	*Kabuli*	*Ca_Kabuli_Scaffold1496*	290018	(C/A)
CakSNP14667	*Kabuli*	*Ca_Kabuli_Scaffold1498*	3474	(A/C)
CakSNP14668	*Kabuli*	*Ca_Kabuli_Scaffold1498*	3657	(C/T)
CakSNP14669	*Kabuli*	*Ca_Kabuli_Scaffold1498*	3641	(T/C)
CakSNP14670	*Kabuli*	*Ca_Kabuli_Scaffold1504*	160623	(G/T)
CakSNP14671	*Kabuli*	*Ca_Kabuli_Scaffold1504*	189700	(A/G)
CakSNP14672	*Kabuli*	*Ca_Kabuli_Scaffold1504*	189699	(C/T)
CakSNP14673	*Kabuli*	*Ca_Kabuli_Scaffold1504*	189681	(C/T)
CakSNP14674	*Kabuli*	*Ca_Kabuli_Scaffold1504*	189664	(G/A)
CakSNP14675	*Kabuli*	*Ca_Kabuli_Scaffold1504*	189663	(G/A)
CakSNP14676	*Kabuli*	*Ca_Kabuli_Scaffold1504*	197789	(G/A)
CakSNP14677	*Kabuli*	*Ca_Kabuli_Scaffold1504*	197790	(G/A)

SNP IDs	Cultivars	Chromosomes/scaffolds	Physical positions (bp)	SNPs
CakSNP14678	*Kabuli*	*Ca_Kabuli_Scaffold1504*	197819	(T/G)
CakSNP14679	*Kabuli*	*Ca_Kabuli_Scaffold1505*	33297	(A/C)
CakSNP14680	*Kabuli*	*Ca_Kabuli_Scaffold151*	267060	(T/G)
CakSNP14681	*Kabuli*	*Ca_Kabuli_Scaffold151*	275743	(T/A)
CakSNP14682	*Kabuli*	*Ca_Kabuli_Scaffold1521*	20734	(C/A)
CakSNP14683	*Kabuli*	*Ca_Kabuli_Scaffold1521*	20779	(T/C)
CakSNP14684	*Kabuli*	*Ca_Kabuli_Scaffold1521*	20851	(C/T)
CakSNP14685	*Kabuli*	*Ca_Kabuli_Scaffold1545*	111476	(G/A)
CakSNP14686	*Kabuli*	*Ca_Kabuli_Scaffold1545*	113148	(T/C)
CakSNP14687	*Kabuli*	*Ca_Kabuli_Scaffold1553*	5078	(G/A)
CakSNP14688	*Kabuli*	*Ca_Kabuli_Scaffold1553*	5115	(C/T)
CakSNP14689	*Kabuli*	*Ca_Kabuli_Scaffold1553*	5125	(A/G)
CakSNP14690	*Kabuli*	*Ca_Kabuli_Scaffold157*	100228	(A/G)
CakSNP14691	*Kabuli*	*Ca_Kabuli_Scaffold157*	100529	(T/C)
CakSNP14692	*Kabuli*	*Ca_Kabuli_Scaffold157*	102779	(C/T)
CakSNP14693	*Kabuli*	*Ca_Kabuli_Scaffold157*	106331	(G/A)
CakSNP14694	*Kabuli*	*Ca_Kabuli_Scaffold157*	106349	(C/T)
CakSNP14695	*Kabuli*	*Ca_Kabuli_Scaffold157*	133602	(A/G)
CakSNP14696	*Kabuli*	*Ca_Kabuli_Scaffold157*	133583	(T/G)
CakSNP14697	*Kabuli*	*Ca_Kabuli_Scaffold157*	141880	(A/G)
CakSNP14698	*Kabuli*	*Ca_Kabuli_Scaffold157*	142444	(A/T)
CakSNP14699	*Kabuli*	*Ca_Kabuli_Scaffold157*	142455	(T/C)
CakSNP14700	*Kabuli*	*Ca_Kabuli_Scaffold157*	142470	(G/T)
CakSNP14701	*Kabuli*	*Ca_Kabuli_Scaffold157*	151081	(G/A)
CakSNP14702	*Kabuli*	*Ca_Kabuli_Scaffold157*	151199	(T/A)
CakSNP14703	*Kabuli*	*Ca_Kabuli_Scaffold157*	151192	(T/C)
CakSNP14704	*Kabuli*	*Ca_Kabuli_Scaffold157*	151177	(A/G)
CakSNP14705	*Kabuli*	*Ca_Kabuli_Scaffold157*	151131	(T/A)
CakSNP14706	*Kabuli*	*Ca_Kabuli_Scaffold157*	213380	(C/G)
CakSNP14707	*Kabuli*	*Ca_Kabuli_Scaffold157*	213398	(T/G)
CakSNP14708	*Kabuli*	*Ca_Kabuli_Scaffold157*	213404	(T/G)
CakSNP14709	*Kabuli*	*Ca_Kabuli_Scaffold157*	213518	(T/C)
CakSNP14710	*Kabuli*	*Ca_Kabuli_Scaffold157*	213487	(G/C)
CakSNP14711	*Kabuli*	*Ca_Kabuli_Scaffold157*	213477	(G/A)
CakSNP14712	*Kabuli*	*Ca_Kabuli_Scaffold157*	229917	(G/A)
CakSNP14713	*Kabuli*	*Ca_Kabuli_Scaffold157*	229900	(G/A)
CakSNP14714	*Kabuli*	*Ca_Kabuli_Scaffold157*	321386	(C/T)
CakSNP14715	*Kabuli*	*Ca_Kabuli_Scaffold157*	326434	(A/G)
CakSNP14716	*Kabuli*	*Ca_Kabuli_Scaffold157*	326467	(C/T)
CakSNP14717	*Kabuli*	*Ca_Kabuli_Scaffold157*	326494	(A/G)
CakSNP14718	*Kabuli*	*Ca_Kabuli_Scaffold157*	326497	(A/G)

SNP IDs	Cultivars	Chromosomes/scaffolds	Physical positions (bp)	SNPs
CakSNP14719	*Kabuli*	*Ca_Kabuli_Scaffold157*	352687	(C/T)
CakSNP14720	*Kabuli*	*Ca_Kabuli_Scaffold157*	352712	(G/A)
CakSNP14721	*Kabuli*	*Ca_Kabuli_Scaffold157*	352747	(T/C)
CakSNP14722	*Kabuli*	*Ca_Kabuli_Scaffold157*	355222	(G/A)
CakSNP14723	*Kabuli*	*Ca_Kabuli_Scaffold157*	469198	(A/C)
CakSNP14724	*Kabuli*	*Ca_Kabuli_Scaffold157*	535057	(T/G)
CakSNP14725	*Kabuli*	*Ca_Kabuli_Scaffold1575*	2230	(C/T)
CakSNP14726	*Kabuli*	*Ca_Kabuli_Scaffold1580*	71861	(G/T)
CakSNP14727	*Kabuli*	*Ca_Kabuli_Scaffold1580*	119410	(T/C)
CakSNP14728	*Kabuli*	*Ca_Kabuli_Scaffold1580*	119678	(T/C)
CakSNP14729	*Kabuli*	*Ca_Kabuli_Scaffold1580*	163148	(A/T)
CakSNP14730	*Kabuli*	*Ca_Kabuli_Scaffold1580*	163194	(T/C)
CakSNP14731	*Kabuli*	*Ca_Kabuli_Scaffold1580*	380649	(A/T)
CakSNP14732	*Kabuli*	*Ca_Kabuli_Scaffold1585*	59010	(C/A)
CakSNP14733	*Kabuli*	*Ca_Kabuli_Scaffold1603*	11828	(C/G)
CakSNP14734	*Kabuli*	*Ca_Kabuli_Scaffold1607*	107222	(T/C)
CakSNP14735	*Kabuli*	*Ca_Kabuli_Scaffold1613*	48917	(T/G)
CakSNP14736	*Kabuli*	*Ca_Kabuli_Scaffold1613*	107664	(T/A)
CakSNP14737	*Kabuli*	*Ca_Kabuli_Scaffold1613*	107750	(T/A)
CakSNP14738	*Kabuli*	*Ca_Kabuli_Scaffold1631*	202418	(A/G)
CakSNP14739	*Kabuli*	*Ca_Kabuli_Scaffold1635*	83227	(G/C)
CakSNP14740	*Kabuli*	*Ca_Kabuli_Scaffold1635*	83345	(A/T)
CakSNP14741	*Kabuli*	*Ca_Kabuli_Scaffold1654*	26432	(G/A)
CakSNP14742	*Kabuli*	*Ca_Kabuli_Scaffold1659*	303945	(T/G)
CakSNP14743	*Kabuli*	*Ca_Kabuli_Scaffold1661*	70434	(A/G)
CakSNP14744	*Kabuli*	*Ca_Kabuli_Scaffold1664*	94989	(G/A)
CakSNP14745	*Kabuli*	*Ca_Kabuli_Scaffold1664*	94984	(A/T)
CakSNP14746	*Kabuli*	*Ca_Kabuli_Scaffold1664*	94930	(G/C)
CakSNP14747	*Kabuli*	*Ca_Kabuli_Scaffold167*	11018	(T/A)
CakSNP14748	*Kabuli*	*Ca_Kabuli_Scaffold167*	11009	(A/T)
CakSNP14749	*Kabuli*	*Ca_Kabuli_Scaffold167*	137869	(C/T)
CakSNP14750	*Kabuli*	*Ca_Kabuli_Scaffold167*	137848	(A/G)
CakSNP14751	*Kabuli*	*Ca_Kabuli_Scaffold167*	239687	(G/A)
CakSNP14752	*Kabuli*	*Ca_Kabuli_Scaffold167*	327829	(T/C)
CakSNP14753	*Kabuli*	*Ca_Kabuli_Scaffold1687*	82935	(A/C)
CakSNP14754	*Kabuli*	*Ca_Kabuli_Scaffold1699*	27556	(A/C)
CakSNP14755	*Kabuli*	*Ca_Kabuli_Scaffold1699*	27607	(T/G)
CakSNP14756	*Kabuli*	*Ca_Kabuli_Scaffold1708*	69664	(A/T)
CakSNP14757	*Kabuli*	*Ca_Kabuli_Scaffold171*	96763	(T/C)
CakSNP14758	*Kabuli*	*Ca_Kabuli_Scaffold171*	183718	(T/C)
CakSNP14759	*Kabuli*	*Ca_Kabuli_Scaffold171*	183720	(C/T)

**SNP IDs**	**Cultivars**	**Chromosomes/scaffolds**	**Physical positions (bp)**	**SNPs**
CakSNP14760	*Kabuli*	*Ca_Kabuli_Scaffold171*	183721	(G/A)
CakSNP14761	*Kabuli*	*Ca_Kabuli_Scaffold171*	183727	(C/T)
CakSNP14762	*Kabuli*	*Ca_Kabuli_Scaffold171*	183759	(T/C)
CakSNP14763	*Kabuli*	*Ca_Kabuli_Scaffold171*	186125	(C/A)
CakSNP14764	*Kabuli*	*Ca_Kabuli_Scaffold171*	208050	(A/G)
CakSNP14765	*Kabuli*	*Ca_Kabuli_Scaffold171*	209964	(G/T)
CakSNP14766	*Kabuli*	*Ca_Kabuli_Scaffold1718*	302	(G/A)
CakSNP14767	*Kabuli*	*Ca_Kabuli_Scaffold1718*	295	(G/A)
CakSNP14768	*Kabuli*	*Ca_Kabuli_Scaffold1718*	289	(C/T)
CakSNP14769	*Kabuli*	*Ca_Kabuli_Scaffold1718*	268	(C/G)
CakSNP14770	*Kabuli*	*Ca_Kabuli_Scaffold1718*	3898	(G/C)
CakSNP14771	*Kabuli*	*Ca_Kabuli_Scaffold1718*	21521	(A/G)
CakSNP14772	*Kabuli*	*Ca_Kabuli_Scaffold1718*	40669	(T/C)
CakSNP14773	*Kabuli*	*Ca_Kabuli_Scaffold1722*	65999	(C/A)
CakSNP14774	*Kabuli*	*Ca_Kabuli_Scaffold1722*	135589	(C/T)
CakSNP14775	*Kabuli*	*Ca_Kabuli_Scaffold1728*	33301	(A/C)
CakSNP14776	*Kabuli*	*Ca_Kabuli_Scaffold174*	17761	(A/C)
CakSNP14777	*Kabuli*	*Ca_Kabuli_Scaffold174*	32366	(T/C)
CakSNP14778	*Kabuli*	*Ca_Kabuli_Scaffold174*	44014	(T/G)
CakSNP14779	*Kabuli*	*Ca_Kabuli_Scaffold174*	61454	(T/C)
CakSNP14780	*Kabuli*	*Ca_Kabuli_Scaffold174*	263899	(G/A)
CakSNP14781	*Kabuli*	*Ca_Kabuli_Scaffold174*	335077	(C/T)
CakSNP14782	*Kabuli*	*Ca_Kabuli_Scaffold174*	341322	(C/T)
CakSNP14783	*Kabuli*	*Ca_Kabuli_Scaffold174*	457627	(G/A)
CakSNP14784	*Kabuli*	*Ca_Kabuli_Scaffold1750*	15510	(C/T)
CakSNP14785	*Kabuli*	*Ca_Kabuli_Scaffold1750*	137106	(A/C)
CakSNP14786	*Kabuli*	*Ca_Kabuli_Scaffold1750*	204638	(G/A)
CakSNP14787	*Kabuli*	*Ca_Kabuli_Scaffold1751*	189367	(T/G)
CakSNP14788	*Kabuli*	*Ca_Kabuli_Scaffold1751*	192417	(C/A)
CakSNP14789	*Kabuli*	*Ca_Kabuli_Scaffold1751*	192713	(G/T)
CakSNP14790	*Kabuli*	*Ca_Kabuli_Scaffold1751*	193026	(T/A)
CakSNP14791	*Kabuli*	*Ca_Kabuli_Scaffold1751*	193013	(T/G)
CakSNP14792	*Kabuli*	*Ca_Kabuli_Scaffold1751*	266583	(G/A)
CakSNP14793	*Kabuli*	*Ca_Kabuli_Scaffold1751*	496626	(G/A)
CakSNP14794	*Kabuli*	*Ca_Kabuli_Scaffold1751*	528896	(A/C)
CakSNP14795	*Kabuli*	*Ca_Kabuli_Scaffold1751*	529005	(C/T)
CakSNP14796	*Kabuli*	*Ca_Kabuli_Scaffold1751*	528979	(T/C)
CakSNP14797	*Kabuli*	*Ca_Kabuli_Scaffold1751*	528973	(C/G)
CakSNP14798	*Kabuli*	*Ca_Kabuli_Scaffold1751*	625364	(G/A)
CakSNP14799	*Kabuli*	*Ca_Kabuli_Scaffold1751*	625362	(C/T)
CakSNP14800	*Kabuli*	*Ca_Kabuli_Scaffold1751*	625355	(C/T)

SNP IDs	Cultivars	Chromosomes/scaffolds	Physical positions (bp)	SNPs
CakSNP14801	*Kabuli*	*Ca_Kabuli_Scaffold1751*	625342	(C/T)
CakSNP14802	*Kabuli*	*Ca_Kabuli_Scaffold1751*	625335	(G/A)
CakSNP14803	*Kabuli*	*Ca_Kabuli_Scaffold1751*	625395	(A/G)
CakSNP14804	*Kabuli*	*Ca_Kabuli_Scaffold1751*	625394	(C/T)
CakSNP14805	*Kabuli*	*Ca_Kabuli_Scaffold1751*	625389	(A/C)
CakSNP14806	*Kabuli*	*Ca_Kabuli_Scaffold1751*	625378	(G/A)
CakSNP14807	*Kabuli*	*Ca_Kabuli_Scaffold1751*	625375	(G/A)
CakSNP14808	*Kabuli*	*Ca_Kabuli_Scaffold1751*	625366	(T/C)
CakSNP14809	*Kabuli*	*Ca_Kabuli_Scaffold1751*	625365	(T/C)
CakSNP14810	*Kabuli*	*Ca_Kabuli_Scaffold1751*	625332	(A/C)
CakSNP14811	*Kabuli*	*Ca_Kabuli_Scaffold1751*	625396	(G/A)
CakSNP14812	*Kabuli*	*Ca_Kabuli_Scaffold1776*	740	(C/T)
CakSNP14813	*Kabuli*	*Ca_Kabuli_Scaffold1777*	164715	(G/A)
CakSNP14814	*Kabuli*	*Ca_Kabuli_Scaffold1777*	164722	(C/G)
CakSNP14815	*Kabuli*	*Ca_Kabuli_Scaffold1777*	164747	(G/A)
CakSNP14816	*Kabuli*	*Ca_Kabuli_Scaffold1777*	164791	(T/G)
CakSNP14817	*Kabuli*	*Ca_Kabuli_Scaffold1777*	164758	(C/T)
CakSNP14818	*Kabuli*	*Ca_Kabuli_Scaffold1777*	164753	(A/G)
CakSNP14819	*Kabuli*	*Ca_Kabuli_Scaffold1777*	164730	(T/G)
CakSNP14820	*Kabuli*	*Ca_Kabuli_Scaffold1777*	169037	(G/T)
CakSNP14821	*Kabuli*	*Ca_Kabuli_Scaffold1777*	343173	(C/T)
CakSNP14822	*Kabuli*	*Ca_Kabuli_Scaffold1777*	343185	(G/A)
CakSNP14823	*Kabuli*	*Ca_Kabuli_Scaffold1777*	448166	(A/G)
CakSNP14824	*Kabuli*	*Ca_Kabuli_Scaffold1777*	448176	(C/T)
CakSNP14825	*Kabuli*	*Ca_Kabuli_Scaffold1777*	448187	(G/T)
CakSNP14826	*Kabuli*	*Ca_Kabuli_Scaffold1777*	448216	(C/T)
CakSNP14827	*Kabuli*	*Ca_Kabuli_Scaffold1779*	957	(G/A)
CakSNP14828	*Kabuli*	*Ca_Kabuli_Scaffold1779*	896	(A/G)
CakSNP14829	*Kabuli*	*Ca_Kabuli_Scaffold1779*	890	(G/A)
CakSNP14830	*Kabuli*	*Ca_Kabuli_Scaffold1802*	17068	(A/G)
CakSNP14831	*Kabuli*	*Ca_Kabuli_Scaffold1802*	17033	(A/G)
CakSNP14832	*Kabuli*	*Ca_Kabuli_Scaffold1802*	17111	(C/T)
CakSNP14833	*Kabuli*	*Ca_Kabuli_Scaffold1802*	17117	(T/C)
CakSNP14834	*Kabuli*	*Ca_Kabuli_Scaffold1802*	17120	(C/T)
CakSNP14835	*Kabuli*	*Ca_Kabuli_Scaffold1802*	17123	(G/T)
CakSNP14836	*Kabuli*	*Ca_Kabuli_Scaffold1802*	17132	(C/T)
CakSNP14837	*Kabuli*	*Ca_Kabuli_Scaffold1802*	17150	(T/C)
CakSNP14838	*Kabuli*	*Ca_Kabuli_Scaffold1802*	17151	(T/C)
CakSNP14839	*Kabuli*	*Ca_Kabuli_Scaffold1802*	17168	(T/C)
CakSNP14840	*Kabuli*	*Ca_Kabuli_Scaffold1802*	17177	(T/A)
CakSNP14841	*Kabuli*	*Ca_Kabuli_Scaffold1835*	9398	(C/A)

SNP IDs	Cultivars	Chromosomes/scaffolds	Physical positions (bp)	SNPs
CakSNP14842	*Kabuli*	*Ca_Kabuli_Scaffold1835*	10541	(C/G)
CakSNP14843	*Kabuli*	*Ca_Kabuli_Scaffold1835*	10563	(T/C)
CakSNP14844	*Kabuli*	*Ca_Kabuli_Scaffold1844*	41899	(C/A)
CakSNP14845	*Kabuli*	*Ca_Kabuli_Scaffold1845*	16292	(C/G)
CakSNP14846	*Kabuli*	*Ca_Kabuli_Scaffold1845*	16317	(C/G)
CakSNP14847	*Kabuli*	*Ca_Kabuli_Scaffold1845*	40886	(T/A)
CakSNP14848	*Kabuli*	*Ca_Kabuli_Scaffold1845*	40872	(T/G)
CakSNP14849	*Kabuli*	*Ca_Kabuli_Scaffold1845*	40871	(T/A)
CakSNP14850	*Kabuli*	*Ca_Kabuli_Scaffold1845*	40842	(T/G)
CakSNP14851	*Kabuli*	*Ca_Kabuli_Scaffold1845*	40821	(T/G)
CakSNP14852	*Kabuli*	*Ca_Kabuli_Scaffold1848*	583	(A/G)
CakSNP14853	*Kabuli*	*Ca_Kabuli_Scaffold1848*	565	(T/C)
CakSNP14854	*Kabuli*	*Ca_Kabuli_Scaffold1848*	562	(G/C)
CakSNP14855	*Kabuli*	*Ca_Kabuli_Scaffold1848*	532	(G/C)
CakSNP14856	*Kabuli*	*Ca_Kabuli_Scaffold186*	11965	(A/G)
CakSNP14857	*Kabuli*	*Ca_Kabuli_Scaffold186*	279942	(A/G)
CakSNP14858	*Kabuli*	*Ca_Kabuli_Scaffold186*	280062	(T/C)
CakSNP14859	*Kabuli*	*Ca_Kabuli_Scaffold186*	280047	(A/G)
CakSNP14860	*Kabuli*	*Ca_Kabuli_Scaffold186*	280023	(C/T)
CakSNP14861	*Kabuli*	*Ca_Kabuli_Scaffold186*	280015	(T/C)
CakSNP14862	*Kabuli*	*Ca_Kabuli_Scaffold186*	280009	(G/T)
CakSNP14863	*Kabuli*	*Ca_Kabuli_Scaffold1866*	106318	(T/A)
CakSNP14864	*Kabuli*	*Ca_Kabuli_Scaffold1866*	106330	(A/T)
CakSNP14865	*Kabuli*	*Ca_Kabuli_Scaffold1866*	106371	(C/A)
CakSNP14866	*Kabuli*	*Ca_Kabuli_Scaffold1866*	106374	(A/T)
CakSNP14867	*Kabuli*	*Ca_Kabuli_Scaffold1866*	106402	(C/T)
CakSNP14868	*Kabuli*	*Ca_Kabuli_Scaffold1866*	110550	(A/G)
CakSNP14869	*Kabuli*	*Ca_Kabuli_Scaffold1866*	110570	(G/A)
CakSNP14870	*Kabuli*	*Ca_Kabuli_Scaffold1866*	110578	(C/A)
CakSNP14871	*Kabuli*	*Ca_Kabuli_Scaffold1899*	148	(T/G)
CakSNP14872	*Kabuli*	*Ca_Kabuli_Scaffold1899*	187	(A/G)
CakSNP14873	*Kabuli*	*Ca_Kabuli_Scaffold1899*	267	(T/A)
CakSNP14874	*Kabuli*	*Ca_Kabuli_Scaffold1899*	200	(G/A)
CakSNP14875	*Kabuli*	*Ca_Kabuli_Scaffold1918*	216231	(T/G)
CakSNP14876	*Kabuli*	*Ca_Kabuli_Scaffold1918*	216213	(T/C)
CakSNP14877	*Kabuli*	*Ca_Kabuli_Scaffold1918*	216180	(C/A)
CakSNP14878	*Kabuli*	*Ca_Kabuli_Scaffold1918*	237787	(G/A)
CakSNP14879	*Kabuli*	*Ca_Kabuli_Scaffold1918*	245929	(T/C)
CakSNP14880	*Kabuli*	*Ca_Kabuli_Scaffold1928*	103092	(A/G)
CakSNP14881	*Kabuli*	*Ca_Kabuli_Scaffold1928*	103114	(G/A)
CakSNP14882	*Kabuli*	*Ca_Kabuli_Scaffold193*	38279	(C/A)

**SNP IDs**	**Cultivars**	**Chromosomes/scaffolds**	**Physical positions (bp)**	**SNPs**
CakSNP14883	*Kabuli*	*Ca_Kabuli_Scaffold193*	186538	(C/T)
CakSNP14884	*Kabuli*	*Ca_Kabuli_Scaffold193*	186523	(T/C)
CakSNP14885	*Kabuli*	*Ca_Kabuli_Scaffold193*	186515	(T/G)
CakSNP14886	*Kabuli*	*Ca_Kabuli_Scaffold193*	186510	(C/A)
CakSNP14887	*Kabuli*	*Ca_Kabuli_Scaffold193*	186584	(A/G)
CakSNP14888	*Kabuli*	*Ca_Kabuli_Scaffold193*	186582	(C/A)
CakSNP14889	*Kabuli*	*Ca_Kabuli_Scaffold193*	186587	(G/T)
CakSNP14890	*Kabuli*	*Ca_Kabuli_Scaffold193*	186588	(C/G)
CakSNP14891	*Kabuli*	*Ca_Kabuli_Scaffold193*	186595	(C/T)
CakSNP14892	*Kabuli*	*Ca_Kabuli_Scaffold193*	186598	(A/G)
CakSNP14893	*Kabuli*	*Ca_Kabuli_Scaffold193*	186611	(G/A)
CakSNP14894	*Kabuli*	*Ca_Kabuli_Scaffold193*	186639	(C/T)
CakSNP14895	*Kabuli*	*Ca_Kabuli_Scaffold193*	186651	(T/C)
CakSNP14896	*Kabuli*	*Ca_Kabuli_Scaffold193*	186657	(G/A)
CakSNP14897	*Kabuli*	*Ca_Kabuli_Scaffold193*	186661	(T/C)
CakSNP14898	*Kabuli*	*Ca_Kabuli_Scaffold193*	242763	(T/C)
CakSNP14899	*Kabuli*	*Ca_Kabuli_Scaffold193*	242733	(G/T)
CakSNP14900	*Kabuli*	*Ca_Kabuli_Scaffold193*	244735	(T/G)
CakSNP14901	*Kabuli*	*Ca_Kabuli_Scaffold1943*	163784	(G/A)
CakSNP14902	*Kabuli*	*Ca_Kabuli_Scaffold1943*	163780	(C/T)
CakSNP14903	*Kabuli*	*Ca_Kabuli_Scaffold1959*	84160	(G/A)
CakSNP14904	*Kabuli*	*Ca_Kabuli_Scaffold1964*	54613	(G/A)
CakSNP14905	*Kabuli*	*Ca_Kabuli_Scaffold1964*	54604	(T/C)
CakSNP14906	*Kabuli*	*Ca_Kabuli_Scaffold1964*	67416	(G/C)
CakSNP14907	*Kabuli*	*Ca_Kabuli_Scaffold198*	631160	(G/A)
CakSNP14908	*Kabuli*	*Ca_Kabuli_Scaffold198*	631174	(C/T)
CakSNP14909	*Kabuli*	*Ca_Kabuli_Scaffold198*	631185	(G/A)
CakSNP14910	*Kabuli*	*Ca_Kabuli_Scaffold198*	641266	(C/T)
CakSNP14911	*Kabuli*	*Ca_Kabuli_Scaffold198*	641252	(A/G)
CakSNP14912	*Kabuli*	*Ca_Kabuli_Scaffold198*	641243	(A/G)
CakSNP14913	*Kabuli*	*Ca_Kabuli_Scaffold1981*	265110	(A/C)
CakSNP14914	*Kabuli*	*Ca_Kabuli_Scaffold1981*	265126	(G/T)
CakSNP14915	*Kabuli*	*Ca_Kabuli_Scaffold1981*	265216	(A/G)
CakSNP14916	*Kabuli*	*Ca_Kabuli_Scaffold1981*	265191	(T/G)
CakSNP14917	*Kabuli*	*Ca_Kabuli_Scaffold1981*	269879	(A/G)
CakSNP14918	*Kabuli*	*Ca_Kabuli_Scaffold1981*	385683	(T/A)
CakSNP14919	*Kabuli*	*Ca_Kabuli_Scaffold1984*	212011	(G/A)
CakSNP14920	*Kabuli*	*Ca_Kabuli_Scaffold1984*	215969	(G/C)
CakSNP14921	*Kabuli*	*Ca_Kabuli_Scaffold1984*	292507	(A/C)
CakSNP14922	*Kabuli*	*Ca_Kabuli_Scaffold1984*	292511	(G/C)
CakSNP14923	*Kabuli*	*Ca_Kabuli_Scaffold1985*	98611	(C/T)

SNP IDs	Cultivars	Chromosomes/scaffolds	Physical positions (bp)	SNPs
CakSNP14924	*Kabuli*	*Ca_Kabuli_Scaffold1985*	98659	(C/T)
CakSNP14925	*Kabuli*	*Ca_Kabuli_Scaffold1991*	62009	(C/A)
CakSNP14926	*Kabuli*	*Ca_Kabuli_Scaffold1991*	62121	(G/A)
CakSNP14927	*Kabuli*	*Ca_Kabuli_Scaffold1991*	62085	(A/C)
CakSNP14928	*Kabuli*	*Ca_Kabuli_Scaffold1991*	62115	(G/A)
CakSNP14929	*Kabuli*	*Ca_Kabuli_Scaffold1991*	62131	(G/A)
CakSNP14930	*Kabuli*	*Ca_Kabuli_Scaffold1991*	62154	(T/A)
CakSNP14931	*Kabuli*	*Ca_Kabuli_Scaffold1991*	62147	(G/A)
CakSNP14932	*Kabuli*	*Ca_Kabuli_Scaffold1991*	62422	(G/A)
CakSNP14933	*Kabuli*	*Ca_Kabuli_Scaffold2*	137309	(T/G)
CakSNP14934	*Kabuli*	*Ca_Kabuli_Scaffold2*	504258	(C/T)
CakSNP14935	*Kabuli*	*Ca_Kabuli_Scaffold2*	504253	(C/T)
CakSNP14936	*Kabuli*	*Ca_Kabuli_Scaffold2*	504248	(G/A)
CakSNP14937	*Kabuli*	*Ca_Kabuli_Scaffold202*	16084	(T/C)
CakSNP14938	*Kabuli*	*Ca_Kabuli_Scaffold202*	92532	(C/A)
CakSNP14939	*Kabuli*	*Ca_Kabuli_Scaffold202*	96456	(T/C)
CakSNP14940	*Kabuli*	*Ca_Kabuli_Scaffold202*	96444	(G/A)
CakSNP14941	*Kabuli*	*Ca_Kabuli_Scaffold202*	96532	(G/T)
CakSNP14942	*Kabuli*	*Ca_Kabuli_Scaffold202*	96589	(A/T)
CakSNP14943	*Kabuli*	*Ca_Kabuli_Scaffold202*	96580	(T/G)
CakSNP14944	*Kabuli*	*Ca_Kabuli_Scaffold202*	96576	(A/G)
CakSNP14945	*Kabuli*	*Ca_Kabuli_Scaffold202*	99123	(C/T)
CakSNP14946	*Kabuli*	*Ca_Kabuli_Scaffold2027*	100867	(G/C)
CakSNP14947	*Kabuli*	*Ca_Kabuli_Scaffold2027*	152763	(C/A)
CakSNP14948	*Kabuli*	*Ca_Kabuli_Scaffold2030*	13855	(G/A)
CakSNP14949	*Kabuli*	*Ca_Kabuli_Scaffold2030*	13822	(C/T)
CakSNP14950	*Kabuli*	*Ca_Kabuli_Scaffold2030*	13807	(G/A)
CakSNP14951	*Kabuli*	*Ca_Kabuli_Scaffold2030*	13889	(C/T)
CakSNP14952	*Kabuli*	*Ca_Kabuli_Scaffold2030*	13874	(G/T)
CakSNP14953	*Kabuli*	*Ca_Kabuli_Scaffold2033*	115044	(C/T)
CakSNP14954	*Kabuli*	*Ca_Kabuli_Scaffold2033*	118686	(C/T)
CakSNP14955	*Kabuli*	*Ca_Kabuli_Scaffold2033*	118779	(G/T)
CakSNP14956	*Kabuli*	*Ca_Kabuli_Scaffold2033*	118760	(T/G)
CakSNP14957	*Kabuli*	*Ca_Kabuli_Scaffold2033*	119057	(T/G)
CakSNP14958	*Kabuli*	*Ca_Kabuli_Scaffold2033*	119166	(T/C)
CakSNP14959	*Kabuli*	*Ca_Kabuli_Scaffold2033*	119645	(G/A)
CakSNP14960	*Kabuli*	*Ca_Kabuli_Scaffold2033*	119648	(G/A)
CakSNP14961	*Kabuli*	*Ca_Kabuli_Scaffold2033*	119703	(C/T)
CakSNP14962	*Kabuli*	*Ca_Kabuli_Scaffold2033*	119715	(G/T)
CakSNP14963	*Kabuli*	*Ca_Kabuli_Scaffold2033*	119728	(G/A)
CakSNP14964	*Kabuli*	*Ca_Kabuli_Scaffold2039*	116734	(A/G)

SNP IDs	Cultivars	Chromosomes/scaffolds	Physical positions (bp)	SNPs
CakSNP14965	*Kabuli*	*Ca_Kabuli_Scaffold2040*	139597	(A/G)
CakSNP14966	*Kabuli*	*Ca_Kabuli_Scaffold2040*	139585	(A/C)
CakSNP14967	*Kabuli*	*Ca_Kabuli_Scaffold2040*	139584	(G/A)
CakSNP14968	*Kabuli*	*Ca_Kabuli_Scaffold2040*	139581	(G/A)
CakSNP14969	*Kabuli*	*Ca_Kabuli_Scaffold2040*	139560	(G/T)
CakSNP14970	*Kabuli*	*Ca_Kabuli_Scaffold2040*	139550	(G/A)
CakSNP14971	*Kabuli*	*Ca_Kabuli_Scaffold2040*	139548	(C/T)
CakSNP14972	*Kabuli*	*Ca_Kabuli_Scaffold2040*	139544	(C/A)
CakSNP14973	*Kabuli*	*Ca_Kabuli_Scaffold2040*	139541	(T/C)
CakSNP14974	*Kabuli*	*Ca_Kabuli_Scaffold2040*	139538	(G/A)
CakSNP14975	*Kabuli*	*Ca_Kabuli_Scaffold2040*	139545	(G/A)
CakSNP14976	*Kabuli*	*Ca_Kabuli_Scaffold2040*	139603	(C/T)
CakSNP14977	*Kabuli*	*Ca_Kabuli_Scaffold205*	74448	(T/G)
CakSNP14978	*Kabuli*	*Ca_Kabuli_Scaffold205*	74430	(A/G)
CakSNP14979	*Kabuli*	*Ca_Kabuli_Scaffold205*	74397	(G/A)
CakSNP14980	*Kabuli*	*Ca_Kabuli_Scaffold205*	74399	(C/T)
CakSNP14981	*Kabuli*	*Ca_Kabuli_Scaffold205*	74424	(T/C)
CakSNP14982	*Kabuli*	*Ca_Kabuli_Scaffold205*	74432	(G/A)
CakSNP14983	*Kabuli*	*Ca_Kabuli_Scaffold205*	74433	(G/A)
CakSNP14984	*Kabuli*	*Ca_Kabuli_Scaffold206*	58349	(C/A)
CakSNP14985	*Kabuli*	*Ca_Kabuli_Scaffold206*	62985	(G/A)
CakSNP14986	*Kabuli*	*Ca_Kabuli_Scaffold208*	344503	(G/T)
CakSNP14987	*Kabuli*	*Ca_Kabuli_Scaffold208*	344515	(A/G)
CakSNP14988	*Kabuli*	*Ca_Kabuli_Scaffold208*	344519	(A/T)
CakSNP14989	*Kabuli*	*Ca_Kabuli_Scaffold208*	344533	(C/T)
CakSNP14990	*Kabuli*	*Ca_Kabuli_Scaffold208*	344556	(T/G)
CakSNP14991	*Kabuli*	*Ca_Kabuli_Scaffold2097*	54210	(A/C)
CakSNP14992	*Kabuli*	*Ca_Kabuli_Scaffold210*	293799	(G/A)
CakSNP14993	*Kabuli*	*Ca_Kabuli_Scaffold210*	355284	(G/T)
CakSNP14994	*Kabuli*	*Ca_Kabuli_Scaffold210*	533483	(G/A)
CakSNP14995	*Kabuli*	*Ca_Kabuli_Scaffold210*	533416	(G/A)
CakSNP14996	*Kabuli*	*Ca_Kabuli_Scaffold210*	617431	(T/A)
CakSNP14997	*Kabuli*	*Ca_Kabuli_Scaffold2104*	61757	(T/C)
CakSNP14998	*Kabuli*	*Ca_Kabuli_Scaffold2104*	61745	(T/G)
CakSNP14999	*Kabuli*	*Ca_Kabuli_Scaffold2104*	61734	(T/G)
CakSNP15000	*Kabuli*	*Ca_Kabuli_Scaffold2104*	61723	(G/A)
CakSNP15001	*Kabuli*	*Ca_Kabuli_Scaffold2104*	61710	(C/T)
CakSNP15002	*Kabuli*	*Ca_Kabuli_Scaffold2104*	61705	(C/T)
CakSNP15003	*Kabuli*	*Ca_Kabuli_Scaffold2152*	372404	(A/T)
CakSNP15004	*Kabuli*	*Ca_Kabuli_Scaffold2166*	228653	(G/C)
CakSNP15005	*Kabuli*	*Ca_Kabuli_Scaffold2166*	228710	(G/A)

**SNP IDs**	**Cultivars**	**Chromosomes/scaffolds**	**Physical positions (bp)**	**SNPs**
CakSNP15006	*Kabuli*	*Ca_Kabuli_Scaffold2170*	4821	(G/A)
CakSNP15007	*Kabuli*	*Ca_Kabuli_Scaffold2192*	67485	(A/G)
CakSNP15008	*Kabuli*	*Ca_Kabuli_Scaffold2192*	67589	(A/C)
CakSNP15009	*Kabuli*	*Ca_Kabuli_Scaffold221*	53841	(T/G)
CakSNP15010	*Kabuli*	*Ca_Kabuli_Scaffold2248*	5538	(A/T)
CakSNP15011	*Kabuli*	*Ca_Kabuli_Scaffold2248*	5562	(C/T)
CakSNP15012	*Kabuli*	*Ca_Kabuli_Scaffold2248*	5570	(T/C)
CakSNP15013	*Kabuli*	*Ca_Kabuli_Scaffold2248*	5520	(C/A)
CakSNP15014	*Kabuli*	*Ca_Kabuli_Scaffold2249*	4446	(A/C)
CakSNP15015	*Kabuli*	*Ca_Kabuli_Scaffold2269*	58091	(A/T)
CakSNP15016	*Kabuli*	*Ca_Kabuli_Scaffold2269*	123799	(A/C)
CakSNP15017	*Kabuli*	*Ca_Kabuli_Scaffold2269*	126013	(A/C)
CakSNP15018	*Kabuli*	*Ca_Kabuli_Scaffold2301*	70721	(A/G)
CakSNP15019	*Kabuli*	*Ca_Kabuli_Scaffold2330*	27894	(A/C)
CakSNP15020	*Kabuli*	*Ca_Kabuli_Scaffold2330*	64157	(T/C)
CakSNP15021	*Kabuli*	*Ca_Kabuli_Scaffold2330*	65482	(T/G)
CakSNP15022	*Kabuli*	*Ca_Kabuli_Scaffold2330*	65617	(G/A)
CakSNP15023	*Kabuli*	*Ca_Kabuli_Scaffold2330*	65650	(C/T)
CakSNP15024	*Kabuli*	*Ca_Kabuli_Scaffold2330*	83061	(C/T)
CakSNP15025	*Kabuli*	*Ca_Kabuli_Scaffold2330*	83064	(A/G)
CakSNP15026	*Kabuli*	*Ca_Kabuli_Scaffold2330*	83077	(G/C)
CakSNP15027	*Kabuli*	*Ca_Kabuli_Scaffold2330*	83112	(A/G)
CakSNP15028	*Kabuli*	*Ca_Kabuli_Scaffold2330*	83116	(G/T)
CakSNP15029	*Kabuli*	*Ca_Kabuli_Scaffold235*	67258	(G/T)
CakSNP15030	*Kabuli*	*Ca_Kabuli_Scaffold2371*	139149	(A/G)
CakSNP15031	*Kabuli*	*Ca_Kabuli_Scaffold2373*	3818	(A/G)
CakSNP15032	*Kabuli*	*Ca_Kabuli_Scaffold2373*	3815	(T/A)
CakSNP15033	*Kabuli*	*Ca_Kabuli_Scaffold2373*	3811	(C/T)
CakSNP15034	*Kabuli*	*Ca_Kabuli_Scaffold2373*	3793	(C/T)
CakSNP15035	*Kabuli*	*Ca_Kabuli_Scaffold2373*	3923	(T/C)
CakSNP15036	*Kabuli*	*Ca_Kabuli_Scaffold2373*	3871	(A/T)
CakSNP15037	*Kabuli*	*Ca_Kabuli_Scaffold2373*	3862	(C/T)
CakSNP15038	*Kabuli*	*Ca_Kabuli_Scaffold2373*	12916	(C/A)
CakSNP15039	*Kabuli*	*Ca_Kabuli_Scaffold2373*	12969	(G/C)
CakSNP15040	*Kabuli*	*Ca_Kabuli_Scaffold2373*	12978	(A/T)
CakSNP15041	*Kabuli*	*Ca_Kabuli_Scaffold2373*	62495	(C/T)
CakSNP15042	*Kabuli*	*Ca_Kabuli_Scaffold2373*	62494	(A/G)
CakSNP15043	*Kabuli*	*Ca_Kabuli_Scaffold2373*	62473	(G/A)
CakSNP15044	*Kabuli*	*Ca_Kabuli_Scaffold2373*	62434	(G/A)
CakSNP15045	*Kabuli*	*Ca_Kabuli_Scaffold2373*	62433	(A/G)
CakSNP15046	*Kabuli*	*Ca_Kabuli_Scaffold2373*	127884	(A/C)

SNP IDs	Cultivars	Chromosomes/scaffolds	Physical positions (bp)	SNPs
CakSNP15047	*Kabuli*	*Ca_Kabuli_Scaffold2373*	127851	(C/T)
CakSNP15048	*Kabuli*	*Ca_Kabuli_Scaffold2373*	127905	(C/T)
CakSNP15049	*Kabuli*	*Ca_Kabuli_Scaffold2373*	127870	(T/G)
CakSNP15050	*Kabuli*	*Ca_Kabuli_Scaffold2373*	127855	(A/C)
CakSNP15051	*Kabuli*	*Ca_Kabuli_Scaffold2392*	35402	(A/C)
CakSNP15052	*Kabuli*	*Ca_Kabuli_Scaffold2392*	198120	(G/T)
CakSNP15053	*Kabuli*	*Ca_Kabuli_Scaffold2392*	314304	(T/C)
CakSNP15054	*Kabuli*	*Ca_Kabuli_Scaffold2392*	314322	(T/C)
CakSNP15055	*Kabuli*	*Ca_Kabuli_Scaffold2392*	314323	(G/A)
CakSNP15056	*Kabuli*	*Ca_Kabuli_Scaffold2392*	314343	(G/A)
CakSNP15057	*Kabuli*	*Ca_Kabuli_Scaffold2392*	314344	(T/C)
CakSNP15058	*Kabuli*	*Ca_Kabuli_Scaffold2392*	314349	(C/G)
CakSNP15059	*Kabuli*	*Ca_Kabuli_Scaffold2392*	314353	(G/A)
CakSNP15060	*Kabuli*	*Ca_Kabuli_Scaffold2392*	314345	(G/A)
CakSNP15061	*Kabuli*	*Ca_Kabuli_Scaffold2392*	314401	(A/C)
CakSNP15062	*Kabuli*	*Ca_Kabuli_Scaffold240*	6231	(T/A)
CakSNP15063	*Kabuli*	*Ca_Kabuli_Scaffold240*	6193	(G/A)
CakSNP15064	*Kabuli*	*Ca_Kabuli_Scaffold240*	6152	(C/T)
CakSNP15065	*Kabuli*	*Ca_Kabuli_Scaffold240*	119157	(G/A)
CakSNP15066	*Kabuli*	*Ca_Kabuli_Scaffold240*	119301	(T/C)
CakSNP15067	*Kabuli*	*Ca_Kabuli_Scaffold240*	119297	(C/A)
CakSNP15068	*Kabuli*	*Ca_Kabuli_Scaffold240*	119278	(T/C)
CakSNP15069	*Kabuli*	*Ca_Kabuli_Scaffold240*	119269	(C/T)
CakSNP15070	*Kabuli*	*Ca_Kabuli_Scaffold240*	119267	(G/C)
CakSNP15071	*Kabuli*	*Ca_Kabuli_Scaffold240*	119265	(C/G)
CakSNP15072	*Kabuli*	*Ca_Kabuli_Scaffold2415*	117840	(A/G)
CakSNP15073	*Kabuli*	*Ca_Kabuli_Scaffold2415*	117871	(A/C)
CakSNP15074	*Kabuli*	*Ca_Kabuli_Scaffold2415*	117882	(A/G)
CakSNP15075	*Kabuli*	*Ca_Kabuli_Scaffold2415*	117970	(G/A)
CakSNP15076	*Kabuli*	*Ca_Kabuli_Scaffold242*	293872	(A/G)
CakSNP15077	*Kabuli*	*Ca_Kabuli_Scaffold250*	38270	(A/G)
CakSNP15078	*Kabuli*	*Ca_Kabuli_Scaffold2501*	287	(C/T)
CakSNP15079	*Kabuli*	*Ca_Kabuli_Scaffold2501*	282	(C/A)
CakSNP15080	*Kabuli*	*Ca_Kabuli_Scaffold2501*	251	(T/G)
CakSNP15081	*Kabuli*	*Ca_Kabuli_Scaffold2501*	242	(C/G)
CakSNP15082	*Kabuli*	*Ca_Kabuli_Scaffold2516*	58947	(T/C)
CakSNP15083	*Kabuli*	*Ca_Kabuli_Scaffold2521*	57762	(T/C)
CakSNP15084	*Kabuli*	*Ca_Kabuli_Scaffold2521*	57725	(G/A)
CakSNP15085	*Kabuli*	*Ca_Kabuli_Scaffold2521*	57709	(G/T)
CakSNP15086	*Kabuli*	*Ca_Kabuli_Scaffold2557*	110378	(C/T)
CakSNP15087	*Kabuli*	*Ca_Kabuli_Scaffold2557*	110414	(T/G)

SNP IDs	Cultivars	Chromosomes/scaffolds	Physical positions (bp)	SNPs
CakSNP15088	*Kabuli*	*Ca_Kabuli_Scaffold2557*	110437	(C/T)
CakSNP15089	*Kabuli*	*Ca_Kabuli_Scaffold2557*	110441	(T/A)
CakSNP15090	*Kabuli*	*Ca_Kabuli_Scaffold2569*	58152	(T/G)
CakSNP15091	*Kabuli*	*Ca_Kabuli_Scaffold2575*	101222	(C/T)
CakSNP15092	*Kabuli*	*Ca_Kabuli_Scaffold2617*	467848	(C/T)
CakSNP15093	*Kabuli*	*Ca_Kabuli_Scaffold263*	85006	(G/A)
CakSNP15094	*Kabuli*	*Ca_Kabuli_Scaffold263*	85003	(C/T)
CakSNP15095	*Kabuli*	*Ca_Kabuli_Scaffold263*	84998	(G/A)
CakSNP15096	*Kabuli*	*Ca_Kabuli_Scaffold263*	84976	(A/G)
CakSNP15097	*Kabuli*	*Ca_Kabuli_Scaffold263*	84970	(T/C)
CakSNP15098	*Kabuli*	*Ca_Kabuli_Scaffold263*	84952	(T/G)
CakSNP15099	*Kabuli*	*Ca_Kabuli_Scaffold2720*	30699	(C/T)
CakSNP15100	*Kabuli*	*Ca_Kabuli_Scaffold2720*	30701	(G/A)
CakSNP15101	*Kabuli*	*Ca_Kabuli_Scaffold2720*	30760	(T/C)
CakSNP15102	*Kabuli*	*Ca_Kabuli_Scaffold2720*	177550	(G/T)
CakSNP15103	*Kabuli*	*Ca_Kabuli_Scaffold2720*	177578	(T/C)
CakSNP15104	*Kabuli*	*Ca_Kabuli_Scaffold2722*	52416	(G/T)
CakSNP15105	*Kabuli*	*Ca_Kabuli_Scaffold2722*	52397	(G/T)
CakSNP15106	*Kabuli*	*Ca_Kabuli_Scaffold2722*	67309	(C/A)
CakSNP15107	*Kabuli*	*Ca_Kabuli_Scaffold2722*	75609	(A/G)
CakSNP15108	*Kabuli*	*Ca_Kabuli_Scaffold2728*	1371	(G/A)
CakSNP15109	*Kabuli*	*Ca_Kabuli_Scaffold273*	202514	(T/G)
CakSNP15110	*Kabuli*	*Ca_Kabuli_Scaffold2738*	1307	(G/A)
CakSNP15111	*Kabuli*	*Ca_Kabuli_Scaffold275*	208095	(G/A)
CakSNP15112	*Kabuli*	*Ca_Kabuli_Scaffold275*	208067	(C/A)
CakSNP15113	*Kabuli*	*Ca_Kabuli_Scaffold275*	208786	(G/C)
CakSNP15114	*Kabuli*	*Ca_Kabuli_Scaffold275*	230606	(A/G)
CakSNP15115	*Kabuli*	*Ca_Kabuli_Scaffold275*	347422	(C/A)
CakSNP15116	*Kabuli*	*Ca_Kabuli_Scaffold275*	347384	(G/A)
CakSNP15117	*Kabuli*	*Ca_Kabuli_Scaffold275*	347361	(C/T)
CakSNP15118	*Kabuli*	*Ca_Kabuli_Scaffold275*	479497	(C/T)
CakSNP15119	*Kabuli*	*Ca_Kabuli_Scaffold275*	562610	(C/T)
CakSNP15120	*Kabuli*	*Ca_Kabuli_Scaffold2763*	4688	(A/C)
CakSNP15121	*Kabuli*	*Ca_Kabuli_Scaffold2763*	4706	(G/T)
CakSNP15122	*Kabuli*	*Ca_Kabuli_Scaffold2763*	4719	(G/A)
CakSNP15123	*Kabuli*	*Ca_Kabuli_Scaffold2763*	4732	(C/G)
CakSNP15124	*Kabuli*	*Ca_Kabuli_Scaffold2763*	4738	(C/G)
CakSNP15125	*Kabuli*	*Ca_Kabuli_Scaffold2763*	4804	(A/T)
CakSNP15126	*Kabuli*	*Ca_Kabuli_Scaffold2763*	4800	(T/A)
CakSNP15127	*Kabuli*	*Ca_Kabuli_Scaffold2763*	4787	(A/G)
CakSNP15128	*Kabuli*	*Ca_Kabuli_Scaffold2763*	4782	(C/T)

SNP IDs	Cultivars	Chromosomes/scaffolds	Physical positions (bp)	SNPs
CakSNP15129	*Kabuli*	*Ca_Kabuli_Scaffold2763*	25756	(T/G)
CakSNP15130	*Kabuli*	*Ca_Kabuli_Scaffold2763*	25769	(A/G)
CakSNP15131	*Kabuli*	*Ca_Kabuli_Scaffold2763*	25771	(T/G)
CakSNP15132	*Kabuli*	*Ca_Kabuli_Scaffold2763*	43207	(G/A)
CakSNP15133	*Kabuli*	*Ca_Kabuli_Scaffold2763*	49533	(T/A)
CakSNP15134	*Kabuli*	*Ca_Kabuli_Scaffold2763*	82193	(C/A)
CakSNP15135	*Kabuli*	*Ca_Kabuli_Scaffold2763*	82232	(C/T)
CakSNP15136	*Kabuli*	*Ca_Kabuli_Scaffold2763*	82249	(G/A)
CakSNP15137	*Kabuli*	*Ca_Kabuli_Scaffold2763*	82250	(T/C)
CakSNP15138	*Kabuli*	*Ca_Kabuli_Scaffold2763*	82253	(G/C)
CakSNP15139	*Kabuli*	*Ca_Kabuli_Scaffold2763*	82262	(G/C)
CakSNP15140	*Kabuli*	*Ca_Kabuli_Scaffold2763*	82261	(C/G)
CakSNP15141	*Kabuli*	*Ca_Kabuli_Scaffold2763*	82265	(G/T)
CakSNP15142	*Kabuli*	*Ca_Kabuli_Scaffold2763*	82302	(T/A)
CakSNP15143	*Kabuli*	*Ca_Kabuli_Scaffold2763*	82319	(T/C)
CakSNP15144	*Kabuli*	*Ca_Kabuli_Scaffold2763*	82328	(A/G)
CakSNP15145	*Kabuli*	*Ca_Kabuli_Scaffold2763*	82342	(C/T)
CakSNP15146	*Kabuli*	*Ca_Kabuli_Scaffold2763*	82369	(G/A)
CakSNP15147	*Kabuli*	*Ca_Kabuli_Scaffold2763*	82417	(C/T)
CakSNP15148	*Kabuli*	*Ca_Kabuli_Scaffold2763*	82397	(T/C)
CakSNP15149	*Kabuli*	*Ca_Kabuli_Scaffold2763*	95823	(G/A)
CakSNP15150	*Kabuli*	*Ca_Kabuli_Scaffold2763*	99364	(A/G)
CakSNP15151	*Kabuli*	*Ca_Kabuli_Scaffold2763*	99368	(C/G)
CakSNP15152	*Kabuli*	*Ca_Kabuli_Scaffold2763*	99549	(C/A)
CakSNP15153	*Kabuli*	*Ca_Kabuli_Scaffold2763*	104549	(G/C)
CakSNP15154	*Kabuli*	*Ca_Kabuli_Scaffold2763*	104583	(T/C)
CakSNP15155	*Kabuli*	*Ca_Kabuli_Scaffold2763*	107087	(A/G)
CakSNP15156	*Kabuli*	*Ca_Kabuli_Scaffold2763*	107033	(T/A)
CakSNP15157	*Kabuli*	*Ca_Kabuli_Scaffold2763*	107314	(A/G)
CakSNP15158	*Kabuli*	*Ca_Kabuli_Scaffold2763*	107437	(T/C)
CakSNP15159	*Kabuli*	*Ca_Kabuli_Scaffold2763*	107376	(C/T)
CakSNP15160	*Kabuli*	*Ca_Kabuli_Scaffold2763*	107413	(A/G)
CakSNP15161	*Kabuli*	*Ca_Kabuli_Scaffold2763*	108560	(T/G)
CakSNP15162	*Kabuli*	*Ca_Kabuli_Scaffold2763*	108599	(T/A)
CakSNP15163	*Kabuli*	*Ca_Kabuli_Scaffold2763*	108763	(T/G)
CakSNP15164	*Kabuli*	*Ca_Kabuli_Scaffold2763*	117440	(C/T)
CakSNP15165	*Kabuli*	*Ca_Kabuli_Scaffold2763*	117430	(A/C)
CakSNP15166	*Kabuli*	*Ca_Kabuli_Scaffold2763*	125716	(C/T)
CakSNP15167	*Kabuli*	*Ca_Kabuli_Scaffold280*	90875	(G/A)
CakSNP15168	*Kabuli*	*Ca_Kabuli_Scaffold280*	90950	(C/A)
CakSNP15169	*Kabuli*	*Ca_Kabuli_Scaffold280*	99357	(A/C)

SNP IDs	Cultivars	Chromosomes/scaffolds	Physical positions (bp)	SNPs
CakSNP15170	*Kabuli*	*Ca_Kabuli_Scaffold280*	101752	(G/C)
CakSNP15171	*Kabuli*	*Ca_Kabuli_Scaffold280*	111160	(C/A)
CakSNP15172	*Kabuli*	*Ca_Kabuli_Scaffold2812*	56649	(C/T)
CakSNP15173	*Kabuli*	*Ca_Kabuli_Scaffold2812*	56658	(G/A)
CakSNP15174	*Kabuli*	*Ca_Kabuli_Scaffold2812*	56672	(C/G)
CakSNP15175	*Kabuli*	*Ca_Kabuli_Scaffold2812*	56702	(G/A)
CakSNP15176	*Kabuli*	*Ca_Kabuli_Scaffold2812*	56714	(G/C)
CakSNP15177	*Kabuli*	*Ca_Kabuli_Scaffold2812*	56648	(C/G)
CakSNP15178	*Kabuli*	*Ca_Kabuli_Scaffold2812*	89844	(A/C)
CakSNP15179	*Kabuli*	*Ca_Kabuli_Scaffold2812*	89940	(C/T)
CakSNP15180	*Kabuli*	*Ca_Kabuli_Scaffold2812*	89888	(T/A)
CakSNP15181	*Kabuli*	*Ca_Kabuli_Scaffold2812*	89887	(A/T)
CakSNP15182	*Kabuli*	*Ca_Kabuli_Scaffold2815*	25626	(C/G)
CakSNP15183	*Kabuli*	*Ca_Kabuli_Scaffold2815*	25655	(T/G)
CakSNP15184	*Kabuli*	*Ca_Kabuli_Scaffold2815*	25668	(T/G)
CakSNP15185	*Kabuli*	*Ca_Kabuli_Scaffold2827*	171	(C/T)
CakSNP15186	*Kabuli*	*Ca_Kabuli_Scaffold2827*	172	(G/A)
CakSNP15187	*Kabuli*	*Ca_Kabuli_Scaffold2827*	179	(T/G)
CakSNP15188	*Kabuli*	*Ca_Kabuli_Scaffold2827*	210	(A/G)
CakSNP15189	*Kabuli*	*Ca_Kabuli_Scaffold2827*	285	(T/G)
CakSNP15190	*Kabuli*	*Ca_Kabuli_Scaffold2827*	258	(T/C)
CakSNP15191	*Kabuli*	*Ca_Kabuli_Scaffold2827*	240	(T/G)
CakSNP15192	*Kabuli*	*Ca_Kabuli_Scaffold2827*	222	(C/T)
CakSNP15193	*Kabuli*	*Ca_Kabuli_Scaffold284*	242841	(A/T)
CakSNP15194	*Kabuli*	*Ca_Kabuli_Scaffold284*	242778	(T/C)
CakSNP15195	*Kabuli*	*Ca_Kabuli_Scaffold284*	522764	(T/G)
CakSNP15196	*Kabuli*	*Ca_Kabuli_Scaffold284*	613764	(G/A)
CakSNP15197	*Kabuli*	*Ca_Kabuli_Scaffold284*	613861	(C/T)
CakSNP15198	*Kabuli*	*Ca_Kabuli_Scaffold284*	613850	(C/T)
CakSNP15199	*Kabuli*	*Ca_Kabuli_Scaffold284*	613811	(G/A)
CakSNP15200	*Kabuli*	*Ca_Kabuli_Scaffold284*	826690	(T/C)
CakSNP15201	*Kabuli*	*Ca_Kabuli_Scaffold284*	826644	(G/A)
CakSNP15202	*Kabuli*	*Ca_Kabuli_Scaffold2845*	7107	(G/A)
CakSNP15203	*Kabuli*	*Ca_Kabuli_Scaffold2848*	2403	(C/A)
CakSNP15204	*Kabuli*	*Ca_Kabuli_Scaffold2848*	2444	(C/A)
CakSNP15205	*Kabuli*	*Ca_Kabuli_Scaffold2848*	2517	(G/A)
CakSNP15206	*Kabuli*	*Ca_Kabuli_Scaffold2848*	2464	(A/G)
CakSNP15207	*Kabuli*	*Ca_Kabuli_Scaffold2853*	14852	(C/G)
CakSNP15208	*Kabuli*	*Ca_Kabuli_Scaffold2853*	14916	(A/G)
CakSNP15209	*Kabuli*	*Ca_Kabuli_Scaffold2853*	14917	(G/A)
CakSNP15210	*Kabuli*	*Ca_Kabuli_Scaffold2853*	15100	(T/C)

**SNP IDs**	**Cultivars**	**Chromosomes/scaffolds**	**Physical positions (bp)**	**SNPs**
CakSNP15211	*Kabuli*	*Ca_Kabuli_Scaffold2853*	15086	(G/A)
CakSNP15212	*Kabuli*	*Ca_Kabuli_Scaffold287*	3224	(G/A)
CakSNP15213	*Kabuli*	*Ca_Kabuli_Scaffold287*	163600	(G/C)
CakSNP15214	*Kabuli*	*Ca_Kabuli_Scaffold287*	163591	(C/A)
CakSNP15215	*Kabuli*	*Ca_Kabuli_Scaffold287*	163576	(G/A)
CakSNP15216	*Kabuli*	*Ca_Kabuli_Scaffold290*	179998	(T/C)
CakSNP15217	*Kabuli*	*Ca_Kabuli_Scaffold290*	179993	(C/T)
CakSNP15218	*Kabuli*	*Ca_Kabuli_Scaffold290*	179984	(G/T)
CakSNP15219	*Kabuli*	*Ca_Kabuli_Scaffold290*	179940	(G/A)
CakSNP15220	*Kabuli*	*Ca_Kabuli_Scaffold290*	179937	(C/G)
CakSNP15221	*Kabuli*	*Ca_Kabuli_Scaffold290*	179969	(A/T)
CakSNP15222	*Kabuli*	*Ca_Kabuli_Scaffold290*	211634	(A/C)
CakSNP15223	*Kabuli*	*Ca_Kabuli_Scaffold290*	212536	(A/C)
CakSNP15224	*Kabuli*	*Ca_Kabuli_Scaffold290*	212514	(C/T)
CakSNP15225	*Kabuli*	*Ca_Kabuli_Scaffold290*	255161	(G/A)
CakSNP15226	*Kabuli*	*Ca_Kabuli_Scaffold290*	728010	(T/A)
CakSNP15227	*Kabuli*	*Ca_Kabuli_Scaffold290*	824906	(C/T)
CakSNP15228	*Kabuli*	*Ca_Kabuli_Scaffold2907*	31202	(G/A)
CakSNP15229	*Kabuli*	*Ca_Kabuli_Scaffold2950*	32650	(C/T)
CakSNP15230	*Kabuli*	*Ca_Kabuli_Scaffold2950*	32703	(A/C)
CakSNP15231	*Kabuli*	*Ca_Kabuli_Scaffold2950*	32739	(C/A)
CakSNP15232	*Kabuli*	*Ca_Kabuli_Scaffold2950*	32776	(T/A)
CakSNP15233	*Kabuli*	*Ca_Kabuli_Scaffold2950*	33081	(A/G)
CakSNP15234	*Kabuli*	*Ca_Kabuli_Scaffold2950*	33207	(C/T)
CakSNP15235	*Kabuli*	*Ca_Kabuli_Scaffold296*	41422	(T/G)
CakSNP15236	*Kabuli*	*Ca_Kabuli_Scaffold296*	59691	(G/A)
CakSNP15237	*Kabuli*	*Ca_Kabuli_Scaffold296*	59758	(A/G)
CakSNP15238	*Kabuli*	*Ca_Kabuli_Scaffold296*	59883	(A/C)
CakSNP15239	*Kabuli*	*Ca_Kabuli_Scaffold296*	361664	(T/C)
CakSNP15240	*Kabuli*	*Ca_Kabuli_Scaffold299*	199050	(A/T)
CakSNP15241	*Kabuli*	*Ca_Kabuli_Scaffold299*	199067	(A/T)
CakSNP15242	*Kabuli*	*Ca_Kabuli_Scaffold299*	199068	(G/T)
CakSNP15243	*Kabuli*	*Ca_Kabuli_Scaffold299*	199087	(G/A)
CakSNP15244	*Kabuli*	*Ca_Kabuli_Scaffold299*	199073	(G/A)
CakSNP15245	*Kabuli*	*Ca_Kabuli_Scaffold299*	204540	(G/A)
CakSNP15246	*Kabuli*	*Ca_Kabuli_Scaffold300*	111914	(C/T)
CakSNP15247	*Kabuli*	*Ca_Kabuli_Scaffold300*	111924	(C/T)
CakSNP15248	*Kabuli*	*Ca_Kabuli_Scaffold300*	111926	(G/A)
CakSNP15249	*Kabuli*	*Ca_Kabuli_Scaffold300*	111933	(C/T)
CakSNP15250	*Kabuli*	*Ca_Kabuli_Scaffold300*	111936	(C/A)
CakSNP15251	*Kabuli*	*Ca_Kabuli_Scaffold300*	111972	(A/T)

**SNP IDs**	**Cultivars**	**Chromosomes/scaffolds**	**Physical positions (bp)**	**SNPs**
CakSNP15252	*Kabuli*	*Ca_Kabuli_Scaffold300*	111973	(A/T)
CakSNP15253	*Kabuli*	*Ca_Kabuli_Scaffold300*	111976	(C/T)
CakSNP15254	*Kabuli*	*Ca_Kabuli_Scaffold300*	112011	(T/C)
CakSNP15255	*Kabuli*	*Ca_Kabuli_Scaffold300*	112006	(G/A)
CakSNP15256	*Kabuli*	*Ca_Kabuli_Scaffold300*	112005	(G/A)
CakSNP15257	*Kabuli*	*Ca_Kabuli_Scaffold300*	111959	(C/T)
CakSNP15258	*Kabuli*	*Ca_Kabuli_Scaffold300*	111964	(C/T)
CakSNP15259	*Kabuli*	*Ca_Kabuli_Scaffold3016*	32790	(C/T)
CakSNP15260	*Kabuli*	*Ca_Kabuli_Scaffold303*	18213	(G/A)
CakSNP15261	*Kabuli*	*Ca_Kabuli_Scaffold305*	49263	(G/A)
CakSNP15262	*Kabuli*	*Ca_Kabuli_Scaffold305*	49266	(T/G)
CakSNP15263	*Kabuli*	*Ca_Kabuli_Scaffold305*	49278	(C/A)
CakSNP15264	*Kabuli*	*Ca_Kabuli_Scaffold305*	138929	(G/A)
CakSNP15265	*Kabuli*	*Ca_Kabuli_Scaffold306*	15514	(C/T)
CakSNP15266	*Kabuli*	*Ca_Kabuli_Scaffold306*	19215	(C/A)
CakSNP15267	*Kabuli*	*Ca_Kabuli_Scaffold306*	85993	(T/A)
CakSNP15268	*Kabuli*	*Ca_Kabuli_Scaffold306*	88389	(T/A)
CakSNP15269	*Kabuli*	*Ca_Kabuli_Scaffold3084*	4409	(G/A)
CakSNP15270	*Kabuli*	*Ca_Kabuli_Scaffold308_2*	9421	(G/A)
CakSNP15271	*Kabuli*	*Ca_Kabuli_Scaffold308_2*	143696	(C/G)
CakSNP15272	*Kabuli*	*Ca_Kabuli_Scaffold308_2*	143813	(C/G)
CakSNP15273	*Kabuli*	*Ca_Kabuli_Scaffold308_2*	144354	(C/T)
CakSNP15274	*Kabuli*	*Ca_Kabuli_Scaffold308_2*	144508	(C/T)
CakSNP15275	*Kabuli*	*Ca_Kabuli_Scaffold308_2*	145639	(G/T)
CakSNP15276	*Kabuli*	*Ca_Kabuli_Scaffold308_2*	152546	(A/G)
CakSNP15277	*Kabuli*	*Ca_Kabuli_Scaffold308_2*	152576	(G/T)
CakSNP15278	*Kabuli*	*Ca_Kabuli_Scaffold311*	86652	(C/A)
CakSNP15279	*Kabuli*	*Ca_Kabuli_Scaffold311*	86677	(C/T)
CakSNP15280	*Kabuli*	*Ca_Kabuli_Scaffold311*	86658	(C/T)
CakSNP15281	*Kabuli*	*Ca_Kabuli_Scaffold311*	86670	(G/A)
CakSNP15282	*Kabuli*	*Ca_Kabuli_Scaffold3116*	159	(T/G)
CakSNP15283	*Kabuli*	*Ca_Kabuli_Scaffold314*	432453	(T/C)
CakSNP15284	*Kabuli*	*Ca_Kabuli_Scaffold314*	432471	(A/G)
CakSNP15285	*Kabuli*	*Ca_Kabuli_Scaffold314*	535269	(A/C)
CakSNP15286	*Kabuli*	*Ca_Kabuli_Scaffold314*	535266	(A/C)
CakSNP15287	*Kabuli*	*Ca_Kabuli_Scaffold314*	535263	(T/C)
CakSNP15288	*Kabuli*	*Ca_Kabuli_Scaffold314*	610535	(C/G)
CakSNP15289	*Kabuli*	*Ca_Kabuli_Scaffold314*	624361	(C/T)
CakSNP15290	*Kabuli*	*Ca_Kabuli_Scaffold3155*	7438	(A/C)
CakSNP15291	*Kabuli*	*Ca_Kabuli_Scaffold3155*	7442	(T/C)
CakSNP15292	*Kabuli*	*Ca_Kabuli_Scaffold3155*	7443	(A/G)

SNP IDs	Cultivars	Chromosomes/scaffolds	Physical positions (bp)	SNPs
CakSNP15293	*Kabuli*	*Ca_Kabuli_Scaffold3155*	7444	(A/G)
CakSNP15294	*Kabuli*	*Ca_Kabuli_Scaffold3155*	7455	(T/G)
CakSNP15295	*Kabuli*	*Ca_Kabuli_Scaffold3155*	7462	(A/G)
CakSNP15296	*Kabuli*	*Ca_Kabuli_Scaffold3155*	7464	(T/A)
CakSNP15297	*Kabuli*	*Ca_Kabuli_Scaffold3155*	7472	(A/G)
CakSNP15298	*Kabuli*	*Ca_Kabuli_Scaffold3155*	7477	(C/G)
CakSNP15299	*Kabuli*	*Ca_Kabuli_Scaffold3155*	7480	(G/A)
CakSNP15300	*Kabuli*	*Ca_Kabuli_Scaffold3155*	9404	(C/T)
CakSNP15301	*Kabuli*	*Ca_Kabuli_Scaffold3155*	9746	(A/T)
CakSNP15302	*Kabuli*	*Ca_Kabuli_Scaffold3170*	56261	(A/G)
CakSNP15303	*Kabuli*	*Ca_Kabuli_Scaffold322*	55475	(G/A)
CakSNP15304	*Kabuli*	*Ca_Kabuli_Scaffold322*	55479	(G/T)
CakSNP15305	*Kabuli*	*Ca_Kabuli_Scaffold322*	55589	(A/T)
CakSNP15306	*Kabuli*	*Ca_Kabuli_Scaffold3228*	2926	(C/T)
CakSNP15307	*Kabuli*	*Ca_Kabuli_Scaffold3228*	23138	(A/G)
CakSNP15308	*Kabuli*	*Ca_Kabuli_Scaffold3228*	23190	(G/C)
CakSNP15309	*Kabuli*	*Ca_Kabuli_Scaffold3228*	23315	(C/A)
CakSNP15310	*Kabuli*	*Ca_Kabuli_Scaffold3228*	23270	(A/C)
CakSNP15311	*Kabuli*	*Ca_Kabuli_Scaffold324*	15569	(A/C)
CakSNP15312	*Kabuli*	*Ca_Kabuli_Scaffold324*	15574	(G/A)
CakSNP15313	*Kabuli*	*Ca_Kabuli_Scaffold324*	15575	(G/C)
CakSNP15314	*Kabuli*	*Ca_Kabuli_Scaffold324*	15581	(G/C)
CakSNP15315	*Kabuli*	*Ca_Kabuli_Scaffold324*	15590	(G/T)
CakSNP15316	*Kabuli*	*Ca_Kabuli_Scaffold324*	15593	(G/A)
CakSNP15317	*Kabuli*	*Ca_Kabuli_Scaffold324*	15594	(C/A)
CakSNP15318	*Kabuli*	*Ca_Kabuli_Scaffold324*	15614	(C/T)
CakSNP15319	*Kabuli*	*Ca_Kabuli_Scaffold324*	15619	(A/G)
CakSNP15320	*Kabuli*	*Ca_Kabuli_Scaffold324*	15636	(G/A)
CakSNP15321	*Kabuli*	*Ca_Kabuli_Scaffold3254_2*	15616	(A/G)
CakSNP15322	*Kabuli*	*Ca_Kabuli_Scaffold3254_2*	28089	(T/C)
CakSNP15323	*Kabuli*	*Ca_Kabuli_Scaffold3254_2*	31210	(T/A)
CakSNP15324	*Kabuli*	*Ca_Kabuli_Scaffold3254_2*	31217	(C/T)
CakSNP15325	*Kabuli*	*Ca_Kabuli_Scaffold3254_2*	31219	(A/C)
CakSNP15326	*Kabuli*	*Ca_Kabuli_Scaffold3254_2*	31224	(T/G)
CakSNP15327	*Kabuli*	*Ca_Kabuli_Scaffold3254_2*	31229	(G/T)
CakSNP15328	*Kabuli*	*Ca_Kabuli_Scaffold3254_2*	31242	(C/A)
CakSNP15329	*Kabuli*	*Ca_Kabuli_Scaffold3254_2*	31344	(A/G)
CakSNP15330	*Kabuli*	*Ca_Kabuli_Scaffold3254_2*	31325	(T/A)
CakSNP15331	*Kabuli*	*Ca_Kabuli_Scaffold3254_2*	31322	(G/A)
CakSNP15332	*Kabuli*	*Ca_Kabuli_Scaffold3254_2*	31311	(G/A)
CakSNP15333	*Kabuli*	*Ca_Kabuli_Scaffold3254_2*	31303	(T/C)

**SNP IDs**	**Cultivars**	**Chromosomes/scaffolds**	**Physical positions (bp)**	**SNPs**
CakSNP15334	*Kabuli*	*Ca_Kabuli_Scaffold3254_2*	39157	(T/A)
CakSNP15335	*Kabuli*	*Ca_Kabuli_Scaffold3254_2*	46644	(G/A)
CakSNP15336	*Kabuli*	*Ca_Kabuli_Scaffold3254_2*	186218	(G/A)
CakSNP15337	*Kabuli*	*Ca_Kabuli_Scaffold3254_2*	186203	(C/G)
CakSNP15338	*Kabuli*	*Ca_Kabuli_Scaffold3254_2*	186243	(A/C)
CakSNP15339	*Kabuli*	*Ca_Kabuli_Scaffold3284*	2735	(T/C)
CakSNP15340	*Kabuli*	*Ca_Kabuli_Scaffold332*	203691	(T/A)
CakSNP15341	*Kabuli*	*Ca_Kabuli_Scaffold332*	204783	(A/G)
CakSNP15342	*Kabuli*	*Ca_Kabuli_Scaffold332*	204785	(G/A)
CakSNP15343	*Kabuli*	*Ca_Kabuli_Scaffold332*	204952	(G/A)
CakSNP15344	*Kabuli*	*Ca_Kabuli_Scaffold3321*	17935	(C/A)
CakSNP15345	*Kabuli*	*Ca_Kabuli_Scaffold3337*	17963	(C/T)
CakSNP15346	*Kabuli*	*Ca_Kabuli_Scaffold3337*	17999	(T/G)
CakSNP15347	*Kabuli*	*Ca_Kabuli_Scaffold3337*	18022	(C/T)
CakSNP15348	*Kabuli*	*Ca_Kabuli_Scaffold3337*	18026	(T/A)
CakSNP15349	*Kabuli*	*Ca_Kabuli_Scaffold3337*	17959	(A/G)
CakSNP15350	*Kabuli*	*Ca_Kabuli_Scaffold3337*	17956	(G/A)
CakSNP15351	*Kabuli*	*Ca_Kabuli_Scaffold334*	43160	(G/A)
CakSNP15352	*Kabuli*	*Ca_Kabuli_Scaffold335*	60951	(T/C)
CakSNP15353	*Kabuli*	*Ca_Kabuli_Scaffold335*	60952	(T/C)
CakSNP15354	*Kabuli*	*Ca_Kabuli_Scaffold335*	60956	(T/C)
CakSNP15355	*Kabuli*	*Ca_Kabuli_Scaffold335*	60957	(T/C)
CakSNP15356	*Kabuli*	*Ca_Kabuli_Scaffold335*	60960	(T/C)
CakSNP15357	*Kabuli*	*Ca_Kabuli_Scaffold335*	60961	(C/T)
CakSNP15358	*Kabuli*	*Ca_Kabuli_Scaffold335*	60962	(T/G)
CakSNP15359	*Kabuli*	*Ca_Kabuli_Scaffold335*	60906	(T/G)
CakSNP15360	*Kabuli*	*Ca_Kabuli_Scaffold335*	135942	(T/A)
CakSNP15361	*Kabuli*	*Ca_Kabuli_Scaffold335*	136017	(C/G)
CakSNP15362	*Kabuli*	*Ca_Kabuli_Scaffold335*	135969	(C/T)
CakSNP15363	*Kabuli*	*Ca_Kabuli_Scaffold335*	135976	(C/T)
CakSNP15364	*Kabuli*	*Ca_Kabuli_Scaffold335*	136077	(G/C)
CakSNP15365	*Kabuli*	*Ca_Kabuli_Scaffold335*	136076	(G/C)
CakSNP15366	*Kabuli*	*Ca_Kabuli_Scaffold335*	136073	(G/A)
CakSNP15367	*Kabuli*	*Ca_Kabuli_Scaffold335*	136051	(T/A)
CakSNP15368	*Kabuli*	*Ca_Kabuli_Scaffold335*	136030	(G/A)
CakSNP15369	*Kabuli*	*Ca_Kabuli_Scaffold335*	136129	(T/C)
CakSNP15370	*Kabuli*	*Ca_Kabuli_Scaffold335*	136109	(G/T)
CakSNP15371	*Kabuli*	*Ca_Kabuli_Scaffold335*	136106	(G/A)
CakSNP15372	*Kabuli*	*Ca_Kabuli_Scaffold335*	136096	(T/C)
CakSNP15373	*Kabuli*	*Ca_Kabuli_Scaffold335*	136091	(G/A)
CakSNP15374	*Kabuli*	*Ca_Kabuli_Scaffold336*	468863	(T/A)

SNP IDs	Cultivars	Chromosomes/scaffolds	Physical positions (bp)	SNPs
CakSNP15375	*Kabuli*	*Ca_Kabuli_Scaffold336*	563363	(C/T)
CakSNP15376	*Kabuli*	*Ca_Kabuli_Scaffold336*	618427	(C/T)
CakSNP15377	*Kabuli*	*Ca_Kabuli_Scaffold336*	771169	(C/T)
CakSNP15378	*Kabuli*	*Ca_Kabuli_Scaffold336*	771175	(C/T)
CakSNP15379	*Kabuli*	*Ca_Kabuli_Scaffold3362*	69878	(C/A)
CakSNP15380	*Kabuli*	*Ca_Kabuli_Scaffold338*	14666	(T/G)
CakSNP15381	*Kabuli*	*Ca_Kabuli_Scaffold3397*	45363	(T/C)
CakSNP15382	*Kabuli*	*Ca_Kabuli_Scaffold342*	311411	(G/A)
CakSNP15383	*Kabuli*	*Ca_Kabuli_Scaffold342*	311394	(A/C)
CakSNP15384	*Kabuli*	*Ca_Kabuli_Scaffold342*	359376	(A/C)
CakSNP15385	*Kabuli*	*Ca_Kabuli_Scaffold3422*	13376	(T/A)
CakSNP15386	*Kabuli*	*Ca_Kabuli_Scaffold346_1*	67110	(G/T)
CakSNP15387	*Kabuli*	*Ca_Kabuli_Scaffold346_1*	105461	(A/G)
CakSNP15388	*Kabuli*	*Ca_Kabuli_Scaffold346_1*	105521	(A/C)
CakSNP15389	*Kabuli*	*Ca_Kabuli_Scaffold346_1*	105523	(G/A)
CakSNP15390	*Kabuli*	*Ca_Kabuli_Scaffold346_1*	105546	(C/T)
CakSNP15391	*Kabuli*	*Ca_Kabuli_Scaffold349*	51381	(A/T)
CakSNP15392	*Kabuli*	*Ca_Kabuli_Scaffold349*	51396	(G/A)
CakSNP15393	*Kabuli*	*Ca_Kabuli_Scaffold349*	51414	(C/T)
CakSNP15394	*Kabuli*	*Ca_Kabuli_Scaffold349*	51435	(C/T)
CakSNP15395	*Kabuli*	*Ca_Kabuli_Scaffold349*	51548	(T/C)
CakSNP15396	*Kabuli*	*Ca_Kabuli_Scaffold349*	51637	(T/A)
CakSNP15397	*Kabuli*	*Ca_Kabuli_Scaffold349*	51624	(G/A)
CakSNP15398	*Kabuli*	*Ca_Kabuli_Scaffold349*	174027	(G/C)
CakSNP15399	*Kabuli*	*Ca_Kabuli_Scaffold349*	174039	(A/T)
CakSNP15400	*Kabuli*	*Ca_Kabuli_Scaffold3514*	42134	(G/A)
CakSNP15401	*Kabuli*	*Ca_Kabuli_Scaffold3514*	42135	(G/T)
CakSNP15402	*Kabuli*	*Ca_Kabuli_Scaffold3514*	42156	(G/A)
CakSNP15403	*Kabuli*	*Ca_Kabuli_Scaffold3514*	42163	(T/C)
CakSNP15404	*Kabuli*	*Ca_Kabuli_Scaffold3514*	42164	(A/G)
CakSNP15405	*Kabuli*	*Ca_Kabuli_Scaffold3514*	42175	(G/A)
CakSNP15406	*Kabuli*	*Ca_Kabuli_Scaffold352*	22672	(C/T)
CakSNP15407	*Kabuli*	*Ca_Kabuli_Scaffold352*	22675	(G/A)
CakSNP15408	*Kabuli*	*Ca_Kabuli_Scaffold352*	22693	(C/T)
CakSNP15409	*Kabuli*	*Ca_Kabuli_Scaffold352*	22708	(A/G)
CakSNP15410	*Kabuli*	*Ca_Kabuli_Scaffold352*	22787	(A/G)
CakSNP15411	*Kabuli*	*Ca_Kabuli_Scaffold352*	22777	(T/G)
CakSNP15412	*Kabuli*	*Ca_Kabuli_Scaffold352*	22726	(A/C)
CakSNP15413	*Kabuli*	*Ca_Kabuli_Scaffold36*	167556	(C/T)
CakSNP15414	*Kabuli*	*Ca_Kabuli_Scaffold36*	226511	(A/G)
CakSNP15415	*Kabuli*	*Ca_Kabuli_Scaffold362*	84943	(A/G)

**SNP IDs**	**Cultivars**	**Chromosomes/scaffolds**	**Physical positions (bp)**	**SNPs**
CakSNP15416	*Kabuli*	*Ca_Kabuli_Scaffold362*	84945	(T/C)
CakSNP15417	*Kabuli*	*Ca_Kabuli_Scaffold362*	84954	(T/C)
CakSNP15418	*Kabuli*	*Ca_Kabuli_Scaffold362*	84972	(C/A)
CakSNP15419	*Kabuli*	*Ca_Kabuli_Scaffold362*	84988	(T/G)
CakSNP15420	*Kabuli*	*Ca_Kabuli_Scaffold362*	84994	(G/A)
CakSNP15421	*Kabuli*	*Ca_Kabuli_Scaffold362*	164620	(C/T)
CakSNP15422	*Kabuli*	*Ca_Kabuli_Scaffold362*	164789	(T/C)
CakSNP15423	*Kabuli*	*Ca_Kabuli_Scaffold362*	164787	(C/T)
CakSNP15424	*Kabuli*	*Ca_Kabuli_Scaffold362*	196556	(G/A)
CakSNP15425	*Kabuli*	*Ca_Kabuli_Scaffold362*	219176	(T/C)
CakSNP15426	*Kabuli*	*Ca_Kabuli_Scaffold366*	61810	(C/T)
CakSNP15427	*Kabuli*	*Ca_Kabuli_Scaffold369*	162735	(T/G)
CakSNP15428	*Kabuli*	*Ca_Kabuli_Scaffold3693*	81068	(C/T)
CakSNP15429	*Kabuli*	*Ca_Kabuli_Scaffold3693*	81080	(C/A)
CakSNP15430	*Kabuli*	*Ca_Kabuli_Scaffold3693*	81204	(C/T)
CakSNP15431	*Kabuli*	*Ca_Kabuli_Scaffold3724*	68378	(G/A)
CakSNP15432	*Kabuli*	*Ca_Kabuli_Scaffold374*	132907	(A/C)
CakSNP15433	*Kabuli*	*Ca_Kabuli_Scaffold374*	132896	(T/C)
CakSNP15434	*Kabuli*	*Ca_Kabuli_Scaffold374*	132873	(A/G)
CakSNP15435	*Kabuli*	*Ca_Kabuli_Scaffold374*	132859	(C/T)
CakSNP15436	*Kabuli*	*Ca_Kabuli_Scaffold374*	132858	(G/A)
CakSNP15437	*Kabuli*	*Ca_Kabuli_Scaffold374*	132849	(A/C)
CakSNP15438	*Kabuli*	*Ca_Kabuli_Scaffold374*	132843	(C/T)
CakSNP15439	*Kabuli*	*Ca_Kabuli_Scaffold374*	198368	(T/C)
CakSNP15440	*Kabuli*	*Ca_Kabuli_Scaffold377*	393535	(G/T)
CakSNP15441	*Kabuli*	*Ca_Kabuli_Scaffold377*	393538	(G/A)
CakSNP15442	*Kabuli*	*Ca_Kabuli_Scaffold379*	69075	(A/C)
CakSNP15443	*Kabuli*	*Ca_Kabuli_Scaffold379*	144442	(G/A)
CakSNP15444	*Kabuli*	*Ca_Kabuli_Scaffold379*	177306	(T/C)
CakSNP15445	*Kabuli*	*Ca_Kabuli_Scaffold379*	177354	(G/A)
CakSNP15446	*Kabuli*	*Ca_Kabuli_Scaffold379*	177340	(C/T)
CakSNP15447	*Kabuli*	*Ca_Kabuli_Scaffold379*	227608	(A/T)
CakSNP15448	*Kabuli*	*Ca_Kabuli_Scaffold379*	227604	(C/A)
CakSNP15449	*Kabuli*	*Ca_Kabuli_Scaffold379*	227597	(T/A)
CakSNP15450	*Kabuli*	*Ca_Kabuli_Scaffold38*	69499	(A/G)
CakSNP15451	*Kabuli*	*Ca_Kabuli_Scaffold38*	413691	(G/A)
CakSNP15452	*Kabuli*	*Ca_Kabuli_Scaffold38*	413713	(A/C)
CakSNP15453	*Kabuli*	*Ca_Kabuli_Scaffold38*	413719	(G/A)
CakSNP15454	*Kabuli*	*Ca_Kabuli_Scaffold38*	457392	(C/A)
CakSNP15455	*Kabuli*	*Ca_Kabuli_Scaffold38*	457596	(T/A)
CakSNP15456	*Kabuli*	*Ca_Kabuli_Scaffold38*	520372	(T/G)

SNP IDs	Cultivars	Chromosomes/scaffolds	Physical positions (bp)	SNPs
CakSNP15457	*Kabuli*	*Ca_Kabuli_Scaffold38*	520311	(G/A)
CakSNP15458	*Kabuli*	*Ca_Kabuli_Scaffold38*	520377	(T/C)
CakSNP15459	*Kabuli*	*Ca_Kabuli_Scaffold38*	537059	(A/T)
CakSNP15460	*Kabuli*	*Ca_Kabuli_Scaffold38*	537063	(C/T)
CakSNP15461	*Kabuli*	*Ca_Kabuli_Scaffold3819*	450	(T/A)
CakSNP15462	*Kabuli*	*Ca_Kabuli_Scaffold382*	36686	(A/T)
CakSNP15463	*Kabuli*	*Ca_Kabuli_Scaffold382*	36703	(A/T)
CakSNP15464	*Kabuli*	*Ca_Kabuli_Scaffold382*	36704	(G/T)
CakSNP15465	*Kabuli*	*Ca_Kabuli_Scaffold382*	36709	(G/A)
CakSNP15466	*Kabuli*	*Ca_Kabuli_Scaffold382*	36674	(G/A)
CakSNP15467	*Kabuli*	*Ca_Kabuli_Scaffold3824*	26207	(G/A)
CakSNP15468	*Kabuli*	*Ca_Kabuli_Scaffold3824*	26267	(G/A)
CakSNP15469	*Kabuli*	*Ca_Kabuli_Scaffold3824*	26327	(G/A)
CakSNP15470	*Kabuli*	*Ca_Kabuli_Scaffold3824*	26387	(G/A)
CakSNP15471	*Kabuli*	*Ca_Kabuli_Scaffold3865*	12265	(T/A)
CakSNP15472	*Kabuli*	*Ca_Kabuli_Scaffold3865*	65093	(T/C)
CakSNP15473	*Kabuli*	*Ca_Kabuli_Scaffold3865*	65127	(C/A)
CakSNP15474	*Kabuli*	*Ca_Kabuli_Scaffold3865*	65129	(T/A)
CakSNP15475	*Kabuli*	*Ca_Kabuli_Scaffold387*	3263	(G/A)
CakSNP15476	*Kabuli*	*Ca_Kabuli_Scaffold387*	3219	(C/A)
CakSNP15477	*Kabuli*	*Ca_Kabuli_Scaffold387*	3309	(T/C)
CakSNP15478	*Kabuli*	*Ca_Kabuli_Scaffold387*	3316	(G/A)
CakSNP15479	*Kabuli*	*Ca_Kabuli_Scaffold3945*	3367	(G/A)
CakSNP15480	*Kabuli*	*Ca_Kabuli_Scaffold3945*	3371	(G/A)
CakSNP15481	*Kabuli*	*Ca_Kabuli_Scaffold3945*	3880	(G/T)
CakSNP15482	*Kabuli*	*Ca_Kabuli_Scaffold3945*	3879	(T/C)
CakSNP15483	*Kabuli*	*Ca_Kabuli_Scaffold3945*	7260	(T/A)
CakSNP15484	*Kabuli*	*Ca_Kabuli_Scaffold3945*	13468	(C/T)
CakSNP15485	*Kabuli*	*Ca_Kabuli_Scaffold3945*	13440	(C/T)
CakSNP15486	*Kabuli*	*Ca_Kabuli_Scaffold3945*	13892	(C/G)
CakSNP15487	*Kabuli*	*Ca_Kabuli_Scaffold3945*	18891	(A/T)
CakSNP15488	*Kabuli*	*Ca_Kabuli_Scaffold3945*	18892	(G/C)
CakSNP15489	*Kabuli*	*Ca_Kabuli_Scaffold3945*	19359	(G/A)
CakSNP15490	*Kabuli*	*Ca_Kabuli_Scaffold3945*	19366	(G/T)
CakSNP15491	*Kabuli*	*Ca_Kabuli_Scaffold3945*	19367	(A/T)
CakSNP15492	*Kabuli*	*Ca_Kabuli_Scaffold3945*	19368	(A/C)
CakSNP15493	*Kabuli*	*Ca_Kabuli_Scaffold396*	130566	(T/C)
CakSNP15494	*Kabuli*	*Ca_Kabuli_Scaffold396*	130671	(A/G)
CakSNP15495	*Kabuli*	*Ca_Kabuli_Scaffold396*	130639	(C/A)
CakSNP15496	*Kabuli*	*Ca_Kabuli_Scaffold396*	130636	(G/A)
CakSNP15497	*Kabuli*	*Ca_Kabuli_Scaffold396*	130635	(G/T)

SNP IDs	Cultivars	Chromosomes/scaffolds	Physical positions (bp)	SNPs
CakSNP15498	*Kabuli*	*Ca_Kabuli_Scaffold396*	130624	(A/G)
CakSNP15499	*Kabuli*	*Ca_Kabuli_Scaffold396*	130621	(C/A)
CakSNP15500	*Kabuli*	*Ca_Kabuli_Scaffold396*	195890	(A/G)
CakSNP15501	*Kabuli*	*Ca_Kabuli_Scaffold396*	297325	(G/C)
CakSNP15502	*Kabuli*	*Ca_Kabuli_Scaffold398*	246956	(C/T)
CakSNP15503	*Kabuli*	*Ca_Kabuli_Scaffold398*	247004	(C/T)
CakSNP15504	*Kabuli*	*Ca_Kabuli_Scaffold398*	441570	(C/T)
CakSNP15505	*Kabuli*	*Ca_Kabuli_Scaffold398*	441605	(C/A)
CakSNP15506	*Kabuli*	*Ca_Kabuli_Scaffold40*	386189	(A/C)
CakSNP15507	*Kabuli*	*Ca_Kabuli_Scaffold40*	497729	(T/C)
CakSNP15508	*Kabuli*	*Ca_Kabuli_Scaffold40*	542951	(C/G)
CakSNP15509	*Kabuli*	*Ca_Kabuli_Scaffold40*	548453	(A/G)
CakSNP15510	*Kabuli*	*Ca_Kabuli_Scaffold40*	636910	(C/T)
CakSNP15511	*Kabuli*	*Ca_Kabuli_Scaffold40*	691226	(C/G)
CakSNP15512	*Kabuli*	*Ca_Kabuli_Scaffold40*	871303	(A/C)
CakSNP15513	*Kabuli*	*Ca_Kabuli_Scaffold40*	911602	(T/G)
CakSNP15514	*Kabuli*	*Ca_Kabuli_Scaffold40*	968798	(G/A)
CakSNP15515	*Kabuli*	*Ca_Kabuli_Scaffold40*	996604	(T/C)
CakSNP15516	*Kabuli*	*Ca_Kabuli_Scaffold40*	1014875	(G/A)
CakSNP15517	*Kabuli*	*Ca_Kabuli_Scaffold40*	1014834	(T/A)
CakSNP15518	*Kabuli*	*Ca_Kabuli_Scaffold40*	1014826	(C/T)
CakSNP15519	*Kabuli*	*Ca_Kabuli_Scaffold40*	1014805	(C/T)
CakSNP15520	*Kabuli*	*Ca_Kabuli_Scaffold40*	1039218	(T/C)
CakSNP15521	*Kabuli*	*Ca_Kabuli_Scaffold40*	1039233	(A/T)
CakSNP15522	*Kabuli*	*Ca_Kabuli_Scaffold40*	1049594	(G/A)
CakSNP15523	*Kabuli*	*Ca_Kabuli_Scaffold40*	1050111	(C/T)
CakSNP15524	*Kabuli*	*Ca_Kabuli_Scaffold40*	1050164	(C/T)
CakSNP15525	*Kabuli*	*Ca_Kabuli_Scaffold40*	1050255	(T/C)
CakSNP15526	*Kabuli*	*Ca_Kabuli_Scaffold40*	1050175	(G/C)
CakSNP15527	*Kabuli*	*Ca_Kabuli_Scaffold40*	1052597	(T/C)
CakSNP15528	*Kabuli*	*Ca_Kabuli_Scaffold40*	1052983	(G/A)
CakSNP15529	*Kabuli*	*Ca_Kabuli_Scaffold40*	1053215	(G/A)
CakSNP15530	*Kabuli*	*Ca_Kabuli_Scaffold401*	49065	(G/A)
CakSNP15531	*Kabuli*	*Ca_Kabuli_Scaffold4011*	93334	(T/C)
CakSNP15532	*Kabuli*	*Ca_Kabuli_Scaffold4011*	93347	(C/T)
CakSNP15533	*Kabuli*	*Ca_Kabuli_Scaffold4011*	96223	(G/C)
CakSNP15534	*Kabuli*	*Ca_Kabuli_Scaffold4011*	97602	(T/C)
CakSNP15535	*Kabuli*	*Ca_Kabuli_Scaffold4011*	130271	(G/A)
CakSNP15536	*Kabuli*	*Ca_Kabuli_Scaffold4011*	130213	(G/A)
CakSNP15537	*Kabuli*	*Ca_Kabuli_Scaffold404*	273203	(C/A)
CakSNP15538	*Kabuli*	*Ca_Kabuli_Scaffold404*	273209	(C/A)

SNP IDs	Cultivars	Chromosomes/scaffolds	Physical positions (bp)	SNPs
CakSNP15539	*Kabuli*	*Ca_Kabuli_Scaffold404*	273220	(G/A)
CakSNP15540	*Kabuli*	*Ca_Kabuli_Scaffold404*	273245	(C/T)
CakSNP15541	*Kabuli*	*Ca_Kabuli_Scaffold404*	285941	(G/A)
CakSNP15542	*Kabuli*	*Ca_Kabuli_Scaffold404*	285952	(T/C)
CakSNP15543	*Kabuli*	*Ca_Kabuli_Scaffold404*	285958	(C/G)
CakSNP15544	*Kabuli*	*Ca_Kabuli_Scaffold404*	285987	(G/A)
CakSNP15545	*Kabuli*	*Ca_Kabuli_Scaffold404*	285999	(T/G)
CakSNP15546	*Kabuli*	*Ca_Kabuli_Scaffold404*	286013	(T/A)
CakSNP15547	*Kabuli*	*Ca_Kabuli_Scaffold404*	355859	(G/A)
CakSNP15548	*Kabuli*	*Ca_Kabuli_Scaffold4057*	16503	(C/G)
CakSNP15549	*Kabuli*	*Ca_Kabuli_Scaffold41*	14657	(C/A)
CakSNP15550	*Kabuli*	*Ca_Kabuli_Scaffold41*	122583	(G/C)
CakSNP15551	*Kabuli*	*Ca_Kabuli_Scaffold4102*	1035	(C/G)
CakSNP15552	*Kabuli*	*Ca_Kabuli_Scaffold4102*	1136	(A/G)
CakSNP15553	*Kabuli*	*Ca_Kabuli_Scaffold4102*	1160	(T/C)
CakSNP15554	*Kabuli*	*Ca_Kabuli_Scaffold4102*	1163	(G/T)
CakSNP15555	*Kabuli*	*Ca_Kabuli_Scaffold4102*	1346	(C/T)
CakSNP15556	*Kabuli*	*Ca_Kabuli_Scaffold411*	60078	(C/T)
CakSNP15557	*Kabuli*	*Ca_Kabuli_Scaffold411*	323122	(T/C)
CakSNP15558	*Kabuli*	*Ca_Kabuli_Scaffold411*	323140	(T/C)
CakSNP15559	*Kabuli*	*Ca_Kabuli_Scaffold411*	323141	(G/A)
CakSNP15560	*Kabuli*	*Ca_Kabuli_Scaffold411*	323147	(T/C)
CakSNP15561	*Kabuli*	*Ca_Kabuli_Scaffold411*	323161	(G/A)
CakSNP15562	*Kabuli*	*Ca_Kabuli_Scaffold411*	323162	(T/C)
CakSNP15563	*Kabuli*	*Ca_Kabuli_Scaffold411*	323167	(C/G)
CakSNP15564	*Kabuli*	*Ca_Kabuli_Scaffold411*	323179	(T/G)
CakSNP15565	*Kabuli*	*Ca_Kabuli_Scaffold411*	323114	(C/G)
CakSNP15566	*Kabuli*	*Ca_Kabuli_Scaffold411*	323215	(T/C)
CakSNP15567	*Kabuli*	*Ca_Kabuli_Scaffold415*	8479	(A/C)
CakSNP15568	*Kabuli*	*Ca_Kabuli_Scaffold415*	8529	(G/A)
CakSNP15569	*Kabuli*	*Ca_Kabuli_Scaffold418*	6092	(C/G)
CakSNP15570	*Kabuli*	*Ca_Kabuli_Scaffold418*	6094	(T/G)
CakSNP15571	*Kabuli*	*Ca_Kabuli_Scaffold418*	13769	(T/G)
CakSNP15572	*Kabuli*	*Ca_Kabuli_Scaffold418*	13804	(T/G)
CakSNP15573	*Kabuli*	*Ca_Kabuli_Scaffold418*	13808	(A/G)
CakSNP15574	*Kabuli*	*Ca_Kabuli_Scaffold418*	13811	(G/A)
CakSNP15575	*Kabuli*	*Ca_Kabuli_Scaffold418*	13812	(C/A)
CakSNP15576	*Kabuli*	*Ca_Kabuli_Scaffold418*	151140	(G/T)
CakSNP15577	*Kabuli*	*Ca_Kabuli_Scaffold418*	151205	(C/T)
CakSNP15578	*Kabuli*	*Ca_Kabuli_Scaffold418*	151256	(G/A)
CakSNP15579	*Kabuli*	*Ca_Kabuli_Scaffold419*	73537	(T/G)

SNP IDs	Cultivars	Chromosomes/scaffolds	Physical positions (bp)	SNPs
CakSNP15580	*Kabuli*	*Ca_Kabuli_Scaffold420*	34145	(T/G)
CakSNP15581	*Kabuli*	*Ca_Kabuli_Scaffold420*	42532	(G/A)
CakSNP15582	*Kabuli*	*Ca_Kabuli_Scaffold420*	42739	(C/T)
CakSNP15583	*Kabuli*	*Ca_Kabuli_Scaffold420*	42811	(C/T)
CakSNP15584	*Kabuli*	*Ca_Kabuli_Scaffold420*	42877	(T/C)
CakSNP15585	*Kabuli*	*Ca_Kabuli_Scaffold420*	43372	(C/T)
CakSNP15586	*Kabuli*	*Ca_Kabuli_Scaffold420*	92756	(C/T)
CakSNP15587	*Kabuli*	*Ca_Kabuli_Scaffold420*	92759	(A/G)
CakSNP15588	*Kabuli*	*Ca_Kabuli_Scaffold420*	92784	(C/T)
CakSNP15589	*Kabuli*	*Ca_Kabuli_Scaffold420*	92813	(G/T)
CakSNP15590	*Kabuli*	*Ca_Kabuli_Scaffold420*	92865	(G/T)
CakSNP15591	*Kabuli*	*Ca_Kabuli_Scaffold420*	92846	(C/T)
CakSNP15592	*Kabuli*	*Ca_Kabuli_Scaffold420*	92801	(A/C)
CakSNP15593	*Kabuli*	*Ca_Kabuli_Scaffold420*	149744	(C/G)
CakSNP15594	*Kabuli*	*Ca_Kabuli_Scaffold420*	242536	(G/T)
CakSNP15595	*Kabuli*	*Ca_Kabuli_Scaffold420*	242503	(C/T)
CakSNP15596	*Kabuli*	*Ca_Kabuli_Scaffold420*	242487	(A/G)
CakSNP15597	*Kabuli*	*Ca_Kabuli_Scaffold420*	242922	(A/C)
CakSNP15598	*Kabuli*	*Ca_Kabuli_Scaffold420*	254661	(T/G)
CakSNP15599	*Kabuli*	*Ca_Kabuli_Scaffold420*	341146	(T/A)
CakSNP15600	*Kabuli*	*Ca_Kabuli_Scaffold420*	341184	(A/G)
CakSNP15601	*Kabuli*	*Ca_Kabuli_Scaffold420*	341202	(T/G)
CakSNP15602	*Kabuli*	*Ca_Kabuli_Scaffold420*	341206	(G/A)
CakSNP15603	*Kabuli*	*Ca_Kabuli_Scaffold421_1*	25389	(G/A)
CakSNP15604	*Kabuli*	*Ca_Kabuli_Scaffold421_1*	71804	(A/G)
CakSNP15605	*Kabuli*	*Ca_Kabuli_Scaffold421_1*	71770	(G/T)
CakSNP15606	*Kabuli*	*Ca_Kabuli_Scaffold421_2*	142859	(G/C)
CakSNP15607	*Kabuli*	*Ca_Kabuli_Scaffold421_2*	341095	(C/A)
CakSNP15608	*Kabuli*	*Ca_Kabuli_Scaffold421_2*	341136	(C/A)
CakSNP15609	*Kabuli*	*Ca_Kabuli_Scaffold421_2*	446961	(G/A)
CakSNP15610	*Kabuli*	*Ca_Kabuli_Scaffold421_2*	446976	(C/A)
CakSNP15611	*Kabuli*	*Ca_Kabuli_Scaffold421_2*	446979	(C/T)
CakSNP15612	*Kabuli*	*Ca_Kabuli_Scaffold424*	32402	(C/G)
CakSNP15613	*Kabuli*	*Ca_Kabuli_Scaffold4331*	4044	(T/G)
CakSNP15614	*Kabuli*	*Ca_Kabuli_Scaffold44*	66309	(A/C)
CakSNP15615	*Kabuli*	*Ca_Kabuli_Scaffold44*	66280	(G/A)
CakSNP15616	*Kabuli*	*Ca_Kabuli_Scaffold44*	87412	(G/A)
CakSNP15617	*Kabuli*	*Ca_Kabuli_Scaffold4414*	276	(C/T)
CakSNP15618	*Kabuli*	*Ca_Kabuli_Scaffold4452*	20003	(G/A)
CakSNP15619	*Kabuli*	*Ca_Kabuli_Scaffold4452*	20005	(G/A)
CakSNP15620	*Kabuli*	*Ca_Kabuli_Scaffold4452*	20038	(A/G)

SNP IDs	Cultivars	Chromosomes/scaffolds	Physical positions (bp)	SNPs
CakSNP15621	*Kabuli*	*Ca_Kabuli_Scaffold4452*	20010	(G/A)
CakSNP15622	*Kabuli*	*Ca_Kabuli_Scaffold4452*	29414	(G/T)
CakSNP15623	*Kabuli*	*Ca_Kabuli_Scaffold4452*	36715	(A/T)
CakSNP15624	*Kabuli*	*Ca_Kabuli_Scaffold4452*	69950	(A/C)
CakSNP15625	*Kabuli*	*Ca_Kabuli_Scaffold4452*	70041	(T/C)
CakSNP15626	*Kabuli*	*Ca_Kabuli_Scaffold450*	132367	(C/T)
CakSNP15627	*Kabuli*	*Ca_Kabuli_Scaffold450*	132427	(A/T)
CakSNP15628	*Kabuli*	*Ca_Kabuli_Scaffold450*	197287	(G/A)
CakSNP15629	*Kabuli*	*Ca_Kabuli_Scaffold450*	197272	(G/A)
CakSNP15630	*Kabuli*	*Ca_Kabuli_Scaffold450*	197262	(T/G)
CakSNP15631	*Kabuli*	*Ca_Kabuli_Scaffold450*	197230	(C/A)
CakSNP15632	*Kabuli*	*Ca_Kabuli_Scaffold450*	197226	(G/A)
CakSNP15633	*Kabuli*	*Ca_Kabuli_Scaffold450*	250303	(G/T)
CakSNP15634	*Kabuli*	*Ca_Kabuli_Scaffold451*	103555	(G/C)
CakSNP15635	*Kabuli*	*Ca_Kabuli_Scaffold451*	103596	(A/G)
CakSNP15636	*Kabuli*	*Ca_Kabuli_Scaffold451*	117231	(A/G)
CakSNP15637	*Kabuli*	*Ca_Kabuli_Scaffold451*	117300	(C/A)
CakSNP15638	*Kabuli*	*Ca_Kabuli_Scaffold4511*	40072	(C/A)
CakSNP15639	*Kabuli*	*Ca_Kabuli_Scaffold4511*	40014	(C/T)
CakSNP15640	*Kabuli*	*Ca_Kabuli_Scaffold4511*	40089	(C/T)
CakSNP15641	*Kabuli*	*Ca_Kabuli_Scaffold452*	1892	(G/A)
CakSNP15642	*Kabuli*	*Ca_Kabuli_Scaffold452*	1881	(A/G)
CakSNP15643	*Kabuli*	*Ca_Kabuli_Scaffold452*	1841	(A/G)
CakSNP15644	*Kabuli*	*Ca_Kabuli_Scaffold452*	1837	(A/C)
CakSNP15645	*Kabuli*	*Ca_Kabuli_Scaffold452*	1836	(A/G)
CakSNP15646	*Kabuli*	*Ca_Kabuli_Scaffold452*	1835	(C/T)
CakSNP15647	*Kabuli*	*Ca_Kabuli_Scaffold453*	303193	(T/C)
CakSNP15648	*Kabuli*	*Ca_Kabuli_Scaffold453*	303201	(G/C)
CakSNP15649	*Kabuli*	*Ca_Kabuli_Scaffold453*	303246	(A/G)
CakSNP15650	*Kabuli*	*Ca_Kabuli_Scaffold453*	303248	(C/T)
CakSNP15651	*Kabuli*	*Ca_Kabuli_Scaffold453*	303259	(C/G)
CakSNP15652	*Kabuli*	*Ca_Kabuli_Scaffold46*	39438	(C/A)
CakSNP15653	*Kabuli*	*Ca_Kabuli_Scaffold46*	39470	(C/T)
CakSNP15654	*Kabuli*	*Ca_Kabuli_Scaffold461*	20652	(C/A)
CakSNP15655	*Kabuli*	*Ca_Kabuli_Scaffold461*	20629	(A/T)
CakSNP15656	*Kabuli*	*Ca_Kabuli_Scaffold461*	20603	(C/T)
CakSNP15657	*Kabuli*	*Ca_Kabuli_Scaffold461*	20599	(C/A)
CakSNP15658	*Kabuli*	*Ca_Kabuli_Scaffold4620*	2699	(T/C)
CakSNP15659	*Kabuli*	*Ca_Kabuli_Scaffold4620*	23442	(T/C)
CakSNP15660	*Kabuli*	*Ca_Kabuli_Scaffold4641*	511	(G/A)
CakSNP15661	*Kabuli*	*Ca_Kabuli_Scaffold4641*	547	(A/C)

**SNP IDs**	**Cultivars**	**Chromosomes/scaffolds**	**Physical positions (bp)**	**SNPs**
CakSNP15662	*Kabuli*	*Ca_Kabuli_Scaffold4641*	553	(C/A)
CakSNP15663	*Kabuli*	*Ca_Kabuli_Scaffold4641*	556	(G/C)
CakSNP15664	*Kabuli*	*Ca_Kabuli_Scaffold4662*	7683	(T/C)
CakSNP15665	*Kabuli*	*Ca_Kabuli_Scaffold4662*	7651	(T/C)
CakSNP15666	*Kabuli*	*Ca_Kabuli_Scaffold4662*	7638	(C/T)
CakSNP15667	*Kabuli*	*Ca_Kabuli_Scaffold4662*	7632	(A/G)
CakSNP15668	*Kabuli*	*Ca_Kabuli_Scaffold4695*	5685	(A/G)
CakSNP15669	*Kabuli*	*Ca_Kabuli_Scaffold470*	7175	(A/G)
CakSNP15670	*Kabuli*	*Ca_Kabuli_Scaffold473*	24996	(G/A)
CakSNP15671	*Kabuli*	*Ca_Kabuli_Scaffold473*	24984	(T/C)
CakSNP15672	*Kabuli*	*Ca_Kabuli_Scaffold473*	24973	(T/C)
CakSNP15673	*Kabuli*	*Ca_Kabuli_Scaffold475*	135286	(C/A)
CakSNP15674	*Kabuli*	*Ca_Kabuli_Scaffold475*	135290	(G/A)
CakSNP15675	*Kabuli*	*Ca_Kabuli_Scaffold477*	51898	(C/T)
CakSNP15676	*Kabuli*	*Ca_Kabuli_Scaffold4777*	10546	(G/T)
CakSNP15677	*Kabuli*	*Ca_Kabuli_Scaffold4777*	16224	(A/T)
CakSNP15678	*Kabuli*	*Ca_Kabuli_Scaffold4777*	16239	(G/T)
CakSNP15679	*Kabuli*	*Ca_Kabuli_Scaffold4777*	55347	(G/T)
CakSNP15680	*Kabuli*	*Ca_Kabuli_Scaffold4777*	72624	(G/A)
CakSNP15681	*Kabuli*	*Ca_Kabuli_Scaffold4777*	76950	(C/A)
CakSNP15682	*Kabuli*	*Ca_Kabuli_Scaffold4777*	106820	(A/G)
CakSNP15683	*Kabuli*	*Ca_Kabuli_Scaffold4777*	112193	(A/C)
CakSNP15684	*Kabuli*	*Ca_Kabuli_Scaffold48*	261719	(A/G)
CakSNP15685	*Kabuli*	*Ca_Kabuli_Scaffold48*	420925	(A/G)
CakSNP15686	*Kabuli*	*Ca_Kabuli_Scaffold480*	67375	(T/C)
CakSNP15687	*Kabuli*	*Ca_Kabuli_Scaffold4836*	5866	(T/A)
CakSNP15688	*Kabuli*	*Ca_Kabuli_Scaffold4836*	5891	(A/T)
CakSNP15689	*Kabuli*	*Ca_Kabuli_Scaffold4836*	5909	(G/A)
CakSNP15690	*Kabuli*	*Ca_Kabuli_Scaffold4836*	5846	(C/T)
CakSNP15691	*Kabuli*	*Ca_Kabuli_Scaffold484*	203947	(C/T)
CakSNP15692	*Kabuli*	*Ca_Kabuli_Scaffold484*	209786	(A/C)
CakSNP15693	*Kabuli*	*Ca_Kabuli_Scaffold484*	243567	(C/T)
CakSNP15694	*Kabuli*	*Ca_Kabuli_Scaffold484*	243575	(T/A)
CakSNP15695	*Kabuli*	*Ca_Kabuli_Scaffold484*	647868	(C/T)
CakSNP15696	*Kabuli*	*Ca_Kabuli_Scaffold484*	647866	(A/G)
CakSNP15697	*Kabuli*	*Ca_Kabuli_Scaffold484*	647809	(A/T)
CakSNP15698	*Kabuli*	*Ca_Kabuli_Scaffold484*	660749	(C/G)
CakSNP15699	*Kabuli*	*Ca_Kabuli_Scaffold485*	103572	(C/T)
CakSNP15700	*Kabuli*	*Ca_Kabuli_Scaffold485*	103546	(A/G)
CakSNP15701	*Kabuli*	*Ca_Kabuli_Scaffold485*	103528	(A/C)
CakSNP15702	*Kabuli*	*Ca_Kabuli_Scaffold485*	123578	(T/C)

SNP IDs	Cultivars	Chromosomes/scaffolds	Physical positions (bp)	SNPs
CakSNP15703	*Kabuli*	*Ca_Kabuli_Scaffold485*	123642	(G/A)
CakSNP15704	*Kabuli*	*Ca_Kabuli_Scaffold495*	128200	(A/T)
CakSNP15705	*Kabuli*	*Ca_Kabuli_Scaffold496*	53652	(G/A)
CakSNP15706	*Kabuli*	*Ca_Kabuli_Scaffold496*	53657	(A/T)
CakSNP15707	*Kabuli*	*Ca_Kabuli_Scaffold496*	53616	(G/T)
CakSNP15708	*Kabuli*	*Ca_Kabuli_Scaffold496*	53587	(A/G)
CakSNP15709	*Kabuli*	*Ca_Kabuli_Scaffold496*	175429	(G/A)
CakSNP15710	*Kabuli*	*Ca_Kabuli_Scaffold496*	175493	(G/A)
CakSNP15711	*Kabuli*	*Ca_Kabuli_Scaffold498*	146772	(A/C)
CakSNP15712	*Kabuli*	*Ca_Kabuli_Scaffold498*	148658	(G/T)
CakSNP15713	*Kabuli*	*Ca_Kabuli_Scaffold498*	164638	(T/C)
CakSNP15714	*Kabuli*	*Ca_Kabuli_Scaffold498*	164615	(G/C)
CakSNP15715	*Kabuli*	*Ca_Kabuli_Scaffold498*	164590	(T/A)
CakSNP15716	*Kabuli*	*Ca_Kabuli_Scaffold50*	153593	(T/A)
CakSNP15717	*Kabuli*	*Ca_Kabuli_Scaffold50*	153599	(C/G)
CakSNP15718	*Kabuli*	*Ca_Kabuli_Scaffold50*	153631	(C/T)
CakSNP15719	*Kabuli*	*Ca_Kabuli_Scaffold50*	153734	(A/C)
CakSNP15720	*Kabuli*	*Ca_Kabuli_Scaffold50*	153851	(T/C)
CakSNP15721	*Kabuli*	*Ca_Kabuli_Scaffold50*	191625	(T/C)
CakSNP15722	*Kabuli*	*Ca_Kabuli_Scaffold50*	201219	(C/G)
CakSNP15723	*Kabuli*	*Ca_Kabuli_Scaffold50*	448866	(G/A)
CakSNP15724	*Kabuli*	*Ca_Kabuli_Scaffold510*	12378	(T/A)
CakSNP15725	*Kabuli*	*Ca_Kabuli_Scaffold510*	12383	(G/T)
CakSNP15726	*Kabuli*	*Ca_Kabuli_Scaffold510*	12394	(C/A)
CakSNP15727	*Kabuli*	*Ca_Kabuli_Scaffold510*	12414	(C/T)
CakSNP15728	*Kabuli*	*Ca_Kabuli_Scaffold510*	12434	(C/T)
CakSNP15729	*Kabuli*	*Ca_Kabuli_Scaffold511*	10450	(T/G)
CakSNP15730	*Kabuli*	*Ca_Kabuli_Scaffold511*	10520	(G/T)
CakSNP15731	*Kabuli*	*Ca_Kabuli_Scaffold511*	173376	(C/A)
CakSNP15732	*Kabuli*	*Ca_Kabuli_Scaffold511*	173377	(A/G)
CakSNP15733	*Kabuli*	*Ca_Kabuli_Scaffold511*	173378	(G/A)
CakSNP15734	*Kabuli*	*Ca_Kabuli_Scaffold513*	7188	(A/C)
CakSNP15735	*Kabuli*	*Ca_Kabuli_Scaffold5163*	2301	(A/G)
CakSNP15736	*Kabuli*	*Ca_Kabuli_Scaffold5185*	1091	(G/A)
CakSNP15737	*Kabuli*	*Ca_Kabuli_Scaffold520*	143359	(C/T)
CakSNP15738	*Kabuli*	*Ca_Kabuli_Scaffold520*	143358	(G/A)
CakSNP15739	*Kabuli*	*Ca_Kabuli_Scaffold520*	143356	(C/G)
CakSNP15740	*Kabuli*	*Ca_Kabuli_Scaffold520*	143342	(G/A)
CakSNP15741	*Kabuli*	*Ca_Kabuli_Scaffold520*	143336	(T/C)
CakSNP15742	*Kabuli*	*Ca_Kabuli_Scaffold520*	143334	(G/A)
CakSNP15743	*Kabuli*	*Ca_Kabuli_Scaffold520*	143333	(A/T)

SNP IDs	Cultivars	Chromosomes/scaffolds	Physical positions (bp)	SNPs
CakSNP15744	*Kabuli*	*Ca_Kabuli_Scaffold520*	143324	(G/A)
CakSNP15745	*Kabuli*	*Ca_Kabuli_Scaffold520*	143317	(T/A)
CakSNP15746	*Kabuli*	*Ca_Kabuli_Scaffold520*	143300	(G/A)
CakSNP15747	*Kabuli*	*Ca_Kabuli_Scaffold520*	143294	(G/A)
CakSNP15748	*Kabuli*	*Ca_Kabuli_Scaffold520*	143291	(G/A)
CakSNP15749	*Kabuli*	*Ca_Kabuli_Scaffold520*	143375	(G/A)
CakSNP15750	*Kabuli*	*Ca_Kabuli_Scaffold520*	143372	(G/A)
CakSNP15751	*Kabuli*	*Ca_Kabuli_Scaffold520*	143355	(C/T)
CakSNP15752	*Kabuli*	*Ca_Kabuli_Scaffold520*	143390	(A/G)
CakSNP15753	*Kabuli*	*Ca_Kabuli_Scaffold520*	143391	(A/C)
CakSNP15754	*Kabuli*	*Ca_Kabuli_Scaffold528*	191764	(A/G)
CakSNP15755	*Kabuli*	*Ca_Kabuli_Scaffold528*	191835	(A/G)
CakSNP15756	*Kabuli*	*Ca_Kabuli_Scaffold528*	232014	(A/C)
CakSNP15757	*Kabuli*	*Ca_Kabuli_Scaffold53*	49273	(A/G)
CakSNP15758	*Kabuli*	*Ca_Kabuli_Scaffold53*	123293	(C/A)
CakSNP15759	*Kabuli*	*Ca_Kabuli_Scaffold531*	2668	(C/T)
CakSNP15760	*Kabuli*	*Ca_Kabuli_Scaffold531*	2674	(C/T)
CakSNP15761	*Kabuli*	*Ca_Kabuli_Scaffold531*	65242	(T/G)
CakSNP15762	*Kabuli*	*Ca_Kabuli_Scaffold531*	101520	(C/G)
CakSNP15763	*Kabuli*	*Ca_Kabuli_Scaffold531*	124019	(T/G)
CakSNP15764	*Kabuli*	*Ca_Kabuli_Scaffold5328*	70	(G/A)
CakSNP15765	*Kabuli*	*Ca_Kabuli_Scaffold5328*	85	(C/T)
CakSNP15766	*Kabuli*	*Ca_Kabuli_Scaffold5328*	86	(C/T)
CakSNP15767	*Kabuli*	*Ca_Kabuli_Scaffold5328*	117	(C/T)
CakSNP15768	*Kabuli*	*Ca_Kabuli_Scaffold535*	156836	(G/A)
CakSNP15769	*Kabuli*	*Ca_Kabuli_Scaffold5358*	2561	(C/A)
CakSNP15770	*Kabuli*	*Ca_Kabuli_Scaffold5358*	3329	(C/A)
CakSNP15771	*Kabuli*	*Ca_Kabuli_Scaffold537*	216656	(A/G)
CakSNP15772	*Kabuli*	*Ca_Kabuli_Scaffold537*	216669	(T/A)
CakSNP15773	*Kabuli*	*Ca_Kabuli_Scaffold537*	216846	(C/T)
CakSNP15774	*Kabuli*	*Ca_Kabuli_Scaffold537*	216829	(C/G)
CakSNP15775	*Kabuli*	*Ca_Kabuli_Scaffold537*	216778	(T/A)
CakSNP15776	*Kabuli*	*Ca_Kabuli_Scaffold537*	223108	(T/C)
CakSNP15777	*Kabuli*	*Ca_Kabuli_Scaffold543*	20780	(T/G)
CakSNP15778	*Kabuli*	*Ca_Kabuli_Scaffold543*	20819	(T/C)
CakSNP15779	*Kabuli*	*Ca_Kabuli_Scaffold543*	20827	(G/T)
CakSNP15780	*Kabuli*	*Ca_Kabuli_Scaffold543*	143169	(C/T)
CakSNP15781	*Kabuli*	*Ca_Kabuli_Scaffold543*	143182	(G/A)
CakSNP15782	*Kabuli*	*Ca_Kabuli_Scaffold543*	143185	(A/G)
CakSNP15783	*Kabuli*	*Ca_Kabuli_Scaffold543*	143191	(G/A)
CakSNP15784	*Kabuli*	*Ca_Kabuli_Scaffold543*	266748	(A/C)

SNP IDs	Cultivars	Chromosomes/scaffolds	Physical positions (bp)	SNPs
CakSNP15785	*Kabuli*	*Ca_Kabuli_Scaffold543*	266729	(A/C)
CakSNP15786	*Kabuli*	*Ca_Kabuli_Scaffold543*	266848	(A/C)
CakSNP15787	*Kabuli*	*Ca_Kabuli_Scaffold545*	17699	(A/C)
CakSNP15788	*Kabuli*	*Ca_Kabuli_Scaffold545*	138848	(T/C)
CakSNP15789	*Kabuli*	*Ca_Kabuli_Scaffold545*	139118	(A/T)
CakSNP15790	*Kabuli*	*Ca_Kabuli_Scaffold545*	146720	(A/G)
CakSNP15791	*Kabuli*	*Ca_Kabuli_Scaffold545*	147549	(G/A)
CakSNP15792	*Kabuli*	*Ca_Kabuli_Scaffold545*	147568	(T/G)
CakSNP15793	*Kabuli*	*Ca_Kabuli_Scaffold545*	226799	(G/A)
CakSNP15794	*Kabuli*	*Ca_Kabuli_Scaffold545*	226769	(G/C)
CakSNP15795	*Kabuli*	*Ca_Kabuli_Scaffold548*	17462	(G/A)
CakSNP15796	*Kabuli*	*Ca_Kabuli_Scaffold548*	231396	(G/A)
CakSNP15797	*Kabuli*	*Ca_Kabuli_Scaffold548*	231338	(G/A)
CakSNP15798	*Kabuli*	*Ca_Kabuli_Scaffold5511*	353	(G/A)
CakSNP15799	*Kabuli*	*Ca_Kabuli_Scaffold5511*	361	(G/A)
CakSNP15800	*Kabuli*	*Ca_Kabuli_Scaffold5511*	366	(G/A)
CakSNP15801	*Kabuli*	*Ca_Kabuli_Scaffold5511*	376	(C/T)
CakSNP15802	*Kabuli*	*Ca_Kabuli_Scaffold5511*	387	(T/C)
CakSNP15803	*Kabuli*	*Ca_Kabuli_Scaffold5511*	388	(G/A)
CakSNP15804	*Kabuli*	*Ca_Kabuli_Scaffold5511*	365	(T/G)
CakSNP15805	*Kabuli*	*Ca_Kabuli_Scaffold5511*	396	(G/A)
CakSNP15806	*Kabuli*	*Ca_Kabuli_Scaffold5511*	458	(G/A)
CakSNP15807	*Kabuli*	*Ca_Kabuli_Scaffold5511*	447	(A/C)
CakSNP15808	*Kabuli*	*Ca_Kabuli_Scaffold5511*	434	(C/T)
CakSNP15809	*Kabuli*	*Ca_Kabuli_Scaffold5511*	404	(T/G)
CakSNP15810	*Kabuli*	*Ca_Kabuli_Scaffold553*	302105	(T/C)
CakSNP15811	*Kabuli*	*Ca_Kabuli_Scaffold553*	302098	(T/C)
CakSNP15812	*Kabuli*	*Ca_Kabuli_Scaffold553*	310715	(A/G)
CakSNP15813	*Kabuli*	*Ca_Kabuli_Scaffold553*	310759	(T/A)
CakSNP15814	*Kabuli*	*Ca_Kabuli_Scaffold553*	402702	(T/C)
CakSNP15815	*Kabuli*	*Ca_Kabuli_Scaffold553*	441845	(T/C)
CakSNP15816	*Kabuli*	*Ca_Kabuli_Scaffold553*	441881	(T/C)
CakSNP15817	*Kabuli*	*Ca_Kabuli_Scaffold553*	441898	(G/A)
CakSNP15818	*Kabuli*	*Ca_Kabuli_Scaffold553*	550789	(G/C)
CakSNP15819	*Kabuli*	*Ca_Kabuli_Scaffold553*	607503	(G/T)
CakSNP15820	*Kabuli*	*Ca_Kabuli_Scaffold553*	607572	(T/C)
CakSNP15821	*Kabuli*	*Ca_Kabuli_Scaffold553*	794383	(A/G)
CakSNP15822	*Kabuli*	*Ca_Kabuli_Scaffold553*	794404	(G/A)
CakSNP15823	*Kabuli*	*Ca_Kabuli_Scaffold553*	794431	(T/C)
CakSNP15824	*Kabuli*	*Ca_Kabuli_Scaffold553*	794439	(G/T)
CakSNP15825	*Kabuli*	*Ca_Kabuli_Scaffold561*	46046	(T/G)

SNP IDs	Cultivars	Chromosomes/scaffolds	Physical positions (bp)	SNPs
CakSNP15826	*Kabuli*	*Ca_Kabuli_Scaffold561*	49174	(G/A)
CakSNP15827	*Kabuli*	*Ca_Kabuli_Scaffold561*	95414	(A/C)
CakSNP15828	*Kabuli*	*Ca_Kabuli_Scaffold561*	95386	(G/A)
CakSNP15829	*Kabuli*	*Ca_Kabuli_Scaffold562*	221706	(T/C)
CakSNP15830	*Kabuli*	*Ca_Kabuli_Scaffold562*	221696	(T/G)
CakSNP15831	*Kabuli*	*Ca_Kabuli_Scaffold562*	221686	(C/A)
CakSNP15832	*Kabuli*	*Ca_Kabuli_Scaffold562*	221713	(G/A)
CakSNP15833	*Kabuli*	*Ca_Kabuli_Scaffold562*	221714	(C/T)
CakSNP15834	*Kabuli*	*Ca_Kabuli_Scaffold562*	221735	(G/A)
CakSNP15835	*Kabuli*	*Ca_Kabuli_Scaffold562*	221795	(C/T)
CakSNP15836	*Kabuli*	*Ca_Kabuli_Scaffold562*	221748	(C/A)
CakSNP15837	*Kabuli*	*Ca_Kabuli_Scaffold562*	221758	(G/A)
CakSNP15838	*Kabuli*	*Ca_Kabuli_Scaffold562*	221769	(T/A)
CakSNP15839	*Kabuli*	*Ca_Kabuli_Scaffold562*	221778	(G/A)
CakSNP15840	*Kabuli*	*Ca_Kabuli_Scaffold562*	221779	(G/A)
CakSNP15841	*Kabuli*	*Ca_Kabuli_Scaffold562*	221788	(G/A)
CakSNP15842	*Kabuli*	*Ca_Kabuli_Scaffold562*	221828	(T/A)
CakSNP15843	*Kabuli*	*Ca_Kabuli_Scaffold562*	221792	(C/A)
CakSNP15844	*Kabuli*	*Ca_Kabuli_Scaffold562*	255362	(G/A)
CakSNP15845	*Kabuli*	*Ca_Kabuli_Scaffold562*	255295	(G/A)
CakSNP15846	*Kabuli*	*Ca_Kabuli_Scaffold562*	289080	(C/A)
CakSNP15847	*Kabuli*	*Ca_Kabuli_Scaffold562*	290071	(C/T)
CakSNP15848	*Kabuli*	*Ca_Kabuli_Scaffold5714*	24882	(A/C)
CakSNP15849	*Kabuli*	*Ca_Kabuli_Scaffold5714*	36359	(G/C)
CakSNP15850	*Kabuli*	*Ca_Kabuli_Scaffold5730*	239	(G/A)
CakSNP15851	*Kabuli*	*Ca_Kabuli_Scaffold5730*	192	(A/G)
CakSNP15852	*Kabuli*	*Ca_Kabuli_Scaffold5730*	227	(G/A)
CakSNP15853	*Kabuli*	*Ca_Kabuli_Scaffold575*	36084	(T/C)
CakSNP15854	*Kabuli*	*Ca_Kabuli_Scaffold575*	36135	(T/C)
CakSNP15855	*Kabuli*	*Ca_Kabuli_Scaffold575*	36238	(C/T)
CakSNP15856	*Kabuli*	*Ca_Kabuli_Scaffold575*	45254	(C/T)
CakSNP15857	*Kabuli*	*Ca_Kabuli_Scaffold575*	45250	(T/C)
CakSNP15858	*Kabuli*	*Ca_Kabuli_Scaffold575*	45239	(G/T)
CakSNP15859	*Kabuli*	*Ca_Kabuli_Scaffold575*	45236	(G/A)
CakSNP15860	*Kabuli*	*Ca_Kabuli_Scaffold575*	45233	(G/A)
CakSNP15861	*Kabuli*	*Ca_Kabuli_Scaffold575*	45215	(C/A)
CakSNP15862	*Kabuli*	*Ca_Kabuli_Scaffold575*	45192	(C/T)
CakSNP15863	*Kabuli*	*Ca_Kabuli_Scaffold575*	45279	(G/C)
CakSNP15864	*Kabuli*	*Ca_Kabuli_Scaffold575*	45245	(G/A)
CakSNP15865	*Kabuli*	*Ca_Kabuli_Scaffold575*	45237	(C/T)
CakSNP15866	*Kabuli*	*Ca_Kabuli_Scaffold575*	45234	(C/T)

SNP IDs	Cultivars	Chromosomes/scaffolds	Physical positions (bp)	SNPs
CakSNP15867	*Kabuli*	*Ca_Kabuli_Scaffold575*	110772	(G/T)
CakSNP15868	*Kabuli*	*Ca_Kabuli_Scaffold575*	110785	(C/T)
CakSNP15869	*Kabuli*	*Ca_Kabuli_Scaffold575*	110765	(C/T)
CakSNP15870	*Kabuli*	*Ca_Kabuli_Scaffold575*	110759	(C/T)
CakSNP15871	*Kabuli*	*Ca_Kabuli_Scaffold575*	110753	(G/A)
CakSNP15872	*Kabuli*	*Ca_Kabuli_Scaffold575*	117287	(T/G)
CakSNP15873	*Kabuli*	*Ca_Kabuli_Scaffold5792*	65166	(A/C)
CakSNP15874	*Kabuli*	*Ca_Kabuli_Scaffold5792*	86779	(C/A)
CakSNP15875	*Kabuli*	*Ca_Kabuli_Scaffold5792*	86716	(T/C)
CakSNP15876	*Kabuli*	*Ca_Kabuli_Scaffold584*	53203	(G/T)
CakSNP15877	*Kabuli*	*Ca_Kabuli_Scaffold589*	72891	(T/G)
CakSNP15878	*Kabuli*	*Ca_Kabuli_Scaffold590*	88429	(A/T)
CakSNP15879	*Kabuli*	*Ca_Kabuli_Scaffold590*	88420	(T/C)
CakSNP15880	*Kabuli*	*Ca_Kabuli_Scaffold598*	300825	(A/C)
CakSNP15881	*Kabuli*	*Ca_Kabuli_Scaffold598*	300940	(C/T)
CakSNP15882	*Kabuli*	*Ca_Kabuli_Scaffold598*	404254	(G/C)
CakSNP15883	*Kabuli*	*Ca_Kabuli_Scaffold598*	404275	(G/T)
CakSNP15884	*Kabuli*	*Ca_Kabuli_Scaffold599*	349605	(T/G)
CakSNP15885	*Kabuli*	*Ca_Kabuli_Scaffold5997*	742	(G/C)
CakSNP15886	*Kabuli*	*Ca_Kabuli_Scaffold5997*	679	(C/T)
CakSNP15887	*Kabuli*	*Ca_Kabuli_Scaffold601*	55483	(A/G)
CakSNP15888	*Kabuli*	*Ca_Kabuli_Scaffold601*	71998	(T/A)
CakSNP15889	*Kabuli*	*Ca_Kabuli_Scaffold605*	23769	(A/C)
CakSNP15890	*Kabuli*	*Ca_Kabuli_Scaffold605*	23773	(T/C)
CakSNP15891	*Kabuli*	*Ca_Kabuli_Scaffold605*	23774	(A/G)
CakSNP15892	*Kabuli*	*Ca_Kabuli_Scaffold605*	23775	(A/G)
CakSNP15893	*Kabuli*	*Ca_Kabuli_Scaffold605*	23793	(A/G)
CakSNP15894	*Kabuli*	*Ca_Kabuli_Scaffold605*	23795	(T/A)
CakSNP15895	*Kabuli*	*Ca_Kabuli_Scaffold605*	23803	(A/G)
CakSNP15896	*Kabuli*	*Ca_Kabuli_Scaffold605*	23808	(C/G)
CakSNP15897	*Kabuli*	*Ca_Kabuli_Scaffold605*	23811	(G/A)
CakSNP15898	*Kabuli*	*Ca_Kabuli_Scaffold605*	23816	(T/A)
CakSNP15899	*Kabuli*	*Ca_Kabuli_Scaffold605*	84761	(A/C)
CakSNP15900	*Kabuli*	*Ca_Kabuli_Scaffold605*	84765	(T/C)
CakSNP15901	*Kabuli*	*Ca_Kabuli_Scaffold605*	84766	(A/G)
CakSNP15902	*Kabuli*	*Ca_Kabuli_Scaffold605*	84767	(A/G)
CakSNP15903	*Kabuli*	*Ca_Kabuli_Scaffold605*	84785	(A/G)
CakSNP15904	*Kabuli*	*Ca_Kabuli_Scaffold605*	84787	(T/A)
CakSNP15905	*Kabuli*	*Ca_Kabuli_Scaffold605*	84795	(A/G)
CakSNP15906	*Kabuli*	*Ca_Kabuli_Scaffold605*	84800	(C/G)
CakSNP15907	*Kabuli*	*Ca_Kabuli_Scaffold605*	84803	(G/A)

**SNP IDs**	**Cultivars**	**Chromosomes/scaffolds**	**Physical positions (bp)**	**SNPs**
CakSNP15908	*Kabuli*	*Ca_Kabuli_Scaffold605*	84807	(A/G)
CakSNP15909	*Kabuli*	*Ca_Kabuli_Scaffold62*	46079	(C/T)
CakSNP15910	*Kabuli*	*Ca_Kabuli_Scaffold62*	46085	(C/T)
CakSNP15911	*Kabuli*	*Ca_Kabuli_Scaffold62*	46087	(G/A)
CakSNP15912	*Kabuli*	*Ca_Kabuli_Scaffold62*	46088	(C/T)
CakSNP15913	*Kabuli*	*Ca_Kabuli_Scaffold624*	254901	(C/T)
CakSNP15914	*Kabuli*	*Ca_Kabuli_Scaffold624*	254950	(G/A)
CakSNP15915	*Kabuli*	*Ca_Kabuli_Scaffold624*	254959	(T/C)
CakSNP15916	*Kabuli*	*Ca_Kabuli_Scaffold624*	254971	(G/A)
CakSNP15917	*Kabuli*	*Ca_Kabuli_Scaffold624*	254992	(C/T)
CakSNP15918	*Kabuli*	*Ca_Kabuli_Scaffold624*	254942	(A/T)
CakSNP15919	*Kabuli*	*Ca_Kabuli_Scaffold6339*	190	(T/G)
CakSNP15920	*Kabuli*	*Ca_Kabuli_Scaffold6367*	6825	(G/T)
CakSNP15921	*Kabuli*	*Ca_Kabuli_Scaffold6367*	10856	(T/G)
CakSNP15922	*Kabuli*	*Ca_Kabuli_Scaffold6367*	10842	(T/A)
CakSNP15923	*Kabuli*	*Ca_Kabuli_Scaffold6367*	13651	(G/A)
CakSNP15924	*Kabuli*	*Ca_Kabuli_Scaffold6367*	23667	(G/A)
CakSNP15925	*Kabuli*	*Ca_Kabuli_Scaffold6367*	25674	(T/C)
CakSNP15926	*Kabuli*	*Ca_Kabuli_Scaffold637*	107671	(G/C)
CakSNP15927	*Kabuli*	*Ca_Kabuli_Scaffold637*	107661	(A/C)
CakSNP15928	*Kabuli*	*Ca_Kabuli_Scaffold637*	107636	(T/C)
CakSNP15929	*Kabuli*	*Ca_Kabuli_Scaffold637*	107613	(C/G)
CakSNP15930	*Kabuli*	*Ca_Kabuli_Scaffold6403*	1553	(T/A)
CakSNP15931	*Kabuli*	*Ca_Kabuli_Scaffold642*	28715	(A/T)
CakSNP15932	*Kabuli*	*Ca_Kabuli_Scaffold642*	615176	(T/C)
CakSNP15933	*Kabuli*	*Ca_Kabuli_Scaffold645*	91908	(C/T)
CakSNP15934	*Kabuli*	*Ca_Kabuli_Scaffold653*	145231	(A/G)
CakSNP15935	*Kabuli*	*Ca_Kabuli_Scaffold653*	183054	(T/C)
CakSNP15936	*Kabuli*	*Ca_Kabuli_Scaffold653*	183063	(G/A)
CakSNP15937	*Kabuli*	*Ca_Kabuli_Scaffold653*	183069	(C/A)
CakSNP15938	*Kabuli*	*Ca_Kabuli_Scaffold653*	183074	(C/G)
CakSNP15939	*Kabuli*	*Ca_Kabuli_Scaffold653*	183078	(T/G)
CakSNP15940	*Kabuli*	*Ca_Kabuli_Scaffold653*	183091	(T/C)
CakSNP15941	*Kabuli*	*Ca_Kabuli_Scaffold653*	183143	(T/A)
CakSNP15942	*Kabuli*	*Ca_Kabuli_Scaffold653*	183135	(T/C)
CakSNP15943	*Kabuli*	*Ca_Kabuli_Scaffold653*	183112	(T/C)
CakSNP15944	*Kabuli*	*Ca_Kabuli_Scaffold653*	189129	(C/T)
CakSNP15945	*Kabuli*	*Ca_Kabuli_Scaffold653*	197878	(A/T)
CakSNP15946	*Kabuli*	*Ca_Kabuli_Scaffold661*	180364	(G/T)
CakSNP15947	*Kabuli*	*Ca_Kabuli_Scaffold661*	180362	(A/C)
CakSNP15948	*Kabuli*	*Ca_Kabuli_Scaffold661*	180348	(C/T)

SNP IDs	Cultivars	Chromosomes/scaffolds	Physical positions (bp)	SNPs
CakSNP15949	*Kabuli*	*Ca_Kabuli_Scaffold661*	180302	(G/T)
CakSNP15950	*Kabuli*	*Ca_Kabuli_Scaffold661*	311222	(G/T)
CakSNP15951	*Kabuli*	*Ca_Kabuli_Scaffold661*	311182	(C/T)
CakSNP15952	*Kabuli*	*Ca_Kabuli_Scaffold663*	16068	(G/A)
CakSNP15953	*Kabuli*	*Ca_Kabuli_Scaffold663*	16042	(C/A)
CakSNP15954	*Kabuli*	*Ca_Kabuli_Scaffold663*	16029	(G/A)
CakSNP15955	*Kabuli*	*Ca_Kabuli_Scaffold663*	16056	(C/T)
CakSNP15956	*Kabuli*	*Ca_Kabuli_Scaffold663*	16059	(G/A)
CakSNP15957	*Kabuli*	*Ca_Kabuli_Scaffold663*	16061	(A/G)
CakSNP15958	*Kabuli*	*Ca_Kabuli_Scaffold663*	156613	(G/C)
CakSNP15959	*Kabuli*	*Ca_Kabuli_Scaffold674*	91979	(T/A)
CakSNP15960	*Kabuli*	*Ca_Kabuli_Scaffold674*	360388	(C/T)
CakSNP15961	*Kabuli*	*Ca_Kabuli_Scaffold674*	360401	(A/G)
CakSNP15962	*Kabuli*	*Ca_Kabuli_Scaffold674*	360421	(G/A)
CakSNP15963	*Kabuli*	*Ca_Kabuli_Scaffold674*	360406	(C/T)
CakSNP15964	*Kabuli*	*Ca_Kabuli_Scaffold674*	360349	(G/A)
CakSNP15965	*Kabuli*	*Ca_Kabuli_Scaffold674*	360464	(G/A)
CakSNP15966	*Kabuli*	*Ca_Kabuli_Scaffold674*	360457	(G/C)
CakSNP15967	*Kabuli*	*Ca_Kabuli_Scaffold674*	360411	(G/A)
CakSNP15968	*Kabuli*	*Ca_Kabuli_Scaffold674*	360408	(G/T)
CakSNP15969	*Kabuli*	*Ca_Kabuli_Scaffold674*	608623	(T/G)
CakSNP15970	*Kabuli*	*Ca_Kabuli_Scaffold674*	652382	(G/A)
CakSNP15971	*Kabuli*	*Ca_Kabuli_Scaffold674*	652373	(A/G)
CakSNP15972	*Kabuli*	*Ca_Kabuli_Scaffold674*	826136	(T/C)
CakSNP15973	*Kabuli*	*Ca_Kabuli_Scaffold681*	18376	(C/G)
CakSNP15974	*Kabuli*	*Ca_Kabuli_Scaffold682*	157238	(G/C)
CakSNP15975	*Kabuli*	*Ca_Kabuli_Scaffold682*	578200	(T/G)
CakSNP15976	*Kabuli*	*Ca_Kabuli_Scaffold684*	78894	(G/A)
CakSNP15977	*Kabuli*	*Ca_Kabuli_Scaffold684*	78883	(T/G)
CakSNP15978	*Kabuli*	*Ca_Kabuli_Scaffold684*	78871	(C/G)
CakSNP15979	*Kabuli*	*Ca_Kabuli_Scaffold684*	78842	(G/A)
CakSNP15980	*Kabuli*	*Ca_Kabuli_Scaffold684*	78844	(A/G)
CakSNP15981	*Kabuli*	*Ca_Kabuli_Scaffold684*	78864	(C/T)
CakSNP15982	*Kabuli*	*Ca_Kabuli_Scaffold684*	78893	(C/T)
CakSNP15983	*Kabuli*	*Ca_Kabuli_Scaffold686*	206111	(G/A)
CakSNP15984	*Kabuli*	*Ca_Kabuli_Scaffold686*	206044	(T/C)
CakSNP15985	*Kabuli*	*Ca_Kabuli_Scaffold686*	206042	(A/G)
CakSNP15986	*Kabuli*	*Ca_Kabuli_Scaffold702*	247644	(G/C)
CakSNP15987	*Kabuli*	*Ca_Kabuli_Scaffold711*	153694	(C/T)
CakSNP15988	*Kabuli*	*Ca_Kabuli_Scaffold711*	153680	(C/A)
CakSNP15989	*Kabuli*	*Ca_Kabuli_Scaffold711*	153667	(G/A)

SNP IDs	Cultivars	Chromosomes/scaffolds	Physical positions (bp)	SNPs
CakSNP15990	*Kabuli*	*Ca_Kabuli_Scaffold711*	153697	(G/A)
CakSNP15991	*Kabuli*	*Ca_Kabuli_Scaffold711*	153699	(A/G)
CakSNP15992	*Kabuli*	*Ca_Kabuli_Scaffold711*	153706	(G/A)
CakSNP15993	*Kabuli*	*Ca_Kabuli_Scaffold7127*	1878	(C/T)
CakSNP15994	*Kabuli*	*Ca_Kabuli_Scaffold7144*	234	(A/T)
CakSNP15995	*Kabuli*	*Ca_Kabuli_Scaffold716*	128208	(G/T)
CakSNP15996	*Kabuli*	*Ca_Kabuli_Scaffold716*	212326	(G/A)
CakSNP15997	*Kabuli*	*Ca_Kabuli_Scaffold716*	212374	(G/A)
CakSNP15998	*Kabuli*	*Ca_Kabuli_Scaffold716*	269220	(T/C)
CakSNP15999	*Kabuli*	*Ca_Kabuli_Scaffold716*	269230	(A/C)
CakSNP16000	*Kabuli*	*Ca_Kabuli_Scaffold716*	269242	(A/G)
CakSNP16001	*Kabuli*	*Ca_Kabuli_Scaffold716*	269266	(G/T)
CakSNP16002	*Kabuli*	*Ca_Kabuli_Scaffold716*	269271	(G/A)
CakSNP16003	*Kabuli*	*Ca_Kabuli_Scaffold716*	269281	(T/G)
CakSNP16004	*Kabuli*	*Ca_Kabuli_Scaffold716*	269287	(G/A)
CakSNP16005	*Kabuli*	*Ca_Kabuli_Scaffold716*	269335	(G/A)
CakSNP16006	*Kabuli*	*Ca_Kabuli_Scaffold716*	269332	(C/T)
CakSNP16007	*Kabuli*	*Ca_Kabuli_Scaffold716*	269314	(G/A)
CakSNP16008	*Kabuli*	*Ca_Kabuli_Scaffold716*	269299	(T/C)
CakSNP16009	*Kabuli*	*Ca_Kabuli_Scaffold719*	11850	(C/T)
CakSNP16010	*Kabuli*	*Ca_Kabuli_Scaffold720*	60880	(G/A)
CakSNP16011	*Kabuli*	*Ca_Kabuli_Scaffold720*	370624	(T/G)
CakSNP16012	*Kabuli*	*Ca_Kabuli_Scaffold720*	370639	(T/A)
CakSNP16013	*Kabuli*	*Ca_Kabuli_Scaffold720*	370725	(G/A)
CakSNP16014	*Kabuli*	*Ca_Kabuli_Scaffold724*	25019	(G/A)
CakSNP16015	*Kabuli*	*Ca_Kabuli_Scaffold724*	25722	(G/C)
CakSNP16016	*Kabuli*	*Ca_Kabuli_Scaffold731*	315093	(G/A)
CakSNP16017	*Kabuli*	*Ca_Kabuli_Scaffold731*	315114	(C/T)
CakSNP16018	*Kabuli*	*Ca_Kabuli_Scaffold731*	315135	(G/T)
CakSNP16019	*Kabuli*	*Ca_Kabuli_Scaffold731*	315138	(C/T)
CakSNP16020	*Kabuli*	*Ca_Kabuli_Scaffold731*	315141	(G/A)
CakSNP16021	*Kabuli*	*Ca_Kabuli_Scaffold731*	332497	(G/A)
CakSNP16022	*Kabuli*	*Ca_Kabuli_Scaffold731*	332468	(G/T)
CakSNP16023	*Kabuli*	*Ca_Kabuli_Scaffold731*	332467	(G/C)
CakSNP16024	*Kabuli*	*Ca_Kabuli_Scaffold731*	332465	(T/A)
CakSNP16025	*Kabuli*	*Ca_Kabuli_Scaffold731*	332464	(G/T)
CakSNP16026	*Kabuli*	*Ca_Kabuli_Scaffold731*	332458	(A/T)
CakSNP16027	*Kabuli*	*Ca_Kabuli_Scaffold731*	332528	(G/C)
CakSNP16028	*Kabuli*	*Ca_Kabuli_Scaffold731*	357674	(G/A)
CakSNP16029	*Kabuli*	*Ca_Kabuli_Scaffold731*	357645	(G/T)
CakSNP16030	*Kabuli*	*Ca_Kabuli_Scaffold731*	357644	(G/C)

**SNP IDs**	**Cultivars**	**Chromosomes/scaffolds**	**Physical positions (bp)**	**SNPs**
CakSNP16031	*Kabuli*	*Ca_Kabuli_Scaffold731*	357642	(T/A)
CakSNP16032	*Kabuli*	*Ca_Kabuli_Scaffold731*	357641	(G/T)
CakSNP16033	*Kabuli*	*Ca_Kabuli_Scaffold731*	357635	(A/T)
CakSNP16034	*Kabuli*	*Ca_Kabuli_Scaffold731*	357715	(A/T)
CakSNP16035	*Kabuli*	*Ca_Kabuli_Scaffold731*	362123	(G/A)
CakSNP16036	*Kabuli*	*Ca_Kabuli_Scaffold731*	362118	(C/A)
CakSNP16037	*Kabuli*	*Ca_Kabuli_Scaffold731*	362114	(A/C)
CakSNP16038	*Kabuli*	*Ca_Kabuli_Scaffold731*	362548	(T/A)
CakSNP16039	*Kabuli*	*Ca_Kabuli_Scaffold731*	362553	(C/T)
CakSNP16040	*Kabuli*	*Ca_Kabuli_Scaffold731*	362554	(T/A)
CakSNP16041	*Kabuli*	*Ca_Kabuli_Scaffold731*	362555	(T/G)
CakSNP16042	*Kabuli*	*Ca_Kabuli_Scaffold731*	362556	(C/A)
CakSNP16043	*Kabuli*	*Ca_Kabuli_Scaffold731*	362574	(T/C)
CakSNP16044	*Kabuli*	*Ca_Kabuli_Scaffold731*	362581	(A/G)
CakSNP16045	*Kabuli*	*Ca_Kabuli_Scaffold731*	362582	(T/C)
CakSNP16046	*Kabuli*	*Ca_Kabuli_Scaffold731*	362594	(G/T)
CakSNP16047	*Kabuli*	*Ca_Kabuli_Scaffold731*	362610	(C/T)
CakSNP16048	*Kabuli*	*Ca_Kabuli_Scaffold731*	372255	(G/A)
CakSNP16049	*Kabuli*	*Ca_Kabuli_Scaffold731*	372253	(G/T)
CakSNP16050	*Kabuli*	*Ca_Kabuli_Scaffold731*	372250	(G/A)
CakSNP16051	*Kabuli*	*Ca_Kabuli_Scaffold731*	372244	(C/T)
CakSNP16052	*Kabuli*	*Ca_Kabuli_Scaffold731*	372243	(A/T)
CakSNP16053	*Kabuli*	*Ca_Kabuli_Scaffold731*	372215	(A/G)
CakSNP16054	*Kabuli*	*Ca_Kabuli_Scaffold731*	372209	(G/C)
CakSNP16055	*Kabuli*	*Ca_Kabuli_Scaffold731*	372183	(C/T)
CakSNP16056	*Kabuli*	*Ca_Kabuli_Scaffold731*	372959	(A/C)
CakSNP16057	*Kabuli*	*Ca_Kabuli_Scaffold7355*	1606	(C/G)
CakSNP16058	*Kabuli*	*Ca_Kabuli_Scaffold7355*	1690	(T/C)
CakSNP16059	*Kabuli*	*Ca_Kabuli_Scaffold7355*	3608	(A/G)
CakSNP16060	*Kabuli*	*Ca_Kabuli_Scaffold7355*	3602	(T/C)
CakSNP16061	*Kabuli*	*Ca_Kabuli_Scaffold7355*	3578	(C/T)
CakSNP16062	*Kabuli*	*Ca_Kabuli_Scaffold7355*	3572	(A/G)
CakSNP16063	*Kabuli*	*Ca_Kabuli_Scaffold7355*	3566	(C/T)
CakSNP16064	*Kabuli*	*Ca_Kabuli_Scaffold752*	382375	(G/A)
CakSNP16065	*Kabuli*	*Ca_Kabuli_Scaffold752*	497452	(T/A)
CakSNP16066	*Kabuli*	*Ca_Kabuli_Scaffold752*	497454	(A/G)
CakSNP16067	*Kabuli*	*Ca_Kabuli_Scaffold753*	8312	(A/G)
CakSNP16068	*Kabuli*	*Ca_Kabuli_Scaffold758*	16480	(G/T)
CakSNP16069	*Kabuli*	*Ca_Kabuli_Scaffold7650*	2686	(C/T)
CakSNP16070	*Kabuli*	*Ca_Kabuli_Scaffold7715*	3434	(A/T)
CakSNP16071	*Kabuli*	*Ca_Kabuli_Scaffold772*	53192	(C/T)

SNP IDs	Cultivars	Chromosomes/scaffolds	Physical positions (bp)	SNPs
CakSNP16072	*Kabuli*	*Ca_Kabuli_Scaffold772*	53196	(C/T)
CakSNP16073	*Kabuli*	*Ca_Kabuli_Scaffold775*	89238	(T/C)
CakSNP16074	*Kabuli*	*Ca_Kabuli_Scaffold775*	89239	(C/T)
CakSNP16075	*Kabuli*	*Ca_Kabuli_Scaffold775*	279828	(A/G)
CakSNP16076	*Kabuli*	*Ca_Kabuli_Scaffold775*	356107	(C/A)
CakSNP16077	*Kabuli*	*Ca_Kabuli_Scaffold775*	448604	(G/A)
CakSNP16078	*Kabuli*	*Ca_Kabuli_Scaffold775*	448743	(T/C)
CakSNP16079	*Kabuli*	*Ca_Kabuli_Scaffold776*	188906	(C/T)
CakSNP16080	*Kabuli*	*Ca_Kabuli_Scaffold776*	188895	(C/T)
CakSNP16081	*Kabuli*	*Ca_Kabuli_Scaffold776*	188888	(G/A)
CakSNP16082	*Kabuli*	*Ca_Kabuli_Scaffold7765*	503	(C/T)
CakSNP16083	*Kabuli*	*Ca_Kabuli_Scaffold7765*	536	(C/A)
CakSNP16084	*Kabuli*	*Ca_Kabuli_Scaffold7765*	549	(G/A)
CakSNP16085	*Kabuli*	*Ca_Kabuli_Scaffold783*	201617	(T/C)
CakSNP16086	*Kabuli*	*Ca_Kabuli_Scaffold783*	244209	(T/C)
CakSNP16087	*Kabuli*	*Ca_Kabuli_Scaffold7842*	727	(C/T)
CakSNP16088	*Kabuli*	*Ca_Kabuli_Scaffold7842*	1078	(G/A)
CakSNP16089	*Kabuli*	*Ca_Kabuli_Scaffold7842*	1075	(C/T)
CakSNP16090	*Kabuli*	*Ca_Kabuli_Scaffold7842*	1068	(C/A)
CakSNP16091	*Kabuli*	*Ca_Kabuli_Scaffold7842*	1042	(T/C)
CakSNP16092	*Kabuli*	*Ca_Kabuli_Scaffold7842*	1024	(T/G)
CakSNP16093	*Kabuli*	*Ca_Kabuli_Scaffold787*	4363	(A/G)
CakSNP16094	*Kabuli*	*Ca_Kabuli_Scaffold794*	50197	(T/G)
CakSNP16095	*Kabuli*	*Ca_Kabuli_Scaffold794*	50201	(T/G)
CakSNP16096	*Kabuli*	*Ca_Kabuli_Scaffold794*	50225	(C/G)
CakSNP16097	*Kabuli*	*Ca_Kabuli_Scaffold794*	50239	(G/A)
CakSNP16098	*Kabuli*	*Ca_Kabuli_Scaffold794*	50240	(C/A)
CakSNP16099	*Kabuli*	*Ca_Kabuli_Scaffold794*	50236	(A/G)
CakSNP16100	*Kabuli*	*Ca_Kabuli_Scaffold799*	3956	(G/A)
CakSNP16101	*Kabuli*	*Ca_Kabuli_Scaffold799*	3930	(A/G)
CakSNP16102	*Kabuli*	*Ca_Kabuli_Scaffold799*	3928	(C/T)
CakSNP16103	*Kabuli*	*Ca_Kabuli_Scaffold801*	67115	(C/T)
CakSNP16104	*Kabuli*	*Ca_Kabuli_Scaffold806*	129263	(A/G)
CakSNP16105	*Kabuli*	*Ca_Kabuli_Scaffold806*	129265	(G/T)
CakSNP16106	*Kabuli*	*Ca_Kabuli_Scaffold809*	48678	(T/C)
CakSNP16107	*Kabuli*	*Ca_Kabuli_Scaffold809*	48650	(C/A)
CakSNP16108	*Kabuli*	*Ca_Kabuli_Scaffold809*	53417	(C/G)
CakSNP16109	*Kabuli*	*Ca_Kabuli_Scaffold809*	53425	(T/C)
CakSNP16110	*Kabuli*	*Ca_Kabuli_Scaffold809*	53431	(G/A)
CakSNP16111	*Kabuli*	*Ca_Kabuli_Scaffold809*	53444	(G/A)
CakSNP16112	*Kabuli*	*Ca_Kabuli_Scaffold809*	53474	(G/A)

SNP IDs	Cultivars	Chromosomes/scaffolds	Physical positions (bp)	SNPs
CakSNP16113	*Kabuli*	*Ca_Kabuli_Scaffold809*	53464	(G/A)
CakSNP16114	*Kabuli*	*Ca_Kabuli_Scaffold809*	53470	(C/G)
CakSNP16115	*Kabuli*	*Ca_Kabuli_Scaffold811*	81373	(C/T)
CakSNP16116	*Kabuli*	*Ca_Kabuli_Scaffold812*	34686	(C/T)
CakSNP16117	*Kabuli*	*Ca_Kabuli_Scaffold812*	246208	(A/C)
CakSNP16118	*Kabuli*	*Ca_Kabuli_Scaffold812*	332746	(T/C)
CakSNP16119	*Kabuli*	*Ca_Kabuli_Scaffold812*	332758	(T/C)
CakSNP16120	*Kabuli*	*Ca_Kabuli_Scaffold812*	528815	(C/G)
CakSNP16121	*Kabuli*	*Ca_Kabuli_Scaffold8226*	21624	(G/A)
CakSNP16122	*Kabuli*	*Ca_Kabuli_Scaffold8226*	21592	(T/G)
CakSNP16123	*Kabuli*	*Ca_Kabuli_Scaffold8226*	21590	(T/C)
CakSNP16124	*Kabuli*	*Ca_Kabuli_Scaffold8226*	21572	(T/A)
CakSNP16125	*Kabuli*	*Ca_Kabuli_Scaffold842*	13898	(T/C)
CakSNP16126	*Kabuli*	*Ca_Kabuli_Scaffold842*	13905	(C/G)
CakSNP16127	*Kabuli*	*Ca_Kabuli_Scaffold842*	13928	(G/T)
CakSNP16128	*Kabuli*	*Ca_Kabuli_Scaffold842*	13946	(T/C)
CakSNP16129	*Kabuli*	*Ca_Kabuli_Scaffold845*	28172	(G/T)
CakSNP16130	*Kabuli*	*Ca_Kabuli_Scaffold845*	45557	(A/G)
CakSNP16131	*Kabuli*	*Ca_Kabuli_Scaffold845*	71041	(A/G)
CakSNP16132	*Kabuli*	*Ca_Kabuli_Scaffold845*	102612	(A/G)
CakSNP16133	*Kabuli*	*Ca_Kabuli_Scaffold845*	129326	(C/G)
CakSNP16134	*Kabuli*	*Ca_Kabuli_Scaffold845*	129370	(A/G)
CakSNP16135	*Kabuli*	*Ca_Kabuli_Scaffold845*	130569	(G/C)
CakSNP16136	*Kabuli*	*Ca_Kabuli_Scaffold845*	131911	(A/G)
CakSNP16137	*Kabuli*	*Ca_Kabuli_Scaffold845*	136796	(A/G)
CakSNP16138	*Kabuli*	*Ca_Kabuli_Scaffold845*	139268	(G/A)
CakSNP16139	*Kabuli*	*Ca_Kabuli_Scaffold845*	154556	(T/C)
CakSNP16140	*Kabuli*	*Ca_Kabuli_Scaffold845*	205934	(T/C)
CakSNP16141	*Kabuli*	*Ca_Kabuli_Scaffold848*	37282	(G/A)
CakSNP16142	*Kabuli*	*Ca_Kabuli_Scaffold848*	37309	(G/A)
CakSNP16143	*Kabuli*	*Ca_Kabuli_Scaffold848*	39934	(T/G)
CakSNP16144	*Kabuli*	*Ca_Kabuli_Scaffold848*	39968	(C/T)
CakSNP16145	*Kabuli*	*Ca_Kabuli_Scaffold848*	39984	(G/T)
CakSNP16146	*Kabuli*	*Ca_Kabuli_Scaffold848*	39985	(C/T)
CakSNP16147	*Kabuli*	*Ca_Kabuli_Scaffold848*	39990	(A/G)
CakSNP16148	*Kabuli*	*Ca_Kabuli_Scaffold848*	39994	(C/T)
CakSNP16149	*Kabuli*	*Ca_Kabuli_Scaffold848*	89976	(C/A)
CakSNP16150	*Kabuli*	*Ca_Kabuli_Scaffold848*	114078	(T/C)
CakSNP16151	*Kabuli*	*Ca_Kabuli_Scaffold848*	158548	(T/C)
CakSNP16152	*Kabuli*	*Ca_Kabuli_Scaffold848*	158557	(G/A)
CakSNP16153	*Kabuli*	*Ca_Kabuli_Scaffold848*	158569	(C/T)

SNP IDs	Cultivars	Chromosomes/scaffolds	Physical positions (bp)	SNPs
CakSNP16154	*Kabuli*	*Ca_Kabuli_Scaffold848*	158570	(A/G)
CakSNP16155	*Kabuli*	*Ca_Kabuli_Scaffold848*	158611	(C/T)
CakSNP16156	*Kabuli*	*Ca_Kabuli_Scaffold848*	158638	(G/A)
CakSNP16157	*Kabuli*	*Ca_Kabuli_Scaffold848*	158626	(G/T)
CakSNP16158	*Kabuli*	*Ca_Kabuli_Scaffold848*	158594	(A/G)
CakSNP16159	*Kabuli*	*Ca_Kabuli_Scaffold857*	5227	(G/A)
CakSNP16160	*Kabuli*	*Ca_Kabuli_Scaffold8586*	4415	(A/G)
CakSNP16161	*Kabuli*	*Ca_Kabuli_Scaffold859*	9382	(A/G)
CakSNP16162	*Kabuli*	*Ca_Kabuli_Scaffold8641*	502	(G/A)
CakSNP16163	*Kabuli*	*Ca_Kabuli_Scaffold8641*	472	(C/T)
CakSNP16164	*Kabuli*	*Ca_Kabuli_Scaffold8641*	466	(C/T)
CakSNP16165	*Kabuli*	*Ca_Kabuli_Scaffold8641*	460	(T/C)
CakSNP16166	*Kabuli*	*Ca_Kabuli_Scaffold8646*	2056	(A/T)
CakSNP16167	*Kabuli*	*Ca_Kabuli_Scaffold8646*	2073	(G/A)
CakSNP16168	*Kabuli*	*Ca_Kabuli_Scaffold8648*	3938	(C/T)
CakSNP16169	*Kabuli*	*Ca_Kabuli_Scaffold8648*	3977	(A/G)
CakSNP16170	*Kabuli*	*Ca_Kabuli_Scaffold8648*	4058	(C/A)
CakSNP16171	*Kabuli*	*Ca_Kabuli_Scaffold8648*	4050	(A/G)
CakSNP16172	*Kabuli*	*Ca_Kabuli_Scaffold8648*	4036	(A/C)
CakSNP16173	*Kabuli*	*Ca_Kabuli_Scaffold8648*	4013	(G/A)
CakSNP16174	*Kabuli*	*Ca_Kabuli_Scaffold8648*	3992	(G/A)
CakSNP16175	*Kabuli*	*Ca_Kabuli_Scaffold866*	27325	(C/T)
CakSNP16176	*Kabuli*	*Ca_Kabuli_Scaffold866*	27293	(T/G)
CakSNP16177	*Kabuli*	*Ca_Kabuli_Scaffold866*	27279	(C/T)
CakSNP16178	*Kabuli*	*Ca_Kabuli_Scaffold87*	182677	(C/G)
CakSNP16179	*Kabuli*	*Ca_Kabuli_Scaffold87*	182724	(G/A)
CakSNP16180	*Kabuli*	*Ca_Kabuli_Scaffold87*	182769	(G/C)
CakSNP16181	*Kabuli*	*Ca_Kabuli_Scaffold87*	182788	(A/C)
CakSNP16182	*Kabuli*	*Ca_Kabuli_Scaffold873*	265386	(C/T)
CakSNP16183	*Kabuli*	*Ca_Kabuli_Scaffold873*	265395	(C/A)
CakSNP16184	*Kabuli*	*Ca_Kabuli_Scaffold873*	265396	(C/T)
CakSNP16185	*Kabuli*	*Ca_Kabuli_Scaffold873*	265399	(C/A)
CakSNP16186	*Kabuli*	*Ca_Kabuli_Scaffold873*	265458	(C/T)
CakSNP16187	*Kabuli*	*Ca_Kabuli_Scaffold873*	265406	(T/C)
CakSNP16188	*Kabuli*	*Ca_Kabuli_Scaffold875*	49179	(T/C)
CakSNP16189	*Kabuli*	*Ca_Kabuli_Scaffold875*	51703	(C/G)
CakSNP16190	*Kabuli*	*Ca_Kabuli_Scaffold875*	51702	(T/A)
CakSNP16191	*Kabuli*	*Ca_Kabuli_Scaffold875*	51701	(T/C)
CakSNP16192	*Kabuli*	*Ca_Kabuli_Scaffold875*	51714	(T/A)
CakSNP16193	*Kabuli*	*Ca_Kabuli_Scaffold875*	51704	(C/A)
CakSNP16194	*Kabuli*	*Ca_Kabuli_Scaffold875*	62738	(A/G)

SNP IDs	Cultivars	Chromosomes/scaffolds	Physical positions (bp)	SNPs
CakSNP16195	*Kabuli*	*Ca_Kabuli_Scaffold876*	152311	(G/T)
CakSNP16196	*Kabuli*	*Ca_Kabuli_Scaffold876*	188605	(C/G)
CakSNP16197	*Kabuli*	*Ca_Kabuli_Scaffold876*	297059	(T/C)
CakSNP16198	*Kabuli*	*Ca_Kabuli_Scaffold876*	297024	(T/G)
CakSNP16199	*Kabuli*	*Ca_Kabuli_Scaffold876*	297023	(G/A)
CakSNP16200	*Kabuli*	*Ca_Kabuli_Scaffold876*	297022	(G/A)
CakSNP16201	*Kabuli*	*Ca_Kabuli_Scaffold876*	297003	(C/T)
CakSNP16202	*Kabuli*	*Ca_Kabuli_Scaffold876*	296995	(C/T)
CakSNP16203	*Kabuli*	*Ca_Kabuli_Scaffold877*	9813	(G/A)
CakSNP16204	*Kabuli*	*Ca_Kabuli_Scaffold877*	9851	(C/T)
CakSNP16205	*Kabuli*	*Ca_Kabuli_Scaffold88*	9982	(C/A)
CakSNP16206	*Kabuli*	*Ca_Kabuli_Scaffold88*	16398	(C/T)
CakSNP16207	*Kabuli*	*Ca_Kabuli_Scaffold88*	110743	(A/G)
CakSNP16208	*Kabuli*	*Ca_Kabuli_Scaffold88*	240551	(T/C)
CakSNP16209	*Kabuli*	*Ca_Kabuli_Scaffold88*	240550	(G/A)
CakSNP16210	*Kabuli*	*Ca_Kabuli_Scaffold88*	240525	(A/G)
CakSNP16211	*Kabuli*	*Ca_Kabuli_Scaffold88*	274006	(A/G)
CakSNP16212	*Kabuli*	*Ca_Kabuli_Scaffold882*	117376	(G/A)
CakSNP16213	*Kabuli*	*Ca_Kabuli_Scaffold882*	118266	(G/T)
CakSNP16214	*Kabuli*	*Ca_Kabuli_Scaffold882*	232549	(C/A)
CakSNP16215	*Kabuli*	*Ca_Kabuli_Scaffold882*	232570	(T/G)
CakSNP16216	*Kabuli*	*Ca_Kabuli_Scaffold882*	232605	(T/A)
CakSNP16217	*Kabuli*	*Ca_Kabuli_Scaffold882*	358449	(T/A)
CakSNP16218	*Kabuli*	*Ca_Kabuli_Scaffold882*	570871	(T/A)
CakSNP16219	*Kabuli*	*Ca_Kabuli_Scaffold882*	571210	(A/T)
CakSNP16220	*Kabuli*	*Ca_Kabuli_Scaffold882*	573367	(T/A)
CakSNP16221	*Kabuli*	*Ca_Kabuli_Scaffold882*	573706	(A/T)
CakSNP16222	*Kabuli*	*Ca_Kabuli_Scaffold887_1*	26025	(A/C)
CakSNP16223	*Kabuli*	*Ca_Kabuli_Scaffold887_1*	26134	(C/A)
CakSNP16224	*Kabuli*	*Ca_Kabuli_Scaffold887_1*	26121	(A/C)
CakSNP16225	*Kabuli*	*Ca_Kabuli_Scaffold887_1*	26108	(G/T)
CakSNP16226	*Kabuli*	*Ca_Kabuli_Scaffold887_1*	139386	(T/C)
CakSNP16227	*Kabuli*	*Ca_Kabuli_Scaffold887_1*	139396	(G/C)
CakSNP16228	*Kabuli*	*Ca_Kabuli_Scaffold887_1*	139580	(G/T)
CakSNP16229	*Kabuli*	*Ca_Kabuli_Scaffold887_1*	144885	(T/C)
CakSNP16230	*Kabuli*	*Ca_Kabuli_Scaffold887_1*	260488	(A/C)
CakSNP16231	*Kabuli*	*Ca_Kabuli_Scaffold892*	24349	(T/C)
CakSNP16232	*Kabuli*	*Ca_Kabuli_Scaffold892*	24323	(A/G)
CakSNP16233	*Kabuli*	*Ca_Kabuli_Scaffold892*	24317	(G/T)
CakSNP16234	*Kabuli*	*Ca_Kabuli_Scaffold892*	24316	(T/A)
CakSNP16235	*Kabuli*	*Ca_Kabuli_Scaffold892*	35527	(T/C)

SNP IDs	Cultivars	Chromosomes/scaffolds	Physical positions (bp)	SNPs
CakSNP16236	*Kabuli*	*Ca_Kabuli_Scaffold892*	35501	(A/G)
CakSNP16237	*Kabuli*	*Ca_Kabuli_Scaffold892*	35495	(G/T)
CakSNP16238	*Kabuli*	*Ca_Kabuli_Scaffold892*	35494	(T/A)
CakSNP16239	*Kabuli*	*Ca_Kabuli_Scaffold892*	35489	(A/G)
CakSNP16240	*Kabuli*	*Ca_Kabuli_Scaffold892*	35514	(G/T)
CakSNP16241	*Kabuli*	*Ca_Kabuli_Scaffold895*	18621	(C/A)
CakSNP16242	*Kabuli*	*Ca_Kabuli_Scaffold895*	18693	(G/C)
CakSNP16243	*Kabuli*	*Ca_Kabuli_Scaffold895*	20978	(T/C)
CakSNP16244	*Kabuli*	*Ca_Kabuli_Scaffold895*	158250	(A/G)
CakSNP16245	*Kabuli*	*Ca_Kabuli_Scaffold901*	91817	(C/T)
CakSNP16246	*Kabuli*	*Ca_Kabuli_Scaffold901*	91849	(T/C)
CakSNP16247	*Kabuli*	*Ca_Kabuli_Scaffold908*	15845	(C/A)
CakSNP16248	*Kabuli*	*Ca_Kabuli_Scaffold908*	15841	(G/A)
CakSNP16249	*Kabuli*	*Ca_Kabuli_Scaffold908*	15840	(G/A)
CakSNP16250	*Kabuli*	*Ca_Kabuli_Scaffold908*	15816	(C/T)
CakSNP16251	*Kabuli*	*Ca_Kabuli_Scaffold908*	15813	(G/T)
CakSNP16252	*Kabuli*	*Ca_Kabuli_Scaffold908*	15810	(A/G)
CakSNP16253	*Kabuli*	*Ca_Kabuli_Scaffold908*	15799	(C/T)
CakSNP16254	*Kabuli*	*Ca_Kabuli_Scaffold908*	15786	(G/A)
CakSNP16255	*Kabuli*	*Ca_Kabuli_Scaffold908*	15785	(G/A)
CakSNP16256	*Kabuli*	*Ca_Kabuli_Scaffold908*	15822	(C/T)
CakSNP16257	*Kabuli*	*Ca_Kabuli_Scaffold908*	15834	(C/A)
CakSNP16258	*Kabuli*	*Ca_Kabuli_Scaffold913*	14282	(A/C)
CakSNP16259	*Kabuli*	*Ca_Kabuli_Scaffold913*	332494	(G/C)
CakSNP16260	*Kabuli*	*Ca_Kabuli_Scaffold913*	403513	(G/A)
CakSNP16261	*Kabuli*	*Ca_Kabuli_Scaffold913*	403572	(C/G)
CakSNP16262	*Kabuli*	*Ca_Kabuli_Scaffold913*	403553	(T/C)
CakSNP16263	*Kabuli*	*Ca_Kabuli_Scaffold913*	403531	(A/C)
CakSNP16264	*Kabuli*	*Ca_Kabuli_Scaffold914*	18826	(A/G)
CakSNP16265	*Kabuli*	*Ca_Kabuli_Scaffold914*	18810	(C/A)
CakSNP16266	*Kabuli*	*Ca_Kabuli_Scaffold914*	18809	(C/T)
CakSNP16267	*Kabuli*	*Ca_Kabuli_Scaffold914*	18750	(C/A)
CakSNP16268	*Kabuli*	*Ca_Kabuli_Scaffold914*	38792	(A/G)
CakSNP16269	*Kabuli*	*Ca_Kabuli_Scaffold914*	38776	(C/A)
CakSNP16270	*Kabuli*	*Ca_Kabuli_Scaffold914*	38775	(C/T)
CakSNP16271	*Kabuli*	*Ca_Kabuli_Scaffold914*	38716	(C/A)
CakSNP16272	*Kabuli*	*Ca_Kabuli_Scaffold914*	53125	(T/A)
CakSNP16273	*Kabuli*	*Ca_Kabuli_Scaffold914*	53132	(C/T)
CakSNP16274	*Kabuli*	*Ca_Kabuli_Scaffold914*	53270	(A/G)
CakSNP16275	*Kabuli*	*Ca_Kabuli_Scaffold916*	1036	(A/G)
CakSNP16276	*Kabuli*	*Ca_Kabuli_Scaffold916*	275497	(T/C)

**SNP IDs**	**Cultivars**	**Chromosomes/scaffolds**	**Physical positions (bp)**	**SNPs**
CakSNP16277	*Kabuli*	*Ca_Kabuli_Scaffold916*	275510	(A/T)
CakSNP16278	*Kabuli*	*Ca_Kabuli_Scaffold916*	275512	(G/T)
CakSNP16279	*Kabuli*	*Ca_Kabuli_Scaffold916*	275519	(A/C)
CakSNP16280	*Kabuli*	*Ca_Kabuli_Scaffold916*	275493	(A/T)
CakSNP16281	*Kabuli*	*Ca_Kabuli_Scaffold919*	119274	(T/C)
CakSNP16282	*Kabuli*	*Ca_Kabuli_Scaffold922*	111921	(C/A)
CakSNP16283	*Kabuli*	*Ca_Kabuli_Scaffold922*	121629	(T/C)
CakSNP16284	*Kabuli*	*Ca_Kabuli_Scaffold93*	205677	(G/A)
CakSNP16285	*Kabuli*	*Ca_Kabuli_Scaffold93*	506983	(C/A)
CakSNP16286	*Kabuli*	*Ca_Kabuli_Scaffold93*	578138	(G/A)
CakSNP16287	*Kabuli*	*Ca_Kabuli_Scaffold93*	578146	(A/G)
CakSNP16288	*Kabuli*	*Ca_Kabuli_Scaffold93*	578158	(A/G)
CakSNP16289	*Kabuli*	*Ca_Kabuli_Scaffold93*	640373	(A/C)
CakSNP16290	*Kabuli*	*Ca_Kabuli_Scaffold93*	640389	(G/T)
CakSNP16291	*Kabuli*	*Ca_Kabuli_Scaffold93*	640430	(C/T)
CakSNP16292	*Kabuli*	*Ca_Kabuli_Scaffold93*	640487	(A/C)
CakSNP16293	*Kabuli*	*Ca_Kabuli_Scaffold93*	640459	(G/T)
CakSNP16294	*Kabuli*	*Ca_Kabuli_Scaffold93*	640448	(T/C)
CakSNP16295	*Kabuli*	*Ca_Kabuli_Scaffold93*	640445	(C/T)
CakSNP16296	*Kabuli*	*Ca_Kabuli_Scaffold93*	640444	(G/T)
CakSNP16297	*Kabuli*	*Ca_Kabuli_Scaffold93*	645328	(A/C)
CakSNP16298	*Kabuli*	*Ca_Kabuli_Scaffold93*	645344	(G/T)
CakSNP16299	*Kabuli*	*Ca_Kabuli_Scaffold93*	645442	(A/C)
CakSNP16300	*Kabuli*	*Ca_Kabuli_Scaffold93*	645414	(G/T)
CakSNP16301	*Kabuli*	*Ca_Kabuli_Scaffold93*	645403	(T/C)
CakSNP16302	*Kabuli*	*Ca_Kabuli_Scaffold93*	645400	(C/T)
CakSNP16303	*Kabuli*	*Ca_Kabuli_Scaffold93*	645399	(G/T)
CakSNP16304	*Kabuli*	*Ca_Kabuli_Scaffold93*	645402	(G/A)
CakSNP16305	*Kabuli*	*Ca_Kabuli_Scaffold93*	651020	(C/T)
CakSNP16306	*Kabuli*	*Ca_Kabuli_Scaffold93*	651018	(C/T)
CakSNP16307	*Kabuli*	*Ca_Kabuli_Scaffold93*	651009	(A/G)
CakSNP16308	*Kabuli*	*Ca_Kabuli_Scaffold93*	650981	(T/C)
CakSNP16309	*Kabuli*	*Ca_Kabuli_Scaffold93*	650968	(G/A)
CakSNP16310	*Kabuli*	*Ca_Kabuli_Scaffold93*	650958	(G/A)
CakSNP16311	*Kabuli*	*Ca_Kabuli_Scaffold93*	650955	(T/C)
CakSNP16312	*Kabuli*	*Ca_Kabuli_Scaffold931*	13212	(T/C)
CakSNP16313	*Kabuli*	*Ca_Kabuli_Scaffold9323*	5263	(A/G)
CakSNP16314	*Kabuli*	*Ca_Kabuli_Scaffold937*	132087	(C/G)
CakSNP16315	*Kabuli*	*Ca_Kabuli_Scaffold937*	134580	(C/G)
CakSNP16316	*Kabuli*	*Ca_Kabuli_Scaffold937*	134707	(G/A)
CakSNP16317	*Kabuli*	*Ca_Kabuli_Scaffold948*	12188	(C/T)

SNP IDs	Cultivars	Chromosomes/scaffolds	Physical positions (bp)	SNPs
CakSNP16318	*Kabuli*	*Ca_Kabuli_Scaffold948*	21152	(C/T)
CakSNP16319	*Kabuli*	*Ca_Kabuli_Scaffold953*	42329	(T/C)
CakSNP16320	*Kabuli*	*Ca_Kabuli_Scaffold953*	57413	(A/T)
CakSNP16321	*Kabuli*	*Ca_Kabuli_Scaffold956*	21441	(C/A)
CakSNP16322	*Kabuli*	*Ca_Kabuli_Scaffold956*	27612	(A/G)
CakSNP16323	*Kabuli*	*Ca_Kabuli_Scaffold956*	27710	(T/G)
CakSNP16324	*Kabuli*	*Ca_Kabuli_Scaffold956*	27693	(T/C)
CakSNP16325	*Kabuli*	*Ca_Kabuli_Scaffold956*	28015	(A/C)
CakSNP16326	*Kabuli*	*Ca_Kabuli_Scaffold956*	28014	(T/A)
CakSNP16327	*Kabuli*	*Ca_Kabuli_Scaffold956*	29736	(T/A)
CakSNP16328	*Kabuli*	*Ca_Kabuli_Scaffold959*	56173	(C/T)
CakSNP16329	*Kabuli*	*Ca_Kabuli_Scaffold959*	56159	(G/A)
CakSNP16330	*Kabuli*	*Ca_Kabuli_Scaffold959*	56156	(G/A)
CakSNP16331	*Kabuli*	*Ca_Kabuli_Scaffold959*	56117	(G/A)
CakSNP16332	*Kabuli*	*Ca_Kabuli_Scaffold962*	149524	(C/T)
CakSNP16333	*Kabuli*	*Ca_Kabuli_Scaffold962*	149545	(C/T)
CakSNP16334	*Kabuli*	*Ca_Kabuli_Scaffold962*	149580	(G/A)
CakSNP16335	*Kabuli*	*Ca_Kabuli_Scaffold962*	149585	(A/G)
CakSNP16336	*Kabuli*	*Ca_Kabuli_Scaffold962*	149645	(G/T)
CakSNP16337	*Kabuli*	*Ca_Kabuli_Scaffold962*	149631	(G/A)
CakSNP16338	*Kabuli*	*Ca_Kabuli_Scaffold962*	149589	(G/A)
CakSNP16339	*Kabuli*	*Ca_Kabuli_Scaffold962*	149583	(G/A)
CakSNP16340	*Kabuli*	*Ca_Kabuli_Scaffold962*	149577	(G/T)
CakSNP16341	*Kabuli*	*Ca_Kabuli_Scaffold962*	149689	(G/A)
CakSNP16342	*Kabuli*	*Ca_Kabuli_Scaffold962*	149687	(A/T)
CakSNP16343	*Kabuli*	*Ca_Kabuli_Scaffold962*	149676	(T/C)
CakSNP16344	*Kabuli*	*Ca_Kabuli_Scaffold962*	149661	(G/A)
CakSNP16345	*Kabuli*	*Ca_Kabuli_Scaffold962*	149649	(C/T)
CakSNP16346	*Kabuli*	*Ca_Kabuli_Scaffold962*	149644	(C/T)
CakSNP16347	*Kabuli*	*Ca_Kabuli_Scaffold962*	149628	(G/A)
CakSNP16348	*Kabuli*	*Ca_Kabuli_Scaffold962*	149613	(G/T)
CakSNP16349	*Kabuli*	*Ca_Kabuli_Scaffold963*	77099	(C/T)
CakSNP16350	*Kabuli*	*Ca_Kabuli_Scaffold98*	287956	(T/A)
CakSNP16351	*Kabuli*	*Ca_Kabuli_Scaffold98*	287933	(T/C)
CakSNP16352	*Kabuli*	*Ca_Kabuli_Scaffold98*	388565	(T/C)
CakSNP16353	*Kabuli*	*Ca_Kabuli_Scaffold98*	434956	(A/G)
CakSNP16354	*Kabuli*	*Ca_Kabuli_Scaffold98*	440243	(G/C)
CakSNP16355	*Kabuli*	*Ca_Kabuli_Scaffold98*	447314	(G/C)
CakSNP16356	*Kabuli*	*Ca_Kabuli_Scaffold98*	447311	(G/A)
CakSNP16357	*Kabuli*	*Ca_Kabuli_Scaffold98*	485305	(G/C)
CakSNP16358	*Kabuli*	*Ca_Kabuli_Scaffold98*	485302	(G/A)

SNP IDs	Cultivars	Chromosomes/scaffolds	Physical positions (bp)	SNPs
CakSNP16359	*Kabuli*	*Ca_Kabuli_Scaffold98*	533710	(T/C)
CakSNP16360	*Kabuli*	*Ca_Kabuli_Scaffold98*	533677	(T/A)
CakSNP16361	*Kabuli*	*Ca_Kabuli_Scaffold98*	533812	(G/C)
CakSNP16362	*Kabuli*	*Ca_Kabuli_Scaffold98*	562979	(G/A)
CakSNP16363	*Kabuli*	*Ca_Kabuli_Scaffold98*	563016	(T/C)
CakSNP16364	*Kabuli*	*Ca_Kabuli_Scaffold98*	563048	(A/C)
CakSNP16365	*Kabuli*	*Ca_Kabuli_Scaffold981*	84091	(G/A)
CakSNP16366	*Kabuli*	*Ca_Kabuli_Scaffold981*	84088	(C/G)
CakSNP16367	*Kabuli*	*Ca_Kabuli_Scaffold981*	84087	(C/A)
CakSNP16368	*Kabuli*	*Ca_Kabuli_Scaffold981*	84072	(G/A)
CakSNP16369	*Kabuli*	*Ca_Kabuli_Scaffold981*	84057	(A/G)
CakSNP16370	*Kabuli*	*Ca_Kabuli_Scaffold981*	84034	(C/T)
CakSNP16371	*Kabuli*	*Ca_Kabuli_Scaffold981*	84026	(G/C)
CakSNP16372	*Kabuli*	*Ca_Kabuli_Scaffold981*	84019	(C/T)
CakSNP16373	*Kabuli*	*Ca_Kabuli_Scaffold981*	84007	(C/G)
CakSNP16374	*Kabuli*	*Ca_Kabuli_Scaffold999*	77761	(A/T)
CakSNP16375	*Kabuli*	*Ca_Kabuli_Scaffold999*	77763	(C/T)
CakSNP16376	*Kabuli*	*Ca_Kabuli_Scaffold999*	77767	(A/T)
