# Supplementary material for: Genome-Wide Scans for Delineation of Candidate Genes Regulating Seed-Protein Content in Chickpea
Source: Front Plant Sci. 2016 Mar 23;7:302. doi: 10.3389/fpls.2016.00302 (PMC4803732; doi:10.3389/fpls.2016.00302)
Supplement: Supplementary file 3 [file Image_1.PDF]

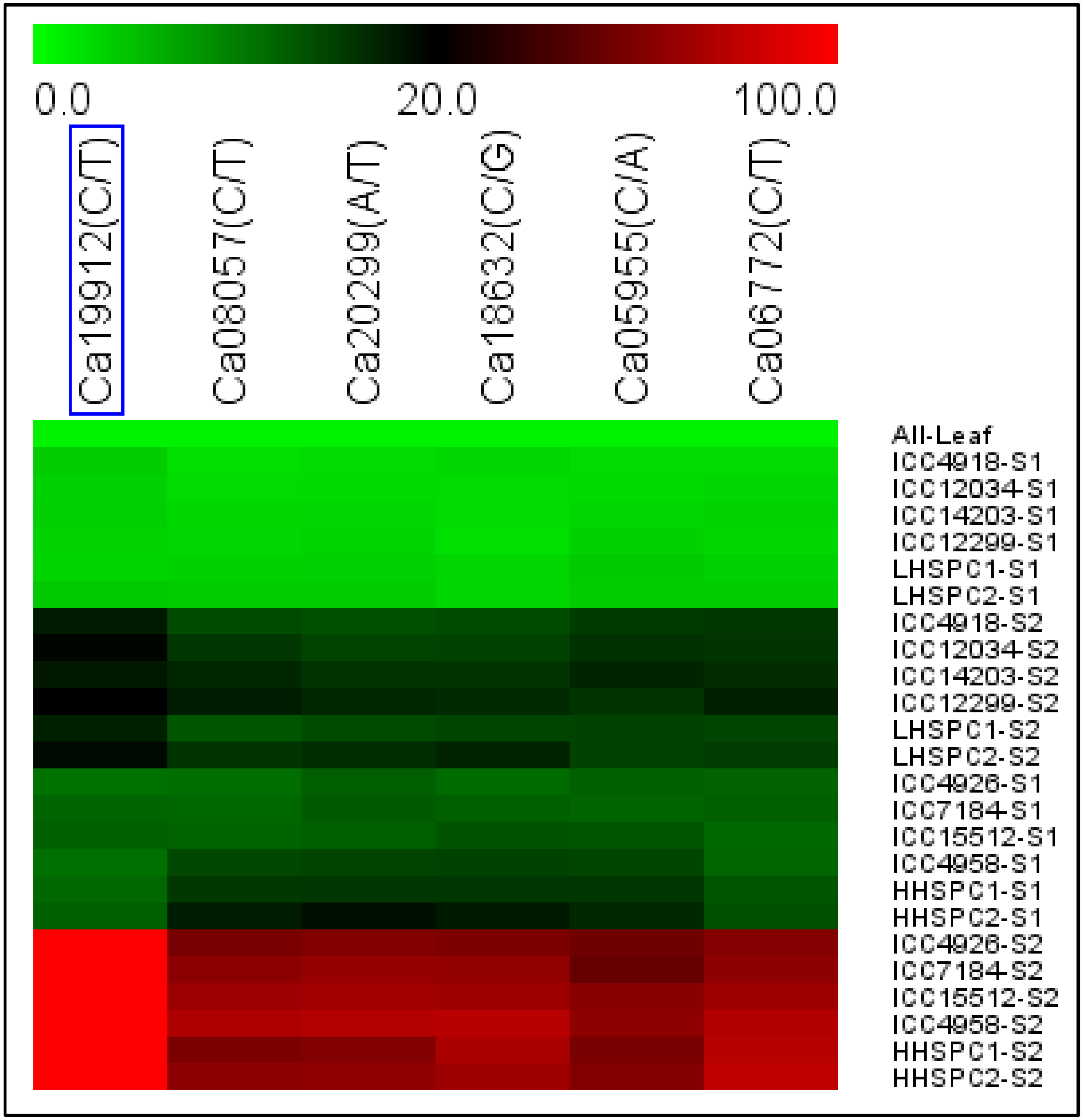


**Figure S1.** Hierarchical cluster display illustrating the differential expression profiles of SPC-associated six SNPs-carrying genes (validated by association mapping and selective genotyping in a mapping population) in the leaves as well as early cell division phase and late seed maturation developmental stages of eight chickpea accessions with contrasting levels of low (ICC 4918, IC 12034, ICC 14203 and ICC 12299 with 15.6-16.5%) and high (ICC 4926, ICC 7184, ICC 15512 and ICC 4958 with 21.5-22.4%) seed protein content in chickpea. One strong SPC-associated non-synonymous SNP-containing zinc finger transcription factor gene exhibiting pronounced up-regulation in two seed developmental stages of high than that of low SPC-containing accessions, parents and RIL mapping individuals are highlighted with blue box. The colour scale at the top signifies average log signal expression value of genes in various tissues and developmental stages; in which green, black and red colour represent low, medium and high level of expression, respectively. The details regarding identities of SNPs-containing genes are mentioned in the Table 2. The tissues and genes used for expression profiling are illustrated on the right and top side of expression map, respectively. Seed1: Seed development stage 1 [10-20 days after podding (DAP)] and Seed2: Seed development stage 2 (21-30 DAP). LHSPC (low homozygous seed protein containing) and HHSPC (high homozygous seed protein containing) RIL mapping individuals.
